# Differential Timing for Glucose Assimilation in *Prochlorococcus* and Coexistent Microbial Populations in the North Pacific Subtropical Gyre

**DOI:** 10.1128/spectrum.02466-22

**Published:** 2022-09-13

**Authors:** María del Carmen Muñoz-Marín, Solange Duhamel, Karin M. Björkman, Jonathan D. Magasin, Jesús Díez, David M. Karl, José M. García-Fernández

**Affiliations:** a Departamento de Bioquímica y Biología Molecular, Campus de Excelencia Internacional Agroalimentario, Universidad de Córdoba, Córdoba, Spain; b Lamont-Doherty Earth Observatory of Columbia University, Division of Biology and Paleo Environment, Palisades, New York, USA; c Daniel K. Inouye Center for Microbial Oceanography: Research and Education (C-MORE), University of Hawaii at Manoa, C-MORE Hale, Honolulu, Hawaii, USA; d Ocean Sciences Department, University of California, Santa Cruz, California, USA; Nanyang Technological University

**Keywords:** glucose assimilation, cyanobacteria, *Prochlorococcus*, diel cycles, assimilation, glucose transport, picocyanobacteria

## Abstract

The marine cyanobacterium *Prochlorococcus* can utilize glucose as a source of carbon. However, the relative importance of inorganic and organic carbon assimilation and the timing of glucose assimilation are still poorly understood in these numerically dominant cyanobacteria. Here, we investigated whole microbial community and group-specific primary production and glucose assimilation using incubations with radioisotopes combined with flow cytometry cell sorting. We also studied changes in the microbial community structure in response to glucose enrichments and analyzed the transcription of *Prochlorocccus* genes involved in carbon metabolism and photosynthesis. Our results showed a diel variation for glucose assimilation in *Prochlorococcus*, with maximum assimilation at midday and minimum at midnight (~2-fold change), which was different from that of the total microbial community. This suggests that the timing in glucose assimilation in *Prochlorococcus* is coupled to photosynthetic light reactions producing energy, it being more convenient for *Prochlorococcus* to show maximum glucose uptake precisely when the rest of microbial populations have their minimum glucose uptake. Many transcriptional responses to glucose enrichment occurred after 12- and 24-h periods, but community composition did not change. High-light *Prochlorococcus* strains were the most impacted by glucose addition, with transcript-level increases observed for genes in pathways for glucose metabolism, such as the pentose phosphate pathway, the Entner-Doudoroff pathway, glycolysis, respiration, and glucose transport. While *Prochlorococcus* C assimilation from glucose represented less than 0.1% of the bacterium’s photosynthetic C fixation, increased assimilation during the day and *glcH* gene upregulation upon glucose enrichment indicate an important role of mixotrophic C assimilation by natural populations of *Prochlorococcus.*

**IMPORTANCE** Several studies have demonstrated that *Prochlorococcus*, the most abundant photosynthetic organism on Earth, can assimilate organic molecules, such as amino acids, amino sugars, ATP, phosphonates, and dimethylsulfoniopropionate. This autotroph can also assimilate small amounts of glucose, supporting the hypothesis that *Prochlorococcus* is mixotrophic. Our results show, for the first time, a diel variability in glucose assimilation by natural populations of *Prochlorococcus* with maximum assimilation during midday. Based on our previous results, this indicates that *Prochlorococcus* could maximize glucose uptake by using ATP made during the light reactions of photosynthesis. Furthermore, *Prochlorococcus* showed a different timing of glucose assimilation from the total population, which may offer considerable fitness advantages over competitors “temporal niches.” Finally, we observed transcriptional changes in some of the genes involved in carbon metabolism, suggesting that *Prochlorococcus* can use both pathways previously proposed in cyanobacteria to metabolize glucose.

## INTRODUCTION

*Prochlorococcus* is the most abundant photosynthetic organism on Earth, contributing substantially to total primary production (1–4). The outstanding relevance of this microorganism in the field of marine microbiology and ecology has been demonstrated by a large series of studies published since its discovery ca. 35 years ago (5). Because of its abundance, *Prochlorococcus* is also one of the main microbial players in biogeochemical cycles (6).

Early studies of this cyanobacterium focused on photosynthesis, and it was widely considered an obligate photoautotrophic organism. More recently, *Prochlorococcus* was shown to take up and use organic compounds, such as amino acids (containing nitrogen) (7, 8), dimethylsulfionopropionate (DMSP [containing sulfur]) (9), or phosphonates and ATP (containing phosphorus) (10, 11), which recently has been reviewed (12). Since *Prochlorococcus* thrives in oligotrophic regions of the ocean, it was thought that these organic molecules are taken up because they provide limiting elements. In this context, the discovery of glucose assimilation in *Prochlorococcus* was surprising (13), since this molecule is devoid of limiting elements, containing only carbon, oxygen, and hydrogen. However, it contains potential energy which could be used by *Prochlorococcus*. Previous studies have shed some light on this hypothesis. Glucose addition to *Prochlorococcus* culture medium induced changes in the expression of a number of genes, including *glcH*, which encodes a multiphasic transporter with a high-affinity substrate constant (*K_s_*) in the nanomolar range (14). The ubiquity of this gene in all sequenced genomes of *Prochlorococcus* and marine *Synechococcus* and the diversity of kinetics of the transporter (*K_s_* and *V*_max_ parameters) (15) suggest that *glcH* is very important for *Prochlorococcus* and *Synechococcus* and has been subjected to selective evolution in their genomes (12, 15).

The effects of glucose addition on the *Prochlorococcus* transcriptome and proteome have been studied in culture. Results from the transcriptome showed increased expression of genes related to glucose metabolism (13). Proteomic analysis showed some changes which were reproducible but quantitatively small (15), including for proteins related to glucose metabolism. Moreover, the expression of *glcH* has been shown to increase with higher concentrations of glucose in *Prochlorococcus* cultures, but to decrease in the dark (16). However, less is known about *Prochlorococcus* glucose utilization in the wild: glucose assimilation was demonstrated in natural populations of *Prochlorococcus* in the Atlantic (14) and in the Southwest Pacific (11) Oceans; furthermore, it was shown that *Prochlorococcus* glucose assimilation in the field was reduced in the dark and in the presence of photosynthesis inhibitors (11), in agreement with previous laboratory studies (13, 15). However, organic carbon, including glucose, may represent a large fraction of the carbon assimilated by phytoplankton adapted to living under low light such as at the base of the photic layer (17).

Here, we further investigated the effects of glucose addition on *Prochlorococcus* glucose metabolism in field experiments carried out at Station ALOHA in the North Pacific Subtropical Gyre (NPSG). Over daily light-dark cycles, we measured glucose turnover rates and assimilation by the whole microbial community as well as in flow-sorted *Prochlorococcus*. Paired experiments measuring primary production were conducted in order to assess the relative contribution of glucose assimilation to *Prochlorococcus* total carbon assimilation. Furthermore, we used metagenomics to investigate the effect of glucose enrichment on the composition of natural populations (both picocyanobacteria and heterotrophic bacteria) and metatranscriptomics (microarrays) to identify transcription changes of genes involved in carbon metabolism and photosynthesis pathways in *Prochlorococcus* populations.

## RESULTS

### *Prochlorococcus* cell abundance.

*Prochlorococcus* dominated phytoplankton abundances, with an average of (1.23 ± 0.35) × 10^5^ cells mL^−1^, with *Synechococcus*, *Crocosphaera*, and picophytoeukaryotes contributing (1.47 ± 0.39) × 10^3^, (3.77 ± 0.97) × 10^2^, and (8.32 ± 1.92) × 10^2^ cells mL^−1^, respectively (average ± standard deviation [SD]; *n* = 20; biological and technical replicates, averaging samples taken at the same depth but at different times) (see Fig. S1A in the supplemental material).

*Prochlorococcus* cell abundance showed a diel cycle with increasing cell abundance during the daylight period and lowest values during the dark period (ranging from 0.7 × 10^5^ to 1.8 × 10^5^ cells mL^−1^) (Fig. S1B).

### Inorganic carbon fixation rates (primary production).

**FIG 1:**
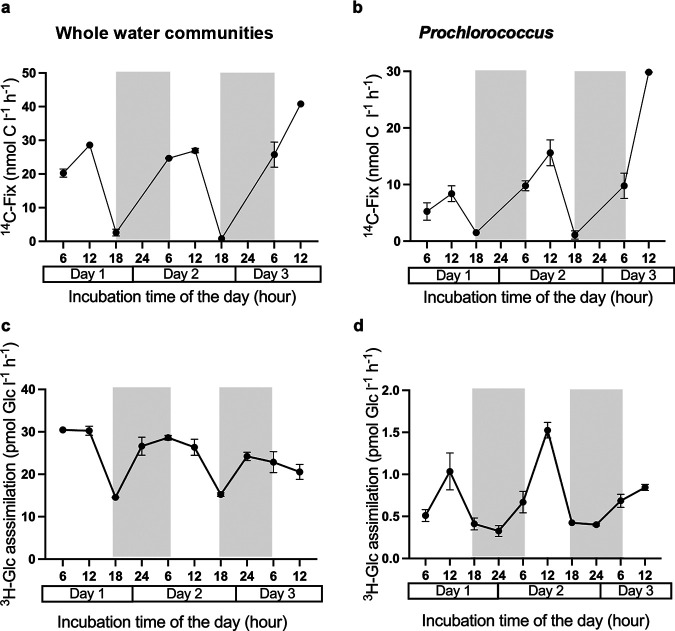
Rates of inorganic carbon fixation and glucose assimilation over the time. (a and b) Inorganic carbon fixation rates over time by the whole water communities (a) (total, >0.2 μm; nanomoles of C per liter per hour), and by *Prochlorococcus* (b) (nanomoles of C per liter per hour). (c and d) Glucose assimilation over time by the whole water communities (c) (total, >0.2 μm; picomoles of Glc per liter per hour) and by *Prochlorococcus* as a group (d) (picomoles of Glc per liter per hour). The shaded area represents the dark period. Glc, glucose.

Rates of inorganic carbon fixation by the whole water communities (cells retained by a polycarbonate filter of 0.2 μm porosity) (Fig. 1a and Table 1) ranged from 0.8 ± 0.0 nmol C L^−1^ h^−1^ at night (incubations from 18:30 to 4:00) to 28.6 ± 0.8 nmol C L^−1^ h^−1^ at midday (incubations from noon to 16:00). On average, carbon fixation during daylight was 25.1 ± 3.1 nmol C L^−1^ h^−1^ (*n* = 12). On a per cell level, *Prochlorococcus* also showed a pronounced diel cycling in carbon fixation with undetectable values at night and 13.1 ± 8.0 nmol C L^−1^ h^−1^ (1.23 ± 0.67 fg C cell^−1^ h^−1^) during the day (*n* = 12) (Fig. 1b and Table 1). As a taxon-specific group, *Prochlorococcus* represented 41.5% ±16.5% of the total carbon fixation during the day (34.8% ± 10% in the morning and 49.6% ± 20% in the afternoon; *n* = 11).

**TABLE 1:** Inorganic carbon fixation and glucose assimilation rates measured during the diel study^*a*^

Sample no.	Time of day	^14^C-PP-WW (nmol C L^−1^ h^−1^)	[^3^H]Glc-WW (pmol Glc L^−1^ h^−1^)	[^14^C]Glc-WW (pmol glc L^−1^ h^−1^)	PRO cell no. (10^8^ L^−1^)	^14^C-PP-PRO (nmol L^−1^ h^−1^)^*b*^	[^3^H]Glc-PRO (pmol Glc L^−1^ h^−1^)^*c*^
1 (day 1)	6:00–10:00	20.3 ± 1.2	30.4 ± 0.3	64.5 ± 13.2	0.89	5.25 ± 1.54	0.51 ± 0.06
						**26% WW**	**1.7% WW**
							**0.06% PRO**

2	12:00–16:00	28.6 ± 0.8	30.3 ± 1.1	54.1 ± 2	1.41	8.40 ± 1.38	1.03 ± 0.21
						**30% WW**	**3.4% WW**
							**0.08% PRO**

3	18:00–22:00	2.6 ± 1.0	14.5 ± 0.1	28.2 ± 0.6	1.57	1.51 ± 0.45	0.40 ± 0.06
						**67% WW**	**2.8% WW**
						−^*d*^	**0.18% PRO**

4	24:00–4:00	−	26.6 ± 2.1	55.2	0.80	−^*d*^	0.32 ± 0.06
							**1.23% WW**

5 (day 2)	6:00–10:00	24.7 ± 0.1	28.6 ± 0.5	57.1 ± 1.1	0.88	9.79 ± 0.88	0.67 ± 0.13
						**40% WW**	**2.3% WW**
							**0.04% PRO**

6	12:00–16:00	27.0 ± 0.7	26.3 ± 1.9	55.2 ± 1.9	1.90	15.62 ± 2.27	1.52 ± 0.00
						**58% WW**	**5.8% WW**
							**0.06% PRO**

7	18:00–22:00	0.8 ± 0.00	15.2 ± 0.4	28.7 ± 1.4	1.43	1.10 ± 0.75	0.42 ± 0.01
						**100% WW**	**2.8% WW**
							**0.31% PRO**

8	24:00–4:00	−	24.2 ± 1.0	45.1 ± 1.4	0.93	−	0.40 ± 0.01
							**1.65% WW**

9 (day 3)	6:00–10:00	25.7 ± 3.7	23.0 ± 2.4	46.0 ± 10.2	1.10	9.79 ± 2.22	0.69 ± 0.08
						**39% WW**	**3% WW**
							**0.04% PRO**

10	12:00–16:00	23.8	20.6 ± 1.7	41.4 ± 1.9	1.40	29.83 ± 0.06	0.85 ± 0.04
						**100% WW**	**4.1% WW**
							**0.02% PRO**

a Shown are rates of inorganic carbon fixation (^14^C-PP [primary productivity]) and assimilation of glucose ([^3^H]Glc and [^14^C]Glc) by the whole water communities (WW), *Prochlorococcus* cell abundance (PRO cell no.), *Prochlorococcus* sodium bicarbonate fixation (^14^C-PP-PRO), and glucose assimilation ([^3^H]Glc-PRO) during the diel study. The values presented in the table are the average of two technical replicates ± SD.

b Percentages in boldface in the ^14^C-PP-PRO column show the relative contribution by *Prochlorococcus* to whole water (WW) primary production.

c Percentages in boldface in the [^3^H]Glc-PRO column show the percentage of glucose assimilation by *Prochlorococcus* relative to community glucose assimilation (WW) and the carbon contribution from glucose to inorganic carbon fixation by *Prochlorococcus*.

d −, measured but not detected at night.

### Glucose assimilation.

The two bioassays conducted using [^3^H]Glc during HOT 296 indicated an ambient glucose concentration of 1.1 ± 0.1 nmol L^−1^. The [^3^H]Glc added, on average, 1.9 ± 0.2 nmol L^−1^ (*n* = 20). Taking both the ambient and added glucose concentrations into account, the assimilation of glucose by the whole community (>0.2 μm) (Fig. 1c and Table 1) varied over the diel cycle by approximately a factor of 2 (from a minimum value of 14.5 ± 0.1 to a maximum value of 30.4 ± 0.3 pmol [^3^H]Glc L^−1^ h^−1^), with on average 24.0 ± 5.6 pmol [^3^H]Glc L^−1^ h^−1^ (*n* = 20). A diel pattern was observed for the whole water communities, with lower values in incubations started at dusk (18:00), averaging 14.9 ± 0.5 pmol [^3^H]Glc L^−1^ h^−1^ (*n* = 4), and higher values in incubations started at midnight and early morning, averaging 25.6 ± 2.7 pmol [^3^H]Glc L^−1^ h^−1^ (*n* = 8) (Fig. 1c and Table 1). The ambient glucose concentration and the assimilation of glucose were also measured using [^14^C]Glc as a potentially better tracker of the fate of carbon from glucose amendments. The ambient glucose concentrations were 0.9 ± 0.1 nmol L^−1^ (*n* = 5) and 0.5 ± 0.2 nmol L^−1^, respectively, during the HOT 295 and 298 cruises. Taking both the ambient and added glucose concentrations into account, the [^14^C]Glc assimilation by the whole water communities showed the same pattern as the [^3^H]Glc assimilation, although assimilation varied by approximately a factor of 2.6 over the day (27.7 to 73.9 pmol [^14^C]Glc L^−1^ h^−1^), with on average 47.1 ± 12.6 pmol Glc L^−1^ h^−1^ (*n* = 20) (Table 1). Glucose assimilation based on [^14^C]Glc yielded nearly twice the assimilation rate values measured using [^3^H]Glc, indicating that while more sensitive than ^14^C, C uptake based on the ^3^H-labeled compound likely underestimated glucose assimilation. However, due to the much lower specific activity of [^14^C]Glc than [^3^H]Glc and the necessity to keep the glucose enrichments low at ~2 nmol L^−1^ in these experiments, the radioactivity in *Prochlorococcus* sorted cells was not significantly different from that in blank samples, and [^14^C]Glc assimilation cannot be reported for *Prochlorococcus*.

*Prochlorococcus* showed a different diel pattern in [^3^H]Glc assimilation than the whole water communities, with (2.5 ± 0.6) × 10^−4^ fg C cell^−1^ h^−1^ at night (*n* = 8) and (4.9 ± 0.8) × 10^−4^ fg C cell^−1^ h^−1^ during the day (*n* = 12) (Table 2). Similarly, as a taxon-specific group, *Prochlorococcus* showed a pronounced diel cycle, with maximum values at noon of 0.87 ± 0.36 pmol Glc L^−1^ h^−1^ (*n* = 12) and minimum values at midnight of 0.39 ± 0.05 pmol Glc L^−1^ h^−1^ (*n* = 8), an ~2.3-fold change (*n* = 4) (Fig. 1d and Tables 1 and 2).

**TABLE 2:** List of inorganic carbon fixation and glucose assimilation rates by the whole water communities, and by *Prochlorococcus* and *Synechococcus* reported for natural samples^*a*^

Parameter	Rate for:	
	Whole water communities	*Prochlorococcus*
Carbon fixation ([^14^C]sodium bicarbonate)	25.1 ± 3.1 nmol C L^−1^ h^−1^ (*n* = 11)^*b*^	13.1 ± 8.0 nmol C L^−1^ h^−1^ (*n* = 12)^*b*^^,^^*c*^ or 1.23 ± 0.67 fg C cell^−1^ h^−1^ (*n* = 12)^*b*^
		0.9–1.6 fg C cell^−1^ h^−1^^*d*^
		1.2 fg C cell^−1^ h^−1^^*e*^
Glucose		
[^3^H]glucose	0.024 ± 0.006 nmol Glc L^−1^ h^−1^ (*n* = 20)^*b*^	8.7 ± 3.6 (day; *n* = 12)/3.9 ± 0.5 (night; *n* = 8) 10^−4^ nmol Glc L^−1^ h^−1^^*b*^^,^^*c*^ or 4.9 ± 0.8 (day; *n* = 12)/2.5 ± 0.6 (night; *n* = 8) 10^−4^ fg C cell^−1^ h^−1^^*b*^
		2.1 × 10^−4^ fg C cell^−1^ h^−1^^*f*^
		1.9 × 10^−4^ fg C cell^−1^ h^−1^^*g*^

[^14^C]glucose	0.047 ± 0.012 nmol Glc L^−1^ h^−1^ (*n* = 19)^*b*^	Undetectable^*b*^

a Rates of inorganic carbon fixation and glucose assimilation by the whole water communities and by *Prochlorococcus* and *Synechococcus* reported for natural samples. In this study, the detection limits are defined as 2× the blank before it was subtracted from the sample.

b This study.

c Determined using cell abundances.

d Determined in the subtropical North Atlantic Ocean (3).

e Determined in the subtropical and tropical Northeast Atlantic Ocean (4).

f Calculated on the basis of the reported data. Determined in the South Pacific Ocean (11).

g Determined in the Atlantic Ocean (14).

*Prochlorococcus* represented 2.9% ± 1.3% of the glucose assimilation by the whole water communities (*n* = 20), with 3.4% ± 1.4% during daylight (*n* = 12) and 2.1% ± 0.8% during the night (*n* = 8) (Table 1). On average, the assimilation of carbon from glucose by the whole microbial community represented 0.6% ± 0.2% of the carbon fixed by primary production. The assimilation of carbon from glucose by *Prochlorococcus* represented circa 0.05% ± 0.02% of their carbon fixed by primary production.

### Glucose effect on the bacterial community composition: 16S rRNA sequences.

We measured shifts in microbial community composition following glucose enrichment based on 16S rRNA gene tag sequencing. In all samples, the total and picoplankton communities were dominated by *Alphaproteobacteria* (mean of 40% of total sequences in each sample versus 3% for all other *Proteobacteria* combined), in particular by the SAR11 clade, and cyanobacteria (38%), mainly *Prochlorococcus* (36%) (Fig. S2A and supplemental material). Amplicon sequence variants (ASVs) of an unknown phylum were rare (<0.01%) (Fig. S2A). The remaining ASVs were mainly from *Bacteroidetes* (*Flavobacteria*), *Actinobacteria* (the OM-1 clade bacteria like “*Candidatus* Actinomarina”), *Planctomycetes*, and *Euryarchaeota* (Fig. S2A and supplemental material).

There were no large shifts in the relative abundances of these taxa in response to glucose amendments over the 24-h incubation period based on nonmetric multidimensional scaling (NMDS) and permutational multivariate analysis of variance (PERMANOVA) of Bray-Curtis distances between samples (supplemental material). Thirty-three ASVs had significant (*P* < 0.05) changes in abundance in response to glucose addition, but they were rare community members in each sample (<0.1%) (supplemental material, Fig. S2B, and Table S2B).

### Transcription of photosystem I and C fixation genes in high- and low-light *Prochlorococcus* ecotypes.

The microarray targeted 1,200 genes from the dominant *Prochlorococcus* strains at Station ALOHA (Table S3). On average, 53% (637 genes) were transcribed at detectable levels in each sample and 65% (775 genes) across all samples (Table S4A). Thus, the population was transcriptionally active over the diverse set of metabolic pathways represented in the microarray (Tables S3 and S4A). Indeed, on average 64% ± 33% (mean ± SD) of genes were detected for each of the 34 strains represented on the microarray, with more genes detected for high-light (HL) strains (84% ± 13%; *n* = 24) than for low-light (LL) strains (18% ± 9%; *n* = 10) (Table S4B). Within each sample, most transcripts belonged to HL adapted ecotypes from unknown clades (HOT208_60m_813O14, HOT208_60m_813G15), followed by clades II (AS9606, MIT0604) and I (MIT9515, MED4) (Fig. S3A). LL strains were also detected in every sample, primarily from clades I (PAC1, NATL1/2) and II/III (SS120, MIT0602) and less often from clade IV (MIT9313, MIT0303) (Table S4B). The only strain not detected was the LL strain MIT0603, which has only 2 genes represented on the microarray compared to 36 genes on average for other strains (Table S4B). Generally, we observed higher transcript levels for HL strains than for LL strains in the array (Fig. S3B).

Across all ecotypes, photosystem I (PSI) and C fixation pathways were highly transcribed in all samples (Fig. S3C), and notably transcription was higher for HL clade I than for other HL clades. In contrast, LL clade I had higher levels of transcription for PSI and respiration but lower levels for C fixation than other LL members (Fig. S3C).

### *Prochlorococcus* expression patterns upon glucose addition.

**FIG 2:**
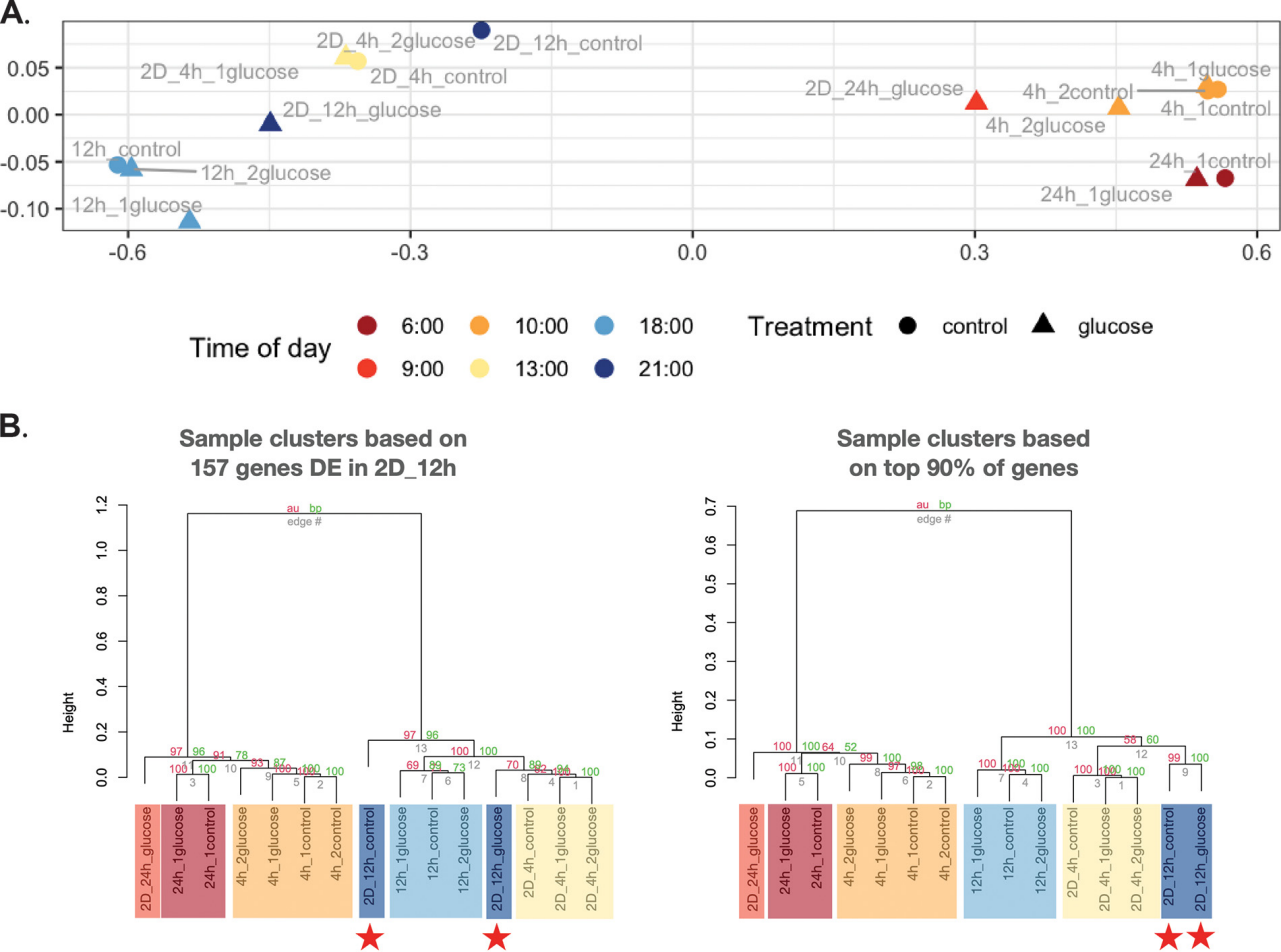
(A) NMDS of *Prochlorococcus* metatranscriptomes. For each of the 15 samples, the metatranscriptome consisted of the transcript levels (log_2_ normalized) of the top 90% of genes. A 2-dimensional NMDS was performed on Euclidean distances calculated over 1 − the Pearson correlation matrix for the sample metatranscriptomes. Stress was ~0. In the legend, the time of day indicates when the mRNA was fixed. Samples cluster primarily by day (*x *>* *0) and night (*x *<* *0) and secondarily by time of day regardless of glucose or control. (B) Sample dendrograms based on the genes that were differentially expressed (DE) in the 2D_12h glucose samples versus control (left) and based on the top 90% of genes used in the NMDS analysis (right). For both dendrograms, correlation-based Euclidean distances were calculated between samples, followed by Ward D2 clustering. Ten thousand bootstraps were performed, and approximate unbiased *P* values were calculated for the clusters (red percentages indicate significant if >95%) as well as traditional bootstrap support (green percentages), using the approach of Suzuki and Shimodaira (70) implemented in the R pvclust package. As in the NMDS, samples cluster first by day versus night and then by time of day. The exceptions are the 2D_12h samples (indicated by red stars) in the left dendrogram, which suggests a disruption in diel expression patterns for the 157 genes that were DE in 2D_12h.

*Prochlorococcus* metatranscriptomes clustered primarily by the hour of the day, which suggests that overall gene expression was influenced more by diel cycles than by glucose addition (Fig. 2A and Fig. S4). For example, most of the genes with higher transcription levels during the day and lower during the night were related to carbon fixation (*rbcSL*), energy metabolism (*atpA*), and photosynthesis (*psaAB*) (Fig. S4). In contrast, higher transcription values during the night and lower values during the day were observed in pathways such as respiration (*coxA*/*cyoBC*) and pentose phosphate (*tal*, *zwf*). Moreover, we observed ammonia assimilation (*glnA*) peaking in the evening and the circadian rhythm (*kaiB*) with a maximum at dawn (Fig. S4).

For most incubations, glucose treatments and matched controls had similar metatranscriptomes in the NMDS analysis. The proportions of transcripts from each of the clades remained stable over the incubations from 4 to 24 h (Fig. S3A). Hence, we interpret differences in metatranscriptome positions in the NMDS between glucose treatments and matched controls as responses to glucose amendment. For the 4-h incubations that terminated in the day (10:00 or 13:00), differences were small. We suspect that 4-h incubation was too short to observe a transcriptional response to glucose because larger metatranscriptome changes occurred in most of the incubations longer than 4 h. For example, metatranscriptome shifts were apparent in the NMDS for two of the three 12-h incubations that terminated at night (2D_12h_glucose at 21:00 and 12h_1glucose at 18:00) and for the 24-h incubation that terminated in daylight (2D_24h_glucose at 9:00) in comparison to 4-h incubations that also terminated in the morning (10:00 or 13:00) (Fig. 2A).

**FIG 3:**
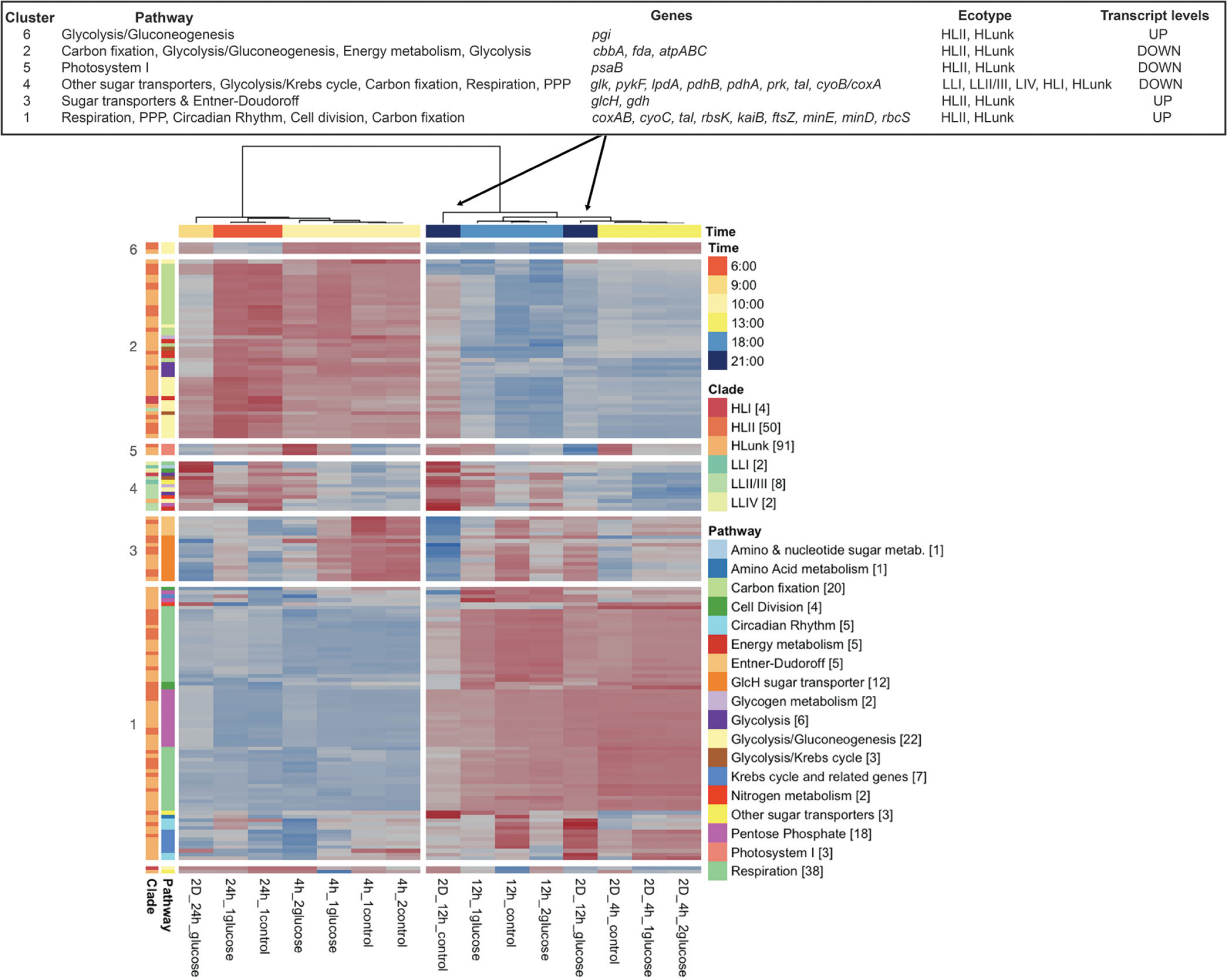
Heat map showing the 157 *Prochlorococcus* genes that were DE in 2D_12h glucose versus control (marked by arrows). Samples were hierarchically clustered (70) based on the Euclidean distances between 1 − their Pearson correlations of the log_2_ transcript levels for the 157 DE genes. The two main sample clusters were significant (both had 97% support using multistep-multiscale bootstrap resampling with 10,000 bootstraps) (69, 71). Genes (rows) had their log_2_ transcript levels standardized (mean = 0, SD = 1) prior to gene hierarchical clustering. Thus, transcription intensities (blue-red scale) should only be compared genewise, having lower transcription levels in blue or higher levels in red. Genes were hierarchically clustered using the same approach as the samples, and eight significant clusters were identified (>95% support). The bottom three genes are not members of the clusters. Row-side annotations include the numbers of DE genes from the category in brackets. A summary table showing the main changes between 2D_12h_control and 2D_12h_glucose is located in the upper panel.

**FIG 4:**
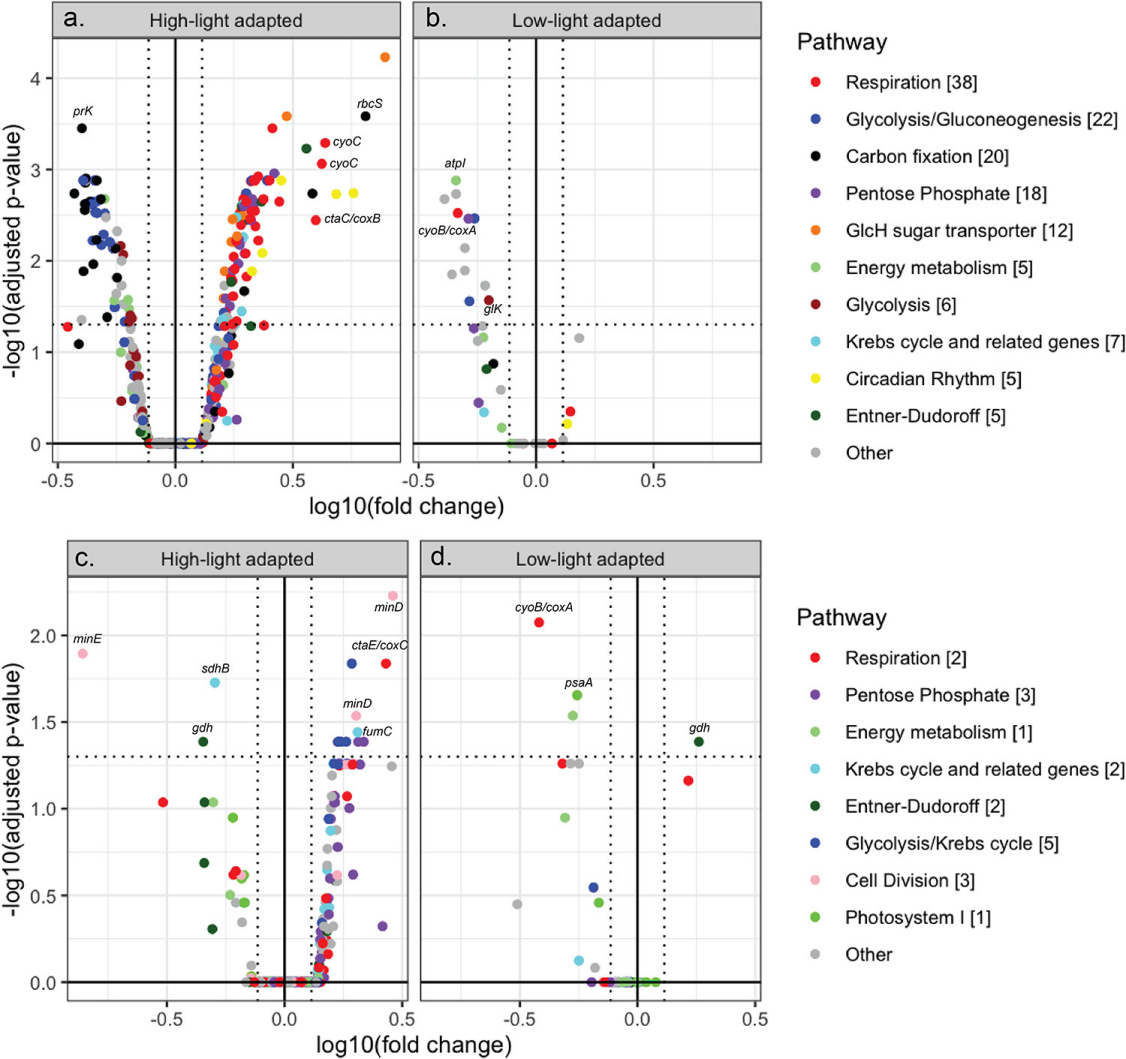
Significantly differentially expressed (DE) genes in 2D_12h and 24h_glucose samples shown (separately) for high-light (a and c) and low-light (b and d) adapted *Prochlorococcus*. (Top panels) A total of 157 DE genes from *Prochlorococcus* were identified in 2D_12h, the 12-h incubation that terminated at 21:00. (Bottom panels) A total of 19 DE genes from *Prochlorococcus* were identified in 24h_glucose, the 24-h incubation that terminated in daylight at 09:00. The pathways and counts for the DE genes are in the legend. The DE genes are in the upper left and right sections of each plot, delimited by vertical dotted lines for fold changes of >1.3 and a horizontal dotted line for adjusted *P* values of <0.05. Genes outside these sections were detected but not DE. A few key genes with the biggest changes have been shown on the graph.

A total of 174 genes were significantly differentially expressed (DE) in response to glucose (Table S4C). HL *Prochlorococcus* had the majority of DE genes in 2D_12h (145 of 157 genes; Table S4C). Sample metatranscriptomes clustered mainly by time of the day in an NMDS analysis, which used most detected genes, as well as in an analysis that used only the 157 DE genes in 2D_12h (Fig. S4B). However, the 2D_12h samples did not cluster together: rather, glucose addition resulted in transcript levels at 21:00 that were similar to those in the 2D_4h incubations at 13:00 (Fig. 2B, left panel). This suggests that diel expression patterns were maintained for most incubations but were perturbed by glucose addition in 2D_12h. We visualized the response in a heat map (see Fig. 3 below). For HL *Prochlorococcus*, the sample clusters were distinguished by pathways (mentioned earlier) that responded to glucose: elevated transcript levels for respiration (*coxAB*, *cyoC*), the pentose phosphate pathway (*tal*, *rbsK*) (gene cluster 1), sugar transporters (*glcH*) and the Entner-Doudoroff pathway (*gdh*) (gene cluster 3), and decreased transcript levels for C fixation (*prk*), glycolysis (*cbbA*, *pykF*), and energy metabolism (*atpABC*) (gene cluster 2). A gene set enrichment analysis corroborated the HL increases for respiration, the pentose phosphate pathway, and glucose transporter genes and the decreases for glycolysis genes (supplemental material). Intriguingly, 5 *kaiB* genes were DE with increases from 2.1- to 5.7-fold, and all of them (circadian rhythm genes in cluster 1, HLI, and HLunk) had their highest levels in 2D_12h_glucose. LL *Prochlorococcus* had only 12 DE genes, which mainly decreased in response to glucose amendment, including one *tal* gene (pentose phosphate) and one *coxA* gene (respiration), whereas DE genes in these two pathways only increased for HL strains (in gene cluster 1) (Fig. 3, Fig. 4b, and Table S4C).

Most of the 19 DE genes identified in the 24-h incubations that terminated in the light at 06:00 (24h_glucose versus 24h_control) belong to pathways related to glucose metabolism (Fig. 4c and d and Table S4C). For HL, increases occurred for respiration (*coxC*), the pentose phosphate pathway (*opcA*), and pyruvate metabolism (*pdhB*), as well as for cell division (*minD*) (Fig. 4c and Table S4C). The respiration increases were corroborated by the gene set enrichment analysis (supplemental material). Few genes were DE for LL, but they included *coxA*, which decreased, in contrast to strict increases for HL respiration genes (also in 2D_12h) (Fig. 4d and Table S4C).

Only 1 DE gene was identified in the 4-h incubations that terminated in the light at 13:00 (2D_4h_glucose versus 2D_4h_control), *pdhA* (encoding pyruvate dehydrogenase) from the LLI strain NATL2, which decreased in the presence of glucose (Table S4C).

## DISCUSSION

### *Prochlorococcus* carbon assimilation.

*Prochlorococcus* abundances showed a clear diel cycle with increases during the day and decreases during the latter half of the night as described in previous works (18, 19) (see Fig. S1B in the supplemental material). C assimilation by *Prochlorococcus* determined using [^3^H]Glc and [^14^C]sodium bicarbonate showed a similar diel pattern. As expected, natural *Prochlorococcus* populations showed a pronounced diel cycle in carbon fixation, with undetectable values at night and a maximum of 13.1 ± 8.0 nmol C L^−1^ h^−1^ (or 1.23 ± 0.67 fg C cell^−1^ h^−1^) fixed between noon and 16:00 (Fig. 1b and Table 2). Similar per cell rates of carbon fixation by *Prochlorococcus* have been measured in the upper euphotic zone at Station ALOHA and in the Atlantic Ocean (3, 4, 8, 20–22) (Table 2).

An interesting aspect of our results was the fact that *Prochlorococcus* showed a clear diel pattern in glucose assimilation, with maximum values during the day (~2-fold change) (Fig. 1d and Table 2). However, a contrasting diel pattern was observed for the whole water communities, with higher values from midnight to early morning and low values at sunset (~2-fold change). Previous studies, carried out in the Pacific and Atlantic Oceans, showed similar per cell rates in daylight incubations (11, 14). Light stimulates the cyanobacterial assimilation of amino acid (8, 11, 23–26), DMSP (27, 28), and ATP (11, 25, 29); this has also been observed for the assimilation of glucose in natural populations of *Prochlorococcus* (11), where it is an active process (13, 15). However, this is the first study showing that glucose assimilation in natural *Prochlorococcus* populations follows a diel pattern; previous studies have shown data related to light/darkness, but in laboratory studies and never in diel experiments.

The fact that *Prochlorococcus* glucose assimilation rates peak during the light period, while rates in the whole water communities peak during the night to early morning, could provide *Prochlorococcus* some advantages over the rest of the community. One of the advantages is the coupling of the energy produced by photosynthesis to glucose assimilation, since it is actively transported (15). This is reflected in the increased percentage of the total glucose assimilation by *Prochlorococcus* during daylight versus night (from 2.1% in the night to 3.4% in the day, a ca. 60% increase) (Table 1). We hypothesize that *Prochlorococcus* is using glucose when it is available during the light period but also during the dark period, being important by phytoplankton adapted to living at the base of the photic layer, when photosynthesiss cannot provide ATP (17). However, we propose glucose uptake is more efficient when *Prochlorococcus* is using the sunlight energy during the day, saving energy involved in synthesizing and maintaining the transporters.

Moreover, coupling light availability with cellular processes would facilitate adaptation to daily environmental changes (30). *Prochlorococcus* could thus be using some of the sugars that are lost by other microorganisms through death and sloppy feeding by zooplankton during the day (coevolved mutualism) (31). Finally, the different timing of glucose assimilation compared to the total population may also offer considerable fitness advantages over competitors in “temporal niches” (32). In this way, *Prochlorococcus* could scavenge more efficiently glucose than if it was showing a glucose uptake maximum synchronized with the other bacteria, avoiding part of the overwhelming competition. Furthermore, since GlcH shows a multiphasic kinetics (14), depending on the environmental concentration of glucose, this transporter could shift from one phase to the other. When there is higher glucose concentration, it will use the phase with lower affinity for glucose but higher *V*_max_; when there is very low glucose, it will shift to the phase with higher affinity for glucose but lower *V*_max_. Therefore, we propose that the differential diel pattern of glucose uptake between *Prochlorococcus* and the rest of bacteria and also the multiphasic kinetic of this transporter could help *Prochlorococcus* to avoid the competition for the glucose and maximize its uptake efficiency.

Interestingly, previous studies on the diel rhythmicity of amino acid assimilation by *Prochlorococcus* in surface areas of the Atlantic Ocean showed maximal assimilation values at the beginning of the dark period and minimal values around midday (33); this is almost exactly the opposite rhythm that we found for glucose assimilation in *Prochlorococcus*. This contrast is striking, especially if we consider that both amino acid and glucose assimilations are active processes stimulated by light. A possible explanation for the difference might be based on the fact that amino acids are an important source of N in oligotrophic environments; since N is an essential element for the production of many cell compounds required before division, a maximum of amino acid assimilation at the beginning of the dark period might boost protein synthesis prior to *Prochlorococcus* cell division, as proposed by Mary and coworkers (33). In contrast, glucose can be directly used for general metabolic needs in *Prochlorococcus* (13), and therefore it would be more efficient to take up most glucose at midday, coupling the energy consumed by this process to the light photosynthetic reactions. Regardless of the difference of rhythms between glucose and amino acid assimilation in *Prochlorococcus*, the results show that not all light-stimulated assimilation processes are regulated the same way in marine picocyanobacteria.

Ambient glucose concentrations in surface seawater at Station ALOHA showed an average value of 1.1 ± 0.1 nmol L^−1^ (*n* = 4), similar to concentrations previously reported (11, 14, 34). Based on these estimates, *Prochlorococcus* contributed approximately 3.4% ± 1.4% (*n* = 12) of the total glucose assimilation in surface seawater during the light period, very similar to previous reports from the North Atlantic Ocean (2.6 to 3.7%) (14) and the Western tropical South Pacific Ocean (~5%) (11). Glucose assimilation by *Prochlorococcus* at the surface represented a small fraction (<1%) of its total (inorganic plus organic) C assimilation, similar to values previously reported (11, 14) (Table 1). It is worth noting that in a previous study carried out in the Atlantic Ocean (14), the percentage of total glucose assimilation assigned to *Prochlorococcus* was overestimated due to errors in the calculation. The corrected data show that *Prochlorococcus* glucose assimilation in the Atlantic Ocean was also lower than 1% of the total C assimilation.

While [^14^C]Glc would have been a better tracer of C assimilation from glucose, due to low specific activity of the ^14^C-radiolabeled compounds, *Prochlorococcus* cell-specific assimilation rates determined from the [^14^C]Glc experiments were below the detection limit. Still, the fact that bulk glucose assimilation rates were nearly 2 times higher using ^14^C-labeled compared to ^3^H-labeled Glc indicates that we likely underestimated the C uptake when using a ^3^H-labeled glucose. Using [^3^H]Glc could lead to underestimation of the C fraction assimilated from glucose due to a possible loss of ^3^H in exchange reactions with H_2_O or the fact that assimilated ^3^H can create problems of self-absorption (35, 36). It should be noted that the contribution of glucose assimilation to *Prochlorococcus* metabolism could be underestimated if glucose is being used for energy rather than for biosynthesis. Furthermore, glucose is one of the pool of diverse dissolved organic C molecules present in the ocean (37, 38) that *Prochlorococcus* might be able to use (14, 39), which could make the contribution of organic C to the total C assimilation much higher.

Cell-specific glucose assimilation by *Synechococcus* was previously determined in the Western Tropical South Pacific Ocean and was higher than that of *Prochlorococcus* (4.3 ×10^−4^ fg C cell^−1^ h^−1^), likely attributable to *Synechococcus* having a larger biomass (11). In the present study, the *Synechoccocus* population was also sorted after [^3^H]Glc incubation, but due to the low *Synechococcus* abundances at Station ALOHA (~100-fold lower cell concentration than *Prochlorococcus*), results were not significantly different from the blanks. Still, it is possible that *Prochlorococcus* and *Synechococcus* compete for glucose. Experiments performed in laboratory cultures revealed that glucose transport in *Prochlorococcus* and *Synechococcus* displays multiphasic kinetics with high efficiency (calculated by dividing the assimilation rate by the *K_s_* constant, between 0.01 and 20 μmol L^−1^) (15). A comparison of the assimilation efficiency demonstrated *Prochlorococcus* to be 7 times more efficient than *Synechococcus* (15), which could be an advantage for *Prochlorococcus* in oligotrophic areas where *Prochlorococcus* spp. coexist with *Synechococcus*, such as Station ALOHA.

### Effects of glucose on *Prochlorococcus* metabolism.

As anticipated from previous studies, the SAR11 clade (*Proteobacteria*) and *Prochlorococcus* were highly abundant in all surface samples at Station ALOHA, followed by *Bacteroidetes* and *Actinobacteria* (40, 41).

Our results did not show differences in community composition after glucose enrichment (Fig. S2A). It is possible that the incubation duration was too short to see changes in the microbial community or that the glucose concentration was too low to induce changes during the short incubation period. Higher abundance of *Prochlorococcus* upon addition of glucose and mannitol was observed in oligotrophic areas of the South Pacific (39); however, the authors used a 4,000-fold-higher glucose concentration and longer incubation times than we did (400 μM and 78-h maximum versus the 0.1 μM and 24-h maximum used in our study).

The population showed active transcription over the diverse metabolic pathways of the 33 strains identified. Many of the same *Prochlorococcus* strains detected in our results were also found in a previous study using Agilent microarrays at Station ALOHA (42). In both studies, photosynthesis and carbon fixation genes have been the most highly transcribed across all taxa and samples (this study and reference 42).

Generally, we observed higher percentages of detected genes and higher transcript levels for HL strains than for LL strains (Table S4B and Fig. S3B). A previous study at Station ALOHA also found much smaller proportions of LL clade transcripts relative to HL clades of *Prochlorococcus* at the surface (43). Our results might suggest that either HL strains had higher relative cell abundances (consistent with the fact that samples were collected from the surface in our work), were transcriptionally more active (44, 45), or both.

Moreover, we found differences in the transcripts across the clades of HL and LL ecotypes, with high transcription levels in PSI and C fixation pathways in HLI and LLI clades. As discussed above, these values might be related to the cell abundances of these clades: in fact, a relatively high contribution of *Prochlorococcus* HLI and LLI in this North Pacific region has been observed previously in surface waters (46–49). LLI strains are usually restricted to deeper depths at Station ALOHA when the water column is stratified; however, contrary to other clades, LLI strains are present in the euphotic zone in mixed water (46).

*Prochlorococcus* strains showed the majority of the transcriptional changes 12 h and 24 h after glucose enrichment (Fig. 4). Only one gene responded significantly in a 4-h incubation (*pdhA* from NATL2 in experiment 2), which suggests that in most cases, *Prochlorococcus* might require between 12 and 24 h from the moment that glucose is taken up until the transcriptional response of the glucose metabolism is detectable. Moreover, the surface light flux in the second experiment averaged 42.1 E m^−2^ day^−1^ versus 33 E m^−2^ day^−1^ during the first experiment (Table S1B), which could explain why most changes were observed during the second experiment, where higher light could have stimulated the glucose assimilation.

Clustering of the transcriptomic data showed a striking result (Fig. 2B, left): a glucose-amended sample taken at 21:00 h (night) grouped with unamended samples taken at 13:00 h (midday). This suggests that *Prochlorococcus* has a transcriptomic response to glucose addition that is similar to that of samples with no glucose in the middle of the day; in other words, increased glucose availability in the night supports a metabolic response that somehow mimics that of cells growing in the light.

A total of 174 genes were significantly DE in response to glucose after 12-h or 24-h incubations (Fig. 4 and Table S4C). The effect of glucose enrichment on the transcriptome of HL strains showed increases for respiration (*coxAB*, *cyoC*), the pentose phosphate pathway (*tal*, *rbsK*), the Entner-Doudoroff pathway (*gdh*), glycolysis (*pgi*), glucose transport (*glcH*), the Krebs cycle (*fumC*), pyruvate metabolism (*pdhB*), and cell division (*minD*). It has been proposed that *Prochlorococcus* might use two pathways to metabolize glucose—the Entner-Doudoroff and pentose phosphate pathways (12, 13, 15, 50)—and small changes in gene expression and quantitative proteomics have been demonstrated upon glucose addition to the culture media (13, 15). These previous studies showed results from the expression of a few genes in a single strain, carried out by quantitative reverse transcription-PCR (qRT-PCR) in the laboratory. Our results show, for the first time in natural populations at the HOT station, that *Prochlorococcus* could utilize glucose by both pathways (the Entner-Doudoroff, and pentose phosphate pathways) using microarrays to study the expression of many genes in several strains.

Changes in the expression of a glycolytic enzyme (phosphoglucose isomerase [*pgi*]) were also observed as previously reported (15). Glycolysis is not active since *Prochlorococcus* lacks phosphofructokinase (50). However, even if it lacks the enzymes involved in the initial steps of glycolysis, this cyanobacterium still has a few genes which could be involved in glucose assimilation with the production of reducing equivalents and/or the production of ATP as a result of the metabolization of glyceraldehyde 3-phosphate and phosphoenolpyruvate (13, 50).

Periodicities of the transcripts of genes involved in physiological processes, such as carbon fixation, energy metabolism, photosynthesis, respiration, pentose phosphate, and circadian rhythm, tracked the timing of its activities relative to the light-dark cycle as previously described (51). We observed high *glcH* transcription levels during the day at 13:00; however, the maximum transcript level was observed during the night at 21:00 (~8-fold change) (supplemental material and Table S4C). The highest transcriptional changes of *glcH* during the night could indicate the synthesis of the glucose transporter in order to be ready during the day, when light is stimulating the glucose assimilation according to the diel pattern.

An interesting result for HL strains (HLII and HLunk) was that glucose addition led to transcript level increases for the circadian gene *kaiB* (2.1- to 5.7-fold) at 21:00. Transcriptomic changes in *kaiB* might affect diel expression patterns, which could explain the similarity of the metabolic response of samples with glucose addition to those with no glucose in the middle of the day described above. In fact, several studies have shown that circadian clocks are connected to cyanobacterial metabolism (52–54).

Finally, we observed differences in the transcription profiles between the different ecotypes. Few genes were DE in LL *Prochlorococcus* strains, but those that were DE showed different patterns after glucose addition compared to HL strains. In fact, the LL ecotype was clustered in the heat map as an independent gene group (gene cluster 4). Genes from LL clades involved in pentose phosphate and respiration decreased after glucose addition, whereas genes in the same pathways increased in HL strains after 12 and 24 h of glucose addition (Fig. 4b and d). Other studies have also found that coexisting *Prochlorococcus* populations respond differently upon addition of nutrients (55). Subpopulations with different abilities to utilize glucose even at the same depth could be explained by many factors like competition, environmental conditions, or genetics. In fact, there is a diversity of kinetics in glucose assimilation in the *Prochlorococcus* strains (15), which could suggest that the glucose assimilation has been subjected to diversification along *Prochlorococcus* evolution.

Overall, our results indicated that *Prochlorococcus* shows synchronous timing in gene expression and in glucose assimilation presumably coupled to the light cycle. Diurnal glucose assimilation could allow *Prochlorococcus* to optimize glucose assimilation by using ATP made during the light period, coupling this process to photosynthesis. Furthermore, it also could provide some advantages over the rest of the community, which showed a different timing for glucose assimilation.

The relative contributions of the different metabolic pathways to metabolize glucose in different subpopulations of *Prochlorococcus* and the possibility that glucose could induce changes in the transcriptional rhythms should be further investigated to understand the impact of mixotrophy on the marine cyanobacterial populations and their consequences for global biogeochemical cycles.

## MATERIALS AND METHODS

### Field sampling.

Seawater for all incubation experiments was collected at Station ALOHA (22°45′N, 158°00′W) in the NPSG during the KM1715 (HOT 296) cruise on 5 to 9 October 2017 (see Table S1A in the supplemental material). Seawater was sampled from a depth of 7 m using the R/V *Kilo Moana*’s uncontaminated seawater system (https://www.soest.hawaii.edu/UMC/cms/KiloMoana.php). The surface light flux values measured during the experiments are shown in Table S1B.

### Incubation experiments with radiolabeled substrates.

Separate incubation experiments were carried out to determine primary production, using [^14^C]sodium bicarbonate (MP Biomedical product no. 117441H; specific activity, 2.22 TBq mmol^−1^), and the turnover and assimilation of glucose by *Prochlorococcus* and the whole microbial community, using both [^14^C]glucose ([^14^C]Glc) and [^3^H]Glc {Perkin-Elmer product no. NEC042X, glucose, d-[^14^C(U)], specific activity, 9.7 GBq mmol^−1^, 97% radiochemical purity; Perkin-Elmer product no. NET100C, glucose, d-[6-^3^H(N)], specific activity, 1.83 TBq mmol^−1^, 97% radiochemical purity}. Radiolabeled glucose was also used to estimate the ambient glucose concentration in the seawater. These incubations were performed in 60-mL or 100-mL clear polycarbonate bottles that had been acid cleaned and rinsed with ultrapure water before being rinsed with sample seawater. The seawater samples were spiked with the relevant radioisotope and incubated in on-deck incubators with surface seawater cooling and blue shielding at 60% of full surface light to simulate a 5-m depth.

### Estimation of ambient glucose concentrations.

To estimate the ambient concentration of glucose in surface seawaters at Station ALOHA, concentration series bioassays were carried out on the HOT 295, 296, and 298 cruises (September, October, and December 2017, respectively) following the procedure described by Wright and Hobbie (56) and modified by Zubkov and Tarran (57). Further details of the bioassay method have been described by Zubkov et al. (34). Additional details are provided in the supplemental material.

### Diel assimilation.

Seawater samples were collected every 6 h for a total of 10 samplings over a 54-h period (Table S1A). Duplicate 60-mL bottles were spiked with radiolabeled glucose to a target addition of 2 nM glucose or with [^14^C]sodium bicarbonate (final activity of ~150 MBq L^−1^). Each sample set included a paraformaldehyde-killed control (0.24% final concentration) that was incubated alongside the live samples. The diel samples were incubated for 4 h and terminated by adding paraformaldehyde (0.24% final concentration) to stop the assimilation of the radiolabel and preserve the sample. The primary production incubations were treated the same way as the glucose incubations during daylight hours, but these samples were kept in the incubator overnight (from 18:30 to 04:00), under the assumption that primary productivity during the night would be negligible. At the end of the incubation period, 5-mL subsamples from each incubation were filtered onto 0.2-μm-pore polycarbonate filters, rinsed with filtered seawater, and placed either into plastic scintillation vials (Simport SnapTwist scintillation vials; 7 mL) for glucose or into 20-mL borosilicate scintillation vials for [^14^C]sodium bicarbonate. The latter were acidified (1 mL 2N HCl) and vented for 24 h before the addition of scintillation cocktail. The total radioactivity subsamples for [^14^C]bicarbonate were trapped with β-phenethylamine (Sigma-Aldrich no. 407267). Ultima Gold LLT (Perkin Elmer) was used as the scintillation cocktail for all radioassays in a Packard Tri-Carb liquid scintillation counter. Dissolved inorganic carbon concentrations for HOT 296 were from the HOT Data Organization and Graphical System (http://hahana.soest.hawaii.edu/hot/hot-dogs), and a correction of 1.06 for preferential assimilation of ^12^C relative to ^14^C was applied (58).

Subsamples for flow cytometric cell counts were taken in duplicate 2-mL samples, fixed with paraformaldehyde (0.24% final concentration), and incubated for 15 min in the dark before being flash frozen, and then were stored at −80°C until further laboratory-based processing. The remaining sample volume from each incubation was concentrated as described by Duhamel et al. (3, 11) for cell sorting to determine cell- and group-specific assimilation of glucose and inorganic carbon by *Prochlorococcus*. Further details of the flow cytometry and cell sorting method are described in the supplemental material.

### Incubation experiments with glucose for genomics-based approaches.

For molecular approaches, seawater was collected into 2-L polycarbonate bottles in two technical replicates for control and for glucose-amended samples (Table S1A and B). The glucose treatments were spiked with 0.1 μM nonradiolabeled glucose and incubated alongside the controls (unamended) in the on-deck incubators.

For the first experiment (6 October), surface seawater was sampled at 06:20, while for the second experiment (7 October) surface seawater was collected at 09:20 (Table S1A and B). The same bottles were used for both experiments.

Samples were collected for these two experiments, and for each one, two technical replicates were collected at 3 different time points: after 4 h, 12 h, and 24 h of incubation in the presence of glucose or in the control treatment (Table S1B). Different bottles with the same treatment and collected at the same time were considered technical replicates. The first and second experiments were considered biological replicates. Therefore, samples are identified as 4h_glucose, 4h_control, 12h_glucose, 12h_control, 24h_glucose, 24h_control, 2D_4h_glucose, 2D_4h_control, 2D_12h_glucose, 2D_12h_control, 2D_24h_glucose, and 2D_24h_control, with the second experiment identified as “2D,” and are considered biological replicates. The different technical replicates are labeled with the number 1 or 2 before the treatment: e.g., 4h_1glucose and 4h_2glucose. Sufficient environmental RNA was obtained for two technical replicates in 4 samplings (4h_1control, 4h_2control, 4h_1glucose, 4h_2glucose, 12h_1glucose, 12h_2glucose, 2D_4h_1glucose, and 2D_4h_2glucose).

At each sampling time point, RNA and DNA samples were collected by filtering 4 L and 1 L of seawater, respectively, onto 0.22-μm-pore-size Sterivex cartridges (Millipore Corp., Billerica, MA, USA) using a peristaltic pump set at low rate to maintain low pressure. Filters were opened and carefully placed in sterile 2-mL bead-beating tubes with sterile glass beads and stored at −80°C until extraction. Further details of the incubation experiments are described in Table S1A and B.

### DNA extraction.

DNA extractions were carried out with a modification of the Qiagen DNeasy plant kit (59). Briefly, 400 μL lysis buffer (AP1 buffer) was added to the bead-beating tubes, followed by three sequential freeze-thaw cycles using liquid nitrogen and a 65°C water bath. The tubes were agitated for 2 min with a Vortex-Genie 2 bead beater (Scientific Industries, Inc.) and incubated for 1 h at 55°C with 20 mg mL^−1^ proteinase K (Qiagen). Samples were treated for 10 min at 65°C with 4 μL RNase A (100 mg mL^−1^), and then the filters were removed using sterile needles. The tubes were centrifuged for 5 min at 16,873 × *g* at 4°C, and the supernatant was further purified using the manufacturer’s protocol (Qiagen). Samples were eluted using 100 μL of the elution buffer (AE buffer) and stored at 20°C.

Sufficient environmental DNA was obtained for two technical replicates in only 5 samples (2D_4h_glucose, 2D_12h_glucose, 2D_12h_control, 2D_24h_glucose, 2D_24h_glucose), and those samples are identified as 2D_4h_1glucose, 2D_4h_2glucose, 2D_12h_1glucose, 2D_12h_2glucose, 2D_12h_1control, 2D_12h_2control, 2D_24h_1glucose, 2D_24h_2glucose, 2D_24h_1glucose, and 2D_24h_2glucose.

### Sequence processing.

V3 and V4 regions of 16S rRNA genes were amplified, sequenced, and analyzed by the STAB-VIDA Company (Lisbon, Portugal) using the following primers (60): forward primer S-d-Bact-0341-b-S-17 (5′-CCTACGGGNGGCWGCAG-3′) and reverse primer S-d-Bact-0785-a-A-21 (5′-GACTACHVGGGTATCTAATCC-3′). DNA samples were checked for quantity and integrity by 1% agarose gel electrophoresis, a Qubit fluorometer (Thermo Fisher Scientific, MA, USA), and a 2100 Bioanalyzer (Agilent Technologies, Santa Clara, CA, USA) prior to library construction using the Illumina 16S Metagenomic Sequencing Library Preparation protocol (61). The generated DNA fragments (DNA libraries) were sequenced with the lllumina MiSeq platform using MiSeq reagent kit v2 to produce paired-end sequencing reads (2 × 250 bp). FastQC (62) was used to inspect the quality of the raw sequencing reads. The analysis of the generated raw sequence data was carried out using QIIME2 v2018.2 (63). The QIIME2 plugin for DADA2 (denoise paired) (64) was used to process the raw reads into amplicon sequence variants (ASVs), which provide higher phylogenetic resolution than operational taxonomic units (OTUs). Reads were trimmed of primers at the 5′ end using the primer lengths and truncated at the 3′ end so that total lengths were 250 bp (R1), and 235 bp (R2). Reads were removed if they had Phred quality scores of <20 on average, <17 for two consecutive bases, or >2 expected errors. Quality-filtered reads were then dereplicated, denoised (ASV inference using the core DADA2 algorithm), merged, and filtered for chimeras.

The 1534 ASVs identified by DADA2 were additionally filtered for chimeras using the uchime3_denovo algorithm implemented in vsearch v2.13.3 (65), which identified 53 chimeras, and the NCBI 16S rRNA chimera detection pipeline based on uchime2_ref (66), which identified 452 chimeras. One of the 452 chimeras (ASV.348) was retained because it was observed in 12 samples (198 sequences total) and had taxonomically consistent parents (genus *Coxiella*). From the 1,030 nonchimeric ASVs, we removed 231 ASVs with ≤10 total sequences and another 81 ASVs that were detected in only 1 sample with <30 sequences to produce a final set of 718 ASVs (Table S2A). The ASVs were classified by taxon using a QIIME2 scikit-learn fitted classifier that had been trained on the SILVA database (release 128 QIIME) clustered at 97% similarity (Table S2A).

### RNA extraction and processing for hybridization to the microarray.

Environmental RNA containing transcripts from *Prochlorococcus* cells was extracted using an Ambion RiboPure Bacteria kit (Ambion, Thermo Fisher), with modifications that included mechanical lysis using glass beads (Biospec, Bartlesville, OK). The extracted RNA was treated with a Turbo-DNA-free DNase kit (Ambion, Thermo Fisher) to remove genomic DNA.

RNA concentration, purity, and quality were determined using a NanoDrop 1000 instrument (Thermo Scientific, Waltham, MA, USA), a 2100 Bioanalyzer (Agilent Technologies, Santa Clara, CA, USA), and an RNA 6000 Nano kit (Agilent Technologies). Only samples with RNA integrity values of >7.0 and *A*_260_/*A*_230_ and A_260_/A_280_ ratios of ≥1.8 were processed further. Double-stranded cDNA (ds-cDNA) was synthesized from environmental RNA samples and amplified following the procedure previously described by Shilova et al. (42). Briefly, 400 ng RNA from each sample was used, and 1 μL of a 1:100 dilution (corresponding to 4.7 aM ERCC-0016) of RNA spike-in mix 1 (External RNA Control Consortium; Ambion) (67) was added before amplification was performed to monitor the technical performance of the assay showing linear amplification of specific probes (67). ds-cDNA was synthesized and amplified using a TransPlex whole-transcriptome amplification kit (WTA-2; Sigma-Aldrich, St. Louis, MO, USA) and antibody-inactivated hot-start *Taq* DNA polymerase (Sigma-Aldrich). The amplified cDNA was purified with a GenElute PCR cleanup kit (Sigma-Aldrich), and the concentration, purity, and quality of ds-cDNA were determined using a NanoDrop 1000 instrument, a 2100 Bioanalyzer, and an Agilent DNA 7500 kit (Agilent Technologies). A total RNA concentration of 400 ng yielded on average 12 μg of ds-cDNA. The labeling and hybridization of cDNA samples (2.0 μg of ds-cDNA) to the microarray were done at the facility Centro de Investigación Principe Felipe (Valencia, Spain) according to the Agilent Technology protocol for arrays. *Prochlorococcus* arrays were designed using the eArray web-based tool (Agilent Technology, Inc. [https://earray.chem.agilent.com/earray/]) as described in the supplemental material. All data analyses were performed using R (www.R-project.org) and packages distributed by the Bioconductor project (68) as described in the supplemental material.

### Data availability.

Microarray data have been deposited at NCBI Gene Expression Omnibus (GEO) under accession no. GSE154594. The 16S raw sequences have been deposited in the Sequence Read Archive (SRA) under BioProject no. PRJNA758505. The main scripts for the 16S abundances and the microarray probes are available in GitHub (https://github.com/jdmagasin/ProchlorococcusGlucoseAssimilation). The fcs files have been deposited in the Flow Repository under accession no. FR-FCM-Z5NF.
