# Supplementary material for: Differential Timing for Glucose Assimilation in Prochlorococcus and Coexistent Microbial Populations in the North Pacific Subtropical Gyre
Source: Microbiol Spectr. 2022 Sep 13;10(5):e02466-22. doi: 10.1128/spectrum.02466-22 (PMC9602893; doi:10.1128/spectrum.02466-22)
Supplement: Supplemental file 1 — Supplemental material. Download spectrum.02466-22-s0001.pdf, PDF file, 6.6 MB [file spectrum.02466-22-s0001.pdf]

Supplemental Information for

Differential timing for glucose assimilation in *Prochlorococcus* and coexistent microbial populations at the North Pacific Subtropical Gyre

María del Carmen Muñoz-Marín, Solange Duhamel, Karin M. Björkman, Jonathan D. Magasin, Jesús Díez-Dapena, David M. Karl, and José M. García-Fernández

Corresponding author:
María del Carmen Muñoz-Marin
Email address: b32mumam@uco.es

This file includes:

Supplementary Information
Figures and captions for Supplemental Information
References for supplementary information

Supplementary Information

Extended description of Material and Methods described in the main text.

Estimation of ambient glucose concentrations

Surface seawater was amended with increasing amounts of radiolabeled glucose, either as ^{3}H-Glc (HOT 296, 298) or ^{14}C-Glc (HOT 295, 298), incubated in on-deck incubators and subsampled in a time-course fashion. Radioactive glucose was added at five to six different concentrations within a target range of 0.2–2.0 nmol Glc l^{-1}. Each target concentration was run as duplicate incubations. The incubations were subsampled after 0.5, 1, 1.5, 2, and 4 hours (h). The 4 h timepoint was used as validation for the 4-h incubations used in the diel study. On HOT 295 incubations started at 12:30 PM and used ^{14}C-Glc only. During HOT 296 two bioassays were run using ^{3}H-Glc with incubations started at 07:10 on 6th Oct, and at 09:40 on the 7th of Oct. Two additional bioassays were performed on HOT 298 using both ^{3}H- and ^{14}C-Glc and started at noon on Dec 14th. At each sampling time point, 10 ml were filtered onto 0.2 µm pore size polycarbonate filters, rinsed with filtered seawater and placed into plastic scintillation vials (Simport snap-twist vials, 7 ml). A small subsample (25 µl) was also collected from each incubation bottle to determine the total radioactivity and calculate the added glucose concentration from the specific radioactivity provided by the manufacturer of each isotope.

Glucose turnover times were determined by dividing the total radioactivity by the assimilation rate, where the net rate of assimilation was derived from the linear regression of the increase in particulate radioactivity over time. By plotting the turnover times against the

concentrations of added glucose, the ambient concentration of glucose can be derived from where the linear regression line intersects the x-axis (y=0, absolute value). The final glucose assimilation rates were calculated from the ambient + added glucose concentrations.

Flow cytometry counting and cell sorting

Phytoplankton groups were enumerated using a BD Influx flow cytometer equipped with a forward scatter (FSC) detector with small particle option (BD Biosciences, San Jose, CA, USA). *Prochlorococcus*, *Synechococcus, Crocosphaera* and pigmented eukaryotes (<5-µm (1)) were enumerated in unstained samples following published protocols (2). Briefly, cells were identified based on red fluorescence signals vs FSC, then further gated by FSC and orange fluorescence. The high phycoerythrin (orange) signal in *Synechococcus* and *Crocosphaera* was used to distinguish them from *Prochlorococcus* and pigmented eukaryotes. A 488 plus a 457 nm (200 and 300 mW solid state, respectively) laser focused into the same pinhole resolved dim surface *Prochlorococcus* population from background noise in a FSC vs red fluorescence plot. Potential particle aggregates were discarded using a pulse width vs. forward scatter plot. Calibration and alignment were done using 1-µm yellow-green microspheres (Polysciences, USA).

Group-specific rates of ^{3}H-glucose /^{14}C-glucose assimilation and primary production by *Prochlorococcus* were determined by measuring the amount of radioactivity assimilated into populations sorted using the BD Influx (100 µm nozzle tip, sheath solution (sodium chloride 6 g L^{-1} in ultrapure water and filtered in-line through a 0.22-µm Sterivex™ filter unit), 1.0 drop single mode) according to Duhamel et al., (1, 3).

The drop delay was calibrated using Accudrop Beads (BD Biosciences, USA) and sorting efficiency was verified manually by sorting a specified number of 1-µm yellow-green microspheres (Polysciences, USA) onto a glass slide and counting the beads under an epifluorescence microscope. We systematically recovered 100% of the targeted beads before sorting cells. For each sample, 50,000 *Prochlorococcus* cells were sorted, filtered onto 0.2-µm polycarbonate membranes, rinsed with filtered seawater, and assayed by liquid scintillation counting (dpm $cell^{-1}$). The ^{14}C-labeled samples were acidified with 1 mL of 2M HCl for 24 h to remove any unincorporated ^{14}C-sodium bicarbonate before adding the scintillation cocktail. Radioactivity per cell (dpm $cell^{-1}$) measured in the killed control samples was subtracted from radioactivity per cell measured in the respective sample. On average, radioactivity in the killed controls for 50,000 *Prochlorococcus* cells sorted (i.e., blanks) was $5 \pm 2x10^{-5}$ and $4.7 \pm 1.3x10^{-4}$ dpm $cell^{-1}$ for ^{3}H-glucose and ^{14}C-sodium bicarbonate, respectively. Detection limits are defined as 2X the killed control before it is subtracted from the sample. The cell-specific assimilation rate (nmol $cell^{-1}$ h^{-1}) was calculated by dividing the radioactivity per cell (dpm $cell^{-1}$) by the total microbial activity (dpm l^{-1}) measured in the same treatment, and then multiplied by the total microbial assimilation rate at ambient plus added organic substrate concentration (S_a+S*, nmol l^{-1} h^{-1}) as described in Duhamel and coworkers (3).

16S rRNA analysis

A total of 718 ASVs were identified within the 16S rRNA data sets (Methods, Table S2.1). The total numbers of reads assigned to ASVs were similar across samples (mean 132,955 reads in ASVs, range 101,003 to 156,099). Rarefaction curves (with vegan R package

function rarefaction) showed that ASV counts saturated well before 101K reads for all samples. Therefore, sequencing depth in each sample was sufficient to capture the richness of the microbial community. Rarefying to 101K reads, samples had a mean of 404 ASVs (s.d.=33).

Proteobacteria and cyanobacteria dominated all samples and had stable relative abundances (means were 0.43±0.04 and 0.38±0.02, respectively) despite different sample collection dates, treatments, and incubation times (Fig. S2A). Alpha-proteobacteria dominated and Delta- and Gamma-proteobacteria were rare (mean relative abundances were 0.40, 0.02, and 0.01, respectively). *Prochlorococcus* dominated cyanobacteria (mean 0.36) and *Synechococcus*, the only other identified cyanobacterial genus, was rare (<0.01).

NMDS analysis of the 16S rRNA ASV abundances was performed using vegan. First, the ASV count matrix was scaled to normalize sequencing depths (decostand ('total')). Then metaMDS was used with Bray-Curtis dissimilarities and auto-transformation disabled, but default parameters otherwise. The NMDS stress was 0.08. Samples did not cluster by treatment or incubation time . Indeed, the relative abundance of *Bacteroidetes* was higher in samples collected on October 6 (0.12) than on October 7 (0.08; $p<0.01$, Welch two-sample *t*-test).

To identify ASVs that had statistically significant abundance changes in response to glucose, we used the edgeR and limma packages (4, 5). EdgeR transforms sequence count data so that they can be used with the same limma functions as in the microarray differential expression (DE) analysis. Counts for the 718 ASVs were imported into an DGEList, and then rare ASVs were removed as described in (6) and implemented in filterByExpr, leaving 678 ASVs. Normalization factors to correct for different sequencing depths were calculated using the TMM approach described by (7) and implemented in calcNormFactors. Sequencing depths were similar (101-156K reads) so the normalization factors had a narrow range (0.93 to 1.10). The normalized ASV count matrix was transformed to log counts per million (cpm). Next, we used the limma-trend method (8, 9) to identify differentially abundant ASVs, with steps nearly identical to those in the DE analysis. Linear models for the ASV abundances were created (lmFit) and empirical Bayesian inference performed with moderated *t*-statistics relative to a 1.2× fold change (treat with trend=TRUE). ASVs that had fold changes significantly larger than 1.2× ($p<0.05$) had to pass a final test: The ASV had to have >50 reads in at least one of the samples compared, for example, in at least one of the four samples in the 2D_12h_glucose vs. 2D_12h_control comparison. Although an ASV with 50 reads would comprise only 0.04% of the reads in a sample (mean; max 0.05%), that ASV would be more abundant than 41% of the other ASVs detected in the sample (on average).

A total of 33 differentially abundant (DA) ASVs were identified in five comparisons of matched glucose vs. controls (Table S2B). None had more than a few hundred reads in any of the compared glucose and control replicates (maximum of 363 reads; Fig. S2B). All of the DA ASVs had 0 reads in one of the conditions compared and >0 reads in the other condition (Table S2B), which indicated that the ASV changed from undetected to detected. Although detected, the DA ASVs had small relative abundances (medians < 0.001 and maximum =

0.005; Fig. S2B) In comparison, the 100th most abundant ASV in each sample had relative abundance slightly >0.001. Moreover, PERMANOVA analyses (with vegan adonis2 using Bray-Curtis dissimilarities and 999 permutations, blocked by experiment) indicated that glucose addition did not have a significant impact on community composition (p>0.05, whether samples were represented by ASV or phylum abundance profiles). Therefore, we interpret the abundance changes in response to glucose as too small to be biologically meaningful. This is consistent with the NMDS results which did not show samples clustering by treatment.

Design of the Prochlorococcus array

The *Prochlorococcus* oligonucleotide expression array was designed using *Prochlorococcus* genes and the eArray Web-based tool (Agilent Technology Inc.; https://earray.chem.agilent.com/earray/) similarly to the array design previously described by (10, 11). The gene sequences were obtained from the National Center of Biotechnology Information (NCBI; https://www.ncbi.nlm.nih.gov). Briefly, six probes of 60 nucleotides in length were designed for each gene, and a total of 7,501 probes (1,326 genes) were designed for *Prochlorococcus*. The probes were designed based on the sequenced genomes of the strains more abundant at Station ALOHA for specific core genes involved in carbon metabolism and photosynthesis pathways (Fig. S3D). These probes were replicated 4 times in the 8 × 60K array slides, which allowed internal evaluation of signals. The sequences of all oligonucleotide probes were tested *in silico* for possible cross-hybridization as described below. The probe sequences were used as queries in the BLASTN against the following available nt databases in June 2017: Marine microbes, Microbial Eukaryote Transcription, and Non-redundant Nucleotides NCBI SRA website and all rRNA databases from Silva as of February 2, 2016.

Agilent technology allows 5% nt mismatch in the whole probe region; thus, sequences with a range of 95% to 100% nt identity to the target probe are detected. Therefore, all probes with BLASTN hits with ≥95% over 100% of the nt length were deleted. Next, the probe sequences that passed the cross-hybridization filter were clustered using CD-HIT-EST (12, 13) at 95% nt similarity to select unique probes for *Prochlorococcus*.

In addition, standard control probes (IS-62976-8-V2_60Kby8_GX_EQC_201000210 with ERCC control probes added) were included randomly as part of the Agilent Technology array to feature locations on the microarray slide. The final design of the microarray was synthesized on a platforms of ca. 62,976 experimental probes and 1,319 control probes on each 8 × 60K array slide. The probe sequences are available at NCBI Gene Expression Omnibus (GEO) under accession number GSE154594.

Microarray data analysis

All data analyses were performed with R (www.R-project.org) and packages from the Bioconductor Project (14), specifically, using the Biobase (15), Linear Models for Microarray LIMMA (16), arrayQualityMetrics (17) and affyPLM (18, 19). These packages were mainly utilized via software that was developed for the MicroTOOLs environmental microarray (10, 20), which we adapted slightly to the *Prochlorococcus* microarrays. As in the prior study (20) arrays were normalized by quantiles and gene intensities were calculated by

median polishing. Gene detection was done separately for each gene in each sample. Specifically, each gene *g* was detected in each sample *s* if it had a signal to noise ratio $SNR_{gs} \geq 5$, where $SNR_{gs} = S_{gi} / BG_s$ and BG_s was the background intensity in *s*. We defined BG_s based on the lowest detected ERCC mRNA spike-in transcripts. For each sample ERCC spike-in transcript intensities were linearly modeled. Then we identified in *s* the least concentrated ERCC with a modelled intensity that was twice the median of measured intensities for Agilent negative control probes (structural hairpins). BG_s was the modelled intensity for this ERCC. On average 448 genes (mean) were detected in each sample (min 416, max 538). In total, 775 detected genes were detected across the samples (union). Raw and normalized microarray data for *Prochlorococcus* were submitted to NCBI GEO under accession number GSE154594.

As in the prior study (20), differentially expressed (DE) genes were identified using the LIMMA functions lmFit, eBayes, and topTreat. Empirical Bayes is well suited to studies with few samples because it pools them to estimate the variances for each gene's linear model (21). To identify biologically relevant DE, we looked for genes with fold changes that were at least 1.3× different (not simply >0) between treatments and matched controls (Benjamini-Hochberg adjusted *p*-value < 0.05). DE genes always had changes >1.5-fold (mean 2.3-fold) and were mainly identified in the experiment 2 at 12h (2D_12h_glucose vs. 2D_12h_control). DE genes were required to be above detection cut offs in at least one of the treatment or control samples. Most DE genes (157 genes) were identified in the 12-h incubations that terminated in the dark at 21:00 ("2D_12h" for 2D_12h_glucose vs. 2D_12_control). Although we did not have replicates for 2D_12h, our DE tests borrowed information from all 15 microarrays to determine which genes had fold changes that were significant relative to their estimated gene variances (21, 22).

Differentially expressed pathways

The gene differential expression analysis identified individual genes in specific *Prochlorococcus* strains that responded to glucose addition. We also used a complementary approach, an Ensemble of Gene Set Enrichment Analyses (EGSEA;(23)), to identify differentially expressed pathways from HL and LL *Prochlorococcus*. EGSEA identifies sets of genes that collectively show significant differential expression based on a consensus of 12 GSEA algorithms. As in a previous study by Shilova and colleagues (20), gene sets were defined by pathway and phylogroup, HL or LL in the present work (Table S5A). Each pathway included genes that would change in the same direction and thus reinforce any signal detected by EGSEA. Differentially expressed pathways for HL and LL were tested in the same treatments versus controls as in the DE gene analysis (Table S4C). The *p*-values from the 12 GSEA algorithms were combined using Wilkinson's method (24) and then corrected for multiple testing using the approach of Benjamini and Hochberg (25). Adjusted *p*-values < 0.01 were significant.

In comparison to the DE results in experiment 2 after 12 h for HL *Prochlorococcus* (main text), EGSEA corroborated increases for respiration, pentose phosphate pathway, and sugar transporter (*glcH*) genes, as well as the decreases for *pykF* genes in glycolysis (Table S5B). DE results that were not corroborated by EGSEA were due to the adjusted *p*-value exceeding 0.01. However, the direction of change identified by EGSEA was always consistent with the

reported DE genes. For example, Entner-Dudoroff had 5 *gdh* gene targets (from 5 distinct strains) with transcript level increases (1.7-3.6-fold; Table S4C), whereas EGSEA found that collectively the 23 genes detected in the pathway (Table S5A) did not change significantly (*p*=0.20) despite an average 1.6-fold increase. Similarly, for the Krebs Cycle the DE analysis identified 6 *fumC* and 1 *ppc* targets (1.6-2.0-fold increases), whereas EGSEA found that the 40 detected genes did not change significantly (*p*=0.02; average 1.4-fold increase). EGSEA also found insignificant increases for the 25 detected RuBisCO genes (*p*=0.02; average 1.5-fold increase), whereas the DE analysis found 4 *rbcS* genes that increased (2.0-6.5-fold). Interestingly, EGSEA identified small but significant decreases from photosystem I genes (average 1.3-fold decrease) even though only 3 of the 40 detected genes decreased (and none increased) in the DE analysis. These results underscore the robustness of the DE results by a complementary approach, EGSEA.

Note that the “Circadian rhythm” gene set included only *kaiC* genes (Table S5A) and does not capture the *kaiB* increases described in the main text. The full EGSEA results are in Table S5C.

Captions for Supplemental Information

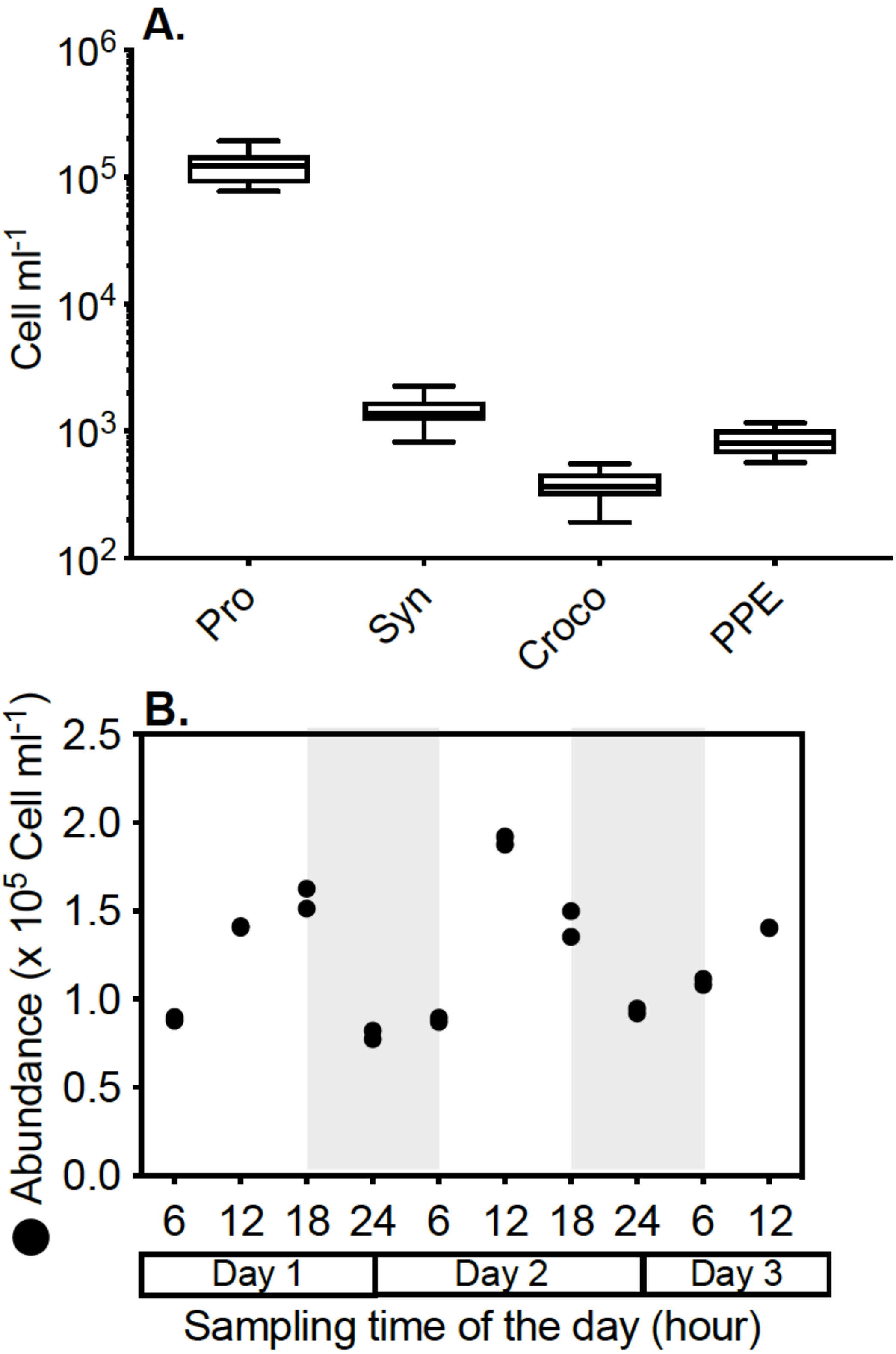

Figure S1. (A) Boxes and whiskers plot of picophytoplankton cell abundances (cell ml^{-1}). *Prochlorococcus* (Pro), *Synechococcus* (Syn), *Crocosphaera* (Croco) and picophytoeukaryotes (PPE). The box extends from the 25th to 75th percentiles and the line

within the box represents the median. Whiskers represent the minimum and maximum values. **(B) Cell abundances (10^5 cells L^{-1}, black dots) during the three sampling days.** The shaded area represents the dark period.

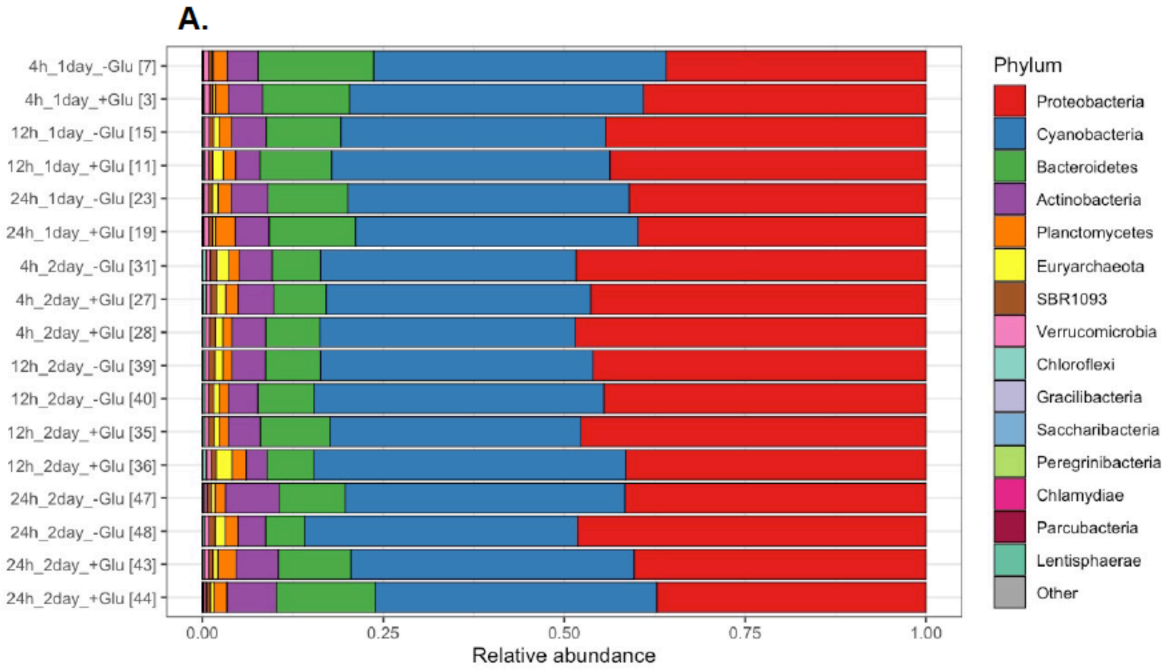

B.

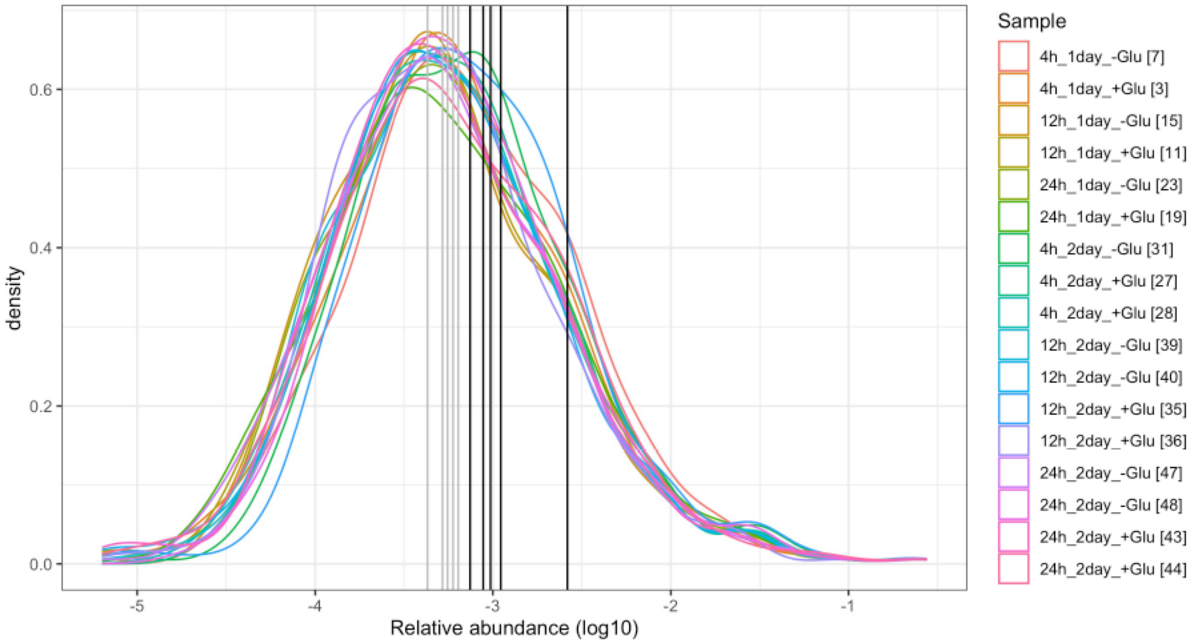

Figure S2. (A) Phylum relative abundances in the 16S rRNA data sets. The 718 ASVs were binned by phylum. Rare ASVs or those with unknown phylum were binned in Other. Samples are grouped by experiment (first 6 are experiment 1), then incubation time, and then treatment. Row labels include sample ID numbers in brackets. **(B) Distribution of relative abundances for detected ASVs for each sample.** Vertical lines indicate the maxima (black) and median (gray) relative abundances of the DA ASVs identified in any of the five comparisons, with black for maxima and gray for medians.

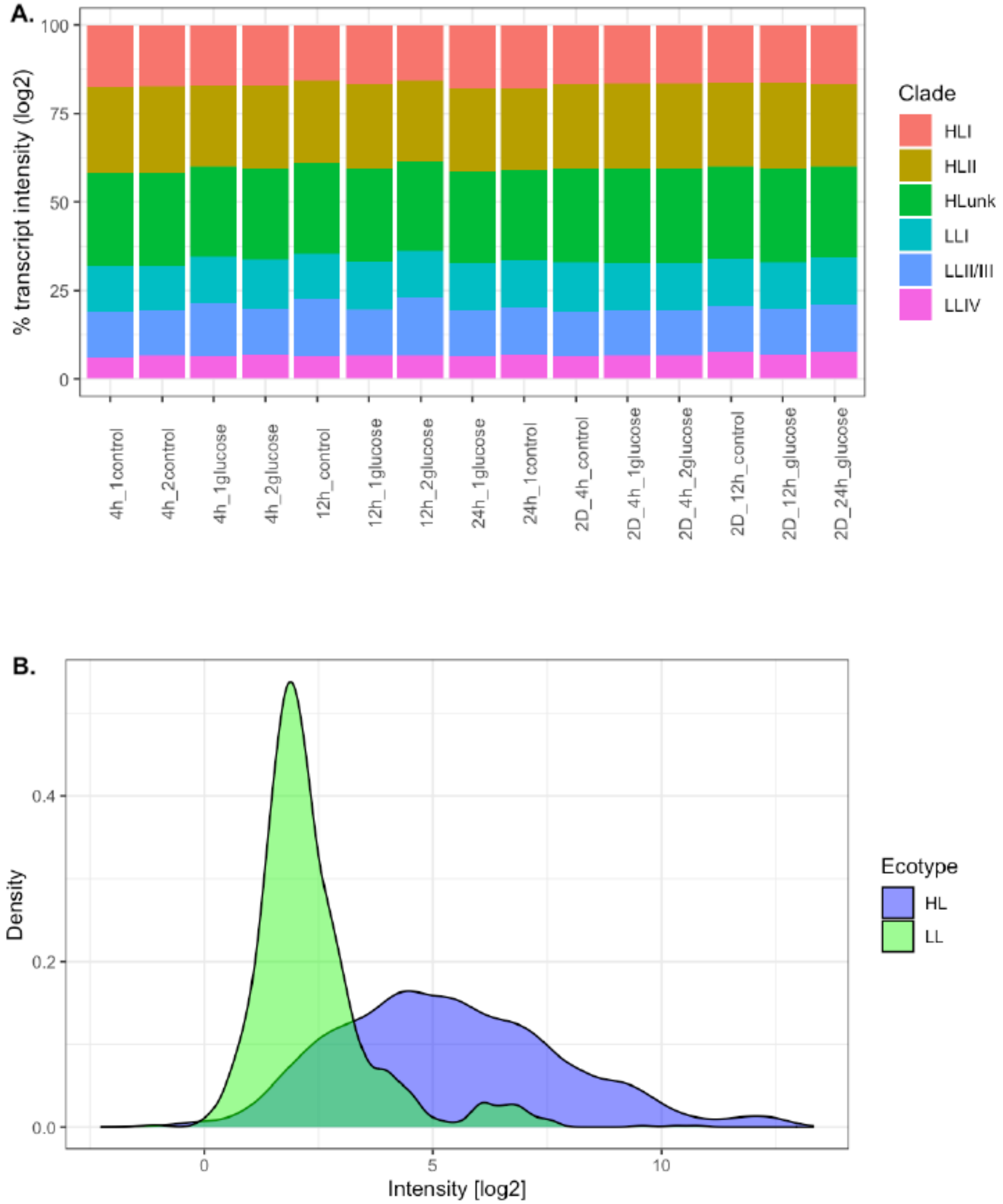
A.
% transcript intensity (log2)
100
75
50
25
0
4h_1control
4h_2control
4h_1glucose
4h_2glucose
12h_control
12h_1glucose
12h_2glucose
24h_1glucose
24h_1control
2D_4h_control
2D_4h_1glucose
2D_4h_2glucose
2D_12h_control
2D_12h_glucose
2D_24h_glucose
Clade
HLI
HLII
HLunk
LLI
LLII/III
LLIV
B.
Density
0.4
0.2
0.0
0
5
10
Intensity [log2]
Ecotype
HL
LL

C.

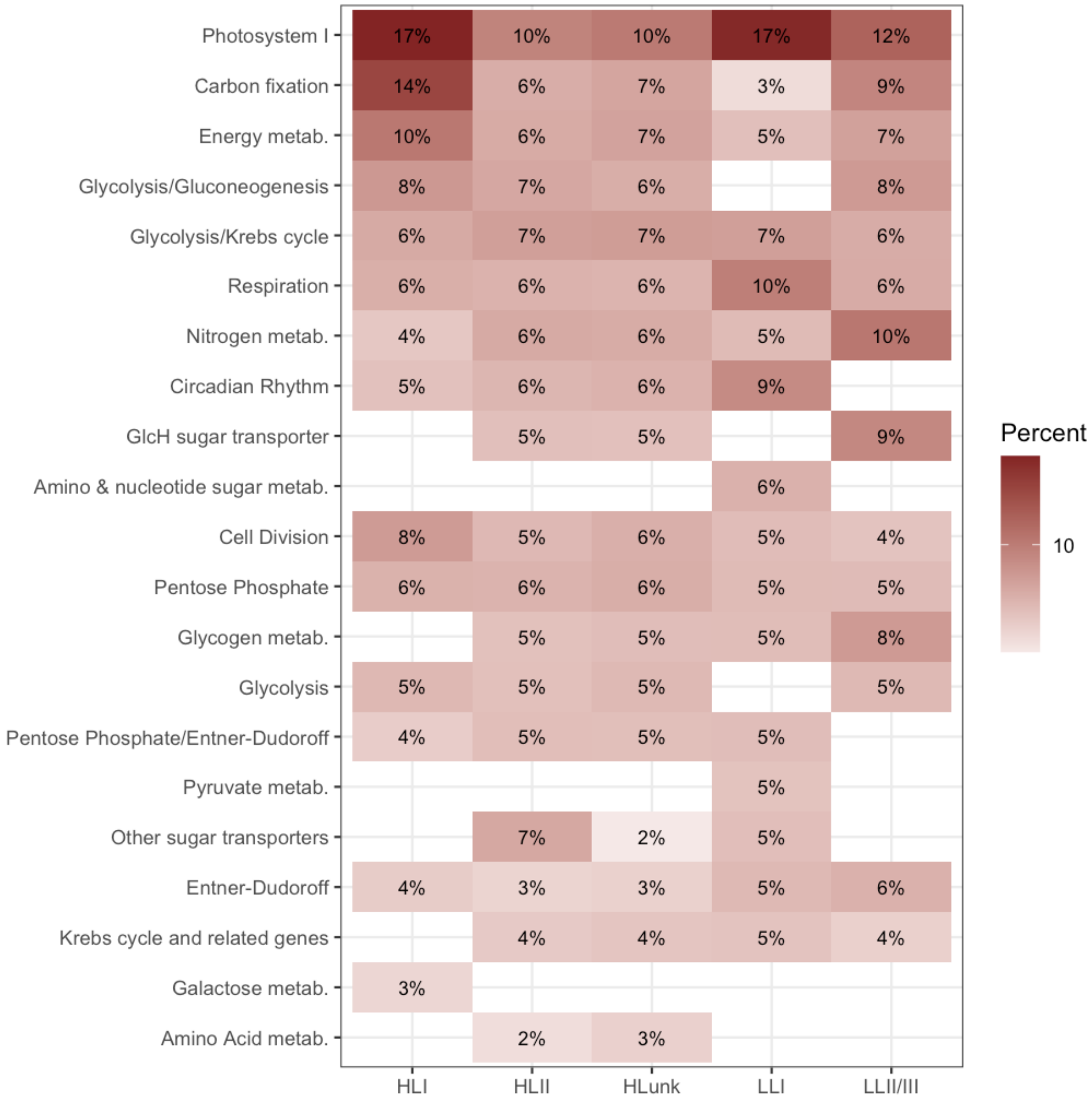

Photosystem I
Carbon fixation
Energy metab.
Glycolysis/Gluconeogenesis
Glycolysis/Krebs cycle
Respiration
Nitrogen metab.
Circadian Rhythm
GlcH sugar transporter
Amino & nucleotide sugar metab.
Cell Division
Pentose Phosphate
Glycogen metab.
Glycolysis
Pentose Phosphate/Entner-Dudoroff
Pyruvate metab.
Other sugar transporters
Entner-Dudoroff
Krebs cycle and related genes
Galactose metab.
Amino Acid metab.
17% 10% 10% 17% 12%
14% 6% 7% 3% 9%
10% 6% 7% 5% 7%
8% 7% 6% 8%
6% 7% 7% 7% 6%
6% 6% 6% 10% 6%
4% 6% 6% 5% 10%
5% 6% 6% 9%
5% 5% 9%
6%
8% 5% 6% 5% 4%
6% 6% 6% 5% 5%
5% 5% 5% 8%
5% 5% 5% 5%
4% 5% 5% 5%
5%
7% 2% 5%
4% 3% 3% 5% 6%
4% 4% 5% 4%
3%
2% 3%
HLI
HLII
HLunk
LLI
LLII/III
Percent
10

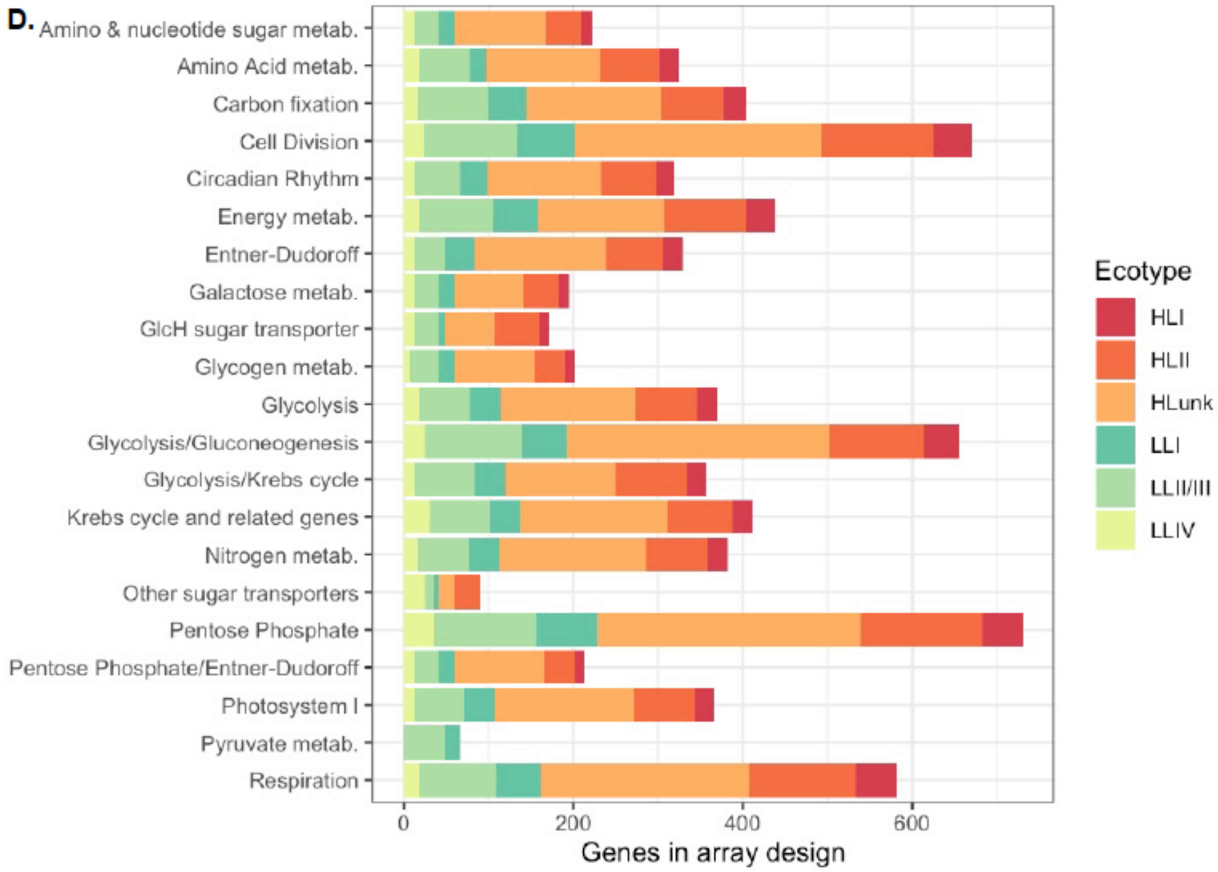

Figure S3. (A) Total transcript levels (normalized, log_2) for each *Prochlorococcus* clade across the samples. Sample labels indicate the incubation time (4h, 12h and 24h) in the presence of glucose or in the control treatment, and the replicate (1 or 2). Samples from the second experiment ("2D") were biological replicates. **(B) Distribution of normalized log_2 transcript levels for detected genes from High Light (HL) and Low Light (LL) ecotypes of *Prochlorococcus*. (C) Heat map showing the relative transcription levels across pathways (rows) in the controls for each *Prochlorococcus* clade (column).** For each clade, pathways were normalized for different gene counts by taking the mean transcript levels (in control samples) of genes in each pathway. Percentages were calculated with respect to the sum of the clade´s pathway means. The percentages shown are the averages over control samples. Photosystem I had the highest percentages and is therefore the top row. Empty cells indicate pathways that were not detected in the controls. **(D) Bar plot showing a summarize of the array design.** Each bar represents a pathway of gene counts, colored by HL vs LL (same colors as the heat map).

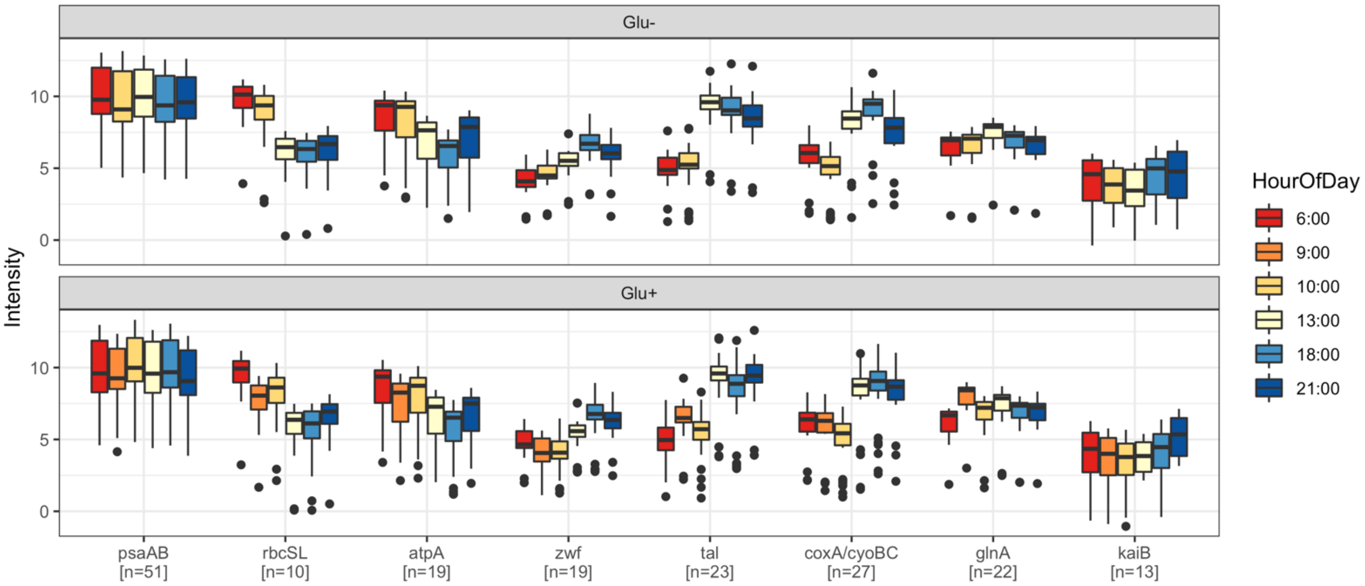

Figure S4. Transcription intensity for selected genes known to have diel expression patterns for HL *Prochlorococcus*. For each gene, intensities differed between day versus night samples, consistent with the times of higher expression observed in Zinser et al 2009. Photosynthesis genes *psaAB* were an exception, possibly because their diel expression patterns had small amplitude changes (26).

A.

TIME	Friday 6-Oct-2017	Saturday 7-Oct-2017	Sunday 8-Oct-2017	Monday 9-Oct-2017
00:20		Sampling and spiking substrates for: ASSIM: PP and Glu-S4	Sampling and spiking substrates for: ASSIM: PP and Glu-S8	
01:20	Arrive ALOHA (2:00)			
02:20				
03:20				
04:20		ASSIM: Add PFA-S4	ASSIM: Add PFA-S8	
05:20				
06:20	Sampling and spiking substrates for: - GENOM: Exp.1 - ASSIM: PP and Glu- S1	GENOM: Filter SW 24h Exp.1 Sampling and spiking substrates for: ASSIM: PP and Glu- S5	Sampling and spiking substrates for: ASSIM: PP and Glu-S9	
07:20				
08:20				Arrive Pier 35
09:20		Sampling and spiking substrates for: - GENOM: Exp. 2	GENOM: Filter SW 24h Exp. 2	
10:20	GENOM: Filter SW 4h Exp. 1 ASSIM: Add PFA-S1	ASSIM: Add PFA-S5	ASSIM: Add PFA-S9	
11:20				
12:20	Sampling and spiking substrates for: ASSIM: PP and Glu-S2	Sampling and spiking substrates for: ASSIM: PP and Glu- S6	Sampling and spiking substrates for: ASSIM: PP and Glu-S10	
13:20		GENOM: Filter SW 4h Exp. 2		
14:20				
15:20				
16:20	ASSIM: Add PFA-S2	ASSIM: Add PFA-S6	ASSIM: Add PFA-S10	
17:20				
18:20	GENOM: Filter SW 12h Exp.1 Sampling and spiking substrates for: - ASSIM: PP and Glu- S3	Sampling and spiking substrates for: ASSIM: PP and Glu- S7		
19:20				
20:20				
21:20		GENOM: Filter SW 12h Exp. 2		
22:20	ASSIM: Add PFA-S3	ASSIM: Add PFA-S7		
23:20				

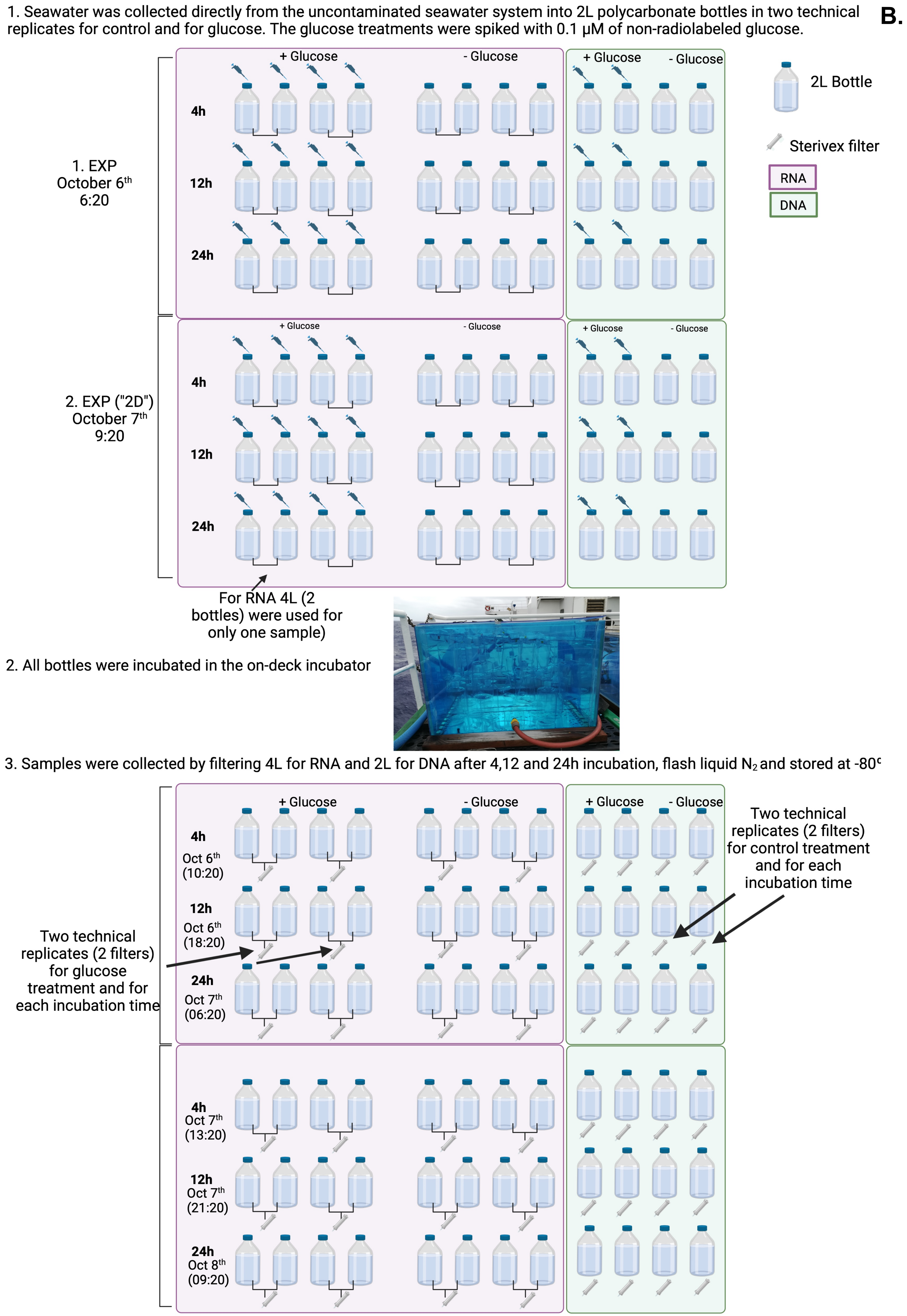
B.
1. Seawater was collected directly from the uncontaminated seawater system into 2L polycarbonate bottles in two technical replicates for control and for glucose. The glucose treatments were spiked with 0.1 μM of non-radiolabeled glucose.
2L Bottle
Sterivex filter
RNA
DNA
1. EXP
October 6th
6:20
2. EXP ("2D")
October 7th
9:20
+ Glucose
- Glucose
4h
12h
24h
For RNA 4L (2 bottles) were used for only one sample)
2. All bottles were incubated in the on-deck incubator
3. Samples were collected by filtering 4L for RNA and 2L for DNA after 4,12 and 24h incubation, flash liquid N2 and stored at -80c
4h
Oct 6th
(10:20)
12h
Oct 6th
(18:20)
24h
Oct 7th
(06:20)
4h
Oct 7th
(13:20)
12h
Oct 7th
(21:20)
24h
Oct 8th
(09:20)
Two technical replicates (2 filters) for glucose treatment and for each incubation time
Two technical replicates (2 filters) for control treatment and for each incubation time

C.

**Sample#**	**Date**	**Start**	**End**	**Light flux (E m^{-2})**
**Diel study**				(per 4 hour)
1	10/6/2017	0640	1040	5.0
2	10/6/2017	1245	1645	14.9
3	10/6/2017	1830	2235	0.0
4	10/7/2017	0035	0435	0.0
5	10/7/2017	0635	1035	10.7
6	10/7/2017	1245	1640	17.7
7	10/7/2017	1830	2235	0.0
8	10/8/2017	0030	0430	0.0
9	10/8/2017	0640	1040	12.3
10	10/8/2017	1240	1640	17.6
**Genomics Exp. 1**			**Incubation time (h)**	**Total light (E m^{-2})**
1	10/6/17	1200	4	18.4
2	10/6/17	1200	12	20.9
3	10/6/17	1200	24	39.4
**Genomics Exp. 2**				
1	10/7/2017	1600	4	3.3
2	10/7/2017	1600	12	3.3
3	10/7/2017	1600	24	40.4

Table S1. (A) Schematic illustration of the experimental design during the HOT 296 cruise. Green color indicates the incubation experiments with glucose for genomics-based approaches. For each experiment, 72 L were collected into 2 L polycarbonate bottles. 24 L were collected for DNA and 48 L for RNA.

For each genomic approach, two technical replicates were collected at 3 different times: after 4h, 12h and 24h of incubation, in the presence of glucose or in the control treatment. At each sampling time point, RNA and DNA samples were collected by filtering 4 L and 1 L of seawater. Yellow color indicates the incubation experiments with radiolabeled substrates. Seawater (SW) samples were collected every 6 h for a total of 10 samplings (S) over a 54-h period. Duplicate 60 ml bottles were spiked with radiolabeled glucose to a target addition of 2 nM glucose or with ^{14}C-sodium bicarbonate (final activity approximately 150 MBq l^{-1}). The diel samples were incubated for 4 h and terminated by adding paraformaldehyde (PFA) to stop the assimilation of the radiolabel and preserve the sample. The shaded area represents the dark period. **(B) Schematic illustration of the experimental design for the metatranscriptomic and transcriptomic experiments during the HOT 296 cruise. (C) Date and sampling times for the diel and genomic incubation experiments and surface light flux during the incubations.**

A.

ASV ID	Num reads	Annotation	Annotation	Annotation	Annotation	Annotation	Annotation	Annotation
ASV.1	561071	K_Bacteria	P_Cyanobacteria	C_Cyanobacteria	O_SubsectionI	F_FamilyI	G_Prochlorococcus	S_uncultured bacterium
ASV.2	180570	K_Bacteria	P_Cyanobacteria	C_Cyanobacteria	O_SubsectionI	F_FamilyI	G_Prochlorococcus	S_uncultured bacterium
ASV.3	93181	K_Bacteria	P_Proteobacteria	C_Alphaproteobacteria	O_Rhodospirillales	F_Rhodospirillaceae	G_AEGEAN-169 marine group	S_uncultured bacterium
ASV.4	70430	K_Bacteria	P_Actinobacteria	C_Acidimicrobiia	O_Acidimicrobiales	F_OM1 clade	G_Candidatus Actinomarina	S_uncultured bacterium
ASV.6	60204	K_Bacteria	P_Proteobacteria	C_Alphaproteobacteria	O_SAR11 clade	F_Surface 2	G_uncultured bacterium	S_unknown
ASV.5	58725	K_Bacteria	P_Proteobacteria	C_Alphaproteobacteria	O_SAR11 clade	F_Surface 1	G_unknown	S_unknown
ASV.7	52432	K_Bacteria	P_Proteobacteria	C_Alphaproteobacteria	O_SAR11 clade	F_Surface 1	G_uncultured bacterium	S_unknown
ASV.8	51639	K_Bacteria	P_Proteobacteria	C_Alphaproteobacteria	O_SAR11 clade	F_Surface 4	G_uncultured bacterium	S_unknown
ASV.9	42513	K_Bacteria	P_Proteobacteria	C_Alphaproteobacteria	O_SAR11 clade	F_Surface 1	G_uncultured bacterium	S_unknown
ASV.10	39645	K_Bacteria	P_Proteobacteria	C_Alphaproteobacteria	O_SAR11 clade	F_Surface 1	G_uncultured bacterium	S_unknown
ASV.11	26283	K_Bacteria	P_Planctomycetes	C_Planctomycetacia	O_Planctomycetales	F_Planctomycetaceae	G_Pirellula	S_uncultured planctomycete
ASV.12	25880	K_Bacteria	P_Bacteroidetes	C_Flavobacteriia	O_Flavobacteriales	F_Flavobacteriaceae	G_NS2b marine group	S_uncultured bacterium
ASV.13	22076	K_Bacteria	P_Proteobacteria	C_Alphaproteobacteria	O_Rickettsiales	F_SAR116 clade	G_uncultured bacterium	S_unknown
ASV.15	21033	K_Bacteria	P_Proteobacteria	C_Alphaproteobacteria	O_SAR11 clade	F_Surface 2	G_uncultured bacterium	S_unknown
ASV.14	20657	K_Bacteria	P_Proteobacteria	C_Alphaproteobacteria	O_SAR11 clade	F_Surface 2	G_uncultured bacterium	S_unknown
ASV.17	20519	K_Bacteria	P_Proteobacteria	C_Alphaproteobacteria	O_Rickettsiales	F_SAR116 clade	G_uncultured bacterium	S_unknown
ASV.16	20158	K_Bacteria	P_Proteobacteria	C_Alphaproteobacteria	O_Rhodospirillales	F_Rhodospirillaceae	G_AEGEAN-169 marine group	S_uncultured bacterium
ASV.18	17098	K_Bacteria	P_Actinobacteria	C_Acidimicrobiia	O_Acidimicrobiales	F_OM1 clade	G_Candidatus Actinomarina	S_uncultured bacterium
ASV.19	17041	K_Bacteria	P_Proteobacteria	C_Alphaproteobacteria	O_SAR11 clade	F_Surface 1	G_uncultured bacterium	S_unknown
ASV.20	16501	K_Bacteria	P_Proteobacteria	C_Alphaproteobacteria	O_Rhodospirillales	F_Rhodospirillaceae	G_AEGEAN-169 marine group	S_uncultured bacterium
ASV.21	16000	K_Bacteria	P_Proteobacteria	C_Alphaproteobacteria	O_Rhodobacterales	F_Rhodobacteraceae	G_uncultured	S_unknown
ASV.22	15404	K_Bacteria	P_Proteobacteria	C_Alphaproteobacteria	O_Rickettsiales	F_SAR116 clade	G_uncultured marine bacterium	S_unknown
ASV.23	14982	K_Bacteria	P_SBR1093	C_marine metagenome	O_unknown	F_unknown	G_unknown	S_unknown
ASV.24	13818	K_Bacteria	P_Cyanobacteria	C_Cyanobacteria	O_SubsectionI	F_FamilyI	G_Prochlorococcus	S_uncultured bacterium
ASV.25	13063	K_Bacteria	P_Proteobacteria	C_Deltaproteobacteria	O_SAR324 clade(Marine group B)	F_uncultured bacterium	G_unknown	S_unknown
ASV.27	12850	K_Bacteria	P_Proteobacteria	C_Alphaproteobacteria	O_Rickettsiales	F_SAR116 clade	G_uncultured marine bacterium	S_unknown
ASV.26	11717	K_Bacteria	P_Cyanobacteria	C_Cyanobacteria	O_SubsectionI	F_FamilyI	G_Prochlorococcus	S_uncultured bacterium
ASV.29	11680	K_Bacteria	P_Cyanobacteria	C_Cyanobacteria	O_SubsectionI	F_FamilyI	G_Prochlorococcus	S_uncultured bacterium
ASV.28	11602	K_Bacteria	P_Proteobacteria	C_Alphaproteobacteria	O_Rhodobacterales	F_Rhodobacteraceae	G_unknown	S_unknown
ASV.30	10420	K_Bacteria	P_Proteobacteria	C_Alphaproteobacteria	O_Rickettsiales	F_SAR116 clade	G_uncultured bacterium	S_unknown
ASV.31	9146	K_Bacteria	P_Cyanobacteria	C_Cyanobacteria	O_SubsectionI	F_FamilyI	G_Prochlorococcus	S_uncultured bacterium
ASV.41	8991	K_Bacteria	P_Cyanobacteria	C_Cyanobacteria	O_SubsectionI	F_FamilyI	G_Synechococcus	S_unknown
ASV.33	8660	K_Bacteria	P_Proteobacteria	C_Alphaproteobacteria	O_Rhodospirillales	F_Rhodospirillaceae	G_AEGEAN-169 marine group	S_uncultured bacterium
ASV.32	8444	K_Bacteria	P_Proteobacteria	C_Alphaproteobacteria	O_SAR11 clade	F_Surface 2	G_uncultured bacterium	S_unknown
ASV.34	8085	K_Bacteria	P_Proteobacteria	C_Alphaproteobacteria	O_SAR11 clade	F_Surface 1	G_uncultured bacterium	S_unknown
ASV.36	8073	K_Bacteria	P_Proteobacteria	C_Alphaproteobacteria	O_SAR11 clade	F_Surface 1	G_uncultured bacterium	S_unknown
ASV.37	8042	K_Bacteria	P_Proteobacteria	C_Alphaproteobacteria	O_Rhodobacterales	F_Rhodobacteraceae	G_unknown	S_unknown
ASV.35	7860	K_Bacteria	P_Proteobacteria	C_Alphaproteobacteria	O_Rickettsiales	F_S25-593	G_uncultured bacterium	S_unknown
ASV.38	7769	K_Bacteria	P_Proteobacteria	C_Alphaproteobacteria	O_SAR11 clade	F_Surface 1	G_uncultured bacterium	S_unknown
ASV.73	7736	K_Bacteria	P_Bacteroidetes	C_Flavobacteriia	O_Flavobacteriales	F_NS7 marine group	G_uncultured bacterium	S_unknown
ASV.40	7568	K_Bacteria	P_Proteobacteria	C_Alphaproteobacteria	O_Rhodospirillales	F_Rhodospirillaceae	G_AEGEAN-169 marine group	S_uncultured bacterium
ASV.42	7457	K_Bacteria	P_Bacteroidetes	C_Flavobacteriia	O_Flavobacteriales	F_Flavobacteriaceae	G_NS4 marine group	S_uncultured bacterium
ASV.39	7429	K_Bacteria	P_Proteobacteria	C_Alphaproteobacteria	O_SAR11 clade	F_Surface 1	G_unknown	S_unknown
ASV.43	7181	K_Bacteria	P_Proteobacteria	C_Alphaproteobacteria	O_Rhodospirillales	F_Rhodospirillaceae	G_AEGEAN-169 marine group	S_uncultured bacterium
ASV.47	7178	K_Bacteria	P_Planctomycetes	C_Phycisphaerae	O_Phycisphaerales	F_Phycisphaeraceae	G_Urania-1B-19 marine sediment gr	S_uncultured bacterium
ASV.67	6899	K_Bacteria	P_Bacteroidetes	C_Sphingobacteriia	O_Sphingobacteriales	F_uncultured	G_uncultured Sphingobacteriia bacte	S_unknown
ASV.44	6885	K_Bacteria	P_Proteobacteria	C_Alphaproteobacteria	O_SAR11 clade	F_Surface 2	G_uncultured bacterium	S_unknown
ASV.53	6835	K_Bacteria	P_Bacteroidetes	C_Flavobacteriia	O_Flavobacteriales	F_Flavobacteriaceae	G_NS4 marine group	S_uncultured bacterium
ASV.49	6460	K_Bacteria	P_Bacteroidetes	C_Flavobacteriia	O_Flavobacteriales	F_Flavobacteriaceae	G_NS4 marine group	S_uncultured bacterium

ASV.45	6343	K_Bacteria	P_Cyanobacteria	C_Cyanobacteria	O_SubsectionI	F_FamilyI	G_Prochlorococcus	S_uncultured bacterium
ASV.50	6326	K_Bacteria	P_Actinobacteria	C_Acidimicrobiia	O_Acidimicrobiales	F_Sva0996 marine group	G_uncultured bacterium	S_unknown
ASV.58	6316	K_Bacteria	P_Bacteroidetes	C_Flavobacteriia	O_Flavobacteriales	F_Flavobacteriaceae	G_unknown	S_unknown
ASV.46	6300	K_Bacteria	P_Proteobacteria	C_Alphaproteobacteria	O_Rickettsiales	F_S25-593	G_uncultured bacterium	S_unknown
ASV.48	6173	K_Bacteria	P_Proteobacteria	C_Alphaproteobacteria	O_Rhodospirillales	F_Rhodospirillaceae	G_AEGEAN-169 marine group	S_uncultured bacterium
ASV.52	6045	K_Archaea	P_Euryarchaeota	C_Thermoplasmata	O_Thermoplasmatales	F_Marine Group II	G_marine metagenome	S_unknown
ASV.51	5953	K_Bacteria	P_Proteobacteria	C_Alphaproteobacteria	O_SAR11 clade	F_unknown	G_unknown	S_unknown
ASV.54	5874	K_Bacteria	P_Proteobacteria	C_Alphaproteobacteria	O_SAR11 clade	F_Surface 4	G_uncultured bacterium	S_unknown
ASV.55	5812	K_Bacteria	P_Proteobacteria	C_Alphaproteobacteria	O_Rhodospirillales	F_Rhodospirillaceae	G_uncultured	S_uncultured bacterium
ASV.56	5777	K_Bacteria	P_Proteobacteria	C_Alphaproteobacteria	O_Rhodospirillales	F_Rhodospirillaceae	G_AEGEAN-169 marine group	S_uncultured bacterium
ASV.59	5546	K_Bacteria	P_Proteobacteria	C_Alphaproteobacteria	O_SAR11 clade	F_Surface 1	G_uncultured bacterium	S_unknown
ASV.60	5532	K_Bacteria	P_Cyanobacteria	C_Cyanobacteria	O_SubsectionI	F_FamilyI	G_Prochlorococcus	S_uncultured bacterium
ASV.65	5322	K_Bacteria	P_Proteobacteria	C_Gammaproteobacteria	O_Thiotrichales	F_Thiotrichaceae	G_Thiothrix	S_unknown
ASV.62	5313	K_Bacteria	P_Bacteroidetes	C_Flavobacteriia	O_Flavobacteriales	F_Flavobacteriaceae	G_NS4 marine group	S_uncultured bacterium
ASV.57	5273	K_Bacteria	P_Cyanobacteria	C_Cyanobacteria	O_SubsectionI	F_FamilyI	G_Prochlorococcus	S_uncultured bacterium
ASV.61	5182	K_Bacteria	P_Proteobacteria	C_Deltaproteobacteria	O_SAR324 clade(Marine group B)	F_uncultured bacterium	G_unknown	S_unknown
ASV.64	5145	K_Bacteria	P_Proteobacteria	C_Alphaproteobacteria	O_Rickettsiales	F_SAR116 clade	G_uncultured bacterium	S_unknown
ASV.74	5085	K_Bacteria	P_Cyanobacteria	C_Cyanobacteria	O_SubsectionIII	F_FamilyI	G_uncultured	S_unknown
ASV.66	5029	K_Bacteria	P_Bacteroidetes	C_Flavobacteriia	O_Flavobacteriales	F_Flavobacteriaceae	G_NS5 marine group	S_uncultured bacterium
ASV.70	4922	K_Bacteria	P_Bacteroidetes	C_Flavobacteriia	O_Flavobacteriales	F_NS7 marine group	G_uncultured bacterium	S_unknown
ASV.63	4868	K_Bacteria	P_Proteobacteria	C_Alphaproteobacteria	O_Rhizobiales	F_PS1 clade	G_uncultured bacterium	S_unknown
ASV.68	4817	K_Bacteria	P_Actinobacteria	C_Acidimicrobiia	O_Acidimicrobiales	F_OM1 clade	G_Candidatus Actinomarina	S_uncultured bacterium
ASV.75	4710	K_Bacteria	P_Proteobacteria	C_Alphaproteobacteria	O_Rhodobacterales	F_Rhodobacteraceae	G_uncultured	S_uncultured bacterium
ASV.72	4520	K_Bacteria	P_Proteobacteria	C_Alphaproteobacteria	O_SAR11 clade	F_Surface 1	G_uncultured bacterium	S_unknown
ASV.84	4455	K_Bacteria	P_Proteobacteria	C_Alphaproteobacteria	O_Rhizobiales	F_OCS116 clade	G_uncultured bacterium	S_unknown
ASV.77	4426	K_Bacteria	P_Proteobacteria	C_Alphaproteobacteria	O_SAR11 clade	F_Surface 1	G_uncultured bacterium	S_unknown
ASV.76	4424	K_Bacteria	P_Cyanobacteria	C_Chloroplast	O_uncultured bacterium	F_unknown	G_unknown	S_unknown
ASV.79	4355	K_Bacteria	P_Bacteroidetes	C_Flavobacteriia	O_Flavobacteriales	F_Flavobacteriaceae	G_NS5 marine group	S_uncultured bacterium
ASV.78	4350	K_Bacteria	P_Proteobacteria	C_Alphaproteobacteria	O_SAR11 clade	F_Surface 4	G_uncultured bacterium	S_unknown
ASV.91	4291	K_Bacteria	P_Bacteroidetes	C_Flavobacteriia	O_Flavobacteriales	F_Flavobacteriaceae	G_NS4 marine group	S_uncultured bacterium
ASV.69	4252	K_Bacteria	P_Bacteroidetes	C_Flavobacteriia	O_Flavobacteriales	F_Flavobacteriaceae	G_NS4 marine group	S_uncultured bacterium
ASV.81	4137	K_Bacteria	P_Bacteroidetes	C_Flavobacteriia	O_Flavobacteriales	F_Flavobacteriaceae	G_NS4 marine group	S_uncultured bacterium
ASV.80	4104	K_Bacteria	P_Proteobacteria	C_Alphaproteobacteria	O_Rhodospirillales	F_Rhodospirillaceae	G_uncultured	S_uncultured bacterium
ASV.87	3982	K_Bacteria	P_Proteobacteria	C_Alphaproteobacteria	O_Rhodospirillales	F_Rhodospirillaceae	G_OM75 clade	S_uncultured bacterium
ASV.82	3822	K_Bacteria	P_Bacteroidetes	C_Flavobacteriia	O_Flavobacteriales	F_Cryomorphaceae	G_uncultured	S_uncultured Owenweeksia sp.
ASV.88	3771	K_Bacteria	P_Bacteroidetes	C_Flavobacteriia	O_Flavobacteriales	F_Flavobacteriaceae	G_NS2b marine group	S_uncultured bacterium
ASV.71	3767	K_Bacteria	P_Proteobacteria	C_Alphaproteobacteria	O_Rhodobacterales	F_Rhodobacteraceae	G_uncultured	S_unknown
ASV.83	3754	K_Bacteria	P_Proteobacteria	C_Alphaproteobacteria	O_SAR11 clade	F_Surface 1	G_uncultured bacterium	S_unknown
ASV.101	3710	K_Bacteria	P_Verrucomicrobia	C_Opitutae	O_Puniceicoccales	F_Puniceicoccaceae	G_marine group	S_uncultured bacterium
ASV.92	3707	K_Bacteria	P_Bacteroidetes	C_Flavobacteriia	O_Flavobacteriales	F_NS7 marine group	G_uncultured bacterium	S_unknown
ASV.99	3688	K_Bacteria	P_Bacteroidetes	C_Flavobacteriia	O_Flavobacteriales	F_Cryomorphaceae	G_uncultured	S_uncultured Owenweeksia sp.
ASV.93	3663	K_Bacteria	P_Bacteroidetes	C_Flavobacteriia	O_Flavobacteriales	F_Flavobacteriaceae	G_unknown	S_unknown
ASV.94	3639	K_Bacteria	P_Bacteroidetes	C_Cytophagia	O_Cytophagales	F_Flammeovirgaceae	G_Marinoscillum	S_uncultured bacterium
ASV.86	3638	K_Bacteria	P_Proteobacteria	C_Alphaproteobacteria	O_Rickettsiales	F_SAR116 clade	G_uncultured bacterium	S_unknown
ASV.85	3577	K_Bacteria	P_Actinobacteria	C_Acidimicrobiia	O_Acidimicrobiales	F_OM1 clade	G_Candidatus Actinomarina	S_uncultured bacterium
ASV.113	3573	K_Bacteria	P_Bacteroidetes	C_Flavobacteriia	O_Flavobacteriales	F_Flavobacteriaceae	G_Aquibacter	S_uncultured bacterium
ASV.90	3530	K_Bacteria	P_Proteobacteria	C_Gammaproteobacteria	O_Thiotrichales	F_Thiotrichaceae	G_Thiothrix	S_uncultured bacterium
ASV.95	3512	K_Bacteria	P_Bacteroidetes	C_Flavobacteriia	O_Flavobacteriales	F_Flavobacteriaceae	G_NS4 marine group	S_uncultured bacterium
ASV.97	3457	K_Bacteria	P_Bacteroidetes	C_Flavobacteriia	O_Flavobacteriales	F_Cryomorphaceae	G_uncultured	S_uncultured Owenweeksia sp.
ASV.89	3348	K_Bacteria	P_Cyanobacteria	C_Cyanobacteria	O_SubsectionI	F_FamilyI	G_Prochlorococcus	S_uncultured bacterium

ASV.98	3335	K_Bacteria	P_Cyanobacteria	C_Chloroplast	O_uncultured bacterium	F_unknown	G_unknown	S_unknown
ASV.105	3255	K_Bacteria	P_Proteobacteria	C_Alphaproteobacteria	O_Rhodospirillales	F_Rhodospirillaceae	G_uncultured	S_unknown
ASV.104	3246	K_Bacteria	P_Bacteroidetes	C_Flavobacteriia	O_Flavobacteriales	F_Flavobacteriaceae	G_NS5 marine group	S_uncultured bacterium
ASV.100	3163	K_Bacteria	P_Bacteroidetes	C_Flavobacteriia	O_Flavobacteriales	F_Flavobacteriaceae	G_NS5 marine group	S_uncultured bacterium
ASV.102	3152	K_Bacteria	P_Proteobacteria	C_Alphaproteobacteria	O_Rickettsiales	F_S25-593	G_uncultured bacterium	S_unknown
ASV.96	3141	K_Bacteria	P_Proteobacteria	C_Alphaproteobacteria	O_Rickettsiales	F_SAR116 clade	G_uncultured bacterium	S_unknown
ASV.379	2941	K_Bacteria	P_Cyanobacteria	C_Cyanobacteria	O_SubsectionI	F_FamilyI	G_Prochlorococcus	S_uncultured bacterium
ASV.108	2892	K_Bacteria	P_Proteobacteria	C_Alphaproteobacteria	O_Rhodospirillales	F_Rhodospirillaceae	G_AEGEAN-169 marine group	S_uncultured bacterium
ASV.110	2878	K_Bacteria	P_Proteobacteria	C_Gammaproteobacteria	O_Legionellales	F_Coxiellaceae	G_Coxiella	S_unknown
ASV.106	2870	K_Bacteria	P_Bacteroidetes	C_Flavobacteriia	O_Flavobacteriales	F_Flavobacteriaceae	G_NS5 marine group	S_uncultured bacterium
ASV.111	2859	K_Bacteria	P_Proteobacteria	C_Alphaproteobacteria	O_SAR11 clade	F_Chesapeake-Delaware Bay	G_uncultured bacterium	S_unknown
ASV.109	2760	K_Bacteria	P_Bacteroidetes	C_Flavobacteriia	O_Flavobacteriales	F_Flavobacteriaceae	G_NS5 marine group	S_uncultured bacterium
ASV.115	2732	K_Bacteria	P_Bacteroidetes	C_Flavobacteriia	O_Flavobacteriales	F_Flavobacteriaceae	G_NS4 marine group	S_uncultured bacterium
ASV.107	2730	K_Bacteria	P_Actinobacteria	C_Acidimicrobiia	O_Acidimicrobiales	F_OM1 clade	G_Candidatus Actinomarina	S_uncultured bacterium
ASV.153	2652	K_Bacteria	P_Bacteroidetes	C_Flavobacteriia	O_Flavobacteriales	F_Flavobacteriaceae	G_uncultured	S_uncultured bacterium
ASV.103	2510	K_Bacteria	P_Cyanobacteria	C_Cyanobacteria	O_SubsectionI	F_FamilyI	G_Prochlorococcus	S_uncultured bacterium
ASV.112	2457	K_Bacteria	P_Proteobacteria	C_Alphaproteobacteria	O_SAR11 clade	F_Surface 1	G_uncultured bacterium	S_unknown
ASV.114	2445	K_Bacteria	P_Bacteroidetes	C_Flavobacteriia	O_Flavobacteriales	F_Flavobacteriaceae	G_NS4 marine group	S_uncultured bacterium
ASV.118	2443	K_Bacteria	P_Proteobacteria	C_Alphaproteobacteria	O_Rhodospirillales	F_Rhodospirillaceae	G_AEGEAN-169 marine group	S_uncultured bacterium
ASV.116	2385	K_Bacteria	P_Proteobacteria	C_Alphaproteobacteria	O_Rickettsiales	F_SAR116 clade	G_uncultured bacterium	S_unknown
ASV.119	2327	K_Bacteria	P_Proteobacteria	C_Alphaproteobacteria	O_Rhodospirillales	F_Rhodospirillaceae	G_AEGEAN-169 marine group	S_uncultured bacterium
ASV.134	2233	K_Bacteria	P_Bacteroidetes	C_Flavobacteriia	O_Flavobacteriales	F_Flavobacteriaceae	G_NS5 marine group	S_uncultured bacterium
ASV.136	2226	K_Bacteria	P_Chloroflexi	C_SAR202 clade	O_marine metagenome	F_unknown	G_unknown	S_unknown
ASV.186	2221	K_Bacteria	P_Cyanobacteria	C_Chloroplast	O_uncultured bacterium	F_unknown	G_unknown	S_unknown
ASV.152	2185	K_Bacteria	P_Bacteroidetes	C_Flavobacteriia	O_Flavobacteriales	F_Flavobacteriaceae	G_Polaribacter 4	S_uncultured bacterium
ASV.124	2179	K_Bacteria	P_Cyanobacteria	C_Chloroplast	O_uncultured bacterium	F_unknown	G_unknown	S_unknown
ASV.120	2176	K_Bacteria	P_Bacteroidetes	C_Flavobacteriia	O_Flavobacteriales	F_NS9 marine group	G_uncultured marine bacterium	S_unknown
ASV.121	2171	K_Bacteria	P_Bacteroidetes	C_Flavobacteriia	O_Flavobacteriales	F_NS9 marine group	G_uncultured marine bacterium	S_unknown
ASV.122	2166	K_Bacteria	P_Bacteroidetes	C_Flavobacteriia	O_Flavobacteriales	F_NS9 marine group	G_uncultured marine bacterium	S_unknown
ASV.126	2159	K_Bacteria	P_Bacteroidetes	C_Flavobacteriia	O_Flavobacteriales	F_Flavobacteriaceae	G_NS5 marine group	S_uncultured bacterium
ASV.123	2148	K_Bacteria	P_Proteobacteria	C_Deltaproteobacteria	O_Bradymonadales	F_uncultured bacterium	G_unknown	S_unknown
ASV.132	2116	K_Bacteria	P_Proteobacteria	C_Alphaproteobacteria	O_SAR11 clade	F_uncultured bacterium	G_unknown	S_unknown
ASV.125	2103	K_Bacteria	P_Proteobacteria	C_Alphaproteobacteria	O_SAR11 clade	F_Surface 1	G_uncultured bacterium	S_unknown
ASV.128	2054	K_Bacteria	P_Proteobacteria	C_Alphaproteobacteria	O_Rickettsiales	F_S25-593	G_uncultured bacterium	S_unknown
ASV.127	2050	K_Bacteria	P_Proteobacteria	C_Alphaproteobacteria	O_Rickettsiales	F_SAR116 clade	G_uncultured marine bacterium	S_unknown
ASV.139	2050	K_Bacteria	P_Proteobacteria	C_Alphaproteobacteria	O_Rickettsiales	F_SAR116 clade	G_uncultured bacterium	S_unknown
ASV.133	2042	K_Bacteria	P_Bacteroidetes	C_Flavobacteriia	O_Flavobacteriales	F_Flavobacteriaceae	G_NS5 marine group	S_uncultured bacterium
ASV.117	2010	K_Bacteria	P_Proteobacteria	C_Alphaproteobacteria	O_Rhodospirillales	F_Rhodospirillaceae	G_uncultured	S_uncultured bacterium
ASV.141	1991	K_Bacteria	P_Actinobacteria	C_Acidimicrobiia	O_Acidimicrobiales	F_Acidimicrobiaceae	G_unknown	S_unknown
ASV.135	1991	K_Bacteria	P_Proteobacteria	C_Deltaproteobacteria	O_SAR324 clade(Marine group B)	F_uncultured bacterium	G_unknown	S_unknown
ASV.130	1973	K_Bacteria	P_Proteobacteria	C_Alphaproteobacteria	O_SAR11 clade	F_Chesapeake-Delaware Bay	G_uncultured bacterium	S_unknown
ASV.158	1970	K_Bacteria	P_Proteobacteria	C_Alphaproteobacteria	O_Rhizobiales	F_Rhodobiaceae	G_Rhodobium	S_uncultured alpha proteobacteri
ASV.138	1965	K_Bacteria	P_Verrucomicrobia	C_Opitutae	O_MB11C04 marine group	F_uncultured bacterium	G_unknown	S_unknown
ASV.131	1963	K_Archaea	P_Euryarchaeota	C_Thermoplasmata	O_Thermoplasmatales	F_Marine Group II	G_uncultured marine archaeon	S_unknown
ASV.142	1939	K_Bacteria	P_Bacteroidetes	C_Flavobacteriia	O_Flavobacteriales	F_Flavobacteriaceae	G_NS5 marine group	S_uncultured bacterium
ASV.163	1892	K_Bacteria	P_Planctomycetes	C_Phycisphaerae	O_Phycisphaerales	F_Phycisphaeraceae	G_Urania-1B-19 marine sediment gı	S_uncultured bacterium
ASV.143	1866	K_Bacteria	P_Bacteroidetes	C_Flavobacteriia	O_Flavobacteriales	F_Flavobacteriaceae	G_NS5 marine group	S_uncultured bacterium
ASV.145	1856	K_Bacteria	P_Proteobacteria	C_Alphaproteobacteria	O_Rhodospirillales	F_Rhodospirillaceae	G_AEGEAN-169 marine group	S_uncultured bacterium
ASV.149	1849	K_Bacteria	P_Bacteroidetes	C_Flavobacteriia	O_Flavobacteriales	F_Flavobacteriaceae	G_NS4 marine group	S_uncultured bacterium
ASV.129	1821	K_Archaea	P_Euryarchaeota	C_Thermoplasmata	O_Thermoplasmatales	F_Marine Group II	G_uncultured marine group II euryar	S_unknown

ASV.144	1808	K_Bacteria	P_Chloroflexi	C_SAR202 clade	O_marine metagenome	F_unknown	G_unknown	S_unknown
ASV.140	1795	K_Bacteria	P_Proteobacteria	C_Alphaproteobacteria	O_Rhodobacterales	F_Rhodobacteraceae	G_uncultured	S_unknown
ASV.172	1769	K_Archaea	P_Euryarchaeota	C_Thermoplasmata	O_Thermoplasmatales	F_Marine Group III	G_unknown	S_unknown
ASV.146	1754	K_Bacteria	P_Bacteroidetes	C_Flavobacteriia	O_Flavobacteriales	F_Flavobacteriaceae	G_unknown	S_unknown
ASV.159	1738	K_Bacteria	P_Proteobacteria	C_Alphaproteobacteria	O_Rickettsiales	F_SAR116 clade	G_uncultured bacterium	S_unknown
ASV.224	1728	K_Bacteria	P_Cyanobacteria	C_Chloroplast	O_uncultured bacterium	F_unknown	G_unknown	S_unknown
ASV.175	1714	K_Bacteria	P_Proteobacteria	C_Alphaproteobacteria	O_Rhizobiales	F_Rhodobiaceae	G_Rhodobium	S_uncultured alpha proteobacteri
ASV.154	1698	K_Bacteria	P_Proteobacteria	C_Deltaproteobacteria	O_Bdellovibrionales	F_Bdellovibrionaceae	G_OM27 clade	S_uncultured delta proteobacteri
ASV.147	1683	K_Bacteria	P_Proteobacteria	C_Alphaproteobacteria	O_SAR11 clade	F_Surface 1	G_uncultured bacterium	S_unknown
ASV.137	1627	K_Bacteria	P_Proteobacteria	C_Alphaproteobacteria	O_Rickettsiales	F_SAR116 clade	G_uncultured bacterium	S_unknown
ASV.156	1608	K_Bacteria	P_Proteobacteria	C_Alphaproteobacteria	O_Rickettsiales	F_SAR116 clade	G_uncultured bacterium	S_unknown
ASV.164	1573	K_Bacteria	P_Proteobacteria	C_Alphaproteobacteria	O_Rhodospirillales	F_Rhodospirillaceae	G_uncultured	S_uncultured bacterium
ASV.160	1550	K_Bacteria	P_Proteobacteria	C_Alphaproteobacteria	O_SAR11 clade	F_Surface 4	G_uncultured bacterium	S_unknown
ASV.176	1530	K_Bacteria	P_Bacteroidetes	C_Bacteroidetes Incertae Se	O_Order III	F_Unknown Family	G_Balneola	S_uncultured bacterium
ASV.378	1500	K_Bacteria	P_Cyanobacteria	C_Cyanobacteria	O_SubsectionIII	F_FamilyI	G_uncultured	S_unknown
ASV.148	1499	K_Bacteria	P_Proteobacteria	C_Alphaproteobacteria	O_Rickettsiales	F_SAR116 clade	G_uncultured bacterium	S_unknown
ASV.167	1497	K_Bacteria	P_Proteobacteria	C_Alphaproteobacteria	O_Rickettsiales	F_SAR116 clade	G_uncultured bacterium	S_unknown
ASV.151	1497	K_Bacteria	P_Proteobacteria	C_Alphaproteobacteria	O_SAR11 clade	F_unknown	G_unknown	S_unknown
ASV.188	1490	K_Bacteria	P_Proteobacteria	C_Alphaproteobacteria	O_Rickettsiales	F_SAR116 clade	G_uncultured bacterium	S_unknown
ASV.155	1490	K_Bacteria	P_Bacteroidetes	C_Cytophagia	O_Cytophagales	F_Flammeovirgaceae	G_Marinoscillum	S_uncultured bacterium
ASV.150	1488	K_Bacteria	P_Proteobacteria	C_Alphaproteobacteria	O_Rickettsiales	F_S25-593	G_uncultured bacterium	S_unknown
ASV.169	1476	K_Bacteria	P_Bacteroidetes	C_Flavobacteriia	O_Flavobacteriales	F_Flavobacteriaceae	G_unknown	S_unknown
ASV.170	1475	K_Bacteria	P_Proteobacteria	C_Alphaproteobacteria	O_Rickettsiales	F_S25-593	G_uncultured bacterium	S_unknown
ASV.356	1419	K_Bacteria	P_Cyanobacteria	C_Chloroplast	O_uncultured bacterium	F_unknown	G_unknown	S_unknown
ASV.380	1417	K_Bacteria	P_Cyanobacteria	C_Cyanobacteria	O_SubsectionI	F_FamilyI	G_Prochlorococcus	S_uncultured bacterium
ASV.180	1410	K_Archaea	P_Euryarchaeota	C_Thermoplasmata	O_Thermoplasmatales	F_Marine Group II	G_uncultured marine group II euryar	S_unknown
ASV.179	1398	K_Bacteria	P_Chloroflexi	C_SAR202 clade	O_marine metagenome	F_unknown	G_unknown	S_unknown
ASV.168	1395	K_Bacteria	P_Proteobacteria	C_Alphaproteobacteria	O_Rhodospirillales	F_Rhodospirillaceae	G_OM75 clade	S_uncultured bacterium
ASV.173	1395	K_Bacteria	P_Cyanobacteria	C_Chloroplast	O_uncultured marine eukaryote	F_unknown	G_unknown	S_unknown
ASV.209	1385	K_Bacteria	P_Proteobacteria	C_Deltaproteobacteria	O_unknown	F_unknown	G_unknown	S_unknown
ASV.181	1380	K_Bacteria	P_Verrucomicrobia	C_Opitutae	O_Puniceicoccales	F_Puniceicoccaceae	G_Pelagicoccus	S_unknown
ASV.184	1353	K_Bacteria	P_Proteobacteria	C_Alphaproteobacteria	O_Rhodospirillales	F_Rhodospirillaceae	G_AEGEAN-169 marine group	S_uncultured bacterium
ASV.192	1352	K_Bacteria	P_Bacteroidetes	C_Flavobacteriia	O_Flavobacteriales	F_NS9 marine group	G_uncultured marine bacterium	S_unknown
ASV.161	1350	K_Bacteria	P_Proteobacteria	C_SPOTSOCT00m83	O_uncultured bacterium	F_unknown	G_unknown	S_unknown
ASV.177	1347	K_Bacteria	P_Cyanobacteria	C_Chloroplast	O_uncultured bacterium	F_unknown	G_unknown	S_unknown
ASV.258	1339	K_Archaea	P_Euryarchaeota	C_Thermoplasmata	O_Thermoplasmatales	F_Marine Group II	G_unknown	S_unknown
ASV.185	1334	K_Archaea	P_Euryarchaeota	C_Thermoplasmata	O_Thermoplasmatales	F_Marine Group II	G_unknown	S_unknown
ASV.165	1333	K_Bacteria	P_Proteobacteria	C_Gammaproteobacteria	O_Oceanospirillales	F_OM182 clade	G_uncultured bacterium	S_unknown
ASV.182	1295	K_Bacteria	P_Bacteroidetes	C_Flavobacteriia	O_Flavobacteriales	F_Flavobacteriaceae	G_NS5 marine group	S_uncultured bacterium
ASV.157	1274	K_Bacteria	P_Proteobacteria	C_Alphaproteobacteria	O_SAR11 clade	F_Surface 2	G_uncultured bacterium	S_unknown
ASV.162	1273	K_Bacteria	P_Proteobacteria	C_Alphaproteobacteria	O_SAR11 clade	F_Surface 2	G_uncultured bacterium	S_unknown
ASV.174	1268	K_Bacteria	P_Proteobacteria	C_Gammaproteobacteria	O_Oceanospirillales	F_OM182 clade	G_uncultured bacterium	S_unknown
ASV.193	1265	K_Bacteria	P_Proteobacteria	C_Alphaproteobacteria	O_Rhodobacterales	F_Rhodobacteraceae	G_unknown	S_unknown
ASV.382	1251	K_Bacteria	P_Cyanobacteria	C_Chloroplast	O_uncultured bacterium	F_unknown	G_unknown	S_unknown
ASV.205	1231	K_Bacteria	P_Proteobacteria	C_Deltaproteobacteria	O_Bdellovibrionales	F_Bdellovibrionaceae	G_OM27 clade	S_uncultured bacterium
ASV.187	1229	K_Bacteria	P_Proteobacteria	C_Alphaproteobacteria	O_SAR11 clade	F_Surface 2	G_uncultured bacterium	S_unknown
ASV.171	1215	K_Bacteria	P_Verrucomicrobia	C_Opitutae	O_Puniceicoccales	F_Puniceicoccaceae	G_marine group	S_uncultured bacterium
ASV.201	1212	K_Bacteria	P_Proteobacteria	C_Alphaproteobacteria	O_Rickettsiales	F_SAR116 clade	G_uncultured bacterium	S_unknown
ASV.191	1205	K_Bacteria	P_Proteobacteria	C_Alphaproteobacteria	O_SAR11 clade	F_unknown	G_unknown	S_unknown
ASV.178	1201	K_Bacteria	P_Proteobacteria	C_Alphaproteobacteria	O_Rhodospirillales	F_Rhodospirillaceae	G_uncultured	S_uncultured bacterium

ASV.198	1196	K_Bacteria	P_Proteobacteria	C_Alphaproteobacteria	O_Rhodospirillales	F_Rhodospirillaceae	G_uncultured	S_uncultured bacterium
ASV.189	1158	K_Bacteria	P_Proteobacteria	C_Alphaproteobacteria	O_SAR11 clade	F_unknown	G_unknown	S_unknown
ASV.183	1155	K_Bacteria	P_Proteobacteria	C_Alphaproteobacteria	O_SAR11 clade	F_Surface 1	G_unknown	S_unknown
ASV.212	1155	K_Bacteria	P_Bacteroidetes	C_Flavobacteriia	O_Flavobacteriales	F_Flavobacteriaceae	G_NS5 marine group	S_uncultured bacterium
ASV.196	1143	K_Bacteria	P_Proteobacteria	C_Alphaproteobacteria	O_SAR11 clade	F_uncultured bacterium	G_unknown	S_unknown
ASV.194	1141	K_Bacteria	P_Bacteroidetes	C_Flavobacteriia	O_Flavobacteriales	F_Flavobacteriaceae	G_NS4 marine group	S_uncultured bacterium
ASV.197	1139	K_Bacteria	P_Proteobacteria	C_Alphaproteobacteria	O_Rickettsiales	F_SAR116 clade	G_uncultured bacterium	S_unknown
ASV.222	1102	K_Archaea	P_Euryarchaeota	C_Thermoplasmata	O_Thermoplasmatales	F_Marine Group II	G_unknown	S_unknown
ASV.207	1096	K_Bacteria	P_Cyanobacteria	C_Chloroplast	O_uncultured bacterium	F_unknown	G_unknown	S_unknown
ASV.216	1096	K_Bacteria	P_Actinobacteria	C_Acidimicrobiia	O_Acidimicrobiales	F_Sva0996 marine group	G_uncultured bacterium	S_unknown
ASV.203	1095	K_Bacteria	P_Proteobacteria	C_Deltaproteobacteria	O_Bradymonadales	F_uncultured delta proteoba	G_unknown	S_unknown
ASV.231	1087	K_Bacteria	P_Bacteroidetes	C_Flavobacteriia	O_Flavobacteriales	F_Cryomorphaceae	G_uncultured	S_uncultured Owenweeksia sp.
ASV.202	1085	K_Bacteria	P_Proteobacteria	C_Alphaproteobacteria	O_Rhodospirillales	F_Rhodospirillaceae	G_AEGEAN-169 marine group	S_uncultured bacterium
ASV.195	1083	K_Bacteria	P_Proteobacteria	C_Alphaproteobacteria	O_Rickettsiales	F_SAR116 clade	G_uncultured marine bacterium	S_unknown
ASV.383	1071	K_Bacteria	P_Cyanobacteria	C_Cyanobacteria	O_SubsectionI	F_FamilyI	G_Prochlorococcus	S_uncultured bacterium
ASV.204	1068	K_Bacteria	P_Proteobacteria	C_Gammaproteobacteria	O_Legionellales	F_Coxiellaceae	G_Coxiella	S_unknown
ASV.200	1065	K_Bacteria	P_Proteobacteria	C_Alphaproteobacteria	O_Rhodospirillales	F_Rhodospirillaceae	G_uncultured	S_uncultured bacterium
ASV.210	1058	K_Bacteria	P_Proteobacteria	C_Alphaproteobacteria	O_Rhodospirillales	F_Rhodospirillaceae	G_uncultured	S_uncultured bacterium
ASV.385	1054	K_Bacteria	P_Proteobacteria	C_Deltaproteobacteria	O_SAR324 clade(Marine group B)	F_uncultured bacterium	G_unknown	S_unknown
ASV.238	1049	K_Bacteria	P_Bacteroidetes	C_Flavobacteriia	O_Flavobacteriales	F_NS9 marine group	G_uncultured Flavobacteriales bacte	S_unknown
ASV.225	1033	K_Bacteria	P_Bacteroidetes	C_Flavobacteriia	O_Flavobacteriales	F_Flavobacteriaceae	G_NS4 marine group	S_uncultured bacterium
ASV.208	1030	K_Bacteria	P_Bacteroidetes	C_Flavobacteriia	O_Flavobacteriales	F_NS9 marine group	G_uncultured bacterium	S_unknown
ASV.221	1029	K_Bacteria	P_Bacteroidetes	C_Flavobacteriia	O_Flavobacteriales	F_unknown	G_unknown	S_unknown
ASV.213	1022	K_Bacteria	P_Cyanobacteria	C_ML635J-21	O_uncultured bacterium	F_unknown	G_unknown	S_unknown
ASV.214	994	K_Bacteria	P_Proteobacteria	C_Alphaproteobacteria	O_Rhodospirillales	F_Rhodospirillaceae	G_AEGEAN-169 marine group	S_uncultured bacterium
ASV.218	987	K_Bacteria	P_Verrucomicrobia	C_Opitutae	O_Puniceicoccales	F_Puniceicoccaceae	G_Pelagicoccus	S_unknown
ASV.262	986	K_Bacteria	P_Actinobacteria	C_Actinobacteria	O_PeM15	F_uncultured bacterium	G_unknown	S_unknown
ASV.166	972	K_Bacteria	P_Proteobacteria	C_Alphaproteobacteria	O_SAR11 clade	F_Surface 1	G_uncultured bacterium	S_unknown
ASV.219	947	K_Bacteria	P_Proteobacteria	C_Alphaproteobacteria	O_Rhodospirillales	F_Rhodospirillaceae	G_uncultured	S_unknown
ASV.271	947	K_Bacteria	P_Proteobacteria	C_Deltaproteobacteria	O_Bdellovibrionales	F_Bdellovibrionaceae	G_OM27 clade	S_uncultured bacterium
ASV.217	931	K_Bacteria	P_Proteobacteria	C_Alphaproteobacteria	O_Rhodospirillales	F_Rhodospirillaceae	G_AEGEAN-169 marine group	S_uncultured bacterium
ASV.266	925	K_Bacteria	P_Bacteroidetes	C_Flavobacteriia	O_Flavobacteriales	F_NS9 marine group	G_uncultured bacterium	S_unknown
ASV.223	921	K_Bacteria	P_Proteobacteria	C_Alphaproteobacteria	O_Rhodospirillales	F_Rhodospirillaceae	G_uncultured	S_uncultured bacterium
ASV.220	921	K_Bacteria	P_Proteobacteria	C_Alphaproteobacteria	O_SAR11 clade	F_Surface 1	G_uncultured bacterium	S_unknown
ASV.206	919	K_Bacteria	P_Proteobacteria	C_Alphaproteobacteria	O_Rickettsiales	F_SAR116 clade	G_uncultured marine bacterium	S_unknown
ASV.386	892	K_Bacteria	P_Proteobacteria	C_Alphaproteobacteria	O_Rhodospirillales	F_Rhodospirillaceae	G_uncultured	S_uncultured bacterium
ASV.240	891	K_Bacteria	P_Bacteroidetes	C_Flavobacteriia	O_Flavobacteriales	F_Flavobacteriaceae	G_NS4 marine group	S_unknown
ASV.233	891	K_Bacteria	P_Bacteroidetes	C_Flavobacteriia	O_Flavobacteriales	F_Flavobacteriaceae	G_uncultured	S_uncultured bacterium
ASV.229	886	K_Bacteria	P_Proteobacteria	C_Alphaproteobacteria	O_SAR11 clade	F_unknown	G_unknown	S_unknown
ASV.234	871	K_Bacteria	P_Proteobacteria	C_Gammaproteobacteria	O_Oceanospirillales	F_OM182 clade	G_unknown	S_unknown
ASV.226	849	K_Bacteria	P_Planctomycetes	C_Planctomycetacia	O_Planctomycetales	F_Planctomycetaceae	G_Pir4 lineage	S_unknown
ASV.199	844	K_Bacteria	P_Proteobacteria	C_Alphaproteobacteria	O_Rhizobiales	F_PS1 clade	G_uncultured bacterium	S_unknown
ASV.241	842	K_Bacteria	P_Bacteroidetes	C_Flavobacteriia	O_Flavobacteriales	F_Flavobacteriaceae	G_NS5 marine group	S_uncultured bacterium
ASV.235	836	K_Bacteria	P_Proteobacteria	C_Alphaproteobacteria	O_Rhodospirillales	F_Rhodospirillaceae	G_AEGEAN-169 marine group	S_uncultured bacterium
ASV.387	824	K_Bacteria	P_Bacteroidetes	C_Cytophagia	O_Cytophagales	F_Flammeovirgaceae	G_Marinoscillum	S_uncultured bacterium
ASV.227	818	K_Bacteria	P_Bacteroidetes	C_Flavobacteriia	O_Flavobacteriales	F_Flavobacteriaceae	G_NS5 marine group	S_uncultured bacterium
ASV.236	815	K_Bacteria	P_Bacteroidetes	C_Flavobacteriia	O_Flavobacteriales	F_Flavobacteriaceae	G_NS5 marine group	S_uncultured bacterium
ASV.228	811	K_Bacteria	P_Proteobacteria	C_Deltaproteobacteria	O_Bradymonadales	F_uncultured delta proteoba	G_unknown	S_unknown
ASV.296	809	K_Bacteria	P_Bacteroidetes	C_Flavobacteriia	O_Flavobacteriales	F_Flavobacteriaceae	G_NS5 marine group	S_uncultured bacterium
ASV.232	806	K_Bacteria	P_Cyanobacteria	C_ML635J-21	O_uncultured bacterium	F_unknown	G_unknown	S_unknown

ASV.190	796	K_Bacteria	P_Cyanobacteria	C_Cyanobacteria	O_SubsectionI	F_FamilyI	G_Prochlorococcus	S_uncultured bacterium
ASV.388	778	K_Bacteria	P_Proteobacteria	C_Alphaproteobacteria	O_SAR11 clade	F_Surface 1	G_uncultured bacterium	S_unknown
ASV.254	762	K_Bacteria	P_Bacteroidetes	C_Sphingobacteriia	O_Sphingobacteriales	F_Saprospiraceae	G_uncultured	S_unknown
ASV.230	757	K_Bacteria	P_Bacteroidetes	C_Flavobacteriia	O_Flavobacteriales	F_NS9 marine group	G_uncultured bacterium	S_unknown
ASV.211	744	K_Bacteria	P_Proteobacteria	C_Alphaproteobacteria	O_Rickettsiales	F_SAR116 clade	G_uncultured marine bacterium	S_unknown
ASV.250	735	K_Bacteria	P_Proteobacteria	C_Alphaproteobacteria	O_SAR11 clade	F_Chesapeake-Delaware Bay	G_uncultured bacterium	S_unknown
ASV.243	725	K_Bacteria	P_Proteobacteria	C_Alphaproteobacteria	O_Rickettsiales	F_SAR116 clade	G_uncultured bacterium	S_unknown
ASV.239	722	K_Bacteria	P_Proteobacteria	C_Alphaproteobacteria	O_Rhizobiales	F_PS1 clade	G_uncultured bacterium	S_unknown
ASV.244	719	K_Bacteria	P_Proteobacteria	C_Alphaproteobacteria	O_Rhodospirillales	F_Rhodospirillaceae	G_AEGEAN-169 marine group	S_uncultured bacterium
ASV.263	718	K_Bacteria	P_Bacteroidetes	C_Flavobacteriia	O_Flavobacteriales	F_Flavobacteriaceae	G_NS5 marine group	S_uncultured bacterium
ASV.245	714	K_Bacteria	P_Bacteroidetes	C_Flavobacteriia	O_Flavobacteriales	F_Cryomorphaceae	G_uncultured	S_unknown
ASV.255	702	K_Bacteria	P_Proteobacteria	C_Alphaproteobacteria	O_Rickettsiales	F_SAR116 clade	G_uncultured marine bacterium	S_unknown
ASV.278	701	K_Bacteria	P_Bacteroidetes	C_Flavobacteriia	O_Flavobacteriales	F_Flavobacteriaceae	G_NS4 marine group	S_uncultured bacterium
ASV.389	695	K_Bacteria	P_Bacteroidetes	C_Flavobacteriia	O_Flavobacteriales	F_Cryomorphaceae	G_uncultured	S_uncultured Owenweeksia sp.
ASV.246	695	K_Bacteria	P_Cyanobacteria	C_Chloroplast	O_uncultured bacterium	F_unknown	G_unknown	S_unknown
ASV.267	688	K_Bacteria	P_Proteobacteria	C_Alphaproteobacteria	O_Rhodospirillales	F_Rhodospirillaceae	G_AEGEAN-169 marine group	S_uncultured bacterium
ASV.247	682	K_Bacteria	P_Proteobacteria	C_Alphaproteobacteria	O_Rhizobiales	F_PS1 clade	G_uncultured bacterium	S_unknown
ASV.215	680	K_Bacteria	P_Proteobacteria	C_Alphaproteobacteria	O_SAR11 clade	F_Surface 1	G_uncultured bacterium	S_unknown
ASV.251	678	K_Bacteria	P_Bacteroidetes	C_Flavobacteriia	O_Flavobacteriales	F_Flavobacteriaceae	G_NS5 marine group	S_uncultured bacterium
ASV.320	672	K_Bacteria	P_Proteobacteria	C_Gammaproteobacteria	O_Oceanospirillales	F_OM182 clade	G_uncultured bacterium	S_unknown
ASV.237	661	K_Bacteria	P_Proteobacteria	C_Alphaproteobacteria	O_Rickettsiales	F_SAR116 clade	G_uncultured bacterium	S_unknown
ASV.390	647	K_Archaea	P_Euryarchaeota	C_Thermoplasmata	O_Thermoplasmatales	F_Marine Group II	G_unknown	S_unknown
ASV.269	647	K_Bacteria	P_Proteobacteria	C_Alphaproteobacteria	O_Rhodospirillales	F_Rhodospirillaceae	G_AEGEAN-169 marine group	S_uncultured bacterium
ASV.268	642	K_Bacteria	P_Cyanobacteria	C_Chloroplast	O_uncultured diatom	F_unknown	G_unknown	S_unknown
ASV.391	637	K_Bacteria	P_Proteobacteria	C_Alphaproteobacteria	O_SAR11 clade	F_unknown	G_unknown	S_unknown
ASV.242	631	K_Bacteria	P_Proteobacteria	C_Alphaproteobacteria	O_Rhodospirillales	F_Rhodospirillaceae	G_AEGEAN-169 marine group	S_uncultured bacterium
ASV.252	620	K_Bacteria	P_Cyanobacteria	C_Chloroplast	O_uncultured bacterium	F_unknown	G_unknown	S_unknown
ASV.259	612	K_Bacteria	P_Chloroflexi	C_SAR202 clade	O_marine metagenome	F_unknown	G_unknown	S_unknown
ASV.392	602	K_Bacteria	P_Proteobacteria	C_Alphaproteobacteria	O_SAR11 clade	F_Surface 2	G_uncultured bacterium	S_unknown
ASV.393	595	K_Bacteria	P_Proteobacteria	C_Alphaproteobacteria	O_Rickettsiales	F_SAR116 clade	G_uncultured bacterium	S_unknown
ASV.272	588	K_Bacteria	P_Gracilibacteria	C_uncultured organism	O_unknown	F_unknown	G_unknown	S_unknown
ASV.290	588	K_Archaea	P_Euryarchaeota	C_Thermoplasmata	O_Thermoplasmatales	F_Marine Group II	G_unknown	S_unknown
ASV.302	582	K_Bacteria	P_Proteobacteria	C_Alphaproteobacteria	O_Rickettsiales	F_Mitochondria	G_marine metagenome	S_unknown
ASV.253	582	K_Bacteria	P_Proteobacteria	C_Deltaproteobacteria	O_Bradymonadales	F_uncultured delta proteoba	G_unknown	S_unknown
ASV.394	579	K_Archaea	P_Euryarchaeota	C_Thermoplasmata	O_Thermoplasmatales	F_Marine Group II	G_uncultured marine group II euryar	S_unknown
ASV.299	572	K_Bacteria	P_Bacteroidetes	C_Flavobacteriia	O_Flavobacteriales	F_NS9 marine group	G_unknown	S_unknown
ASV.279	571	K_Bacteria	P_Proteobacteria	C_Alphaproteobacteria	O_Rhodospirillales	F_Rhodospirillaceae	G_AEGEAN-169 marine group	S_uncultured bacterium
ASV.256	562	K_Bacteria	P_Proteobacteria	C_Alphaproteobacteria	O_Rhodospirillales	F_Rhodospirillaceae	G_uncultured	S_uncultured bacterium
ASV.257	559	K_Bacteria	P_Proteobacteria	C_Alphaproteobacteria	O_SAR11 clade	F_Surface 1	G_uncultured bacterium	S_unknown
ASV.270	552	K_Bacteria	P_Bacteroidetes	C_Flavobacteriia	O_Flavobacteriales	F_Flavobacteriaceae	G_NS2b marine group	S_uncultured bacterium
ASV.280	551	K_Bacteria	P_Bacteroidetes	C_Flavobacteriia	O_Flavobacteriales	F_Flavobacteriaceae	G_NS5 marine group	S_uncultured bacterium
ASV.395	544	K_Archaea	P_Euryarchaeota	C_Thermoplasmata	O_Thermoplasmatales	F_Marine Group II	G_uncultured marine group II euryar	S_unknown
ASV.297	536	K_Bacteria	P_Proteobacteria	C_Deltaproteobacteria	O_Bdellovibrionales	F_Bdellovibrionaceae	G_OM27 clade	S_uncultured delta proteobacteri
ASV.281	534	K_Bacteria	P_Bacteroidetes	C_Flavobacteriia	O_Flavobacteriales	F_Flavobacteriaceae	G_Aquibacter	S_uncultured bacterium
ASV.264	530	K_Bacteria	P_Proteobacteria	C_Alphaproteobacteria	O_Rhodospirillales	F_Rhodospirillaceae	G_AEGEAN-169 marine group	S_uncultured bacterium
ASV.275	524	K_Bacteria	P_Proteobacteria	C_Alphaproteobacteria	O_Rhodospirillales	F_Rhodospirillaceae	G_AEGEAN-169 marine group	S_uncultured bacterium
ASV.273	516	K_Bacteria	P_Bacteroidetes	C_Flavobacteriia	O_Flavobacteriales	F_Flavobacteriaceae	G_Tenacibaculum	S_uncultured bacterium
ASV.248	513	K_Bacteria	P_Proteobacteria	C_Alphaproteobacteria	O_Rhodospirillales	F_Rhodospirillaceae	G_AEGEAN-169 marine group	S_uncultured bacterium
ASV.276	512	K_Bacteria	P_Proteobacteria	C_Alphaproteobacteria	O_SAR11 clade	F_Surface 1	G_uncultured bacterium	S_unknown
ASV.260	512	K_Bacteria	P_Bacteroidetes	C_Flavobacteriia	O_Flavobacteriales	F_Flavobacteriaceae	G_NS5 marine group	S_uncultured bacterium

ASV.291	502	K_Bacteria	P_Proteobacteria	C_Gammaproteobacteria	O_Oceanospirillales	F_OM182 clade	G_uncultured gamma proteobacteriu	S_unknown
ASV.265	496	K_Bacteria	P_Bacteroidetes	C_Flavobacteriia	O_Flavobacteriales	F_Flavobacteriaceae	G_NS4 marine group	S_unknown
ASV.277	494	K_Bacteria	P_Cyanobacteria	C_ML635J-21	O_uncultured bacterium	F_unknown	G_unknown	S_unknown
ASV.249	492	K_Bacteria	P_Proteobacteria	C_Alphaproteobacteria	O_Rhodospirillales	F_Rhodospirillaceae	G_AEGEAN-169 marine group	S_uncultured bacterium
ASV.285	486	K_Bacteria	P_Proteobacteria	C_Alphaproteobacteria	O_Rhodospirillales	F_Rhodospirillaceae	G_AEGEAN-169 marine group	S_uncultured bacterium
ASV.284	481	K_Bacteria	P_Proteobacteria	C_Deltaproteobacteria	O_Bradymonadales	F_uncultured bacterium	G_unknown	S_unknown
ASV.274	480	K_Bacteria	P_Proteobacteria	C_Alphaproteobacteria	O_SAR11 clade	F_Chesapeake-Delaware Bay	G_uncultured bacterium	S_unknown
ASV.396	480	K_Bacteria	P_Bacteroidetes	C_Flavobacteriia	O_Flavobacteriales	F_Flavobacteriaceae	G_NS4 marine group	S_uncultured bacterium
ASV.286	476	K_Bacteria	P_Proteobacteria	C_Deltaproteobacteria	O_Bradymonadales	F_uncultured bacterium	G_unknown	S_unknown
ASV.287	470	K_Bacteria	P_Proteobacteria	C_Alphaproteobacteria	O_Rhodospirillales	F_Rhodospirillaceae	G_AEGEAN-169 marine group	S_uncultured bacterium
ASV.397	469	K_Bacteria	P_Proteobacteria	C_Alphaproteobacteria	O_Rhizobiales	F_PS1 clade	G_uncultured bacterium	S_unknown
ASV.300	463	K_Bacteria	P_Proteobacteria	C_Alphaproteobacteria	O_Rhodospirillales	F_Rhodospirillaceae	G_AEGEAN-169 marine group	S_uncultured bacterium
ASV.261	453	K_Bacteria	P_Proteobacteria	C_Alphaproteobacteria	O_Rickettsiales	F_SAR116 clade	G_uncultured marine bacterium	S_unknown
ASV.301	453	K_Bacteria	P_Proteobacteria	C_Alphaproteobacteria	O_Rickettsiales	F_S25-593	G_uncultured bacterium	S_unknown
ASV.398	447	K_Archaea	P_Euryarchaeota	C_Thermoplasmata	O_Thermoplasmatales	F_Marine Group II	G_uncultured archaeon	S_unknown
ASV.399	447	K_Bacteria	P_Verrucomicrobia	C_Opitutae	O_Puniceicoccales	F_Puniceicoccaceae	G_marine group	S_uncultured bacterium
ASV.282	445	K_Bacteria	P_Bacteroidetes	C_Flavobacteriia	O_Flavobacteriales	F_NS9 marine group	G_uncultured bacterium	S_unknown
ASV.292	442	K_Bacteria	P_Proteobacteria	C_Alphaproteobacteria	O_Rhodospirillales	F_Rhodospirillaceae	G_AEGEAN-169 marine group	S_uncultured bacterium
ASV.283	437	K_Bacteria	P_Proteobacteria	C_Alphaproteobacteria	O_Rickettsiales	F_SAR116 clade	G_uncultured bacterium	S_unknown
ASV.293	435	K_Bacteria	P_Bacteroidetes	C_Flavobacteriia	O_Flavobacteriales	F_Flavobacteriaceae	G_NS5 marine group	S_uncultured bacterium
ASV.400	429	K_Bacteria	P_Bacteroidetes	C_Flavobacteriia	O_Flavobacteriales	F_NS9 marine group	G_hydrothermal vent metagenome	S_unknown
ASV.294	427	K_Bacteria	P_Proteobacteria	C_Alphaproteobacteria	O_SAR11 clade	F_unknown	G_unknown	S_unknown
ASV.304	421	K_Bacteria	P_Cyanobacteria	C_Chloroplast	O_unknown	F_unknown	G_unknown	S_unknown
ASV.288	417	K_Bacteria	P_Verrucomicrobia	C_OPB35 soil group	O_unknown	F_unknown	G_unknown	S_unknown
ASV.375	395	K_Bacteria	P_Bacteroidetes	C_Flavobacteriia	O_Flavobacteriales	F_NS9 marine group	G_uncultured marine bacterium	S_unknown
ASV.343	372	K_Bacteria	P_Bacteroidetes	C_Flavobacteriia	O_Flavobacteriales	F_Flavobacteriaceae	G_Muricauda	S_uncultured bacterium
ASV.376	369	K_Bacteria	P_Bacteroidetes	C_Flavobacteriia	O_Flavobacteriales	F_Cryomorphaceae	G_uncultured	S_uncultured Owenweeksia sp.
ASV.401	368	K_Bacteria	P_Bacteroidetes	C_Flavobacteriia	O_Flavobacteriales	F_Flavobacteriaceae	G_NS4 marine group	S_uncultured bacterium
ASV.295	366	K_Bacteria	P_Cyanobacteria	C_Chloroplast	O_uncultured bacterium	F_unknown	G_unknown	S_unknown
ASV.298	363	K_Bacteria	P_Proteobacteria	C_Deltaproteobacteria	O_Bradymonadales	F_uncultured delta proteoba	G_unknown	S_unknown
ASV.303	356	K_Bacteria	P_Bacteroidetes	C_Flavobacteriia	O_Flavobacteriales	F_NS9 marine group	G_uncultured bacterium	S_unknown
ASV.310	355	K_Bacteria	P_Gracilibacteria	C_uncultured organism	O_unknown	F_unknown	G_unknown	S_unknown
ASV.316	354	K_Bacteria	P_Proteobacteria	C_Alphaproteobacteria	O_Rickettsiales	F_Mitochondria	G_uncultured bacterium	S_unknown
ASV.321	353	K_Bacteria	P_Proteobacteria	C_Deltaproteobacteria	O_Bdellovibrionales	F_Bdellovibrionaceae	G_OM27 clade	S_uncultured bacterium
ASV.306	350	K_Bacteria	P_Bacteroidetes	C_Flavobacteriia	O_Flavobacteriales	F_Flavobacteriaceae	G_Tenacibaculum	S_uncultured bacterium
ASV.311	349	K_Bacteria	P_Bacteroidetes	C_Flavobacteriia	O_Flavobacteriales	F_Flavobacteriaceae	G_Ulvibacter	S_uncultured bacterium
ASV.313	348	K_Bacteria	P_Cyanobacteria	C_Chloroplast	O_uncultured bacterium	F_unknown	G_unknown	S_unknown
ASV.317	348	K_Bacteria	P_Cyanobacteria	C_Chloroplast	O_uncultured diatom	F_unknown	G_unknown	S_unknown
ASV.402	347	K_Archaea	P_Euryarchaeota	C_Thermoplasmata	O_Thermoplasmatales	F_Marine Group II	G_uncultured archaeon	S_unknown
ASV.307	345	K_Bacteria	P_Proteobacteria	C_Alphaproteobacteria	O_Rickettsiales	F_S25-593	G_uncultured bacterium	S_unknown
ASV.289	344	K_Bacteria	P_Proteobacteria	C_Alphaproteobacteria	O_SAR11 clade	F_Surface 1	G_uncultured bacterium	S_unknown
ASV.403	342	K_Bacteria	P_Proteobacteria	C_Alphaproteobacteria	O_SAR11 clade	F_Surface 1	G_uncultured bacterium	S_unknown
ASV.404	342	K_Bacteria	P_Bacteroidetes	C_Flavobacteriia	O_Flavobacteriales	F_Flavobacteriaceae	G_NS4 marine group	S_uncultured bacterium
ASV.405	340	K_Bacteria	P_Proteobacteria	C_Gammaproteobacteria	O_Oceanospirillales	F_OM182 clade	G_uncultured bacterium	S_unknown
ASV.406	338	K_Bacteria	P_Bacteroidetes	C_Flavobacteriia	O_Flavobacteriales	F_Flavobacteriaceae	G_NS5 marine group	S_uncultured bacterium
ASV.308	336	K_Bacteria	P_Proteobacteria	C_Alphaproteobacteria	O_Rhodospirillales	F_Rhodospirillaceae	G_uncultured	S_unknown
ASV.318	335	K_Bacteria	P_Planctomycetes	C_Planctomycetacia	O_Planctomycetales	F_Planctomycetaceae	G_Rubripirellula	S_unknown
ASV.314	335	K_Bacteria	P_Proteobacteria	C_Alphaproteobacteria	O_Alphaproteobacteria Incertae Sed	F_Unknown Family	G_uncultured	S_uncultured bacterium
ASV.407	331	K_Bacteria	P_Proteobacteria	C_Alphaproteobacteria	O_Rhodospirillales	F_Rhodospirillaceae	G_OM75 clade	S_uncultured bacterium
ASV.408	330	K_Bacteria	P_Cyanobacteria	C_Chloroplast	O_uncultured bacterium	F_unknown	G_unknown	S_unknown

ASV.322	329	K_Bacteria	P_Proteobacteria	C_Deltaproteobacteria	O_Bdellovibrionales	F_Bdellovibrionaceae	G_OM27 clade	S_uncultured bacterium
ASV.327	328	K_Bacteria	P_Proteobacteria	C_Deltaproteobacteria	O_Bdellovibrionales	F_Bdellovibrionaceae	G_OM27 clade	S_uncultured delta proteobacteri
ASV.409	328	K_Bacteria	P_Cyanobacteria	C_Chloroplast	O_uncultured bacterium	F_unknown	G_unknown	S_unknown
ASV.410	325	K_Bacteria	P_Proteobacteria	C_Alphaproteobacteria	O_SAR11 clade	F_Surface 1	G_unknown	S_unknown
ASV.411	322	K_Bacteria	P_Planctomycetes	C_Planctomycetacia	O_Planctomycetales	F_Planctomycetaceae	G_Pir4 lineage	S_unknown
ASV.412	320	K_Bacteria	P_Proteobacteria	C_Gammaproteobacteria	O_Thiotrichales	F_Thiotrichaceae	G_Thiothrix	S_unknown
ASV.305	315	K_Bacteria	P_Proteobacteria	C_Alphaproteobacteria	O_Rickettsiales	F_SAR116 clade	G_uncultured bacterium	S_unknown
ASV.331	314	K_Bacteria	P_Bacteroidetes	C_Flavobacteriia	O_Flavobacteriales	F_Flavobacteriaceae	G_Ulvibacter	S_uncultured bacterium
ASV.413	309	K_Bacteria	P_Proteobacteria	C_Gammaproteobacteria	O_Thiotrichales	F_Thiotrichaceae	G_Thiothrix	S_unknown
ASV.414	308	K_Bacteria	P_Proteobacteria	C_Alphaproteobacteria	O_Rickettsiales	F_S25-593	G_uncultured bacterium	S_unknown
ASV.415	303	K_Bacteria	P_Bacteroidetes	C_Flavobacteriia	O_Flavobacteriales	F_Cryomorphaceae	G_uncultured	S_uncultured Owenweeksia sp.
ASV.416	303	K_Bacteria	P_Proteobacteria	C_Alphaproteobacteria	O_SAR11 clade	F_Surface 1	G_uncultured bacterium	S_unknown
ASV.324	300	K_Bacteria	P_Bacteroidetes	C_Sphingobacteriia	O_Sphingobacteriales	F_Saprospiraceae	G_uncultured	S_uncultured Bacteroidetes bacte
ASV.315	299	K_Bacteria	P_Bacteroidetes	C_Flavobacteriia	O_Flavobacteriales	F_Flavobacteriaceae	G_NS5 marine group	S_uncultured bacterium
ASV.312	298	K_Bacteria	P_Proteobacteria	C_Alphaproteobacteria	O_Rickettsiales	F_S25-593	G_uncultured bacterium	S_unknown
ASV.417	293	K_Bacteria	P_Bacteroidetes	C_Flavobacteriia	O_Flavobacteriales	F_Flavobacteriaceae	G_Pseudofulvibacter	S_uncultured bacterium
ASV.323	292	K_Bacteria	P_Cyanobacteria	C_Chloroplast	O_uncultured bacterium	F_unknown	G_unknown	S_unknown
ASV.309	292	K_Bacteria	P_Proteobacteria	C_Alphaproteobacteria	O_Rhodospirillales	F_Rhodospirillaceae	G_AEGEAN-169 marine group	S_uncultured bacterium
ASV.319	291	K_Bacteria	P_Proteobacteria	C_Deltaproteobacteria	O_Bradymonadales	F_uncultured delta proteoba	G_unknown	S_unknown
ASV.418	290	K_Bacteria	P_Cyanobacteria	C_Cyanobacteria	O_SubsectionI	F_FamilyI	G_Prochlorococcus	S_uncultured bacterium
ASV.328	288	K_Bacteria	P_Bacteroidetes	C_Flavobacteriia	O_Flavobacteriales	F_Schleiferiaceae	G_Schleiferia	S_uncultured bacterium
ASV.419	287	K_Archaea	P_Euryarchaeota	C_Thermoplasmata	O_Thermoplasmatales	F_Marine Group II	G_unknown	S_unknown
ASV.337	283	K_Bacteria	P_Bacteroidetes	C_Flavobacteriia	O_Flavobacteriales	F_Flavobacteriaceae	G_Tenacibaculum	S_uncultured bacterium
ASV.332	283	K_Bacteria	P_Planctomycetes	C_Planctomycetacia	O_Planctomycetales	F_Planctomycetaceae	G_Blastopirellula	S_uncultured planctomycete
ASV.333	282	K_Bacteria	P_Saccharibacteria	C_uncultured bacterium	O_unknown	F_unknown	G_unknown	S_unknown
ASV.420	282	K_Bacteria	P_Proteobacteria	C_Deltaproteobacteria	O_SAR324 clade(Marine group B)	F_uncultured bacterium	G_unknown	S_unknown
ASV.329	280	K_Bacteria	P_Cyanobacteria	C_Chloroplast	O_uncultured bacterium	F_unknown	G_unknown	S_unknown
ASV.421	279	K_Bacteria	P_Cyanobacteria	C_Chloroplast	O_uncultured bacterium	F_unknown	G_unknown	S_unknown
ASV.371	273	K_Bacteria	P_Bacteroidetes	C_Cytophagia	O_Cytophagales	F_Flammeovirgaceae	G_Marinoscillum	S_uncultured bacterium
ASV.422	273	K_Bacteria	P_Proteobacteria	C_Alphaproteobacteria	O_Rickettsiales	F_SAR116 clade	G_uncultured bacterium	S_unknown
ASV.423	269	K_Bacteria	P_Proteobacteria	C_Alphaproteobacteria	O_Rhodospirillales	F_Rhodospirillaceae	G_uncultured	S_uncultured bacterium
ASV.334	268	K_Bacteria	P_Cyanobacteria	C_ML635J-21	O_uncultured bacterium	F_unknown	G_unknown	S_unknown
ASV.335	266	K_Bacteria	P_Proteobacteria	C_Gammaproteobacteria	O_Legionellales	F_Coxiellaceae	G_Coxiella	S_uncultured bacterium
ASV.325	263	K_Bacteria	P_Cyanobacteria	C_ML635J-21	O_uncultured bacterium	F_unknown	G_unknown	S_unknown
ASV.424	261	K_Bacteria	P_Proteobacteria	C_Alphaproteobacteria	O_Rickettsiales	F_SAR116 clade	G_uncultured marine bacterium	S_unknown
ASV.326	257	K_Bacteria	P_Bacteroidetes	C_Flavobacteriia	O_Flavobacteriales	F_Flavobacteriaceae	G_NS4 marine group	S_uncultured bacterium
ASV.339	255	K_Bacteria	P_Planctomycetes	C_Phycisphaerae	O_Phycisphaerales	F_Phycisphaeraceae	G_CL500-3	S_uncultured Phycisphaeraceae b
ASV.425	254	K_Bacteria	P_Cyanobacteria	C_Chloroplast	O_uncultured bacterium	F_unknown	G_unknown	S_unknown
ASV.336	253	K_Bacteria	P_Verrucomicrobia	C_Opitutae	O_Puniceicoccales	F_Puniceicoccaceae	G_marine group	S_uncultured bacterium
ASV.426	248	K_Archaea	P_Euryarchaeota	C_Thermoplasmata	O_Thermoplasmatales	F_Marine Group II	G_unknown	S_unknown
ASV.330	247	K_Bacteria	P_Proteobacteria	C_Deltaproteobacteria	O_Bradymonadales	F_uncultured delta proteoba	G_unknown	S_unknown
ASV.427	243	K_Bacteria	P_Cyanobacteria	C_Chloroplast	O_uncultured bacterium	F_unknown	G_unknown	S_unknown
ASV.428	241	K_Bacteria	P_Proteobacteria	C_Alphaproteobacteria	O_SAR11 clade	F_Surface 2	G_uncultured bacterium	S_unknown
ASV.429	235	K_Bacteria	P_Proteobacteria	C_Deltaproteobacteria	O_Bdellovibrionales	F_Bdellovibrionaceae	G_OM27 clade	S_uncultured bacterium
ASV.430	234	K_Bacteria	P_Proteobacteria	C_Alphaproteobacteria	O_Rhodospirillales	F_Rhodospirillaceae	G_AEGEAN-169 marine group	S_uncultured bacterium
ASV.340	229	K_Bacteria	P_Verrucomicrobia	C_Opitutae	O_Puniceicoccales	F_Puniceicoccaceae	G_Pelagicoccus	S_uncultured bacterium
ASV.431	227	K_Bacteria	P_Proteobacteria	C_Deltaproteobacteria	O_Bdellovibrionales	F_Bdellovibrionaceae	G_OM27 clade	S_uncultured bacterium
ASV.433	224	K_Archaea	P_Euryarchaeota	C_Thermoplasmata	O_Thermoplasmatales	F_Marine Group II	G_unknown	S_unknown
ASV.432	224	K_Bacteria	P_Proteobacteria	C_Deltaproteobacteria	O_Bdellovibrionales	F_Bdellovibrionaceae	G_OM27 clade	S_uncultured delta proteobacteri
ASV.434	223	K_Bacteria	P_Proteobacteria	C_Alphaproteobacteria	O_SAR11 clade	F_Surface 1	G_uncultured bacterium	S_unknown

ASV.344	218	K_Bacteria	P_Peregrinibacteria	C_uncultured bacterium	O_unknown	F_unknown	G_unknown	S_unknown
ASV.377	217	K_Bacteria	P_Bacteroidetes	C_Flavobacteriia	O_Flavobacteriales	F_Flavobacteriaceae	G_NS4 marine group	S_uncultured bacterium
ASV.435	217	K_Bacteria	P_Bacteroidetes	C_Sphingobacteriia	O_Sphingobacteriales	F_Saprospiraceae	G_Aureispira	S_uncultured bacterium
ASV.436	215	K_Bacteria	P_Bacteroidetes	C_Flavobacteriia	O_Flavobacteriales	F_NS7 marine group	G_uncultured bacterium	S_unknown
ASV.345	214	K_Bacteria	P_Bacteroidetes	C_Flavobacteriia	O_Flavobacteriales	F_NS7 marine group	G_uncultured bacterium	S_unknown
ASV.437	212	K_Bacteria	P_Proteobacteria	C_Alphaproteobacteria	O_Rhodospirillales	F_Rhodospirillaceae	G_uncultured	S_uncultured bacterium
ASV.338	212	K_Bacteria	P_Proteobacteria	C_Deltaproteobacteria	O_Bradymonadales	F_uncultured delta proteoba	G_unknown	S_unknown
ASV.438	210	K_Bacteria	P_Proteobacteria	C_Deltaproteobacteria	O_Bdellovibrionales	F_Bdellovibrionaceae	G_OM27 clade	S_unknown
ASV.346	208	K_Bacteria	P_Proteobacteria	C_Deltaproteobacteria	O_Bdellovibrionales	F_Bdellovibrionaceae	G_OM27 clade	S_uncultured delta proteobacteri
ASV.341	206	K_Bacteria	P_Cyanobacteria	C_Chloroplast	O_uncultured bacterium	F_unknown	G_unknown	S_unknown
ASV.439	205	K_Bacteria	P_Proteobacteria	C_Deltaproteobacteria	O_Bdellovibrionales	F_Bdellovibrionaceae	G_OM27 clade	S_uncultured bacterium
ASV.440	204	K_Bacteria	P_Cyanobacteria	C_Cyanobacteria	O_SubsectionI	F_FamilyI	G_Prochlorococcus	S_uncultured bacterium
ASV.347	201	K_Bacteria	P_Cyanobacteria	C_ML635J-21	O_uncultured bacterium	F_unknown	G_unknown	S_unknown
ASV.372	199	K_Bacteria	P_Proteobacteria	C_Alphaproteobacteria	O_Rickettsiales	F_S25-593	G_uncultured bacterium	S_unknown
ASV.348	198	K_Bacteria	P_Proteobacteria	C_Gammaproteobacteria	O_Legionellales	F_Coxiellaceae	G_Coxiella	S_uncultured bacterium
ASV.352	198	K_Bacteria	P_Chlamydiae	C_Chlamydiae	O_Chlamydiales	F_unknown	G_unknown	S_unknown
ASV.349	195	K_Bacteria	P_Proteobacteria	C_Deltaproteobacteria	O_Bdellovibrionales	F_Bdellovibrionaceae	G_Bdellovibrio	S_uncultured bacterium
ASV.342	191	K_Bacteria	P_Planctomycetes	C_Planctomycetacia	O_Planctomycetales	F_Planctomycetaceae	G_Rhodopirellula	S_unknown
ASV.441	190	K_Bacteria	P_Proteobacteria	C_Alphaproteobacteria	O_SAR11 clade	F_Surface 2	G_uncultured bacterium	S_unknown
ASV.442	185	K_Bacteria	P_Bacteroidetes	C_Flavobacteriia	O_Flavobacteriales	F_Cryomorphaceae	G_uncultured	S_uncultured Owenweeksia sp.
ASV.443	185	K_Bacteria	P_Bacteroidetes	C_Flavobacteriia	O_Flavobacteriales	F_Flavobacteriaceae	G_Tenacibaculum	S_uncultured bacterium
ASV.361	181	K_Bacteria	P_Proteobacteria	C_Deltaproteobacteria	O_Bdellovibrionales	F_Bdellovibrionaceae	G_OM27 clade	S_unknown
ASV.445	179	K_Bacteria	P_Proteobacteria	C_Alphaproteobacteria	O_SAR11 clade	F_Surface 2	G_uncultured bacterium	S_unknown
ASV.444	179	K_Bacteria	P_Bacteroidetes	C_Flavobacteriia	O_Flavobacteriales	F_Flavobacteriaceae	G_Aquibacter	S_uncultured bacterium
ASV.350	178	K_Bacteria	P_Bacteroidetes	C_Sphingobacteriia	O_Sphingobacteriales	F_Saprospiraceae	G_Aureispira	S_uncultured bacterium
ASV.446	172	K_Bacteria	P_Bacteroidetes	C_Flavobacteriia	O_Flavobacteriales	F_NS9 marine group	G_uncultured bacterium	S_unknown
ASV.351	172	K_Bacteria	P_Cyanobacteria	C_Chloroplast	O_uncultured bacterium	F_unknown	G_unknown	S_unknown
ASV.365	169	K_Bacteria	P_Bacteroidetes	C_Flavobacteriia	O_Flavobacteriales	F_NS9 marine group	G_uncultured Flavobacteriales bacte	S_unknown
ASV.447	168	K_Bacteria	P_Proteobacteria	C_Gammaproteobacteria	O_Legionellales	F_Coxiellaceae	G_Coxiella	S_uncultured bacterium
ASV.448	167	K_Bacteria	P_Verrucomicrobia	C_Opitutae	O_Puniceicoccales	F_Puniceicoccaceae	G_marine group	S_uncultured bacterium
ASV.449	166	K_Archaea	P_Euryarchaeota	C_Thermoplasmata	O_Thermoplasmatales	F_Marine Group III	G_uncultured marine archaeon	S_unknown
ASV.450	166	K_Bacteria	P_Proteobacteria	C_Alphaproteobacteria	O_Rickettsiales	F_S25-593	G_uncultured bacterium	S_unknown
ASV.452	163	K_Bacteria	P_Proteobacteria	C_Alphaproteobacteria	O_Rhizobiales	F_PS1 clade	G_uncultured bacterium	S_unknown
ASV.451	163	K_Bacteria	P_Proteobacteria	C_Alphaproteobacteria	O_SAR11 clade	F_Surface 1	G_uncultured bacterium	S_unknown
ASV.454	162	K_Bacteria	P_Proteobacteria	C_Alphaproteobacteria	O_SAR11 clade	F_Surface 1	G_uncultured bacterium	S_unknown
ASV.455	162	K_Bacteria	P_Bacteroidetes	C_Sphingobacteriia	O_Sphingobacteriales	F_Saprospiraceae	G_Aureispira	S_uncultured bacterium
ASV.453	162	K_Bacteria	P_Proteobacteria	C_Deltaproteobacteria	O_Bdellovibrionales	F_Bdellovibrionaceae	G_OM27 clade	S_uncultured marine microorgani
ASV.354	160	K_Bacteria	P_Cyanobacteria	C_Chloroplast	O_uncultured bacterium	F_unknown	G_unknown	S_unknown
ASV.456	159	K_Bacteria	P_Proteobacteria	C_Alphaproteobacteria	O_SAR11 clade	F_Surface 1	G_unknown	S_unknown
ASV.457	158	K_Bacteria	P_Bacteroidetes	C_Flavobacteriia	O_Flavobacteriales	F_Cryomorphaceae	G_Crocinitomix	S_uncultured bacterium
ASV.357	157	K_Bacteria	P_Gracilibacteria	C_uncultured bacterium	O_unknown	F_unknown	G_unknown	S_unknown
ASV.458	155	K_Bacteria	P_Bacteroidetes	C_Flavobacteriia	O_Flavobacteriales	F_NS9 marine group	G_uncultured marine bacterium	S_unknown
ASV.459	154	K_Bacteria	P_Cyanobacteria	C_Chloroplast	O_uncultured bacterium	F_unknown	G_unknown	S_unknown
ASV.353	152	K_Bacteria	P_Proteobacteria	C_Gammaproteobacteria	O_Legionellales	F_Coxiellaceae	G_Coxiella	S_unknown
ASV.462	147	K_Bacteria	P_Cyanobacteria	C_Chloroplast	O_uncultured diatom	F_unknown	G_unknown	S_unknown
ASV.461	147	K_Bacteria	P_Proteobacteria	C_Alphaproteobacteria	O_Rickettsiales	F_S25-593	G_uncultured bacterium	S_unknown
ASV.460	147	K_Bacteria	P_Proteobacteria	C_Alphaproteobacteria	O_Rhodobacterales	F_Rhodobacteraceae	G_Roseovarius	S_uncultured bacterium
ASV.463	145	K_Bacteria	P_Cyanobacteria	C_Chloroplast	O_uncultured bacterium	F_unknown	G_unknown	S_unknown
ASV.464	145	K_Bacteria	P_Planctomycetes	C_Planctomycetacia	O_Planctomycetales	F_Planctomycetaceae	G_Blastopirellula	S_uncultured planctomycete
ASV.465	144	K_Bacteria	P_Cyanobacteria	C_Chloroplast	O_uncultured bacterium	F_unknown	G_unknown	S_unknown

ASV.362	142	K_Bacteria	P_Proteobacteria	C_Alphaproteobacteria	O_Rickettsiales	F_Mitochondria	G_unknown	S_unknown
ASV.358	142	K_Bacteria	P_Proteobacteria	C_Deltaproteobacteria	O_Bradymonadales	F_uncultured bacterium	G_unknown	S_unknown
ASV.373	141	K_Bacteria	P_Proteobacteria	C_Alphaproteobacteria	O_SAR11 clade	F_unknown	G_unknown	S_unknown
ASV.381	138	K_Bacteria	P_Proteobacteria	C_Alphaproteobacteria	O_Sphingomonadales	F_Sphingomonadaceae	G_Sphingomonas	S_unknown
ASV.355	137	K_Bacteria	P_Proteobacteria	C_Deltaproteobacteria	O_Bdellovibrionales	F_Bdellovibrionaceae	G_OM27 clade	S_uncultured delta proteobacteri
ASV.467	136	K_Bacteria	P_Proteobacteria	C_Alphaproteobacteria	O_Rhodobacterales	F_Rhodobacteraceae	G_unknown	S_unknown
ASV.468	136	K_Bacteria	P_Proteobacteria	C_Gammaproteobacteria	O_Legionellales	F_Coxiellaceae	G_Coxiella	S_unknown
ASV.466	136	K_Bacteria	P_Planctomycetes	C_Planctomycetacia	O_Planctomycetales	F_Planctomycetaceae	G_Blastopirellula	S_uncultured bacterium
ASV.469	135	K_Bacteria	P_Verrucomicrobia	C_OPB35 soil group	O_uncultured Verrucomicrobia bacte	F_unknown	G_unknown	S_unknown
ASV.470	135	K_Bacteria	P_Bacteroidetes	C_Flavobacteriia	O_Flavobacteriales	F_NS9 marine group	G_unknown	S_unknown
ASV.471	133	K_Bacteria	P_Cyanobacteria	C_Cyanobacteria	O_SubsectionI	F_FamilyI	G_Prochlorococcus	S_uncultured bacterium
ASV.472	132	K_Bacteria	P_Cyanobacteria	C_Chloroplast	O_uncultured bacterium	F_unknown	G_unknown	S_unknown
ASV.474	131	K_Bacteria	P_Bacteroidetes	C_Flavobacteriia	O_Flavobacteriales	F_Flavobacteriaceae	G_Tenacibaculum	S_uncultured bacterium
ASV.473	131	K_Bacteria	P_Proteobacteria	C_Gammaproteobacteria	O_Oceanospirillales	F_OM182 clade	G_uncultured gamma proteobacteri	S_unknown
ASV.359	128	K_Bacteria	P_Proteobacteria	C_Alphaproteobacteria	O_Rhodospirillales	F_unknown	G_unknown	S_unknown
ASV.475	128	K_Bacteria	P_Cyanobacteria	C_Chloroplast	O_uncultured bacterium	F_unknown	G_unknown	S_unknown
ASV.476	128	K_Bacteria	P_Bacteroidetes	C_Flavobacteriia	O_Flavobacteriales	F_Flavobacteriaceae	G_NS2b marine group	S_uncultured bacterium
ASV.477	126	K_Bacteria	P_Proteobacteria	C_Gammaproteobacteria	O_Legionellales	F_Coxiellaceae	G_Coxiella	S_uncultured bacterium
ASV.363	126	K_Bacteria	P_Cyanobacteria	C_ML635J-21	O_uncultured bacterium	F_unknown	G_unknown	S_unknown
ASV.478	125	K_Bacteria	P_Verrucomicrobia	C_Arctic97B-4 marine group	O_uncultured bacterium	F_unknown	G_unknown	S_unknown
ASV.480	124	K_Bacteria	P_Proteobacteria	C_Alphaproteobacteria	O_SAR11 clade	F_unknown	G_unknown	S_unknown
ASV.479	124	K_Bacteria	P_Proteobacteria	C_Alphaproteobacteria	O_Rhodospirillales	F_Rhodospirillaceae	G_AEGEAN-169 marine group	S_uncultured bacterium
ASV.481	123	K_Bacteria	P_Planctomycetes	C_Planctomycetacia	O_Planctomycetales	F_Planctomycetaceae	G_Bythopirellula	S_uncultured bacterium
ASV.484	122	K_Bacteria	P_Proteobacteria	C_Alphaproteobacteria	O_Rickettsiales	F_SAR116 clade	G_uncultured bacterium	S_unknown
ASV.483	122	K_Bacteria	P_Cyanobacteria	C_ML635J-21	O_uncultured bacterium	F_unknown	G_unknown	S_unknown
ASV.482	122	K_Bacteria	P_Bacteroidetes	C_Cytophagia	O_Cytophagales	F_Flammeovirgaceae	G_uncultured	S_unknown
ASV.485	121	K_Bacteria	P_Proteobacteria	C_Deltaproteobacteria	O_Bradymonadales	F_unknown	G_unknown	S_unknown
ASV.487	120	K_Bacteria	P_Proteobacteria	C_Alphaproteobacteria	O_SAR11 clade	F_Surface 1	G_uncultured bacterium	S_unknown
ASV.486	120	K_Bacteria	P_Proteobacteria	C_Alphaproteobacteria	O_SAR11 clade	F_uncultured bacterium	G_unknown	S_unknown
ASV.488	118	K_Bacteria	P_Bacteroidetes	C_Cytophagia	O_Cytophagales	F_Flammeovirgaceae	G_Marinoscillum	S_uncultured bacterium
ASV.360	118	K_Bacteria	P_Proteobacteria	C_Alphaproteobacteria	O_Rickettsiales	F_S25-593	G_uncultured bacterium	S_unknown
ASV.491	117	K_Bacteria	P_Bacteroidetes	C_Flavobacteriia	O_Flavobacteriales	F_Cryomorphaceae	G_uncultured	S_uncultured Bacteroidetes bacte
ASV.490	117	K_Bacteria	P_Proteobacteria	C_Alphaproteobacteria	O_SAR11 clade	F_uncultured bacterium	G_unknown	S_unknown
ASV.489	117	K_Bacteria	P_Cyanobacteria	C_Cyanobacteria	O_SubsectionI	F_FamilyI	G_Prochlorococcus	S_uncultured bacterium
ASV.492	114	K_Bacteria	P_Bacteroidetes	C_Flavobacteriia	O_Flavobacteriales	F_Flavobacteriaceae	G_NS5 marine group	S_uncultured bacterium
ASV.493	114	K_Bacteria	P_Proteobacteria	C_Deltaproteobacteria	O_Bradymonadales	F_uncultured delta proteoba	G_unknown	S_unknown
ASV.494	113	K_Bacteria	P_Proteobacteria	C_Alphaproteobacteria	O_SAR11 clade	F_Surface 4	G_uncultured bacterium	S_unknown
ASV.495	111	K_Bacteria	P_Bacteroidetes	C_Flavobacteriia	O_Flavobacteriales	F_NS7 marine group	G_uncultured bacterium	S_unknown
ASV.496	111	K_Bacteria	P_Proteobacteria	C_Gammaproteobacteria	O_Legionellales	F_Coxiellaceae	G_Coxiella	S_unknown
ASV.497	110	K_Bacteria	P_Proteobacteria	C_Deltaproteobacteria	O_Bdellovibrionales	F_Bdellovibrionaceae	G_OM27 clade	S_uncultured delta proteobacteri
ASV.498	109	K_Bacteria	P_Cyanobacteria	C_Cyanobacteria	O_SubsectionI	F_FamilyI	G_Prochlorococcus	S_uncultured bacterium
ASV.499	108	K_Bacteria	P_Proteobacteria	C_Alphaproteobacteria	O_Rickettsiales	F_S25-593	G_uncultured bacterium	S_unknown
ASV.366	108	K_Bacteria	P_Bacteroidetes	C_Flavobacteriia	O_Flavobacteriales	F_NS9 marine group	G_uncultured marine bacterium	S_unknown
ASV.501	107	K_Bacteria	P_Bacteroidetes	C_Flavobacteriia	O_Flavobacteriales	F_Cryomorphaceae	G_Fluviicola	S_uncultured bacterium
ASV.500	107	K_Bacteria	P_Proteobacteria	C_Alphaproteobacteria	O_Rhodospirillales	F_Rhodospirillaceae	G_AEGEAN-169 marine group	S_uncultured bacterium
ASV.364	106	K_Bacteria	P_Bacteroidetes	C_Flavobacteriia	O_Flavobacteriales	F_Flavobacteriaceae	G_NS5 marine group	S_uncultured bacterium
ASV.503	104	K_Bacteria	P_Proteobacteria	C_Deltaproteobacteria	O_Bdellovibrionales	F_Bdellovibrionaceae	G_OM27 clade	S_uncultured delta proteobacteri
ASV.502	104	K_Bacteria	P_Proteobacteria	C_Alphaproteobacteria	O_Rhodospirillales	F_Rhodospirillaceae	G_uncultured	S_uncultured bacterium
ASV.505	103	K_Bacteria	P_Proteobacteria	C_SPOTSOCT00m83	O_uncultured bacterium	F_unknown	G_unknown	S_unknown
ASV.504	103	K_Bacteria	P_Proteobacteria	C_Alphaproteobacteria	O_Rickettsiales	F_SAR116 clade	G_uncultured bacterium	S_unknown

ASV.506	102	K_Bacteria	P_Proteobacteria	C_Alphaproteobacteria	O_Rhodospirillales	F_Rhodospirillaceae	G_AEGEAN-169 marine group	S_uncultured bacterium
ASV.507	101	K_Bacteria	P_Cyanobacteria	C_Chloroplast	O_uncultured bacterium	F_unknown	G_unknown	S_unknown
ASV.367	100	K_Bacteria	P_Planctomycetes	C_OM190	O_unknown	F_unknown	G_unknown	S_unknown
ASV.508	98	K_Bacteria	P_Bacteroidetes	C_Cytophagia	O_Cytophagales	F_Flammeovirgaceae	G_Marinoscillum	S_uncultured bacterium
ASV.368	98	K_Bacteria	P_Proteobacteria	C_Alphaproteobacteria	O_Rickettsiales	F_SAR116 clade	G_uncultured bacterium	S_unknown
ASV.509	97	K_Bacteria	P_Bacteroidetes	C_Flavobacteriia	O_Flavobacteriales	F_Flavobacteriaceae	G_NS4 marine group	S_uncultured bacterium
ASV.510	96	K_Bacteria	P_Cyanobacteria	C_Chloroplast	O_uncultured bacterium	F_unknown	G_unknown	S_unknown
ASV.512	94	K_Bacteria	P_Bacteroidetes	C_Flavobacteriia	O_Flavobacteriales	F_NS9 marine group	G_uncultured Flavobacteriales bacte	S_unknown
ASV.511	94	K_Bacteria	P_Cyanobacteria	C_Cyanobacteria	O_SubsectionI	F_FamilyI	G_Prochlorococcus	S_uncultured bacterium
ASV.514	93	K_Bacteria	P_Proteobacteria	C_Deltaproteobacteria	O_Bdellovibrionales	F_Bdellovibrionaceae	G_Bdellovibrio	S_uncultured bacterium
ASV.515	93	K_Bacteria	P_Planctomycetes	C_Planctomycetacia	O_Planctomycetales	F_Planctomycetaceae	G_Pir4 lineage	S_uncultured planctomycete
ASV.513	93	K_Bacteria	P_Proteobacteria	C_Alphaproteobacteria	O_Rickettsiales	F_SAR116 clade	G_uncultured bacterium	S_unknown
ASV.516	92	K_Bacteria	P_Proteobacteria	C_Alphaproteobacteria	O_Rhizobiales	F_PS1 clade	G_uncultured bacterium	S_unknown
ASV.517	91	K_Bacteria	P_Proteobacteria	C_Alphaproteobacteria	O_Alphaproteobacteria Incertae Sed	F_Unknown Family	G_uncultured	S_unknown
ASV.518	90	K_Bacteria	P_Proteobacteria	C_Alphaproteobacteria	O_Rickettsiales	F_Mitochondria	G_unknown	S_unknown
ASV.519	88	K_Bacteria	P_Proteobacteria	C_Alphaproteobacteria	O_Sphingomonadales	F_Erythrobacteraceae	G_unknown	S_unknown
ASV.522	87	K_Bacteria	P_Cyanobacteria	C_ML635J-21	O_uncultured bacterium	F_unknown	G_unknown	S_unknown
ASV.520	87	K_Bacteria	P_Bacteroidetes	C_Flavobacteriia	O_Flavobacteriales	F_Cryomorphaceae	G_uncultured	S_uncultured Owenweeksia sp.
ASV.521	87	K_Bacteria	P_Verrucomicrobia	C_Arctic97B-4 marine group	O_uncultured bacterium	F_unknown	G_unknown	S_unknown
ASV.523	84	K_Bacteria	P_Proteobacteria	C_Alphaproteobacteria	O_SAR11 clade	F_Surface 1	G_uncultured bacterium	S_unknown
ASV.525	82	K_Bacteria	P_Cyanobacteria	C_Chloroplast	O_uncultured bacterium	F_unknown	G_unknown	S_unknown
ASV.524	82	K_Bacteria	P_Cyanobacteria	C_Chloroplast	O_uncultured bacterium	F_unknown	G_unknown	S_unknown
ASV.527	81	K_Bacteria	P_Proteobacteria	C_Alphaproteobacteria	O_Rickettsiales	F_Rickettsiaceae	G_uncultured	S_uncultured bacterium
ASV.526	81	K_Bacteria	P_Proteobacteria	C_Alphaproteobacteria	O_Rhizobiales	F_PS1 clade	G_uncultured bacterium	S_unknown
ASV.369	80	K_Bacteria	P_Proteobacteria	C_Alphaproteobacteria	O_Rhodospirillales	F_AT-s3-44	G_uncultured alpha proteobacterium	S_unknown
ASV.528	80	K_Bacteria	P_Cyanobacteria	C_ML635J-21	O_uncultured bacterium	F_unknown	G_unknown	S_unknown
ASV.529	80	K_Bacteria	P_Cyanobacteria	C_ML635J-21	O_uncultured bacterium	F_unknown	G_unknown	S_unknown
ASV.530	79	K_Bacteria	P_Cyanobacteria	C_Chloroplast	O_uncultured bacterium	F_unknown	G_unknown	S_unknown
ASV.532	77	K_Bacteria	P_Cyanobacteria	C_Chloroplast	O_uncultured bacterium	F_unknown	G_unknown	S_unknown
ASV.531	77	K_Bacteria	P_Proteobacteria	C_Deltaproteobacteria	O_Oligoflexales	F_053A03-B-DI-P58	G_uncultured bacterium	S_unknown
ASV.533	76	K_Bacteria	P_Bacteroidetes	C_Flavobacteriia	O_Flavobacteriales	F_NS7 marine group	G_uncultured bacterium	S_unknown
ASV.536	76	K_Bacteria	P_Cyanobacteria	C_Chloroplast	O_uncultured bacterium	F_unknown	G_unknown	S_unknown
ASV.535	76	K_Bacteria	P_Chloroflexi	C_Caldilineae	O_Caldilineales	F_Caldilineaceae	G_uncultured	S_uncultured bacterium
ASV.534	76	K_Bacteria	P_Verrucomicrobia	C_Arctic97B-4 marine group	O_uncultured bacterium	F_unknown	G_unknown	S_unknown
ASV.537	74	K_Bacteria	P_Proteobacteria	C_Deltaproteobacteria	O_Bdellovibrionales	F_Bdellovibrionaceae	G_OM27 clade	S_uncultured delta proteobacteri
ASV.540	73	K_Bacteria	P_Proteobacteria	C_Alphaproteobacteria	O_Alphaproteobacteria Incertae Sed	F_Unknown Family	G_uncultured	S_uncultured bacterium
ASV.538	73	K_Bacteria	P_Proteobacteria	C_Deltaproteobacteria	O_Bdellovibrionales	F_Bdellovibrionaceae	G_OM27 clade	S_uncultured delta proteobacteri
ASV.539	73	K_Bacteria	P_Proteobacteria	C_Gammaproteobacteria	O_Legionellales	F_Coxiellaceae	G_Coxiella	S_unknown
ASV.541	72	K_Bacteria	P_Cyanobacteria	C_Chloroplast	O_uncultured bacterium	F_unknown	G_unknown	S_unknown
ASV.542	72	K_Bacteria	P_Cyanobacteria	C_Chloroplast	O_uncultured bacterium	F_unknown	G_unknown	S_unknown
ASV.545	72	K_Bacteria	P_Proteobacteria	C_Deltaproteobacteria	O_SAR324 clade(Marine group B)	F_uncultured bacterium	G_unknown	S_unknown
ASV.543	72	K_Bacteria	P_Cyanobacteria	C_Chloroplast	O_uncultured bacterium	F_unknown	G_unknown	S_unknown
ASV.544	72	K_Bacteria	P_Bacteroidetes	C_Flavobacteriia	O_Flavobacteriales	F_Flavobacteriaceae	G_NS5 marine group	S_uncultured bacterium
ASV.546	71	K_Bacteria	P_Verrucomicrobia	C_Opitutae	O_Puniceicoccales	F_Puniceicoccaceae	G_marine group	S_uncultured bacterium
ASV.547	70	K_Bacteria	P_Proteobacteria	C_Alphaproteobacteria	O_SAR11 clade	F_Surface 2	G_uncultured bacterium	S_unknown
ASV.548	70	K_Bacteria	P_Cyanobacteria	C_Chloroplast	O_uncultured bacterium	F_unknown	G_unknown	S_unknown
ASV.550	69	K_Bacteria	P_Proteobacteria	C_Alphaproteobacteria	O_SAR11 clade	F_unknown	G_unknown	S_unknown
ASV.549	69	K_Bacteria	P_Proteobacteria	C_Alphaproteobacteria	O_Rhodospirillales	F_Rhodospirillaceae	G_AEGEAN-169 marine group	S_uncultured bacterium
ASV.551	69	K_Bacteria	P_Bacteroidetes	C_Flavobacteriia	O_Flavobacteriales	F_NS9 marine group	G_uncultured marine bacterium	S_unknown
ASV.370	69	K_Bacteria	P_Cyanobacteria	C_Melainabacteria	O_Caenarcaniphilales	F_uncultured bacterium	G_unknown	S_unknown

ASV.374	68	K_Bacteria	P_Cyanobacteria	C_Chloroplast	O_uncultured bacterium	F_unknown	G_unknown	S_unknown
ASV.552	68	K_Bacteria	P_Proteobacteria	C_Alphaproteobacteria	O_SAR11 clade	F_Surface 1	G_uncultured bacterium	S_unknown
ASV.555	67	K_Bacteria	P_Bacteroidetes	C_Flavobacteriia	O_Flavobacteriales	F_Cryomorphaceae	G_uncultured	S_uncultured Owenweeksia sp.
ASV.553	67	K_Bacteria	P_Proteobacteria	C_Deltaproteobacteria	O_SAR324 clade(Marine group B)	F_uncultured SAR324 cluster	G_unknown	S_unknown
ASV.554	67	K_Bacteria	P_Planctomycetes	C_Planctomycetacia	O_Planctomycetales	F_Planctomycetaceae	G_Blastopirellula	S_unknown
ASV.558	65	K_Bacteria	P_Cyanobacteria	C_Chloroplast	O_uncultured bacterium	F_unknown	G_unknown	S_unknown
ASV.557	65	K_Bacteria	P_Cyanobacteria	C_Chloroplast	O_uncultured bacterium	F_unknown	G_unknown	S_unknown
ASV.556	65	K_Bacteria	P_Verrucomicrobia	C_Opitutae	O_Puniceicoccales	F_Puniceicoccaceae	G_marine group	S_uncultured bacterium
ASV.559	64	K_Bacteria	P_Verrucomicrobia	C_Opitutae	O_Puniceicoccales	F_Puniceicoccaceae	G_marine group	S_uncultured bacterium
ASV.560	64	K_Bacteria	P_Bacteroidetes	C_Flavobacteriia	O_Flavobacteriales	F_Flavobacteriaceae	G_NS5 marine group	S_uncultured bacterium
ASV.561	63	K_Bacteria	P_Planctomycetes	C_Planctomycetacia	O_Planctomycetales	F_Planctomycetaceae	G_Pirellula	S_uncultured bacterium
ASV.562	62	K_Bacteria	P_Proteobacteria	C_unknown	O_unknown	F_unknown	G_unknown	S_unknown
ASV.563	61	K_Bacteria	P_Bacteroidetes	C_Flavobacteriia	O_Flavobacteriales	F_NS7 marine group	G_uncultured bacterium	S_unknown
ASV.564	60	K_Bacteria	P_Bacteroidetes	C_Flavobacteriia	O_Flavobacteriales	F_Flavobacteriaceae	G_uncultured	S_uncultured bacterium
ASV.565	60	K_Bacteria	P_Proteobacteria	C_Alphaproteobacteria	O_SAR11 clade	F_uncultured bacterium	G_unknown	S_unknown
ASV.568	58	K_Bacteria	P_Cyanobacteria	C_Chloroplast	O_uncultured diatom	F_unknown	G_unknown	S_unknown
ASV.566	58	K_Bacteria	P_Proteobacteria	C_Gammaproteobacteria	O_Legionellales	F_Coxiellaceae	G_Coxiella	S_unknown
ASV.567	58	K_Bacteria	P_Cyanobacteria	C_ML635J-21	O_uncultured bacterium	F_unknown	G_unknown	S_unknown
ASV.571	57	K_Bacteria	P_Proteobacteria	C_Alphaproteobacteria	O_Rhodospirillales	F_Rhodospirillaceae	G_uncultured	S_uncultured bacterium
ASV.572	57	K_Bacteria	P_Bacteroidetes	C_Flavobacteriia	O_Flavobacteriales	F_NS9 marine group	G_unknown	S_unknown
ASV.569	57	K_Bacteria	P_Proteobacteria	C_Deltaproteobacteria	O_Bdellovibrionales	F_Bdellovibrionaceae	G_OM27 clade	S_uncultured marine microorgani
ASV.570	57	K_Bacteria	P_Chloroflexi	C_Caldilineae	O_Caldilineales	F_Caldilineaceae	G_uncultured	S_uncultured bacterium
ASV.574	56	K_Bacteria	P_Proteobacteria	C_Alphaproteobacteria	O_Rickettsiales	F_Mitochondria	G_Proteobacteria bacterium JGI 000	S_unknown
ASV.573	56	K_Bacteria	P_Bacteroidetes	C_Flavobacteriia	O_Flavobacteriales	F_Flavobacteriaceae	G_NS5 marine group	S_uncultured bacterium
ASV.577	55	K_Bacteria	P_Cyanobacteria	C_ML635J-21	O_unknown	F_unknown	G_unknown	S_unknown
ASV.575	55	K_Bacteria	P_Proteobacteria	C_Deltaproteobacteria	O_Oligoflexales	F_0319-6G20	G_uncultured organism	S_unknown
ASV.578	55	K_Bacteria	P_Proteobacteria	C_Deltaproteobacteria	O_Bradymonadales	F_unknown	G_unknown	S_unknown
ASV.576	55	K_Bacteria	P_Planctomycetes	C_Planctomycetacia	O_Planctomycetales	F_Planctomycetaceae	G_Rhodopirellula	S_uncultured bacterium
ASV.579	54	K_Bacteria	P_Cyanobacteria	C_Chloroplast	O_uncultured bacterium	F_unknown	G_unknown	S_unknown
ASV.580	54	K_Bacteria	P_Bacteroidetes	C_Flavobacteriia	O_Flavobacteriales	F_Cryomorphaceae	G_uncultured	S_unknown
ASV.586	53	K_Bacteria	P_Proteobacteria	C_Gammaproteobacteria	O_Legionellales	F_Coxiellaceae	G_Coxiella	S_uncultured bacterium
ASV.583	53	K_Bacteria	P_Cyanobacteria	C_Chloroplast	O_uncultured bacterium	F_unknown	G_unknown	S_unknown
ASV.585	53	K_Bacteria	P_Cyanobacteria	C_Chloroplast	O_Bigelowiella natans	F_unknown	G_unknown	S_unknown
ASV.587	53	K_Archaea	P_Euryarchaeota	C_Thermoplasmata	O_Thermoplasmatales	F_Marine Group III	G_uncultured marine archaeon	S_unknown
ASV.584	53	K_Bacteria	P_SBR1093	C_marine metagenome	O_unknown	F_unknown	G_unknown	S_unknown
ASV.582	53	K_Bacteria	P_Cyanobacteria	C_Chloroplast	O_uncultured bacterium	F_unknown	G_unknown	S_unknown
ASV.581	53	K_Bacteria	P_Proteobacteria	C_Deltaproteobacteria	O_Bradymonadales	F_uncultured bacterium	G_unknown	S_unknown
ASV.589	52	K_Bacteria	P_Parcubacteria	C_uncultured organism	O_unknown	F_unknown	G_unknown	S_unknown
ASV.588	52	K_Bacteria	P_unknown	C_unknown	O_unknown	F_unknown	G_unknown	S_unknown
ASV.591	51	K_Bacteria	P_Cyanobacteria	C_Cyanobacteria	O_SubsectionI	F_FamilyI	G_Synechococcus	S_uncultured bacterium
ASV.590	51	K_Bacteria	P_Proteobacteria	C_Alphaproteobacteria	O_unknown	F_unknown	G_unknown	S_unknown
ASV.592	50	K_Bacteria	P_Cyanobacteria	C_Chloroplast	O_uncultured bacterium	F_unknown	G_unknown	S_unknown
ASV.596	49	K_Bacteria	P_Cyanobacteria	C_Melainabacteria	O_Vampirovibrionales	F_uncultured bacterium	G_unknown	S_unknown
ASV.595	49	K_Bacteria	P_Proteobacteria	C_Deltaproteobacteria	O_Bdellovibrionales	F_Bdellovibrionaceae	G_Bdellovibrio	S_uncultured delta proteobacteri
ASV.594	49	K_Bacteria	P_unknown	C_unknown	O_unknown	F_unknown	G_unknown	S_unknown
ASV.593	49	K_Bacteria	P_Bacteroidetes	C_Flavobacteriia	O_Flavobacteriales	F_Cryomorphaceae	G_Fluviicola	S_uncultured bacterium
ASV.597	49	K_Bacteria	P_Proteobacteria	C_Alphaproteobacteria	O_Rhodobacterales	F_Rhodobacteraceae	G_Roseovarius	S_uncultured bacterium
ASV.598	48	K_Bacteria	P_Proteobacteria	C_Alphaproteobacteria	O_Rickettsiales	F_S25-593	G_uncultured bacterium	S_unknown
ASV.599	48	K_Bacteria	P_Cyanobacteria	C_ML635J-21	O_uncultured bacterium	F_unknown	G_unknown	S_unknown
ASV.600	47	K_Bacteria	P_Proteobacteria	C_Deltaproteobacteria	O_Bdellovibrionales	F_Bdellovibrionaceae	G_OM27 clade	S_uncultured delta proteobacteri

ASV.601	47	K_Archaea	P_Euryarchaeota	C_Thermoplasmata	O_Thermoplasmatales	F_Marine Group II	G_uncultured marine archaeon	S_unknown
ASV.603	45	K_Bacteria	P_Cyanobacteria	C_Chloroplast	O_uncultured bacterium	F_unknown	G_unknown	S_unknown
ASV.602	45	K_Bacteria	P_Proteobacteria	C_Alphaproteobacteria	O_unknown	F_unknown	G_unknown	S_unknown
ASV.606	43	K_Bacteria	P_Bacteroidetes	C_Flavobacteriia	O_Flavobacteriales	F_NS9 marine group	G_uncultured bacterium	S_unknown
ASV.609	43	K_Bacteria	P_Bacteroidetes	C_Flavobacteriia	O_Flavobacteriales	F_NS9 marine group	G_unknown	S_unknown
ASV.605	43	K_Bacteria	P_Proteobacteria	C_Deltaproteobacteria	O_Bdellovibrionales	F_Bdellovibrionaceae	G_OM27 clade	S_uncultured marine microorgani
ASV.607	43	K_Bacteria	P_Bacteroidetes	C_Flavobacteriia	O_Flavobacteriales	F_Flavobacteriaceae	G_Tenacibaculum	S_uncultured bacterium
ASV.608	43	K_Bacteria	P_Actinobacteria	C_Actinobacteria	O_PeM15	F_uncultured bacterium	G_unknown	S_unknown
ASV.604	43	K_Bacteria	P_Proteobacteria	C_Alphaproteobacteria	O_Rhizobiales	F_PS1 clade	G_uncultured bacterium	S_unknown
ASV.610	42	K_Bacteria	P_Cyanobacteria	C_Melainabacteria	O_Caenarcaniphilales	F_uncultured bacterium	G_unknown	S_unknown
ASV.612	42	K_Bacteria	P_Proteobacteria	C_Deltaproteobacteria	O_Bdellovibrionales	F_Bdellovibrionaceae	G_Bdellovibrio	S_uncultured bacterium
ASV.611	42	K_Bacteria	P_Proteobacteria	C_Alphaproteobacteria	O_Rickettsiales	F_SAR116 clade	G_uncultured bacterium	S_unknown
ASV.614	41	K_Bacteria	P_Cyanobacteria	C_Chloroplast	O_uncultured bacterium	F_unknown	G_unknown	S_unknown
ASV.613	41	K_Bacteria	P_Cyanobacteria	C_Chloroplast	O_uncultured bacterium	F_unknown	G_unknown	S_unknown
ASV.615	40	K_Bacteria	P_Proteobacteria	C_unknown	O_unknown	F_unknown	G_unknown	S_unknown
ASV.616	40	K_Bacteria	P_Bacteroidetes	C_Flavobacteriia	O_Flavobacteriales	F_Flavobacteriaceae	G_Pseudofulvibacter	S_uncultured bacterium
ASV.618	39	K_Bacteria	P_Proteobacteria	C_Deltaproteobacteria	O_Bdellovibrionales	F_Bdellovibrionaceae	G_OM27 clade	S_unknown
ASV.617	39	K_Bacteria	P_Proteobacteria	C_Alphaproteobacteria	O_Rhodobacterales	F_Rhodobacteraceae	G_uncultured	S_unknown
ASV.621	38	K_Bacteria	P_Chloroflexi	C_SAR202 clade	O_uncultured Chloroflexi bacterium	F_unknown	G_unknown	S_unknown
ASV.619	38	K_Bacteria	P_Bacteroidetes	C_Flavobacteriia	O_Flavobacteriales	F_Flavobacteriaceae	G_NS5 marine group	S_uncultured bacterium
ASV.620	38	K_Bacteria	P_Proteobacteria	C_Deltaproteobacteria	O_Bradymonadales	F_uncultured delta proteoba	G_unknown	S_unknown
ASV.622	37	K_Bacteria	P_Proteobacteria	C_Alphaproteobacteria	O_Rickettsiales	F_S25-593	G_uncultured bacterium	S_unknown
ASV.623	36	K_Bacteria	P_Planctomycetes	C_Planctomycetacia	O_Planctomycetales	F_Planctomycetaceae	G_Planctomyces	S_uncultured Planctomyces sp.
ASV.626	35	K_Bacteria	P_Cyanobacteria	C_Chloroplast	O_uncultured bacterium	F_unknown	G_unknown	S_unknown
ASV.624	35	K_Bacteria	P_Bacteroidetes	C_Flavobacteriia	O_Flavobacteriales	F_NS9 marine group	G_unknown	S_unknown
ASV.627	35	K_Bacteria	P_Bacteroidetes	C_Flavobacteriia	O_Flavobacteriales	F_Flavobacteriaceae	G_NS5 marine group	S_uncultured bacterium
ASV.625	35	K_Bacteria	P_Proteobacteria	C_Alphaproteobacteria	O_Rickettsiales	F_SAR116 clade	G_uncultured bacterium	S_unknown
ASV.631	34	K_Bacteria	P_Proteobacteria	C_Alphaproteobacteria	O_Rickettsiales	F_SAR116 clade	G_uncultured bacterium	S_unknown
ASV.628	34	K_Bacteria	P_Proteobacteria	C_Alphaproteobacteria	O_SAR11 clade	F_unknown	G_unknown	S_unknown
ASV.630	34	K_Bacteria	P_Proteobacteria	C_Deltaproteobacteria	O_Bdellovibrionales	F_Bdellovibrionaceae	G_OM27 clade	S_uncultured delta proteobacteri
ASV.629	34	K_Bacteria	P_Proteobacteria	C_Alphaproteobacteria	O_Rhodospirillales	F_Rhodospirillaceae	G_AEGEAN-169 marine group	S_uncultured bacterium
ASV.633	33	K_Bacteria	P_Bacteroidetes	C_Flavobacteriia	O_Flavobacteriales	F_NS7 marine group	G_uncultured bacterium	S_unknown
ASV.634	33	K_Bacteria	P_Cyanobacteria	C_ML635J-21	O_uncultured bacterium	F_unknown	G_unknown	S_unknown
ASV.632	33	K_Bacteria	P_Proteobacteria	C_Alphaproteobacteria	O_Rickettsiales	F_S25-593	G_uncultured bacterium	S_unknown
ASV.635	33	K_Bacteria	P_Bacteroidetes	C_Bacteroidetes Incertae Sed	O_Order III	F_Unknown Family	G_Balneola	S_uncultured bacterium
ASV.637	32	K_Bacteria	P_Cyanobacteria	C_ML635J-21	O_uncultured bacterium	F_unknown	G_unknown	S_unknown
ASV.636	32	K_Bacteria	P_Bacteroidetes	C_Flavobacteriia	O_Flavobacteriales	F_Flavobacteriaceae	G_NS5 marine group	S_uncultured bacterium
ASV.639	32	K_Bacteria	P_Cyanobacteria	C_ML635J-21	O_uncultured bacterium	F_unknown	G_unknown	S_unknown
ASV.642	32	K_Bacteria	P_Proteobacteria	C_Deltaproteobacteria	O_SAR324 clade(Marine group B)	F_uncultured bacterium	G_unknown	S_unknown
ASV.641	32	K_Bacteria	P_Planctomycetes	C_Phycisphaerae	O_Phycisphaerales	F_Phycisphaeraceae	G_FS140-16B-02 marine group	S_unknown
ASV.640	32	K_Bacteria	P_Cyanobacteria	C_Chloroplast	O_uncultured bacterium	F_unknown	G_unknown	S_unknown
ASV.638	32	K_Bacteria	P_Proteobacteria	C_Alphaproteobacteria	O_Alphaproteobacteria Incertae Sed	F_Unknown Family	G_uncultured	S_uncultured bacterium
ASV.645	31	K_Bacteria	P_Bacteroidetes	C_Flavobacteriia	O_Flavobacteriales	F_Flavobacteriaceae	G_Tenacibaculum	S_uncultured bacterium
ASV.644	31	K_Bacteria	P_Cyanobacteria	C_Chloroplast	O_uncultured diatom	F_unknown	G_unknown	S_unknown
ASV.646	31	K_Bacteria	P_Bacteroidetes	C_Flavobacteriia	O_Flavobacteriales	F_Cryomorphaceae	G_Crocinitomix	S_uncultured bacterium
ASV.643	31	K_Archaea	P_Euryarchaeota	C_Thermoplasmata	O_Thermoplasmatales	F_Marine Group II	G_uncultured marine archaeon	S_unknown
ASV.649	30	K_Bacteria	P_Cyanobacteria	C_Melainabacteria	O_Vampirovibrionales	F_uncultured bacterium	G_unknown	S_unknown
ASV.648	30	K_Bacteria	P_Proteobacteria	C_Deltaproteobacteria	O_Bdellovibrionales	F_Bdellovibrionaceae	G_OM27 clade	S_uncultured delta proteobacteri
ASV.647	30	K_Bacteria	P_Cyanobacteria	C_Chloroplast	O_uncultured bacterium	F_unknown	G_unknown	S_unknown
ASV.652	30	K_Bacteria	P_Verrucomicrobia	C_Opitutae	O_Puniceicoccales	F_Puniceicoccaceae	G_marine group	S_uncultured bacterium

ASV.650	30	K_Bacteria	P_Cyanobacteria	C_Chloroplast	O_uncultured bacterium	F_unknown	G_unknown	S_unknown
ASV.651	30	K_Bacteria	P_Bacteroidetes	C_Sphingobacteriia	O_Sphingobacteriales	F_Saprospiraceae	G_unknown	S_unknown
ASV.653	29	K_Bacteria	P_Bacteroidetes	C_Flavobacteriia	O_Flavobacteriales	F_NS7 marine group	G_uncultured bacterium	S_unknown
ASV.654	29	K_Bacteria	P_Proteobacteria	C_Alphaproteobacteria	O_Rhodospirillales	F_Rhodospirillaceae	G_uncultured	S_unknown
ASV.656	29	K_Bacteria	P_Cyanobacteria	C_ML635J-21	O_uncultured bacterium	F_unknown	G_unknown	S_unknown
ASV.655	29	K_Bacteria	P_Bacteroidetes	C_Bacteroidia	O_Bacteroidales	F_Marinilabiaceae	G_Marinifilum	S_uncultured bacterium
ASV.657	28	K_Bacteria	P_Proteobacteria	C_Deltaproteobacteria	O_Bdellovibrionales	F_Bdellovibrionaceae	G_OM27 clade	S_uncultured delta proteobacteri
ASV.661	27	K_Bacteria	P_Proteobacteria	C_Deltaproteobacteria	O_Bdellovibrionales	F_Bdellovibrionaceae	G_OM27 clade	S_uncultured delta proteobacteri
ASV.662	27	K_Bacteria	P_Bacteroidetes	C_Flavobacteriia	O_Flavobacteriales	F_Cryomorphaceae	G_uncultured	S_uncultured bacterium
ASV.659	27	K_Bacteria	P_Planctomycetes	C_Planctomycetacia	O_Planctomycetales	F_Planctomycetaceae	G_Blastopirellula	S_uncultured bacterium
ASV.663	27	K_Bacteria	P_Proteobacteria	C_Deltaproteobacteria	O_unknown	F_unknown	G_unknown	S_unknown
ASV.660	27	K_Bacteria	P_Proteobacteria	C_Alphaproteobacteria	O_Rhodobacterales	F_Rhodobacteraceae	G_uncultured	S_unknown
ASV.658	27	K_Bacteria	P_Planctomycetes	C_Phycisphaerae	O_Phycisphaerales	F_Phycisphaeraceae	G_CL500-3	S_uncultured bacterium
ASV.664	26	K_Bacteria	P_Cyanobacteria	C_Melainabacteria	O_Vampirovibrionales	F_uncultured bacterium	G_unknown	S_unknown
ASV.667	24	K_Bacteria	P_Proteobacteria	C_Proteobacteria Incertae Se	O_Unknown Order	F_Unknown Family	G_Candidatus Tenderia	S_uncultured bacterium
ASV.666	24	K_Bacteria	P_Cyanobacteria	C_Chloroplast	O_uncultured diatom	F_unknown	G_unknown	S_unknown
ASV.665	24	K_Bacteria	P_Proteobacteria	C_Deltaproteobacteria	O_Bdellovibrionales	F_Bdellovibrionaceae	G_Bdellovibrio	S_uncultured bacterium
ASV.669	23	K_Bacteria	P_Planctomycetes	C_OM190	O_unknown	F_unknown	G_unknown	S_unknown
ASV.668	23	K_Bacteria	P_Proteobacteria	C_Alphaproteobacteria	O_Alphaproteobacteria Incertae Sed	F_uncultured	G_uncultured bacterium	S_unknown
ASV.671	23	K_Bacteria	P_Planctomycetes	C_Phycisphaerae	O_Phycisphaerales	F_Phycisphaeraceae	G_Urania-1B-19 marine sediment gr	S_uncultured bacterium
ASV.670	23	K_Bacteria	P_Proteobacteria	C_Alphaproteobacteria	O_SAR11 clade	F_uncultured bacterium	G_unknown	S_unknown
ASV.675	22	K_Bacteria	P_Proteobacteria	C_Alphaproteobacteria	O_Caulobacterales	F_Hyphomonadaceae	G_uncultured	S_uncultured organism
ASV.672	22	K_Bacteria	P_Planctomycetes	C_BD7-11	O_uncultured bacterium	F_unknown	G_unknown	S_unknown
ASV.673	22	K_Bacteria	P_Cyanobacteria	C_ML635J-21	O_uncultured bacterium	F_unknown	G_unknown	S_unknown
ASV.677	22	K_Bacteria	P_Planctomycetes	C_Planctomycetacia	O_Planctomycetales	F_Planctomycetaceae	G_Blastopirellula	S_uncultured bacterium
ASV.676	22	K_Bacteria	P_Proteobacteria	C_Alphaproteobacteria	O_Alphaproteobacteria Incertae Sed	F_Unknown Family	G_uncultured	S_uncultured bacterium
ASV.674	22	K_Bacteria	P_Peregrinibacteria	C_Candidatus Peribacteria	O_uncultured bacterium	F_unknown	G_unknown	S_unknown
ASV.678	21	K_Bacteria	P_Proteobacteria	C_Alphaproteobacteria	O_Rickettsiales	F_Mitochondria	G_marine metagenome	S_unknown
ASV.679	21	K_Bacteria	P_Proteobacteria	C_Deltaproteobacteria	O_Bdellovibrionales	F_Bdellovibrionaceae	G_OM27 clade	S_uncultured marine microorgani
ASV.680	20	K_Bacteria	P_Proteobacteria	C_Deltaproteobacteria	O_Bradymonadales	F_uncultured delta proteoba	G_unknown	S_unknown
ASV.684	19	K_Bacteria	P_Lentisphaerae	C_unknown	O_unknown	F_unknown	G_unknown	S_unknown
ASV.682	19	K_Bacteria	P_Proteobacteria	C_Deltaproteobacteria	O_Myxococcales	F_Nannocystaceae	G_uncultured	S_unknown
ASV.683	19	K_Bacteria	P_Bacteroidetes	C_Flavobacteriia	O_Flavobacteriales	F_Cryomorphaceae	G_Fluviicola	S_uncultured bacterium
ASV.681	19	K_Bacteria	P_Proteobacteria	C_Deltaproteobacteria	O_Bdellovibrionales	F_Bdellovibrionaceae	G_OM27 clade	S_uncultured delta proteobacteri
ASV.686	18	K_Bacteria	P_Proteobacteria	C_Alphaproteobacteria	O_Rickettsiales	F_LWSR-14	G_uncultured bacterium	S_unknown
ASV.685	18	K_Bacteria	P_Proteobacteria	C_Deltaproteobacteria	O_Myxococcales	F_Eel-36e1D6	G_uncultured delta proteobacterium	S_unknown
ASV.384	17	K_Bacteria	P_Proteobacteria	C_Alphaproteobacteria	O_Rhizobiales	F_Methylobacteriaceae	G_Methylobacterium	S_uncultured bacterium
ASV.690	17	K_Bacteria	P_Proteobacteria	C_Deltaproteobacteria	O_Bdellovibrionales	F_Bdellovibrionaceae	G_Bdellovibrio	S_uncultured delta proteobacteri
ASV.689	17	K_Bacteria	P_Verrucomicrobia	C_Opitutae	O_Puniceicoccales	F_Puniceicoccaceae	G_Cerasicoccus	S_uncultured Verrucomicrobia ba
ASV.688	17	K_Bacteria	P_Planctomycetes	C_Planctomycetacia	O_Planctomycetales	F_Planctomycetaceae	G_Planctomyces	S_uncultured planctomycete
ASV.687	17	K_Bacteria	P_Planctomycetes	C_Phycisphaerae	O_Phycisphaerales	F_Phycisphaeraceae	G_SM1A02	S_unknown
ASV.691	17	K_Bacteria	P_Bacteroidetes	C_Flavobacteriia	O_Flavobacteriales	F_NS7 marine group	G_uncultured bacterium	S_unknown
ASV.692	17	K_Bacteria	P_Bacteroidetes	C_Flavobacteriia	O_Flavobacteriales	F_Flavobacteriaceae	G_NS5 marine group	S_uncultured bacterium
ASV.695	16	K_Bacteria	P_Cyanobacteria	C_Chloroplast	O_uncultured bacterium	F_unknown	G_unknown	S_unknown
ASV.693	16	K_Bacteria	P_Proteobacteria	C_Gammaproteobacteria	O_HOC36	F_uncultured bacterium	G_unknown	S_unknown
ASV.694	16	K_Bacteria	P_Proteobacteria	C_Alphaproteobacteria	O_Alphaproteobacteria Incertae Sed	F_Unknown Family	G_uncultured	S_uncultured bacterium
ASV.699	15	K_Bacteria	P_Bacteroidetes	C_Flavobacteriia	O_Flavobacteriales	F_NS9 marine group	G_uncultured bacterium	S_unknown
ASV.700	15	K_Bacteria	P_Proteobacteria	C_Alphaproteobacteria	O_Rickettsiales	F_Rickettsiaceae	G_uncultured	S_uncultured bacterium
ASV.701	15	K_Bacteria	P_Bacteroidetes	C_Flavobacteriia	O_Flavobacteriales	F_Flavobacteriaceae	G_Aquibacter	S_uncultured bacterium
ASV.697	15	K_Bacteria	P_Planctomycetes	C_Phycisphaerae	O_Phycisphaerales	F_Phycisphaeraceae	G_Urania-1B-19 marine sediment gr	S_uncultured bacterium

ASV.702	15	K_Bacteria	P_Bacteroidetes	C_Cytophagia	O_Cytophagales	F_unknown	G_unknown	S_unknown
ASV.696	15	K_Bacteria	P_Bacteroidetes	C_Sphingobacteriia	O_Sphingobacteriales	F_Saprospiraceae	G_Portibacter	S_unknown
ASV.698	15	K_Bacteria	P_Proteobacteria	C_Alphaproteobacteria	O_unknown	F_unknown	G_unknown	S_unknown
ASV.705	14	K_Bacteria	P_Bacteroidetes	C_Flavobacteriia	O_Flavobacteriales	F_unknown	G_unknown	S_unknown
ASV.706	14	K_Bacteria	P_Proteobacteria	C_Alphaproteobacteria	O_Rickettsiales	F_SM2D12	G_unknown	S_unknown
ASV.703	14	K_Bacteria	P_Proteobacteria	C_Alphaproteobacteria	O_Rickettsiales	F_SM2D12	G_unknown	S_unknown
ASV.704	14	K_Bacteria	P_Bacteroidetes	C_Sphingobacteriia	O_Sphingobacteriales	F_Saprospiraceae	G_uncultured	S_unknown
ASV.708	13	K_Bacteria	P_Proteobacteria	C_Alphaproteobacteria	O_Alphaproteobacteria Incertae Sed	F_Unknown Family	G_uncultured	S_uncultured bacterium
ASV.707	13	K_Bacteria	P_Bacteroidetes	C_Cytophagia	O_Cytophagales	F_Flammeovirgaceae	G_unknown	S_unknown
ASV.712	12	K_Bacteria	P_Cyanobacteria	C_Chloroplast	O_uncultured diatom	F_unknown	G_unknown	S_unknown
ASV.711	12	K_Bacteria	P_Bacteroidetes	C_Cytophagia	O_Cytophagales	F_Flammeovirgaceae	G_uncultured	S_uncultured Bacteroidetes bacte
ASV.710	12	K_Bacteria	P_Bacteroidetes	C_Flavobacteriia	O_Flavobacteriales	F_Flavobacteriaceae	G_Tenacibaculum	S_uncultured bacterium
ASV.709	12	K_Bacteria	P_Bacteroidetes	C_Flavobacteriia	O_Flavobacteriales	F_Flavobacteriaceae	G_NS4 marine group	S_uncultured bacterium
ASV.713	12	K_Bacteria	P_Bacteroidetes	C_Flavobacteriia	O_Flavobacteriales	F_Flavobacteriaceae	G_Lutibacter	S_uncultured bacterium
ASV.715	11	K_Bacteria	P_Planctomycetes	C_Phycisphaerae	O_Phycisphaerales	F_Phycisphaeraceae	G_SM1A02	S_uncultured organism
ASV.718	11	K_Bacteria	P_Cyanobacteria	C_Chloroplast	O_uncultured bacterium	F_unknown	G_unknown	S_unknown
ASV.716	11	K_Bacteria	P_Bacteroidetes	C_Bacteroidetes Incertae Se	O_Order II	F_Rhodothermaceae	G_uncultured	S_uncultured bacterium
ASV.717	11	K_Bacteria	P_Bacteroidetes	C_Flavobacteriia	O_Flavobacteriales	F_Flavobacteriaceae	G_NS4 marine group	S_uncultured bacterium
ASV.714	11	K_Bacteria	P_Actinobacteria	C_Acidimicrobiia	O_Acidimicrobiales	F_OM1 clade	G_unknown	S_unknown

Sequence
TGGGGAATTTTCCGCAATGGGCGAAAGCCTGACGGAGCAACGCCGCGTGAGGGACGAAGGCCTCTGGGCTGTAAACCTCTTTT
TGGGGAATTTTCCGCAATGGGCGAAAGCCTGACGGAGCAACGCCGCGTGAGGGACGAAGGCCTCTGGGCTGTAAACCTCTTTT
TAGGGAATATTGGACAATGGGGGCAACCCTGATCCAGCAATGCAGCGTGAGTGACGAAGGCCTTAGGGTTGTAAAACTCTTTC
CAGGGAATATTGCGCAATGAGCGAAAGCTTGACGCAGCGACACCGCGTGTGGGATGACGGATCTAGGTTTGTAAACCACTTTC
TGGGGAATCTTGCACAATGGGGGAAACCCTGATGCAGCGATGCCGCGTGAGTGAAGAAGGCCCTTGGGTTGTAAAACTCTTTC
TGGGGAATCTTGCACAATGGAGGAAACTCTGATGCAGCGATGCCGCGTGAGTGAAGAAGGCCTTTGGGTTGTAAAGCTCTTTC
TGGGGAATCTTGCACAATGGAGGAAACTCTGATGCAGCGATGCCGCGTGAGTGAAGAAGGCCCTTGGGTTGTAAAGCTCTTTC
TGGGGAATATTGGACAATGGGGGCAACCCTGATCCAGCGATGCCGCGTGAGTGATGAAGGCCCTAGGGTTGTAAAACTCTTTC
TAGGGAATATTGCACAATGGAGGAAACTCTGATGCAGCGATGCCGCGTGAGTGAAGAAGGCCTTTGGGTTGTAAAGCTCTTTC
TAGGGAATCTTGCACAATGGGGGAAACCCTGATGCAGCGATGCCGCGTGAGTGAAGAAGGCCTTTGGGTTGTAAAGCTCTTTC
TCGAGAATCTTCGGCAATGGGCGAAAGCCTGACCGAGCGACGCCGCGTGCGGGATGAAGGCCCTCGGGTTGTAAACCGCTGTC
TAAGGAATATTGGTCAATGGACGAAAGTCTGAACCAGCCATGCCGCGTGCAGGATGACTGCCCTATGGGTTGTAAACTGCTTTT
TCGGGAATATTGGACAATGGAGGAAACTCTGATCCAGCAATGCCGCGTGTGTGATGAAGGCCCTAGGGTTGTAAAGCACTTTC
TGGGGAATCTTGCACAATGGGGGAAACCCTGATGCAGCGATGCCGCGTGAGTGAAGAAGGCCTTTGGGTTGTAAAACTCTTTC
TGGGGAATCTTGCACAATGGGGGAAACCCTGATGCAGCGATGCCGCGTGAGTGAAGAAGGCCCTTGGGTTGTAAAACTCTTTC
TGGGGAATATTGGACAATGGGGGCAACCCTGATCCAGCAATGCCGCGTGTGTGATGAAGGCCCTAGGGTTGTAAAGCACTTTC
TAGGGAATATTGGACAATGGGGGAAACCCTGATCCAGCAATGCCGCGTGAGTGAAGAAGGCCCTCGGGTTGTAAAACTCTTTC
CAGGGAATATTGCGCAATGAGCGAAAGCTTGACGCAGCGACACCGCGTGTGGGATGACGGATCTAGGTTTGTAAACCACTTTC
TAGGGAATATTGCACAATGGAGGAAACTCTGATGCAGCGATGCCGCGTGAGTGAAGAAGGCCTTTGGGTTGTAAAGCTCTTTC
TAGGGAATATTGGACAATGGGGGAAACCCTGATCCAGCAATGCCGCGTGAGTGAAGAAGGCCCTCGGGTTGTAAAACTCTTTC
TGGGGAATCTTGGACAATGGGGGCAACCCTGATCCAGCCATGCCGCGTGAGTGAAGAAGGCCCTAGGGTCGTAAAACTCTTTC
TGGGGAATATTGGACAATGGAGGCAACTCTGATCCAGCAATGCCGCGTGTGTGATGAAGGCCCTAGGGTTGTAAAGCACTTTC
TGAGGAATATTGGGCAATGGACGAAAGTCTGACCCAGCGATGCCGCGTGAAGGATGAAGGCCTTAGGGTTGTAAACTTCTGTC
TGGGGAATTTTCCGCAATGGGCGAAAGCCTGACGGAGCAACGCCGCGTGAGGGATGAAGGCCTCTGGGCTGTAAACCTCTTTT
TAGGGAATATTGCGCAATGGGGGCAACCCTGACGCAGCAATGCCGCGTGAGTGAAGAAGGCCTTCGGGTCGTAAAGCTCTTTT
TGGGGAATATTGGACAATGGAGGCAACTCTGATCCAGCAATGCCGCGTGTGTGATGAAGGCCCTAGGGTTGTAAAGCACTTTC
TGGGGAATTTTCCGCAATGGGCGAAAGCCTGACGGAGCAACGCCGCGTGAGGGACGAAGGCCTCTGGGCTGTAAACCTCTTTT
TGGGGAATTTTCCGCAATGGGCGAAAGCCTGACGGAGCAACGCCGCGTGAGGGACGAAGGCCTCTGGGCTGTAAACCTCTTTT
TAGGGAATCTTGGACAATGGGGGCAACCCTGATCCAGCCATGCCGCGTGAGTGAAGAAGGCCCTAGGGTCGTAAAGCTCTTTC
TGGGGAATATTGGACAATGGGGGCAACCCTGATCCAGCAATGCCGCGTGTGTGATGAAGGCCCTAGGGTTGTAAAGCACTTTC
TGGGGAATTTTCCGCAATGGGCGAAAGCCTGACGGAGCAACGCCGCGTGAGGGAAGAAGGCCTCTGGGCTGTAAACCTCTTTT
TGGGGAATTTTCCGCAATGGGCGAAAGCCTGACGGAGCAACGCCGCGTGAGGGATGAAGGCCTCTGGGCTGTAAACCTCTTTT
TAGGGAATATTGGACAATGGGGGAAACCCTGATCCAGCAATGCCGCGTGAGTGAAGAAGGCCCTCGGGTTGTAAAACTCTTTC
TGGGGAATCTTGCACAATGGGGGAAACCCTGATGCAGCGATGCCGCGTGAGTGAAGAAGGCCCTTGGGTTGTAAAACTCTTTC
TGGGGAATCTTGCACAATGGAGGAAACTCTGATGCAGCGATGCCGCGTGAGTGAAGAAGGCCTTTGGGTTGTAAAGCTCTTTC
TAGGGAATCTTGCACAATGGGGGAAACCCTGATGCAGCGATGCCGCGTGAGTGAAGAAGGCCTTTGGGTTGTAAAGCTCTTTC
TAGGGAATCTTGGACAATGGGGGCAACCCTGATCCAGCCATGCCGCGTGAGTGAAGAAGGCCTTAGGGTCGTAAAGCTCTTTC
TGGGGAATATTGGACAATGGGGGAAACCCTGATCCAGCAATGCCGCGTGAGTGATGAAGGCCTTAGGGTTGTAAAGCTCTTTC
TAGGGAATATTGCACAATGGAGGAAACTCTGATGCAGCGATGCCGCGTGAGTGAAGAAGGCCTTTGGGTTGTAAAGCTCTTTC
TGAGGAATATTGGTCAATGGACGAAAGTCTGAACCAGCCATGCCGCGTGAAGGATGACGGCCCTACGGGTTGTAAACTTCTTTT
TAGGGAATATTGGACAATGGGGGAAACCCTGATCCAGCAATGCCGCGTGAGTGAAGAAGGCCTTAGGGTTGTAAAACTCTTTC
TGAGGAATATTGGACAATGGACGAAAGTCTGATCCAGCCATGCCGCGTGCAGGATGACGGCCCTATGGGTTGTAAACTGCTTTT
TGGGGAATCTTGCACAATGGAGGAAACTCTGATGCAGCGATGCCGCGTGAGTGAAGAAGGCCTTTGGGTTGTAAAGCTCTTTC
TAGGGAATATTGGACAATGGGGGCAACCCTGATCCAGCAATGCAGCGTGAGTGACGAAGGCCTTAGGGTTGTAAAACTCTTTC
TAACGAATATTCCGCAATGCACGAAAGTGTGACGGAGCAATGCCGCGTGCAGGATGAATCCCCTCGGGGTGTAAACTGCTGTCA
TAGGGAATATTGGACAATGGGGGAAACCCTGATCCAGCCATCCCGCGTGCAGGAAGAATGCCCTATGGGTTGTAAACTGCTTTT
TGGGGAATCTTGCACAATGGGGGAAACCCTGATGCAGCGATGCCGCGTGAGTGAAGAAGGCCCTTGGGTTGTAAAACTCTTTC
TGAGGAATATTGGACAATGGACGAAAGTCTGATCCAGCTATGCCGCGTGCAGGATGACAGCCCTACGGGTTGTAAACTGCTTTT
TGAGGAATATTGGACAATGGACGAAAGTCTGATCCAGCCATGCCGCGTGCAGGATGACGGCCCTATGGGTTGTAAACTGCTTTT

TGGGGAATTTTCCGCAATGGGCGAAAGCCTGACGGAGCAACGCCGCGTGAGGGACGAAGGCCTCTGGGCTGTAAACCTCTTTT
TAGGGAATCTTGCGCAATGGGGGCAACCCTGACGCAGCAACGCCGCGTGTGGGATGACGGCTTTCGGGTTGTAAACCACTTTC
TAAGGAATATTGGACAATGGGCGAAAGCCTGATCCAGCCATGCCGCGTGCAGGATGACTGCCCTATGGGTTGTAAACTGCTTTT
TGGGGAATATTGGACAATGGGGGAAACCCTGATCCAGCAATGCCGCGTGAGTGATGAAGGCCTTAGGGTTGTAAAGCTCTTTC
TAGGGAATATTGGACAATGGGGGAAACCCTGATCCAGCAATGCCGCGTGAGTGAAGAAGGCCCTCGGGTTGTAAAACTCTTTC
GCGCGAAAACTTCACACTGCAGGAAACTGTGATGAGGGAACTCCTAGTGCATGCACTATGTGTATGTAGATCCTACTCTTAACA
TAAGGAATCTTGCACAATGGGGGAAACCCTGATGCAGCGATGCCGCGTGAGTGAAGAAGGCCCTTGGGTTGTAAAACTCTTTC
TGGGGAATATTGGACAATGGGGGCAACCCTGATCCAGCGATGCCGCGTGAGTGATGAAGGCCTTAGGGTTGTAAAACTCTTTC
TGGGGAATATTGGACAATGGGCGAAAGCCTGATCCAGCCATGCCGCGTGAGTGAAGAAGGCCTTAGGGTTGTAAAACTCTTTC
TAGGGAATATTGGACAATGGGGGAAACCCTGATCCAGCAATGCCGCGTGAGTGAAGAAGGCCTTAGGGTTGTAAAACTCTTTC
TAGGGAATCTTGCACAATGGGGGAAACCCTGATGCAGCGATGCCGCGTGAGTGAAGAAGGCCTTTGGGTTGTAAAGCTCTTTC
TGGGGAATTTTCCGCAATGGGCGAAAGCCTGACGGAGCAACGCCGCGTGAGGGACGAAGGCCTCTGGGCTGTAAACCTCTTTT
TGGGGAATATTGGACAATGGGCGAAAGCCTGATCCAGCAATGCAGCGTGTGTGAAGAAGGCCTGAGGGTTGTAAAGCACTTT
TGAGGAATATTGGACAATGGACGAAAGTCTGATCCAGCTATGCCGCGTGCAGGATGACAGCCCTACGGGTTGTAAACTGCTTT
TGGGGAATTTTCCGCAATGGGCGAAAGCCTGACGGAGCAACGCCGCGTGAGGGACGAAGGCCTTTGGGCTGTAAACCTCTTTT
TAGGGAATTTTGCGCAATGGGCGAAAGCCTGACGCAGCAATGCCGCGTGAGTGAAGAAGGCCCTCGGGTCGTAAAGCTCTTTA
TGGGGAATATTGGACAATGGGGGCAACCCTGATCCAGCAATGCCGCGTGTGTGATGAAGGCCCTAGGGTTGTAAAGCACTTTC
TGGGGAATTTTCCGCAATGGGCGAAAGCCTGACGGAGCAATACCGCGTGAGGGAGGAAGGCCTTTGGGTTGTAAACCTCTTTT
TAAGGAATATTGGACAATGGAGGCAACTCTGATCCAGCCATGCCGCGTGAAGGAAGACGGCCTTATGGGTTGTAAACTTCTTTT
TGAGGAATATTGGTCAATGGACGAAAGTCTGAACCAGCCATGCCGCGTGAAGGATGACGGCCCTACGGGTTGTAAACTTCTTT
TGGGGAATATTGCGCAATGGGGGAAACCCTGACGCAGCGATGCCGCGTGAGTGAAGAAGGCCCTAGGGTTGTAAAACTCTTT
CAGGGAATATTGTGCAATGAACGAAAGTTTGACACAGCGACACCGCGTGTGGGATGACGGATCTAGGTTTGTAAACCACTTTC
TAGGGAATCTTGGACAATGGGCGAAAGCCTGATCCAGCCATGCCGCGTGAGTGATGAAGGCCCTAGGGTTGTAAAGCTCTTTC
TAAGGAATCTTGCACAATGGAGGAAACTCTGATGCAGCGATGCCGCGTGAGTGAAGAAGGCCCTTGGGTTGTAAAGCTCTTTC
TAGGGAATCTTGCACAATGGGCGAAAGCCTGATGCAGCCATGCCGCGTGAGTGAAGAAGGCTCTAGGGTTGTAAAACTCTTTC
TAGGGAATATTGCACAATGGAGGAAACTCTGATGCAGCGATGCCGCGTGAGTGAAGAAGGCCCTTGGGTTGTAAAGCTCTTTC
TGAGGAATTTTCCGCAATGGGCGAAAGCCTGACGGAGCAATACCGCGTGAGGGATGAAGGATTTTGGTCTGTAAACCTCTTTT
TGAGGAATATTGGACAATGGAGGCAACTCTGATCCAGCCATGCCGCGTGCAGGAAGACGGCCCTATGGGTTGTAAACTGCTTT
TGGGGAATATTGGACAATGGGGGCAACCCTGATCCAGCGATGCCGCGTGAGTGATGAAGGCCCTAGGGTTGTAAAACTCTTTC
TGAGGAATATTGGACAATGGACGAAAGTCTGATCCAGCTATGCCGCGTGCAGGATGACGGCCCTATGGGTTGTAAACTGCTTT
TGAGGAATATTGGACAATGGACGAAAGTCTGATCCAGCTATGCCGCGTGCAGGATGACAGCCCTACGGGTTGTAAACTGCTTT
TGAGGAATATTGGACAATGGACGAAAGTCTGATCCAGCCATGCCGCGTGCAGGATGACGGCCCTATGGGTTGTAAACTGCTTT
TGGGGAATATTGCACAATGGGCGCAAGCCTGATGCAGCCATGCCGCGTGAGTGAAGAAGGCCTTAGGGTTGTAAAGCTCTTTC
TGGGGAATATTGGACAATGGGGGAAACCCTGATCCAGCAATGCCGCGTGTGTGAAGAAGGCCTTAGGGTTGTAAAGCACTTTC
TGAGGAATATTGCGCAATGGAGGAAACTCTGACGCAGCCATGCCGCGTGCAGGAAGACTGCCCTATGGGTTGTAAACTGCTTT
TAAGGAATATTGGTCAATGGACGAAAGTCTGAACCAGCCATGCCGCGTGCAGGATGACGGCCCTATGGGTTGTAAACTGCTTT
TGGGGAATCTTGGACAATGGGGGCAACCCTGATCCAGCCATGCCGCGTGAGTGAAGAAGGCCCTAGGGTCGTAAAACTCTTTC
TAGGGAATCTTGCACAATGGGGGAAACCCTGATGCAGCGATGCCGCGTGAGTGAAGAAGGCCTTTGGGTTGTAAAGCTCTTTC
TTTCGAATCATTCACAATGGGGGAAACCCTGATGGTGCAACGCCGCGTGAGGGATGAAGGCCTTCGGGTCGTAAACCTCTGTCA
TGAGGAATATTGGTCAATGGACGAAAGTCTGAACCAGCCATGCCGCGTGAAGGATGACTGCCCTATGGGTTGTAAACTTCTTTT
TGAGGAATATTGCGCAATGGAGGAAACTCTGACGCAGCCATGCCGCGTGCAGGATGAATGCCCTATGGGTTGTAAACTGCTTT
TGAGGAATATTGGACAATGGAGGCAACTCTGATCCAGCCATGCCGCGTGCAGGAAGACTGCCTTATGGGTTGTAAACTGCTTTT
TAGGGAATATTGGTCAATGGGCGAGAGCCTGAACCAGCCATGCCGCGTGCAGGAAGACGGCCTTCTGGGTTGTAAACTGCTTT
TGGGGAATATTGGACAATGGGGGCAACCCTGATCCAGCAATGCCGCGTGTGTGATGAAGGCCTTAGGGTTGTAAAGCACTTTC
CAGGGAATATTGCGCAATGAGCGAAAGCTTGACGCAGCGACACCGCGTGTGGGATGACGGATCTAGGTTTGTAAACCACTTTC
TGAGGAATATTGGACAATGGAGGCAACTCTGATCCAGCCATGCCGCGTGCAGGAAGACTGCCCTATGGGTTGTAAACTGCTTTT
TGGGGAATATTGGACAATGGGCGAAAGCCTGATCCAGCAATGCAGCGTGTGTGAAGAAGGCCTGCGGGTTGTAAAGCACTTT
TGAGGAATATTGGACAATGGACGAAAGTCTGATCCAGCCATGCCGCGTGCAGGATGACGGCCCTATGGGTTGTAAACTGCTTT
TGAGGAATATTGCGCAATGGAGGAAACTCTGACGCAGCCATGCCGCGTGCAGGAAGACTGCCCTATGGGTTGTAAACTGCTTT
TGGGGAATTTTCCGCAATGGGCGAAAGCCTGACGGAGCAACGCCGCGTGAGGGACGAAGGCCTCTGGGCTGTAAACCTCTTTT

TGAGGAATTTTCTGCAATGGGCGAAAGCCTGACAGAGCAATACCGCGTGAGGGATGACTGCCTACGGGTTGTAAACCTCTTTTC
TGGGGAATATTGGACAATGGGGGAAACCCTGATCCAGCAATGCCGCGTGTGTGAAGAAGGCCTTAGGGTTGTAAAACACTTTC
TGAGGAATATTGGACAATGGAGGCAACTCTGATCCAGCCATGCCGCGTGCAGGAAGACTGCCCTATGGGTTGTAAACTGCTTTT
TGAGGAATATTGGACAATGGAGGCAACTCTGATCCAGCCATGCCGCGTGCAGGAAGAAGGCCCTACGGGTTGTAAACTGCTTT
TGGGGAATATTGGACAATGGGGGAAACCCTGATCCAGCAATGCCGCGTGAGTGATGAAGGCCTTAGGGTTGTAAAACTCTTTC
TCGGGAATATTGGACAATGGAGGAAACTCTGATCCAGCAATGCCGCGTGTGTGATGAAGGCCCTAGGGTTGTAAAGCACTTTC
TGGGGAATTTTCCGCAATGGGCGAAAGCCTGACGGAGCAACGCCGCGTGAGGGACGAAGGCCTCTGGGCTGTAAACCTCTTTT
TAGGGAATATTGGACAATGGGGGAAACCCTGATCCAGCAATGCCGCGTGAGTGAAGAAGGCCCTCGGGTTGTAAAACTCTTTC
TGGGGAATATTGGACAATGGGCGAAAGCCTGATCCAGCAATGCCGCGTGTGCGATGAAGGCCTTCGGGTCGTAAAGCACTTTC
TGAGGAATATTGGACAATGGAGGAAACTCTGATCCAGCCATGCCGCGTGCAGGATGAATGCCCTATGGGTTGTAAACTGCTTT
TGGGGAATCTTGCACAATGGAGGAAACTCTGATGCAGCGATGCCGCGTGAGTGAAGAAGGCCTTTGGGTTGTAAAGCTCTTTT
TGAGGAATATTGGACAATGGAGGCAACTCTGATCCAGCCATGCCGCGTGCAGGAAGACTGCCCTATGGGTTGTAAACTGCTTTT
TGAGGAATATTGGACAATGGACGAAAGTCTGATCCAGCCATGCCGCGTGCAGGATGAATGCCCTATGGGTTGTAAACTGCTTT
CAGGGAATATTGTGCAATGAACGAAAGTTTGACACAGCGACACCGCGTGTGGGATGACGGATCTAGGTTTGTAAACCACTTTCA
TGAGGAATATTGGGCAATGGAGGCAACTCTGACCCAGCCATGCCGCGTGCAGGAAGACGGCCCTATGGGTTGTAAACTGCTTT
TGGGGAATTTTCCGCAATGGGCGAAAGCCTGACGGAGCAACGCCGCGTGAGGGACGAAGGCCTCTGGGCTGTAAACCTCTTTT
TAGGGAATCTTGCACAATGGGGGAAACCCTGATGCAGCGATGCCGCGTGAGTGAAGAAGGCCTTTGGGTTGTAAAGCTCTTTC
TGAGGAATATTGGACAATGGACGAAAGTCTGATCCAGCTATGCCGCGTGCAGGACGACGGCCCTATGGGTTGTAAACTGCTTT
TAGGGAATATTGGACAATGGGGGAAACCCTGATCCAGCAATGCCGCGTGAGTGAAGAAGGCCCTCGGGTTGTAAAACTCTTTC
TGGGGAATATTGGACAATGGGGGCAACCCTGATCCAGCAATGCCGCGTGTGTGATGAAGGCCTTAGGGTTGTAAAGCACTTTC
TAGGGAATATTGGACAATGGGGGAAACCCTGATCCAGCAATGCCGCGTGAGTGAAGAAGGCCCTCGGGTTGTAAAACTCTTTC
TGAGGAATATTGGACAATGGAGGAAACTCTGATCCAGCCATGCCGCGTGCAGGATGAATGCCCTATGGGTTGTAAACTGCTTT
CAGGGAATCTTGCACAATGGGCGAAAGCCTGATGCAGCGACGCCGCGTGAGGGATGAAGGTTTTAGGATCGTAAACCTCTTTT
TGGGGAATTTTCTGCAATGGGCGAAAGCCTGACAGAGCAATACCGCGTGAGGGATGAAGGCTCGTGGGTCGTAAACCTCTTTT
TGAGGAATATTGGGCAATGGAGGGAACTCTGACCCAGCCATGCCGCGTGCAGGAAGACTGCCCTATGGGTTGTAAACTGCTTT
TGAGGAATTTTCTGCAATGGGCGAAAGCCTGACAGAGCAATACCGCGTGAGGGATGACTGCCTACGGGTTGTAAACCTCTTTTC
TAAGGAATATTGCACAATGGAGGAAACTCTGATGCAGCCATGCCGCGTGCAGGAAGACTGCCCTATGGGTTGTAAACTGCTTTT
TGAGGAATATTGCACAATGGAGGAAACTCTGATGCAGCCATGCCGCGTGCAGGACGACTGCCCTATGGGTTGTAAACTGCTTTT
TGAGGAATATTGCACAATGGAGGAAACTCTGATGCAGCCATGCCGCGTGCAGGATGACGGCCCTACGGGTTGTAAACTGCTTT
TGAGGAATATTGGACAATGGAGGAAACTCTGATCCAGCCATGCCGCGTGCAGGAAGACTGCCCTATGGGTTGTAAACTGCTTT
TGGGGAATCTTGCGCAATGGGCGAAAGCCTGACGCAGCAACGCCGCGTGTGTGAAGAAGGCCCTCGGGTCGTAAAGCACTATA
TGGGGAATCTTGCACAATGGGGGAAACCCTGATGCAGCGATGCCGCGTGAGTGAAGAAGGCCTTTGGGTTGTAAAACTCTTTC
TAAGGAATCTTGCACAATGGAGGAAACTCTGATGCAGCGATGCCGCGTGAGTGAAGAAGGCCTTTGGGTTGTAAAGCTCTTTC
TGGGGAATATTGGACAATGGGGGAAACCCTGATCCAGCAATGCCGCGTGAGTGATGAAGGCCCTAGGGTTGTAAAACTCTTTC
TGGGGAATATTGGACAATGGAGGCAACTCTGATCCAGCAATGCCGCGTGTGTGATGAAGGCCTTAGGGTTGTAAAGCACTTTC
TCGGGAATATTGGACAATGGAGGAAACTCTGATCCAGCAATGCCGCGTGTGTGATGAAGGCCCTAGGGTTGTAAAGCACTTTC
TGAGGAATATTGGACAATGGAGGCAACTCTGATCCAGCCATGCCGCGTGCAGGAAGACGGCCCTATGGGTTGTAAACTGCTTT
TGGGGAATATTGGACAATGGGCGAAAGCCTGATCCAGCCATGCCGCGTGAGTGAAGAAGGCCTTAGGGTTGTAAAACTCTTTC
TAGGGAATATTGCACAATGGGCGAAAGCCTGATGCAGCAACGCCGCGTGCGGGAAGAAGGCCCTAGGGTTGTAAACCGCTTTC
TAGGGAATTTTGCGCAATGGGCGAAAGCCTGACGCAGCAATGCCGCGTGAGTGAAGAAGGCCTTCGGGTCGTAAAGCTCTTTA
TGGGGAATCTTGCACAATGGAGGAAACTCTGATGCAGCGATGCCGCGTGAGTGAAGAAGGCCTTTGGGTTGTAAAGCTCTTTT
TGGGGAATATTGGACAATGGGGGCAACCCTGATCCAGCCATGCCGCGTGAGTGAAGAAGGCCCTAGGGTTGTAAAGCTCTTTC
TTTCGAATCATTCACAATGGGCGAAAGCCTGATGATGCAATGCCGCGTGGAGGATGAAGGCCCTAGGGTCGTAAACTCCTGTCA
GCGCGAAAACTTGGCAATGCGAGAAATCGTGACCAGGGAACTCCAAGTGGCAGGGGTAAGACCCTGTCTTTTTCCGACTCTAAA
TGAGGAATATTGGACAATGGAGGAAACTCTGATCCAGCCATGCCGCGTGCAGGAAGACGGCCCTATGGGTTGTAAACTGCTTT
TAACGAATATTCCGCAATGCACGAAAGTGTGACGGAGCAATGCCGCGTGCAGGATGAATCCCCTCGGGGTGTAAACTGCTGTCA
TGAGGAATATTGGACAATGGAGGCAACTCTGATCCAGCCATGCCGCGTGCAGGAAGACAGCCCTATGGGTTGTAAACTGCTTTT
TAGGGAATATTGGACAATGGGGGGAACCCTGATCCAGCAATGCCGCGTGAGTGAAGAAGGCCTTAGGGTTGTAAAACTCTTTC
TGAGGAATATTGGACAATGGACGAAAGTCTGATCCAGCTATGCCGCGTGCAGGATGACAGCCCTACGGGTTGTAAACTGCTTTT
GCGCGAAAACTTCACACTGCAGGAAACTGTGATGAGGGAACTCCAAGTGCGTGCACTTAGTGTGCGCTTTTGTTGACTATTAAT

CAGGGAATCTTGCACAATGGGCGAAAGCCTGATGCAGCGACGCCGCGTGAGGGATGAAGGTTTTAGGATCGTAAACCTCTTTT
TAGGGAATCTTGGACAATGGGCGAAAGCCTGATCCAGCCATGCCGCGTGAGTGATGAAGGCCCTAGGGTTGTAAAACTCTTTC
GCGCGAAAACTTCGCAATGCGGGAAACCGTGACGAGGGAACTCCAAGTGTCTATGCAATGCATAGACTGTTTTTCAGCCTAAAT
TAAGGAATATTGGACAATGGGCGAAAGCCTGATCCAGCCATGCCGCGTGCAGGATGACTGCCCTATGGGTTGTAAACTGCTTTT
TCGGGAATATTGGACAATGGAGGAAACTCTGATCCAGCAATGCCGCGTGTGTGATGAAGGCCTTAGGGTTGTAAAGCACTTTC
TGGGGAATTTTCTGCAATGGGCGAAAGCCTGACAGAGCAATACCGCGTGAGGGATGAAGGCTCGTGGGTCGTAAACCTCTTTT
TGGGGAATATTGGACAATGGGGGCAACCCTGATCCAGCCATGCCGCGTGAGTGAAGAAGGCCCTAGGGTTGTAAAGCTCTTTC
TGGGGAATCTTGGACAATGGGCGCAAGCCTGATCCAGCCACGCCGCGTGAGTGATGAAGGCCTTAGGGTTGTAAAGCTCTGTC
TAAGGAATCTTGCACAATGGAGGAAACTCTGATGCAGCGATGCCGCGTGAGTGAAGAAGGCCTTTGGGTTGTAAAGCTCTTTC
TGGGGAATATTGGACAATGGGGGCAACCCTGATCCAGCAATGCCGCGTGTGTGATGAAGGCCTTAGGGTTGTAAAGCACTTTC
TGGGGAATATTGGACAATGGGGGCAACCCTGATCCAGCAATGCCGCGTGTGTGATGAAGGCCCTAGGGTTGTAAAGCACTTTC
TGGGGAATATTGGACAATGGGCGAAAGCCTGATCCAGCCATGCCGCGTGAGTGAAGAAGGCCTTAGGGTTGTAAAACTCTTTC
TGGGGAATATTGGACAATGGGGGCAACCCTGATCCAGCGATGCCGCGTGAGTGATGAAGGCCTTCGGGTTGTAAAACTCTTTC
TGAGGAATCTTGCGCAATGGGCGAAAGCCTGACGCAGCCACGCCGCGTGCCGGATGAAGGCCCTATGGGTTGTAAACGGCTTT
TGGGGAATTTTCCGCAATGGGCGAAAGCCTGACGGAGCAATACCGCGTGAGGGAGGAAGGCCTTTGGGTTGTAAACCTCTTTT
TCGGGAATATTGGACAATGGAGGAAACTCTGATCCAGCAATGCCGCGTGTGTGATGAAGGCCCTAGGGTTGTAAAGCACTTTC
TGGGGAATATTGGACAATGGGGGCAACCCTGATCCAGCAATGCCGCGTGTGTGATGAAGGCCCTAGGGTTGTAAAGCACTTTC
TAAGGAATCTTGCACAATGGGGGAAACCCTGATGCAGCGATGCCGCGTGAGTGAAGAAGGCCTTTGGGTTGTAAAACTCTTTC
TGGGGAATATTGGACAATGGGGGCAACCCTGATCCAGCAATGCCGCGTGTGTGACGAAGGCCTTAGGGTTGTAAAGCACTTTC
TAAGGAATATTGGGCAATGGGCGCAAGCCTGACCCAGCCATGCCGCGTGCAGGATGACGACCCTATGGGTTGTAAACTGCTTTT
TGGGGAATATTGGACAATGGGGGAAACCCTGATCCAGCAATGCCGCGTGAGTGATGAAGGCCTTAGGGTTGTAAAGCTCTTTC
TGAGGAATATTGGACAATGGAGGCAACTCTGATCCAGCCATGCCGCGTGCAGGAAGACTGCCCTATGGGTTGTAAACTGCTTTT
TGGGGAATATTGGACAATGGGGGAAACCCTGATCCAGCAATGCCGCGTGAGTGATGAAGGCCTTAGGGTTGTAAAGCTCTTTC
TGGGGAATTTTCCGCAATGGGCGAAAGCCTGACGGAGCAATACCGCGTGAGGGATGAAGGCTTACTGAGTTGTAAACCTCTTT
TGGGGAATTTTCCGCAATGGGCGAAAGCCTGACGGAGCAACGCCGCGTGAGGGACGAAGGCCTCTGGGCTGTAAACCTCTTTT
GCGCGAAAACTTCACACTGCAGGAAACTGTGATGAGGGAACTCCAAGTGACTGCCCAATGGGTAGCCTTTTCTTGAATCTTAATA
CAGGGAATCTTGCACAATGGGCGAAAGCCTGATGCAGCGACGCCGCGTGAGGGATGAAGGTTCTAGGATCGTAAACCTCTTTT
TGGGGAATATTGGACAATGGGGGAAACCCTGATCCAGCAATGCCGCGTGTGTGAAGAAGGCCCTAGGGTTGTAAAGCACTTTC
TGAGGAATTTTCTGCAATGGGCGCAAGCCTGACAGAGCAATACCGCGTGAGGGATGACGGCCTATGGGTTGTAAACCTCTTTTC
TGGGGAATTTTGCGCAATGGGGGAAACCCTGACGCAGCAATGCCGCGTGAGCGATGAAGGCCTGATGGTTGTAAAGCTCTTTC
TTTCGAATCTTTCACAATGGGCGCAAGCCTGATGGAGCAACGCCGCGTGGGGGATGACGGCCTTCGGGTTGTAAACCCCTGTCA
TAGGGAATATTGGACAATGGGGGAAACCCTGATCCAGCAATGCCGCGTGAGTGAAGAAGGCCCTCGGGTTGTAAAACTCTTTC
TGAGGAATATTGCGCAATGGAGGAAACTCTGACGCAGCCATACCGCGTGTAGGAAGAATGTCCTATGGATTGTAAACTACTTTT
TGGGGAATATTGCACAATGGGCGAAAGCCTGATGCAGCAATACCGCGTGTGTGAAGAAGGCCTGCGGGTTGTAAAGCACTTTC
TGGGGAATTTTCTGCAATGGGCGAAAGCCTGACAGAGCAATACCGCGTGAGGGATGAAGGCCTGTGGGTCGTAAACCTCTTTT
GCGCGAAAACTTCACACTGCAGGAAACTGTGATGAGGGAACTCCAAGTGCATGCACTATGTGTATGCTTTTCTTAACTATTAATC
GCGCGAAAACTTCACACTGCAGGAAACTGTGATGAGGGAACTCCAAGTGACTGCACAATGTGTAGCCTTTTCTTGAATCTTAATA
TTAGGAATATTGTGCAATGGGGGAAACCCTGACACAGCCATGCCGCGTGTGTGAAGAAGGCCCTAGGGTTGTAAAGCACTTTC
TGAGGAATATTGGACAATGGAGGCAACTCTGATCCAGCCATGCCGCGTGCAGGAAGACTGCCCTATGGGTTGTAAACTGCTTTT
TGGGGAATCTTGCACAATGGGGGAAACCCTGATGCAGCGATGCCGCGTGAGTGAAGAAGGCCCTTGGGTTGTAAAACTCTTTC
TGGGGAATCTTGCACAATGGGGGAAACCCTGATGCAGCGATGCCGCGTGAGTGAAGAAGGCCCTTGGGTTGTAAAACTCTTTC
TTAGGAATATTGTGCAATGGGGGAAACCCTGACACAGCCATGCCGCGTGTGTGAAGAAGGCCCTAGGGTTGTAAAGCACTTTC
TAGGGAATCTTGGACAATGGGCGCAAGCCTGATCCAGCCATGCCGCGTGAGTGAAGAAGGCCCTAGGGTCGTAAAGCTCTTTC
TGAGGAATTTTCCGCAATGGGCGAAAGCCTGACGGAGCAATACCGCGTGAGGGATGAAGGATTTTGGTCTGTAAACCTCTTTTC
TGGGGAATATTGCGCAATGGGCGAAAGCCTGACGCAGCCAACCCGCGTGTGTGATGAAGGCCCTAGGGTTGTAAAGCACTGTC
TGGGGAATCTTGCACAATGGGGGAAACCCTGATGCAGCGATGCCGCGTGAGTGAAGAAGGCCCTTGGGTTGTAAAACTCTTTC
TTTCGAATCATTCACAATGGGGGAAACCCTGATGGTGCAACGCCGCGTGAGGGATGAAGGCCTTCGGGTCGTAAACCTCTGTCA
TGGGGAATATTGGACAATGGGGGCAACCCTGATCCAGCAATGCCGCGTGTGTGACGAAGGCCCTAGGGTTGTAAAGCACTTTC
TAAGGAATCTTGCACAATGGGGGAAACCCTGATGCAGCGATGCCGCGTGAGTGAAGAAGGCCCTTGGGTTGTAAAACTCTTTC
TGGGGAATATTGGACAATGGGCGAAAGCCTGATCCAGCCATGCCGCGTGAGTGAAGAAGGCCTTAGGGTTGTAAAACTCTTTC

TGGGGAATATTGGACAATGGGCGCAAGCCTGATCCAGCCATGCCGCGTGAGTGAAGAAGGCCTTAGGGTTGTAAAACTCTTTC
TAAGGAATCTTGCACAATGGGGGAAACCCTGATGCAGCGATGCCGCGTGAGTGAAGAAGGCCCTTGGGTTGTAAAACTCTTTC
TGGGGAATCTTGCACAATGGAGGAAACTCTGATGCAGCGATGCCGCGTGAGTGAAGAAGGCCTTTGGGTTGTAAAGCTCTTTC
TGAGGAATATTGGACAATGGAGGAAACTCTGATCCAGCCATGCCGCGTGCAGGATGAATGCCCTACGGGTTGTAAACTGCTTTT
TAAGGAATCTTGCACAATGGGGGAAACCCTGATGCAGCGATGCCGCGTGAGTGAAGAAGGCCTTTGGGTTGTAAAACTCTTTC
TGAGGAATATTGGACAATGGACGAAAGTCTGATCCAGCCATGCCGCGTGCAGGATGACGGCCCTATGGGTTGTAAACTGCTTTT
TGGGGAATATTGGACAATGGGGGCAACCCTGATCCAGCAATGCCGCGTGTGTGATGAAGGCCTTAGGGTTGTAAAGCACTTTC
GCGCGAAAACTTGGCAATGCGAGAAATCGTGACCAGGGAACTCCAAGTGCGTAGGGTAAGACCTACGCTTTTCTTGACTCTTCA
TGAGGAATTTTCCGCAATGGGCGAAAGCCTGACGGAGCAATACCGCGTGAGGGATGAAGGATTTTGGTCTGTAAACCTCTTTTC
TAGGGAATCTTGCGCAATGGGCGAAAGCCTGACGCAGCAACGCCGCGTGGGGGATGAATGCCTTCGGGTTGTAAACCCCTTTC
TGGGGAATATTGCGCAATGGGCGGAAGCCTGACGCAGCAACGCCGCGTGAGTGAAGAAGGTCTTCGGATTGTAAAGCTCTGT
TGAGGAATATTGCGCAATGGAGGAAACTCTGACGCAGCCATGCCGCGTGCAGGAAGACGGCCCTACGGGTTGTAAACTGCTTT
TAGGGAATATTGGACAATGGGGGAAACCCTGATCCAGCAATGCCGCGTGAGTGAAGAAGGCCTTCGGGTTGTAAAACTCTTTC
TGGGGAATATTGGACAATGGAGGCAACTCTGATCCAGCAATGCCGCGTGTGTGATGAAGGCCCTAGGGTTGTAAAGCACTTTC
TGGGGAATTTTCCGCAATGGGCGAAAGCCTGACGGAGCAACGCCGCGTGAGGGACGAAGGCCTCTGGGCTGTAAACCTCTTTT
TGGGGAATATTGGACAATGGGCGAAAGCCTGATCCAGCAATGCCGCGTGTGTGATGAAGGCCTTCGGGTCGTAAAGCACTTTC
TGGGGAATATTGGACAATGGGCGAAAGCCTGATCCAGCCATGCCGCGTGAGTGAAGAAGGCCTTAGGGTTGTAAAACTCTTTC
TGGGGAATATTGGACAATGGGCGCAAGCCTGATCCAGCCATGCCGCGTGAGTGAAGAAGGCCCTAGGGTTGTAAAACTCTTTC
TAGGGAATTTTGCGCAATGGGCGAAAGCCTGACGCAGCAATGCCGCGTGAGTGAAGAAGGCCTTCGGGTCGTAAAGCTCTTTA
TGAGGAATATTGCGCAATGGACGAAAGTCTGACGCAGCCATGCCGCGTGCAGGATGACGGCGCTACGCGTTGTAAACTGCTTT
TGAGGAATATTGGACAATGGACGAAAGTCTGATCCAGCTATGCCGCGTGCAGGATGACAGCCCTACGGGTTGTAAACTGCTTTT
TGAGGAATATTGCGCAATGGAGGAAACTCTGACGCAGCCATGCCGCGTGCAGGAATAAGGCCCTATGGGTTGTAAACTGCTTT
TGAGGAATATTGGTCAATGGACGAAAGTCTGAACCAGCCATGCCGCGTGCAGGATGACTGCCCTATGGGTTGTAAACTGCTTTT
TAGGGAATATTGCGCAATGGACGAAAGTCTGACGCAGCGATGCCGCGTGAGTGATGACGCCTTTCGGGGTGTAAAGCTCTGTC
TAGGGAATATTGGACAATGGGGGAAACCCTGATCCAGCAATGCCGCGTGAGTGAAGAAGGCCTTCGGGTTGTAAAACTCTTTC
TTTCGAATCTTTCACAATGGGCGCAAGCCTGATGGAGCAACGCCGCGTGGGGGATGACTGCCTTCGGGTTGTAAACCCCTGTCA
TGGGGAATATTGCGCAATGGGCGAAAGCCTGACGCAGCAACGCCGCGTGCGGGATGAAGGCCTTCGGGTTGTAAACCGCTTTC
TAGGGAATATTGCACAATGGAGGAAACTCTGATGCAGCGATGCCGCGTGAGTGAAGAAGGCCTTTGGGTTGTAAAGCTCTTTC
TGGGGAATATTGGACAATGGGCGAAAGCCTGATCCAGCCATGCCGCGTGAGTGAAGAAGGCCCTAGGGTTGTAAAACTCTTTC
TGGGGAATCTTGCGCAATGGGCGAAAGCCTGACGCAGCCACGCCGCGTGAGTGATGAAGGCCCTCGGGTCGTAAAGCTCTGTC
TAGGGAATATTGGACAATGGGGGAAACCCTGATCCAGCAATGCCGCGTGAGTGAAGAAGGCCTTCGGGTTGTAAAACTCTTTC
TGAGGAATATTGCGCAATGGAGGAAACTCTGACGCAGCCATGCCGCGTGCAGGAAGACGGTCTTATGGATTGTAAACTGCTTT
TGGGGAATATTGGACAATGGGCGAAAGCCTGATCCAGCCATGCCGCGTGAGTGAAGAAGGCCTTAGGGTTGTAAAACTCTTTC
TAGGGAATATTGCACAATGGAGGAAACTCTGATGCAGCGATGCCGCGTGAGTGAAGAAGGCCTTTGGGTTGTAAAGCTCTTTC
TGGGGAATATTGGACAATGGAGGCAACTCTGATCCAGCAATGCCGCGTGTGTGATGAAGGCCCTAGGGTTGTAAAGCACTTTC
TGGGGAATATTGGACAATGGGCGAAAGCCTGATCCAGCCATGCCGCGTGAGTGAAGAAGGCCTTAGGGTTGTAAAACTCTTTC
TGAGGAATATTGGACAATGGACGAAAGTCTGATCCAGCCATGCCGCGTGCAGGATGAATGCCTTATGGGTTGTAAACTGCTTTT
TGAGGAATATTGGACAATGGGCGGAAGCCTGATCCAGCCATGCCGCGTGCAGGAAGACTGCCCTATGGGTTGTAAACTGCTTT
TGGGGAATCTTGCACAATGGGGGAAACCCTGATGCAGCGATGCCGCGTGAGTGAAGAAGGCCTTTGGGTTGTAAAACTCTTTC
TAGGGAATATTGCACAATGGGGGAAACCCTGATGCAGCCATGCCGCGTGTGTGAAGAAGGCCCTAGGGTTGTAAAGCACTTTC
TCGAGAATCTTCCGCAATGGGCGAAAGCCTGACGGAGCGACGCCGCGTGCGGGATGAAGGCCTTCGGGTTGTAAACCGCTGTC
TGGGGAATATTGCGCAATGGGGGAAACCCTGACGCAGCCATGCCGCGTGAGTGACGAAGGCCTTAGGGTTGTAAAGCTCTTTC
TGAGGAATATTGGACAATGGAGGCAACTCTGATCCAGCCATGCCGCGTGCAGGAAGACTGCCCTATGGGTTGTAAACTGCTTTT
TAGGGAATATTGGACAATGGGGGAAACCCTGATCCAGCAATGCCGCGTGAGTGAAGAAGGCCTTCGGGTTGTAAAACTCTTTC
TAAGGAATATTGGGCAATGGGCGCAAGCCTGACCCAGCCATGCCGCGTGCAGGATGACGACCCTATGGGTTGTAAACTGCTTTT
TGAGGAATATTGGACAATGGAGGCAACTCTGATCCAGCCATGCCGCGTGCAGGAAGACGGCCCTATGGGTTGTAAACTGCTTTT
TGAGGAATATTGGACAATGGAGGCAACTCTGATCCAGCCATGCCGCGTGCAGGAAGACTGCCCTATGGGTTGTAAACTGCTTTT
TGAGGAATCTTGCGCAATGGGCGAAAGCCTGACGCAGCAACGCCGCGTGTGTGATGAAGGCCCTAGGGTTGTAAAGCACTGAG
TGAGGAATATTGGACAATGGAGGAAACTCTGATCCAGCCATGCCGCGTGCAGGATGAATGCCCTATGGGTTGTAAACTGCTTTT
TGAGGAATATTGCGCAATGGACGAAAGTCTGACGCAGCGATGCCGCGTGAGTGATGACGCCTTTCGGGGTGTAAAACTCTGTC

TGGGGAATTTTCCGCAATGGGCGAAAGCCTGACGGAGCAACGCCGCGTGAGGGACGAAGGCCTCTGGGCTGTAAACCTCTTTT
TGGGGAATCTTGCACAATGGGGGAAACCCTGATGCAGCGATGCCGCGTGAGTGAAGAAGGCCTTTGGGTTGTAAAGCTCTTTC
TAAGGAATATTGGTCAATGGGGGCAACCCTGAACCAGCCATCCCGCGTGAAGGATGACAGCCCTATGGGTCGTAAACTTCTTTT
TGAGGAATATTGCGCAATGGACGAAAGTCTGACGCAGCCATGCCGCGTGCAGGATGACGGCGCTATGCGTTGTAAACTGCTTT
TGGGGAATATTGGACAATGGAGGCAACTCTGATCCAGCAATGCCGCGTGTGTGATGAAGGCCCTAGGGTTGTAAAGCACTTTC
TGGGGAATCTTGCACAATGGAGGAAACTCTGATGCAGCGATGCCGCGTGAGTGAAGAAGGCCCTTGGGTTGTAAAGCTCTTTT
TGGGGAATATTGGACAATGGGGGCAACCCTGATCCAGCAATGCCGCGTGTGTGATGAAGGCCCTAGGGTTGTAAAGCACTTTC
TGGGGAATATTGCGCAATGGGGGAAACCCTGACGCAGCCATGCCGCGTGAGTGACGAAGGCCTTAGGGTTGTAAAGCTCTTTC
TAGGGAATATTGGACAATGGGGGAAACCCTGATCCAGCAATGCCGCGTGAGTGAAGAAGGCCTTAGGGTTGTAAAACTCTTTC
TGAGGAATATTGGACAATGGAGGAAACTCTGATCCAGCCATGCCGCGTGCAGGATGAATGCCCTATGGGTTGTAAACTGCTTT
TGAGGAATATTGGTCAATGGACGAAAGTCTGAACCAGCCATGCCGCGTGCAGGATGACGGCCCTACGGGTTGTAAACTGCTTT
TGGGGAATATTGGACAATGGAGGAAACTCTGATCCAGCAATGCCGCGTGTGTGATGAAGGCCTTAGGGTTGTAAAGCACTTTC
TAAGGAATATTGGACAATGGACGAAAGTCTGATCCAGCCATGCCGCGTGCAGGATGACTGCCCTATGGGTTGTAAACTGCTTTT
TGAGGAATATTGCGCAATGGAGGAAACTCTGACGCAGCCATGCCGCGTGCAGGAAGACGGCCCTACGGGTTGTAAACTGCTTT
TGAGGAATTTTCTGCAATGGGCGCAAGCCTGACAGAGCAATACCGCGTGAGGGATGACTGCCTATGGGTTGTAAACCTCTTTTC
TAGGGAATATTGGACAATGGGGGAAACCCTGATCCAGCAATGCCGCGTGAGTGAAGAAGGCCTTCGGGTTGTAAAACTCTTTC
TAGGGAATATTGCGCAATGGGGGAAACCCTGACGCAGCGATGCCGCGTGAGTGAAGAAGGCCTTAGGGTTGTAAAACTCTTTC
TAGGGAATCTTGCACAATGGGGGAAACCCTGATGCAGCGATGCCGCGTGAGTGAAGAAGGCCTTTGGGTTGTAAAGCTCTTTC
TGAGGAATATTGGACAATGGAGGAAACTCTGATCCAGCCATGCCGCGTGCAGGAAGACGGCCCTATGGGTTGTAAACTGCTTT
TTAGGAATATTGTGCAATGGGGGAAACCCTGACACAGCCATGCCGCGTGTGTGAAGAAGGCCCTAGGGTTGTAAAGCACTTTC
TGGGGAATATTGGACAATGGGGGCAACCCTGATCCAGCAATGCCGCGTGTGTGATGAAGGCCCTAGGGTTGTAAAGCACTTTC
GCGCGAAAACTTCACACTGCAGGAAACTGTGATGAGGGAACTCCAAGTGCATGCACTATGTGTATGCTTTTCTTAACTATTAATC
TAGGGAATATTGGACAATGGGGGGAACCCTGATCCAGCAATGCCGCGTGAGTGAAGAAGGCCTTCGGGTTGTAAAACTCTTTC
TGGGGAATTTTCCGCAATGGGCGAAAGCCTGACGGAGCAATCTCGCGTGAGGGATGACGGCCTATGGGTTGTAAACCTCTTTT
TGGGGAATCTTGCACAATGGGGGAAACCCTGATGCAGCGATGCCGCGTGAGTGAAGAAGGCCTTTGGGTTGTAAAACTCTTTC
TAGGGAATATTGGACAATGGGGGAAACCCTGATCCAGCAATGCCGCGTGAGTGAAGAAGGCCTTCGGGTTGTAAAACTCTTTC
TGGGGAATTTTCTGCAATGGGCGCAAGCCTGACAGAGCAATGCCGCGTGAGGGATGACGGCCTTTGGGTTGTAAACCTCTTTT
CTAAGAATATTGCACAATGGACGAAAGTCTGATGCAGCGACACCGCGTGAAGGATGAAGGCCCTAGGGTTGTAAACTTCTTTTA
TGGGGAATCTTGCACAATGGGGGAAACCCTGATGCAGCGATGCCGCGTGAGTGAAGAAGGCCTTTGGGTTGTAAAACTCTTTC
TGGGGAATATTGGACAATGGGGGCAACCCTGATCCAGCAATGCCGCGTGTGTGATGAAGGCCTTAGGGTTGTAAAGCACTTTC
TAGGGAATCTTCCGCAATGGACGAAAGTCTGACGGAGCAACACCGCGTGGTTGATGACGCCCCTTGGGGTGTAAAGACCTTTTC
GCGCGAAAACTTCACACTGCAGGAAACTGTGATGAGGGAACTCCAAGTGCGCGCACTTATGTGTGCGCTTTTCTTGACTATTAAT
TGAGGAATATTGGACAATGGGCGAAAGCCTGATCCAGCAATGTTACGTGAGTGAAGAAAGCTATTTTGGTTGTAAAGCTCTTTC
TGGGGAATATTGCGCAATGGACGAAAGTCTGACGCAGCAACGCCGCGTGAGTGAAGAAGGCCTTCGGGTTGTAAAGCTCTGTT
GCGCGAAAACTTCACACTGCAGGAAACTGTGATGAGGGAACTCCAAGTGACTGCCCAATGGGTAGCCTTTTCTTGAATCTTAATA
TGAGGAATATTGCGCAATGGACGAAAGTCTGACGCAGCCATGCCGCGTGCAGGATGACGGCGCTACGCGTTGTAAACTGCTTT
TAGGGAATATTGGACAATGGGGGAAACCCTGATCCAGCAATGCCGCGTGAGTGAAGAAGGCCTTAGGGTTGTAAAACTCTTTC
TGGGGAATATTGGACAATGGGCGAAAGCCTGATCCAGCCATGCCGCGTGAGTGAAGAAGGCCTTAGGGTTGTAAAACTCTTTC
TGGGGAATCTTGCACAATGGAGGAAACTCTGATGCAGCGATGCCGCGTGAGTGAAGAAGGCCTTTGGGTTGTAAAGCTCTTTC
TAAGGAATATTGGTCAATGGACGAAAGTCTGAACCAGCCATGCCGCGTGCAGGATGACGGCCCTATGGGTTGTAAACTGCTTTT
TAAGGAATATTGGACAATGGAGGAAACTCTGATCCAGCCATGCCGCGTGCAGGATGAATGCCCTATGGGTTGTAAACTGCTTTT
GCGCGAAAACTTCACACTGCAGGAAACTGTGATGAGGGAACTCCAAGTGCGTGCACTTAGTGTGCGCTTTTGTTGACTATTAAT
TAGGGAATATTGGACAATGGGGGAAACCCTGATCCAGCTACGCCGCGTGAGTGATGAAGGCCTTCGGGTTGTAAAGCTCTGTC
TGAGGAATATTGGACAATGGAGGCAACTCTGATCCAGCCATGCCGCGTGCAGGAAGACTGCCCTATGGGTTGTAAACTGCTTTT
TAGGGAATATTGGACAATGGGGGAAACCCTGATCCAGCAATGCCGCGTGAGTGAAGAAGGCCTTCGGGTTGTAAAACTCTTTC
TAGGGAATATTGGACAATGGGGGAAACCCTGATCCAGCAATGCCGCGTGAGTGAAGAAGGCCTTAGGGTTGTAAAACTCTTTC
TGAGGAATATTGGTCAATGGAGGCAACTCTGAACCAGCCATGCCGCGTGCAGGAAGACTGCCCTATGGGTTGTAAACTGCTTTT
TAGGGAATATTGGACAATGGGGGAAACCCTGATCCAGCAATGCCGCGTGAGTGAAGAAGGCCTTAGGGTTGTAAAACTCTTTC
TAAGGAATCTTGCACAATGGAGGAAACTCTGATGCAGCGATGCCGCGTGAGTGAAGAAGGCCTTTGGGTTGTAAAGCTCTTTC
TGAGGAATATTGGACAATGGAGGAAACTCTGATCCAGCCATGCCGCGTGCAGGATGAATGCCCTATGGGTTGTAAACTGCTTT

TGAGGAATATTGTGCAATGGGCGAAAGCCTGACACAGCCATGCCGCGTGTGTGAAGAAGGCCCTAGGGTTGTAAAGCACTTTC
TGAGGAATATTGGACAATGGACGAAAGTCTGATCCAGCCATGCCGCGTGCAGGATGAATGCCTTATGGGTTGTAAACTGCTTTT
TGAGGAATATTGCGCAATGGACGAAAGTCTGACGCAGCGATGCCGCGTGAGTGATGACGCCTTTCGAGGTGTAAAGCTCTGTC
TAGGGAATATTGGACAATGGGGGAAACCCTGATCCAGCAATGCCGCGTGAGTGAAGAAGGCCTTCGGGTTGTAAAACTCTTTC
TAGGGAATATTGGACAATGGGGGAAACCCTGATCCAGCAATGCCGCGTGAGTGAAGAAGGCCTTCGGGTTGTAAAACTCTTTC
TGGGGAATATTGCGCAATGGGCGAAAGCCTGACGCAGCAACGCCGCGTGCGTGAAGAAGGCCCTCGGGTCGTAAAGCGCTAT
TGGGGAATCTTGCACAATGGAGGAAACTCTGATGCAGCGATGCCGCGTGAGTGAAGAAGGCCCTTGGGTTGTAAAGCTCTTTT
TGAGGAATATTGGACAATGGACGAAAGTCTGATCCAGCCATGCCGCGTGCAGGATGACGGCCTTATGGGTTGTAAACTGCTTTT
TGGGGAATCTTGCGCAATGGGCGAAAGCCTGACGCAGCAACGCCGCGTGTGTGAAGAAGGCCCTCGGGTCGTAAAGCACTATA
TAGGGAATATTGGACAATGGGGGAAACCCTGATCCAGCAATGCCGCGTGAGTGAAGAAGGCCTTCGGGTTGTAAAACTCTTTC
TGGGGAATATTGCGCAATGGGGGAAACCCTGACGCAGCCATGCCGCGTGAGTGACGAAGGCCTTAGGGTTGTAAAGCTCTTTC
TAGGGAATATTGGACAATGGGGGAAACCCTGATCCAGCAATGCCGCGTGAGTGAAGAAGGCCTTCGGGTTGTAAAACTCTTTC
TGGGGAATATTGGACAATGGAGGCAACTCTGATCCAGCAATGCCGCGTGTGTGATGAAGGCCCTAGGGTTGTAAAGCACTTTC
TGGGGAATATTGGACAATGGGGGAAACCCTGATCCAGCAATGCCGCGTGAGTGATGAAGGCCCTAGGGTTGTAAAACTCTTTC
TCGCGAAACCTTGACAATGCGGGAAACCGTGATCAGGGGACTCCAAGTGCCAGGAGTAAGATCCTGTGCTTTTCATCTCTCTTCA
TTTCGAATCATTCACAATGGGGGAAACCCTGATGGTGCAACGCCGCGTGAGGGATGAAGGCCTTCGGGTCGTAAACCTCTGTCA
TGAGGAATATTGCGCAATGGAGGAAACTCTGACGCAGCCATGCCGCGTGCAGGAATAAGGCCCTATGGGTTGTAAACTGCTTT
TAGGGAATATTGGACAATGGGGGAAACCCTGATCCAGCAATGCCGCGTGAGTGAAGAAGGCCTTCGGGTTGTAAAACTCTTTC
TCGGGAATATTGGACAATGGAGGAAACTCTGATCCAGCAATGCCGCGTGTGTGATGAAGGCCTTAGGGTTGTAAAGCACTTTC
TGAGGAATATTGGACAATGGAGGAAACTCTGATCCAGCCATGCCGCGTGCAGGATGACTGCCCTACGGGTTGTAAACTGCTTTT
TGAGGAATATTGCGCAATGGACGAAAGTCTGACGCAGCCATGCCGCGTGAAGGATGACGGCGCTACGCGTTGTAAACTTCTTTT
TAAGGAATCTTGCACAATGGGGGAAACCCTGATGCAGCGATGCCGCGTGAGTGAAGAAGGCCCTTGGGTTGTAAAACTCTTTC
TGAGGAATTTTCCGCAATGGGCGAAAGCCTGACGGAGCAATACCGCGTGAAGGATTGACGGCCCACGGGTTGTAAACTTCTTTT
TCGAGAATCATTCACAATGGGGGAAACCCTGATGGTGCGACGCCGCGTGGGGGATGAAGGTCTTCGGATTGTAAACCCCTGTC
TGAGGAATATTGCACAATGGAGGAAACTCTGATGCAGCCATGCCGCGTGCAGGACGACTGCCCTATGGGTTGTAAACTGCTTTT
TGAGGAATATTGGACAATGGGCGGGAGCCTGATCCAGCCATGCCGCGTGCAGGATGACGGCCCTATGGGTTGTAAACTGCTTT
TGAGGAATATTGCGCAATGGAGGAAACTCTGACGCAGCCATGCCGCGTGCAGGAAGACGGCCCTACGGGTTGTAAACTGCTTT
TGAGGAATATTGGACAATGGACGAAAGTCTGATCCAGCTATGCCGCGTGCAGGATGACGGCCCTACGGGTTGTAAACTGCTTTT
TGAGGAATTTTCTGCAATGGGCGCAAGCCTGACAGAGCAATACCGCGTGAGGGATGACTGCCTACGGGTTGTAAACCTCTTTTC
TGGGGAATATTGCGCAATGGAGGAAACTCTGACGCAGCAATGCCGCGTGTGTGAAGAAGGTCTTCGGATTGTAAAGCACTGTT
TGAGGAATATTGCGCAATGGACGAAAGTCTGACGCAGCCATGCCGCGTGAAGGATGACTGCCCTATGGGTTGTAAACTTCTTTT
TGAGGAATCTTCCGCAATGGGCGAAAGCCTGACGGAGCAACACCGCGTGGGGGATGAAGGTCGTAAGATTGTAAACTCCTTTT
TGGGGAATATTGGACAATGGGCGCAAGCCTGATCCAGCAATACCGCGTGAGTGATGACGGCCCTCGGGTTGTAAAGCTCTTTTA
TGGGGAATATTGGACAATGGGCGAAAGCCTGATCCAGTCATGCCGCGTGTGTGATGAAGGCCTTCGGGTTGTAAAGCACTGTC
TGAGGAATATTGGTCAATGGAGGCAACTCTGAACCAGCCATGCCGCGTGCAGGAAGACTGCCCTATGGGTTGTAAACTGCTTTT
TGAGGAATATTGGACAATGGAGGCAACTCTGATCCAGCCATGCCGCGTGCAGGAAGACTGCCCTATGGGTTGTAAACTGCTTTT
TGAGGAATTTTCTACAATGGACGAAAGTCTGATAGAGCAATACCGCGTGAGGGATGACTGCCTACGGGTTGTAAACCTCTTTTC
TGGGGAATTTTCCGCAATGGGCGAAAGCCTGACGGAGCAATACCGCGTGAGGGATTAAGGCCTGTGGGTTGTAAACCTCTTTT
TCGCGAAACCTTGACAATGCGGGAAACCGTGATCAGGGGACTCCAAGTGCCAGGAGTAAGATCCTGTGCTTTTCTTTTCTCTTCA
TGGGGAATATTGGACAATGGGGGAAACCCTGATCCAGCAATGCCGCGTGAGTGAAGAAGGCCTTAGGGTTGTAAAACTCTTTC
TAGGGAATATTGCACAATGGAGGAAACTCTGATGCAGCGATGCCGCGTGAGTGAAGAAGGCCTTTGGGTTGTAAAGCTCTTTC
TAGGGAATATTGCACAATGGAGGAAACTCTGATGCAGCGATGCCGCGTGAGTGAAGAAGGCCCTTGGGTTGTAAAGCTCTTTC
TGAGGAATATTGGACAATGGACGAAAGTCTGATCCAGCCATGCCGCGTGCAGGATGACGGCCCTATGGGTTGTAAACTGCTTTT
TTAGGAATATTGTGCAATGGGGGAAACCCTGACACAGCCATGCCGCGTGTGTGAAGAAGGCCCTAGGGTTGTAAAGCACTTTC
TGAGGAATATTGGACAATGGAGGCAACTCTGATCCAGCCATGCCGCGTGCAGGAAGACTGCCCTATGGGTTGTAAACTGCTTTT
TGGGGAATATTGGACAATGGGGGAAACCCTGATCCAGCCATGCCGCGTGAGTGAAGAAGGCCCTAGGGTTGTAAAGCTCTTTC
TCGAGAATCTTCGGCAATGGACGAAAGTCTGACCGAGCGACGCCGCGTGCGGGATGAAGGCCCTCGGGTTGTAAACCGCTGTC
TGAGGAATATTGGACAATGGGGGCAACCCTGATCCAGCCATGCCGCGTGAGTGAAGAAGGCCTTAGGGTTGTAAAGCTCTTTC
TGGGGAATATTGGACAATGGGGGAAACCCTGATCCAGCAATGCCGCGTGTGTGAAGAAGGCCCTAGGGTTGTAAAGCACTTTC
TGAGGAATTTTCCGCAATGGGCGAAAGCCTGACGGAGCAATACCGCGTGAGGGATGAAGGATTTTGGTCTGTAAACCTCTTTTC

TGGGGAATATTGCGCAATGGACGAAAGTCTGACGCAGCTATGCCGCGTGTGTGATGAAGGCCTTAGGGTTGTAAAGCACTGT
TGGGGAATATTGCACAATGGGGGAAACCCTGATGCAGCTACGCCGCGTGAGTGATGAAGGCCCTCGGGTTGTAAAGCTCTGTT
TGAGGAATTTTCCGCAATGGGCGAAAGCCTGACGGAGCAATACCGCGTGAGGGATGAAGGATTTTGGTCTGTAAACCTCTTTT
TGGGGAATCTTGCACAATGGAGGAAACTCTGATGCAGCGATGCCGCGTGAGTGAAGAAGGCCCTTGGGTTGTAAAGCTCTTT
TCGAGAATCTTCCGCAATGGGCGAAAGCCTGACGGAGCGACGCCGCGTGCGGGATGAAGGCCTTCGGGTTGTAAACCGCTGT
TGGGGAATATTGGACAATGGGCGAAAGCCTGATCCAGCAATGCAGCGTGTGTGAAGAAGGCCTGAGGGTTGTAAAGCACTTT
TGGGGAATATTGGACAATGGGGGCAACCCTGATCCAGCAATGCCGCGTGTGTGATGAAGGCCTTAGGGTTGTAAAGCACTTT
TGAGGAATATTGGACAATGGAGGCAACTCTGATCCAGCCATGCCGCGTGCAGGAAGACTGCCCTATGGGTTGTAAACTGCTTTT
TGGGGAATATTGGACAATGGGCGAAAGCCTGATCCAGCAATGCAGCGTGTGTGAAGAAGGCCTGAGGGTTGTAAAGCACTTT
TGGGGAATATTGGACAATGGGGGAAACCCTGATCCAGCAATGCCGCGTGAGTGACGAAGGCCTTAGGGTTGTAAAGCTCTTT
TGAGGAATATTGCGCAATGGAGGAAACTCTGACGCAGCCATGCCGCGTGCAGGAAGACGGCCCTACGGGTTGTAAACTGCTTT
TAGGGAATATTGCACAATGGAGGAAACTCTGATGCAGCGATGCCGCGTGAGTGAAGAAGGCCTTTGGGTTGTAAAGCTCTTT
TAAGGAATATTGGACAATGGGCGCAAGCCTGATCCAGCCATCCCGCGTGCAGGATGACGGCCCTATGGGTTGTAAACTGCTTTT
TGAGGAATATTGGACAATGGAGGAAACTCTGATCCAGCCATGCCGCGTGCAGGAAGACGGCCCTATGGGTTGTAAACTGCTTT
TGGGGAATATTGGACAATGGGGGAAACCCTGATCCAGCAATGCCGCGTGAGTGACGAAGGCCTTAGGGTTGTAAAACTCTTT
TGAGGAATATTGGACAATGGAGGCAACTCTGATCCAGCCATGCCGCGTGAAGGATGACTGCCCTATGGGTTGTAAACTTCTTTT
TGGGGAATTTTCTGCAATGGGCGAAAGCCTGACAGAGCAATACCGCGTGAGGGATGAAGGCCCGTGGGTCGTAAACCTCTTTT
TAGGGAATATTGGACAATGGGGGAAACCCTGATCCAGCAATGCCGCGTGAGTGAAGAAGGCCCTCGGGTTGTAAAACTCTTT
TGAGGAATATTGCGCAATGGGCGAAAGCCTGACGCAGCAACGCCGCGTGTGTGATGAAGGCCTTAGGGTTGTAAAGCACTGA
TGGGGAATTTTCCGCAATGGGCGAAAGCCTGACGGAGCAACGCCGCGTGAGGGACGAAGGCCTCTGGGCTGTAAACCTCTTTT
TGAGGAATATTGGACAATGGACGAAAGTCTGATCCAGCCATACCGCGTGCAGGAAGACAGCCCTACGGGTCGTAAACTGCTTT
GCGCGAAAACTTCACACTGCAGGAAACTGTGATGAGGGAACTCCAAGTGCGCGCACTTATGTGTGCGCTTTTCTTGACTATTAA
TGAGGAATATTGGTCAATGGAGGCAACTCTGAACCAGCCATGCCGCGTGCAGGAAGACTGCCCTATGGGTTGTAAACTGCTTTT
TCGAGAATCTTCCGCAATGGACGAAAGTCTGACGGAGCGACGCCGCGTGCTGGATGAAGGCCTTCGGGTTGTAAACAGCTGT
TAGGGAATCTTCCACAATGGACGAAAGTCTGATGGAGCAACGCCGCGTGCAGGATGAAGGCCTTCGGGTCGTAAACTGCTTTTA
TAGGGAATATTGCGCAATGGGGGCAACCCTGACGCAGCAATGCCGCGTGAGTGAAGAAGGCCTTCGGGTCGTAAAGCTCTTTT
TGGGGAATTTTCTGCAATGGGCGCAAGCCTGACAGAGCAATGCCGCGTGAGGGATGACGGCCTTTGGGTTGTAAACCTCTTTT
TGAGGAATTTTCTGCAATGGGCGCAAGCCTGACAGAGCAATACCGCGTGAGGGATGACTGCCTACGGGTTGTAAACCTCTTTT
TAAGGAATATTGGGCAATGGGCGCAAGCCTGACCCAGCCATGCCGCGTGCAGGATGACGACCCTATGGGTTGTAAACTGCTTT
TGGGGAATATTGGACAATGGGGGCAACCCTGATCCAGCAATGCCGCGTGTGTGATGAAGGCCTTAGGGTTGTAAAGCACTTT
TGGGGAATATTGGACAATGGGCGAAAGCCTGATCCAGCCATGCCGCGTGAGTGAAGAAGGCCTTAGGGTTGTAAAACTCTTT
TGAGGAATATTGCGCAATGGACGAAAGTCTGACGCAGCGATGCCGCGTGAGTGATGACGCCCTTCGGGGTGTAAAGCTCTGT
TGGGGAATATTGGACAATGGGCGAAAGCCTGATCCAGCAATGCCGCGTGTGTGATGAAGGCCTTCGGGTCGTAAAGCACTTT
TGGGGAATATTGCGCAATGGACGAAAGTCTGACGCAGCGATGCCGCGTGAGTGATGACGCCCTTCGGGGTGTAAAGCTCTGT
TGGGGAATATTGGACAATGGAGGCAACTCTGATCCAGCAATGCCGCGTGTGTGATGAAGGCCCTAGGGTTGTAAAGCACTTT
TGAGGAATATTGGACAATGGACGAAAGTCTGATCCAGCTATGCCGCGTGCAGGATGACAGCCCTACGGGTTGTAAACTGCTTT
TAACGAATATTCCGCAATGCGCGAAAGCGTGACGGGGCAATGCCGCGTGTGGGATGAAGCCCTTCGGGGTGTAAACCACTGT
TGAGGAATTTTCCGCAATGGGCGAAAGCCTGACGGAGCAATACCGCGTGAGGGATGAAGGATTTTGGTCTGTAAACCTCTTTT
TTTCGAATCATTCACAATGGGGGAAACCCTGATGGTGCAACGCCGCGTGAGGGATGAAAGCCTTCGGGTCGTAAACCTCTGTCA
GCGCGAAAACTTGGCAATGCGAGCAATCGTGACCAGGGAACTCCAAGTGCGTAGGGTAAGACCTACGCTTTTCTTGACTCTTCA
TGGGGAATATTGCGCAATGGGCGGAAGCCTGACGCAGCAACGCCGCGTGAGTGAAGAAGGCCTTCGGGTTGTAAAGCTCTGT
TGGGGAATTTTCTGCAATGGGCGAAAGCCTGACAGAGCAATACCGCGTGAGGGATGAAGGCCCGTGGGTCGTAAACCTCTTTT
TGGGGAATCTTGCACAATGGGGGAAACCCTGATGCAGCGATGCCGCGTGAGTGAAGAAGGCCCTTGGGTTGTAAAACTCTTT
TGGGGAATATTGGACAATGGGCGAAAGCCTGATCCAGTCATGCCGCGTGTGTGATGAAGGCCTTAGGGTTGTAAAGCACTGT
TAGGGAATATTGGACAATGGGGGAAACCCTGATCCAGCAATGCCGCGTGAGTGAAGAAGGCCTTAGGGTTGTAAAACTCTTT
TTTCGAATCTTTCACAATGGGCGCAAGCCTGATGGAGCAACGCCGCGTGGGGGATGAAGGTCTTCGGATTGTAAACCCCTGTCA
TGGGGAATATTGGACAATGGGCGAAAGCCTGATCCAGTCATGCCGCGTGTGTGATGAAGGCCTTAGGGTTGTAAAGCACTGT
GCGCGAAAACTTCACACTGCAGGAAACTGTGATGAGGGAACTCCAAGTGACTGCACAATGTGTAGCCTTTTCTTGAATCTTAATA
TGGGGAATATTGGACAATGGGCGAAAGCCTGATCCAGCTACGCCGCGTGAGTGATGAAGGCCCTCGGGTTGTAAAGCTCTGTT
TAGGGAATCTTGCACAATGGGGGGAACCCTGATGCAGCGATGCCGCGTGAGTGAAGAAGGCCTTTGGGTTGTAAAGCTCTTT

TGAGGAATCTTCCGCAATGGGCGAAAGCCTGACGGAGCGACACCGCGTGGAGGACGACGGCCTTTTGGTTGTAAACTCCTTTTC
TGAGGAATATTGGACAATGGACGAAAGTCTGATCCAGCCATGCCGCGTGCAGGATGACGGCCTTATGGGTTGTAAACTGCTTTT
TAAGGAATATTGGTCAATGGAGGCAACTCTGAACCAGCCATGCCGCGTGTATGATGAAGGCCCTTTGGGTTGTAAAGTACTTTT
TGAGGAATATTGGTCAATGGACGAAAGTCTGAACCAGCCATGCCGCGTGAAGGATGACTGCCCTATGGGTTGTAAACTTCTTTT
TGAGGAATATTGGTCAATGGACGAAAGTCTGAACCAGCCATGCCGCGTGAAGGATGACGGCCCTACGGGTTGTAAACTTCTTTT
TGGGGAATATTGGACAATGGGGGCAACCCTGATCCAGCCATGCCGCGTGAGTGAAGAAGGCCCTAGGGTTGTAAAACTCTTTC
TGGGGAATATTGCGCAATGGGCGAAAGCCTGACGCAGCAACGCCGCGTGAGTGAAGAAGGCCTTCGGGTTGTAAAGCTCTGT
TGGGGAATATTGCACAATGGGGGAAACCCTGATGCAGCTACGCCGCGTGTGTGATTGAAGGCCTTCGGGTTGTAAAGCACTGT
TGGGGAATATTGGACAATGGGCGAAAGCCTGATCCAGCGACGCCGCGTGGGTGATGAAGGCCTTCGGGTTGTAAAGCCCTGT
TGGGGAATTTTCCGCAATGGGCGAAAGCCTGACGGAGCAATACCGCGTGAGGGATGACGGCCTGTGGGTTGTAAACCTCTTTT
TGGGGAATATTGGACAATGGGCGAAAGCCTGATCCAGTCATGCCGCGTGTGTGATGAAGGCCTTCGGGTTGTAAAGCACTGTC
TGGGGAATTTTCCGCAATGGGCGAAAGCCTGACGGAGCAACGCCGCGTGAGGGACGAAGGCCTCTGGGCTGTAAACCTCTTTT
TGAGGAATATTGCGCAATGGGCGAAAGCCTGACGCAGCGACGCCACGTGAGCGATGAAGTCTCTAGGGATGTAAAGCTCTGTC
TGGGGAATATTGGACAATGGGGGAAACCCTGATCCAGCAATGCCGCGTGAGTGACGAAGGCCTTAGGGTTGTAAAACTCTTTC
TGGGGAATATTGGACAATGGGCGAAAGCCTGATCCAGCAATGCCGCGTGTGTGATGAAGGCCTTCGGGTCGTAAAGCACTTTC
CCGAGAATCATTCGCAATGGGCGCAAGCCTGACGATGCGACGCCGCGTGAACGAAGAAGGTCTTCGGATTGTAAAGTTCTTTTA
TAGGGAATATTGCACAATGGAGGAAACTCTGATGCAGCGACGCCGCGTGAGTGATGAAGGCCCTCGGGTTGTAAAGCTCTGTT
TCGAGAATCTTCCGCAATGGACGAAAGTCTGACGGAGCGACGCCGCGTGCGGGATGAAGGCCCTCGGGTTGTAAACCGCTGTC
TGGGGAATCTTGCACAATGGGGGAAACCCTGATGCAGCGATGCCGCGTGAGTGAAGAAGGCCCTTGGGTTGTAAAACTCTTTC
TGAGGAATATTGCGCAATGGAGGAAACTCTGACGCAGCCATGCCGCGTGCAGGAAGACGGCCCTACGGGTTGTAAACTGCTTT
TGAGGAATATTGGTCAATGGGCGCAAGCCTGAACCAGCCATGCCGCGTGCAGGATGACTGCCCTATGGGTTGTAAACTGCTTTT
TGGGGAATATTGGACAATGGGCGCAAGCCTGATCCAGCCATGCCGCGTGTGTGATGAAGGCCTTCGGGTTGTAAAGCACTGTC
TGGGGAATCTTGCACAATGGGGGAAACCCTGATGCAGCGATGCCGCGTGAGTGAAGAAGGCCCTTGGGTTGTAAAACTCTTTC
TGAGGAATATTGGACAATGGAGGCAACTCTGATCCAGCCATGCCGCGTGCAGGAAGACTGCCCTATGGGTTGTAAACTGCTTTT
TAAGGAATATTGGTCAATGGAGGAAACTCTGAACCAGCCATGCCGCGTGTGTGATGAAGGCCCTATGGGTTGTAAAGCACTTTT
TGAGGAATATTGCGCAATGGACGAAAGTCTGACGCAGCCATGCCGCGTGCAGGATGACGGCGCTATGCGTTGTAAACTGCTTT
TGAGGAATTTTCCGCAATGGGCGAAAGCCTGACGGAGCAATACCGCGTGAGGGATGAAGGATTTTGGTCTGTAAACCTCTTTTC
TGAGGAATATTGCGCAATGGACGAAAGTCTGACGCAGCCATGCCGCGTGCAGGATGACGGCGCTACGCGTTGTAAACTGCTTT
TGGGGAATATTGGACAATGGGCGAAAGCCTGATCCAGCAATGCCGCGTGTGCGATGAAGGCTTTCGGGTCGTAAAGCACTTTC
TTTCGAATCATTCACAATGGGGGAAACCCTGATGGTGCAACGCCGCGTGAGGGATGAAGGCCTTCGGGTCGTAAACCTCTGTCA
GCGCGAAAACTTCGCAATGCGGGAAACCGTGACGAGGGAACTCCAAGTGTCTATGCAACGCATAGACTGTTTTTCAGCCTAAAT
TGGGGAATATTGGACAATGGGGGAAACCCTGATCCAGCAATGCCGCGTGAGTGACGAAGGCCTTAGGGTTGTAAAACTCTTTC
TAGGGAATATTGCGCAATGGGGGAAACCCTGACGCAGCGATGCCGCGTGAGTGAAGAAGGCCTTAGGGTTGTAAAACTCTTTC
TGGGGAATCTTGCACAATGGAGGAAACTCTGATGCAGCGATGCCGCGTGAGTGAAGAAGGCCTTTGGGTTGTAAAGCTCTTTC
TAGGGAATCTTGCACAATGGGGGAAACCCTGATGCAGCGATGCCGCGTGAGTGAAGAAGGCCTTTGGGTTGTAAAGCTCTTTC
TAAGGAATATTGGTCAATGGAGGCAACTCTGAACCAGCCATGCCGCGTGTATGATGAAGGCCCTTTGGGTTGTAAAGTACTTTT
TCGGGAATATTGGACAATGGGCGCAAGCCTGATCCAGCTACGCCAAGTGAGTGATGAAGGCCTCCGGGTTGTAAAGCTCTGTC
TGAGGAATTTTCCGCAATGGGCGAAAGCCTGACGGAGCAATACCGCGTGAGGGATGACGGCCTTTGGGTTGTAAACCTCTTTT
TGGGGAATCTTGCACAATGGAGGAAACTCTGATGCAGCGATGCCGCGTGAGTGAAGAAGGCCTTTGGGTTGTAAAGCTCTTTC
TGAGGAATATTGGGCAATGGAGGCAACTCTGACCCAGCCATGCCGCGTGAAGGAAGAAGGTCCTACGGATTGTAAACTTCTTTT
TAAAGAATCTTTCGCAATGGGGGAAACCCTGACGAAGCAATACCGCGTGGGTGATGACGGTCGTAAGATTGTAAAGCCCTTTTC
TGAGGAATATTGCACAATGGAGGAAACTCTGATGCAGCCATGCCGCGTGCAGGATGAATGCCCTATGGGTTGTAAACTGCTTTT
TGAGGAATTTTCCGCAATGGGCGAAAGCCTGACGGAGCAATACCGCGTGAGGGATGAAGGATTTTGGTCTGTAAACCTCTTTTC
TGGGGAATATTGGACAATGGGCGAAAGCCTGATCCAGCAATGCCGCGTGTGCGATGAAGGCCTTCGGGTCGTAAAGCACTTTC
TGGGGAATTTTCCGCAATGGGCGAAAGCCTGACGGAGCAATACCGCGTGAGGGATGACGGCCTGTGGGTTGTAAACCTCTTTT
TGGGGAATATTGGACAATGGGGGAAACCCTGATCCAGCAATGCCGCGTGAGTGATGAAGGCCTTAGGGTTGTAAAGCTCTTTC
TGGGGAATCTTAGACAATGGGCGCAAGCCTGATCTAGCCATGCCGCGTGAGTGATGAAGGCCTTAGGGTCGTAAAGCTCTTTCC
TGGGGAATTTTCTGCAATGGGCGAAAGCCTGACAGAGCAATACCGCGTGAAGGATGAAGGCCCGTGGGTCGTAAACTTCTTTT
TCGAGAATCTTCCGCAATGGACGAAAGTCTGACGGAGCGACGCCGCGTGCGGGATGAAGGCCTTCGGGTTGTAAACCGCTGTC
TGGGGAATTTTCTGCAATGGGCGCAAGCCTGACAGAGCAATACCGCGTGAAGGATGAAGGCCCGTGGGTCGTAAACTTCTTTT

TGAGGAATATTGGACAATGGGCGAAAGCCTGATCCAGCAATGTTACGTGAGTGATGAAAACTATTTGGTTGTAAAGCTCTTTC
TGGGGAATATTGCGCAATGGAGGAAACTCTGACGCAGCAACGCCGCGTGTGTGAAGAAGGCCCTCGGGTCGTAAAGCACTAT
TAAGGAATCTTGCACAATGGGGGAAACCCTGATGCAGCGATGCCGCGTGAGTGAAGAAGGCCTTTGGGTTGTAAAACTCTTTC
TGGGGAATATTGGACAATGGGCGAAAGCCTGATCCAGCAATGCCGCGTGAGTGATGAAGGCCCTAGGGTTGTAAAGCTCTTT
TGGGGAATATTGGGCAATGGGCGAAAGCCTGACCCAGCTACGCCGCGTGAGTGATGAAGGCCTTAGGGTTGTAAAGCTCTGT
TGGGGAATCTTAGACAATGGGGGCAACCCTGATCTAGCCATGCCGCGTGAGTGATGAAGGCCCTAGGGTCGTAAAGCTCTTTC
TGGGGAATATTGGACAATGGGCGAAAGCCTGATCCAGCAATGCCGCGTGTGTGATGAAGGCCTTCGGGTCGTAAAGCACTTT
TCGAGAATCTTCCGCAATGGACGAAAGTCTGACGGAGCAATGCCGCGTGGTGGATGAAGGCCTTCGGGTTGTAAACACCTGTC
TCGAGAATCATTCACAATGGGCGAAAGCCTGATGGTGCGACGCCGCGTGGGGGATGAATGGCTTCGGTCAGTAAACCCCTGTC
TGAGGAATATTGCGCAATGGACGAAAGTCTGACGCAGCCATGCCGCGTGAAGGATGACGGCGCTACGCGTTGTAAACTTCTTT
TGGGGAATTTTCCGCAATGGGCGAAAGCCTGACGGAGCAACGCCGCGTGAGGGACGAAGGCCTCTGGGCTGTAAACCTCTTT
TGGGGAATTTTCCGCAATGGGCGAAAGCCTGACGGAGCAATACCGCGTGAGGGATGACGGCCTGTGGGTTGTAAACCTCTTT
TGAGGAATATTGGTCAATGGGCGCAAGCCTGAACCAGCCATGCCGCGTGCAGGATGACTGCCCTATGGGTTGTAAACTGCTTT
TGAGGAATATTGTGCAATGGGCGAAAGCCTGACACAGCCATGCCGCGTGTGTGAAGAAGGCCCTAGGGTTGTAAAGCACTTT
CTAGGAATATTGGACAATGGGGGAAACCCTGATCCAGCGACGCCGCGTGTGTGATGAAGGTCTTCGGATTGTAAAGCACTTTC
TGAGGAATTTTCCGCAATGGGCGAAAGCCTGACGGAGCAATACCGCGTGAGGGATGACGGCCTTTGGGTTGTAAACCTCTTTT
TAAGGAATATTGGTCAATGGACGCAAGTCTGAACCAGCCATGCCGCGTGCAGGATGACGGCCCTATGGGTTGTAAACTGCTTT
TGGGGAATATTGGACAATGGGCGAAAGCCTGATCCAGCAATGCCGCGTGTGTGATGAAGGCCTTCGGGTCGTAAAGCACTTT
TGAGGAATATTGCGCAATGGGCGAAAGCCTGACGCAGCGACACCACGTGAGTGATGACGCCTTTCGGGGTGTAAAGCTCTGT
TCGAGAATCATTCACAATGGGCGCAAGCCTGATGGTGCGACGCCGCGTGGGGGATGAAGGGCTTCGGTCTGTAAACCCCTGT
TGGGGAATCTTGCACAATGGGGGAAACCCTGATGCAGCGATGCCGCGTGAGTGAAGAAGGCCTTTGGGTTGTAAAACTCTTT
TAGGGAATATTGGACAATGGGGGAAACCCTGATCCAGCAATGCCGCGTGAGTGAAGAAGGCCTTAGGGTTGTAAAACTCTTT
TCGAGAATCTTCGGCAATGGACGAAAGTCTGACCGAGCGATGCCGCGTGCGGGATGAAGGCCTTCGGGTTGTAAACCGCTGT
TGGGGAATATTGGACAATGGGGGCAACCCTGATCCAGCAATGCCGCGTGTGTGATGAAGGCCTTAGGGTTGTAAAGCACTTT
TGAGGAATATTGCGCAATGGACGAAAGTCTGACGCAGCGATGCCGCGTGAGTGATGACGCCTTTCGAGGTGTAAAGCTCTGT
TAGGGAATATTGGTCAATGGGGGGAACCCTGAACCAGCCATGCCGCGTGAAGGAAGAAGGCTTTCTGAGTTGTAAACTTCTTT
TGGGGAATATTGCGCAATGGAGGAAACTCTGACGCAGCAACGCCGCGTGTGTGAAGAAGGTTTTCGGATCGTAAAGCACTGT
TGGGGAATCTTGCACAATGGAGGAAACTCTGATGCAGCGATGCCGCGTGAGTGAAGAAGGCCCTTGGGTTGTAAAGCTCTTT
TAAGGAATCTTGCACAATGGGGGAAACCCTGATGCAGCGATGCCGCGTGAGTGAAGAAGGCCCTTGGGTTGTAAAACTCTTTC
TAAGGAATATTGGGCAATGGGCGAAAGCCTGACCCAGCCATGCCGCGTGCAGGATGACGACCCTATGGGTTGTAAACTGCTTT
TGGGGAATATTGGACAATGGGGGAAACCCTGATCCAGCAATGCCGCGTGAGTGATGAAGGCCCTAGGGTTGTAAAACTCTTT
TGAGGAATATTGCGCAATGGACGAAAGTCTGACGCAGCCATGCCGCGTGAAGGATGAATGCCCTAGTGGTTGTAAACTTCTTT
TAAGGAATCTTGCACAATGGAGGAAACTCTGATGCAGCGATGCCGCGTGAGTGAAGAAGGCCTTTGGGTTGTAAAACTCTTTC
TGGGGAATTTTCCGCAATGGGCGAAAGCCTGACGGAGCAACGCCGCGTGAGGGACGAAGGCTTTTGGGCTGTAAACCTCTTT
TGAGGAATATTGGACAATGGAGGAAACTCTGATCCAGCCATGCCGCGTGCAGGAAGACGGCCCTATGGGTTGTAAACTGCTTT
TGGGGAATATTGCGCAATGGGCGGAAGCCTGACGCAGCAACGCCGCGTGAGTGAAGAAGGTCTTCGGATTGTAAAGCTCTGT
TGGGGAATATTGGACAATGGGGGCAACCCTGATCCAGCGATGCCGCGTGAGTGATGAAGGCCCTAGGGTTGTAAAGCTCTTT
TGAGGAATATTGGTCAATGGACGAAAGTCTGAACCAGCCATGCCGCGTGAAGGATGACGGCCCTACGGGTTGTAAACTTCTTT
TGGGGAATATTGGACAATGGGCGAAAGCCTGATCCAGCAATGCCGCGTGTGCGATGAAGGCCTTCGGGTCGTAAAGCACTTT
TGGGGAATATTGCGCAATGGACGAAAGTCTGACGCAGCCACGCCGCGTGAGTGATGAAGGCCTTAGGGTTGTAAAGCTCTGT
TGGGGAATTTTCCGCAATGGGCGAAAGCCTGACGGAGCAACGCCGCGTGAGGGACGAAGGCCTCTGGGCTGTAAACCTCTTT
TGGGGAATATTGGACAATGGGGGAAACCCTGATCCAGCAATGCCGCGTGAGTGATGAAGGCCTTAGGGTTGTAAAGCTCTTT
TGAGGAATATTGCACAATGGAGGAAACTCTGATGCAGCCATGCCGCGTGCAGGATGAATGCCCTATGGGTTGTAAACTGCTTT
TGAGGAATATTGGACAATGGACGCAAGTCTGATCCAGCCATGCCGCGTGCAGGAAGAATGCCCTATGGGTTGTAAACTGCTTT
TAGGGAATATTGGACAATGGGGGAAACCCTGATCCAGCAATGCCGCGTGAGTGAAGAAGGCCCTCGGGTTGTAAAACTCTTTC
TGAGGGATATTGGACAATGGAGGAAACTCTGATCCAGCCATGCCGCGTGCAGGATGAATGCCCTACGGGTTGTAAACTGCTTT
TGGGGAATATTGGACAATGGGCGAAAGCCTGATCCAGCTACGCCGCGTGTGTGACGAAGGCCTTAGGGTTGTAAAGCACTGT
TGGGGAATATTGGACAATGGGCGAAAGCCTGATCCAGCCATGCCGCGTGAGTGAAGAAGGCCTTAGGGTTGTAAAACTCTTT
TGGGGAATATTGCACAATGGGCGAAAGCCTGATGCAGCAATACCGCGTGTGTGAAGAAGGCCTGCGGGTTGTAAAGCACTTT
TGGGGAATATTGGACAATGGGGGCAACCCTGATCCAGCAATGCCGCGTGTGTGATGAAGGCCTTAGGGTTGTAAAGCACTTT

TAGGGAATATTGGACAATGGGGGAAACCCTGATCCAGCAATGCCGCGTGAGTGAAGAAGGCCTTCGGGTTGTAAAACTCTTTC
TGAGGAATTTTCCGCAATGGGCGAAAGCCTGACGGAGCAATACCGCGTGAGGGATGAAGGATTTTGGTCTGTAAACCTCTTTT
TCGAGAATCTTCCGCAATGGGCGAAAGCCTGACGGAGCGACACCGCGTGTGGGATGAAGGCCCTCGGGTTGTAAACCACTGTC
TAAGGAATATTGGGCAATGGGCGCAAGCCTGACCCAGCCATGCCGCGTGCAGGATGACGACCCTATGGGTTGTAAACTGCTTT
TCGGGAATATTGGACAATGGGGGAAACCCTGATCCAGCAATGCCGCGTGTGTGATGAAGGCCTTAGGGTTGTAAAACACTTTA
TGAGGAATATTGGACAATGGACGAAAGTCTGATCCAGCTATGCCGCGTGCAGGATGACAGCCCTACGGGTTGTAAACTGCTTT
TGAGGAATTTTCCGCAATGGGCGAAAGCCTGACGGAGCAATACCGCGTGAGGGATGAAGGATTTTGGTCTGTAAACCTCTTTT
TGAGGAATATTGCGCAATGGACGAAAGTCTGACGCAGCCATGCCGCGTGCAGGATGACGGCGCTACGCGTTGTAAACTGCTTT
TGGGGAATTTTCCGCAATGGGCGAAAGCCTGACGGAGCAACGCCGCGTGAGGGACGAAGGCCTCTGGGCTGTAAACCTCTTTT
TAGGGAATATTGCGCAATGGGGGAAACCCTGACGCAGCAACGCCGCGTGAGTGACGAAGGCTCTAGGGTCGTAAAGCTCTGT
TCGAGAATCTTCCGCAATGGGCGAAAGCCTGACGGAGCGACGCCGCGTGCGGGATGAAGGCCTTCGGGTTGTAAACCGCTGT
TGGGGAATATTGGACAATGGGGGCAACCCTGATCCAGCAATGCCGCGTGTGTGATGAAGGCCTTAGGGTTGTAAAGCACTTT
TGGGGAATATTGCGCAATGGGGGAAACCCTGACGCAGCCATGCCGCGTGAGTGACGAAGGCCTTAGGGTTGTAAAGCTCTTT
TGAGGAATATTGCGCAATGGAGGAAACTCTGACGCAGCCATGCCGCGTGAGTGAAGAAGGCCTTAGGGTTGTAAAGCTCTTT
TGAGGAATATTGGACAATGGGCGAAAGCCTGATCCAGCAATGTTACGTGAGTGATGAAAGCTATTTTGGTTGTAAAGCTCTTT
TGGGGAATATTGGACAATGGGCGAAAGCCTGATCCAGCAATGCCGCGTGAGTGATGAAGGCCTTAGGGTTGTAAAGCTCTTT
TGGGGAATATTGCGCAATGGACGAAAGTCTGACGCAGCGATGCCGCGTGAGTGATGACGCCTTTCGAGGTGTAAAGCTCTGT
TGAGGAATATTGCGCAATGGAGGAAACTCTGACGCAGCCATGCCGCGTGCAGGAAGACGGCCCTATGGGTTGTAAACTGCTTT
TCGAGAATCATTCACAATGGGCGCAAGCCTGATGGTGCGACGCCGCGTGGGGGATGAAGGGCTTCGGTCTGTAAACCCCTGTC
TGGGGAATCTTGCACAATGGAGGAAACTCTGATGCAGCGATGCCGCGTGAGTGAAGAAGGCCTTTGGGTTGTAAAGCTCTTT
TGGGGAATTTTCTGCAATGGGCGAAAGCCTGACAGAGCAATACCGCGTGAGGGATGAAGGCCTGTGGGTCGTAAACCTCTTT
TGGGGAATTTTCTGCAATGGGCGCAAGCCTGACAGAGCAATACCGCGTGAGGGATGAAGGCCTGTGGGTCGTAAACCTCTTT
TGGGGAATATTGGACAATGGGCGAAAGCCTGATCCAGCAATACCACGTGAGTGATGAAGGCCTTAGGGTTGTAAAACTCTTTC
TGGGGAATATTGCGCAATGGGGGAAACCCTGACGCAGCCATGCCGCGTGAGTGAGGAAGGCCTTAGGGTTGTAAAGCTCTTT
TGGGGAATATTGGACAATGGGCGCAAGCCTGATCCAGCCATGCCGCGTGAGTGATGAAGGCCTTAGGGTTGTAAAGCTCTTTC
TGAGGAATATTGCGCAATGGGCGAAAGCCTGACGCAGCGACGCCACGTGAGCGATGAAGTCTTTAGGGATGTAAAGCTCTGT
TGGGGAATATTGCGCAATGGACGAAAGTCTGACGCAGCGATGCCGCGTGAGTGACGACGCCTTTCGGGGTGTAAAACTCTGT
TGGGGAATTTTCTGCAATGGGCGCAAGCCTGACAGAGCAATGCCGCGTGAGGGATGACGGCCTTTGGGTTGTAAACCTCTTTT
TGAGGAATTTTCCGCAATGGGCGAAAGCCTGACGGAGCAATACCGCGTGGGGGATGAAGGATTTTGGTCTGTAAACCTCTTTT
TGGGGAATATTGCGCAATGGGCGAAAGCCTGACGCAGCAACGCCGCGTGTGTGAAGAAGGCCTTCGGGTTGTAAAGCACTGT
TGAGGAATATTGGTCAATGGACGAAAGTCTGAACCAGCCATGCCGCGTGAAGGATGACGGCCCTACGGGTTGTAAACTTCTTT
TGGGGAATTTTCTGCAATGGGCGAAAGCCTGACAGAGCAATACCGCGTGAAGGATGAAGGCCCGTGGGTCGTAAACTTCTTTT
TGAGGAATATTGCACAATGGGGGAAACCCTGATGCAGCAACGCCGCGTGGAGGATGAAGGTTTTTGGATTGTAAACTCCTTTT
TCGAGAATCATTCACAATGGGCGCAAGCCTGATGGTGCGACGCCGCGTGGGGGATGAAGGGCTTCGGTCTGTAAACCCCTGTC
TGGGGAATATTGGACAATGGGCGGAAGCCTGATCCAGCCACGCCGCGTGAGTGATGAAGGCCTTAGGGTTGTAAAGCTCTGT
TGAGGAATATTGGACAATGGGGGCAACCCTGATCCAGCCATGCCGCGTGAGTGAAGAAGGCCTTAGGGTTGTAAAGCTCTTTC
TGGGGAATATTGGACAATGGGCGAAAGCCTGATCCAGCTACGCCGCGTGTGTGATGAAGGCCTTAGGGTTGTAAAGCACTGT
TGGGGAATATTGGACAATGGGCGAAAGCCTGATCCAGCAATGCCGCGTGTGCGATGAAGGCCTTCGGGTCGTAAAGCACTTT
TGGGGAATTTTCTGCAATGGGCGAAAGCCTGACAGAGCGATACCGCGTGAGGGATGACGGCCTGTGGGTTGTAAACCTCTTTT
TGAGGAATTTTCCGCAATGGGCGAAAGCCTGACGGAGCAATACCGCGTGGGGGATGAAGGATTTTGGTCTGTAAACCTCTTTT
TAGGGAATCTTGCGCAATGGGCGAAAGCCTGACGCAGCAATGCCGCGTGAGTGAAGAAGGCCTTCGGGTCGTAAAGCTCTTT
TGGGGAATTTTCTGCAATGGGCGAAAGCCTGACAGAGCAATACCGCGTGAGGGATGAAGGCCTGTGGGTCGTAAACCTCTTTT
TAAGGAATATTGGACAATGGAGGCAACTCTGATCCAGCCATGCCGCGTGAAGGAAGACAGCCTTATGGGTTGTAAACTTCTTTT
TTTCGAATCATTCACAATGGGGGAAACCCTGATGGTGCAACGCCGCGTGAGGGATGAAAGCCTTCGGGTCGTAAACCTCTGTC
TAAGGAATCTTGCACAATGGGGGAAACCCTGATGCAGCGATGCCGCGTGAGTGAAGAAGGCCTTTGGGTTGTAAAACTCTTTC
TGAGGAATTTTCCGCAATGGGCGAAAGCCTGACGGAGCAATACCGCGTGAGGGATGAAGGATTTTGGTCTGTAAACCTCTTTT
TAAGGAATCTTGCACAATGGGGGAAACCCTGATGCAGCGATGCCGCGTGAGTGAAGAAGGCCTTTGGGTTGTAAAACTCTTTC
TAGGGAATATTGGACAATGGGGGAAACCCTGATCCAGCAATGCCGCGTGAGTGAAGAAGGCCCTCGGGTTGTAAAACTCTTTC
TAAGGAATATTGCACAATGGAGGAAACTCTGATGCAGCCATGCCGCGTGCAGGAAGAATGCCCTATGGGTTGTAAACTGCTTT
TAGGGAATTTTGCGCAATGGAGGAAACTCTGACGCAGCAATACCGTGTGCGGGAAGACGCTTCGTGGAGTGTAAACCGCTGT

TCTTTTCTCAAGGAGGAAGAACTGACGTTACTTGAGGAATAAGCATCGGCTAACTCTGTGCCAGCAGCCGCGGTAATACAGAGG
TAGGGAATATTGCACAATGGAGGAAACTCTGATGCAGCGATGCCGCGTGAGTGAAGAAGGCCCTTGGGTTGTAAAGCTCTTTC
TGAGGAATATTGCGCAATGGAGGAAACTCTGACGCAGCCATGCCGCGTGCAGGAAGACGGCCCTACGGGTTGTAAACTGCTTT
TGGGGAATATTGCACAATGGAGGAAACTCTGATGCAGCAATGTCGCGTGAGTGAAGAAGGCCCTTGGGTCGTAAAGCTCTTTT
TCGAGAATCTTCCGCAATGGACGAAAGTCTGACGGAGCGACGCCGCGTGCGGGATGAAGGCCTTCGGGTTGTAAACCGCTGTC
TGAGGAATTTTCCGCAATGGGCGAAAGCCTGACGGAGCAATACCGCGTGAGGGATGAAGGATTTTGGTCTGTAAACCTCTTTTG
TGAGGAATTTTCCGCAATGGGCGAAAGCCTGACGGAGCAATACCGCGTGGGGGATGAAGTATTTTGGTATGTAAACCTCTTTTG
TTTCGAATCATTCACAATGGGGGAAACCCTGATGGTGCAACGCCGCGTGAGGGATGAAGGCCTTCGGGTCGTAAACCTCTGTCA
TTTCGAATCATTCACAATGGGGGAAACCCTGATGGTGCAACGCCGCGTGAGGGATGAAGGCCTTCGGGTCGTAAACCTCTGTCA
TGAGGAATATTGGACAATGGAGGAAACTCTGATCCAGCCATGCCGCGTGCAGGAAGACTGCCCTATGGGTTGTAAACTGCTTTT
TCGAGAATCTTTGTCAATGGACGAAAGTCTGAACAAGCGACGCCGCGTGCGGGATGAAGGCCCTCGGGTTGTAAACCGCTGTC
TGGGGAATATTGGACAATGGGCGCGAGCCTGATCCAGCAATACCGCGTGTGTGAAGAAGGCCTTAGGGTTGTAAAGCACTTTC
TGAGGAATATTGGTCAATGGACGAAAGTCTGAACCAGCCATGCCGCGTGAAGGATGACTGCCCTATGGGTTGTAAACTTCTTTT
TGAGGAATATTGGTCAATGGAGGCAACTCTGAACCAGCCATGCCGCGTGCAGGAAGACTGCCCTATGGGTTGTAAACTGCTTTT
TAAGGAATCTTGCACAATGGGGGAAACCCTGATGCAGCGATGCCGCGTGAGTGAAGAAGGCCCTTGGGTTGTAAAACTCTTTC
TGGGGAATTTTCCGCAATGGGCGAAAGCCTGACGGAGCAATCTCGCGTGAGGGATGACTGCCCTACGTGGTTGTAAACCTCTTT
TGGGGAATATTGGACAATGGGCGAAAGCCTGATCCAGCAATGCCGCGTGTGCGATGAAGGCCTTCGGGTCGTAAAGCACTTTC
TGAGGAATATTGCGCAATGGGCGAAAGCCTGACGCAGCGACGCCACGTGAGCGATGAAGTCTTTCGGGATGTAAAGCTCTGTC
TGGGGAATATTGGACAATGGGGGCAACCCTGATCCAGCCATGCCGCGTGAGTGAAGAAGGCCCTAGGGTTGTAAAACTCTTTC
TGAGGAATATTGCGCAATGGACGAAAGTCTGACGCAGCCATGCCGCGTGCAGGATGACGGCGCTACGCGTTGTAAACTGCTTT
TCGGGAATATTGGACAATGGGGGAAACCCTGATCCAGCCACGCCAAGTGAGTGATGAAGGCCTTAGGGTTGTAAAACTCTGTC
TGAGGAATATTGCACAATGGGGGAAACCCTGATGCAGCAACGCCGCGTGGAGGATGAAGGTTTTTGGATTGTAAACTCCTTTT
TGAGGAATCTTGGACAATGGGCGAAAGCCTGATCCAGCAATATTACGTGAGTGATGACAGCTATTTGGTTGTAAAGCTCTTTCA
TGAGGAATATTGGACAATGGAGGCAACTCTGATCCAGCCATGCCGCGTGCAGGAAGACGGCCCTATGGGTTGTAAACTGCTTTT
TGAGGAATATTGCGCAATGGGGGCAACCCTGACGCAGCAATACCGCGTGAGCGATGACGTCTCTAGGGATGTAAAGCTCTGTC
TTGGGAATATTGCGCAATGGGGGCAACCCTGACGCAGCGACGCCGCGTGAGCGAAGAAGGCCTTCGGGTTGTAAAGCTCTGTC
TGGGGAATATTGCGCAATGGGGGAAACCCTGACGCAGCAACGCCGCGTGTGTGAAGAAGGCCCTCGGGTCGTAAAGCACTGT
TCGAGAATCTTCGGCAATGGGCGCAAGCCTGACCGAGCGACGCCGCGTGCGGGATGAAGGCCCTCGGGTTGTAAACCGCTGTC
TGGGGAATTTTCTGCAATGGGCGAAAGCCTGACAGAGCAATACCGCGTGAGGGATGAAGGCCCGTGGGTCGTAAACCTCTTTT
TGAGGAATATTGGTCAATGGACGAAAGTCTGAACCAGCCATGCCGCGTGCAGGATGACTGCCCTATGGGTTGTAAACTGCTTTT
TGGGGAATATTGGACAATGGGCGAAAGCCTGATCCAGCAATGCCGCGTGTGCGATGAAGGCTTTCGGGTCGTAAAGCACTTTC
TGAGGAATTTTCCGCAATGGGCGAAAGCCTGACGGAGCAATACCGCGTGAGGGATGACGGCCTTTGGGTTGTAAACCTCTTTT
TGAGGAATTTTCCGCAATGGGCGAAAGCCTGACGGAGCAATACCGCGTGGAGGATGAAAGCCTATGGGTTGTAAACTCCTTTT
GCGCGAAAACTTCGCAATGCGGGAAACCGTGACGAGGGAACTCCAAGTGTCTATGCAATGCATAGACTGTTTTTCAGCCTAAAT
TGAGGAATATTGGGCAATGGACGCAAGTCTGACCCAGCGATGCCGCGTGAAGGATGAAGGCCTTAGGGTTGTAAACTTCTGTC
TGAGGAATTTTCCGCAATGGGCGAAAGCCTGACGGAGCAATACCGCGTGAGGGATGAAGGATTTTGGTCTGTAAACCTCTTTTG
TGGGGAATATTGCGCAATGGAGGAAACTCTGACGCAGCAACGCCGCGTGTGTGAAGAAGGCCCTCGGGTCGTAAAGCACTATA
TCGAGAATCTTCCGCAATGGGCGAAAGCCTGACGGAGCGACGCCGCGTGGTGGATGAAGTCCTTAGGGACGTAAACACCTTTT
TGAAGAATTTTGGACAATGAACGAAAGTTTGATCCAGCAATGTTACGTGAGTGAAAGAAGGCCATTTTGGTCGTAAAGCTCTTT
TGGGGAATTTTCCGCAATGGGCGAAAGCCTGACGGAGCAACGCCGCGTGAGGGATGAAGGCCTCTGGGCTGTAAACCTCTTTT
TGAGGAATATTGGACAATGGGCGAAAGCCTGATCCAGCAATGTTACGTGAGTGAAGACGGCTATTTTGGTTGTAAAGCTCTTTG
TGAGGAATTTTCCGCAATGGGCGAAAGCCTGACGGAGCAATACCGCGTGAGGGATGACGGATTTTGGTCTGTAAACCTCTTTTG
TAGGGAATATTGCGCAATGGGCGAAAGCCTGACGCAGCAACGCCGCGTGGAGGACGAAGCCCCTAGGGGCGTAAACTCCTGTC
TAGGGAATATTGCGCAATGGGGGAAACCCTGACGCAGCGACGCCGCGTGAGTGATGAAGGCCTTAGGGTTGTAAAGCTCTGT
TCGAGAATCTTCTGCAATGAGCGCAAGCTTGACAGAGCGACGCCGCGTGTGGGATTGAAGGCCTAAGGGTTGTAAACCACTGT
TGAGGAATATTGGGCAATGGGCGCAAGCCTGACCCAGCCATGCCGCGTGCAGGAAGAATGCCCTATGGGTTGTAAACTGCTTT
TGGGGAATCTTGGACAATGGGCGCAAGCCTGATCCAGCCATGCCGCGTGAGTGATGAAGGCCCTAGGGTCGTAAAGCTCTTTC
TGGGGAATATTGGACAATGGGGGAAACCCTGATCCAGCAATGCCGCGTGAGTGAAGAAGGCCCTAGGGTTGTAAAGCTCTTTC
TGAGGAATATTGCGCAATGGGCGAAAGCCTGACGCAGCGACGCCACGTGAGCGATGAAGTCTTTAGGGATGTAAAGCTCTGTC
TGGGGAATATTGGACAATGGGCGAAAGCCTGATCCAGCTACGCCGCGTGAGTGATGAAGGCCCTCGGGTTGTAAAGCTCTGTT

GCGCGAAAACTTGGCAATGCGAGAAATCGTGACCAGGGAACTCCAAGTGGCTGGGGTAAGACCCAGTCTTTTCTTGACTCTTCA
TGGGGAATTTTCTGCAATGGGCGAAAGCCTGACAGAGCAATACCGCGTGAGGGATGAAGGCCCGTGGGTCGTAAACCTCTTTT
TGAGGAATATTGGACAATGGGCGAAAGCCTGATCCAGCAATGTTACGTGAGTGATGAAAGCTATTTTGGTTGTAAAGCTCTTT
TGAGGAATATTGCGCAATGGAGGAAACTCTGACGCAGCCATGCCGCGTGCAGGAAGACGGCCTTATGGGTTGTAAACTGCTTT
TGAGGAATATTGCGCAATGGACGAAAGTCTGACGCAGCCATGCCGCGTGAAGGATGACGGCGCTACGCGTTGTAAACTTCTTT
TCGGGAATATTGGACAATGGGCGCAAGCCTGATCCAGCTACGCCAAGTGAGTGATGAAGGCCTCCGGGTTGTAAAGCTCTGTC
TGAGGAATATTGGTCAATGGAGGCAACTCTGAACCAGCCATGCCGCGTGCAGGAAGACTGCCCTATGGGTTGTAAACTGCTTTT
TGGGGAATATTGCGCAATGGGCGAAAGCCTGACGCAGCAACGCCGCGTGCGGGATGAAGGCCTTCGGGTTGTAAACCGCTTT
TGGGGAATATTGCGCAATGGGGGAAACCCTGACGCAGCCATGCCGCGTGAGTGACGAAGGCCTTAGGGTTGTAAAGCTCTTT
TAGGGAATATTGCGCAATGGGCGAAAGCCTGACGCAGCAATACCGTGTGCGGGAAGACGCTCCGTGGAGTGTAAACCGCTGT
TAGGGAATCTTGCGCAATGGGCGAAAGCCTGACGCAGCAACGCCGCGTGTGTGATGAAGGCTCTAGGGTCGTAAAGCACTGT
TGGGGAATATTGGACAATGGGGGCAACCCTGATCCAGCAATGCCGCGTGTGTGAAGAAGGCCCTAGGGTTGTAAAGCACTTT
TGGGGAATTTTCTGCAATGGGCGCAAGCCTGACAGAGCAATACCGCGTGAAGGATGAAGGCCCGTGGGTCGTAAACTTCTTTT
TGGGGAATTTTCTGCAATGGGCGCAAGCCTGACAGAGCAATACCGCGTGAGGGATGAAGGCCTGTGGGTCGTAAACCTCTTTT
TAGGGAATCTTGCGCAATGGGCGAAAGCCTGACGCAGCAATACCGCGTGACTGATGAAGGCCCTTGGGTTGTAAAAGTCTTTTA
TGAGGAATATTGGACAATGGAGGCAACTCTGATCCAGCCATGCCGCGTGAAGGATGACTGCCCTATGGGTTGTAAACTTCTTTT
TGGGGAATATTGCGCAATGGGCGAAAGCCTGACGCAGCCACGCCGCGTGAGTGATGAAGGCCCTCGGGTCGTAAAGCTCTGT
TGGGGAATCTTGGACAATGGGCGAAAGCCTGATCCAGCCATGCCGCGTGAGTGATGAAGGCCCTAGGGTCGTAAAGCTCTTTC
CAGGGAATCTTCCACAATGGGCGAAAGCCTGATGGAGCGACGCCGCGTGGGGGATGAAGGTTCTCGGATCGTAAACCCCTTTT
TGAGGAATATTGGACAATGGAGGCAACTCTGATCCAGCCATGCCGCGTGCAGGAAGACTGCCCTATGGGTTGTAAACTGCTTTT
TGGGGAATCTTGCGCAATGGACGAAAGTCTGACGCAGCAACGCCGCGTGGGTGATGAAGGCTCTTGGGTCGTAAAGCCCTGT
TGGGGAATATTGGACAATGGGGGCAACCCTGATCCAGCAATGCCGCGTGAGTGAAGAAGGCCTTAGGGTTGTAAAACTCTTTC
TCGAGAATCTTCGGCAATGGGCGAAAGCCTGACCGAGCGACGCCGCGTGCGGGATGACGGCCCTTGGGTTGTAAACCGCTGTC
TGAGGAATTTTCCGCAATGGGCGAAAGCCTGACGGAGCAATACCGCGTGAGGGATGAAGGATTTTGGTCTGTAAACCTCTTTT
TGAGGAATATTGCGCAATGGACGAAAGTCTGACGCAGCCATGCCGCGTGAAGGATGACGGCGCTACGCGTTGTAAACTTCTTT
TGAGGAATATTGGACAATGGAGGCAACTCTGATCCAGCCATGCCGCGTGCAGGAAGACTGCCCTATGGGTTGTAAACTGCTTTT
TCGGGAATATTGGACAATGGGGGCAACCCTGATCCAGCAATGCCGCGTGTGTGATGAAGGCCCTAGGGTTGTAAAGCACTTTC
TGGGGAATATTGGACAATGGGGGCAACCCTGATCCAGCAATGCCGCGTGTGTGATGAAGGCCTTAGGGTTGTAAAACACTTTC
TAAGGAATCTTGCACAATGGAGGAAACTCTGATGCAGCGATGCCGCGTGAGTGAAGAAGGCCTTTGGGTTGTAAAACTCTTTC
TGGGGAATATTGGACAATGGGCGAAAGCCTGATCCAGCTACGCCGCGTGTGTGATGAAGGCCTTAGGGTTGTAAAGCACTGT
TAGGGAATATTGGACAATGGGGGCAACCCTGATCCAGCAATGCAGCGTGAGTGACGAAGGCCTTAGGGTTGTAAAACTCTTTC
TGAGGAATATTGGTCAATGGACGAAAGTCTGAACCAGCCATGCCGCGTGAAGGATGACTGCCCTATGGGTTGTAAACTTCTTTT
TGAGGAATATTGCGCAATGGGCGAAAGCCTGACGCAGCGACGCCACGTGAGCGATGAAGTCTTTCGGGATGTAAAGCTCTGT
TGGGGAATATTGGACAATGGGGGAAACCCTGATCCAGCAATGCCGCGTGAGTGATGAAGGCCCTAGGGTTGTAAAACTCTTTC
TGAGGAATCTTGCGCAATGGGCGAAAGCCTGACGCAGCCACGCCGCGTGCCGGATGAAGGCCCTATGGGTTGTAAACGGCTTT
TGGGGAATATTGCGCAATGGACGAAAGTCTGACGCAGCGATGCCGCGTGAGTGATGACGCCTTTCGAGGTGTAAAGCTCTGT
TGAGGAATATTGGACAATGGAGGAAACTCTGATCCAGCCATGCCGCGTGCAGGAAGACGGCCCTATGGGTTGTAAACTGCTTT
TGAGGAATATTGCGCAATGGGCGAAAGCCTGACGCAGCAACGCCGCGTGAGCGACGAAGCCTTTCGGGGTGTAAAGCTCTGT
TAGGGAATTTTGCGCAATGGGCGAAAGCCTGACGCAGCAATGCCGCGTGAGTGAAGAAGGCCCTCGGGTCGTAAAGCTCTTTA
TAACGAATATTCCGCAATGCGCGAAAGCGTGACGGAGCAATGCCGCGTGTGGGATGAAGCTTTTCGGAGTGTAAACCACTGTC
TGGGGAATTTTCCGCAATGGGCGAAAGCCTGACGGAGCAATACCGCGTGAGGGATGAAGGCTTACTGAGTTGTAAACCTCTTT
TGAGGAATATTGGACAATGGGGGAAACCCTGATCCAGCCATGCCGCGTGAGTGAAGAAGGCCTTAGGGTTGTAAAGCTCTTTC
TGAGGAATATTGGTCAATGGAGGCAACTCTGAACCAGCCATGCCGCGTGCAGGAAGACTGCCCTATGGGTTGTAAACTGCTTTT
TGGGGAATTTTCCGCAATGGGCGAAAGCCTGACGGAGCAATCTCGCGTGAGGGATGACGGCCTATGGGTTGTAAACCTCTTTT
TGAGGAATATTGGTCAATGGAGGCAACTCTGAACCAGCCATGCCGCGTGAAGGAAGAATGTCCTACGGATTGTAAACTTCTTTT
GCGCGAAAACTTGGCAATGCGAGCAATCGTGACCAGGGAACTCCAAGTGCTTGGGGTAAGACCCAAGCTTTTCTTGACTCTTCA
TAGGGAATCTTGCGCAATGGGCGAAAGCCTGACGCAGCAATTCCGCGTGGAGGATGACGGCCTTCGGGTTGTAAACTCCTTTC
TGGGGAATATTGCGCAATGGGCGAAAGCCTGACGCAGCTACGCCGCGTGTGTGACGAAGGCCCTCGGGTCGTAAAGCACTGT
TGGGGAATTTTCCGCAATGGGCGAAAGCCTGACGGAGCAATACCGCGTGAGGGATGAAGGCTTAATGAGTTGTAAACCTCTTT
TTTCGAATCATTCACAATGGGGGAAACCCTGATGGTGCAACGCCGCGTGAGGGATGAAGGCCTTCGGGTCGTAAACCTCTGTCA

TGAGGAATTTTCCGCAATGGGCGAAAGCCTGACGGAGCAATACCGCGTGAGGGATGAAGGATTTTGGTCTGTAAACCTCTTTT
TAAGGAATATTGGACAATGGGCGGAAGCCTGATCCAGCCATCCCGCGTGAAGGATGAAGGCCCTCTGGGTTGTAAACTTCTTT
TGAGGAATATTGGTCAATGGACGAAAGTCTGAACCAGCCATGCCGCGTGAAGGATGACGGCCCTACGGGTTGTAAACTTCTTT
TGGGGAATATTGGACAATGGGGGAAACCCTGATCCAGCAATGCCGCGTGAGTGAAGAAGGCCCTAGGGTTGTAAAACTCTTT
TGAGGAATATTGCGCAATGGACGAAAGTCTGACGCAGCGATGCCGCGTGAGTGATGACGCCCTTCGGGGTGTAAAGCTCTGT
TGAGGAATATTGGTCAATGGGCGCAAGCCTGAACCAGCCATGTCGCGTGCAGGATGACGGCCCTATGGGTTGTAAACTGCTTT
TGGGGAATATTGGACAATGGGCGAAAGCCTGATCCAGCTACGCCGCGTGAGTGATGAAGGCCCTCGGGTTGTAAAGCTCTGT
TGGGGAATATTGGACAATGGGCGAAAGCCTGATCCAGCTACGCCGCGTGTGTGATGAAGGCCTTAGGGTTGTAAAGCACTGT
TGAGGAATATTGGTCAATGGACGAAAGTCTGAACCAGCCATGCCGCGTGCAGGATGACGGCCCTACGGGTTGTAAACTGCTTT
TCGAGAATCTTCCGCAATGGACGAAAGTCTGACGGAGCAATGCCGCGTGGTGGATGAAGGCCCTCGGGTTGTAAACACCTGTC
TGGGGAATTTTGCGCAATGGGCGAAAGCCTGACGCAGCAATGCCGCGTGAGCGATGAAGGCCTGATGGTTGTAAAGCTCTTT
TGGGGAATCTTAGACAATGGGGGAAACCCTGATCTAGCCATGCCGCGTGAGTGACGAAGGCCCTAGGGTCGTAAAGCTCTTTC
TAACGAATCTTCCGCAATGCACGAAAGTGTGACGGGGCAATGCCGCGTGTGGGATGAAGCCCTTCGGGGTGTAAACCACTGTC
TAGGGAATCTTGCGCAATGGGCGAAAGCCTGACGCAGCGACACCGCGTGGAGGATGAAGGTTTTTGGATCGTAAACTCCTGT
TGGGGAATATTGGACAATGGGCGAAAGCCTGATCCAGCAATGCCGCGTGTGTGAAGAAGGCCTGCGGGTTGTAAAGCACTTT
TGGGGAATTTTCCGCAATGGGCGAAAGCCTGACGGAGCAATCTCGCGTGAGGGATGACTGCCCTACGTGGTTGTAAACCTCTT
TAGGGAATATTGCGCAATGGGGGAAACCCTGACGCAGCGACGCCGCGTGAGTGATGAAGGCTCTTGGGTTGTAAAGCTCTGT
TCGAGAATCTTCCGCAATGGGCGAAAGCCTGACGGAGCGACACCGCGTGTAGGATGAAGGCCTTAGGGTTGTAAACTACTGTC
TGAGGAATATTGGACAATGGGGGCAACCCTGATCCAGCCATGCCGCGTGAGTGACGAAGGCCCTAGGGTTGTAAAGCTCTTTC
TAACGAATATTCCGCAATGCGCGAAAGCGTGACGGAGCAATGCCGCGTGCAGGATGAACCCCCTAGGGGTGTAAACTGCTGTC
TAAGGAATCTTGCACAATGGAGGAAACTCTGATGCAGCGATGCCGCGTGAGTGAAGAAGGCCTTTGGGTTGTAAAACTCTTTC
TGGGGAATCTTGCACAATGGGCGAAAGCCTGATGCAGCCATGCCGCGTGAATGATGAAGGCCTTAGGGTTGTAAAATTCTTTC
TCGAGAATCTTCCCCAATGGGCGCAAGCCTGAGGGAGCGACGCCGCGTGCAGGATGAAGGTCTTCGGATTGTAAACTGCTGTC
TGAGGAATATTGCGCAATGGACGAAAGTCTGACGCAGCGATGCCGCGTGAGTGATGACGCCTTTTGGGGTGTAAAGCTCTGT
TCGAGAATCTTCCGCAATGGACGAAAGTCTGACGGAGCAATGCCGCGTGGTGGATGAAGGCCCTCGGGTTGTAAACACCTGTC
TGAGGAATATTGGACAATGGGGGAAACCCTGATCCAGCCATGCCGCGTGAGTGAAGAAGGCCTTAGGGTTGTAAAGCTCTTT
TGAGGAATCTTCCGCAATGGGGGCAACCCTGACGGAGCGACACCGCGTGAAGGATGAAACCATTTTTTGGCGTAAACTTCTGT
TGAGGAATATTGGACAATGGGCGAAAGCCTGATCCAGCAATGTTACGTGAGTGATGAAAGCTATTTTGGTTGTAAAACTCTTT
TCGGGAATATTGGACAATGGGGGAAACCCTGATCCAGCCACGCCAAGTGAGTGATGAAGGCCTTAGGGTTGTAAAACTCTGTC
TGGGGAATATTGCGCAATGGAGGAAACTCTGACGCAGCAACGCCGCGTGTGTGAAGAAGGTTTTCGGATCGTAAAGCACTGT
TCGAGAATCTTCCGCAATGCGCGAAAGCGTGACGGAGCAATGCCGCGTGAGGGATGAAGGCCTTCGGGTTGTAAACCTCTGTC
TGGGGAATATTGCGCAATGGGCGAAAGCCTGACGCAGCCACGCCGCGTGAGCGATGAAGGTTTTCGGATCGTAAAGCTCTGT
TGAGGAATATTGGGCAATGGAGGCAACTCTGACCCAGCCATGCCGCGTGCAGGAAGAAGGTCCTATGGATTGTAAACTGCTTT
TGGGGAATATTGGGCAATGGGCGAAAGCCTGACCCAGCTACGCCGCGTGAGTGATAAGGCCTTCGGGTTGTAAAACTCTGTC
TGAGGAATTTTGGACAATGGGGGAAACCCTGATCCAGCAATACCACGTGAGTGAAGAAAGCCTTCGGGTTGTAAAGCTCTTTT
TGGGGAATCTTGCGCAATGGGCGAAAGCCTGACGCAGCGACGCCGCGTGAGCGATGAAGGCCTTAGGGTTGTAAAGCTCTGT
TGGGGAATATTGGACAATGGGCGCAAGCCTGATCCAGCCATGCCGCGTGAGTGATGAAGGCCTTAGGGTTGTAAAGCTCTTTT
TAGGGAATATTGCACAATGGAGGAAACTCTGATGCAGCGACGCCGCGTGAGTGATGACGGTCTTCGGATTGTAAAGCTCTGTT
TTTCGAATCATTCACAATGGGCGAAAGCCTGATGGTGCAATGCCGCGTGGGGGATGAAGGTCTTCGGATCGTAAACCCCTGTC
TCGAGAATCTTCGGCAATGCGCGAAAGCGTGACCGAGCGACGCCGCGTGCGGGATGAAGGCCTTCGGGTTGTAAACCGCTGT
TAACGAATATTCCGCAATGCGCGAAAGCGTGACGGAGCAATGCCGCGTGCAGGATGAAGCGGTTCTGCCGTGTAAACTGCTGT
TGAGGAATATTGGTCAATGGACGAAAGTCTGAACCAGCCATGCCGCGTGAAGGATGACTGCCCTATGGGTTGTAAACTTCTTTT
TGAGGAATATTGGACAATGGAGGAAACTCTGATCCAGCCATGCCGCGTGCAGGAAGACGGCCCTATGGGTTGTAAACTGCTTT
TGGGGAATTTTCTGCAATGGGCGAAAGCCTGACAGAGCAATACCGCGTGAGGGATGAAGGCCCGTGGGTCGTAAACCTCTTTT
TGGGGAATATTGGACAATGGGCGAAAGCCTGATCCAGCAATGCCGCGTGTGTGAAGAAGGCCTGCGGGTTGTAAAGCACTTT
TGAGGAATATTGGACAATGGGGGCAACCCTGATCCAGCCATGCCGCGTGAGTGAAGAAGGCCTTAGGGTTGTAAAGCTCTTTT
TGAGGAATATTGCGCAATGGACGAAAGTCTGACGCAGCCATGCCGCGTGAAGGATGACTGCCCTATGGGTTGTAAACTTCTTTT
TGGGGAATATTGGACAATGGGCGAAAGCCTGATCCAGCAATGCCACGTGAGTGATGAAGGCCTTCGGGTTGTAAAGCTCTTTT
TGAGGAATATTGGACAATGGGCGAGAGCCTGATCCAGCCATGCCGCGTGCAGGATGACTGCCCTATGGGTTGTAAACTGCTTT
TAACGAATATTCCGCAATGCGCGAAAGCGTGACGGAGCAATGCCGCGTGCAGGATGAAGCCCCTCGGGGTGTAAACTGCTGTC

TAGGGAATATTGGGCAATGGAGGCAACTCTGACCCAGCCATGCCGCGTGCAGGAAGACGGCGTTCTGCGTTGTAAACTGCTTT
TAAGGAATATTGGACAATGGGCGCAAGCCTGATCCAGCTATCCCGCGTGCAGGATGACTGCCCTATGGGTTGTAAACTGCTTTT
TGGGGAATCTTGCGCAATGGGGGCAACCCTGACGCAGCCATGCCGCGTGTGTGATGAAGGCCTTCGGGTTGTAAAGCACTTTC
TGAGGAATATTGCGCAATGGAGGAAACTCTGACGCAGCCATACCGCGTGCAGGATGACAGTCCTACGGATTGTAAACTGCTTTT
TGGGGAATATTGGACAATGGGCGCAAGCCTGATCCAGCCATGCCGCGTGAGTGAAGAAGGCCTTAGGGTTGTAAAACTCTTTC
TGAGGAATATTGGACAATGGAGGAAACTCTGATCCAGCCATGCCGCGTGAGTGAAGAAGGCCTTAGGGTTGTAAAACTCTTTC
TAAGGAATATTGGACAATGGGCGCAAGCCTGATCCAGCCATCCCGCGTGCAGGATGACGGCCCTATGGGTTGTAAACTGCTTTT
TGAGGAATATTGGACAATGGGGGCAACCCTGATCCAGCCATGCCGCGTGAGTGAAGAAGGCCTTAGGGTTGTAAAGCTCTTTT
TAGGGAATATTGGTCAATGGGCGCAAGCCTGAACCAGCCATGCCGCGTGCAGGATGAAAGCCTTAAGGGTTGTAAACTGCTTT
TAGGGAATTTTCCGCAATGGGCGAAAGCCTGACGGAGCAATACCGCGTGAGGGATGACTGCCTATGGGTTGTAAACCTCTTTT
TAAGGAATATTGGTCAATGGAGGCAACTCTGAACCAGCCATGCCGCGTGCAGGAAGAAGGCGTTCTGCGTCGTAAACTGCTTT
TGAGGAATATTGGACAATGGGCGAGAGCCTGATCCAGCCATGCCGCGTGCAGGAAGACTGCCCTATGGGTTGTAAACTGCTTT
GCTTTTATACAGGAAGAAAAATCCCGACGTGTCGGGGCTTGACGGTACTGTAAGAATAAGGATCGGCTAACTCCGTGCCAGCA
TGAGGAATATTGGACAATGGAGGCAACTCTGATCCAGCCATGCCGCGTGCAGGAAGACTGCCCTATGGGTTGTAAACTGCTTTT
TAACGAATCTTCCGCAATGCGCGAAAGCGTGACGGAGCAATGCCGCGTGTGGGATGAAGCGGTTTCGCCGTGTAAACCACTGT
TGGGGAATTTTCTGCAATGGGCGAAAGCCTGACAGAGCGATACCGCGTGAGGGATGACGGCCTGTGGGTTGTAAACCTCTTTT
TAAGGAATATTGCGCAATGGGCGAAAGCCTGACGCAGCCACGCCGCGTGGAGGAAGACACCCCTATGGGGCGTAAACTCCTTT
GCTTTTATACAGGAAGAAAACCTTCGACGTGTCGAAGCTTGACGGTACTGTAAGAATAAGGATCGGCTAACTCCGTGCCAGCAG
CAGGGAATCTTGCGCAATGGGCGAAAGCCTGACGCAGCGACGCCGCGTGGGGGATGAAGGCCTTCGGGTTGTAAACCCCTTT

B.

SequenceID	Reads 4h_1day_+Glu [3]	Reads 4h_1day_-Glu [7]	log2FC	adj.P.Val	Phylum	Class	Order	Notes
ASV.484	122	0	5,930712295	4,52E-08	Proteobacteria	Alphaproteobacteria	Rickettsiales	
ASV.512	0	94	-5,374848273	4,52E-08	Bacteroidetes	Flavobacteriia	Flavobacteriales	
ASV.555	67	0	5,085349791	4,52E-08	Bacteroidetes	Flavobacteriia	Flavobacteriales	
ASV.39	327	0	7,338217892	1,23E-05	Proteobacteria	Alphaproteobacteria		SAR11 clade
ASV.96	206	0	6,676798948	1,57E-05	Proteobacteria	Alphaproteobacteria	Rickettsiales	
ASV.130	160	0	6,316274941	5,75E-05	Proteobacteria	Alphaproteobacteria		SAR11 clade
ASV.378	0	363	-7,298087345	9,51E-05	Cyanobacteria			
ASV.191	0	64	-4,836465714	0,000103954	Proteobacteria	Alphaproteobacteria		SAR11 clade
ASV.177	60	0	4,931100377	0,000136942	Cyanobacteria			chloroplast
ASV.68	265	0	7,037004024	0,0001505	Actinobacteria	Acidimicrobiia	Acidimicrobiales	
ASV.74	340	0	7,394121071	0,00026543	Cyanobacteria			
ASV.144	54	0	4,784342136	0,00136491	Chloroflexi			SAR202 clade
ASV.223	53	0	4,758362548	0,00136491	Proteobacteria	Alphaproteobacteria	Rhodospirillales	
ASV.246	68	0	5,106098389	0,001946528	Cyanobacteria			chloroplast
ASV.299	57	0	4,859586576	0,002054593	Bacteroidetes	Flavobacteriia	Flavobacteriales	
ASV.482	0	62	-4,792289995	0,021249397	Bacteroidetes	Cytophagia	Cytophagales	

SequenceID	Reads 12h_1day_+Glu [11]	Reads 12h_1day_-Glu [15]	log2FC	adj.P.Val	Phylum	Class	Order	Notes
ASV.456	0	159	-6,164779924	4,55E-08	Proteobacteria	Alphaproteobacteria		SAR11 clade
ASV.498	0	109	-5,629276249	4,55E-08	Cyanobacteria			
ASV.549	0	69	-4,986414999	4,55E-08	Proteobacteria	Alphaproteobacteria	Rhodospirillales	
ASV.556	0	65	-4,903056302	4,55E-08	Verrucomicrobia	Opitutae	Puniceicoccales	
ASV.479	0	57	-4,720330093	6,96E-07	Proteobacteria	Alphaproteobacteria	Rhodospirillales	
ASV.378	0	173	-6,284896958	0,000236878	Cyanobacteria			
ASV.248	0	65	-4,903056302	0,004620823	Proteobacteria	Alphaproteobacteria	Rhodospirillales	
ASV.224	0	53	-4,619485085	0,018456266	Cyanobacteria			chloroplast

SequenceID	Reads 24h_1day_+Glu [19]	Reads 24h_1day_-Glu [23]	log2FC	adj.P.Val	Phylum	Class	Order	Notes
ASV.499	0	108	-5,725196826	4,53E-08	Proteobacteria	Alphaproteobacteria	Rickettsiales	
ASV.511	0	94	-5,52895427	4,53E-08	Cyanobacteria	Cyanobacteria		
ASV.516	92	0	5,501203331	4,53E-08	Proteobacteria	Alphaproteobacteria	Rhizobiales	
ASV.479	0	67	-5,052991621	4,67E-07	Proteobacteria	Alphaproteobacteria	Rhodospirillales	
ASV.444	0	73	-5,173150647	5,12E-06	Bacteroidetes	Flavobacteriia	Flavobacteriales	
ASV.410	0	111	-5,763987918	8,53E-06	Proteobacteria	Alphaproteobacteria		SAR11 clade
ASV.415	0	66	-5,031954859	0,000628768	Bacteroidetes	Flavobacteriia	Flavobacteriales	
ASV.179	0	55	-4,777712927	0,000795649	Chloroflexi			SAR202 clade

SequenceID	Reads 4h_2day_+Glu [27]	Reads 4h_2day_+Glu [28]	Reads 4h_2day_-Glu [31]	log2FC	adj.P.Val	Phylum	Class	Order	Notes
ASV.508	0	0	98	-5,927926382	2,86E-08	Bacteroidetes	Cytophagia	Cytophagales	
ASV.587	0	0	53	-5,061117965	2,86E-08	Euryarchaeota	Thermoplasmata	Thermoplasmatales	
ASV.248	54	46	0	4,668052031	0,006101764	Proteobacteria	Alphaproteobacteria	Rhodospirillales	
ASV.141	81	72	0	5,263605705	0,025502732	Actinobacteria	Acidimicrobiia	Acidimicrobiales	

SequenceID	Reads 12h_2day_+Glu [35]	Reads 12h_2day_+Glu [36]	Reads 12h_2day_-Glu [39]	Reads 12h_2day_-Glu [40]	log2FC	adj.P.Val	Phylum	Class	Order	Notes
ASV.177	84	108	0	0	5,727279742	0,000106907	Cyanobacteria			chloroplast
ASV.248	0	0	68	48	-4,89211669	0,003322831	Proteobacteria	Alphaproteobacteria	Rhodospirillales	

Table S2. (A) List of 718 ASVs identified within the 16S rRNA data sets. This spreadsheet shows the ASV ID, total number of reads, scikit-learn annotation and sequence. **(B) List of 33 differentially abundant (DA) ASVs in response to glucose for each incubation.** The 33 DA ASVs always had fold changes that exceeded 1.2 and p-values <0.05.

ID_Gene	ProbeID	Strain	Ecotye	ID_Gene	Sequence	Gene symbol	Description	Protein_ID2	Protein ID	Location	Pathway	COG Function
CAE18667.1_215	CUST_1180_PI442190954	MED4	HLI	CAE18667.1_215	AATAGAGGATGGACTAGCAGAAGATGATTGGGAGGGTTGGTCAGCCCTAAATAAAGAGAT	eno	Enolase	PMM0208	CAE18667.1	201456..202748	Glycolysis/Gluconeogenesis	COG0148
CAE18667.1_215	CUST_1181_PI442190954	MED4	HLI	CAE18667.1_215	GGCTATTCAGAAAGCAGGATTTATTCCTGGTGAGCAGGTATCGTTAGCATTAGACGTAGC	eno	Enolase	PMM0208	CAE18667.1	201456..202748	Glycolysis/Gluconeogenesis	COG0148
CAE18667.1_215	CUST_1182_PI442190954	MED4	HLI	CAE18667.1_215	GCTGGTTTTACAAGTGTTATCAGTCATAGAAGCGGCGAAACAGAAGATACAACGATTGCC	eno	Enolase	PMM0208	CAE18667.1	201456..202748	Glycolysis/Gluconeogenesis	COG0148
CAE18667.1_215	CUST_1183_PI442190954	MED4	HLI	CAE18667.1_215	GGCTCTTAAGGATTGAGGAAGAGTTGGGTGATCAAGCTAGATTTGCTGGTGATTTAGGAT	eno	Enolase	PMM0208	CAE18667.1	201456..202748	Glycolysis/Gluconeogenesis	COG0148
CAE18667.1_215	CUST_1184_PI442190954	MED4	HLI	CAE18667.1_215	CGAGCCGGGCAAATTAAAACTGGCTCTTTGAGCAGAAGTGAAAGAATTGCTAAGTACAAT	eno	Enolase	PMM0208	CAE18667.1	201456..202748	Glycolysis/Gluconeogenesis	COG0148
CAE18667.1_215	CUST_1185_PI442190954	MED4	HLI	CAE18667.1_215	AGACGAAGGTGGTTTTGCTCCAGAATTATCATCTAGTGAAGCAGCAGGAGATTTGCTTTT	eno	Enolase	PMM0208	CAE18667.1	201456..202748	Glycolysis/Gluconeogenesis	COG0148
CAE18684.1_232	CUST_1269_PI442190954	MED4	HLI	CAE18684.1_232	AGATGACTCTATAAAAATAGGTTGCAATTATTCAATGATGAGAGGTTGGGATAGAGACCT	eda	2-dehydro-3-deoxyphosphogluconate aldolase	PMM0225	CAE18684.1	219107..219748	Entner-Dudoroff	COG0800
CAE18684.1_232	CUST_1270_PI442190954	MED4	HLI	CAE18684.1_232	GCTGCCGGAGGACTATCTATATCAGATATACCGCTATATAAATCTTTAGGATATAAAGCT	eda	2-dehydro-3-deoxyphosphogluconate aldolase	PMM0225	CAE18684.1	219107..219748	Entner-Dudoroff	COG0800
CAE18684.1_232	CUST_1271_PI442190954	MED4	HLI	CAE18684.1_232	TGGATAAATTACGTTTCAAATTTAAGATTAAAGTTCCCAAGTCTTAATCTAGGCTCTGCT	eda	2-dehydro-3-deoxyphosphogluconate aldolase	PMM0225	CAE18684.1	219107..219748	Entner-Dudoroff	COG0800
CAE18684.1_232	CUST_1272_PI442190954	MED4	HLI	CAE18684.1_232	TGGGGATATGGGTTTCAAAAATAATAAAATAGATCCAAAGATATATGAATGGCTCAGAAG	eda	2-dehydro-3-deoxyphosphogluconate aldolase	PMM0225	CAE18684.1	219107..219748	Entner-Dudoroff	COG0800
CAE18684.1_232	CUST_1273_PI442190954	MED4	HLI	CAE18684.1_232	ACTATTGAAAAAGATTTAGTTGAACTAGAAATAGAGCTTTTAATACAGGCAGGTTTTACA	eda	2-dehydro-3-deoxyphosphogluconate aldolase	PMM0225	CAE18684.1	219107..219748	Entner-Dudoroff	COG0800
CAE18684.1_232	CUST_1274_PI442190954	MED4	HLI	CAE18684.1_232	AATTCCTGGAATCAAGCATTTAAAAGATCTTAAACAAGCAATAGATTTGAATTGTAAAAT	eda	2-dehydro-3-deoxyphosphogluconate aldolase	PMM0225	CAE18684.1	219107..219748	Entner-Dudoroff	COG0800
AAP99281.1_235	CUST_1295_PI442190958	SS120	LLII/III	AAP99281.1_235	AATCAACTTCTTCGAATTGAAGATCAATTAGGTGCTCAGGCTGTCTACGCAGGATCTGTT	eno	Enolase		AAP99281.1	231829..233127	Glycolysis/Gluconeogenesis	COG0148
AAP99281.1_235	CUST_1296_PI442190958	SS120	LLII/III	AAP99281.1_235	TGAAACTGAAGATACTACAATCGCTGATCTAGCAGTGGCAACAAGAGCTGGTCAAATCAA	eno	Enolase		AAP99281.1	231829..233127	Glycolysis/Gluconeogenesis	COG0148
AAP99281.1_235	CUST_1297_PI442190958	SS120	LLII/III	AAP99281.1_235	TAAGGTCCAACTAGTTGGGGATGATTTGTTTGTCACAAATACAAAACGATTGCGCCAAGG	eno	Enolase		AAP99281.1	231829..233127	Glycolysis/Gluconeogenesis	COG0148
AAP99281.1_235	CUST_1298_PI442190958	SS120	LLII/III	AAP99281.1_235	GAAAAAGCTGGATTTCGTCCAGGAGAAGAGATTTCTTTGGCTTTAGATGTTGCAAGCACT	eno	Enolase		AAP99281.1	231829..233127	Glycolysis/Gluconeogenesis	COG0148
AAP99281.1_235	CUST_1299_PI442190958	SS120	LLII/III	AAP99281.1_235	AGAAGCTTCTTTCAGATAAGGGTTTATCTACTGCAGTTGGAGATGAAGGAGGCTTTGCAC	eno	Enolase		AAP99281.1	231829..233127	Glycolysis/Gluconeogenesis	COG0148
AAP99281.1_235	CUST_1300_PI442190958	SS120	LLII/III	AAP99281.1_235	ATGCTGCCAATAATCTTGATTTTCAAGAATTTATGTTGGTGCCTCATGGTGCTGAGAGCT	eno	Enolase		AAP99281.1	231829..233127	Glycolysis/Gluconeogenesis	COG0148
AAP99300.1_254	CUST_1399_PI442190958	SS120	LLII/III	AAP99300.1_254	ACCTTGTTGGGATATTGAACTTCAAAAGAGAGCTAAAGCATTGGGTCAAGCTTTAGTGCC	eda	2-dehydro-3-deoxyphosphogluconate aldolase		AAP99300.1	251069..251758	Entner-Dudoroff	COG0800
AAP99300.1_254	CUST_1400_PI442190958	SS120	LLII/III	AAP99300.1_254	TATCGCAGTTGGAAGAGAGTTAATACAAAAAGAATCTGTAGACCCCCAGCTGGCAGAATG	eda	2-dehydro-3-deoxyphosphogluconate aldolase		AAP99300.1	251069..251758	Entner-Dudoroff	COG0800
AAP99300.1_254	CUST_1401_PI442190958	SS120	LLII/III	AAP99300.1_254	AGCTGCAGGAGGAATGTCAATCATCAATATAAACAGTTGGTTAGAACAAGGGTATGGCGC	eda	2-dehydro-3-deoxyphosphogluconate aldolase		AAP99300.1	251069..251758	Entner-Dudoroff	COG0800
AAP99300.1_254	CUST_1402_PI442190958	SS120	LLII/III	AAP99300.1_254	CAACAAGCAAAAAACTTTGGATATCGAATAATCAAGCTATTCCCTGCCTCAGTTATAGGA	eda	2-dehydro-3-deoxyphosphogluconate aldolase		AAP99300.1	251069..251758	Entner-Dudoroff	COG0800
AAP99300.1_254	CUST_1403_PI442190958	SS120	LLII/III	AAP99300.1_254	TGCATCTTTAACTTATCAGCCTTTATTAATTGTCTTGCGAATCACTGAAGACGATCTTGA	eda	2-dehydro-3-deoxyphosphogluconate aldolase		AAP99300.1	251069..251758	Entner-Dudoroff	COG0800
AAP99300.1_254	CUST_1404_PI442190958	SS120	LLII/III	AAP99300.1_254	ACACTGCAATCTTTAGGAGTTAGAAATATTGAAATTGCTTGGTCTTCTCACAAAAACTGG	eda	2-dehydro-3-deoxyphosphogluconate aldolase		AAP99300.1	251069..251758	Entner-Dudoroff	COG0800
CAE18779.1_330	CUST_1784_PI442190954	MED4	HLI	CAE18779.1_330	TCCTATAAAAAGAACCCTTACTGGAGAAATTGAATTTAAAAAAAATGAAGACACACAGAA	minE	septum site-determining protein MinE	PMM0320	CAE18779.1	305233..305553	Cell Division	COG0851
CAE18779.1_330	CUST_1785_PI442190954	MED4	HLI	CAE18779.1_330	TGAGGAAGTAGCTGTAAGTCTAGAAACCGAAGATAGAATGACAGCATTAGTTGCGAATCT	minE	septum site-determining protein MinE	PMM0320	CAE18779.1	305233..305553	Cell Division	COG0851
CAE18779.1_330	CUST_1786_PI442190954	MED4	HLI	CAE18779.1_330	AGTTCATTGACAACTGATCTTTTGGATAAAATGAGAAAAGAAATACTTGATGTGGTTGCT	minE	septum site-determining protein MinE	PMM0320	CAE18779.1	305233..305553	Cell Division	COG0851
CAE18779.1_330	CUST_1787_PI442190954	MED4	HLI	CAE18779.1_330	AGGCAGAGAAACGGCTAGTGCCAATACTGCTAGAGAAAGATTGCAATTAGTATTAGCTCA	minE	septum site-determining protein MinE	PMM0320	CAE18779.1	305233..305553	Cell Division	COG0851
CAE18780.1_331	CUST_1788_PI442190954	MED4	HLI	CAE18780.1_331	GCTCTTTCAGCATGCAAAGAAGCCATAGTGGTTACAAATCCTGAATTATCTGCAGTTCGT	minD	septum site-determining protein MinD	PMM0321	CAE18780.1	305556..306371	Cell Division	COG2894
CAE18780.1_331	CUST_1789_PI442190954	MED4	HLI	CAE18780.1_331	CCAGCTGGTGATCCTAGGATGCTTGACTGGATGAAACCTGAAGATATGAAACAAATCAGT	minD	septum site-determining protein MinD	PMM0321	CAE18780.1	305556..306371	Cell Division	COG2894
CAE18780.1_331	CUST_1790_PI442190954	MED4	HLI	CAE18780.1_331	CCTAGCAAATAGTGGAGCTACAACAGCCGTGTTAGATGCTGATTTTGGATTGAGGAATTT	minD	septum site-determining protein MinD	PMM0321	CAE18780.1	305556..306371	Cell Division	COG2894
CAE18780.1_331	CUST_1791_PI442190954	MED4	HLI	CAE18780.1_331	TGCTGGGTATTGTTTTAGAGGATGAACAAGTAATAATAAGTACAAACAGAGGGGAACCCC	minD	septum site-determining protein MinD	PMM0321	CAE18780.1	305556..306371	Cell Division	COG2894
CAE18780.1_331	CUST_1792_PI442190954	MED4	HLI	CAE18780.1_331	TCAAAGGCTTACAGGAAAAGATATTCCAATTATCGATCCAAAGAATGAAGGGAAAAGCCT	minD	septum site-determining protein MinD	PMM0321	CAE18780.1	305556..306371	Cell Division	COG2894
CAE18780.1_331	CUST_1793_PI442190954	MED4	HLI	CAE18780.1_331	ACAAGAATTGTCGTCTTGATCAAGCATTAGTTAAACACAAAAAGGAGCCGAATTTAGCTC	minD	septum site-determining protein MinD	PMM0321	CAE18780.1	305556..306371	Cell Division	COG2894
CAE18781.1_332	CUST_1794_PI442190954	MED4	HLI	CAE18781.1_332	TGCTTTCGCCGGAAAAAATGGAAATAAAAATGCTTCTGTTGCCTCGCTTTATCTAAACCC	minC	septum site-determining protein MinC	PMM0322	CAE18781.1	306489..307151	Cell Division	COG0850
CAE18781.1_332	CUST_1795_PI442190954	MED4	HLI	CAE18781.1_332	ACTAAGAATCTGCGAAATTGTAGCTATAGGCCCAAAAGAGAAGCCTAAGGGTCAACATCC	minC	septum site-determining protein MinC	PMM0322	CAE18781.1	306489..307151	Cell Division	COG0850
CAE18781.1_332	CUST_1796_PI442190954	MED4	HLI	CAE18781.1_332	GGACATACTTCACAAAGGCACGGTTAGATCAGGGGATCGAATATCTTCAAATGGTGATCT	minC	septum site-determining protein MinC	PMM0322	CAE18781.1	306489..307151	Cell Division	COG0850
CAE18781.1_332	CUST_1797_PI442190954	MED4	HLI	CAE18781.1_332	TGGTGACGTAAATCCAGGAGCTATTATTTCCGCAAATAATAACGTTTATGTATGGGGAAA	minC	septum site-determining protein MinC	PMM0322	CAE18781.1	306489..307151	Cell Division	COG0850
CAE18781.1_332	CUST_1798_PI442190954	MED4	HLI	CAE18781.1_332	TGAGATTGATCAAAATTTTTTCGACAAATTAATTACTAAAGAAGCCCCAGCTGCAGCAAA	minC	septum site-determining protein MinC	PMM0322	CAE18781.1	306489..307151	Cell Division	COG0850
CAE18781.1_332	CUST_1799_PI442190954	MED4	HLI	CAE18781.1_332	TCAACTCAACGTTTTTAAAAATCAAAGACCTTGAAAATCAATTACCACTAGATCCTTCAT	minC	septum site-determining protein MinC	PMM0322	CAE18781.1	306489..307151	Cell Division	COG0850
AAP99408.1_362	CUST_1963_PI442190958	SS120	LLII/III	AAP99408.1_362	GAAGGTGCAGTGAGTCTTGAAACTGAAGATAGAATGACTGCATTAGTTGCCAATCTTCCA	minE	septum site-determining protein MinE		AAP99408.1	345276..345596	Cell Division	COG0851
AAP99408.1_362	CUST_1964_PI442190958	SS120	LLII/III	AAP99408.1_362	TGGAACAAATGAGAGAAGAAATTCTTAGCGTTGTAGCTAAATATGTAGAAATAGATTTTG	minE	septum site-determining protein MinE		AAP99408.1	345276..345596	Cell Division	COG0851
AAP99408.1_362	CUST_1965_PI442190958	SS120	LLII/III	AAP99408.1_362	GCTCGAGAAAGGCTGCAATTAGTACTCGCTCATGACAGAACTGATTTAAGTCCAGATCTC	minE	septum site-determining protein MinE		AAP99408.1	345276..345596	Cell Division	COG0851
AAP99408.1_362	CUST_1966_PI442190958	SS120	LLII/III	AAP99408.1_362	ATGACACTTCGAGACATTCTAAACAAATTGATGGGCAGACAGCCTGCGAGTGCTGCAAAA	minE	septum site-determining protein MinE		AAP99408.1	345276..345596	Cell Division	COG0851
AAP99409.1_363	CUST_1967_PI442190958	SS120	LLII/III	AAP99409.1_363	GCAGTAAGAGATGCTGATCGGGTAATTGGACTCCTAAATACACATGGGATCAAACCTGTC	minD	septum site-determining protein MinD		AAP99409.1	345602..346417	Cell Division	COG2894
AAP99409.1_363	CUST_1968_PI442190958	SS120	LLII/III	AAP99409.1_363	CTTCTCAAGAAGGTTCTGGAATAGCAGACAAATTCCGCCGACTGATGCAAACAAAGATTT	minD	septum site-determining protein MinD		AAP99409.1	345602..346417	Cell Division	COG2894
AAP99409.1_363	CUST_1969_PI442190958	SS120	LLII/III	AAP99409.1_363	TTACCGGCTGGCAATCCAAGGATGCTTGATTGGCTTAAGCCAGAAGATATGAAAAAAATA	minD	septum site-determining protein MinD		AAP99409.1	345602..346417	Cell Division	COG2894
AAP99409.1_363	CUST_1970_PI442190958	SS120	LLII/III	AAP99409.1_363	TAGAAGAGACTTGTCGCCTTGATCAAGCCTTGGTAAAGCATAAACAAGAGCCAAACCTAT	minD	septum site-determining protein MinD		AAP99409.1	345602..346417	Cell Division	COG2894
AAP99409.1_363	CUST_1971_PI442190958	SS120	LLII/III	AAP99409.1_363	CAGAAAAGGCTCCCCTACTGCTGTTCTTGATGCTGATTTTGGCTTACGTAATCTAGATCT	minD	septum site-determining protein MinD		AAP99409.1	345602..346417	Cell Division	COG2894
AAP99409.1_363	CUST_1972_PI442190958	SS120	LLII/III	AAP99409.1_363	AGGGTGCGACCAAAAATGATGGAAAGCCAAGAAATGCTATCAATTGAAGATGTGACAGAT	minD	septum site-determining protein MinD		AAP99409.1	345602..346417	Cell Division	COG2894
AAP99410.1_364	CUST_1973_PI442190958	SS120	LLII/III	AAP99410.1_364	ATTTTAATTCTCGGAGATGTGAATCCAGGCGCAACAGTTTTAGCTGGTGGGAATGTAATG	minC	septum site-determining protein MinC		AAP99410.1	346556..347221	Cell Division	COG0850
AAP99410.1_364	CUST_1974_PI442190958	SS120	LLII/III	AAP99410.1_364	TGCAGGCAAATATGGAAATAATCAAGCCAAAATAACTGCTCTTCAGCTTAGGCCTGTTCA	minC	septum site-determining protein MinC		AAP99410.1	346556..347221	Cell Division	COG0850
AAP99410.1_364	CUST_1975_PI442190958	SS120	LLII/III	AAP99410.1_364	TGAATCAACTAACGCAGAAAGTATTGTTAGCGCTTCTGCATTAGGCCTAAATGCAAACCT	minC	septum site-determining protein MinC		AAP99410.1	346556..347221	Cell Division	COG0850
AAP99410.1_364	CUST_1976_PI442190958	SS120	LLII/III	AAP99410.1_364	GCAGAAGAGGCAACTATCCAAGAGGAAGTAATTGTCATTAAACCAGCAAGAACCACCTGA	minC	septum site-determining protein MinC		AAP99410.1	346556..347221	Cell Division	COG0850
AAP99410.1_364	CUST_1977_PI442190958	SS120	LLII/III	AAP99410.1_364	ACGCAGCATTGTTATTTCATAAAGGGACTCTAAGGTCTGGAGAAGTATTGGAAGCTGATG	minC	septum site-determining protein MinC		AAP99410.1	346556..347221	Cell Division	COG0850
AAP99410.1_364	CUST_1978_PI442190958	SS120	LLII/III	AAP99410.1_364	AGGGTACCAAAAAGGATTCATCAAAATTAACTCTGGAGCATTACCCCTAACTTGCAAAGA	minC	septum site-determining protein MinC		AAP99410.1	346556..347221	Cell Division	COG0850
AAP99486.1_440	CUST_2387_PI442190958	SS120	LLII/III	AAP99486.1_440	TTTTCATGGCCTACATGTAACTCTAGGCGCGATAATGATTTTAATTGTCTGGTGGCAAGC	ctaE/coxC	Cytochrome c oxidase subunit III		AAP99486.1	422970..423572	Respiration	COG1845
AAP99486.1_440	CUST_2388_PI442190958	SS120	LLII/III	AAP99486.1_440	AGGCGAAAGAATCAGAAAAATGCCAAAGATGGTTACTAATAACCGCTGGCCTTGGAATAG	ctaE/coxC	Cytochrome c oxidase subunit III		AAP99486.1	422970..423572	Respiration	COG1845
AAP99486.1_440	CUST_2389_PI442190958	SS120	LLII/III	AAP99486.1_440	AAAGCAGTCAACCCACTGCTACCTGACGCTATATACGAATTAGAACTACCTCTACCAACA	ctaE/coxC	Cytochrome c oxidase subunit III		AAP99486.1	422970..423572	Respiration	COG1845
AAP99486.1_440	CUST_2390_PI442190958	SS120	LLII/III	AAP99486.1_440	TTTACTTTGCCCTTTGGGCTTACCGACAATCTTTACGCAAGTACTTTCTATGCTCTCACT	ctaE/coxC	Cytochrome c oxidase subunit III		AAP99486.1	422970..423572	Respiration	COG1845
AAP99486.1_440	CUST_2391_PI442190958	SS120	LLII/III	AAP99486.1_440	AGCCGCAGAACTCTATTGGCACTTTGTTGATGGGATTTGGGTAATTTTATTCATCATCCT	ctaE/coxC	Cytochrome c oxidase subunit III		AAP99486.1	422970..423572	Respiration	COG1845
AAP99486.1_440	CUST_2392_PI442190958	SS120	LLII/III	AAP99486.1_440	ACACTAAATACCATTTTGCTGCTTGTTAGCAGTGCAACCTTCCATAGAGCTGGGAAG	ctaE/coxC	Cytochrome c oxidase subunit III		AAP99486.1	422970..423572	Respiration	COG1845
AAP99487.1_441	CUST_2393_PI442190958	SS120	LLII/III	AAP99487.1_441	GCCTTATGGCTATGGACAAAGCCAACTGTCCAAAGAAATTCCCAATTCCTTTATTGAGGA	cyoB/coxA	Cytochrome c oxidase subunit I		AAP99487.1	423572..425230	Respiration	COG0843
AAP99487.1_441	CUST_2394_PI442190958	SS120	LLII/III	AAP99487.1_441	GTAGTGTTGGGGCTCTCCTAATGGCTCTAAGTACTCTTCCATTCCTTTGGAATGTTTTCC	cyoB/coxA	Cytochrome c oxidase subunit I		AAP99487.1	423572..425230	Respiration	COG0843
AAP99487.1_441	CUST_2395_PI442190958	SS120	LLII/III	AAP99487.1_441	GCTAGGACTTAATGGAATGCCAAGACGAGTAGCTGAATACGACCCTCAATTCACTTTTGT	cyoB/coxA	Cytochrome c oxidase subunit I		AAP99487.1	423572..425230	Respiration	COG0843
AAP99487.1_441	CUST_2396_PI442190958	SS120	LLII/III	AAP99487.1_441	TTATTGCTGTACCGACAGGAATAAAATTCTTTAACTGGGTAGCAACTCTATGGGGAGGGC	cyoB/coxA	Cytochrome c oxidase subunit I		AAP99487.1	423572..425230	Respiration	COG0843
AAP99487.1_441	CUST_2397_PI442190958	SS120	LLII/III	AAP99487.1_441	GGTGTGGGCTCACCATATGTTTACAAGTGGTACTCCTCCTTGGATGAGACTCTTTTTTAC	cyoB/coxA	Cytochrome c oxidase subunit I		AAP99487.1	423572..425230	Respiration	COG0843
AAP99487.1_441	CUST_2398_PI442190958	SS120	LLII/III	AAP99487.1_441	AAACCTCTTTTTGGCTATGCCACAATGGTTTACTCAATCATGGGGATTGTAGTTTTGGGA	cyoB/coxA	Cytochrome c oxidase subunit I		AAP99487.1	423572..425230	Respiration	COG0843
AAP99488.1_442	CUST_2399_PI442190958	SS120	LLII/III	AAP99488.1_442	ACGGCATAGCAATAGAAGACAATCTGCCTTTAGAGATATTTTGGACAGCTGTACCTGCAA	ctaC/coxB	Cytochrome c oxidase subunit II		AAP99488.1	425227..426060	Respiration	COG1622
AAP99488.1_442	CUST_2400_PI442190958	SS120	LLII/III	AAP99488.1_442	TGTTTGTTGGTCTTTATAGCTACGACATATACGAACGAATGGGTGGGATGCAATTAGCCT	ctaC/coxB	Cytochrome c oxidase subunit II		AAP99488.1	425227..426060	Respiration	COG1622
AAP99488.1_442	CUST_2401_PI442190958	SS120	LLII/III	AAP99488.1_442	CTCTGGAGAACTTCATGTTCCAGCAGGTCGTCCCGTAAGTATGAAAATGGATTCAAAAGA	ctaC/coxB	Cytochrome c oxidase subunit II		AAP99488.1	425227..426060	Respiration	COG1622
AAP99488.1_442	CUST_2402_PI442190958	SS120	LLII/III	AAP99488.1_442	AGAAGAGAATCCTGAAGGAGCTCTATCCCCCGTTAATGTTGAAGTAACTGCAATGCAATT	ctaC/coxB	Cytochrome c oxidase subunit II		AAP99488.1	425227..426060	Respiration	COG1622
AAP99488.1_442	CUST_2403_PI442190958	SS120	LLII/III	AAP99488.1_442	CCTTTTGGGTCCCTGAATTCCGACTAAAGCAAGATATAATTCCCGGTCAACCAACAATTT	ctaC/coxB	Cytochrome c oxidase subunit II		AAP99488.1	425227..426060	Respiration	COG1622
AAP99488.1_442	CUST_2404_PI442190958	SS120	LLII/III	AAP99488.1_442	GGGGGAATGAGATCAACTGTAGTAGTTGAGGAGCCGGAAGAATATGAATCTTGGTTCAAC	ctaC/coxB	Cytochrome c oxidase subunit II		AAP99488.1	425227..426060	Respiration	COG1622
AAP99498.1_452	CUST_2452_PI442190958	SS120	LLII/III	AAP99498.1_452	ACTTCAGTTGTAGGATTAATGGGTAATGCAGGACAAGCAAATTACGCTTCTGCAAAAGCA	gdh	3-oxoacyl-acyl-carrier-protein reductase		AAP99498.1	434454..435206	Entner-Dudoroff	COG1028
AAP99498.1_452	CUST_2453_PI442190958	SS120	LLII/III	AAP99498.1_452	AAAGAGTTAGATGCAGAACCAATACTTTCAGCGATCCCATTAGGGCATTTTGGGAGTACT	gdh	3-oxoacyl-acyl-carrier-protein reductase		AAP99498.1	434454..435206	Entner-Dudoroff	COG1028
AAP99498.1_452	CUST_2454_PI442190958	SS120	LLII/III	AAP99498.1_452	GTGTTTTCCTGTGCACTAAAGCAGTTTCAAGATCAATGCTTAAACAAAGAAAAGGGCGCA	gdh	3-oxoacyl-acyl-carrier-protein reductase		AAP99498.1	434454..435206	Entner-Dudoroff	COG1028
AAP99498.1_452	CUST_2455_PI442190958	SS120	LLII/III	AAP99498.1_452	GGACAGTTCGTTTTCTAGCTGCAGATCCAGCCTCTTCTTATATAACAGGGCAAGTTCTTC	gdh	3-oxoacyl-acyl-carrier-protein reductase		AAP99498.1	434454..435206	Entner-Dudoroff	COG1028
AAP99498.1_452	CUST_2456_PI442190958	SS120	LLII/III	AAP99498.1_452	CCAAAGCAGAAGAAGTAGTCTCCTTAATCAAAAGCACTGGAGGGAAAGCATATGCATTGC	gdh	3-oxoacyl-acyl-carrier-protein reductase		AAP99498.1	434454..435206	Entner-Dudoroff	COG1028
AAP99498.1_452	CUST_2457_PI442190958	SS120	LLII/III	AAP99498.1_452	GTGCTGCAAAAGAATTTGCTAGTAGAGGTATCACAGTAAATGCTGTTGCACCTGGCTTTA	gdh	3-oxoacyl-acyl-carrier-protein reductase		AAP99498.1	434454..435206	Entner-Dudoroff	COG1028
CAE18903.1_499	CUST_2539_PI442190954	MED4	HLI	CAE18903.1_499	GCAGATGGGATGACTTTTGCTGGCTTCTTTGCTGCTTACCTCACATATAAAGCAGTTAAT	ctaE/coxC	Cytochrome c oxidase subunit III	PMM0444	CAE18903.1	422877..423479	Respiration	COG1845
CAE18903.1_499	CUST_2540_PI442190954	MED4	HLI	CAE18903.1_499	GCCGTTCGGACTAACGGATAATTTATTTGCAAGTACTTTTTATGCCTTAACAGGCTTTCA	ctaE/coxC	Cytochrome c oxidase subunit III	PMM0444	CAE18903.1	422877..423479	Respiration	COG1845
CAE18903.1_499	CUST_2541_PI442190954	MED4	HLI	CAE18903.1_499	TGGCAAACAAGACCAAAGGAAGGAAGAATTACTAATCAAAATATGTTCCCATTAGAAGCT	ctaE/coxC	Cytochrome c oxidase subunit III	PMM0444	CAE18903.1	422877..423479	Respiration	COG1845
CAE18903.1_499	CUST_2542_PI442190954	MED4	HLI	CAE18903.1_499	ACAATTTTATTGCTTGTAAGTAGCGCGACTTTTCATAAAGCAGGAAAAGCTCTTCTTAAA	ctaE/coxC	Cytochrome c oxidase subunit III	PMM0444	CAE18903.1	422877..423479	Respiration	COG1845
CAE18903.1_499	CUST_2543_PI442190954	MED4	HLI	CAE18903.1_499	ACTCAAAAATGGTTGCTAGTCACTGCTCTACTTGGGATTACTTTTTAATATGTCAACTA	ctaE/coxC	Cytochrome c oxidase subunit III	PMM0444	CAE18903.1	422877..423479	Respiration	COG1845
CAE18903.1_499	CUST_2544_PI442190954	MED4	HLI	CAE18903.1_499	ATTGAGGCGAGTGAACATCATGAAGACTTTAGGATGTTTGGATTAATAACTTTTTGATA	ctaE/coxC	Cytochrome c oxidase subunit III	PMM0444	CAE18903.1	422877..423479	Respiration	COG1845
CAE18904.1_500	CUST_2545_PI442190954	MED4	HLI	CAE18904.1_500	CCAAAGGTAACCGGCAAGTTAATGAGCGAAAAACTGGGGATACTACATTTTGCCATAACT	cyoB/coxA	Cytochrome c oxidase subunit I	PMM0445	CAE18904.1	423492..425117	Respiration	COG0843
CAE18904.1_500	CUST_2546_PI442190954	MED4	HLI	CAE18904.1_500	TGGGAAGGATTCTGGGGATAATCCTTGGAATGCTTTAACTCCTGAATGGTTAACATCCTC	cyoB/coxA	Cytochrome c oxidase subunit I	PMM0445	CAE18904.1	423492..425117	Respiration	COG0843
CAE18904.1_500	CUST_2547_PI442190954	MED4	HLI	CAE18904.1_500	CAACATTGGCTTGGTCTTAATGGAATGCCTAGAAGAGTTGCGGATACGATCCACAATTT	cyoB/coxA	Cytochrome c oxidase subunit I	PMM0445	CAE18904.1	423492..425117	Respiration	COG0843
CAE18904.1_500	CUST_2548_PI442190954	MED4	HLI	CAE18904.1_500	ACCACAATGGTTTTTTCAATAATGGGAATAGTTGTGCTTGGTCTAGTTGTGTGGGCTCAT	cyoB/coxA	Cytochrome c oxidase subunit I	PMM0445	CAE18904.1	423492..425117	Respiration	COG0843
CAE18904.1_500	CUST_2549_PI442190954	MED4	HLI	CAE18904.1_500	GGAAGGCGAAGCACCTTTAGTTGAAGAACCTTATGGTTATGGCAAACCAATTTCTCAAGA	cyoB/coxA	Cytochrome c oxidase subunit I	PMM0445	CAE18904.1	423492..425117	Respiration	COG0843
CAE18904.1_500	CUST_2550_PI442190954	MED4	HLI	CAE18904.1_500	TTGGCGGTATTACAGGAGTTGCTCTTGCTCAAGTACCTTTTGATATTCACGTGCATGATA	cyoB/coxA	Cytochrome c oxidase subunit I	PMM0445	CAE18904.1	423492..425117	Respiration	COG0843
CAE18905.1_501	CUST_2551_PI442190954	MED4	HLI	CAE18905.1_501	GCCCTTATCACGGGGGGATGAGGGCTTCAATAATAGTTGAAGAAGAATCTGACTACAAAG	ctaC/coxB	Cytochrome c oxidase subunit II	PMM0446	CAE18905.1	425114..425917	Respiration	COG1622
CAE18905.1_501	CUST_2552_PI442190954	MED4	HLI	CAE18905.1_501	TTATCCGAAAGGGGAATTTATTTCTGGGGAGCTTCATGTTCCAGTTGATCGCAAAGTTTC	ctaC/coxB	Cytochrome c oxidase subunit II	PMM0446	CAE18905.1	425114..425917	Respiration	COG1622
CAE18905.1_501	CUST_2553_PI442190954	MED4	HLI	CAE18905.1_501	ACGATCGAATGGGAGGTATGAAAGAACTAAATCATAGCCATGAAATGATGACCTCCAACA	ctaC/coxB	Cytochrome c oxidase subunit II	PMM0446	CAE18905.1	425114..425917	Respiration	COG1622
CAE18905.1_501	CUST_2554_PI442190954	MED4	HLI	CAE18905.1_501	ATGCTTTTTGGGTGCCAGAATTCAGAATAAAACAAGATATTATTCCTGGCCAACCTACGA	ctaC/coxB	Cytochrome c oxidase subunit II	PMM0446	CAE18905.1	425114..425917	Respiration	COG1622
CAE18905.1_501	CUST_2555_PI442190954	MED4	HLI	CAE18905.1_501	TCTTTTTGGATCGGATTTAATGTTAACTTACTTCCTGTTGAAGCAAGTATAAATGCGCCT	ctaC/coxB	Cytochrome c oxidase subunit II	PMM0446	CAE18905.1	425114..425917	Respiration	COG1622
CAE18905.1_501	CUST_2556_PI442190954	MED4	HLI	CAE18905.1_501	TGAGGTTGCTAGCAATAATTTATCTGTTGAAGTTTCAGCTATGCAATTTGCCTTTTTATT	ctaC/coxB	Cytochrome c oxidase subunit II	PMM0446	CAE18905.1	425114..425917	Respiration	COG1622
CAE18912.1_509	CUST_2595_PI442190954	MED4	HLI	CAE18912.1_509	AAAGTTGCTTTAATTACAGGGGCTAGCAGGGGCATTGGTAAAGAAATTGCCTTAGAACTT	gdh	3-oxoacyl-acyl-carrier-protein reductase	PMM0453	CAE18912.1	432615..433364	Entner-Dudoroff	COG1028
CAE18912.1_509	CUST_2596_PI442190954	MED4	HLI	CAE18912.1_509	CGGAATAATTGGAAATCCTGGACAAGCAAATTATTCTGCCGCAAAAGCAGGTGTTATTGG	gdh	3-oxoacyl-acyl-carrier-protein reductase	PMM0453	CAE18912.1	432615..433364	Entner-Dudoroff	COG1028
CAE18912.1_509	CUST_2597_PI442190954	MED4	HLI	CAE18912.1_509	CTGGCATAACAAGAGATGGGCTCTTAATGCGAATGAAATCAGAACAATGGGATGACGTTC	gdh	3-oxoacyl-acyl-carrier-protein reductase	PMM0453	CAE18912.1	432615..433364	Entner-Dudoroff	COG1028
CAE18912.1_509	CUST_2598_PI442190954	MED4	HLI	CAE18912.1_509	TCGAGTAATGCTGGAAGCTATATAACAGGACAAACAATAAGTATTGATGGGGGCATGAGT	gdh	3-oxoacyl-acyl-carrier-protein reductase	PMM0453	CAE18912.1	432615..433364	Entner-Dudoroff	COG1028
CAE18912.1_509	CUST_2599_PI442190954	MED4	HLI	CAE18912.1_509	ACCTGTGCAAAAGAATTTGCTTCAAGAGGGATAAATGTTAATGCTATAGCTCCAGGTTTT	gdh	3-oxoacyl-acyl-carrier-protein reductase	PMM0453	CAE18912.1	432615..433364	Entner-Dudoroff	COG1028
CAE18912.1_509	CUST_2600_PI442190954	MED4	HLI	CAE18912.1_509	ACGAAGAGATTATCAAAGCCATACCATTAGGAAAGCTAGGTAGTTGTTCACAGATAGCAA	gdh	3-oxoacyl-acyl-carrier-protein reductase	PMM0453	CAE18912.1	432615..433364	Entner-Dudoroff	COG1028
AAP99564.1_518	CUST_2813_PI442190958	SS120	LLII/III	AAP99564.1_518	TGTTGATTAAGGTTGCATCAACTTGGGAAGGGATTAAGGCTGCTGAAGTTCTTGAAAGTG	tal	Transaldolase		AAP99564.1	495609..496616	Pentose Phosphate	COG0176
AAP99564.1_518	CUST_2814_PI442190958	SS120	LLII/III	AAP99564.1_518	TGGTATAAAGCAGAAACAGGGAAGGACTCTTATGCTGGAGAAGAAGATCCAGGTGTAGTT	tal	Transaldolase		AAP99564.1	495609..496616	Pentose Phosphate	COG0176
AAP99564.1_518	CUST_2815_PI442190958	SS120	LLII/III	AAP99564.1_518	TGGAGCAGCTTTTGAGTAAAAGGCTTTCTTATATTGAGAGCGGTTCAAATGCTGAGCTTT	tal	Transaldolase		AAP99564.1	495609..496616	Pentose Phosphate	COG0176
AAP99564.1_518	CUST_2816_PI442190958	SS120	LLII/III	AAP99564.1_518	TTCATTGTAATTTAACGTTGTTATTTGGCTTTTCTCAGGCAGTTGCATGTGCAGAGGCAG	tal	Transaldolase		AAP99564.1	495609..496616	Pentose Phosphate	COG0176
AAP99564.1_518	CUST_2817_PI442190958	SS120	LLII/III	AAP99564.1_518	ATTCCTGGGAGAGTATCTACTGAAGTTGATGCAAGATTGAGTTTTAATACGCAGGCAACT	tal	Transaldolase		AAP99564.1	495609..496616	Pentose Phosphate	COG0176
AAP99564.1_518	CUST_2818_PI442190958	SS120	LLII/III	AAP99564.1_518	AGCAGGCTGTGATTTGCTTACGATTGCACCAAAATTTTTAGAACAATTGGAAAACTCTGA	tal	Transaldolase		AAP99564.1	495609..496616	Pentose Phosphate	COG0176
CAE18978.1_576	CUST_2963_PI442190954	MED4	HLI	CAE18978.1_576	AGAGGATCAAATGGCAAGTGAAAAGCTTAGTGAAGGAATAACAGGCTTCAGCAAAGCAAT	tal	Transaldolase	PMM0519	CAE18978.1	490726..491727	Pentose Phosphate	COG0176
CAE18978.1_576	CUST_2964_PI442190954	MED4	HLI	CAE18978.1_576	CGCCTTTCGTAGGGCGAATATTGGATTGGCATAAAGCAAAAACTGGCAAAGATAACTTTG	tal	Transaldolase	PMM0519	CAE18978.1	490726..491727	Pentose Phosphate	COG0176
CAE18978.1_576	CUST_2965_PI442190954	MED4	HLI	CAE18978.1_576	TCTACAGAAGTTGATGCAAGACTAAGTTTTGATACTGAAGCAACAGTTACAAAAGCGAGA	tal	Transaldolase	PMM0519	CAE18978.1	490726..491727	Pentose Phosphate	COG0176
CAE18978.1_576	CUST_2966_PI442190954	MED4	HLI	CAE18978.1_576	AAGATCGCTTCAACTTGGGAAGGAATTAAGGCAGCTGAAATTTTAGAAAAAGAAGGTATT	tal	Transaldolase	PMM0519	CAE18978.1	490726..491727	Pentose Phosphate	COG0176
CAE18978.1_576	CUST_2967_PI442190954	MED4	HLI	CAE18978.1_576	AATGCAATTTAACTTTACTTTTTAACTTCTGCCAAGCTGTAGCCTGTGCTAATGCAAAAA	tal	Transaldolase	PMM0519	CAE18978.1	490726..491727	Pentose Phosphate	COG0176
CAE18978.1_576	CUST_2968_PI442190954	MED4	HLI	CAE18978.1_576	TGTGACCTTCTAACTATTGCTCCAAAATTTTTAGACGAACTTAATAGAGAGGAAGGGGAA	tal	Transaldolase	PMM0519	CAE18978.1	490726..491727	Pentose Phosphate	COG0176
AAP99596.1_550	CUST_2993_PI442190958	SS120	LLII/III	AAP99596.1_550	TCGTCAAACCACTCTTGGTTATATCGACAATTTACGTGAATCCTTTGTCCCAGAAGACCG	rbcL	Ribulose 1,5-bisphosphate carboxylase large subunit		AAP99596.1	524454..525866	Carbon fixation	COG1850
AAP99596.1_550	CUST_2994_PI442190958	SS120	LLII/III	AAP99596.1_550	GCGAAGCATAGTCCAGAGTTGGCTATTGCACTTGAAACCTGGAAGGAAATTAAATTCGAG	rbcL	Ribulose 1,5-bisphosphate carboxylase large subunit		AAP99596.1	524454..525866	Carbon fixation	COG1850
AAP99596.1_550	CUST_2995_PI442190958	SS120	LLII/III	AAP99596.1_550	TCTTAATTGCACAGCTACTACTCCTGAAGAGATGTATGAGCGTGCAGAGTTCGCTAAAGA	rbcL	Ribulose 1,5-bisphosphate carboxylase large subunit		AAP99596.1	524454..525866	Carbon fixation	COG1850
AAP99596.1_550	CUST_2996_PI442190958	SS120	LLII/III	AAP99596.1_550	TATGCCTATTATCATGCATGACTACATAACTGGAGGCTTTACTGCTAACACAGGCTTGGC	rbcL	Ribulose 1,5-bisphosphate carboxylase large subunit		AAP99596.1	524454..525866	Carbon fixation	COG1850
AAP99596.1_550	CUST_2997_PI442190958	SS120	LLII/III	AAP99596.1_550	CATATCCATAGAGCAATGCATGCGGTGATTGATCGTCATCCAAAGCATGGTATCCATTTC	rbcL	Ribulose 1,5-bisphosphate carboxylase large subunit		AAP99596.1	524454..525866	Carbon fixation	COG1850
AAP99596.1_550	CUST_2998_PI442190958	SS120	LLII/III	AAP99596.1_550	TAGCTTTAGAGGCTTGTGTTAAGGCACGTAATGCAGGTAGAGAGATCGAGAAGGAAAGTC	rbcL	Ribulose 1,5-bisphosphate carboxylase large subunit		AAP99596.1	524454..525866	Carbon fixation	COG1850
AAP99597.1_551	CUST_2999_PI442190958	SS120	LLII/III	AAP99597.1_551	CGAATGGTTGGTTACGATGCTTATACCCAAAGTCAGGGTACAGCTTTCGTAGTCTTTGAA	rbcS	Ribulose bisphosphate carboxylase small subunit		AAP99597.1	525970..526311	Carbon fixation	COG4451
AAP99597.1_551	CUST_3000_PI442190958	SS120	LLII/III	AAP99597.1_551	GATTTGAATGTCGTTGTTAATGAGCTTGAAGCTTGCCACCGCGCATATCCAGATC	rbcS	Ribulose bisphosphate carboxylase small subunit		AAP99597.1	525970..526311	Carbon fixation	COG4451
AAP99597.1_551	CUST_3001_PI442190958	SS120	LLII/III	AAP99597.1_551	GTGGCTCCATGCAGACCTATTGGTCTTATTGGAAATTGCCTTTCTTTGGTGAGAAAGATT	rbcS	Ribulose bisphosphate carboxylase small subunit		AAP99597.1	525970..526311	Carbon fixation	COG4451
AAP99597.1_551	CUST_3002_PI442190958	SS120	LLII/III	AAP99597.1_551	TTTGGCTTTTTGCCTCCTATGTCACAGGACGAAATTTACGACCAAATCGCTTACATCATC	rbcS	Ribulose bisphosphate carboxylase small subunit		AAP99597.1	525970..526311	Carbon fixation	COG4451
CAE19009.1_607	CUST_3139_PI442190954	MED4	HLI	CAE19009.1_607	GCATGATTACATAACTGGTGGATTTACAGCAAATACTGGCCTTGCAAACTGGTGTCGTAA	rbcL	Ribulose 1,5-bisphosphate carboxylase large subunit		CAE19009.1	519087..520502	Carbon fixation	COG1850
AAP99743.1_697	CUST_3653_PI442190958	SS120	LLII/III	AAP99743.1_697	CAGACACAAGCAGGAGAAGCAATGAGGAATGATTCTGAATATTCCCATATTGCAGCTTGG	sdhA	Succinate dehydrogenase flavoprotein subunit		AAP99743.1	638919..640841	Krebs cycle and related genes	COG1053
AAP99743.1_697	CUST_3654_PI442190958	SS120	LLII/III	AAP99743.1_697	TATCAAGATGTTCGAATCCCTTACGGAGTTGACTGTCCTAATCCTGAACTTGAGAAGGCA	sdhA	Succinate dehydrogenase flavoprotein subunit		AAP99743.1	638919..640841	Krebs cycle and related genes	COG1053
AAP99743.1_697	CUST_3655_PI442190958	SS120	LLII/III	AAP99743.1_697	GTGGAATCACAAGAAACAAGACAGGTTTAGAAGCTGGACTGAATGAGGTCAGGTTTTTGC	sdhA	Succinate dehydrogenase flavoprotein subunit		AAP99743.1	638919..640841	Krebs cycle and related genes	COG1053
AAP99743.1_697	CUST_3656_PI442190958	SS120	LLII/III	AAP99743.1_697	TGCATGGTGCCAATCGTTTAGGAGCAAATGCCCTTTTACAGTGTTTATCTGATGGTTATT	sdhA	Succinate dehydrogenase flavoprotein subunit		AAP99743.1	638919..640841	Krebs cycle and related genes	COG1053
AAP99743.1_697	CUST_3657_PI442190958	SS120	LLII/III	AAP99743.1_697	AGATGTATGAGCGAATAGTGGGAGAGGACCCGTTTAAAATGCCTATGCGAATTTATCCTG	sdhA	Succinate dehydrogenase flavoprotein subunit		AAP99743.1	638919..640841	Krebs cycle and related genes	COG1053
AAP99743.1_697	CUST_3658_PI442190958	SS120	LLII/III	AAP99743.1_697	GATGGACGCTCTGTTTATCTGGACCTTCGTGATGCAATCCTTAGTAAAGGATCAGATGTG	sdhA	Succinate dehydrogenase flavoprotein subunit		AAP99743.1	638919..640841	Krebs cycle and related genes	COG1053
AAP99744.1_698	CUST_3659_PI442190958	SS120	LLII/III	AAP99744.1_698	GGGTTTCTTATAAATGGACAAGCTCATGGGCCTAACACTGCTACAACTATTTGCCAGTTG	sdhB	Succinate dehydrogenase iron-sulphur protein subunit		AAP99744.1	640838..641578	Krebs cycle and related genes	COG0479
AAP99744.1_698	CUST_3660_PI442190958	SS120	LLII/III	AAP99744.1_698	TCTGCCTTTCAAACAGCAACATGTATTGGTTGTGGAGCTTGTGTAGCAAGTTGTAAGAAT	sdhB	Succinate dehydrogenase iron-sulphur protein subunit		AAP99744.1	640838..641578	Krebs cycle and related genes	COG0479
AAP99744.1_698	CUST_3661_PI442190958	SS120	LLII/III	AAP99744.1_698	AGCTTATTTGTTGCTGCCAAGATTGCTCATTTAGGACAGTTGCCACAAGGACAATTAGAT	sdhB	Succinate dehydrogenase iron-sulphur protein subunit		AAP99744.1	640838..641578	Krebs cycle and related genes	COG0479
AAP99744.1_698	CUST_3662_PI442190958	SS120	LLII/III	AAP99744.1_698	TCAAACCCTAACTCTTGAGCCATGGAGAGCAAAAGCTTTTCAGATAATTCAGGATCTCGT	sdhB	Succinate dehydrogenase iron-sulphur protein subunit		AAP99744.1	640838..641578	Krebs cycle and related genes	COG0479
AAP99744.1_698	CUST_3663_PI442190958	SS120	LLII/III	AAP99744.1_698	GCCAAGACTCGCAGGCTGATAAGGGCAAGTTTGATGAATATGTTTTGGAAAACCTTTCTA	sdhB	Succinate dehydrogenase iron-sulphur protein subunit		AAP99744.1	640838..641578	Krebs cycle and related genes	COG0479
AAP99744.1_698	CUST_3664_PI442190958	SS120	LLII/III	AAP99744.1_698	ATCTTGAATGTGAGGCGGTTTGTCCTAAGGAGATTTCAGCAAGTTGGATTAGTTGGATGA	sdhB	Succinate dehydrogenase iron-sulphur protein subunit		AAP99744.1	640838..641578	Krebs cycle and related genes	COG0479
AAP99810.1_764	CUST_3972_PI442190958	SS120	LLII/III	AAP99810.1_764	TCTAATAATATGGGAATGCTTCGATATACCAGTGGGGGCAACTTCAAAATCCCGACTGTT	pdhB	Pyruvate dehydrogenase E1 component beta subunit		AAP99810.1	695189..696172	Glycolysis/Krebs cycle	COG0022
AAP99810.1_764	CUST_3973_PI442190958	SS120	LLII/III	AAP99810.1_764	CTTGGAGCTGAACATAGCCAGAGGTTGGAAGCTTATTTTCATGCAGTTCCTGGAATTAAA	pdhB	Pyruvate dehydrogenase E1 component beta subunit		AAP99810.1	695189..696172	Glycolysis/Krebs cycle	COG0022
AAP99810.1_764	CUST_3974_PI442190958	SS120	LLII/III	AAP99810.1_764	AGGAGGGATTGGAGCTGAGTTGATGGCTTTAATTAATGAACACTGTTTTGATGATCTGGA	pdhB	Pyruvate dehydrogenase E1 component beta subunit		AAP99810.1	695189..696172	Glycolysis/Krebs cycle	COG0022
AAP99810.1_764	CUST_3975_PI442190958	SS120	LLII/III	AAP99810.1_764	TGCATGTAGTACACCAACCAATGCAAAAGGTTTAATGAAGGCTGCTATAAGAGATGACAA	pdhB	Pyruvate dehydrogenase E1 component beta subunit		AAP99810.1	695189..696172	Glycolysis/Krebs cycle	COG0022
AAP99810.1_764	CUST_3976_PI442190958	SS120	LLII/III	AAP99810.1_764	TCTCACAGAAGAGTTGCCTGAAGGTGATTATTTATGTTCGCTTGATCAAGCTGATCTAGT	pdhB	Pyruvate dehydrogenase E1 component beta subunit		AAP99810.1	695189..696172	Glycolysis/Krebs cycle	COG0022
AAP99810.1_764	CUST_3977_PI442190958	SS120	LLII/III	AAP99810.1_764	GGGTTGCGCCCAATAGTTGAAGGAATGAATATGGGGTTCTTGCTTTTAGCGTTT	pdhB	Pyruvate dehydrogenase E1 component beta subunit		AAP99810.1	695189..696172	Glycolysis/Krebs cycle	COG0022
AAP99886.1_840	CUST_4387_PI442190958	SS120	LLII/III	AAP99886.1_840	AGCCTTCTATGAATCTAATTTGGCTTTAACTCAACAGCCGACGCCTCCTTTCAGTTTCTA	glgC	Glucose-1-phosphate adenylyltransferase		AAP99886.1	768769..770064	Glycogen Metabolism	COG0448
AAP99886.1_840	CUST_4388_PI442190958	SS120	LLII/III	AAP99886.1_840	AGCTGAAGAAAGAATTGCTTTAAGAAAAGGAGGTGGAATTCCTCTTGGAGTAGGTCAGGG	glgC	Glucose-1-phosphate adenylyltransferase		AAP99886.1	768769..770064	Glycogen Metabolism	COG0448
AAP99886.1_840	CUST_4389_PI442190958	SS120	LLII/III	AAP99886.1_840	AGCTGATCAGGGCTTCTATATTCGCAATGGAATAGTTGTTGTTGTTAAAAACGCCAGCAT	glgC	Glucose-1-phosphate adenylyltransferase		AAP99886.1	768769..770064	Glycogen Metabolism	COG0448
AAP99886.1_840	CUST_4390_PI442190958	SS120	LLII/III	AAP99886.1_840	AACCCTCTGGAGAAGCTCTTAAAGCAATGGCTGTAGATACTTCACGTTTTGGACTTTCTC	glgC	Glucose-1-phosphate adenylyltransferase		AAP99886.1	768769..770064	Glycogen Metabolism	COG0448
AAP99886.1_840	CUST_4391_PI442190958	SS120	LLII/III	AAP99886.1_840	AAAGACTTTGGGAAGGAAGTTATCCCAGAAGCCTTAAGTAGGGGTGATGCTCTAAAAAGC	glgC	Glucose-1-phosphate adenylyltransferase		AAP99886.1	768769..770064	Glycogen Metabolism	COG0448
AAP99886.1_840	CUST_4392_PI442190958	SS120	LLII/III	AAP99886.1_840	ACGACCGTCAAAAGGGCGATTTTGGATAAGAATACACGAATTGGAGAGAATGTCACAATC	glgC	Glucose-1-phosphate adenylyltransferase		AAP99886.1	768769..770064	Glycogen Metabolism	COG0448
AAP99887.1_841	CUST_4393_PI442190958	SS120	LLII/III	AAP99887.1_841	AATGGAATGTCTCCAATAATGGATGCGGTTGTTTTGTCTACGATTGCGAGTTATGCACAA	edd	6-Phosphogluconate dehydratase		AAP99887.1	770220..771638	Pentose Phosphate/Entner-Dudorof	COG2084
AAP99887.1_841	CUST_4394_PI442190958	SS120	LLII/III	AAP99887.1_841	TTACGAACTAAGGATCCAACCGACGGAGCTGATCTTGTTGAGAAAATAATGGACAAAGCA	edd	6-Phosphogluconate dehydratase		AAP99887.1	770220..771638	Pentose Phosphate/Entner-Dudorof	COG2084
AAP99887.1_841	CUST_4395_PI442190958	SS120	LLII/III	AAP99887.1_841	TGAGAGAGTCAAGGCGGAAAAAGTATTTGGCAAGCTATCTATCGAAAGAACTGACCTTGG	edd	6-Phosphogluconate dehydratase		AAP99887.1	770220..771638	Pentose Phosphate/Entner-Dudorof	COG2084
AAP99887.1_841	CUST_4396_PI442190958	SS120	LLII/III	AAP99887.1_841	CTTCATATGCCTTCTATTGCTCAAATTTGGAAAGGAGGCTGCATCATACGTTCCACACTC	edd	6-Phosphogluconate dehydratase		AAP99887.1	770220..771638	Pentose Phosphate/Entner-Dudorof	COG2084
AAP99887.1_841	CUST_4397_PI442190958	SS120	LLII/III	AAP99887.1_841	ATCCATGGTTTGCCGAACAAATTAAAAATAGATTGCCAGGGCTTACAAAAACAGTGGCTG	edd	6-Phosphogluconate dehydratase		AAP99887.1	770220..771638	Pentose Phosphate/Entner-Dudorof	COG2084
AAP99887.1_841	CUST_4398_PI442190958	SS120	LLII/III	AAP99887.1_841	TATAGAACTGCTAGGTTGCCACAAAATCTAGTTCAGGCTATGCGTGATTGCTTTGGATCA	edd	6-Phosphogluconate dehydratase		AAP99887.1	770220..771638	Pentose Phosphate/Entner-Dudorof	COG2084
AAP99899.1_853	CUST_4456_PI442190958	SS120	LLII/III	AAP99899.1_853	TAACTGATCCTTCAGAAGCTTCTGATTTTGTTGCAAAAACCAAAGTCGATGCTCTTGCGA	cbbA	Fructose-1,6-bisphosphate aldolase class II		AAP99899.1	779131..780204	Glycolysis/Gluconeogenesis	COG0191
AAP99899.1_853	CUST_4457_PI442190958	SS120	LLII/III	AAP99899.1_853	GGTGCCTATAAATTCACAAGGAAGCCAACCGGTGAAGTTCTTGCAATTAGTAGAATTGCA	cbbA	Fructose-1,6-bisphosphate aldolase class II		AAP99899.1	779131..780204	Glycolysis/Gluconeogenesis	COG0191
AAP99899.1_853	CUST_4458_PI442190958	SS120	LLII/III	AAP99899.1_853	AAAGCAATACCAAACACTCACCTTGTAATGCATGGATCAAGCTCTGTTCCTCAAGAATGG	cbbA	Fructose-1,6-bisphosphate aldolase class II		AAP99899.1	779131..780204	Glycolysis/Gluconeogenesis	COG0191
AAP99899.1_853	CUST_4459_PI442190958	SS120	LLII/III	AAP99899.1_853	GGTAAGGAAAGTAAACATCGACACTGATAACCGTCTTGCATTTACTGCAGCTGTTAGGGA	cbbA	Fructose-1,6-bisphosphate aldolase class II		AAP99899.1	779131..780204	Glycolysis/Gluconeogenesis	COG0191
AAP99899.1_853	CUST_4460_PI442190958	SS120	LLII/III	AAP99899.1_853	CAGGTTTGTCTTGACCGCTATCAACAATTTTGGTGTGCTGGACAGGCAAGTAAGATTAAA	cbbA	Fructose-1,6-bisphosphate aldolase class II		AAP99899.1	779131..780204	Glycolysis/Gluconeogenesis	COG0191
AAP99899.1_853	CUST_4461_PI442190958	SS120	LLII/III	AAP99899.1_853	GCAGCAGCTGATCCAACCAATTTTGATCCAAGGCATTTCAACAAACCAGCTAGAAAATAT	cbbA	Fructose-1,6-bisphosphate aldolase class II		AAP99899.1	779131..780204	Glycolysis/Gluconeogenesis	COG0191
AAP99900.1_854	CUST_4462_PI442190958	SS120	LLII/III	AAP99900.1_854	TTAGATGGTCTTGTGGAAAGGTCTTCTGACTATTACGCACAAGGTGCTCGTTTTGCTAAA	fda	Fructose-1,6-bisphosphate aldolase class I		AAP99900.1	780355..781422	Glycolysis/Gluconeogenesis	COG0191
AAP99900.1_854	CUST_4463_PI442190958	SS120	LLII/III	AAP99900.1_854	TCCATCAAAACTATCTATAAAGGAAAATGCTTGGGGCCTAGCACGTTATGCGAGATCAGT	fda	Fructose-1,6-bisphosphate aldolase class I		AAP99900.1	780355..781422	Glycolysis/Gluconeogenesis	COG0191
AAP99900.1_854	CUST_4464_PI442190958	SS120	LLII/III	AAP99900.1_854	GTAGCAGGGTCTCAACCATCATCTGATGAAGCTCTGTTCGTTGCAGGGTACAAATATTAA	fda	Fructose-1,6-bisphosphate aldolase class I		AAP99900.1	780355..781422	Glycolysis/Gluconeogenesis	COG0191
AAP99900.1_854	CUST_4465_PI442190958	SS120	LLII/III	AAP99900.1_854	CTTCAGCACTCATGTCTTAAAGAATGGGGTGGCAAAAATTTAGAAGCTGGACAGAAAGCT	fda	Fructose-1,6-bisphosphate aldolase class I		AAP99900.1	780355..781422	Glycolysis/Gluconeogenesis	COG0191
AAP99900.1_854	CUST_4466_PI442190958	SS120	LLII/III	AAP99900.1_854	AGAGATACTTATGGATGGTTCTCACAGCATAAACAAAACTGCCGAAGTGCAAGAAGAGGT	fda	Fructose-1,6-bisphosphate aldolase class I		AAP99900.1	780355..781422	Glycolysis/Gluconeogenesis	COG0191
AAP99900.1_854	CUST_4467_PI442190958	SS120	LLII/III	AAP99900.1_854	CCAGGAATTAAAGTTGATAAAGGCTTACGCCCATTAGCGGGTGGTAATGATGTTGAAACA	fda	Fructose-1,6-bisphosphate aldolase class I		AAP99900.1	780355..781422	Glycolysis/Gluconeogenesis	COG0191
AAP99905.1_859	CUST_4491_PI442190958	SS120	LLII/III	AAP99905.1_859	AGTCCTATGAAAGAAGCAATGGCTGAGGCTCTTGCAAAAGGGGAAAATTTCTCTCATTTT	prk	Phosphoribulokinase		AAP99905.1	784660..785559	Carbon Fixation	COG3954
AAP99905.1_859	CUST_4492_PI442190958	SS120	LLII/III	AAP99905.1_859	CGGTCAGTTCACTCCATGGGAAGAAATTCCCAAAGGTACTGATGTACTTTTCTACGAAGG	prk	Phosphoribulokinase		AAP99905.1	784660..785559	Carbon Fixation	COG3954
AAP99905.1_859	CUST_4493_PI442190958	SS120	LLII/III	AAP99905.1_859	TTTGACAAGCTCGAGGAACTCTTCCAAACATATGGACAAAATGGAGGTGGCAAAAAAAGA	prk	Phosphoribulokinase		AAP99905.1	784660..785559	Carbon Fixation	COG3954
AAP99905.1_859	CUST_4494_PI442190958	SS120	LLII/III	AAP99905.1_859	TGTCAAGTCCAACAAGTATTGTTGTAAATGGAGGCAAAATGGGTTTTGCTATGGAGCTAA	prk	Phosphoribulokinase		AAP99905.1	784660..785559	Carbon Fixation	COG3954
AAP99905.1_859	CUST_4495_PI442190958	SS120	LLII/III	AAP99905.1_859	TGCTGAAAGGGGATATTCAGCAGAAACAATTGTAGAAACAATCTTGAGAAGAATGCCTGA	prk	Phosphoribulokinase		AAP99905.1	784660..785559	Carbon Fixation	COG3954
AAP99905.1_859	CUST_4496_PI442190958	SS120	LLII/III	AAP99905.1_859	TGCCCTCAATTTAGTCGTACTGATATAAACTTTCAACGAGTTCCAACTATTGACACCTCA	prk	Phosphoribulokinase		AAP99905.1	784660..785559	Carbon Fixation	COG3954
AAP99967.1_921	CUST_4814_PI442190958	SS120	LLII/III	AAP99967.1_921	GCAGGCAATGGACATGAAATTATTAAAGCCCGGAGATCAAGTAGTAGAAACAGCAGGAAC	pykF	Pyruvate kinase		AAP99967.1	839129..840913	Glycolysis	COG0469
AAP99967.1_921	CUST_4815_PI442190958	SS120	LLII/III	AAP99967.1_921	GTATGGGGTGTATCTCCCTTGTTGATAGATGCTCAAAAAAGCACAAGCAAAACCTTTGGA	pykF	Pyruvate kinase		AAP99967.1	839129..840913	Glycolysis	COG0469
AAP99967.1_921	CUST_4816_PI442190958	SS120	LLII/III	AAP99967.1_921	ACTTCCCCTAACTAAAAGTGGTGCAACTGCTCATAATGTGAGTAAGTTTCGTCCTGCTAC	pykF	Pyruvate kinase		AAP99967.1	839129..840913	Glycolysis	COG0469
AAP99967.1_921	CUST_4817_PI442190958	SS120	LLII/III	AAP99967.1_921	TCCTAATGCAATTAGTGCCGCAGTAAGTACTATTGCAAGACAATTAAACGCAGCAGCAAT	pykF	Pyruvate kinase		AAP99967.1	839129..840913	Glycolysis	COG0469
AAP99967.1_921	CUST_4818_PI442190958	SS120	LLII/III	AAP99967.1_921	CCATGGCAAAAATAGCAAGAAGAATCGAGAGGGATTACCCACAAAGAGCTTTAGAAAGCC	pykF	Pyruvate kinase		AAP99967.1	839129..840913	Glycolysis	COG0469
AAP99967.1_921	CUST_4819_PI442190958	SS120	LLII/III	AAP99967.1_921	GATGCAGTAATGCTTTCTAACGAAACAGCAGTGGGAGATTATCCAATAGAAGCTGTCGAA	pykF	Pyruvate kinase		AAP99967.1	839129..840913	Glycolysis	COG0469
AAP99990.1_944	CUST_4935_PI442190958	SS120	LLII/III	AAP99990.1_944	TATATATGCAGAACTTATTGATATCAATGCATATCATCAACCGGGCGTAGAGGCCGGCAA	pgi	Glucose-6-phosphate isomerase		AAP99990.1	860990..862591	Glycolysis/Gluconeogenesis	COG0166
AAP99990.1_944	CUST_4936_PI442190958	SS120	LLII/III	AAP99990.1_944	TCGGGTTTTTTGCAAGGCACTAGGTTAGCTCTATCAGAAAACGATCGTCAAAGTATAACT	pgi	Glucose-6-phosphate isomerase		AAP99990.1	860990..862591	Glycolysis/Gluconeogenesis	COG0166
AAP99990.1_944	CUST_4937_PI442190958	SS120	LLII/III	AAP99990.1_944	TGGAAATAAAGGATCTACAGATCAACATGCTTATGTACAACAACTAAGGGATGGAATTGA	pgi	Glucose-6-phosphate isomerase		AAP99990.1	860990..862591	Glycolysis/Gluconeogenesis	COG0166
AAP99990.1_944	CUST_4938_PI442190958	SS120	LLII/III	AAP99990.1_944	GGTTGTACTTCCTTATCAGGATAGGCTTCAGGTTTTCAGTAAATATCTTCAACAACTTGT	pgi	Glucose-6-phosphate isomerase		AAP99990.1	860990..862591	Glycolysis/Gluconeogenesis	COG0166
AAP99990.1_944	CUST_4939_PI442190958	SS120	LLII/III	AAP99990.1_944	TCCATCTGCATTACTTGCTGCATCATGGTATTTTTCTGGAGCTGGTAAAGGAAAGAGAGA	pgi	Glucose-6-phosphate isomerase		AAP99990.1	860990..862591	Glycolysis/Gluconeogenesis	COG0166
AAP99990.1_944	CUST_4940_PI442190958	SS120	LLII/III	AAP99990.1_944	TGGAGAAGATATAGACAGTTTTCTTGATGGAGCCTCGCAAATGGATCAAATAACACGACG	pgi	Glucose-6-phosphate isomerase		AAP99990.1	860990..862591	Glycolysis/Gluconeogenesis	COG0166
AAQ00083.1_1036	CUST_5426_PI442190958	SS120	LLII/III	AAQ00083.1_1036	TAGAAAAGCATCATCACGAAGTTGCTGGCCCAGGACAACATGAGTTAGGAATGAAATTTG	glnA	Glutamine synthetase/ glutamate--ammonialigase		AAQ00083.1	952441..953862	Nitrogen Metabolism	COG0174
AAQ00083.1_1036	CUST_5427_PI442190958	SS120	LLII/III	AAQ00083.1_1036	ACTTTCATGCCAAAACCTGTATGGAACGATAACGGTACAGGTATGCATGTTCATCAAAGC	glnA	Glutamine synthetase/ glutamate--ammonialigase		AAQ00083.1	952441..953862	Nitrogen Metabolism	COG0174
AAQ00083.1_1036	CUST_5428_PI442190958	SS120	LLII/III	AAQ00083.1_1036	TTGAGTTCAGGTCAGGAGATGCTCTTGCAAATCCATACTTAGCCTTTAGTGCAATGATGA	glnA	Glutamine synthetase/ glutamate--ammonialigase		AAQ00083.1	952441..953862	Nitrogen Metabolism	COG0174
AAQ00083.1_1036	CUST_5429_PI442190958	SS120	LLII/III	AAQ00083.1_1036	CCAACCAATGTTCTATGGGGAAGGAACATATGCAAATCTCTCCCAAACAGCAAAATGGTA	glnA	Glutamine synthetase/ glutamate--ammonialigase		AAQ00083.1	952441..953862	Nitrogen Metabolism	COG0174
AAQ00083.1_1036	CUST_5430_PI442190958	SS120	LLII/III	AAQ00083.1_1036	AGAAATGAAGTACGAAGAAGTCCAACAACTCCGTCAAAGGCCTCATCCTCATGAATTCTT	glnA	Glutamine synthetase/ glutamate--ammonialigase		AAQ00083.1	952441..953862	Nitrogen Metabolism	COG0174
AAQ00083.1_1036	CUST_5431_PI442190958	SS120	LLII/III	AAQ00083.1_1036	GCCTTTACAAATCCAACAACAAATAGTTATAAGCGTCTTGTTCCTGGCTTCGAAGCTCCA	glnA	Glutamine synthetase/ glutamate--ammonialigase		AAQ00083.1	952441..953862	Nitrogen Metabolism	COG0174
AAQ00110.1_1063	CUST_5584_PI442190958	SS120	LLII/III	AAQ00110.1_1063	AACTACCAATAATGGTCTTAATAGACCCAGAGGCTGGGCTATTTGGAGCTGCATGTAAAG	glk	Glucokinase		AAQ00110.1	983438..984484	Glycolysis	COG0837
AAQ00110.1_1063	CUST_5585_PI442190958	SS120	LLII/III	AAQ00110.1_1063	TTCCATTTTTGCAATTCTTGGAGCTGGTACAGGTCTTGGTATGGCAAGAGGCATAATAAC	glk	Glucokinase		AAQ00110.1	983438..984484	Glycolysis	COG0837
AAQ00110.1_1063	CUST_5586_PI442190958	SS120	LLII/III	AAQ00110.1_1063	TACAAGAACTATCTACAGAAGGGCTTTGGATTGGAGGTGGAACGGCGATAAAGCATTTAG	glk	Glucokinase		AAQ00110.1	983438..984484	Glycolysis	COG0837
AAQ00110.1_1063	CUST_5587_PI442190958	SS120	LLII/III	AAQ00110.1_1063	GCGGACATAGAGAATTCTCCCCTCGAAATAATAAGGAATGGGAACTATGCCAATGGCTTA	glk	Glucokinase		AAQ00110.1	983438..984484	Glycolysis	COG0837
AAQ00110.1_1063	CUST_5588_PI442190958	SS120	LLII/III	AAQ00110.1_1063	TGAAAGAGTATCAATCGCAGCCAAAGGAGGCGATCCAATTATGCGAGAAGTTCTAGAAAT	glk	Glucokinase		AAQ00110.1	983438..984484	Glycolysis	COG0837
AAQ00110.1_1063	CUST_5589_PI442190958	SS120	LLII/III	AAQ00110.1_1063	AAGAGTAGTAAGTGGAACTGGTCTTGCAAATATTGCTCGTTGGAGATTAAGCCAAGAGGA	glk	Glucokinase		AAQ00110.1	983438..984484	Glycolysis	COG0837
AAQ00169.1_1122	CUST_5922_PI442190958	SS120	LLII/III	AAQ00169.1_1122	ATGGAACCACCTGGTCATTTTGATCCAGAAGCCATTAGGAATGAAAAAGCCAAAGTACTG	zwf	glucose-6-phosphate dehydrogenase zwf		AAQ00169.1	1051431..1052951	Pentose Phosphate	COG0364
AAQ00169.1_1122	CUST_5923_PI442190958	SS120	LLII/III	AAQ00169.1_1122	CCTAAGAATCCAACCTGATGAAGGAGCTGAGTTCAAGTTTGATGTGAAGTCTCCAGGTTC	zwf	glucose-6-phosphate dehydrogenase zwf		AAQ00169.1	1051431..1052951	Pentose Phosphate	COG0364
AAQ00169.1_1122	CUST_5924_PI442190958	SS120	LLII/III	AAQ00169.1_1122	ATTGGCGATGGCAAGGAGTCCCTTTTTATATTCGTACTGGGAAAAGATTGGCAAAAGAA	zwf	glucose-6-phosphate dehydrogenase zwf		AAQ00169.1	1051431..1052951	Pentose Phosphate	COG0364
AAQ00169.1_1122	CUST_5925_PI442190958	SS120	LLII/III	AAQ00169.1_1122	AGGTGGCTCTAAGAAAAGACCATTGATTGGTTACCGAGATGAACCTGGTGTTAATCCAAA	zwf	glucose-6-phosphate dehydrogenase zwf		AAQ00169.1	1051431..1052951	Pentose Phosphate	COG0364
AAQ00169.1_1122	CUST_5926_PI442190958	SS120	LLII/III	AAQ00169.1_1122	TTCCAACTTATCCTTATGAATCCAGAACTTGGGGCCCTTCAGAAGCTGATGCATTACTAG	zwf	glucose-6-phosphate dehydrogenase zwf		AAQ00169.1	1051431..1052951	Pentose Phosphate	COG0364
AAQ00169.1_1122	CUST_5927_PI442190958	SS120	LLII/III	AAQ00169.1_1122	TATGTAAGGCTTTTAGCTGATGCAATGCTTGGAGATCCTACTCTTTTCACGAGAAGCGAT	zwf	glucose-6-phosphate dehydrogenase zwf		AAQ00169.1	1051431..1052951	Pentose Phosphate	COG0364
AAQ00170.1_1123	CUST_5928_PI442190958	SS120	LLII/III	AAQ00170.1_1123	CCAACACAAAACAACCAAGTTGAAACTGATGTAGCACTTCTACTTGAAAGCAGCAGAGGT	opcA	Glucose-6-P dehydrogenase subunit opcA		AAQ00170.1	1052991..1054304	Pentose Phosphate	COG3429
AAQ00170.1_1123	CUST_5929_PI442190958	SS120	LLII/III	AAQ00170.1_1123	GAAGACCCATCTCTCTTAAATCAACTGGCTTTGCCTGCTAGAAGGATCATCATTGACAGT	opcA	Glucose-6-P dehydrogenase subunit opcA		AAQ00170.1	1052991..1054304	Pentose Phosphate	COG3429
AAQ00170.1_1123	CUST_5930_PI442190958	SS120	LLII/III	AAQ00170.1_1123	AACATGTCATTCAAGGATTACTTTTAGCCTCTTGGATAGCCGATAGACTGGGGTGGAAAC	opcA	Glucose-6-P dehydrogenase subunit opcA		AAQ00170.1	1052991..1054304	Pentose Phosphate	COG3429
AAQ00170.1_1123	CUST_5931_PI442190958	SS120	LLII/III	AAQ00170.1_1123	GATCTCAATTGGCTAAGGCTAAGAACTTGGAGAGAAACATTAGCAATGGTCTTCGACCCA	opcA	Glucose-6-P dehydrogenase subunit opcA		AAQ00170.1	1052991..1054304	Pentose Phosphate	COG3429
AAQ00170.1_1123	CUST_5932_PI442190958	SS120	LLII/III	AAQ00170.1_1123	CGTGAACTTTCGATTAATGCAATTACCTGTAGGGAATCCAAGCATTCACCCTGGTCAAAT	opcA	Glucose-6-P dehydrogenase subunit opcA		AAQ00170.1	1052991..1054304	Pentose Phosphate	COG3429
AAQ00170.1_1123	CUST_5933_PI442190958	SS120	LLII/III	AAQ00170.1_1123	CTGTAAACCAGAGGAAGCTCATAAAACAGGTCTATGCGTAATACTTGCATCGGAGTCTGG	opcA	Glucose-6-P dehydrogenase subunit opcA		AAQ00170.1	1052991..1054304	Pentose Phosphate	COG3429

AAQ00202.1_1155	CUST_6105_PI442190958	SS120	LLII/III	AAQ00202.1_1155	TCATTTGGCATCACTATGCATACAGCTTGGCGAAATAAACCCACTAGAAGAGCTCTAAGT	rbsK	Sugar kinase, ribokinase family		AAQ00202.1	1086518..1087342	Pentose Phosphate	COG0524
AAQ00202.1_1155	CUST_6106_PI442190958	SS120	LLII/III	AAQ00202.1_1155	AGTTTTGCTGCTGGCCTAACATATGGAATGGCAGCAAACATGGATATTCAAAAAGCAATT	rbsK	Sugar kinase, ribokinase family		AAQ00202.1	1086518..1087342	Pentose Phosphate	COG0524
AAQ00202.1_1155	CUST_6107_PI442190958	SS120	LLII/III	AAQ00202.1_1155	ATATCGAATGGGTGTCCTTCCTTTCCGTTGACAAAATGCCTAGACAGGGCTTGATAAATA	rbsK	Sugar kinase, ribokinase family		AAQ00202.1	1086518..1087342	Pentose Phosphate	COG0524
AAQ00202.1_1155	CUST_6108_PI442190958	SS120	LLII/III	AAQ00202.1_1155	GGCTGCAGCCTCAAGGAATGGATGATTTACCCTGGTCCATATTAAAAGAATTTGATGGAG	rbsK	Sugar kinase, ribokinase family		AAQ00202.1	1086518..1087342	Pentose Phosphate	COG0524
AAQ00202.1_1155	CUST_6109_PI442190958	SS120	LLII/III	AAQ00202.1_1155	ACTGCTGCTGACAGTAAAGCTATTCAATTTTGCAGAGAAGCAAAGATTCTTTGCGCTACT	rbsK	Sugar kinase, ribokinase family		AAQ00202.1	1086518..1087342	Pentose Phosphate	COG0524
AAQ00202.1_1155	CUST_6110_PI442190958	SS120	LLII/III	AAQ00202.1_1155	AGTCGCAGCAGTACAAATGTCAAAACTAACTGATGTGCCTGTTCACTTCTTCACTTCTCT	rbsK	Sugar kinase, ribokinase family		AAQ00202.1	1086518..1087342	Pentose Phosphate	COG0524
CAE18471.1_12	CUST_67_PI442190954	MED4	HLI	CAE18471.1_12	ACCTCAATCCTATGAACGTAGTCAAGTCCAGAAATAGTTTAGGTGGAACAGGATTCGATC	argH	Argininosuccinate lyase	PMM0012	CAE18471.1	14585..15964	Amino Acid metabolism	COG0165
AAP99058.1_12	CUST_67_PI442190958	SS120	LLII/III	AAP99058.1_12	TGCCTCAGAAAAAGAATCCTGATGTACCTGAATTAGTAAGAGGTAAAACGGGCCGTGTTT	argH	Argininosuccinate lyase		AAP99058.1	14770..16158	Amino Acid metabolism	COG0165
CAE18471.1_12	CUST_68_PI442190954	MED4	HLI	CAE18471.1_12	AGCAGATTATCTAGTATTTAAGAAGGTTCCTTTCAGAGAGGCATATCAAGTGGTTGGAGA	argH	Argininosuccinate lyase	PMM0012	CAE18471.1	14585..15964	Amino Acid metabolism	COG0165
AAP99058.1_12	CUST_68_PI442190958	SS120	LLII/III	AAP99058.1_12	CTGAAAAAGTCGTAGCAGCTAGGATTAGTGATGGTGGAACAGGTTTTGATCGAGTTCGAG	argH	Argininosuccinate lyase		AAP99058.1	14770..16158	Amino Acid metabolism	COG0165
AAQ00356.1_1309	CUST_6858_PI442190958	SS120	LLII/III	AAQ00356.1_1309	CATTGTGTGAGTCTACAGGGGCAGATGTTAATGATGTAGCTCTTGCTATTGGCATGGATA	ugd	UDP-glucose 6-dehydrogenase/nucleotide sugar dehydrogenase		AAQ00356.1	1212167..1213582	Amino sugar and nucleotide sugar r	COG1004
AAQ00356.1_1309	CUST_6859_PI442190958	SS120	LLII/III	AAQ00356.1_1309	CTTGACTGGGAGAAACTTTCAAAAATAATGCGGTCTCCATCCTGGCTGTTTGATACAAGG	ugd	UDP-glucose 6-dehydrogenase/nucleotide sugar dehydrogenase		AAQ00356.1	1212167..1213582	Amino sugar and nucleotide sugar r	COG1004
AAQ00356.1_1309	CUST_6860_PI442190958	SS120	LLII/III	AAQ00356.1_1309	TCTTTTATTTGAGGGTAAATGGGAATTTTCTAAATCGGTTGAATTGGCAGCAAACGGCGC	ugd	UDP-glucose 6-dehydrogenase/nucleotide sugar dehydrogenase		AAQ00356.1	1212167..1213582	Amino sugar and nucleotide sugar r	COG1004
AAQ00356.1_1309	CUST_6861_PI442190958	SS120	LLII/III	AAQ00356.1_1309	TCTCAGACCAGGCCCAGGATTTGGGGGAAGTTGCTTTAAGAAAGATATATTAAACTTGGT	ugd	UDP-glucose 6-dehydrogenase/nucleotide sugar dehydrogenase		AAQ00356.1	1212167..1213582	Amino sugar and nucleotide sugar r	COG1004
AAQ00356.1_1309	CUST_6862_PI442190958	SS120	LLII/III	AAQ00356.1_1309	GCAATTGAGGCTAAATCGTTCGGGATTAATATCTGGAGACTTGGCGATGGAGCTCTAAAT	ugd	UDP-glucose 6-dehydrogenase/nucleotide sugar dehydrogenase		AAQ00356.1	1212167..1213582	Amino sugar and nucleotide sugar r	COG1004
AAQ00356.1_1309	CUST_6863_PI442190958	SS120	LLII/III	AAQ00356.1_1309	AGCTGTGCTTGGCTTTGCCTTTAAAGCAAATACTAATGATACTAGAGAATCTCCTGCGAT	ugd	UDP-glucose 6-dehydrogenase/nucleotide sugar dehydrogenase		AAQ00356.1	1212167..1213582	Amino sugar and nucleotide sugar r	COG1004
AAQ00358.1_1311	CUST_6870_PI442190958	SS120	LLII/III	AAQ00358.1_1311	TATGTGCATGTTTTAGACTTGGCAGAAGCCCACAAGTCTGCATTAGAATGCTTATTTGCT	galE	UDP-glucose 4-epimerase		AAQ00358.1	1214631..1215674	Galactose metabolism	COG1087
AAQ00358.1_1311	CUST_6871_PI442190958	SS120	LLII/III	AAQ00358.1_1311	AACAAACGTAGCTGGAGGACAAATCAAGCAAGTAGAGGTTTTTGGAAATGATTGGCCTAC	galE	UDP-glucose 4-epimerase		AAQ00358.1	1214631..1215674	Galactose metabolism	COG1087
AAQ00358.1_1311	CUST_6872_PI442190958	SS120	LLII/III	AAQ00358.1_1311	GTGCTCCTTTTGATTGGAGAATAGCTTGTCTTAGATATTTTAATCCTGTTGGGGCGCACC	galE	UDP-glucose 4-epimerase		AAQ00358.1	1214631..1215674	Galactose metabolism	COG1087
AAQ00358.1_1311	CUST_6873_PI442190958	SS120	LLII/III	AAQ00358.1_1311	GCACGCCTTAATTGGTATCCTAAGAGGTCTATAGAAGATATGTGTCGTGACACGTGGAGA	galE	UDP-glucose 4-epimerase		AAQ00358.1	1214631..1215674	Galactose metabolism	COG1087
AAQ00358.1_1311	CUST_6874_PI442190958	SS120	LLII/III	AAQ00358.1_1311	ACATTCTCGAGAGTCAATAACTGTGAAGTTCCTTATGTTTTTGCTGCAAGGAGACCAGGA	galE	UDP-glucose 4-epimerase		AAQ00358.1	1214631..1215674	Galactose metabolism	COG1087
AAQ00358.1_1311	CUST_6875_PI442190958	SS120	LLII/III	AAQ00358.1_1311	GAACCTGCGCAATTACTTATTCTTAATTTGGGGAATGGTTTTGGACTTAGCGTGTTGGAA	galE	UDP-glucose 4-epimerase		AAQ00358.1	1214631..1215674	Galactose metabolism	COG1087
CAE18471.1_12	CUST_69_PI442190954	MED4	HLI	CAE18471.1_12	AGGACTTCCAAGAGGATAAAGAGCCAATTTTTGACACAGTAGATACTATTTCTTCTTGCT	argH	Argininosuccinate lyase	PMM0012	CAE18471.1	14585..15964	Amino Acid metabolism	COG0165
AAP99058.1_12	CUST_69_PI442190958	SS120	LLII/III	AAP99058.1_12	TATTCCTTTTCGAGAGGCATATCAAATTGTTGGACGCGTTGTTAGGCTTTGCATTCAGAA	argH	Argininosuccinate lyase		AAP99058.1	14770..16158	Amino Acid metabolism	COG0165
CAE18471.1_12	CUST_70_PI442190954	MED4	HLI	CAE18471.1_12	ACGGGTCGAGTTTATGGACATCTTCAATCTTTATTAACCATGATAAAGGGAGTGCCTCTT	argH	Argininosuccinate lyase	PMM0012	CAE18471.1	14585..15964	Amino Acid metabolism	COG0165
AAP99058.1_12	CUST_70_PI442190958	SS120	LLII/III	AAP99058.1_12	TCTTGAACGTTTGAAAGAGACAGTTGAAGCCGATTTTTCTAATGCTACTGATGTTGCAGA	argH	Argininosuccinate lyase		AAP99058.1	14770..16158	Amino Acid metabolism	COG0165
CAE18471.1_12	CUST_71_PI442190954	MED4	HLI	CAE18471.1_12	CTGATAAGTGTGCGACTGGAAGTAGCTTAATGCCTCAGAAGAAGAATCCAGATGTTCCTG	argH	Argininosuccinate lyase	PMM0012	CAE18471.1	14585..15964	Amino Acid metabolism	COG0165
AAP99058.1_12	CUST_71_PI442190958	SS120	LLII/III	AAP99058.1_12	TGGACATTTGCAAAGTCTCTTGACCATGATGAAAGGCCTTCCTTTAGCTTATAACAAAGA	argH	Argininosuccinate lyase		AAP99058.1	14770..16158	Amino Acid metabolism	COG0165
AAQ00406.1_1359	CUST_7145_PI442190958	SS120	LLII/III	AAQ00406.1_1359	GTTGCAGTAAGGGGAGAGGAGGTTCCATGCACCTTTTTTCTAAAGAACACCATTTATTGG	pdhA	Pyruvate dehydrogenase E1 component alpha subunit		AAQ00406.1	1263536..1264630	Glycolysis/Krebs cycle	COG1071
AAQ00406.1_1359	CUST_7146_PI442190958	SS120	LLII/III	AAQ00406.1_1359	ATTTGGAGGAAAGCAAATGCATTTGGTATGAAAGGAGAAGAAGTTGACGGGATGGATGTT	pdhA	Pyruvate dehydrogenase E1 component alpha subunit		AAQ00406.1	1263536..1264630	Glycolysis/Krebs cycle	COG1071
AAQ00406.1_1359	CUST_7147_PI442190958	SS120	LLII/III	AAQ00406.1_1359	GAAATTGATCAGGAAGTTGCAGATGCGGTTGAATTTGCAATTGGATCAGCTGATCCAAAA	pdhA	Pyruvate dehydrogenase E1 component alpha subunit		AAQ00406.1	1263536..1264630	Glycolysis/Krebs cycle	COG1071
AAQ00406.1_1359	CUST_7148_PI442190958	SS120	LLII/III	AAQ00406.1_1359	GATTTAGAGGCCATTCACTGGCAGATCCCGATGAACTTAGATCAGCAGAAGAAAAAGACT	pdhA	Pyruvate dehydrogenase E1 component alpha subunit		AAQ00406.1	1263536..1264630	Glycolysis/Krebs cycle	COG1071
AAQ00406.1_1359	CUST_7149_PI442190958	SS120	LLII/III	AAQ00406.1_1359	TCTGTTACAGCAGCGTTTTTTGGTGACGGTACATGCAATAACGGTCAATTTTATGAGTGT	pdhA	Pyruvate dehydrogenase E1 component alpha subunit		AAQ00406.1	1263536..1264630	Glycolysis/Krebs cycle	COG1071
AAQ00406.1_1359	CUST_7150_PI442190958	SS120	LLII/III	AAQ00406.1_1359	TTTGTTGTTGAAAACAACAAATGGGCTATTGGGATGGCTCATGATCGAGCAACTAGTGAT	pdhA	Pyruvate dehydrogenase E1 component alpha subunit		AAQ00406.1	1263536..1264630	Glycolysis/Krebs cycle	COG1071
CAE18471.1_12	CUST_72_PI442190954	MED4	HLI	CAE18471.1_12	TAGGGTTAATATTTCGCCCTTAGGCGCAGCAGCTTTAGCGGGGACTAAAATTAAAATAGA	argH	Argininosuccinate lyase	PMM0012	CAE18471.1	14585..15964	Amino Acid metabolism	COG0165
AAP99058.1_12	CUST_72_PI442190958	SS120	LLII/III	AAP99058.1_12	ACTGTTGAATTTACAGCTGCGGCATCTTTAATAATGACGCATATAAGTCGTTTAGCTGAT	argH	Argininosuccinate lyase		AAP99058.1	14770..16158	Amino Acid metabolism	COG0165
AAQ00427.1_1380	CUST_7268_PI442190958	SS120	LLII/III	AAQ00427.1_1380	AAGCTCTTGAAGGGGAAGTTCAAGTAACTGTAATTGCAACTGGTTTTGATGGGAATCAGC	ftsZ	cell division protein FtsZ		AAQ00427.1	1286755..1287870	Cell Division	COG0206
AAQ00427.1_1380	CUST_7269_PI442190958	SS120	LLII/III	AAQ00427.1_1380	AATGACTGAAGCTGGAACATCATTACTTGGAATAGGCATTGGATCTGGTCGTTCTAGAGC	ftsZ	cell division protein FtsZ		AAQ00427.1	1286755..1287870	Cell Division	COG0206
AAQ00427.1_1380	CUST_7270_PI442190958	SS120	LLII/III	AAQ00427.1_1380	CAGGCGGGAAAGACATGACATTAGAAGACATGACCTCTGCTTCAGAGGTTATTTACGATG	ftsZ	cell division protein FtsZ		AAQ00427.1	1286755..1287870	Cell Division	COG0206
AAQ00427.1_1380	CUST_7271_PI442190958	SS120	LLII/III	AAQ00427.1_1380	ATGCAGATGATGTTTTACGAATGGGAGTGAAAGGCATAACCGACATAATCACTTTGCCTG	ftsZ	cell division protein FtsZ		AAQ00427.1	1286755..1287870	Cell Division	COG0206
AAQ00427.1_1380	CUST_7272_PI442190958	SS120	LLII/III	AAQ00427.1_1380	ATCGACAAACACCAAACAAAGAGCCTGGAGCAAGTATCCCTGAATTCCTAAGACTTAGAC	ftsZ	cell division protein FtsZ		AAQ00427.1	1286755..1287870	Cell Division	COG0206
AAQ00427.1_1380	CUST_7273_PI442190958	SS120	LLII/III	AAQ00427.1_1380	AAGTGTTGACACTTTAATTGTCATCCCTAACGATCGCCTTAAAGATGCAATTGCAGGAGC	ftsZ	cell division protein FtsZ		AAQ00427.1	1286755..1287870	Cell Division	COG0206
AAQ00448.1_1401	CUST_7376_PI442190958	SS120	LLII/III	AAQ00448.1_1401	TCTTGTTAATTGCATGGAGCAATCAGATACTGCAATAACAACCATCCGAATCTGCATGGG	glcH	GPH family sugar transporter		AAQ00448.1	1304392..1305750	GlcH sugar transporter	COG2211
AAQ00448.1_1401	CUST_7377_PI442190958	SS120	LLII/III	AAQ00448.1_1401	CCAGTTGCTTGGTCTTTTGCTTTCGTTGTCAGGTTATCGATCATCTAATGATTGCGTTGA	glcH	GPH family sugar transporter		AAQ00448.1	1304392..1305750	GlcH sugar transporter	COG2211
AAQ00448.1_1401	CUST_7378_PI442190958	SS120	LLII/III	AAQ00448.1_1401	CCAGATGCTATAGACGCTGATCCAGATAAACCTGCAGGAATTTATACAGCTTGGATGGTC	glcH	GPH family sugar transporter		AAQ00448.1	1304392..1305750	GlcH sugar transporter	COG2211
AAQ00448.1_1401	CUST_7379_PI442190958	SS120	LLII/III	AAQ00448.1_1401	AATTCTGGAGTCTTTACTCAAACAAGAATGGCCGCATTAAGGCACTTTTCAAGGGTTCAT	glcH	GPH family sugar transporter		AAQ00448.1	1304392..1305750	GlcH sugar transporter	COG2211
AAQ00448.1_1401	CUST_7380_PI442190958	SS120	LLII/III	AAQ00448.1_1401	ATCATTGGTCTTTATTTGCTTCTCTGGTGTGGGCTCCAATTAATGCAAACAGTTTCGCTA	glcH	GPH family sugar transporter		AAQ00448.1	1304392..1305750	GlcH sugar transporter	COG2211
AAQ00448.1_1401	CUST_7381_PI442190958	SS120	LLII/III	AAQ00448.1_1401	TGCTGGGGTCTCTCTCCATTTGCAAAGAAAGCAAGAAAACCAATAATTACTTCTGAACCG	glcH	GPH family sugar transporter		AAQ00448.1	1304392..1305750	GlcH sugar transporter	COG2211
AAQ00467.1_1420	CUST_7488_PI442190958	SS120	LLII/III	AAQ00467.1_1420	TTCGAATCAGTTCAGGTGTGCCTGATTTAGATGATATGTGTGGTGGAGGGTATTTTCAGG	kaiC	circadian clock protein KaiC		AAQ00467.1	1324116..1325621	Circadian Rhythm	COG0467
AAQ00467.1_1420	CUST_7489_PI442190958	SS120	LLII/III	AAQ00467.1_1420	CGCAATCAAGAAAGAGCCATTATTTTTGCTTACGAAGAGTCAAGGGCTCAGCTTCTTAGG	kaiC	circadian clock protein KaiC		AAQ00467.1	1324116..1325621	Circadian Rhythm	COG0467
AAQ00467.1_1420	CUST_7490_PI442190958	SS120	LLII/III	AAQ00467.1_1420	TAAGAGGAGAAATGGCTCGAGCTATTAATGTGTTTAAGATGCGTGGATCTTGGCATGACA	kaiC	circadian clock protein KaiC		AAQ00467.1	1324116..1325621	Circadian Rhythm	COG0467
AAQ00467.1_1420	CUST_7491_PI442190958	SS120	LLII/III	AAQ00467.1_1420	GCGACCAGTTGGGGGATTGATTTTGAACAGATGGAAGCTGATGGGTTATTAAAGATCATT	kaiC	circadian clock protein KaiC		AAQ00467.1	1324116..1325621	Circadian Rhythm	COG0467
AAQ00467.1_1420	CUST_7492_PI442190958	SS120	LLII/III	AAQ00467.1_1420	TTGAGCAAATTTTTAGCGGTGCTCCCCATCGTATTAGTGGTGAGGATTCTATATCAGGTG	kaiC	circadian clock protein KaiC		AAQ00467.1	1324116..1325621	Circadian Rhythm	COG0467
AAQ00467.1_1420	CUST_7493_PI442190958	SS120	LLII/III	AAQ00467.1_1420	ATAACAGAATTTAAACCATCTCGAATGGCTATTGACTCTCTTTCTGCGCTAGCTCGTGGG	kaiC	circadian clock protein KaiC		AAQ00467.1	1324116..1325621	Circadian Rhythm	COG0467
AAQ00468.1_1421	CUST_7494_PI442190958	SS120	LLII/III	AAQ00468.1_1421	AATTGTCTGAAAGTGATTTCTTTTCTGGATCTCCTGACTCCGAATTCAGCTCCGATGAGG	kaiB	Circadian clock protein KaiB		AAQ00468.1	1325722..1326075	Circadian Rhythm	COG4251
AAQ00468.1_1421	CUST_7495_PI442190958	SS120	LLII/III	AAQ00468.1_1421	CCCGTCAGAAGAATAATTGGAGACCTTTCTGATCGTGAGCGAGTATTGATTGGTTTGGAT	kaiB	Circadian clock protein KaiB		AAQ00468.1	1325722..1326075	Circadian Rhythm	COG4251
AAQ00468.1_1421	CUST_7496_PI442190958	SS120	LLII/III	AAQ00468.1_1421	CTTAAAAACCCACAGCTAGCAGAGGAAGATAAGATACTTGCAACCCCAACTTTGGCAAAA	kaiB	Circadian clock protein KaiB		AAQ00468.1	1325722..1326075	Circadian Rhythm	COG4251
AAQ00468.1_1421	CUST_7497_PI442190958	SS120	LLII/III	AAQ00468.1_1421	TGTTGCTGGGAATACTCCTAATTCCATGAGAGCTTTGACTACATTGAGAGAAATTTTGCA	kaiB	Circadian clock protein KaiB		AAQ00468.1	1325722..1326075	Circadian Rhythm	COG4251
AAQ00636.1_1589	CUST_8222_PI442190958	SS120	LLII/III	AAQ00636.1_1589	TCACCGCCATTGATATAGGAGTGCTAAAACTTCGTAGCAGTAATGGAAATTGGGATTCCA	atpC	ATP synthase epsilon subunit		AAQ00636.1	1449179..1449589	Energy Metabolism	COG0355
AAQ00636.1_1589	CUST_8223_PI442190958	SS120	LLII/III	AAQ00636.1_1589	AGTTAATGCTGCAGAACTAGGAAAAAGCATTGACAAAGCCACAGCAGAAAAAGAGTTCGA	atpC	ATP synthase epsilon subunit		AAQ00636.1	1449179..1449589	Energy Metabolism	COG0355
AAQ00636.1_1589	CUST_8224_PI442190958	SS120	LLII/III	AAQ00636.1_1589	AAATTAAAAGCAAAAGAGAGTCTAAACAAAGCCAGAGCAAGGTCTCAAGCAGTTGGGGAA	atpC	ATP synthase epsilon subunit		AAQ00636.1	1449179..1449589	Energy Metabolism	COG0355
AAQ00636.1_1589	CUST_8225_PI442190958	SS120	LLII/III	AAQ00636.1_1589	CTAAGAGTACTTGCGCCAGACAAGAGTGTGTTTGACGACACAGTGGAAGAGGTCATACTC	atpC	ATP synthase epsilon subunit		AAQ00636.1	1449179..1449589	Energy Metabolism	COG0355
AAQ00636.1_1589	CUST_8226_PI442190958	SS120	LLII/III	AAQ00636.1_1589	AAGAGGTCATACTCCCAAGCACTACAGGTCTGCTGGGGATCCTCCC	atpC	ATP synthase epsilon subunit		AAQ00636.1	1449179..1449589	Energy Metabolism	COG0355
AAQ00636.1_1589	CUST_8227_PI442190958	SS120	LLII/III	AAQ00636.1_1589	TTCGAACAAGCTAAAGCAGCATTAAATAAACTTGAGGATCAAGCAGGTAATTCAG	atpC	ATP synthase epsilon subunit		AAQ00636.1	1449179..1449589	Energy Metabolism	COG0355
AAQ00648.1_1601	CUST_8290_PI442190958	SS120	LLII/III	AAQ00648.1_1601	GTCACGCAATTTGCCGCTGAACTTCGTGAATACTTGAAAACCAGTAAGCCAGATTACATA	atpA	ATP synthase alpha subunit		AAQ00648.1	1460526..1462043	Energy Metabolism	COG0056
AAQ00648.1_1601	CUST_8291_PI442190958	SS120	LLII/III	AAQ00648.1_1601	CCTTTGAATCTTGCTGAGCAGGTAGCAGTTGTTTATGCAGGAGTCAAAGGTTTGATTGAT	atpA	ATP synthase alpha subunit		AAQ00648.1	1460526..1462043	Energy Metabolism	COG0056
AAQ00648.1_1601	CUST_8292_PI442190958	SS120	LLII/III	AAQ00648.1_1601	TAGATGAAGCTACCCAGCAACAACTTGAGAGAGGTAAACGTTTGAGAGAGTTGCTTAAGC	atpA	ATP synthase alpha subunit		AAQ00648.1	1460526..1462043	Energy Metabolism	COG0056
AAQ00648.1_1601	CUST_8293_PI442190958	SS120	LLII/III	AAQ00648.1_1601	GCAATTAATGTCGGTATTTCAGTTAGTCGTGTTGGAGGTGCAGCTCAAACTAAAGCGATT	atpA	ATP synthase alpha subunit		AAQ00648.1	1460526..1462043	Energy Metabolism	COG0056
AAQ00648.1_1601	CUST_8294_PI442190958	SS120	LLII/III	AAQ00648.1_1601	GATGGGTAGTGGATCCATGACAGCTTTACCAATAATTGAGACTCAAGCTGGAGATGTGTC	atpA	ATP synthase alpha subunit		AAQ00648.1	1460526..1462043	Energy Metabolism	COG0056
AAQ00648.1_1601	CUST_8295_PI442190958	SS120	LLII/III	AAQ00648.1_1601	ACACAATTGTTGTAAATGCAAGTGCTTCAGAAGCAGCTGCATTGCAATATTTAGCACCTT	atpA	ATP synthase alpha subunit		AAQ00648.1	1460526..1462043	Energy Metabolism	COG0056
AAQ00653.1_1606	CUST_8317_PI442190958	SS120	LLII/III	AAQ00653.1_1606	GCTGATATAAACACAACCGTCGCACTTGCGCTTTTGGTGTCACTTTCATATTTTTATGCG	atpI	ATP synthase subunit A		AAQ00653.1	1464186..1464911	Energy Metabolism	COG0356
AAQ00653.1_1606	CUST_8318_PI442190958	SS120	LLII/III	AAQ00653.1_1606	CACCAAGCCTCTTTCACTTTCCTTCCGTTTATTTGGAAATATTCTGGCGGATGAACTTGT	atpI	ATP synthase subunit A		AAQ00653.1	1464186..1464911	Energy Metabolism	COG0356
AAQ00653.1_1606	CUST_8319_PI442190958	SS120	LLII/III	AAQ00653.1_1606	TACCTTTTGCTGCATTAGAGGTTGGTCAACACCTTTATTGGCAGATAGGGAAGTTAAGGA	atpI	ATP synthase subunit A		AAQ00653.1	1464186..1464911	Energy Metabolism	COG0356
AAQ00653.1_1606	CUST_8320_PI442190958	SS120	LLII/III	AAQ00653.1_1606	GCCTTGATTTTTGCAACTCTTGCTGCTTACTACATAGGTGAAGCAGTGGAAGAACATCAT	atpI	ATP synthase subunit A		AAQ00653.1	1464186..1464911	Energy Metabolism	COG0356
AAQ00653.1_1606	CUST_8321_PI442190958	SS120	LLII/III	AAQ00653.1_1606	CAGGTTTTTATGACTTCATGGATTCTTATAGGTGCACTGCTAACCCTTGTTGTGGTTGGC	atpI	ATP synthase subunit A		AAQ00653.1	1464186..1464911	Energy Metabolism	COG0356
AAQ00653.1_1606	CUST_8322_PI442190958	SS120	LLII/III	AAQ00653.1_1606	GCAATTGGGGAGGAGCTTTAATCCCATGGAAGTTAATTGAACTCCCTAGTGGAGAGTTAG	atpI	ATP synthase subunit A		AAQ00653.1	1464186..1464911	Energy Metabolism	COG0356
AAQ00664.1_1617	CUST_8374_PI442190958	SS120	LLII/III	AAQ00664.1_1617	CACAGGGCTCAATGCACCAGCAAAATTCGATAAAGAAATTGCTCTTGAGATTGCAAAGTC	fumC	Fumarate hydratase class II		AAQ00664.1	1472949..1474346	Krebs cycle and related genes	COG0114
AAQ00664.1_1617	CUST_8375_PI442190958	SS120	LLII/III	AAQ00664.1_1617	AACAAGTTCGCCATTATGGCAAGCCATGATGCATTAGTCCATACAATGTCTCAGTTGAAA	fumC	Fumarate hydratase class II		AAQ00664.1	1472949..1474346	Krebs cycle and related genes	COG0114
AAQ00664.1_1617	CUST_8376_PI442190958	SS120	LLII/III	AAQ00664.1_1617	GCCTCCGCATGGAAGGAACAACTTATTGCATCTCGTAATCGACTCAACAAAAGCTTAAAT	fumC	Fumarate hydratase class II		AAQ00664.1	1472949..1474346	Krebs cycle and related genes	COG0114
AAQ00664.1_1617	CUST_8377_PI442190958	SS120	LLII/III	AAQ00664.1_1617	ACTATGGCAGGTAGTGGTGGGCATTTACAAATGAATTCGTACAAACCTTTAATCGCGTTC	fumC	Fumarate hydratase class II		AAQ00664.1	1472949..1474346	Krebs cycle and related genes	COG0114
AAQ00664.1_1617	CUST_8378_PI442190958	SS120	LLII/III	AAQ00664.1_1617	AAATAGTTAATGATCTACGCCTTTTATCCTGTGGGCCAAGAGGAGGGCTAGGAGAACTAA	fumC	Fumarate hydratase class II		AAQ00664.1	1472949..1474346	Krebs cycle and related genes	COG0114
AAQ00664.1_1617	CUST_8379_PI442190958	SS120	LLII/III	AAQ00664.1_1617	CACAAGCTTGACTCCGATAATCGGTTACGAAAAAGCTAGTAAGATTGCACAATGTGCTCA	fumC	Fumarate hydratase class II		AAQ00664.1	1472949..1474346	Krebs cycle and related genes	COG0114
AAQ00712.1_1665	CUST_8637_PI442190958	SS120	LLII/III	AAQ00712.1_1665	CTTGGCAACACATGTCTATATGGAGGTACTGGCGGAAAATTATTTGCTCTTGGAAAAGCA	glsF	Ferredoxin-dependent glutamate synthase		AAQ00712.1	1529478..1534052	Nitrogen Metabolism	COG0069
AAQ00712.1_1665	CUST_8638_PI442190958	SS120	LLII/III	AAQ00712.1_1665	TAGTGCTAGGAGAAACTGGCAGAAATGTTGGAGCAGGAATGACTGGAGGAATAACTTTTT	glsF	Ferredoxin-dependent glutamate synthase		AAQ00712.1	1529478..1534052	Nitrogen Metabolism	COG0069
AAQ00712.1_1665	CUST_8639_PI442190958	SS120	LLII/III	AAQ00712.1_1665	ATGAATGTTCGTCTTATAGGCGAAGCTAATGATTATGTAGGGAAAGGCATTAACGGCGGA	glsF	Ferredoxin-dependent glutamate synthase		AAQ00712.1	1529478..1534052	Nitrogen Metabolism	COG0069
AAQ00712.1_1665	CUST_8640_PI442190958	SS120	LLII/III	AAQ00712.1_1665	ATTATTCCAATAGCAAACACGGATAGGAGTGTTTGTGCTCGTATTTCTGGCGAGATAGCA	glsF	Ferredoxin-dependent glutamate synthase		AAQ00712.1	1529478..1534052	Nitrogen Metabolism	COG0069
AAQ00712.1_1665	CUST_8641_PI442190958	SS120	LLII/III	AAQ00712.1_1665	AAAGGAACACTTGATCTAACCTTTAAAGGTGCTTCAGGACAAAGCTTTGGCGCATTCTTA	glsF	Ferredoxin-dependent glutamate synthase		AAQ00712.1	1529478..1534052	Nitrogen Metabolism	COG0069
AAQ00712.1_1665	CUST_8642_PI442190958	SS120	LLII/III	AAQ00712.1_1665	GTAAGAAATAGTGGGGTAGAAACAGTGGTTGAAGGAGCAGGCGATCATTGCTGTGAATAC	glsF	Ferredoxin-dependent glutamate synthase		AAQ00712.1	1529478..1534052	Nitrogen Metabolism	COG0069
AAQ00716.1_1669	CUST_8659_PI442190958	SS120	LLII/III	AAQ00716.1_1669	GCAAAGTTGCTATTGCTCCGATTCCCTTAGGAACTGCTGACTTGATGATTCATCATGTTC	psaA	Photosystem I P700 chlorophyll A apoprotein A1 PsaA		AAQ00716.1	1537494..1539815	Photosystem I	COG2885
AAQ00716.1_1669	CUST_8660_PI442190958	SS120	LLII/III	AAQ00716.1_1669	GTGGCAGAGGCTACTGGCAGGAACTTTTTGAGTCCATAATTTGGGCACATAATAAATTGA	psaA	Photosystem I P700 chlorophyll A apoprotein A1 PsaA		AAQ00716.1	1537494..1539815	Photosystem I	COG2885
AAQ00716.1_1669	CUST_8661_PI442190958	SS120	LLII/III	AAQ00716.1_1669	AATTTTGCACAAAGTTCTATCACTATTAACGGTTGGCTTCGTGACTTCCTTTGGGCTCAG	psaA	Photosystem I P700 chlorophyll A apoprotein A1 PsaA		AAQ00716.1	1537494..1539815	Photosystem I	COG2885
AAQ00716.1_1669	CUST_8662_PI442190958	SS120	LLII/III	AAQ00716.1_1669	CTGTTGTTATCTTCCATTTTTCTTGGAAGATGCAAAGTGATGTCTGGGGGCTAACAGGTG	psaA	Photosystem I P700 chlorophyll A apoprotein A1 PsaA		AAQ00716.1	1537494..1539815	Photosystem I	COG2885
AAQ00716.1_1669	CUST_8663_PI442190958	SS120	LLII/III	AAQ00716.1_1669	ATTAGCATGTATGGCCTAATGTTCCTTGGTGCTCATTTTGTATGGGCATTCAGCCTTATG	psaA	Photosystem I P700 chlorophyll A apoprotein A1 PsaA		AAQ00716.1	1537494..1539815	Photosystem I	COG2885
AAQ00716.1_1669	CUST_8664_PI442190958	SS120	LLII/III	AAQ00716.1_1669	AGTTGGAACTGGAGACTTAGTAGGCGCTGGTAATTTACCTGGAAGTGTTCTAAGTGAAGT	psaA	Photosystem I P700 chlorophyll A apoprotein A1 PsaA		AAQ00716.1	1537494..1539815	Photosystem I	COG2885
AAQ00717.1_1670	CUST_8665_PI442190958	SS120	LLII/III	AAQ00717.1_1670	GGATGTTCCTATTTGGCCACTTGATATGGGCTACTGGTTTTATGTTCTTGATCTCCTGGA	psaB	Photosystem I P700 chlorophyll A apoprotein A2 PsaB		AAQ00717.1	1539838..1542081	Photosystem I	COG2885
AAQ00717.1_1670	CUST_8666_PI442190958	SS120	LLII/III	AAQ00717.1_1670	ACTGCATTGATCCTTATTAAAGGAGCTCTTGATGCACGAGGATCCAAACTCATTCCTGAT	psaB	Photosystem I P700 chlorophyll A apoprotein A2 PsaB		AAQ00717.1	1539838..1542081	Photosystem I	COG2885
AAQ00717.1_1670	CUST_8667_PI442190958	SS120	LLII/III	AAQ00717.1_1670	GATAGTTCAGCTTGGGATGCTACTTACCTCGCTATGTTCTGGGCACTTAATACAATTGCT	psaB	Photosystem I P700 chlorophyll A apoprotein A2 PsaB		AAQ00717.1	1539838..1542081	Photosystem I	COG2885
AAQ00717.1_1670	CUST_8668_PI442190958	SS120	LLII/III	AAQ00717.1_1670	TTGGATGGACATGATTAACAGGCAAGATGCCTTAACTAATTTCCTTCCAATTGGACCTGC	psaB	Photosystem I P700 chlorophyll A apoprotein A2 PsaB		AAQ00717.1	1539838..1542081	Photosystem I	COG2885
AAQ00717.1_1670	CUST_8669_PI442190958	SS120	LLII/III	AAQ00717.1_1670	AGAACTAATAGAGACTCTTGTGTGGGCTCATCAACGTACTCCAATTGCCAATCTTGTTGG	psaB	Photosystem I P700 chlorophyll A apoprotein A2 PsaB		AAQ00717.1	1539838..1542081	Photosystem I	COG2885
AAQ00717.1_1670	CUST_8670_PI442190958	SS120	LLII/III	AAQ00717.1_1670	GTTCAGGCCCGTTTAGTTGGTTTAACACACTTCACTGTTGGTAACTTGTAACCTTGGT	psaB	Photosystem I P700 chlorophyll A apoprotein A2 PsaB		AAQ00717.1	1539838..1542081	Photosystem I	COG2885
AAQ00774.1_1727	CUST_8970_PI442190958	SS120	LLII/III	AAQ00774.1_1727	TGCTCGTAGGAAAACTGGTGCTAAAGATCTTTCGAGCTTAAGAGCTATTCCATGGGTTTT	ppc	Phosphoenolpyruvate carboxylase		AAQ00774.1	1580807..1583812	Krebs cycle and related genes	COG2352
AAQ00774.1_1727	CUST_8971_PI442190958	SS120	LLII/III	AAQ00774.1_1727	ACAGTTGTCAGTAGATCTACGTAATCGCACAATAGTGCCTTTAGGCTTTCTTCAAGTGGC	ppc	Phosphoenolpyruvate carboxylase		AAQ00774.1	1580807..1583812	Krebs cycle and related genes	COG2352
AAQ00774.1_1727	CUST_8972_PI442190958	SS120	LLII/III	AAQ00774.1_1727	GTTGGTTTGGTGTTGGAACAGCTTTGGAAGAAGAACTTAAGTCTGATCCAGACCACATTG	ppc	Phosphoenolpyruvate carboxylase		AAQ00774.1	1580807..1583812	Krebs cycle and related genes	COG2352
AAQ00774.1_1727	CUST_8973_PI442190958	SS120	LLII/III	AAQ00774.1_1727	CCGCTCTAGGCAACATTACAGAGCCCTTGTTCATGACAATCCTGATTAGTTGCTTTTTT	ppc	Phosphoenolpyruvate carboxylase		AAQ00774.1	1580807..1583812	Krebs cycle and related genes	COG2352
AAQ00774.1_1727	CUST_8974_PI442190958	SS120	LLII/III	AAQ00774.1_1727	TCGACAGCCACCTATGAGTGAGACTTTGTTAACCGAAGGTGATATTGGACGTACGTATAG	ppc	Phosphoenolpyruvate carboxylase		AAQ00774.1	1580807..1583812	Krebs cycle and related genes	COG2352
AAQ00774.1_1727	CUST_8975_PI442190958	SS120	LLII/III	AAQ00774.1_1727	GTAGAAGTGAACTCTTAAGAGGTGCTTTGTTAACCATTAATGGCATTGCGGCTGGGATG	ppc	Phosphoenolpyruvate carboxylase		AAQ00774.1	1580807..1583812	Krebs cycle and related genes	COG2352
AAQ00798.1_1751	CUST_9106_PI442190958	SS120	LLII/III	AAQ00798.1_1751	GAAGCTTGGGGTGGCAAGATTGTTGTTAAAGGTGTTCATATTGGAGATGATGCTCGTAAG	lldD	alpha-hydroxy-acidoxidizingprotein (L-lactate)		AAQ00798.1	1606671..1607843	Pyruvate metabolism	COG1304
AAQ00798.1_1751	CUST_9107_PI442190958	SS120	LLII/III	AAQ00798.1_1751	TTTGTTAGATGGAGGTATTAGACGTGGGGGCGATGTTATTAAAGCCTTATGTCTTGGAGC	lldD	alpha-hydroxy-acidoxidizingprotein (L-lactate)		AAQ00798.1	1606671..1607843	Pyruvate metabolism	COG1304
AAQ00798.1_1751	CUST_9108_PI442190958	SS120	LLII/III	AAQ00798.1_1751	TATCTAATCATGGAGCACGCCAATTAGATAGCGTTGCTCCCACTATTCGTGTTTTACCTG	lldD	alpha-hydroxy-acidoxidizingprotein (L-lactate)		AAQ00798.1	1606671..1607843	Pyruvate metabolism	COG1304
AAQ00798.1_1751	CUST_9109_PI442190958	SS120	LLII/III	AAQ00798.1_1751	AAATGCTTCCTTATCTTTCTCAAATGGTTGTTAAACCCTGCTGGATGACTCAGTGGTTGG	lldD	alpha-hydroxy-acidoxidizingprotein (L-lactate)		AAQ00798.1	1606671..1607843	Pyruvate metabolism	COG1304
AAQ00798.1_1751	CUST_9110_PI442190958	SS120	LLII/III	AAQ00798.1_1751	TCCGCAATAGTTGTGACGATTGATACCCCTGTTTCAGGATTAAGAGAACGTGATTTGCTT	lldD	alpha-hydroxy-acidoxidizingprotein (L-lactate)		AAQ00798.1	1606671..1607843	Pyruvate metabolism	COG1304
AAQ00798.1_1751	CUST_9111_PI442190958	SS120	LLII/III	AAQ00798.1_1751	CGGGCCAGGTGTTGCGAGGGCAATTGAAATTATTAAAACTGATGTTTTACGTACGATGAA	lldD	alpha-hydroxy-acidoxidizingprotein (L-lactate)		AAQ00798.1	1606671..1607843	Pyruvate metabolism	COG1304
AAQ00799.1_1752	CUST_9112_PI442190958	SS120	LLII/III	AAQ00799.1_1752	ACTGAAATTCTTGGATTCAATAAATGCAAAATATCCTGCCGAACATAATGTTGGTCATTT	dld	D-lactate dehydrogenase, NADH independent		AAQ00799.1	1607961..1609661	Pyruvate metabolism	COG0277
AAQ00799.1_1752	CUST_9113_PI442190958	SS120	LLII/III	AAQ00799.1_1752	AGATGGCGCATTTTCTTTGCATGGTATTCCACTTAGATTTTGTCGTTAAAAAGGAGTCG	dld	D-lactate dehydrogenase, NADH independent		AAQ00799.1	1607961..1609661	Pyruvate metabolism	COG0277
AAQ00799.1_1752	CUST_9114_PI442190958	SS120	LLII/III	AAQ00799.1_1752	TGTTGCAGGAGCAGCTCCTGCTAGATATAAAATACTTAATCCGGACTTAGCAGGAGATCT	dld	D-lactate dehydrogenase, NADH independent		AAQ00799.1	1607961..1609661	Pyruvate metabolism	COG0277
AAQ00799.1_1752	CUST_9115_PI442190958	SS120	LLII/III	AAQ00799.1_1752	TGCAATTTATAGCAGATATTCTTCCTAACCACCTGCCAATAAGGCTACTATCTTTCCGCA	dld	D-lactate dehydrogenase, NADH independent		AAQ00799.1	1607961..1609661	Pyruvate metabolism	COG0277
AAQ00799.1_1752	CUST_9116_PI442190958	SS120	LLII/III	AAQ00799.1_1752	ATGAATTCTTTGCCTGACATGGGAGAATATATGCACTTTAGTTACTTTGATGGATCTGCT	dld	D-lactate dehydrogenase, NADH independent		AAQ00799.1	1607961..1609661	Pyruvate metabolism	COG0277
AAQ00799.1_1752	CUST_9117_PI442190958	SS120	LLII/III	AAQ00799.1_1752	TCTTTGTTGGTACAAATAATGCCGAACATTTGACGCATTTAAGAGAGAAAATATTGACAA	dld	D-lactate dehydrogenase, NADH independent		AAQ00799.1	1607961..1609661	Pyruvate metabolism	COG0277
AAQ00810.1_1763	CUST_9172_PI442190958	SS120	LLII/III	AAQ00810.1_1763	TTAGCAATGGAACATGAAAGACGCTCTGCTTGTAATGATATTGCGTTTGCTTCCAGACAA	glmS	Glutamine--fructose-6-phosphatetransaminase(isomerizing)		AAQ00810.1	1620235..1622139	Amino Acid metabolism	COG0449
AAQ00810.1_1763	CUST_9173_PI442190958	SS120	LLII/III	AAQ00810.1_1763	GCACGACAAGTGTCTAATATTCTTGATATAGGAGCGGGATTAGAAGTTGGTGTTGCAGCT	glmS	Glutamine--fructose-6-phosphatetransaminase(isomerizing)		AAQ00810.1	1620235..1622139	Amino Acid metabolism	COG0449
AAQ00810.1_1763	CUST_9174_PI442190958	SS120	LLII/III	AAQ00810.1_1763	TGTTTTTGAAAAAGCTTTAAGCAATGCGCAAGAAGCTAAAGCTAGGGATGCATGCTTGAT	glmS	Glutamine--fructose-6-phosphatetransaminase(isomerizing)		AAQ00810.1	1620235..1622139	Amino Acid metabolism	COG0449
AAQ00810.1_1763	CUST_9175_PI442190958	SS120	LLII/III	AAQ00810.1_1763	ACATGCAGAAGGATATCCTGCTGGTGAAATGAAACACGGTCCAATTGCCTTATTAGATAA	glmS	Glutamine--fructose-6-phosphatetransaminase(ison		AAQ00810.1	1620235..1622139	Amino Acid metabolism	COG0449
AAQ00810.1_1763	CUST_9176_PI442190958	SS120	LLII/III	AAQ00810.1_1763	GGCAGAGGAATCAATTATCCAATAGCCTTGGAAGCAGCATTAAAGCTTAAGGAAATTAGT	glmS	Glutamine--fructose-6-phosphatetransaminase(isomerizing)		AAQ00810.1	1620235..1622139	Amino Acid metabolism	COG0449
AAQ00810.1_1763	CUST_9177_PI442190958	SS120	LLII/III	AAQ00810.1_1763	TCACATTCGCAGCTCATAGAGGTTTGGATGTTGATCAACCAAGAAATCTAGCTAAAAGTGT	glmS	Glutamine--fructose-6-phosphatetransaminase(isomerizing)		AAQ00810.1	1620235..1622139	Amino Acid metabolism	COG0449
CAE19009.1_607	CUST_3142_PI442190954	MED4	HLI	CAE19009.1_607	AATATTTGGAGATGATTCTTGTCTTCAATTCGGAGGAGGAACTCATGGTCATCCATGGGG	rbcL	Ribulose 1,5-bisphosphate carboxylase large subunit	PMM0550	CAE19009.1	519087..520502	Carbon fixation	COG1850
CAE19009.1_607	CUST_3143_PI442190954	MED4	HLI	CAE19009.1_607	GCTAATACCCCTGAAGAACTATACGAACGTGCAGAGTTTGCTAAAGAGCTTGATATGCCA	rbcL	Ribulose 1,5-bisphosphate carboxylase large subunit	PMM0550	CAE19009.1	519087..520502	Carbon fixation	COG1850
CAE19010.1_608	CUST_3145_PI442190954	MED4	HLI	CAE19010.1_608	ATTGGTTATGACGCTTATACACAAAGTCAAGGTACAGCTTTTGCAGTTTTCAAGGACGC	rbcS	Ribulose bisphosphate carboxylase small subunit	PMM0551	CAE19010.1	520592..520933	Carbon fixation	COG4451
CAE19010.1_608	CUST_3146_PI442190954	MED4	HLI	CAE19010.1_608	GCTCCCTTTCTTTGGGGAAAAAGATCTTAACTTGGTTGTAAGTGAGTTAGAAGCATGCCA	rbcS	Ribulose bisphosphate carboxylase small subunit	PMM0551	CAE19010.1	520592..520933	Carbon fixation	COG4451
CAE19010.1_608	CUST_3147_PI442190954	MED4	HLI	CAE19010.1_608	TTGCCTACATTATTGCTCAAGGATGGAGTCCTGTTATTGAGCATGTACATCCAAGTGGAA	rbcS	Ribulose bisphosphate carboxylase small subunit	PMM0551	CAE19010.1	520592..520933	Carbon fixation	COG4451
CAE19010.1_608	CUST_3148_PI442190954	MED4	HLI	CAE19010.1_608	TTCCAGAGCTCAGTTGGTGACTATCAAACAGTTGCTACCCTGGAAACATTCGGTTTTTTA	rbcS	Ribulose bisphosphate carboxylase small subunit	PMM0551	CAE19010.1	520592..520933	Carbon fixation	COG4451
CAE19055.1_653	CUST_3387_PI442190954	MED4	HLI	CAE19055.1_653	GATTTACATGCAATCGTTGGAGCAGGAACTGGTCTAGGCATTGCAAAAGGCATAATATCT	glk	Glucokinase	PMM0596	CAE19055.1	565152..566189	Glycolysis	COG0837
CAE19055.1_653	CUST_3388_PI442190954	MED4	HLI	CAE19055.1_653	TGAAGTGGAAAGAATATGGTTAGATGCTTACGCCTCATTATTGGGTGACGTTGCTCTTCA	glk	Glucokinase	PMM0596	CAE19055.1	565152..566189	Glycolysis	COG0837
CAE19055.1_653	CUST_3389_PI442190954	MED4	HLI	CAE19055.1_653	GGGTTATCAAGAATAGCTGAGTGGAGACTTAGCAAACCTGATGCCAAAAACCATCCTCTA	glk	Glucokinase	PMM0596	CAE19055.1	565152..566189	Glycolysis	COG0837
CAE19055.1_653	CUST_3390_PI442190954	MED4	HLI	CAE19055.1_653	AGTTTTAGCTAGCGAAGGTGGTCATGTTGAATACTCGCCAAAGTCAGATTTAGAATGGGA	glk	Glucokinase	PMM0596	CAE19055.1	565152..566189	Glycolysis	COG0837
CAE19055.1_653	CUST_3391_PI442190954	MED4	HLI	CAE19055.1_653	AGATGAAGAGTTTGGATTATTTAGCGCAGCCTGCAGAGCAAAAATGCTTTCACAAAAAGA	glk	Glucokinase	PMM0596	CAE19055.1	565152..566189	Glycolysis	COG0837
CAE19055.1_653	CUST_3392_PI442190954	MED4	HLI	CAE19055.1_653	GGAGGGTTATGGATTTCTGGAGGAACCGCTCCAAAACATTTCATAAACTTTAAATCAGGT	glk	Glucokinase	PMM0596	CAE19055.1	565152..566189	Glycolysis	COG0837
CAE19228.1_868	CUST_4396_PI442190954	MED4	HLI	CAE19228.1_868	TAAAATCAATGGCGGTTGATACCTCTAAGTTCGGTTTAACCAAGGAATCGGCATCAGAGA	glgC	Glucose-1-phosphate adenylyltransferase	PMM0769	CAE19228.1	732893..734188	Glycogen Metabolism	COG0448
CAE19228.1_868	CUST_4397_PI442190954	MED4	HLI	CAE19228.1_868	AGAGGATTGAATTAAGAAAAGGTGGTGGTACGCCTCTTGGAGTAGGAGAAGGTTCAACTA	glgC	Glucose-1-phosphate adenylyltransferase	PMM0769	CAE19228.1	732893..734188	Glycogen Metabolism	COG0448
CAE19228.1_868	CUST_4398_PI442190954	MED4	HLI	CAE19228.1_868	TGTGAAGGAACTATTTTGAAATCTTGTAGCATCTTGCATTGTGTCTTAGGGGTGAGAAGT	glgC	Glucose-1-phosphate adenylyltransferase	PMM0769	CAE19228.1	732893..734188	Glycogen Metabolism	COG0448
CAE19228.1_868	CUST_4399_PI442190954	MED4	HLI	CAE19228.1_868	ACCGTATCTTGCCTCTATGGGCATTTATGTTTTCAGTAGAAAGACACTTTTTGATCTCTT	glgC	Glucose-1-phosphate adenylyltransferase	PMM0769	CAE19228.1	732893..734188	Glycogen Metabolism	COG0448
CAE19228.1_868	CUST_4400_PI442190954	MED4	HLI	CAE19228.1_868	AGGAGCATTTTTTGAATCAAACTTAGCATTAACACAACAACCAAAACCTCCGTTTAGTTT	glgC	Glucose-1-phosphate adenylyltransferase	PMM0769	CAE19228.1	732893..734188	Glycogen Metabolism	COG0448
CAE19228.1_868	CUST_4401_PI442190954	MED4	HLI	CAE19228.1_868	AGTTCCCTAGTTATACAGATTTTGGTAAAGATATAATCCCTGAGGCATTAAGTAGAGGGG	glgC	Glucose-1-phosphate adenylyltransferase	PMM0769	CAE19228.1	732893..734188	Glycogen Metabolism	COG0448
CAE19229.1_869	CUST_4402_PI442190954	MED4	HLI	CAE19229.1_869	AACCTTTGTTTGATGCAGTCGTTCTTGCTACTATTGCTAGTTACGCTCAAGGGATGGATA	edd	6-Phosphogluconate dehydratase	PMM0770	CAE19229.1	734287..735705	Pentose Phosphate/Entner-Dudorof	COG2084
CAE19229.1_869	CUST_4403_PI442190954	MED4	HLI	CAE19229.1_869	TGGCCAAAAAGGTACTGGATTGTGGACTGTTGTTAGTGCCTTAGAACTTGGTATATCAGT	edd	6-Phosphogluconate dehydratase	PMM0770	CAE19229.1	734287..735705	Pentose Phosphate/Entner-Dudorof	COG2084
CAE19229.1_869	CUST_4404_PI442190954	MED4	HLI	CAE19229.1_869	GCAAGTGTCGTTTCTTCTTCAACTAAGGCTGGTATTCCAGTGCCTTGTTTATCAAGTACT	edd	6-Phosphogluconate dehydratase	PMM0770	CAE19229.1	734287..735705	Pentose Phosphate/Entner-Dudorof	COG2084
CAE19229.1_869	CUST_4405_PI442190954	MED4	HLI	CAE19229.1_869	TGCAAGGGTTATGAGTTCCCTTAAGGTTCAACGTAGTGAAATCGAGAAAACAATTCCGAT	edd	6-Phosphogluconate dehydratase	PMM0770	CAE19229.1	734287..735705	Pentose Phosphate/Entner-Dudorof	COG2084
CAE19229.1_869	CUST_4406_PI442190954	MED4	HLI	CAE19229.1_869	CAAGCCATGAGAGATTGTTTTGGTTCACATACTTACGAAAGAGTTGACAAGGAAGGAAGT	edd	6-Phosphogluconate dehydratase	PMM0770	CAE19229.1	734287..735705	Pentose Phosphate/Entner-Dudorof	COG2084
CAE19229.1_869	CUST_4407_PI442190954	MED4	HLI	CAE19229.1_869	ATGAATGGATCACAAATGGCTGAGGTTTTTGGTCTTTGGAATAATACTGATGAATTGGCA	edd	6-Phosphogluconate dehydratase	PMM0770	CAE19229.1	734287..735705	Pentose Phosphate/Entner-Dudorof	COG2084
CAE19240.1_881	CUST_4464_PI442190954	MED4	HLI	CAE19240.1_881	GGAGTTAGTGTTGAGGGAGAATTAGGTTGCTTAGGATCCTTAGAGACAGGGAAAGGTGAA	fda	Fructose-1,6-bisphosphate aldolase class I	PMM0781	CAE19240.1	742975..744048	Glycolysis/Gluconeogenesis	COG0191
CAE19240.1_881	CUST_4465_PI442190954	MED4	HLI	CAE19240.1_881	TCCTGAAGAAGCTGCAGACTTTGTAGCGAAAACAAAAGTTGATGCTTTAGCCATAGCAAT	fda	Fructose-1,6-bisphosphate aldolase class I	PMM0781	CAE19240.1	742975..744048	Glycolysis/Gluconeogenesis	COG0191
CAE19240.1_881	CUST_4466_PI442190954	MED4	HLI	CAE19240.1_881	TCGATACTGATAATAGACTTGCATTTACAGCCGCAGTTAGAGAAGCTGCTTTTGCAGACA	fda	Fructose-1,6-bisphosphate aldolase class I	PMM0781	CAE19240.1	742975..744048	Glycolysis/Gluconeogenesis	COG0191
CAE19240.1_881	CUST_4467_PI442190954	MED4	HLI	CAE19240.1_881	GGTGCATATAAATTCACAAGAAAGCCAACAGGAGAGGTTCTTGCGATAAGCAGAATTGCT	fda	Fructose-1,6-bisphosphate aldolase class I	PMM0781	CAE19240.1	742975..744048	Glycolysis/Gluconeogenesis	COG0191
CAE19240.1_881	CUST_4468_PI442190954	MED4	HLI	CAE19240.1_881	TGGTGCGAAGGTCAAGCAAGTAAGATCAAACAAAGCAGCACAAATTATTACGCTGATCTT	fda	Fructose-1,6-bisphosphate aldolase class I	PMM0781	CAE19240.1	742975..744048	Glycolysis/Gluconeogenesis	COG0191
CAE19240.1_881	CUST_4469_PI442190954	MED4	HLI	CAE19240.1_881	ATGGACGGATCTTTAGAGGCAGATGCAAAAACACCCGCAAGTTATGAATATAATGTTGCC	fda	Fructose-1,6-bisphosphate aldolase class I	PMM0781	CAE19240.1	742975..744048	Glycolysis/Gluconeogenesis	COG0191
CAE19244.1_885	CUST_4487_PI442190954	MED4	HLI	CAE19244.1_885	ACAGATTTGAAAGAATGCCTATGAAAAAAGCAATGGCAGAAGCACTCTCAAAAGGGGAGA	prk	Phosphoribulokinase		CAE19244.1	746750..747649	Carbon Fixation	COG3954
CAE19244.1_885	CUST_4488_PI442190954	MED4	HLI	CAE19244.1_885	AACATAATGCAAGACTTGGAACATCATTAGAACCAGGTCAATTCACCCCCTGGGAAGATA	prk	Phosphoribulokinase		CAE19244.1	746750..747649	Carbon Fixation	COG3954
CAE19244.1_885	CUST_4489_PI442190954	MED4	HLI	CAE19244.1_885	GAGCATAGTTGTTAATGGAGGGAAAATGGGTTTTGCCATGGAATTGATCCTCACTCCAAT	prk	Phosphoribulokinase		CAE19244.1	746750..747649	Carbon Fixation	COG3954
CAE19244.1_885	CUST_4490_PI442190954	MED4	HLI	CAE19244.1_885	TTTCTACGAAGGTTTACATGGTGGTGTTGAGGGAGAAGGATACAATGTTTCCTCCTATGC	prk	Phosphoribulokinase		CAE19244.1	746750..747649	Carbon Fixation	COG3954
CAE19244.1_885	CUST_4491_PI442190954	MED4	HLI	CAE19244.1_885	TGGGGGATTGACTTTCAATATCTTTTGGGAATGATTCATGATTCATTCATGTCAAGCCCA	prk	Phosphoribulokinase		CAE19244.1	746750..747649	Carbon Fixation	COG3954
CAE19244.1_885	CUST_4492_PI442190954	MED4	HLI	CAE19244.1_885	ATGGTGAATCAGGTGGAGGTAAAAAAAGATATTATCTTCACAGCACTGAAGAAGCACAGG	prk	Phosphoribulokinase		CAE19244.1	746750..747649	Carbon Fixation	COG3954
CAE19349.1_1017	CUST_5081_PI442190954	MED4	HLI	CAE19349.1_1017	GTTGATATAAATGCTTATGATCAACCTGGTGTAGAAGCTGGTAAAAAAGCAGCCGCGGAA	pgi	Glucose-6-phosphate isomerase	PMM0890	CAE19349.1	851817..853397	Glycolysis/Gluconeogenesis	COG0166
CAE19349.1_1017	CUST_5082_PI442190954	MED4	HLI	CAE19349.1_1017	ACCTTGGATAAGTTGAGTTGTTTAACACTAGGTGCCTTAATTGCTCTATTTGAGCGAGCT	pgi	Glucose-6-phosphate isomerase	PMM0890	CAE19349.1	851817..853397	Glycolysis/Gluconeogenesis	COG0166
CAE19349.1_1017	CUST_5083_PI442190954	MED4	HLI	CAE19349.1_1017	TCCTGCTGCTTTACTATCATCAGCATGGTTTTTTCTGGTGATGGAATAGGTAAGAGAGA	pgi	Glucose-6-phosphate isomerase	PMM0890	CAE19349.1	851817..853397	Glycolysis/Gluconeogenesis	COG0166
CAE19349.1_1017	CUST_5084_PI442190954	MED4	HLI	CAE19349.1_1017	GGCGAAGAATTGTCAATCAAAGAAATTACTTCTCTTCTAAGGAATTCATCAGCTGAGCCA	pgi	Glucose-6-phosphate isomerase	PMM0890	CAE19349.1	851817..853397	Glycolysis/Gluconeogenesis	COG0166
CAE19349.1_1017	CUST_5085_PI442190954	MED4	HLI	CAE19349.1_1017	CGTCAGATGTGTTTTGGTAATGATGACTATCTTATAAATGGAGACTGGTCTAAGCCAAGT	pgi	Glucose-6-phosphate isomerase	PMM0890	CAE19349.1	851817..853397	Glycolysis/Gluconeogenesis	COG0166
CAE19349.1_1017	CUST_5086_PI442190954	MED4	HLI	CAE19349.1_1017	AGGAAGGGTGAAATCGTACATCAAGGAATATCTGTTTTTGGTAATAAAGGCTCTACTGAT	pgi	Glucose-6-phosphate isomerase	PMM0890	CAE19349.1	851817..853397	Glycolysis/Gluconeogenesis	COG0166
CAE19371.1_1039	CUST_5199_PI442190954	MED4	HLI	CAE19371.1_1039	CCAAATCCGGTTCAACAGCCCGTAATGTGAGTAAATTCAGACCTCCTACTCCTATTCTTG	pykF	Pyruvate kinase	PMM0912	CAE19371.1	872358..874148	Glycolysis	COG0469
CAE19371.1_1039	CUST_5200_PI442190954	MED4	HLI	CAE19371.1_1039	TCAGACTGCAGGGACACTAACAGGTATAAGTGGTTCGACTGATCTAATAAAGGTTGGGTT	pykF	Pyruvate kinase	PMM0912	CAE19371.1	872358..874148	Glycolysis	COG0469
CAE19371.1_1039	CUST_5201_PI442190954	MED4	HLI	CAE19371.1_1039	AGAAAAGTGGTAACTCGAGGAATATCAGTAGGGGAGATAGGTGTAACTGGCAAAGCAAGA	pykF	Pyruvate kinase	PMM0912	CAE19371.1	872358..874148	Glycolysis	COG0469
CAE19371.1_1039	CUST_5202_PI442190954	MED4	HLI	CAE19371.1_1039	GAGCGATGTGTCGCGAGAAGATTGCAATTAGTATGGGGAGTGACTCCTTTATTAGTCAAG	pykF	Pyruvate kinase	PMM0912	CAE19371.1	872358..874148	Glycolysis	COG0469
CAE19371.1_1039	CUST_5203_PI442190954	MED4	HLI	CAE19371.1_1039	GTTACAACTCAATGAAGGGGTTGTTTATATGGGGCAGATAGAAGACGATGAGGATGCACT	pykF	Pyruvate kinase	PMM0912	CAE19371.1	872358..874148	Glycolysis	COG0469
CAE19371.1_1039	CUST_5204_PI442190954	MED4	HLI	CAE19371.1_1039	CTAGTACTATTCCAAATGCAATAAGCGCCGCAGTCAGCAACATTGCTAGACAACTTGATG	pykF	Pyruvate kinase	PMM0912	CAE19371.1	872358..874148	Glycolysis	COG0469
CAE19379.1_1047	CUST_5245_PI442190954	MED4	HLI	CAE19379.1_1047	AATTTTATCGATATGAAGTACGAAGAGGTTCAACAATTAAGGCAGAGGCCCCACCCTCAT	glnA	Glutamine synthetase/ glutamate--ammonialigase	PMM0920	CAE19379.1	881367..882788	Nitrogen Metabolism	COG0174
CAE19379.1_1047	CUST_5246_PI442190954	MED4	HLI	CAE19379.1_1047	TCCAGGTTTTGAAGCTCCGGTAAATCTAGTTTACTCAGAGGGTAATAGATCAGCAGCTGT	glnA	Glutamine synthetase/ glutamate--ammonialigase	PMM0920	CAE19379.1	881367..882788	Nitrogen Metabolism	COG0174
CAE19379.1_1047	CUST_5247_PI442190954	MED4	HLI	CAE19379.1_1047	CTCAGACAGCAAGGTGGTATATCGGAGGTATTTAAAACACGCTCCATCATTCCTAGCAT	glnA	Glutamine synthetase/ glutamate--ammonialigase	PMM0920	CAE19379.1	881367..882788	Nitrogen Metabolism	COG0174
CAE19379.1_1047	CUST_5248_PI442190954	MED4	HLI	CAE19379.1_1047	ACGATAATGGAACAGGCATGCATGTTCATCAGAGTTTATGGGAAAGTGGTCAACCTCTAT	glnA	Glutamine synthetase/ glutamate--ammonialigase	PMM0920	CAE19379.1	881367..882788	Nitrogen Metabolism	COG0174
CAE19379.1_1047	CUST_5249_PI442190954	MED4	HLI	CAE19379.1_1047	GTCGCTGGAGCAGGACAACATGAACTTGGTATAAAATTTGATTCCTTAATTAGCTCCGCT	glnA	Glutamine synthetase/ glutamate--ammonialigase	PMM0920	CAE19379.1	881367..882788	Nitrogen Metabolism	COG0174
CAE19379.1_1047	CUST_5250_PI442190954	MED4	HLI	CAE19379.1_1047	AGATCAGGTGATGCACTTGCTAACCCTATTTAGCATTTTCTGTAATGATGCTAGCTGGT	glnA	Glutamine synthetase/ glutamate--ammonialigase	PMM0920	CAE19379.1	881367..882788	Nitrogen Metabolism	COG0174
CAE19389.1_1058	CUST_5303_PI442190954	MED4	HLI	CAE19389.1_1058	ATTTCCAACAACATGGGTATGCTCAGATATACCAGTGGCGGTAACTATAAAATACCCGCA	pdhB	Pyruvate dehydrogenase E1 component beta subuni	PMM0930	CAE19389.1	890595..891578	Glycolysis/Krebs cycle	COG0022
CAE19389.1_1058	CUST_5304_PI442190954	MED4	HLI	CAE19389.1_1058	TTAGGCGCTGAACATAGTCAAAGATTAGAGGCATACTTTCACGCAGTTCCTGGGATTAAG	pdhB	Pyruvate dehydrogenase E1 component beta subuni	PMM0930	CAE19389.1	890595..891578	Glycolysis/Krebs cycle	COG0022
CAE19389.1_1058	CUST_5305_PI442190954	MED4	HLI	CAE19389.1_1058	TGTTGCTTGTAGCACTCCTACTAATGCAAAGGCATTAATGAAAGCTGCTATAAGGGACAA	pdhB	Pyruvate dehydrogenase E1 component beta subuni	PMM0930	CAE19389.1	890595..891578	Glycolysis/Krebs cycle	COG0022
CAE19389.1_1058	CUST_5306_PI442190954	MED4	HLI	CAE19389.1_1058	TGTTGAGGAGTGTATGAAGACTGGAGGTATTGGAGCAGAATTAATTGCTTTGATTACCGA	pdhB	Pyruvate dehydrogenase E1 component beta subuni	PMM0930	CAE19389.1	890595..891578	Glycolysis/Krebs cycle	COG0022
CAE19389.1_1058	CUST_5307_PI442190954	MED4	HLI	CAE19389.1_1058	TGAGACATCATTGCTTAAAGGCTGTTGAGGAATTAGATAAGAAAAATATTGACGTGGAAT	pdhB	Pyruvate dehydrogenase E1 component beta subuni	PMM0930	CAE19389.1	890595..891578	Glycolysis/Krebs cycle	COG0022
CAE19389.1_1058	CUST_5308_PI442190954	MED4	HLI	CAE19389.1_1058	GGAATTGCCTGAAGGAGATTATATATGTTCTTTAGATCAAGCTGATTTAGTCAAAGAGGG	pdhB	Pyruvate dehydrogenase E1 component beta subuni	PMM0930	CAE19389.1	890595..891578	Glycolysis/Krebs cycle	COG0022
CAE19503.1_1210	CUST_5971_PI442190954	MED4	HLI	CAE19503.1_1210	TATCCTGCCGGGGGAGGATCAGTAATAGCTAAAAGACTAAGGGAATTAACGAACAGTGAA	rbsK	Sugar kinase, ribokinase family	PMM1044	CAE19503.1	986582..987559	Pentose Phosphate	COG0524
CAE19503.1_1210	CUST_5972_PI442190954	MED4	HLI	CAE19503.1_1210	AACCCAAAATATGTTATCAAAACAGAAGGTGAGAATGGCGGGATAGTATTCCCAGGAGGT	rbsK	Sugar kinase, ribokinase family	PMM1044	CAE19503.1	986582..987559	Pentose Phosphate	COG0524
CAE19503.1_1210	CUST_5973_PI442190954	MED4	HLI	CAE19503.1_1210	ACTAAAAAAAATAAGGTTGACTCATATGGATGCGGCGATTCTTTCGCGGCAGGAATTCTT	rbsK	Sugar kinase, ribokinase family	PMM1044	CAE19503.1	986582..987559	Pentose Phosphate	COG0524
CAE19503.1_1210	CUST_5974_PI442190954	MED4	HLI	CAE19503.1_1210	TGATGATTTAGATTGGGCTATCCTTGATGAGATGGATGGAGTTTTTATTACGGCAGGCGA	rbsK	Sugar kinase, ribokinase family	PMM1044	CAE19503.1	986582..987559	Pentose Phosphate	COG0524
CAE19503.1_1210	CUST_5975_PI442190954	MED4	HLI	CAE19503.1_1210	TGTAGGATGGCGAGATAAACCAACAAGAAAAGGTTTCAGTGTAATTGATTCTGATGGCGA	rbsK	Sugar kinase, ribokinase family	PMM1044	CAE19503.1	986582..987559	Pentose Phosphate	COG0524
CAE19503.1_1210	CUST_5976_PI442190954	MED4	HLI	CAE19503.1_1210	TGTACGACGCCCAGAGTAGGGTTAAATATAATTAACGAATCCGAGATATTTTTAGATGGA	rbsK	Sugar kinase, ribokinase family	PMM1044	CAE19503.1	986582..987559	Pentose Phosphate	COG0524
CAE19532.1_1239	CUST_6133_PI442190954	MED4	HLI	CAE19532.1_1239	AGACTTAAAAGTTGGCGTGAATCATTAGCAATGATTTTTGATCCTCCATCCAGGAGACCT	opcA	Glucose-6-P dehydrogenase subunit opcA	PMM1073	CAE19532.1	1015278..1016579	Pentose Phosphate	COG3429
CAE19532.1_1239	CUST_6134_PI442190954	MED4	HLI	CAE19532.1_1239	ACCCTGTACCTTTGGGAAATCCAAGTATTCATTCAGGCCAAGTTATTGGATTAAGGTTGA	opcA	Glucose-6-P dehydrogenase subunit opcA	PMM1073	CAE19532.1	1015278..1016579	Pentose Phosphate	COG3429
CAE19532.1_1239	CUST_6135_PI442190954	MED4	HLI	CAE19532.1_1239	TCACAGATCAGGGTATTAGGTTAATTATTGATAGTGCAAATGGGTCGCCCAAAAGATGCT	opcA	Glucose-6-P dehydrogenase subunit opcA	PMM1073	CAE19532.1	1015278..1016579	Pentose Phosphate	COG3429
CAE19532.1_1239	CUST_6136_PI442190954	MED4	HLI	CAE19532.1_1239	TGAGACTTGAAGCTGGAGGTATGGCTGATATGCAATTAATAGAGCAAGTTGTTCCAAACT	opcA	Glucose-6-P dehydrogenase subunit opcA	PMM1073	CAE19532.1	1015278..1016579	Pentose Phosphate	COG3429
CAE19532.1_1239	CUST_6137_PI442190954	MED4	HLI	CAE19532.1_1239	TGATGACCTATCTATTAAAGATTTACCTATTTGGTTATGGTGGAATGGCAGCCTTGATGA	opcA	Glucose-6-P dehydrogenase subunit opcA	PMM1073	CAE19532.1	1015278..1016579	Pentose Phosphate	COG3429
CAE19532.1_1239	CUST_6138_PI442190954	MED4	HLI	CAE19532.1_1239	GGGAAGTAGTAGAGGAAATACTAGTCCACTTTTTGAAAATGCTATTAAGGTCGCAGTTCA	opcA	Glucose-6-P dehydrogenase subunit opcA	PMM1073	CAE19532.1	1015278..1016579	Pentose Phosphate	COG3429
CAE19533.1_1240	CUST_6139_PI442190954	MED4	HLI	CAE19533.1_1240	AAAAGCAAAAGTTCTTCAAGCTTCAAAACTTGCCGATGAGAATGAACCCTGGAATTGCTG	zwf	glucose-6-phosphate dehydrogenase zwf	PMM1074	CAE19533.1	1016587..1018110	Pentose Phosphate	COG0364
CAE19533.1_1240	CUST_6140_PI442190954	MED4	HLI	CAE19533.1_1240	GAGATATGCTCCAAAATCATTTAACTCAAATGCTTGCTGTAACAGCGATGGAGCCTCCTG	zwf	glucose-6-phosphate dehydrogenase zwf	PMM1074	CAE19533.1	1016587..1018110	Pentose Phosphate	COG0364
CAE19533.1_1240	CUST_6141_PI442190954	MED4	HLI	CAE19533.1_1240	GGAAGACGCTCCTTGGAAGTTACCATTCATAAATACGAATCAATGACTTGGGGACCTCC	zwf	glucose-6-phosphate dehydrogenase zwf	PMM1074	CAE19533.1	1016587..1018110	Pentose Phosphate	COG0364
CAE19533.1_1240	CUST_6142_PI442190954	MED4	HLI	CAE19533.1_1240	TGGCGATGGCAAGGAGTTCCTTTCTTCTTAAGAACAGGTAAAAGACTACCGAAAAGACTT	zwf	glucose-6-phosphate dehydrogenase zwf	PMM1074	CAE19533.1	1016587..1018110	Pentose Phosphate	COG0364
CAE19533.1_1240	CUST_6143_PI442190954	MED4	HLI	CAE19533.1_1240	TTCTTACGATGAATCTTTTGGAGAGCCTTCTGACGAAGGTTATGTAAGGTTGTTGGCTGA	zwf	glucose-6-phosphate dehydrogenase zwf	PMM1074	CAE19533.1	1016587..1018110	Pentose Phosphate	COG0364
CAE19533.1_1240	CUST_6144_PI442190954	MED4	HLI	CAE19533.1_1240	ATGCTCTCAGATCCTACCTTGTTTACAAGGAGTGATGAGGTAGAAGCTGCTTGGAAACTT	zwf	glucose-6-phosphate dehydrogenase zwf	PMM1074	CAE19533.1	1016587..1018110	Pentose Phosphate	COG0364
CAE19668.1_1395	CUST_6915_PI442190954	MED4	HLI	CAE19668.1_1395	TTTGGGAAAGACTGGCCTACTAGGGATGGCACCTGTTTAAGAGATTATGTTCATGTTGAG	galE	UDP-glucose 4-epimerase	PMM1209	CAE19668.1	1158248..1159339	Galactose metabolism	COG1087
CAE19668.1_1395	CUST_6916_PI442190954	MED4	HLI	CAE19668.1_1395	AGCAGTAGTGCCGCCGTTTATGGTAACAATCAAAATTGTCCCATCAAAGAAGATAGTTCA	galE	UDP-glucose 4-epimerase	PMM1209	CAE19668.1	1158248..1159339	Galactose metabolism	COG1087
CAE19668.1_1395	CUST_6917_PI442190954	MED4	HLI	CAE19668.1_1395	TGAGTTCTCAGAGAGGAGGATAGGAGATTGTGGAGTGCTTTTTGCAGATAGTAGATTGTC	galE	UDP-glucose 4-epimerase	PMM1209	CAE19668.1	1158248..1159339	Galactose metabolism	COG1087
CAE19668.1_1395	CUST_6918_PI442190954	MED4	HLI	CAE19668.1_1395	TGGAAGGCAAGAAAAACATTAGATGATATATGTAGGGATGGTTGGAGATGGGGTTTGAAA	galE	UDP-glucose 4-epimerase	PMM1209	CAE19668.1	1158248..1159339	Galactose metabolism	COG1087
CAE19668.1_1395	CUST_6919_PI442190954	MED4	HLI	CAE19668.1_1395	AATCCTGTAGGAGCTCATCACTCTGGAATAATAGGAGAAAATCCTATCTCAAACAAAAAT	galE	UDP-glucose 4-epimerase	PMM1209	CAE19668.1	1158248..1159339	Galactose metabolism	COG1087
CAE19668.1_1395	CUST_6920_PI442190954	MED4	HLI	CAE19668.1_1395	AGATGGTCACGAAAAAGCATTACGCTTGTTATTAAATTCAAAACATAATTTGATCAAAGT	galE	UDP-glucose 4-epimerase	PMM1209	CAE19668.1	1158248..1159339	Galactose metabolism	COG1087
CAE19720.1_1448	CUST_7222_PI442190954	MED4	HLI	CAE19720.1_1448	TTGGTATGGACACAAGGATTGGAGAAAAATTCCTAAATGCAGGACCTGGATATGGAGGAA	ugd	UDP-glucose 6-dehydrogenase/nucleotide sugar del	PMM1261	CAE19720.1	1214864..1216288	Amino sugar and nucleotide sugar r	COG1004
CAE19720.1_1448	CUST_7223_PI442190954	MED4	HLI	CAE19720.1_1448	GGAGAGGGCTCTTGGACTCTTTGCAAATCAATAAAGACATCGCTATAGATGCTGATGCA	ugd	UDP-glucose 6-dehydrogenase/nucleotide sugar del	PMM1261	CAE19720.1	1214864..1216288	Amino sugar and nucleotide sugar r	COG1004
CAE19720.1_1448	CUST_7224_PI442190954	MED4	HLI	CAE19720.1_1448	ACGACACTAGAGAATCTCCTGCTATTCAAATATGTAAAGACCTAATTGAAGAAGGCGCAG	ugd	UDP-glucose 6-dehydrogenase/nucleotide sugar del	PMM1261	CAE19720.1	1214864..1216288	Amino sugar and nucleotide sugar r	COG1004
CAE19720.1_1448	CUST_7225_PI442190954	MED4	HLI	CAE19720.1_1448	TGGCCTAAATGAAGTTGCTTCATATTGGCAAAAGGTAGTAGATTTAAATGTTTGGCAACA	ugd	UDP-glucose 6-dehydrogenase/nucleotide sugar del	PMM1261	CAE19720.1	1214864..1216288	Amino sugar and nucleotide sugar r	COG1004

CAE19720.1_1448	CUST_7226_PI442190954	MED4	HLI	CAE19720.1_1448	AGCACAAAGAATAAGTTCTATTAATTCGATATCTGCAATATGTGAAACTACAGGTGCTGA	ugd	UDP-glucose 6-dehydrogenase/nucleotide sugar del	PMM1261	CAE19720.1	1214864..1216288	Amino sugar and nucleotide sugar r	COG1004
CAE19720.1_1448	CUST_7227_PI442190954	MED4	HLI	CAE19720.1_1448	TTGGTCTGATATCTCTAGATTAATGAGATCACCTAAATGGTTATTTGATACAAGGGGGAT	ugd	UDP-glucose 6-dehydrogenase/nucleotide sugar del	PMM1261	CAE19720.1	1214864..1216288	Amino sugar and nucleotide sugar r	COG1004
CAE19747.1_1475	CUST_7368_PI442190954	MED4	HLI	CAE19747.1_1475	CTTATCGCGATCATGTTCATGCGCTTAGTGCAGGTGTTCCATCTTTTGAAGTAATGAGTG	pdhA	Pyruvate dehydrogenase E1 component alpha subuni	PMM1288	CAE19747.1	1239069..1240106	Glycolysis/Krebs cycle	COG1071
CAE19747.1_1475	CUST_7369_PI442190954	MED4	HLI	CAE19747.1_1475	TCAGAGGTCATTCTTTAGCCGACCCAGATGAATTAAGAGCTGAGGAAGAGAAGGAATTCT	pdhA	Pyruvate dehydrogenase E1 component alpha subuni	PMM1288	CAE19747.1	1239069..1240106	Glycolysis/Krebs cycle	COG1071
CAE19747.1_1475	CUST_7370_PI442190954	MED4	HLI	CAE19747.1_1475	GAAAAGCATCAGCTTTCGGTATGCATGGTGAAGAAGTTGATGGGATGGATGTATTAGCTG	pdhA	Pyruvate dehydrogenase E1 component alpha subuni	PMM1288	CAE19747.1	1239069..1240106	Glycolysis/Krebs cycle	COG1071
CAE19747.1_1475	CUST_7371_PI442190954	MED4	HLI	CAE19747.1_1475	AGAGCTGTTGAAAGAGCAAGAGCTGGAGAGGGTCCAACATTACTGGAATGTTTAACTTAC	pdhA	Pyruvate dehydrogenase E1 component alpha subuni	PMM1288	CAE19747.1	1239069..1240106	Glycolysis/Krebs cycle	COG1071
CAE19747.1_1475	CUST_7372_PI442190954	MED4	HLI	CAE19747.1_1475	CAACAGGATGCAGTAAAGGCAGAGGTGGTTCTATGCATTTGTTCTCAAAGGAACATCATT	pdhA	Pyruvate dehydrogenase E1 component alpha subuni	PMM1288	CAE19747.1	1239069..1240106	Glycolysis/Krebs cycle	COG1071
CAE19747.1_1475	CUST_7373_PI442190954	MED4	HLI	CAE19747.1_1475	AAAATAATAAATGGGCTATTGGCATGGCTCATGACAGAGCCACAAGCAACCCAGAAATAT	pdhA	Pyruvate dehydrogenase E1 component alpha subuni	PMM1288	CAE19747.1	1239069..1240106	Glycolysis/Krebs cycle	COG1071
CAE19768.1_1497	CUST_7492_PI442190954	MED4	HLI	CAE19768.1_1497	AATTGCAGGAGCTCCACTTCAAGAAGCCTTTAGAAATGCTGATGATGTTTTAAGGATGGG	ftsZ	cell division protein FtsZ	PMM1309	CAE19768.1	1262202..1263317	Cell Division	COG0206
CAE19768.1_1497	CUST_7493_PI442190954	MED4	HLI	CAE19768.1_1497	GTTGGGATAGTAACCAAGCCATTTTCATTTGAAGGTAAAAGGAGAATGCGTCAGGCAGAA	ftsZ	cell division protein FtsZ	PMM1309	CAE19768.1	1262202..1263317	Cell Division	COG0206
CAE19768.1_1497	CUST_7494_PI442190954	MED4	HLI	CAE19768.1_1497	GATTAGTTAACGTTGATTTTGCTGATGTTAGGTCTGTAATGACTGAAGCTGGCACTGCCC	ftsZ	cell division protein FtsZ	PMM1309	CAE19768.1	1262202..1263317	Cell Division	COG0206
CAE19768.1_1497	CUST_7495_PI442190954	MED4	HLI	CAE19768.1_1497	AATGGAAGGCGAAATACAGGTAACTGTTATTGCAACAGGTTTCGAAACCAATCAACCGTT	ftsZ	cell division protein FtsZ	PMM1309	CAE19768.1	1262202..1263317	Cell Division	COG0206
CAE19768.1_1497	CUST_7496_PI442190954	MED4	HLI	CAE19768.1_1497	ATAGGTATTGGTTCTGGTAGATCTAGAGCATTAGAGGCTGCTCAAGCCGCAATGAATAGT	ftsZ	cell division protein FtsZ	PMM1309	CAE19768.1	1262202..1263317	Cell Division	COG0206
CAE19768.1_1497	CUST_7497_PI442190954	MED4	HLI	CAE19768.1_1497	AGAAGCGGCAAGAATTGATGGAGCTAAAGGTTGTGTGATAAATATTACAGGTGGGAAAGA	ftsZ	cell division protein FtsZ	PMM1309	CAE19768.1	1262202..1263317	Cell Division	COG0206
CAE19782.1_1512	CUST_7569_PI442190954	MED4	HLI	CAE19782.1_1512	CCATATGCAAAAAAAGCTAGACGGCCATCTGCGAAAGCGGAACCAATTAAACTTCAATTT	glcH	GPH family sugar transporter	PMM1323	CAE19782.1	1274850..1276199	GlcH sugar transporter	COG2211
CAE19782.1_1512	CUST_7570_PI442190954	MED4	HLI	CAE19782.1_1512	CTTGGTCACTACTTCCTGATGCAATAGATGAAGATCCAGAAAAACCCGCAGGCCTTTATA	glcH	GPH family sugar transporter	PMM1323	CAE19782.1	1274850..1276199	GlcH sugar transporter	COG2211
CAE19782.1_1512	CUST_7571_PI442190954	MED4	HLI	CAE19782.1_1512	ACTTTGGTGTGCCTTGCAGTTAATGCAAACAGTCGCGTTAATTTATGTAGAGGATGTTCT	glcH	GPH family sugar transporter	PMM1323	CAE19782.1	1274850..1276199	GlcH sugar transporter	COG2211
CAE19782.1_1512	CUST_7572_PI442190954	MED4	HLI	CAE19782.1_1512	TGTGCCTTCAGAAATTGCTAACTGGGTTCCAATACCATTCCAAATAAGTGCCCTAATAGG	glcH	GPH family sugar transporter	PMM1323	CAE19782.1	1274850..1276199	GlcH sugar transporter	COG2211
CAE19782.1_1512	CUST_7573_PI442190954	MED4	HLI	CAE19782.1_1512	TCCTATTCATAATGTTGATTGTCATAATCTGTTTAATTGGAATAGGAGCTTCAACCGCGT	glcH	GPH family sugar transporter	PMM1323	CAE19782.1	1274850..1276199	GlcH sugar transporter	COG2211
CAE19782.1_1512	CUST_7574_PI442190954	MED4	HLI	CAE19782.1_1512	ACTGCTTGGATGGTTCTTATTCAGAAAATTGGCATTGGACTAAGTGTTCAAGTATTAGG	glcH	GPH family sugar transporter	PMM1323	CAE19782.1	1274850..1276199	GlcH sugar transporter	COG2211
CAE19801.1_1531	CUST_7680_PI442190954	MED4	HLI	CAE19801.1_1531	GGTTATTTTCAGGATTCAATAATTCTGGCAACTGGCGCGACTGGTACGGGTAAAACTATG	kaiC	possible circadian clock protein KaiC	PMM1342	CAE19801.1	1293980..1295509	Circadian Rhythm	COG0467
CAE19801.1_1531	CUST_7681_PI442190954	MED4	HLI	CAE19801.1_1531	ACCTAAGAGATTGGCAATTGACTCTCTCTCTGCATTGGCTAGAGGAGTAAGTTTGAATGC	kaiC	possible circadian clock protein KaiC	PMM1342	CAE19801.1	1293980..1295509	Circadian Rhythm	COG0467
CAE19801.1_1531	CUST_7682_PI442190954	MED4	HLI	CAE19801.1_1531	GGGGAGATGGCTAGAGCCATAAATGTTTTTAAAATGAGAGGCTCATGGCATGATAAAAGG	kaiC	possible circadian clock protein KaiC	PMM1342	CAE19801.1	1293980..1295509	Circadian Rhythm	COG0467
CAE19801.1_1531	CUST_7683_PI442190954	MED4	HLI	CAE19801.1_1531	GCGGGTTTCTTCACAAATACTGCAGAAGAATTTATGGGAAGTCATTCTATTACCGACTCT	kaiC	possible circadian clock protein KaiC	PMM1342	CAE19801.1	1293980..1295509	Circadian Rhythm	COG0467
CAE19801.1_1531	CUST_7684_PI442190954	MED4	HLI	CAE19801.1_1531	AGCGGTGCACCTCATAGAGTTATTTCTGATCAGAGTATTCCAAACGTTTTTAAAGGAATT	kaiC	possible circadian clock protein KaiC	PMM1342	CAE19801.1	1293980..1295509	Circadian Rhythm	COG0467
CAE19801.1_1531	CUST_7685_PI442190954	MED4	HLI	CAE19801.1_1531	TGCTTACAACAATAAAGAAAGAGCCATACTTTTTGCCTATGAAGAATCTAGGGCTCAACT	kaiC	possible circadian clock protein KaiC	PMM1342	CAE19801.1	1293980..1295509	Circadian Rhythm	COG0467
CAE19802.1_1532	CUST_7686_PI442190954	MED4	HLI	CAE19802.1_1532	TTTTGATGAATTATCAGAGAGTGAATATAGTGGAGGTACAAAAAATTAA	kaiB	Circadian clock protein KaiB	PMM1343	CAE19802.1	1295572..1295895	Circadian Rhythm	COG4251
CAE19802.1_1532	CUST_7687_PI442190954	MED4	HLI	CAE19802.1_1532	TTGGTGATTTGTCAGATAGAGAAAAGGTTTTAATTGGATTAGATTTACTTTTTGATGAAT	kaiB	Circadian clock protein KaiB	PMM1343	CAE19802.1	1295572..1295895	Circadian Rhythm	COG4251
CAE19802.1_1532	CUST_7688_PI442190954	MED4	HLI	CAE19802.1_1532	GCTGAAGAAGATAAGATTTTGGCAACCCCAACTCTTGCAAAGATTTTACCTCCTCCTGTT	kaiB	Circadian clock protein KaiB	PMM1343	CAE19802.1	1295572..1295895	Circadian Rhythm	COG4251
CAE19802.1_1532	CUST_7689_PI442190954	MED4	HLI	CAE19802.1_1532	AGAGAATGAATTTAAAGGAGTTTATGCTCTAAAAGTAATAGATGTTCTTAAACAACCTCA	kaiB	Circadian clock protein KaiB	PMM1343	CAE19802.1	1295572..1295895	Circadian Rhythm	COG4251
CAE19802.1_1532	CUST_7690_PI442190954	MED4	HLI	CAE19802.1_1532	AACTTTACGTAGCAGGTAACACTCCCAATTCAATGAGAGCTTTGAATACTCTTAAAGAAA	kaiB	Circadian clock protein KaiB	PMM1343	CAE19802.1	1295572..1295895	Circadian Rhythm	COG4251
CAE19897.1_1678	CUST_8232_PI442190954	MED4	HLI	CAE19897.1_1678	AGGTGCAAGAATGAGAGTCGGCTTATCGGCATTAACTATGGCTGAACATTTTAGAGATGT	atpB	ATP synthase beta subunit	PMM1438	CAE19897.1	1375858..1377318	Energy Metabolism	COG0055
CAE19897.1_1678	CUST_8233_PI442190954	MED4	HLI	CAE19897.1_1678	TACATCAATTCAAGCAGTATATGTTCCAGCGGATGATTTAACTGATCCTGCTCCCGCTAC	atpB	ATP synthase beta subunit	PMM1438	CAE19897.1	1375858..1377318	Energy Metabolism	COG0055
CAE19897.1_1678	CUST_8234_PI442190954	MED4	HLI	CAE19897.1_1678	CTCTGTTAGGAAGAATGCCTTCTGCTGTTGGGTATCAACCAACTCTAGGAACTGATGTTG	atpB	ATP synthase beta subunit	PMM1438	CAE19897.1	1375858..1377318	Energy Metabolism	COG0055
CAE19897.1_1678	CUST_8235_PI442190954	MED4	HLI	CAE19897.1_1678	GTGATGAACATTACAAGACAGCTCGCGCGGTTCAATCAACCTTACAAAGATACAAAGAGC	atpB	ATP synthase beta subunit	PMM1438	CAE19897.1	1375858..1377318	Energy Metabolism	COG0055
CAE19897.1_1678	CUST_8236_PI442190954	MED4	HLI	CAE19897.1_1678	GTATTTATCCAGCAGTTGATCCTCTAGATTCAACCAGCACGATGCTTCAACCCTCAGTAG	atpB	ATP synthase beta subunit	PMM1438	CAE19897.1	1375858..1377318	Energy Metabolism	COG0055
CAE19897.1_1678	CUST_8237_PI442190954	MED4	HLI	CAE19897.1_1678	AGCTGGTGAACTGGATGATTTACCCGAACAAGCTTTTTACTTGGTAGGTAATATCGATGA	atpB	ATP synthase beta subunit	PMM1438	CAE19897.1	1375858..1377318	Energy Metabolism	COG0055
CAE19909.1_1690	CUST_8295_PI442190954	MED4	HLI	CAE19909.1_1690	CCCAGTTGTGCAAACATTGTTGCCATTAGATCCTCAAGGAATTGCTGAGGATAATGATGA	atpG	ATP synthase gamma subunit	PMM1450	CAE19909.1	1386093..1387043	Energy Metabolism	COG0224
CAE19909.1_1690	CUST_8296_PI442190954	MED4	HLI	CAE19909.1_1690	TCAGCTGCTTCAGAACTTGCCTGTAGAATGACAGCTATGAATAATGCGAGTGATAATGCT	atpG	ATP synthase gamma subunit	PMM1450	CAE19909.1	1386093..1387043	Energy Metabolism	COG0224
CAE19909.1_1690	CUST_8297_PI442190954	MED4	HLI	CAE19909.1_1690	AAGCAAGACAAGCTGCTATAACTCAGGAAATACTTGAAGTAGTTGGAGGTTCAGCAGTCT	atpG	ATP synthase gamma subunit	PMM1450	CAE19909.1	1386093..1387043	Energy Metabolism	COG0224
CAE19909.1_1690	CUST_8298_PI442190954	MED4	HLI	CAE19909.1_1690	AGGAACGTAAAAAAAATCTCCTTAGTTTGCATAACTGCCGATAGAGGTTTATGTGGGGGA	atpG	ATP synthase gamma subunit	PMM1450	CAE19909.1	1386093..1387043	Energy Metabolism	COG0224
CAE19909.1_1690	CUST_8299_PI442190954	MED4	HLI	CAE19909.1_1690	ACGCAGAGTTGATCAAACAAGGTTATGAACCTAACTTGATTTTAGTTGGCAAAAAGGCAA	atpG	ATP synthase gamma subunit	PMM1450	CAE19909.1	1386093..1387043	Energy Metabolism	COG0224
CAE19909.1_1690	CUST_8300_PI442190954	MED4	HLI	CAE19909.1_1690	AGCTAGAACAAGTACCAACAGCTATAGATTCTGAAGGAATAACTAATGATGTCTTAGCCG	atpG	ATP synthase gamma subunit	PMM1450	CAE19909.1	1386093..1387043	Energy Metabolism	COG0224
CAE19910.1_1691	CUST_8301_PI442190954	MED4	HLI	CAE19910.1_1691	CAGGTGTTAAGGGGCTTATTGACGAGGTACCTGTTGAAGATGTAACAAAATTTGCTGCTG	atpA	ATP synthase alpha subunit	PMM1451	CAE19910.1	1387064..1388581	Energy Metabolism	COG0056
CAE19910.1_1691	CUST_8302_PI442190954	MED4	HLI	CAE19910.1_1691	AGGCTTAAGACCTGCAATAAATGTTGGTATTTCAGTTAGTAGAGTTGGTGGAGCTGCACA	atpA	ATP synthase alpha subunit	PMM1451	CAE19910.1	1387064..1388581	Energy Metabolism	COG0056
CAE19910.1_1691	CUST_8303_PI442190954	MED4	HLI	CAE19910.1_1691	ACAAGCTTACAGACAAATGTCTCTTCTTTTGAGAAGGCCACCAGGAAGAGAAGCTTATCC	atpA	ATP synthase alpha subunit	PMM1451	CAE19910.1	1387064..1388581	Energy Metabolism	COG0056
CAE19910.1_1691	CUST_8304_PI442190954	MED4	HLI	CAE19910.1_1691	GATATGGGTGGTGGATCAATGACAGCTTTGCCAATTATTGAAACTCAAGCTGGAGATGTC	atpA	ATP synthase alpha subunit	PMM1451	CAE19910.1	1387064..1388581	Energy Metabolism	COG0056
CAE19910.1_1691	CUST_8305_PI442190954	MED4	HLI	CAE19910.1_1691	ATTTGATGAATTAGCAGCATTTTCTCAGTTCGCTTCTGATCTCGATGAGGCTACTCAGCA	atpA	ATP synthase alpha subunit	PMM1451	CAE19910.1	1387064..1388581	Energy Metabolism	COG0056
CAE19910.1_1691	CUST_8306_PI442190954	MED4	HLI	CAE19910.1_1691	TACACTGGAGCTGCAATAGCTGAGCATTTCATGTACCAAGGCAAAGCTACTCTTGTTATT	atpA	ATP synthase alpha subunit	PMM1451	CAE19910.1	1387064..1388581	Energy Metabolism	COG0056
CAE19925.1_1707	CUST_8387_PI442190954	MED4	HLI	CAE19925.1_1707	TAATAATTCCCAAAAATGAACCAGGGAGCTCAATCATGCCTGGGAAAGTTAATCCAACCC	fumC	Fumarate hydratase class II	PMM1466	CAE19925.1	1399018..1400403	Krebs cycle and related genes	COG0114
CAE19925.1_1707	CUST_8388_PI442190954	MED4	HLI	CAE19925.1_1707	AATGGTATGCACTCAAGTAATGGGTTTTGAATACGCAGTATCAATAGCTAATGCCAGCGG	fumC	Fumarate hydratase class II	PMM1466	CAE19925.1	1399018..1400403	Krebs cycle and related genes	COG0114
CAE19925.1_1707	CUST_8389_PI442190954	MED4	HLI	CAE19925.1_1707	TGGCATCTCATGATCGATTAGCCCAAGTAATGGGTCAAATAAAAATCCTAGCAAGTGCAT	fumC	Fumarate hydratase class II	PMM1466	CAE19925.1	1399018..1400403	Krebs cycle and related genes	COG0114
CAE19925.1_1707	CUST_8390_PI442190954	MED4	HLI	CAE19925.1_1707	ACTGCATTGGTTCCCAAAATAGGTTATGAAAAAGCAGCAGAAATTGCGAATCTTGCTTTT	fumC	Fumarate hydratase class II	PMM1466	CAE19925.1	1399018..1400403	Krebs cycle and related genes	COG0114
CAE19925.1_1707	CUST_8391_PI442190954	MED4	HLI	CAE19925.1_1707	ACCACTAGGTGGTACGGCAGTTGGTACAGGTATTAATTGTCCAAAAGATTTTTCAAAAGA	fumC	Fumarate hydratase class II	PMM1466	CAE19925.1	1399018..1400403	Krebs cycle and related genes	COG0114
CAE19925.1_1707	CUST_8392_PI442190954	MED4	HLI	CAE19925.1_1707	ACTGAATCATGCGATAAGTAACTTCAGACTAAAATTAGTTGAAGGGATACAACCAAACCC	fumC	Fumarate hydratase class II	PMM1466	CAE19925.1	1399018..1400403	Krebs cycle and related genes	COG0114
CAE19971.1_1754	CUST_8641_PI442190954	MED4	HLI	CAE19971.1_1754	ACCTGTCTTTATGGAGCAACAGGAGGCAAGTTATTTGCTTTAGGTAAATCCGGAGAAAGA	glsF	Ferredoxin-dependent glutamate synthase	PMM1512	CAE19971.1	1450922..1455487	Nitrogen Metabolism	COG0069
CAE19971.1_1754	CUST_8642_PI442190954	MED4	HLI	CAE19971.1_1754	TCACAGAAGGTTCGGGCGACCATTGTTGTGAATATATGACTGGTGGCAAGATAGTAATTT	glsF	Ferredoxin-dependent glutamate synthase	PMM1512	CAE19971.1	1450922..1455487	Nitrogen Metabolism	COG0069
CAE19971.1_1754	CUST_8643_PI442190954	MED4	HLI	CAE19971.1_1754	AGAAATCAAGAACACTGACAGAAGTGTATGTGCAAAAATATCAGGTGAAATTGCGGGGCT	glsF	Ferredoxin-dependent glutamate synthase	PMM1512	CAE19971.1	1450922..1455487	Nitrogen Metabolism	COG0069
CAE19971.1_1754	CUST_8644_PI442190954	MED4	HLI	CAE19971.1_1754	TGGATTTAATGGTGAACTAAATCTCAATTTCAAAGGATACGCAGGGCAGAGTTTTGGTGC	glsF	Ferredoxin-dependent glutamate synthase	PMM1512	CAE19971.1	1450922..1455487	Nitrogen Metabolism	COG0069
CAE19971.1_1754	CUST_8645_PI442190954	MED4	HLI	CAE19971.1_1754	TGTTTGCAAAGGAATGAATGGAGGTGTACTTACTATTGTTCCTCCCCAAGTAGATGAGAA	glsF	Ferredoxin-dependent glutamate synthase	PMM1512	CAE19971.1	1450922..1455487	Nitrogen Metabolism	COG0069
CAE19971.1_1754	CUST_8646_PI442190954	MED4	HLI	CAE19971.1_1754	AGGTTCAACAGGAAGAAATATTGGAGCAGGAATGACTGGTGGAATAGCTTATATACTTGA	glsF	Ferredoxin-dependent glutamate synthase	PMM1512	CAE19971.1	1450922..1455487	Nitrogen Metabolism	COG0069
CAE19982.1_1765	CUST_8695_PI442190954	MED4	HLI	CAE19982.1_1765	CCATGTATCTTGCAATGTTCTGGGCTTTGAACTTGATAGCATGGGTAACTTTCTACTGGC	psaB	Photosystem I P700 chlorophyll A apoprotein A2 Psa	PMM1523	CAE19982.1	1461905..1464133	Photosystem I	COG2885
CAE19982.1_1765	CUST_8696_PI442190954	MED4	HLI	CAE19982.1_1765	AGCAAGTCTGGCGGCAAACTCTTTGCCAGGAAACCATTACTGGATGGATTTAATTAATAG	psaB	Photosystem I P700 chlorophyll A apoprotein A2 Psa	PMM1523	CAE19982.1	1461905..1464133	Photosystem I	COG2885
CAE19982.1_1765	CUST_8697_PI442190954	MED4	HLI	CAE19982.1_1765	AGAGATAAACCAGTTGCACTTTCAATTGTTCAAGCAAGACTAGTTGGACTGGCGCATTTT	psaB	Photosystem I P700 chlorophyll A apoprotein A2 Psa	PMM1523	CAE19982.1	1461905..1464133	Photosystem I	COG2885
CAE19982.1_1765	CUST_8698_PI442190954	MED4	HLI	CAE19982.1_1765	GTCATCTTGTATGGGCGACTGGATTCATGTTCTTGATATCTTGGAGAGGATACTGGCAAG	psaB	Photosystem I P700 chlorophyll A apoprotein A2 Psa	PMM1523	CAE19982.1	1461905..1464133	Photosystem I	COG2885
CAE19982.1_1765	CUST_8699_PI442190954	MED4	HLI	CAE19982.1_1765	TGGCCCAGCAGATTTCTTAGTTCATCATGCTATAGCTCTTGGACTTCATACAACTGCATT	psaB	Photosystem I P700 chlorophyll A apoprotein A2 Psa	PMM1523	CAE19982.1	1461905..1464133	Photosystem I	COG2885
CAE19982.1_1765	CUST_8700_PI442190954	MED4	HLI	CAE19982.1_1765	ACTCTGCTCAGCTAATTAATGGGTACAACCCTTTCGGTGTTAATTCACTTTCTGTCTGGG	psaB	Photosystem I P700 chlorophyll A apoprotein A2 Psa	PMM1523	CAE19982.1	1461905..1464133	Photosystem I	COG2885
CAE19983.1_1766	CUST_8701_PI442190954	MED4	HLI	CAE19983.1_1766	TATGGTTCAGCTATAAGTATGTATGGCCTGATGTTTTTGGAGCACACTTTATCTGGGCA	psaA	Photosystem I P700 chlorophyll A apoprotein A1 Psa	PMM1524	CAE19983.1	1464162..1466465	Photosystem I	COG2885
CAE19983.1_1766	CUST_8702_PI442190954	MED4	HLI	CAE19983.1_1766	GAATCAATTGTTTGGGCTCATAACAAACTGAAAGTTGCTCCAACAATTCAACCAAGGGCA	psaA	Photosystem I P700 chlorophyll A apoprotein A1 Psa	PMM1524	CAE19983.1	1464162..1466465	Photosystem I	COG2885
CAE19983.1_1766	CUST_8703_PI442190954	MED4	HLI	CAE19983.1_1766	CACGATTGATTCCTGATAAAGCATCCTTAGGATTTAGATTCCCATGTGATGGCCCTGGTA	psaA	Photosystem I P700 chlorophyll A apoprotein A1 Psa	PMM1524	CAE19983.1	1464162..1466465	Photosystem I	COG2885
CAE19983.1_1766	CUST_8704_PI442190954	MED4	HLI	CAE19983.1_1766	GTTGGGACTTCAATATTGGCCGGTACAGCTGAAGCCTTACCTCATAAAGCTATAAGTGAA	psaA	Photosystem I P700 chlorophyll A apoprotein A1 Psa	PMM1524	CAE19983.1	1464162..1466465	Photosystem I	COG2885
CAE19983.1_1766	CUST_8705_PI442190954	MED4	HLI	CAE19983.1_1766	TAACGGAAGTTTAGTTGAAGTAGGTGGAAAGGTTGCTATTGCTCCAATCCCCTTAGGAAC	psaA	Photosystem I P700 chlorophyll A apoprotein A1 Psa	PMM1524	CAE19983.1	1464162..1466465	Photosystem I	COG2885
CAE19983.1_1766	CUST_8706_PI442190954	MED4	HLI	CAE19983.1_1766	ATTACTCAAGGTCGTGCAGTTGGTGTTACTCACTTCTTAGTGGGTGGTATTGCTACTACT	psaA	Photosystem I P700 chlorophyll A apoprotein A1 Psa	PMM1524	CAE19983.1	1464162..1466465	Photosystem I	COG2885
CAE20034.1_1817	CUST_8978_PI442190954	MED4	HLI	CAE20034.1_1817	ACAGAGTAGATTTCTTTTGCCAAGCTGGTTTGGAGTGGGTACGGCTTTATCTGTAGAATT	ppc	Phosphoenolpyruvate carboxylase	PMM1575	CAE20034.1	1502738..1505707	Krebs cycle and related genes	COG2352
CAE20034.1_1817	CUST_8979_PI442190954	MED4	HLI	CAE20034.1_1817	GCAAGAAGGAAAAAAGGCGCAAAGGATTTATCAAGTTTAAGGGCAATCCCTTGGGTATTT	ppc	Phosphoenolpyruvate carboxylase	PMM1575	CAE20034.1	1502738..1505707	Krebs cycle and related genes	COG2352
CAE20034.1_1817	CUST_8980_PI442190954	MED4	HLI	CAE20034.1_1817	TGGTTAACAACAGACTTGATGCTACACCAGAATGGAATGACTTAATGACTAGGTTGGCAG	ppc	Phosphoenolpyruvate carboxylase	PMM1575	CAE20034.1	1502738..1505707	Krebs cycle and related genes	COG2352
CAE20034.1_1817	CUST_8981_PI442190954	MED4	HLI	CAE20034.1_1817	AGTCGTAGTGAATTATTAAGAGGAGCACTTTTAACAATTAATGGAATTGCGGCAGGGATG	ppc	Phosphoenolpyruvate carboxylase	PMM1575	CAE20034.1	1502738..1505707	Krebs cycle and related genes	COG2352
CAE20034.1_1817	CUST_8982_PI442190954	MED4	HLI	CAE20034.1_1817	AGCGAAATATTACGTTGACACACTTGGGAGTAAAGAAAATTCAAAGTCGTTTGAGGAGAT	ppc	Phosphoenolpyruvate carboxylase	PMM1575	CAE20034.1	1502738..1505707	Krebs cycle and related genes	COG2352
CAE20034.1_1817	CUST_8983_PI442190954	MED4	HLI	CAE20034.1_1817	TGGGATTTTTACAAGTCTCACTTTTAAGAAGACTTCGAGATCAAACAAGACAACCTCCAA	ppc	Phosphoenolpyruvate carboxylase	PMM1575	CAE20034.1	1502738..1505707	Krebs cycle and related genes	COG2352
CAE20065.1_1848	CUST_9153_PI442190954	MED4	HLI	CAE20065.1_1848	GGGAGCTCTGAAGTTGAAGGAAATTAGTTATATTAATGCTGCAGGTTATCCTGCTGGGGA	glmS	Glutamine--fructose-6-phosphatetransaminase(ison	PMM1606	CAE20065.1	1535851..1537746	Amino Acid metabolism	COG0449
CAE20065.1_1848	CUST_9154_PI442190954	MED4	HLI	CAE20065.1_1848	AGTAATGCTCAAGAAGCAAAAGCGAGAGATGCTTATTTAATTGGTGTTGCCCCTCAATGT	glmS	Glutamine--fructose-6-phosphatetransaminase(ison	PMM1606	CAE20065.1	1535851..1537746	Amino Acid metabolism	COG0449
CAE20065.1_1848	CUST_9155_PI442190954	MED4	HLI	CAE20065.1_1848	AGTGCTGGAATTGAAATTGGTGTTGCTGCTACAAAAACTTTCTTTGCGCAACTTCTTTCT	glmS	Glutamine--fructose-6-phosphatetransaminase(ison	PMM1606	CAE20065.1	1535851..1537746	Amino Acid metabolism	COG0449
CAE20065.1_1848	CUST_9156_PI442190954	MED4	HLI	CAE20065.1_1848	TCACATTGCTTCTCATAGAGGACTTGATGTTGATCAACCAAGGAATCTAGCTAAAAGCGT	glmS	Glutamine--fructose-6-phosphatetransaminase(ison	PMM1606	CAE20065.1	1535851..1537746	Amino Acid metabolism	COG0449
CAE20065.1_1848	CUST_9157_PI442190954	MED4	HLI	CAE20065.1_1848	ATGACCATACCTTCAACAAATGAATGGATTTCACCTCTTCTTGCGATTGTTCCTCTTCAA	glmS	Glutamine--fructose-6-phosphatetransaminase(ison	PMM1606	CAE20065.1	1535851..1537746	Amino Acid metabolism	COG0449
CAE20065.1_1848	CUST_9158_PI442190954	MED4	HLI	CAE20065.1_1848	TGGGCCAATAGCTTTACTTGATAAAAAAGTACCTGTAATTTCAATAGCTACTCCAGGGCA	glmS	Glutamine--fructose-6-phosphatetransaminase(ison	PMM1606	CAE20065.1	1535851..1537746	Amino Acid metabolism	COG0449
WP_012006816.1_12	CUST_67_PI442190926	MIT9215	HLII	WP_012006816.1_12	ACAGGAAGCAGCTTAATGCCGCAGAAAAAAAATCCTGATGTTCCTGAATTGATAAGAGGT	argH	Argininosuccinate lyase		WP_012006816.:	14488..15867	Amino Acid metabolism	COG0165
WP_012006816.1_12	CUST_68_PI442190926	MIT9215	HLII	WP_012006816.1_12	GAAGAGTGTATGGACATCTTCAGGCATTGTTAACTATGGTAAAAGGTGTGCCGCTTTCAT	argH	Argininosuccinate lyase		WP_012006816.:	14488..15867	Amino Acid metabolism	COG0165
WP_012006816.1_12	CUST_69_PI442190926	MIT9215	HLII	WP_012006816.1_12	TGGCAGATTACTTAGTTGGTAAAGACGTTCCTTTTAGGACTGCTTATCAAGTTGTTGGCG	argH	Argininosuccinate lyase		WP_012006816.:	14488..15867	Amino Acid metabolism	COG0165
WP_012006816.1_12	CUST_70_PI442190926	MIT9215	HLII	WP_012006816.1_12	ACCTCATAACGTCGTTAAATCAAGAAATAGTGAAGGTGGTACAGGTTTTGTTCAGGTAGA	argH	Argininosuccinate lyase		WP_012006816.:	14488..15867	Amino Acid metabolism	COG0165
WP_012006816.1_12	CUST_71_PI442190926	MIT9215	HLII	WP_012006816.1_12	TGCAGTTAGTGATAGAGAGATTTTTGTATTGAGTTTGTTTCTGCTTCTGCTTTGTCAATGTC	argH	Argininosuccinate lyase		WP_012006816.:	14488..15867	Amino Acid metabolism	COG0165
WP_012006816.1_12	CUST_72_PI442190926	MIT9215	HLII	WP_012006816.1_12	AAAATTGGTGAGTTTAAGAAATTTCATCCCGAATTTGATGAGGATGTTTTTTCGGATCTT	argH	Argininosuccinate lyase		WP_012006816.:	14488..15867	Amino Acid metabolism	COG0165
WP_012007014.1_220	CUST_1213_PI442190926	MIT9215	HLII	WP_012007014.1_220	GAAGAGGAATTGGGAAATCAAGCAAGATTCGCTGGAGCTTTAGGTTTAGGTCCCAAAAAT	eno	Enolase		WP_012007014.:	208199..209491	Glycolysis/Gluconeogenesis	COG0148
WP_012007014.1_220	CUST_1214_PI442190926	MIT9215	HLII	WP_012007014.1_220	GCAGATTTATCTGTTGCTACAAGATCGGGTCAGATAAAGACAGGCTCTTTGAGCAGAAGT	eno	Enolase		WP_012007014.:	208199..209491	Glycolysis/Gluconeogenesis	COG0148
WP_012007014.1_220	CUST_1215_PI442190926	MIT9215	HLII	WP_012007014.1_220	TCCAATAGTTTCAATAGAGGATGGTTTAGCTGAGGATGATTGGGAGGGTTGGTCAGAATT	eno	Enolase		WP_012007014.:	208199..209491	Glycolysis/Gluconeogenesis	COG0148
WP_012007014.1_220	CUST_1216_PI442190926	MIT9215	HLII	WP_012007014.1_220	GGACTTATTATTAGAAGCAATTCAAAAAGCCGGATTTAAGCCCGGTGAGCAGGTGTCTTT	eno	Enolase		WP_012007014.:	208199..209491	Glycolysis/Gluconeogenesis	COG0148
WP_012007014.1_220	CUST_1217_PI442190926	MIT9215	HLII	WP_012007014.1_220	AAATCATTACTTGATCAAAAAGGTCTATCTACTGCCGTAGGCGATGAGGGTGGATTTGCA	eno	Enolase		WP_012007014.:	208199..209491	Glycolysis/Gluconeogenesis	COG0148
WP_012007014.1_220	CUST_1218_PI442190926	MIT9215	HLII	WP_012007014.1_220	GCTCACGCCCCAAATAGTCTTGATTTTCAGGAATTTATGCTTGTCCCACATGGAGTTAAT	eno	Enolase		WP_012007014.:	208199..209491	Glycolysis/Gluconeogenesis	COG0148
WP_012007028.1_237	CUST_1304_PI442190926	MIT9215	HLII	WP_012007028.1_237	AACTTTGAAAGTATTGATTTCATTGCTGCTGGAGGCCTATCAATCAATGATGTAAAAACT	eda	2-dehydro-3-deoxyphosphogluconate aldolase		WP_012007028.:	226078..226707	Entner-Dudoroff	COG0800
WP_012007028.1_237	CUST_1305_PI442190926	MIT9215	HLII	WP_012007028.1_237	TGGATTTTGTATACGAAATCAAACTCAAATTTCCAAGAATTAATTTAGGCTCTGCCTCCA	eda	2-dehydro-3-deoxyphosphogluconate aldolase		WP_012007028.:	226078..226707	Entner-Dudoroff	COG0800
WP_012007028.1_237	CUST_1306_PI442190926	MIT9215	HLII	WP_012007028.1_237	AGGTAGCTTAGTAAAATTAGGCTTAAAGAATATTGAAATAAGTTGGTCAAACAACGAAAA	eda	2-dehydro-3-deoxyphosphogluconate aldolase		WP_012007028.:	226078..226707	Entner-Dudoroff	COG0800
WP_012007028.1_237	CUST_1307_PI442190926	MIT9215	HLII	WP_012007028.1_237	AGGATATAAAGCAATCGTGATTGGAGATAAAGGTATTATTAAAAAACAATTTGATCCCAA	eda	2-dehydro-3-deoxyphosphogluconate aldolase		WP_012007028.:	226078..226707	Entner-Dudoroff	COG0800
WP_012007028.1_237	CUST_1308_PI442190926	MIT9215	HLII	WP_012007028.1_237	TCTGGGATAAAGATCTTTTCAATTATTCGAAGTCAAAAAACTATTTATTAATTCCTGGAA	eda	2-dehydro-3-deoxyphosphogluconate aldolase		WP_012007028.:	226078..226707	Entner-Dudoroff	COG0800
WP_012007028.1_237	CUST_1309_PI442190926	MIT9215	HLII	WP_012007028.1_237	AGAAGATTCTTTTTCTGATTTGTTACTAAAAGAATCTTTTTTTTTACTCATAAAACCGGA	eda	2-dehydro-3-deoxyphosphogluconate aldolase		WP_012007028.:	226078..226707	Entner-Dudoroff	COG0800
WP_002806062.1_334	CUST_1818_PI442190926	MIT9215	HLII	WP_002806062.1_334	AGAACTATTTCAGGAGAAATAAAATTCAAAAAAACTGATAAAGCTAATAAAGATATCAAA	minE	septum site-determining protein MinE		WP_002806062.:	312096..312422	Cell Division	COG0851
WP_002806062.1_334	CUST_1819_PI442190926	MIT9215	HLII	WP_002806062.1_334	GTGGCAGTAAGTTTAGAGACTGAGGATAGAATGACTGCATTAGTTGCCAATTTACCAATA	minE	septum site-determining protein MinE		WP_002806062.:	312096..312422	Cell Division	COG0851
WP_002806062.1_334	CUST_1820_PI442190926	MIT9215	HLII	WP_002806062.1_334	AGTTCCTTAACAACTGATCTTTTAGATAAAATGAGGAAAGAGATTCTTGATGTCGTTGCT	minE	septum site-determining protein MinE		WP_002806062.:	312096..312422	Cell Division	COG0851
WP_002806062.1_334	CUST_1821_PI442190926	MIT9215	HLII	WP_002806062.1_334	AGGCAGAGAAACATCTAGTGCAAATACAGCTAGAGAAAGATTGCAGCTTGTACTTGCTCA	minE	septum site-determining protein MinE		WP_002806062.:	312096..312422	Cell Division	COG0851
WP_012007114.1_335	CUST_1822_PI442190926	MIT9215	HLII	WP_012007114.1_335	AATAGAGGCGAGCCACTGACACTTTCAGATGGCAGATCTCCTGCAAAAAAATGTTATTTG	minD	septum site-determining protein MinD		WP_012007114.:	312424..313239	Cell Division	COG2894
WP_012007114.1_335	CUST_1823_PI442190926	MIT9215	HLII	WP_012007114.1_335	CATAGCACTTGCTAATAGTGGAGCAACAACTGCCGTATTAGATGCTGATTTTGGCTTGAG	minD	septum site-determining protein MinD		WP_012007114.:	312424..313239	Cell Division	COG2894
WP_012007114.1_335	CUST_1824_PI442190926	MIT9215	HLII	WP_012007114.1_335	GATTTTGTCTTAGTAGATTGCCCTGCTGGTGTAGAAGATGGCTTTAAAAATGCCCTTGCA	minD	septum site-determining protein MinD		WP_012007114.:	312424..313239	Cell Division	COG2894
WP_012007114.1_335	CUST_1825_PI442190926	MIT9215	HLII	WP_012007114.1_335	AATCTTGCTCTTTTACCAGCTGGAGATCCAAGAATGTTAGATTGGATGAAGCCCGAAGAT	minD	septum site-determining protein MinD		WP_012007114.:	312424..313239	Cell Division	COG2894
WP_012007114.1_335	CUST_1826_PI442190926	MIT9215	HLII	WP_012007114.1_335	ATCGCATCATTTACACGGCTCAAGATGTTCTAGACAGTAATTGTCGTCTTGACCAAGCAT	minD	septum site-determining protein MinD		WP_012007114.:	312424..313239	Cell Division	COG2894
WP_012007114.1_335	CUST_1827_PI442190926	MIT9215	HLII	WP_012007114.1_335	AGAGTTCGCCCTAACATGATGGCTAGTCAAGAAATGTTATCTATCGAAGATGTGCAAGGG	minD	septum site-determining protein MinD		WP_012007114.:	312424..313239	Cell Division	COG2894
WP_012007115.1_336	CUST_1828_PI442190926	MIT9215	HLII	WP_012007115.1_336	AGGAATCGCATTCGCGGGGAAAAGTGGAAATAAAAATGCCTCTATTGCATCACTTTATCT	minC	septum site-determining protein MinC		WP_012007115.:	313351..314013	Cell Division	COG0850
WP_012007115.1_336	CUST_1829_PI442190926	MIT9215	HLII	WP_012007115.1_336	AGCTTTTCTTCCATCGAGGGGCGTTTAGAAGCAAAAATTTTCGCTGTCAATGAGTCAATA	minC	septum site-determining protein MinC		WP_012007115.:	313351..314013	Cell Division	COG0850
WP_012007115.1_336	CUST_1830_PI442190926	MIT9215	HLII	WP_012007115.1_336	TCTTCAAATGGAAACCTATGCATTATTGGAGACGTTAATCCAGGAGCAATAGTTTCCGCT	minC	septum site-determining protein MinC		WP_012007115.:	313351..314013	Cell Division	COG0850
WP_012007115.1_336	CUST_1831_PI442190926	MIT9215	HLII	WP_012007115.1_336	ACCCTTTGCAGTTAAGAATTGCAGATGTAATTGCTATTGGACCAAAGGATAAGCCTAAGA	minC	septum site-determining protein MinC		WP_012007115.:	313351..314013	Cell Division	COG0850
WP_012007115.1_336	CUST_1832_PI442190926	MIT9215	HLII	WP_012007115.1_336	CCAGAAATTGCGATAATAGATAAACAAACGATAATCATAAAACCGCACATAATCGAGAGT	minC	septum site-determining protein MinC		WP_012007115.:	313351..314013	Cell Division	COG0850
WP_012007115.1_336	CUST_1833_PI442190926	MIT9215	HLII	WP_012007115.1_336	TGTTCAATTCAAAAAAGAAAGAAGATATTCTTCACAAAGGAACAATACGATCAGGTGAAA	minC	septum site-determining protein MinC		WP_012007115.:	313351..314013	Cell Division	COG0850
WP_012007271.1_494	CUST_2636_PI442190926	MIT9215	HLII	WP_012007271.1_494	TTGGACTTATAACTTTCCTAATTGCTGACGGAATGACTTTTGCTGGATTCTTTGCTGCTT	cyoC	heme-copper oxidase subunit III		WP_012007271.:	460180..460782	Respiration	COG1845
WP_012007271.1_494	CUST_2637_PI442190926	MIT9215	HLII	WP_012007271.1_494	TGTCACTTTAGGCACTTTAATGATATTAATTATTGCTTGGCAATCGAGAATCAATGGCGG	cyoC	heme-copper oxidase subunit III		WP_012007271.:	460180..460782	Respiration	COG1845
WP_012007271.1_494	CUST_2638_PI442190926	MIT9215	HLII	WP_012007271.1_494	AGCAGTAAATCCATTACCCGATGGTGCTATTTATGAATTAGAACTACCAATACCTACACT	cyoC	heme-copper oxidase subunit III		WP_012007271.:	460180..460782	Respiration	COG1845
WP_012007271.1_494	CUST_2639_PI442190926	MIT9215	HLII	WP_012007271.1_494	ATTTCCATTAGAAGCTGTTGAGTTGTACTGGCATTTTGTAGATGGAATATGGGTTATTTT	cyoC	heme-copper oxidase subunit III		WP_012007271.:	460180..460782	Respiration	COG1845
WP_012007271.1_494	CUST_2640_PI442190926	MIT9215	HLII	WP_012007271.1_494	AGTGCAACTTTCCATAAAGCAGGTAAAGCACTTTTAAAAGATAAAAACTCTGATTCACAA	cyoC	heme-copper oxidase subunit III		WP_012007271.:	460180..460782	Respiration	COG1845
WP_012007271.1_494	CUST_2641_PI442190926	MIT9215	HLII	WP_012007271.1_494	TTTGGATTAACCGATAATTTATTTGCAAGTACTTTTTATGCTCTTACTGGTTTTCATGGA	cyoC	heme-copper oxidase subunit III		WP_012007271.:	460180..460782	Respiration	COG1845
WP_012007272.1_495	CUST_2642_PI442190926	MIT9215	HLII	WP_012007272.1_495	GCCACCGGTTGAAAATTGGGAAGAAGAAGCTCCATTAGTTGAAGAACCTTATGGTTATGG	cyoB/coxA	Cytochrome c oxidase subunit I		WP_012007272.:	460789..462414	Respiration	COG0843
WP_012007272.1_495	CUST_2643_PI442190926	MIT9215	HLII	WP_012007272.1_495	TGGAGGTATTACAGGAGTTGCTTTGGCACAGGTCCCTTTTGATATTCACGTACATGATAC	cyoB/coxA	Cytochrome c oxidase subunit I		WP_012007272.:	460789..462414	Respiration	COG0843
WP_012007272.1_495	CUST_2644_PI442190926	MIT9215	HLII	WP_012007272.1_495	CCTTAGTGTGAGAAATGGAAAAGATGCTGGAGACAATCCTTGGAATGCTCTTACACCTGA	cyoB/coxA	Cytochrome c oxidase subunit I		WP_012007272.:	460789..462414	Respiration	COG0843
WP_012007272.1_495	CUST_2645_PI442190926	MIT9215	HLII	WP_012007272.1_495	TGGATGCGATTGTTCTTTACTATCGCAACAGCATTTATTGCTGTTCCAACGGGGATAAAA	cyoB/coxA	Cytochrome c oxidase subunit I		WP_012007272.:	460789..462414	Respiration	COG0843
WP_012007272.1_495	CUST_2646_PI442190926	MIT9215	HLII	WP_012007272.1_495	CTTTTATTGGATTTAACTTGTGCTTCGCTCCTCAACATTGGCTCGGTTTAAACGGAATGC	cyoB/coxA	Cytochrome c oxidase subunit I		WP_012007272.:	460789..462414	Respiration	COG0843
WP_012007272.1_495	CUST_2647_PI442190926	MIT9215	HLII	WP_012007272.1_495	ATGGGGATAGTTGTTTTAGGTTTAGTTGTTTGGGCGCATCACATGTTTACTAGTGGAACG	cyoB/coxA	Cytochrome c oxidase subunit I		WP_012007272.:	460789..462414	Respiration	COG0843
WP_012007273.1_496	CUST_2648_PI442190926	MIT9215	HLII	WP_012007273.1_496	AAAATATCCAATTATTTGCGCAGAATTATGTGGCCCATATCATGGTGGGATGAGAGCCTC	ctaC/coxB	Cytochrome c oxidase subunit II		WP_012007273.:	462411..463214	Respiration	COG1622
WP_012007273.1_496	CUST_2649_PI442190926	MIT9215	HLII	WP_012007273.1_496	TCTTTTTGGATTGGAACCAATGTAAATTTGCTCCCAGCTGAAGCAAGTATTAATGCACCA	ctaC/coxB	Cytochrome c oxidase subunit II		WP_012007273.:	462411..463214	Respiration	COG1622
WP_012007273.1_496	CUST_2650_PI442190926	MIT9215	HLII	WP_012007273.1_496	TGCTTTTTGGGTACCTGAGTTCAGAATTAAACAGGATATTATTCCTGGTCAACCAACTGT	ctaC/coxB	Cytochrome c oxidase subunit II		WP_012007273.:	462411..463214	Respiration	COG1622
WP_012007273.1_496	CUST_2651_PI442190926	MIT9215	HLII	WP_012007273.1_496	AGTTACAACATCTACGACAGAATGGAGGTATGAAAGAGCTAAATCATAACCACGAAATG	ctaC/coxB	Cytochrome c oxidase subunit II		WP_012007273.:	462411..463214	Respiration	COG1622
WP_012007273.1_496	CUST_2652_PI442190926	MIT9215	HLII	WP_012007273.1_496	TGCATTTCTATTCAATTATCCCAAAGGCAAATTTATATCAGGAGAACTACACGTTCCAGT	ctaC/coxB	Cytochrome c oxidase subunit II		WP_012007273.:	462411..463214	Respiration	COG1622
WP_012007273.1_496	CUST_2653_PI442190926	MIT9215	HLII	WP_012007273.1_496	ACTTTTCAAAATTCTTTTCATCATAGGGTTAAATTATTTTTATAGGAATGACAATGGCGGT	ctaC/coxB	Cytochrome c oxidase subunit II		WP_012007273.:	462411..463214	Respiration	COG1622
WP_012007280.1_504	CUST_2691_PI442190926	MIT9215	HLII	WP_012007280.1_504	TTGTTGGATTAATTGGTAATCCCGGTCAAGCAAATTATTCTGCAGCCAAAGCTGGAGTTA	gdh	3-oxoacyl-acyl-carrier-protein reductase		WP_012007280.:	469792..470541	Entner-Dudoroff	COG1028
WP_012007280.1_504	CUST_2692_PI442190926	MIT9215	HLII	WP_012007280.1_504	GCTAGCAGAGGAATTGGTAAAGAGATTGCTTTAGAACTTAGCCGCTTAGGAGCAGAAGTT	gdh	3-oxoacyl-acyl-carrier-protein reductase		WP_012007280.:	469792..470541	Entner-Dudoroff	COG1028
WP_012007280.1_504	CUST_2693_PI442190926	MIT9215	HLII	WP_012007280.1_504	TGCTGGCATTACTAGAGATGGACTATTGATGAGAATGAAATCGGAACAATGGGATGACGT	gdh	3-oxoacyl-acyl-carrier-protein reductase		WP_012007280.:	469792..470541	Entner-Dudoroff	COG1028
WP_012007280.1_504	CUST_2694_PI442190926	MIT9215	HLII	WP_012007280.1_504	ACTTGCGCTAAAGAATTTGCTTCAAGGAGTATAAACGTAAATGCAATAGCTCCTGGTTTC	gdh	3-oxoacyl-acyl-carrier-protein reductase		WP_012007280.:	469792..470541	Entner-Dudoroff	COG1028
WP_012007280.1_504	CUST_2695_PI442190926	MIT9215	HLII	WP_012007280.1_504	TCAAGTAATGCTGGAAGTTATATCACGGGACAAACAATTAGTATTGACGGAGGTATGAGT	gdh	3-oxoacyl-acyl-carrier-protein reductase		WP_012007280.:	469792..470541	Entner-Dudoroff	COG1028
WP_012007280.1_504	CUST_2696_PI442190926	MIT9215	HLII	WP_012007280.1_504	ACCGATGAGATTCTTAAAGCTATTCCTTTAGGGAAATTAGGAAGTTGCACTCAAATTGCA	gdh	3-oxoacyl-acyl-carrier-protein reductase		WP_012007280.:	469792..470541	Entner-Dudoroff	COG1028
WP_041484446.1_570	CUST_3053_PI442190926	MIT9215	HLII	WP_041484446.1_570	CTACAGAAGTTGATGCAAGACTAAGCTTTGACACCGAAGCTACGGTAGAAAAAGCGAGAA	tal	Transaldolase		WP_041484446.:	526690..527691	Pentose Phosphate	COG0176
WP_041484446.1_570	CUST_3054_PI442190926	MIT9215	HLII	WP_041484446.1_570	CGGCAACTTGGGAGGGAATTAAGGCAGCTGAAATTTTAGAAAAAGAGGGTATAAAGTGCA	tal	Transaldolase		WP_041484446.:	526690..527691	Pentose Phosphate	COG0176
WP_041484446.1_570	CUST_3055_PI442190926	MIT9215	HLII	WP_041484446.1_570	TAACTTTTGCCAAGCGGTAACTTGTGCCAATGCAAAGATAACTCTAATTTCTCCGTTCGT	tal	Transaldolase		WP_041484446.:	526690..527691	Pentose Phosphate	COG0176
WP_041484446.1_570	CUST_3056_PI442190926	MIT9215	HLII	WP_041484446.1_570	AAAGCTTAGTGAAGGTATCACTGGATTCAGTAAGGCTATAGAAGAATTGGAGGAGCTGCT	tal	Transaldolase		WP_041484446.:	526690..527691	Pentose Phosphate	COG0176
WP_041484446.1_570	CUST_3057_PI442190926	MIT9215	HLII	WP_041484446.1_570	GGATTGGTATAAGGCAAAAACTGGTAAAAATAGTTTTGTTGGTGCTGAAGACCCTGGTGT	tal	Transaldolase		WP_041484446.:	526690..527691	Pentose Phosphate	COG0176
WP_041484446.1_570	CUST_3058_PI442190926	MIT9215	HLII	WP_041484446.1_570	AGCAGGTTGCGATCTTTTAACAATCGCACCAAAATTTCTTGAGGAACTGAAAAAAGAAAA	tal	Transaldolase		WP_041484446.:	526690..527691	Pentose Phosphate	COG0176
WP_002805854.1_601	CUST_3229_PI442190926	MIT9215	HLII	WP_002805854.1_601	GGTATTCACGTATGGCATATGCCTGCACTACTAGCGATTTTTGGAGATGACTCTTGTCTT	rbcL	Ribulose 1,5-bisphosphate carboxylase large subunit		WP_002805854.:	555111..556526	Carbon fixation	COG1850
WP_011376108.1_602	CUST_3233_PI442190926	MIT9215	HLII	WP_011376108.1_602	ACCCTGATCATCATGTAAGAATCATCGGATACGATGCTTACACTCAAAGCCAAGGAACAG	rbcS	Ribulose bisphosphate carboxylase small subunit		WP_011376108.:	556620..556961	Carbon fixation	COG4451
WP_011376108.1_602	CUST_3234_PI442190926	MIT9215	HLII	WP_011376108.1_602	TGGAAGCTCCCATTCTTTGGGGAAAAAGATCTTAACTTGGTTGTAAGTGAATTAGAGGCA	rbcS	Ribulose bisphosphate carboxylase small subunit		WP_011376108.:	556620..556961	Carbon fixation	COG4451
WP_011376108.1_602	CUST_3235_PI442190926	MIT9215	HLII	WP_011376108.1_602	CCAAATTGCATACATAATTGCTCAAGGCTGGAGTCCTGTTATTGAGCATGTTCACCCTAG	rbcS	Ribulose bisphosphate carboxylase small subunit		WP_011376108.:	556620..556961	Carbon fixation	COG4451
WP_011376108.1_602	CUST_3236_PI442190926	MIT9215	HLII	WP_011376108.1_602	CAGAGCACAGTAGGCGACTATCAAACAGTTGCAACCCTGGAAACATTCGGTTTCTTAC	rbcS	Ribulose bisphosphate carboxylase small subunit		WP_011376108.:	556620..556961	Carbon fixation	COG4451
WP_012007413.1_649	CUST_3483_PI442190926	MIT9215	HLII	WP_012007413.1_649	GTGCTTTGGCGGGTTATGGATTTCTGGAGGAACCGCATCAAAACATTTCAAAAACTTTAA	glk	Glucokinase		WP_012007413.:	602516..603550	Glycolysis	COG0837
WP_012007413.1_649	CUST_3484_PI442190926	MIT9215	HLII	WP_012007413.1_649	GAGCGGGTACTGGTTTGGGCATTGCAAGAGGAATAATATCAGGGAAAAAGGTAAAAGTTT	glk	Glucokinase		WP_012007413.:	602516..603550	Glycolysis	COG0837
WP_012007413.1_649	CUST_3485_PI442190926	MIT9215	HLII	WP_012007413.1_649	AGTTGAGAGGATTTGGCTAGGTGCTTATGCATCTTTATTGGGAGATGTTGCTCTTCAAGA	glk	Glucokinase		WP_012007413.:	602516..603550	Glycolysis	COG0837
WP_012007413.1_649	CUST_3486_PI442190926	MIT9215	HLII	WP_012007413.1_649	AATTATTAGCGGCACTGGTTTATCAAGAATTGCCGAATGGAGACTAAGCAAACCTGATGC	glk	Glucokinase		WP_012007413.:	602516..603550	Glycolysis	COG0837
WP_012007413.1_649	CUST_3487_PI442190926	MIT9215	HLII	WP_012007413.1_649	AGATGAAGAGTTTGGACTTTTTAGTGCAGCCTGCAGAGCAAAAATGCTTTTAAAAACCTA	glk	Glucokinase		WP_012007413.:	602516..603550	Glycolysis	COG0837
WP_012007413.1_649	CUST_3488_PI442190926	MIT9215	HLII	WP_012007413.1_649	TCTACTATCCAAAATGGATCCCATTCTGAAGGTGCTAATAATGATCTGCATGCCATTGTT	glk	Glucokinase		WP_012007413.:	602516..603550	Glycolysis	COG0837
WP_012007577.1_820	CUST_4352_PI442190926	MIT9215	HLII	WP_012007577.1_820	TTATGGGAGCTGATTTCTTTGAATCGCCTGAAGAGAGGATTGAATTAAGAAGAGGAGGCG	glgC	Glucose-1-phosphate adenylyltransferase		WP_012007577.:	753349..754644	Glycogen Metabolism	COG0448
WP_012007577.1_820	CUST_4353_PI442190926	MIT9215	HLII	WP_012007577.1_820	GATGATTATTGGGAAGATATCGGAACGATTGGGGCATTCTTTGAGTCAAATCTCGCGTTG	glgC	Glucose-1-phosphate adenylyltransferase		WP_012007577.:	753349..754644	Glycogen Metabolism	COG0448
WP_012007577.1_820	CUST_4354_PI442190926	MIT9215	HLII	WP_012007577.1_820	AGTGAAAAGCCCACTGGTAAGAAGTTGAAAGCAATGGCAGTAGATACTTCAAAATTTGGA	glgC	Glucose-1-phosphate adenylyltransferase		WP_012007577.:	753349..754644	Glycogen Metabolism	COG0448
WP_012007577.1_820	CUST_4355_PI442190926	MIT9215	HLII	WP_012007577.1_820	GCGGAACGCCTCTTGGAGTAGGGGAAGGAACTACAGTAAAAAGAGCAATTCTTGATA	glgC	Glucose-1-phosphate adenylyltransferase		WP_012007577.:	753349..754644	Glycogen Metabolism	COG0448
WP_012007577.1_820	CUST_4356_PI442190926	MIT9215	HLII	WP_012007577.1_820	ATTTCCTAGTTATACAGACTTTGGTAAGGACATAATTCCTGAAGCCCTAAATAGGGGAGA	glgC	Glucose-1-phosphate adenylyltransferase		WP_012007577.:	753349..754644	Glycogen Metabolism	COG0448
WP_012007577.1_820	CUST_4357_PI442190926	MIT9215	HLII	WP_012007577.1_820	ATTGTGTTTTAGGTGTAAGAACTAGGATTGAAAGTGATTCGGTTCTTGAGGATACTCTCG	glgC	Glucose-1-phosphate adenylyltransferase		WP_012007577.:	753349..754644	Glycogen Metabolism	COG0448
WP_012007578.1_821	CUST_4358_PI442190926	MIT9215	HLII	WP_012007578.1_821	AGAACCAATAGACTTCCTCAGAATCTCGTTCAGGCTATGAGAGATTGTTTTGGCTCTCAT	edd	phosphogluconate dehydrogenase (NADP(+)-dependent, decarboxyla		WP_012007578.:	754740..756158	Pentose Phosphate/Entner-Dudorof	COG2084
WP_012007578.1_821	CUST_4359_PI442190926	MIT9215	HLII	WP_012007578.1_821	GCTAATGTAGTTTCATTGTCAACAAAAGCTGGTATACCTGTGCCATGTCTATCCAGTACT	edd	phosphogluconate dehydrogenase (NADP(+)-dependent, decarboxyla		WP_012007578.:	754740..756158	Pentose Phosphate/Entner-Dudorof	COG2084
WP_012007578.1_821	CUST_4360_PI442190926	MIT9215	HLII	WP_012007578.1_821	GCAGTATATAACTATGGTTTGAATATGCCCTCAATTGCTCAGATATGGAAAGGTGGTTGC	edd	phosphogluconate dehydrogenase (NADP(+)-dependent, decarboxyla		WP_012007578.:	754740..756158	Pentose Phosphate/Entner-Dudorof	COG2084
WP_012007578.1_821	CUST_4361_PI442190926	MIT9215	HLII	WP_012007578.1_821	GATGGGATGAAACCTTTATTTGATGCTGTAGTCCTTGCCACAATTGCTAGCTATGCACAA	edd	phosphogluconate dehydrogenase (NADP(+)-dependent, decarboxyla		WP_012007578.:	754740..756158	Pentose Phosphate/Entner-Dudorof	COG2084
WP_012007578.1_821	CUST_4362_PI442190926	MIT9215	HLII	WP_012007578.1_821	GGGTATGGACTGTTGTAAGTGCTTTAGAACTGGGGGTATCAGTCCCAACTATTTATGCA	edd	phosphogluconate dehydrogenase (NADP(+)-dependent, decarboxyla		WP_012007578.:	754740..756158	Pentose Phosphate/Entner-Dudorof	COG2084
WP_012007578.1_821	CUST_4363_PI442190926	MIT9215	HLII	WP_012007578.1_821	TGCAGAAGCTTATGACCTTATGAAAAGAGTAAAAGGTATGAATGGGAAGCAGATGTCAGA	edd	phosphogluconate dehydrogenase (NADP(+)-dependent, decarboxyla		WP_012007578.:	754740..756158	Pentose Phosphate/Entner-Dudorof	COG2084
WP_012007589.1_832	CUST_4419_PI442190926	MIT9215	HLII	WP_012007589.1_832	GCTGATCCTATGAAAGTGGCTGAGATGACTATAAGAACTATGGAAAGATGCGTTCCTGCG	fda	Fructose-1,6-bisphosphate aldolase class I		WP_012007589.:	763429..764496	Glycolysis/Gluconeogenesis	COG0191
WP_012007589.1_832	CUST_4420_PI442190926	MIT9215	HLII	WP_012007589.1_832	GCTGAAGTACAAGAAGAGGTGATTAAGCAGGTCTACATTGCTTGTCAGGCAAATGGAGTA	fda	Fructose-1,6-bisphosphate aldolase class I		WP_012007589.:	763429..764496	Glycolysis/Gluconeogenesis	COG0191
WP_012007589.1_832	CUST_4421_PI442190926	MIT9215	HLII	WP_012007589.1_832	ATAGGAAAGCATTATGGAATGTTTCATTCTCCTATGGAAGAGCGCTACAGCACTCATGCT	fda	Fructose-1,6-bisphosphate aldolase class I		WP_012007589.:	763429..764496	Glycolysis/Gluconeogenesis	COG0191
WP_012007589.1_832	CUST_4422_PI442190926	MIT9215	HLII	WP_012007589.1_832	TTGTCCTTCAAAATTATCCATACAGGAAAATGCATGGGGATTGGCAAGGTATGCGAGATC	fda	Fructose-1,6-bisphosphate aldolase class I		WP_012007589.:	763429..764496	Glycolysis/Gluconeogenesis	COG0191
WP_012007589.1_832	CUST_4423_PI442190926	MIT9215	HLII	WP_012007589.1_832	GTCGCAGGATCTCAGCCTTCATCTGATGAGCAGCTATTTGTTGCTGGATACACTTATTAA	fda	Fructose-1,6-bisphosphate aldolase class I		WP_012007589.:	763429..764496	Glycolysis/Gluconeogenesis	COG0191
WP_012007589.1_832	CUST_4424_PI442190926	MIT9215	HLII	WP_012007589.1_832	ATAAAGGTTTGAATCCACTTCCTGGAGGAGGAGATGTAGAAACATTTTGCTCTGGTCTGG	fda	Fructose-1,6-bisphosphate aldolase class I		WP_012007589.:	763429..764496	Glycolysis/Gluconeogenesis	COG0191
WP_012007593.1_836	CUST_4442_PI442190926	MIT9215	HLII	WP_012007593.1_836	TTCCAACCCCTGACGAGAGCTTTGTGATCATACATTTCAGAAAAGGAGCAAGAGAGAAAT	prk	Phosphoribulokinase		WP_012007593.:	767116..768015	Carbon Fixation	COG3954
WP_012007593.1_836	CUST_4443_PI442190926	MIT9215	HLII	WP_012007593.1_836	GGCAGAGGCTCTTTCTAAAGGTGAAAATTTTTCTCACTTTGGCCCAGAGGCTAATCTTTT	prk	Phosphoribulokinase		WP_012007593.:	767116..768015	Carbon Fixation	COG3954
WP_012007593.1_836	CUST_4444_PI442190926	MIT9215	HLII	WP_012007593.1_836	GATATTCCTAAGGGAACAGACGTACTTTTTTATGAAGGGTTACATGGCGGTGTGGAAGGT	prk	Phosphoribulokinase		WP_012007593.:	767116..768015	Carbon Fixation	COG3954
WP_012007593.1_836	CUST_4445_PI442190926	MIT9215	HLII	WP_012007593.1_836	TAGGAATGATTCACGATTCATTCATGTCTAGCCCTACCAGCATTGTTGTAAATGGTGGGA	prk	Phosphoribulokinase		WP_012007593.:	767116..768015	Carbon Fixation	COG3954
WP_012007593.1_836	CUST_4446_PI442190926	MIT9215	HLII	WP_012007593.1_836	TGGTGAAACTGGGGGAGGGAAGAAAAGATATTATCTACATAGTCCTGAAGAAGCTGAAGA	prk	Phosphoribulokinase		WP_012007593.:	767116..768015	Carbon Fixation	COG3954
WP_012007593.1_836	CUST_4447_PI442190926	MIT9215	HLII	WP_012007593.1_836	TGTGGCATCTTATGCTGATTTACTTGTTGGCGTTGTTCCGATAACAAATTTAGAGTGGAT	prk	Phosphoribulokinase		WP_012007593.:	767116..768015	Carbon Fixation	COG3954
WP_012007666.1_912	CUST_4847_PI442190926	MIT9215	HLII	WP_012007666.1_912	TGGGAATGCTTAGATATACAAGCGGCGGAAATTATAAGATACCAGCAGTTGTTCGAGGTC	pdhB	Pyruvate dehydrogenase E1 component beta subunit		WP_012007666.:	831329..832312	Glycolysis/Krebs cycle	COG0022
WP_012007666.1_912	CUST_4848_PI442190926	MIT9215	HLII	WP_012007666.1_912	GTGCAGAATTAATTGCCTTGATAACAGAAGAGTGTTTTGACGATCTTGATGCCCGACCAA	pdhB	Pyruvate dehydrogenase E1 component beta subunit		WP_012007666.:	831329..832312	Glycolysis/Krebs cycle	COG0022
WP_012007666.1_912	CUST_4849_PI442190926	MIT9215	HLII	WP_012007666.1_912	GAATTACCTGAGGGGGATTATACATGCGCTTTAGATCAGGCTGACGTTGTAAAAGAAGGT	pdhB	Pyruvate dehydrogenase E1 component beta subunit		WP_012007666.:	831329..832312	Glycolysis/Krebs cycle	COG0022
WP_012007666.1_912	CUST_4850_PI442190926	MIT9215	HLII	WP_012007666.1_912	GTTGGTCGCCAACTAGGTGCTGAACATAGTCAAAGACTTGAAGCATATTTTCATGCAGTT	pdhB	Pyruvate dehydrogenase E1 component beta subunit		WP_012007666.:	831329..832312	Glycolysis/Krebs cycle	COG0022
WP_012007666.1_912	CUST_4851_PI442190926	MIT9215	HLII	WP_012007666.1_912	TGAGACATCACTGCCTTAAAGCGGTTGAAGAATTAGAAAAAAAAGGAATAGATGTTGAGT	pdhB	Pyruvate dehydrogenase E1 component beta subunit		WP_012007666.:	831329..832312	Glycolysis/Krebs cycle	COG0022
WP_012007666.1_912	CUST_4852_PI442190926	MIT9215	HLII	WP_012007666.1_912	TGTTGCATGTAGTACTCCTACAAATGCTAAAGGTTTAATGAAAGCAGCTATAAGAGATGA	pdhB	Pyruvate dehydrogenase E1 component beta subunit		WP_012007666.:	831329..832312	Glycolysis/Krebs cycle	COG0022
WP_012007675.1_923	CUST_4904_PI442190926	MIT9215	HLII	WP_012007675.1_923	CCAACCCAACTACAAATAGTTACAAACGATTGGTTCCAGGATTCGAAGCGCCTGTAAATC	glnA	Glutamine synthetase/ glutamate--ammonialigase		WP_012007675.:	840097..841518	Nitrogen Metabolism	COG0174
WP_012007675.1_923	CUST_4905_PI442190926	MIT9215	HLII	WP_012007675.1_923	ATTCCTTTAACAGGTCCAAGCCCTAAAGCTAAAAGATTAGAATTCCGATCAGGTGACGCA	glnA	Glutamine synthetase/ glutamate--ammonialigase		WP_012007675.:	840097..841518	Nitrogen Metabolism	COG0174
WP_012007675.1_923	CUST_4906_PI442190926	MIT9215	HLII	WP_012007675.1_923	TGATCCTGGTGATGGAGTGGATGTAGATTTATTTGAACTTCCAGCTGATGAACTTGCCAA	glnA	Glutamine synthetase/ glutamate--ammonialigase		WP_012007675.:	840097..841518	Nitrogen Metabolism	COG0174
WP_012007675.1_923	CUST_4907_PI442190926	MIT9215	HLII	WP_012007675.1_923	TTTTTGGTGAAGGAGCATATGCAAATTTATCTCAAACAGCAAGATGGTACATCGGAGGCA	glnA	Glutamine synthetase/ glutamate--ammonialigase		WP_012007675.:	840097..841518	Nitrogen Metabolism	COG0174
WP_012007675.1_923	CUST_4908_PI442190926	MIT9215	HLII	WP_012007675.1_923	TGCCCAAGCCTGTATTTAACGATAATGGAACTGGAATGCATGTTCACCAAAGTTTATGGA	glnA	Glutamine synthetase/ glutamate--ammonialigase		WP_012007675.:	840097..841518	Nitrogen Metabolism	COG0174
WP_012007675.1_923	CUST_4909_PI442190926	MIT9215	HLII	WP_012007675.1_923	CGCCGGCCAACACGAACTTGGAATGAAATTCGATTCGTTAATAAATTCTGCAGATAACGT	glnA	Glutamine synthetase/ glutamate--ammonialigase		WP_012007675.:	840097..841518	Nitrogen Metabolism	COG0174
WP_012007684.1_931	CUST_4949_PI442190926	MIT9215	HLII	WP_012007684.1_931	AGAAAAATTGTATCAAGAGGAATTTCAATAGGGGAAATCGGTGTTACAGGTAAAGCAAGA	pykF	Pyruvate kinase		WP_012007684.:	848739..850532	Glycolysis	COG0469
WP_012007684.1_931	CUST_4950_PI442190926	MIT9215	HLII	WP_012007684.1_931	GCAAATTGCTCAGGAGATGGGAATCCTAAATCAAGGAGATTTAGTAGTTCAAACCGCAGG	pykF	Pyruvate kinase		WP_012007684.:	848739..850532	Glycolysis	COG0469
WP_012007684.1_931	CUST_4951_PI442190926	MIT9215	HLII	WP_012007684.1_931	TTTGGCAACAACTACAGAGAGGAGTGTAGCTAGAAGATTGCAACTTGTTTGGGGAGTTAC	pykF	Pyruvate kinase		WP_012007684.:	848739..850532	Glycolysis	COG0469
WP_012007684.1_931	CUST_4952_PI442190926	MIT9215	HLII	WP_012007684.1_931	AGCTAGACAACTTGATGCAGGAGCTATAATCCCTTTAACAAAATCAGGTTCTACCGCTCG	pykF	Pyruvate kinase		WP_012007684.:	848739..850532	Glycolysis	COG0469
WP_012007684.1_931	CUST_4953_PI442190926	MIT9215	HLII	WP_012007684.1_931	AAGGCTATTGAAAGCCACTTACCCAGTACTATCCCAAATGCTATTAGTGCAGCAGTAAGT	pykF	Pyruvate kinase		WP_012007684.:	848739..850532	Glycolysis	COG0469
WP_012007684.1_931	CUST_4954_PI442190926	MIT9215	HLII	WP_012007684.1_931	TGTAGAGGCAGTTGAAACGATGGCAACTATAGCAAGAAGAATTGAAAGGGATTACCCACT	pykF	Pyruvate kinase		WP_012007684.:	848739..850532	Glycolysis	COG0469
WP_012007704.1_953	CUST_5067_PI442190926	MIT9215	HLII	WP_012007704.1_953	GCATATGATCAACCTGGTGTTGAAGCTGGGAAGAAAGCAGCCGCAAATATTATTGAGTAT	pgi	Glucose-6-phosphate isomerase		WP_012007704.:	869426..871009	Glycolysis/Gluconeogenesis	COG0166
WP_012007704.1_953	CUST_5068_PI442190926	MIT9215	HLII	WP_012007704.1_953	TGTTTTTCACTAGGTGCCTTAATCGCTTTATTTGAGAGGGCTGTATCCTTCTACGCTGAA	pgi	Glucose-6-phosphate isomerase		WP_012007704.:	869426..871009	Glycolysis/Gluconeogenesis	COG0166
WP_012007704.1_953	CUST_5069_PI442190926	MIT9215	HLII	WP_012007704.1_953	GAGGTGCATCATTAATGGACGAGGCCACACGTACAGTTGATTTTAAAAATAATCCCGCAG	pgi	Glucose-6-phosphate isomerase		WP_012007704.:	869426..871009	Glycolysis/Gluconeogenesis	COG0166
WP_012007704.1_953	CUST_5070_PI442190926	MIT9215	HLII	WP_012007704.1_953	TCCGTTTTTGGCAATAAAGGATCTACAGATCAACATGCTTATGTTCAGCAACTGAGAGAT	pgi	Glucose-6-phosphate isomerase		WP_012007704.:	869426..871009	Glycolysis/Gluconeogenesis	COG0166
WP_012007704.1_953	CUST_5071_PI442190926	MIT9215	HLII	WP_012007704.1_953	TCCGTGAAATGTGTTTCGGTAATGATAATTATTTAGTTAAGGGCGATTGGTCAAATCCGA	pgi	Glucose-6-phosphate isomerase		WP_012007704.:	869426..871009	Glycolysis/Gluconeogenesis	COG0166
WP_012007704.1_953	CUST_5072_PI442190926	MIT9215	HLII	WP_012007704.1_953	TGGAGATGGTATTGGAAAGAGAGATATGGTCGTATTACCTTATAGAGATAGGTTGCAGGT	pgi	Glucose-6-phosphate isomerase		WP_012007704.:	869426..871009	Glycolysis/Gluconeogenesis	COG0166
WP_012007871.1_1110	CUST_5822_PI442190926	MIT9215	HLII	WP_012007871.1_1110	GGCCATGTTGAGTGGATTAATTTCTTAAGAGTCGATAAATTGCCGAAGCCAGGAACCATC	rbsK	Sugar kinase, ribokinase family		WP_012007871.:	996259..997125	Pentose Phosphate	COG0524
WP_012007871.1_1110	CUST_5823_PI442190926	MIT9215	HLII	WP_012007871.1_1110	TTCAAGATTCTCTCGAATATGGGAATTAAGTTGCATGTGGCTTGGCGTGATAAACCAACG	rbsK	Sugar kinase, ribokinase family		WP_012007871.:	996259..997125	Pentose Phosphate	COG0524
WP_012007871.1_1110	CUST_5824_PI442190926	MIT9215	HLII	WP_012007871.1_1110	TCTTATGGATGTGGCGATTCTTTTGCTGCTGGGATTCTTTATGGAATGGCATCTAAATGG	rbsK	Sugar kinase, ribokinase family		WP_012007871.:	996259..997125	Pentose Phosphate	COG0524
WP_012007871.1_1110	CUST_5825_PI442190926	MIT9215	HLII	WP_012007871.1_1110	AAAGTCCATTGAATATCCAGCTGGCGGTGGATCTATTATCGCGAAAATACTTTCGGATTT	rbsK	Sugar kinase, ribokinase family		WP_012007871.:	996259..997125	Pentose Phosphate	COG0524

WP_012007871.1_1110	CUST_5826_PI442190926	MIT9215	HLII	WP_012007871.1_1110	AGTTTAAATCTTGCTAAAGTAATGGGAAGAGATGCCAGTGAATTTTTTGGCCCATATGCA	rbsK	Sugar kinase, ribokinase family	WP_012007871.:	996259..997125	Pentose Phosphate	COG0524
WP_012007871.1_1110	CUST_5827_PI442190926	MIT9215	HLII	WP_012007871.1_1110	AACCGAGGGAGAGAAGGGAGGAATAATATTTCCTGGTGGAAGATATAAGGCTCTAAAAAA	rbsK	Sugar kinase, ribokinase family	WP_012007871.:	996259..997125	Pentose Phosphate	COG0524
WP_012007898.1_1139	CUST_5986_PI442190926	MIT9215	HLII	WP_012007898.1_1139	TAATACTTGGTTGTGAATCAGTGGAATGTATGAGACTTGAAGCAGGGGGAATGGCCAATA	opcA	Glucose-6-P dehydrogenase subunit opcA	WP_012007898.:	1024757..1026058	Pentose Phosphate	COG3429
WP_012007898.1_1139	CUST_5987_PI442190926	MIT9215	HLII	WP_012007898.1_1139	ATGAATTACCTTCTTGGTTGTGGTGGAATGGTAGCTTGGATGAATCCCCTGAAATTTTCG	opcA	Glucose-6-P dehydrogenase subunit opcA	WP_012007898.:	1024757..1026058	Pentose Phosphate	COG3429
WP_012007898.1_1139	CUST_5988_PI442190926	MIT9215	HLII	WP_012007898.1_1139	TAGGTTGAAAAATTGGAGGGAATCATTGGCAATGATTTTTGATCCACCTTCGAGGAGACC	opcA	Glucose-6-P dehydrogenase subunit opcA	WP_012007898.:	1024757..1026058	Pentose Phosphate	COG3429
WP_012007898.1_1139	CUST_5989_PI442190926	MIT9215	HLII	WP_012007898.1_1139	TCCCTTATCTTTGGGAAATCCCAGTATTCATTTAGGACAAGTTATTGGATTGAGGTTGAT	opcA	Glucose-6-P dehydrogenase subunit opcA	WP_012007898.:	1024757..1026058	Pentose Phosphate	COG3429
WP_012007898.1_1139	CUST_5990_PI442190926	MIT9215	HLII	WP_012007898.1_1139	AGCATTGTTTTTGATCTCATGGATTAGCGATAAACTTGGTTGGTCCTTTATAAGTGTTGA	opcA	Glucose-6-P dehydrogenase subunit opcA	WP_012007898.:	1024757..1026058	Pentose Phosphate	COG3429
WP_012007898.1_1139	CUST_5991_PI442190926	MIT9215	HLII	WP_012007898.1_1139	TTCACGAAATATGGTTTAAGGTTAATAATTGATACTGCTCTTGGATCTCCTAACAGATGT	opcA	Glucose-6-P dehydrogenase subunit opcA	WP_012007898.:	1024757..1026058	Pentose Phosphate	COG3429
WP_012007899.1_1140	CUST_5992_PI442190926	MIT9215	HLII	WP_012007899.1_1140	AATATGGAGAGGGAGGAAATATCTCAAATCGACTCAAAGGGTACAGACAGGAAGAGGGTG	zwf	glucose-6-phosphate dehydrogenase zwf	WP_012007899.:	1026061..1027584	Pentose Phosphate	COG0364
WP_012007899.1_1140	CUST_5993_PI442190926	MIT9215	HLII	WP_012007899.1_1140	CTGACCCAACCTTATTTACTCGAAGTGATGAAGTAGAGGCAGCCTGGAAACTTTATACGC	zwf	glucose-6-phosphate dehydrogenase zwf	WP_012007899.:	1026061..1027584	Pentose Phosphate	COG0364
WP_012007899.1_1140	CUST_5994_PI442190926	MIT9215	HLII	WP_012007899.1_1140	CAACAGAAACTTATATTGCGACAAAGTTTTTGTGGATAACTGGCGTTGGCAAGGGGTTC	zwf	glucose-6-phosphate dehydrogenase zwf	WP_012007899.:	1026061..1027584	Pentose Phosphate	COG0364
WP_012007899.1_1140	CUST_5995_PI442190926	MIT9215	HLII	WP_012007899.1_1140	GACTCAAATGCTTGCTGTTACTGCTATGGAACCTCCTGGAAAATTTGAACCAGAAGCAAT	zwf	glucose-6-phosphate dehydrogenase zwf	WP_012007899.:	1026061..1027584	Pentose Phosphate	COG0364
WP_012007899.1_1140	CUST_5996_PI442190926	MIT9215	HLII	WP_012007899.1_1140	TCCCCTTGGAAGTTACCTATTTATAACTATGAATCTATGACGTGGGGACCTCCTGAGTCT	zwf	glucose-6-phosphate dehydrogenase zwf	WP_012007899.:	1026061..1027584	Pentose Phosphate	COG0364
WP_012007899.1_1140	CUST_5997_PI442190926	MIT9215	HLII	WP_012007899.1_1140	AGGTGCTACTTTCAAATTTGAGGTAAAATCTCCTGGTTCTGGAATGAAATCCAGACCTGT	zwf	glucose-6-phosphate dehydrogenase zwf	WP_012007899.:	1026061..1027584	Pentose Phosphate	COG0364
WP_041484402.1_1330	CUST_6932_PI442190926	MIT9215	HLII	WP_041484402.1_1330	TCACTAATTTGTGGAGCAGCGAACTTTCTAAATTAACAGCAAATGCATTTTTGGCGCAAA	ugd	UDP-glucose 6-dehydrogenase/nucleotide sugar dehydrogenase	WP_041484402.:	1200739..1202151	Amino sugar and nucleotide sugar r	COG1004
WP_041484402.1_1330	CUST_6933_PI442190926	MIT9215	HLII	WP_041484402.1_1330	TGGACTAAATGAAGTTGCGGGTTACTGGAGGAAGGTAATTGAAATAAATTCTTGGCAACA	ugd	UDP-glucose 6-dehydrogenase/nucleotide sugar dehydrogenase	WP_041484402.:	1200739..1202151	Amino sugar and nucleotide sugar r	COG1004
WP_041484402.1_1330	CUST_6934_PI442190926	MIT9215	HLII	WP_041484402.1_1330	AACCTTGTTGGGAATTTCAATCAAACTTATATAGTGCATTTGAAAATGCCCACGCAGTCA	ugd	UDP-glucose 6-dehydrogenase/nucleotide sugar dehydrogenase	WP_041484402.:	1200739..1202151	Amino sugar and nucleotide sugar r	COG1004
WP_041484402.1_1330	CUST_6935_PI442190926	MIT9215	HLII	WP_041484402.1_1330	GGAAAAGATAATAGAATTGGCTCTAAATTTTTAAAATCTGGGCCTGGATTTGGGGGGAGT	ugd	UDP-glucose 6-dehydrogenase/nucleotide sugar dehydrogenase	WP_041484402.:	1200739..1202151	Amino sugar and nucleotide sugar r	COG1004
WP_041484402.1_1330	CUST_6936_PI442190926	MIT9215	HLII	WP_041484402.1_1330	CGAGAGAGTCTCCTGCTATTTCCATATGCAGAAATCTAATAAATGAGGGAGCTTTTCTGT	ugd	UDP-glucose 6-dehydrogenase/nucleotide sugar dehydrogenase	WP_041484402.:	1200739..1202151	Amino sugar and nucleotide sugar r	COG1004
WP_041484402.1_1330	CUST_6937_PI442190926	MIT9215	HLII	WP_041484402.1_1330	TCAATTTCGGCAATTTGTGAAGTTACAGGAGCAGATATAAAGGATGTTTCTTTTGCAATT	ugd	UDP-glucose 6-dehydrogenase/nucleotide sugar dehydrogenase	WP_041484402.:	1200739..1202151	Amino sugar and nucleotide sugar r	COG1004
WP_012008076.1_1332	CUST_6944_PI442190926	MIT9215	HLII	WP_012008076.1_1332	AGCAGCGCTTCAATATATGGGGAACCTGAATATTTACCTATTGATGAGATGCATCCCCTT	galE	UDP-glucose 4-epimerase	WP_012008076.:	1203391..1204395	Galactose metabolism	COG1087
WP_012008076.1_1332	CUST_6945_PI442190926	MIT9215	HLII	WP_012008076.1_1332	TCCACTTGGGGCACATCAGTTTGGATTTATTGGAGATGATCCATTCTCAAAAAATTCACA	galE	UDP-glucose 4-epimerase	WP_012008076.:	1203391..1204395	Galactose metabolism	COG1087
WP_012008076.1_1332	CUST_6946_PI442190926	MIT9215	HLII	WP_012008076.1_1332	AAGGTGATGTCTCTTCATGCTACGCAGATCCATCTAGGGCAAATACTTTTTTAGATTGGA	galE	UDP-glucose 4-epimerase	WP_012008076.:	1203391..1204395	Galactose metabolism	COG1087
WP_012008076.1_1332	CUST_6947_PI442190926	MIT9215	HLII	WP_012008076.1_1332	TGCCATCTATTATCAGAGCGGCACTTGGATTGAGTCGTAAAATAAAAATTTATGGCGATG	galE	UDP-glucose 4-epimerase	WP_012008076.:	1203391..1204395	Galactose metabolism	COG1087
WP_012008076.1_1332	CUST_6948_PI442190926	MIT9215	HLII	WP_012008076.1_1332	TGGAAGAGGTTTTAGCGTTCTGGAAGTAATTAATACATTTGAAATGGTAACTGGAAAGCA	galE	UDP-glucose 4-epimerase	WP_012008076.:	1203391..1204395	Galactose metabolism	COG1087
WP_012008076.1_1332	CUST_6949_PI442190926	MIT9215	HLII	WP_012008076.1_1332	TATGTAGGAGATTCAGTTAAGAGTCCTTTAGATTACTATGATGTAAACGTGCTTGGGACT	galE	UDP-glucose 4-epimerase	WP_012008076.:	1203391..1204395	Galactose metabolism	COG1087
WP_012008130.1_1387	CUST_7270_PI442190926	MIT9215	HLII	WP_012008130.1_1387	CTTACAAAGCCAAAGAATTAGGTTATTACCCTGAGGTTGTCTTGGCAGGCCGCAAAATTA	ugd	UDP-glucose 6-dehydrogenase/nucleotide sugar dehydrogenase	WP_012008130.:	1263195..1264538	Amino sugar and nucleotide sugar r	COG1004
WP_012008130.1_1387	CUST_7271_PI442190926	MIT9215	HLII	WP_012008130.1_1387	TATCTTGCTCCAAGTATCAAAGTAGCTGAAGCCGCAAAGGTTATTGAGAATACACAGCGT	ugd	UDP-glucose 6-dehydrogenase/nucleotide sugar dehydrogenase	WP_012008130.:	1263195..1264538	Amino sugar and nucleotide sugar r	COG1004
WP_012008130.1_1387	CUST_7272_PI442190926	MIT9215	HLII	WP_012008130.1_1387	AAGTAACAAGTGGGAGTACCCCATATGTAGCTGAAAAAGTTGACATGCTTTATAGAATGA	ugd	UDP-glucose 6-dehydrogenase/nucleotide sugar dehydrogenase	WP_012008130.:	1263195..1264538	Amino sugar and nucleotide sugar r	COG1004
WP_012008130.1_1387	CUST_7273_PI442190926	MIT9215	HLII	WP_012008130.1_1387	AAAGGCGGCATCAACAAAATGGAATTTTATTAATTTTAGACCAGGTTTAGTAGGAGGTCA	ugd	UDP-glucose 6-dehydrogenase/nucleotide sugar dehydrogenase	WP_012008130.:	1263195..1264538	Amino sugar and nucleotide sugar r	COG1004
WP_012008130.1_1387	CUST_7274_PI442190926	MIT9215	HLII	WP_012008130.1_1387	AGGGATAACTTTCAAAGAAAATTGCCCAGATATTAGAAACACGAAAGTTATTGATTTGAT	ugd	UDP-glucose 6-dehydrogenase/nucleotide sugar dehydrogenase	WP_012008130.:	1263195..1264538	Amino sugar and nucleotide sugar r	COG1004
WP_012008130.1_1387	CUST_7275_PI442190926	MIT9215	HLII	WP_012008130.1_1387	TGCTTTGATAAATGAATTGGCAATTATTTTCAATAAAATTGGGATTGATACACATGATGT	ugd	UDP-glucose 6-dehydrogenase/nucleotide sugar dehydrogenase	WP_012008130.:	1263195..1264538	Amino sugar and nucleotide sugar r	COG1004
WP_012008167.1_1423	CUST_7473_PI442190926	MIT9215	HLII	WP_012008167.1_1423	AGTTCCACTAGAAGCTACCTTATCCGATGCGATTGATCTTACTGGACCGATTAAACCTCT		polysaccharide biosynthesis/export family protein	WP_012008167.:	1300315..1301595	Other sugar transporters	COG1596
WP_012008167.1_1423	CUST_7474_PI442190926	MIT9215	HLII	WP_012008167.1_1423	ACCCTTTCTGATGTTATTAGAAAAGCCGGCGGCATAACTTCTAAAACTGATTTATCTCGA		polysaccharide biosynthesis/export family protein	WP_012008167.:	1300315..1301595	Other sugar transporters	COG1596
WP_012008167.1_1423	CUST_7475_PI442190926	MIT9215	HLII	WP_012008167.1_1423	TCAGGTATTTCTCCAAAATTCATCTCAGTAAATGTTTTTGGTAGAGTCGAAACTCCTGGA		polysaccharide biosynthesis/export family protein	WP_012008167.:	1300315..1301595	Other sugar transporters	COG1596
WP_012008167.1_1423	CUST_7476_PI442190926	MIT9215	HLII	WP_012008167.1_1423	TTCATATTCAGCAAGGGCGGTAAAAGGCTCTAAAAGAAATCCCTATTTAAAAGAAGATGA		polysaccharide biosynthesis/export family protein	WP_012008167.:	1300315..1301595	Other sugar transporters	COG1596
WP_012008167.1_1423	CUST_7477_PI442190926	MIT9215	HLII	WP_012008167.1_1423	TGGAAAAACTACTGGTGTATTAAATGAGTTCACTGCTCCATTTGTTGGGATTTATTCAAC		polysaccharide biosynthesis/export family protein	WP_012008167.:	1300315..1301595	Other sugar transporters	COG1596
WP_012008167.1_1423	CUST_7478_PI442190926	MIT9215	HLII	WP_012008167.1_1423	TGGAGATAGTTTGTTTTTTCCTAAATTAAGTAATGCTGATCCAAATCAAATCCCAAAGTC		polysaccharide biosynthesis/export family protein	WP_012008167.:	1300315..1301595	Other sugar transporters	COG1596
WP_012008176.1_1432	CUST_7527_PI442190926	MIT9215	HLII	WP_012008176.1_1432	ACTGCAGCATTCTTTGGGGATGGGACTTGCAATAATGGGCAGTTTTTTGAATGTTTAAAT	pdhA	Pyruvate dehydrogenase E1 component alpha subunit	WP_012008176.:	1309929..1311002	Glycolysis/Krebs cycle	COG1071
WP_012008176.1_1432	CUST_7528_PI442190926	MIT9215	HLII	WP_012008176.1_1432	CACAAAGAGCAATAGAACGTGCTAGAGCAGGAGAAGGTCCGACACTTTTAGAATGTTTGA	pdhA	Pyruvate dehydrogenase E1 component alpha subunit	WP_012008176.:	1309929..1311002	Glycolysis/Krebs cycle	COG1071
WP_012008176.1_1432	CUST_7529_PI442190926	MIT9215	HLII	WP_012008176.1_1432	CAGCTGCTTTTGGTATGCAAGGCGAGGAAATTGATGGAATGGATGTATTAGCAGTTAGAG	pdhA	Pyruvate dehydrogenase E1 component alpha subunit	WP_012008176.:	1309929..1311002	Glycolysis/Krebs cycle	COG1071
WP_012008176.1_1432	CUST_7530_PI442190926	MIT9215	HLII	WP_012008176.1_1432	CGGAGTCAGTTAAAAATGCTATTGAAGCACCCGAACCCCCATCGCAAGAATTAACTAAAT	pdhA	Pyruvate dehydrogenase E1 component alpha subunit	WP_012008176.:	1309929..1311002	Glycolysis/Krebs cycle	COG1071
WP_012008176.1_1432	CUST_7531_PI442190926	MIT9215	HLII	WP_012008176.1_1432	AAGGCCGAGGTGGATCCATGCACTTATTTTCAAAAGAGCATCACTTACTAGGAGGATATG	pdhA	Pyruvate dehydrogenase E1 component alpha subunit	WP_012008176.:	1309929..1311002	Glycolysis/Krebs cycle	COG1071
WP_012008176.1_1432	CUST_7532_PI442190926	MIT9215	HLII	WP_012008176.1_1432	CCAGTTGCTTTAGGAGCAGCCTTTTCAAGTAAATACAAAAAGGAAGTCGCCGGTAATTCT	pdhA	Pyruvate dehydrogenase E1 component alpha subunit	WP_012008176.:	1309929..1311002	Glycolysis/Krebs cycle	COG1071
WP_012008198.1_1453	CUST_7650_PI442190926	MIT9215	HLII	WP_012008198.1_1453	ACCATTTTCTTTTGAAGGGAAAAGAAGAATGCGTCAAGCAGAAGAAGGAATCGCAAGACT	ftsZ	cell division protein FtsZ	WP_012008198.:	1333132..1334241	Cell Division	COG0206
WP_012008198.1_1453	CUST_7651_PI442190926	MIT9215	HLII	WP_012008198.1_1453	TTGTTGATCAAGAAGCAAATATAATAGTTGGTGCAGTGGTTGATGAGGCAATGGAAGGGG	ftsZ	cell division protein FtsZ	WP_012008198.:	1333132..1334241	Cell Division	COG0206
WP_012008198.1_1453	CUST_7652_PI442190926	MIT9215	HLII	WP_012008198.1_1453	GCCCAGGATTAGTTAATGTTGATTTCGCAGATGTAAGATCAGTCATGACAGAAGCGGGTA	ftsZ	cell division protein FtsZ	WP_012008198.:	1333132..1334241	Cell Division	COG0206
WP_012008198.1_1453	CUST_7653_PI442190926	MIT9215	HLII	WP_012008198.1_1453	TAGAGGCAGCACAGGCAGCAATGAATAGTCCTTTATTAGAAGCAGCTAGAATTGATGGAG	ftsZ	cell division protein FtsZ	WP_012008198.:	1333132..1334241	Cell Division	COG0206
WP_012008198.1_1453	CUST_7654_PI442190926	MIT9215	HLII	WP_012008198.1_1453	ATAGCAGGAGCACCTCTTCAAGAAGCATTTAGGAATGCTGATGATGTTTTAAGGATGGGA	ftsZ	cell division protein FtsZ	WP_012008198.:	1333132..1334241	Cell Division	COG0206
WP_012008198.1_1453	CUST_7655_PI442190926	MIT9215	HLII	WP_012008198.1_1453	CACCCGTAGTTGCAGAAGTGGCTAAACAAAGTGGTGCTTTAACAGTAGGCATAGTAAC	ftsZ	cell division protein FtsZ	WP_012008198.:	1333132..1334241	Cell Division	COG0206
WP_012008208.1_1468	CUST_7728_PI442190926	MIT9215	HLII	WP_012008208.1_1468	ACGAAGACCCAGAGAAACCAGCAGGATTATATACTGCGTGGATGGTACTTATTCAGAAGA	glcH	GPH family sugar transporter	WP_012008208.:	1345728..1347074	GlcH sugar transporter	COG2211
WP_012008208.1_1468	CUST_7729_PI442190926	MIT9215	HLII	WP_012008208.1_1468	TACTGAACGTACCAACATATATTGCGAAGTGGATCCCGATACCTTTCCAAATTAGCGCTT	glcH	GPH family sugar transporter	WP_012008208.:	1345728..1347074	GlcH sugar transporter	COG2211
WP_012008208.1_1468	CUST_7730_PI442190926	MIT9215	HLII	WP_012008208.1_1468	AATCTGTCTTATTGGCATTGGAGCTTCAACCGCTTTTCTTATCCCTTGGTCACTACTTCC	glcH	GPH family sugar transporter	WP_012008208.:	1345728..1347074	GlcH sugar transporter	COG2211
WP_012008208.1_1468	CUST_7731_PI442190926	MIT9215	HLII	WP_012008208.1_1468	TTCTTCTCTGGTGCGCCTTACAATTAATGCAAACAGTAGCGTTAATCTATGTCGAGGATG	glcH	GPH family sugar transporter	WP_012008208.:	1345728..1347074	GlcH sugar transporter	COG2211
WP_012008208.1_1468	CUST_7732_PI442190926	MIT9215	HLII	WP_012008208.1_1468	ACAGGATTTCAGCGTTAAACTTTGGAGCGATTATGTGGATTATTTCATGTACAGCAGCTT	glcH	GPH family sugar transporter	WP_012008208.:	1345728..1347074	GlcH sugar transporter	COG2211
WP_012008208.1_1468	CUST_7733_PI442190926	MIT9215	HLII	WP_012008208.1_1468	ACCTTCATTATCAAAAATTTCAGGCGCTGGAGATAGTTTATTCCTAAATGCCAGCAACAT	glcH	GPH family sugar transporter	WP_012008208.:	1345728..1347074	GlcH sugar transporter	COG2211
WP_012008224.1_1487	CUST_7841_PI442190926	MIT9215	HLII	WP_012008224.1_1487	CTTGATGATATGTGTGGTGGGGGATATTTTCAAGATTCCATCATTCTCGCTACTGGAGCT	kaiC	circadian clock protein KaiC	WP_012008224.:	1364860..1366398	Circadian Rhythm	COG0467
WP_012008224.1_1487	CUST_7842_PI442190926	MIT9215	HLII	WP_012008224.1_1487	CCAGAGGTGTGAGCTTGAATGCATTTAGACAGTTCGTAATAGCCGTAACGGGTTATACAA	kaiC	circadian clock protein KaiC	WP_012008224.:	1364860..1366398	Circadian Rhythm	COG0467
WP_012008224.1_1487	CUST_7843_PI442190926	MIT9215	HLII	WP_012008224.1_1487	TGAGATGGCACGAGCTTTAAATGTCTTTAAAATGCGGGGATCATGGCATGATAAACGAAT	kaiC	circadian clock protein KaiC	WP_012008224.:	1364860..1366398	Circadian Rhythm	COG0467
WP_012008224.1_1487	CUST_7844_PI442190926	MIT9215	HLII	WP_012008224.1_1487	TGAACAAATATTTAGTGGTGCGCCTCACAGAGTTGTGCCTGATCAAAATGTTCAAAATGT	kaiC	circadian clock protein KaiC	WP_012008224.:	1364860..1366398	Circadian Rhythm	COG0467
WP_012008224.1_1487	CUST_7845_PI442190926	MIT9215	HLII	WP_012008224.1_1487	ACTTTTTGCATATGAAGAATCTAGGGCTCAATTGCTTCGGAATGCTACTAGTTGGGGTAT	kaiC	circadian clock protein KaiC	WP_012008224.:	1364860..1366398	Circadian Rhythm	COG0467
WP_012008224.1_1487	CUST_7846_PI442190926	MIT9215	HLII	WP_012008224.1_1487	AAAAACGCAAATTACTCAATTTAAACCAAAGAGGATGGCAATTGATTCTCTCTCTGCGTT	kaiC	circadian clock protein KaiC	WP_012008224.:	1364860..1366398	Circadian Rhythm	COG0467
WP_002807968.1_1488	CUST_7847_PI442190926	MIT9215	HLII	WP_002807968.1_1488	AGTTTTAATTGGTTTAGATCTACTTTTTGATGAATTAACTGAAACTGAATATAGTGGAGA	kaiB	Circadian clock protein KaiB	WP_002807968.:	1366470..1366787	Circadian Rhythm	COG4251
WP_002807968.1_1488	CUST_7848_PI442190926	MIT9215	HLII	WP_002807968.1_1488	AGCAACACCAACTTTAGCCAAAATTTTACCGCCACCAGTGAGAAAAATAATTGGTGATCT	kaiB	Circadian clock protein KaiB	WP_002807968.:	1366470..1366787	Circadian Rhythm	COG4251
WP_002807968.1_1488	CUST_7849_PI442190926	MIT9215	HLII	WP_002807968.1_1488	AGGAGTTTATGCTTTGAAGGTTATTGATGTACTTAAGCAGCCACAACTTGCAGAAGAAGA	kaiB	Circadian clock protein KaiB	WP_002807968.:	1366470..1366787	Circadian Rhythm	COG4251
WP_002807968.1_1488	CUST_7850_PI442190926	MIT9215	HLII	WP_002807968.1_1488	AACTCTATGTTGCTGGAAATACTCCTAATTCAATGCGAGCTTTAAATACTTTAAGAGAAA	kaiB	Circadian clock protein KaiB	WP_002807968.:	1366470..1366787	Circadian Rhythm	COG4251
WP_012008307.1_1557	CUST_8172_PI442190926	MIT9215	HLII	WP_012008307.1_1557	AAATGGTAATAGAGGTTGGGCAACAGTAAAACCCGCTCCTGATCCAAAAGTTAATGATGC	ctaC/coxB	Cytochrome c oxidase subunit II	WP_012008307.:	1423139..1424074	Respiration	COG1622
WP_012008307.1_1557	CUST_8173_PI442190926	MIT9215	HLII	WP_012008307.1_1557	TGGACCAATTGACGTAATTTCGAGGCAATGGAACTGGGAGTTTGTTTATCCAAATGGAAT	ctaC/coxB	Cytochrome c oxidase subunit II	WP_012008307.:	1423139..1424074	Respiration	COG1622
WP_012008307.1_1557	CUST_8174_PI442190926	MIT9215	HLII	WP_012008307.1_1557	AGGATGTTCAGGGACAATGGCTTGGATTTTAGTTTTCAATAGAGCTCCCAAGTATGATGA	ctaC/coxB	Cytochrome c oxidase subunit II	WP_012008307.:	1423139..1424074	Respiration	COG1622
WP_012008307.1_1557	CUST_8175_PI442190926	MIT9215	HLII	WP_012008307.1_1557	TCTCTAACACCAACTAAAGAGGGAGTATTTAGATTAAGAGATGCAATGTTTAGTGGCGCA	ctaC/coxB	Cytochrome c oxidase subunit II	WP_012008307.:	1423139..1424074	Respiration	COG1622
WP_012008307.1_1557	CUST_8176_PI442190926	MIT9215	HLII	WP_012008307.1_1557	TGTAAAAAAAGAACTTCAAAATGGATTAAATCCTGCTGGCAGTTTGTACCAGAAAAGGCT	ctaC/coxB	Cytochrome c oxidase subunit II	WP_012008307.:	1423139..1424074	Respiration	COG1622
WP_012008307.1_1557	CUST_8177_PI442190926	MIT9215	HLII	WP_012008307.1_1557	TGGAGAACCTTTAGAGGGCAATCTAAAACTTGAAATTATTTGGACAATAGTTCCTCTTCT	ctaC/coxB	Cytochrome c oxidase subunit II	WP_012008307.:	1423139..1424074	Respiration	COG1622
WP_012008308.1_1558	CUST_8178_PI442190926	MIT9215	HLII	WP_012008308.1_1558	GAATGGCTACTTCCCTCTCCACCTCCTCACGAAAATTTTGAAGAAGATATCCCAACTGTT	cyoB/coxA	Cytochrome c oxidase subunit I	WP_012008308.:	1424083..1425774	Respiration	COG0843
WP_012008308.1_1558	CUST_8179_PI442190926	MIT9215	HLII	WP_012008308.1_1558	TCATTGGGCTGGTTTATTGGGAATGCCTAGAAGAGTTGCATCATATGATCCTGAATTTGC	cyoB/coxA	Cytochrome c oxidase subunit I	WP_012008308.:	1424083..1425774	Respiration	COG0843
WP_012008308.1_1558	CUST_8180_PI442190926	MIT9215	HLII	WP_012008308.1_1558	ATGCTCATTGCTGTTCCAACAGGTGTAAAAGTATTTGCATGGATAGCTACATTATGGGGC	cyoB/coxA	Cytochrome c oxidase subunit I	WP_012008308.:	1424083..1425774	Respiration	COG0843
WP_012008308.1_1558	CUST_8181_PI442190926	MIT9215	HLII	WP_012008308.1_1558	TTGAATATGGTAAGTTCATGGGTCAGAGGAAAATCAGCTCCACCTAATCCTTGGAAAGCT	cyoB/coxA	Cytochrome c oxidase subunit I	WP_012008308.:	1424083..1425774	Respiration	COG0843
WP_012008308.1_1558	CUST_8182_PI442190926	MIT9215	HLII	WP_012008308.1_1558	TGCTTGCAACAGTGCCAGTAGATATACATGTTGGAAATACATATTTTGTGGTTGCCCATT	cyoB/coxA	Cytochrome c oxidase subunit I	WP_012008308.:	1424083..1425774	Respiration	COG0843
WP_012008308.1_1558	CUST_8183_PI442190926	MIT9215	HLII	WP_012008308.1_1558	TGGAATGTACTAGCGAGTATTGGAGCTTTTATTCTTGGCGTTGCCTCTATCCCTTTTATC	cyoB/coxA	Cytochrome c oxidase subunit I	WP_012008308.:	1424083..1425774	Respiration	COG0843
WP_012008309.1_1559	CUST_8184_PI442190926	MIT9215	HLII	WP_012008309.1_1559	TACGTTGAGTGGTCTGAACTCTCTTTCAGCTTACAAAGTGGAGCATTTGGAGGAATGTTT	cyoC	heme-copper oxidase subunit III	WP_012008309.:	1425778..1426374	Respiration	COG1845
WP_012008309.1_1559	CUST_8185_PI442190926	MIT9215	HLII	WP_012008309.1_1559	TTAAAATTAGTGCACCTGTCTGGTACCCCGAAGGAATCAATGGGATTGATGTGAAAATGC	cyoC	heme-copper oxidase subunit III	WP_012008309.:	1425778..1426374	Respiration	COG1845
WP_012008309.1_1559	CUST_8186_PI442190926	MIT9215	HLII	WP_012008309.1_1559	GGCGGAGAGATGGGTGTTGAATCAGTAAGCCTTTTTTGGCATTTTGTTGATGTTATTTGG	cyoC	heme-copper oxidase subunit III	WP_012008309.:	1425778..1426374	Respiration	COG1845
WP_012008309.1_1559	CUST_8187_PI442190926	MIT9215	HLII	WP_012008309.1_1559	ACTTACTGGTTTCCATGGCCTACACGTAATTACAGGGATATTACTAATGGGGCTTATGTT	cyoC	heme-copper oxidase subunit III	WP_012008309.:	1425778..1426374	Respiration	COG1845
WP_012008309.1_1559	CUST_8188_PI442190926	MIT9215	HLII	WP_012008309.1_1559	TGGGGTTTCAGAATTATTTGGTTCATAACCATGTTGATGGGAAGTTACTTTGTTTATGGT	cyoC	heme-copper oxidase subunit III	WP_012008309.:	1425778..1426374	Respiration	COG1845
WP_012008309.1_1559	CUST_8189_PI442190926	MIT9215	HLII	WP_012008309.1_1559	GCGCACAGAAAATCAGAATTTAACTTTAAATCATAAGCCAGGTCACATTAAACATGACGG	cyoC	heme-copper oxidase subunit III	WP_012008309.:	1425778..1426374	Respiration	COG1845
WP_002806495.1_1598	CUST_8364_PI442190926	MIT9215	HLII	WP_002806495.1_1598	CGGAAAAAATTAAAGCCTTAAAACAAGTTTCGAAAGCTGAGGCTAGGATTCAAGCCGCAA	atpC	ATP synthase epsilon subunit	WP_002806495.:	1456892..1457296	Energy Metabolism	COG0355
WP_002806495.1_1598	CUST_8365_PI442190926	MIT9215	HLII	WP_002806495.1_1598	TGCTGAAATTGGTTCTGAAATTAATGTTCAAAATGCCGAACAAGATCTTAAAGAAGCAAA	atpC	ATP synthase epsilon subunit	WP_002806495.:	1456892..1457296	Energy Metabolism	COG0355
WP_002806495.1_1598	CUST_8366_PI442190926	MIT9215	HLII	WP_002806495.1_1598	TTGGCGTTTTAAGGCTAAGAATGAATTCTCAATGGAAATCCATAGCATTAATGGGAGGCT	atpC	ATP synthase epsilon subunit	WP_002806495.:	1456892..1457296	Energy Metabolism	COG0355
WP_002806495.1_1598	CUST_8367_PI442190926	MIT9215	HLII	WP_002806495.1_1598	AGGTGAAGCTGAAGAAGTAATCCTTCCTAGTACTACTGGTCAGCTTGGTATATTACCGGG	atpC	ATP synthase epsilon subunit	WP_002806495.:	1456892..1457296	Energy Metabolism	COG0355
WP_002805160.1_1609	CUST_8423_PI442190926	MIT9215	HLII	WP_002805160.1_1609	CAATCATTGAAACTCAGGCAGGGGACGTTTCGGCTTACATTCCAACTAACGTTATTTCAA	atpA	ATP synthase alpha subunit	WP_002805160.:	1466582..1468099	Energy Metabolism	COG0056
WP_002805160.1_1609	CUST_8424_PI442190926	MIT9215	HLII	WP_002805160.1_1609	CCACCAGGAAGAGAGGCTTATCCTGGAGACGTGTTTTACTTACACAGCAGATTACTAGAA	atpA	ATP synthase alpha subunit	WP_002805160.:	1466582..1468099	Energy Metabolism	COG0056
WP_002805160.1_1609	CUST_8425_PI442190926	MIT9215	HLII	WP_002805160.1_1609	TGAGCTAGCTGCTTTTTCTCAATTTGCATCTGATCTTGATGAAGCAACTCAGCAACAACT	atpA	ATP synthase alpha subunit	WP_002805160.:	1466582..1468099	Energy Metabolism	COG0056
WP_002805160.1_1609	CUST_8427_PI442190926	MIT9215	HLII	WP_002805160.1_1609	AGTTATTGAAGCAACCTCAATTCTCTCCGCTAAACCTTGCAGAACAAGTTGCAGTTGTTT	atpA	ATP synthase alpha subunit	WP_002805160.:	1466582..1468099	Energy Metabolism	COG0056
WP_002805160.1_1609	CUST_8428_PI442190926	MIT9215	HLII	WP_002805160.1_1609	AGGTCTTATTGATGAGGTTCCTGTTGAGGAAGTCACTAAATTCGCAACTGAACTTAGGGA	atpA	ATP synthase alpha subunit	WP_002805160.:	1466582..1468099	Energy Metabolism	COG0056
WP_012008357.1_1614	CUST_8451_PI442190926	MIT9215	HLII	WP_012008357.1_1614	TACTAGTGCAATTCAGGCATTGATTTTTGCAACTTTAGCGGCCTACTACATTGGAGAAGC	atpI	ATP synthase subunit A	WP_012008357.:	1470122..1470847	Energy Metabolism	COG0356
WP_012008357.1_1614	CUST_8452_PI442190926	MIT9215	HLII	WP_012008357.1_1614	GTGGAGAATTAGGAGCACCTACCGCGGATATCAATACAACTATAGCCTTGGCTTTATTGG	atpI	ATP synthase subunit A	WP_012008357.:	1470122..1470847	Energy Metabolism	COG0356
WP_012008357.1_1614	CUST_8453_PI442190926	MIT9215	HLII	WP_012008357.1_1614	GGACTTCAAAACTTGCTTGAGTTCCTTTGGGATTACATAAGAGATCTTGCAAGGACGCAA	atpI	ATP synthase subunit A	WP_012008357.:	1470122..1470847	Energy Metabolism	COG0356
WP_012008357.1_1614	CUST_8454_PI442190926	MIT9215	HLII	WP_012008357.1_1614	TGCAGGCTTAAGCAATAAGGGTTGGAGATACTTCGAATACTATGTTCACCCAACTCCTAT	atpI	ATP synthase subunit A	WP_012008357.:	1470122..1470847	Energy Metabolism	COG0356
WP_012008357.1_1614	CUST_8455_PI442190926	MIT9215	HLII	WP_012008357.1_1614	AGCACTAGAAGTTGGTCAACATCTTTATTGGCAAATTGGAAATATCAGACTTCATGGGCA	atpI	ATP synthase subunit A	WP_012008357.:	1470122..1470847	Energy Metabolism	COG0356
WP_012008357.1_1614	CUST_8456_PI442190926	MIT9215	HLII	WP_012008357.1_1614	TGGTGTTCTAGTCTTTTTAGTTCCGCTAATTCTCCCAATACCTGTTATGTTCCTAGGCTT	atpI	ATP synthase subunit A	WP_012008357.:	1470122..1470847	Energy Metabolism	COG0356
WP_012008366.1_1624	CUST_8507_PI442190926	MIT9215	HLII	WP_012008366.1_1624	ATGCGAAGCCTTATCAATGGTTTGCACTCAGATCATGGGTCTTGAATATGCAGTTTCAAT	fumC	class II fumarate hydratase	WP_012008366.:	1478521..1479906	Krebs cycle and related genes	COG0114
WP_012008366.1_1624	CUST_8508_PI442190926	MIT9215	HLII	WP_012008366.1_1624	CTGGTTACAGCCATAGTGCCAAAAGTTGGTTATGAAAAAGCAGCTGAAATTGCAAACCTT	fumC	class II fumarate hydratase	WP_012008366.:	1478521..1479906	Krebs cycle and related genes	COG0114
WP_012008366.1_1624	CUST_8509_PI442190926	MIT9215	HLII	WP_012008366.1_1624	TTGTATTTCTTACCTCTGGGAGGAACTGCAGTTGGAACAGGGATTAATTGTCCAAAAGGA	fumC	class II fumarate hydratase	WP_012008366.:	1478521..1479906	Krebs cycle and related genes	COG0114
WP_012008366.1_1624	CUST_8510_PI442190926	MIT9215	HLII	WP_012008366.1_1624	TGGCCTCTCATGATCGTCTAGCTCAAGTAATGAGTCAGTTAAAAATATTAGCAGGTGCAT	fumC	class II fumarate hydratase	WP_012008366.:	1478521..1479906	Krebs cycle and related genes	COG0114
WP_012008366.1_1624	CUST_8511_PI442190926	MIT9215	HLII	WP_012008366.1_1624	AATTATTCCTCAAAATGAACCTGGAAGTTCTATCATGCCGGGTAAAGTGAATCCAACTCA	fumC	class II fumarate hydratase	WP_012008366.:	1478521..1479906	Krebs cycle and related genes	COG0114
WP_012008366.1_1624	CUST_8512_PI442190926	MIT9215	HLII	WP_012008366.1_1624	TGGCAAATTCTAGCGGCACTTTACAGATGAATGAATATAAGCCTCTTATTGGGTTTAAT	fumC	class II fumarate hydratase	WP_012008366.:	1478521..1479906	Krebs cycle and related genes	COG0114
WP_012008407.1_1669	CUST_8750_PI442190926	MIT9215	HLII	WP_012008407.1_1669	AATCAGGTGAAAGGTTTGCTGTAAGAAATAGTGGAGCAGTAGCTGTAACTGAAGGAGCAG	glsF	Ferredoxin-dependent glutamate synthase	WP_012008407.:	1528216..1532790	Nitrogen Metabolism	COG0069
WP_012008407.1_1669	CUST_8751_PI442190926	MIT9215	HLII	WP_012008407.1_1669	ATCATTGCTGTGAGTATATGACAGGGGGCAAAATTGTAATCCTTGGCTCTACAGGAAGAA	glsF	Ferredoxin-dependent glutamate synthase	WP_012008407.:	1528216..1532790	Nitrogen Metabolism	COG0069
WP_012008407.1_1669	CUST_8752_PI442190926	MIT9215	HLII	WP_012008407.1_1669	AATGGAACTGTATTGGAGGACTCGATTTTGACAGATGCTCAATTCATAGATGCGGTGACT	glsF	Ferredoxin-dependent glutamate synthase	WP_012008407.:	1528216..1532790	Nitrogen Metabolism	COG0069
WP_012008407.1_1669	CUST_8753_PI442190926	MIT9215	HLII	WP_012008407.1_1669	GCAATAAGGGTTTTGAGGGCGCAATTAATCTAATTTTTAATGGTCATGCGGGTCAAAGCT	glsF	Ferredoxin-dependent glutamate synthase	WP_012008407.:	1528216..1532790	Nitrogen Metabolism	COG0069
WP_012008407.1_1669	CUST_8754_PI442190926	MIT9215	HLII	WP_012008407.1_1669	TAGATAAGAATTCATCGGAGCAAGTCATTTTGGGCAATACTTGTCTGTATGGCGCTACGG	glsF	Ferredoxin-dependent glutamate synthase	WP_012008407.:	1528216..1532790	Nitrogen Metabolism	COG0069
WP_012008407.1_1669	CUST_8755_PI442190926	MIT9215	HLII	WP_012008407.1_1669	TGAGATCAAAAATACCGATAGAAGTGTTTGTGCAAAAATTTCAGGAGAGCTTGCTCAGCA	glsF	Ferredoxin-dependent glutamate synthase	WP_012008407.:	1528216..1532790	Nitrogen Metabolism	COG0069
WP_002807071.1_1680	CUST_8808_PI442190926	MIT9215	HLII	WP_002807071.1_1680	ATAGGCAAGATGCATTAAGTGCTTTCTTGCCCATTGGTCCTGCTGATTTCTTAGTTCATC	psaB	Photosystem I P700 chlorophyll A apoprotein A2 PsaB	WP_002807071.:	1539154..1541382	Photosystem I	COG2885
WP_002807071.1_1680	CUST_8809_PI442190926	MIT9215	HLII	WP_002807071.1_1680	GCAAGAATTAATTGAGACATTAGTTTGGGCTCATCAGCGCACACCAATAGCTAACCTTGT	psaB	Photosystem I P700 chlorophyll A apoprotein A2 PsaB	WP_002807071.:	1539154..1541382	Photosystem I	COG2885
WP_002807071.1_1680	CUST_8810_PI442190926	MIT9215	HLII	WP_002807071.1_1680	ACGGATATAATCCGTTCGGAGTAAACGCACTATCTCCTTGGGCTTGGATGTTCTTATTTG	psaB	Photosystem I P700 chlorophyll A apoprotein A2 PsaB	WP_002807071.:	1539154..1541382	Photosystem I	COG2885
WP_002807071.1_1680	CUST_8811_PI442190926	MIT9215	HLII	WP_002807071.1_1680	TGGGTAACTTTCTACTGGCATTGGAAACATCTAGCAATATGGCAGGGTAATGTAGCTCAA	psaB	Photosystem I P700 chlorophyll A apoprotein A2 PsaB	WP_002807071.:	1539154..1541382	Photosystem I	COG2885
WP_002807071.1_1680	CUST_8812_PI442190926	MIT9215	HLII	WP_002807071.1_1680	AACTGCATTAATCCTCATTAAAGGAGCTCTTGATGCTAGAGGAACAAAGTTGATCCCCGA	psaB	Photosystem I P700 chlorophyll A apoprotein A2 PsaB	WP_002807071.:	1539154..1541382	Photosystem I	COG2885
WP_002807071.1_1680	CUST_8813_PI442190926	MIT9215	HLII	WP_002807071.1_1680	CCTACTAGTGCTGCTAGTGTCGCAGCAAATTCTTTACCTGGTAATCATTACTGGATGGAT	psaB	Photosystem I P700 chlorophyll A apoprotein A2 PsaB	WP_002807071.:	1539154..1541382	Photosystem I	COG2885
WP_012008418.1_1681	CUST_8814_PI442190926	MIT9215	HLII	WP_012008418.1_1681	ATTGTTTGGGCTCACAACAAATTAAAAGTTGCCCCAACAATTCAACCAAGGGCTTTATCT	psaA	Photosystem I P700 chlorophyll A apoprotein A1 PsaA	WP_012008418.:	1541410..1543695	Photosystem I	COG2885
WP_012008418.1_1681	CUST_8815_PI442190926	MIT9215	HLII	WP_012008418.1_1681	TGGTGGAAAAATTGCAATGAAAGCGATACCACTTGGAACTGCTGACTTAATGATCCATCA	psaA	Photosystem I P700 chlorophyll A apoprotein A1 PsaA	WP_012008418.:	1541410..1543695	Photosystem I	COG2885
WP_012008418.1_1681	CUST_8816_PI442190926	MIT9215	HLII	WP_012008418.1_1681	AGAGACTTCTTATGGGCACAAGCTTCTCAGGTTTTGACAAGCTATGGTCAATCAATCAGT	psaA	Photosystem I P700 chlorophyll A apoprotein A1 PsaA	WP_012008418.:	1541410..1543695	Photosystem I	COG2885
WP_012008418.1_1681	CUST_8817_PI442190926	MIT9215	HLII	WP_012008418.1_1681	CTGATAAAGCTTCTCTAGGTTTCAGATTCCCTTGTGATGGTCCAGGAAGAGGAGGTACTT	psaA	Photosystem I P700 chlorophyll A apoprotein A1 PsaA	WP_012008418.:	1541410..1543695	Photosystem I	COG2885
WP_012008418.1_1681	CUST_8818_PI442190926	MIT9215	HLII	WP_012008418.1_1681	ATTACAACCTGTCTTCGCGCAGTGGGTACAAAATATACAATCTTCAGGAATCGGTACGAC	psaA	Photosystem I P700 chlorophyll A apoprotein A1 PsaA	WP_012008418.:	1541410..1543695	Photosystem I	COG2885
WP_012008418.1_1681	CUST_8819_PI442190926	MIT9215	HLII	WP_012008418.1_1681	TTTTAGGAGCACACTTTATTTGGGCCTTTAGTTTGATGTTCCTCTTCAGTGGACGAGGAT	psaA	Photosystem I P700 chlorophyll A apoprotein A1 PsaA	WP_012008418.:	1541410..1543695	Photosystem I	COG2885
WP_012008463.1_1735	CUST_9111_PI442190926	MIT9215	HLII	WP_012008463.1_1735	GTTGGGGTTTTTGCAGGTTTCACTTTTAAGAAGATTAAGAGACCAGACAAGACAACCCCC	ppc	Phosphoenolpyruvate carboxylase	WP_012008463.:	1584117..1587086	Krebs cycle and related genes	COG2352
WP_012008463.1_1735	CUST_9112_PI442190926	MIT9215	HLII	WP_012008463.1_1735	TAGAAGTGAACTATTGAGGGGGGCACTTTTAACTATCAATGGAATAGCAGCTGGCATGAG	ppc	Phosphoenolpyruvate carboxylase	WP_012008463.:	1584117..1587086	Krebs cycle and related genes	COG2352
WP_012008463.1_1735	CUST_9113_PI442190926	MIT9215	HLII	WP_012008463.1_1735	TAGACTTGACGCTACCCCAGAATGGAATCAATTAATGTCTAGGTTGGCAGAAACGTCAAG	ppc	Phosphoenolpyruvate carboxylase	WP_012008463.:	1584117..1587086	Krebs cycle and related genes	COG2352
WP_012008463.1_1735	CUST_9114_PI442190926	MIT9215	HLII	WP_012008463.1_1735	TATTTGGTTGGACACAAAGTAGATTTCTTTTGCCAAGTTGGTTTGGAGTGGGTACTGCAT	ppc	Phosphoenolpyruvate carboxylase	WP_012008463.:	1584117..1587086	Krebs cycle and related genes	COG2352
WP_012008463.1_1735	CUST_9116_PI442190926	MIT9215	HLII	WP_012008463.1_1735	TCTAAGGTGGATTTGGAAGTTGCCAGATATTATGTTGATACTCTTGGAAGTAGGGAAAAT	ppc	Phosphoenolpyruvate carboxylase	WP_012008463.:	1584117..1587086	Krebs cycle and related genes	COG2352
WP_012008491.1_1766	CUST_9289_PI442190926	MIT9215	HLII	WP_012008491.1_1766	GCGTTAAAACTCAAAGAAATTAGTTACATTCACGCGGCTGGATATCCAGCTGGTGAGATG	glmS	Glutamine--fructose-6-phosphatetransaminase(isomerizing)	WP_012008491.:	1617261..1619156	Amino Acid metabolism	COG0449
WP_012008491.1_1766	CUST_9290_PI442190926	MIT9215	HLII	WP_012008491.1_1766	AGCTCGATAGGAAGGCAGGTTCCAAATATAATTGATATTTGCGCAGGAATAGAAGTGGGA	glmS	Glutamine--fructose-6-phosphatetransaminase(isomerizing)	WP_012008491.:	1617261..1619156	Amino Acid metabolism	COG0449
WP_012008491.1_1766	CUST_9291_PI442190926	MIT9215	HLII	WP_012008491.1_1766	TGCTCAAGAAGCAAAAGCTAGAGATTCATTCTTGATTGGGATTGCCCCTGAATGTAATGG	glmS	Glutamine--fructose-6-phosphatetransaminase(isomerizing)	WP_012008491.:	1617261..1619156	Amino Acid metabolism	COG0449
WP_012008491.1_1766	CUST_9292_PI442190926	MIT9215	HLII	WP_012008491.1_1766	TGCAGCTCATAGAGGTTTAGACGTGGATCAACCAAGAAATTTAGCTAAAAGTGTAACTGT	glmS	Glutamine--fructose-6-phosphatetransaminase(isomerizing)	WP_012008491.:	1617261..1619156	Amino Acid metabolism	COG0449
WP_012008491.1_1766	CUST_9293_PI442190926	MIT9215	HLII	WP_012008491.1_1766	ACGGTCCAATAGCTTTATTAGATAAAAAAGTCCCTGTAATTTCTATTGCTTCCCCTGGTG	glmS	Glutamine--fructose-6-phosphatetransaminase(isomerizing)	WP_012008491.:	1617261..1619156	Amino Acid metabolism	COG0449
WP_012008491.1_1766	CUST_9294_PI442190926	MIT9215	HLII	WP_012008491.1_1766	TGAAAGTTCCTTCCTATAATCAATGGATTTCACCTTTACTTAACATAGTGCCTTTACAAT	glmS	Glutamine--fructose-6-phosphatetransaminase(isomerizing)	WP_012008491.:	1617261..1619156	Amino Acid metabolism	COG0449
WP_011817489.1_12	CUST_67_PI442190916	AS9606	HLII	WP_011817489.1_12	ACTTGGCAGATTACTTAGTGGGTAAAGATGTCCCTTTTAGGACCGCCTATCAAGTTGTTG	argH	Argininosuccinate lyase	WP_011817489.:	14527..15906	Amino Acid metabolism	COG0165
WP_011817489.1_12	CUST_69_PI442190916	AS9606	HLII	WP_011817489.1_12	ATAAATGTGCAACGGGAAGTAGCTTAATGCCGCAGAAAAAAAATCCTGACGTTCCAGAAT	argH	Argininosuccinate lyase	WP_011817489.:	14527..15906	Amino Acid metabolism	COG0165
WP_011817489.1_12	CUST_70_PI442190916	AS9606	HLII	WP_011817489.1_12	AGTTAGTGACAGAGATTTTTGTATAGAGTTTGTTTCTGCATCTGCCTTGTTGATGTCGCA	argH	Argininosuccinate lyase	WP_011817489.:	14527..15906	Amino Acid metabolism	COG0165
WP_011817489.1_12	CUST_71_PI442190916	AS9606	HLII	WP_011817489.1_12	TGTCGTTAAATCAAGAAATAGCGAAGGAGGTACAGGTTTTGTTCAGGTAGAAAAAGAGGT	argH	Argininosuccinate lyase	WP_011817489.:	14527..15906	Amino Acid metabolism	COG0165
WP_011817700.1_219	CUST_1211_PI442190916	AS9606	HLII	WP_011817700.1_219	TTCAAAAAGCCGGATTTAAGCCTGGTGAGCAGGTATCTTTAGCTTTAGATGCTGCTAGTA	eno	Enolase	WP_011817700.:	211249..212541	Glycolysis/Gluconeogenesis	COG0148
WP_011817700.1_219	CUST_1212_PI442190916	AS9606	HLII	WP_011817700.1_219	CACTAGATCGGGTCAGATCAAGACTGGCTCTTTGAGCAGAAGTGAAAGGATTGCAAAATA	eno	Enolase	WP_011817700.:	211249..212541	Glycolysis/Gluconeogenesis	COG0148
WP_011817700.1_219	CUST_1213_PI442190916	AS9606	HLII	WP_011817700.1_219	AGAGGACGGTTTAGCTGAGGATGATTGGGAGGGTTGGTCAGAATTAAACAAAGAATTAGG	eno	Enolase	WP_011817700.:	211249..212541	Glycolysis/Gluconeogenesis	COG0148
WP_011817700.1_219	CUST_1214_PI442190916	AS9606	HLII	WP_011817700.1_219	AATCATTACTTGATCAAAAAGGTCTATCTACTGCTGTAGGCGATGAGGGTGGATTTGCCC	eno	Enolase	WP_011817700.:	211249..212541	Glycolysis/Gluconeogenesis	COG0148
WP_011817700.1_219	CUST_1215_PI442190916	AS9606	HLII	WP_011817700.1_219	AAAATTGAGGAGGAATTAGGAAATCAAGCAAGATTCGCTGGAGCTCTAGGTTTAGGTCCT	eno	Enolase	WP_011817700.:	211249..212541	Glycolysis/Gluconeogenesis	COG0148
WP_011817700.1_219	CUST_1216_PI442190916	AS9606	HLII	WP_011817700.1_219	TGGTTTCACAAGTGTTATAAGTCATAGAAGTGGTGAAACTGAAGATACAACAATCGCAGA	eno	Enolase	WP_011817700.:	211249..212541	Glycolysis/Gluconeogenesis	COG0148
WP_011817718.1_237	CUST_1303_PI442190916	AS9606	HLII	WP_011817718.1_237	TCCAAGAATTAATTTAGGCTCCGCCTCCATAGTTAATAAGCAATCAATAGAAGATTCTTT	eda	2-dehydro-3-deoxyphosphogluconate aldolase	WP_011817718.:	229148..229774	Entner-Dudoroff	COG0800
WP_011817718.1_237	CUST_1304_PI442190916	AS9606	HLII	WP_011817718.1_237	ATTTTTCGATGATGAAATTTTGGGATAAAGATCTTTTCAATTATGCGCAGTCAAAAAGTT	eda	2-dehydro-3-deoxyphosphogluconate aldolase	WP_011817718.:	229148..229774	Entner-Dudoroff	COG0800
WP_011817718.1_237	CUST_1305_PI442190916	AS9606	HLII	WP_011817718.1_237	AGATAGTTCGATAGATATACTCAACTATACAAATATTGATTTCATTGCTGCTGGAGGACT	eda	2-dehydro-3-deoxyphosphogluconate aldolase	WP_011817718.:	229148..229774	Entner-Dudoroff	COG0800
WP_011817718.1_237	CUST_1306_PI442190916	AS9606	HLII	WP_011817718.1_237	AGATTCTTTTTCGGAGTTCCTGAAAACTGAGTCTTTTTTTTTACTTATAAAACCTGAAGA	eda	2-dehydro-3-deoxyphosphogluconate aldolase	WP_011817718.:	229148..229774	Entner-Dudoroff	COG0800
WP_011817718.1_237	CUST_1307_PI442190916	AS9606	HLII	WP_011817718.1_237	ATTAAAGAATATTGAAATAAGTTGGTCTAACAACGAAAATTGGTTCGATTTTGTATCCGA	eda	2-dehydro-3-deoxyphosphogluconate aldolase	WP_011817718.:	229148..229774	Entner-Dudoroff	COG0800
WP_011817718.1_237	CUST_1308_PI442190916	AS9606	HLII	WP_011817718.1_237	AGGGTATAAAGCAGTCGTAATTGGAGATAAAGCTATCAAAAATAAAAAATTTGATCCAAA	eda	2-dehydro-3-deoxyphosphogluconate aldolase	WP_011817718.:	229148..229774	Entner-Dudoroff	COG0800
WP_011817807.1_334	CUST_1817_PI442190916	AS9606	HLII	WP_011817807.1_334	ATCAAAAGAACTATTTCAGGAGAAATAAAATTCAAAAAAGCTGATAAAGCTAATAAAGAT	minE	septum site-determining protein MinE	WP_011817807.:	315202..315528	Cell Division	COG0851
WP_011817807.1_334	CUST_1818_PI442190916	AS9606	HLII	WP_011817807.1_334	GAGGTGGCAGTAAGTTTAGAGACTGAGGATAGAATGACTGCATTAGTTGCCAATTTACCA	minE	septum site-determining protein MinE	WP_011817807.:	315202..315528	Cell Division	COG0851
WP_011817807.1_334	CUST_1819_PI442190916	AS9606	HLII	WP_011817807.1_334	TGACAGAGTTGACATGAGTTCTTTGACAACTGATCTTTTAGATAAAATGAGGAAAGAGAT	minE	septum site-determining protein MinE	WP_011817807.:	315202..315528	Cell Division	COG0851
WP_011817807.1_334	CUST_1820_PI442190916	AS9606	HLII	WP_011817807.1_334	AGGCAGAGAAACGTCTAGTGCTAATACAGCAAGAGAAAGATTACAACTTGTACTGGCTCA	minE	septum site-determining protein MinE	WP_011817807.:	315202..315528	Cell Division	COG0851
WP_080512707.1_335	CUST_1821_PI442190916	AS9606	HLII	WP_080512707.1_335	AGAGTTCGCCCTAACATGATGGCTAGTCAAGAAATGTTATCCATCGAAGATGTTCAAGGG	minD	septum site-determining protein MinD	WP_080512707.:	315530..316345	Cell Division	COG2894
WP_080512707.1_335	CUST_1822_PI442190916	AS9606	HLII	WP_080512707.1_335	ACATAAAAAGGAGCCTAATCTTGCTCTTCTACCTGCTGGAGATCCAAGGATGTTGGATTG	minD	septum site-determining protein MinD	WP_080512707.:	315530..316345	Cell Division	COG2894
WP_080512707.1_335	CUST_1823_PI442190916	AS9606	HLII	WP_080512707.1_335	GGTTGGTAAAACAACTTTAACTGCAAACCTAGGGATAGCACTTGCTAATAGCGGAGCAAC	minD	septum site-determining protein MinD	WP_080512707.:	315530..316345	Cell Division	COG2894
WP_080512707.1_335	CUST_1824_PI442190916	AS9606	HLII	WP_080512707.1_335	CCTGCTGGTGTAGAAGATGGCTTTAAAAATGCTCTTGCAGCCTGTAAAGAAGCTATTGTT	minD	septum site-determining protein MinD	WP_080512707.:	315530..316345	Cell Division	COG2894
WP_080512707.1_335	CUST_1825_PI442190916	AS9606	HLII	WP_080512707.1_335	AATCGCATCATTTATACAGCTCAAGATGTTCTAGACAAGAACTGTCGTCTCGACCAAGCA	minD	septum site-determining protein MinD	WP_080512707.:	315530..316345	Cell Division	COG2894
WP_080512707.1_335	CUST_1826_PI442190916	AS9606	HLII	WP_080512707.1_335	TCCGCAGTGAGGGATGCAGATAGAGTAATAGGAATTCTTAATACTTCAGATATTGAGCCT	minD	septum site-determining protein MinD	WP_080512707.:	315530..316345	Cell Division	COG2894
WP_011817809.1_336	CUST_1827_PI442190916	AS9606	HLII	WP_011817809.1_336	GGGGCAATAGTTTCCGCTAAGAAAAATATTTACGTTTGGGGCAAATTACTAGGGATCGCA	minC	septum site-determining protein MinC	WP_011817809.:	316457..317119	Cell Division	COG0850
WP_011817809.1_336	CUST_1828_PI442190916	AS9606	HLII	WP_011817809.1_336	ACGATATTCTTCACAAAGGAACAGTACGATCGGGAGAAAGAATATCTTCAAACGGAAACC	minC	septum site-determining protein MinC	WP_011817809.:	316457..317119	Cell Division	COG0850
WP_011817809.1_336	CUST_1829_PI442190916	AS9606	HLII	WP_011817809.1_336	AGCAATTGGACCAAAAGAAAAGCCCAAAAATTGCTATCCAGAAATTGCAGTAATAGATCA	minC	septum site-determining protein MinC	WP_011817809.:	316457..317119	Cell Division	COG0850
WP_011817809.1_336	CUST_1830_PI442190916	AS9606	HLII	WP_011817809.1_336	TCCATCAAGGAACCTTTAGAAGCAAAAATTTTCGCAGTCAATGAGTCAATAAGTTCTCAT	minC	septum site-determining protein MinC	WP_011817809.:	316457..317119	Cell Division	COG0850
WP_011817809.1_336	CUST_1831_PI442190916	AS9606	HLII	WP_011817809.1_336	CGCAGGGAAAAGTGGAAATAAAAATGCCTCTATTTCATCACTTTATTTAAACCCTTTACA	minC	septum site-determining protein MinC	WP_011817809.:	316457..317119	Cell Division	COG0850
WP_011817809.1_336	CUST_1832_PI442190916	AS9606	HLII	WP_011817809.1_336	GTTCCTTATGCATTTACTCAAATAATAGAGATACAATAATAAGTGGAAAGTTCTTAAAAA	minC	septum site-determining protein MinC	WP_011817809.:	316457..317119	Cell Division	COG0850
WP_011817955.1_471	CUST_2503_PI442190916	AS9606	HLII	WP_011817955.1_471	GGTCTTGTAACTTTCCTAATTGCGGACGGAATGACTTTTGCTGGATTCTTTGCTGCTTAT	cyoC	heme-copper oxidase subunit III	WP_011817955.:	434046..434648	Respiration	COG1845
WP_011817955.1_471	CUST_2504_PI442190916	AS9606	HLII	WP_011817955.1_471	TGCTTGGCAATCGAGAATCAATGGCGGAAGATTAACTAGTCAAAATATGTTCCCCTTAGA	cyoC	heme-copper oxidase subunit III	WP_011817955.:	434046..434648	Respiration	COG1845
WP_011817955.1_471	CUST_2505_PI442190916	AS9606	HLII	WP_011817955.1_471	AGTGCAACTTTCCATAAAGCAGGCAAAGCACTTTTAAAAGATAAAAACTCTGATTCCCAA	cyoC	heme-copper oxidase subunit III	WP_011817955.:	434046..434648	Respiration	COG1845
WP_011817955.1_471	CUST_2506_PI442190916	AS9606	HLII	WP_011817955.1_471	TGCAAGTACTTTTTATGCTCTTACTGGTTTTCATGGATTACATGTCACTTTAGGAACTTT	cyoC	heme-copper oxidase subunit III	WP_011817955.:	434046..434648	Respiration	COG1845
WP_011817955.1_471	CUST_2507_PI442190916	AS9606	HLII	WP_011817955.1_471	AGCAGTAAATCCATTACCTGATGGTGCTATTTATGAATTAGAACTACCAATACCTACACT	cyoC	heme-copper oxidase subunit III	WP_011817955.:	434046..434648	Respiration	COG1845
WP_011817955.1_471	CUST_2508_PI442190916	AS9606	HLII	WP_011817955.1_471	AGCTGTTGAATTGTACTGGCATTTTGTAGATGGAATATGGGTTATTTTATTTATTATCTT	cyoC	heme-copper oxidase subunit III	WP_011817955.:	434046..434648	Respiration	COG1845
WP_011817956.1_472	CUST_2509_PI442190916	AS9606	HLII	WP_011817956.1_472	TGTTTTTGGAGGTATTACAGGAGTTGCTTTGGCACAGGTACCTTTCGATATTCACGTACA	cyoB/coxA	Cytochrome c oxidase subunit I	WP_011817956.:	434649..436274	Respiration	COG0843
WP_011817956.1_472	CUST_2510_PI442190916	AS9606	HLII	WP_011817956.1_472	AACGCCCCCTTGGATGAGATTATTCTTTACTATCGCAACAGCATTTATTGCTGTTCCAAC	cyoB/coxA	Cytochrome c oxidase subunit I	WP_011817956.:	434649..436274	Respiration	COG0843
WP_011817956.1_472	CUST_2511_PI442190916	AS9606	HLII	WP_011817956.1_472	GCTTTGCTCCTCAACATTGGCTTGGTTTAAATGGAATGCCAAGAAGAGTTGCAGAATATG	cyoB/coxA	Cytochrome c oxidase subunit I	WP_011817956.:	434649..436274	Respiration	COG0843
WP_011817956.1_472	CUST_2512_PI442190916	AS9606	HLII	WP_011817956.1_472	CTCTTACACCTGAATGGTTAACATCTTCTCCACCTCCAGTGGAAAATTGGGAAGGAGAAG	cyoB/coxA	Cytochrome c oxidase subunit I	WP_011817956.:	434649..436274	Respiration	COG0843
WP_011817956.1_472	CUST_2513_PI442190916	AS9606	HLII	WP_011817956.1_472	AATAATGGGGATAGTAGTTTTAGGTTTAGTTGTTTGGGCGCATCACATGTTTACCAGTGG	cyoB/coxA	Cytochrome c oxidase subunit I	WP_011817956.:	434649..436274	Respiration	COG0843
WP_011817956.1_472	CUST_2514_PI442190916	AS9606	HLII	WP_011817956.1_472	GGTTGCAACATTATGGGGAGGTAAAATTTCCATCAATAGTGCAATACTATTCTCTTGCGG	cyoB/coxA	Cytochrome c oxidase subunit I	WP_011817956.:	434649..436274	Respiration	COG0843
WP_011817957.1_473	CUST_2515_PI442190916	AS9606	HLII	WP_011817957.1_473	CGATAATTTGCGCAGAATTATGTGGCCCATATCATGGAGGGATGAGAGCCTCAATAATTG	ctaC/coxB	Cytochrome c oxidase subunit II	WP_011817957.:	436271..437074	Respiration	COG1622
WP_011817957.1_473	CUST_2516_PI442190916	AS9606	HLII	WP_011817957.1_473	AATGGAATCCAAAGACGTCATTCATGCTTTTTGGGTGCCAGAGTTCAGAATTAAACAGGA	ctaC/coxB	Cytochrome c oxidase subunit II	WP_011817957.:	436271..437074	Respiration	COG1622
WP_011817957.1_473	CUST_2517_PI442190916	AS9606	HLII	WP_011817957.1_473	TCCCAAGGGCAATTTCATATCAGGAGAACTACACGTTCCTGTTGATCAAAAAGTATCAAT	ctaC/coxB	Cytochrome c oxidase subunit II	WP_011817957.:	436271..437074	Respiration	COG1622
WP_011817957.1_473	CUST_2518_PI442190916	AS9606	HLII	WP_011817957.1_473	AGCTACAACATCTACGATCGAATGGGAGGGATGAAAGAACTAAATCATAACCACGATATG	ctaC/coxB	Cytochrome c oxidase subunit II	WP_011817957.:	436271..437074	Respiration	COG1622
WP_011817957.1_473	CUST_2519_PI442190916	AS9606	HLII	WP_011817957.1_473	TCTTTTTGGATTGGTTTTAATGTAAATTTGCTTCCAGCTGAAGCAAGTATTAATGCACCA	ctaC/coxB	Cytochrome c oxidase subunit II	WP_011817957.:	436271..437074	Respiration	COG1622
WP_011817957.1_473	CUST_2520_PI442190916	AS9606	HLII	WP_011817957.1_473	ATCAGATAGGAGATGGTATAGCTTTAGAGGGGAATTTAAGCTTAGAAATTGTATGGACAA	ctaC/coxB	Cytochrome c oxidase subunit II	WP_011817957.:	436271..437074	Respiration	COG1622
WP_011817965.1_481	CUST_2559_PI442190916	AS9606	HLII	WP_011817965.1_481	ATCCCGGTCAAGCAAATTATTCTGCAGCTAAAGCTGGAGTTATTGGATTCACCAAAACTT	gdh	3-oxoacyl-acyl-carrier-protein reductase	WP_011817965.:	443774..444523	Entner-Dudoroff	COG1028
WP_011817965.1_481	CUST_2560_PI442190916	AS9606	HLII	WP_011817965.1_481	AGCTAGCAGAGGAATTGGTAAAGAAATTGCTTTAGAACTAAGCCGCTTAGGAGCAGAAGT	gdh	3-oxoacyl-acyl-carrier-protein reductase	WP_011817965.:	443774..444523	Entner-Dudoroff	COG1028
WP_011817965.1_481	CUST_2561_PI442190916	AS9606	HLII	WP_011817965.1_481	TGCTGGTATTACTAGAGATGGACTGTTGATGAGAATGAAATCAGAACAATGGGATGATGT	gdh	3-oxoacyl-acyl-carrier-protein reductase	WP_011817965.:	443774..444523	Entner-Dudoroff	COG1028
WP_011817965.1_481	CUST_2562_PI442190916	AS9606	HLII	WP_011817965.1_481	TCAAGTAATGCTGGAAGTTACATTACAGGGCAAACAATAAGTATAGACGGAGGTATGAGT	gdh	3-oxoacyl-acyl-carrier-protein reductase	WP_011817965.:	443774..444523	Entner-Dudoroff	COG1028
WP_011817965.1_481	CUST_2563_PI442190916	AS9606	HLII	WP_011817965.1_481	AAGAGAGGATTCTGTAAGTTCAGCCTTTGAAGAAATCATAAAAATCAATGGCTCCATTGA	gdh	3-oxoacyl-acyl-carrier-protein reductase	WP_011817965.:	443774..444523	Entner-Dudoroff	COG1028
WP_011817965.1_481	CUST_2564_PI442190916	AS9606	HLII	WP_011817965.1_481	TGCTTCAAGAGGTATAAACGTAAATGCAATAGCTCCAGGCTTTATAGAAACAGAAATGAC	gdh	3-oxoacyl-acyl-carrier-protein reductase	WP_011817965.:	443774..444523	Entner-Dudoroff	COG1028
WP_011818027.1_547	CUST_2922_PI442190916	AS9606	HLII	WP_011818027.1_547	GTTGATGCGAGACTGAGCTTTGACACTGACGCTACGGTAGAAAAAGCAAGAAAATTGATC	tal	Transaldolase	WP_011818027.:	500786..501787	Pentose Phosphate	COG0176
WP_011818027.1_547	CUST_2923_PI442190916	AS9606	HLII	WP_011818027.1_547	AACGTGGGAGGGAATTAAGGCAGCTGAAATTTTGGAAAAAGAGGGTATTAAGTGCAACTT	tal	Transaldolase	WP_011818027.:	500786..501787	Pentose Phosphate	COG0176
WP_011818027.1_547	CUST_2924_PI442190916	AS9606	HLII	WP_011818027.1_547	ACTTCTGCCAAGCTGTAACTTGTGCCAATGCAAAGATAACTCTAATTTCTCCCTTCGTTG	tal	Transaldolase	WP_011818027.:	500786..501787	Pentose Phosphate	COG0176
WP_011818027.1_547	CUST_2925_PI442190916	AS9606	HLII	WP_011818027.1_547	TTAGAAGACCAAATGGCAAGTGAAAAGCTTAGTGAAGGAATCACTGGATTCAGTAAGGCT	tal	Transaldolase	WP_011818027.:	500786..501787	Pentose Phosphate	COG0176
WP_011818027.1_547	CUST_2926_PI442190916	AS9606	HLII	WP_011818027.1_547	AGATTGGCATAAAGCAAAAACTGGTAAAACTAGTTTTGTTGGTGCTGAAGATCCTGGTGT	tal	Transaldolase	WP_011818027.:	500786..501787	Pentose Phosphate	COG0176
WP_011818027.1_547	CUST_2927_PI442190916	AS9606	HLII	WP_011818027.1_547	AGAAGTAATGGGAGCGAGTTTTAGAAATCTCGATGAAATAAAAGAATTAGCAGGTTGCGA	tal	Transaldolase	WP_011818027.:	500786..501787	Pentose Phosphate	COG0176
WP_002805854.1_578	CUST_3099_PI442190916	AS9606	HLII	WP_002805854.1_578	TATTCACGTATGGCATATGCCTGCACTCCTAGCGATTTTTGGAGATGATTCTTGTCTTCA	rbcL	Ribulose 1,5-bisphosphate carboxylase large subunit	WP_002805854.:	529175..530590	Carbon fixation	COG1850
WP_002805854.1_578	CUST_3100_PI442190916	AS9606	HLII	WP_002805854.1_578	ATGCTGGTCGCGAAATCGAAAAAGAGAGTAGAGACATTCTTATGGAAGCTGCTAAACACA	rbcL	Ribulose 1,5-bisphosphate carboxylase large subunit	WP_002805854.:	529175..530590	Carbon fixation	COG1850
WP_011376108.1_579	CUST_3104_PI442190916	AS9606	HLII	WP_011376108.1_579	ACCCTGATCATCATGTAAGAATCATCGGATACGATGCTTACACTCAAAGCCAAGGAACAG	rbcS	Ribulose bisphosphate carboxylase small subunit	WP_011376108.:	530681..531022	Carbon fixation	COG4451
WP_011376108.1_579	CUST_3105_PI442190916	AS9606	HLII	WP_011376108.1_579	TGGAAGCTCCCATTCTTTGGGGAAAAAGACCTTAACTTGGTTGTAAGTGAATTAGAGGCA	rbcS	Ribulose bisphosphate carboxylase small subunit	WP_011376108.:	530681..531022	Carbon fixation	COG4451
WP_011376108.1_579	CUST_3106_PI442190916	AS9606	HLII	WP_011376108.1_579	AATTGCATACATAATTGCTCAAGGCTGGAGTCCTGTTATTGAGCATGTTCATCCAAGTGG	rbcS	Ribulose bisphosphate carboxylase small subunit	WP_011376108.:	530681..531022	Carbon fixation	COG4451
WP_011376108.1_579	CUST_3107_PI442190916	AS9606	HLII	WP_011376108.1_579	CAGAGCACAGTAGGCGACTATCAAACAGTTGCAACCCTGGAAACATTCGGTTTCTTAC	rbcS	Ribulose bisphosphate carboxylase small subunit	WP_011376108.:	530681..531022	Carbon fixation	COG4451
WP_011818100.1_624	CUST_3344_PI442190916	AS9606	HLII	WP_011818100.1_624	TTGGTTAGGTGCTTATGCCTCTTTATTGGGAGATGTTGCTCTTCAAGAATTGTGCTTTGG	glk	Glucokinase	WP_011818100.:	575153..576187	Glycolysis	COG0837
WP_011818100.1_624	CUST_3345_PI442190916	AS9606	HLII	WP_011818100.1_624	GGATTATTAGCGGCACTGGTTTATCAAGAATTGCCGAATGGAGACTAAGCAAACCTGATG	glk	Glucokinase	WP_011818100.:	575153..576187	Glycolysis	COG0837
WP_011818100.1_624	CUST_3346_PI442190916	AS9606	HLII	WP_011818100.1_624	TGGATCCAATTCTGAAAATACTAATAATGATTTGCATGCCATTGTTGGAGCGGGGACTGG	glk	Glucokinase	WP_011818100.:	575153..576187	Glycolysis	COG0837
WP_011818100.1_624	CUST_3347_PI442190916	AS9606	HLII	WP_011818100.1_624	CAGGGGAAAAGGTAAAAGTTTTAGCTAGTGAAGGTGGTCATGTAGAGTACTCCCCAAAGT	glk	Glucokinase	WP_011818100.:	575153..576187	Glycolysis	COG0837
WP_011818100.1_624	CUST_3348_PI442190916	AS9606	HLII	WP_011818100.1_624	AGATGAAGAGTTTGGACTTTTTAGTGCAGCCTGCAGAGCAAAAATGCTTTTAAAAACTTA	glk	Glucokinase	WP_011818100.:	575153..576187	Glycolysis	COG0837
WP_011818100.1_624	CUST_3349_PI442190916	AS9606	HLII	WP_011818100.1_624	TGCAGCGAGAAAAGAACTACCTGAAAAAATTTGTAATCTTTCTAAAGAAGGTGATCAGGT	glk	Glucokinase	WP_011818100.:	575153..576187	Glycolysis	COG0837
WP_011818274.1_785	CUST_4182_PI442190916	AS9606	HLII	WP_011818274.1_785	ATTGAGAGTGATTCGGTTCTTGAGGACACTCTTGTTATGGGTGCCGATTTCTTTGAATCT	glgC	Glucose-1-phosphate adenylyltransferase	WP_011818274.:	720461..721756	Glycogen Metabolism	COG0448
WP_011818274.1_785	CUST_4183_PI442190916	AS9606	HLII	WP_011818274.1_785	AGGCAATGGCAGTAGATACTTCAAAATTTGGATTAAGTAAGGAGTCAGCGGCCGAAAAAC	glgC	Glucose-1-phosphate adenylyltransferase	WP_011818274.:	720461..721756	Glycogen Metabolism	COG0448
WP_011818274.1_785	CUST_4184_PI442190916	AS9606	HLII	WP_011818274.1_785	GGGCATTCTTTGAGTCAAACCTTGCATTGACTGAGCAACCAAAACCTCCATTTAGTTTTT	glgC	Glucose-1-phosphate adenylyltransferase	WP_011818274.:	720461..721756	Glycogen Metabolism	COG0448
WP_011818274.1_785	CUST_4185_PI442190916	AS9606	HLII	WP_011818274.1_785	TGAATTAAGAAAAGGAGGCGGAACCCCTCTTGGAGTAGGTGAAGGAACTACTGTAAAAAG	glgC	Glucose-1-phosphate adenylyltransferase	WP_011818274.:	720461..721756	Glycogen Metabolism	COG0448

WP_011818274.1_785	CUST_4186_PI442190916	AS9606	HLII	WP_011818274.1_785	TCGAGTAGAGGAAGCAGATAAGCCAGAATTAGGCTTTTATATCAGAAATGGAATTGTCGT	glgC	Glucose-1-phosphate adenylyltransferase	WP_011818274.	720461..721756	Glycogen Metabolism	COG0448
WP_011818274.1_785	CUST_4187_PI442190916	AS9606	HLII	WP_011818274.1_785	TTTCTCCCCCCTTCTAAACTTGTTGATGCTCAAATTACTGATTCAATAGTTTGTGAAGGT	glgC	Glucose-1-phosphate adenylyltransferase	WP_011818274.	720461..721756	Glycogen Metabolism	COG0448
WP_011818275.1_786	CUST_4188_PI442190916	AS9606	HLII	WP_011818275.1_786	AGACTTCCTCAGAATCTTGTTCAGGCAATGAGAGACTGTTTTGGCTCCCATACATATGAA	edd	phosphogluconate dehydrogenase (NADP(+)-dependent, decarboxyla	WP_011818275.	721853..723271	Pentose Phosphate/Entner-Dudorof	COG2084
WP_011818275.1_786	CUST_4189_PI442190916	AS9606	HLII	WP_011818275.1_786	TAGCTAAGGTGGTTTCTCTATCCACAAAGGCAGGTATACCAGTCCCATGTTTATCCAGTA	edd	phosphogluconate dehydrogenase (NADP(+)-dependent, decarboxyla	WP_011818275.	721853..723271	Pentose Phosphate/Entner-Dudorof	COG2084
WP_011818275.1_786	CUST_4190_PI442190916	AS9606	HLII	WP_011818275.1_786	ATGAAACCTTTATTTGATGCTGTAGTCCTTGCCACAATTGCTAGCTATGCCCAAGGTATG	edd	phosphogluconate dehydrogenase (NADP(+)-dependent, decarboxyla	WP_011818275.	721853..723271	Pentose Phosphate/Entner-Dudorof	COG2084
WP_011818275.1_786	CUST_4191_PI442190916	AS9606	HLII	WP_011818275.1_786	TAGATAAAGCTGGCCAGAAAGGTACAGGTCTATGGACTGTTGTAAGTGCTCTAGAACTGG	edd	phosphogluconate dehydrogenase (NADP(+)-dependent, decarboxyla	WP_011818275.	721853..723271	Pentose Phosphate/Entner-Dudorof	COG2084
WP_011818275.1_786	CUST_4192_PI442190916	AS9606	HLII	WP_011818275.1_786	TGCAGAAGCTTATGACCTTATGAAGAGAGTCAAAGGTATGAATGGACAGCAGATGTCAGA	edd	phosphogluconate dehydrogenase (NADP(+)-dependent, decarboxyla	WP_011818275.	721853..723271	Pentose Phosphate/Entner-Dudorof	COG2084
WP_011818275.1_786	CUST_4193_PI442190916	AS9606	HLII	WP_011818275.1_786	TTGGCCCAGGAGGCTCAGGTCATTTTGTAAAAACTGTTCATAACGGAATTGAATATGGAA	edd	phosphogluconate dehydrogenase (NADP(+)-dependent, decarboxyla	WP_011818275.	721853..723271	Pentose Phosphate/Entner-Dudorof	COG2084
WP_011818286.1_797	CUST_4250_PI442190916	AS9606	HLII	WP_011818286.1_797	AGGTGAAGCGGAAGATGGTCATGGATTTGAAGGTGAACTTTCTACAGATATGCTTCTGAC	cbbA	Fructose-1,6-bisphosphate aldolase class II	WP_011818286.	730539..731612	Glycolysis/Gluconeogenesis	COG0191
WP_011818286.1_797	CUST_4251_PI442190916	AS9606	HLII	WP_011818286.1_797	GGAGTTAGGAAAGTCAACATTGATACTGATAACAGACTTGCTTTCACTGCCGCGGTTAGA	cbbA	Fructose-1,6-bisphosphate aldolase class II	WP_011818286.	730539..731612	Glycolysis/Gluconeogenesis	COG0191
WP_011818286.1_797	CUST_4252_PI442190916	AS9606	HLII	WP_011818286.1_797	AGCATTTGCTGATAAGGCAAACTTCGACCCAAGACATTTCAACAAGCCTGCTAGAAAATA	cbbA	Fructose-1,6-bisphosphate aldolase class II	WP_011818286.	730539..731612	Glycolysis/Gluconeogenesis	COG0191
WP_011818286.1_797	CUST_4255_PI442190916	AS9606	HLII	WP_011818286.1_797	AATTTGGCGGTGAAATTCCTCAAACATATGGTGTTCCTGTTGAAGAAATTCAAGAGGGAA	cbbA	Fructose-1,6-bisphosphate aldolase class II	WP_011818286.	730539..731612	Glycolysis/Gluconeogenesis	COG0191
WP_011818287.1_798	CUST_4256_PI442190916	AS9606	HLII	WP_011818287.1_798	AAGGAAGCGATGTTGAAGGAGGTCAAAAAGCATTAATAGCAAGAGCTCAAGCAAACTCTG	fda	Fructose-1,6-bisphosphate aldolase class I	WP_011818287.	731760..732827	Glycolysis/Gluconeogenesis	COG0191
WP_011818287.1_798	CUST_4257_PI442190916	AS9606	HLII	WP_011818287.1_798	GAGAATGCTTGGGGATTAGCAAGGTATGCAAGATCAGTTCAAGAATCTGGGTTAGTACCA	fda	Fructose-1,6-bisphosphate aldolase class I	WP_011818287.	731760..732827	Glycolysis/Gluconeogenesis	COG0191
WP_011818287.1_798	CUST_4258_PI442190916	AS9606	HLII	WP_011818287.1_798	GAAGGTTGCTGAAATGACAATTAGAACTATGGAAAGATGCGTTCCTGCATCTGTTCCTGG	fda	Fructose-1,6-bisphosphate aldolase class I	WP_011818287.	731760..732827	Glycolysis/Gluconeogenesis	COG0191
WP_011818287.1_798	CUST_4259_PI442190916	AS9606	HLII	WP_011818287.1_798	CCAGGTATAAAGGTCGATAAAGGCCTAAATCCACTTCCAGGAGGAGGAGATGTAGAAACT	fda	Fructose-1,6-bisphosphate aldolase class I	WP_011818287.	731760..732827	Glycolysis/Gluconeogenesis	COG0191
WP_011818287.1_798	CUST_4260_PI442190916	AS9606	HLII	WP_011818287.1_798	GCTTCAAAAGGTGCCTATGTAGCAGGATCTCAACCTTCATCTGATGAGCAATTGTTTGTG	fda	Fructose-1,6-bisphosphate aldolase class I	WP_011818287.	731760..732827	Glycolysis/Gluconeogenesis	COG0191
WP_011818287.1_798	CUST_4261_PI442190916	AS9606	HLII	WP_011818287.1_798	TTAGTTGAAAGAGCCGCAAAATATTATGAACAAGGTGCCAGATTTGCAAAGTGGAGAGCA	fda	Fructose-1,6-bisphosphate aldolase class I	WP_011818287.	731760..732827	Glycolysis/Gluconeogenesis	COG0191
WP_011818291.1_802	CUST_4279_PI442190916	AS9606	HLII	WP_011818291.1_802	TTTTTTATGAAGGTTTGCATGGCGGGGTAGAAGGTGATGGATACAATGTTGCCTCTTATG	prk	Phosphoribulokinase	WP_011818291.	735446..736342	Carbon Fixation	COG3954
WP_011818291.1_802	CUST_4280_PI442190916	AS9606	HLII	WP_011818291.1_802	TCCCAACTCCTGATGAGAGCTTTGTGATCATCATTTCAGAAAAGGGGCAAGAGAGAAAT	prk	Phosphoribulokinase	WP_011818291.	735446..736342	Carbon Fixation	COG3954
WP_011818291.1_802	CUST_4281_PI442190916	AS9606	HLII	WP_011818291.1_802	CAGAAGAACATAATACAAGACTTGGGACATCCCTAGAACCTGGGCAATTTACTCCATGGG	prk	Phosphoribulokinase	WP_011818291.	735446..736342	Carbon Fixation	COG3954
WP_011818291.1_802	CUST_4282_PI442190916	AS9606	HLII	WP_011818291.1_802	AAAGCTATGGCAGAAGCTCTTTCAAAAGGTGAAAATTTCTCTCATTTTGGTCCAGAGGCT	prk	Phosphoribulokinase	WP_011818291.	735446..736342	Carbon Fixation	COG3954
WP_011818291.1_802	CUST_4283_PI442190916	AS9606	HLII	WP_011818291.1_802	AACGCAGAAAGAGGTTACTCAGCGGAAACCATTGTGGATACAATATTGAGAAGAATGCCT	prk	Phosphoribulokinase	WP_011818291.	735446..736342	Carbon Fixation	COG3954
WP_011818291.1_802	CUST_4284_PI442190916	AS9606	HLII	WP_011818291.1_802	TGTCAAGTCCAACAAGTATCGTTGTAAATGGAGGAAAAATGGGATTCGCAATGGAACTAA	prk	Phosphoribulokinase	WP_011818291.	735446..736342	Carbon Fixation	COG3954
WP_011818370.1_878	CUST_4686_PI442190916	AS9606	HLII	WP_011818370.1_878	CAACTTGGTGCTGAGCATAGTCAAAGACTTGAAGCATATTTTCATGCAGTTCCTGGCATA	pdhB	Pyruvate dehydrogenase E1 component beta subunit	WP_011818370.	799701..800684	Glycolysis/Krebs cycle	COG0022
WP_011818370.1_878	CUST_4687_PI442190916	AS9606	HLII	WP_011818370.1_878	GTGGGTGCAGCAATGACTGGCTTAAGACCAATAGTAGAAGGAATGAATATGGGGTTTTTG	pdhB	Pyruvate dehydrogenase E1 component beta subunit	WP_011818370.	799701..800684	Glycolysis/Krebs cycle	COG0022
WP_011818370.1_878	CUST_4688_PI442190916	AS9606	HLII	WP_011818370.1_878	GGTGCAGAATTAATTGCCTTGATAACAGAAGAGTGTTTTGATGATCTTGATGCCCGACCA	pdhB	Pyruvate dehydrogenase E1 component beta subunit	WP_011818370.	799701..800684	Glycolysis/Krebs cycle	COG0022
WP_011818370.1_878	CUST_4689_PI442190916	AS9606	HLII	WP_011818370.1_878	AGGGTGATTATACTTGCGCTTTAGATCAGGCTGACGTTGTAAAAGAAGGGCGTGATATTA	pdhB	Pyruvate dehydrogenase E1 component beta subunit	WP_011818370.	799701..800684	Glycolysis/Krebs cycle	COG0022
WP_011818370.1_878	CUST_4690_PI442190916	AS9606	HLII	WP_011818370.1_878	ATATACAAGTGGCGGAAATTATAAAATACCAGCAGTAGTTCGAGGACCTGGTGGAGTTGG	pdhB	Pyruvate dehydrogenase E1 component beta subunit	WP_011818370.	799701..800684	Glycolysis/Krebs cycle	COG0022
WP_011818370.1_878	CUST_4691_PI442190916	AS9606	HLII	WP_011818370.1_878	TGTTGCATGTAGTACACCCACAAATGCTAAAGGTTTAATGAAAGCAGCTATAAGAGATGA	pdhB	Pyruvate dehydrogenase E1 component beta subunit	WP_011818370.	799701..800684	Glycolysis/Krebs cycle	COG0022
WP_011818380.1_889	CUST_4743_PI442190916	AS9606	HLII	WP_011818380.1_889	CGCACTTGCAAATCCTTACTTAGCATTCTCTGTAATGATGCTTGCCGGTATTGATGGAAT	glnA	Glutamine synthetase/ glutamate--ammonialigase	WP_011818380.	808459..809880	Nitrogen Metabolism	COG0174
WP_011818380.1_889	CUST_4744_PI442190916	AS9606	HLII	WP_011818380.1_889	CCTGTTTTTAACGACAACGGAACAGGAATGCATGTTCACCAAAGTTTATGGAAGAGTGGA	glnA	Glutamine synthetase/ glutamate--ammonialigase	WP_011818380.	808459..809880	Nitrogen Metabolism	COG0174
WP_011818380.1_889	CUST_4745_PI442190916	AS9606	HLII	WP_011818380.1_889	AGATGGTACATCGGAGGCATACTTAAACATGCGCCATCATTCTTAGCATTTACTAACCCA	glnA	Glutamine synthetase/ glutamate--ammonialigase	WP_011818380.	808459..809880	Nitrogen Metabolism	COG0174
WP_011818380.1_889	CUST_4746_PI442190916	AS9606	HLII	WP_011818380.1_889	GAGGGTAATAGATCAGCTGCTGTAAGAATACCTTTAACAGGACCAAGCCCCAAAGCTAAA	glnA	Glutamine synthetase/ glutamate--ammonialigase	WP_011818380.	808459..809880	Nitrogen Metabolism	COG0174
WP_011818380.1_889	CUST_4747_PI442190916	AS9606	HLII	WP_011818380.1_889	GTGCCGGCCAACACGAGCTTGGAATGAAATTTGATTCGTTAATAAATGCTGCTGATAAC	glnA	Glutamine synthetase/ glutamate--ammonialigase	WP_011818380.	808459..809880	Nitrogen Metabolism	COG0174
WP_011818380.1_889	CUST_4748_PI442190916	AS9606	HLII	WP_011818380.1_889	AATTGATACAGTGCCTTCATCTCTTAATGATTCACTTAATGCGCTAAAAGCAGACAAGGA	glnA	Glutamine synthetase/ glutamate--ammonialigase	WP_011818380.	808459..809880	Nitrogen Metabolism	COG0174
WP_011818389.1_897	CUST_4789_PI442190916	AS9606	HLII	WP_011818389.1_897	TGGCGATCTTATTACCCTCCAGCTTAATGAAGGTGTTATATACATGGGACAAATTGAAGA	pykF	Pyruvate kinase	WP_011818389.	817097..818887	Glycolysis	COG0469
WP_011818389.1_897	CUST_4790_PI442190916	AS9606	HLII	WP_011818389.1_897	GCAAATTGCTCAAGAGATGGGCATCCTAAATCAAGGAGATTTAGTAGTTCAAACCGCAGG	pykF	Pyruvate kinase	WP_011818389.	817097..818887	Glycolysis	COG0469
WP_011818389.1_897	CUST_4791_PI442190916	AS9606	HLII	WP_011818389.1_897	CCTATCTTGGCAACTACTACAGAAAGAAGTGTAGCGAGAAGATTGCAACTTGTTTGGGGA	pykF	Pyruvate kinase	WP_011818389.	817097..818887	Glycolysis	COG0469
WP_011818389.1_897	CUST_4792_PI442190916	AS9606	HLII	WP_011818389.1_897	ATCCCTTTAACTAAATCAGGTTCTACCGCTCGAAATGTAAGTAAGTTCAGACCTCCAACA	pykF	Pyruvate kinase	WP_011818389.	817097..818887	Glycolysis	COG0469
WP_011818389.1_897	CUST_4793_PI442190916	AS9606	HLII	WP_011818389.1_897	GCCACTTACCCAGTACGATCCCAAATGCTATTAGTGCAGCAGTAAGCAATATAGCTAGAC	pykF	Pyruvate kinase	WP_011818389.	817097..818887	Glycolysis	COG0469
WP_011818389.1_897	CUST_4794_PI442190916	AS9606	HLII	WP_011818389.1_897	GCAGTTGAAACGATGGCAACCATAGCAAGAAGAATTGAAAGGGATTATCCACTTAAGGCT	pykF	Pyruvate kinase	WP_011818389.	817097..818887	Glycolysis	COG0469
WP_011818410.1_919	CUST_4904_PI442190916	AS9606	HLII	WP_011818410.1_919	ATCCAGCAGCACTATTATCATCCGCATGGTATTTAACTGGGGATGGTATTGGAAAGAGAG	pgi	Glucose-6-phosphate isomerase	WP_011818410.	837779..839362	Glycolysis/Gluconeogenesis	COG0166
WP_011818410.1_919	CUST_4905_PI442190916	AS9606	HLII	WP_011818410.1_919	TTCACTAGGTGCCTTAATCGCTTTATTTGAAAGGGCTGTATCCTTCTACGCTGAATTGGT	pgi	Glucose-6-phosphate isomerase	WP_011818410.	837779..839362	Glycolysis/Gluconeogenesis	COG0166
WP_011818410.1_919	CUST_4906_PI442190916	AS9606	HLII	WP_011818410.1_919	ATGCATATGATCAACCTGGAGTTGAAGCTGGAAAGAAAGCAGCTGCAAATATTATTGAGT	pgi	Glucose-6-phosphate isomerase	WP_011818410.	837779..839362	Glycolysis/Gluconeogenesis	COG0166
WP_011818410.1_919	CUST_4907_PI442190916	AS9606	HLII	WP_011818410.1_919	TTCCGTTTTTGGTAATAAAGGATCTACAGATCAGCATGCCTATGTTCAGCAACTGAGAGA	pgi	Glucose-6-phosphate isomerase	WP_011818410.	837779..839362	Glycolysis/Gluconeogenesis	COG0166
WP_011818410.1_919	CUST_4908_PI442190916	AS9606	HLII	WP_011818410.1_919	ACTCCGTGAAATGTGTTTCGGTAATGATAATTATTTAGTTAAGGGCGATTGGTCAAATCC	pgi	Glucose-6-phosphate isomerase	WP_011818410.	837779..839362	Glycolysis/Gluconeogenesis	COG0166
WP_011818410.1_919	CUST_4909_PI442190916	AS9606	HLII	WP_011818410.1_919	AGGAACCAGATCAGCACTCTCTAGTGAAAACAGACAAAGTATCACTATTACGTTAGAAAA	pgi	Glucose-6-phosphate isomerase	WP_011818410.	837779..839362	Glycolysis/Gluconeogenesis	COG0166
WP_011818582.1_1078	CUST_5667_PI442190916	AS9606	HLII	WP_011818582.1_1078	AACCGAGGGAGAGAAGGGAGGCATAATATTCCCAGGAGGAAGATATAAGGCTCTTAAAAA	rbsK	Sugar kinase, ribokinase family	WP_011818582.	964467..965336	Pentose Phosphate	COG0524
WP_011818582.1_1078	CUST_5668_PI442190916	AS9606	HLII	WP_011818582.1_1078	CAGGCGTCATTTCTCATTCTGAAAAATCTCTTGAGTATCCAGCTGGTGGTGGCTCTATTA	rbsK	Sugar kinase, ribokinase family	WP_011818582.	964467..965336	Pentose Phosphate	COG0524
WP_011818582.1_1078	CUST_5669_PI442190916	AS9606	HLII	WP_011818582.1_1078	TCTTATGGATGTGGCGATTCATTTGCTGCTGGGATTCTTTATGGAATGGCATCTAAATGG	rbsK	Sugar kinase, ribokinase family	WP_011818582.	964467..965336	Pentose Phosphate	COG0524
WP_011818582.1_1078	CUST_5670_PI442190916	AS9606	HLII	WP_011818582.1_1078	TGCTAAAGTAATGGGAAGAGACGCAAGTGAATTTTTCGGCCCATATGCAAATAGTGATGA	rbsK	Sugar kinase, ribokinase family	WP_011818582.	964467..965336	Pentose Phosphate	COG0524
WP_011818582.1_1078	CUST_5671_PI442190916	AS9606	HLII	WP_011818582.1_1078	CTCTCAAATATGGGAATTAAGATGCATGTAGCTTGGCGTGATAAACCAACTAGAAGAGGA	rbsK	Sugar kinase, ribokinase family	WP_011818582.	964467..965336	Pentose Phosphate	COG0524
WP_011818582.1_1078	CUST_5672_PI442190916	AS9606	HLII	WP_011818582.1_1078	AATTGACTCTCTAGGTGAAAGAGCAATAACAGTTATTGGTGAAAGGTTAGCTCCAACTCA	rbsK	Sugar kinase, ribokinase family	WP_011818582.	964467..965336	Pentose Phosphate	COG0524
WP_011818610.1_1107	CUST_5832_PI442190916	AS9606	HLII	WP_011818610.1_1107	TAACACTTGTGTAATACTTGGCTGTGAATCAGTGGAATGTATGAGACTTGAAGCAGGGGG	opcA	Glucose-6-P dehydrogenase subunit opcA	WP_011818610.	992976..994277	Pentose Phosphate	COG3429
WP_011818610.1_1107	CUST_5833_PI442190916	AS9606	HLII	WP_011818610.1_1107	TAGATTGAAAAATTGGAGGGAATCATTGGCAATGATTTTTGATCCGCCTTCGAGGAGACC	opcA	Glucose-6-P dehydrogenase subunit opcA	WP_011818610.	992976..994277	Pentose Phosphate	COG3429
WP_011818610.1_1107	CUST_5834_PI442190916	AS9606	HLII	WP_011818610.1_1107	TACCTTCATGGTTGTGGTGGAATGGAAGCTTGGATGAATCGCCTGAAATTTTCGAATATT	opcA	Glucose-6-P dehydrogenase subunit opcA	WP_011818610.	992976..994277	Pentose Phosphate	COG3429
WP_011818610.1_1107	CUST_5835_PI442190916	AS9606	HLII	WP_011818610.1_1107	TCCCTTATCTTTGGGAAATCCAAGTATTCATTTAGGACAAGTCATTGGATTGAGGCTGAT	opcA	Glucose-6-P dehydrogenase subunit opcA	WP_011818610.	992976..994277	Pentose Phosphate	COG3429
WP_011818610.1_1107	CUST_5836_PI442190916	AS9606	HLII	WP_011818610.1_1107	CGCAGGAGATCATATGATTCAAGCGTTGTTTTTGATTTCATGGATTAGCGATAAACTTGG	opcA	Glucose-6-P dehydrogenase subunit opcA	WP_011818610.	992976..994277	Pentose Phosphate	COG3429
WP_011818610.1_1107	CUST_5837_PI442190916	AS9606	HLII	WP_011818610.1_1107	ACTGATTATGGTCTAAGGTTAATAATTGATACTGCTCTTGGATCGCCTCAAAGATGTTTA	opcA	Glucose-6-P dehydrogenase subunit opcA	WP_011818610.	992976..994277	Pentose Phosphate	COG3429
WP_011818611.1_1108	CUST_5838_PI442190916	AS9606	HLII	WP_011818611.1_1108	CCCGTAGTGATGAGGTAGAGGCAGCCTGGAAACTTTATACGCCATTAATAGAATTGATGG	zwf	glucose-6-phosphate dehydrogenase zwf	WP_011818611.	994280..995803	Pentose Phosphate	COG0364
WP_011818611.1_1108	CUST_5839_PI442190916	AS9606	HLII	WP_011818611.1_1108	TCAAGCTTCCAAACTTGCTGACGAAAATCAACCGTGGAATTGTTGCATAAGAGGACAATA	zwf	glucose-6-phosphate dehydrogenase zwf	WP_011818611.	994280..995803	Pentose Phosphate	COG0364
WP_011818611.1_1108	CUST_5840_PI442190916	AS9606	HLII	WP_011818611.1_1108	CGTTGATAACTGGCGTTGGCAAGGAGTGCCTTTTTATTTGAGAACTGGTAAAAGACTACC	zwf	glucose-6-phosphate dehydrogenase zwf	WP_011818611.	994280..995803	Pentose Phosphate	COG0364
WP_011818611.1_1108	CUST_5841_PI442190916	AS9606	HLII	WP_011818611.1_1108	CGGGATATGCTTCAAAATCATATGACTCAAATGCTTGCAGTTACTGCAATGGAACCTCCT	zwf	glucose-6-phosphate dehydrogenase zwf	WP_011818611.	994280..995803	Pentose Phosphate	COG0364
WP_011818611.1_1108	CUST_5842_PI442190916	AS9606	HLII	WP_011818611.1_1108	TGGAGAGGGAGGGAATATTTCAAATCGACTCAAAGGATATAGGCAGGAAGATGGTGTTAA	zwf	glucose-6-phosphate dehydrogenase zwf	WP_011818611.	994280..995803	Pentose Phosphate	COG0364
WP_011818611.1_1108	CUST_5843_PI442190916	AS9606	HLII	WP_011818611.1_1108	GGAGAACCATCAGATGAAGGCTATGTAAGATTATTAGCTGATGCAATGCTTTCTGACCCA	zwf	glucose-6-phosphate dehydrogenase zwf	WP_011818611.	994280..995803	Pentose Phosphate	COG0364
WP_011818811.1_1297	CUST_6751_PI442190916	AS9606	HLII	WP_011818811.1_1297	GCGAGGCTACAGGTGCAGAAGTTAATGAAGTGTCCAATGCAATAGGATTTGATAGTAGGA	ugd	UDP-glucose 6-dehydrogenase/nucleotide sugar dehydrogenase	WP_011818811.	1157399..1158823	Amino sugar and nucleotide sugar r	COG1004
WP_011818811.1_1297	CUST_6752_PI442190916	AS9606	HLII	WP_011818811.1_1297	TGGAAGTGGACAAGGTAAGTGGAGATATCATAAAGATATATATGAGGCAGCCATGGGGGT	ugd	UDP-glucose 6-dehydrogenase/nucleotide sugar dehydrogenase	WP_011818811.	1157399..1158823	Amino sugar and nucleotide sugar r	COG1004
WP_011818811.1_1297	CUST_6753_PI442190916	AS9606	HLII	WP_011818811.1_1297	ACACAAGGGAATCTCCCGCTATCAAAATAGTTAAAGACCTTCTCGAAAATGGAGCCGAAA	ugd	UDP-glucose 6-dehydrogenase/nucleotide sugar dehydrogenase	WP_011818811.	1157399..1158823	Amino sugar and nucleotide sugar r	COG1004
WP_011818811.1_1297	CUST_6754_PI442190916	AS9606	HLII	WP_011818811.1_1297	TTTTGGCCTGAATGAAGTTGCAGAATACTGGGAACAGGTAGTTAAGTTTAATGAATGGCA	ugd	UDP-glucose 6-dehydrogenase/nucleotide sugar dehydrogenase	WP_011818811.	1157399..1158823	Amino sugar and nucleotide sugar r	COG1004
WP_011818811.1_1297	CUST_6755_PI442190916	AS9606	HLII	WP_011818811.1_1297	CTCAGACCCACAAGTTGACCCTGATATTATTGCACTAGATCTTGGAATTGAGGAGAATGT	ugd	UDP-glucose 6-dehydrogenase/nucleotide sugar dehydrogenase	WP_011818811.	1157399..1158823	Amino sugar and nucleotide sugar r	COG1004
WP_011818811.1_1297	CUST_6756_PI442190916	AS9606	HLII	WP_011818811.1_1297	TGGAGTAGTGAATTATCTAAGCTTGCTGCCAATGCTTTTTAGCTCAAAGAATAAGCTCA	ugd	UDP-glucose 6-dehydrogenase/nucleotide sugar dehydrogenase	WP_011818811.	1157399..1158823	Amino sugar and nucleotide sugar r	COG1004
WP_011818848.1_1335	CUST_6978_PI442190916	AS9606	HLII	WP_011818848.1_1335	AGAGAGATTGAAAAACTTCCTATTTACGGATCCGATTGGCCTACTAAAGATGGGACATGT	galE	UDP-glucose 4-epimerase	WP_011818848.	1199934..1201001	Galactose metabolism	COG1087
WP_011818848.1_1335	CUST_6979_PI442190916	AS9606	HLII	WP_011818848.1_1335	AGTGGGAACCTAAGAGAAACCTAAAAGATATTTGCAAAGACTCATGGCGTTGGTTTATCA	galE	UDP-glucose 4-epimerase	WP_011818848.	1199934..1201001	Galactose metabolism	COG1087
WP_011818848.1_1335	CUST_6980_PI442190916	AS9606	HLII	WP_011818848.1_1335	AAGCGACGATAAAAGATGGAAAATAGCTAACTTGAGGTATTTCAATCCTTGTGGAGCTCA	galE	UDP-glucose 4-epimerase	WP_011818848.	1199934..1201001	Galactose metabolism	COG1087
WP_011818848.1_1335	CUST_6981_PI442190916	AS9606	HLII	WP_011818848.1_1335	TGAGCCGACTTACCTTAATCTCAATATTGGAACGGGTACAGGTATAAGTGTACTAGAACT	galE	UDP-glucose 4-epimerase	WP_011818848.	1199934..1201001	Galactose metabolism	COG1087
WP_011818848.1_1335	CUST_6982_PI442190916	AS9606	HLII	WP_011818848.1_1335	TGTCAAATTCCATATTACTTTACTGAAAAAAGAAAAGGTGATGCTGCTTTCGTTGTTGCG	galE	UDP-glucose 4-epimerase	WP_011818848.	1199934..1201001	Galactose metabolism	COG1087
WP_011818848.1_1335	CUST_6983_PI442190916	AS9606	HLII	WP_011818848.1_1335	TCAGAAAATGGAATCCTTTCACCTCTTAATCCATATGGAAATACAAAATTAAGTAACGAA	galE	UDP-glucose 4-epimerase	WP_011818848.	1199934..1201001	Galactose metabolism	COG1087
WP_011818906.1_1391	CUST_7301_PI442190916	AS9606	HLII	WP_011818906.1_1391	AGAGAGCATCACCTACTAGGAGGATATGCATTTATTGGAGAGGGAATTCCTGTTGCCTTA	pdhA	Pyruvate dehydrogenase E1 component alpha subunit	WP_011818906.	1268563..1269636	Glycolysis/Krebs cycle	COG1071
WP_011818906.1_1391	CUST_7302_PI442190916	AS9606	HLII	WP_011818906.1_1391	TCATGTTCATGCACTAAGTGCGGGTGTTCCCTCATTTGAAGTAATGAGTGAACTTTTTGG	pdhA	Pyruvate dehydrogenase E1 component alpha subunit	WP_011818906.	1268563..1269636	Glycolysis/Krebs cycle	COG1071
WP_011818906.1_1391	CUST_7303_PI442190916	AS9606	HLII	WP_011818906.1_1391	ATGGCTCATGATAGAGCTACTAGTAATCCTGAAATCTGGAGAAAAGCGTCTGCTTTTGGC	pdhA	Pyruvate dehydrogenase E1 component alpha subunit	WP_011818906.	1268563..1269636	Glycolysis/Krebs cycle	COG1071
WP_011818906.1_1391	CUST_7304_PI442190916	AS9606	HLII	WP_011818906.1_1391	AAGGAAGTTGCTGGAAATAGTAATAGTGATGCTGTAACTGCAGCATTTTTTGGGGATGGG	pdhA	Pyruvate dehydrogenase E1 component alpha subunit	WP_011818906.	1268563..1269636	Glycolysis/Krebs cycle	COG1071
WP_011818906.1_1391	CUST_7305_PI442190916	AS9606	HLII	WP_011818906.1_1391	AGCAATTGAGCGAGCTAGGGCAGGAGAAGGTCCCACACTTTTAGAATGTTTAACTTATAG	pdhA	Pyruvate dehydrogenase E1 component alpha subunit	WP_011818906.	1268563..1269636	Glycolysis/Krebs cycle	COG1071
WP_011818906.1_1391	CUST_7306_PI442190916	AS9606	HLII	WP_011818906.1_1391	TTGATGCTGAAATAGCGGAGTCAGTTAAAAATGCAATTGAAGCTCCTGAACCTCCTTCAG	pdhA	Pyruvate dehydrogenase E1 component alpha subunit	WP_011818906.	1268563..1269636	Glycolysis/Krebs cycle	COG1071
WP_011818927.1_1412	CUST_7424_PI442190916	AS9606	HLII	WP_011818927.1_1412	AGTTAATGTTGATTTTGCAGACGTAAGATCAGTTATGACAGAAGCTGGCACTGCTCTCCT	ftsZ	cell division protein FtsZ	WP_011818927.	1291754..1292869	Cell Division	COG0206
WP_011818927.1_1412	CUST_7425_PI442190916	AS9606	HLII	WP_011818927.1_1412	CTATAGAGGCGGCACAGGCTGCAATGAATAGTCCTTTACTAGAAGCTGCTAGAATTGATG	ftsZ	cell division protein FtsZ	WP_011818927.	1291754..1292869	Cell Division	COG0206
WP_011818927.1_1412	CUST_7426_PI442190916	AS9606	HLII	WP_011818927.1_1412	TGTTGATCAAGAAGCAAATATTATTGTTGGTGCAGTCGTTGATGAAGCAATGGAAGGGGA	ftsZ	cell division protein FtsZ	WP_011818927.	1291754..1292869	Cell Division	COG0206
WP_011818927.1_1412	CUST_7427_PI442190916	AS9606	HLII	WP_011818927.1_1412	CCCGTTGTCGCAGAAGTAGCAAAACAAAGTGGTGCTCTAACTGTAGGTATAGTAACCAAA	ftsZ	cell division protein FtsZ	WP_011818927.	1291754..1292869	Cell Division	COG0206
WP_011818927.1_1412	CUST_7428_PI442190916	AS9606	HLII	WP_011818927.1_1412	TTCAAGAAGCATTTAGGAATGCTGATGATGTTCTAAGAATGGGCGTCAAAGGCATAAGCG	ftsZ	cell division protein FtsZ	WP_011818927.	1291754..1292869	Cell Division	COG0206
WP_011818927.1_1412	CUST_7429_PI442190916	AS9606	HLII	WP_011818927.1_1412	AGGAGGGGATCGCAAGACTAGCTGAAAACGTTGATACTCTTATTGTTATTCCAAACGACC	ftsZ	cell division protein FtsZ	WP_011818927.	1291754..1292869	Cell Division	COG0206
WP_011818939.1_1428	CUST_7502_PI442190916	AS9606	HLII	WP_011818939.1_1428	GTCACTACTTCCTGATGCAATAGACGAAGACCCAGAAAAACCAGCAGGATTATATACCGC	glcH	GPH family sugar transporter	WP_011818939.	1304362..1305708	GlcH sugar transporter	COG2211
WP_011818939.1_1428	CUST_7503_PI442190916	AS9606	HLII	WP_011818939.1_1428	AATGTTCCAACATACATTGCGAAGTGGATCCCGATACCTTTCCAAATTAGCGCTTTAGTG	glcH	GPH family sugar transporter	WP_011818939.	1304362..1305708	GlcH sugar transporter	COG2211
WP_011818939.1_1428	CUST_7504_PI442190916	AS9606	HLII	WP_011818939.1_1428	CCATTCGCGAAAAAAGCAAGAAGGCCTTCAGGGAAAGTTGAAGCAATAACACTTCAATTC	glcH	GPH family sugar transporter	WP_011818939.	1304362..1305708	GlcH sugar transporter	COG2211
WP_011818939.1_1428	CUST_7505_PI442190916	AS9606	HLII	WP_011818939.1_1428	TCTTCTCTGGTGCGCCTTACAATTGATGCAAACAGTAGCGTTAATCTATGTAGAGGATGT	glcH	GPH family sugar transporter	WP_011818939.	1304362..1305708	GlcH sugar transporter	COG2211
WP_011818939.1_1428	CUST_7506_PI442190916	AS9606	HLII	WP_011818939.1_1428	TTTAATTTTCATAATCTGTCTTATTGGCATTGGGGCTTCAACCGCTTTTCTTATCCCTTG	glcH	GPH family sugar transporter	WP_011818939.	1304362..1305708	GlcH sugar transporter	COG2211
WP_011818939.1_1428	CUST_7507_PI442190916	AS9606	HLII	WP_011818939.1_1428	ACAGAATTTCAGCTTTAAACTATGGAGCGATTTTATGGATTATTTCATGTACGGCAGCTT	glcH	GPH family sugar transporter	WP_011818939.	1304362..1305708	GlcH sugar transporter	COG2211
WP_011818958.1_1447	CUST_7615_PI442190916	AS9606	HLII	WP_011818958.1_1447	ACCAAAAAGGATGGCAATTGATTCTCTCTCTGCATTAGCCAGAGGTGTAAGTTTGAATGC	kaiC	circadian clock protein KaiC	WP_011818958.	1323503..1325032	Circadian Rhythm	COG0467
WP_011818958.1_1447	CUST_7616_PI442190916	AS9606	HLII	WP_011818958.1_1447	ATATGTGTGGTGGAGGATATTTTCAGGATTCCATCATTCTCGCTACTGGAGCTACTGGTA	kaiC	circadian clock protein KaiC	WP_011818958.	1323503..1325032	Circadian Rhythm	COG0467
WP_011818958.1_1447	CUST_7617_PI442190916	AS9606	HLII	WP_011818958.1_1447	GGTGAGATGGCAAGAGCTCTAAATGTTTTTAAAATGCGGGGATCATGGCATGATAAACGA	kaiC	circadian clock protein KaiC	WP_011818958.	1323503..1325032	Circadian Rhythm	COG0467
WP_011818958.1_1447	CUST_7618_PI442190916	AS9606	HLII	WP_011818958.1_1447	ACTTTTTGCATATGAAGAATCTAGGGCTCAATTGCTTAGGAATGCAACTAGCTGGGGAAT	kaiC	circadian clock protein KaiC	WP_011818958.	1323503..1325032	Circadian Rhythm	COG0467
WP_011818958.1_1447	CUST_7619_PI442190916	AS9606	HLII	WP_011818958.1_1447	TTTAGCGGCGCCCCTCACAGAATTGTACCTGATCAAAATGTTCAAAATGTTTTTAAAGGA	kaiC	circadian clock protein KaiC	WP_011818958.	1323503..1325032	Circadian Rhythm	COG0467
WP_011818958.1_1447	CUST_7620_PI442190916	AS9606	HLII	WP_011818958.1_1447	TGCTGTTACTGGTTATACAAAACAAGAGGAGATAGCAGGCTTCTTTACTAATACTGCAGA	kaiC	circadian clock protein KaiC	WP_011818958.	1323503..1325032	Circadian Rhythm	COG0467
WP_011818959.1_1448	CUST_7621_PI442190916	AS9606	HLII	WP_011818959.1_1448	TGGTTTAGATCTCCTTTTTGATGAATTAACTGAAACTGAATATAGTGGAGATAAATA	kaiB	Circadian clock protein KaiB	WP_011818959.	1325093..1325410	Circadian Rhythm	COG4251
WP_011818959.1_1448	CUST_7622_PI442190916	AS9606	HLII	WP_011818959.1_1448	ACCGCCTCCTGTGAGAAAAATAATAGGTGATCTCTCAGATAGAGAGAAAGTATTAATTGG	kaiB	Circadian clock protein KaiB	WP_011818959.	1325093..1325410	Circadian Rhythm	COG4251
WP_011818959.1_1448	CUST_7623_PI442190916	AS9606	HLII	WP_011818959.1_1448	CTCAAGCAACCCCAACTTGCAGAAGAAGATAAAATTTTAGCCACCCCAACCTTAGCTAAA	kaiB	Circadian clock protein KaiB	WP_011818959.	1325093..1325410	Circadian Rhythm	COG4251
WP_011818959.1_1448	CUST_7624_PI442190916	AS9606	HLII	WP_011818959.1_1448	TGAGAGCCTTAAATACTTTAAGAGGAATTTTAGAAAATGAATTCAAAGGTGTTTACGCTT	kaiB	Circadian clock protein KaiB	WP_011818959.	1325093..1325410	Circadian Rhythm	COG4251
WP_011818959.1_1448	CUST_7625_PI442190916	AS9606	HLII	WP_011818959.1_1448	TGGCAGCAAGAAAAACATACATTTTAAAACTCTATGTTGCTGGAAATACACCTAATTCAA	kaiB	Circadian clock protein KaiB	WP_011818959.	1325093..1325410	Circadian Rhythm	COG4251
WP_011819054.1_1518	CUST_7969_PI442190916	AS9606	HLII	WP_011819054.1_1518	CCAGAAAAAATTAAAGCCTTGAAAGAGATTTCAAAGGCTGAAGCTAGGATTCAAGCAGCA	atpC	ATP synthase epsilon subunit	WP_011819054.	1387557..1387961	Energy Metabolism	COG0355
WP_011819054.1_1518	CUST_7970_PI442190916	AS9606	HLII	WP_011819054.1_1518	TGCGGAAATTGGTTCTGAAATTAATGTTCAAAATGCTGAACAAGATCTTAAAGAAGCAAA	atpC	ATP synthase epsilon subunit	WP_011819054.	1387557..1387961	Energy Metabolism	COG0355
WP_011819054.1_1518	CUST_7971_PI442190916	AS9606	HLII	WP_011819054.1_1518	TAAGGCTAAGAATGAATTCCCAGTGGAAATCAATAGCTCTTATGGGAGGCTTCGCTGAAA	atpC	ATP synthase epsilon subunit	WP_011819054.	1387557..1387961	Energy Metabolism	COG0355
WP_011819054.1_1518	CUST_7972_PI442190916	AS9606	HLII	WP_011819054.1_1518	GCGAAGCTGAAGAAGTAATCCTTCCTAGTACTACTGGTCAACTTGGGATACTACCAGGAC	atpC	ATP synthase epsilon subunit	WP_011819054.	1387557..1387961	Energy Metabolism	COG0355
WP_011819066.1_1529	CUST_8028_PI442190916	AS9606	HLII	WP_011819066.1_1529	CTTCTTTTAAAAAGACCACCAGGAAGAGAGGCTTATCCTGGAGACGTGTTCTACTTGCAC	atpA	ATP synthase alpha subunit	WP_011819066.	1397246..1398763	Energy Metabolism	COG0056
WP_011819066.1_1529	CUST_8029_PI442190916	AS9606	HLII	WP_011819066.1_1529	ACTCAGGCAGGAGACGTTTCGGCTTACATTCCAACTAATGTTATTTCAATTACGGATGGA	atpA	ATP synthase alpha subunit	WP_011819066.	1397246..1398763	Energy Metabolism	COG0056
WP_011819066.1_1529	CUST_8030_PI442190916	AS9606	HLII	WP_011819066.1_1529	ACCAGCTATTAATGTTGGTATATCTGTTAGTCGTGTTGGAGGAGCAGCTCAGACAAAAGC	atpA	ATP synthase alpha subunit	WP_011819066.	1397246..1398763	Energy Metabolism	COG0056
WP_011819066.1_1529	CUST_8031_PI442190916	AS9606	HLII	WP_011819066.1_1529	AAAGCAACCTCAATTCTCTCCTCTAAACCTTGCAGAACAAGTTGCAGTTGTTTATGCAGG	atpA	ATP synthase alpha subunit	WP_011819066.	1397246..1398763	Energy Metabolism	COG0056
WP_011819066.1_1529	CUST_8032_PI442190916	AS9606	HLII	WP_011819066.1_1529	GTAGATTACTAGAAAGAGCAGCAAAACTTTCTGATGCAATGGGCGGGGGTTCTATGACAG	atpA	ATP synthase alpha subunit	WP_011819066.	1397246..1398763	Energy Metabolism	COG0056
WP_011819066.1_1529	CUST_8033_PI442190916	AS9606	HLII	WP_011819066.1_1529	TGCATCTGATCTTGATGAAGCAACTCAGCAACAACTTGAAAGAGGCAAAAGACTAAGAGA	atpA	ATP synthase alpha subunit	WP_011819066.	1397246..1398763	Energy Metabolism	COG0056
WP_011819070.1_1534	CUST_8055_PI442190916	AS9606	HLII	WP_011819070.1_1534	GGCGCTTTAATTCCGTGGAGATTGATTAAGTTACCAAGTGGAGAATTAGGAGCACCTACT	atpI	ATP synthase subunit A	WP_011819070.	1400787..1401512	Energy Metabolism	COG0356
WP_011819070.1_1534	CUST_8056_PI442190916	AS9606	HLII	WP_011819070.1_1534	GCATTGATTTTCGCAACTTTAGCGGCCTACTACATTGGAGAAGCTGTTGAAGAACATCAT	atpI	ATP synthase subunit A	WP_011819070.	1400787..1401512	Energy Metabolism	COG0356
WP_011819070.1_1534	CUST_8057_PI442190916	AS9606	HLII	WP_011819070.1_1534	AGGCCTTCAAAACTTGCTTGAGTTCCTTTGGGATTACATAAGAGATCTTGCTAGGACTCA	atpI	ATP synthase subunit A	WP_011819070.	1400787..1401512	Energy Metabolism	COG0356
WP_011819070.1_1534	CUST_8058_PI442190916	AS9606	HLII	WP_011819070.1_1534	GCCTTGGCTTTATTGGTTTCACTGTCTTATTTCTATGCAGGTTTAAGCAATAAGGGTTGG	atpI	ATP synthase subunit A	WP_011819070.	1400787..1401512	Energy Metabolism	COG0356
WP_011819070.1_1534	CUST_8059_PI442190916	AS9606	HLII	WP_011819070.1_1534	AGCATTAGAAGTTGGTCAACATCTTTATTGGCAAATTGGAAATATCAGACTTCATGGGCA	atpI	ATP synthase subunit A	WP_011819070.	1400787..1401512	Energy Metabolism	COG0356
WP_011819070.1_1534	CUST_8060_PI442190916	AS9606	HLII	WP_011819070.1_1534	TGTTCACCCAACTCCTATAATGCTTCCTTTCAAAATTCTAGAGGACTTTACGAAACCTTT	atpI	ATP synthase subunit A	WP_011819070.	1400787..1401512	Energy Metabolism	COG0356
WP_011819079.1_1544	CUST_8112_PI442190916	AS9606	HLII	WP_011819079.1_1544	TTGGTTACAGCCATAGTGCCAAAAGTTGGTTATGAAAAAGCAGCTGAAATTGCAAACCTT	fumC	class II fumarate hydratase	WP_011819079.	1409186..1410571	Krebs cycle and related genes	COG0114
WP_011819079.1_1544	CUST_8113_PI442190916	AS9606	HLII	WP_011819079.1_1544	GGTTTGTACTCAGATAATGGGTCTTGAATATGCAGTCTCAATGGCAAATTCTAGCGGCAC	fumC	class II fumarate hydratase	WP_011819079.	1409186..1410571	Krebs cycle and related genes	COG0114
WP_011819079.1_1544	CUST_8114_PI442190916	AS9606	HLII	WP_011819079.1_1544	TCAAAATGAACCTGGAAGTTCTATTATGCCAGGGAAAGTGAATCCAACTCAATGCGAAGC	fumC	class II fumarate hydratase	WP_011819079.	1409186..1410571	Krebs cycle and related genes	COG0114
WP_011819079.1_1544	CUST_8115_PI442190916	AS9606	HLII	WP_011819079.1_1544	TGGCCTCGCATGATCGTCTTGCTCAAGTAATGAGTCAGATAAAAATATTAGCAAGTGCAT	fumC	class II fumarate hydratase	WP_011819079.	1409186..1410571	Krebs cycle and related genes	COG0114
WP_011819079.1_1544	CUST_8116_PI442190916	AS9606	HLII	WP_011819079.1_1544	TGAATTGTATTTCTTACCTCTGGGAGGAACTGCGGTTGGTACAGGGATTAATTGTCCAAA	fumC	class II fumarate hydratase	WP_011819079.	1409186..1410571	Krebs cycle and related genes	COG0114
WP_011819079.1_1544	CUST_8117_PI442190916	AS9606	HLII	WP_011819079.1_1544	TCCCCTTACTTTTGGGCAAGAAATATCAGGATGGTCAGAGCAACTTAAAGATGCTGAGAA	fumC	class II fumarate hydratase	WP_011819079.	1409186..1410571	Krebs cycle and related genes	COG0114
WP_011819125.1_1588	CUST_8356_PI442190916	AS9606	HLII	WP_011819125.1_1588	GCGCAGGACAAAGCTTTGGTGCCTTTTTATTGAAGGGAATGAATGTTCAATTAATCGGAG	glsF	Ferredoxin-dependent glutamate synthase	WP_011819125.	1458439..1463013	Nitrogen Metabolism	COG0069
WP_011819125.1_1588	CUST_8357_PI442190916	AS9606	HLII	WP_011819125.1_1588	GGAGAAAGATTTGCAGTAAGAAATAGTGGTGCTACAGCAGTAACAGAAGGAGCAGGTGAT	glsF	Ferredoxin-dependent glutamate synthase	WP_011819125.	1458439..1463013	Nitrogen Metabolism	COG0069
WP_011819125.1_1588	CUST_8358_PI442190916	AS9606	HLII	WP_011819125.1_1588	AAATACAGATAGAAGTGTTTGTGCGAAAATATCAGGCGAAATCGCAGAACTTCACGGCAA	glsF	Ferredoxin-dependent glutamate synthase	WP_011819125.	1458439..1463013	Nitrogen Metabolism	COG0069
WP_011819125.1_1588	CUST_8359_PI442190916	AS9606	HLII	WP_011819125.1_1588	AGAGAAAACTTCCTCCGAACAGGTCATTTTAGGAAACACCTGTCTTTATGGAGCAACAGG	glsF	Ferredoxin-dependent glutamate synthase	WP_011819125.	1458439..1463013	Nitrogen Metabolism	COG0069
WP_011819125.1_1588	CUST_8360_PI442190916	AS9606	HLII	WP_011819125.1_1588	GGTGGGAAAGTAGTTATTCTAGGTTCCATAGGAAGGAATATTAGTGCAGGCATGACTGGT	glsF	Ferredoxin-dependent glutamate synthase	WP_011819125.	1458439..1463013	Nitrogen Metabolism	COG0069
WP_011819125.1_1588	CUST_8361_PI442190916	AS9606	HLII	WP_011819125.1_1588	ACAAGAGTACTTTTAGATTAATAGTTCCCCCAAGCGAAGAAGAAATGCTTGGTATAAAGA	glsF	Ferredoxin-dependent glutamate synthase	WP_011819125.	1458439..1463013	Nitrogen Metabolism	COG0069
WP_011819135.1_1599	CUST_8410_PI442190916	AS9606	HLII	WP_011819135.1_1599	TCCTGGACGTGGAGGTACTTGTGATAGTTCATCATGGGACGCTATGTATTTAGCTATGTT	psaB	Photosystem I P700 chlorophyll A apoprotein A2 PsaB	WP_011819135.	1469416..1471644	Photosystem I	COG2885
WP_011819135.1_1599	CUST_8411_PI442190916	AS9606	HLII	WP_011819135.1_1599	TCCTATTCGGACATTTAGTTTGGGCTACTGGTTTCATGTTCCTAATATCATGGCGTGGTT	psaB	Photosystem I P700 chlorophyll A apoprotein A2 PsaB	WP_011819135.	1469416..1471644	Photosystem I	COG2885
WP_011819135.1_1599	CUST_8412_PI442190916	AS9606	HLII	WP_011819135.1_1599	TGGGTAACTTTCTACTGGCATTGGAAACATTTAGCAATTTGGCAGGGTAATGTAGCACAA	psaB	Photosystem I P700 chlorophyll A apoprotein A2 PsaB	WP_011819135.	1469416..1471644	Photosystem I	COG2885
WP_011819135.1_1599	CUST_8413_PI442190916	AS9606	HLII	WP_011819135.1_1599	TAGGTCTTCATACAACTGCTTTAATCCTTATCAAGGGGGCTCTTGATGCTAGAGGTACCA	psaB	Photosystem I P700 chlorophyll A apoprotein A2 PsaB	WP_011819135.	1469416..1471644	Photosystem I	COG2885
WP_011819135.1_1599	CUST_8414_PI442190916	AS9606	HLII	WP_011819135.1_1599	CCTTGTTGGCTGGAGAGATAAGCCAGTAGCACTCTCAATTGTTCAAGCTAGATTAGTTGG	psaB	Photosystem I P700 chlorophyll A apoprotein A2 PsaB	WP_011819135.	1469416..1471644	Photosystem I	COG2885
WP_011819135.1_1599	CUST_8415_PI442190916	AS9606	HLII	WP_011819135.1_1599	TTGGTAACATTCTTACTTCGGTGCATTCGTGATTGCATCAACTTCTGGTAAGTTTGGCT	psaB	Photosystem I P700 chlorophyll A apoprotein A2 PsaB	WP_011819135.	1469416..1471644	Photosystem I	COG2885
WP_011819136.1_1600	CUST_8416_PI442190916	AS9606	HLII	WP_011819136.1_1600	GAGCTCTATCTATTACTCAGGGACGTGCAGTAGGTGTTACACACTTCCTAGTAGGTGGTA	psaA	Photosystem I P700 chlorophyll A apoprotein A1 PsaA	WP_011819136.	1471672..1473975	Photosystem I	COG2885
WP_011819136.1_1600	CUST_8417_PI442190916	AS9606	HLII	WP_011819136.1_1600	GTTTAGTAGAAGTTGGCGGAAAGGTAGCTATAGCGCCAATTCCATTAGGTACAGCTGATT	psaA	Photosystem I P700 chlorophyll A apoprotein A1 PsaA	WP_011819136.	1471672..1473975	Photosystem I	COG2885
WP_011819136.1_1600	CUST_8418_PI442190916	AS9606	HLII	WP_011819136.1_1600	GGAAGAGGTGGTACATGTCAAGTTTCTTCATGGGATCACGTTTTCTTAGCTCTCTTCTGG	psaA	Photosystem I P700 chlorophyll A apoprotein A1 PsaA	WP_011819136.	1471672..1473975	Photosystem I	COG2885
WP_011819136.1_1600	CUST_8419_PI442190916	AS9606	HLII	WP_011819136.1_1600	CTTCTCTTGGAAAATGCAGAGTGATGTTTGGGGACTTACTGGTGGTAATTTCGCACAAAG	psaA	Photosystem I P700 chlorophyll A apoprotein A1 PsaA	WP_011819136.	1471672..1473975	Photosystem I	COG2885
WP_011819136.1_1600	CUST_8420_PI442190916	AS9606	HLII	WP_011819136.1_1600	TGCTCATTTTATATGGGCGTTCAGTTTAATGTTCCTCTTCAGCGGTAGAGGATATTGGCA	psaA	Photosystem I P700 chlorophyll A apoprotein A1 PsaA	WP_011819136.	1471672..1473975	Photosystem I	COG2885
WP_011819136.1_1600	CUST_8421_PI442190916	AS9606	HLII	WP_011819136.1_1600	TTGTTCGAATCAATTGTTTGGGCACACAATAAACTAAAGGTTGCACCAACTATTCAGCCC	psaA	Photosystem I P700 chlorophyll A apoprotein A1 PsaA	WP_011819136.	1471672..1473975	Photosystem I	COG2885
WP_011819187.1_1655	CUST_8712_PI442190916	AS9606	HLII	WP_011819187.1_1655	TGGCAGAAACATCAAGGTCTCACTACCGAAAATTAGTGCATGAGAATCCTGACTTGTTGA	ppc	Phosphoenolpyruvate carboxylase	WP_011819187.	1513603..1516572	Krebs cycle and related genes	COG2352
WP_011819187.1_1655	CUST_8713_PI442190916	AS9606	HLII	WP_011819187.1_1655	TGGGGTTTTTGCAAGTTTCACTTTTAAGAAGATTAAGAGACCAGACAAGACAACCCCCAA	ppc	Phosphoenolpyruvate carboxylase	WP_011819187.	1513603..1516572	Krebs cycle and related genes	COG2352
WP_011819187.1_1655	CUST_8714_PI442190916	AS9606	HLII	WP_011819187.1_1655	GGCCTGCAAGAAGAAAAAAAGGTGCAAAAGATTTATCAAGTTTACGAGCTATTCCATGGG	ppc	Phosphoenolpyruvate carboxylase	WP_011819187.	1513603..1516572	Krebs cycle and related genes	COG2352
WP_011819187.1_1655	CUST_8715_PI442190916	AS9606	HLII	WP_011819187.1_1655	TACAGCAGAAGTGAACTATTAAGGGGAGCACTTTTAACTATTAATGGGATAGCAGCTGGT	ppc	Phosphoenolpyruvate carboxylase	WP_011819187.	1513603..1516572	Krebs cycle and related genes	COG2352
WP_011819187.1_1655	CUST_8717_PI442190916	AS9606	HLII	WP_011819187.1_1655	TCCACAACAAATTGAATTACTAAGAGTCTTGCATCAAAGATGGCCATTTTTTAGGATGCT	ppc	Phosphoenolpyruvate carboxylase	WP_011819187.	1513603..1516572	Krebs cycle and related genes	COG2352
WP_011819200.1_1667	CUST_8782_PI442190916	AS9606	HLII	WP_011819200.1_1667	AACTCCCCATTGAAGCTGTCCTATCAGATGCTATTGATTTGACTGGACCGATAAAACCTC		polysaccharide biosynthesis/export family protein	WP_011819200.	1527979..1529232	Other sugar transporters	COG1596
WP_011819200.1_1667	CUST_8783_PI442190916	AS9606	HLII	WP_011819200.1_1667	AGAGATGTACCTCTTGGAAAAGGCGGAGGCAAAAAGAGAGCGATTATTGATTTTAGTACT		polysaccharide biosynthesis/export family protein	WP_011819200.	1527979..1529232	Other sugar transporters	COG1596
WP_011819200.1_1667	CUST_8784_PI442190916	AS9606	HLII	WP_011819200.1_1667	AGTAGTTTTTTTGGAAAAACTACTGGTGTAATAAAAGAGATTACAGCTCCTTTTGTGGGT		polysaccharide biosynthesis/export family protein	WP_011819200.	1527979..1529232	Other sugar transporters	COG1596
WP_011819200.1_1667	CUST_8785_PI442190916	AS9606	HLII	WP_011819200.1_1667	TCAAAAGATCAAGTGAAAATCTAACTACTATTTCTGATGTAATTAGAAAAGCTGGTGGCA		polysaccharide biosynthesis/export family protein	WP_011819200.	1527979..1529232	Other sugar transporters	COG1596
WP_011819200.1_1667	CUST_8786_PI442190916	AS9606	HLII	WP_011819200.1_1667	TTACTCCAAAATTTATTACAGTAAGTCTTTTTGGTAGAGTTGAAACTACTGGAGTGGTAA		polysaccharide biosynthesis/export family protein	WP_011819200.	1527979..1529232	Other sugar transporters	COG1596
WP_011819200.1_1667	CUST_8787_PI442190916	AS9606	HLII	WP_011819200.1_1667	TTCTTATTCTGCAAATGCAAAAAGAGGATCCAAAAGAAATCCTTATTTAAAAAAAGACGA		polysaccharide biosynthesis/export family protein	WP_011819200.	1527979..1529232	Other sugar transporters	COG1596
WP_011819220.1_1687	CUST_8897_PI442190916	AS9606	HLII	WP_011819220.1_1687	TTGAGTTACCATATCGCAGCTCATAGAGGCCTTGATGTGGATCAACCAAGAAATTTAGCT	glmS	Glutamine--fructose-6-phosphatetransaminase(isomerizing)	WP_011819220.	1548284..1550179	Amino Acid metabolism	COG0449
WP_011819220.1_1687	CUST_8898_PI442190916	AS9606	HLII	WP_011819220.1_1687	ATTGCCTCTCCCGGTGAAGTTTTTGATAAAGTTATTAGTAATGCACAAGAAGCGAAAGCT	glmS	Glutamine--fructose-6-phosphatetransaminase(isomerizing)	WP_011819220.	1548284..1550179	Amino Acid metabolism	COG0449
WP_011819220.1_1687	CUST_8899_PI442190916	AS9606	HLII	WP_011819220.1_1687	ATTCATGCTGCTGGATATCCTGCTGGTGAAATGAAACATGGTCCAATAGCTTTATTAGAT	glmS	Glutamine--fructose-6-phosphatetransaminase(isomerizing)	WP_011819220.	1548284..1550179	Amino Acid metabolism	COG0449
WP_011819220.1_1687	CUST_8900_PI442190916	AS9606	HLII	WP_011819220.1_1687	GGAAGGCAGGTTTCTAATATAATTGATATCTGTGCAGGAATAGAAGTTGGAGTAGCTGCA	glmS	Glutamine--fructose-6-phosphatetransaminase(isomerizing)	WP_011819220.	1548284..1550179	Amino Acid metabolism	COG0449
WP_011819220.1_1687	CUST_8901_PI442190916	AS9606	HLII	WP_011819220.1_1687	TGGATTAGCCATAAAATTTGCTCAAATTAAAGGCAACCAAAGTCCTGATGAAATAGGAAA	glmS	Glutamine--fructose-6-phosphatetransaminase(isomerizing)	WP_011819220.	1548284..1550179	Amino Acid metabolism	COG0449
WP_011819220.1_1687	CUST_8902_PI442190916	AS9606	HLII	WP_011819220.1_1687	GGGATAGCTCCTGAATGTAATGGAACTGAAATCTTTGATTATTTAATGAAAGTTCCCTCC	glmS	Glutamine--fructose-6-phosphatetransaminase(isomerizing)	WP_011819220.	1548284..1550179	Amino Acid metabolism	COG0449
010.1_WP_079333896.	CUST_13_PI442190715	HOT208_60m_813E23		010.1_WP_079333896.	GTGCTTTGGCGGGTTATGGATTTCTGGAGGAACCGCATCAAAACATTTCAAAAACTTTAA	glk	Glucokinase	WP_079333896.	1546..2580	Glycolysis	COG0837
010.1_WP_079333896.	CUST_14_PI442190715	HOT208_60m_813E23		010.1_WP_079333896.	TCTTTCCAAAGAAGGGGATCAGCTAATGATTGAAGTTGAGAGGATTTGGTTAGGCGCTTA	glk	Glucokinase	WP_079333896.	1546..2580	Glycolysis	COG0837
010.1_WP_079333896.	CUST_15_PI442190715	HOT208_60m_813E23		010.1_WP_079333896.	AATTATTAGCGGCAGTGGCTTATCAAGAATTGCAGAATGGAGACTAAGCAAACCTGATGC	glk	Glucokinase	WP_079333896.	1546..2580	Glycolysis	COG0837
010.1_WP_079333896.	CUST_16_PI442190715	HOT208_60m_813E23		010.1_WP_079333896.	ATCCAGAATGGATCACAATCTGAAGGTACTAACAATGATTTGCATGCCATTGTTGGAGCC	glk	Glucokinase	WP_079333896.	1546..2580	Glycolysis	COG0837
010.1_WP_079333896.	CUST_17_PI442190715	HOT208_60m_813E23		010.1_WP_079333896.	AGTTTTAGCTAGCGAAGGTGGTCATGTTGAGTACTCCCCAAAGTCAAAATTAGAATGGGA	glk	Glucokinase	WP_079333896.	1546..2580	Glycolysis	COG0837
010.1_WP_079333896.	CUST_18_PI442190715	HOT208_60m_813E23		010.1_WP_079333896.	TGAATGTAATTTTAGATGAAGAGTTTGGACTTTTAGTGCAGCCTGCAGAGCAAAAATGC	glk	Glucokinase	WP_079333896.	1546..2580	Glycolysis	COG0837
108.1_WP_011862568.	CUST_409_PI442190715	HOT208_60m_813E23		108.1_WP_011862568.	ACGATGCTTACACTCAAAGTCAAGGAACAGCTTTTGTAGTTTTCCAAGGTCGTTAA	rbcS	Ribulose bisphosphate carboxylase small subunit	WP_011862568.	2452..2793	Carbon fixation	COG4451
108.1_WP_011862568.	CUST_410_PI442190715	HOT208_60m_813E23		108.1_WP_011862568.	GCGAATTAGAGGCATGCCATAGAGCATACCCTGATCATCATGTAAGAATCATCGGATACG	rbcS	Ribulose bisphosphate carboxylase small subunit	WP_011862568.	2452..2793	Carbon fixation	COG4451
108.1_WP_011862568.	CUST_411_PI442190715	HOT208_60m_813E23		108.1_WP_011862568.	CTTATTGGTCTTATTGGAAACTCCCATTCTTTGGGGAAAAAGATCTTAACTTGATAGTG	rbcS	Ribulose bisphosphate carboxylase small subunit	WP_011862568.	2452..2793	Carbon fixation	COG4451
108.1_WP_011862568.	CUST_412_PI442190715	HOT208_60m_813E23		108.1_WP_011862568.	AATTGCTCAAGGTTGGAGTCCAGTTATTGAGCATGTTCATCCAAGTGGATGTATGCAAAC	rbcS	Ribulose bisphosphate carboxylase small subunit	WP_011862568.	2452..2793	Carbon fixation	COG4451
108.1_WP_011862568.	CUST_413_PI442190715	HOT208_60m_813E23		108.1_WP_011862568.	CTGGAAACATTCGGTTTCTTACCACCGATGACCCAGGAAGAAATATATGACCAAATTGCC	rbcS	Ribulose bisphosphate carboxylase small subunit	WP_011862568.	2452..2793	Carbon fixation	COG4451
108.1_77	CUST_418_PI442190715	HOT208_60m_813E23		108.1_77	GAAACTTGGAAGGAAATTAAGTTCGAGTTTGACACTGTCGACAAGCTTGATGTTCAGGGT	rbcL	Ribulose 1,5-bisphosphate carboxylase large subunit		2883..3744	Carbon fixation	COG1850
011.1_WP_079333908.	CUST_448_PI442190715	HOT208_60m_813E23		011.1_WP_079333908.	CGATGATTATTGGGAAGATATTGGAACCATTGGGGCATTCTTTGAGTCCAATCTTGCCTT	glgC	Glucose-1-phosphate adenylyltransferase	WP_079333908.	<1..1295	Glycogen Metabolism	COG0448
011.1_WP_079333908.	CUST_449_PI442190715	HOT208_60m_813E23		011.1_WP_079333908.	AGATTTCTTTGAATCGCCTGAAGAGAGGATTGAATTAAGAAAAGGAGGCGGAACACCTCT	glgC	Glucose-1-phosphate adenylyltransferase	WP_079333908.	<1..1295	Glycogen Metabolism	COG0448
011.1_WP_079333908.	CUST_450_PI442190715	HOT208_60m_813E23		011.1_WP_079333908.	TTAGGGGTAAGAAGCAGGATTGAAAGTGATTCGGTTCTTGAGGATACTCTTGTTATGGGG	glgC	Glucose-1-phosphate adenylyltransferase	WP_079333908.	<1..1295	Glycogen Metabolism	COG0448
011.1_WP_079333908.	CUST_451_PI442190715	HOT208_60m_813E23		011.1_WP_079333908.	AGTGAAAAGCCAACTGGAGAGAAGTTGAAGGCAATGGCAGTAGATACTTCAAAATTTGGA	glgC	Glucose-1-phosphate adenylyltransferase	WP_079333908.	<1..1295	Glycogen Metabolism	COG0448
011.1_WP_079333908.	CUST_452_PI442190715	HOT208_60m_813E23		011.1_WP_079333908.	TTTCCAATCTACACAAGACCAAGATTTCTCCCCCCTTCTAAACTCGTAGATGCTCAAATT	glgC	Glucose-1-phosphate adenylyltransferase	WP_079333908.	<1..1295	Glycogen Metabolism	COG0448
011.1_WP_079333908.	CUST_453_PI442190715	HOT208_60m_813E23		011.1_WP_079333908.	ACGGATTTTGGTAAGGACATAATTCCTGAAGCACTTGATAGGGGAGATACTCTTAAAAGT	glgC	Glucose-1-phosphate adenylyltransferase	WP_079333908.	<1..1295	Glycogen Metabolism	COG0448
012.1_WP_079333914.	CUST_781_PI442190715	HOT208_60m_813E23		012.1_WP_079333914.	TGGTTGTGGTGGAATGGAAGCTTGGATGAATCGCCAGAAATCTTCGAATTTTTTACTGAT	opcA	Glucose-6-P dehydrogenase subunit opcA	WP_079333914.	2384..3685	Pentose Phosphate	COG3429
012.1_WP_079333914.	CUST_782_PI442190715	HOT208_60m_813E23		012.1_WP_079333914.	GCTGTGAATCAGTGGAGTGTATGAGACTTGAAGCAGGGGGAATGGCTAATATGGAATTAA	opcA	Glucose-6-P dehydrogenase subunit opcA	WP_079333914.	2384..3685	Pentose Phosphate	COG3429
012.1_WP_079333914.	CUST_783_PI442190715	HOT208_60m_813E23		012.1_WP_079333914.	GGGTTAGATTGAAAAATTGGAGAGAATCATTGGCAATGATTTTTGATCCGCCCTCGAGGA	opcA	Glucose-6-P dehydrogenase subunit opcA	WP_079333914.	2384..3685	Pentose Phosphate	COG3429
012.1_WP_079333914.	CUST_784_PI442190715	HOT208_60m_813E23		012.1_WP_079333914.	TGTTTTTGATTTCATGGATTAGCGATAAGCTTGGTTGGTCATTTCTGAGAGTTGAAAGGG	opcA	Glucose-6-P dehydrogenase subunit opcA	WP_079333914.	2384..3685	Pentose Phosphate	COG3429
012.1_WP_079333914.	CUST_785_PI442190715	HOT208_60m_813E23		012.1_WP_079333914.	TCCCTTATCTTTGGGAAATCCAAGTATTCATTTAGGACAAGTTATTGGATTGAGGCTGAT	opcA	Glucose-6-P dehydrogenase subunit opcA	WP_079333914.	2384..3685	Pentose Phosphate	COG3429
012.1_WP_079333914.	CUST_786_PI442190715	HOT208_60m_813E23		012.1_WP_079333914.	ACATCAGAGTATGATGTTAGTAAATTGTTGGGAAGTAGTAGAGGAAATACCAGCCCTCTT	opcA	Glucose-6-P dehydrogenase subunit opcA	WP_079333914.	2384..3685	Pentose Phosphate	COG3429
126.1_WP_079334412.	CUST_890_PI442190715	HOT208_60m_813E23		126.1_WP_079334412.	ATTCTTATGGATGTGGCGATTCTTTTGCGGCTGGGATTCTTTATGGAATGGCATCTAAAT	rbsK	Sugar kinase, ribokinase family	WP_079334412.	2996..3865	Pentose Phosphate	COG0524
126.1_WP_079334412.	CUST_891_PI442190715	HOT208_60m_813E23		126.1_WP_079334412.	TTGCTAAAGTAATGGGAAGAGACGCCAGTGAATTTTTCGGCCCATATGCAAATAGTGATG	rbsK	Sugar kinase, ribokinase family	WP_079334412.	2996..3865	Pentose Phosphate	COG0524
126.1_WP_079334412.	CUST_892_PI442190715	HOT208_60m_813E23		126.1_WP_079334412.	ACTATTAAAACCGAGGGAGAGAAGGGTGGCATAATATTCCCTGGAGGAAGATATAAGGCT	rbsK	Sugar kinase, ribokinase family	WP_079334412.	2996..3865	Pentose Phosphate	COG0524
126.1_WP_079334412.	CUST_893_PI442190715	HOT208_60m_813E23		126.1_WP_079334412.	ATTACCAAAACCCGGAGTTATTTCTCATTCTGAAAAGTCCCTTGAGTATCCAGCTGGTGG	rbsK	Sugar kinase, ribokinase family	WP_079334412.	2996..3865	Pentose Phosphate	COG0524
126.1_WP_079334412.	CUST_894_PI442190715	HOT208_60m_813E23		126.1_WP_079334412.	AATTGACTCTCAAGGTGAAAGAGCAATAACAGTTATTGGAGAAAGGTTAGCTCCAACTCA	rbsK	Sugar kinase, ribokinase family	WP_079334412.	2996..3865	Pentose Phosphate	COG0524
126.1_WP_079334412.	CUST_895_PI442190715	HOT208_60m_813E23		126.1_WP_079334412.	CTCTCAAATATGGGAATTAAGTTGCATGTAGCTTGGCGTGATATACCAACTAGAAGAGGA	rbsK	Sugar kinase, ribokinase family	WP_079334412.	2996..3865	Pentose Phosphate	COG0524
183.1_WP_079334572.	CUST_1693_PI442190715	HOT208_60m_813E23		183.1_WP_079334572.	CCAATAGACTTCCTCAGAATCTTGTGCAGGCAATGAGAGACTGTTTTGGCTCTCATACAT	edd	phosphogluconate dehydrogenase (NADP(+)-dependent, decarboxyla	WP_079334572.	6152..7084	Pentose Phosphate/Entner-Dudorof	COG2084
183.1_WP_079334572.	CUST_1694_PI442190715	HOT208_60m_813E23		183.1_WP_079334572.	ATGAAACCTTTATTTGATGCTGTAGTCCTTGCCACAATTGCTAGCTATGCCCAAGGTATG	edd	phosphogluconate dehydrogenase (NADP(+)-dependent, decarboxyla	WP_079334572.	6152..7084	Pentose Phosphate/Entner-Dudorof	COG2084
183.1_WP_079334572.	CUST_1695_PI442190715	HOT208_60m_813E23		183.1_WP_079334572.	ACAGGTTTATGGACTGTTGTGAGTGCTCTAGAACTGGGGGTATCAGTCCCAACTATTTAT	edd	phosphogluconate dehydrogenase (NADP(+)-dependent, decarboxyla	WP_079334572.	6152..7084	Pentose Phosphate/Entner-Dudorof	COG2084
183.1_WP_079334572.	CUST_1696_PI442190715	HOT208_60m_813E23		183.1_WP_079334572.	GCTAAGGTAGTTTCTCTATCCACAAAGGCAGGTATACCAGTCCCATGTTTATCCAGTACT	edd	phosphogluconate dehydrogenase (NADP(+)-dependent, decarboxyla	WP_079334572.	6152..7084	Pentose Phosphate/Entner-Dudorof	COG2084
183.1_WP_079334572.	CUST_1697_PI442190715	HOT208_60m_813E23		183.1_WP_079334572.	GCAGTATATAACTATGGTTTGAATATGCCGTCAATTGCACAAATATGGAAAGGTGGTTGC	edd	phosphogluconate dehydrogenase (NADP(+)-dependent, decarboxyla	WP_079334572.	6152..7084	Pentose Phosphate/Entner-Dudorof	COG2084
183.1_WP_079334572.	CUST_1698_PI442190715	HOT208_60m_813E23		183.1_WP_079334572.	GATGATATAACTGGAGATGATGTCGTGGAAAAAATATTAGATAAAGCTGGCCAGAAAGGT	edd	phosphogluconate dehydrogenase (NADP(+)-dependent, decarboxyla	WP_079334572.	6152..7084	Pentose Phosphate/Entner-Dudorof	COG2084
196.1_375	CUST_1875_PI442190715	HOT208_60m_813E23		196.1_375	ATGATGCAATAGAGAGCTTACTAACAAAAATGGCTGCCAAAGTTGAAGACGGACCATGTG	edd	phosphogluconate dehydrogenase (NADP(+)-dependent, decarboxylating)		85..593	Pentose Phosphate/Entner-Dudorof	COG2084
196.1_375	CUST_1876_PI442190715	HOT208_60m_813E23		196.1_375	TAAAGCTGGGCCAGCAACAGATGCTGTCATTGATAATATTTCTGGATATCTCGAGGAAGG	edd	phosphogluconate dehydrogenase (NADP(+)-dependent, decarboxylating)		85..593	Pentose Phosphate/Entner-Dudorof	COG2084
196.1_375	CUST_1877_PI442190715	HOT208_60m_813E23		196.1_375	AATACTCTTGAAAGTAAAAGTTTTGGATACATCGGAATGGGAGTCTCTGGGGGTGCCAAA	edd	phosphogluconate dehydrogenase (NADP(+)-dependent, decarboxylating)		85..593	Pentose Phosphate/Entner-Dudorof	COG2084
196.1_375	CUST_1878_PI442190715	HOT208_60m_813E23		196.1_375	TGAAGAATTCTTACAGGGTCGAGGCCTTGGGAAGAATATAGAAGGAGCTGAAACTCTTCA	edd	phosphogluconate dehydrogenase (NADP(+)-dependent, decarboxylating)		85..593	Pentose Phosphate/Entner-Dudorof	COG2084
196.1_375	CUST_1879_PI442190715	HOT208_60m_813E23		196.1_375	AGGCACATTTTGGTTTAGTAGGTCTTGGTGTTATGGGCGAAAATTTAGTTCTTAACGCAG	edd	phosphogluconate dehydrogenase (NADP(+)-dependent, decarboxylating)		85..593	Pentose Phosphate/Entner-Dudorof	COG2084
196.1_375	CUST_1880_PI442190715	HOT208_60m_813E23		196.1_375	TCTGGGGGTGCCAAAGGAGCTCTAGAGGGTCCAAGCATGATGCCC	edd	phosphogluconate dehydrogenase (NADP(+)-dependent, decarboxylating)		85..593	Pentose Phosphate/Entner-Dudorof	COG2084
008.1_WP_079333861.	CUST_2361_PI442190715	HOT208_60m_813E23		008.1_WP_079333861.	CGTTGATAACTGGCGTTGGCAAGGGGTTCCTTTTTATTTGAGAACAGGTAAAAGACTACC	zwf	glucose-6-phosphate dehydrogenase zwf	WP_079333861.	<1..1384	Pentose Phosphate	COG0364
008.1_WP_079333861.	CUST_2362_PI442190715	HOT208_60m_813E23		008.1_WP_079333861.	ATGCTTCAAAATCATATGACCCAAATGCTTGCTGTTACTGCTATGGAACCTCCTGGAAAG	zwf	glucose-6-phosphate dehydrogenase zwf	WP_079333861.	<1..1384	Pentose Phosphate	COG0364
008.1_WP_079333861.	CUST_2363_PI442190715	HOT208_60m_813E23		008.1_WP_079333861.	TAGCTGATGCAATGCTTTCTGACCCAACCCTATTTACTCGAAGTGATGAAGTAGAAGCAG	zwf	glucose-6-phosphate dehydrogenase zwf	WP_079333861.	<1..1384	Pentose Phosphate	COG0364
008.1_WP_079333861.	CUST_2364_PI442190715	HOT208_60m_813E23		008.1_WP_079333861.	TTACTTCATCTGAAACAGTGGGCGTTGAAGATAGAGCAGGTTATTACGAAAGCTCTGGTG	zwf	glucose-6-phosphate dehydrogenase zwf	WP_079333861.	<1..1384	Pentose Phosphate	COG0364
008.1_WP_079333861.	CUST_2365_PI442190715	HOT208_60m_813E23		008.1_WP_079333861.	CTTGCTGACGAAAATGAACCTTGGAATTGTTGCATAAGAGGTCAATATGGAGAAGGAGGG	zwf	glucose-6-phosphate dehydrogenase zwf	WP_079333861.	<1..1384	Pentose Phosphate	COG0364
008.1_WP_079333861.	CUST_2366_PI442190715	HOT208_60m_813E23		008.1_WP_079333861.	TCCTGCCCCAAATCAACTTATCCTTAGAATTCAGCCAAATGAAGGTGCTACTTTCAAATT	zwf	glucose-6-phosphate dehydrogenase zwf	WP_079333861.	<1..1384	Pentose Phosphate	COG0364

009.1_WP_011376344.:	CUST_2522_PI442190715	HOT208_60m_813E23 009.1_WP_011376344.:	AGGTGAAGCGGAAGATGGTCATGGATTTGAAGGTGAACTTTCTACAGATATGCTTCTGAC	cbbA	Fructose-1,6-bisphosphate aldolase class II	WP_011376344.:	1078..2151	Glycolysis/Gluconeogenesis	COG0191
009.1_WP_011376344.:	CUST_2524_PI442190715	HOT208_60m_813E23 009.1_WP_011376344.:	AGCATTTGCTGATAAGGCAAACTTCGACCCAAGACATTTCAACAAGCCTGCTAGAAAATA	cbbA	Fructose-1,6-bisphosphate aldolase class II	WP_011376344.:	1078..2151	Glycolysis/Gluconeogenesis	COG0191
009.1_WP_011376344.:	CUST_2525_PI442190715	HOT208_60m_813E23 009.1_WP_011376344.:	TACAAGCAGTTCTGGTGCGAAGGTCAAGCAAGCAAGATCAAACAAAACAGCACTAATTAT	cbbA	Fructose-1,6-bisphosphate aldolase class II	WP_011376344.:	1078..2151	Glycolysis/Gluconeogenesis	COG0191
009.1_WP_011376344.:	CUST_2527_PI442190715	HOT208_60m_813E23 009.1_WP_011376344.:	TGGCGGTGAAATCCCTCAAACTTATGGTGTTCCTGTTGAAGAAATTCAAGAGGGAATAAG	cbbA	Fructose-1,6-bisphosphate aldolase class II	WP_011376344.:	1078..2151	Glycolysis/Gluconeogenesis	COG0191
092.1_WP_079334001.:	CUST_2590_PI442190715	HOT208_60m_813E23 092.1_WP_079334001.:	GAGACGTTAACCCAGGAGCAATAGTTTCCGCTGAGAAAAATATTTACGTTTGGGGCAAAT	minC	septum site-determining protein MinC	WP_079334001.:	5406..6068	Cell Division	COG0850
092.1_WP_079334001.:	CUST_2591_PI442190715	HOT208_60m_813E23 092.1_WP_079334001.:	TGCAGATGTGGTAGCTATTGGACCAAAGGATAAGCCCAAAAATTGTTATCCAGAAGTTGC	minC	septum site-determining protein MinC	WP_079334001.:	5406..6068	Cell Division	COG0850
092.1_WP_079334001.:	CUST_2592_PI442190715	HOT208_60m_813E23 092.1_WP_079334001.:	AGGAATCGCGTTCGCAGGGAAAAGTGGAAATAAAAATGCCTCTATTGCATCACTTTATCT	minC	septum site-determining protein MinC	WP_079334001.:	5406..6068	Cell Division	COG0850
092.1_WP_079334001.:	CUST_2593_PI442190715	HOT208_60m_813E23 092.1_WP_079334001.:	TTCACAAAGGAACAGTACGATCCGGTGAAAGAATATCTTCAAATGGAAACCTATGCATCA	minC	septum site-determining protein MinC	WP_079334001.:	5406..6068	Cell Division	COG0850
092.1_WP_079334001.:	CUST_2594_PI442190715	HOT208_60m_813E23 092.1_WP_079334001.:	TCTTCCATCAAGGGACATTTAGATGCAAAAATTTTCGCAGTCAATGAGACAATAAGTTCT	minC	septum site-determining protein MinC	WP_079334001.:	5406..6068	Cell Division	COG0850
092.1_WP_079334001.:	CUST_2595_PI442190715	HOT208_60m_813E23 092.1_WP_079334001.:	AGAGAAACAATCTTGAGTGGAAAATCTTTAAAAATAGATTCAGCTTTTGTAGAAGAGCAA	minC	septum site-determining protein MinC	WP_079334001.:	5406..6068	Cell Division	COG0850
092.1_WP_025913401.:	CUST_2596_PI442190715	HOT208_60m_813E23 092.1_WP_025913401.:	CTGCCGGAGATCCAAGGATGTTGGATTGGATGAAGCCCGAAGATATGAAAAAAATTAGTG	minD	septum site-determining protein MinD	WP_025913401.:	6180..6995	Cell Division	COG2894
092.1_WP_025913401.:	CUST_2597_PI442190715	HOT208_60m_813E23 092.1_WP_025913401.:	AGAGTTCGCCCTAACATGATGGCTAGTCAAGAAATGTTATCCATCGAAGATGTTCAAGGG	minD	septum site-determining protein MinD	WP_025913401.:	6180..6995	Cell Division	COG2894
092.1_WP_025913401.:	CUST_2598_PI442190715	HOT208_60m_813E23 092.1_WP_025913401.:	GTAGAAGATGGCTTTAAAAATGCTCTAGCGGCCTGCAAAGAAGCTATTGTGGTCACTAAC	minD	septum site-determining protein MinD	WP_025913401.:	6180..6995	Cell Division	COG2894
092.1_WP_025913401.:	CUST_2599_PI442190715	HOT208_60m_813E23 092.1_WP_025913401.:	CCAGAACTATCCGCAGTGCGGGATGCGGATAGAGTAATAGGGATTCTTAATACTTCAGAT	minD	septum site-determining protein MinD	WP_025913401.:	6180..6995	Cell Division	COG2894
092.1_WP_025913401.:	CUST_2600_PI442190715	HOT208_60m_813E23 092.1_WP_025913401.:	AGCGGAGCAACAACTGCTGTGTTAGATGCCGATTTTGGCTTAAGAAATTTAGATCTTCTT	minD	septum site-determining protein MinD	WP_025913401.:	6180..6995	Cell Division	COG2894
092.1_WP_025913401.:	CUST_2601_PI442190715	HOT208_60m_813E23 092.1_WP_025913401.:	GCACTAATAGAGGGGAACCACTGACACTTTCAGATGGTAGATCTCCTGCAAAAAGATGTT	minD	septum site-determining protein MinD	WP_025913401.:	6180..6995	Cell Division	COG2894
092.1_WP_025890423.:	CUST_2602_PI442190715	HOT208_60m_813E23 092.1_WP_025890423.:	TCTCAGGAGAAATAAAATTCAAAAAAACTGATAAAACTGATAAAGTTGATAAATCTAACA	minE	septum site-determining protein MinE	WP_025890423.:	6997..7341	Cell Division	COG0851
092.1_WP_025890423.:	CUST_2603_PI442190715	HOT208_60m_813E23 092.1_WP_025890423.:	AGCAGTAAGTTTAGAGACTGAGGATAGAATGACTGCATTAGTTGCCAATTTACCAATCAA	minE	septum site-determining protein MinE	WP_025890423.:	6997..7341	Cell Division	COG0851
092.1_WP_025890423.:	CUST_2604_PI442190715	HOT208_60m_813E23 092.1_WP_025890423.:	AATGAGGAAAGAAATTCTCGATGTTGTTGCTAAATACGTAGAGATTGATTTTGATGAAGT	minE	septum site-determining protein MinE	WP_025890423.:	6997..7341	Cell Division	COG0851
092.1_WP_025890423.:	CUST_2605_PI442190715	HOT208_60m_813E23 092.1_WP_025890423.:	ACTTGTACTTGCTCATGACAGAGTTGATATGAGTTCCTTAACAAC	minE	septum site-determining protein MinE	WP_025890423.:	6997..7341	Cell Division	COG0851
092.1_WP_025890423.:	CUST_2606_PI442190715	HOT208_60m_813E23 092.1_WP_025890423.:	AGGCAGAGAAACGTCTAGTGCAAATACGGCTAGAGAAAGATTACAACTTGTACTTGCTCA	minE	septum site-determining protein MinE	WP_025890423.:	6997..7341	Cell Division	COG0851
095.1_WP_025880735.:	CUST_3013_PI442190715	HOT208_60m_813E23 095.1_WP_025880735.:	TGGCAATCGAGAATCAATGGCGGAAGATTAACCAGTCAAAATATGTTTCCATTGGAAGCT	cyoC	heme-copper oxidase subunit III	WP_025880735.:	14251..14853	Respiration	COG1845
095.1_WP_025880735.:	CUST_3014_PI442190715	HOT208_60m_813E23 095.1_WP_025880735.:	TGCAAGCACTTTTTATGCTCTTACGGGGTTTCATGGATTACATGTCACTTTAGGCACTTT	cyoC	heme-copper oxidase subunit III	WP_025880735.:	14251..14853	Respiration	COG1845
095.1_WP_025880735.:	CUST_3015_PI442190715	HOT208_60m_813E23 095.1_WP_025880735.:	TTGGTCTTATAACTTTCCTAATTGCGGACGGAATGACTTTTGCTGGATTCTTTGCTGCTT	cyoC	heme-copper oxidase subunit III	WP_025880735.:	14251..14853	Respiration	COG1845
095.1_WP_025880735.:	CUST_3016_PI442190715	HOT208_60m_813E23 095.1_WP_025880735.:	AGTGCAACTTTCCATAAAGCAGGCAAAGCACTTTTAAAAGATAAAAACTCAGATTCCCAA	cyoC	heme-copper oxidase subunit III	WP_025880735.:	14251..14853	Respiration	COG1845
095.1_WP_025880735.:	CUST_3017_PI442190715	HOT208_60m_813E23 095.1_WP_025880735.:	AGCAGTAAATCCATTACCAGATGGTGCTATTTATGAATTAGAACTACCAATACCTACTCT	cyoC	heme-copper oxidase subunit III	WP_025880735.:	14251..14853	Respiration	COG1845
095.1_WP_025880735.:	CUST_3018_PI442190715	HOT208_60m_813E23 095.1_WP_025880735.:	AGCTCTAAAGAAATTCAAAAAAATAATTCTGAAGTTAATGAAACACATGAAGACTTCAGA	cyoC	heme-copper oxidase subunit III	WP_025880735.:	14251..14853	Respiration	COG1845
095.1_WP_079334103.:	CUST_3019_PI442190715	HOT208_60m_813E23 095.1_WP_079334103.:	GCTTTGCTCCTCAACATTGGCTTGGTTTAAATGGAATGCCAAGAAGAGTTGCAGAATATG	cyoB/coxA	Cytochrome c oxidase subunit I	WP_079334103.:	14860..16485	Respiration	COG0843
095.1_WP_079334103.:	CUST_3020_PI442190715	HOT208_60m_813E23 095.1_WP_079334103.:	GAGATAACCCTTGGAATGCTCTTACACCTGAATGGTTAACATCTTCTCCACCTCCAGTTG	cyoB/coxA	Cytochrome c oxidase subunit I	WP_079334103.:	14860..16485	Respiration	COG0843
095.1_WP_079334103.:	CUST_3021_PI442190715	HOT208_60m_813E23 095.1_WP_079334103.:	CCCCTTGGATGAGATTATTCTTTACTATCGCCACAGCATTTATTGCTGTTCCGACAGGTA	cyoB/coxA	Cytochrome c oxidase subunit I	WP_079334103.:	14860..16485	Respiration	COG0843
095.1_WP_079334103.:	CUST_3022_PI442190715	HOT208_60m_813E23 095.1_WP_079334103.:	AATGGGGATAGTAGTTTTAGGTTTAGTTGTTTGGGCGCATCACATGTTTACGAGTGGAAC	cyoB/coxA	Cytochrome c oxidase subunit I	WP_079334103.:	14860..16485	Respiration	COG0843
095.1_WP_079334103.:	CUST_3023_PI442190715	HOT208_60m_813E23 095.1_WP_079334103.:	TGCTTTAGCACAGGTACCTTTCGATATTCACGTACATGATACCTATTTCGTTGTAGCCCA	cyoB/coxA	Cytochrome c oxidase subunit I	WP_079334103.:	14860..16485	Respiration	COG0843
095.1_WP_079334103.:	CUST_3024_PI442190715	HOT208_60m_813E23 095.1_WP_079334103.:	TGATCCTCAATTTCAGTTCGTTAATCAAATTAGTAGCCTTGGGGCTCTTTTGATGGCT	cyoB/coxA	Cytochrome c oxidase subunit I	WP_079334103.:	14860..16485	Respiration	COG0843
095.1_WP_025887633.:	CUST_3025_PI442190715	HOT208_60m_813E23 095.1_WP_025887633.:	AATGGAATCCAAAGACGTCATTCATGCTTTTTGGGTGCCAGAGTTCAGAATTAAACAGGA	ctaC/coxB	Cytochrome c oxidase subunit II	WP_025887633.:	16482..17285	Respiration	COG1622
095.1_WP_025887633.:	CUST_3026_PI442190715	HOT208_60m_813E23 095.1_WP_025887633.:	GTGGCCCATATCATGGAGGGATGAGAGCCTCCATAATTGTTGAAGAAGAATCTGATTACA	ctaC/coxB	Cytochrome c oxidase subunit II	WP_025887633.:	16482..17285	Respiration	COG1622
095.1_WP_025887633.:	CUST_3027_PI442190715	HOT208_60m_813E23 095.1_WP_025887633.:	AGCTACAACATCTACGATCGGATGGGAGGGATGAAAGAACTAAATCATAACCACGAAATG	ctaC/coxB	Cytochrome c oxidase subunit II	WP_025887633.:	16482..17285	Respiration	COG1622
095.1_WP_025887633.:	CUST_3028_PI442190715	HOT208_60m_813E23 095.1_WP_025887633.:	TCTTTTTGGATTGGTTTTAATGTAAATTTGCTTCCAGCCGAAGCAAGTATCAATGCACCA	ctaC/coxB	Cytochrome c oxidase subunit II	WP_025887633.:	16482..17285	Respiration	COG1622
095.1_WP_025887633.:	CUST_3029_PI442190715	HOT208_60m_813E23 095.1_WP_025887633.:	TCCCAAAGGCAATTTCATATCAGGAGAACTACACGTTCCTGTTGATCAAAAAGTAACAAT	ctaC/coxB	Cytochrome c oxidase subunit II	WP_025887633.:	16482..17285	Respiration	COG1622
095.1_WP_025887633.:	CUST_3030_PI442190715	HOT208_60m_813E23 095.1_WP_025887633.:	ACAGCCTACTATTCTAAATTTCACTCCCACAAAAGTAGGAAAATATCCGATAATTTGCGC	ctaC/coxB	Cytochrome c oxidase subunit II	WP_025887633.:	16482..17285	Respiration	COG1622
112.1_23	CUST_99_PI442190815	HOT208_60m_813102 112.1_23	AAATGGCCGCTAGCTCAACCCATGAGATTTTTGGGTCATAATGGCGAAATAAATACTCTC	glnA	Glutamine synthetase/ glutamate--ammonialigase		<1..848	Nitrogen Metabolism	COG0174
112.1_23	CUST_100_PI442190815	HOT208_60m_813102 112.1_23	AGGAGATAGTGATTCAGGAGATGGAGCGGGCATATTATGTTCAATTCCATGGGGATATCT	glnA	Glutamine synthetase/ glutamate--ammonialigase		<1..848	Nitrogen Metabolism	COG0174
112.1_23	CUST_101_PI442190815	HOT208_60m_813102 112.1_23	AAAGTGATTCATCAAATCTTGATGCAACCCTTGAAATCAATATTCGTTCAGGTCAGGCCA	glnA	Glutamine synthetase/ glutamate--ammonialigase		<1..848	Nitrogen Metabolism	COG0174
112.1_23	CUST_102_PI442190815	HOT208_60m_813102 112.1_23	TGGAGAAAAGTGCCTGTTAATAATGAAATCTTAGGTCCTTTAGCTAAAGCAAATGCTCCA	glnA	Glutamine synthetase/ glutamate--ammonialigase		<1..848	Nitrogen Metabolism	COG0174
112.1_23	CUST_103_PI442190815	HOT208_60m_813102 112.1_23	AGGTAATATCAACTGGGCTAAAGCTTCAGAAACACATATAGATGATTTTGGGGAGAGTT	glnA	Glutamine synthetase/ glutamate--ammonialigase		<1..848	Nitrogen Metabolism	COG0174
112.1_23	CUST_104_PI442190815	HOT208_60m_813102 112.1_23	TTTATTTTGCCTCACTAAGTTCTCAAACAGTTGTTTACAAAGGTATGGTTCGCTCTGAAA	glnA	Glutamine synthetase/ glutamate--ammonialigase		<1..848	Nitrogen Metabolism	COG0174
148.1_WP_079330482.:	CUST_462_PI442190815	HOT208_60m_813102 148.1_WP_079330482.:	ACTTCTGCCAAGCAGTAACTTGTGCCAATGCAAAGATAACTCTAATTTCTCCGTTCGTTG	tal	Transaldolase	WP_079330482.:	22681..23682	Pentose Phosphate	COG0176
148.1_WP_079330482.:	CUST_463_PI442190815	HOT208_60m_813102 148.1_WP_079330482.:	AGATTGCTGCAACTTGGGAGGGAATTAAGGCAGCTGAAATTTTGGAAAAAGAGGGTATTA	tal	Transaldolase	WP_079330482.:	22681..23682	Pentose Phosphate	COG0176
148.1_WP_079330482.:	CUST_464_PI442190815	HOT208_60m_813102 148.1_WP_079330482.:	CTACAGAAGTTGATGCAAGACTTAGCTTTGACACCGAAGCTACGGTAGAAAAAGCGAGAA	tal	Transaldolase	WP_079330482.:	22681..23682	Pentose Phosphate	COG0176
148.1_WP_079330482.:	CUST_465_PI442190815	HOT208_60m_813102 148.1_WP_079330482.:	CATCTTGGATTGGCATAAAGCAAAAACTGGTAAAACCAGTTTTGTTGGTGCTGAAGACCC	tal	Transaldolase	WP_079330482.:	22681..23682	Pentose Phosphate	COG0176
148.1_WP_079330482.:	CUST_466_PI442190815	HOT208_60m_813102 148.1_WP_079330482.:	AGCAGGTTGCGATCTTTTGACAATTGCGCCAAAATTTCTTGAGGAACTGAAAAAAGAAAA	tal	Transaldolase	WP_079330482.:	22681..23682	Pentose Phosphate	COG0176
148.1_WP_079330482.:	CUST_467_PI442190815	HOT208_60m_813102 148.1_WP_079330482.:	TGGCAAGTGAAAAGCTTAGTGAAGGTATCACTGGGTTCAGTAAAGCTATAGAAGAATTGG	tal	Transaldolase	WP_079330482.:	22681..23682	Pentose Phosphate	COG0176
148.1_WP_025955299.:	CUST_824_PI442190815	HOT208_60m_813102 148.1_WP_025955299.:	CCAAAGCTGGAGTTATTGGATTTACCAAAACCTGCGCTAAAGAATTTGCCTCAAGAGGTA	gdh	3-oxoacyl-acyl-carrier-protein reductase	WP_025955299.:	79915..80664	Entner-Dudoroff	COG1028
148.1_WP_025955299.:	CUST_825_PI442190815	HOT208_60m_813102 148.1_WP_025955299.:	ATCACTGGAGCTAGCAGAGGAATTGGTAAAGAAATTGCTTTAGAACTAAGCCGATTGGGA	gdh	3-oxoacyl-acyl-carrier-protein reductase	WP_025955299.:	79915..80664	Entner-Dudoroff	COG1028
148.1_WP_025955299.:	CUST_826_PI442190815	HOT208_60m_813102 148.1_WP_025955299.:	TCAAGTGATGCAGGAAGTTATATTACAGGGCAAACAATCAGTATAGACGGGGGTATGAGT	gdh	3-oxoacyl-acyl-carrier-protein reductase	WP_025955299.:	79915..80664	Entner-Dudoroff	COG1028
148.1_WP_025955299.:	CUST_827_PI442190815	HOT208_60m_813102 148.1_WP_025955299.:	AGTGGTAGCATCGTAAATATTTCATCTGTTGTTGGCATAATTGGTAATCCTGGTCAAGCA	gdh	3-oxoacyl-acyl-carrier-protein reductase	WP_025955299.:	79915..80664	Entner-Dudoroff	COG1028
148.1_WP_025955299.:	CUST_828_PI442190815	HOT208_60m_813102 148.1_WP_025955299.:	TGCTGGAATTACTAGAGATGGACTATTGATGAGAATGAAATCGGAACAATGGGATGATGT	gdh	3-oxoacyl-acyl-carrier-protein reductase	WP_025955299.:	79915..80664	Entner-Dudoroff	COG1028
148.1_WP_025955299.:	CUST_829_PI442190815	HOT208_60m_813102 148.1_WP_025955299.:	TGCAATCGCTCCAGGTTTTATAGAAACAGAGATGACTGAAAAACTTAATACTGAAGAGAT	gdh	3-oxoacyl-acyl-carrier-protein reductase	WP_025955299.:	79915..80664	Entner-Dudoroff	COG1028
148.1_WP_079330590.:	CUST_865_PI442190815	HOT208_60m_813102 148.1_WP_079330590.:	TATCCAATAATTTGTGCAGAATTATGCGGCCCTTATCATGGAGGAATGAGAGCCTCCATA	ctaC/coxB	Cytochrome c oxidase subunit II	WP_079330590.:	87618..88421	Respiration	COG1622
148.1_WP_079330590.:	CUST_866_PI442190815	HOT208_60m_813102 148.1_WP_079330590.:	CATGCTTTTTGGGTCCCAGAGTTCAGAATTAAACAGGATATTATTCCTGGACAACCCACT	ctaC/coxB	Cytochrome c oxidase subunit II	WP_079330590.:	87618..88421	Respiration	COG1622
148.1_WP_079330590.:	CUST_867_PI442190815	HOT208_60m_813102 148.1_WP_079330590.:	TCCCAAGGGCAATTTCATATCAGGTGAACTACACGTTCCTGTTGATCAAAAAGTATCAAT	ctaC/coxB	Cytochrome c oxidase subunit II	WP_079330590.:	87618..88421	Respiration	COG1622
148.1_WP_079330590.:	CUST_868_PI442190815	HOT208_60m_813102 148.1_WP_079330590.:	TCTTTTTGGATTGGTTTTAATGTAAATTTGCTCCCAGCAGAAGCAAGTATTAATGCACCA	ctaC/coxB	Cytochrome c oxidase subunit II	WP_079330590.:	87618..88421	Respiration	COG1622
148.1_WP_079330590.:	CUST_869_PI442190815	HOT208_60m_813102 148.1_WP_079330590.:	AGCTACAACATCTACGATCGAATGGGAGGGATGAAAGAACTAAATCATAATCACGAAATG	ctaC/coxB	Cytochrome c oxidase subunit II	WP_079330590.:	87618..88421	Respiration	COG1622
148.1_WP_079330590.:	CUST_870_PI442190815	HOT208_60m_813102 148.1_WP_079330590.:	GGCGATGGTATAGCTTTAGAGGGAAATTTAAGCTTAGAAATTGTATGGACAATTATCCCT	ctaC/coxB	Cytochrome c oxidase subunit II	WP_079330590.:	87618..88421	Respiration	COG1622
148.1_WP_079330592.:	CUST_871_PI442190815	HOT208_60m_813102 148.1_WP_079330592.:	TTACACCTGAATGGTTAACTTCTTCTCCACCTCCAGTTGAAAATTGGGAAGGAGAAGCTC	cyoB/coxA	Cytochrome c oxidase subunit I	WP_079330592.:	88418..90052	Respiration	COG0843
148.1_WP_079330592.:	CUST_872_PI442190815	HOT208_60m_813102 148.1_WP_079330592.:	GAACGCCCCCTTGGATGAGATTATTCTTTACTATTGCCACAGCATTTATTGCTGTTCCAA	cyoB/coxA	Cytochrome c oxidase subunit I	WP_079330592.:	88418..90052	Respiration	COG0843
148.1_WP_079330592.:	CUST_873_PI442190815	HOT208_60m_813102 148.1_WP_079330592.:	CTCCTCAACATTGGCTTGGTTTAAATGGAATGCCCAGAAGAGTTGCAGAATATGATCCTC	cyoB/coxA	Cytochrome c oxidase subunit I	WP_079330592.:	88418..90052	Respiration	COG0843
148.1_WP_079330592.:	CUST_874_PI442190815	HOT208_60m_813102 148.1_WP_079330592.:	TGCTTTGGCACAGGTACCTTTTGATATTCACGTACATGATACCTATTTCGTAGTAGCCCA	cyoB/coxA	Cytochrome c oxidase subunit I	WP_079330592.:	88418..90052	Respiration	COG0843
148.1_WP_079330592.:	CUST_875_PI442190815	HOT208_60m_813102 148.1_WP_079330592.:	TTGGGTTGCAACATTATGGGGCGGTAAAATTTCCATCAATAGTGCAATGCTATTCTCTTG	cyoB/coxA	Cytochrome c oxidase subunit I	WP_079330592.:	88418..90052	Respiration	COG0843
148.1_WP_079330592.:	CUST_876_PI442190815	HOT208_60m_813102 148.1_WP_079330592.:	TAATTAATGTATTCCTCAGCGTGAGAAATGGGAAAGATGCTGGCGATAACCCTTGGAATG	cyoB/coxA	Cytochrome c oxidase subunit I	WP_079330592.:	88418..90052	Respiration	COG0843
148.1_WP_025880735.:	CUST_877_PI442190815	HOT208_60m_813102 148.1_WP_025880735.:	TTGGTCTTATAACTTTCCTAATTGCGGACGGAATGACTTTTGCTGGATTCTTTGCTGCTT	cyoC	heme-copper oxidase subunit III	WP_025880735.:	90049..90651	Respiration	COG1845
148.1_WP_025880735.:	CUST_878_PI442190815	HOT208_60m_813102 148.1_WP_025880735.:	TTGCTTGGCAGTCGAGAATCAATGGCGGAAGATTAACTAGTCAAAATATGTTCCCATTGG	cyoC	heme-copper oxidase subunit III	WP_025880735.:	90049..90651	Respiration	COG1845
148.1_WP_025880735.:	CUST_879_PI442190815	HOT208_60m_813102 148.1_WP_025880735.:	TGCAAGTACTTTCTATGCCCTTACTGGTTTTCATGGATTACATGTCACTTTAGGCACTTT	cyoC	heme-copper oxidase subunit III	WP_025880735.:	90049..90651	Respiration	COG1845
148.1_WP_025880735.:	CUST_880_PI442190815	HOT208_60m_813102 148.1_WP_025880735.:	AGTGCAACTTTCCATAAAGCAGGCAAAGCACTTTTAAAAGATAAAAACTCAGATTCCCAA	cyoC	heme-copper oxidase subunit III	WP_025880735.:	90049..90651	Respiration	COG1845
148.1_WP_025880735.:	CUST_881_PI442190815	HOT208_60m_813102 148.1_WP_025880735.:	AGCAGTAAATCCATTACCAGATGGTGCTATTTATGAATTAGAACTACCAATACCTACTCT	cyoC	heme-copper oxidase subunit III	WP_025880735.:	90049..90651	Respiration	COG1845
148.1_WP_025880735.:	CUST_882_PI442190815	HOT208_60m_813102 148.1_WP_025880735.:	AGCTCAAAAGAAATTCAGAAAAATAATTCCGAAGTTAATGAAACACATGAAGACTTCAGA	cyoC	heme-copper oxidase subunit III	WP_025880735.:	90049..90651	Respiration	COG1845
150.1_218	CUST_1124_PI442190815	HOT208_60m_813102 150.1_218	ATGTAAGGAAATACTTGATGGGCTTCACGATTCACAGTTTCCCCTAAAGGTTTGGCAAAC	fumC	class II fumarate hydratase		<1..300	Krebs cycle and related genes	COG0114
150.1_218	CUST_1125_PI442190815	HOT208_60m_813102 150.1_218	AATTGCGAATTTCAATTTAGGTTTAATAGATAAAAGGAAAAAAGATTTG	fumC	class II fumarate hydratase		<1..300	Krebs cycle and related genes	COG0114
150.1_218	CUST_1126_PI442190815	HOT208_60m_813102 150.1_218	TTCCAATTGAGTTAATTTATTCGCTCACCCTCATAAAAAAAGCTGCTTCAATTGCGAATT	fumC	class II fumarate hydratase		<1..300	Krebs cycle and related genes	COG0114
150.1_218	CUST_1127_PI442190815	HOT208_60m_813102 150.1_218	GAACAATAGAGGTTCCCATTGAAGCTTTGTGGGGCGCTCAAACACAAAGATCGATAATAA	fumC	class II fumarate hydratase		<1..300	Krebs cycle and related genes	COG0114
150.1_WP_032524993.:	CUST_1179_PI442190815	HOT208_60m_813102 150.1_WP_032524993.:	TACTAGTGCAATTCAGGCATTGATTTTTGCAACTTTAGCGGCCTACTACATTGGAGAAGC	atpI	ATP synthase subunit A	WP_032524993.:	7975..8700	Energy Metabolism	COG0356
150.1_WP_032524993.:	CUST_1180_PI442190815	HOT208_60m_813102 150.1_WP_032524993.:	AAAGGCCTTCAAAACTTGCTTGAGTTCCTCTGGGATTACATAAGAGATCTTGCTAGGACG	atpI	ATP synthase subunit A	WP_032524993.:	7975..8700	Energy Metabolism	COG0356
150.1_WP_032524993.:	CUST_1181_PI442190815	HOT208_60m_813102 150.1_WP_032524993.:	TACCAAGTGGAGAATTAGGAGCACCTACTGCTGATATCAATACGACTATAGCCTTGGCTT	atpI	ATP synthase subunit A	WP_032524993.:	7975..8700	Energy Metabolism	COG0356
150.1_WP_032524993.:	CUST_1182_PI442190815	HOT208_60m_813102 150.1_WP_032524993.:	TGCAGGTTTAAGCAATAAGGGTTGGAGATACTTCGAATACTATGTACACCCAACTCCGAT	atpI	ATP synthase subunit A	WP_032524993.:	7975..8700	Energy Metabolism	COG0356
150.1_WP_032524993.:	CUST_1183_PI442190815	HOT208_60m_813102 150.1_WP_032524993.:	AGAAGTTGGTCAACATCTTTATTGGAAAATTGGAAATATCAGACTACATGGGCAGGTTTT	atpI	ATP synthase subunit A	WP_032524993.:	7975..8700	Energy Metabolism	COG0356
150.1_WP_032524993.:	CUST_1184_PI442190815	HOT208_60m_813102 150.1_WP_032524993.:	ATGCTTCCTTTCAAAATTCTAGAGGACTTTACGAAACCTTTATCACTCTCTTTCAGGCTA	atpI	ATP synthase subunit A	WP_032524993.:	7975..8700	Energy Metabolism	COG0356
150.1_WP_011863563.:	CUST_1207_PI442190815	HOT208_60m_813102 150.1_WP_011863563.:	AGTTATTAAAGCAACCTCAATTCTCTCCGCTGAACCTTGCAGAACAAGTTGCAGTTGTTT	atpA	ATP synthase alpha subunit	WP_011863563.:	10722..12239	Energy Metabolism	COG0056
150.1_WP_011863563.:	CUST_1208_PI442190815	HOT208_60m_813102 150.1_WP_011863563.:	AGCTGCTTTTTCTCAATTTGCATCTGATCTTGATGAAGCAACTCAGCAACAACTTGAACG	atpA	ATP synthase alpha subunit	WP_011863563.:	10722..12239	Energy Metabolism	COG0056
150.1_WP_011863563.:	CUST_1209_PI442190815	HOT208_60m_813102 150.1_WP_011863563.:	TATTAATGTAGGTATTTCTGTTAGCCGTGTTGGGGGAGCCGCTCAGACAAAAGCAATTAA	atpA	ATP synthase alpha subunit	WP_011863563.:	10722..12239	Energy Metabolism	COG0056
150.1_WP_011863563.:	CUST_1210_PI442190815	HOT208_60m_813102 150.1_WP_011863563.:	TGAAACTCAGGCAGGGGACGTTTCGGCTTACATCCCAACTAATGTTATTTCAATTACAGA	atpA	ATP synthase alpha subunit	WP_011863563.:	10722..12239	Energy Metabolism	COG0056
150.1_WP_011863563.:	CUST_1211_PI442190815	HOT208_60m_813102 150.1_WP_011863563.:	GATTACTAGAAAGAGCAGCAAAACTTTCTGATGCTATGGGAGGAGGCTCTATGACAGCTC	atpA	ATP synthase alpha subunit	WP_011863563.:	10722..12239	Energy Metabolism	COG0056
150.1_WP_011863563.:	CUST_1212_PI442190815	HOT208_60m_813102 150.1_WP_011863563.:	GAACCTGCTGCTTTACAGTACTTAGCACCTTATACTGGTGCAGCAATTGCTGAACATTTT	atpA	ATP synthase alpha subunit	WP_011863563.:	10722..12239	Energy Metabolism	COG0056
150.1_WP_079330710.:	CUST_1269_PI442190815	HOT208_60m_813102 150.1_WP_079330710.:	ATTCAGAGAAAATTAAAGCCTTGAAAGAGGTTTCAAAGGCTGAGGCTAGGATTCAAGCGG	atpC	ATP synthase epsilon subunit	WP_079330710.:	21514..21918	Energy Metabolism	COG0355
150.1_WP_079330710.:	CUST_1270_PI442190815	HOT208_60m_813102 150.1_WP_079330710.:	TGCTGAAATTGGTTCTGAAATTAATGTTCAAAATGCTGAACAAGATCTTAAAGAAGCAAA	atpC	ATP synthase epsilon subunit	WP_079330710.:	21514..21918	Energy Metabolism	COG0355
150.1_WP_079330710.:	CUST_1271_PI442190815	HOT208_60m_813102 150.1_WP_079330710.:	TAAGGCTAAGAATGAATTCCCAGTGGAAATCAATAGCTCTTATGGAGGCTTCGCTGAAA	atpC	ATP synthase epsilon subunit	WP_079330710.:	21514..21918	Energy Metabolism	COG0355
150.1_WP_079330710.:	CUST_1272_PI442190815	HOT208_60m_813102 150.1_WP_079330710.:	CCAAGGCGAAGCTGAAGAAGTGATCCTTCCTAGTACTACTGGTCAACTTGGTATACTACC	atpC	ATP synthase epsilon subunit	WP_079330710.:	21514..21918	Energy Metabolism	COG0355
152.1_WP_079330836.:	CUST_1661_PI442190815	HOT208_60m_813102 152.1_WP_079330836.:	TCATGGTTGTGGTGGAATGGAAGCTTGGATGAATCGCCTGAAATCTTCGAATTTTTTACT	opcA	Glucose-6-P dehydrogenase subunit opcA	WP_079330836.:	21433..22734	Pentose Phosphate	COG3429
152.1_WP_079330836.:	CUST_1662_PI442190815	HOT208_60m_813102 152.1_WP_079330836.:	GGGTTAGATTGAAAAATTGGAGGGAATCATTGGCAATGATTTTTGATCCGCCCTCTAGGA	opcA	Glucose-6-P dehydrogenase subunit opcA	WP_079330836.:	21433..22734	Pentose Phosphate	COG3429
152.1_WP_079330836.:	CUST_1663_PI442190815	HOT208_60m_813102 152.1_WP_079330836.:	TGTGAATCAGTGGAGTGCATGAGACTTGAAGCAGGGGGAATGGCTAATATGGAATTAATA	opcA	Glucose-6-P dehydrogenase subunit opcA	WP_079330836.:	21433..22734	Pentose Phosphate	COG3429
152.1_WP_079330836.:	CUST_1664_PI442190815	HOT208_60m_813102 152.1_WP_079330836.:	AATCCCTTATCTTTGGGAAATCCAAGTATTCATTTAGGACAAGTTATTGGGTTGAGGCTT	opcA	Glucose-6-P dehydrogenase subunit opcA	WP_079330836.:	21433..22734	Pentose Phosphate	COG3429
152.1_WP_079330836.:	CUST_1665_PI442190815	HOT208_60m_813102 152.1_WP_079330836.:	TCATGGATTTGCGATAAGCTTGGTTGGTCATTTCTAAGAGTTGAAAGGGATATAGAATCA	opcA	Glucose-6-P dehydrogenase subunit opcA	WP_079330836.:	21433..22734	Pentose Phosphate	COG3429
152.1_WP_079330836.:	CUST_1666_PI442190815	HOT208_60m_813102 152.1_WP_079330836.:	ACATCAGAGTATGATGTAAGTAAATTGTTGGGAAGTAGTAGAGGTAATACCAGCCCTCTT	opcA	Glucose-6-P dehydrogenase subunit opcA	WP_079330836.:	21433..22734	Pentose Phosphate	COG3429
152.1_WP_079330838.:	CUST_1667_PI442190815	HOT208_60m_813102 152.1_WP_079330838.:	CGTTGATAACTGGCGTTGGCAAGGGGTTCCTTTTTATTTGAGAACAGGTAAAAGACTACC	zwf	glucose-6-phosphate dehydrogenase zwf	WP_079330838.:	22737..24260	Pentose Phosphate	COG0364
152.1_WP_079330838.:	CUST_1668_PI442190815	HOT208_60m_813102 152.1_WP_079330838.:	ACAAATGCTTGCTGTTACTGCTATGGAACCTCCTGGAAAGTTTGAACCAGAAGCAATAAG	zwf	glucose-6-phosphate dehydrogenase zwf	WP_079330838.:	22737..24260	Pentose Phosphate	COG0364
152.1_WP_079330838.:	CUST_1669_PI442190815	HOT208_60m_813102 152.1_WP_079330838.:	TAATGCTTTCTGACCCAACCCTATTTACTCGAAGTGATGAAGTAGAAGCAGCCTGGAAAC	zwf	glucose-6-phosphate dehydrogenase zwf	WP_079330838.:	22737..24260	Pentose Phosphate	COG0364
152.1_WP_079330838.:	CUST_1670_PI442190815	HOT208_60m_813102 152.1_WP_079330838.:	TCCTGCCCCAAATCAACTTATCCTTAGAATTCAGCCAAATGAAGGGGCTACTTTCAAATT	zwf	glucose-6-phosphate dehydrogenase zwf	WP_079330838.:	22737..24260	Pentose Phosphate	COG0364
152.1_WP_079330838.:	CUST_1671_PI442190815	HOT208_60m_813102 152.1_WP_079330838.:	ACTTGCTGACGAAAATGAACCATGGAATTGTTGCATAAGAGGTCAATATGGAGAAGGAGG	zwf	glucose-6-phosphate dehydrogenase zwf	WP_079330838.:	22737..24260	Pentose Phosphate	COG0364
152.1_WP_079330838.:	CUST_1672_PI442190815	HOT208_60m_813102 152.1_WP_079330838.:	GACCTGTTGAGATGGAATTTTCTTATGATGAATCATTTGGAGAACCCTCAGATGAGGGCT	zwf	glucose-6-phosphate dehydrogenase zwf	WP_079330838.:	22737..24260	Pentose Phosphate	COG0364
153.1_WP_079330982.:	CUST_2163_PI442190815	HOT208_60m_813102 153.1_WP_079330982.:	ATGCAAATTGCTCAGGAGATGGGGATCCTAAATCAAGGAGATTTAGTAGTCCAAACAGCA	pykF	Pyruvate kinase	WP_079330982.:	46991..48781	Glycolysis	COG0469
153.1_WP_079330982.:	CUST_2164_PI442190815	HOT208_60m_813102 153.1_WP_079330982.:	GCCACTTACCCAGTACGATCCCAAATGCTATTAGTGCAGCAGTAAGCAATATAGCTAGAC	pykF	Pyruvate kinase	WP_079330982.:	46991..48781	Glycolysis	COG0469
153.1_WP_079330982.:	CUST_2165_PI442190815	HOT208_60m_813102 153.1_WP_079330982.:	CTTGGCAACTACTACTGAGAGAAGTGTAGCGAGAAGATTGCAACTTGTTTGGGGAGTTAC	pykF	Pyruvate kinase	WP_079330982.:	46991..48781	Glycolysis	COG0469
153.1_WP_079330982.:	CUST_2166_PI442190815	HOT208_60m_813102 153.1_WP_079330982.:	CCCTTTAACTAAATCAGGCTCTACCGCTCGAAATGTAAGTAAGTTCAGACCACCAACACC	pykF	Pyruvate kinase	WP_079330982.:	46991..48781	Glycolysis	COG0469
153.1_WP_079330982.:	CUST_2167_PI442190815	HOT208_60m_813102 153.1_WP_079330982.:	AGAAAGATTGTATCAAGAGGAATTTCAATAGGGGAAGTCGGTGTTACAGGTAAAGCTAGA	pykF	Pyruvate kinase	WP_079330982.:	46991..48781	Glycolysis	COG0469
153.1_WP_079330982.:	CUST_2168_PI442190815	HOT208_60m_813102 153.1_WP_079330982.:	TGGTGACCTTATTACACTCCAGCTTAATGAAGGTGTTATATACATGGGACAAATTGAAGA	pykF	Pyruvate kinase	WP_079330982.:	46991..48781	Glycolysis	COG0469
153.1_WP_079331011.:	CUST_2209_PI442190815	HOT208_60m_813102 153.1_WP_079331011.:	ACGTTTATGCCCAAGCCTGTATTTAACGATAATGGAACTGGAATGCATGTTCACCAAAGT	glnA	Glutamine synthetase/ glutamate--ammonialigase	WP_079331011.:	56002..57423	Nitrogen Metabolism	COG0174
153.1_WP_079331011.:	CUST_2210_PI442190815	HOT208_60m_813102 153.1_WP_079331011.:	TGCTGGTGCCGGTCAACACGAGCTTGGTATGAAATTTGATTCATTAATAAATTCTGCTGA	glnA	Glutamine synthetase/ glutamate--ammonialigase	WP_079331011.:	56002..57423	Nitrogen Metabolism	COG0174
153.1_WP_079331011.:	CUST_2211_PI442190815	HOT208_60m_813102 153.1_WP_079331011.:	TCTGAGGGTAATAGATCAGCTGCGGTAAGAATACCTTTAACAGGACCAAGTCCTAAAGCT	glnA	Glutamine synthetase/ glutamate--ammonialigase	WP_079331011.:	56002..57423	Nitrogen Metabolism	COG0174
153.1_WP_079331011.:	CUST_2212_PI442190815	HOT208_60m_813102 153.1_WP_079331011.:	CAGGTGATGCACTCGCAAATCCTTACTTAGCATTCTCTGTAATGATGCTTGCTGGTATTG	glnA	Glutamine synthetase/ glutamate--ammonialigase	WP_079331011.:	56002..57423	Nitrogen Metabolism	COG0174
153.1_WP_079331011.:	CUST_2213_PI442190815	HOT208_60m_813102 153.1_WP_079331011.:	TCTCAAACTGCAAGATGGTATATCGGAGGCATACTTAAGCATGCTCCATCATTCTTAGCA	glnA	Glutamine synthetase/ glutamate--ammonialigase	WP_079331011.:	56002..57423	Nitrogen Metabolism	COG0174
153.1_WP_079331011.:	CUST_2214_PI442190815	HOT208_60m_813102 153.1_WP_079331011.:	GAGGTTCAACAACTAAGACAAAGGCCTCATCCTCATGAATTCTTCATGTATTACGATGCA	glnA	Glutamine synthetase/ glutamate--ammonialigase	WP_079331011.:	56002..57423	Nitrogen Metabolism	COG0174
158.1_550	CUST_2860_PI442190815	HOT208_60m_813102 158.1_550	TTAGTGGTGCACCTCACAGAGTTGTACCTGATCAAAATGTTCAAAATGTTTTTAGAGGAA	kaiC	circadian clock protein KaiC		<1..361	Circadian Rhythm	COG0467
158.1_550	CUST_2861_PI442190815	HOT208_60m_813102 158.1_550	AGAATTTATCATTACAAATAGTGGCCCAGAAATAAGGGATTCTTTTTCTAATTTTGAACA	kaiC	circadian clock protein KaiC		<1..361	Circadian Rhythm	COG0467
158.1_550	CUST_2862_PI442190815	HOT208_60m_813102 158.1_550	AGGTGAGATGGCACGAGCATTAAATGTTTTTAAAATGCGGGGATCATGGCATGATAAAAG	kaiC	circadian clock protein KaiC		<1..361	Circadian Rhythm	COG0467
158.1_550	CUST_2863_PI442190815	HOT208_60m_813102 158.1_550	TGGGAAGTCATTCTATAACTGATTCTCATATCTCAACAATTACAGACACCATATTGTTGC	kaiC	circadian clock protein KaiC		<1..361	Circadian Rhythm	COG0467
158.1_550	CUST_2864_PI442190815	HOT208_60m_813102 158.1_550	TGCTGTCACTGGTTATACAAAACAAGAGGAGATAGCAGGCTTTTTTACTAATACTGCGGA	kaiC	circadian clock protein KaiC		<1..361	Circadian Rhythm	COG0467
170.1_WP_011862568.:	CUST_3454_PI442190815	HOT208_60m_813102 170.1_WP_011862568.:	ATCGGATACGATGCTTACACTCAAAGTCAAGGAACAGCTTTTGTAGTTTTCCAAGGACGT	rbcS	Ribulose bisphosphate carboxylase small subunit	WP_011862568.:	6237..6578	Carbon fixation	COG4451
170.1_WP_011862568.:	CUST_3455_PI442190815	HOT208_60m_813102 170.1_WP_011862568.:	GGGGAAAAAGATCTTAACTTGATCGTGAGCGAATTAGAGGCATGCCATAGAGCATATCCT	rbcS	Ribulose bisphosphate carboxylase small subunit	WP_011862568.:	6237..6578	Carbon fixation	COG4451
170.1_WP_011862568.:	CUST_3456_PI442190815	HOT208_60m_813102 170.1_WP_011862568.:	AATTGCTCAAGGTTGGAGTCCAGTTATTGAGCATGTTCATCCAAGTGGATGTATGCAAAC	rbcS	Ribulose bisphosphate carboxylase small subunit	WP_011862568.:	6237..6578	Carbon fixation	COG4451
170.1_WP_011862568.:	CUST_3457_PI442190815	HOT208_60m_813102 170.1_WP_011862568.:	CTGGAAACATTCGGTTTCTTACCACCGATGACCCAGGAAGAAATATATGACCAAATTGCC	rbcS	Ribulose bisphosphate carboxylase small subunit	WP_011862568.:	6237..6578	Carbon fixation	COG4451
172.1_WP_079331374.:	CUST_3628_PI442190815	HOT208_60m_813102 172.1_WP_079331374.:	TCGCCTCCCCTGGTGAGGTTTTTGATAAAGTTATCAGTAATGCTCAAGAAGCAAAAGCTA	glmS	Glutamine--fructose-6-phosphatetransaminase(isomerizing)	WP_079331374.:	24005..25900	Amino Acid metabolism	COG0449
172.1_WP_079331374.:	CUST_3629_PI442190815	HOT208_60m_813102 172.1_WP_079331374.:	GGAAGACAGGTCTCAAATATAATTGACATCTGTGCAGGAATAGAAGTTGGAGTTGCAGCA	glmS	Glutamine--fructose-6-phosphatetransaminase(isomerizing)	WP_079331374.:	24005..25900	Amino Acid metabolism	COG0449
172.1_WP_079331374.:	CUST_3630_PI442190815	HOT208_60m_813102 172.1_WP_079331374.:	ACATGCAGCTGGATATCCTGCTGGAGAAATGAAACACGGTCCAATAGCTTTATTAGATAA	glmS	Glutamine--fructose-6-phosphatetransaminase(isomerizing)	WP_079331374.:	24005..25900	Amino Acid metabolism	COG0449
172.1_WP_079331374.:	CUST_3631_PI442190815	HOT208_60m_813102 172.1_WP_079331374.:	TCCGCCGTTACTGGAAGATCTCATAGAGCAACATAATAAATCTTCAGAAAAGCTAGCACA	glmS	Glutamine--fructose-6-phosphatetransaminase(isomerizing)	WP_079331374.:	24005..25900	Amino Acid metabolism	COG0449
172.1_WP_079331374.:	CUST_3632_PI442190815	HOT208_60m_813102 172.1_WP_079331374.:	TGCATCTCATAGAGGACTTGACGTGGATCAACCAAGAAATTTAGCTAAAAGTGTAACTGT	glmS	Glutamine--fructose-6-phosphatetransaminase(isomerizing)	WP_079331374.:	24005..25900	Amino Acid metabolism	COG0449
172.1_WP_079331374.:	CUST_3633_PI442190815	HOT208_60m_813102 172.1_WP_079331374.:	TTGCTCCTGAATGTAACGGAACTGAAATCTTTGATTATTTAATGAAAGTCCCTTCCTCCA	glmS	Glutamine--fructose-6-phosphatetransaminase(isomerizing)	WP_079331374.:	24005..25900	Amino Acid metabolism	COG0449
173.1_WP_079331443.:	CUST_4088_PI442190815	HOT208_60m_813102 173.1_WP_079331443.:	AGATATCGGAACAATTGGAGCATTCTTTGAGTCAAACCTTGCATTGACTGAGCAACCAAA	glgC	Glucose-1-phosphate adenylyltransferase	WP_079331443.:	23484..24779	Glycogen Metabolism	COG0448
173.1_WP_079331443.:	CUST_4089_PI442190815	HOT208_60m_813102 173.1_WP_079331443.:	ACGCCTCTTGGAGTTGGTGAAGGGACCACTGTAAAAAGAGCAATTCTTGATAAGAATACA	glgC	Glucose-1-phosphate adenylyltransferase	WP_079331443.:	23484..24779	Glycogen Metabolism	COG0448
173.1_WP_079331443.:	CUST_4090_PI442190815	HOT208_60m_813102 173.1_WP_079331443.:	TTGAAAGTGACTCGGTTCTTGAGGATACTCTTGTTATGGGAGCCGATTTCTTTGAATCCC	glgC	Glucose-1-phosphate adenylyltransferase	WP_079331443.:	23484..24779	Glycogen Metabolism	COG0448
173.1_WP_079331443.:	CUST_4091_PI442190815	HOT208_60m_813102 173.1_WP_079331443.:	TGGATTAAGTAAGGAGTCAGCTGCTGAGAAACCCTATTTAGCCTCTATGGGGATTTACGT	glgC	Glucose-1-phosphate adenylyltransferase	WP_079331443.:	23484..24779	Glycogen Metabolism	COG0448
173.1_WP_079331443.:	CUST_4092_PI442190815	HOT208_60m_813102 173.1_WP_079331443.:	ACGGATTTTGGTAAGGACATAATTCCAGAAGCCCTCAATAGAGGCGATACTCTTAAAAGT	glgC	Glucose-1-phosphate adenylyltransferase	WP_079331443.:	23484..24779	Glycogen Metabolism	COG0448
173.1_WP_079331443.:	CUST_4093_PI442190815	HOT208_60m_813102 173.1_WP_079331443.:	ACCCCCTTCTAAACTTGTAGATGCTCAAATTACTGATTCAATTGTTTGTGAAGGCACAAT	glgC	Glucose-1-phosphate adenylyltransferase	WP_079331443.:	23484..24779	Glycogen Metabolism	COG0448
173.1_WP_079331444.:	CUST_4094_PI442190815	HOT208_60m_813102 173.1_WP_079331444.:	AGAACCAATAGACTTCCTCAGAACCTTGTTCAGGCAATGAGAGACTGTTTTGGTTCTCAT	edd	phosphogluconate dehydrogenase (NADP(+)-dependent, decarboxyla	WP_079331444.:	24875..26293	Pentose Phosphate/Entner-Dudorof	COG2084
173.1_WP_079331444.:	CUST_4095_PI442190815	HOT208_60m_813102 173.1_WP_079331444.:	GCTAAAGTTGTTTCTTTATCCACAAAAGCTGGTATACCAGTCCCATGTCTATCCAGTACC	edd	phosphogluconate dehydrogenase (NADP(+)-dependent, decarboxyla	WP_079331444.:	24875..26293	Pentose Phosphate/Entner-Dudorof	COG2084
173.1_WP_079331444.:	CUST_4096_PI442190815	HOT208_60m_813102 173.1_WP_079331444.:	GCAGTATATAACTATGGTTTGAATATGCCGTCAATTGCTCAAATATGGAAGGTGGTTGC	edd	phosphogluconate dehydrogenase (NADP(+)-dependent, decarboxyla	WP_079331444.:	24875..26293	Pentose Phosphate/Entner-Dudorof	COG2084
173.1_WP_079331444.:	CUST_4097_PI442190815	HOT208_60m_813102 173.1_WP_079331444.:	TCAGATGGAATGAAACCTTTATTTGATGCGGTAGTTCTTGCCACAATAGCAAGCTATGCC	edd	phosphogluconate dehydrogenase (NADP(+)-dependent, decarboxyla	WP_079331444.:	24875..26293	Pentose Phosphate/Entner-Dudorof	COG2084
173.1_WP_079331444.:	CUST_4098_PI442190815	HOT208_60m_813102 173.1_WP_079331444.:	GGTTATGGACTGTTGTAAGTGCTCTAGAACTGGGGGTATCAGTCCCAACAATTTATGCAT	edd	phosphogluconate dehydrogenase (NADP(+)-dependent, decarboxyla	WP_079331444.:	24875..26293	Pentose Phosphate/Entner-Dudorof	COG2084
173.1_WP_079331444.:	CUST_4099_PI442190815	HOT208_60m_813102 173.1_WP_079331444.:	TGCTGAAGCTTATGACCTTATGAAGAGAGTCAAAGGTATGAACGGACAGCAAATGTCAGA	edd	phosphogluconate dehydrogenase (NADP(+)-dependent, decarboxyla	WP_079331444.:	24875..26293	Pentose Phosphate/Entner-Dudorof	COG2084
173.1_WP_011376344.:	CUST_4153_PI442190815	HOT208_60m_813102 173.1_WP_011376344.:	AGGTGAAGCGGAAGATGGTCATGGATTTGAAGGTGAACTTTCTACAGATATGCTTCTGAC	cbbA	Fructose-1,6-bisphosphate aldolase class II	WP_011376344.:	33559..34632	Glycolysis/Gluconeogenesis	COG0191
173.1_WP_011376344.:	CUST_4155_PI442190815	HOT208_60m_813102 173.1_WP_011376344.:	AGCAAGTTTGTCTTGATAGATACAAGCAGTTCTGGTGCGAAGGTCAAGCAAGTAAGATCA	cbbA	Fructose-1,6-bisphosphate aldolase class II	WP_011376344.:	33559..34632	Glycolysis/Gluconeogenesis	COG0191
173.1_WP_011376344.:	CUST_4156_PI442190815	HOT208_60m_813102 173.1_WP_011376344.:	AGCATTTGCTGATAAGGCAAACTTCGACCCAAGACATTTCAACAAGCCTGCTAGAAAATA	cbbA	Fructose-1,6-bisphosphate aldolase class II	WP_011376344.:	33559..34632	Glycolysis/Gluconeogenesis	COG0191
173.1_WP_011376344.:	CUST_4157_PI442190815	HOT208_60m_813102 173.1_WP_011376344.:	TAGGAAAGTCAACATTGATACTGATACAGACTTGCTTTCACTGCAGCGGTTAGGAGAGGC	cbbA	Fructose-1,6-bisphosphate aldolase class II	WP_011376344.:	33559..34632	Glycolysis/Gluconeogenesis	COG0191
173.1_WP_011376344.:	CUST_4158_PI442190815	HOT208_60m_813102 173.1_WP_011376344.:	AGCACTTCCAAATACCCACCTTGTAATGCATGGATCTAGTTCAGTTCCTCAAGAATGGTT	cbbA	Fructose-1,6-bisphosphate aldolase class II	WP_011376344.:	33559..34632	Glycolysis/Gluconeogenesis	COG0191
173.1_WP_079331453.:	CUST_4159_PI442190815	HOT208_60m_813102 173.1_WP_079331453.:	GTTAATGGAGCAGATTGCCCAACAAAGGCTGATCCTATGAAGGTTGCTGAAATGACAATA	fda	Fructose-1,6-bisphosphate aldolase class I	WP_079331453.:	34780..35847	Glycolysis/Gluconeogenesis	COG0191
173.1_WP_079331453.:	CUST_4160_PI442190815	HOT208_60m_813102 173.1_WP_079331453.:	CACTCATGCTTAAAGGCTTGGAAAGGAAGCGACGTTGAAGGAGGTCAAAAAGCATTAATA	fda	Fructose-1,6-bisphosphate aldolase class I	WP_079331453.:	34780..35847	Glycolysis/Gluconeogenesis	COG0191
173.1_WP_079331453.:	CUST_4161_PI442190815	HOT208_60m_813102 173.1_WP_079331453.:	TGCATCTGTTCCTGGAATAGTTTTCTTATCAGGTGGATTAAGCGAAGAAGCTGCTTCTGT	fda	Fructose-1,6-bisphosphate aldolase class I	WP_079331453.:	34780..35847	Glycolysis/Gluconeogenesis	COG0191
173.1_WP_079331453.:	CUST_4162_PI442190815	HOT208_60m_813102 173.1_WP_079331453.:	CCAGGTATAAAGGTCGATAAAGGCCTAAATCCACTTCCAGGAGGAGGAGATGTAGAAACT	fda	Fructose-1,6-bisphosphate aldolase class I	WP_079331453.:	34780..35847	Glycolysis/Gluconeogenesis	COG0191
173.1_WP_079331453.:	CUST_4163_PI442190815	HOT208_60m_813102 173.1_WP_079331453.:	CCTAGATGGGTTAGTTGAAAGAGCCGCAAAATATTATGAACAAGGTGCCAGATTTGCCAA	fda	Fructose-1,6-bisphosphate aldolase class I	WP_079331453.:	34780..35847	Glycolysis/Gluconeogenesis	COG0191
173.1_WP_079331453.:	CUST_4164_PI442190815	HOT208_60m_813102 173.1_WP_079331453.:	AGAGCTCAAGCAAACTCTGAAGCTTCAAAAGGTGCTTATATAGCAGGATCTCAACCTTCA	fda	Fructose-1,6-bisphosphate aldolase class I	WP_079331453.:	34780..35847	Glycolysis/Gluconeogenesis	COG0191
173.1_WP_025881193.:	CUST_4182_PI442190815	HOT208_60m_813102 173.1_WP_025881193.:	TTTTTTATGAAGGTTTGCATGGCGGGGTAGAAGGTGATGGATACAATGTTGCCTCTTATG	prk	Phosphoribulokinase	WP_025881193.:	38467..39363	Carbon Fixation	COG3954
173.1_WP_025881193.:	CUST_4183_PI442190815	HOT208_60m_813102 173.1_WP_025881193.:	GCTATGGCAGCAGCTCTTTCAAAAGGTGAAAATTTCTCTCATTTTGGTCCAGAGGCTAAT	prk	Phosphoribulokinase	WP_025881193.:	38467..39363	Carbon Fixation	COG3954
173.1_WP_025881193.:	CUST_4184_PI442190815	HOT208_60m_813102 173.1_WP_025881193.:	CCCACAATTGACACCTCTAATCCTTTCATATGCAGGAATATCCCAACTCCTGATGAGAGC	prk	Phosphoribulokinase	WP_025881193.:	38467..39363	Carbon Fixation	COG3954
173.1_WP_025881193.:	CUST_4185_PI442190815	HOT208_60m_813102 173.1_WP_025881193.:	CAAGACTTGGGACATCCTTAGAACCTGGACAATTTACTCCATGGGAAGATATTCCTGAGG	prk	Phosphoribulokinase	WP_025881193.:	38467..39363	Carbon Fixation	COG3954
173.1_WP_025881193.:	CUST_4186_PI442190815	HOT208_60m_813102 173.1_WP_025881193.:	ACGCAGAAAGAGGTTACTCAGCAGAAACCATTGTGGATACTATATTGAGAAGAATGCCTG	prk	Phosphoribulokinase	WP_025881193.:	38467..39363	Carbon Fixation	COG3954
173.1_WP_025881193.:	CUST_4187_PI442190815	HOT208_60m_813102 173.1_WP_025881193.:	TGTCAAGTCCAACAAGTATCGTTGTAAATGGAGGAAAAATGGGATTCGCAATGGAACTAA	prk	Phosphoribulokinase	WP_025881193.:	38467..39363	Carbon Fixation	COG3954
020.1_WP_079329973.:	CUST_5448_PI442190815	HOT208_60m_813102 020.1_WP_079329973.:	TGGTTCGATTTTGTATCCGATATCAAAATTAAATATCCAAGAATTAATTTAGGCTCCGCC	eda	2-dehydro-3-deoxyphosphogluconate aldolase	WP_079329973.:	57294..57920	Entner-Dudoroff	COG0800
020.1_WP_079329973.:	CUST_5449_PI442190815	HOT208_60m_813102 020.1_WP_079329973.:	ATTTTTCGATGATGAAATTTTGGGATAAAGATCTTTTCAATTATGCGCAGTCAAAAAGTT	eda	2-dehydro-3-deoxyphosphogluconate aldolase	WP_079329973.:	57294..57920	Entner-Dudoroff	COG0800
020.1_WP_079329973.:	CUST_5450_PI442190815	HOT208_60m_813102 020.1_WP_079329973.:	AGGGTATAAAGCAGTCGTTAATTGGGGATAAAGCTATCAAAAATAAAAAATTTGATCCAAA	eda	2-dehydro-3-deoxyphosphogluconate aldolase	WP_079329973.:	57294..57920	Entner-Dudoroff	COG0800
020.1_WP_079329973.:	CUST_5451_PI442190815	HOT208_60m_813102 020.1_WP_079329973.:	AGATAGTTCGATAGATATACTCAACTATAGAAATATTGATTTCATTGCTGCTGGAGGACT	eda	2-dehydro-3-deoxyphosphogluconate aldolase	WP_079329973.:	57294..57920	Entner-Dudoroff	COG0800
020.1_WP_079329973.:	CUST_5452_PI442190815	HOT208_60m_813102 020.1_WP_079329973.:	AGATTCTTTTTCGGAGTTCCTGAAAACTGAGTCTTTTTTTTACTTATAAAACCTGAAGA	eda	2-dehydro-3-deoxyphosphogluconate aldolase	WP_079329973.:	57294..57920	Entner-Dudoroff	COG0800
020.1_WP_079329973.:	CUST_5453_PI442190815	HOT208_60m_813102 020.1_WP_079329973.:	GATCTTAAGGAAGCGATAGATTTAAATTGCAAAATTATCAAATTTACCCAATAAACAGT	eda	2-dehydro-3-deoxyphosphogluconate aldolase	WP_079329973.:	57294..57920	Entner-Dudoroff	COG0800
206.1_1078	CUST_5623_PI442190815	HOT208_60m_813102 206.1_1078	ATGGTTTGCACTCAGATAATGGGTCTTGAATATGGAGTTTCAATGGCCAATTATAGCGGC	fumC	class II fumarate hydratase		<1..458	Krebs cycle and related genes	COG0114
206.1_1078	CUST_5624_PI442190815	HOT208_60m_813102 206.1_1078	ATTCCTCAAAATGAACCTGGAGTTCTATCATGCGGGTAAAGTGAATCCAACTCAATCGC	fumC	class II fumarate hydratase		<1..458	Krebs cycle and related genes	COG0114
206.1_1078	CUST_5625_PI442190815	HOT208_60m_813102 206.1_1078	ACTAATGTTAGTTACAGCCATAGTGCCAAAAGTTGGTTATGAAAAAGCAGCTGAAATTGC	fumC	class II fumarate hydratase		<1..458	Krebs cycle and related genes	COG0114
206.1_1078	CUST_5626_PI442190815	HOT208_60m_813102 206.1_1078	GGCACTTTGCAGACGAATGAATATAAGCCTCTTATTGGGTTTAATATTCTCACAAGT	fumC	class II fumarate hydratase		<1..458	Krebs cycle and related genes	COG0114
206.1_1078	CUST_5627_PI442190815	HOT208_60m_813102 206.1_1078	AGAAAACTTCAGAATAAAATTAGTTGATGGGATGGAACCTAATCAAAAAAAAATGAAGTT	fumC	class II fumarate hydratase		<1..458	Krebs cycle and related genes	COG0114
206.1_1078	CUST_5628_PI442190815	HOT208_60m_813102 206.1_1078	TGTGCATTATTTAAAATTTCAAATGATATAAAAATTCTATCTTCTGGTCCAAGATCAGGA	fumC	class II fumarate hydratase		<1..458	Krebs cycle and related genes	COG0114
022.1_WP_079330068.:	CUST_6674_PI442190815	HOT208_60m_813102 022.1_WP_079330068.:	GAAGAGTTGTCAATCTTTGGCAATGATTGGCCAACTAAAGATGGTACATGCGTGAGAGAC	galE	UDP-glucose 4-epimerase	WP_079330068.:	23657..24706	Galactose metabolism	COG1087
022.1_WP_079330068.:	CUST_6675_PI442190815	HOT208_60m_813102 022.1_WP_079330068.:	AGTTGGAGCTCATGATTCAGGGATCCTAGGAGAAGAACCCAAGAGTAATCCTACGAATTT	galE	UDP-glucose 4-epimerase	WP_079330068.:	23657..24706	Galactose metabolism	COG1087
022.1_WP_079330068.:	CUST_6676_PI442190815	HOT208_60m_813102 022.1_WP_079330068.:	GTGGCAGATAATAGCTTGGCTTGATGAGGTTGGAGACCTGTCAAGACAATAGAA	galE	UDP-glucose 4-epimerase	WP_079330068.:	23657..24706	Galactose metabolism	COG1087
022.1_WP_079330068.:	CUST_6677_PI442190815	HOT208_60m_813102 022.1_WP_079330068.:	AATAATATTTCTATTCCCTACAAATTTGTTGAGAGGAGAAAAGGGGACGCGGCTAGTGTG	galE	UDP-glucose 4-epimerase	WP_079330068.:	23657..24706	Galactose metabolism	COG1087
022.1_WP_079330068.:	CUST_6678_PI442190815	HOT208_60m_813102 022.1_WP_079330068.:	TGCCTCATTAAAGTCCGTCGAAGAATCAGTATCTAATCCATTGAAATATTGGGATGCAAA	galE	UDP-glucose 4-epimerase	WP_079330068.:	23657..24706	Galactose metabolism	COG1087
022.1_WP_079330068.:	CUST_6679_PI442190815	HOT208_60m_813102 022.1_WP_079330068.:	AGACGAAAATACTTTTCAAGAGCCCATTAATCCATATGGGAATACGAAAATGACAATCGA	galE	UDP-glucose 4-epimerase	WP_079330068.:	23657..24706	Galactose metabolism	COG1087
022.1_WP_079330072.:	CUST_6686_PI442190815	HOT208_60m_813102 022.1_WP_079330072.:	ACCGATAATAGGATAGGTTCAAAATTTCTTGATGCAGGGCCTGGATTTGGAGGTAGTTGT	ugd	UDP-glucose 6-dehydrogenase/nucleotide sugar dehydrogenase	WP_079330072.:	25975..27420	Amino sugar and nucleotide sugar r	COG1004
022.1_WP_079330072.:	CUST_6687_PI442190815	HOT208_60m_813102 022.1_WP_079330072.:	GGAGCTGATGCAATAGTTGTCCTCACAGAATGGCCTGAATATTCATCTTTAGATTGGGGA	ugd	UDP-glucose 6-dehydrogenase/nucleotide sugar dehydrogenase	WP_079330072.:	25975..27420	Amino sugar and nucleotide sugar r	COG1004
022.1_WP_079330072.:	CUST_6688_PI442190815	HOT208_60m_813102 022.1_WP_079330072.:	CCAGAAGTCGCAAATTTTTGGGAAAGTGTTGTTACTTTGAACAATTGGCATCAGCACAGA	ugd	UDP-glucose 6-dehydrogenase/nucleotide sugar dehydrogenase	WP_079330072.:	25975..27420	Amino sugar and nucleotide sugar r	COG1004
022.1_WP_079330072.:	CUST_6689_PI442190815	HOT208_60m_813102 022.1_WP_079330072.:	ATTGGAGTACTTTGTGAAGCAACTGGTGCAGATGTTAGAGAAGTAGCAAGAGCAATAGGT	ugd	UDP-glucose 6-dehydrogenase/nucleotide sugar dehydrogenase	WP_079330072.:	25975..27420	Amino sugar and nucleotide sugar r	COG1004
022.1_WP_079330072.:	CUST_6690_PI442190815	HOT208_60m_813102 022.1_WP_079330072.:	TGGAGTAGTGAACTTGCAAAATTAACCGCAAATGCCTTTCTAGCACAAAGAATTAGCTCA	ugd	UDP-glucose 6-dehydrogenase/nucleotide sugar dehydrogenase	WP_079330072.:	25975..27420	Amino sugar and nucleotide sugar r	COG1004
022.1_WP_079330072.:	CUST_6691_PI442190815	HOT208_60m_813102 022.1_WP_079330072.:	ACGATACGAGAGAATCTGCGGCAATAAGTATTTGTAAAGATCTTTTAGATGAAGGGGCTT	ugd	UDP-glucose 6-dehydrogenase/nucleotide sugar dehydrogenase	WP_079330072.:	25975..27420	Amino sugar and nucleotide sugar r	COG1004
230.1_1325	CUST_6875_PI442190815	HOT208_60m_813102 230.1_1325	TGATCCTGATGGTCAGGATGTAGCGGGTAACTTTGACTTGTCTGGTCTTATTGAGAGAAT	kaiC	circadian clock protein KaiC		<1..422	Circadian Rhythm	COG0467
230.1_1325	CUST_6876_PI442190815	HOT208_60m_813102 230.1_1325	TGATGACTACTGAAAGGGTGGATGATTACGGCCCGATTGCTAGATATGGTGTAGAAGAAT	kaiC	circadian clock protein KaiC		<1..422	Circadian Rhythm	COG0467
230.1_1325	CUST_6877_PI442190815	HOT208_60m_813102 230.1_1325	AGAGTTGCAATAGATTCAATAACTGCTGTCTTTCAACAGTATGATGCTATTTACGTTGTT	kaiC	circadian clock protein KaiC		<1..422	Circadian Rhythm	COG0467
230.1_1325	CUST_6878_PI442190815	HOT208_60m_813102 230.1_1325	AGAAATGCCGCAAGTTTTGGTTGGGATTTACAAGAATTAATTGATCAAAATAAACTTTTT	kaiC	circadian clock protein KaiC		<1..422	Circadian Rhythm	COG0467
230.1_1325	CUST_6879_PI442190815	HOT208_60m_813102 230.1_1325	TGTATCTGATAATGTAGTCTTATTAAGAAATGTTCTTGAATCAGAGAAGAGAAGAAGAAC	kaiC	circadian clock protein KaiC		<1..422	Circadian Rhythm	COG0467
230.1_1325	CUST_6880_PI442190815	HOT208_60m_813102 230.1_1325	TTGTTAGAAGGGAAATATTTAGATTGATAGCAAGATTAAAAGAAATAGGTGTGAC	kaiC	circadian clock protein KaiC		<1..422	Circadian Rhythm	COG0467
024.1_WP_079330093.:	CUST_6921_PI442190815	HOT208_60m_813102 024.1_WP_079330093.:	ACGATGGTTAAAGGGGTGCCACTGTTCTTACAATAAGGATTTTCAAGAGGATAAAGAGCCA	argH	Argininosuccinate lyase	WP_079330093.:	<1..1320	Amino Acid metabolism	COG0165
024.1_WP_079330093.:	CUST_6922_PI442190815	HOT208_60m_813102 024.1_WP_079330093.:	GCCACAGGAAGTAGCTTAATGCCGCAGAAAAAAATCCTGACGTTCCAGAATTGATAAGA	argH	Argininosuccinate lyase	WP_079330093.:	<1..1320	Amino Acid metabolism	COG0165
024.1_WP_079330093.:	CUST_6923_PI442190815	HOT208_60m_813102 024.1_WP_079330093.:	AGGAGCTGCAGCTTTAGCTGGAACAAAAATAAAAATAGATATAGGCACTTTACCGCGTTAGA	argH	Argininosuccinate lyase	WP_079330093.:	<1..1320	Amino Acid metabolism	COG0165
024.1_WP_079330093.:	CUST_6924_PI442190815	HOT208_60m_813102 024.1_WP_079330093.:	GCGAAGGTGGCACAGGTTTTGTTCAAGTAGAAAAAGAGGTAAATAATTGGCAAAAAAGGT	argH	Argininosuccinate lyase	WP_079330093.:	<1..1320	Amino Acid metabolism	COG0165
024.1_WP_079330093.:	CUST_6925_PI442190815	HOT208_60m_813102 024.1_WP_079330093.:	TGTTCCTTTTAGGACTGCTTATCAAGTTGTTGGTCAAATGGTTAAATATTGCTTGGAGAG	argH	Argininosuccinate lyase	WP_079330093.:	<1..1320	Amino Acid metabolism	COG0165
024.1_WP_079330093.:	CUST_6926_PI442190815	HOT208_60m_813102 024.1_WP_079330093.:	TGCAGTAAGTGATGAGATTTTTGTATAGAGTTTGTTTCTGCCTCTGCTTTGTTGATGTC	argH	Argininosuccinate lyase	WP_079330093.:	<1..1320	Amino Acid metabolism	COG0165
025.1_WP_079330103.:	CUST_7033_PI442190815	HOT208_60m_813102 025.1_WP_079330103.:	GGCGTTCAGTTTAATGTTCCTCTTCAGCGGAAGAGGATATTGGCAAGAATTGTTCGAATC	psaA	Photosystem I P700 chlorophyll A apoprotein A1 PsaA	WP_079330103.:	876..3179	Photosystem I	COG2885
025.1_WP_079330103.:	CUST_7034_PI442190815	HOT208_60m_813102 025.1_WP_079330103.:	TCCCTGATAAAGCTTCTTAGGATTTAGATTCCCTTGTATGGTCCTGGAAGAGGTGGTA	psaA	Photosystem I P700 chlorophyll A apoprotein A1 PsaA	WP_079330103.:	876..3179	Photosystem I	COG2885
025.1_WP_079330103.:	CUST_7035_PI442190815	HOT208_60m_813102 025.1_WP_079330103.:	TCAATAGTTATTTTCCACTTCTCCTGGAAAATGCAGAGTGATGTTTGGGGACTCACTGGT	psaA	Photosystem I P700 chlorophyll A apoprotein A1 PsaA	WP_079330103.:	876..3179	Photosystem I	COG2885
025.1_WP_079330103.:	CUST_7036_PI442190815	HOT208_60m_813102 025.1_WP_079330103.:	ACACAATAAACTTAAGGTTGCACCAACCATTCAACCCCGAGCTCATCTATTACTCAGGG	psaA	Photosystem I P700 chlorophyll A apoprotein A1 PsaA	WP_079330103.:	876..3179	Photosystem I	COG2885
025.1_WP_079330103.:	CUST_7037_PI442190815	HOT208_60m_813102 025.1_WP_079330103.:	TCGCACAAAGTTCTATTACTATCAATGGTTGGCTAAGAGATTTCCTCTGGGCACAAGCTT	psaA	Photosystem I P700 chlorophyll A apoprotein A1 PsaA	WP_079330103.:	876..3179	Photosystem I	COG2885
025.1_WP_079330103.:	CUST_7038_PI442190815	HOT208_60m_813102 025.1_WP_079330103.:	AAGTTTAGTAGAAGTTGGTGGAAAGGTTGCTATAGCCCCAATTCCATTAGGAACAGCTGA	psaA	Photosystem I P700 chlorophyll A apoprotein A1 PsaA	WP_079330103.:	876..3179	Photosystem I	COG2885
025.1_WP_079330105.:	CUST_7039_PI442190815	HOT208_60m_813102 025.1_WP_079330105.:	CCTTGTTGGCTGGAGAGATAAGCCAGTTGCACTTTCAATTGTTCAAGCTAGATTAGTTGG	psaB	Photosystem I P700 chlorophyll A apoprotein A2 PsaB	WP_079330105.:	3207..5435	Photosystem I	COG2885
025.1_WP_079330105.:	CUST_7041_PI442190815	HOT208_60m_813102 025.1_WP_079330105.:	CATGGGTAACTTTCTATTGGCATTGGAAACACCTAGCAATATGGCAGGGTAATGTAGCAC	psaB	Photosystem I P700 chlorophyll A apoprotein A2 PsaB	WP_079330105.:	3207..5435	Photosystem I	COG2885
025.1_WP_079330105.:	CUST_7042_PI442190815	HOT208_60m_813102 025.1_WP_079330105.:	GACGAGGAGGTACTTGTGATAGTTCATCTTGGGCTGGGCGCTATGTACTTAGCTATGTTCTGGG	psaB	Photosystem I P700 chlorophyll A apoprotein A2 PsaB	WP_079330105.:	3207..5435	Photosystem I	COG2885
025.1_WP_079330105.:	CUST_7043_PI442190815	HOT208_60m_813102 025.1_WP_079330105.:	TATCGGTCCTGCAGATTTCTTAGTGCATCATGCTATCGCTTTAGGTCTTCATACAACTGC	psaB	Photosystem I P700 chlorophyll A apoprotein A2 PsaB	WP_079330105.:	3207..5435	Photosystem I	COG2885
025.1_WP_079330105.:	CUST_7044_PI442190815	HOT208_60m_813102 025.1_WP_079330105.:	AATGATGTACGGCTTTAACGCTCTACTATCAGATCTACAAGTTCAGCAAGTCTTGCTGC	psaB	Photosystem I P700 chlorophyll A apoprotein A2 PsaB	WP_079330105.:	3207..5435	Photosystem I	COG2885
025.1_WP_079330121.:	CUST_7095_PI442190815	HOT208_60m_813102 025.1_WP_079330121.:	GGAAACACCTGTCTTTATGGAGCAACAGGTGGGAAATTATTTGCATTAGGAAATCGGGA	glsF	Ferredoxin-dependent glutamate synthase	WP_079330121.:	11838..15467	Nitrogen Metabolism	COG0069
025.1_WP_079330121.:	CUST_7096_PI442190815	HOT208_60m_813102 025.1_WP_079330121.:	AAGAAATAGTGGTGCTACAGCGGTAACTGAAGGAGCAGGTGATCATTGTTGTGAATACAT	glsF	Ferredoxin-dependent glutamate synthase	WP_079330121.:	11838..15467	Nitrogen Metabolism	COG0069

025.1_WP_079330121.:	CUST_7097_PI442190815	HOT208_60m_813102		025.1_WP_079330121.:	TCGCAGAACTTCACGGAAACACTGGCTTCAATGGCGAACTCAACTTAAATTTTAAAGGAT	glsF	Ferredoxin-dependent glutamate synthase	WP_079330121.:	11838..15467	Nitrogen Metabolism	COG0069
025.1_WP_079330121.:	CUST_7098_PI442190815	HOT208_60m_813102		025.1_WP_079330121.:	CACAGGAAGGAATATTGGTGCGGGCATGACTGGTGGAATAGCTTTTATAATCGATGAAAA	glsF	Ferredoxin-dependent glutamate synthase	WP_079330121.:	11838..15467	Nitrogen Metabolism	COG0069
025.1_WP_079330121.:	CUST_7099_PI442190815	HOT208_60m_813102		025.1_WP_079330121.:	TGCAGGACAAAGCTTTGGGGCCTTTTTATTGAAAGGAATGAATGTTCAATTAATCGGAGA	glsF	Ferredoxin-dependent glutamate synthase	WP_079330121.:	11838..15467	Nitrogen Metabolism	COG0069
025.1_WP_079330121.:	CUST_7100_PI442190815	HOT208_60m_813102		025.1_WP_079330121.:	ACAAGAGTACTTTCAAATTAATAGTTCCCCCAAGCGAAGAAGAAATGCTTGGCATAAAGA	glsF	Ferredoxin-dependent glutamate synthase	WP_079330121.:	11838..15467	Nitrogen Metabolism	COG0069
031.1_WP_079330199.:	CUST_8106_PI442190815	HOT208_60m_813102		031.1_WP_079330199.:	AGTGCATCTGATTTGCTTGAGGTTCTCTTACTGGCAAAAGAAATGGGACTTCTTGATCAA	ppc	Phosphoenolpyruvate carboxylase	WP_079330199.:	6041..8045	Krebs cycle and related genes	COG2352
031.1_WP_079330199.:	CUST_8107_PI442190815	HOT208_60m_813102		031.1_WP_079330199.:	TGCCTCTTTTTGAAACTGTGGAAGACCTTAAAAGAGCACCAGAAGTAATGGAAAAGTTGT	ppc	Phosphoenolpyruvate carboxylase	WP_079330199.:	6041..8045	Krebs cycle and related genes	COG2352
031.1_WP_079330199.:	CUST_8108_PI442190815	HOT208_60m_813102		031.1_WP_079330199.:	AGGAAACTTTTTCAGTTTTTAAAATGGTTAAGAGACTGCAGCAAGAATTTGGTAGCCGCA	ppc	Phosphoenolpyruvate carboxylase	WP_079330199.:	6041..8045	Krebs cycle and related genes	COG2352
031.1_WP_079330199.:	CUST_8109_PI442190815	HOT208_60m_813102		031.1_WP_079330199.:	GCAAGTTTAGATATTCGTCAAGAGAGTACAAGGCATAGTGACGCTATACAAGAGCTTACA	ppc	Phosphoenolpyruvate carboxylase	WP_079330199.:	6041..8045	Krebs cycle and related genes	COG2352
031.1_WP_079330199.:	CUST_8110_PI442190815	HOT208_60m_813102		031.1_WP_079330199.:	TGGCTCATTGACGAATTAAATACAAAAAGGCCTCTAATTCCATCTGATGTTAACTGGACA	ppc	Phosphoenolpyruvate carboxylase	WP_079330199.:	6041..8045	Krebs cycle and related genes	COG2352
093.1_1740	CUST_8949_PI442190815	HOT208_60m_813102		093.1_1740	GATATTTTCAAGATTCCATTATTCTCGCCACTGGAGCTACTGGTACGGGCAAAACAATGC	kaiC	circadian clock protein KaiC		<1..617	Circadian Rhythm	COG0467
093.1_1740	CUST_8950_PI442190815	HOT208_60m_813102		093.1_1740	TGGATGGAATAAGTGTGTTTGCCTTAGGAGCAATGAGGTTAACGCAGAGATCCTCAAATA	kaiC	circadian clock protein KaiC		<1..617	Circadian Rhythm	COG0467
093.1_1740	CUST_8951_PI442190815	HOT208_60m_813102		093.1_1740	AATGGCAATTGATTCTCTCTCTGCATTAGCCAGAGGTGTAAGTTTGAATGCATTTAGGCA	kaiC	circadian clock protein KaiC		<1..617	Circadian Rhythm	COG0467
093.1_1740	CUST_8952_PI442190815	HOT208_60m_813102		093.1_1740	ACTTTTTGCATATGAAGAATCTAGAGCTCAATTGCTTAGAAATGCTACTAGCTGGGGTAT	kaiC	circadian clock protein KaiC		<1..617	Circadian Rhythm	COG0467
093.1_1740	CUST_8953_PI442190815	HOT208_60m_813102		093.1_1740	TTTGTGCATATCCTGAATCAACTGGTTTAGAGGATCACTTACAAATAATAAAACGCAAA	kaiC	circadian clock protein KaiC		<1..617	Circadian Rhythm	COG0467
093.1_1740	CUST_8954_PI442190815	HOT208_60m_813102		093.1_1740	TGTTCTTGAGTCAGAGAAGAGAAGAAGAACTTTAGAAGTTTTAAAGTTAAGAGGTACTGT	kaiC	circadian clock protein KaiC		<1..617	Circadian Rhythm	COG0467
WP_012194568.1_12	CUST_67_PI442190936	MIT9211	LLII/III	WP_012194568.1_12	GAACAGGCTTTGCTCAAGTGTCTGCACAGTTGGAAAATTGGCAAAATCAGTTTTCTTCTT	argH	Argininosuccinate lyase	WP_012194568.:	14906..16294	Amino Acid metabolism	COG0165
WP_012194568.1_12	CUST_68_PI442190936	MIT9211	LLII/III	WP_012194568.1_12	TTGGTCTAGCGAAGAATTTTCGTTTGTAAAATTAACCGATCGATGCGCAACTGGTAGCAG	argH	Argininosuccinate lyase	WP_012194568.:	14906..16294	Amino Acid metabolism	COG0165
WP_012194568.1_12	CUST_69_PI442190936	MIT9211	LLII/III	WP_012194568.1_12	GCAATGTCTATTCTCTTAGAAGAGGGTTTGGAGTTTTCTTTAGATCGCCTGGGATCAGCC	argH	Argininosuccinate lyase	WP_012194568.:	14906..16294	Amino Acid metabolism	COG0165
WP_012194568.1_12	CUST_70_PI442190936	MIT9211	LLII/III	WP_012194568.1_12	GATGTACCTGAACTTGTTAGAGGAAAGTCAGGAAGAGTCTTTGGCCATCTCCAAGCTTTG	argH	Argininosuccinate lyase	WP_012194568.:	14906..16294	Amino Acid metabolism	COG0165
WP_012194568.1_12	CUST_71_PI442190936	MIT9211	LLII/III	WP_012194568.1_12	TTTGCTTAAGGATCTTTCTTTTGATCAATGGCAGGATATGCACCCTGCTTTTGATCAGGA	argH	Argininosuccinate lyase	WP_012194568.:	14906..16294	Amino Acid metabolism	COG0165
WP_012194568.1_12	CUST_72_PI442190936	MIT9211	LLII/III	WP_012194568.1_12	GGTCATGCTCAAAGGTCTGCCTCTTGCATATAACAAAGATTTTCAGGAAGATAAAGAGGC	argH	Argininosuccinate lyase	WP_012194568.:	14906..16294	Amino Acid metabolism	COG0165
WP_012194783.1_224	CUST_1229_PI442190936	MIT9211	LLII/III	WP_012194783.1_224	GAAGATACGACTATTGCTGACCTTTCTGTAGCCACTAGAGCCGGTCAAATAAAAACTGGG	eno	Enolase	WP_012194783.:	218269..219567	Glycolysis/Gluconeogenesis	COG0148
WP_012194783.1_224	CUST_1230_PI442190936	MIT9211	LLII/III	WP_012194783.1_224	GGAGCAAATACTTTTAGGGAGGCTCTTCGCATGGGAGCAGAAGTCTTTCATACTCTTAAG	eno	Enolase	WP_012194783.:	218269..219567	Glycolysis/Gluconeogenesis	COG0148
WP_012194783.1_224	CUST_1231_PI442190936	MIT9211	LLII/III	WP_012194783.1_224	TCTGAAAAAGGTTTATCTACTGCTGTTGGAGATGAAGGTGGTTTCGCACCAAATTTGCAA	eno	Enolase	WP_012194783.:	218269..219567	Glycolysis/Gluconeogenesis	COG0148
WP_012194783.1_224	CUST_1232_PI442190936	MIT9211	LLII/III	WP_012194783.1_224	CCAATAGTATCGATTGAAGATGGGCTCGCAGAAGATGATTGGGAAGGTTGGGGATTATTA	eno	Enolase	WP_012194783.:	218269..219567	Glycolysis/Gluconeogenesis	COG0148
WP_012194783.1_224	CUST_1233_PI442190936	MIT9211	LLII/III	WP_012194783.1_224	AGGTGAAAAGGTCCAACTGGTTGGCGATGATTTGTTTGTTACAAATACCAGTAGATTGCA	eno	Enolase	WP_012194783.:	218269..219567	Glycolysis/Gluconeogenesis	COG0148
WP_012194783.1_224	CUST_1234_PI442190936	MIT9211	LLII/III	WP_012194783.1_224	AAGATGAATTGGGATCAGAGGCTACTTATGCAGGGTCTAATGATTTAGGACCTCTTTGCG	eno	Enolase	WP_012194783.:	218269..219567	Glycolysis/Gluconeogenesis	COG0148
WP_012194897.1_339	CUST_1847_PI442190936	MIT9211	LLII/III	WP_012194897.1_339	TAGGAGAAGGTATTTGGTGCTTTGCGAAAGATATTGCCGATGCTTGTAGTTTTGCAGATG	ugd	UDP-glucose 6-dehydrogenase/nucleotide sugar dehydrogenase	WP_012194897.:	322093..323562	Amino sugar and nucleotide sugar r	COG1004
WP_012194897.1_339	CUST_1848_PI442190936	MIT9211	LLII/III	WP_012194897.1_339	TTCGATGCAGGTCTGCAATTTTGGCGAGTAGGAGATGGATCGGAAAGGATAAGTAATTAG	ugd	UDP-glucose 6-dehydrogenase/nucleotide sugar dehydrogenase	WP_012194897.:	322093..323562	Amino sugar and nucleotide sugar r	COG1004
WP_012194897.1_339	CUST_1849_PI442190936	MIT9211	LLII/III	WP_012194897.1_339	CATAGAATCTCAAGATTAGTGGTCAAGAAGCTTTTTGGCACTTTGTCCGGGAAAAGGGTG	ugd	UDP-glucose 6-dehydrogenase/nucleotide sugar dehydrogenase	WP_012194897.:	322093..323562	Amino sugar and nucleotide sugar r	COG1004
WP_012194897.1_339	CUST_1850_PI442190936	MIT9211	LLII/III	WP_012194897.1_339	GCTCAGAGAATAAGTTCAATAAATTCAATTGGCGCTTTATGTGAGGCCACTGGAGCAGAT	ugd	UDP-glucose 6-dehydrogenase/nucleotide sugar dehydrogenase	WP_012194897.:	322093..323562	Amino sugar and nucleotide sugar r	COG1004
WP_012194897.1_339	CUST_1851_PI442190936	MIT9211	LLII/III	WP_012194897.1_339	TAGGGAATCTTCGGCTATTAACATCGCAAAGGATTTGTTAGATGAAGGCGCTCAGCTATC	ugd	UDP-glucose 6-dehydrogenase/nucleotide sugar dehydrogenase	WP_012194897.:	322093..323562	Amino sugar and nucleotide sugar r	COG1004
WP_012194897.1_339	CUST_1852_PI442190936	MIT9211	LLII/III	WP_012194897.1_339	CTTGTTTATTTATCACGCTACTTTGGCTTACCTGAAGTGGCTGATTTTTGGGAGGGAGTG	ugd	UDP-glucose 6-dehydrogenase/nucleotide sugar dehydrogenase	WP_012194897.:	322093..323562	Amino sugar and nucleotide sugar r	COG1004
WP_012194911.1_353	CUST_1918_PI442190936	MIT9211	LLII/III	WP_012194911.1_353	ACCCTTTCAGGAGATATAAAGTTAAAGGAGCAATCAACAGAATCATCTGCCTCAA	minE	septum site-determining protein MinE	WP_012194911.:	334356..334667	Cell Division	COG0851
WP_012194911.1_353	CUST_1919_PI442190936	MIT9211	LLII/III	WP_012194911.1_353	TCTTGAAACAGAAGATAGGATGACAGCCCTGGTTGCGAATCTACCTATTAAGAGAACCCT	minE	septum site-determining protein MinE	WP_012194911.:	334356..334667	Cell Division	COG0851
WP_012194911.1_353	CUST_1920_PI442190936	MIT9211	LLII/III	WP_012194911.1_353	GATCTTTTAGACCAAATGAGGGAGGAGATCCTGAGCGTGGTAGCCAAGTATGTAGAGATT	minE	septum site-determining protein MinE	WP_012194911.:	334356..334667	Cell Division	COG0851
WP_012194911.1_353	CUST_1921_PI442190936	MIT9211	LLII/III	WP_012194911.1_353	CAGCAAGGGCGAGAGAACGTCTTCAGCTAGTGCTTGCACATGATAGAACTGATTTAAG	minE	septum site-determining protein MinE	WP_012194911.:	334356..334667	Cell Division	COG0851
WP_012194912.1_354	CUST_1922_PI442190936	MIT9211	LLII/III	WP_012194912.1_354	TCCTGCTGGAGTAGAGGATGGATTCAAAAATGCAGTTGCTGCATCAAAAGAAGCAATTAT	minD	septum site-determining protein MinD	WP_012194912.:	334672..335487	Cell Division	COG2894
WP_012194912.1_354	CUST_1923_PI442190936	MIT9211	LLII/III	WP_012194912.1_354	TCCTTCAAAAGAAGGTTCAACCCTAAGGGACAAGTTTCGCAGACTGATGCAAACAAAAAT	minD	septum site-determining protein MinD	WP_012194912.:	334672..335487	Cell Division	COG2894
WP_012194912.1_354	CUST_1924_PI442190936	MIT9211	LLII/III	WP_012194912.1_354	CAGGGAATCCTCGGATGCTTGATTGGCTTAAGCCTGATGATATGAAGCGAATAGTAGAAA	minD	septum site-determining protein MinD	WP_012194912.:	334672..335487	Cell Division	COG2894
WP_012194912.1_354	CUST_1925_PI442190936	MIT9211	LLII/III	WP_012194912.1_354	CCCAAGAAGTGCTTGAAGAGAATTGCAGACTAGATCAAGCACTCGTTAAGCACAAACAAC	minD	septum site-determining protein MinD	WP_012194912.:	334672..335487	Cell Division	COG2894
WP_012194912.1_354	CUST_1926_PI442190936	MIT9211	LLII/III	WP_012194912.1_354	AGAGATGCTATCGATTGATGACGTGACCGATATCCTTGCACTTCCATTACTAGGACTTGT	minD	septum site-determining protein MinD	WP_012194912.:	334672..335487	Cell Division	COG2894
WP_012194912.1_354	CUST_1927_PI442190936	MIT9211	LLII/III	WP_012194912.1_354	ATAGAGGAGAACCTCTTACACTTATTGGCAGCAATTCACCAGCAGCAAGATGCTATTCAA	minD	septum site-determining protein MinD	WP_012194912.:	334672..335487	Cell Division	COG2894
WP_012194913.1_355	CUST_1928_PI442190936	MIT9211	LLII/III	WP_012194913.1_355	GTCCCAGAAACAATTGTCAGTGCAGCTGCATTGGGCTTCATAAGTGTACTGAATATGAAA	minC	septum site-determining protein MinC	WP_012194913.:	335628..336269	Cell Division	COG0850
WP_012194913.1_355	CUST_1929_PI442190936	MIT9211	LLII/III	WP_012194913.1_355	TTCAGGTGAAATTCTAGAGGCGAATAGCGACTTATTGATTCACGGTGATGTAAACCCAGG	minC	septum site-determining protein MinC	WP_012194913.:	335628..336269	Cell Division	COG0850
WP_012194913.1_355	CUST_1930_PI442190936	MIT9211	LLII/III	WP_012194913.1_355	TGAGACCTGTTCAATTGAGAATCTCCAATAAAATTGCAAGAGGTCCCAAGGAGAGACCTG	minC	septum site-determining protein MinC	WP_012194913.:	335628..336269	Cell Division	COG0850
WP_012194913.1_355	CUST_1931_PI442190936	MIT9211	LLII/III	WP_012194913.1_355	GTGCAATTGTTTCTGCTGGTGGAGATGTAATGATTTGGGGAAGACTCCTTGGGATAG	minC	septum site-determining protein MinC	WP_012194913.:	335628..336269	Cell Division	COG0850
WP_012194913.1_355	CUST_1932_PI442190936	MIT9211	LLII/III	WP_012194913.1_355	AACACTCGAGAGAGGTTTTCTAGAGATCGACTGTGGTGATAATTTCCTTAACTCCAAAGA	minC	septum site-determining protein MinC	WP_012194913.:	335628..336269	Cell Division	COG0850
WP_012194913.1_355	CUST_1933_PI442190936	MIT9211	LLII/III	WP_012194913.1_355	TGCAACAAACAGCAAAAGACCAGTCATTCCTTCTAAATACAGAAAAGAGGAAAACATCT	minC	septum site-determining protein MinC	WP_012194913.:	335628..336269	Cell Division	COG0850
WP_012194995.1_438	CUST_2374_PI442190936	MIT9211	LLII/III	WP_012194995.1_438	AAAAACACATTTCCTCTAGAGGCAGCTGAACTCTATTGGCACTTCGTAGATGGAATCTGG	cyoC	heme-copper oxidase subunit III	WP_012194995.:	416909..417529	Respiration	COG1845
WP_012194995.1_438	CUST_2375_PI442190936	MIT9211	LLII/III	WP_012194995.1_438	TTTGCTGGATTTTTTGCAGCTTACTTGACCTTTAAAGCAGTCAACCCATTACTCCCTGAT	cyoC	heme-copper oxidase subunit III	WP_012194995.:	416909..417529	Respiration	COG1845
WP_012194995.1_438	CUST_2376_PI442190936	MIT9211	LLII/III	WP_012194995.1_438	TTCACTTTGCCTTTTGGCCTTACCGACAATTTATACGCGAGTACTTTCTACGCTCTTACT	cyoC	heme-copper oxidase subunit III	WP_012194995.:	416909..417529	Respiration	COG1845
WP_012194995.1_438	CUST_2377_PI442190936	MIT9211	LLII/III	WP_012194995.1_438	ACTATTTTGCTTCTTGTAAGTAGCGCAACATTTCACCGTGCTGGTAAAGCATTACAACGT	cyoC	heme-copper oxidase subunit III	WP_012194995.:	416909..417529	Respiration	COG1845
WP_012194995.1_438	CUST_2378_PI442190936	MIT9211	LLII/III	WP_012194995.1_438	GGTTTCCATGGACTTCATGTAACACTTGGGACTCTGATGATTTTGATTGTTTGGTGGCAA	cyoC	heme-copper oxidase subunit III	WP_012194995.:	416909..417529	Respiration	COG1845
WP_012194995.1_438	CUST_2379_PI442190936	MIT9211	LLII/III	WP_012194995.1_438	TCAACATGAGCACGAAGACCATAGATTATTTGGGCTTATAGCTTTTCTCGTTGCTGACGG	cyoC	heme-copper oxidase subunit III	WP_012194995.:	416909..417529	Respiration	COG1845
WP_012194996.1_439	CUST_2380_PI442190936	MIT9211	LLII/III	WP_012194996.1_439	CTCAAGTTCCTTTCGATATCCACGTTCATGATACCTACTTCGTAGTAGCCCACTTCCACT	cyoB/coxA	Cytochrome c oxidase subunit I	WP_012194996.:	417533..419191	Respiration	COG0843
WP_012194996.1_439	CUST_2381_PI442190936	MIT9211	LLII/III	WP_012194996.1_439	GCTAGGTCTTAATGGAATGCCAAGGCGTGTAGCTGAATATGATCCTCAATTTGCTTTTGT	cyoB/coxA	Cytochrome c oxidase subunit I	WP_012194996.:	417533..419191	Respiration	COG0843
WP_012194996.1_439	CUST_2382_PI442190936	MIT9211	LLII/III	WP_012194996.1_439	CCCTGGATGAGGCTCTTCTTTACAATTGCGACAGCATTTATAGCAGTACCAACAGGTATA	cyoB/coxA	Cytochrome c oxidase subunit I	WP_012194996.:	417533..419191	Respiration	COG0843
WP_012194996.1_439	CUST_2383_PI442190936	MIT9211	LLII/III	WP_012194996.1_439	CACTGGTATCCAAAATTCACTGGAAGAATGCTCAATGAGAATATTGGCCGGCTTCACTTC	cyoB/coxA	Cytochrome c oxidase subunit I	WP_012194996.:	417533..419191	Respiration	COG0843
WP_012194996.1_439	CUST_2384_PI442190936	MIT9211	LLII/III	WP_012194996.1_439	GGAATATTATATATAGCGCTTTTAAAGGCCCAATCGCTGGTGACAATCCTTGGCAAGCAT	cyoB/coxA	Cytochrome c oxidase subunit I	WP_012194996.:	417533..419191	Respiration	COG0843
WP_012194996.1_439	CUST_2385_PI442190936	MIT9211	LLII/III	WP_012194996.1_439	CCCCTCTAGTCACCAAGCCTTATGGTTATGGTCAGAAGCCACAAAAAGAAAAGAATCCTT	cyoB/coxA	Cytochrome c oxidase subunit I	WP_012194996.:	417533..419191	Respiration	COG0843
WP_012194997.1_440	CUST_2386_PI442190936	MIT9211	LLII/III	WP_012194997.1_440	GAGAGCTTCATGTGCCGATTGGACAACCAGTGACTATGAAGATGGAGTCAAAAGATGTTA	ctaC/coxB	Cytochrome c oxidase subunit II	WP_012194997.:	419188..420063	Respiration	COG1622
WP_012194997.1_440	CUST_2387_PI442190936	MIT9211	LLII/III	WP_012194997.1_440	GTTTCGTCTCAAGCAAGACGTGATCCCTGGTCAACCAACAATATTAAATTTCACACCGAC	ctaC/coxB	Cytochrome c oxidase subunit II	WP_012194997.:	419188..420063	Respiration	COG1622
WP_012194997.1_440	CUST_2388_PI442190936	MIT9211	LLII/III	WP_012194997.1_440	GCTATGACATATATGAGCGAATGGGTGGGATGGAATCACTAGGGGCTAATAATCAACACC	ctaC/coxB	Cytochrome c oxidase subunit II	WP_012194997.:	419188..420063	Respiration	COG1622
WP_012194997.1_440	CUST_2389_PI442190936	MIT9211	LLII/III	WP_012194997.1_440	TTATTCAATTTCGAAGGAAACCAGGACAAACAGGTGATGGCCTTGCAATAGAAGACAACC	ctaC/coxB	Cytochrome c oxidase subunit II	WP_012194997.:	419188..420063	Respiration	COG1622
WP_012194997.1_440	CUST_2390_PI442190936	MIT9211	LLII/III	WP_012194997.1_440	TTTGGGTAGGCAGCAACGTGAATCTACTTCCTGTTGTTGCTAGTTCTAATGCTCCAATAT	ctaC/coxB	Cytochrome c oxidase subunit II	WP_012194997.:	419188..420063	Respiration	COG1622
WP_012194997.1_440	CUST_2391_PI442190936	MIT9211	LLII/III	WP_012194997.1_440	TCAAATGGCTTCTAAACAAAGAATTTGGGGTGGCATTGGCTCTAAAGACAAGTCTATGCA	ctaC/coxB	Cytochrome c oxidase subunit II	WP_012194997.:	419188..420063	Respiration	COG1622
WP_012195008.1_450	CUST_2437_PI442190936	MIT9211	LLII/III	WP_012195008.1_450	TTCTTGCCACAGACCCAGCAGCAGCTTATATAACTGGACAAGTTTTACAAGTAGATGGAG	gdh	3-oxoacyl-acyl-carrier-protein reductase	WP_012195008.:	427659..428411	Entner-Dudoroff	COG1028
WP_012195008.1_450	CUST_2438_PI442190936	MIT9211	LLII/III	WP_012195008.1_450	TAGCAAGACCCATGCTTAAGCAGAAAACAGGTCGGATTGTAAATATCACTTCCGTCGTAG	gdh	3-oxoacyl-acyl-carrier-protein reductase	WP_012195008.:	427659..428411	Entner-Dudoroff	COG1028
WP_012195008.1_450	CUST_2439_PI442190936	MIT9211	LLII/III	WP_012195008.1_450	CTAACAAAAAGCACAGCCAAAGAGTTTGCCAGTAGAGGCATAACAGTTAATGCAGTAGCA	gdh	3-oxoacyl-acyl-carrier-protein reductase	WP_012195008.:	427659..428411	Entner-Dudoroff	COG1028
WP_012195008.1_450	CUST_2440_PI442190936	MIT9211	LLII/III	WP_012195008.1_450	TCAGAACAAATCCTCTCTGCAATTCCACTAGGTGAATTTGGAAGTCCAGAGCATATTGCT	gdh	3-oxoacyl-acyl-carrier-protein reductase	WP_012195008.:	427659..428411	Entner-Dudoroff	COG1028
WP_012195008.1_450	CUST_2441_PI442190936	MIT9211	LLII/III	WP_012195008.1_450	ACAGAAGATTGGCAGTCAGTTATTAATCTCAACTTGACCGGGGTCTTCCTTTGTACAAGG	gdh	3-oxoacyl-acyl-carrier-protein reductase	WP_012195008.:	427659..428411	Entner-Dudoroff	COG1028
WP_012195008.1_450	CUST_2442_PI442190936	MIT9211	LLII/III	WP_012195008.1_450	TTAGTAACTGGAGCCAGTAGAGGTATTGGCGAGCAATTGCTTTAAATCTTGCTACAACA	gdh	3-oxoacyl-acyl-carrier-protein reductase	WP_012195008.:	427659..428411	Entner-Dudoroff	COG1028
WP_012195075.1_518	CUST_2813_PI442190936	MIT9211	LLII/III	WP_012195075.1_518	ATTGATGACTTGGAGATTAGGCTGCTTGAAAGGATTGCCATCCTTGAAGAAGGTAAGTCT	tal	Transaldolase	WP_012195075.:	488681..489700	Pentose Phosphate	COG0176
WP_012195075.1_518	CUST_2814_PI442190936	MIT9211	LLII/III	WP_012195075.1_518	GGTATAAAGCAAATACGGGGATAGCCAATTATCCAGGCCCTGAAGATCCAGGAGTAATTT	tal	Transaldolase	WP_012195075.:	488681..489700	Pentose Phosphate	COG0176
WP_012195075.1_518	CUST_2815_PI442190936	MIT9211	LLII/III	WP_012195075.1_518	CCTGGAACGTGAAGGAATTCACTGTAACCTCACTTTGCTATTTAATTTCTGTCAAGCGGC	tal	Transaldolase	WP_012195075.:	488681..489700	Pentose Phosphate	COG0176
WP_012195075.1_518	CUST_2816_PI442190936	MIT9211	LLII/III	WP_012195075.1_518	TATGGGTGCCAGCTTTAGAAATATTGAAGAGATTGTTGAATTGGCTGGATTGCGATTTGCT	tal	Transaldolase	WP_012195075.:	488681..489700	Pentose Phosphate	COG0176
WP_012195075.1_518	CUST_2817_PI442190936	MIT9211	LLII/III	WP_012195075.1_518	GTAGGGTCTCAACTGAGGTTGATGCTCGTCTTAGCTTTGATACTCAAGCAACAGTTGAAA	tal	Transaldolase	WP_012195075.:	488681..489700	Pentose Phosphate	COG0176
WP_012195075.1_518	CUST_2818_PI442190936	MIT9211	LLII/III	WP_012195075.1_518	TGAAAAGAAGCTTGACTCTTTGAATCCTAAGCCTGTTGGGCAAAAGATTGACATTGACCA	tal	Transaldolase	WP_012195075.:	488681..489700	Pentose Phosphate	COG0176
WP_012195105.1_549	CUST_2989_PI442190936	MIT9211	LLII/III	WP_012195105.1_549	CCCTTGAGACCTGGAAAGAGATCAAGTTTGAGTTCGATACGGTTGACAAGTTAGACGTTC	rbcL	Ribulose 1,5-bisphosphate carboxylase large subunit	WP_012195105.:	517060..518472	Carbon fixation	COG1850
WP_012195105.1_549	CUST_2990_PI442190936	MIT9211	LLII/III	WP_012195105.1_549	TTGTGTTAAAGCACGTAATGCCGGTAGAGAGATTGAGAAAGAAAGCCGTGACATCTTGAT	rbcL	Ribulose 1,5-bisphosphate carboxylase large subunit	WP_012195105.:	517060..518472	Carbon fixation	COG1850
WP_012195105.1_549	CUST_2991_PI442190936	MIT9211	LLII/III	WP_012195105.1_549	TACAGGAACTGTTGTTGGAAAACTAGAAGGTGATCGCCAGACCACTCTTGGCTATATAGA	rbcL	Ribulose 1,5-bisphosphate carboxylase large subunit	WP_012195105.:	517060..518472	Carbon fixation	COG1850
WP_012195105.1_549	CUST_2992_PI442190936	MIT9211	LLII/III	WP_012195105.1_549	ATGCCCATAATCATGCATGACTACATAACAGGTGGCTTTACTGCTAATACAGGACTAGCC	rbcL	Ribulose 1,5-bisphosphate carboxylase large subunit	WP_012195105.:	517060..518472	Carbon fixation	COG1850
WP_012195105.1_549	CUST_2993_PI442190936	MIT9211	LLII/III	WP_012195105.1_549	CAAGCTGGCTCAACAAGAAACTGGTGAAGTTAAAGGTCATTATCTTAACTGCACAGCGAC	rbcL	Ribulose 1,5-bisphosphate carboxylase large subunit	WP_012195105.:	517060..518472	Carbon fixation	COG1850
WP_012195105.1_549	CUST_2994_PI442190936	MIT9211	LLII/III	WP_012195105.1_549	AACATAAACTCACAACCTTTCCAGAGATGGAGAGAGCGTTTCGAATTTGTAGCTGAAGCT	rbcL	Ribulose 1,5-bisphosphate carboxylase large subunit	WP_012195105.:	517060..518472	Carbon fixation	COG1850
WP_012195106.1_550	CUST_2995_PI442190936	MIT9211	LLII/III	WP_012195106.1_550	CATGTTCGAATGGTTGGTTACGACGCTTATACCCAAAGTCAGGGCACTTGCTTC	rbcS	Ribulose bisphosphate carboxylase small subunit	WP_012195106.:	518572..518913	Carbon fixation	COG4451
WP_012195106.1_550	CUST_2996_PI442190936	MIT9211	LLII/III	WP_012195106.1_550	GAGAGAAGGATTTGAATGTTGTAGTTAGTGAGCTAGAAGCTTGTCATCGCGCATACCCTG	rbcS	Ribulose bisphosphate carboxylase small subunit	WP_012195106.:	518572..518913	Carbon fixation	COG4451
WP_012195106.1_550	CUST_2997_PI442190936	MIT9211	LLII/III	WP_012195106.1_550	GAACACGTTCATCCAAGTGGCTCTATGCAGACCTACTGGTCTTATTGGAAGTTACCTTTC	rbcS	Ribulose bisphosphate carboxylase small subunit	WP_012195106.:	518572..518913	Carbon fixation	COG4451
WP_012195106.1_550	CUST_2998_PI442190936	MIT9211	LLII/III	WP_012195106.1_550	CTTCTTACCGCCGATGACCCAGGACGAAATTTACGACCAAATTGCTTACATCATTGCTC	rbcS	Ribulose bisphosphate carboxylase small subunit	WP_012195106.:	518572..518913	Carbon fixation	COG4451
WP_012195106.1_550	CUST_2999_PI442190936	MIT9211	LLII/III	WP_012195106.1_550	ATGCCTTTCCAGAGCACAGTTGGTGACTATCAGACAGTTGCCACCCTGGAAACATTC	rbcS	Ribulose bisphosphate carboxylase small subunit	WP_012195106.:	518572..518913	Carbon fixation	COG4451
WP_012195268.1_698	CUST_3661_PI442190936	MIT9211	LLII/III	WP_012195268.1_698	GCAGATGTCGTCAAGGAAGGTAAGGATTTGACATTGCTTACATACTCCCGAATGAGACAT	pdhB	Pyruvate dehydrogenase E1 component beta subunit	WP_012195268.:	625463..626446	Glycolysis/Krebs cycle	COG0022
WP_012195268.1_698	CUST_3662_PI442190936	MIT9211	LLII/III	WP_012195268.1_698	GTATTTCCATGCAGTCCCTGGGATAAAGATAGTTGCTTGCAGTACACCTACTAATGCGAA	pdhB	Pyruvate dehydrogenase E1 component beta subunit	WP_012195268.:	625463..626446	Glycolysis/Krebs cycle	COG0022
WP_012195268.1_698	CUST_3663_PI442190936	MIT9211	LLII/III	WP_012195268.1_698	CAATGGTCAACTTGAGAACCTAACGATTATTCAACCCCACCAAATTGTTGAATCGGCAGA	pdhB	Pyruvate dehydrogenase E1 component beta subunit	WP_012195268.:	625463..626446	Glycolysis/Krebs cycle	COG0022
WP_012195268.1_698	CUST_3664_PI442190936	MIT9211	LLII/III	WP_012195268.1_698	GCACTCCCTCAACTGGAGGAGAAAGGAATTGATGCAGAATTGATTGATTTAATCAGCCTG	pdhB	Pyruvate dehydrogenase E1 component beta subunit	WP_012195268.:	625463..626446	Glycolysis/Krebs cycle	COG0022
WP_012195268.1_698	CUST_3665_PI442190936	MIT9211	LLII/III	WP_012195268.1_698	CAATATGGGGATGTTGAGGTATACCAGTGGAGGTAATTTCACTATCCCGACTGTTGTTCG	pdhB	Pyruvate dehydrogenase E1 component beta subunit	WP_012195268.:	625463..626446	Glycolysis/Krebs cycle	COG0022
WP_012195268.1_698	CUST_3666_PI442190936	MIT9211	LLII/III	WP_012195268.1_698	GGCTTCGCCCAATTGTGGAGGGAATGAATATGGGATTTCTTCTTCTTGCCTTTAATCAAA	pdhB	Pyruvate dehydrogenase E1 component beta subunit	WP_012195268.:	625463..626446	Glycolysis/Krebs cycle	COG0022
WP_012195400.1_830	CUST_4352_PI442190936	MIT9211	LLII/III	WP_012195400.1_830	CTCTTGTGGTCCAAACAGCTGGCACAATAACTGGAGTAAGTGGATCAACTGACCTAATAA	pykF	Pyruvate kinase	WP_012195400.:	741389..743131	Glycolysis	COG0469
WP_012195400.1_830	CUST_4353_PI442190936	MIT9211	LLII/III	WP_012195400.1_830	GTTGCCCGTGGAAATTCTGAAGGCAATAATTCTGTTTGTGGGAAAGTGAGAATTGCAAAG	pykF	Pyruvate kinase	WP_012195400.:	741389..743131	Glycolysis	COG0469
WP_012195400.1_830	CUST_4354_PI442190936	MIT9211	LLII/III	WP_012195400.1_830	AGAAACTACAACTAAAACATTTAACTTAGCCATGGAGATGGCCCAGGGTATGGGCTTACT	pykF	Pyruvate kinase	WP_012195400.:	741389..743131	Glycolysis	COG0469
WP_012195400.1_830	CUST_4355_PI442190936	MIT9211	LLII/III	WP_012195400.1_830	GCCATCTACCAAGCACCATTCCAAATGCAATTAGTGCTGCTGTTAGCTCTATATCAAGAC	pykF	Pyruvate kinase	WP_012195400.:	741389..743131	Glycolysis	COG0469
WP_012195400.1_830	CUST_4356_PI442190936	MIT9211	LLII/III	WP_012195400.1_830	AAGCAAATTCAGGCCTCCAACCCCTATAGTTGCTGTAACTAATGAAACTGATGTTGCAAG	pykF	Pyruvate kinase	WP_012195400.:	741389..743131	Glycolysis	COG0469
WP_012195400.1_830	CUST_4357_PI442190936	MIT9211	LLII/III	WP_012195400.1_830	GGTGCTACAGATATCCTAAGAGATGGTGATATGGTCACACTAGTCCTAAAGGAAGGGACT	pykF	Pyruvate kinase	WP_012195400.:	741389..743131	Glycolysis	COG0469
WP_012195427.1_857	CUST_4498_PI442190936	MIT9211	LLII/III	WP_012195427.1_857	CAATGGCCTGGTATTCATCAGGATCAGCAAAAGGTTTGAAAGATATGGTTGTACTGCCAT	pgi	Glucose-6-phosphate isomerase	WP_012195427.:	767200..768798	Glycolysis/Gluconeogenesis	COG0166
WP_012195427.1_857	CUST_4499_PI442190936	MIT9211	LLII/III	WP_012195427.1_857	CTGGAGTCGAGGCAGGTAAGAAGGCAGCAGCTGATATATTGAATCTACAGCTACAAATTG	pgi	Glucose-6-phosphate isomerase	WP_012195427.:	767200..768798	Glycolysis/Gluconeogenesis	COG0166
WP_012195427.1_857	CUST_4500_PI442190936	MIT9211	LLII/III	WP_012195427.1_857	AATCGGCAGAGCTTAACAATTACGCTAAAAACATTTGACCCTCTTAGCTTAGGTGCCTTG	pgi	Glucose-6-phosphate isomerase	WP_012195427.:	767200..768798	Glycolysis/Gluconeogenesis	COG0166
WP_012195427.1_857	CUST_4501_PI442190936	MIT9211	LLII/III	WP_012195427.1_857	ATAATAAGTTACCTGGTGATTACCTTTCTGGGTTTATGCAAGGTACGCGCTTGGCACTTA	pgi	Glucose-6-phosphate isomerase	WP_012195427.:	767200..768798	Glycolysis/Gluconeogenesis	COG0166
WP_012195427.1_857	CUST_4502_PI442190936	MIT9211	LLII/III	WP_012195427.1_857	TCAGGGATTATCAGTATATGGGAACAAAGGCTCTACTGACCAGCATGCATATGTACAACA	pgi	Glucose-6-phosphate isomerase	WP_012195427.:	767200..768798	Glycolysis/Gluconeogenesis	COG0166
WP_012195427.1_857	CUST_4503_PI442190936	MIT9211	LLII/III	WP_012195427.1_857	GGTGCCTCCAAAATGGATGAACTTACCCGAAATAATGAATTATTAGATAATCCAGCAGCC	pgi	Glucose-6-phosphate isomerase	WP_012195427.:	767200..768798	Glycolysis/Gluconeogenesis	COG0166
WP_012195528.1_958	CUST_5047_PI442190936	MIT9211	LLII/III	WP_012195528.1_958	CAGGAGGTGGGAAAAAAAGATATTACCTTCACAGCCAAGAAGAAGCCGATGAACACAATG	prk	Phosphoribulokinase	WP_012195528.:	868171..869073	Carbon Fixation	COG3954
WP_012195528.1_958	CUST_5048_PI442190936	MIT9211	LLII/III	WP_012195528.1_958	CGCTTGCAAAAGGAGAGAACTTTTCTCATTTTGGACCTGAGGCAAACTTGTTTGACAAAC	prk	Phosphoribulokinase	WP_012195528.:	868171..869073	Carbon Fixation	COG3954
WP_012195528.1_958	CUST_5049_PI442190936	MIT9211	LLII/III	WP_012195528.1_958	CTGATGTACTCTTCTACGAGGGTCTACATGGTGGTGTAGTTGGAGATGGTTATGACGTTG	prk	Phosphoribulokinase	WP_012195528.:	868171..869073	Carbon Fixation	COG3954
WP_012195528.1_958	CUST_5050_PI442190936	MIT9211	LLII/III	WP_012195528.1_958	TTCATGTCAAGTCCTACAAGCATTGTAGTCAATGGAGGGAAAATGGGTTTTGCAATGGAA	prk	Phosphoribulokinase	WP_012195528.:	868171..869073	Carbon Fixation	COG3954
WP_012195528.1_958	CUST_5051_PI442190936	MIT9211	LLII/III	WP_012195528.1_958	TGCCCACAATTTAGCCGTACAGATATTAACTTCCAACGTGTACCAACGATAGATACATCT	prk	Phosphoribulokinase	WP_012195528.:	868171..869073	Carbon Fixation	COG3954
WP_012195528.1_958	CUST_5052_PI442190936	MIT9211	LLII/III	WP_012195528.1_958	GCATCCCTCGCTGATCTTCTGGTTGGAGTAGTACCTATTACAAACTTAGAATGGATACA	prk	Phosphoribulokinase	WP_012195528.:	868171..869073	Carbon Fixation	COG3954
WP_012195532.1_962	CUST_5071_PI442190936	MIT9211	LLII/III	WP_012195532.1_962	AATCAAGCAACAAAGCACCAATTACTATGCTGACCTTTATGGGAAAGGAGCATTAGAGCC	cbbA	Fructose-1,6-bisphosphate aldolase class II	WP_012195532.:	872174..873247	Glycolysis/Gluconeogenesis	COG0191
WP_012195532.1_962	CUST_5072_PI442190936	MIT9211	LLII/III	WP_012195532.1_962	CTCTGGCTATAGCTATTGGAACAAGCCATGGAGCATACAAATTCACAAGAAAGCCAACAG	cbbA	Fructose-1,6-bisphosphate aldolase class II	WP_012195532.:	872174..873247	Glycolysis/Gluconeogenesis	COG0191
WP_012195532.1_962	CUST_5073_PI442190936	MIT9211	LLII/III	WP_012195532.1_962	CAAAAGATATGTTGCTTACAGACCCAGCGGAGGCTGCTGATTTTGTTCAAAAAACCAAAG	cbbA	Fructose-1,6-bisphosphate aldolase class II	WP_012195532.:	872174..873247	Glycolysis/Gluconeogenesis	COG0191
WP_012195532.1_962	CUST_5074_PI442190936	MIT9211	LLII/III	WP_012195532.1_962	CCAAACACTCATCTAGTGATGCATGGCTCTAGTTCAGTGCCTCAAGAATGGCTAGATATG	cbbA	Fructose-1,6-bisphosphate aldolase class II	WP_012195532.:	872174..873247	Glycolysis/Gluconeogenesis	COG0191
WP_012195532.1_962	CUST_5075_PI442190936	MIT9211	LLII/III	WP_012195532.1_962	TACAAGTAACAAAGACTGTAGTTGATTTTGCCCACTCAGTTGGAGTCAGTGTTGAGGGTG	cbbA	Fructose-1,6-bisphosphate aldolase class II	WP_012195532.:	872174..873247	Glycolysis/Gluconeogenesis	COG0191
WP_012195532.1_962	CUST_5076_PI442190936	MIT9211	LLII/III	WP_012195532.1_962	TGAATATTGATACTGATAATAGGTTGGCTTATTTTACTGCAGCTGTAAGAGAGGCTGCGGCTG	cbbA	Fructose-1,6-bisphosphate aldolase class II	WP_012195532.:	872174..873247	Glycolysis/Gluconeogenesis	COG0191
WP_012195544.1_974	CUST_5135_PI442190936	MIT9211	LLII/III	WP_012195544.1_974	CGCACTAAAGATTCAGTTGACGGCACGGATCTTGTAGAAAAAATCATGGATAAAGCAGGG	edd	phosphogluconate dehydrogenase (NADP(+)-dependent, decarboxyla	WP_012195544.:	880680..882098	Pentose Phosphate/Entner-Dudorof	COG2084
WP_012195544.1_974	CUST_5136_PI442190936	MIT9211	LLII/III	WP_012195544.1_974	GCTTTGGAGTTAGGTGCTTCCGTTCCTACAATTTATGCATCACTAAATGCACGAGTGATG	edd	phosphogluconate dehydrogenase (NADP(+)-dependent, decarboxyla	WP_012195544.:	880680..882098	Pentose Phosphate/Entner-Dudorof	COG2084
WP_012195544.1_974	CUST_5137_PI442190936	MIT9211	LLII/III	WP_012195544.1_974	GAACTTGGCACTCCTTCAGATGGGATGTCTCCAATAATGGATGCAGTAGTATTGTCACA	edd	phosphogluconate dehydrogenase (NADP(+)-dependent, decarboxyla	WP_012195544.:	880680..882098	Pentose Phosphate/Entner-Dudorof	COG2084
WP_012195544.1_974	CUST_5138_PI442190936	MIT9211	LLII/III	WP_012195544.1_974	TATTAGATTCCTGGTTCCTCAATCAAGTTGACAAACGAATTGCTGGATTGGCAAAGGTGG	edd	phosphogluconate dehydrogenase (NADP(+)-dependent, decarboxyla	WP_012195544.:	880680..882098	Pentose Phosphate/Entner-Dudorof	COG2084
WP_012195544.1_974	CUST_5139_PI442190936	MIT9211	LLII/III	WP_012195544.1_974	GGTGATCAGATGGCTGACGTCTTTGCATATTGGAATAGCACAGAAGAACTGTCCTCTTAT	edd	phosphogluconate dehydrogenase (NADP(+)-dependent, decarboxyla	WP_012195544.:	880680..882098	Pentose Phosphate/Entner-Dudorof	COG2084
WP_012195544.1_974	CUST_5140_PI442190936	MIT9211	LLII/III	WP_012195544.1_974	TGCGTGATTGTTTCGGCTCTCATACTTATGAACGGACAGATAAGGAGGGTTTTTTCCATA	edd	phosphogluconate dehydrogenase (NADP(+)-dependent, decarboxyla	WP_012195544.:	880680..882098	Pentose Phosphate/Entner-Dudorof	COG2084
WP_012195545.1_975	CUST_5141_PI442190936	MIT9211	LLII/III	WP_012195545.1_975	ACAGGTGATTCCCTTAAGGCTATGGCTGTAGATACATCTAGGTTTGGTCTAACTGCTCAA	glgC	Glucose-1-phosphate adenylyltransferase	WP_012195545.:	882260..883555	Glycogen Metabolism	COG0448
WP_012195545.1_975	CUST_5142_PI442190936	MIT9211	LLII/III	WP_012195545.1_975	GTTGGTGAAGGATCCATTTTAAAGGCTTGTAGCATTCATCATTGTGTATTGGGCGTCAGA	glgC	Glucose-1-phosphate adenylyltransferase	WP_012195545.:	882260..883555	Glycogen Metabolism	COG0448
WP_012195545.1_975	CUST_5143_PI442190936	MIT9211	LLII/III	WP_012195545.1_975	TACCTCTGGGAGTCGGTCAAGGAACTACTGTAAAAAGAGCAATTCTTGATAAAACGCCC	glgC	Glucose-1-phosphate adenylyltransferase	WP_012195545.:	882260..883555	Glycogen Metabolism	COG0448
WP_012195545.1_975	CUST_5144_PI442190936	MIT9211	LLII/III	WP_012195545.1_975	GTTATGGGTTCCGATTTTTACGAATCAGGAGAAGAGAGAATTGCCCTAAGATCTGGTGGG	glgC	Glucose-1-phosphate adenylyltransferase	WP_012195545.:	882260..883555	Glycogen Metabolism	COG0448
WP_012195545.1_975	CUST_5145_PI442190936	MIT9211	LLII/III	WP_012195545.1_975	TATAGGAACTATTGGCGCATTCTTTGAGTCAAATCTTGCACTTACTCAACAGCCAAAGCC	glgC	Glucose-1-phosphate adenylyltransferase	WP_012195545.:	882260..883555	Glycogen Metabolism	COG0448
WP_012195545.1_975	CUST_5146_PI442190936	MIT9211	LLII/III	WP_012195545.1_975	AAGACTTTGGCAAGGAAGTTATACCCGAAGCTCTTAACCGTGGAGATGTATTGAAGAGTT	glgC	Glucose-1-phosphate adenylyltransferase	WP_012195545.:	882260..883555	Glycogen Metabolism	COG0448
WP_012195579.1_1008	CUST_5332_PI442190936	MIT9211	LLII/III	WP_012195579.1_1008	CAACATGAGTTGGGCATGAAATTTGCTTCGCTAATAAATGCTGCTGACAACGTAATGACC	glnA	Glutamine synthetase/ glutamate--ammonialigase	WP_012195579.:	918609..920030	Nitrogen Metabolism	COG0174
WP_012195579.1_1008	CUST_5333_PI442190936	MIT9211	LLII/III	WP_012195579.1_1008	AGCAAAGAAGTATGGAAAGACTGCGACTTTTATGCCTAAGCCAGTTTGGAATGACAATGG	glnA	Glutamine synthetase/ glutamate--ammonialigase	WP_012195579.:	918609..920030	Nitrogen Metabolism	COG0174
WP_012195579.1_1008	CUST_5334_PI442190936	MIT9211	LLII/III	WP_012195579.1_1008	CTTTCAAAAATATCAACAGTTCCATCTTCGTTAAACGACGCTCTTGATGCCCTCAAGGCA	glnA	Glutamine synthetase/ glutamate--ammonialigase	WP_012195579.:	918609..920030	Nitrogen Metabolism	COG0174
WP_012195579.1_1008	CUST_5335_PI442190936	MIT9211	LLII/III	WP_012195579.1_1008	CCAAGTCCAAAAGCAAAAAGGCTTGAATTCAGGTCTGGAGATGCTCTTGCTAATCCTTAT	glnA	Glutamine synthetase/ glutamate--ammonialigase	WP_012195579.:	918609..920030	Nitrogen Metabolism	COG0174
WP_012195579.1_1008	CUST_5336_PI442190936	MIT9211	LLII/III	WP_012195579.1_1008	TGCATGTGCATCAAAGTTTATGGAAAAATGGTCAGCCTCTATTTTATGGTGAGGGGACAT	glnA	Glutamine synthetase/ glutamate--ammonialigase	WP_012195579.:	918609..920030	Nitrogen Metabolism	COG0174
WP_012195579.1_1008	CUST_5337_PI442190936	MIT9211	LLII/III	WP_012195579.1_1008	ATAGTTACAAGAGGTTAGTCCCAGGATTCGAAGCACCAGTGAATTTGGTTTACTCCCAAG	glnA	Glutamine synthetase/ glutamate--ammonialigase	WP_012195579.:	918609..920030	Nitrogen Metabolism	COG0174
WP_012195606.1_1035	CUST_5487_PI442190936	MIT9211	LLII/III	WP_012195606.1_1035	TTGATGAACTTCCGATAATGGCATTAACAGATAAAGAGGTTGGCCTATTTTGCGCAGCAT	glk	Glucokinase	WP_012195606.:	949451..950491	Glycolysis	COG0837
WP_012195606.1_1035	CUST_5488_PI442190936	MIT9211	LLII/III	WP_012195606.1_1035	TTTACAAGAACTATGCTATGGAGGCTTATGGATTGGTGGAGGAACTGCTTCAAAGCATCT	glk	Glucokinase	WP_012195606.:	949451..950491	Glycolysis	COG0837
WP_012195606.1_1035	CUST_5489_PI442190936	MIT9211	LLII/III	WP_012195606.1_1035	CAAGTGAAGGAGGTCATAAAGAATTTGCACCGCGAACTCAAAATGAATGGGAACTAACCA	glk	Glucokinase	WP_012195606.:	949451..950491	Glycolysis	COG0837
WP_012195606.1_1035	CUST_5490_PI442190936	MIT9211	LLII/III	WP_012195606.1_1035	GAAAAACTTTGCAGTTATAGGAGCAGGAACTGGTCTTGGCATCGCAAGAGGTATTACAAC	glk	Glucokinase	WP_012195606.:	949451..950491	Glycolysis	COG0837
WP_012195606.1_1035	CUST_5491_PI442190936	MIT9211	LLII/III	WP_012195606.1_1035	ACTGGACTAGGAAATATAGCTCGATGGAGACTTATGAAACAAGACTCAAAATCGCACCCT	glk	Glucokinase	WP_012195606.:	949451..950491	Glycolysis	COG0837
WP_012195606.1_1035	CUST_5492_PI442190936	MIT9211	LLII/III	WP_012195606.1_1035	TCCCATAATGAATGAAGTATTAAATATTTGGCTTAGCGCCTATGGATCAGTAATTGGGGA	glk	Glucokinase	WP_012195606.:	949451..950491	Glycolysis	COG0837
WP_012195666.1_1093	CUST_5818_PI442190936	MIT9211	LLII/III	WP_012195666.1_1093	GTTTACCAATATGAAGCTAGAACATGGGGACCAGCCGAATCTGATGCCCTATTAGCTAAA	zwf	glucose-6-phosphate dehydrogenase zwf	WP_012195666.:	1016525..1018048	Pentose Phosphate	COG0364
WP_012195666.1_1093	CUST_5819_PI442190936	MIT9211	LLII/III	WP_012195666.1_1093	TTTAAGAATCCAACCAAATGAGGGGGCTGAATTTCGATTTGAGGTTAAATCCCCAGGTTC	zwf	glucose-6-phosphate dehydrogenase zwf	WP_012195666.:	1016525..1018048	Pentose Phosphate	COG0364
WP_012195666.1_1093	CUST_5820_PI442190936	MIT9211	LLII/III	WP_012195666.1_1093	ATTCTCAAGGTGGAAATAAAAATAGCCCGATTCTTGGATACAGACAGGAACCAGGAGTGG	zwf	glucose-6-phosphate dehydrogenase zwf	WP_012195666.:	1016525..1018048	Pentose Phosphate	COG0364
WP_012195666.1_1093	CUST_5821_PI442190936	MIT9211	LLII/III	WP_012195666.1_1093	GCGATCCAACACTGTTTACCAGAAGTGATGAAGTAGAAGCAGCATGGAGGTTATATACCC	zwf	glucose-6-phosphate dehydrogenase zwf	WP_012195666.:	1016525..1018048	Pentose Phosphate	COG0364
WP_012195666.1_1093	CUST_5822_PI442190936	MIT9211	LLII/III	WP_012195666.1_1093	GGCAAGGAGTTCCTTTTTACGTTCGAACTGGTAAAAGGTTAGCCAAAAGGACAAGTGAAG	zwf	glucose-6-phosphate dehydrogenase zwf	WP_012195666.:	1016525..1018048	Pentose Phosphate	COG0364
WP_012195666.1_1093	CUST_5823_PI442190936	MIT9211	LLII/III	WP_012195666.1_1093	GAAAGCGCAGGTGCTCTAAGAGATATGGTACAGAATCATCTGACTCAAATGCTTGCGATA	zwf	glucose-6-phosphate dehydrogenase zwf	WP_012195666.:	1016525..1018048	Pentose Phosphate	COG0364
WP_012195667.1_1094	CUST_5824_PI442190936	MIT9211	LLII/III	WP_012195667.1_1094	TTGATATTGAAGGCGATCATCCTGTTCAGGCTCTTTTATTCGGCAACATGGATTGCAGAT	opcA	Glucose-6-P dehydrogenase subunit opcA	WP_012195667.:	1018080..1019387	Pentose Phosphate	COG3429
WP_012195667.1_1094	CUST_5825_PI442190936	MIT9211	LLII/III	WP_012195667.1_1094	AAAATAGCCACACCAATTCGCAGGTTGATAATTGACAGTGCCCTAGGAAATCCCTTTAAC	opcA	Glucose-6-P dehydrogenase subunit opcA	WP_012195667.:	1018080..1019387	Pentose Phosphate	COG3429
WP_012195667.1_1094	CUST_5826_PI442190936	MIT9211	LLII/III	WP_012195667.1_1094	GTCCGTGATGACAAAGAGATAGTCAAATTCAAATTAATGCCATTGCCCGTAGGCAATCCA	opcA	Glucose-6-P dehydrogenase subunit opcA	WP_012195667.:	1018080..1019387	Pentose Phosphate	COG3429
WP_012195667.1_1094	CUST_5827_PI442190936	MIT9211	LLII/III	WP_012195667.1_1094	TATTATTCTTGCTTCTGAATCAGGAGAATGTATGAGACTTGAGGCAGGAGGCAGTGGCAAG	opcA	Glucose-6-P dehydrogenase subunit opcA	WP_012195667.:	1018080..1019387	Pentose Phosphate	COG3429
WP_012195667.1_1094	CUST_5828_PI442190936	MIT9211	LLII/III	WP_012195667.1_1094	AGTATCCACCCTGGCCAAATTATTGGTATAAGGTTGATATCCAAACCTAAAGAGGAGCCT	opcA	Glucose-6-P dehydrogenase subunit opcA	WP_012195667.:	1018080..1019387	Pentose Phosphate	COG3429
WP_012195667.1_1094	CUST_5829_PI442190936	MIT9211	LLII/III	WP_012195667.1_1094	TATGGAGCTTTTAGAGGAAGTAGTCCCACACCAAAAAAACTCTGCTGAGATGGATGTCG	opcA	Glucose-6-P dehydrogenase subunit opcA	WP_012195667.:	1018080..1019387	Pentose Phosphate	COG3429
WP_012195700.1_1126	CUST_6005_PI442190936	MIT9211	LLII/III	WP_012195700.1_1126	TGTATCCTCAACCTAAAAAACAAGTGGCTACAGAAGGTGCTCAAGGTGGTTACGTAATCC	rbsK	Sugar kinase, ribokinase family	WP_012195700.:	1051194..1052036	Pentose Phosphate	COG0524
WP_012195700.1_1126	CUST_6006_PI442190936	MIT9211	LLII/III	WP_012195700.1_1126	GATAATGGTCTTAAGTTACATGTTGCCTGGAAAGATGAGCCGACCAGGAAAGGATTAGT	rbsK	Sugar kinase, ribokinase family	WP_012195700.:	1051194..1052036	Pentose Phosphate	COG0524
WP_012195700.1_1126	CUST_6007_PI442190936	MIT9211	LLII/III	WP_012195700.1_1126	TTAAAGGTCTTCCCAAGCCAGGAGATATTATTCACGCAAGCAACTTTAGAGAGGAACCAG	rbsK	Sugar kinase, ribokinase family	WP_012195700.:	1051194..1052036	Pentose Phosphate	COG0524
WP_012195700.1_1126	CUST_6008_PI442190936	MIT9211	LLII/III	WP_012195700.1_1126	ATAACAGCTACAGACGCCAAGGGTATTCAACTTTGCAGAAATGCAAAAAACATTGTTGCC	rbsK	Sugar kinase, ribokinase family	WP_012195700.:	1051194..1052036	Pentose Phosphate	COG0524
WP_012195700.1_1126	CUST_6009_PI442190936	MIT9211	LLII/III	WP_012195700.1_1126	CAGCTGGCTGGGAAATTAACAAAGCCTTAAACCTAGCAGCAAAATGTGGTGCTATATGTA	rbsK	Sugar kinase, ribokinase family	WP_012195700.:	1051194..1052036	Pentose Phosphate	COG0524
WP_012195700.1_1126	CUST_6010_PI442190936	MIT9211	LLII/III	WP_012195700.1_1126	AAATCACAAAATAATTGACTCATATGGCTGCGGGATAGTTTTGCAGCAGGACTAACTGC	rbsK	Sugar kinase, ribokinase family	WP_012195700.:	1051194..1052036	Pentose Phosphate	COG0524
WP_012195835.1_1256	CUST_6630_PI442190936	MIT9211	LLII/III	WP_012195835.1_1256	GTTTAAAAGCTGTGGAGGAATCAGTGGAGAATCCTTTGCTTTATTGGGATGTCAACGTCG	galE	UDP-glucose 4-epimerase	WP_012195835.:	1157228..1158295	Galactose metabolism	COG1087
WP_012195835.1_1256	CUST_6631_PI442190936	MIT9211	LLII/III	WP_012195835.1_1256	GACAGGAATTCGTGATTACATTCATGTCATGGATTTAGCAGAAGCTCACCATGCAGCATT	galE	UDP-glucose 4-epimerase	WP_012195835.:	1157228..1158295	Galactose metabolism	COG1087
WP_012195835.1_1256	CUST_6632_PI442190936	MIT9211	LLII/III	WP_012195835.1_1256	AAAGGCTGCTATTGAGCAAATACTTAGTGATCTTTTTCAACACACTGACCAACAGTGGCG	galE	UDP-glucose 4-epimerase	WP_012195835.:	1157228..1158295	Galactose metabolism	COG1087
WP_012195835.1_1256	CUST_6633_PI442190936	MIT9211	LLII/III	WP_012195835.1_1256	TATGTCGTGATGGTTGGAAATGGCAAATGCTTAATCCCCAGGGCTATTTAAATAAAGCCA	galE	UDP-glucose 4-epimerase	WP_012195835.:	1157228..1158295	Galactose metabolism	COG1087
WP_012195835.1_1256	CUST_6634_PI442190936	MIT9211	LLII/III	WP_012195835.1_1256	AAATGTCAATGACTGCAAAGTACCTTATACTTTTGCAATAGGCGATTGGGGATGTCCC	galE	UDP-glucose 4-epimerase	WP_012195835.:	1157228..1158295	Galactose metabolism	COG1087
WP_012195835.1_1256	CUST_6635_PI442190936	MIT9211	LLII/III	WP_012195835.1_1256	CCACCAATCTGGAGAGATTGGAGAGTCCCCATTAGGTAAACCAAACAATTTGTTCCCTTA	galE	UDP-glucose 4-epimerase	WP_012195835.:	1157228..1158295	Galactose metabolism	COG1087
WP_012195841.1_1262	CUST_6666_PI442190936	MIT9211	LLII/III	WP_012195841.1_1262	TTCTTGGAGCGGGAAAGATATACCCTTCCCTGTTAAAAGGATACGAGATATGAGCCTAGG	ldhA	lactate dehydrogenase	WP_012195841.:	1162882..1163835	Pyruvate metabolism	COG1052
WP_012195841.1_1262	CUST_6667_PI442190936	MIT9211	LLII/III	WP_012195841.1_1262	TGCCTTCAGGCGTGGAGAAAGTCCTTGGATTAAAAAGGTTTCAGAGTTGCAAGATTTAC	ldhA	lactate dehydrogenase	WP_012195841.:	1162882..1163835	Pyruvate metabolism	COG1052
WP_012195841.1_1262	CUST_6668_PI442190936	MIT9211	LLII/III	WP_012195841.1_1262	TTAGAAGGATATGGGACCGATGTATGGACACAAGAGCCTCCAACAGACAAAGATCAGTTA	ldhA	lactate dehydrogenase	WP_012195841.:	1162882..1163835	Pyruvate metabolism	COG1052
WP_012195841.1_1262	CUST_6669_PI442190936	MIT9211	LLII/III	WP_012195841.1_1262	GGATAGGCGGCTGCTTAGCTAGGAAATTCACAGCATTATCAAATAATGTTGGATTCACG	ldhA	lactate dehydrogenase	WP_012195841.:	1162882..1163835	Pyruvate metabolism	COG1052
WP_012195841.1_1262	CUST_6670_PI442190936	MIT9211	LLII/III	WP_012195841.1_1262	CTGCCTACTATAGTCACGAATCATTAGAAGAGGCTAGGACCAAGGCCTGCAAAAATTGTC	ldhA	lactate dehydrogenase	WP_012195841.:	1162882..1163835	Pyruvate metabolism	COG1052
WP_012195841.1_1262	CUST_6671_PI442190936	MIT9211	LLII/III	WP_012195841.1_1262	TCAAGAGGTGCCATAGTGAAAGACAAGAGCATAATCTATGATGCTCTTGTCTCACACAAA	ldhA	lactate dehydrogenase	WP_012195841.:	1162882..1163835	Pyruvate metabolism	COG1052
WP_012195855.1_1276	CUST_6750_PI442190936	MIT9211	LLII/III	WP_012195855.1_1276	TTAGATAAAGCAGGAGCTAAATACCCTGCAGAACATAATGTCGGCCACTTGTACTTTGCT	dld	D-lactate dehydrogenase, NADH independent	WP_012195855.:	1176737..1178437	Pyruvate metabolism	COG0277
WP_012195855.1_1276	CUST_6751_PI442190936	MIT9211	LLII/III	WP_012195855.1_1276	GTCAGAAGCTTCTTTTCAAATGGGACATTTCCTATGCATGGTTTTTCATTGGGATTTCGT	dld	D-lactate dehydrogenase, NADH independent	WP_012195855.:	1176737..1178437	Pyruvate metabolism	COG0277
WP_012195855.1_1276	CUST_6752_PI442190936	MIT9211	LLII/III	WP_012195855.1_1276	AGATGTTGCTTTGCCTAGGAACAACAAAAATTGGCATGAAATTCTTCCTGAAAGTGCGTT	dld	D-lactate dehydrogenase, NADH independent	WP_012195855.:	1176737..1178437	Pyruvate metabolism	COG0277
WP_012195855.1_1276	CUST_6753_PI442190936	MIT9211	LLII/III	WP_012195855.1_1276	TGCTCTCAGTCCGAAGGTGAAGATTTGCTTTTACATAGATATGTAGCAGGAATTGCACCA	dld	D-lactate dehydrogenase, NADH independent	WP_012195855.:	1176737..1178437	Pyruvate metabolism	COG0277
WP_012195855.1_1276	CUST_6754_PI442190936	MIT9211	LLII/III	WP_012195855.1_1276	AAGATATTGCATAACCACGCTCAACTGTTACCAGATCACCTTCCAGTCAGAATAAGGCAA	dld	D-lactate dehydrogenase, NADH independent	WP_012195855.:	1176737..1178437	Pyruvate metabolism	COG0277
WP_012195855.1_1276	CUST_6755_PI442190936	MIT9211	LLII/III	WP_012195855.1_1276	TTTTGATCAACTGCCTGACATGGCAGAGTATATGCATAAAAGCTTTTTTGATGGAGCAGA	dld	D-lactate dehydrogenase, NADH independent	WP_012195855.:	1176737..1178437	Pyruvate metabolism	COG0277
WP_012195887.1_1304	CUST_6907_PI442190936	MIT9211	LLII/III	WP_012195887.1_1304	GTTACTGATGCAGTTGAATTTGCACTTGCAGCTAAAGACCCTGACCCAAGTGAATTAACT	pdhA	Pyruvate dehydrogenase E1 component alpha subunit	WP_012195887.:	1202814..1203896	Glycolysis/Krebs cycle	COG1071
WP_012195887.1_1304	CUST_6908_PI442190936	MIT9211	LLII/III	WP_012195887.1_1304	TTTCGAATGCCTCAACATGGCCCAACTATGGAAGCTGCCAATAATTTTTGTTGTTGAAAA	pdhA	Pyruvate dehydrogenase E1 component alpha subunit	WP_012195887.:	1202814..1203896	Glycolysis/Krebs cycle	COG1071
WP_012195887.1_1304	CUST_6909_PI442190936	MIT9211	LLII/III	WP_012195887.1_1304	AAAGAACATCATCTTTTGGGAGGCTATGCATTTATTGGAGAAGGGATTCCAGTCGCTCTA	pdhA	Pyruvate dehydrogenase E1 component alpha subunit	WP_012195887.:	1202814..1203896	Glycolysis/Krebs cycle	COG1071
WP_012195887.1_1304	CUST_6910_PI442190936	MIT9211	LLII/III	WP_012195887.1_1304	TGGGCACAAAGAGATCCATTAAAGAACCTTGCCAAGGTTCTTGTATCAAAAGAATTGGCA	pdhA	Pyruvate dehydrogenase E1 component alpha subunit	WP_012195887.:	1202814..1203896	Glycolysis/Krebs cycle	COG1071
WP_012195887.1_1304	CUST_6911_PI442190936	MIT9211	LLII/III	WP_012195887.1_1304	GAAAGGGCCCTTGAAAGAGCTAGAGCAGGAGAAGGACCTTCTTTAATTGAATGTCTTACC	pdhA	Pyruvate dehydrogenase E1 component alpha subunit	WP_012195887.:	1202814..1203896	Glycolysis/Krebs cycle	COG1071
WP_012195887.1_1304	CUST_6912_PI442190936	MIT9211	LLII/III	WP_012195887.1_1304	AGATAAAAATAGCGATGCTGTAACAGCCGCCTTCTTTGGAGATGGTACGTGTAATAACGG	pdhA	Pyruvate dehydrogenase E1 component alpha subunit	WP_012195887.:	1202814..1203896	Glycolysis/Krebs cycle	COG1071
WP_086934912.1_1326	CUST_7032_PI442190936	MIT9211	LLII/III	WP_086934912.1_1326	TCAAGCGTTTTATAGACAAGGTGCTAGTAAAGAAGCTGGAGCAAGCATTCCTGAGTTTCT	ftsZ	cell division protein FtsZ	WP_086934912.:	1226592..1227710	Cell Division	COG0206
WP_086934912.1_1326	CUST_7033_PI442190936	MIT9211	LLII/III	WP_086934912.1_1326	TGAAAGACTTGCAGAGAATGTTGACACTCTGATTGTGATTCCCAATGACCGTCTCAAAGA	ftsZ	cell division protein FtsZ	WP_086934912.:	1226592..1227710	Cell Division	COG0206
WP_086934912.1_1326	CUST_7034_PI442190936	MIT9211	LLII/III	WP_086934912.1_1326	AAGGCATCAGTGACATAATTACATGTCCTGGTCTTGTAAATGTGGACTTCGCAGATGTCA	ftsZ	cell division protein FtsZ	WP_086934912.:	1226592..1227710	Cell Division	COG0206
WP_086934912.1_1326	CUST_7035_PI442190936	MIT9211	LLII/III	WP_086934912.1_1326	AAGATATGACCCTAGAAGATATGACTACAGCATCAGAGGTGATTGCTGATGTGGTCGACC	ftsZ	cell division protein FtsZ	WP_086934912.:	1226592..1227710	Cell Division	COG0206
WP_086934912.1_1326	CUST_7036_PI442190936	MIT9211	LLII/III	WP_086934912.1_1326	GCAGTTATTGACCCTGAACTTGACGGAGAGGTACAAGTCACTGTTATAGCAACAGGTTTC	ftsZ	cell division protein FtsZ	WP_086934912.:	1226592..1227710	Cell Division	COG0206
WP_086934912.1_1326	CUST_7037_PI442190936	MIT9211	LLII/III	WP_086934912.1_1326	AAATGCAGGAGCACCTCTTCAAGAAGCATTCAGAAATGCAGATGACATTCTAAGAATGGG	ftsZ	cell division protein FtsZ	WP_086934912.:	1226592..1227710	Cell Division	COG0206
WP_041391579.1_1346	CUST_7142_PI442190936	MIT9211	LLII/III	WP_041391579.1_1346	GCTTGCACCTTTCGCGAAAAAAGCAAGAAAGCCAATAGAAAAGTCTGAGCCATTTAAACT	glcH	GPH family sugar transporter	WP_041391579.:	1244376..1245728	GlcH sugar transporter	COG2211
WP_041391579.1_1346	CUST_7143_PI442190936	MIT9211	LLII/III	WP_041391579.1_1346	ATGCTGCTGCCGCCACTATCTTCCGATTTCTCATTAGGGAACCTTTTAAACATAACTAGT	glcH	GPH family sugar transporter	WP_041391579.:	1244376..1245728	GlcH sugar transporter	COG2211
WP_041391579.1_1346	CUST_7144_PI442190936	MIT9211	LLII/III	WP_041391579.1_1346	GCAATTGACGCTGATGTCCAGAAAAACCTGCAGGAATTTATACAGCCTGGATGGTCTTAATT	glcH	GPH family sugar transporter	WP_041391579.:	1244376..1245728	GlcH sugar transporter	COG2211
WP_041391579.1_1346	CUST_7145_PI442190936	MIT9211	LLII/III	WP_041391579.1_1346	CATCATTACTTGTAGGCAGCGGGATATACATAATGAAAGGTTGGAATGACACCCCCTCTC	glcH	GPH family sugar transporter	WP_041391579.:	1244376..1245728	GlcH sugar transporter	COG2211
WP_041391579.1_1346	CUST_7146_PI442190936	MIT9211	LLII/III	WP_041391579.1_1346	AATGCTTCTTTTATTGTTAACAATTACGCTGGTTGGTTTTGGCGCATCAACTGCATACCT	glcH	GPH family sugar transporter	WP_041391579.:	1244376..1245728	GlcH sugar transporter	COG2211
WP_041391579.1_1346	CUST_7147_PI442190936	MIT9211	LLII/III	WP_041391579.1_1346	ACCAACAGGGTTTTCAAAATGGATCCCCATTCCTTTTCAAATAAGTGCTCTATTAGGCCT	glcH	GPH family sugar transporter	WP_041391579.:	1244376..1245728	GlcH sugar transporter	COG2211
WP_086934913.1_1366	CUST_7258_PI442190936	MIT9211	LLII/III	WP_086934913.1_1366	GGATTAGTTCGGGTGTCCCTGATTTAGATGAAATGTGCGGAGGAGGTTATTTTCAAGACT	kaiC	circadian clock protein KaiC	WP_086934913.:	1264399..1265904	Circadian Rhythm	COG0467
WP_086934913.1_1366	CUST_7259_PI442190936	MIT9211	LLII/III	WP_086934913.1_1366	ACTCTCTTTCTGCATTGGCTCGTGGCGTAAGTTTGAATGCATTTAGGCAGTTTGTAATAG	kaiC	circadian clock protein KaiC	WP_086934913.:	1264399..1265904	Circadian Rhythm	COG0467
WP_086934913.1_1366	CUST_7260_PI442190936	MIT9211	LLII/III	WP_086934913.1_1366	GAGTTACTGGATATGCAAAGCAGGAAGAGATTGCCGGCTTTTTTACAAATACTGCAGAGG	kaiC	circadian clock protein KaiC	WP_086934913.:	1264399..1265904	Circadian Rhythm	COG0467
WP_086934913.1_1366	CUST_7261_PI442190936	MIT9211	LLII/III	WP_086934913.1_1366	CCTGAGATAAAGGACTCTTTTAGTAACTTTGAGCAAATCTTTAGCGGAGCCCCTCATCGG	kaiC	circadian clock protein KaiC	WP_086934913.:	1264399..1265904	Circadian Rhythm	COG0467
WP_086934913.1_1366	CUST_7262_PI442190936	MIT9211	LLII/III	WP_086934913.1_1366	CACGAGCACAACTTCTTAGAAACGCAACAAGTTGGGGTATTGACTTTGAAGAGATGGAAA	kaiC	circadian clock protein KaiC	WP_086934913.:	1264399..1265904	Circadian Rhythm	COG0467
WP_086934913.1_1366	CUST_7263_PI442190936	MIT9211	LLII/III	WP_086934913.1_1366	GTAGAGATTAGAGGGGAGATGGCTAGGGCTGTAAATGTATTTAAGATGCGTGGTTCTTGG	kaiC	circadian clock protein KaiC	WP_086934913.:	1264399..1265904	Circadian Rhythm	COG0467
WP_012195949.1_1367	CUST_7264_PI442190936	MIT9211	LLII/III	WP_012195949.1_1367	TGAAGAATTGTCTGGGAATGAATTTTTATCATCTATTGCCAGTCCCAATTCAGATGAGGA	kaiB	Circadian clock protein KaiB	WP_012195949.:	1266019..1266363	Circadian Rhythm	COG4251
WP_012195949.1_1367	CUST_7265_PI442190936	MIT9211	LLII/III	WP_012195949.1_1367	CCCTGTCCGGCGTATTATTGGAGATCTATCAGACAGAGAAGAGTACTTATTGGGCTTGA	kaiB	Circadian clock protein KaiB	WP_012195949.:	1266019..1266363	Circadian Rhythm	COG4251
WP_012195949.1_1367	CUST_7266_PI442190936	MIT9211	LLII/III	WP_012195949.1_1367	TCAACTAGCAGAAGAGGATAAAATATTAGCGACTCCAACCTTGACCAAAATTCTCCCCCC	kaiB	Circadian clock protein KaiB	WP_012195949.:	1266019..1266363	Circadian Rhythm	COG4251
WP_012195949.1_1367	CUST_7267_PI442190936	MIT9211	LLII/III	WP_012195949.1_1367	CTTAGAAAATGAATTTAAGGGGGTATATGCCTTGAAGGTGATTGATATTTT	kaiB	Circadian clock protein KaiB	WP_012195949.:	1266019..1266363	Circadian Rhythm	COG4251
WP_012195949.1_1367	CUST_7268_PI442190936	MIT9211	LLII/III	WP_012195949.1_1367	GGCCGGCAATATACACCAAATTCAATGCGAGCATTGAAAACACTCCATGAAATCTTAGAAAA	kaiB	Circadian clock protein KaiB	WP_012195949.:	1266019..1266363	Circadian Rhythm	COG4251
WP_012196110.1_1523	CUST_8020_PI442190936	MIT9211	LLII/III	WP_012196110.1_1523	CGACTCAAAAAATCAAAGCAAAAGAAATGCTTAACAGAGCCAGAGCAAGGGTTCAAGCAA	atpC	ATP synthase epsilon subunit	WP_012196110.:	1390968..1391375	Energy Metabolism	COG0355
WP_012196110.1_1523	CUST_8021_PI442190936	MIT9211	LLII/III	WP_012196110.1_1523	GCCGAAAGCGAATTCGAACAGGCCAAAACTGAATTTGGTCAATTAGAAGGTCAAGAAAAT	atpC	ATP synthase epsilon subunit	WP_012196110.:	1390968..1391375	Energy Metabolism	COG0355
WP_012196110.1_1523	CUST_8022_PI442190936	MIT9211	LLII/III	WP_012196110.1_1523	AGAAGTTGAGTCTGATGAAGTTACTGTTCTAGTTAATGCTGCCGAGCTCGCTAGTGAGAT	atpC	ATP synthase epsilon subunit	WP_012196110.:	1390968..1391375	Energy Metabolism	COG0355

WP_012196110.1_1523	CUST_8023_PI442190936	MIT9211	LLII/III	WP_012196110.1_1523	GTCACTGCTATTGATACCGGAGTTCTAAGGGTACGAACAAATGGGAATTGGGACTCAATT	atpC	ATP synthase epsilon subunit	WP_012196110.:	1390968..1391375	Energy Metabolism	COG0355
WP_012196110.1_1523	CUST_8024_PI442190936	MIT9211	LLII/III	WP_012196110.1_1523	GAAGAAGTTATTCTTCCAAGCACAACAGGACTTTTGGGAATTTGCCAGGGCATATCTCA	atpC	ATP synthase epsilon subunit	WP_012196110.:	1390968..1391375	Energy Metabolism	COG0355
WP_012196122.1_1534	CUST_8081_PI442190936	MIT9211	LLII/III	WP_012196122.1_1534	AGGTGCCTGTTGAACAAGTAACTCAATTTGCTGGCGAACTCCGTGAATATTTAAAAACGA	atpA	ATP synthase alpha subunit	WP_012196122.:	1401107..1402621	Energy Metabolism	COG0056
WP_012196122.1_1534	CUST_8082_PI442190936	MIT9211	LLII/III	WP_012196122.1_1534	ACGATTAAGAGAACTTCTTAAACAGGCTCAGTTTGCTCCACTTAATCTTGCAGAGCAGGT	atpA	ATP synthase alpha subunit	WP_012196122.:	1401107..1402621	Energy Metabolism	COG0056
WP_012196122.1_1534	CUST_8083_PI442190936	MIT9211	LLII/III	WP_012196122.1_1534	CTCAGTTTGATGAATTGGCAGCATTCTCTCAATTCGCTTCTGATCTTGATGAAGCCACTC	atpA	ATP synthase alpha subunit	WP_012196122.:	1401107..1402621	Energy Metabolism	COG0056
WP_012196122.1_1534	CUST_8084_PI442190936	MIT9211	LLII/III	WP_012196122.1_1534	CAGCTATTAATGTTGGTATTTCAGTTAGTAGGGTAGGTGGTGCTGCTCAGACGAAAGCAA	atpA	ATP synthase alpha subunit	WP_012196122.:	1401107..1402621	Energy Metabolism	COG0056
WP_012196122.1_1534	CUST_8085_PI442190936	MIT9211	LLII/III	WP_012196122.1_1534	CAAAGCTTTCTGAATCAATGGGAAGTGGATCAATGACTGCATTGCCGATTATTGAAACGC	atpA	ATP synthase alpha subunit	WP_012196122.:	1401107..1402621	Energy Metabolism	COG0056
WP_012196122.1_1534	CUST_8086_PI442190936	MIT9211	LLII/III	WP_012196122.1_1534	TTGCGTGAGAAAGGAGCTCTTGAATACACAATTGTTGTTAATGCAAGTGCTTCTGAAGCT	atpA	ATP synthase alpha subunit	WP_012196122.:	1401107..1402621	Energy Metabolism	COG0056
WP_012196127.1_1539	CUST_8108_PI442190936	MIT9211	LLII/III	WP_012196127.1_1539	GGCTTGTTTACAAGTGCTATTCAGGCCTTGATCTTTGCAACTTTGGCTGCTTACTACATA	atpI	ATP synthase subunit A	WP_012196127.:	1404774..1405499	Energy Metabolism	COG0356
WP_012196127.1_1539	CUST_8109_PI442190936	MIT9211	LLII/III	WP_012196127.1_1539	CCTCTTTCTCTTTCTTTCCGTTTATTCGGGAACATTTTGGCGGATGAATTAGTCGTTGCA	atpI	ATP synthase subunit A	WP_012196127.:	1404774..1405499	Energy Metabolism	COG0356
WP_012196127.1_1539	CUST_8110_PI442190936	MIT9211	LLII/III	WP_012196127.1_1539	ATAGGAACTCTTTTCTTGTTTATTTTTGTGAGCAATTGGGGAGGAGCTTTGGTCCCTTGG	atpI	ATP synthase subunit A	WP_012196127.:	1404774..1405499	Energy Metabolism	COG0356
WP_012196127.1_1539	CUST_8111_PI442190936	MIT9211	LLII/III	WP_012196127.1_1539	CGTTACTTCGAGTATTACGTTCATCCCACTCCAATCATGCTTCCCTTCAAGATTGTCGAA	atpI	ATP synthase subunit A	WP_012196127.:	1404774..1405499	Energy Metabolism	COG0356
WP_012196127.1_1539	CUST_8112_PI442190936	MIT9211	LLII/III	WP_012196127.1_1539	GGGATTACATACGTGATTTGGCTAGGACCCAAATAGGAGAAAAGGTTTATCGCGATTGGA	atpI	ATP synthase subunit A	WP_012196127.:	1404774..1405499	Energy Metabolism	COG0356
WP_012196127.1_1539	CUST_8113_PI442190936	MIT9211	LLII/III	WP_012196127.1_1539	GGCTTCTTATTGGTGCCCTCTTAACTCTTGTTGTTGTCGGTACCAAAAAAATGGAACGAG	atpI	ATP synthase subunit A	WP_012196127.:	1404774..1405499	Energy Metabolism	COG0356
WP_012196138.1_1550	CUST_8166_PI442190936	MIT9211	LLII/III	WP_012196138.1_1550	TGCAGAAGTTGCCGAAAAAATATGGTAGAGGGCATTAGACCAAACGAAGAGAAAATTGCA	fumC	class II fumarate hydratase	WP_012196138.:	1413464..1414864	Krebs cycle and related genes	COG0114
WP_012196138.1_1550	CUST_8167_PI442190936	MIT9211	LLII/III	WP_012196138.1_1550	AGGATAGAAGTCAGTCTCAGAGAGCTATATCCACTTCCATTAGGAGGTACAGCCATAGGG	fumC	class II fumarate hydratase	WP_012196138.:	1413464..1414864	Krebs cycle and related genes	COG0114
WP_012196138.1_1550	CUST_8168_PI442190936	MIT9211	LLII/III	WP_012196138.1_1550	AAGTCAACCCAACTCAATGTGAAGCCATGTCAATGGTATGCATGCAAGTAATAGGTCTTG	fumC	class II fumarate hydratase	WP_012196138.:	1413464..1414864	Krebs cycle and related genes	COG0114
WP_012196138.1_1550	CUST_8169_PI442190936	MIT9211	LLII/III	WP_012196138.1_1550	AAAAATAGTCAATGATATCCGACTCTTGTCATGTGGTCCAAGAGCTGGGTTTTCCGAACT	fumC	class II fumarate hydratase	WP_012196138.:	1413464..1414864	Krebs cycle and related genes	COG0114
WP_012196138.1_1550	CUST_8170_PI442190936	MIT9211	LLII/III	WP_012196138.1_1550	CAATGCCACTAAGATTGCCAAATATGCTTATGAGACAGGCGTAAGTTTACGAGAAGCAGC	fumC	class II fumarate hydratase	WP_012196138.:	1413464..1414864	Krebs cycle and related genes	COG0114
WP_012196138.1_1550	CUST_8171_PI442190936	MIT9211	LLII/III	WP_012196138.1_1550	AGGTGGGGGGCACCTACAAATGAATGCCTACAAACCATTAATAGGTTCAATCTTCTAAA	fumC	class II fumarate hydratase	WP_012196138.:	1413464..1414864	Krebs cycle and related genes	COG0114
WP_086934918.1_1598	CUST_8431_PI442190936	MIT9211	LLII/III	WP_086934918.1_1598	CCGATAGAAGCGTTTGTGCCCGTATATCCGGTGAGATAGCTCAACTATATGGAAACAAAG	glnA	Glutamine synthetase/ glutamate--ammonialigase	WP_086934918.:	1469067..1473590	Nitrogen Metabolism	COG0174
WP_086934918.1_1598	CUST_8432_PI442190936	MIT9211	LLII/III	WP_086934918.1_1598	TGCCTCCTAGCAATAATCAAAATGTCTCCAATCATGTAATCCTTGGCAACACATGCCTTT	glnA	Glutamine synthetase/ glutamate--ammonialigase	WP_086934918.:	1469067..1473590	Nitrogen Metabolism	COG0174
WP_086934918.1_1598	CUST_8433_PI442190936	MIT9211	LLII/III	WP_086934918.1_1598	TTTGCAAGGAATGAACATTCGCTTAGTGGGGGAAGCTAACGACTATGTAGGTAAAGGAAT	glnA	Glutamine synthetase/ glutamate--ammonialigase	WP_086934918.:	1469067..1473590	Nitrogen Metabolism	COG0174
WP_086934918.1_1598	CUST_8434_PI442190936	MIT9211	LLII/III	WP_086934918.1_1598	TAAAGTAATTGTCCCACCAAGTGAGAAAAGTAAAGTTGGCCTAGAGGCAGTGGACATAGC	glnA	Glutamine synthetase/ glutamate--ammonialigase	WP_086934918.:	1469067..1473590	Nitrogen Metabolism	COG0174
WP_086934918.1_1598	CUST_8435_PI442190936	MIT9211	LLII/III	WP_086934918.1_1598	AGGAGGATTTGCTCAAGCCTCTCTTAGAAGAGCACTTAGTACAAACTCAAAGCACCAAAG	glnA	Glutamine synthetase/ glutamate--ammonialigase	WP_086934918.:	1469067..1473590	Nitrogen Metabolism	COG0174
WP_086934918.1_1598	CUST_8436_PI442190936	MIT9211	LLII/III	WP_086934918.1_1598	TTAAAGGTAATCTTAATCTGACATTTAAGGGTTCGGCAGGACAAAGTTTTGGCGCCTTCC	glnA	Glutamine synthetase/ glutamate--ammonialigase	WP_086934918.:	1469067..1473590	Nitrogen Metabolism	COG0174
WP_012196191.1_1602	CUST_8453_PI442190936	MIT9211	LLII/III	WP_012196191.1_1602	ACTTTTTGAGTCAATCGTTTGGGCCCATAACAAATTGAAAGTGGCTCCAACAATTCAACC	psaA	Photosystem I P700 chlorophyll A apoprotein A1 PsaA	WP_012196191.:	1477076..1479397	Photosystem I	COG2885
WP_012196191.1_1602	CUST_8454_PI442190936	MIT9211	LLII/III	WP_012196191.1_1602	AGTTCTTTACTCAAGAAGTTCTCGTCTCATTCCTGACAAAGCTCAACTTGGCTTCCGTTT	psaA	Photosystem I P700 chlorophyll A apoprotein A1 PsaA	WP_012196191.:	1477076..1479397	Photosystem I	COG2885
WP_012196191.1_1602	CUST_8455_PI442190936	MIT9211	LLII/III	WP_012196191.1_1602	GACTAATGTTCCTTGGTGCTCACTTTGTTTGGGCATTCAGCCTTATGTTCCTATTCAGTG	psaA	Photosystem I P700 chlorophyll A apoprotein A1 PsaA	WP_012196191.:	1477076..1479397	Photosystem I	COG2885
WP_012196191.1_1602	CUST_8456_PI442190936	MIT9211	LLII/III	WP_012196191.1_1602	GAAGTATTCAATGGAAATGTCATTGAAGTTGGAGGAAAAGTTGCTATTGGGCCGATCCCA	psaA	Photosystem I P700 chlorophyll A apoprotein A1 PsaA	WP_012196191.:	1477076..1479397	Photosystem I	COG2885
WP_012196191.1_1602	CUST_8457_PI442190936	MIT9211	LLII/III	WP_012196191.1_1602	GAAATTTTGCCCAAAGCTCAATCACAATTAATGGTTGGCTTAGGGATTTCCTATGGGCAC	psaA	Photosystem I P700 chlorophyll A apoprotein A1 PsaA	WP_012196191.:	1477076..1479397	Photosystem I	COG2885
WP_012196191.1_1602	CUST_8458_PI442190936	MIT9211	LLII/III	WP_012196191.1_1602	GGCACATGTCAGGTTTCCTCATGGGATCATGTTTTCCTTGGCCTATTTTGGATGTATAAC	psaA	Photosystem I P700 chlorophyll A apoprotein A1 PsaA	WP_012196191.:	1477076..1479397	Photosystem I	COG2885
WP_012196192.1_1603	CUST_8459_PI442190936	MIT9211	LLII/III	WP_012196192.1_1603	GTGGTACATGTGATAGTTCGGCTTGGGATGCTACTTATTTAGCAATGTTCTGGGCTCTAA	psaB	Photosystem I P700 chlorophyll A apoprotein A2 PsaB	WP_012196192.:	1479419..1481647	Photosystem I	COG2885
WP_012196192.1_1603	CUST_8460_PI442190936	MIT9211	LLII/III	WP_012196192.1_1603	AACCTTGTAGGTTGGAGAGATAAGCCAGTTGCCTTATCAATTGTTCAAGCACGTCTAGTT	psaB	Photosystem I P700 chlorophyll A apoprotein A2 PsaB	WP_012196192.:	1479419..1481647	Photosystem I	COG2885
WP_012196192.1_1603	CUST_8461_PI442190936	MIT9211	LLII/III	WP_012196192.1_1603	GCTGACTTCTTGGTTCACCATGCAATTGCTTTAGGACTACACACAACTGCCTTAATACTG	psaB	Photosystem I P700 chlorophyll A apoprotein A2 PsaB	WP_012196192.:	1479419..1481647	Photosystem I	COG2885
WP_012196192.1_1603	CUST_8462_PI442190936	MIT9211	LLII/III	WP_012196192.1_1603	TGCTAGAGGCACTAAATTAATCCCTGACAAGAAAGACTTTGGCTATGCATTCCCTTGTGA	psaB	Photosystem I P700 chlorophyll A apoprotein A2 PsaB	WP_012196192.:	1479419..1481647	Photosystem I	COG2885
WP_012196192.1_1603	CUST_8463_PI442190936	MIT9211	LLII/III	WP_012196192.1_1603	GTATGGCATTGATGCGTTATTAGCTAATGCAAATAGTTCTGCAACCTTGGCTGCAAACAG	psaB	Photosystem I P700 chlorophyll A apoprotein A2 PsaB	WP_012196192.:	1479419..1481647	Photosystem I	COG2885
WP_012196192.1_1603	CUST_8464_PI442190936	MIT9211	LLII/III	WP_012196192.1_1603	GGAAACTTTGTTACCTTTGGTGCCTTTGTGATAGCTTCTACTTCGGGCAAGTTTGGATAG	psaB	Photosystem I P700 chlorophyll A apoprotein A2 PsaB	WP_012196192.:	1479419..1481647	Photosystem I	COG2885
WP_012196246.1_1659	CUST_8762_PI442190936	MIT9211	LLII/III	WP_012196246.1_1659	GGATTTATCTAGTCTTAGGGCCATACCTTGGGTCTTTGGTTGGACTCAGAGTAGGTTTCT	ppc	Phosphoenolpyruvate carboxylase	WP_012196246.:	1519264..1522287	Krebs cycle and related genes	COG2352
WP_012196246.1_1659	CUST_8763_PI442190936	MIT9211	LLII/III	WP_012196246.1_1659	TTGCAAGCGTCTGTAGAGCTTAGAAACCGTACGATTGTACCTTTAGGGTTTCTTCAAGTG	ppc	Phosphoenolpyruvate carboxylase	WP_012196246.:	1519264..1522287	Krebs cycle and related genes	COG2352
WP_012196246.1_1659	CUST_8764_PI442190936	MIT9211	LLII/III	WP_012196246.1_1659	AAGAAGATCCTGCACAAATTGAGCTTTTGAAAATGCTTCATCAGCGTTGGCCTTTCTTTC	ppc	Phosphoenolpyruvate carboxylase	WP_012196246.:	1519264..1522287	Krebs cycle and related genes	COG2352
WP_012196246.1_1659	CUST_8765_PI442190936	MIT9211	LLII/III	WP_012196246.1_1659	ATGACAACCCTGACTTGGTGCCATTTTTTCAAGAAGTGACGCCAATTGAAGAAATCAGTA	ppc	Phosphoenolpyruvate carboxylase	WP_012196246.:	1519264..1522287	Krebs cycle and related genes	COG2352
WP_012196246.1_1659	CUST_8766_PI442190936	MIT9211	LLII/III	WP_012196246.1_1659	AGTGAATTATTGAGAGGAGCTTTGTTGACTATTAATGGCATTGCTGCGGGAATGCGAAAT	ppc	Phosphoenolpyruvate carboxylase	WP_012196246.:	1519264..1522287	Krebs cycle and related genes	COG2352
WP_012196246.1_1659	CUST_8767_PI442190936	MIT9211	LLII/III	WP_012196246.1_1659	TTTGGTTACTAATCAGTTAGATGCAACCCCTAGTTGGAATGAGCTGATGACTCGATTGGC	ppc	Phosphoenolpyruvate carboxylase	WP_012196246.:	1519264..1522287	Krebs cycle and related genes	COG2352
WP_012196282.1_1695	CUST_8962_PI442190936	MIT9211	LLII/III	WP_012196282.1_1695	TTTTGAGTAATGCTCAAGAAGCTAAGGCAAGAGATGCAAGTTTGATAGGGATTTGCCCAC	glmS	Glutamine--fructose-6-phosphatetransaminase(isomerizing)	WP_012196282.:	1557551..1559455	Amino Acid metabolism	COG0449
WP_012196282.1_1695	CUST_8963_PI442190936	MIT9211	LLII/III	WP_012196282.1_1695	GACGCGGCATTAATTACCCAATTGCTCTAGAAGGCGCACTCAAATTAAAAGAGATCAGTT	glmS	Glutamine--fructose-6-phosphatetransaminase(isomerizing)	WP_012196282.:	1557551..1559455	Amino Acid metabolism	COG0449
WP_012196282.1_1695	CUST_8964_PI442190936	MIT9211	LLII/III	WP_012196282.1_1695	GAGCACCAAAGGCGATCAGTTCTGGATAACAATGATTTTGCTTCTTTCCAGTTGGGAATA	glmS	Glutamine--fructose-6-phosphatetransaminase(isomerizing)	WP_012196282.:	1557551..1559455	Amino Acid metabolism	COG0449
WP_012196282.1_1695	CUST_8965_PI442190936	MIT9211	LLII/III	WP_012196282.1_1695	CGGCTGGCGAAATGAAGCATGGACCTATTGCTTTATTGGATCAAAGAGTACCTGTAATTT	glmS	Glutamine--fructose-6-phosphatetransaminase(isomerizing)	WP_012196282.:	1557551..1559455	Amino Acid metabolism	COG0449
WP_012196282.1_1695	CUST_8966_PI442190936	MIT9211	LLII/III	WP_012196282.1_1695	GCACTGCAAGTTCCCAATATTCTTGATATAGGAGCAGGTATAGAAGTTGGTGTAGCAGCA	glmS	Glutamine--fructose-6-phosphatetransaminase(isomerizing)	WP_012196282.:	1557551..1559455	Amino Acid metabolism	COG0449
WP_012196282.1_1695	CUST_8967_PI442190936	MIT9211	LLII/III	WP_012196282.1_1695	AATCAATTGGCCGAGTCCTTAGCTCATCGTTTCGCCGATACAGAAGATGTCATTTTCTTA	glmS	Glutamine--fructose-6-phosphatetransaminase(isomerizing)	WP_012196282.:	1557551..1559455	Amino Acid metabolism	COG0449
WP_011862039.1_13	CUST_68_PI442190907	MIT9301	HLII	WP_011862039.1_13	TTAATGCCGCAGAGAAAAATCCCGATGTTCCAGAATTGATAAGAGGTAAGACGGGAAGA	argH	Argininosuccinate lyase	WP_011862039.:	14576..15955	Amino Acid metabolism	COG0165
WP_011862039.1_13	CUST_69_PI442190907	MIT9301	HLII	WP_011862039.1_13	TGTCGTTAAATCAAGAAATAGCGAGGGTGGAACAGGTTTTGCTCAGGTAGAAAAAGAGGT	argH	Argininosuccinate lyase	WP_011862039.:	14576..15955	Amino Acid metabolism	COG0165
WP_011862039.1_13	CUST_70_PI442190907	MIT9301	HLII	WP_011862039.1_13	AGTGTATGGACATCTTCAGGCATTATTAACTATGGTCAAAGGGGTACCTCTCTCATACA	argH	Argininosuccinate lyase	WP_011862039.:	14576..15955	Amino Acid metabolism	COG0165
WP_011862039.1_13	CUST_72_PI442190907	MIT9301	HLII	WP_011862039.1_13	AGGCACTTTACAGCTGCAGAATTGGGCTTTGAAAAGATTTATAAAAATAGTATTGATGCT	argH	Argininosuccinate lyase	WP_011862039.:	14576..15955	Amino Acid metabolism	COG0165
WP_011862039.1_13	CUST_73_PI442190907	MIT9301	HLII	WP_011862039.1_13	TGCAGAAACAATTTCTTCTTGTATTACGGCAATGACTATTCTACTCAATGAAGGTATCGA	argH	Argininosuccinate lyase	WP_011862039.:	14576..15955	Amino Acid metabolism	COG0165
WP_011862251.1_221	CUST_1220_PI442190907	MIT9301	HLII	WP_011862251.1_221	AGATCGGGGCAGATCAAGACCGGCTCTTTGAGTAGAAGTGAAAGGATTGCAAAATATAAT	eno	Enolase	WP_011862251.:	212228..213520	Glycolysis/Gluconeogenesis	COG0148
WP_011862251.1_221	CUST_1221_PI442190907	MIT9301	HLII	WP_011862251.1_221	GAAGAGGAATTGGGAAATCAAGCAAGATTCGCTGGAGCTTTAGGTTTAGGTCCCAAAAAT	eno	Enolase	WP_011862251.:	212228..213520	Glycolysis/Gluconeogenesis	COG0148
WP_011862251.1_221	CUST_1222_PI442190907	MIT9301	HLII	WP_011862251.1_221	AGAGGACGGTTTAGCTGAGGATGATTGGGAGGGTTGGTCAGAATTAAACAAAGAATTAGG	eno	Enolase	WP_011862251.:	212228..213520	Glycolysis/Gluconeogenesis	COG0148
WP_011862251.1_221	CUST_1223_PI442190907	MIT9301	HLII	WP_011862251.1_221	CCGGATTCAAGCCTGGTGAGCAGGTATCTTTAGCTTTAGATGCTGCTAGTACTGAATTTT	eno	Enolase	WP_011862251.:	212228..213520	Glycolysis/Gluconeogenesis	COG0148
WP_011862251.1_221	CUST_1224_PI442190907	MIT9301	HLII	WP_011862251.1_221	GCCCCGAATTTGTCATCAAGCGAAGAGGCAGGGGACTTATTATTAGAAGCAATTCAAAAA	eno	Enolase	WP_011862251.:	212228..213520	Glycolysis/Gluconeogenesis	COG0148
WP_011862251.1_221	CUST_1225_PI442190907	MIT9301	HLII	WP_011862251.1_221	ATTAGTCATAGAAGTGGTGAGACTGAAGATACAACAATTGCTGATTTGTCTGTCGCCACG	eno	Enolase	WP_011862251.:	212228..213520	Glycolysis/Gluconeogenesis	COG0148
WP_011862268.1_238	CUST_1310_PI442190907	MIT9301	HLII	WP_011862268.1_238	AGGGTATAAATCCGTCGTAATTGGAGATAAAGCCATCAAAAATAAAAAATTTGATCCAAA	eda	2-dehydro-3-deoxyphosphogluconate aldolase	WP_011862268.:	230112..230738	Entner-Dudoroff	COG0800
WP_011862268.1_238	CUST_1311_PI442190907	MIT9301	HLII	WP_011862268.1_238	AGTTGGTCCAACAACAAAAATTGGTTAGATTTGTTATCCGATATCAAAACTAAATATCCA	eda	2-dehydro-3-deoxyphosphogluconate aldolase	WP_011862268.:	230112..230738	Entner-Dudoroff	COG0800
WP_011862268.1_238	CUST_1312_PI442190907	MIT9301	HLII	WP_011862268.1_238	AAAGGATCTTAAGGACGCGACAAATTTAAATTGCAACATTATCAAAATTTACCCAATAAA	eda	2-dehydro-3-deoxyphosphogluconate aldolase	WP_011862268.:	230112..230738	Entner-Dudoroff	COG0800
WP_011862268.1_238	CUST_1313_PI442190907	MIT9301	HLII	WP_011862268.1_238	GGATTAAATTTTTCGATGATGAAATTTTGGGATAAAGATCTTTTCAATTATGCACAGTCA	eda	2-dehydro-3-deoxyphosphogluconate aldolase	WP_011862268.:	230112..230738	Entner-Dudoroff	COG0800
WP_011862268.1_238	CUST_1314_PI442190907	MIT9301	HLII	WP_011862268.1_238	AGATAATTCGATAGATATACTCAAGTATAAAAATATTGATTTCATTGCTGCTGGAGGACT	eda	2-dehydro-3-deoxyphosphogluconate aldolase	WP_011862268.:	230112..230738	Entner-Dudoroff	COG0800
WP_011862268.1_238	CUST_1315_PI442190907	MIT9301	HLII	WP_011862268.1_238	AGGGAAGATTCTTTTTGTTATTTATCGCTGAAAGAATCTTTTTTTTTACTTATAAAACCT	eda	2-dehydro-3-deoxyphosphogluconate aldolase	WP_011862268.:	230112..230738	Entner-Dudoroff	COG0800
WP_011862356.1_335	CUST_1822_PI442190907	MIT9301	HLII	WP_011862356.1_335	AAAAAAACTGATAAAACTGATAAAGCTAATAAAGATATCAAAAAGTAA	minE	septum site-determining protein MinE	WP_011862356.:	314788..315123	Cell Division	COG0851
WP_011862356.1_335	CUST_1823_PI442190907	MIT9301	HLII	WP_011862356.1_335	AGTAGCAAATTTACCAATCAAAAGAACTATTGACGGAGAAATAAAGTTTAAAAAAACTGA	minE	septum site-determining protein MinE	WP_011862356.:	314788..315123	Cell Division	COG0851
WP_011862356.1_335	CUST_1824_PI442190907	MIT9301	HLII	WP_011862356.1_335	TGAGATTGATTTTGAAGAGGTGGCAGTAAGTTTAGAAACTGAAGACAGAATGACTGCACT	minE	septum site-determining protein MinE	WP_011862356.:	314788..315123	Cell Division	COG0851
WP_011862356.1_335	CUST_1825_PI442190907	MIT9301	HLII	WP_011862356.1_335	ACAACTTGTACTTGCTCACGACAGAGTTGATATGAGTTCTTTAACAACTGATCTTTTGGA	minE	septum site-determining protein MinE	WP_011862356.:	314788..315123	Cell Division	COG0851
WP_011862356.1_335	CUST_1826_PI442190907	MIT9301	HLII	WP_011862356.1_335	ACACTCAGAGATCTTATAAACAAATTATTAGGCAGAGAAACGGCTAGCGCCAACACAGCA	minE	septum site-determining protein MinE	WP_011862356.:	314788..315123	Cell Division	COG0851
WP_011862357.1_336	CUST_1827_PI442190907	MIT9301	HLII	WP_011862357.1_336	ATCTGTTCAGGTAAAGGAGGGGTTGGTAAAACCACTTTAACTGCAAATCTAGGCATAGCT	minD	septum site-determining protein MinD	WP_011862357.:	315125..315940	Cell Division	COG2894
WP_011862357.1_336	CUST_1828_PI442190907	MIT9301	HLII	WP_011862357.1_336	GACATAAAAAGGAACCTAATCTTGCTCTTCTACCCGCTGGAGATCCAAGGATGTTGGATT	minD	septum site-determining protein MinD	WP_011862357.:	315125..315940	Cell Division	COG2894
WP_011862357.1_336	CUST_1829_PI442190907	MIT9301	HLII	WP_011862357.1_336	TGTGGTTACTAACCCAGAATTATCTGCAGTACGCGACGCCGATAGAGTAATAGGAATTCT	minD	septum site-determining protein MinD	WP_011862357.:	315125..315940	Cell Division	COG2894
WP_011862357.1_336	CUST_1830_PI442190907	MIT9301	HLII	WP_011862357.1_336	TTGCTAATAGCGGAGCCACAACCGCTGTATTAGATGCTGATTTTGGCTTGAGAAATTTAG	minD	septum site-determining protein MinD	WP_011862357.:	315125..315940	Cell Division	COG2894
WP_011862357.1_336	CUST_1831_PI442190907	MIT9301	HLII	WP_011862357.1_336	GCATCATTTATACAGCTCAAGATGTTCTTGACAAGAATTGTCGTCTTGACCAAGCGCTGG	minD	septum site-determining protein MinD	WP_011862357.:	315125..315940	Cell Division	COG2894
WP_011862357.1_336	CUST_1832_PI442190907	MIT9301	HLII	WP_011862357.1_336	TGCTTAGTGAGAAATTTGATTTTGTCTTAGTAGATTGTCCTGCTGGCGTCGAAGATGGCT	minD	septum site-determining protein MinD	WP_011862357.:	315125..315940	Cell Division	COG2894
WP_011862358.1_337	CUST_1833_PI442190907	MIT9301	HLII	WP_011862358.1_337	GGGGTCGCATTCGCGGGGAAAAGTGGAAATAAAAGTGCCTCTATTTCATCACTTTATTA	minC	septum site-determining protein MinC	WP_011862358.:	316052..316714	Cell Division	COG0850
WP_011862358.1_337	CUST_1834_PI442190907	MIT9301	HLII	WP_011862358.1_337	TGGAGACGTTAATCCAGGAGCAATAGTTTCCGCCAAAAAAAACATTTACGTTTGGGGTAA	minC	septum site-determining protein MinC	WP_011862358.:	316052..316714	Cell Division	COG0850
WP_011862358.1_337	CUST_1835_PI442190907	MIT9301	HLII	WP_011862358.1_337	TTCTTCACGAAGGAACAGTTCGATCGGGTGAAAGAATATCTTCAAATGGAAACCTATGCA	minC	septum site-determining protein MinC	WP_011862358.:	316052..316714	Cell Division	COG0850
WP_011862358.1_337	CUST_1836_PI442190907	MIT9301	HLII	WP_011862358.1_337	GCAGATGTAATAGCTATTGGACCAAAGGATAAGCCCAATAATTTTTACCCAGAAATTGCA	minC	septum site-determining protein MinC	WP_011862358.:	316052..316714	Cell Division	COG0850
WP_011862358.1_337	CUST_1837_PI442190907	MIT9301	HLII	WP_011862358.1_337	TCCATCAAGGAACCTTTAGAAGTAAATATTTTCGCAGTCAATGAATCAATAAGTGCTCAA	minC	septum site-determining protein MinC	WP_011862358.:	316052..316714	Cell Division	COG0850
WP_011862358.1_337	CUST_1838_PI442190907	MIT9301	HLII	WP_011862358.1_337	GTGGAAAGTCTTTAAAAATAGATTCAACTTTTTGTAAAGAGCAAGAGATTCAAAATAAGT	minC	septum site-determining protein MinC	WP_011862358.:	316052..316714	Cell Division	COG0850
WP_011862466.1_444	CUST_2382_PI442190907	MIT9301	HLII	WP_011862466.1_444	TGCTTGGCAATCGAGAATCAAGAGCGGAAGATTAACAAGTCAAAATATGTTCCCTTTGGA	cyoC	heme-copper oxidase subunit III	WP_011862466.:	408466..409068	Respiration	COG1845
WP_011862466.1_444	CUST_2383_PI442190907	MIT9301	HLII	WP_011862466.1_444	TTGGTCTTATAACTTTCCTAATTGCGGATGGAATGACTTTTGCTGGATTCTTTGCTGCTT	cyoC	heme-copper oxidase subunit III	WP_011862466.:	408466..409068	Respiration	COG1845
WP_011862466.1_444	CUST_2384_PI442190907	MIT9301	HLII	WP_011862466.1_444	AGTGCAACTTTCCATAAAGCCGGCAAAGCACTTTTAAAAGATAAAAACTCTGAATCCCAA	cyoC	heme-copper oxidase subunit III	WP_011862466.:	408466..409068	Respiration	COG1845
WP_011862466.1_444	CUST_2385_PI442190907	MIT9301	HLII	WP_011862466.1_444	AGCAGTAAATCCATTACCTGATGGTGCTATTTATGAATTAGAACTCCCAATACCTACACT	cyoC	heme-copper oxidase subunit III	WP_011862466.:	408466..409068	Respiration	COG1845
WP_011862466.1_444	CUST_2386_PI442190907	MIT9301	HLII	WP_011862466.1_444	TTTGCAAGTACTTTTTATGCTCTTACTGGTTTTCATGGATTACACGTCACTTTAGGAACT	cyoC	heme-copper oxidase subunit III	WP_011862466.:	408466..409068	Respiration	COG1845
WP_011862466.1_444	CUST_2387_PI442190907	MIT9301	HLII	WP_011862466.1_444	AGCTCAAAAGAAATTCAAAAAAATAATTCTGAAGTCAACGAAACACATGAAGACTTCAGA	cyoC	heme-copper oxidase subunit III	WP_011862466.:	408466..409068	Respiration	COG1845
WP_011862467.1_445	CUST_2388_PI442190907	MIT9301	HLII	WP_011862467.1_445	TGGAAAAGATGCTGGAGATAACCCTTGGAATGCTCTTACACCTGAGTGGTTAACATCTTC	cyoB/coxA	Cytochrome c oxidase subunit I	WP_011862467.:	409075..410700	Respiration	COG0843
WP_011862467.1_445	CUST_2389_PI442190907	MIT9301	HLII	WP_011862467.1_445	TTCGTTTTTGGAGGTATTACAGGAGTTGCTTTGGCACAGGTACCTTTCGATATTCACGTA	cyoB/coxA	Cytochrome c oxidase subunit I	WP_011862467.:	409075..410700	Respiration	COG0843
WP_011862467.1_445	CUST_2390_PI442190907	MIT9301	HLII	WP_011862467.1_445	GCTTTGCTCCTCAACATTGGCTTGGTTTAAATGGAATGCCAAGAAGAGTTGCAGAATATG	cyoB/coxA	Cytochrome c oxidase subunit I	WP_011862467.:	409075..410700	Respiration	COG0843
WP_011862467.1_445	CUST_2391_PI442190907	MIT9301	HLII	WP_011862467.1_445	CCTCCAGTTGAAAATTGGGAAGGAGAAGCTCCATTAGTTGAAGAACCCTATGGTTATGGT	cyoB/coxA	Cytochrome c oxidase subunit I	WP_011862467.:	409075..410700	Respiration	COG0843
WP_011862467.1_445	CUST_2392_PI442190907	MIT9301	HLII	WP_011862467.1_445	CCTTGGATGAGATTGTTCTTTACAATTGCCACAGCATTTATCGCTGTTCCAACGGGTATA	cyoB/coxA	Cytochrome c oxidase subunit I	WP_011862467.:	409075..410700	Respiration	COG0843
WP_011862467.1_445	CUST_2393_PI442190907	MIT9301	HLII	WP_011862467.1_445	ATGGGGATAGTAGTTTTAGGTCTAGTTGTTTGGGCGCATCACATGTTTACAAGTGGAACG	cyoB/coxA	Cytochrome c oxidase subunit I	WP_011862467.:	409075..410700	Respiration	COG0843
WP_011862468.1_446	CUST_2394_PI442190907	MIT9301	HLII	WP_011862468.1_446	GTGGCCCATATCATGGAGGGATGAGAGCCTCCATAATTGTTGAAGAAGAATCTGATTACA	ctaC/coxB	Cytochrome c oxidase subunit II	WP_011862468.:	410697..411500	Respiration	COG1622
WP_011862468.1_446	CUST_2395_PI442190907	MIT9301	HLII	WP_011862468.1_446	ATTCATGCTTTTTGGGTGCCAGAGTTCAGAATTAAACAGGATATTATTCCTGGGCAACCT	ctaC/coxB	Cytochrome c oxidase subunit II	WP_011862468.:	410697..411500	Respiration	COG1622
WP_011862468.1_446	CUST_2396_PI442190907	MIT9301	HLII	WP_011862468.1_446	TCCCAAGGGCAATTTCATATCAGGAGAACTACACGTTCCTGTTGATCAAAAAGTATCAAT	ctaC/coxB	Cytochrome c oxidase subunit II	WP_011862468.:	410697..411500	Respiration	COG1622
WP_011862468.1_446	CUST_2397_PI442190907	MIT9301	HLII	WP_011862468.1_446	TCTTTTTGGATTGGTTTTAATGTAAATTTGCTCCCAACGGAAGCAAGTATTAATGCACCA	ctaC/coxB	Cytochrome c oxidase subunit II	WP_011862468.:	410697..411500	Respiration	COG1622
WP_011862468.1_446	CUST_2398_PI442190907	MIT9301	HLII	WP_011862468.1_446	AGCTACAACATCTACGATCGAATGGGAGGTATGAAAGAACTAAATCATAACCACGAAATG	ctaC/coxB	Cytochrome c oxidase subunit II	WP_011862468.:	410697..411500	Respiration	COG1622
WP_011862468.1_446	CUST_2399_PI442190907	MIT9301	HLII	WP_011862468.1_446	AGTTCTAATTCTGAAAAAATATGGGCTGGAATAAGTCAAACTTCTGATAATGAAATAGCA	ctaC/coxB	Cytochrome c oxidase subunit II	WP_011862468.:	410697..411500	Respiration	COG1622
WP_011862476.1_454	CUST_2439_PI442190907	MIT9301	HLII	WP_011862476.1_454	AATTATTCTGCAGCCAAAGCTGGCGTTATTGGATTCACCAAAACTTGCGCTAAAGAATTT	gdh	3-oxoacyl-acyl-carrier-protein reductase	WP_011862476.:	418245..418994	Entner-Dudoroff	COG1028
WP_011862476.1_454	CUST_2440_PI442190907	MIT9301	HLII	WP_011862476.1_454	TGGAGCTAGCAGAGGAATTGGTAAAGAAATTGCTTTAGAACTAAGTCGCTTTGGAGCAGA	gdh	3-oxoacyl-acyl-carrier-protein reductase	WP_011862476.:	418245..418994	Entner-Dudoroff	COG1028
WP_011862476.1_454	CUST_2441_PI442190907	MIT9301	HLII	WP_011862476.1_454	TCAAGTGATGCAGGAAGGTACATTACAGGTCAGACAATCAGTATAGACGGAGGTATGAGT	gdh	3-oxoacyl-acyl-carrier-protein reductase	WP_011862476.:	418245..418994	Entner-Dudoroff	COG1028
WP_011862476.1_454	CUST_2442_PI442190907	MIT9301	HLII	WP_011862476.1_454	TGCTGGTATTACTCGAGATGGACTATTGATGAGAATGAAATCGGAACAATGGGATGATGT	gdh	3-oxoacyl-acyl-carrier-protein reductase	WP_011862476.:	418245..418994	Entner-Dudoroff	COG1028
WP_011862476.1_454	CUST_2443_PI442190907	MIT9301	HLII	WP_011862476.1_454	AGTGGTAGTATCGTAAATATTTCATCTGTTGTTGGAATAATTGGTAATCCCGGTCAAGCA	gdh	3-oxoacyl-acyl-carrier-protein reductase	WP_011862476.:	418245..418994	Entner-Dudoroff	COG1028
WP_011862476.1_454	CUST_2444_PI442190907	MIT9301	HLII	WP_011862476.1_454	TTTCAAGAGAGGATTCTGTCAGTTCAGCTTTTGAAGAAATCATAAAAGTTAATGGCTCCA	gdh	3-oxoacyl-acyl-carrier-protein reductase	WP_011862476.:	418245..418994	Entner-Dudoroff	COG1028
WP_011862538.1_520	CUST_2800_PI442190907	MIT9301	HLII	WP_011862538.1_520	CGTGTATCTACAGAAGTTGATGCAAGACTGAGCTTTGACACCCAAGCTACGGTAGAAAAA	tal	Transaldolase	WP_011862538.:	475195..476196	Pentose Phosphate	COG0176
WP_011862538.1_520	CUST_2801_PI442190907	MIT9301	HLII	WP_011862538.1_520	AGATTGCTGCAACTTGGGAGGGAATTAAGGCAGCTGAAATTTTGGAAAAAGAGGGTATTA	tal	Transaldolase	WP_011862538.:	475195..476196	Pentose Phosphate	COG0176
WP_011862538.1_520	CUST_2802_PI442190907	MIT9301	HLII	WP_011862538.1_520	TTGTGCCAATGCAAAGATAACTCTAATTTCTCCTTTCGTTGGCCGTATATTGGATTGGCA	tal	Transaldolase	WP_011862538.:	475195..476196	Pentose Phosphate	COG0176
WP_011862538.1_520	CUST_2803_PI442190907	MIT9301	HLII	WP_011862538.1_520	AAAGCTTAGTGAAGGCATCACTGGATTCAGTAAGGCTATAGAAGAATTGGAAGAGCTGCT	tal	Transaldolase	WP_011862538.:	475195..476196	Pentose Phosphate	COG0176
WP_011862538.1_520	CUST_2804_PI442190907	MIT9301	HLII	WP_011862538.1_520	TGTTGGCGCTGAAGACCCTGGTGTTATTTCGGTTACGCAAATATATAAGTACTTTAAAGA	tal	Transaldolase	WP_011862538.:	475195..476196	Pentose Phosphate	COG0176
WP_011862538.1_520	CUST_2805_PI442190907	MIT9301	HLII	WP_011862538.1_520	AGCAGGTTGCGATCTTTTAACAATTGCACCAAAATTTCTTGAGGAGCTGAAAAAAGAAAA	tal	Transaldolase	WP_011862538.:	475195..476196	Pentose Phosphate	COG0176
WP_011862567.1_551	CUST_2978_PI442190907	MIT9301	HLII	WP_011862567.1_551	TGCTCTAGAAACTTGGAAGGAAATTAAGTTCGAGTTTGACACTGTCGACAAGCTTGACGT	rbcL	Ribulose 1,5-bisphosphate carboxylase large subunit	WP_011862567.:	503604..505019	Carbon fixation	COG1850
WP_011862568.1_552	CUST_2982_PI442190907	MIT9301	HLII	WP_011862568.1_552	ATCGGATACGATGCTTACACTCAAAGTCAAGGAACAGCTTTTGTAGTTTTCCAAGGACGT	rbcS	Ribulose bisphosphate carboxylase small subunit	WP_011862568.:	505109..505450	Carbon fixation	COG4451
WP_011862568.1_552	CUST_2983_PI442190907	MIT9301	HLII	WP_011862568.1_552	ACTCCCATTCTTTGGGGAAAAAGATCTTAACTTGATCGTGAGCGAATTAGAGGCATGTCA	rbcS	Ribulose bisphosphate carboxylase small subunit	WP_011862568.:	505109..505450	Carbon fixation	COG4451
WP_011862568.1_552	CUST_2984_PI442190907	MIT9301	HLII	WP_011862568.1_552	AGTCCAGTTATTGAGCATGTTCATCCAAGTGGATGTATGCAAACTTATTGGTCTTATTGG	rbcS	Ribulose bisphosphate carboxylase small subunit	WP_011862568.:	505109..505450	Carbon fixation	COG4451
WP_011862568.1_552	CUST_2985_PI442190907	MIT9301	HLII	WP_011862568.1_552	CCACCGATGACCCAGGAAGAAATATATGACCAAATTGCGTACATAATTGCTCAAGGTTGG	rbcS	Ribulose bisphosphate carboxylase small subunit	WP_011862568.:	505109..505450	Carbon fixation	COG4451
WP_011862568.1_552	CUST_2986_PI442190907	MIT9301	HLII	WP_011862568.1_552	TTCCAGAGCACAGTAAGCGACTATCAAACAGTTGCAACCCTGGAAACATTCGGTTTCTTA	rbcS	Ribulose bisphosphate carboxylase small subunit	WP_011862568.:	505109..505450	Carbon fixation	COG4451
WP_011862612.1_597	CUST_3222_PI442190907	MIT9301	HLII	WP_011862612.1_597	TCTGAAGATACTAATAATGATTTGCATGTCATTGTTGGAGCGGGGACTGGCTTAGGGATT	glk	Glucokinase	WP_011862612.:	549579..550613	Glycolysis	COG0837
WP_011862612.1_597	CUST_3223_PI442190907	MIT9301	HLII	WP_011862612.1_597	GATATCTTGTGAAAGAATTGTTAGCGGCACTGGTTTATCAAGAATTGCCGAATGGAGGCT	glk	Glucokinase	WP_011862612.:	549579..550613	Glycolysis	COG0837
WP_011862612.1_597	CUST_3224_PI442190907	MIT9301	HLII	WP_011862612.1_597	GAGGATTTGGTTAGGCGCTTATGCCTCATTATTGGGAGATGTTGCTCTTCAAGAATTGTG	glk	Glucokinase	WP_011862612.:	549579..550613	Glycolysis	COG0837
WP_011862612.1_597	CUST_3225_PI442190907	MIT9301	HLII	WP_011862612.1_597	CAGGGGAAAAGGTAAAAGTTTTAGCTAGTGAAGGTGGTCATGTAGAGTACTCCCCAAAGT	glk	Glucokinase	WP_011862612.:	549579..550613	Glycolysis	COG0837
WP_011862612.1_597	CUST_3226_PI442190907	MIT9301	HLII	WP_011862612.1_597	AGATGAAGAGTTTGGACTTTTTAGTGCAGCCTGCAGAGCAAAAATGCTTTTAAAAACTTA	glk	Glucokinase	WP_011862612.:	549579..550613	Glycolysis	COG0837
WP_011862612.1_597	CUST_3227_PI442190907	MIT9301	HLII	WP_011862612.1_597	TGGTGGATTATGGATTTCTGGAGGAACTGCGTCAAAACATTTCAAAAACTTTAAATCAGA	glk	Glucokinase	WP_011862612.:	549579..550613	Glycolysis	COG0837
WP_011862806.1_787	CUST_4223_PI442190907	MIT9301	HLII	WP_011862806.1_787	TATTGGGAAGATATTGGAACCATTGGGGCATTCTTTGAGTCAAACCTTGCATTGACTGAG	glgC	Glucose-1-phosphate adenylyltransferase	WP_011862806.:	720123..721418	Glycogen Metabolism	COG0448
WP_011862806.1_787	CUST_4224_PI442190907	MIT9301	HLII	WP_011862806.1_787	GTGACTCAGTTCTTGAGGATACTCTTGTTATGGGAGCCGATTTCTTTGAATCCCCTGAAG	glgC	Glucose-1-phosphate adenylyltransferase	WP_011862806.:	720123..721418	Glycogen Metabolism	COG0448
WP_011862806.1_787	CUST_4225_PI442190907	MIT9301	HLII	WP_011862806.1_787	GTGAAAAGCCCACTGGAGAGAAGTTGAAGGCAATGGCAGTAGATACTTCAAAATTTGGAT	glgC	Glucose-1-phosphate adenylyltransferase	WP_011862806.:	720123..721418	Glycogen Metabolism	COG0448
WP_011862806.1_787	CUST_4226_PI442190907	MIT9301	HLII	WP_011862806.1_787	AGGAGTCAGCTGCCGAAAAACCATATCTAGCCTCTATGGGTATTTACGTTTTTAGCAGAA	glgC	Glucose-1-phosphate adenylyltransferase	WP_011862806.:	720123..721418	Glycogen Metabolism	COG0448
WP_011862806.1_787	CUST_4227_PI442190907	MIT9301	HLII	WP_011862806.1_787	CGGAACGCCTCTTGGAGTAGGTGAAGGGACTACTGTAAAAAGAGCAATTCTTGATAAGAA	glgC	Glucose-1-phosphate adenylyltransferase	WP_011862806.:	720123..721418	Glycogen Metabolism	COG0448
WP_011862806.1_787	CUST_4228_PI442190907	MIT9301	HLII	WP_011862806.1_787	ACCCCCTTCTAAGCTTGTAGATGCACAAATTACTGATTCAATTGTTGTGAAGGTACAAT	glgC	Glucose-1-phosphate adenylyltransferase	WP_011862806.:	720123..721418	Glycogen Metabolism	COG0448
WP_011862807.1_788	CUST_4229_PI442190907	MIT9301	HLII	WP_011862807.1_788	TAGAACCAATAGACTTCCTCAGAATCTCGTTCAGGCAATGAGAGACTGTTTTGGCTCTCA	edd	phosphogluconate dehydrogenase (NADP(+)-dependent, decarboxyla	WP_011862807.:	721514..722932	Pentose Phosphate/Entner-Dudorof	COG2084
WP_011862807.1_788	CUST_4230_PI442190907	MIT9301	HLII	WP_011862807.1_788	AGCTAAAGTAGTCTCTCTATCCACAAAGGCAGGTATACCAGTGCCATGTTTATCCAGTAC	edd	phosphogluconate dehydrogenase (NADP(+)-dependent, decarboxyla	WP_011862807.:	721514..722932	Pentose Phosphate/Entner-Dudorof	COG2084
WP_011862807.1_788	CUST_4231_PI442190907	MIT9301	HLII	WP_011862807.1_788	ATGAAACCTTTATTTGATGCTGTAGTCCTTGCCACAATTGCTAGCTATGCCCAAGGTATG	edd	phosphogluconate dehydrogenase (NADP(+)-dependent, decarboxyla	WP_011862807.:	721514..722932	Pentose Phosphate/Entner-Dudorof	COG2084
WP_011862807.1_788	CUST_4232_PI442190907	MIT9301	HLII	WP_011862807.1_788	ACAGGGTTATGGACTGTTGTAAGTGCTCTAGAACTGGGGGTATCAGTCCCAACTATTTAT	edd	phosphogluconate dehydrogenase (NADP(+)-dependent, decarboxyla	WP_011862807.:	721514..722932	Pentose Phosphate/Entner-Dudorof	COG2084
WP_011862807.1_788	CUST_4233_PI442190907	MIT9301	HLII	WP_011862807.1_788	GATGAGATAACTGGAGATGATGTCGTGGAAAAAATATTAGATAAAGCTGGCCAGAAAGGT	edd	phosphogluconate dehydrogenase (NADP(+)-dependent, decarboxyla	WP_011862807.:	721514..722932	Pentose Phosphate/Entner-Dudorof	COG2084
WP_011862807.1_788	CUST_4234_PI442190907	MIT9301	HLII	WP_011862807.1_788	TGCAGAAGCTTATGACCTTATGAAGAGAGTCAAAGGTATGAATGGACAGCAGATGTCAGA	edd	phosphogluconate dehydrogenase (NADP(+)-dependent, decarboxyla	WP_011862807.:	721514..722932	Pentose Phosphate/Entner-Dudorof	COG2084
WP_011376344.1_799	CUST_4291_PI442190907	MIT9301	HLII	WP_011376344.1_799	GAAATGGAGTTAGAAAAGTCAACATTGATACCGATAACAGACTTGCTTTCACTGCCGCGG	cbbA	Fructose-1,6-bisphosphate aldolase class II	WP_011376344.:	730199..731272	Glycolysis/Gluconeogenesis	COG0191
WP_011376344.1_799	CUST_4292_PI442190907	MIT9301	HLII	WP_011376344.1_799	AGTTTGTCTTGATAGATACAAGCAGTTCTGGTGCGAAGGTCAAGCAAGTAAGATCAAGCA	cbbA	Fructose-1,6-bisphosphate aldolase class II	WP_011376344.:	730199..731272	Glycolysis/Gluconeogenesis	COG0191
WP_011376344.1_799	CUST_4293_PI442190907	MIT9301	HLII	WP_011376344.1_799	CTCCCAAATACCCATCTTGTAATGCATGGATCTAGTTCAGTTCCTCAGGAATGGTTGGAT	cbbA	Fructose-1,6-bisphosphate aldolase class II	WP_011376344.:	730199..731272	Glycolysis/Gluconeogenesis	COG0191
WP_011376344.1_799	CUST_4294_PI442190907	MIT9301	HLII	WP_011376344.1_799	CAGCATTTGCTGATAAGGCAAACTTTGACCCAAGACATTTCAACAAGCCTGCTAGAAAAT	cbbA	Fructose-1,6-bisphosphate aldolase class II	WP_011376344.:	730199..731272	Glycolysis/Gluconeogenesis	COG0191
WP_011818291.1_803	CUST_4312_PI442190907	MIT9301	HLII	WP_011818291.1_803	CGTACTTTTTTATGAAGGTTTGCATGGCGGGGTAGAAGGTGATGGATATAATGTGGCATC	prk	Phosphoribulokinase	WP_011818291.:	733884..734780	Carbon Fixation	COG3954
WP_011818291.1_803	CUST_4313_PI442190907	MIT9301	HLII	WP_011818291.1_803	TCCTGATGAGAGTTTTGTGATCATACATTTCAGAAAAGGGGCAAGAGAGAAATGGGGGAT	prk	Phosphoribulokinase	WP_011818291.:	733884..734780	Carbon Fixation	COG3954
WP_011818291.1_803	CUST_4314_PI442190907	MIT9301	HLII	WP_011818291.1_803	ACAATACAAGACTTGGGACATCCTTAGAACCAGGGCAATTCACTCCATGGGAAGATATTC	prk	Phosphoribulokinase	WP_011818291.:	733884..734780	Carbon Fixation	COG3954
WP_011818291.1_803	CUST_4315_PI442190907	MIT9301	HLII	WP_011818291.1_803	AATGCAGAAAGAGGGTACTCAGCTGAAACCATTGTGGATACCATATTGAGAAGAATGCCT	prk	Phosphoribulokinase	WP_011818291.:	733884..734780	Carbon Fixation	COG3954
WP_011818291.1_803	CUST_4316_PI442190907	MIT9301	HLII	WP_011818291.1_803	AGGTGATAGTTACCACAGGTTTGAAAGAATGCCTATGAAAAAGCTATGGCAGAGGCTCT	prk	Phosphoribulokinase	WP_011818291.:	733884..734780	Carbon Fixation	COG3954
WP_011818291.1_803	CUST_4317_PI442190907	MIT9301	HLII	WP_011818291.1_803	TTATGTCAAGTCCCACAAGTATCGTTGTAAATGGAGGGAAAATGGGATTCGCAATGGAAC	prk	Phosphoribulokinase	WP_011818291.:	733884..734780	Carbon Fixation	COG3954
WP_011862899.1_879	CUST_4718_PI442190907	MIT9301	HLII	WP_011862899.1_879	TGGGTGCAGCAATGACTGGCTTAAGACCAATAGTAGAAGGAATGAATATGGTTTTTGC	pdhB	Pyruvate dehydrogenase E1 component beta subunit	WP_011862899.:	798152..799135	Glycolysis/Krebs cycle	COG0022
WP_011862899.1_879	CUST_4719_PI442190907	MIT9301	HLII	WP_011862899.1_879	TGGCATAAAAATCGTTGCATGTAGTACACCCACAAATGCCAAAGGTTTAATGAAAGCTGC	pdhB	Pyruvate dehydrogenase E1 component beta subunit	WP_011862899.:	798152..799135	Glycolysis/Krebs cycle	COG0022
WP_011862899.1_879	CUST_4720_PI442190907	MIT9301	HLII	WP_011862899.1_879	ACCTGAGGGTGATTATATTTGCGCTTTAGATCAGGCTGACGTTGTAAAAGCAGGTAAAGA	pdhB	Pyruvate dehydrogenase E1 component beta subunit	WP_011862899.:	798152..799135	Glycolysis/Krebs cycle	COG0022
WP_011862899.1_879	CUST_4721_PI442190907	MIT9301	HLII	WP_011862899.1_879	GGTGCAGAATTAATTGCCTTGATAACAGAAGAGTGTTTTGATGATCTTGATGCCCGACCA	pdhB	Pyruvate dehydrogenase E1 component beta subunit	WP_011862899.:	798152..799135	Glycolysis/Krebs cycle	COG0022
WP_011862899.1_879	CUST_4722_PI442190907	MIT9301	HLII	WP_011862899.1_879	ATATACAAGTGGCGGAAATTATAAATACCAACAGTAGTTCGAGGCCCTGGAGGAGTTGG	pdhB	Pyruvate dehydrogenase E1 component beta subunit	WP_011862899.:	798152..799135	Glycolysis/Krebs cycle	COG0022
WP_011862899.1_879	CUST_4723_PI442190907	MIT9301	HLII	WP_011862899.1_879	TTGGGCGTCAACTTGGTGCTGAGCATAGTCAAAGACTTGAAGCATATTTTCATGCG	pdhB	Pyruvate dehydrogenase E1 component beta subunit	WP_011862899.:	798152..799135	Glycolysis/Krebs cycle	COG0022
WP_011862910.1_890	CUST_4775_PI442190907	MIT9301	HLII	WP_011862910.1_890	TTTTAACGATAACGGAACAGGAATGCATGCTCACCAAAGTTTATGGAAGAGTGGACAGC	glnA	Glutamine synthetase/ glutamate--ammonialigase	WP_011862910.:	806913..808334	Nitrogen Metabolism	COG0174
WP_011862910.1_890	CUST_4776_PI442190907	MIT9301	HLII	WP_011862910.1_890	GTGCAGGCCAACACGAGCTTGGAATGAAATTTGATTCGTTAATAAATGCTGCTGATAACG	glnA	Glutamine synthetase/ glutamate--ammonialigase	WP_011862910.:	806913..808334	Nitrogen Metabolism	COG0174
WP_011862910.1_890	CUST_4777_PI442190907	MIT9301	HLII	WP_011862910.1_890	CAGGTGACGCACTTGCAAATCCTTACTTAGCATTCTCTGTAATGATGCTTGCTGGTATTG	glnA	Glutamine synthetase/ glutamate--ammonialigase	WP_011862910.:	806913..808334	Nitrogen Metabolism	COG0174
WP_011862910.1_890	CUST_4778_PI442190907	MIT9301	HLII	WP_011862910.1_890	AGATGGTACATCGGAGGCATACTTAAACATGCGCCATCATTCTTAGCATTTACTAACCCA	glnA	Glutamine synthetase/ glutamate--ammonialigase	WP_011862910.:	806913..808334	Nitrogen Metabolism	COG0174
WP_011862910.1_890	CUST_4779_PI442190907	MIT9301	HLII	WP_011862910.1_890	TCCAGGATTCGAAGCACCAGTAAATCTAGTTTATTCTGAGGGTAATAGATCAGCTGCGGT	glnA	Glutamine synthetase/ glutamate--ammonialigase	WP_011862910.:	806913..808334	Nitrogen Metabolism	COG0174
WP_011862910.1_890	CUST_4780_PI442190907	MIT9301	HLII	WP_011862910.1_890	GAGGTACAACAACTAAGACAAAGGCCTCATCCACATGAATTCTTCATGTATTACGATGCA	glnA	Glutamine synthetase/ glutamate--ammonialigase	WP_011862910.:	806913..808334	Nitrogen Metabolism	COG0174
WP_011862918.1_898	CUST_4820_PI442190907	MIT9301	HLII	WP_011862918.1_898	CCCAGTACGATCCCAAATGCTATTAGCGCAGCAGTAAGCAATATAGCTAGACAACTTGAA	pykF	Pyruvate kinase	WP_011862918.:	815554..817344	Glycolysis	COG0469
WP_011862918.1_898	CUST_4821_PI442190907	MIT9301	HLII	WP_011862918.1_898	GCAACTACTACAGAAAGAAGTGTAGCGAGAAGATTGCAACTTGTTTGGGGAGTTACTCCA	pykF	Pyruvate kinase	WP_011862918.:	815554..817344	Glycolysis	COG0469
WP_011862918.1_898	CUST_4822_PI442190907	MIT9301	HLII	WP_011862918.1_898	ACTAAATCAGGTTCTACCGCTCGAAATGTAAGTAAGTTCAGACCACCAACACCCATCTTG	pykF	Pyruvate kinase	WP_011862918.:	815554..817344	Glycolysis	COG0469
WP_011862918.1_898	CUST_4823_PI442190907	MIT9301	HLII	WP_011862918.1_898	GCAAATTGCTCAAGAGATGGGGATCCTTAATCAAGGAGATTTAGTAGTTCAAACCGCAGG	pykF	Pyruvate kinase	WP_011862918.:	815554..817344	Glycolysis	COG0469
WP_011862918.1_898	CUST_4824_PI442190907	MIT9301	HLII	WP_011862918.1_898	TGGAGATCTCATTACTCTCCAGCTTAATGAAGGTGTCATATACATGGGCCAAATTGAAGA	pykF	Pyruvate kinase	WP_011862918.:	815554..817344	Glycolysis	COG0469
WP_011862918.1_898	CUST_4825_PI442190907	MIT9301	HLII	WP_011862918.1_898	AGAAGGATTGTATCAAGAGGAATTTCAATAGGGGAAATCGGTGTTACAGGTAAAGCAAGA	pykF	Pyruvate kinase	WP_011862918.:	815554..817344	Glycolysis	COG0469
WP_011862940.1_920	CUST_4938_PI442190907	MIT9301	HLII	WP_011862940.1_920	AAATGCATATGATCAACCTGGAGTTGAAGCTGGGAAGAAAGCAGCCGCAAATATTATTGA	pgi	Glucose-6-phosphate isomerase	WP_011862940.:	836239..837822	Glycolysis/Gluconeogenesis	COG0166
WP_011862940.1_920	CUST_4939_PI442190907	MIT9301	HLII	WP_011862940.1_920	TTTCACTAGGTGCCTTAATCGCTTTATTTGAGAGGGCTGTATCCTTCTACGCTGAATTGG	pgi	Glucose-6-phosphate isomerase	WP_011862940.:	836239..837822	Glycolysis/Gluconeogenesis	COG0166
WP_011862940.1_920	CUST_4940_PI442190907	MIT9301	HLII	WP_011862940.1_920	CAGCAGCACTATTGTCATCAGCATGGTATTTAACTGGTGATGGCACTGGAAAGAGAGATA	pgi	Glucose-6-phosphate isomerase	WP_011862940.:	836239..837822	Glycolysis/Gluconeogenesis	COG0166
WP_011862940.1_920	CUST_4941_PI442190907	MIT9301	HLII	WP_011862940.1_920	ACTCCGTGAAATGTGTTTCGGTAATGATAATTATTTGGTTAAGGGCGATTGGTCAAATCC	pgi	Glucose-6-phosphate isomerase	WP_011862940.:	836239..837822	Glycolysis/Gluconeogenesis	COG0166
WP_011862940.1_920	CUST_4942_PI442190907	MIT9301	HLII	WP_011862940.1_920	TTCCGTTTTGGTAATAAAGGATCTACAGATCAACATGCTTATGTTCAGCAACTCAGAGA	pgi	Glucose-6-phosphate isomerase	WP_011862940.:	836239..837822	Glycolysis/Gluconeogenesis	COG0166
WP_011862940.1_920	CUST_4943_PI442190907	MIT9301	HLII	WP_011862940.1_920	AGGAACCAGATCAGCACTCTCTAGTGAAAACAGACAAAGTATCACCATAACATTAGAAAA	pgi	Glucose-6-phosphate isomerase	WP_011862940.:	836239..837822	Glycolysis/Gluconeogenesis	COG0166
WP_011863103.1_1080	CUST_5699_PI442190907	MIT9301	HLII	WP_011863103.1_1080	AACCGAGGGAGAGAAGGGAGGCATAATATTCCCAGGAGGAAGATATAAGGCTCTTAAAAA	rbsK	Sugar kinase, ribokinase family	WP_011863103.:	963338..964207	Pentose Phosphate	COG0524
WP_011863103.1_1080	CUST_5700_PI442190907	MIT9301	HLII	WP_011863103.1_1080	TTCTCATTCTGAAAATCTCTTGAGTATCCAGCTGGTGGCGGCTCTATTATCGCGAAAAT	rbsK	Sugar kinase, ribokinase family	WP_011863103.:	963338..964207	Pentose Phosphate	COG0524
WP_011863103.1_1080	CUST_5701_PI442190907	MIT9301	HLII	WP_011863103.1_1080	TCTTATGGATGTGGCGATTCATTTGCTGCTGGGATTCTTTATGGAATGGCATCTAAATGG	rbsK	Sugar kinase, ribokinase family	WP_011863103.:	963338..964207	Pentose Phosphate	COG0524
WP_011863103.1_1080	CUST_5702_PI442190907	MIT9301	HLII	WP_011863103.1_1080	TGCTAAAGTAATGGGAAGAGACGCAAGTGAATTTTTCGGCCCATATGCAAATAGTGATGA	rbsK	Sugar kinase, ribokinase family	WP_011863103.:	963338..964207	Pentose Phosphate	COG0524
WP_011863103.1_1080	CUST_5703_PI442190907	MIT9301	HLII	WP_011863103.1_1080	AATTGACTCTCAAGGTGAAAGAGCAATAACAGTTATTGGTGAAAGGTTAGCTCCAACTCA	rbsK	Sugar kinase, ribokinase family	WP_011863103.:	963338..964207	Pentose Phosphate	COG0524
WP_011863103.1_1080	CUST_5704_PI442190907	MIT9301	HLII	WP_011863103.1_1080	CTCTCAAATATGGGAATTAAGATGCATGTAGCTTGGCGTGATAAACCAACTAGAAGAGGA	rbsK	Sugar kinase, ribokinase family	WP_011863103.:	963338..964207	Pentose Phosphate	COG0524
WP_011863131.1_1109	CUST_5862_PI442190907	MIT9301	HLII	WP_011863131.1_1109	TAACACTTGTGTAATACTTGGCTGTGAATCAGTGGAATGTATGAGACTTGAAGCAGGGGG	opcA	Glucose-6-P dehydrogenase subunit opcA	WP_011863131.:	991842..993143	Pentose Phosphate	COG3429
WP_011863131.1_1109	CUST_5863_PI442190907	MIT9301	HLII	WP_011863131.1_1109	ATCTATTGAGGAATTACCTTCATGGTTGTGGTGGAATGGAAGCTTGGATGAATCACCTGA	opcA	Glucose-6-P dehydrogenase subunit opcA	WP_011863131.:	991842..993143	Pentose Phosphate	COG3429
WP_011863131.1_1109	CUST_5864_PI442190907	MIT9301	HLII	WP_011863131.1_1109	AATTGGGTTAGGTTGAAAAATTGGAGGGAATCATTGGCAATGATTTTTGATCCGCCTTCG	opcA	Glucose-6-P dehydrogenase subunit opcA	WP_011863131.:	991842..993143	Pentose Phosphate	COG3429
WP_011863131.1_1109	CUST_5865_PI442190907	MIT9301	HLII	WP_011863131.1_1109	TCTTTGGGGAATCCAAGTATTCATTCAGGACAAGTGATTGGATTGAGGTTGATTTCAAGA	opcA	Glucose-6-P dehydrogenase subunit opcA	WP_011863131.:	991842..993143	Pentose Phosphate	COG3429
WP_011863131.1_1109	CUST_5866_PI442190907	MIT9301	HLII	WP_011863131.1_1109	CGCAGGGGATCATATGATTCAAGCTTTGTTTTTGATCTCATGGATTAGCGATAAACTTGG	opcA	Glucose-6-P dehydrogenase subunit opcA	WP_011863131.:	991842..993143	Pentose Phosphate	COG3429
WP_011863131.1_1109	CUST_5867_PI442190907	MIT9301	HLII	WP_011863131.1_1109	GGGGTACAGAATCAACAAAAATAGAGTTTGAAAGAATTAATGGCGAAATAATTTCTGCGT	opcA	Glucose-6-P dehydrogenase subunit opcA	WP_011863131.:	991842..993143	Pentose Phosphate	COG3429
WP_011863132.1_1110	CUST_5868_PI442190907	MIT9301	HLII	WP_011863132.1_1110	AAAACTTGCTGACGAAAATGAACCGTGGAATTGTTGCATAAGAGGTCAATATGGAGAGGG	zwf	glucose-6-phosphate dehydrogenase zwf	WP_011863132.:	993146..994669	Pentose Phosphate	COG0364
WP_011863132.1_1110	CUST_5869_PI442190907	MIT9301	HLII	WP_011863132.1_1110	TTCAAAACCATATGACACAAATGCTTGCTGTTACTGCTATGGAACCTCCTGGGAAGTTTG	zwf	glucose-6-phosphate dehydrogenase zwf	WP_011863132.:	993146..994669	Pentose Phosphate	COG0364
WP_011863132.1_1110	CUST_5870_PI442190907	MIT9301	HLII	WP_011863132.1_1110	ATAAATCCTGCCCCAAATCAACTTATCCTTAGAATTCAGCCAAATGAAGGGGCGACTTTC	zwf	glucose-6-phosphate dehydrogenase zwf	WP_011863132.:	993146..994669	Pentose Phosphate	COG0364
WP_011863132.1_1110	CUST_5871_PI442190907	MIT9301	HLII	WP_011863132.1_1110	CTGACCCAACTTTATTTACTCGAAGCGATGAAGTAGAGGCAGCCTGGAAACTTATACCC	zwf	glucose-6-phosphate dehydrogenase zwf	WP_011863132.:	993146..994669	Pentose Phosphate	COG0364
WP_011863132.1_1110	CUST_5872_PI442190907	MIT9301	HLII	WP_011863132.1_1110	ATGACGTGGGGACCTCCTGAATCTGATCAATTACTCTCAAAAGATAATATTTTCTGGCGT	zwf	glucose-6-phosphate dehydrogenase zwf	WP_011863132.:	993146..994669	Pentose Phosphate	COG0364
WP_011863132.1_1110	CUST_5873_PI442190907	MIT9301	HLII	WP_011863132.1_1110	AGGCAGGAAGATGGTGTTAATTCCAATAGCACAACAGAAACTTATATCGCGACAAAAGTT	zwf	glucose-6-phosphate dehydrogenase zwf	WP_011863132.:	993146..994669	Pentose Phosphate	COG0364
WP_011863330.1_1308	CUST_6853_PI442190907	MIT9301	HLII	WP_011863330.1_1308	TATTTGCGAAAAGACTGGTGCAGAAATCAAAGAGGTTTCTGCTGCTATTGGTTCCGATAC	ugd	UDP-glucose 6-dehydrogenase/nucleotide sugar dehydrogenase	WP_011863330.:	1165603..1167018	Amino sugar and nucleotide sugar r	COG1004
WP_011863330.1_1308	CUST_6854_PI442190907	MIT9301	HLII	WP_011863330.1_1308	GGAAGACATTTTGGAGGCTTGTAAATCAGCCGATGCAATAGTTATCTTAACTGAGTGGCA	ugd	UDP-glucose 6-dehydrogenase/nucleotide sugar dehydrogenase	WP_011863330.:	1165603..1167018	Amino sugar and nucleotide sugar r	COG1004
WP_011863330.1_1308	CUST_6855_PI442190907	MIT9301	HLII	WP_011863330.1_1308	AGGATTGGAAATAAATTCTTATTATCAAGTCCTGGTTTTGGCGGGAGCTGCTTCCAAAAA	ugd	UDP-glucose 6-dehydrogenase/nucleotide sugar dehydrogenase	WP_011863330.:	1165603..1167018	Amino sugar and nucleotide sugar r	COG1004
WP_011863330.1_1308	CUST_6856_PI442190907	MIT9301	HLII	WP_011863330.1_1308	GCCTTCTTGGGTTTTTGATACAAGAGGCATAATTAATCGTAGGAAATCGATGTAACTCC	ugd	UDP-glucose 6-dehydrogenase/nucleotide sugar dehydrogenase	WP_011863330.:	1165603..1167018	Amino sugar and nucleotide sugar r	COG1004
WP_011863330.1_1308	CUST_6857_PI442190907	MIT9301	HLII	WP_011863330.1_1308	TGGAGCAGTGAATTGTCTAAATTAACAGCAAATGCTTTTTTAGCCCAAAAGAAATAAGTTCA	ugd	UDP-glucose 6-dehydrogenase/nucleotide sugar dehydrogenase	WP_011863330.:	1165603..1167018	Amino sugar and nucleotide sugar r	COG1004
WP_011863330.1_1308	CUST_6858_PI442190907	MIT9301	HLII	WP_011863330.1_1308	GATCATGTCGCAAAATATTGGGAAAATGTAATTTCTTTGAATAATTGGCAAAAGGAACGT	ugd	UDP-glucose 6-dehydrogenase/nucleotide sugar dehydrogenase	WP_011863330.:	1165603..1167018	Amino sugar and nucleotide sugar r	COG1004
WP_011863347.1_1325	CUST_6950_PI442190907	MIT9301	HLII	WP_011863347.1_1325	TTGAAGTGGTGCTCAAATGCTTATGGTTTGCGATGGGTATCTCTAAGGTATTTCAATGCC	galE	UDP-glucose 4-epimerase	WP_011863347.:	1181603..1182595	Galactose metabolism	COG1087
WP_011863347.1_1325	CUST_6951_PI442190907	MIT9301	HLII	WP_011863347.1_1325	ATGATCCTGAAACACATATAATTCCCTTGGCTATAAGGGCCTTAGGTGATTCTGGCGAAA	galE	UDP-glucose 4-epimerase	WP_011863347.:	1181603..1182595	Galactose metabolism	COG1087
WP_011863347.1_1325	CUST_6952_PI442190907	MIT9301	HLII	WP_011863347.1_1325	TGGATTTGGCTTCTGCCCACTTAAAAGCAATAGAATATTTAGCTGAAGGTGGAATGTCAA	galE	UDP-glucose 4-epimerase	WP_011863347.:	1181603..1182595	Galactose metabolism	COG1087
WP_011863347.1_1325	CUST_6953_PI442190907	MIT9301	HLII	WP_011863347.1_1325	AGCAGGTCAAATTAAAATACTGTGAAAGAAGAGAGGAAGACCCATCCTGTTTATTTGCTG	galE	UDP-glucose 4-epimerase	WP_011863347.:	1181603..1182595	Galactose metabolism	COG1087
WP_011863347.1_1325	CUST_6954_PI442190907	MIT9301	HLII	WP_011863347.1_1325	TCCTCTTCTTGTTTCAGTATATGGTGAAGCAAAAAATGTTCCAATTAATGAGTCAGAACCA	galE	UDP-glucose 4-epimerase	WP_011863347.:	1181603..1182595	Galactose metabolism	COG1087
WP_011863347.1_1325	CUST_6955_PI442190907	MIT9301	HLII	WP_011863347.1_1325	GAATTGGCAACCAGAATTTTCAAACTTAGATAATATTCTTAGATCAGCATGGAAATGGCA	galE	UDP-glucose 4-epimerase	WP_011863347.:	1181603..1182595	Galactose metabolism	COG1087
WP_011863356.1_1335	CUST_7007_PI442190907	MIT9301	HLII	WP_011863356.1_1335	ACTCATGATGGCACTGGAATTAGAGACTACATACATGTAATGGATCTTGCTGAAGGGCAT	galE	UDP-glucose 4-epimerase	WP_011863356.:	1191331..1192389	Galactose metabolism	COG1087
WP_011863356.1_1335	CUST_7008_PI442190907	MIT9301	HLII	WP_011863356.1_1335	GCTGAAGAAGAGAGGGTGATGTACCTATTTCAATCGCAGATAATTGTCTTGCCAAAACA	galE	UDP-glucose 4-epimerase	WP_011863356.:	1191331..1192389	Galactose metabolism	COG1087
WP_011863356.1_1335	CUST_7009_PI442190907	MIT9301	HLII	WP_011863356.1_1335	AATCCAATTGGTTGTCATAACTCAGGCCAGATAGGAGAAAGCCCTTTAAATAAACCAACT	galE	UDP-glucose 4-epimerase	WP_011863356.:	1191331..1192389	Galactose metabolism	COG1087
WP_011863356.1_1335	CUST_7010_PI442190907	MIT9301	HLII	WP_011863356.1_1335	[illegible]	galE	UDP-glucose 4-epimerase	WP_011863356.:	1191331..1192389	Galactose metabolism	COG1087
WP_011863356.1_1335	CUST_7011_PI442190907	MIT9301	HLII	WP_011863356.1_1335	[illegible]	galE	UDP-glucose 4-epimerase	WP_011863356.:	1191331..1192389	Galactose metabolism	COG1087
WP_011863356.1_1335	CUST_7012_PI442190907	MIT9301	HLII	WP_011863356.1_1335	[illegible]	galE	UDP-glucose 4-epimerase	WP_011863356.:	1191331..1192389	Galactose metabolism	COG1087
WP_011863403.1_1382	CUST_7280_PI442190907	MIT9301	HLII	WP_011863403.1_1382	[illegible]	pdhA	Pyruvate dehydrogenase E1 component alpha subunit	WP_011863403.:	1241667..1242740	Glycolysis/Krebs cycle	COG1071
WP_011863403.1_1382	CUST_7281_PI442190907	MIT9301	HLII	WP_011863403.1_1382	[illegible]	pdhA	Pyruvate dehydrogenase E1 component alpha subunit	WP_011863403.:	1241667..1242740	Glycolysis/Krebs cycle	COG1071
WP_011863403.1_1382	CUST_7282_PI442190907	MIT9301	HLII	WP_011863403.1_1382	[illegible]	pdhA	Pyruvate dehydrogenase E1 component alpha subunit	WP_011863403.:	1241667..1242740	Glycolysis/Krebs cycle	COG1071
WP_011863403.1_1382	CUST_7283_PI442190907	MIT9301	HLII	WP_011863403.1_1382	[illegible]	pdhA	Pyruvate dehydrogenase E1 component alpha subunit	WP_011863403.:	1241667..1242740	Glycolysis/Krebs cycle	COG1071

WP_011863403.1_1382	CUST_7284_PI442190907	MIT9301	HLII	WP_011863403.1_1382	TGATGCAGAGATATCTGAGTCAGTTAAAAATGCTATTGAAGCTCCCGAACCACCTTCAGA	pdhA	Pyruvate dehydrogenase E1 component alpha subunit	WP_011863403.:	1241667..1242740	Glycolysis/Krebs cycle	COG1071
WP_011863403.1_1382	CUST_7285_PI442190907	MIT9301	HLII	WP_011863403.1_1382	GCAGATCCAGATGAATTAAGGTCTGAAAAAGAGAAGGAGTTTTGGGGAAAAGAGATCCC	pdhA	Pyruvate dehydrogenase E1 component alpha subunit	WP_011863403.:	1241667..1242740	Glycolysis/Krebs cycle	COG1071
WP_011863425.1_1403	CUST_7403_PI442190907	MIT9301	HLII	WP_011863425.1_1403	GAATGCGTCAAGCAGAGGAAGGGATCGCAAGACTAGCTGAAAACGTTGATACTCTTATAG	ftsZ	cell division protein FtsZ	WP_011863425.:	1264859..1265974	Cell Division	COG0206
WP_011863425.1_1403	CUST_7404_PI442190907	MIT9301	HLII	WP_011863425.1_1403	TTAGATCAGTTATGACTGAGGCTGGCACTGCACTTCTTGGAATAGGTATAGGTTCAGGAA	ftsZ	cell division protein FtsZ	WP_011863425.:	1264859..1265974	Cell Division	COG0206
WP_011863425.1_1403	CUST_7405_PI442190907	MIT9301	HLII	WP_011863425.1_1403	CCAGGCAGCAATGAATAGTCCTTTATTAGAAGCAGCAAGAATTGATGGAGCTAAAGGCTG	ftsZ	cell division protein FtsZ	WP_011863425.:	1264859..1265974	Cell Division	COG0206
WP_011863425.1_1403	CUST_7406_PI442190907	MIT9301	HLII	WP_011863425.1_1403	GTCGATGAGGCGATGGAAGGGGAGATACAAGTTACTGTAATTGCTACAGGATTTGAAACA	ftsZ	cell division protein FtsZ	WP_011863425.:	1264859..1265974	Cell Division	COG0206
WP_011863425.1_1403	CUST_7407_PI442190907	MIT9301	HLII	WP_011863425.1_1403	CCCGTTGTTGCAGAAGTAGCCAAACAAAGTGGTGCTTTAACAGTAGGAATAGTAACCAAA	ftsZ	cell division protein FtsZ	WP_011863425.:	1264859..1265974	Cell Division	COG0206
WP_011863425.1_1403	CUST_7408_PI442190907	MIT9301	HLII	WP_011863425.1_1403	GCAGGAGCTCCTCTTCAAGAAGCATTTAGGAATGCTGATGATGTTCTAAGAATGGGAGTC	ftsZ	cell division protein FtsZ	WP_011863425.:	1264859..1265974	Cell Division	COG0206
WP_011863437.1_1418	CUST_7479_PI442190907	MIT9301	HLII	WP_011863437.1_1418	CATTGATTTCTTGTTGGGGATTAGCTCCATTTGCCAAAAAAGCAAGAATGCCTTCAGGAA	glcH	GPH family sugar transporter	WP_011863437.:	1277456..1278802	GlcH sugar transporter	COG2211
WP_011863437.1_1418	CUST_7480_PI442190907	MIT9301	HLII	WP_011863437.1_1418	AAGTGGATCCCGATACCTTTCCAAATTAGCGCTTTAGTGGGTTTACAAATATGGACCAGA	glcH	GPH family sugar transporter	WP_011863437.:	1277456..1278802	GlcH sugar transporter	COG2211
WP_011863437.1_1418	CUST_7481_PI442190907	MIT9301	HLII	WP_011863437.1_1418	TACTTCCTGATGCAATAGACGAAGACCCAGAGAAACCTGCAGGATTATATACTGCTTGGA	glcH	GPH family sugar transporter	WP_011863437.:	1277456..1278802	GlcH sugar transporter	COG2211
WP_011863437.1_1418	CUST_7482_PI442190907	MIT9301	HLII	WP_011863437.1_1418	TCTCCTCTGGTGCGCCTTACAGTTGATGCAAACAGTATCGTTAATCTATGTAGAGGATGT	glcH	GPH family sugar transporter	WP_011863437.:	1277456..1278802	GlcH sugar transporter	COG2211
WP_011863437.1_1418	CUST_7483_PI442190907	MIT9301	HLII	WP_011863437.1_1418	ACAGGATTTCAGCTTTAAACTATGGAGCGATTATGTGGATTGTTTCATGTACGGCAGCTT	glcH	GPH family sugar transporter	WP_011863437.:	1277456..1278802	GlcH sugar transporter	COG2211
WP_011863437.1_1418	CUST_7484_PI442190907	MIT9301	HLII	WP_011863437.1_1418	TTTTCATAATCTGTCTTATTGGAGTAGGAGCTTCAACCGCTTTTCTTATCCCTTGGTCAC	glcH	GPH family sugar transporter	WP_011863437.:	1277456..1278802	GlcH sugar transporter	COG2211
WP_011863455.1_1437	CUST_7592_PI442190907	MIT9301	HLII	WP_011863455.1_1437	CCAAAAAGAATGGCAATTGACTCTCTCTCTGCATTAGCCAGGGGTGTAAGTTTAAATGCA	kaiC	circadian clock protein KaiC	WP_011863455.:	1296596..1298125	Circadian Rhythm	COG0467
WP_011863455.1_1437	CUST_7593_PI442190907	MIT9301	HLII	WP_011863455.1_1437	TGGAGGAGGTTATTTTCAAGATTCCATTATTCTCGCCACTGGAGCTACTGGTACAGGTAA	kaiC	circadian clock protein KaiC	WP_011863455.:	1296596..1298125	Circadian Rhythm	COG0467
WP_011863455.1_1437	CUST_7594_PI442190907	MIT9301	HLII	WP_011863455.1_1437	AGGGCTCAATTACTAAGGAATGCGACTAGCTGGGGAATAGACTTTGAAAAAATGGAAAGT	kaiC	circadian clock protein KaiC	WP_011863455.:	1296596..1298125	Circadian Rhythm	COG0467
WP_011863455.1_1437	CUST_7595_PI442190907	MIT9301	HLII	WP_011863455.1_1437	ACAAGAAGAGATAGCAGGCTTTTTTACTAATACTGCAGAAGAATTTATGGGGAGCCACTC	kaiC	circadian clock protein KaiC	WP_011863455.:	1296596..1298125	Circadian Rhythm	COG0467
WP_011863455.1_1437	CUST_7596_PI442190907	MIT9301	HLII	WP_011863455.1_1437	TTTAGTGGTGCCCCTCATAGAGTTGTACCTGATCAAAATGTTCAAAATGTTTTTAAAGGA	kaiC	circadian clock protein KaiC	WP_011863455.:	1296596..1298125	Circadian Rhythm	COG0467
WP_011863455.1_1437	CUST_7597_PI442190907	MIT9301	HLII	WP_011863455.1_1437	CGGGGATCATGGCATGATAAACGAATTAGAGAATTTATTATTACAAATAGTGGCCCAGAA	kaiC	circadian clock protein KaiC	WP_011863455.:	1296596..1298125	Circadian Rhythm	COG0467
WP_011863456.1_1438	CUST_7598_PI442190907	MIT9301	HLII	WP_011863456.1_1438	TGGTTTAGATTTACTTTTTGATGAATTAACTGAAACTGAATATAGTGGAAATAAAT	kaiB	Circadian clock protein KaiB	WP_011863456.:	1298194..1298511	Circadian Rhythm	COG4251
WP_011863456.1_1438	CUST_7599_PI442190907	MIT9301	HLII	WP_011863456.1_1438	CCGCCACCAGTGAGAAAAATAATAGGTGATCTTTCAGATAGAGAGAAAGTTTTAATTGGT	kaiB	Circadian clock protein KaiB	WP_011863456.:	1298194..1298511	Circadian Rhythm	COG4251
WP_011863456.1_1438	CUST_7600_PI442190907	MIT9301	HLII	WP_011863456.1_1438	ATTGATGTACTTAAGCAACCACAACTTGCAGAAGAAGACAAGATTTTAGCCACCCCAACC	kaiB	Circadian clock protein KaiB	WP_011863456.:	1298194..1298511	Circadian Rhythm	COG4251
WP_011863456.1_1438	CUST_7601_PI442190907	MIT9301	HLII	WP_011863456.1_1438	TGAGAGCTTTAAATACCTTAAGAGAAATTTTAGAAAATGAATTTAAAGGAGTTTATGCTT	kaiB	Circadian clock protein KaiB	WP_011863456.:	1298194..1298511	Circadian Rhythm	COG4251
WP_011863456.1_1438	CUST_7602_PI442190907	MIT9301	HLII	WP_011863456.1_1438	TGGCTGCAAGGAAAACATATATTTTAAAACTCTATGTTGCTGGAAATACTCCTAATTCGA	kaiB	Circadian clock protein KaiB	WP_011863456.:	1298194..1298511	Circadian Rhythm	COG4251
WP_011863551.1_1517	CUST_7994_PI442190907	MIT9301	HLII	WP_011863551.1_1517	AAATCCAGAAAAAATTAAGGCCTTGAAAGAGGTTTCGAAGGCTGAGGCTAGGATTCAAGC	atpC	ATP synthase epsilon subunit	WP_011863551.:	1363319..1363723	Energy Metabolism	COG0355
WP_011863551.1_1517	CUST_7995_PI442190907	MIT9301	HLII	WP_011863551.1_1517	TGCTGAAATTGGATCTGAGATAAATGTTCAGAATGCTGAACAAGACCTTAAAGAAGCAAA	atpC	ATP synthase epsilon subunit	WP_011863551.:	1363319..1363723	Energy Metabolism	COG0355
WP_011863551.1_1517	CUST_7996_PI442190907	MIT9301	HLII	WP_011863551.1_1517	CCCAATGGAAATCAATAGCTCTAATGGGAGGCTTCGCTGAAATTGAATCAGATGAAGTCA	atpC	ATP synthase epsilon subunit	WP_011863551.:	1363319..1363723	Energy Metabolism	COG0355
WP_011863551.1_1517	CUST_7997_PI442190907	MIT9301	HLII	WP_011863551.1_1517	CCTGGACATATCTCTTTAGTTACTGCTATAGATATTGGTGTTTTAAGGGTTAGAA	atpC	ATP synthase epsilon subunit	WP_011863551.:	1363319..1363723	Energy Metabolism	COG0355
WP_011863551.1_1517	CUST_7998_PI442190907	MIT9301	HLII	WP_011863551.1_1517	AGGCGAAGCTGAAGAAGTAATCCTTCCTAGTACTACTGGTCAACTTGGTATACTACCTGG	atpC	ATP synthase epsilon subunit	WP_011863551.:	1363319..1363723	Energy Metabolism	COG0355
WP_011863563.1_1528	CUST_8054_PI442190907	MIT9301	HLII	WP_011863563.1_1528	AATTCTCTCCGTTGAACCTTGCAGAACAAGTTGCAGTTGTTTATGCAGGAGTTAAAGGTC	atpA	ATP synthase alpha subunit	WP_011863563.:	1373011..1374528	Energy Metabolism	COG0056
WP_011863563.1_1528	CUST_8055_PI442190907	MIT9301	HLII	WP_011863563.1_1528	GCTGCTTTTTCTCAATTTGCATCTGATCTTGATGAAGCAACTCAGCAACAACTTGAACGG	atpA	ATP synthase alpha subunit	WP_011863563.:	1373011..1374528	Energy Metabolism	COG0056
WP_011863563.1_1528	CUST_8056_PI442190907	MIT9301	HLII	WP_011863563.1_1528	ACAAGCTCAGGCTTATAGACAAATGTCTCTCCTTTTAAAAAGACCACCAGGAAGAGAGGC	atpA	ATP synthase alpha subunit	WP_011863563.:	1373011..1374528	Energy Metabolism	COG0056
WP_011863563.1_1528	CUST_8057_PI442190907	MIT9301	HLII	WP_011863563.1_1528	CAGCTCTTCCAATTATTGAAACTCAGGCAGGGGACGTTTCTGCTTACATTCCAACTAATG	atpA	ATP synthase alpha subunit	WP_011863563.:	1373011..1374528	Energy Metabolism	COG0056
WP_011863563.1_1528	CUST_8059_PI442190907	MIT9301	HLII	WP_011863563.1_1528	ACTTACACAGTAGATTACTAGAAAGAGCAGCAAAACTTTCTGATGCTATGGGAGGGGGAT	atpA	ATP synthase alpha subunit	WP_011863563.:	1373011..1374528	Energy Metabolism	COG0056
WP_011863567.1_1533	CUST_8081_PI442190907	MIT9301	HLII	WP_011863567.1_1533	CATTGATTTTCGCAACTTTAGCGGCCTACTACATCGGAGAAGCTGTTGAAGAACATCATT	atpI	ATP synthase subunit A	WP_011863567.:	1376551..1377276	Energy Metabolism	COG0356
WP_011863567.1_1533	CUST_8082_PI442190907	MIT9301	HLII	WP_011863567.1_1533	AGGCCTTCAAAACTTGCTTGAGTTTCTTTGGGATTACATAAGAGATCTTGCTAGGACGCA	atpI	ATP synthase subunit A	WP_011863567.:	1376551..1377276	Energy Metabolism	COG0356
WP_011863567.1_1533	CUST_8083_PI442190907	MIT9301	HLII	WP_011863567.1_1533	TGGGGAGGAGCTTTAATTCCTTGGAGATTGATTAAATTACCTAGTGGAGAATTAGGAGCA	atpI	ATP synthase subunit A	WP_011863567.:	1376551..1377276	Energy Metabolism	COG0356
WP_011863567.1_1533	CUST_8084_PI442190907	MIT9301	HLII	WP_011863567.1_1533	TGCAGGTTTAAGTAATAAGGGTTGGAGATACTTCGAATACTATGTTCACCCAACTCCGAT	atpI	ATP synthase subunit A	WP_011863567.:	1376551..1377276	Energy Metabolism	COG0356
WP_011863567.1_1533	CUST_8085_PI442190907	MIT9301	HLII	WP_011863567.1_1533	AGCATTAGAAGTTGGTCAACATCTTTATTGGCAAATTGGAAATATCAGACTACATGGGCA	atpI	ATP synthase subunit A	WP_011863567.:	1376551..1377276	Energy Metabolism	COG0356
WP_011863567.1_1533	CUST_8086_PI442190907	MIT9301	HLII	WP_011863567.1_1533	GTTGGTGTTTTAGTCTTTTTAGTTCCGCTAATTCTTCCAATACCTGTTATGTTCCTAGGA	atpI	ATP synthase subunit A	WP_011863567.:	1376551..1377276	Energy Metabolism	COG0356
WP_011863576.1_1543	CUST_8137_PI442190907	MIT9301	HLII	WP_011863576.1_1543	ATTACAGCCATAGTGCCAAAAGTTGGTTATGAAAAAGCAGCTGAAATTGCAAACCTTGCC	fumC	class II fumarate hydratase	WP_011863576.:	1384941..1386326	Krebs cycle and related genes	COG0114
WP_011863576.1_1543	CUST_8138_PI442190907	MIT9301	HLII	WP_011863576.1_1543	AGATAATGGGCCTTGAATATGCAGTTTCAATGGCAAATTCTAGCGGAACTTTGCAGATGA	fumC	class II fumarate hydratase	WP_011863576.:	1384941..1386326	Krebs cycle and related genes	COG0114
WP_011863576.1_1543	CUST_8139_PI442190907	MIT9301	HLII	WP_011863576.1_1543	GAATTAATCATCCCTCAAAATGAACCTGGAAGTTCTATCATGCCGGGCAAAGTGAATCCG	fumC	class II fumarate hydratase	WP_011863576.:	1384941..1386326	Krebs cycle and related genes	COG0114
WP_011863576.1_1543	CUST_8140_PI442190907	MIT9301	HLII	WP_011863576.1_1543	TGGCCTCGCATGATCGTTTAGCTCAAGTAATGAGTCAGATAAAAATATTAGCAGGTGCTT	fumC	class II fumarate hydratase	WP_011863576.:	1384941..1386326	Krebs cycle and related genes	COG0114
WP_011863576.1_1543	CUST_8141_PI442190907	MIT9301	HLII	WP_011863576.1_1543	ATTGTATTTTTTACCTCTGGGAGGCACTGCAGTTGGAACAGGGATTAATTGTCCAAAAGG	fumC	class II fumarate hydratase	WP_011863576.:	1384941..1386326	Krebs cycle and related genes	COG0114
WP_011863576.1_1543	CUST_8142_PI442190907	MIT9301	HLII	WP_011863576.1_1543	AATAGGAAGAACCCATTTTCAAGATGCAGTGCCCCTTACTTTTGGCCAAGAAATATCTGG	fumC	class II fumarate hydratase	WP_011863576.:	1384941..1386326	Krebs cycle and related genes	COG0114
WP_011863621.1_1589	CUST_8392_PI442190907	MIT9301	HLII	WP_011863621.1_1589	TAGGAAAATCGGGAGAAAGATTTGCGGTTAGAAATAGTGGTGCTACAGCGGTAACAGAAG	glsF	Ferredoxin-dependent glutamate synthase	WP_011863621.:	1435535..1440106	Nitrogen Metabolism	COG0069
WP_011863621.1_1589	CUST_8393_PI442190907	MIT9301	HLII	WP_011863621.1_1589	GGAAGGAATATTGGTGCGGGCATGACTGGCGGAATAGCTTTCATAATCGATGAAAATAAT	glsF	Ferredoxin-dependent glutamate synthase	WP_011863621.:	1435535..1440106	Nitrogen Metabolism	COG0069
WP_011863621.1_1589	CUST_8394_PI442190907	MIT9301	HLII	WP_011863621.1_1589	GGCTTCAATGGCGAACTAAACTTAAATTTCAAAGGATATGCAGGACAAAGCTTTGGTGCC	glsF	Ferredoxin-dependent glutamate synthase	WP_011863621.:	1435535..1440106	Nitrogen Metabolism	COG0069
WP_011863621.1_1589	CUST_8395_PI442190907	MIT9301	HLII	WP_011863621.1_1589	TACAGATAGAAGTGTTTGTGCGAAAATATCAGGCGAAATCGCAGAACTTCATGGCAACAC	glsF	Ferredoxin-dependent glutamate synthase	WP_011863621.:	1435535..1440106	Nitrogen Metabolism	COG0069
WP_011863621.1_1589	CUST_8396_PI442190907	MIT9301	HLII	WP_011863621.1_1589	GAAATCTCCTCCGAACAAGTCATCCTAGGAAATACTTGTCTTTATGGAGCAACAGGTGGG	glsF	Ferredoxin-dependent glutamate synthase	WP_011863621.:	1435535..1440106	Nitrogen Metabolism	COG0069
WP_011863621.1_1589	CUST_8397_PI442190907	MIT9301	HLII	WP_011863621.1_1589	TCTCATTTTAAGAGCACTTTCAAATTGATTGTTCCCCAAGCGAAGAAGAGATGCTTGGT	glsF	Ferredoxin-dependent glutamate synthase	WP_011863621.:	1435535..1440106	Nitrogen Metabolism	COG0069
WP_011819135.1_1600	CUST_8448_PI442190907	MIT9301	HLII	WP_011819135.1_1600	CGTACTCCAATTGCTAACCTTGTTGGCTGGAGAGATAAACCTGTTGCACTTTCAATTGTT	psaB	Photosystem I P700 chlorophyll A apoprotein A2 PsaB	WP_011819135.:	1446493..1448721	Photosystem I	COG2885
WP_011819135.1_1600	CUST_8449_PI442190907	MIT9301	HLII	WP_011819135.1_1600	AATAGACAAGATGCATTAAGTGCTTTCCTACCCATCGGACCTGCAGATTTCCTAGTTCAC	psaB	Photosystem I P700 chlorophyll A apoprotein A2 PsaB	WP_011819135.:	1446493..1448721	Photosystem I	COG2885
WP_011819135.1_1600	CUST_8450_PI442190907	MIT9301	HLII	WP_011819135.1_1600	TATCTCCTTGGGCTTGGATGTTCTTATTTGGTCACTTAGTTGGGCTACTGGTTTCATGT	psaB	Photosystem I P700 chlorophyll A apoprotein A2 PsaB	WP_011819135.:	1446493..1448721	Photosystem I	COG2885
WP_011819135.1_1600	CUST_8451_PI442190907	MIT9301	HLII	WP_011819135.1_1600	AAATTAATTCCTGATAAGAAAGATTTAGGTTATGCATTCCCTTGCGATGGACCTGGGCGC	psaB	Photosystem I P700 chlorophyll A apoprotein A2 PsaB	WP_011819135.:	1446493..1448721	Photosystem I	COG2885
WP_011819135.1_1600	CUST_8452_PI442190907	MIT9301	HLII	WP_011819135.1_1600	AAATTTACTAGCATGGGTAACTTTCTACTGGCATTGGAAACACCTAGCAATTTGGCAGGG	psaB	Photosystem I P700 chlorophyll A apoprotein A2 PsaB	WP_011819135.:	1446493..1448721	Photosystem I	COG2885
WP_011819135.1_1600	CUST_8453_PI442190907	MIT9301	HLII	WP_011819135.1_1600	GTTGGACTAGCACATTTCACGATTGGAAACATTCTTACATTCGGTGCATTTGTTATCGCA	psaB	Photosystem I P700 chlorophyll A apoprotein A2 PsaB	WP_011819135.:	1446493..1448721	Photosystem I	COG2885
WP_011863632.1_1601	CUST_8454_PI442190907	MIT9301	HLII	WP_011863632.1_1601	CCTTTGGGCGCAAGCTTCTCAAGTATTAACAAGTTATGGTCAATCCATAAGCATGTACGG	psaA	Photosystem I P700 chlorophyll A apoprotein A1 PsaA	WP_011863632.:	1448749..1451052	Photosystem I	COG2885
WP_011863632.1_1601	CUST_8455_PI442190907	MIT9301	HLII	WP_011863632.1_1601	GCTTTATCTATCACTCAGGGTAGAGCAGTAGGTGTAACACACTTCCTTGTCGGTGGTATT	psaA	Photosystem I P700 chlorophyll A apoprotein A1 PsaA	WP_011863632.:	1448749..1451052	Photosystem I	COG2885
WP_011863632.1_1601	CUST_8456_PI442190907	MIT9301	HLII	WP_011863632.1_1601	ATGTTCCTCTTTAGTGGACGCGGATATTGGCAAGAATTATTCGAATCAATTGTTTGGGCA	psaA	Photosystem I P700 chlorophyll A apoprotein A1 PsaA	WP_011863632.:	1448749..1451052	Photosystem I	COG2885
WP_011863632.1_1601	CUST_8457_PI442190907	MIT9301	HLII	WP_011863632.1_1601	TAGCAGGTACTGCAGAAGCTCTACCTCACAAAGCTTTGAGTGAAGTTTTTAACGGAAGTT	psaA	Photosystem I P700 chlorophyll A apoprotein A1 PsaA	WP_011863632.:	1448749..1451052	Photosystem I	COG2885
WP_011863632.1_1601	CUST_8458_PI442190907	MIT9301	HLII	WP_011863632.1_1601	GTAGAAGTGGGTGGAAAGGTTGCTATAGCTCCGATTCCATTAGGGACTGCTGATTTAATG	psaA	Photosystem I P700 chlorophyll A apoprotein A1 PsaA	WP_011863632.:	1448749..1451052	Photosystem I	COG2885
WP_011863632.1_1601	CUST_8459_PI442190907	MIT9301	HLII	WP_011863632.1_1601	GATTTAGATTCCCTTGTGATGGACCTGGTAGAGGTGGTACATGTCAAGTTTCTTCATGGG	psaA	Photosystem I P700 chlorophyll A apoprotein A1 PsaA	WP_011863632.:	1448749..1451052	Photosystem I	COG2885
WP_011863677.1_1652	CUST_8731_PI442190907	MIT9301	HLII	WP_011863677.1_1652	TTCCATGGGTATTTGGCTGGACACAAAGTAGATTTCTTTTGCCAAGTTGGTTTGGAGTAG	ppc	Phosphoenolpyruvate carboxylase	WP_011863677.:	1487262..1490231	Krebs cycle and related genes	COG2352
WP_011863677.1_1652	CUST_8732_PI442190907	MIT9301	HLII	WP_011863677.1_1652	ACAGCAGAAGTGAACTATTAAGGGGTGCACTTTTAACTATTAATGGGATAGCAGCTGGCA	ppc	Phosphoenolpyruvate carboxylase	WP_011863677.:	1487262..1490231	Krebs cycle and related genes	COG2352
WP_011863677.1_1652	CUST_8734_PI442190907	MIT9301	HLII	WP_011863677.1_1652	AGGGTTTTTGCAGGTTTCACTTCTAAGAAGATTAAGAGACCAGACAAGACAACCTCCAAT	ppc	Phosphoenolpyruvate carboxylase	WP_011863677.:	1487262..1490231	Krebs cycle and related genes	COG2352
WP_011863677.1_1652	CUST_8735_PI442190907	MIT9301	HLII	WP_011863677.1_1652	TGACATTATCGAAGGTGGATCTGGAAGTTGCTAGATATTATGTTGATACTCTTGGCAGCA	ppc	Phosphoenolpyruvate carboxylase	WP_011863677.:	1487262..1490231	Krebs cycle and related genes	COG2352
WP_011863709.1_1683	CUST_8908_PI442190907	MIT9301	HLII	WP_011863709.1_1683	TACAATTATTGAGTTACCATATTGCAGCTCACAGGGGACTTGACGTGGATCAACCAAGAA	glmS	Glutamine--fructose-6-phosphatetransaminase(isomerizing)	WP_011863709.:	1520256..1522151	Amino Acid metabolism	COG0449
WP_011863709.1_1683	CUST_8909_PI442190907	MIT9301	HLII	WP_011863709.1_1683	ATTCATGCAGCTGGATATCCTGCTGGGGAAATGAAACATGGTCCAATAGCTTTATTAGAT	glmS	Glutamine--fructose-6-phosphatetransaminase(isomerizing)	WP_011863709.:	1520256..1522151	Amino Acid metabolism	COG0449
WP_011863709.1_1683	CUST_8910_PI442190907	MIT9301	HLII	WP_011863709.1_1683	TCCGCCATTACTGGAAGATCTCTTACACAAACATAATAAATCTGCTCAGAAAAGCTAGCGCA	glmS	Glutamine--fructose-6-phosphatetransaminase(isomerizing)	WP_011863709.:	1520256..1522151	Amino Acid metabolism	COG0449
WP_011863709.1_1683	CUST_8911_PI442190907	MIT9301	HLII	WP_011863709.1_1683	GTGCAGGAATAGAAGTTGGAGTTGCAGCAACAAAAACTTTTTTTGCTCAGTTACTTTCGT	glmS	Glutamine--fructose-6-phosphatetransaminase(isomerizing)	WP_011863709.:	1520256..1522151	Amino Acid metabolism	COG0449
WP_011863709.1_1683	CUST_8912_PI442190907	MIT9301	HLII	WP_011863709.1_1683	TCAAACCCAATCTTATTGCAATAACAAATAGGAAAGAGAGTTCCATCGGAAGGCAGGTTT	glmS	Glutamine--fructose-6-phosphatetransaminase(isomerizing)	WP_011863709.:	1520256..1522151	Amino Acid metabolism	COG0449
WP_011863709.1_1683	CUST_8913_PI442190907	MIT9301	HLII	WP_011863709.1_1683	TGCTCAAGAAGCAAAAGCTAGAGATCATATTGATTGGGATTGCTCCTGAATGTAATGG	glmS	Glutamine--fructose-6-phosphatetransaminase(isomerizing)	WP_011863709.:	1520256..1522151	Amino Acid metabolism	COG0449
3.1_WP_036900507.1_	CUST_441_PI442182253	MIT0601	LLII/III	3.1_WP_036900507.1_	GGGTGGTTTTGCAGAAGTGGAAGCTGATGAAGTAACTGTTTTAGTTAATGCTGCAGAACT	atpC	ATP synthase epsilon subunit	WP_036900507.:	11903..12310	Energy Metabolism	COG0355
3.1_WP_036900507.1_	CUST_442_PI442182253	MIT0601	LLII/III	3.1_WP_036900507.1_	TCAACTGAAAAAGTAAAGGCCAAGGAAAGTCTAAATCGAGCTAGAGCAAGAGTTCAGGCA	atpC	ATP synthase epsilon subunit	WP_036900507.:	11903..12310	Energy Metabolism	COG0355
3.1_WP_036900507.1_	CUST_443_PI442182253	MIT0601	LLII/III	3.1_WP_036900507.1_	CTTTAAATGTTCGAGTGCTTGCCCCAGATAAAAGCGTCTTTGACGATACTGCAGAAGAAG	atpC	ATP synthase epsilon subunit	WP_036900507.:	11903..12310	Energy Metabolism	COG0355
3.1_WP_036900507.1_	CUST_444_PI442182253	MIT0601	LLII/III	3.1_WP_036900507.1_	TCCTAGCACTACAGGGTTATTAGGCATATTGCCTGGTCACATATCTATGGTTACAGCCAT	atpC	ATP synthase epsilon subunit	WP_036900507.:	11903..12310	Energy Metabolism	COG0355
3.1_WP_036900507.1_	CUST_445_PI442182253	MIT0601	LLII/III	3.1_WP_036900507.1_	TGGAGATGAAATCAATTCCAGCATCGCTGAAAAAGAATTTGAACAAGCTCAAAGCAAAGT	atpC	ATP synthase epsilon subunit	WP_036900507.:	11903..12310	Energy Metabolism	COG0355
3.1_WP_036900507.1_	CUST_446_PI442182253	MIT0601	LLII/III	3.1_WP_036900507.1_	CATAGATATTGGAGTGCTGAGAGTTCGAAAAGATGGTGGTTGGGACTCTATTGCTCT	atpC	ATP synthase epsilon subunit	WP_036900507.:	11903..12310	Energy Metabolism	COG0355
3.1_WP_036900526.1_	CUST_508_PI442182253	MIT0601	LLII/III	3.1_WP_036900526.1_	CTATGACGACTTAACTAAACAGGCACAAGCTTATCGTCAAATGTCCTTGCTTTTGCGTCG	atpA	ATP synthase alpha subunit	WP_036900526.:	22409..23926	Energy Metabolism	COG0056
3.1_WP_036900526.1_	CUST_509_PI442182253	MIT0601	LLII/III	3.1_WP_036900526.1_	GGTAAGCGTTTAAGAGAGCTTTTGAAACAAGCTCAGTTTGCACCTCTAACCTTGCAGAA	atpA	ATP synthase alpha subunit	WP_036900526.:	22409..23926	Energy Metabolism	COG0056
3.1_WP_036900526.1_	CUST_510_PI442182253	MIT0601	LLII/III	3.1_WP_036900526.1_	AAGCAGGGGACGTTTCGGCTTATATTCCAACCAATGTGATATCAATTACTGATGGTCAGA	atpA	ATP synthase alpha subunit	WP_036900526.:	22409..23926	Energy Metabolism	COG0056
3.1_WP_036900526.1_	CUST_511_PI442182253	MIT0601	LLII/III	3.1_WP_036900526.1_	TACTCTGAAGCTTGAGTTGGCTCAATTTGATGAATTGGCAGCTTTCTCTCAGTTTGCTTC	atpA	ATP synthase alpha subunit	WP_036900526.:	22409..23926	Energy Metabolism	COG0056
3.1_WP_036900526.1_	CUST_512_PI442182253	MIT0601	LLII/III	3.1_WP_036900526.1_	GTCGTGAGGCATATCCAGGAGATGTTTTCTATTGTCATAGTCGTTTGCTTGAGAGAGCTG	atpA	ATP synthase alpha subunit	WP_036900526.:	22409..23926	Energy Metabolism	COG0056
3.1_WP_036900526.1_	CUST_513_PI442182253	MIT0601	LLII/III	3.1_WP_036900526.1_	GCAGTTGTATATGCAGGTGTTAAAGGCTTGATTGATGAAGTGCCTGTTGAACAGGTAACA	atpA	ATP synthase alpha subunit	WP_036900526.:	22409..23926	Energy Metabolism	COG0056
3.1_WP_036900534.1_	CUST_537_PI442182253	MIT0601	LLII/III	3.1_WP_036900534.1_	CCTGATCTTCGCAACTCTTGCTGCTTACTACATTGGTGAAGCAGTAGAAGAACACCATTA	atpI	ATP synthase subunit A	WP_036900534.:	26019..26744	Energy Metabolism	COG0356
3.1_WP_036900534.1_	CUST_538_PI442182253	MIT0601	LLII/III	3.1_WP_036900534.1_	AATGCTGCCATTTAAGATTGTGGAAGACTTTACTAAGCCCCTTTCGCTTTCTTTCCGTTT	atpI	ATP synthase subunit A	WP_036900534.:	26019..26744	Energy Metabolism	COG0356
3.1_WP_036900534.1_	CUST_539_PI442182253	MIT0601	LLII/III	3.1_WP_036900534.1_	GATGCCTTTTATTGGCACCCTATTTTTGTTCATTTTTGTGAGCAATTGGGGAGGAGCTTT	atpI	ATP synthase subunit A	WP_036900534.:	26019..26744	Energy Metabolism	COG0356
3.1_WP_036900534.1_	CUST_540_PI442182253	MIT0601	LLII/III	3.1_WP_036900534.1_	ACCACTGTTGCATTGGCTCTTTTAGTTTCTCTTTCCTATTTCTATGCGGGATTGAGCAAC	atpI	ATP synthase subunit A	WP_036900534.:	26019..26744	Energy Metabolism	COG0356
3.1_WP_036900534.1_	CUST_541_PI442182253	MIT0601	LLII/III	3.1_WP_036900534.1_	TTTCTAGTACCACTTGTTCTACCAGTTCCTGTGATGTTCCTTGGCTTGTTTACAAGTGCT	atpI	ATP synthase subunit A	WP_036900534.:	26019..26744	Energy Metabolism	COG0356
3.1_WP_036900548.1_	CUST_597_PI442182253	MIT0601	LLII/III	3.1_WP_036900548.1_	CCCTTCCATTGGGTGGTACGGCAATAGGGACTGGATTAAATGCTCCTAAGTATTTTGATA	fumC	class II fumarate hydratase	WP_036900548.:	34695..36095	Krebs cycle and related genes	COG0114
3.1_WP_036900548.1_	CUST_598_PI442182253	MIT0601	LLII/III	3.1_WP_036900548.1_	AAGATTGCACAATTCGCACACAAGGAAAACGTAAGTTTAAGGGAAGCAGCTATCGAACTT	fumC	class II fumarate hydratase	WP_036900548.:	34695..36095	Krebs cycle and related genes	COG0114
3.1_WP_036900548.1_	CUST_599_PI442182253	MIT0601	LLII/III	3.1_WP_036900548.1_	GTAGTGGTGGTCAGCTACAGATGAATGTCTACAAACCATTGCTGGGCTTCAATCTTCTAA	fumC	class II fumarate hydratase	WP_036900548.:	34695..36095	Krebs cycle and related genes	COG0114
3.1_WP_036900548.1_	CUST_600_PI442182253	MIT0601	LLII/III	3.1_WP_036900548.1_	CCCTTCAAGCCCGCAAAAAATAAGTTCTCAATTATGGCTAGTCACGATGGCTTAGTCAAT	fumC	class II fumarate hydratase	WP_036900548.:	34695..36095	Krebs cycle and related genes	COG0114
3.1_WP_036900548.1_	CUST_601_PI442182253	MIT0601	LLII/III	3.1_WP_036900548.1_	GTATCACTCTTAAAGATTGTTAATGACATTCGTTTGCTTTCTTGTGGTCCTCGAGCGGGC	fumC	class II fumarate hydratase	WP_036900548.:	34695..36095	Krebs cycle and related genes	COG0114
3.1_WP_036900548.1_	CUST_602_PI442182253	MIT0601	LLII/III	3.1_WP_036900548.1_	CTTGCCAAAGTTGCAGGAAATTCATGCTTGAAGGCCTTCAAGCAAACGAATCAAGAATAG	fumC	class II fumarate hydratase	WP_036900548.:	34695..36095	Krebs cycle and related genes	COG0114
3.1_WP_036900598.1_	CUST_856_PI442182253	MIT0601	LLII/III	3.1_WP_036900598.1_	ATGTCCTGCTAATAGGTGAAGCGAATGATTATGTAGGCAAAGGTATCAATGGAGGCGTAC	glsF	Ferredoxin-dependent glutamate synthase	WP_036900598.:	90008..94594	Nitrogen Metabolism	COG0069
3.1_WP_036900598.1_	CUST_860_PI442182253	MIT0601	LLII/III	3.1_WP_036900598.1_	AGGCAAGCTTGAACTAATCTTTAAAGGTTCTGCCGGACAAAGCTTTGGAGCATTCTTATT	glsF	Ferredoxin-dependent glutamate synthase	WP_036900598.:	90008..94594	Nitrogen Metabolism	COG0069
3.1_WP_036900598.1_	CUST_857_PI442182253	MIT0601	LLII/III	3.1_WP_036900598.1_	AGGAATGTAGGAGCAGGAATGACTGGAGGAGTAGCCTTTCTACTCGATGAAGATCAAAAA	glsF	Ferredoxin-dependent glutamate synthase	WP_036900598.:	90008..94594	Nitrogen Metabolism	COG0069
3.1_WP_036900598.1_	CUST_858_PI442182253	MIT0601	LLII/III	3.1_WP_036900598.1_	ACGTTTTGGAGTGAGAAATAGTGGAGTAAAAGCAGTGATAGAAGGAGCAGGTGACCATTG	glsF	Ferredoxin-dependent glutamate synthase	WP_036900598.:	90008..94594	Nitrogen Metabolism	COG0069
3.1_WP_036900598.1_	CUST_859_PI442182253	MIT0601	LLII/III	3.1_WP_036900598.1_	TTCTTAATACTGATCGAAGTGTATGCGCTCGAGTATCTGGGGAGATTGCTGAATTGCATG	glsF	Ferredoxin-dependent glutamate synthase	WP_036900598.:	90008..94594	Nitrogen Metabolism	COG0069
3.1_WP_036900598.1_	CUST_861_PI442182253	MIT0601	LLII/III	3.1_WP_036900598.1_	TAGTCGAAATTGCTCAACCCAAGTTATTCTTGGGAATACATGCCTCTATGGAGGAACTGG	glsF	Ferredoxin-dependent glutamate synthase	WP_036900598.:	90008..94594	Nitrogen Metabolism	COG0069
3.1_WP_036900601.1_	CUST_878_PI442182253	MIT0601	LLII/III	3.1_WP_036900601.1_	CTAATGTTCCTTGGTGCTCACTTTGTATGGGCATTCAGTCTCATGTTCCTCTTTAGTGGA	psaA	Photosystem I P700 chlorophyll A apoprotein A1 PsaA	WP_036900601.:	98133..100454	Photosystem I	COG2885
3.1_WP_036900601.1_	CUST_879_PI442182253	MIT0601	LLII/III	3.1_WP_036900601.1_	ATCTTCCATTTCTCATGGAAAATGCAAAGTGATGTATGGGGTTTGACAGGTGGGAACTTT	psaA	Photosystem I P700 chlorophyll A apoprotein A1 PsaA	WP_036900601.:	98133..100454	Photosystem I	COG2885
3.1_WP_036900601.1_	CUST_880_PI442182253	MIT0601	LLII/III	3.1_WP_036900601.1_	TCTTCCTGGGACCACGTTTTCCTTGGACTATTTTGGATGTATAACAGCCTTTCAGTTGTT	psaA	Photosystem I P700 chlorophyll A apoprotein A1 PsaA	WP_036900601.:	98133..100454	Photosystem I	COG2885
3.1_WP_036900601.1_	CUST_881_PI442182253	MIT0601	LLII/III	3.1_WP_036900601.1_	TAGGCACAGCTGACTTGATGATCCACGTTCATGCCTTTACTATTCATGTAACCCTTT	psaA	Photosystem I P700 chlorophyll A apoprotein A1 PsaA	WP_036900601.:	98133..100454	Photosystem I	COG2885
3.1_WP_036900601.1_	CUST_882_PI442182253	MIT0601	LLII/III	3.1_WP_036900601.1_	GTACTGGAGAACTTGTCGGAGCTGGAAATTTACCTGGGAATGTTCTCAGTGAAGTTTTCA	psaA	Photosystem I P700 chlorophyll A apoprotein A1 PsaA	WP_036900601.:	98133..100454	Photosystem I	COG2885
3.1_WP_036900601.1_	CUST_883_PI442182253	MIT0601	LLII/III	3.1_WP_036900601.1_	GTCATGAGGGCTTTGGGACGTCCTCAAGATATGTTTAGTGATACAGGCATTCAGTTACAG	psaA	Photosystem I P700 chlorophyll A apoprotein A1 PsaA	WP_036900601.:	98133..100454	Photosystem I	COG2885
3.1_WP_036900602.1_	CUST_884_PI442182253	MIT0601	LLII/III	3.1_WP_036900602.1_	AGTTGGACTTACGCACTTTACTGTGGGTAATTTCGTAACCTTTGGTGCATTCGTAATTGC	psaB	Photosystem I P700 chlorophyll A apoprotein A2 PsaB	WP_036900602.:	100477..102705	Photosystem I	COG2885
3.1_WP_036900602.1_	CUST_885_PI442182253	MIT0601	LLII/III	3.1_WP_036900602.1_	ATTTAATTTGGGCAACAGGGTTTATGTTCCTGATTTCCTGGAGAGGATATTGGCAAGAGC	psaB	Photosystem I P700 chlorophyll A apoprotein A2 PsaB	WP_036900602.:	100477..102705	Photosystem I	COG2885
3.1_WP_036900602.1_	CUST_887_PI442182253	MIT0601	LLII/III	3.1_WP_036900602.1_	GATAAAAAAGACTTTGGTTACGCTTTCCCATGTGATGGTCCTGGTAGAGGAGGTACATGT	psaB	Photosystem I P700 chlorophyll A apoprotein A2 PsaB	WP_036900602.:	100477..102705	Photosystem I	COG2885
3.1_WP_036900602.1_	CUST_888_PI442182253	MIT0601	LLII/III	3.1_WP_036900602.1_	TTTAACTAATTTCCTTCCCATAGGTCCAGCTGATTTCCTAGTACACCACGCAATCGCTTT	psaB	Photosystem I P700 chlorophyll A apoprotein A2 PsaB	WP_036900602.:	100477..102705	Photosystem I	COG2885
3.1_WP_036900602.1_	CUST_889_PI442182253	MIT0601	LLII/III	3.1_WP_036900602.1_	ACCTGTTTTTGCTCAAGCAATTCAGGCATTTAGTGGAAAAGTGATGTATGGCATTGATGC	psaB	Photosystem I P700 chlorophyll A apoprotein A2 PsaB	WP_036900602.:	100477..102705	Photosystem I	COG2885
3.1_WP_036900699.1_	CUST_1183_PI442182253	MIT0601	LLII/III	3.1_WP_036900699.1_	TCGGATGTTGGTTTCTAAGGTAGAGATGACCTTGTCGAAAGTTGACCTGGAAGTAGCTAA	ppc	Phosphoenolpyruvate carboxylase	WP_036900699.:	140299..143319	Krebs cycle and related genes	COG2352
3.1_WP_036900699.1_	CUST_1181_PI442182253	MIT0601	LLII/III	3.1_WP_036900699.1_	TGGTTACTAATCAACTGGATGCTACCCTTCCTGGAATGAACTAATGACAAGATTGGCAG	ppc	Phosphoenolpyruvate carboxylase	WP_036900699.:	140299..143319	Krebs cycle and related genes	COG2352
3.1_WP_036900699.1_	CUST_1182_PI442182253	MIT0601	LLII/III	3.1_WP_036900699.1_	GCATCCGTAGAACTTAGGAATCGTACTATTGTACCTCTGGGCTTTCTTCAGGTTGCTTTG	ppc	Phosphoenolpyruvate carboxylase	WP_036900699.:	140299..143319	Krebs cycle and related genes	COG2352
3.1_WP_036900699.1_	CUST_1184_PI442182253	MIT0601	LLII/III	3.1_WP_036900699.1_	GGTGTAGGGACAGCCTTGGCAGCTGAATTAGAAGCTGATCAAGACCAGATTGAATTATTA	ppc	Phosphoenolpyruvate carboxylase	WP_036900699.:	140299..143319	Krebs cycle and related genes	COG2352
3.1_WP_036900699.1_	CUST_1185_PI442182253	MIT0601	LLII/III	3.1_WP_036900699.1_	GTGGAGATATAGCACGCACGTATAGTCGTAGTGAATTGTTAAGAGGTGCCCTGCTAACTA	ppc	Phosphoenolpyruvate carboxylase	WP_036900699.:	140299..143319	Krebs cycle and related genes	COG2352
3.1_WP_036900699.1_	CUST_1186_PI442182253	MIT0601	LLII/III	3.1_WP_036900699.1_	ATTACAAATTTCTAGCCGACCAGCCCGTAGGAAATCTGGAACGAAGCAATTATCAAGTCT	ppc	Phosphoenolpyruvate carboxylase	WP_036900699.:	140299..143319	Krebs cycle and related genes	COG2352
3.1_WP_036900776.1_	CUST_1389_PI442182253	MIT0601	LLII/III	3.1_WP_036900776.1_	TGAAATGAAGCATGGCCCTATTGCCTTGCTAGATCAAAGAGTGCCTGTTATTTCAATAGC	glmS	Glutamine--fructose-6-phosphatetransaminase(isomerizing)	WP_036900776.:	179174..181078	Amino Acid metabolism	COG0449
3.1_WP_036900776.1_	CUST_1390_PI442182253	MIT0601	LLII/III	3.1_WP_036900776.1_	CAGGGTGTGGTTTACGAAAAGGTTTTAAGTAATGCTCAAGAAGCTAAAGCAAGGGATGCA	glmS	Glutamine--fructose-6-phosphatetransaminase(isomerizing)	WP_036900776.:	179174..181078	Amino Acid metabolism	COG0449
3.1_WP_036900776.1_	CUST_1391_PI442182253	MIT0601	LLII/III	3.1_WP_036900776.1_	AGGAATTAGCTCATCGTTTTTCAGAGACTGAAGATGTGATCTTTTGGGGCGGGGTATTA	glmS	Glutamine--fructose-6-phosphatetransaminase(isomerizing)	WP_036900776.:	179174..181078	Amino Acid metabolism	COG0449
3.1_WP_036900776.1_	CUST_1392_PI442182253	MIT0601	LLII/III	3.1_WP_036900776.1_	CGGACAGCTTTGAATGATGAGGATTTTGCTTCTGTCCAGCTTGGGATTACTAATAGTCCT	glmS	Glutamine--fructose-6-phosphatetransaminase(isomerizing)	WP_036900776.:	179174..181078	Amino Acid metabolism	COG0449
3.1_WP_036900776.1_	CUST_1393_PI442182253	MIT0601	LLII/III	3.1_WP_036900776.1_	CGGCTCAGAGAGGTTTGGATGTTGATCAACCAAGAAATCTAGCCAAAAGTGTAACTGTTG	glmS	Glutamine--fructose-6-phosphatetransaminase(isomerizing)	WP_036900776.:	179174..181078	Amino Acid metabolism	COG0449
3.1_WP_036900776.1_	CUST_1394_PI442182253	MIT0601	LLII/III	3.1_WP_036900776.1_	TGAGGGAGCATTGAAACTTAAAGAGATTAGTTATATCCATGCTGAGGGGTATCCAGCTGG	glmS	Glutamine--fructose-6-phosphatetransaminase(isomerizing)	WP_036900776.:	179174..181078	Amino Acid metabolism	COG0449
3.1_WP_036901016.1_	CUST_2135_PI442182253	MIT0601	LLII/III	3.1_WP_036901016.1_	TGGGCTACTGAAGAATTTGGGTTTGTTCGTTTAACTGATCGATGTTCTACAGGCAGTAGT	argH	Argininosuccinate lyase	WP_036901016.:	325128..326516	Amino Acid metabolism	COG0165
3.1_WP_036901016.1_	CUST_2132_PI442182253	MIT0601	LLII/III	3.1_WP_036901016.1_	GGAGGCACTGGTTTTGATCAAATTCTGAACAGTTGCTTGCTTGGAGTAATAGGTTTTCT	argH	Argininosuccinate lyase	WP_036901016.:	325128..326516	Amino Acid metabolism	COG0165
3.1_WP_036901016.1_	CUST_2133_PI442182253	MIT0601	LLII/III	3.1_WP_036901016.1_	GATCGGGATATACATGAAAGGTTGATTCCGCAACAAGTCGTAGCTTCAAGAGTGAGTCAG	argH	Argininosuccinate lyase	WP_036901016.:	325128..326516	Amino Acid metabolism	COG0165
3.1_WP_036901016.1_	CUST_2134_PI442182253	MIT0601	LLII/III	3.1_WP_036901016.1_	GCAGGGTGTTTGGTCATTTACAAGCTTTATTAGCAATGATGAAAGGCTTACCTTTGGCCT	argH	Argininosuccinate lyase	WP_036901016.:	325128..326516	Amino Acid metabolism	COG0165
3.1_WP_036901016.1_	CUST_2136_PI442182253	MIT0601	LLII/III	3.1_WP_036901016.1_	TTGCCGTTGAATTTACGGCAGCAGCTTCACTGATAATGGTTCATTTGAGTAGATTGTCTG	argH	Argininosuccinate lyase	WP_036901016.:	325128..326516	Amino Acid metabolism	COG0165
3.1_WP_036901016.1_	CUST_2137_PI442182253	MIT0601	LLII/III	3.1_WP_036901016.1_	TAAGAATAAGAGTTAATACTTGCCCTTTAGGAGCAGCCGCTTTGGCTGGAACTTCTTTCC	argH	Argininosuccinate lyase	WP_036901016.:	325128..326516	Amino Acid metabolism	COG0165
4.1_WP_036901156.1_	CUST_2403_PI442182253	MIT0601	LLII/III	4.1_WP_036901156.1_	GTTATGACGCTTATACCCAAAGCCAGGGTACAGCTTTCGTGGTTTTCGAAGGAC	rbcS	Ribulose bisphosphate carboxylase small subunit	WP_036901156.:	44724..45065	Carbon fixation	COG4451
4.1_WP_036901156.1_	CUST_2404_PI442182253	MIT0601	LLII/III	4.1_WP_036901156.1_	TAATGAACTAGAGGCCTGCCATCGTGCATACCCTGATCACCATGTTCGGAT	rbcS	Ribulose bisphosphate carboxylase small subunit	WP_036901156.:	44724..45065	Carbon fixation	COG4451
4.1_WP_036901157.1_	CUST_2408_PI442182253	MIT0601	LLII/III	4.1_WP_036901157.1_	ATACATTGACAACTTACGTGAATCTTTCGTTCCAGAAGACCGTACACGCGGAAACTTCTT	rbcL	Ribulose 1,5-bisphosphate carboxylase large subunit	WP_036901157.:	45162..46574	Carbon fixation	COG1850
4.1_WP_036901156.1_	CUST_2405_PI442182253	MIT0601	LLII/III	4.1_WP_036901156.1_	GTCTTATTGGAAGCTACCTTTCTTCGGCGAGAAGGATCTGAACGTGGTGTTAATGAACT	rbcS	Ribulose bisphosphate carboxylase small subunit	WP_036901156.:	44724..45065	Carbon fixation	COG4451
4.1_WP_036901156.1_	CUST_2406_PI442182253	MIT0601	LLII/III	4.1_WP_036901156.1_	GAAATTTACGATCAAATTGCTTACATTATTGCTCAGGGTTGGAGCCCTGTCATCGAGCAT	rbcS	Ribulose bisphosphate carboxylase small subunit	WP_036901156.:	44724..45065	Carbon fixation	COG4451
4.1_WP_036901156.1_	CUST_2407_PI442182253	MIT0601	LLII/III	4.1_WP_036901156.1_	CTTTCCAGAGCACAGTTGGTGACTATCAAACAGTTGCCACCCTGGAGACTTTC	rbcS	Ribulose bisphosphate carboxylase small subunit	WP_036901156.:	44724..45065	Carbon fixation	COG4451
4.1_WP_036901157.1_	CUST_2409_PI442182253	MIT0601	LLII/III	4.1_WP_036901157.1_	GCTATGCATGCAGTAATTGACCGTCATCCAAAGCATGGTATTCATTTCCGTGTATTAGCA	rbcL	Ribulose 1,5-bisphosphate carboxylase large subunit	WP_036901157.:	45162..46574	Carbon fixation	COG1850
4.1_WP_036901157.1_	CUST_2410_PI442182253	MIT0601	LLII/III	4.1_WP_036901157.1_	CGTAATGCTGGAAGAGAAATTGAAAAAGAAAGTCGCGACATCCTTATGGAAGCGGCTAAG	rbcL	Ribulose 1,5-bisphosphate carboxylase large subunit	WP_036901157.:	45162..46574	Carbon fixation	COG1850
4.1_WP_036901157.1_	CUST_2411_PI442182253	MIT0601	LLII/III	4.1_WP_036901157.1_	ACTACATTACCGGTGGTTTTACTGCTAACACAGGATTAGCCAACTGGTGTCGTAAGAATG	rbcL	Ribulose 1,5-bisphosphate carboxylase large subunit	WP_036901157.:	45162..46574	Carbon fixation	COG1850
4.1_WP_036901157.1_	CUST_2412_PI442182253	MIT0601	LLII/III	4.1_WP_036901157.1_	GTAAAGCTTGCTCAACAAGAAACTGGTGAAGTCAAAGGGCATTACCTTAACTGTACTGCG	rbcL	Ribulose 1,5-bisphosphate carboxylase large subunit	WP_036901157.:	45162..46574	Carbon fixation	COG1850
4.1_WP_036901157.1_	CUST_2413_PI442182253	MIT0601	LLII/III	4.1_WP_036901157.1_	TTGGCTATTGCACTTGAGACATGGAAGGAGATCAAGTTTGAGTTCGATACAGTCGACAAG	rbcL	Ribulose 1,5-bisphosphate carboxylase large subunit	WP_036901157.:	45162..46574	Carbon fixation	COG1850
4.1_WP_036901183.1_	CUST_2578_PI442182253	MIT0601	LLII/III	4.1_WP_036901183.1_	TTCCTGGAAGAGTTTCGACGGAAGTAGATGCTCGTCTTAGTTATGATACAGAAGCGACAA	tal	Transaldolase	WP_036901183.:	73913..74920	Pentose Phosphate	COG0176
4.1_WP_036901183.1_	CUST_2576_PI442182253	MIT0601	LLII/III	4.1_WP_036901183.1_	TGTTAACAATCTCCCCGGCGTTGTTGGATCAATTAAGGAATACACATGCTCCTCTTCTTA	tal	Transaldolase	WP_036901183.:	73913..74920	Pentose Phosphate	COG0176
4.1_WP_036901183.1_	CUST_2577_PI442182253	MIT0601	LLII/III	4.1_WP_036901183.1_	TATTATTTAACTTTTGCCAGGCTGTTGCTTGTGCAGAGGCAGGTGTAACCCTTATTTCTC	tal	Transaldolase	WP_036901183.:	73913..74920	Pentose Phosphate	COG0176
4.1_WP_036901183.1_	CUST_2579_PI442182253	MIT0601	LLII/III	4.1_WP_036901183.1_	GGAGGCTGATTTGATGGCAGATGAGAAGTTAAAAGAGGGAATTGAAAAGTTTAGCAAGGC	tal	Transaldolase	WP_036901183.:	73913..74920	Pentose Phosphate	COG0176
4.1_WP_036901183.1_	CUST_2580_PI442182253	MIT0601	LLII/III	4.1_WP_036901183.1_	AGGCCGCATACTTGATTGGTATAAAGCAAAAACAGGCAAAGATTCATATTTAGGAGCTGA	tal	Transaldolase	WP_036901183.:	73913..74920	Pentose Phosphate	COG0176
4.1_WP_036901183.1_	CUST_2581_PI442182253	MIT0601	LLII/III	4.1_WP_036901183.1_	GCATCTACTTGGGAAGGAATTAAGGCTGCTGAAGTTTTAGAAGAAGAAGGCATTCATTGT	tal	Transaldolase	WP_036901183.:	73913..74920	Pentose Phosphate	COG0176
4.1_WP_036901232.1_	CUST_2955_PI442182253	MIT0601	LLII/III	4.1_WP_036901232.1_	ATCTGACTGGAGTATTTCTATGCTCAAGAGCTGTTGCAAGGCCTATGCTTAAACAAAGGA	gdh	3-oxoacyl-acyl-carrier-protein reductase	WP_036901232.:	136494..137246	Entner-Dudoroff	COG1028
4.1_WP_036901232.1_	CUST_2956_PI442182253	MIT0601	LLII/III	4.1_WP_036901232.1_	GCAGCAAAAGCAGGTGTTATTGGACTTACAAAGAGTTCTGCGAAAGAATTTGCGAGTAGA	gdh	3-oxoacyl-acyl-carrier-protein reductase	WP_036901232.:	136494..137246	Entner-Dudoroff	COG1028
4.1_WP_036901232.1_	CUST_2957_PI442182253	MIT0601	LLII/III	4.1_WP_036901232.1_	TATTCAAGCTCCCCAGAAAAAGCGGAAGAAGTGGTTTCTACAATTAAAGAATCGGGAGGA	gdh	3-oxoacyl-acyl-carrier-protein reductase	WP_036901232.:	136494..137246	Entner-Dudoroff	COG1028
4.1_WP_036901232.1_	CUST_2958_PI442182253	MIT0601	LLII/III	4.1_WP_036901232.1_	GCTGCGTCCTATATAACTGGTCAAGTTTTACAAGTTGATGGGGGGATGGTAATGAGCTAG	gdh	3-oxoacyl-acyl-carrier-protein reductase	WP_036901232.:	136494..137246	Entner-Dudoroff	COG1028
4.1_WP_036901232.1_	CUST_2959_PI442182253	MIT0601	LLII/III	4.1_WP_036901232.1_	TAATTCAGCTCTAATGGAAGGACAAACTGCCTTAGTAACAGGGGCTAGCAGAGGTATAGG	gdh	3-oxoacyl-acyl-carrier-protein reductase	WP_036901232.:	136494..137246	Entner-Dudoroff	COG1028
4.1_WP_036901232.1_	CUST_2960_PI442182253	MIT0601	LLII/III	4.1_WP_036901232.1_	GAAGGATTATAAATATCACCTCTGTAGTGGGACTTATGGCAATGCTGGGCAAGCAAATT	gdh	3-oxoacyl-acyl-carrier-protein reductase	WP_036901232.:	136494..137246	Entner-Dudoroff	COG1028
4.1_WP_036901240.1_	CUST_3003_PI442182253	MIT0601	LLII/III	4.1_WP_036901240.1_	TTGGCCTCTATAGCTATGACATATATGACCGAATGGGAGGAATGCAAACATTAACCCACG	ctaC/coxB	Cytochrome c oxidase subunit II	WP_036901240.:	144355..145179	Respiration	COG1622
4.1_WP_036901240.1_	CUST_3001_PI442182253	MIT0601	LLII/III	4.1_WP_036901240.1_	GGCCATACCATGGAGGTATGAGATCAACAGTTGTAGTGGAAGATCCTGAAGACTACAAGA	ctaC/coxB	Cytochrome c oxidase subunit II	WP_036901240.:	144355..145179	Respiration	COG1622
4.1_WP_036901240.1_	CUST_3002_PI442182253	MIT0601	LLII/III	4.1_WP_036901240.1_	ATGTACCTGTTGGTCAGCCAGTCAGTATGAAAATGGAGTCCAAAGATGTATCATACATGCTT	ctaC/coxB	Cytochrome c oxidase subunit II	WP_036901240.:	144355..145179	Respiration	COG1622
4.1_WP_036901240.1_	CUST_3004_PI442182253	MIT0601	LLII/III	4.1_WP_036901240.1_	ATTGGAGATGGGCTAGCTATTGAAGACAACCTTCCACTTGAAATTTTTTGGACAGCTGTG	ctaC/coxB	Cytochrome c oxidase subunit II	WP_036901240.:	144355..145179	Respiration	COG1622
4.1_WP_036901240.1_	CUST_3005_PI442182253	MIT0601	LLII/III	4.1_WP_036901240.1_	GAAGCTTGGGGACCATTCCTGTTGAAGTAACTGCAATGCAATTTGCTTTTCTTTTTCACT	ctaC/coxB	Cytochrome c oxidase subunit II	WP_036901240.:	144355..145179	Respiration	COG1622
4.1_WP_036901240.1_	CUST_3006_PI442182253	MIT0601	LLII/III	4.1_WP_036901240.1_	TGTCGTCGCAAGCTCCAACGCCCCTATTTATGACGAACTCTTTAAAGTTCTCTTCATTAT	ctaC/coxB	Cytochrome c oxidase subunit II	WP_036901240.:	144355..145179	Respiration	COG1622
4.1_WP_036901241.1_	CUST_3007_PI442182253	MIT0601	LLII/III	4.1_WP_036901241.1_	AGGAGAAGCACCTCTAGTTACCAAACCATATGGGTATGGCCAAGTTGAAATCAATGCTAA	cyoB/coxA	Cytochrome c oxidase subunit I	WP_036901241.:	145176..146837	Respiration	COG0843
4.1_WP_036901241.1_	CUST_3008_PI442182253	MIT0601	LLII/III	4.1_WP_036901241.1_	ATTCGCTTTTGTGAATCAACTCAGTAGTGCAGGTGCTCTATTGATGGCGATAAGTACACT	cyoB/coxA	Cytochrome c oxidase subunit I	WP_036901241.:	145176..146837	Respiration	COG0843
4.1_WP_036901241.1_	CUST_3009_PI442182253	MIT0601	LLII/III	4.1_WP_036901241.1_	GTTTCATTGTAAATTTTGTACTCGGTGGGATTACTGGAGTTGCATTGGCTCAGGTTCCTT	cyoB/coxA	Cytochrome c oxidase subunit I	WP_036901241.:	145176..146837	Respiration	COG0843
4.1_WP_036901241.1_	CUST_3010_PI442182253	MIT0601	LLII/III	4.1_WP_036901241.1_	GATAAAGTTTTTCAATTGGGTGGCAACTCTTGGGGTGGAGAATCTCTCTTAACACAGC	cyoB/coxA	Cytochrome c oxidase subunit I	WP_036901241.:	145176..146837	Respiration	COG0843
4.1_WP_036901241.1_	CUST_3011_PI442182253	MIT0601	LLII/III	4.1_WP_036901241.1_	TTGTTTTAGGATTAGTGGTATGGGCACATCATATGTTTACAAGTGGCACTCCTCCTTGGA	cyoB/coxA	Cytochrome c oxidase subunit I	WP_036901241.:	145176..146837	Respiration	COG0843
4.1_WP_036901241.1_	CUST_3012_PI442182253	MIT0601	LLII/III	4.1_WP_036901241.1_	GTGTACATAATGGTCCTACCAGCATTTGGATTGGTCAGTGAAATACTTCCAGTGCATTGC	cyoB/coxA	Cytochrome c oxidase subunit I	WP_036901241.:	145176..146837	Respiration	COG0843
4.1_WP_036901242.1_	CUST_3013_PI442182253	MIT0601	LLII/III	4.1_WP_036901242.1_	AGTACCAATAAATTCCCACTTGAAGCAGCTGAACTCTATTGGCACTTCGTAGATGGATC	cyoC	heme-copper oxidase subunit III	WP_036901242.:	146834..147448	Respiration	COG1845
4.1_WP_036901242.1_	CUST_3014_PI442182253	MIT0601	LLII/III	4.1_WP_036901242.1_	TGCAGGTTTTTTTGCTGCGTATCTTACTTTTAAAGCAGTCAACCCATTACTACCTGGAGC	cyoC	heme-copper oxidase subunit III	WP_036901242.:	146834..147448	Respiration	COG1845
4.1_WP_036901242.1_	CUST_3015_PI442182253	MIT0601	LLII/III	4.1_WP_036901242.1_	ACAGGATTTCATGGGCTGCATGTCACTTTAGGCACCTTGATGATTTTATAATCTGGTGG	cyoC	heme-copper oxidase subunit III	WP_036901242.:	146834..147448	Respiration	COG1845
4.1_WP_036901242.1_	CUST_3016_PI442182253	MIT0601	LLII/III	4.1_WP_036901242.1_	TTCCTTGTGAGTCAAATGATTGAATATTTCAGCCTTCCCTTCGGCCTCACTGACAATTTG	cyoC	heme-copper oxidase subunit III	WP_036901242.:	146834..147448	Respiration	COG1845
4.1_WP_036901242.1_	CUST_3017_PI442182253	MIT0601	LLII/III	4.1_WP_036901242.1_	CTAGTAAAGCATTGCAGAAAAAGATGCGAAAGTCTGCCAACAATGGTTGCTGGTTACTG	cyoC	heme-copper oxidase subunit III	WP_036901242.:	146834..147448	Respiration	COG1845
4.1_WP_036901242.1_	CUST_3018_PI442182253	MIT0601	LLII/III	4.1_WP_036901242.1_	GATAACTCTCCTGGTTCAACTACCGATCCAAAAACTTAGAAGGTGAACATTCCGACCAT	cyoC	heme-copper oxidase subunit III	WP_036901242.:	146834..147448	Respiration	COG1845
4.1_WP_036901304.1_	CUST_3450_PI442182253	MIT0601	LLII/III	4.1_WP_036901304.1_	GGGAGATTACTTGGCATAGCTCACGCAGGTAAATATGGAAATGATCGCGCAAAAATAACA	minC	septum site-determining protein MinC	WP_036901304.:	229601..230239	Cell Division	COG0850
4.1_WP_036901304.1_	CUST_3451_PI442182253	MIT0601	LLII/III	4.1_WP_036901304.1_	CTTGTTAATACTGGGTGATATCAACCCTGGAGCAGTTATTTCTGCAGGGGGTGATGTAAT	minC	septum site-determining protein MinC	WP_036901304.:	229601..230239	Cell Division	COG0850
4.1_WP_036901304.1_	CUST_3452_PI442182253	MIT0601	LLII/III	4.1_WP_036901304.1_	TCCTTCCCAGAGACTGTTGTAAGCGCAAAATCTCTAGGATTTAAAAGCTCACTCATGCTC	minC	septum site-determining protein MinC	WP_036901304.:	229601..230239	Cell Division	COG0850
4.1_WP_036901304.1_	CUST_3455_PI442182253	MIT0601	LLII/III	4.1_WP_036901304.1_	GGGCTTGCTGAAGAAGCCATTATCGAAGACGGAGTAATTGTTATTAAACCATCAAGA	minC	septum site-determining protein MinC	WP_036901304.:	229601..230239	Cell Division	COG0850
4.1_WP_036901304.1_	CUST_3453_PI442182253	MIT0601	LLII/III	4.1_WP_036901304.1_	GCTTTCATTGCTGATGAAAAAGAACCAAATACAGTTTTCCATGAAGGGACTTTGCGCTCG	minC	septum site-determining protein MinC	WP_036901304.:	229601..230239	Cell Division	COG0850
4.1_WP_036901304.1_	CUST_3454_PI442182253	MIT0601	LLII/III	4.1_WP_036901304.1_	GTTCAACTGAGAATATCAAATAAAATTGCAAGAGGGCCTAAGGAGAAGCCTGAGCAAGGG	minC	septum site-determining protein MinC	WP_036901304.:	229601..230239	Cell Division	COG0850
4.1_WP_036901305.1_	CUST_3456_PI442182253	MIT0601	LLII/III	4.1_WP_036901305.1_	TCGTATATACCGCCCAAGAAGTACTAGAAGAAAGCTGCCGACTAGATCAAGCATTGGTTA	minD	septum site-determining protein MinD	WP_036901305.:	230379..231194	Cell Division	COG2894
4.1_WP_036901305.1_	CUST_3457_PI442182253	MIT0601	LLII/III	4.1_WP_036901305.1_	TCAATATATTCTTATTGACTGTCCGGCCGGGGTCGAAGATGGTTTTAAAAATGCTGTTGC	minD	septum site-determining protein MinD	WP_036901305.:	230379..231194	Cell Division	COG2894
4.1_WP_036901305.1_	CUST_3458_PI442182253	MIT0601	LLII/III	4.1_WP_036901305.1_	TGTTCAGTTGGTGCTGAATAGAGTTCGTCCAAAAATGATGGAAAGCCAAGAGATGCTTTC	minD	septum site-determining protein MinD	WP_036901305.:	230379..231194	Cell Division	COG2894
4.1_WP_036901306.1_	CUST_3462_PI442182253	MIT0601	LLII/III	4.1_WP_036901306.1_	GGCCAAATTAAATTAAAAGAGAAATCATCTGAGAGCTCATCCGAAAACAAATCCGACTA	minE	septum site-determining protein MinE	WP_036901306.:	231199..231519	Cell Division	COG0851
4.1_WP_036901305.1_	CUST_3459_PI442182253	MIT0601	LLII/III	4.1_WP_036901305.1_	CTTTAATAGGCAGCAATTCTCCCGCTGCCAGATGTTATTCCAATATTGCAGGAAGACTTC	minD	septum site-determining protein MinD	WP_036901305.:	230379..231194	Cell Division	COG2894
4.1_WP_036901305.1_	CUST_3460_PI442182253	MIT0601	LLII/III	4.1_WP_036901305.1_	AGGCAATTATCGTAACGAATCCAGAAGTTTCAGCAGTCAGAGATGCCGACAGAGTAATTG	minD	septum site-determining protein MinD	WP_036901305.:	230379..231194	Cell Division	COG2894
4.1_WP_036901305.1_	CUST_3461_PI442182253	MIT0601	LLII/III	4.1_WP_036901305.1_	ACGAATGCTCGATTGGCTAAAGCCAGAGGACATGCAAAAAATAGTTGGAATGCTTAAAGA	minD	septum site-determining protein MinD	WP_036901305.:	230379..231194	Cell Division	COG2894
4.1_WP_036901306.1_	CUST_3463_PI442182253	MIT0601	LLII/III	4.1_WP_036901306.1_	AAACAGAAGATAGGATGACCGCACTAGTAGCAAACCTGCCTATCAAAAGAACCCTTTCAG	minE	septum site-determining protein MinE	WP_036901306.:	231199..231519	Cell Division	COG0851
4.1_WP_036901306.1_	CUST_3464_PI442182253	MIT0601	LLII/III	4.1_WP_036901306.1_	AGATCTTAAGTGTTGTAGCTAAGTATGTGGAAATTGATGTTGAGGAAGGAGCAGTTAGCT	minE	septum site-determining protein MinE	WP_036901306.:	231199..231519	Cell Division	COG0851
4.1_WP_036901306.1_	CUST_3465_PI442182253	MIT0601	LLII/III	4.1_WP_036901306.1_	AGTGCTGCCAAAGCAAGAGAAAGACTTCAATTAGTACTCGCTCATGATCGAACGGATTTA	minE	septum site-determining protein MinE	WP_036901306.:	231199..231519	Cell Division	COG0851
4.1_WP_036901315.1_	CUST_3530_PI442182253	MIT0601	LLII/III	4.1_WP_036901315.1_	TCTATTAATTCTATAGGGGCTTTATGTGAAGCTACTGGAGCCGATGTTAGAGAGGTGGCC	ugd	UDP-glucose 6-dehydrogenase/nucleotide sugar dehydrogenase	WP_036901315.:	242239..243696	Amino sugar and nucleotide sugar r	COG1004
4.1_WP_036901315.1_	CUST_3531_PI442182253	MIT0601	LLII/III	4.1_WP_036901315.1_	TAGCCCTAGTCAAATAGAGCGTGACTTGGGACAGGCAAGGGCTAATTTGAAAAATATTGA	ugd	UDP-glucose 6-dehydrogenase/nucleotide sugar dehydrogenase	WP_036901315.:	242239..243696	Amino sugar and nucleotide sugar r	COG1004
4.1_WP_036901315.1_	CUST_3532_PI442182253	MIT0601	LLII/III	4.1_WP_036901315.1_	AGGGTCAAAGTTTTTGGATGCAGGCCCTGGATTTGGTGGTAGTTGTTTTAAAAAGGATAT	ugd	UDP-glucose 6-dehydrogenase/nucleotide sugar dehydrogenase	WP_036901315.:	242239..243696	Amino sugar and nucleotide sugar r	COG1004
4.1_WP_036901315.1_	CUST_3533_PI442182253	MIT0601	LLII/III	4.1_WP_036901315.1_	GGGATATGGTGGTTTTCTGAAGATATTTATGAAGTATGTTCAGCGGCCGATGCAGTGTTG	ugd	UDP-glucose 6-dehydrogenase/nucleotide sugar dehydrogenase	WP_036901315.:	242239..243696	Amino sugar and nucleotide sugar r	COG1004
4.1_WP_036901315.1_	CUST_3534_PI442182253	MIT0601	LLII/III	4.1_WP_036901315.1_	GAACTAATATTTGGAGTAGTGAGTTGGCAAACTACAGCAAATGCCTTTTTGGCCCAGC	ugd	UDP-glucose 6-dehydrogenase/nucleotide sugar dehydrogenase	WP_036901315.:	242239..243696	Amino sugar and nucleotide sugar r	COG1004
4.1_WP_036901315.1_	CUST_3535_PI442182253	MIT0601	LLII/III	4.1_WP_036901315.1_	CTTGTATATTTGTCGCGACACTTTGGTTTGCCAGAAGTTGCTGATTTTGGGAAGGTGTA	ugd	UDP-glucose 6-dehydrogenase/nucleotide sugar dehydrogenase	WP_036901315.:	242239..243696	Amino sugar and nucleotide sugar r	COG1004
4.1_WP_036901503.1_	CUST_4142_PI442182253	MIT0601	LLII/III	4.1_WP_036901503.1_	GCTGTGAAGGCAGGACAAATTAAAACAGGATCATTAAGTAGGAGCGAGAGAGTCGCTAAA	eno	Enolase	WP_036901503.:	345831..347129	Glycolysis/Gluconeogenesis	COG0148
4.1_WP_036901503.1_	CUST_4139_PI442182253	MIT0601	LLII/III	4.1_WP_036901503.1_	CAATCAAATTGGTTCTCTCACTGAAACCTTGCAAGCAATAGAACTTGCTACTCGCTCAGG	eno	Enolase	WP_036901503.:	345831..347129	Glycolysis/Gluconeogenesis	COG0148
4.1_WP_036901503.1_	CUST_4140_PI442182253	MIT0601	LLII/III	4.1_WP_036901503.1_	ATTGTTTCAATCGAAGATGGGCTGGCTGAAGATGATTGGTTGGGTTGGGAATTATTAACA	eno	Enolase	WP_036901503.:	345831..347129	Glycolysis/Gluconeogenesis	COG0148
4.1_WP_036901503.1_	CUST_4141_PI442182253	MIT0601	LLII/III	4.1_WP_036901503.1_	CATGCGGCTGGAGATTTATTAGTTACAGCAATTGAGAAAGCGGGATTTTCTCCTGGAAAA	eno	Enolase	WP_036901503.:	345831..347129	Glycolysis/Gluconeogenesis	COG0148
4.1_WP_036901503.1_	CUST_4143_PI442182253	MIT0601	LLII/III	4.1_WP_036901503.1_	CCTGAAGACACTTCTTCTGATAATGGTTTATCTACGGCTGTTGGTGATGAAGGGGGTTT	eno	Enolase	WP_036901503.:	345831..347129	Glycolysis/Gluconeogenesis	COG0148
4.1_WP_036901503.1_	CUST_4144_PI442182253	MIT0601	LLII/III	4.1_WP_036901503.1_	TGGTGGTAAAACCTTTGCTAGTGATGCAATGGTTAAGGAGTTAATCGCACTTGTAGGTTC	eno	Enolase	WP_036901503.:	345831..347129	Glycolysis/Gluconeogenesis	COG0148
5.1_WP_036902244.1_	CUST_5549_PI442182253	MIT0601	LLII/III	5.1_WP_036902244.1_	ATTTGCTTTATGAAGAACTATCGGAAAAAGATTTTTTTCTTTGAAAGCAAAGCCTTTGT	kaiB	Circadian clock protein KaiB	WP_036902244.:	42092..42421	Circadian Rhythm	COG4251
5.1_WP_036902244.1_	CUST_5550_PI442182253	MIT0601	LLII/III	5.1_WP_036902244.1_	CCAAGATTCTTCCTCCTCCTGTTAGACGAATTATCGGAGATCTTTCCGATAGGGAGAGAG	kaiB	Circadian clock protein KaiB	WP_036902244.:	42092..42421	Circadian Rhythm	COG4251
5.1_WP_036902244.1_	CUST_5551_PI442182253	MIT0601	LLII/III	5.1_WP_036902244.1_	TGATGTTTTAAAAAATCCACAGTTGGCAGAAGAGGACAAAATTCTTGCCACGCCAACATT	kaiB	Circadian clock protein KaiB	WP_036902244.:	42092..42421	Circadian Rhythm	COG4251
5.1_WP_036902247.1_	CUST_5555_PI442182253	MIT0601	LLII/III	5.1_WP_036902247.1_	TCAATCATTCTGCCAACAGGAGCAACTGGTACAGGCAAGACATCTTGGTTTCTAAATTT	kaiC	circadian clock protein KaiC	WP_036902247.:	42547..44049	Circadian Rhythm	COG0467
5.1_WP_036902244.1_	CUST_5552_PI442182253	MIT0601	LLII/III	5.1_WP_036902244.1_	GAAAACGCTTCATGAAATTTTGGAAACTGAATTTAAAGGGGTATATGC	kaiB	Circadian clock protein KaiB	WP_036902244.:	42092..42421	Circadian Rhythm	COG4251
5.1_WP_036902244.1_	CUST_5553_PI442182253	MIT0601	LLII/III	5.1_WP_036902244.1_	TCTCAAGCTTTATGTAGCTGGGAATACCCCTAATTCTATGAGGGCATTGAAAACGCTTCA	kaiB	Circadian clock protein KaiB	WP_036902244.:	42092..42421	Circadian Rhythm	COG4251
5.1_WP_036902247.1_	CUST_5554_PI442182253	MIT0601	LLII/III	5.1_WP_036902247.1_	GTGTCAGCCTCAATGCCTTTAGGCAATTTGTGATAGGTGTTACTGGTTATGCTAAGCAAG	kaiC	circadian clock protein KaiC	WP_036902247.:	42547..44049	Circadian Rhythm	COG0467
5.1_WP_036902247.1_	CUST_5556_PI442182253	MIT0601	LLII/III	5.1_WP_036902247.1_	GTGGTGCGCCTCATCGTATAAATTCTGACGAGACATCCCTGGAGTATTTAAAGGTATTG	kaiC	circadian clock protein KaiC	WP_036902247.:	42547..44049	Circadian Rhythm	COG0467
5.1_WP_036902247.1_	CUST_5557_PI442182253	MIT0601	LLII/III	5.1_WP_036902247.1_	ATGTGCATACCCTGAATCGACAGGCTTGGAGGATCATCTACAGATAATCAAGACAGAGAT	kaiC	circadian clock protein KaiC	WP_036902247.:	42547..44049	Circadian Rhythm	COG0467
5.1_WP_036902247.1_	CUST_5558_PI442182253	MIT0601	LLII/III	5.1_WP_036902247.1_	AGGGGAGAAATGGCTAGAGCAGTTAATGTATTTAAAATGCGTGGATCCTGGCATGATAAG	kaiC	circadian clock protein KaiC	WP_036902247.:	42547..44049	Circadian Rhythm	COG0467

5.1_WP_036902247.1_	CUST_5559_PI442182253	MIT0601	LLII/III	5.1_WP_036902247.1_	ATTTTGTTTGCATATGAAGAGTCCAGAGCTCAACTTCTAAGGAATGCTACAAGTTGGGGT	kaiC	circadian clock protein KaiC	WP_036902247.	42547..44049	Circadian Rhythm	COG0467
5.1_WP_036903536.1_	CUST_5668_PI442182253	MIT0601	LLII/III	5.1_WP_036903536.1_	AATCGGGTTAAGTGTTCAACTGCTCGGCTTGCTTTTGTCATTTTCAGGCTATATCTCAAC	glcH	GPH family sugar transporter	WP_036903536.	62684..64036	GlcH sugar transporter	COG2211
5.1_WP_036903536.1_	CUST_5669_PI442182253	MIT0601	LLII/III	5.1_WP_036903536.1_	AACAACTGTCTAACACAGCCTGATAGCGCTATAGCAACAATAAGAATGAGCATGGGTTA	glcH	GPH family sugar transporter	WP_036903536.	62684..64036	GlcH sugar transporter	COG2211
5.1_WP_036903536.1_	CUST_5670_PI442182253	MIT0601	LLII/III	5.1_WP_036903536.1_	GATCCCCATACCCTTTCAAGTAAGTGCTCTTTTTGGGCTACAATTTTGGAGCATTCACTC	glcH	GPH family sugar transporter	WP_036903536.	62684..64036	GlcH sugar transporter	COG2211
5.1_WP_036903536.1_	CUST_5671_PI442182253	MIT0601	LLII/III	5.1_WP_036903536.1_	CAACAGCATATTTAATACCATGGTCTCTACTCCCAGATGCAATAGATGCTGACCCGGAAA	glcH	GPH family sugar transporter	WP_036903536.	62684..64036	GlcH sugar transporter	COG2211
5.1_WP_036903536.1_	CUST_5672_PI442182253	MIT0601	LLII/III	5.1_WP_036903536.1_	CCCCATTTGCAAAGAAAGCAAGAAAACCAATGCAAAAGCGAGAACCTTTGCATTTTCAAA	glcH	GPH family sugar transporter	WP_036903536.	62684..64036	GlcH sugar transporter	COG2211
5.1_WP_036903536.1_	CUST_5673_PI442182253	MIT0601	LLII/III	5.1_WP_036903536.1_	GGCTTGATATGGATTGGAGCATGCTTAATTGCAATGATTTTACCTCCAATCTCATCTGGT	glcH	GPH family sugar transporter	WP_036903536.	62684..64036	GlcH sugar transporter	COG2211
5.1_WP_036902296.1_	CUST_5726_PI442182253	MIT0601	LLII/III	5.1_WP_036902296.1_	TAAAAGGGCAGGAGACATTGTTTTCTCCCTGACAGTCCTGTTAATAGGCTCTCCAGTATA	glgC	Glucose-1-phosphate adenylyltransferase	WP_036902296.	73121..73759	Glycogen Metabolism	COG0448
5.1_WP_036902296.1_	CUST_5727_PI442182253	MIT0601	LLII/III	5.1_WP_036902296.1_	ACGTGTGACACCTGTTGGTAAATTCCTTAGACGTTCAAGTCTTGATGAGTTGCCTCAATT	glgC	Glucose-1-phosphate adenylyltransferase	WP_036902296.	73121..73759	Glycogen Metabolism	COG0448
5.1_WP_036902296.1_	CUST_5728_PI442182253	MIT0601	LLII/III	5.1_WP_036902296.1_	AGGTGATATGAGTGTTGTTGGTCCTAGGCCTATTATCCAGGAAGAGGTAATTCGTTATGG	glgC	Glucose-1-phosphate adenylyltransferase	WP_036902296.	73121..73759	Glycogen Metabolism	COG0448
5.1_WP_036902296.1_	CUST_5729_PI442182253	MIT0601	LLII/III	5.1_WP_036902296.1_	TTACTTGTGAAAATAAGCTCTTCTGGACCAGTCTTTTATAAACAAGAAAGGGTTGGCCGT	glgC	Glucose-1-phosphate adenylyltransferase	WP_036902296.	73121..73759	Glycogen Metabolism	COG0448
5.1_WP_036902296.1_	CUST_5730_PI442182253	MIT0601	LLII/III	5.1_WP_036902296.1_	TGTATGAATGAAGTCAATGCTATAAAGCCTGGTATAACTGGCTTATGGCAAATTAGTGGT	glgC	Glucose-1-phosphate adenylyltransferase	WP_036902296.	73121..73759	Glycogen Metabolism	COG0448
5.1_WP_036902296.1_	CUST_5731_PI442182253	MIT0601	LLII/III	5.1_WP_036902296.1_	AGAAGAGTAGATCTAGATATTGCTTATGTACGCAAAAGGAGTCTTTTGATGGACTTTAGA	glgC	Glucose-1-phosphate adenylyltransferase	WP_036902296.	73121..73759	Glycogen Metabolism	COG0448
5.1_WP_036902311.1_	CUST_5776_PI442182253	MIT0601	LLII/III	5.1_WP_036902311.1_	ACGATATACTAAGAATGGGCGTCAAAGGCATTAGTGACATAATCACTTGTCCTGGGTTGG	ftsZ	cell division protein FtsZ	WP_036902311.	80629..81747	Cell Division	COG0206
5.1_WP_036902311.1_	CUST_5774_PI442182253	MIT0601	LLII/III	5.1_WP_036902311.1_	TGATTTCAGATGTAGTGGACCCAGAGGCAAATATTATTGTAGGTGCAGTTATTGACCCGG	ftsZ	cell division protein FtsZ	WP_036902311.	80629..81747	Cell Division	COG0206
5.1_WP_036902311.1_	CUST_5775_PI442182253	MIT0601	LLII/III	5.1_WP_036902311.1_	CATACCAAATGATCGATTAAAAGATGTCAATGCAGCTGCTCCTCTCCAAGAAGCCTTTCG	ftsZ	cell division protein FtsZ	WP_036902311.	80629..81747	Cell Division	COG0206
5.1_WP_036902311.1_	CUST_5777_PI442182253	MIT0601	LLII/III	5.1_WP_036902311.1_	GCTGCAATAAATAGTCCTTTACTTGAAGCCAGTCGCATTGATGGTGCTAAAGGATGTGTT	ftsZ	cell division protein FtsZ	WP_036902311.	80629..81747	Cell Division	COG0206
5.1_WP_036902311.1_	CUST_5778_PI442182253	MIT0601	LLII/III	5.1_WP_036902311.1_	AATGCGTCAAGCCGACGAAGGGATAGAAAGACTTGCAGAAAATGTTGACACATTAATTGT	ftsZ	cell division protein FtsZ	WP_036902311.	80629..81747	Cell Division	COG0206
5.1_WP_036902311.1_	CUST_5779_PI442182253	MIT0601	LLII/III	5.1_WP_036902311.1_	GGCGAAGTCCAAGTAACTGTCATAGCAACTGGATTTAATGGCAGCCAGCCTTATAAAAAT	ftsZ	cell division protein FtsZ	WP_036902311.	80629..81747	Cell Division	COG0206
5.1_WP_036902357.1_	CUST_5900_PI442182253	MIT0601	LLII/III	5.1_WP_036902357.1_	GCAGCACATATCGTGATCATGTTCATGCTTTAAGTGCTGGAGTTCCTGCAAAAGAAGTAA	pdhA	Pyruvate dehydrogenase E1 component alpha subunit	WP_036902357.	104349..105434	Glycolysis/Krebs cycle	COG1071
5.1_WP_036902357.1_	CUST_5901_PI442182253	MIT0601	LLII/III	5.1_WP_036902357.1_	CTGCTGCCTTCTTTGGAGATGGGACTTGCAATAATGGTCAATTCTTCGAATGCTTGAATA	pdhA	Pyruvate dehydrogenase E1 component alpha subunit	WP_036902357.	104349..105434	Glycolysis/Krebs cycle	COG1071
5.1_WP_036902357.1_	CUST_5902_PI442182253	MIT0601	LLII/III	5.1_WP_036902357.1_	GCAAGATCAGGAGAAGGGCCTAGCTTGATTGAATGCCTTACATACAGATTTAGAGGTCAC	pdhA	Pyruvate dehydrogenase E1 component alpha subunit	WP_036902357.	104349..105434	Glycolysis/Krebs cycle	COG1071
5.1_WP_036902357.1_	CUST_5903_PI442182253	MIT0601	LLII/III	5.1_WP_036902357.1_	ACTGCCAATAATTTTCGTTGTTGAAAACAACAAATGGGCTATAGGAATGGCGCATGATCG	pdhA	Pyruvate dehydrogenase E1 component alpha subunit	WP_036902357.	104349..105434	Glycolysis/Krebs cycle	COG1071
5.1_WP_036902357.1_	CUST_5904_PI442182253	MIT0601	LLII/III	5.1_WP_036902357.1_	GTTGTTTGGTAAAGAAACAGGATGCAGCAAAGGTAGAGGAGGATCAATGCACCTCTTTTC	pdhA	Pyruvate dehydrogenase E1 component alpha subunit	WP_036902357.	104349..105434	Glycolysis/Krebs cycle	COG1071
5.1_WP_036902357.1_	CUST_5905_PI442182253	MIT0601	LLII/III	5.1_WP_036902357.1_	AAAGAAATAAACGATGCTGTTGAATATGCTCTTAGCTCTAAAGACCCCGACCCCAACGAA	pdhA	Pyruvate dehydrogenase E1 component alpha subunit	WP_036902357.	104349..105434	Glycolysis/Krebs cycle	COG1071
5.1_WP_081934289.1_	CUST_6055_PI442182253	MIT0601	LLII/III	5.1_WP_081934289.1_	ACCTGCTAATCTTTTACCATTAGATGTTGCACTACCCAGGAACTGTACTGATTGGGATCG	dld	D-lactate dehydrogenase, NADH independent	WP_081934289.	130456..132156	Pyruvate metabolism	COG0277
5.1_WP_081934289.1_	CUST_6053_PI442182253	MIT0601	LLII/III	5.1_WP_081934289.1_	TGACCATGCACAATTATTGCCTGATCACCTTCCTAAGATTGCAAGAGAATATAGACGGCT	dld	D-lactate dehydrogenase, NADH independent	WP_081934289.	130456..132156	Pyruvate metabolism	COG0277
5.1_WP_081934289.1_	CUST_6054_PI442182253	MIT0601	LLII/III	5.1_WP_081934289.1_	GCTTAAAGGTGGAAGGTCAAGATTTACTTTTGCATAGATACGTAGCAGGAATTGCGCCAA	dld	D-lactate dehydrogenase, NADH independent	WP_081934289.	130456..132156	Pyruvate metabolism	COG0277
5.1_WP_081934289.1_	CUST_6056_PI442182253	MIT0601	LLII/III	5.1_WP_081934289.1_	AGGAGCAAAATACCCAGCAGAGCATAATGTAGGTCATCTCTATTTTGCTGAATACGACCT	dld	D-lactate dehydrogenase, NADH independent	WP_081934289.	130456..132156	Pyruvate metabolism	COG0277
5.1_WP_081934289.1_	CUST_6057_PI442182253	MIT0601	LLII/III	5.1_WP_081934289.1_	TGCAGCATCTAAATATGGTAAAGACACATTTCTAATAATTAAATACCTTGGCCGTGGCTT	dld	D-lactate dehydrogenase, NADH independent	WP_081934289.	130456..132156	Pyruvate metabolism	COG0277
5.1_WP_081934289.1_	CUST_6058_PI442182253	MIT0601	LLII/III	5.1_WP_081934289.1_	AACTGGAATATTAGAGTCTTTTCGTATGGGACATTTTCTTTGTATGGTCTTTCATTGGGA	dld	D-lactate dehydrogenase, NADH independent	WP_081934289.	130456..132156	Pyruvate metabolism	COG0277
5.1_WP_036902439.1_	CUST_6151_PI442182253	MIT0601	LLII/III	5.1_WP_036902439.1_	TGGAAGCCAAAAAGGACTTTGGAAGATATTTGTAGAGATGGATGGCAATGGCAAAAGCTA	galE	UDP-glucose 4-epimerase	WP_036902439.	147824..148867	Galactose metabolism	COG1087
5.1_WP_036902439.1_	CUST_6149_PI442182253	MIT0601	LLII/III	5.1_WP_036902439.1_	TATTGATGGCACTGGTGTACGTGATTATATCCACGTAATGGACTTAGCCGAAGCACATTC	galE	UDP-glucose 4-epimerase	WP_036902439.	147824..148867	Galactose metabolism	COG1087
5.1_WP_036902439.1_	CUST_6150_PI442182253	MIT0601	LLII/III	5.1_WP_036902439.1_	TGGTACTGGCAAAGGTACCAGTGTCCTCGAACTTGTTAAGACTTTTGAAGCTGTGAATAA	galE	UDP-glucose 4-epimerase	WP_036902439.	147824..148867	Galactose metabolism	COG1087
5.1_WP_036902439.1_	CUST_6152_PI442182253	MIT0601	LLII/III	5.1_WP_036902439.1_	GCACACCCTTCCGGTAAGATAGGAGAGCATCCTTTGGAGAAGCCAAATAATTTATTTCCT	galE	UDP-glucose 4-epimerase	WP_036902439.	147824..148867	Galactose metabolism	COG1087
5.1_WP_036902439.1_	CUST_6153_PI442182253	MIT0601	LLII/III	5.1_WP_036902439.1_	TGTCAAATCCCATATGTTTTTTCTAACCGAAGACTGGGAGATGTACCATTTGCTGTTGCA	galE	UDP-glucose 4-epimerase	WP_036902439.	147824..148867	Galactose metabolism	COG1087
5.1_WP_036902439.1_	CUST_6154_PI442182253	MIT0601	LLII/III	5.1_WP_036902439.1_	TCAGTAGCTGAATCTGTGAATGAACCTATGCTTTATTGGGATGTAAATGTTGCTGGTGCA	galE	UDP-glucose 4-epimerase	WP_036902439.	147824..148867	Galactose metabolism	COG1087
5.1_WP_052042966.1_	CUST_6865_PI442182253	MIT0601	LLII/III	5.1_WP_052042966.1_	TTCAAGAAAGCCAACGATTGATTCTTATGGATGTGGAGATTGCTTTGCTGCAGGAGTTAC	rbsK	Sugar kinase, ribokinase family	WP_052042966.	274317..275159	Pentose Phosphate	COG0524
5.1_WP_052042966.1_	CUST_6866_PI442182253	MIT0601	LLII/III	5.1_WP_052042966.1_	CAAAGGTAATTACAGCAACCCCACGAGTTGGCCTTCAAGCATTAAAAAAGGCAAATGTAC	rbsK	Sugar kinase, ribokinase family	WP_052042966.	274317..275159	Pentose Phosphate	COG0524
5.1_WP_052042966.1_	CUST_6867_PI442182253	MIT0601	LLII/III	5.1_WP_052042966.1_	AGTTCACTTTTTTACGGCTCTAGGACGCGATGAGGAAGGTCTTAAGAGTGCAAAGATTTT	rbsK	Sugar kinase, ribokinase family	WP_052042966.	274317..275159	Pentose Phosphate	COG0524
5.1_WP_052042966.1_	CUST_6868_PI442182253	MIT0601	LLII/III	5.1_WP_052042966.1_	CTTGGAGAGATAAGCCTACAAGGAAAGGCATTAGCTTTATCGATGATGATGGCGAAAGGA	rbsK	Sugar kinase, ribokinase family	WP_052042966.	274317..275159	Pentose Phosphate	COG0524
5.1_WP_052042966.1_	CUST_6869_PI442182253	MIT0601	LLII/III	5.1_WP_052042966.1_	ATTAGCGAAACCAGGCGAAATAAATCATGCAAATAAATTTACAGTCCTACCCGCTGGAGG	rbsK	Sugar kinase, ribokinase family	WP_052042966.	274317..275159	Pentose Phosphate	COG0524
5.1_WP_052042966.1_	CUST_6870_PI442182253	MIT0601	LLII/III	5.1_WP_052042966.1_	AGTAATTGGAGAAAGGCTTCAGCCAAACTCGGAAGACGATCTTGAATGGTCTATGTTGAA	rbsK	Sugar kinase, ribokinase family	WP_052042966.	274317..275159	Pentose Phosphate	COG0524
5.1_WP_036902638.1_	CUST_7046_PI442182253	MIT0601	LLII/III	5.1_WP_036902638.1_	GTTGGGAGAGCCTCATCGATGTCTTAAATTGCTTGTAACAACTATTGAGAATGGCCAAGC	opcA	Glucose-6-P dehydrogenase subunit opcA	WP_036902638.	307030..308337	Pentose Phosphate	COG3429
5.1_WP_036902638.1_	CUST_7047_PI442182253	MIT0601	LLII/III	5.1_WP_036902638.1_	GAAGCAGGAGGCATGGCAAGCATGGAACTAATAGAAGAAGTTGTGCCAATTCAAAAAAAC	opcA	Glucose-6-P dehydrogenase subunit opcA	WP_036902638.	307030..308337	Pentose Phosphate	COG3429
5.1_WP_036902638.1_	CUST_7048_PI442182253	MIT0601	LLII/III	5.1_WP_036902638.1_	TTGAAGATTTAATACCTGAAGACCTTCCCTCATGGCTTTGGTGGAATGGAGTGCTAGATG	opcA	Glucose-6-P dehydrogenase subunit opcA	WP_036902638.	307030..308337	Pentose Phosphate	COG3429
5.1_WP_036902639.1_	CUST_7052_PI442182253	MIT0601	LLII/III	5.1_WP_036902639.1_	TCACTAGAAGTGATGAAGTTGAGGCGGCTTGGCGCTTATACACACCTTTACTTGAATTAA	zwf	glucose-6-phosphate dehydrogenase zwf	WP_036902639.	308372..309895	Pentose Phosphate	COG0364
5.1_WP_036902638.1_	CUST_7049_PI442182253	MIT0601	LLII/III	5.1_WP_036902638.1_	ATAGATATTGACATTGAAGGCAATAACCCTGTTCAAGGACTACTTCTTGCCGCCTGGATT	opcA	Glucose-6-P dehydrogenase subunit opcA	WP_036902638.	307030..308337	Pentose Phosphate	COG3429
5.1_WP_036902638.1_	CUST_7050_PI442182253	MIT0601	LLII/III	5.1_WP_036902638.1_	CAACAAACAGTTATTTTCCGATTAATGCCTTTACCAGTCGGGAAACCAAGTATCCACCCA	opcA	Glucose-6-P dehydrogenase subunit opcA	WP_036902638.	307030..308337	Pentose Phosphate	COG3429
5.1_WP_036902638.1_	CUST_7051_PI442182253	MIT0601	LLII/III	5.1_WP_036902638.1_	CGTGGCGAGAGACTTTGGCAATGGTATTTGATCCACCTGATCGTCGATCTATACTTAATA	opcA	Glucose-6-P dehydrogenase subunit opcA	WP_036902638.	307030..308337	Pentose Phosphate	COG3429
5.1_WP_036902639.1_	CUST_7053_PI442182253	MIT0601	LLII/III	5.1_WP_036902639.1_	GAACCATGGAAATGCTGTGTAAGAGGGCAATACTCAAAAGGAGGTAACAAAGCAAATCCA	zwf	glucose-6-phosphate dehydrogenase zwf	WP_036902639.	308372..309895	Pentose Phosphate	COG0364
5.1_WP_036902639.1_	CUST_7054_PI442182253	MIT0601	LLII/III	5.1_WP_036902639.1_	TGGGGGAAGTCCTACGTCAAACCAATTAATTCTTAGAATTCAACCCAATGAAGGAGCAGA	zwf	glucose-6-phosphate dehydrogenase zwf	WP_036902639.	308372..309895	Pentose Phosphate	COG0364
5.1_WP_036902639.1_	CUST_7055_PI442182253	MIT0601	LLII/III	5.1_WP_036902639.1_	GCGTTAAGGGATATGGTGCAAAACCATTTAACACAAATGCTTGCGATAACAGCAATGGAA	zwf	glucose-6-phosphate dehydrogenase zwf	WP_036902639.	308372..309895	Pentose Phosphate	COG0364
5.1_WP_036902639.1_	CUST_7056_PI442182253	MIT0601	LLII/III	5.1_WP_036902639.1_	TATACGTACTGGGAAACGACTGGCTAAAAGGACAAGCGAGGTTGTACTTACTTTTCGAGA	zwf	glucose-6-phosphate dehydrogenase zwf	WP_036902639.	308372..309895	Pentose Phosphate	COG0364
5.1_WP_036902639.1_	CUST_7057_PI442182253	MIT0601	LLII/III	5.1_WP_036902639.1_	GCAGGCCTGTTGAGATGGAATTTTCATATGACGAATCATTTGGAGAGCCTTCTGATGAAG	zwf	glucose-6-phosphate dehydrogenase zwf	WP_036902639.	308372..309895	Pentose Phosphate	COG0364
5.1_WP_036902706.1_	CUST_7384_PI442182253	MIT0601	LLII/III	5.1_WP_036902706.1_	TACAAGCTCTACATATTTGGCTAAGTGCTTATGGCTCAGCAGTTGGAGATCTTGCATTGA	glk	Glucokinase	WP_036902706.	375820..376875	Glycolysis	COG0837
5.1_WP_036902706.1_	CUST_7385_PI442182253	MIT0601	LLII/III	5.1_WP_036902706.1_	GGCATCGCAAGAGGAGTAATAACTGAAGGAGGTGTTATATCTCTTCCTAGTGAAGGTGGT	glk	Glucokinase	WP_036902706.	375820..376875	Glycolysis	COG0837
5.1_WP_036902706.1_	CUST_7386_PI442182253	MIT0601	LLII/III	5.1_WP_036902706.1_	AAGGGTTTCCCTTGAAAGAATTGTAAGTGGCAAAGGGCTTGGGTATATAGCAATTTGGAG	glk	Glucokinase	WP_036902706.	375820..376875	Glycolysis	COG0837
5.1_WP_036902706.1_	CUST_7387_PI442182253	MIT0601	LLII/III	5.1_WP_036902706.1_	TGCAACTTTTAAGTCAGCCATGACAAACAAGGGACGCTTTTCGGAGTTTATTAAAGAGCT	glk	Glucokinase	WP_036902706.	375820..376875	Glycolysis	COG0837
5.1_WP_036902706.1_	CUST_7388_PI442182253	MIT0601	LLII/III	5.1_WP_036902706.1_	TTTTGACAGATCCCGAGATAGGTCTATTAGTGCAGCGTGCAAAGCTCATTTAATAGCCA	glk	Glucokinase	WP_036902706.	375820..376875	Glycolysis	COG0837
5.1_WP_036902706.1_	CUST_7389_PI442182253	MIT0601	LLII/III	5.1_WP_036902706.1_	CACAGGTGGCCTATGGATAGCGGGAGGACCAGCTTCAAAAAATATAGATGGAATAACTTC	glk	Glucokinase	WP_036902706.	375820..376875	Glycolysis	COG0837
5.1_WP_036902760.1_	CUST_7546_PI442182253	MIT0601	LLII/III	5.1_WP_036902760.1_	AACATGAATTAGGAATGAAGTTCGCTTCATTGATCAATGCAGCGGACAATGTCATGACCT	glnA	Glutamine synthetase/ glutamate--ammonialigase	WP_036902760.	406243..407664	Nitrogen Metabolism	COG0174
5.1_WP_036902760.1_	CUST_7544_PI442182253	MIT0601	LLII/III	5.1_WP_036902760.1_	CAGGTGATGCTATGGCAAATCCATACTTAGCTTTTAGTGCAATGATGATGGCGGGAATAG	glnA	Glutamine synthetase/ glutamate--ammonialigase	WP_036902760.	406243..407664	Nitrogen Metabolism	COG0174
5.1_WP_036902760.1_	CUST_7545_PI442182253	MIT0601	LLII/III	5.1_WP_036902760.1_	AAATTGATCCTGGTGATGGAGTAGATGTAGACCTTTTCGAGCTTCCCTCAGAGGAACTAG	glnA	Glutamine synthetase/ glutamate--ammonialigase	WP_036902760.	406243..407664	Nitrogen Metabolism	COG0174
5.1_WP_036902760.1_	CUST_7547_PI442182253	MIT0601	LLII/III	5.1_WP_036902760.1_	TCATCTTTAAACGACGCATTAGACGCTCTCAAATCTGACAATGAATACCTCACAGCTGGT	glnA	Glutamine synthetase/ glutamate--ammonialigase	WP_036902760.	406243..407664	Nitrogen Metabolism	COG0174
5.1_WP_036902760.1_	CUST_7548_PI442182253	MIT0601	LLII/III	5.1_WP_036902760.1_	TAAGCGATTAGTTCCAGGATTCGAAGCCCCAGTTAATTTGGTTTACTCACAAGGTAACCG	glnA	Glutamine synthetase/ glutamate--ammonialigase	WP_036902760.	406243..407664	Nitrogen Metabolism	COG0174
5.1_WP_036902760.1_	CUST_7549_PI442182253	MIT0601	LLII/III	5.1_WP_036902760.1_	TCAGCCTTTATTCTTTGGAGAGGGGACATATGCAAACCTTTCTCAGACTGCTAAATGGTA	glnA	Glutamine synthetase/ glutamate--ammonialigase	WP_036902760.	406243..407664	Nitrogen Metabolism	COG0174
5.1_WP_036902820.1_	CUST_7735_PI442182253	MIT0601	LLII/III	5.1_WP_036902820.1_	TGGCATCCCTCTAGGAGTAGGAAAGGGAACAACTGTCAAGAGAGCTATCCTTGATAAAAA	glgC	Glucose-1-phosphate adenylyltransferase	WP_036902820.	442706..444001	Glycogen Metabolism	COG0448
5.1_WP_036902820.1_	CUST_7736_PI442182253	MIT0601	LLII/III	5.1_WP_036902820.1_	CAGGCGATTCTTTGAAGGCAATGGCAGTAGATACCTCGAGATTCGGATTAGATGAAGAAT	glgC	Glucose-1-phosphate adenylyltransferase	WP_036902820.	442706..444001	Glycogen Metabolism	COG0448
5.1_WP_036902820.1_	CUST_7737_PI442182253	MIT0601	LLII/III	5.1_WP_036902820.1_	ACGTAACTATCGTGAACAAAGACAGGATTGAAGAAGCTGACAGACCTGAAGAGGGTTTTT	glgC	Glucose-1-phosphate adenylyltransferase	WP_036902820.	442706..444001	Glycogen Metabolism	COG0448
5.1_WP_036902820.1_	CUST_7738_PI442182253	MIT0601	LLII/III	5.1_WP_036902820.1_	GGAACAATAGGAGCATTTTTTGACTCAAATTTAGCGCTTACTCAGCAACCAAAACCTCCT	glgC	Glucose-1-phosphate adenylyltransferase	WP_036902820.	442706..444001	Glycogen Metabolism	COG0448
5.1_WP_036902820.1_	CUST_7739_PI442182253	MIT0601	LLII/III	5.1_WP_036902820.1_	TCGAGAGATAAACCCTACTTGGCTTCAATGGGAATTTATGTTTTCAGTCGAGAAACGCTT	glgC	Glucose-1-phosphate adenylyltransferase	WP_036902820.	442706..444001	Glycogen Metabolism	COG0448
5.1_WP_036902820.1_	CUST_7740_PI442182253	MIT0601	LLII/III	5.1_WP_036902820.1_	TGTAATGGGATCAGACTTTTATGAATCAGGAGAAGAGAGAATTGCTCTCCGATCTGGAGG	glgC	Glucose-1-phosphate adenylyltransferase	WP_036902820.	442706..444001	Glycogen Metabolism	COG0448
5.1_WP_036902822.1_	CUST_7741_PI442182253	MIT0601	LLII/III	5.1_WP_036902822.1_	CAAAGGACCCTTCCGATGGCTCAAATCTTGTAGAAAAAATTGTGGATAAGGCTGGTCAAA	edd	phosphogluconate dehydrogenase (NADP(+)-dependent, decarboxyla	WP_036902822.	444154..445569	Pentose Phosphate/Entner-Dudorof	COG2084
5.1_WP_036902822.1_	CUST_7742_PI442182253	MIT0601	LLII/III	5.1_WP_036902822.1_	TTAAATAGCTACAGAACTTCTCGGTTGCCTCAAAATTTGATACAGGCGATGCGTGATTGC	edd	phosphogluconate dehydrogenase (NADP(+)-dependent, decarboxyla	WP_036902822.	444154..445569	Pentose Phosphate/Entner-Dudorof	COG2084
5.1_WP_036902822.1_	CUST_7745_PI442182253	MIT0601	LLII/III	5.1_WP_036902822.1_	GGCCATTATGGATGCTGTTGTCTTGTCTACTATTGCAAGTTATGCACAGGGCATTGAAAT	edd	phosphogluconate dehydrogenase (NADP(+)-dependent, decarboxyla	WP_036902822.	444154..445569	Pentose Phosphate/Entner-Dudorof	COG2084
5.1_WP_036902822.1_	CUST_7743_PI442182253	MIT0601	LLII/III	5.1_WP_036902822.1_	CTTGGTTTTGCACTCAAATCCAAAAAAGGCTGCCAGGACTTTCAAAGGTTGTATCTCATG	edd	phosphogluconate dehydrogenase (NADP(+)-dependent, decarboxyla	WP_036902822.	444154..445569	Pentose Phosphate/Entner-Dudorof	COG2084
5.1_WP_036902822.1_	CUST_7744_PI442182253	MIT0601	LLII/III	5.1_WP_036902822.1_	TAGGTGCTTCAGTTCCAACAATATATGCTGCTCTTAATGCCCGTGTTATGAGCTCCTTAA	edd	phosphogluconate dehydrogenase (NADP(+)-dependent, decarboxyla	WP_036902822.	444154..445569	Pentose Phosphate/Entner-Dudorof	COG2084
5.1_WP_036902822.1_	CUST_7746_PI442182253	MIT0601	LLII/III	5.1_WP_036902822.1_	GGCTCCCATACCTATGAGCGTATAGATATGGATGGTGTGTACCACACTGAATGGTTGTAA	edd	phosphogluconate dehydrogenase (NADP(+)-dependent, decarboxyla	WP_036902822.	444154..445569	Pentose Phosphate/Entner-Dudorof	COG2084
5.1_WP_036902839.1_	CUST_7804_PI442182253	MIT0601	LLII/III	5.1_WP_036902839.1_	GCAATAGCTATTGGAACAAGTCATGGCGCTTATAAATTCACAAGGAAGCCAACAGGAGAA	cbbA	Fructose-1,6-bisphosphate aldolase class II	WP_036902839.	452980..454053	Glycolysis/Gluconeogenesis	COG0191
5.1_WP_036902839.1_	CUST_7805_PI442182253	MIT0601	LLII/III	5.1_WP_036902839.1_	AATTATTATGCTGGGCTTTATGCCAAAGGTTCACTTGAACCTAAGACATCAGTTGCTGCA	cbbA	Fructose-1,6-bisphosphate aldolase class II	WP_036902839.	452980..454053	Glycolysis/Gluconeogenesis	COG0191
5.1_WP_036902839.1_	CUST_7806_PI442182253	MIT0601	LLII/III	5.1_WP_036902839.1_	CATTAGAGGCTGATGCAAAAACACCCGCAAGCTATGACTACACAACGTCGAAGTAACAAAA	cbbA	Fructose-1,6-bisphosphate aldolase class II	WP_036902839.	452980..454053	Glycolysis/Gluconeogenesis	COG0191
5.1_WP_036902839.1_	CUST_7807_PI442182253	MIT0601	LLII/III	5.1_WP_036902839.1_	AAGCAAGTTTGTTTAGACAGGTACCAACAATTCTGGTGCGCTGGTAATGCAAGTAAGATC	cbbA	Fructose-1,6-bisphosphate aldolase class II	WP_036902839.	452980..454053	Glycolysis/Gluconeogenesis	COG0191
5.1_WP_036902839.1_	CUST_7808_PI442182253	MIT0601	LLII/III	5.1_WP_036902839.1_	AGCCTTACCCAATACTCATCTTGTTATGCATGGATCTAGTTCAGTTCCACAAGAATGGCT	cbbA	Fructose-1,6-bisphosphate aldolase class II	WP_036902839.	452980..454053	Glycolysis/Gluconeogenesis	COG0191
5.1_WP_036902839.1_	CUST_7809_PI442182253	MIT0601	LLII/III	5.1_WP_036902839.1_	CAGCATCTGCAGATCCTACGAACTTTGACCCAAGACATTTCAACAAACCTGCTAGAAAAT	cbbA	Fructose-1,6-bisphosphate aldolase class II	WP_036902839.	452980..454053	Glycolysis/Gluconeogenesis	COG0191
5.1_WP_036902841.1_	CUST_7810_PI442182253	MIT0601	LLII/III	5.1_WP_036902841.1_	TATCGGTTTTTCTTACGGAAGGGCATTACAACATTCCTGCTTAAAGGAATGGAAAGGCAC	fda	Fructose-1,6-bisphosphate aldolase class I	WP_036902841.	454204..455271	Glycolysis/Gluconeogenesis	COG0191
5.1_WP_036902841.1_	CUST_7811_PI442182253	MIT0601	LLII/III	5.1_WP_036902841.1_	CGCTTTGCTAAATGGAGAGCTGTCCTTCAAATTACTGATGATGGATGCCCATCAAATCTT	fda	Fructose-1,6-bisphosphate aldolase class I	WP_036902841.	454204..455271	Glycolysis/Gluconeogenesis	COG0191
5.1_WP_036902841.1_	CUST_7812_PI442182253	MIT0601	LLII/III	5.1_WP_036902841.1_	GTCTTCCTAGAAGGAACTCTTTTAAAACCTTCGATGACAGTTCAAGGCGCGGAATGTACT	fda	Fructose-1,6-bisphosphate aldolase class I	WP_036902841.	454204..455271	Glycolysis/Gluconeogenesis	COG0191
5.1_WP_036902841.1_	CUST_7813_PI442182253	MIT0601	LLII/III	5.1_WP_036902841.1_	GTTATAGTGCAGGGTCTCAGCCATCGTCAGATGAAGCTCTTTTTGTAGCTGGCTATAAAT	fda	Fructose-1,6-bisphosphate aldolase class I	WP_036902841.	454204..455271	Glycolysis/Gluconeogenesis	COG0191
5.1_WP_036902841.1_	CUST_7814_PI442182253	MIT0601	LLII/III	5.1_WP_036902841.1_	AGATAAAGGACTACGACCTCTCTCTGGAGCCAATGATGTTGAAACTTCTGTTCAGGACT	fda	Fructose-1,6-bisphosphate aldolase class I	WP_036902841.	454204..455271	Glycolysis/Gluconeogenesis	COG0191
5.1_WP_036902841.1_	CUST_7815_PI442182253	MIT0601	LLII/III	5.1_WP_036902841.1_	CCAGAAAAAATAGCAGAATTTACAATTCGGACAATGGAACGATCTGTCCCAGCGAGTGTG	fda	Fructose-1,6-bisphosphate aldolase class I	WP_036902841.	454204..455271	Glycolysis/Gluconeogenesis	COG0191
5.1_WP_036902844.1_	CUST_7836_PI442182253	MIT0601	LLII/III	5.1_WP_036902844.1_	CTGAGAGAGGGTATTCGGCAGAAACAATTGTTGAAACAATCCTTAGAAGGATGCCTGACT	prk	Phosphoribulokinase	WP_036902844.	457913..458818	Carbon Fixation	COG3954
5.1_WP_036902844.1_	CUST_7833_PI442182253	MIT0601	LLII/III	5.1_WP_036902844.1_	CCTAAAGGTACTGATGTTCTTTTTATGAAGGTCTTCATGGCGGTGTCTCTGGAGAAGGG	prk	Phosphoribulokinase	WP_036902844.	457913..458818	Carbon Fixation	COG3954
5.1_WP_036902844.1_	CUST_7834_PI442182253	MIT0601	LLII/III	5.1_WP_036902844.1_	CTATGAAGCAAGCGATGTCTGATGCTCTTGCAAAAGGAGAAAACTTTTCTCACTTCGGAC	prk	Phosphoribulokinase	WP_036902844.	457913..458818	Carbon Fixation	COG3954
5.1_WP_036902844.1_	CUST_7835_PI442182253	MIT0601	LLII/III	5.1_WP_036902844.1_	CATCCCTTGCTGATCTCCTCGTTGGCGTTGTACCAATAACAAATCTTGAATGGATTCAAA	prk	Phosphoribulokinase	WP_036902844.	457913..458818	Carbon Fixation	COG3954
5.1_WP_036902844.1_	CUST_7837_PI442182253	MIT0601	LLII/III	5.1_WP_036902844.1_	AACAGGAGGTGGTCAAAAGCGTTATTACCTCCACAGTCAAGAAGAAGCAGAAGAACATAA	prk	Phosphoribulokinase	WP_036902844.	457913..458818	Carbon Fixation	COG3954
5.1_WP_036902844.1_	CUST_7838_PI442182253	MIT0601	LLII/III	5.1_WP_036902844.1_	CATGTCAAGTCCAACTAGTATCGTTGTTAATGGTGGGAAAATGGGATTCGCAATGGAACT	prk	Phosphoribulokinase	WP_036902844.	457913..458818	Carbon Fixation	COG3954
5.1_WP_036903026.1_	CUST_8388_PI442182253	MIT0601	LLII/III	5.1_WP_036903026.1_	AGGGTCAACAGATCAGCATGCATATGTTCAGCAGTTACGTGATGGACTTGATAACTTCTT	pgi	Glucose-6-phosphate isomerase	WP_036903026.	559622..561220	Glycolysis/Gluconeogenesis	COG0166
5.1_WP_036903026.1_	CUST_8389_PI442182253	MIT0601	LLII/III	5.1_WP_036903026.1_	CAATCGTTTCTGAGAATCCATCTGCACTGTTGGCTTCAGCTTGGTATTCATCTGGAAAAG	pgi	Glucose-6-phosphate isomerase	WP_036903026.	559622..561220	Glycolysis/Gluconeogenesis	COG0166
5.1_WP_036903026.1_	CUST_8390_PI442182253	MIT0601	LLII/III	5.1_WP_036903026.1_	ATGGAATCTTTAGGTAAGAAGTTGGACAGAAATTCTAAAGAGGTCTGCCAAGGGCTTGCT	pgi	Glucose-6-phosphate isomerase	WP_036903026.	559622..561220	Glycolysis/Gluconeogenesis	COG0166
5.1_WP_036903026.1_	CUST_8391_PI442182253	MIT0601	LLII/III	5.1_WP_036903026.1_	AAAAAATTTGACCCTTGTTCGTTAGGTGCGTTAATTGCTCTTTTCGAAAGAGCAGTTGGC	pgi	Glucose-6-phosphate isomerase	WP_036903026.	559622..561220	Glycolysis/Gluconeogenesis	COG0166
5.1_WP_036903026.1_	CUST_8392_PI442182253	MIT0601	LLII/III	5.1_WP_036903026.1_	CATCAACCTGGTGTAGAAGCAGGTAAGAAAGCTGCAGCTGATGTATTATTTTTGCAGAGT	pgi	Glucose-6-phosphate isomerase	WP_036903026.	559622..561220	Glycolysis/Gluconeogenesis	COG0166
5.1_WP_036903026.1_	CUST_8393_PI442182253	MIT0601	LLII/III	5.1_WP_036903026.1_	TCAAGGTAAATCCCCTGGAGATTTTTTATCAGGCTTCTTTCAAGGGACTCGTTTAGCATT	pgi	Glucose-6-phosphate isomerase	WP_036903026.	559622..561220	Glycolysis/Gluconeogenesis	COG0166
5.1_WP_036903087.1_	CUST_8533_PI442182253	MIT0601	LLII/III	5.1_WP_036903087.1_	AGGCACACTCACTGGCATAAGTGGTTCCACTGATTTAATAAAGGTTGGGATAGTTAACGA	pykF	Pyruvate kinase	WP_036903087.	585216..586970	Glycolysis	COG0469
5.1_WP_036903087.1_	CUST_8534_PI442182253	MIT0601	LLII/III	5.1_WP_036903087.1_	TATGGAAATAGCACAAAATTATGGTCTACTCAAACCTGGCGATCTTGTTGTCCAAACGGC	pykF	Pyruvate kinase	WP_036903087.	585216..586970	Glycolysis	COG0469
5.1_WP_036903087.1_	CUST_8535_PI442182253	MIT0601	LLII/III	5.1_WP_036903087.1_	ACCAACTGTCGCCAGAAGACTTCAATTAGTTTGGGGTGTAAGACCATTAGTAGTTCCAAC	pykF	Pyruvate kinase	WP_036903087.	585216..586970	Glycolysis	COG0469
5.1_WP_036903087.1_	CUST_8536_PI442182253	MIT0601	LLII/III	5.1_WP_036903087.1_	GCTATAAGTGCAGCTGTGAGCTCTATTGCTAGACAATTAAATGCAACCGCAATATTGCCT	pykF	Pyruvate kinase	WP_036903087.	585216..586970	Glycolysis	COG0469
5.1_WP_036903087.1_	CUST_8537_PI442182253	MIT0601	LLII/III	5.1_WP_036903087.1_	AGGATTGAGAAAGATTATCCCCAAAGAGCCCTAGAGAGTCATCTTCCTAGTACCATTCCT	pykF	Pyruvate kinase	WP_036903087.	585216..586970	Glycolysis	COG0469
5.1_WP_036903087.1_	CUST_8538_PI442182253	MIT0601	LLII/III	5.1_WP_036903087.1_	AATGAAACTGCTGTTGGAGACTATCCAATTGAAGCAGTGAAAACGATGGCAACTATTGCT	pykF	Pyruvate kinase	WP_036903087.	585216..586970	Glycolysis	COG0469
5.1_WP_052043120.1_	CUST_9162_PI442182253	MIT0601	LLII/III	5.1_WP_052043120.1_	TCAGATCTCTAACAATATGGGGATGTTGCGTTACACAAGCGGAGGTAATTACACAATCCC	pdhB	Pyruvate dehydrogenase E1 component beta subunit	WP_052043120.	691302..692234	Glycolysis/Krebs cycle	COG0022
5.1_WP_052043120.1_	CUST_9163_PI442182253	MIT0601	LLII/III	5.1_WP_052043120.1_	ATGTGCTTCTTTATAATTTGAGCGAGGAAATCCCTGGAGGGGATTATGTTTGTGCTTTGG	pdhB	Pyruvate dehydrogenase E1 component beta subunit	WP_052043120.	691302..692234	Glycolysis/Krebs cycle	COG0022
5.1_WP_052043120.1_	CUST_9164_PI442182253	MIT0601	LLII/III	5.1_WP_052043120.1_	ACGAATGAGACACCATTGCTTAAAAGCTCTGCCTCAATTAGCAGAAAAAGGTATCGATGC	pdhB	Pyruvate dehydrogenase E1 component beta subunit	WP_052043120.	691302..692234	Glycolysis/Krebs cycle	COG0022
5.1_WP_052043120.1_	CUST_9165_PI442182253	MIT0601	LLII/III	5.1_WP_052043120.1_	GAGGTGTTGGGAGGCAATTAGGCGCTGAACATAGTCAAAGACTTGAAGCTTATTTTCATG	pdhB	Pyruvate dehydrogenase E1 component beta subunit	WP_052043120.	691302..692234	Glycolysis/Krebs cycle	COG0022
5.1_WP_052043120.1_	CUST_9166_PI442182253	MIT0601	LLII/III	5.1_WP_052043120.1_	CCCCTACCAATGCTAAAGGTCTAATGAAAGCCGCCATTAGAGATAACAATCCCGTCTTAT	pdhB	Pyruvate dehydrogenase E1 component beta subunit	WP_052043120.	691302..692234	Glycolysis/Krebs cycle	COG0022
5.1_WP_052043120.1_	CUST_9167_PI442182253	MIT0601	LLII/III	5.1_WP_052043120.1_	AAAACTGTTTTGATGATTTGGATGCTCGTCCTATTAGGTTGTCGAGTCAGGACATCCCAA	pdhB	Pyruvate dehydrogenase E1 component beta subunit	WP_052043120.	691302..692234	Glycolysis/Krebs cycle	COG0022
1.1_WP_036918725.1_	CUST_136_PI442182273	MIT0602	LLII/III	1.1_WP_036918725.1_	ACTGGTCGAAGAGAAGGAGATGCCTTTCGAGTAATAGCAGATAATTCATTGGCAACATGT	galE	UDP-glucose 4-epimerase	WP_036918725.	24092..25156	Galactose metabolism	COG1087
1.1_WP_036918725.1_	CUST_137_PI442182273	MIT0602	LLII/III	1.1_WP_036918725.1_	CCTGGGAAATGGCGAATCGCAAACTTACGTTATTTCAATCCAATAGGTGCTCATACTTCA	galE	UDP-glucose 4-epimerase	WP_036918725.	24092..25156	Galactose metabolism	COG1087
1.1_WP_036918725.1_	CUST_138_PI442182273	MIT0602	LLII/III	1.1_WP_036918725.1_	TTTTGCTGGGTTGAAATCAGTTAGAGAATCCGTTGGAAACCCTTTAAAGTATTGGGACGT	galE	UDP-glucose 4-epimerase	WP_036918725.	24092..25156	Galactose metabolism	COG1087
1.1_WP_036918725.1_	CUST_139_PI442182273	MIT0602	LLII/III	1.1_WP_036918725.1_	TGGCAATGATTGGCCAACTCCAGATGGAACTGGAGTAAGAGACTATATACATGTAATGGA	galE	UDP-glucose 4-epimerase	WP_036918725.	24092..25156	Galactose metabolism	COG1087
1.1_WP_036918723.1_	CUST_143_PI442182273	MIT0602	LLII/III	1.1_WP_036918723.1_	TCAGCTCTTTGTGAAGCTACAGGGGCAGATATTAGAGATGTTTCTCTAGCAATAGGAGCT	ugd	UDP-glucose 6-dehydrogenase/nucleotide sugar dehydrogenase	WP_036918723.	25294..26715	Amino sugar and nucleotide sugar r	COG1004
1.1_WP_036918725.1_	CUST_140_PI442182273	MIT0602	LLII/III	1.1_WP_036918725.1_	TTGGACCCCAAAAAGAAGTCTAGAAGATATGTGCTATGACGGTTATAAATGGCAATCTTT	galE	UDP-glucose 4-epimerase	WP_036918725.	24092..25156	Galactose metabolism	COG1087
1.1_WP_036918725.1_	CUST_141_PI442182273	MIT0602	LLII/III	1.1_WP_036918725.1_	TGTTGCAGATGGTCATGAAAAGGCTTTCGAATACTTATTAAGTAAAGAACCACAAGTTCT	galE	UDP-glucose 4-epimerase	WP_036918725.	24092..25156	Galactose metabolism	COG1087
1.1_WP_036918723.1_	CUST_142_PI442182273	MIT0602	LLII/III	1.1_WP_036918723.1_	AGACTCAAGCACTAATCAGGAAGGATCTTGGTCATTTTGCGAATCAATTGTTGAAGCAGC	ugd	UDP-glucose 6-dehydrogenase/nucleotide sugar dehydrogenase	WP_036918723.	25294..26715	Amino sugar and nucleotide sugar r	COG1004
1.1_WP_036918723.1_	CUST_144_PI442182273	MIT0602	LLII/III	1.1_WP_036918723.1_	TTTCTTGAATTCAGGGCCTGGTTTCGGAGGAAGTTGTTTCAAAAAGGATCTCCTGAATTT	ugd	UDP-glucose 6-dehydrogenase/nucleotide sugar dehydrogenase	WP_036918723.	25294..26715	Amino sugar and nucleotide sugar r	COG1004
1.1_WP_036918723.1_	CUST_145_PI442182273	MIT0602	LLII/III	1.1_WP_036918723.1_	ACTAACCTTTGGAGCTCTGAACTGTCAAAGCTAATCTCAAATGCTTTTCTTGCCCAAAGA	ugd	UDP-glucose 6-dehydrogenase/nucleotide sugar dehydrogenase	WP_036918723.	25294..26715	Amino sugar and nucleotide sugar r	COG1004
1.1_WP_036918723.1_	CUST_146_PI442182273	MIT0602	LLII/III	1.1_WP_036918723.1_	AGAGAACCAGCATGGTTGTTTGATACAAGGTCCATTGCAAATACAAAGCAAGCAAAAGAA	ugd	UDP-glucose 6-dehydrogenase/nucleotide sugar dehydrogenase	WP_036918723.	25294..26715	Amino sugar and nucleotide sugar r	COG1004
1.1_WP_036918723.1_	CUST_147_PI442182273	MIT0602	LLII/III	1.1_WP_036918723.1_	TGACGCTGATGCAATTGTAATTTTAACTGAATGGCGTGAATTTAAACAAATAAATTGGGA	ugd	UDP-glucose 6-dehydrogenase/nucleotide sugar dehydrogenase	WP_036918723.	25294..26715	Amino sugar and nucleotide sugar r	COG1004
1.1_WP_036918716.1_	CUST_178_PI442182273	MIT0602	LLII/III	1.1_WP_036918716.1_	AGGAGCTGGGGGCAAGGCGCACAACATTCTCAATCATTTCATAGTTTTTATCATCAATA	pdhB	Pyruvate dehydrogenase E1 component beta subunit	WP_036918716.	31812..32843	Glycolysis/Krebs cycle	COG0022
1.1_WP_036918716.1_	CUST_179_PI442182273	MIT0602	LLII/III	1.1_WP_036918716.1_	TGGATTTATGGGAAAGTCTAAAATAATGGGCTATTCAGATAGCCCATGCCCAACTCCAAA	pdhB	Pyruvate dehydrogenase E1 component beta subunit	WP_036918716.	31812..32843	Glycolysis/Krebs cycle	COG0022
1.1_WP_036918716.1_	CUST_180_PI442182273	MIT0602	LLII/III	1.1_WP_036918716.1_	TGGACTGACTGTAGTTGCACCTGTTACACCTCACGACATGTTTAATACTATATTATGGGC	pdhB	Pyruvate dehydrogenase E1 component beta subunit	WP_036918716.	31812..32843	Glycolysis/Krebs cycle	COG0022
1.1_WP_036918716.1_	CUST_181_PI442182273	MIT0602	LLII/III	1.1_WP_036918716.1_	GCTGCGAAAGTAAAATATTTATCAAATGGCAAGGATTCCTGCCCATTAATAGTTAGGGCA	pdhB	Pyruvate dehydrogenase E1 component beta subunit	WP_036918716.	31812..32843	Glycolysis/Krebs cycle	COG0022
1.1_WP_036918716.1_	CUST_182_PI442182273	MIT0602	LLII/III	1.1_WP_036918716.1_	AGAAGAAGGTTATGAGCCTAGTATCAGGAAGCTAGAATCTGGCAATGATATTACAGTTGT	pdhB	Pyruvate dehydrogenase E1 component beta subunit	WP_036918716.	31812..32843	Glycolysis/Krebs cycle	COG0022
1.1_WP_036918716.1_	CUST_183_PI442182273	MIT0602	LLII/III	1.1_WP_036918716.1_	TGTCTCTTGAGGCAAGTAGAGCTATTGAGGAATTAAGTTCTAAAATGAATATAACCTCAG	pdhB	Pyruvate dehydrogenase E1 component beta subunit	WP_036918716.	31812..32843	Glycolysis/Krebs cycle	COG0022
1.1_WP_036918589.1_	CUST_568_PI442182273	MIT0602	LLII/III	1.1_WP_036918589.1_	GGCAATAGGTATGGCACATGATCGTGCTACTAGTGAACCTGAAATATGGAGAAAAGCTGA	pdhA	Pyruvate dehydrogenase E1 component alpha subunit	WP_036918589.	103613..104686	Glycolysis/Krebs cycle	COG1071
1.1_WP_036918589.1_	CUST_567_PI442182273	MIT0602	LLII/III	1.1_WP_036918589.1_	TTAACCTATAGATTTAGAGGACACTCTCTTGCAGACCCTGACGAATTGCGCTCTTCTGAA	pdhA	Pyruvate dehydrogenase E1 component alpha subunit	WP_036918589.	103613..104686	Glycolysis/Krebs cycle	COG1071
1.1_WP_036918589.1_	CUST_569_PI442182273	MIT0602	LLII/III	1.1_WP_036918589.1_	AAACAGGATGTAGCAAAGGGAGAGGTGGATCAATGCATCTTTTTTCCAAAGAACATCACC	pdhA	Pyruvate dehydrogenase E1 component alpha subunit	WP_036918589.	103613..104686	Glycolysis/Krebs cycle	COG1071
1.1_WP_036918589.1_	CUST_570_PI442182273	MIT0602	LLII/III	1.1_WP_036918589.1_	GGAAAGTGATGCAGTTACAGCTGCTTTTTTGGAGATGGCACTTGCAATATTGGTCAATT	pdhA	Pyruvate dehydrogenase E1 component alpha subunit	WP_036918589.	103613..104686	Glycolysis/Krebs cycle	COG1071
1.1_WP_036918589.1_	CUST_571_PI442182273	MIT0602	LLII/III	1.1_WP_036918589.1_	TGCCTTTATAGGAGAAGGTATTCCTGTGGCACTAGGGTCAGCTTTTACTAGTCGTTACAA	pdhA	Pyruvate dehydrogenase E1 component alpha subunit	WP_036918589.	103613..104686	Glycolysis/Krebs cycle	COG1071
1.1_WP_036918589.1_	CUST_572_PI442182273	MIT0602	LLII/III	1.1_WP_036918589.1_	CTTGGTGCAGCAGATCCTGACCCTGCTGAACTAACAAAATATATTTGGGCGGAAAATTAA	pdhA	Pyruvate dehydrogenase E1 component alpha subunit	WP_036918589.	103613..104686	Glycolysis/Krebs cycle	COG1071
1.1_WP_036918556.1_	CUST_691_PI442182273	MIT0602	LLII/III	1.1_WP_036918556.1_	TCAAGCGTATAGGAACCAAAAAGCAGGAGTCAAATTATCACCTCAATCGCTATATCGGCA	ftsZ	cell division protein FtsZ	WP_036918556.	126726..127847	Cell Division	COG0206
1.1_WP_036918556.1_	CUST_692_PI442182273	MIT0602	LLII/III	1.1_WP_036918556.1_	GGCGCCGTTATTGATGAAGCTCTAGAAGGTGAAGTTCAAGTAACTGTTATAGCAACTGGA	ftsZ	cell division protein FtsZ	WP_036918556.	126726..127847	Cell Division	COG0206
1.1_WP_036918556.1_	CUST_693_PI442182273	MIT0602	LLII/III	1.1_WP_036918556.1_	AAATGCAGATGATGTCTTGCGAATGGGGGTAAAAGGCATAACAGATATAATCACATGCCC	ftsZ	cell division protein FtsZ	WP_036918556.	126726..127847	Cell Division	COG0206
1.1_WP_036918556.1_	CUST_694_PI442182273	MIT0602	LLII/III	1.1_WP_036918556.1_	AGATAAATAAGGAGCCAGGAGCAAGCATCCCTGAATTTTTACGACTCAGACAGCTTAGAC	ftsZ	cell division protein FtsZ	WP_036918556.	126726..127847	Cell Division	COG0206
1.1_WP_036918556.1_	CUST_695_PI442182273	MIT0602	LLII/III	1.1_WP_036918556.1_	CGGAGCCCTCACAGTTGCAATAGTTACTAAGCCTTTTGGATTTGAAGGTCGTAGAAGAAT	ftsZ	cell division protein FtsZ	WP_036918556.	126726..127847	Cell Division	COG0206
1.1_WP_036918556.1_	CUST_696_PI442182273	MIT0602	LLII/III	1.1_WP_036918556.1_	TAGATACTTTGATTGTCATACCTAATGATCGACTGAAAGACGCAATTTCAGGCGCCCCAC	ftsZ	cell division protein FtsZ	WP_036918556.	126726..127847	Cell Division	COG0206
1.1_WP_036918523.1_	CUST_790_PI442182273	MIT0602	LLII/III	1.1_WP_036918523.1_	CTTGCAACTTGTGCGGAGCAATCAGCTAGTGCAATAACTACTATCCGTATATGTATGGGA	glcH	GPH family sugar transporter	WP_036918523.	143658..145013	GlcH sugar transporter	COG2211
1.1_WP_036918523.1_	CUST_791_PI442182273	MIT0602	LLII/III	1.1_WP_036918523.1_	GATGCTATTGATGCCGATCCAGATAAACCTGCCGGTATTATACAGCATGGATGGTTTTG	glcH	GPH family sugar transporter	WP_036918523.	143658..145013	GlcH sugar transporter	COG2211
1.1_WP_036918523.1_	CUST_792_PI442182273	MIT0602	LLII/III	1.1_WP_036918523.1_	GTACCTTCAGTAACTGCAAAATGGATGCCCATACCCTTCCAAATTAGTGCACTTTTTGGA	glcH	GPH family sugar transporter	WP_036918523.	143658..145013	GlcH sugar transporter	COG2211
1.1_WP_036918523.1_	CUST_793_PI442182273	MIT0602	LLII/III	1.1_WP_036918523.1_	AAGCGCTTCCTTAAGATCATTTGTTTATATCTCCTCCTCTGGTGCGGTCTTCAGCTTATG	glcH	GPH family sugar transporter	WP_036918523.	143658..145013	GlcH sugar transporter	COG2211
1.1_WP_036918523.1_	CUST_794_PI442182273	MIT0602	LLII/III	1.1_WP_036918523.1_	GCCCCCTTTGCGAAAAAAGCTAGAAAACCAATTCCTACATCTGAGCCTTTCAAATTTCA	glcH	GPH family sugar transporter	WP_036918523.	143658..145013	GlcH sugar transporter	COG2211
1.1_WP_036918523.1_	CUST_795_PI442182273	MIT0602	LLII/III	1.1_WP_036918523.1_	TGGGAAGAATTACAGGCTTAATAGCAACTTTGACGACTTTGATTTCGTGTTGGGGATTGG	glcH	GPH family sugar transporter	WP_036918523.	143658..145013	GlcH sugar transporter	COG2211
1.1_WP_036918487.1_	CUST_904_PI442182273	MIT0602	LLII/III	1.1_WP_036918487.1_	AGCTCAACTCCTTAGGAATGCTACAAGTTGGGGTATTGATTTTGAGAAGATGGAAAGCGA	kaiC	circadian clock protein KaiC	WP_036918487.	163632..165176	Circadian Rhythm	COG0467
1.1_WP_036918487.1_	CUST_908_PI442182273	MIT0602	LLII/III	1.1_WP_036918487.1_	TTGAACAAATATTTAGTGGCGCACCGCATCGTATAAGTAGCGATGAAAATCTTCCTGGTG	kaiC	circadian clock protein KaiC	WP_036918487.	163632..165176	Circadian Rhythm	COG0467
1.1_WP_036918487.1_	CUST_905_PI442182273	MIT0602	LLII/III	1.1_WP_036918487.1_	ACTCAGTAATACTTGCTACTGGGGCAACAGGGACTGGTAAGACTATGCTTGTCTCTAAAT	kaiC	circadian clock protein KaiC	WP_036918487.	163632..165176	Circadian Rhythm	COG0467
1.1_WP_036918487.1_	CUST_906_PI442182273	MIT0602	LLII/III	1.1_WP_036918487.1_	TAAACAGGAAGAAATAGCGGGGTTTTTTACTAACACTGCTGAAGAGTTTATGGGGAGCCA	kaiC	circadian clock protein KaiC	WP_036918487.	163632..165176	Circadian Rhythm	COG0467
1.1_WP_036918487.1_	CUST_907_PI442182273	MIT0602	LLII/III	1.1_WP_036918487.1_	ATACAAGCCCTCTAGAATGGCAATAGATTCTCTTTCAGCTTTAGCTCGAGGAGTAAGCCT	kaiC	circadian clock protein KaiC	WP_036918487.	163632..165176	Circadian Rhythm	COG0467
1.1_WP_036918487.1_	CUST_909_PI442182273	MIT0602	LLII/III	1.1_WP_036918487.1_	TTACAGACACAATCTTATTGCTTCAATATGTAGAGATTGGAGGAGAGATGGCCCGTGCAG	kaiC	circadian clock protein KaiC	WP_036918487.	163632..165176	Circadian Rhythm	COG0467
1.1_WP_036918486.1_	CUST_910_PI442182273	MIT0602	LLII/III	1.1_WP_036918486.1_	AGAACTAAATGAAAAGTCATTTTTTTCTTCAACTGATAATGAGCACGAGATAGAGAGCAA	kaiB	Circadian clock protein KaiB	WP_036918486.	165243..165590	Circadian Rhythm	COG4251
1.1_WP_036918486.1_	CUST_911_PI442182273	MIT0602	LLII/III	1.1_WP_036918486.1_	TCCTCCCTCCCCCTGTAAGAAGAATTATCGGAGATCTTTCCGATCGAGAAAAGGTTTTAA	kaiB	Circadian clock protein KaiB	WP_036918486.	165243..165590	Circadian Rhythm	COG4251
1.1_WP_036918486.1_	CUST_912_PI442182273	MIT0602	LLII/III	1.1_WP_036918486.1_	AGTAATTGATGTACTTGAGAAGCCTCAACTGGCGGAGGAAGACAAAATACTTGCTACACC	kaiB	Circadian clock protein KaiB	WP_036918486.	165243..165590	Circadian Rhythm	COG4251
1.1_WP_036918486.1_	CUST_913_PI442182273	MIT0602	LLII/III	1.1_WP_036918486.1_	CGCCTTAAACACTCTTCGAGAGATTCTTAAAACAGATTTCAAGGGG	kaiB	Circadian clock protein KaiB	WP_036918486.	165243..165590	Circadian Rhythm	COG4251
1.1_WP_036918486.1_	CUST_914_PI442182273	MIT0602	LLII/III	1.1_WP_036918486.1_	ACATCCTTAAATTGTATGTCGCTGGAAACACTCCAAATTCTATGCGCGCCTTAAACACTC	kaiB	Circadian clock protein KaiB	WP_036918486.	165243..165590	Circadian Rhythm	COG4251
2.1_WP_036917127.1_	CUST_1181_PI442182273	MIT0602	LLII/III	2.1_WP_036917127.1_	ATGTTCGAATGGTTGGCTATGACGCTTATACCCAAAGTCAGGGTACAGCTTTTGTAGTTT	rbcS	Ribulose bisphosphate carboxylase small subunit	WP_036917127.	17549..17890	Carbon fixation	COG4451
2.1_WP_036917127.1_	CUST_1182_PI442182273	MIT0602	LLII/III	2.1_WP_036917127.1_	AAAAAGATTTGAATGTCGTTGTTAGCGAGTTAGAAGCTTGCCACCGTGCATATCCAGATC	rbcS	Ribulose bisphosphate carboxylase small subunit	WP_036917127.	17549..17890	Carbon fixation	COG4451
2.1_WP_036917127.1_	CUST_1183_PI442182273	MIT0602	LLII/III	2.1_WP_036917127.1_	TTCACCCCAGTGGTTCCATGCAGACTTATTGGTCTTATTGGAAGTTACCTTTCTTTGGTG	rbcS	Ribulose bisphosphate carboxylase small subunit	WP_036917127.	17549..17890	Carbon fixation	COG4451
2.1_WP_036917127.1_	CUST_1184_PI442182273	MIT0602	LLII/III	2.1_WP_036917127.1_	TTCTTGCCTCCAATGTCACAGGACGAAATTTACGACCAAATTGCTTACATCATTGCTCAG	rbcS	Ribulose bisphosphate carboxylase small subunit	WP_036917127.	17549..17890	Carbon fixation	COG4451
2.1_WP_036917127.1_	CUST_1185_PI442182273	MIT0602	LLII/III	2.1_WP_036917127.1_	ATGCCTTTTCAGAGCACAGTTGGTGACTATCAAACAGTCGCCACCCTGGAGACATTC	rbcS	Ribulose bisphosphate carboxylase small subunit	WP_036917127.	17549..17890	Carbon fixation	COG4451
2.1_WP_011124704.1_	CUST_1186_PI442182273	MIT0602	LLII/III	2.1_WP_011124704.1_	ATAGTCCTGAGTTAGCTATTGCACTTGAGACCTGGAAGGAGATCAAGTTCGAGTTTGACA	rbcL	Ribulose 1,5-bisphosphate carboxylase large subunit	WP_011124704.	17995..19407	Carbon fixation	COG1850
2.1_WP_011124704.1_	CUST_1187_PI442182273	MIT0602	LLII/III	2.1_WP_011124704.1_	AGCTTTAGAAGCATGTGTTAAGGCACGTAATGCAGGTAGAGAAATCGAGAAGGAAAGTCG	rbcL	Ribulose 1,5-bisphosphate carboxylase large subunit	WP_011124704.	17995..19407	Carbon fixation	COG1850
2.1_WP_011124704.1_	CUST_1188_PI442182273	MIT0602	LLII/III	2.1_WP_011124704.1_	GACCGTCAAACAACTCTTGGGTATATCGACAATTTACGTGAATCCTTCGTCCCAGAAGAC	rbcL	Ribulose 1,5-bisphosphate carboxylase large subunit	WP_011124704.	17995..19407	Carbon fixation	COG1850
2.1_WP_011124704.1_	CUST_1189_PI442182273	MIT0602	LLII/III	2.1_WP_011124704.1_	GTTTACGTCTGTCAGGTGGTGATCAACTTCACACTGGAACAGTTGTAGGTAAGTTGGAAG	rbcL	Ribulose 1,5-bisphosphate carboxylase large subunit	WP_011124704.	17995..19407	Carbon fixation	COG1850
2.1_WP_011124704.1_	CUST_1190_PI442182273	MIT0602	LLII/III	2.1_WP_011124704.1_	GTGGCTTTACTGCTAACACAGGATTAGCTAATTGGTGTCGTAAGAATGGCATGTTGCTTC	rbcL	Ribulose 1,5-bisphosphate carboxylase large subunit	WP_011124704.	17995..19407	Carbon fixation	COG1850
2.1_WP_011124704.1_	CUST_1191_PI442182273	MIT0602	LLII/III	2.1_WP_011124704.1_	GATGAGAACATAAACTCACAGCCCTTCCAGAGATGGAGAGATCGTTTTGAGTTTGTTGCT	rbcL	Ribulose 1,5-bisphosphate carboxylase large subunit	WP_011124704.	17995..19407	Carbon fixation	COG1850
2.1_WP_036917068.1_	CUST_1357_PI442182273	MIT0602	LLII/III	2.1_WP_036917068.1_	TTGTTGGACGTATCCTTGATTGGTATAAAGCAAAAACTGGCAAAGAATCTTACCCAGGGC	tal	Transaldolase	WP_036917068.	46488..47495	Pentose Phosphate	COG0176
2.1_WP_036917068.1_	CUST_1358_PI442182273	MIT0602	LLII/III	2.1_WP_036917068.1_	AAAAGAAGGCATTCATTGTAACTTAACCTTGTTATTTGGCTTTGCTCAAGCCGTTGCCTG	tal	Transaldolase	WP_036917068.	46488..47495	Pentose Phosphate	COG0176
2.1_WP_036917068.1_	CUST_1359_PI442182273	MIT0602	LLII/III	2.1_WP_036917068.1_	CAGTCAGACATAATGGCTGATGAGAAGCTTGAAGAGGGTATTAGAAGTTTCAGCAAGGCT	tal	Transaldolase	WP_036917068.	46488..47495	Pentose Phosphate	COG0176
2.1_WP_036917068.1_	CUST_1360_PI442182273	MIT0602	LLII/III	2.1_WP_036917068.1_	ATGAGGCTGGCATTAGTAATGAAAGAGTCTTAATAAAAGTCGCCTCAACATGGGAAGGAA	tal	Transaldolase	WP_036917068.	46488..47495	Pentose Phosphate	COG0176
2.1_WP_036917068.1_	CUST_1361_PI442182273	MIT0602	LLII/III	2.1_WP_036917068.1_	CGATGAGATTATTGAGTTAGCAGGATGTGATTTGCTTACTATTTCCCCTAAGTTACTGGA	tal	Transaldolase	WP_036917068.	46488..47495	Pentose Phosphate	COG0176
2.1_WP_036917068.1_	CUST_1362_PI442182273	MIT0602	LLII/III	2.1_WP_036917068.1_	ACTCTACAAATCCATCATCCTCAGAAAGCAAGCTTCATTTAAATGAAGGTCAGTTAAGA	tal	Transaldolase	WP_036917068.	46488..47495	Pentose Phosphate	COG0176
2.1_WP_036916967.1_	CUST_1712_PI442182273	MIT0602	LLII/III	2.1_WP_036916967.1_	TGGGGAAGCATATTCATTGCAAGCAAATGTGGCAAATGAAGATGCTGTTAATGAATTGGT	gdh	3-oxoacyl-acyl-carrier-protein reductase	WP_036916967.	106869..107621	Entner-Dudoroff	COG1028
2.1_WP_036916967.1_	CUST_1713_PI442182273	MIT0602	LLII/III	2.1_WP_036916967.1_	AGTTTGCAAGCAGAGGAATAACTGTAAATGCAGTCGCTCCTGGGTTTATTGAGACAGATA	gdh	3-oxoacyl-acyl-carrier-protein reductase	WP_036916967.	106869..107621	Entner-Dudoroff	COG1028
2.1_WP_036916967.1_	CUST_1714_PI442182273	MIT0602	LLII/III	2.1_WP_036916967.1_	TTTTCTAGCAGCAGATCCTGCAGCGAGTTATATAACAGGGCAGGTATTACAAGTTGATGG	gdh	3-oxoacyl-acyl-carrier-protein reductase	WP_036916967.	106869..107621	Entner-Dudoroff	COG1028
2.1_WP_036916967.1_	CUST_1715_PI442182273	MIT0602	LLII/III	2.1_WP_036916967.1_	TAGGAGCTAAAGTCGTAGTGAACTATTCAAGCTCGCCAGAGAAAGCTGAAGAAGTAGTGG	gdh	3-oxoacyl-acyl-carrier-protein reductase	WP_036916967.	106869..107621	Entner-Dudoroff	COG1028
2.1_WP_036916967.1_	CUST_1716_PI442182273	MIT0602	LLII/III	2.1_WP_036916967.1_	ACTTAGACGCCGAAAAAATACTTTCAGCAATTCCCTTAGGAAAATTTGGGACTCCTGAGC	gdh	3-oxoacyl-acyl-carrier-protein reductase	WP_036916967.	106869..107621	Entner-Dudoroff	COG1028
2.1_WP_036916967.1_	CUST_1717_PI442182273	MIT0602	LLII/III	2.1_WP_036916967.1_	GGAGTATTTTTATGCACCAAAGCAGTTTCACGATCAATGCTGAAAGCCAAGAAAGGAGA	gdh	3-oxoacyl-acyl-carrier-protein reductase	WP_036916967.	106869..107621	Entner-Dudoroff	COG1028
2.1_WP_036918124.1_	CUST_1753_PI442182273	MIT0602	LLII/III	2.1_WP_036918124.1_	GTTGAGTTAACTGCGATGCAATTTGCTTTTCTCTTCAATTACCCAGAGGGGGGGATTATT	ctaC/coxB	Cytochrome c oxidase subunit II	WP_036918124.	114777..115595	Respiration	COG1622
2.1_WP_036918124.1_	CUST_1754_PI442182273	MIT0602	LLII/III	2.1_WP_036918124.1_	AAGATGTAATTCACGCTTTTTGGGTTCCGGAGTTTCGCTTAAAGCAAGATGTCATACCTG	ctaC/coxB	Cytochrome c oxidase subunit II	WP_036918124.	114777..115595	Respiration	COG1622
2.1_WP_036918124.1_	CUST_1758_PI442182273	MIT0602	LLII/III	2.1_WP_036918124.1_	AGTAGTTTTATTTGTCGGTCTTTACAGCTATGACATCTATGAAAGGATGGGGGGCATGCA	ctaC/coxB	Cytochrome c oxidase subunit II	WP_036918124.	114777..115595	Respiration	COG1622
2.1_WP_036918124.1_	CUST_1755_PI442182273	MIT0602	LLII/III	2.1_WP_036918124.1_	CGTTCAACCGTTGTTGTGGAAGAGCCAGAAGAATATCAAGCTTGGTTCAAAGAAAACTCA	ctaC/coxB	Cytochrome c oxidase subunit II	WP_036918124.	114777..115595	Respiration	COG1622
2.1_WP_036918124.1_	CUST_1756_PI442182273	MIT0602	LLII/III	2.1_WP_036918124.1_	TCGTATATAGCTTGATTCAGTTTAGGCGAAAGCCAGGGGAAACAGAAGATGGTCTACCTA	ctaC/coxB	Cytochrome c oxidase subunit II	WP_036918124.	114777..115595	Respiration	COG1622
2.1_WP_036918124.1_	CUST_1757_PI442182273	MIT0602	LLII/III	2.1_WP_036918124.1_	GTCAACCAACAGTCCTAAATTTCACGCCAACGAAACCTGGAAGGTATCCAATAATATGCG	ctaC/coxB	Cytochrome c oxidase subunit II	WP_036918124.	114777..115595	Respiration	COG1622
2.1_WP_036916951.1_	CUST_1759_PI442182273	MIT0602	LLII/III	2.1_WP_036916951.1_	CTATTCGATCATGGGGATTGTAATTTTAGGGCTTGTCGTTTGGGCTCACCACATGTTTAC	cyoB/coxA	Cytochrome c oxidase subunit I	WP_036916951.	115592..117238	Respiration	COG0843
2.1_WP_036916951.1_	CUST_1760_PI442182273	MIT0602	LLII/III	2.1_WP_036916951.1_	CAAGTGCCATTTGACGTACATGTTCACGATACCTACTTTGTCGTCGCCCATTTTCATTAC	cyoB/coxA	Cytochrome c oxidase subunit I	WP_036916951.	115592..117238	Respiration	COG0843
2.1_WP_036916951.1_	CUST_1761_PI442182273	MIT0602	LLII/III	2.1_WP_036916951.1_	CAGACGTGTAGCAGAGTATGACCCACAGTTTGCTTTTGTAAATCAAATCAGCAGTGTTGG	cyoB/coxA	Cytochrome c oxidase subunit I	WP_036916951.	115592..117238	Respiration	COG0843
2.1_WP_036916951.1_	CUST_1762_PI442182273	MIT0602	LLII/III	2.1_WP_036916951.1_	TTTTCAATTGGGTAGCTACTCTCTGGGGGGGACGTATTTCATTAAACAGTGCAATTCTCT	cyoB/coxA	Cytochrome c oxidase subunit I	WP_036916951.	115592..117238	Respiration	COG0843
2.1_WP_036916951.1_	CUST_1763_PI442182273	MIT0602	LLII/III	2.1_WP_036916951.1_	CATTAACTCCAGAATGGTTAACTAGTTCACCTCCTCCAGTTGAAAACTGGATCGGCGAAG	cyoB/coxA	Cytochrome c oxidase subunit I	WP_036916951.	115592..117238	Respiration	COG0843
2.1_WP_036916951.1_	CUST_1764_PI442182273	MIT0602	LLII/III	2.1_WP_036916951.1_	ATTACATTTATTGGGTTTAATCTCTGCTTCGCTCCTCAGCATTGGCTCGGTTTAAATGGC	cyoB/coxA	Cytochrome c oxidase subunit I	WP_036916951.	115592..117238	Respiration	COG0843
2.1_WP_036916949.1_	CUST_1765_PI442182273	MIT0602	LLII/III	2.1_WP_036916949.1_	AACTAACAAGGAATCAGAAAAATCAGCCCTACAAGGTCAAGAGCATGAACACTCCGATCA	cyoC	heme-copper oxidase subunit III	WP_036916949.	117244..117852	Respiration	COG1845
2.1_WP_036916949.1_	CUST_1766_PI442182273	MIT0602	LLII/III	2.1_WP_036916949.1_	CTTGGGCTTGCTTTTCTAGCAAGTCAAATGGTGGAATACTTCACTCTTCCATTTGGACTC	cyoC	heme-copper oxidase subunit III	WP_036916949.	117244..117852	Respiration	COG1845
2.1_WP_036916949.1_	CUST_1767_PI442182273	MIT0602	LLII/III	2.1_WP_036916949.1_	ATGGGATGACATTTGCTGGCTTCTTTGCAGCTTATTTAACTTACAAGTCAGTTAACCCCC	cyoC	heme-copper oxidase subunit III	WP_036916949.	117244..117852	Respiration	COG1845
2.1_WP_036916949.1_	CUST_1768_PI442182273	MIT0602	LLII/III	2.1_WP_036916949.1_	TGCCGAGCTGTATTGGCACTTCGTTGATGGAATATGGGTCGTTTTATTCATAATCCTGTA	cyoC	heme-copper oxidase subunit III	WP_036916949.	117244..117852	Respiration	COG1845
2.1_WP_036916949.1_	CUST_1769_PI442182273	MIT0602	LLII/III	2.1_WP_036916949.1_	ATGTAACTCTAGGAACAATAATGATTTTAATTGTCTGGTGGCAAGCTCGCTCTCCGGCTG	cyoC	heme-copper oxidase subunit III	WP_036916949.	117244..117852	Respiration	COG1845
2.1_WP_036916949.1_	CUST_1770_PI442182273	MIT0602	LLII/III	2.1_WP_036916949.1_	AAAGCATTGGAAAGTAAAAATGCAAACAAATGTCAATTTTGGCTTCTTGTTACGGCAGCC	cyoC	heme-copper oxidase subunit III	WP_036916949.	117244..117852	Respiration	COG1845
2.1_WP_052043786.1_	CUST_2159_PI442182273	MIT0602	LLII/III	2.1_WP_052043786.1_	GAGAAGCCTGAAGAGGGTTAGCTGAAGAAGCACGCTTAGAGGATGATGTAATTGTCATT	minC	septum site-determining protein MinC	WP_052043786.	191335..192024	Cell Division	COG0850
2.1_WP_052043786.1_	CUST_2160_PI442182273	MIT0602	LLII/III	2.1_WP_052043786.1_	TTGGAGATGTAAATCCTGGTGCTTCTGTATTAGCTGGTGGGAATGTAATGATTTGGGGAA	minC	septum site-determining protein MinC	WP_052043786.	191335..192024	Cell Division	COG0850
2.1_WP_052043786.1_	CUST_2161_PI442182273	MIT0602	LLII/III	2.1_WP_052043786.1_	TAAAGGCCAAAATAACCGCCCTTCAATTAAGGCCAGTTCAATTGAGAATCGCAAACAAAG	minC	septum site-determining protein MinC	WP_052043786.	191335..192024	Cell Division	COG0850
2.1_WP_052043786.1_	CUST_2162_PI442182273	MIT0602	LLII/III	2.1_WP_052043786.1_	TTTTTACATCAAGGTACTCTTAGGTCCGGTGAAGTCTTAGCCGTAAATAATGATGTGCTT	minC	septum site-determining protein MinC	WP_052043786.	191335..192024	Cell Division	COG0850
2.1_WP_036916612.1_	CUST_2166_PI442182273	MIT0602	LLII/III	2.1_WP_036916612.1_	TTTGAATACGTGTTAATTGATTGCCCTGCAGGAGTCGAAGATGGTTTCAAGAATGCAGTC	minD	septum site-determining protein MinD	WP_036916612.	192165..192980	Cell Division	COG2894
2.1_WP_052043786.1_	CUST_2163_PI442182273	MIT0602	LLII/III	2.1_WP_052043786.1_	AGTGCTTCTGGCTTAGGATTTAAGACATTGCTTAACCCTAAAGAGAGCACATTAAAACAA	minC	septum site-determining protein MinC	WP_052043786.	191335..192024	Cell Division	COG0850
2.1_WP_052043786.1_	CUST_2164_PI442182273	MIT0602	LLII/III	2.1_WP_052043786.1_	AAGAGATAAAGTTATTTTCAGCTCTGTTCTTAAGAAGAACTGGCGTGATAAGATTGAGTT	minC	septum site-determining protein MinC	WP_052043786.	191335..192024	Cell Division	COG0850
2.1_WP_036916612.1_	CUST_2165_PI442182273	MIT0602	LLII/III	2.1_WP_036916612.1_	AAGCATTAGTTAAACATAAACAGGAACCAAACCTCTCTTTGCTTCCTGCTGGCAATCCAA	minD	septum site-determining protein MinD	WP_036916612.	192165..192980	Cell Division	COG2894
2.1_WP_036916612.1_	CUST_2167_PI442182273	MIT0602	LLII/III	2.1_WP_036916612.1_	GAGTCAAGAGATGCTATCCATCGAAGATGTAACTGATATTCTCGCGCTGCCTTTATTGGG	minD	septum site-determining protein MinD	WP_036916612.	192165..192980	Cell Division	COG2894

2.1_WP_036916612.1_	CUST_2168_PI442182273	MIT0602	LLII/III	2.1_WP_036916612.1_	CTCAGCTGTTAGAGATGCTGATAGGGTAATTGGCCTTCTTAATACTCACGGCATTAAGCC	minD	septum site-determining protein MinD	WP_036916612.	192165..192980	Cell Division	COG2894
2.1_WP_036916612.1_	CUST_2169_PI442182273	MIT0602	LLII/III	2.1_WP_036916612.1_	GGGAGTAATTCACCTGCAGCAAGATGTTATTCCAATATCGCAGGACGTCTTAAAGGAGAA	minD	septum site-determining protein MinD	WP_036916612.	192165..192980	Cell Division	COG2894
2.1_WP_036916612.1_	CUST_2170_PI442182273	MIT0602	LLII/III	2.1_WP_036916612.1_	CCCTGGCTAGAAAAGGTTCTCCAACTGCTGTACTAGATGCTGATTTGGTCTTCGTAATC	minD	septum site-determining protein MinD	WP_036916612.	192165..192980	Cell Division	COG2894
2.1_WP_036916610.1_	CUST_2171_PI442182273	MIT0602	LLII/III	2.1_WP_036916610.1_	TGAAACAACAGGAGTCAAATAGTGAATCAATATCGAAGGTGTCAGAAGAAGCATCTTCTT	minE	septum site-determining protein MinE	WP_036916610.	192986..193321	Cell Division	COG0851
2.1_WP_036916610.1_	CUST_2172_PI442182273	MIT0602	LLII/III	2.1_WP_036916610.1_	CTTGGAAGGAGGAGAAGTCAGCCTTGAAACAGAAGACAGAATGACTGCATTAGTAGCAAA	minE	septum site-determining protein MinE	WP_036916610.	192986..193321	Cell Division	COG0851
2.1_WP_036916610.1_	CUST_2173_PI442182273	MIT0602	LLII/III	2.1_WP_036916610.1_	GTCCAGACCTCTTGGACAAAATGAGAGCAGAAATCCTTGATGTGGTTGCGAAATATGTTG	minE	septum site-determining protein MinE	WP_036916610.	192986..193321	Cell Division	COG0851
2.1_WP_036916610.1_	CUST_2174_PI442182273	MIT0602	LLII/III	2.1_WP_036916610.1_	GTGCTGCTAAAGCTCGTGAAAGGCTCCAGTTAGTTCTAGCTCATGACAGAACTGATTTAA	minE	septum site-determining protein MinE	WP_036916610.	192986..193321	Cell Division	COG0851
2.1_WP_036916489.1_	CUST_2838_PI442182273	MIT0602	LLII/III	2.1_WP_036916489.1_	CAATTACTTCGCATTGAAGATGAATTGGGTGCACAAGCTGTTTATGCAGGTGCTATAGGA	eno	Enolase	WP_036916489.	305756..307054	Glycolysis/Gluconeogenesis	COG0148
2.1_WP_036916489.1_	CUST_2839_PI442182273	MIT0602	LLII/III	2.1_WP_036916489.1_	AGTCATAGGAGCGGAGAAACTGAAGATACTACAATTGCTGATCTTGCAGTTGGAACAAGG	eno	Enolase	WP_036916489.	305756..307054	Glycolysis/Gluconeogenesis	COG0148
2.1_WP_036916489.1_	CUST_2840_PI442182273	MIT0602	LLII/III	2.1_WP_036916489.1_	ATCAAATTGGTTCTTTGACAGAAACTCTAGAAGCCATAGAACTCGCAACAAGGTCAGGCT	eno	Enolase	WP_036916489.	305756..307054	Glycolysis/Gluconeogenesis	COG0148
2.1_WP_036916489.1_	CUST_2841_PI442182273	MIT0602	LLII/III	2.1_WP_036916489.1_	GATACAGCTGGTTTTCTGCCAGGCGAACAAATTTCATTGGCTTTAGATGTAGCAAGTACT	eno	Enolase	WP_036916489.	305756..307054	Glycolysis/Gluconeogenesis	COG0148
2.1_WP_036916489.1_	CUST_2842_PI442182273	MIT0602	LLII/III	2.1_WP_036916489.1_	GGGTTTATCTACTGCTGTGGGAGATGAAGGTGGATTTGCTCCTAATCTCACAAGCAATAA	eno	Enolase	WP_036916489.	305756..307054	Glycolysis/Gluconeogenesis	COG0148
2.1_WP_036916489.1_	CUST_2843_PI442182273	MIT0602	LLII/III	2.1_WP_036916489.1_	TCTTGATTTTCAGGAGTTTATGTTGGTCCCTCATGGGGCTAATAATTTTCGGGAAGCTCT	eno	Enolase	WP_036916489.	305756..307054	Glycolysis/Gluconeogenesis	COG0148
4.1_WP_036917404.1_	CUST_4465_PI442182273	MIT0602	LLII/III	4.1_WP_036917404.1_	CTTGATTGCCGTCCAATTAGACTTTCCAGTCAGGATATTCCTACTCCTTATAACGGGCAG	pdhB	Pyruvate dehydrogenase E1 component beta subunit	WP_036917404.	40934..41917	Glycolysis/Krebs cycle	COG0022
4.1_WP_036917404.1_	CUST_4462_PI442182273	MIT0602	LLII/III	4.1_WP_036917404.1_	GTAGACAGCTTGGAGCAGAACATAGTCAGAGGCTAGAAGCTTATTTTCATGCTGTTCCTG	pdhB	Pyruvate dehydrogenase E1 component beta subunit	WP_036917404.	40934..41917	Glycolysis/Krebs cycle	COG0022
4.1_WP_036917404.1_	CUST_4463_PI442182273	MIT0602	LLII/III	4.1_WP_036917404.1_	CCAGAAGGGGACTATATATGTGCGCTTGATCAGGCGGATTTGGTAAAAGAAGGTAAAGAT	pdhB	Pyruvate dehydrogenase E1 component beta subunit	WP_036917404.	40934..41917	Glycolysis/Krebs cycle	COG0022
4.1_WP_036917404.1_	CUST_4464_PI442182273	MIT0602	LLII/III	4.1_WP_036917404.1_	GCACTCCAACAAATGCAAAAGGATTAATGAAAGCTGCCATACGAGATAACAATCCCGTCT	pdhB	Pyruvate dehydrogenase E1 component beta subunit	WP_036917404.	40934..41917	Glycolysis/Krebs cycle	COG0022
4.1_WP_036917404.1_	CUST_4466_PI442182273	MIT0602	LLII/III	4.1_WP_036917404.1_	CATGACAGGCCTAAGGCCAATAGTAGAGGGTATGAATATGGATTTCTGCTCTTAGCTTT	pdhB	Pyruvate dehydrogenase E1 component beta subunit	WP_036917404.	40934..41917	Glycolysis/Krebs cycle	COG0022
4.1_WP_036917404.1_	CUST_4467_PI442182273	MIT0602	LLII/III	4.1_WP_036917404.1_	TATCTAACAACATGGGCATGTTGCGTTATACCAGTGGTGGCAACTTTACTATTCCAACAG	pdhB	Pyruvate dehydrogenase E1 component beta subunit	WP_036917404.	40934..41917	Glycolysis/Krebs cycle	COG0022
4.1_WP_036917506.1_	CUST_4875_PI442182273	MIT0602	LLII/III	4.1_WP_036917506.1_	GGAACAACTGTTAAACGTGCGATTTTGGATAAGAACACGCGTATTGGGGATAATGTCACA	glgC	Glucose-1-phosphate adenylyltransferase	WP_036917506.	115174..116469	Glycogen Metabolism	COG0448
4.1_WP_036917506.1_	CUST_4876_PI442182273	MIT0602	LLII/III	4.1_WP_036917506.1_	TTTTGGCCTTAGCAAAGAATCTGCAAAAGATAGGCCATACTTGGCTTCAATGGGCATATA	glgC	Glucose-1-phosphate adenylyltransferase	WP_036917506.	115174..116469	Glycogen Metabolism	COG0448
4.1_WP_036917506.1_	CUST_4877_PI442182273	MIT0602	LLII/III	4.1_WP_036917506.1_	TTTGCAGGATTCCCTCGTAATGGGATCTGATTTTTATGAGTCAGGAGAAGAAAGAGACGC	glgC	Glucose-1-phosphate adenylyltransferase	WP_036917506.	115174..116469	Glycogen Metabolism	COG0448
4.1_WP_036917506.1_	CUST_4878_PI442182273	MIT0602	LLII/III	4.1_WP_036917506.1_	TGGAAATATTAAAGAGTTCAAAGAGAAGCCTAGTGGTGAAGCTCTTAAGGCAATGGCCGT	glgC	Glucose-1-phosphate adenylyltransferase	WP_036917506.	115174..116469	Glycogen Metabolism	COG0448
4.1_WP_036917506.1_	CUST_4879_PI442182273	MIT0602	LLII/III	4.1_WP_036917506.1_	ACACCCCTCTCATAAAGACTTTGGCAAGGAAGTTATACCAGAAGCATTAAACAGAGGTGA	glgC	Glucose-1-phosphate adenylyltransferase	WP_036917506.	115174..116469	Glycogen Metabolism	COG0448
4.1_WP_036917506.1_	CUST_4880_PI442182273	MIT0602	LLII/III	4.1_WP_036917506.1_	TTGGAACTATTGGGGCTTTCTACGAATCAAACTTGGCTTTAACTCAGCAACCTAAACCTC	glgC	Glucose-1-phosphate adenylyltransferase	WP_036917506.	115174..116469	Glycogen Metabolism	COG0448
4.1_WP_036917507.1_	CUST_4881_PI442182273	MIT0602	LLII/III	4.1_WP_036917507.1_	TCTCCGATTATGGACGCAGTAGTTTTGTCGACTATTGCAAGTTATGCACAAGGCATAGAA	edd	phosphogluconate dehydrogenase (NADP(+)-dependent, decarboxyla	WP_036917507.	116629..118047	Pentose Phosphate/Entner-Dudorof	COG2084
4.1_WP_036917507.1_	CUST_4882_PI442182273	MIT0602	LLII/III	4.1_WP_036917507.1_	AGATGGGTCTGACTTGGTTGAGAAGATAATAGATAAGGCTGGTCAGAAAGGTACTGGCTT	edd	phosphogluconate dehydrogenase (NADP(+)-dependent, decarboxyla	WP_036917507.	116629..118047	Pentose Phosphate/Entner-Dudorof	COG2084
4.1_WP_036917507.1_	CUST_4883_PI442182273	MIT0602	LLII/III	4.1_WP_036917507.1_	CCCCAGAACTTAGTACAAGCTATGCGGGACTGTTTTGGCTCACATACATATGAACGTATT	edd	phosphogluconate dehydrogenase (NADP(+)-dependent, decarboxyla	WP_036917507.	116629..118047	Pentose Phosphate/Entner-Dudorof	COG2084
4.1_WP_036917507.1_	CUST_4884_PI442182273	MIT0602	LLII/III	4.1_WP_036917507.1_	TCAGATCCCTTCCATTGCTCAGATTTGGAAAGGCGGTTGCATAATACGATCTACTCTTTT	edd	phosphogluconate dehydrogenase (NADP(+)-dependent, decarboxyla	WP_036917507.	116629..118047	Pentose Phosphate/Entner-Dudorof	COG2084
4.1_WP_036917507.1_	CUST_4885_PI442182273	MIT0602	LLII/III	4.1_WP_036917507.1_	TTGGTTCAGTGACCAAGTTTCCAGAAGATTGCCAGGTCTTACGAAAGTAGTCTCTATTGC	edd	phosphogluconate dehydrogenase (NADP(+)-dependent, decarboxyla	WP_036917507.	116629..118047	Pentose Phosphate/Entner-Dudorof	COG2084
4.1_WP_036917507.1_	CUST_4886_PI442182273	MIT0602	LLII/III	4.1_WP_036917507.1_	GCACAGGCTGGCGTACCGGTCCCATGTTTTAGTAGCACGCTTGATTATATAAATAGTTAT	edd	phosphogluconate dehydrogenase (NADP(+)-dependent, decarboxyla	WP_036917507.	116629..118047	Pentose Phosphate/Entner-Dudorof	COG2084
4.1_WP_036918207.1_	CUST_4944_PI442182273	MIT0602	LLII/III	4.1_WP_036918207.1_	TAAGGGAAGCTGCAGCTGCTGATCCAACCAACTTTGATCCAAGACATTTTAATAAACCAG	cbbA	Fructose-1,6-bisphosphate aldolase class II	WP_036918207.	125524..126597	Glycolysis/Gluconeogenesis	COG0191
4.1_WP_036918207.1_	CUST_4945_PI442182273	MIT0602	LLII/III	4.1_WP_036918207.1_	CGAAAGCCAACTGGTGAAGTGCTTGCTATTAGTCGAATTGCAGAAATTCATAAGGCCATA	cbbA	Fructose-1,6-bisphosphate aldolase class II	WP_036918207.	125524..126597	Glycolysis/Gluconeogenesis	COG0191
4.1_WP_036918207.1_	CUST_4946_PI442182273	MIT0602	LLII/III	4.1_WP_036918207.1_	AAACACTCATCTTGTAATGCATGGATCAAGCTCTGTCCCTCAAGAATGGCTAGACATGAT	cbbA	Fructose-1,6-bisphosphate aldolase class II	WP_036918207.	125524..126597	Glycolysis/Gluconeogenesis	COG0191
4.1_WP_036918207.1_	CUST_4947_PI442182273	MIT0602	LLII/III	4.1_WP_036918207.1_	GTAGGGGTAAGTGTAGAAGGTGAATTGGGATGTTTAGGATCACTAGAGACAGGCAAAGGA	cbbA	Fructose-1,6-bisphosphate aldolase class II	WP_036918207.	125524..126597	Glycolysis/Gluconeogenesis	COG0191
4.1_WP_036918207.1_	CUST_4948_PI442182273	MIT0602	LLII/III	4.1_WP_036918207.1_	GTAATGATGGATGGCTCCCTAGAGGCTGACGCCAAAACTCCATCAAGTTACGAATATAAC	cbbA	Fructose-1,6-bisphosphate aldolase class II	WP_036918207.	125524..126597	Glycolysis/Gluconeogenesis	COG0191
4.1_WP_036918207.1_	CUST_4949_PI442182273	MIT0602	LLII/III	4.1_WP_036918207.1_	GCAATACCTGAGACATACGGAGTGCCTGTAGAAGAAATACAAGAAGGGATACGTAACGGA	cbbA	Fructose-1,6-bisphosphate aldolase class II	WP_036918207.	125524..126597	Glycolysis/Gluconeogenesis	COG0191
4.1_WP_036918209.1_	CUST_4950_PI442182273	MIT0602	LLII/III	4.1_WP_036918209.1_	AAAAGCTAATTGGAATGTTGGATTCTCTTATGGACGTGCTCTGCAACATTCATGCCTCAA	fda	Fructose-1,6-bisphosphate aldolase class I	WP_036918209.	126748..127815	Glycolysis/Gluconeogenesis	COG0191
4.1_WP_036918209.1_	CUST_4951_PI442182273	MIT0602	LLII/III	4.1_WP_036918209.1_	CGGAAATTACAATTAGGACTATGGAGAGATCTGTTCCGGCTGCAGTACCAGGAATTGTTT	fda	Fructose-1,6-bisphosphate aldolase class I	WP_036918209.	126748..127815	Glycolysis/Gluconeogenesis	COG0191
4.1_WP_036918209.1_	CUST_4952_PI442182273	MIT0602	LLII/III	4.1_WP_036918209.1_	AGCTGATGGTTGTCCATCAAAATTGTCATTAAAGGAAAATGCTTGGGGCTTGGCTAGATA	fda	Fructose-1,6-bisphosphate aldolase class I	WP_036918209.	126748..127815	Glycolysis/Gluconeogenesis	COG0191
4.1_WP_036918209.1_	CUST_4953_PI442182273	MIT0602	LLII/III	4.1_WP_036918209.1_	TATTGCTTGCCAAGACAATGGCGTCTACCTAGAAGGGACATTATTGAAGCCTTCTATGAC	fda	Fructose-1,6-bisphosphate aldolase class I	WP_036918209.	126748..127815	Glycolysis/Gluconeogenesis	COG0191
4.1_WP_036918209.1_	CUST_4954_PI442182273	MIT0602	LLII/III	4.1_WP_036918209.1_	TAGTAGAAAGAGCCTCAGATTATTACGCACAAGGTGCTCGTTTTGCTAAATGGAGAGCTG	fda	Fructose-1,6-bisphosphate aldolase class I	WP_036918209.	126748..127815	Glycolysis/Gluconeogenesis	COG0191
4.1_WP_036918209.1_	CUST_4955_PI442182273	MIT0602	LLII/III	4.1_WP_036918209.1_	TGAGCCTGAGATATTAATGGATGGCTCTCATGGAATAAATCAAACTGCCTCTGTGCAAGA	fda	Fructose-1,6-bisphosphate aldolase class I	WP_036918209.	126748..127815	Glycolysis/Gluconeogenesis	COG0191
4.1_WP_036917523.1_	CUST_4979_PI442182273	MIT0602	LLII/III	4.1_WP_036917523.1_	CTTGGAACAACCTTAGGGCCAGGACAATTCACTCCTTGGGAAGATATTCCTAAGAAAACT	prk	Phosphoribulokinase	WP_036917523.	130967..131866	Carbon Fixation	COG3954
4.1_WP_036917523.1_	CUST_4980_PI442182273	MIT0602	LLII/III	4.1_WP_036917523.1_	GTCGTTGGAGATGGTTATGACGTAGCCAAATATGCAGACTTACTAGTAGGAGTAGTGCCA	prk	Phosphoribulokinase	WP_036917523.	130967..131866	Carbon Fixation	COG3954
4.1_WP_036917523.1_	CUST_4981_PI442182273	MIT0602	LLII/III	4.1_WP_036917523.1_	AGGCATGATTAACGACTCATTTATGTCTAGCCCAACAAGCATAGTAGTGAATGGAGGCAA	prk	Phosphoribulokinase	WP_036917523.	130967..131866	Carbon Fixation	COG3954
4.1_WP_036917523.1_	CUST_4982_PI442182273	MIT0602	LLII/III	4.1_WP_036917523.1_	AGCTCTTGCAAAAGGCGAGAATTTTTCTCACTTTGGTCCTGAAGCAAATCTATTCGACAA	prk	Phosphoribulokinase	WP_036917523.	130967..131866	Carbon Fixation	COG3954
4.1_WP_036917523.1_	CUST_4983_PI442182273	MIT0602	LLII/III	4.1_WP_036917523.1_	TAAAACATACGGAGAGAAAGGAGGTGGCAGCAAGAGATACTACCTTCATAGCCAAGAAGA	prk	Phosphoribulokinase	WP_036917523.	130967..131866	Carbon Fixation	COG3954
4.1_WP_036917523.1_	CUST_4984_PI442182273	MIT0602	LLII/III	4.1_WP_036917523.1_	ACACCCGATGAAAGTTTCGTAATTATCCATTTCCGAAAAGGAGCTAGAGAAAAATGGGGA	prk	Phosphoribulokinase	WP_036917523.	130967..131866	Carbon Fixation	COG3954
4.1_WP_036917552.1_	CUST_5095_PI442182273	MIT0602	LLII/III	4.1_WP_036917552.1_	ATCAATTAAGCGATGTTTTGAAAACTTGTTCGGCCACCGCGATTTTAGTAACACATGACC		Sugar ABC transporter	WP_036917552.	147492..148556	Other sugar transporters	
4.1_WP_036917552.1_	CUST_5092_PI442182273	MIT0602	LLII/III	4.1_WP_036917552.1_	TCAAGAAGCACTTGGTATATGTGATCGTGTAGCAGTAATGCGTGCAGGTGTTATTCATCA		Sugar ABC transporter	WP_036917552.	147492..148556	Other sugar transporters	
4.1_WP_036917552.1_	CUST_5093_PI442182273	MIT0602	LLII/III	4.1_WP_036917552.1_	CTCCACCTCATACCCTAATGTTTGATGAAATATCGCTAGAGCTATTTCCATCGCCCGAAG		Sugar ABC transporter	WP_036917552.	147492..148556	Other sugar transporters	
4.1_WP_036917552.1_	CUST_5094_PI442182273	MIT0602	LLII/III	4.1_WP_036917552.1_	TCGGACTTGCAAGAGCATTAGCTCCTGGAACTTCTTTAGTGCTATTGGATGAACCTTTTA		Sugar ABC transporter	WP_036917552.	147492..148556	Other sugar transporters	
4.1_WP_036917552.1_	CUST_5096_PI442182273	MIT0602	LLII/III	4.1_WP_036917552.1_	TGGTCTAAATGACTTGCAGAAAAGATATCCACATGAATTATCAGGAGGACAACGCCAACG		Sugar ABC transporter	WP_036917552.	147492..148556	Other sugar transporters	
4.1_WP_036917552.1_	CUST_5097_PI442182273	MIT0602	LLII/III	4.1_WP_036917552.1_	ATGCTCGACCCCTCTTGATTTACTAACAAATCCAGATACTCCTTTCATTGGACATTTTGT		Sugar ABC transporter	WP_036917552.	147492..148556	Other sugar transporters	
4.1_WP_036917625.1_	CUST_5308_PI442182273	MIT0602	LLII/III	4.1_WP_036917625.1_	TGTTACTGATGTAGTAGCAAACGGTCATTCATCAGGATCAGGTAGGGTAAGTGGCAAATT	pykF	Pyruvate kinase	WP_036917625.	187501..189264	Glycolysis	COG0469
4.1_WP_036917625.1_	CUST_5309_PI442182273	MIT0602	LLII/III	4.1_WP_036917625.1_	AGAAACCGCTGGAACATTAACAGGCATAAGTGGCTCAACAGATTTGATCAAAGTTGGAAT	pykF	Pyruvate kinase	WP_036917625.	187501..189264	Glycolysis	COG0469
4.1_WP_036917625.1_	CUST_5310_PI442182273	MIT0602	LLII/III	4.1_WP_036917625.1_	GTGTTGCCAGACGACTCCAATTAGTATGGGGAGTCTCGGCTTTACTAATTGAGGAAATAC	pykF	Pyruvate kinase	WP_036917625.	187501..189264	Glycolysis	COG0469
4.1_WP_036917625.1_	CUST_5311_PI442182273	MIT0602	LLII/III	4.1_WP_036917625.1_	CAATCCTTCCACTAACCAAAAGTGGAGCAACGGCTCACAATGTAAGTAAATTTCGTCCAG	pykF	Pyruvate kinase	WP_036917625.	187501..189264	Glycolysis	COG0469
4.1_WP_036917625.1_	CUST_5312_PI442182273	MIT0602	LLII/III	4.1_WP_036917625.1_	AGTCATCTGCCAAGTACTATCCCAAATGCTATAAGCGCAGCAGTAAGTACTATTGCAAGG	pykF	Pyruvate kinase	WP_036917625.	187501..189264	Glycolysis	COG0469
4.1_WP_036917625.1_	CUST_5313_PI442182273	MIT0602	LLII/III	4.1_WP_036917625.1_	TCGAATGAAACGGCTGTTGGTGATTATCCAATAGAAGCTGTCAGAACAATGGCAACAATT	pykF	Pyruvate kinase	WP_036917625.	187501..189264	Glycolysis	COG0469
4.1_WP_052043876.1_	CUST_5445_PI442182273	MIT0602	LLII/III	4.1_WP_052043876.1_	GTCAAAAGGTAAATCAAGGTTTGGCAGTCTATGGCAACAAGGGCTCTACAGATCAACATG	pgi	Glucose-6-phosphate isomerase	WP_052043876.	212066..213667	Glycolysis/Gluconeogenesis	COG0166
4.1_WP_052043876.1_	CUST_5446_PI442182273	MIT0602	LLII/III	4.1_WP_052043876.1_	ATTGTAGATAACCCAGCAGCTTTGTTGGCTTCTGTTTGGTTTTCTTCAGGACAAGGAAAA	pgi	Glucose-6-phosphate isomerase	WP_052043876.	212066..213667	Glycolysis/Gluconeogenesis	COG0166
4.1_WP_052043876.1_	CUST_5447_PI442182273	MIT0602	LLII/III	4.1_WP_052043876.1_	GCTTATCGATATTAATGCATACCATCAACCTGGAGTTGAGGCTGGAAAGAAAGCAGCTTC	pgi	Glucose-6-phosphate isomerase	WP_052043876.	212066..213667	Glycolysis/Gluconeogenesis	COG0166
4.1_WP_052043876.1_	CUST_5448_PI442182273	MIT0602	LLII/III	4.1_WP_052043876.1_	ACTTGGCAGCAAACAATTCTGTATATAAAGTAAACGGAGATTGGTCAGAACCTAGGTCCT	pgi	Glucose-6-phosphate isomerase	WP_052043876.	212066..213667	Glycolysis/Gluconeogenesis	COG0166
4.1_WP_052043876.1_	CUST_5449_PI442182273	MIT0602	LLII/III	4.1_WP_052043876.1_	TCTCCAGGTGATTATCTTTCTGGATTTTTACAAGGGACTCGGTTAGCACTATCTGAGAAT	pgi	Glucose-6-phosphate isomerase	WP_052043876.	212066..213667	Glycolysis/Gluconeogenesis	COG0166
4.1_WP_052043876.1_	CUST_5450_PI442182273	MIT0602	LLII/III	4.1_WP_052043876.1_	ACTCATATTCGCTTGGAGCACTAATTGCTTTGTTTGAAAGAAGTGTTGGTTTATATGCAG	pgi	Glucose-6-phosphate isomerase	WP_052043876.	212066..213667	Glycolysis/Gluconeogenesis	COG0166
4.1_WP_036931469.1_	CUST_5932_PI442182273	MIT0602	LLII/III	4.1_WP_036931469.1_	GGAAGGATGGGCAACCACTTTTCTTCGGTGAAAATACATATGCCAACCTATCTCAAACTG	glnA	Glutamine synthetase/ glutamate--ammonialigase	WP_036931469.	303610..305031	Nitrogen Metabolism	COG0174
4.1_WP_036931469.1_	CUST_5933_PI442182273	MIT0602	LLII/III	4.1_WP_036931469.1_	TGATGCTATAGCAAATCCCTATTTAGCCTTTAGCGCAATGCTAATGGCTGGAATTGATGG	glnA	Glutamine synthetase/ glutamate--ammonialigase	WP_036931469.	303610..305031	Nitrogen Metabolism	COG0174
4.1_WP_036931469.1_	CUST_5934_PI442182273	MIT0602	LLII/III	4.1_WP_036931469.1_	TGGTATATAGGTGGCATTCTTAAACATGCACCTTCTTTCTTAGCCTTCACAAACCCAGGA	glnA	Glutamine synthetase/ glutamate--ammonialigase	WP_036931469.	303610..305031	Nitrogen Metabolism	COG0174
4.1_WP_036931469.1_	CUST_5935_PI442182273	MIT0602	LLII/III	4.1_WP_036931469.1_	TTTTATGCCAAAACCTATCTGGAATGACAATGGAACTGGCATGCATGTCCATCAAAGTCT	glnA	Glutamine synthetase/ glutamate--ammonialigase	WP_036931469.	303610..305031	Nitrogen Metabolism	COG0174
4.1_WP_036931469.1_	CUST_5936_PI442182273	MIT0602	LLII/III	4.1_WP_036931469.1_	GAACTTCCCTCAGAGAACTTGCCAAGATTGCAACAGTTCCATCATCCTTAAATGATGCA	glnA	Glutamine synthetase/ glutamate--ammonialigase	WP_036931469.	303610..305031	Nitrogen Metabolism	COG0174
4.1_WP_036931469.1_	CUST_5937_PI442182273	MIT0602	LLII/III	4.1_WP_036931469.1_	CCAGGGTTTGAAGCCCAGTAAATCTTGTTTACTCCCAAGGTAATAGATCAGCTGCTATT	glnA	Glutamine synthetase/ glutamate--ammonialigase	WP_036931469.	303610..305031	Nitrogen Metabolism	COG0174
4.1_WP_036917878.1_	CUST_6099_PI442182273	MIT0602	LLII/III	4.1_WP_036917878.1_	ATATTACACCAAATATTTATGCAATTCTTGGCGCTGGTACAGGACTTGGTGTGGCAAGAG	glk	Glucokinase	WP_036917878.	334975..336027	Glycolysis	COG0837
4.1_WP_036917878.1_	CUST_6096_PI442182273	MIT0602	LLII/III	4.1_WP_036917878.1_	GGAATTCCAGTCATGGCAATAACCGATCGAAATTGTGGACTATTTGGAGCAGCATGTAAA	glk	Glucokinase	WP_036917878.	334975..336027	Glycolysis	COG0837
4.1_WP_036917878.1_	CUST_6097_PI442182273	MIT0602	LLII/III	4.1_WP_036917878.1_	ACTGGCTTGGTGAATATTGCAAGATGGAGATTGACAAAAGAGGATGCAAAATTCCATCCT	glk	Glucokinase	WP_036917878.	334975..336027	Glycolysis	COG0837
4.1_WP_036917878.1_	CUST_6098_PI442182273	MIT0602	LLII/III	4.1_WP_036917878.1_	GATTAATTACAAGCACCGAAATCAAAGTGCTACCCAGCGAAGGCGGACATAGAGAATTTG	glk	Glucokinase	WP_036917878.	334975..336027	Glycolysis	COG0837
4.1_WP_036917878.1_	CUST_6100_PI442182273	MIT0602	LLII/III	4.1_WP_036917878.1_	GGGGGTGGTCCCGCTGCTAGACATATCGACAACTTTAAATCAGAAAGCTTTTTAAAATCA	glk	Glucokinase	WP_036917878.	334975..336027	Glycolysis	COG0837
4.1_WP_036917878.1_	CUST_6101_PI442182273	MIT0602	LLII/III	4.1_WP_036917878.1_	AGGAGATCCTGTCATGAGAGAAGTATTGGACATATGGATAAGTGCTTATGGATCAGCGGC	glk	Glucokinase	WP_036917878.	334975..336027	Glycolysis	COG0837
4.1_WP_036917952.1_	CUST_6426_PI442182273	MIT0602	LLII/III	4.1_WP_036917952.1_	CTCTTTTGGAACTGATTGAAGACAGCCCATGGCAACTACCTACTTATCCCTATGAAGCTC	zwf	glucose-6-phosphate dehydrogenase zwf	WP_036917952.	402261..403781	Pentose Phosphate	COG0364
4.1_WP_036917952.1_	CUST_6427_PI442182273	MIT0602	LLII/III	4.1_WP_036917952.1_	CATTTTGACCCAGAAGCTATTAGGAATGAAAAAGCCAAAGTTTTGCAAGCCGCTCATCTG	zwf	glucose-6-phosphate dehydrogenase zwf	WP_036917952.	402261..403781	Pentose Phosphate	COG0364
4.1_WP_036917952.1_	CUST_6428_PI442182273	MIT0602	LLII/III	4.1_WP_036917952.1_	GCAGGTGCATTACGAGACATGGTACAGAATCATTTAACTCAAATGCTTGCCATTACAGCT	zwf	glucose-6-phosphate dehydrogenase zwf	WP_036917952.	402261..403781	Pentose Phosphate	COG0364
4.1_WP_036917952.1_	CUST_6429_PI442182273	MIT0602	LLII/III	4.1_WP_036917952.1_	TTAAGAATTCAGCCTAACGAAGGTGCTGATTTTCGATTTGAAGTGAAGTCTCCAGGGTCA	zwf	glucose-6-phosphate dehydrogenase zwf	WP_036917952.	402261..403781	Pentose Phosphate	COG0364
4.1_WP_036917952.1_	CUST_6430_PI442182273	MIT0602	LLII/III	4.1_WP_036917952.1_	CTTACGACGAATCATTTGGAGAGCCTTCAGACGAAGGATACGTTAGGCTTTTAGCTGATG	zwf	glucose-6-phosphate dehydrogenase zwf	WP_036917952.	402261..403781	Pentose Phosphate	COG0364
4.1_WP_036917952.1_	CUST_6431_PI442182273	MIT0602	LLII/III	4.1_WP_036917952.1_	AAGGTTGGCTAAAAGGACTAGCGAAGTTGTTCTAACTTTTAGGGAGGCTCCTGTTCATTT	zwf	glucose-6-phosphate dehydrogenase zwf	WP_036917952.	402261..403781	Pentose Phosphate	COG0364
4.1_WP_036917953.1_	CUST_6432_PI442182273	MIT0602	LLII/III	4.1_WP_036917953.1_	TAAGATTAATCTGCAAAGCCGACAGGAATGACAAAACAGGGTTATGTGTGATCCTAGCCT	opcA	Glucose-6-P dehydrogenase subunit opcA	WP_036917953.	403821..405128	Pentose Phosphate	COG3429
4.1_WP_036917953.1_	CUST_6433_PI442182273	MIT0602	LLII/III	4.1_WP_036917953.1_	CTTAATACCTGAGGATCTACCTTCATGGCTTTGGTGGAATGGAAATCTTGACGAAGACCC	opcA	Glucose-6-P dehydrogenase subunit opcA	WP_036917953.	403821..405128	Pentose Phosphate	COG3429
4.1_WP_036917953.1_	CUST_6434_PI442182273	MIT0602	LLII/III	4.1_WP_036917953.1_	GTTAAGACTAAGAACTTGGAGAGAGACTCTAGCAATGGTGTTCGACCCACCTAACCGTAG	opcA	Glucose-6-P dehydrogenase subunit opcA	WP_036917953.	403821..405128	Pentose Phosphate	COG3429
4.1_WP_036917953.1_	CUST_6435_PI442182273	MIT0602	LLII/III	4.1_WP_036917953.1_	CCGAGGCACCACAAGTCCTTTACTTAGCAATGTGGCTCCAATATCAAAAAAACTGCTTAA	opcA	Glucose-6-P dehydrogenase subunit opcA	WP_036917953.	403821..405128	Pentose Phosphate	COG3429
4.1_WP_036917953.1_	CUST_6436_PI442182273	MIT0602	LLII/III	4.1_WP_036917953.1_	GGGGATCCATCGAGATGTTTAGATGTTCTATCTCAGAGGATAAAAAGTGGCCAAGCAGTT	opcA	Glucose-6-P dehydrogenase subunit opcA	WP_036917953.	403821..405128	Pentose Phosphate	COG3429
4.1_WP_036917953.1_	CUST_6437_PI442182273	MIT0602	LLII/III	4.1_WP_036917953.1_	GGGATGGCCAGAATGGAATTATTGGAAGAGGTAGTTCCAATACAAAACAACCCTGTTGAA	opcA	Glucose-6-P dehydrogenase subunit opcA	WP_036917953.	403821..405128	Pentose Phosphate	COG3429
4.1_WP_036917996.1_	CUST_6609_PI442182273	MIT0602	LLII/III	4.1_WP_036917996.1_	GGTGGGGCTGTAGCAGCAGTGCAAATGTCAAAACTAACCAATCAAAAAGTTCATTTCTTT	rbsK	Sugar kinase, ribokinase family	WP_036917996.	437281..438102	Pentose Phosphate	COG0524
4.1_WP_036917996.1_	CUST_6607_PI442182273	MIT0602	LLII/III	4.1_WP_036917996.1_	ACTTTGATGCTGTGTTTATAACAGCAGGAGATTCAGAGGCAATTAATGCTTGCAGGGAAG	rbsK	Sugar kinase, ribokinase family	WP_036917996.	437281..438102	Pentose Phosphate	COG0524
4.1_WP_036917996.1_	CUST_6608_PI442182273	MIT0602	LLII/III	4.1_WP_036917996.1_	CTTTGTAGATAAAAATGGGGATCGTGCCATAACAGTTGTTGGAGAAAGGCTTCAACCGTC	rbsK	Sugar kinase, ribokinase family	WP_036917996.	437281..438102	Pentose Phosphate	COG0524
4.1_WP_036917996.1_	CUST_6610_PI442182273	MIT0602	LLII/III	4.1_WP_036917996.1_	AAAACAGAAGGCGCTTTAGGTGGGCTAATAACACCAGGTGGTAGATATGAAGCAGTAAAT	rbsK	Sugar kinase, ribokinase family	WP_036917996.	437281..438102	Pentose Phosphate	COG0524
4.1_WP_036917996.1_	CUST_6611_PI442182273	MIT0602	LLII/III	4.1_WP_036917996.1_	CAACTCGGTGCAAAATGTGGGGCAGAATGCGCTTCTCACTTTGGAGCTTATAATAAATAA	rbsK	Sugar kinase, ribokinase family	WP_036917996.	437281..438102	Pentose Phosphate	COG0524
4.1_WP_036917996.1_	CUST_6612_PI442182273	MIT0602	LLII/III	4.1_WP_036917996.1_	GACAGCTTTGCAGCAGGAGTTACTGCAGGATTAGCAGCAAATCTGGAGATAAAAGAAG	rbsK	Sugar kinase, ribokinase family	WP_036917996.	437281..438102	Pentose Phosphate	COG0524
6.1_WP_036919465.1_	CUST_8033_PI442182273	MIT0602	LLII/III	6.1_WP_036919465.1_	TGCCCAAGAAGCTAAAGCTAGAGATGCATCTTTAATTGGCGTAGCACCAATATCAACAGA	glmS	Glutamine--fructose-6-phosphatetransaminase(isomerizing)	WP_036919465.	142782..144686	Amino Acid metabolism	COG0449
6.1_WP_036919465.1_	CUST_8034_PI442182273	MIT0602	LLII/III	6.1_WP_036919465.1_	AGCATGGTCCGATTGCTTTATTGGACAAGCAAGTTCCAGTTATCTCAATCGCAACTAAAG	glmS	Glutamine--fructose-6-phosphatetransaminase(isomerizing)	WP_036919465.	142782..144686	Amino Acid metabolism	COG0449
6.1_WP_036919465.1_	CUST_8035_PI442182273	MIT0602	LLII/III	6.1_WP_036919465.1_	AGATGTAATTTTTCTGGGTAGAGGGATTAATTATCCGATAGCACTGGAGGCTGCCTTGAA	glmS	Glutamine--fructose-6-phosphatetransaminase(isomerizing)	WP_036919465.	142782..144686	Amino Acid metabolism	COG0449
6.1_WP_036919465.1_	CUST_8036_PI442182273	MIT0602	LLII/III	6.1_WP_036919465.1_	GTTTGCAGCCCGTAGGAAAAGTCGTTCCACAGAAGAAATAGTTGATTTGTGTAGCCAATT	glmS	Glutamine--fructose-6-phosphatetransaminase(isomerizing)	WP_036919465.	142782..144686	Amino Acid metabolism	COG0449
6.1_WP_036919465.1_	CUST_8037_PI442182273	MIT0602	LLII/III	6.1_WP_036919465.1_	TGATATAGGAGCAGGGATGGAAGTTGGAGTTGCGGCTACTAAAACTTTTCTTGGACAATT	glmS	Glutamine--fructose-6-phosphatetransaminase(isomerizing)	WP_036919465.	142782..144686	Amino Acid metabolism	COG0449
6.1_WP_036919465.1_	CUST_8038_PI442182273	MIT0602	LLII/III	6.1_WP_036919465.1_	ATTTTGACCATTGGAGTTACTCAATCTGGTGAGACAGCAGATACTCTTGCAGCATTAGCT	glmS	Glutamine--fructose-6-phosphatetransaminase(isomerizing)	WP_036919465.	142782..144686	Amino Acid metabolism	COG0449
6.1_WP_036919443.1_	CUST_8094_PI442182273	MIT0602	LLII/III	6.1_WP_036919443.1_	AAAGAGAAGGTGAAGATGCTCTTCTTCATCGTTATGTTGCAGGGTATGCTCCAACAAGAT	dld	D-lactate dehydrogenase, NADH independent	WP_036919443.	155324..157024	Pyruvate metabolism	COG0277
6.1_WP_036919443.1_	CUST_8095_PI442182273	MIT0602	LLII/III	6.1_WP_036919443.1_	ACCACTAGATGTTGCATTGCCTAGGAATTATACTTCTTGGTACGAGGTTTTGCCTAAGGA	dld	D-lactate dehydrogenase, NADH independent	WP_036919443.	155324..157024	Pyruvate metabolism	COG0277
6.1_WP_036919443.1_	CUST_8096_PI442182273	MIT0602	LLII/III	6.1_WP_036919443.1_	TGGGCATCTCTATGATGCAGAAACTAACCTAAAGGATTTTTATAAGGAACTTGATCCAAC	dld	D-lactate dehydrogenase, NADH independent	WP_036919443.	155324..157024	Pyruvate metabolism	COG0277
6.1_WP_036919443.1_	CUST_8097_PI442182273	MIT0602	LLII/III	6.1_WP_036919443.1_	AACTGAAATGGCAGAGATTTTTCAAATGGGTCATTTTTTATGTATGGTCTTTCATTTGGA	dld	D-lactate dehydrogenase, NADH independent	WP_036919443.	155324..157024	Pyruvate metabolism	COG0277
6.1_WP_036919443.1_	CUST_8098_PI442182273	MIT0602	LLII/III	6.1_WP_036919443.1_	CCTACCTGATAACTTAATGGATAAGTTTCTTCAATTGATAGCTGGTTTAATCCCAGATCA	dld	D-lactate dehydrogenase, NADH independent	WP_036919443.	155324..157024	Pyruvate metabolism	COG0277
6.1_WP_036919443.1_	CUST_8099_PI442182273	MIT0602	LLII/III	6.1_WP_036919443.1_	TTTGGCACAAAATCATTGCCAAATATTTTTCTTTTAAAGAGATCTATTGATAGATTTGCT	dld	D-lactate dehydrogenase, NADH independent	WP_036919443.	155324..157024	Pyruvate metabolism	COG0277
6.1_WP_081936974.1_	CUST_8235_PI442182273	MIT0602	LLII/III	6.1_WP_081936974.1_	ATTGTGCCTTTAGGCTTTCTGCAAGTTGCTTTATTGCGAAGATTACGTGATCAGAATCGT	ppc	Phosphoenolpyruvate carboxylase	WP_081936974.	181059..184073	Krebs cycle and related genes	COG2352
6.1_WP_081936974.1_	CUST_8236_PI442182273	MIT0602	LLII/III	6.1_WP_081936974.1_	AACCTCCTATCAGTGAGACACTATTGGCTGAAGGTGAGGTAGGACGGACATATAGTAGAA	ppc	Phosphoenolpyruvate carboxylase	WP_081936974.	181059..184073	Krebs cycle and related genes	COG2352
6.1_WP_081936974.1_	CUST_8237_PI442182273	MIT0602	LLII/III	6.1_WP_081936974.1_	GCGTTGGTACTGCTTTGGAAGAGGAGCTGAGTGCACAACCAAATCAAATTGACTTATTAA	ppc	Phosphoenolpyruvate carboxylase	WP_081936974.	181059..184073	Krebs cycle and related genes	COG2352
6.1_WP_081936974.1_	CUST_8238_PI442182273	MIT0602	LLII/III	6.1_WP_081936974.1_	ACGCCAATTGAGGAGATTAGTAAGTTGCAAATATCTAGTCGTCCAGCACGAAGAAAAGCA	ppc	Phosphoenolpyruvate carboxylase	WP_081936974.	181059..184073	Krebs cycle and related genes	COG2352
6.1_WP_081936974.1_	CUST_8239_PI442182273	MIT0602	LLII/III	6.1_WP_081936974.1_	TATTAAGAGGTGCATTGTTGACAATTAATGGAATTGCAGCGGGCATGCGTAATACAGGTT	ppc	Phosphoenolpyruvate carboxylase	WP_081936974.	181059..184073	Krebs cycle and related genes	COG2352
6.1_WP_081936974.1_	CUST_8240_PI442182273	MIT0602	LLII/III	6.1_WP_081936974.1_	GCTGAAAGATCACGACAACATTACCGCGCTTTAGTCCATGATAATCCTGACTTAGTTGCT	ppc	Phosphoenolpyruvate carboxylase	WP_081936974.	181059..184073	Krebs cycle and related genes	COG2352
6.1_WP_036919280.1_	CUST_8548_PI442182273	MIT0602	LLII/III	6.1_WP_036919280.1_	ATTTTGGTTATGCATTCCCATGTGATGGTCCAGGTAGAGGTGGTACTTGTGATAGTTCAG	psaB	Photosystem I P700 chlorophyll A apoprotein A2 PsaB	WP_036919280.	225300..227528	Photosystem I	COG2885
6.1_WP_036919280.1_	CUST_8552_PI442182273	MIT0602	LLII/III	6.1_WP_036919280.1_	GATTTCTTGGTCCACCATGCCATCGCTTTAGGTCTTCACACAACTGCATTAATCCTTATC	psaB	Photosystem I P700 chlorophyll A apoprotein A2 PsaB	WP_036919280.	225300..227528	Photosystem I	COG2885
6.1_WP_036919280.1_	CUST_8549_PI442182273	MIT0602	LLII/III	6.1_WP_036919280.1_	TGTTCCTCTTTGGCCATTTAATCTGGGCAACAGGCTTCATGTTCTTAATTTCATGGAGAG	psaB	Photosystem I P700 chlorophyll A apoprotein A2 PsaB	WP_036919280.	225300..227528	Photosystem I	COG2885
6.1_WP_036919280.1_	CUST_8550_PI442182273	MIT0602	LLII/III	6.1_WP_036919280.1_	GGACGCAACTTACCTTGCAATGTTCTGGGCACTTAATACAATTGCTTGGATCACTTTCTA	psaB	Photosystem I P700 chlorophyll A apoprotein A2 PsaB	WP_036919280.	225300..227528	Photosystem I	COG2885
6.1_WP_036919280.1_	CUST_8551_PI442182273	MIT0602	LLII/III	6.1_WP_036919280.1_	CTCTCAATTGATTAATGGGTACAACCCATTTGGTGTTAATGCTCTATCCCCTTGGGCATG	psaB	Photosystem I P700 chlorophyll A apoprotein A2 PsaB	WP_036919280.	225300..227528	Photosystem I	COG2885
6.1_WP_036919280.1_	CUST_8553_PI442182273	MIT0602	LLII/III	6.1_WP_036919280.1_	CTGGCAAGGAAATGTTGCTCAGTTCAATGAATCTGGAACATACCTTATGGATGGTTCAG	psaB	Photosystem I P700 chlorophyll A apoprotein A2 PsaB	WP_036919280.	225300..227528	Photosystem I	COG2885
6.1_WP_036919278.1_	CUST_8554_PI442182273	MIT0602	LLII/III	6.1_WP_036919278.1_	AATCCCACTAGGAACTGCCGACTTGATGATCCATCACGTTCATGCTTTTACTATTCATGT	psaA	Photosystem I P700 chlorophyll A apoprotein A1 PsaA	WP_036919278.	227551..229872	Photosystem I	COG2885
6.1_WP_036919278.1_	CUST_8555_PI442182273	MIT0602	LLII/III	6.1_WP_036919278.1_	GGAAAATGCAGAGTGATGTATGGGGCCTTACTGGTGGAAACTTTGCTCAAAGTTCAATTA	psaA	Photosystem I P700 chlorophyll A apoprotein A1 PsaA	WP_036919278.	227551..229872	Photosystem I	COG2885
6.1_WP_036919278.1_	CUST_8556_PI442182273	MIT0602	LLII/III	6.1_WP_036919278.1_	AGCAGGTAACTTACCTGGAAGTGTCCTGAGCGAAGTTTTCAATGGAAATGTAGTTGAAGT	psaA	Photosystem I P700 chlorophyll A apoprotein A1 PsaA	WP_036919278.	227551..229872	Photosystem I	COG2885
6.1_WP_036919278.1_	CUST_8557_PI442182273	MIT0602	LLII/III	6.1_WP_036919278.1_	GCACATAATAAGTTAAAAGTTGCACCAACTATTCAGCCACGCGCTCTCTCAATCACTCAG	psaA	Photosystem I P700 chlorophyll A apoprotein A1 PsaA	WP_036919278.	227551..229872	Photosystem I	COG2885
6.1_WP_036919278.1_	CUST_8558_PI442182273	MIT0602	LLII/III	6.1_WP_036919278.1_	CAAGAAGCTCAAGACTAATTCCTGATAAAGCTCAACTTGGTTTCCGTTTCCCTTGCGATG	psaA	Photosystem I P700 chlorophyll A apoprotein A1 PsaA	WP_036919278.	227551..229872	Photosystem I	COG2885
6.1_WP_036919278.1_	CUST_8559_PI442182273	MIT0602	LLII/III	6.1_WP_036919278.1_	TCAGTTTAATGTTCCTATTCAGTGGCAGAGGCTACTGGCAGGAACTTTTTGAGTCCATAA	psaA	Photosystem I P700 chlorophyll A apoprotein A1 PsaA	WP_036919278.	227551..229872	Photosystem I	COG2885
6.1_WP_036919270.1_	CUST_8577_PI442182273	MIT0602	LLII/III	6.1_WP_036919270.1_	AATAGTTGTTGTACTTGGATCTACAGGTAGAAATGTTGGCGCTGGAATGACTGGTGGAAT	glsF	Ferredoxin-dependent glutamate synthase	WP_036919270.	233412..237971	Nitrogen Metabolism	COG0069
6.1_WP_036919270.1_	CUST_8578_PI442182273	MIT0602	LLII/III	6.1_WP_036919270.1_	GTAAGGAATAGCGGAGTAAAAGCAGTTGTAGAGGGCACTGGAGATCATTGCTGTGAATAC	glsF	Ferredoxin-dependent glutamate synthase	WP_036919270.	233412..237971	Nitrogen Metabolism	COG0069
6.1_WP_036919270.1_	CUST_8579_PI442182273	MIT0602	LLII/III	6.1_WP_036919270.1_	ACTGCAAAAAAGCTATTTAAGCTTTTAGTCCCCCCAAGCGAAAAAGAGAAGGTTGGCTTA	glsF	Ferredoxin-dependent glutamate synthase	WP_036919270.	233412..237971	Nitrogen Metabolism	COG0069
6.1_WP_036919270.1_	CUST_8580_PI442182273	MIT0602	LLII/III	6.1_WP_036919270.1_	AAGTTTCGGGGCATTTTTGTTACAGGGCATGCATATTCACTTAATCGGAGAAGCTAATGA	glsF	Ferredoxin-dependent glutamate synthase	WP_036919270.	233412..237971	Nitrogen Metabolism	COG0069
6.1_WP_036919270.1_	CUST_8581_PI442182273	MIT0602	LLII/III	6.1_WP_036919270.1_	GCTCAATCATATGGTAACAATGGTTTCCAAGGGAGTCTTGCAATAACTTTTACGGGAGCA	glsF	Ferredoxin-dependent glutamate synthase	WP_036919270.	233412..237971	Nitrogen Metabolism	COG0069
6.1_WP_036919270.1_	CUST_8582_PI442182273	MIT0602	LLII/III	6.1_WP_036919270.1_	CAAATCAAGTAATCCTAGGCAACACATGTTTATATGGGGAACGGGCGGCAAGTTATTTG	glsF	Ferredoxin-dependent glutamate synthase	WP_036919270.	233412..237971	Nitrogen Metabolism	COG0069
6.1_WP_036919763.1_	CUST_8842_PI442182273	MIT0602	LLII/III	6.1_WP_036919763.1_	TAGCTGTATCACTATTTAAAATAGTCAATGATCTGCGTCTCCTGGGGTCTGGACCACGAG	fumC	class II fumarate hydratase	WP_036919763.	293153..294547	Krebs cycle and related genes	COG0114
6.1_WP_036919763.1_	CUST_8840_PI442182273	MIT0602	LLII/III	6.1_WP_036919763.1_	TAAAGGAAGTATCAAAGAACTTTTTTCACTACCTCTTGGAGGGACGGCAATCGGAACAGG	fumC	class II fumarate hydratase	WP_036919763.	293153..294547	Krebs cycle and related genes	COG0114
6.1_WP_036919763.1_	CUST_8841_PI442182273	MIT0602	LLII/III	6.1_WP_036919763.1_	ATGCTCGTCACAAGTTTATCCCCAAAAATTGGTTATGAAAAAGCTAGTGAGATCGCCCAG	fumC	class II fumarate hydratase	WP_036919763.	293153..294547	Krebs cycle and related genes	COG0114
6.1_WP_036919763.1_	CUST_8843_PI442182273	MIT0602	LLII/III	6.1_WP_036919763.1_	TGCAGTAAGTATGGCTGGGAGTGGTGGACATCTACAAATGAACGCTTACAAACCTTTAAT	fumC	class II fumarate hydratase	WP_036919763.	293153..294547	Krebs cycle and related genes	COG0114
6.1_WP_036919763.1_	CUST_8844_PI442182273	MIT0602	LLII/III	6.1_WP_036919763.1_	ACCGGATTACCTTTTCAACCTGCTGTTAATAAGTTTGCCATTATGGCTAGTCATGATGGA	fumC	class II fumarate hydratase	WP_036919763.	293153..294547	Krebs cycle and related genes	COG0114
6.1_WP_036919763.1_	CUST_8845_PI442182273	MIT0602	LLII/III	6.1_WP_036919763.1_	TGCTCATCTACAGAACGTAAGTCTCAGAGAAGCATCAAAGCATTTAGGATATGTAAGCGA	fumC	class II fumarate hydratase	WP_036919763.	293153..294547	Krebs cycle and related genes	COG0114
6.1_WP_036919751.1_	CUST_8899_PI442182273	MIT0602	LLII/III	6.1_WP_036919751.1_	AAGCCTTGATTTTTGCAACTCTTGCTGCTTACTACATTGGTGAAGCAGTAGAAGAGCATC	atpI	ATP synthase subunit A	WP_036919751.	302577..303302	Energy Metabolism	COG0356
6.1_WP_036919751.1_	CUST_8900_PI442182273	MIT0602	LLII/III	6.1_WP_036919751.1_	TACTTCGAGTACTACGTTCATCCAACGCCAATCATGCTTCCGTTCAAGATTGTTGAAGAT	atpI	ATP synthase subunit A	WP_036919751.	302577..303302	Energy Metabolism	COG0356
6.1_WP_036919751.1_	CUST_8901_PI442182273	MIT0602	LLII/III	6.1_WP_036919751.1_	GGTGCATTGCTAACTCTTGTTGTTGTTGGCACTAAAAAGATGGAACGTGATCCTAAAGGA	atpI	ATP synthase subunit A	WP_036919751.	302577..303302	Energy Metabolism	COG0356
6.1_WP_036919751.1_	CUST_8902_PI442182273	MIT0602	LLII/III	6.1_WP_036919751.1_	ACTCTCTTTTTGTTCATTTTTGTGAGCAATTGGGGTGGGGCTTTAATCCCTTGGAAGTTA	atpI	ATP synthase subunit A	WP_036919751.	302577..303302	Energy Metabolism	COG0356
6.1_WP_036919751.1_	CUST_8903_PI442182273	MIT0602	LLII/III	6.1_WP_036919751.1_	CACCTACGGCGGATATAAATACAACCGTCGCATTGGCTCTTTTGGTTTCACTTTCATATT	atpI	ATP synthase subunit A	WP_036919751.	302577..303302	Energy Metabolism	COG0356
6.1_WP_036919751.1_	CUST_8904_PI442182273	MIT0602	LLII/III	6.1_WP_036919751.1_	TGCACTTGAAGTTGGTCAACATCTTTATTGGCAAATAGGAAACGTCAGAATTCATGGGCA	atpI	ATP synthase subunit A	WP_036919751.	302577..303302	Energy Metabolism	COG0356
6.1_WP_036919152.1_	CUST_8927_PI442182273	MIT0602	LLII/III	6.1_WP_036919152.1_	CCTTTAAACCTCGCAGAGCAAGTGGCAGTTGTATATGCAGGTGTAAAAGGTTTGATTGAT	atpA	ATP synthase alpha subunit	WP_036919152.	305455..306972	Energy Metabolism	COG0056
6.1_WP_036919152.1_	CUST_8928_PI442182273	MIT0602	LLII/III	6.1_WP_036919152.1_	GCTATTAATGTTGGTATCTCTGTTAGTCGAGTAGGAGGAGCCGCTCAAACAAAGGCAATT	atpA	ATP synthase alpha subunit	WP_036919152.	305455..306972	Energy Metabolism	COG0056
6.1_WP_036919152.1_	CUST_8929_PI442182273	MIT0602	LLII/III	6.1_WP_036919152.1_	CCAGGAGATGTTTTCTATTGTCACAGTCGTTTGCTTGAAAGAGCTGCAAAGCTTTCTGAT	atpA	ATP synthase alpha subunit	WP_036919152.	305455..306972	Energy Metabolism	COG0056
6.1_WP_036919152.1_	CUST_8930_PI442182273	MIT0602	LLII/III	6.1_WP_036919152.1_	AGTGGATCTATGACAGCTTTGCCGATAATTGAAACACAAGCAGGAGATGTCTCTGCTTAT	atpA	ATP synthase alpha subunit	WP_036919152.	305455..306972	Energy Metabolism	COG0056
6.1_WP_036919152.1_	CUST_8931_PI442182273	MIT0602	LLII/III	6.1_WP_036919152.1_	TATGACGATTTAACAAAACAGGCTCAGGCTTATCGCCAGATGTCTCTTCTTCTAAGACGC	atpA	ATP synthase alpha subunit	WP_036919152.	305455..306972	Energy Metabolism	COG0056
6.1_WP_036919152.1_	CUST_8932_PI442182273	MIT0602	LLII/III	6.1_WP_036919152.1_	TCACAGTTTGCCTCTGATTTAGATGAAGCTACTCAGCAACAACTTGAAAGAGGAAAGCGT	atpA	ATP synthase alpha subunit	WP_036919152.	305455..306972	Energy Metabolism	COG0056
6.1_WP_036919122.1_	CUST_8996_PI442182273	MIT0602	LLII/III	6.1_WP_036919122.1_	TTCCAAGCACTACTGGACTTCTAGGTATATTGCCCGGTCATATATCTATGGTTACGGCAA	atpC	ATP synthase epsilon subunit	WP_036919122.	317892..318299	Energy Metabolism	COG0355
6.1_WP_036919122.1_	CUST_8997_PI442182273	MIT0602	LLII/III	6.1_WP_036919122.1_	TGGGAATCTATTGCCCTAATGGGTGGATTCGCAGAAGTTGAATCTGATGATGTAACAGTT	atpC	ATP synthase epsilon subunit	WP_036919122.	317892..318299	Energy Metabolism	COG0355
6.1_WP_036919122.1_	CUST_8998_PI442182273	MIT0602	LLII/III	6.1_WP_036919122.1_	CACCCTCAGAGTACTTGCTCCAGACAAGAGTGTGTTTGACAACACAGTAGAGGAAGTAAT	atpC	ATP synthase epsilon subunit	WP_036919122.	317892..318299	Energy Metabolism	COG0355
6.1_WP_036919122.1_	CUST_8999_PI442182273	MIT0602	LLII/III	6.1_WP_036919122.1_	AGAGTTAGAGCAAGCTAAAAGCCAATTAAGCAAATTATCCGATCAGGCAAACTCTGCTGA	atpC	ATP synthase epsilon subunit	WP_036919122.	317892..318299	Energy Metabolism	COG0355
6.1_WP_036919122.1_	CUST_9000_PI442182273	MIT0602	LLII/III	6.1_WP_036919122.1_	AGATAAAAGCTAAAGAAAACTTGGACAAAGCAAGAGCAAGAGCACAAGCAGTATCCAACT	atpC	ATP synthase epsilon subunit	WP_036919122.	317892..318299	Energy Metabolism	COG0355
6.1_WP_036919122.1_	CUST_9001_PI442182273	MIT0602	LLII/III	6.1_WP_036919122.1_	AACAGTTTTAGTAAATTCAGCAGAACTAGGTAAAAGTATTAATTCCTCAGATG	atpC	ATP synthase epsilon subunit	WP_036919122.	317892..318299	Energy Metabolism	COG0355
9.1_WP_036919709.1_	CUST_9718_PI442182273	MIT0602	LLII/III	9.1_WP_036919709.1_	CAGCAGCTTTGGCCGGTACATCTTTATCAATTGATAGAAGAAGTACCGCAGAACAATTGG	argH	Argininosuccinate lyase	WP_036919709.	430..1818	Amino Acid metabolism	COG0165
9.1_WP_036919709.1_	CUST_9719_PI442182273	MIT0602	LLII/III	9.1_WP_036919709.1_	TATTCCACAAAATGTAGTTGCTTCTAGAGTCAGTGAGGGAGGTACAGGTTTTGAGCGAGT	argH	Argininosuccinate lyase	WP_036919709.	430..1818	Amino Acid metabolism	COG0165
9.1_WP_036919709.1_	CUST_9720_PI442182273	MIT0602	LLII/III	9.1_WP_036919709.1_	AGCCTAGATGCAGTTAGTGATAGGGATTTCGCTGTTGAGTTTACATCTGCTTCATCATTG	argH	Argininosuccinate lyase	WP_036919709.	430..1818	Amino Acid metabolism	COG0165
9.1_WP_036919709.1_	CUST_9721_PI442182273	MIT0602	LLII/III	9.1_WP_036919709.1_	GGAAGAGTGTTTGGGCATCTTCAAGCTCTCTTGACTATGATTAAAGGTCTTCCTTTAGCA	argH	Argininosuccinate lyase	WP_036919709.	430..1818	Amino Acid metabolism	COG0165
9.1_WP_036919709.1_	CUST_9722_PI442182273	MIT0602	LLII/III	9.1_WP_036919709.1_	ACGGATAGATGTTCTACAGGAAGTAGCTTGATGCCTCAAAAAAAGAATCCTGATGTACCT	argH	Argininosuccinate lyase	WP_036919709.	430..1818	Amino Acid metabolism	COG0165
9.1_WP_036919709.1_	CUST_9723_PI442182273	MIT0602	LLII/III	9.1_WP_036919709.1_	ATGAAAGTTTGAGTAAAGCTGTTGAAGCGGATTTTTCAAATGCTACTGATGTAGCAGATT	argH	Argininosuccinate lyase	WP_036919709.	430..1818	Amino Acid metabolism	COG0165
WP_002807363.1_34	CUST_143_PI442190825	MIT9292		WP_002807363.1_34	ACCATCTCTCATTCTAAAAAGTCCATTGAATATCCAGCTGGCGGTGGATCTATTATCGCG	rbsK	Sugar kinase, ribokinase family	WP_002807363.	21267..22133	Pentose Phosphate	COG0524
WP_002807363.1_34	CUST_144_PI442190825	MIT9292		WP_002807363.1_34	TGTGGCTTGGCGTGATAAACCAACGAGAAGAGGATTTAGTTTAATTGACTCTCAAGGTGA	rbsK	Sugar kinase, ribokinase family	WP_002807363.	21267..22133	Pentose Phosphate	COG0524
WP_002807363.1_34	CUST_145_PI442190825	MIT9292		WP_002807363.1_34	TCTTATGGATGTGGCGATTCTTTTGCTGCTGGGATTCTTTATGGAATGGCATCTAAATGG	rbsK	Sugar kinase, ribokinase family	WP_002807363.	21267..22133	Pentose Phosphate	COG0524
WP_002807363.1_34	CUST_146_PI442190825	MIT9292		WP_002807363.1_34	AACTGAGGGAGAGAAGGGAGGCATAATATTTCCTGGTGGAAGATATAAGGCTCTAAAAAA	rbsK	Sugar kinase, ribokinase family	WP_002807363.	21267..22133	Pentose Phosphate	COG0524
WP_002807363.1_34	CUST_147_PI442190825	MIT9292		WP_002807363.1_34	TCAGAAATTTTTAAAATTGCTAGATCAGCTTCAATTCTATGCACAACACCAAGGGTGGGA	rbsK	Sugar kinase, ribokinase family	WP_002807363.	21267..22133	Pentose Phosphate	COG0524
WP_002807363.1_34	CUST_148_PI442190825	MIT9292		WP_002807363.1_34	AGTTTAAATCTTGCTAAAGTAATGGGAAGAGACGCCAGTGAATTTTTTGGTCCATATGCA	rbsK	Sugar kinase, ribokinase family	WP_002807363.	21267..22133	Pentose Phosphate	COG0524
WP_002805121.1_63	CUST_307_PI442190825	MIT9292		WP_002805121.1_63	TAATACTTGGTTGTGAATCAGTGGAATGTATGAGACTTGAAGCAGGGGGAATGGCCAATA	opcA	Glucose-6-P dehydrogenase subunit opcA	WP_002805121.	49774..51075	Pentose Phosphate	COG3429
WP_002805121.1_63	CUST_308_PI442190825	MIT9292		WP_002805121.1_63	ATGAATTACCTTCTTGGTTGTGGTGGAATGGTAGCTTGGATGAATCCCCTGAAATTTTCG	opcA	Glucose-6-P dehydrogenase subunit opcA	WP_002805121.	49774..51075	Pentose Phosphate	COG3429
WP_002805121.1_63	CUST_309_PI442190825	MIT9292		WP_002805121.1_63	TAGGTTGAAAAATTGGAGGGAATCATTGGCAATGATTTTTGATCCACCTTCGAGGAGACC	opcA	Glucose-6-P dehydrogenase subunit opcA	WP_002805121.	49774..51075	Pentose Phosphate	COG3429
WP_002805121.1_63	CUST_310_PI442190825	MIT9292		WP_002805121.1_63	TCCTTTGTCTTTGGGAAATCCCAGTATTCATTTAGGACAAGTTATTGGATTGAGGTTGAT	opcA	Glucose-6-P dehydrogenase subunit opcA	WP_002805121.	49774..51075	Pentose Phosphate	COG3429
WP_002805121.1_63	CUST_311_PI442190825	MIT9292		WP_002805121.1_63	GCAGGAGATCATATTATTCAAGCGTTGTTTTTGATCTCATGGATTAGCGATAAACTTGGT	opcA	Glucose-6-P dehydrogenase subunit opcA	WP_002805121.	49774..51075	Pentose Phosphate	COG3429
WP_002805121.1_63	CUST_312_PI442190825	MIT9292		WP_002805121.1_63	TTCACGAAATATGGTTTAAGGTTAATAATTGATACTGCTCTTGGATCTCCTAACAGATGT	opcA	Glucose-6-P dehydrogenase subunit opcA	WP_002805121.	49774..51075	Pentose Phosphate	COG3429
WP_002805176.1_64	CUST_313_PI442190825	MIT9292		WP_002805176.1_64	CAGAAACTTATATTGCGACAAAAGTTTTCGTAGATAACTGGCGTTGGCAAGGGGTTCCTT	zwf	glucose-6-phosphate dehydrogenase zwf	WP_002805176.	51078..52601	Pentose Phosphate	COG0364
WP_002805176.1_64	CUST_314_PI442190825	MIT9292		WP_002805176.1_64	ACCCTCAGATGAAGGCTATGTAAGATTATTAGCTGATGCAATGCTTTCTGACCCAACCTT	zwf	glucose-6-phosphate dehydrogenase zwf	WP_002805176.	51078..52601	Pentose Phosphate	COG0364
WP_002805176.1_64	CUST_315_PI442190825	MIT9292		WP_002805176.1_64	GACTCAAATGCTTGCTGTTACTGCTATGGAACCTCCTGGAAAATTTGAACCAGAAGCAAT	zwf	glucose-6-phosphate dehydrogenase zwf	WP_002805176.	51078..52601	Pentose Phosphate	COG0364
WP_002805176.1_64	CUST_316_PI442190825	MIT9292		WP_002805176.1_64	TGGAGAGGGAGGAAATATCTCAAATCGACTCAAAGGGTATAGACAGGAAGAGGGTGTTAA	zwf	glucose-6-phosphate dehydrogenase zwf	WP_002805176.	51078..52601	Pentose Phosphate	COG0364
WP_002805176.1_64	CUST_317_PI442190825	MIT9292		WP_002805176.1_64	TCCCCTTGGAAGTTACCTATTTATAACTATGAATCTATGACGTGGGGACCTCCTGAGTCT	zwf	glucose-6-phosphate dehydrogenase zwf	WP_002805176.	51078..52601	Pentose Phosphate	COG0364
WP_002805176.1_64	CUST_318_PI442190825	MIT9292		WP_002805176.1_64	AGGTGCTACTTTCAAATTTGAGGTAAAATCTCCTGGTTCTGGAATGAAATCCAGACCTGT	zwf	glucose-6-phosphate dehydrogenase zwf	WP_002805176.	51078..52601	Pentose Phosphate	COG0364
WP_002808097.1_262	CUST_1306_PI442190825	MIT9292		WP_002808097.1_262	AAAAGTACCTTTTGAAATAGTTGATCGAAGACCAGGAGATGCTGCTAGTGTAGTTGCGGA	galE	UDP-glucose 4-epimerase	WP_002808097.	232268..233320	Galactose metabolism	COG1087
WP_002808097.1_262	CUST_1307_PI442190825	MIT9292		WP_002808097.1_262	AGGTATTTTAATCCTATTGGAGCTCATTGCTCTGGTTTAATTGGAGAAAACCCAGTGGGT	galE	UDP-glucose 4-epimerase	WP_002808097.	232268..233320	Galactose metabolism	COG1087
WP_002808097.1_262	CUST_1308_PI442190825	MIT9292		WP_002808097.1_262	TGGCAATGATTGGCCTACTATTGATGGCACTGGAGTTAGGGATTATATACATGTTATGGA	galE	UDP-glucose 4-epimerase	WP_002808097.	232268..233320	Galactose metabolism	COG1087
WP_002808097.1_262	CUST_1309_PI442190825	MIT9292		WP_002808097.1_262	AATTGGTTGCCAAAACTTAGTTTAGAAGAAATGTGTAGGGATGGATGGAATTGGAAAATT	galE	UDP-glucose 4-epimerase	WP_002808097.	232268..233320	Galactose metabolism	COG1087
WP_002808097.1_262	CUST_1310_PI442190825	MIT9292		WP_002808097.1_262	AGCAGTAGTGCTACTTTATATGGCCAATCAAGTAAAGAGCTACTTAATGAAGAAACAGAA	galE	UDP-glucose 4-epimerase	WP_002808097.	232268..233320	Galactose metabolism	COG1087
WP_002808097.1_262	CUST_1311_PI442190825	MIT9292		WP_002808097.1_262	AGCCTATTAATCCTTATGGGACTTCAAAGTATGGGATTGAACTTTTTCTTGATGATGTTT	galE	UDP-glucose 4-epimerase	WP_002808097.	232268..233320	Galactose metabolism	COG1087
WP_002807742.1_300	CUST_1534_PI442190825	MIT9292		WP_002807742.1_300	TTGGAACTGATAAAAGAATCGGAAGTGAATTTTTAAATCCAGGGCCTGGCTTTGGTGGAA	ugd	UDP-glucose 6-dehydrogenase/nucleotide sugar dehydrogenase	WP_002807742.	278815..280206	Amino sugar and nucleotide sugar r	COG1004
WP_002807742.1_300	CUST_1535_PI442190825	MIT9292		WP_002807742.1_300	GCAATAAAAGCAGCAGAAGGGGCTGATGCGGTTCTTGTTTTAACAGAATGGAAAGAATTT	ugd	UDP-glucose 6-dehydrogenase/nucleotide sugar dehydrogenase	WP_002807742.	278815..280206	Amino sugar and nucleotide sugar r	COG1004
WP_002807742.1_300	CUST_1536_PI442190825	MIT9292		WP_002807742.1_300	AATTCCATCTCTGCAATTTGCGAGTCTACTGGTGCAAATATAAGTGAAGTTGCTAAGGCT	ugd	UDP-glucose 6-dehydrogenase/nucleotide sugar dehydrogenase	WP_002807742.	278815..280206	Amino sugar and nucleotide sugar r	COG1004
WP_002807742.1_300	CUST_1537_PI442190825	MIT9292		WP_002807742.1_300	TGGATTAAAGGAAGTCGCAGACTACTGGGAAAAGGTCGTTAGTATTAATTTATGGCAACA	ugd	UDP-glucose 6-dehydrogenase/nucleotide sugar dehydrogenase	WP_002807742.	278815..280206	Amino sugar and nucleotide sugar r	COG1004
WP_002807742.1_300	CUST_1538_PI442190825	MIT9292		WP_002807742.1_300	GCCTTGGATTGGAAAAATATTTTTCAAGTAATGAGAAAACCTGCTTGGATTTTTGATGCA	ugd	UDP-glucose 6-dehydrogenase/nucleotide sugar dehydrogenase	WP_002807742.	278815..280206	Amino sugar and nucleotide sugar r	COG1004
WP_002807742.1_300	CUST_1539_PI442190825	MIT9292		WP_002807742.1_300	TTAGCAATATTTGGATTCTCTTTTAAGGCAAATACAAATGATACTAGGGAATCGCCAAGT	ugd	UDP-glucose 6-dehydrogenase/nucleotide sugar dehydrogenase	WP_002807742.	278815..280206	Amino sugar and nucleotide sugar r	COG1004
WP_002805940.1_326	CUST_1683_PI442190825	MIT9292		WP_002805940.1_326	GGACATTCTCTTGCAGATCCAGATGAATTAAGGTCTGAGAAAGAGAAGGAGTTTTGGGGG	pdhA	Pyruvate dehydrogenase E1 component alpha subunit	WP_002805940.	304017..305090	Glycolysis/Krebs cycle	COG1071
WP_002805940.1_326	CUST_1684_PI442190825	MIT9292		WP_002805940.1_326	ACTGCAGCATTCTTTGGGGATGGGACTTGCAATAATGGGCAGTTTTTTGAATGTTTAAAT	pdhA	Pyruvate dehydrogenase E1 component alpha subunit	WP_002805940.	304017..305090	Glycolysis/Krebs cycle	COG1071
WP_002805940.1_326	CUST_1685_PI442190825	MIT9292		WP_002805940.1_326	GCATCACTTACTAGGAGGATATGCATTTATTGGAGAGGGGATTCCAGTTGCTTTAGGAGC	pdhA	Pyruvate dehydrogenase E1 component alpha subunit	WP_002805940.	304017..305090	Glycolysis/Krebs cycle	COG1071
WP_002805940.1_326	CUST_1686_PI442190825	MIT9292		WP_002805940.1_326	CACAAAGAGCAATAGAACGTGCTAGAGCAGGAGAAGGTCCGACACTTTTAGAATGTTTGA	pdhA	Pyruvate dehydrogenase E1 component alpha subunit	WP_002805940.	304017..305090	Glycolysis/Krebs cycle	COG1071
WP_002805940.1_326	CUST_1687_PI442190825	MIT9292		WP_002805940.1_326	TACAGAAATATCGGAGTCAGTTAAAAATGCTATTGAAGCACCTGAACCCCCATCGCAAGA	pdhA	Pyruvate dehydrogenase E1 component alpha subunit	WP_002805940.	304017..305090	Glycolysis/Krebs cycle	COG1071
WP_002805940.1_326	CUST_1688_PI442190825	MIT9292		WP_002805940.1_326	TAAATGGGCTATTGGTATGGCTCACGATAGAGCTACTAGCAATCCTGAGATCTGGAGAAA	pdhA	Pyruvate dehydrogenase E1 component alpha subunit	WP_002805940.	304017..305090	Glycolysis/Krebs cycle	COG1071
WP_002805227.1_347	CUST_1807_PI442190825	MIT9292		WP_002805227.1_347	TTGTTGATCAAGAAGCAAATATAATAGTTGGTGCAGTGGTTGATGAGGCAATGGAAGGGG	ftsZ	cell division protein FtsZ	WP_002805227.	327231..328340	Cell Division	COG0206
WP_002805227.1_347	CUST_1808_PI442190825	MIT9292		WP_002805227.1_347	TAGAGGCAGCACAGGCAGCAATGAATAGTCCTTTATTAGAAGCAGCTAGAATTGATGGAG	ftsZ	cell division protein FtsZ	WP_002805227.	327231..328340	Cell Division	COG0206
WP_002805227.1_347	CUST_1809_PI442190825	MIT9292		WP_002805227.1_347	ACGTAATAGCAGGAGCCCCTCTTCAAGAAGCATTTAGGAATGCTGATGATGTTTTAGGA	ftsZ	cell division protein FtsZ	WP_002805227.	327231..328340	Cell Division	COG0206
WP_002805227.1_347	CUST_1810_PI442190825	MIT9292		WP_002805227.1_347	TGCGTCAAGCAGAAGAAGGAATCGCAAGACTAGCTGAGAATGTAGATACTCTTATCGTCA	ftsZ	cell division protein FtsZ	WP_002805227.	327231..328340	Cell Division	COG0206
WP_002805227.1_347	CUST_1811_PI442190825	MIT9292		WP_002805227.1_347	GCTCCGGTAGTTGCAGAAGTGGCTAAACAAAGTGGTGCTTTAACAGTAGGTATAGTAACC	ftsZ	cell division protein FtsZ	WP_002805227.	327231..328340	Cell Division	COG0206
WP_002805227.1_347	CUST_1812_PI442190825	MIT9292		WP_002805227.1_347	GGCCGAAGAATCAAAAGATGAGCTTCAACAAGCGTTAGAGGGATCTGACCTAGTCTTTAT	ftsZ	cell division protein FtsZ	WP_002805227.	327231..328340	Cell Division	COG0206
WP_002805743.1_363	CUST_1888_PI442190825	MIT9292		WP_002805743.1_363	TAGCTCCATTCGCAAAAAAAGCAAGAAGGCCTTCAGGAAAAGTTGAAGCTATCACTCTTC	glcH	GPH family sugar transporter	WP_002805743.	340621..341967	GlcH sugar transporter	COG2211
WP_002805743.1_363	CUST_1889_PI442190825	MIT9292		WP_002805743.1_363	TTCTTCTCTGGTGCGCCTTACAATTAATGCAAACAGTGGCGTTAATTTATGTCGAGGATG	glcH	GPH family sugar transporter	WP_002805743.	340621..341967	GlcH sugar transporter	COG2211
WP_002805743.1_363	CUST_1890_PI442190825	MIT9292		WP_002805743.1_363	TACTGAACGTACCAACATATATTGCGAAGTGGATCCCGATACCTTTCCAAATTAGCGCTT	glcH	GPH family sugar transporter	WP_002805743.	340621..341967	GlcH sugar transporter	COG2211
WP_002805743.1_363	CUST_1891_PI442190825	MIT9292		WP_002805743.1_363	TACTTCCTGATGCAATAGACGAAGACCCAGAGAAACCAGCAGGATTATATACTGCTTGGA	glcH	GPH family sugar transporter	WP_002805743.	340621..341967	GlcH sugar transporter	COG2211
WP_002805743.1_363	CUST_1892_PI442190825	MIT9292		WP_002805743.1_363	TTTTCATAATCTGTCTTATTGGCATTGGAGCTTCAACCGCTTTTCTTATCCCTTGGTCAC	glcH	GPH family sugar transporter	WP_002805743.	340621..341967	GlcH sugar transporter	COG2211
WP_002805743.1_363	CUST_1893_PI442190825	MIT9292		WP_002805743.1_363	ACAGGATTTCAGCGTTAAACTATGGAGCGATTATGTGGATTATTTCATGTACAGCAGCTT	glcH	GPH family sugar transporter	WP_002805743.	340621..341967	GlcH sugar transporter	COG2211
WP_002807011.1_382	CUST_2001_PI442190825	MIT9292		WP_002807011.1_382	GCCAGAGGTGTAAGCTTGAATGCATTTAGACAGTTCGTAATAGCCGTAACGGGTTATACA	kaiC	circadian clock protein KaiC	WP_002807011.	359736..361274	Circadian Rhythm	COG0467

WP_002807011.1_382	CUST_2002_PI442190825	MIT9292		WP_002807011.1_382	AGATTCCATCATTCTCGCTACTGGAGCTACTGGTACAGGCAAAACAATGCTTGTATCAAA	kaiC	circadian clock protein KaiC	WP_002807011.:	359736..361274	Circadian Rhythm	COG0467
WP_002807011.1_382	CUST_2003_PI442190825	MIT9292		WP_002807011.1_382	TGAGATGGCACGAGCTTTAAATGTCTTTAAAATGCGGGGATCATGGCATGATAAACGAAT	kaiC	circadian clock protein KaiC	WP_002807011.:	359736..361274	Circadian Rhythm	COG0467
WP_002807011.1_382	CUST_2004_PI442190825	MIT9292		WP_002807011.1_382	ACTTTTTGCATATGAAGAATCTAGGGCTCAATTGCTTCGGAATGCTACTAGTTGGGGTAT	kaiC	circadian clock protein KaiC	WP_002807011.:	359736..361274	Circadian Rhythm	COG0467
WP_002807011.1_382	CUST_2005_PI442190825	MIT9292		WP_002807011.1_382	TGAACAAATATTTAGTGGTGCTCCTCACAGAGTTGTGCCTGATCAAAATGTTCAAAATGT	kaiC	circadian clock protein KaiC	WP_002807011.:	359736..361274	Circadian Rhythm	COG0467
WP_002807011.1_382	CUST_2006_PI442190825	MIT9292		WP_002807011.1_382	AAAAACGCAAATTACTCAATTTAAACCAAAAAGGATGGCAATTGATTCTCTCTCTGCGTT	kaiC	circadian clock protein KaiC	WP_002807011.:	359736..361274	Circadian Rhythm	COG0467
WP_002807968.1_383	CUST_2007_PI442190825	MIT9292		WP_002807968.1_383	TGGTTTAGATCTACTTTTTGATGAATTAACTGAAACTGAATATAGTGGAGATAAATAA	kaiB	Circadian clock protein KaiB	WP_002807968.:	361347..361664	Circadian Rhythm	COG4251
WP_002807968.1_383	CUST_2008_PI442190825	MIT9292		WP_002807968.1_383	TGCCGCCACCAGTGAGAAAAATAATTGGTGATCTCTCAGATAGAGAGAAAGTT	kaiB	Circadian clock protein KaiB	WP_002807968.:	361347..361664	Circadian Rhythm	COG4251
WP_002807968.1_383	CUST_2009_PI442190825	MIT9292		WP_002807968.1_383	ACAACTTGCAGAAGAAGATAAGATTTTAGCAACCCCAACTTTAGCCAAAATTTTGCCGCC	kaiB	Circadian clock protein KaiB	WP_002807968.:	361347..361664	Circadian Rhythm	COG4251
WP_002807968.1_383	CUST_2010_PI442190825	MIT9292		WP_002807968.1_383	TTTAGAAAATGAATTCAAAGGAGTTTATGCTTTGAAGGTTATTGATGTACTTAAGCAGCC	kaiB	Circadian clock protein KaiB	WP_002807968.:	361347..361664	Circadian Rhythm	COG4251
WP_002807968.1_383	CUST_2011_PI442190825	MIT9292		WP_002807968.1_383	TGGCAGCAAGAAAAACATATATTTTAAAACTCTATGTTGCTGGAAATACTCCTAATTCAA	kaiB	Circadian clock protein KaiB	WP_002807968.:	361347..361664	Circadian Rhythm	COG4251
WP_033829829.1_455	CUST_2341_PI442190825	MIT9292		WP_033829829.1_455	AAATGGTAATAGAGGTTGGGCAACAGTAAAACCCGCTCCTGATCCAAAAGTTAATGATGC	ctaC/coxB	Cytochrome c oxidase subunit II	WP_033829829.:	421479..422414	Respiration	COG1622
WP_033829829.1_455	CUST_2342_PI442190825	MIT9292		WP_033829829.1_455	AGAGCCCCCAAGTATGATGAAAGTAATGGGGAACCTTTAGAGGGCAATCTAAAACTTGAA	ctaC/coxB	Cytochrome c oxidase subunit II	WP_033829829.:	421479..422414	Respiration	COG1622
WP_033829829.1_455	CUST_2343_PI442190825	MIT9292		WP_033829829.1_455	TGGCGCAGAAAGTCCCTTTTGTTGAGGAGAAAAAAGATTTTGATTATGGACCAATTGACAT	ctaC/coxB	Cytochrome c oxidase subunit II	WP_033829829.:	421479..422414	Respiration	COG1622
WP_033829829.1_455	CUST_2344_PI442190825	MIT9292		WP_033829829.1_455	TCTCTAACACCAACTAAAGAGGGAGTATTTAGATTAAGAGATGCAATGTTTAGTGGCGCA	ctaC/coxB	Cytochrome c oxidase subunit II	WP_033829829.:	421479..422414	Respiration	COG1622
WP_033829829.1_455	CUST_2345_PI442190825	MIT9292		WP_033829829.1_455	TGTAAAAAAAGAACTTCAAAATGGATTAAATCCTGCTGGCAGTTTGTACCAGAAAAGGCT	ctaC/coxB	Cytochrome c oxidase subunit II	WP_033829829.:	421479..422414	Respiration	COG1622
WP_033829829.1_455	CUST_2346_PI442190825	MIT9292		WP_033829829.1_455	TTCGAGGCAATGGAACTGGGAGTTTGTTTATCCAAATGGAATACATAGTTCTGAATTACA	ctaC/coxB	Cytochrome c oxidase subunit II	WP_033829829.:	421479..422414	Respiration	COG1622
WP_002807504.1_456	CUST_2347_PI442190825	MIT9292		WP_002807504.1_456	GAATGGCTACTTCCCTCTCCACCTCCTCACGAAAATTTTGAAGAAGATATCCCAACTGTT	cyoB/coxA	Cytochrome c oxidase subunit I	WP_002807504.:	422423..424114	Respiration	COG0843
WP_002807504.1_456	CUST_2348_PI442190825	MIT9292		WP_002807504.1_456	TCATTGGGCTGGTTTATTGGGAATGCCTAGAAGAGTTGCATCATATGATCCTGAATTTGC	cyoB/coxA	Cytochrome c oxidase subunit I	WP_002807504.:	422423..424114	Respiration	COG0843
WP_002807504.1_456	CUST_2349_PI442190825	MIT9292		WP_002807504.1_456	ATGCTCATTGCTGTTCCAACAGGTGTAAAAGTATTTGCATGGATAGCTACATTATGGGGC	cyoB/coxA	Cytochrome c oxidase subunit I	WP_002807504.:	422423..424114	Respiration	COG0843
WP_002807504.1_456	CUST_2350_PI442190825	MIT9292		WP_002807504.1_456	TTGAATATGGTAAGTTCTTGGGTCAGAGGAAAATCAGCTCCACCTAATCCTTGGAAAGCT	cyoB/coxA	Cytochrome c oxidase subunit I	WP_002807504.:	422423..424114	Respiration	COG0843
WP_002807504.1_456	CUST_2351_PI442190825	MIT9292		WP_002807504.1_456	ACCATTGCTTTTGGAATATTTGCAGCTATTTATCACTGGTTCCCAAAATTCACAGGAAAG	cyoB/coxA	Cytochrome c oxidase subunit I	WP_002807504.:	422423..424114	Respiration	COG0843
WP_002807504.1_456	CUST_2352_PI442190825	MIT9292		WP_002807504.1_456	TGGAATGTAATAGCGAGTATTGGAGCTTTTATTCTTGGCGTTGCCTCTATTCCTTTTATC	cyoB/coxA	Cytochrome c oxidase subunit I	WP_002807504.:	422423..424114	Respiration	COG0843
WP_012008309.1_457	CUST_2353_PI442190825	MIT9292		WP_012008309.1_457	TACGTTGAGTGGTCTGAACTCTCTTTCAGCTTACAAAGTGGAGCATTTGGAGGAATGTTT	cyoC	heme-copper oxidase subunit III	WP_012008309.:	424118..424714	Respiration	COG1845
WP_012008309.1_457	CUST_2354_PI442190825	MIT9292		WP_012008309.1_457	TCTCCCAGATAATTATAAAGGCGGAGAGATGGGTGTTGAATCAGTAAGCCTTTTTTGGCA	cyoC	heme-copper oxidase subunit III	WP_012008309.:	424118..424714	Respiration	COG1845
WP_012008309.1_457	CUST_2355_PI442190825	MIT9292		WP_012008309.1_457	TTTTTGCTTTTTTTGTAGGTTACAGCGTCCTTAAAATTAGTGCACCTGTCTGGTACCCCG	cyoC	heme-copper oxidase subunit III	WP_012008309.:	424118..424714	Respiration	COG1845
WP_012008309.1_457	CUST_2356_PI442190825	MIT9292		WP_012008309.1_457	ACTTACTGGTTTCCATGGCCTACACGTAATTACAGGGATATTACTAATGGGGCTTATGTT	cyoC	heme-copper oxidase subunit III	WP_012008309.:	424118..424714	Respiration	COG1845
WP_012008309.1_457	CUST_2357_PI442190825	MIT9292		WP_012008309.1_457	TGGGGTTTCAGAATTATTTGGTTCATAACCATGTTGATGGGAAGTTACTTTGTTTATGGT	cyoC	heme-copper oxidase subunit III	WP_012008309.:	424118..424714	Respiration	COG1845
WP_012008309.1_457	CUST_2358_PI442190825	MIT9292		WP_012008309.1_457	GCGCACAGAAAATCAGAATTTAACTTTAAATCATAAGCCAGGTCACATAAAACATGACGG	cyoC	heme-copper oxidase subunit III	WP_012008309.:	424118..424714	Respiration	COG1845
WP_002806495.1_488	CUST_2488_PI442190825	MIT9292		WP_002806495.1_488	ATCCGGAGAAAATTAAAGCCTTAAAACAAGTTTCGAAAGCTGAGGCTAGGATTCAAGCCG	atpC	ATP synthase epsilon subunit	WP_002806495.:	447454..447858	Energy Metabolism	COG0355
WP_002806495.1_488	CUST_2489_PI442190825	MIT9292		WP_002806495.1_488	TGCTGAAATTGGTTCTGAAATTAATGTTCAAAATGCCGAACAAGATCTTAAAGAAGCAAA	atpC	ATP synthase epsilon subunit	WP_002806495.:	447454..447858	Energy Metabolism	COG0355
WP_002806495.1_488	CUST_2490_PI442190825	MIT9292		WP_002806495.1_488	TGGCGTTTTAAGGCTAAGAATGAATTCCCAATGGAAATCAATAGCATTAATGGGAGGCTT	atpC	ATP synthase epsilon subunit	WP_002806495.:	447454..447858	Energy Metabolism	COG0355
WP_002806495.1_488	CUST_2491_PI442190825	MIT9292		WP_002806495.1_488	AAATGTTTATCAAGGCGAAGCTGAAGAAGTAATCCTTCCTAGTACCACTGGTCAGCTTGG	atpC	ATP synthase epsilon subunit	WP_002806495.:	447454..447858	Energy Metabolism	COG0355
WP_002805160.1_499	CUST_2547_PI442190825	MIT9292		WP_002805160.1_499	CAAAACTTTCTGATGCAATGGGAGGGGGTTCTATGACAGCTCTTCCAATCATTGAAACTC	atpA	ATP synthase alpha subunit	WP_002805160.:	457142..458659	Energy Metabolism	COG0056
WP_002805160.1_499	CUST_2548_PI442190825	MIT9292		WP_002805160.1_499	TGAAGCAACTCAGCAACAACTTGAAAGAGGCAAAAGACTAAGGGAGTTATTGAAGCAACC	atpA	ATP synthase alpha subunit	WP_002805160.:	457142..458659	Energy Metabolism	COG0056
WP_002805160.1_499	CUST_2549_PI442190825	MIT9292		WP_002805160.1_499	ACAAGTTGCAGTTGTTTATGCAGGAGTTAAAGGTCTTATTGATGAGGTTCCTGTGGAGGA	atpA	ATP synthase alpha subunit	WP_002805160.:	457142..458659	Energy Metabolism	COG0056
WP_002805160.1_499	CUST_2551_PI442190825	MIT9292		WP_002805160.1_499	TGTCTCTTCTTTTAAAAAGACCACCAGGAAGAGAGGCTTATCCTGGAGACGTGTTCTACT	atpA	ATP synthase alpha subunit	WP_002805160.:	457142..458659	Energy Metabolism	COG0056
WP_002805160.1_499	CUST_2552_PI442190825	MIT9292		WP_002805160.1_499	GCAGGGGACGTTTCGGCTTACATTCCAACTAACGTTATTTCAATTACGGATGGACAAATA	atpA	ATP synthase alpha subunit	WP_002805160.:	457142..458659	Energy Metabolism	COG0056
WP_002807601.1_504	CUST_2575_PI442190825	MIT9292		WP_002807601.1_504	GGGAGGAGCTTTAATTCCTTGGAGATTGATTAAGCTACCAAGTGGAGAATTAGGAGCACC	atpI	ATP synthase subunit A	WP_002807601.:	460682..461407	Energy Metabolism	COG0356
WP_002807601.1_504	CUST_2576_PI442190825	MIT9292		WP_002807601.1_504	ACTTGCTTGAGTTCCTCTGGGATTACATAAGAGATCTTGCAAGGACGCAAATAGGTGAAA	atpI	ATP synthase subunit A	WP_002807601.:	460682..461407	Energy Metabolism	COG0356
WP_002807601.1_504	CUST_2577_PI442190825	MIT9292		WP_002807601.1_504	GCATTGATTTTCGCAACTTTAGCGGCCTACTACATTGGAGAAGCTGTTGAAGAACATCAT	atpI	ATP synthase subunit A	WP_002807601.:	460682..461407	Energy Metabolism	COG0356
WP_002807601.1_504	CUST_2578_PI442190825	MIT9292		WP_002807601.1_504	TGCAGGCTTGAGCAATAAAGGTTGGAGATACTTCGAATACTATGTTCACCCAACTCCTAT	atpI	ATP synthase subunit A	WP_002807601.:	460682..461407	Energy Metabolism	COG0356
WP_002807601.1_504	CUST_2579_PI442190825	MIT9292		WP_002807601.1_504	GCTGATGAACTTGTCGTTGGTGTTTTAGTCTTTTTAGTTCCGCTAATCCTCCCAATACCT	atpI	ATP synthase subunit A	WP_002807601.:	460682..461407	Energy Metabolism	COG0356
WP_002807601.1_504	CUST_2580_PI442190825	MIT9292		WP_002807601.1_504	AGCATTAGAAGTTGGTCAACATCTTTATTGGCAAGTTGGAAATATCAGACTTCATGGGCA	atpI	ATP synthase subunit A	WP_002807601.:	460682..461407	Energy Metabolism	COG0356
WP_002805961.1_514	CUST_2631_PI442190825	MIT9292		WP_002805961.1_514	TGGCCTCGCATGATCGTCTAGCACAAGTAATGAGTCAGATAAAAATATTAGCAGGTGCAT	fumC	class II fumarate hydratase	WP_002805961.:	469082..470467	Krebs cycle and related genes	COG0114
WP_002805961.1_514	CUST_2632_PI442190825	MIT9292		WP_002805961.1_514	CGACTCAATGCGAAGCCTTATCAATGGTTTGCACTCAGATAATGGGTCTTGAATATGCAG	fumC	class II fumarate hydratase	WP_002805961.:	469082..470467	Krebs cycle and related genes	COG0114
WP_002805961.1_514	CUST_2633_PI442190825	MIT9292		WP_002805961.1_514	TTGGTTACTGCCATAGTGCCAAAAGTTGGTTATGAAAAAGCAGCTGAAATTGCAAACCTT	fumC	class II fumarate hydratase	WP_002805961.:	469082..470467	Krebs cycle and related genes	COG0114
WP_002805961.1_514	CUST_2634_PI442190825	MIT9292		WP_002805961.1_514	ACCTCTAGGAGGAACTGCAGTTGGTACAGGGATTAATTGTCCAAAAGGATTTTGTGAAGA	fumC	class II fumarate hydratase	WP_002805961.:	469082..470467	Krebs cycle and related genes	COG0114
WP_002805961.1_514	CUST_2635_PI442190825	MIT9292		WP_002805961.1_514	TCAGGAATATATGAATTAATTATCCCTCAAAATGAACCTGGAAGTTCTATCATGCCGGGT	fumC	class II fumarate hydratase	WP_002805961.:	469082..470467	Krebs cycle and related genes	COG0114
WP_002805961.1_514	CUST_2636_PI442190825	MIT9292		WP_002805961.1_514	TCAATAGCAAACTCTAGCGGCACTTTACAGATGAATGAATATAAGCCTCTTATTGGATTT	fumC	class II fumarate hydratase	WP_002805961.:	469082..470467	Krebs cycle and related genes	COG0114
WP_002807955.1_559	CUST_2877_PI442190825	MIT9292		WP_002807955.1_559	TTATATGCCTTGGGTAAATCAGGTGAAAGGTTTGCCGTAAGAAATAGTGGAGCAGTAGCT	glsF	Ferredoxin-dependent glutamate synthase	WP_002807955.:	518773..523347	Nitrogen Metabolism	COG0069
WP_002807955.1_559	CUST_2878_PI442190825	MIT9292		WP_002807955.1_559	CAGGGGGAATAATTGTAATTCTTGGCTCTACAGGAAGGAATATCGGAGCAGGAATGACTG	glsF	Ferredoxin-dependent glutamate synthase	WP_002807955.:	518773..523347	Nitrogen Metabolism	COG0069
WP_002807955.1_559	CUST_2879_PI442190825	MIT9292		WP_002807955.1_559	GTTTGTGCAAAAATTTCAGGAGAGCTTGCTCAGCATTATGGTAATAAGGGTTTTGAGGGC	glsF	Ferredoxin-dependent glutamate synthase	WP_002807955.:	518773..523347	Nitrogen Metabolism	COG0069
WP_002807955.1_559	CUST_2880_PI442190825	MIT9292		WP_002807955.1_559	TAGATAAGAATTCATCGGAGCAAGTCATTTTGGGCAATACTTGTCTGTATGGCGCTACGG	glsF	Ferredoxin-dependent glutamate synthase	WP_002807955.:	518773..523347	Nitrogen Metabolism	COG0069
WP_002807955.1_559	CUST_2881_PI442190825	MIT9292		WP_002807955.1_559	TTGGAGGACTCGATTTTAACAGATGCTCAATTCATAGATGCGGTGACTACTCATGGAGAA	glsF	Ferredoxin-dependent glutamate synthase	WP_002807955.:	518773..523347	Nitrogen Metabolism	COG0069
WP_002807955.1_559	CUST_2882_PI442190825	MIT9292		WP_002807955.1_559	AGGGCGCAATTAATCTAATTTTTAATGGTCATGCGGGTCAAAGCTTTGGAGCCTT	glsF	Ferredoxin-dependent glutamate synthase	WP_002807955.:	518773..523347	Nitrogen Metabolism	COG0069
WP_002807071.1_570	CUST_2932_PI442190825	MIT9292		WP_002807071.1_570	AATAGCTAACCTTGTAGGCTGGAGAGATAAACCAGTGGCACTTTCAATTGTTCAGGCTAG	psaB	Photosystem I P700 chlorophyll A apoprotein A2 PsaB	WP_002807071.:	529714..531942	Photosystem I	COG2885
WP_002807071.1_570	CUST_2933_PI442190825	MIT9292		WP_002807071.1_570	TGGCTTCAATGCTTTACTATCTGATCCTACTAGTGCTGCTAGTGTCGCAGCAAATTCTTT	psaB	Photosystem I P700 chlorophyll A apoprotein A2 PsaB	WP_002807071.:	529714..531942	Photosystem I	COG2885
WP_002807071.1_570	CUST_2934_PI442190825	MIT9292		WP_002807071.1_570	ATAGCATGGGTCACTTTCTACTGGCATTGGAAACATCTAGCAATATGGCAGGGTAATGTA	psaB	Photosystem I P700 chlorophyll A apoprotein A2 PsaB	WP_002807071.:	529714..531942	Photosystem I	COG2885
WP_002807071.1_570	CUST_2935_PI442190825	MIT9292		WP_002807071.1_570	TTACCCATTGGCCCTGCTGATTTCTTAGTTCATCATGCTATTGCACTAGGACTTCATACA	psaB	Photosystem I P700 chlorophyll A apoprotein A2 PsaB	WP_002807071.:	529714..531942	Photosystem I	COG2885
WP_002807071.1_570	CUST_2936_PI442190825	MIT9292		WP_002807071.1_570	ATACAATCCGTTTGGAGTAAATGCACTATCTCCCTGGGCTTGGATGTTCTTATTTGGTCA	psaB	Photosystem I P700 chlorophyll A apoprotein A2 PsaB	WP_002807071.:	529714..531942	Photosystem I	COG2885
WP_002807071.1_570	CUST_2937_PI442190825	MIT9292		WP_002807071.1_570	ACAAAGTTGATCCCTGATAAGAAAGATTTAGGTTACGCTTTCCCTTGTGATGGACCAGGG	psaB	Photosystem I P700 chlorophyll A apoprotein A2 PsaB	WP_002807071.:	529714..531942	Photosystem I	COG2885
WP_002805306.1_571	CUST_2938_PI442190825	MIT9292		WP_002805306.1_571	GAGTGATGTTTGGGGACTTACTGGAGGCAATTTCGCTCAAAGTTCCATAACCATAAATGG	psaA	Photosystem I P700 chlorophyll A apoprotein A1 PsaA	WP_002805306.:	531970..534252	Photosystem I	COG2885
WP_002805306.1_571	CUST_2939_PI442190825	MIT9292		WP_002805306.1_571	CGAGTCAATTGTGTGGGCACACAATAAATTAAAAGTTGCTCCAACAATCCAACCAAGAGC	psaA	Photosystem I P700 chlorophyll A apoprotein A1 PsaA	WP_002805306.:	531970..534252	Photosystem I	COG2885
WP_002805306.1_571	CUST_2940_PI442190825	MIT9292		WP_002805306.1_571	TATTCCTGATAAGGCATCTCTAGGATTTAGATTTCCTTGCGATGGTCCAGGAAGAGGAGG	psaA	Photosystem I P700 chlorophyll A apoprotein A1 PsaA	WP_002805306.:	531970..534252	Photosystem I	COG2885
WP_002805306.1_571	CUST_2941_PI442190825	MIT9292		WP_002805306.1_571	CTGTCAGGTGTCTGCTTGGGATCATGTGTTCCTTGGCTTATTCTGGATGTATAACTGTAT	psaA	Photosystem I P700 chlorophyll A apoprotein A1 PsaA	WP_002805306.:	531970..534252	Photosystem I	COG2885
WP_002805306.1_571	CUST_2942_PI442190825	MIT9292		WP_002805306.1_571	ATGGCTTAGAGACTTCTTGTGGGCACAAGCTTCTCAAGTTTTGACGAGTTATGGTCAATC	psaA	Photosystem I P700 chlorophyll A apoprotein A1 PsaA	WP_002805306.:	531970..534252	Photosystem I	COG2885
WP_002805306.1_571	CUST_2943_PI442190825	MIT9292		WP_002805306.1_571	CATTACTCAAGGTCGGGCAGTAGGCGTAACTCACTTCCTAGTTGGTGGTATAGCTACTAC	psaA	Photosystem I P700 chlorophyll A apoprotein A1 PsaA	WP_002805306.:	531970..534252	Photosystem I	COG2885
WP_002807116.1_623	CUST_3222_PI442190825	MIT9292		WP_002807116.1_623	TACAGTAGAAGTGAACTATTGAGGGGGGCACTTTTAACTATTAATGGCATAGCAGCTGGC	ppc	Phosphoenolpyruvate carboxylase	WP_002807116.:	571652..574621	Krebs cycle and related genes	COG2352
WP_002807116.1_623	CUST_3223_PI442190825	MIT9292		WP_002807116.1_623	TAGACTTGACGCTACCCCAGAATGGAATCAATTAATGTCTAGGTTGGCAGAAACGTCAAG	ppc	Phosphoenolpyruvate carboxylase	WP_002807116.:	571652..574621	Krebs cycle and related genes	COG2352
WP_002807116.1_623	CUST_3225_PI442190825	MIT9292		WP_002807116.1_623	CCAAGTTGGTTTGGAGTGGGTACTGCATTGTCATCTGAATTAAATTCTGATCCACGACAA	ppc	Phosphoenolpyruvate carboxylase	WP_002807116.:	571652..574621	Krebs cycle and related genes	COG2352
WP_002807116.1_623	CUST_3226_PI442190825	MIT9292		WP_002807116.1_623	TGGGTTTTTTGCAGGTTTCACTTTTAAGAAGATTAAGAGACCAGACAAGACAACCCCCAA	ppc	Phosphoenolpyruvate carboxylase	WP_002807116.:	571652..574621	Krebs cycle and related genes	COG2352
WP_002807116.1_623	CUST_3227_PI442190825	MIT9292		WP_002807116.1_623	TCTAAGGTGGATTTGGAAGTTGCCAGATATTATGTTGATACTCTTGGCAGTAGGGAAAAT	ppc	Phosphoenolpyruvate carboxylase	WP_002807116.:	571652..574621	Krebs cycle and related genes	COG2352
WP_002807135.1_654	CUST_3401_PI442190825	MIT9292		WP_002807135.1_654	AGGCAGGTTTCAAATATAATTGATATTTGCGCAGGAATAGAAGTGGGAGTTGCAGCAACA	glmS	Glutamine--fructose-6-phosphatetransaminase(isomerizing)	WP_002807135.:	604793..606688	Amino Acid metabolism	COG0449
WP_002807135.1_654	CUST_3402_PI442190825	MIT9292		WP_002807135.1_654	AGTAATGCTCAAGAAGCAAAAGCTAGAGATTCATTCTTGATTGGATTGCCCCTGAATGC	glmS	Glutamine--fructose-6-phosphatetransaminase(isomerizing)	WP_002807135.:	604793..606688	Amino Acid metabolism	COG0449
WP_002807135.1_654	CUST_3403_PI442190825	MIT9292		WP_002807135.1_654	GCGTTAAAACTCAAAGAAATTAGTTACATTCACGCGGCTGGATATCCAGCTGGTGAGATG	glmS	Glutamine--fructose-6-phosphatetransaminase(isomerizing)	WP_002807135.:	604793..606688	Amino Acid metabolism	COG0449
WP_002807135.1_654	CUST_3404_PI442190825	MIT9292		WP_002807135.1_654	AGTGCCTTTACAATTGTTGAGTTACCATATTGCAGCTCATAGAGGTTTAGACGTGGATCA	glmS	Glutamine--fructose-6-phosphatetransaminase(isomerizing)	WP_002807135.:	604793..606688	Amino Acid metabolism	COG0449
WP_002807135.1_654	CUST_3405_PI442190825	MIT9292		WP_002807135.1_654	AACTGCTGATACAATCGCAGCTATCGATATGGAAATTAAAAGACGGTCTTCGATTGAAGA	glmS	Glutamine--fructose-6-phosphatetransaminase(isomerizing)	WP_002807135.:	604793..606688	Amino Acid metabolism	COG0449
WP_002807135.1_654	CUST_3406_PI442190825	MIT9292		WP_002807135.1_654	ACGGTCCAATAGCTTTATTAGATAAAAAAGTCCCTGTAATTTCTATTGCTTCCCCTGGTG	glmS	Glutamine--fructose-6-phosphatetransaminase(isomerizing)	WP_002807135.:	604793..606688	Amino Acid metabolism	COG0449
WP_002806783.1_776	CUST_4092_PI442190825	MIT9292		WP_002806783.1_776	ACAGGAAGCAGCTTAATGCCGCAGAAAAAAAATCCTGATGTTCCTGAATTGATAAGAGGT	argH	Argininosuccinate lyase	WP_002806783.:	740860..742239	Amino Acid metabolism	COG0165
WP_002806783.1_776	CUST_4093_PI442190825	MIT9292		WP_002806783.1_776	ACGGGAAGAGTTTATGGACATCTTCAGGCATTGTTAACTATGGTAAAAGGTGTTCCGCTT	argH	Argininosuccinate lyase	WP_002806783.:	740860..742239	Amino Acid metabolism	COG0165
WP_002806783.1_776	CUST_4094_PI442190825	MIT9292		WP_002806783.1_776	GGCAGATTACTTAGTTGGTAAAGACGTTCCTTTTAGGACTGCTTATCACGTTGTTGGCGA	argH	Argininosuccinate lyase	WP_002806783.:	740860..742239	Amino Acid metabolism	COG0165
WP_002806783.1_776	CUST_4095_PI442190825	MIT9292		WP_002806783.1_776	ACCTCATAACGTCGTTAAATCAAGAAATAGTGAAGGTGGTACAGGTTTTGTTCAGGTAGA	argH	Argininosuccinate lyase	WP_002806783.:	740860..742239	Amino Acid metabolism	COG0165
WP_002806783.1_776	CUST_4096_PI442190825	MIT9292		WP_002806783.1_776	ACAGCATTGATGCAGTAGTGATAGAGATTTTTGTATTGAGTTTGTTTCTGCTTCTGCTT	argH	Argininosuccinate lyase	WP_002806783.:	740860..742239	Amino Acid metabolism	COG0165
WP_002806783.1_776	CUST_4097_PI442190825	MIT9292		WP_002806783.1_776	ACTGCAGAAACAATATCTTCTTGTATGAAAGCAATGACTATTTTGATTAATGAGGGCATT	argH	Argininosuccinate lyase	WP_002806783.:	740860..742239	Amino Acid metabolism	COG0165
WP_002805405.1_989	CUST_5264_PI442190825	MIT9292		WP_002805405.1_989	TTGTCATCAAGCGAAGAAGCCGGGGACTTATTATTAGAAGCAATTCAAAAAGCCGGATTT	eno	Enolase	WP_002805405.:	939623..940915	Glycolysis/Gluconeogenesis	COG0148
WP_002805405.1_989	CUST_5265_PI442190825	MIT9292		WP_002805405.1_989	TACAAGATCGGGTCAGATAAAGACCGGCTCTTTGAGCAGAAGTGAAAGGATTGCAAAATA	eno	Enolase	WP_002805405.:	939623..940915	Glycolysis/Gluconeogenesis	COG0148
WP_002805405.1_989	CUST_5266_PI442190825	MIT9292		WP_002805405.1_989	TCATTAAAATCATTGCTTGATCAAAAAGGTCTATCTACTGCCGTAGGCGATGAGGGTGGA	eno	Enolase	WP_002805405.:	939623..940915	Glycolysis/Gluconeogenesis	COG0148
WP_002805405.1_989	CUST_5267_PI442190825	MIT9292		WP_002805405.1_989	TCCAATAGTTTCAATAGAGGATGGTTTAGCTGAGGATGATTGGGACGGTTGGTCAGAATT	eno	Enolase	WP_002805405.:	939623..940915	Glycolysis/Gluconeogenesis	COG0148
WP_002805405.1_989	CUST_5268_PI442190825	MIT9292		WP_002805405.1_989	TGAAGAGGAATTGGGAAATCAAGCAAGATTCGCTGGAGTTTTAGGTTTAGGTCCCAAAAA	eno	Enolase	WP_002805405.:	939623..940915	Glycolysis/Gluconeogenesis	COG0148
WP_002805405.1_989	CUST_5269_PI442190825	MIT9292		WP_002805405.1_989	CCTGGCGAGCAGGTGTCTTTAGCTTTAGATGCTGCTAGTACTGAATTTTATAATGATGGC	eno	Enolase	WP_002805405.:	939623..940915	Glycolysis/Gluconeogenesis	COG0148
WP_002807335.1_1006	CUST_5348_PI442190825	MIT9292		WP_002807335.1_1006	AGCTAGTTCGATAGATATCCTTAACTTTGAAAGTATTGATTTCATTGCTGCTGGAGGCCT	eda	2-dehydro-3-deoxyphosphogluconate aldolase	WP_002807335.:	957508..958137	Entner-Dudoroff	COG0800
WP_002807335.1_1006	CUST_5349_PI442190825	MIT9292		WP_002807335.1_1006	TGGATTTTGTATACGAAATCAAACTCAAATTTCCAAGAATTAATTTAGGCTCTGCCTCCA	eda	2-dehydro-3-deoxyphosphogluconate aldolase	WP_002807335.:	957508..958137	Entner-Dudoroff	COG0800
WP_002807335.1_1006	CUST_5350_PI442190825	MIT9292		WP_002807335.1_1006	AGGTTCTTTTTCTGATTTGTTGCTAAAAGAATCTTTTTTTTACTCATAAAACCGGAAGA	eda	2-dehydro-3-deoxyphosphogluconate aldolase	WP_002807335.:	957508..958137	Entner-Dudoroff	COG0800
WP_002807335.1_1006	CUST_5351_PI442190825	MIT9292		WP_002807335.1_1006	AGGTAGCTTAGTAAAATTGGGATTAAAGAATATTGAAATAAGTTGGTCAAACAACGAAAA	eda	2-dehydro-3-deoxyphosphogluconate aldolase	WP_002807335.:	957508..958137	Entner-Dudoroff	COG0800
WP_002807335.1_1006	CUST_5352_PI442190825	MIT9292		WP_002807335.1_1006	TCTGGGATAAAGATCTTTTCAATTATTCGAAGTCAAAAACTATTTATTAATTCCCGGAA	eda	2-dehydro-3-deoxyphosphogluconate aldolase	WP_002807335.:	957508..958137	Entner-Dudoroff	COG0800
WP_002807335.1_1006	CUST_5353_PI442190825	MIT9292		WP_002807335.1_1006	AGGATATAAAGCAATCGTGATTGGAGATAAAGGTATTATTAAAAAAAAATTTGATCCCAA	eda	2-dehydro-3-deoxyphosphogluconate aldolase	WP_002807335.:	957508..958137	Entner-Dudoroff	COG0800
WP_002806062.1_1104	CUST_5866_PI442190825	MIT9292		WP_002806062.1_1104	AGAACTATTTCAGGAGAAATAAAATTCAAAAAACTGATAAAGCTAATAAAGATATCAAA	minE	septum site-determining protein MinE	WP_002806062.:	1044370..1044696	Cell Division	COG0851
WP_002806062.1_1104	CUST_5867_PI442190825	MIT9292		WP_002806062.1_1104	TGGCAGTAAGTTTAGAGACTGAGGATAGAATGACTGCATTAGTTGCCAATTTACCAATCA	minE	septum site-determining protein MinE	WP_002806062.:	1044370..1044696	Cell Division	COG0851
WP_002806062.1_1104	CUST_5868_PI442190825	MIT9292		WP_002806062.1_1104	AGTTCCTTAACAACTGATCTTTTAGATAAAATGAGGAAAGAGATTCTTGATGTCGTTGCT	minE	septum site-determining protein MinE	WP_002806062.:	1044370..1044696	Cell Division	COG0851
WP_002806062.1_1104	CUST_5869_PI442190825	MIT9292		WP_002806062.1_1104	AGGCAGAGAAACGTCTAGTGCAAATACAGCTAGAGAAAGATTACAGCTTGTACTCGCTCA	minE	septum site-determining protein MinE	WP_002806062.:	1044370..1044696	Cell Division	COG0851
WP_002805184.1_1105	CUST_5870_PI442190825	MIT9292		WP_002805184.1_1105	CATAGCACTTGCTAATAGTGGAGCAACAACTGCCGTATTAGATGCTGATTTTGGCTTGAG	minD	septum site-determining protein MinD	WP_002805184.:	1044698..1045513	Cell Division	COG2894
WP_002805184.1_1105	CUST_5871_PI442190825	MIT9292		WP_002805184.1_1105	GATTTTGTCTTAGTAGATTGCCCTGCTGGTGTAGAAGATGGCTTAAAAATGCCCTTGCA	minD	septum site-determining protein MinD	WP_002805184.:	1044698..1045513	Cell Division	COG2894
WP_002805184.1_1105	CUST_5872_PI442190825	MIT9292		WP_002805184.1_1105	AATCTTGCTCTTTTACCAGCTGGAGATCCAAGAATGTTAGATTGGATGAAGCCCGAAGAT	minD	septum site-determining protein MinD	WP_002805184.:	1044698..1045513	Cell Division	COG2894
WP_002805184.1_1105	CUST_5873_PI442190825	MIT9292		WP_002805184.1_1105	TTCGCCCTAACATGATGGCTAGTCAAGAAATGCTATCTATCGAAGATGTGCAAGGGATTC	minD	septum site-determining protein MinD	WP_002805184.:	1044698..1045513	Cell Division	COG2894
WP_002805184.1_1105	CUST_5874_PI442190825	MIT9292		WP_002805184.1_1105	GCTTGTAAAGAGGCTATTGTTGTTACTAACCCAGAATTATCTGCAGTACGCGATGCCGAT	minD	septum site-determining protein MinD	WP_002805184.:	1044698..1045513	Cell Division	COG2894
WP_002805184.1_1105	CUST_5875_PI442190825	MIT9292		WP_002805184.1_1105	AATCGCATCATTTATACGGCTCAAGATGTTCTAGACAGTAATTGTCGTCTTGACCAAGCA	minD	septum site-determining protein MinD	WP_002805184.:	1044698..1045513	Cell Division	COG2894
WP_002808384.1_1106	CUST_5876_PI442190825	MIT9292		WP_002808384.1_1106	GTTTCCGCTAAGAAAAATATTTACGTTTGGGGTAAATTGCAAGGAATCGCATTCGCAGGG	minC	septum site-determining protein MinC	WP_002808384.:	1045625..1046287	Cell Division	COG0850
WP_002808384.1_1106	CUST_5877_PI442190825	MIT9292		WP_002808384.1_1106	AAAAGATTGTCTTCCATAGAGGGGCGTTTAGAAGCAAAAATTTTCGCTGTCAATGAGTCA	minC	septum site-determining protein MinC	WP_002808384.:	1045625..1046287	Cell Division	COG0850
WP_002808384.1_1106	CUST_5878_PI442190825	MIT9292		WP_002808384.1_1106	ACCCTTTACAGTTAAGGATTGCAGATGTAATTGCTATTGGACCAAAGGATAAACCCAAGA	minC	septum site-determining protein MinC	WP_002808384.:	1045625..1046287	Cell Division	COG0850
WP_002808384.1_1106	CUST_5879_PI442190825	MIT9292		WP_002808384.1_1106	GGTGAAAGAATATCTTCAAATGGAACCTATGCATTATTGGAGACGTTAATCCAGGAGCA	minC	septum site-determining protein MinC	WP_002808384.:	1045625..1046287	Cell Division	COG0850
WP_002808384.1_1106	CUST_5880_PI442190825	MIT9292		WP_002808384.1_1106	AAGTTACTATTGTTCAATTCAAAAAAGAAAGAAGATATTCTTCACAAAGGAACAGTACGA	minC	septum site-determining protein MinC	WP_002808384.:	1045625..1046287	Cell Division	COG0850
WP_002808384.1_1106	CUST_5881_PI442190825	MIT9292		WP_002808384.1_1106	CCAGAAATTGCGATAATAGATAACAAAACGATAATCATAAAACCACATATAATCGAAAGT	minC	septum site-determining protein MinC	WP_002808384.:	1045625..1046287	Cell Division	COG0850
WP_002805394.1_1251	CUST_6622_PI442190825	MIT9292		WP_002805394.1_1251	TTGGACTTATAACTTTCCTAATTGCTGACGGAATGACTTTTGCTGGATTCTTTGCTGCTT	cyoC	heme-copper oxidase subunit III	WP_002805394.:	1173034..1173636	Respiration	COG1845
WP_002805394.1_1251	CUST_6623_PI442190825	MIT9292		WP_002805394.1_1251	TGCAAGTACTTTTTATGCTCTTACTGGTTTTCATGGATTACATGTCACTTTAGGCACTT	cyoC	heme-copper oxidase subunit III	WP_002805394.:	1173034..1173636	Respiration	COG1845
WP_002805394.1_1251	CUST_6624_PI442190825	MIT9292		WP_002805394.1_1251	AGCAGTAAATCCATTACCAGATGGTGCTATTTATGAATTAGAACTACCAATCCCTACACT	cyoC	heme-copper oxidase subunit III	WP_002805394.:	1173034..1173636	Respiration	COG1845
WP_002805394.1_1251	CUST_6625_PI442190825	MIT9292		WP_002805394.1_1251	TGGCGGAAGATTAAATAGTCAAAATATATTTCCATTAGAAGCTGTTGAGTTGTACTGGCA	cyoC	heme-copper oxidase subunit III	WP_002805394.:	1173034..1173636	Respiration	COG1845
WP_002805394.1_1251	CUST_6626_PI442190825	MIT9292		WP_002805394.1_1251	AGTGCAACTTTCCATAAAGCAGGTAAATCACTTTTAAAAGATAAAAACTCTGATTCACAA	cyoC	heme-copper oxidase subunit III	WP_002805394.:	1173034..1173636	Respiration	COG1845
WP_002805394.1_1251	CUST_6627_PI442190825	MIT9292		WP_002805394.1_1251	AGCTCAAAAGAAATTCAAAAAAATAATTCTGAAGTTAATGAAACACATGAAGACTTCAGA	cyoC	heme-copper oxidase subunit III	WP_002805394.:	1173034..1173636	Respiration	COG1845
WP_002806073.1_1252	CUST_6628_PI442190825	MIT9292		WP_002806073.1_1252	CTTCTCCGCCTCCGGTTGAAAATTGGGAAAAAGAAGCTCCATTAGTTGAAGAACCTTATG	cyoB/coxA	Cytochrome c oxidase subunit I	WP_002806073.:	1173643..1175268	Respiration	COG0843
WP_002806073.1_1252	CUST_6629_PI442190825	MIT9292		WP_002806073.1_1252	TGGAGGTATTACAGGAGTTGCTTGGCACAGGTCCCTTTTGATATTCACGTACATGATAC	cyoB/coxA	Cytochrome c oxidase subunit I	WP_002806073.:	1173643..1175268	Respiration	COG0843
WP_002806073.1_1252	CUST_6630_PI442190825	MIT9292		WP_002806073.1_1252	TGGATGCGATTGTTCTTTACTATCGCAACAGCATTTATTGCTGTTCCAACGGGGATAAAA	cyoB/coxA	Cytochrome c oxidase subunit I	WP_002806073.:	1173643..1175268	Respiration	COG0843
WP_002806073.1_1252	CUST_6631_PI442190825	MIT9292		WP_002806073.1_1252	CTTTTATTGGATTTAACTTGTGCTTCGCTCCTCAACATTGGCTCGGTTTAAACGGAATGC	cyoB/coxA	Cytochrome c oxidase subunit I	WP_002806073.:	1173643..1175268	Respiration	COG0843
WP_002806073.1_1252	CUST_6632_PI442190825	MIT9292		WP_002806073.1_1252	ATGGGGATAGTTGTTTTAGGTTTAGTTGTTTGGGCGCATCACATGTTTACTAGTGGAACG	cyoB/coxA	Cytochrome c oxidase subunit I	WP_002806073.:	1173643..1175268	Respiration	COG0843
WP_002806073.1_1252	CUST_6633_PI442190825	MIT9292		WP_002806073.1_1252	GTGTGAGAAATGGAAAAGATGCTGGAGATAATCCTTGGAATGCTCTTACAGCTGAATGGT	cyoB/coxA	Cytochrome c oxidase subunit I	WP_002806073.:	1173643..1175268	Respiration	COG0843
WP_002805052.1_1253	CUST_6634_PI442190825	MIT9292		WP_002805052.1_1253	CATGGAGGGATGAGAGCCGCCATAATTGTTGAGGAAGAATCAGACTATAACGATTGGTTT	ctaC/coxB	Cytochrome c oxidase subunit II	WP_002805052.:	1175265..1176068	Respiration	COG1622
WP_002805052.1_1253	CUST_6635_PI442190825	MIT9292		WP_002805052.1_1253	ACAACATCTACGACCGAATGGGAGGGATGAAAGAGCTAAATCATAACCACGAAATGATGA	ctaC/coxB	Cytochrome c oxidase subunit II	WP_002805052.:	1175265..1176068	Respiration	COG1622
WP_002805052.1_1253	CUST_6636_PI442190825	MIT9292		WP_002805052.1_1253	TCTTTTTGGATTGGAACCAATGTAAATTTGCTCCCAGCTGAAGCAAGTATTAATGCACCA	ctaC/coxB	Cytochrome c oxidase subunit II	WP_002805052.:	1175265..1176068	Respiration	COG1622
WP_002805052.1_1253	CUST_6637_PI442190825	MIT9292		WP_002805052.1_1253	TGCTTTTTGGGTACCTGAGTTCAGAATTAAACAGGATATTATTCCTGGTCAACCAACTGT	ctaC/coxB	Cytochrome c oxidase subunit II	WP_002805052.:	1175265..1176068	Respiration	COG1622
WP_002805052.1_1253	CUST_6638_PI442190825	MIT9292		WP_002805052.1_1253	AATTTCACTCCTACAAAAGTAGGAAAATATCCAATAATCTGCGCAGAATTATGTGGCCCA	ctaC/coxB	Cytochrome c oxidase subunit II	WP_002805052.:	1175265..1176068	Respiration	COG1622
WP_002805052.1_1253	CUST_6639_PI442190825	MIT9292		WP_002805052.1_1253	TGCATTTCTATTCAATTATCCCAAAGGCAAATTTATATCAGGAGAACTACACGTTCCAGT	ctaC/coxB	Cytochrome c oxidase subunit II	WP_002805052.:	1175265..1176068	Respiration	COG1622
WP_002806376.1_1261	CUST_6677_PI442190825	MIT9292		WP_002806376.1_1261	CAAGCAAATTATTCTGCAGCCAAAGCTGGAGTTATTGGATTCACCAAAACTTGCGCTAAA	gdh	3-oxoacyl-acyl-carrier-protein reductase	WP_002806376.:	1182645..1183394	Entner-Dudoroff	COG1028
WP_002806376.1_1261	CUST_6678_PI442190825	MIT9292		WP_002806376.1_1261	GCTAGCAGAGGAATTGGTAAAGAGATTGCTTTAGAACTTAGCCGCTTAGGAGCAGAAGTT	gdh	3-oxoacyl-acyl-carrier-protein reductase	WP_002806376.:	1182645..1183394	Entner-Dudoroff	COG1028
WP_002806376.1_1261	CUST_6679_PI442190825	MIT9292		WP_002806376.1_1261	ATGCTGGCATTACTAGAGATGGACTCTTGATGAGAATGAAATCGGAACAATGGATGACG	gdh	3-oxoacyl-acyl-carrier-protein reductase	WP_002806376.:	1182645..1183394	Entner-Dudoroff	COG1028
WP_002806376.1_1261	CUST_6680_PI442190825	MIT9292		WP_002806376.1_1261	TCAAGTAATGCTGGAAGTTATATCACGGGACAAACAATTAGTATTGACGGAGGTATGAGT	gdh	3-oxoacyl-acyl-carrier-protein reductase	WP_002806376.:	1182645..1183394	Entner-Dudoroff	COG1028
WP_002806376.1_1261	CUST_6681_PI442190825	MIT9292		WP_002806376.1_1261	ACCGATGAGATTCTTAAAGCTATTCCTTTAGGGAAATTAGGAAGTTGCACTCAAATTGCA	gdh	3-oxoacyl-acyl-carrier-protein reductase	WP_002806376.:	1182645..1183394	Entner-Dudoroff	COG1028
WP_002806376.1_1261	CUST_6682_PI442190825	MIT9292		WP_002806376.1_1261	TGCTTCAAGGAGTATAAACGTAAATGCAATAGCTCCTGGTTTCATAGAAACAGAGATGAC	gdh	3-oxoacyl-acyl-carrier-protein reductase	WP_002806376.:	1182645..1183394	Entner-Dudoroff	COG1028
WP_002805504.1_1327	CUST_7038_PI442190825	MIT9292		WP_002805504.1_1327	TGAAAATTATTTCAGGGCGCGTATCTACAGAAGTTGATGCAAGATTGAGCTTTGACACCG	tal	Transaldolase	WP_002805504.:	1239608..1240609	Pentose Phosphate	COG0176
WP_002805504.1_1327	CUST_7039_PI442190825	MIT9292		WP_002805504.1_1327	TAACTTTTGCCAAGCGGTAACTTGTGCCAATGCAAAGATAACTCTAATTTCTCCGTTCGT	tal	Transaldolase	WP_002805504.:	1239608..1240609	Pentose Phosphate	COG0176
WP_002805504.1_1327	CUST_7040_PI442190825	MIT9292		WP_002805504.1_1327	ATTGGGAAGGAAAGAATTTTGATTAAGATTGCTGCAACTTGGGAGGGAATTAAGGCAGCT	tal	Transaldolase	WP_002805504.:	1239608..1240609	Pentose Phosphate	COG0176
WP_002805504.1_1327	CUST_7041_PI442190825	MIT9292		WP_002805504.1_1327	AAAGCTTAGTGAAGGTATCACTGGATTCAGTAAGGCTATAGAAGAATTGGAGGAGCTGCT	tal	Transaldolase	WP_002805504.:	1239608..1240609	Pentose Phosphate	COG0176
WP_002805504.1_1327	CUST_7042_PI442190825	MIT9292		WP_002805504.1_1327	GGATTGGTATAAGGCAAAAACTGGTAAAAATAGTTTTGTTGGTGCTGAAGACCCTGGTGT	tal	Transaldolase	WP_002805504.:	1239608..1240609	Pentose Phosphate	COG0176
WP_002805504.1_1327	CUST_7043_PI442190825	MIT9292		WP_002805504.1_1327	AGCAGGTTGCGATCTTTTAACAATCGCACCAAAATTTCTTGAGGAACTGAAAAAAGAAAA	tal	Transaldolase	WP_002805504.:	1239608..1240609	Pentose Phosphate	COG0176
WP_011376108.1_1359	CUST_7220_PI442190825	MIT9292		WP_011376108.1_1359	ATGCTTACACTCAAAGCCAAGGAACAGCTTTTGTTGTTTTCCAAGGACGTTAA	rbcS	Ribulose bisphosphate carboxylase small subunit	WP_011376108.:	1269544..1269885	Carbon fixation	COG4451
WP_011376108.1_1359	CUST_7221_PI442190825	MIT9292		WP_011376108.1_1359	AGAGGCATGTCATAGAGCATACCCTGATCACCATGTAAGAATCATCGGATACGATGCTTA	rbcS	Ribulose bisphosphate carboxylase small subunit	WP_011376108.:	1269544..1269885	Carbon fixation	COG4451
WP_011376108.1_1359	CUST_7222_PI442190825	MIT9292		WP_011376108.1_1359	GTCTTATTGGAAGCTCCCATTCTTTGGGGAAAAAGATCTTAACTTGGTTGTAAGTG	rbcS	Ribulose bisphosphate carboxylase small subunit	WP_011376108.:	1269544..1269885	Carbon fixation	COG4451
WP_011376108.1_1359	CUST_7223_PI442190825	MIT9292		WP_011376108.1_1359	GGTTGGAGTCCTGTTATTGAGCACGTTCATCCTAGTGGAAGTATGCAAACTTATTGGTCT	rbcS	Ribulose bisphosphate carboxylase small subunit	WP_011376108.:	1269544..1269885	Carbon fixation	COG4451
WP_011376108.1_1359	CUST_7224_PI442190825	MIT9292		WP_011376108.1_1359	GCAACCCTGGAAACATTCGGTTTCTTACCACCGATGACCCAGGAAGAAATATATGATCAA	rbcS	Ribulose bisphosphate carboxylase small subunit	WP_011376108.:	1269544..1269885	Carbon fixation	COG4451
WP_002806954.1_1404	CUST_7460_PI442190825	MIT9292		WP_002806954.1_1404	TATCCAAAATGGATCCCATTCTGAAGGTGCTAATAATGATCTGCATGCCATTGTTGGAGC	glk	Glucokinase	WP_002806954.:	1313990..1315024	Glycolysis	COG0837
WP_002806954.1_1404	CUST_7461_PI442190825	MIT9292		WP_002806954.1_1404	TATGCATCTTTATTGGGAGATGTTGCTCTTCAAGAATTGTGCTTTGGCGGGTTATGGATT	glk	Glucokinase	WP_002806954.:	1313990..1315024	Glycolysis	COG0837
WP_002806954.1_1404	CUST_7462_PI442190825	MIT9292		WP_002806954.1_1404	AATTATTAGCGGCACTGGTTTATCAAGAATTGCCGAATGGAGACTAAGCAAACCTGATGC	glk	Glucokinase	WP_002806954.:	1313990..1315024	Glycolysis	COG0837
WP_002806954.1_1404	CUST_7463_PI442190825	MIT9292		WP_002806954.1_1404	ATCAGGGAAAAAGGTAAAAGTTTTAGCTAGTGAAGGTGGCCATGTTGAGTACTCCCCAAA	glk	Glucokinase	WP_002806954.:	1313990..1315024	Glycolysis	COG0837
WP_002806954.1_1404	CUST_7464_PI442190825	MIT9292		WP_002806954.1_1404	AGATGAAGAGTTTGGACTTTTTAGTGCAGCCTGCAGAGCAAAAATGCTTTTAAAAACCTA	glk	Glucokinase	WP_002806954.:	1313990..1315024	Glycolysis	COG0837
WP_002806954.1_1404	CUST_7465_PI442190825	MIT9292		WP_002806954.1_1404	AATCTTTCTAAAGAAGGAGATCAGCTAATGATTGAAGTTGAGAGGATTTGGTTAGGTGCT	glk	Glucokinase	WP_002806954.:	1313990..1315024	Glycolysis	COG0837
WP_002807690.1_1581	CUST_8375_PI442190825	MIT9292		WP_002807690.1_1581	TGTGAGAACTAGGATTGAAAGTGATTCGGTTCTTGAGGATACTCTCGTTATGGGTGCTGA	glgC	Glucose-1-phosphate adenylyltransferase	WP_002807690.:	1470173..1471468	Glycogen Metabolism	COG0448
WP_002807690.1_1581	CUST_8376_PI442190825	MIT9292		WP_002807690.1_1581	GCATTCTTTGAGTCAAATCTCGCGTTGACGGAGCAACCTAAACCTCCATTCAGTTTTAT	glgC	Glucose-1-phosphate adenylyltransferase	WP_002807690.:	1470173..1471468	Glycogen Metabolism	COG0448
WP_002807690.1_1581	CUST_8377_PI442190825	MIT9292		WP_002807690.1_1581	CTGACTTTGGTAAGGACATAATTCCTGAAGCCCTAAATAGGGGCGATTCACTCAAAAGCT	glgC	Glucose-1-phosphate adenylyltransferase	WP_002807690.:	1470173..1471468	Glycogen Metabolism	COG0448
WP_002807690.1_1581	CUST_8378_PI442190825	MIT9292		WP_002807690.1_1581	AGTGAAAAGCCCACTGGTGAGAAGTTGAAAGCAATGGCAGTAGATACTTCAAAATTTGGA	glgC	Glucose-1-phosphate adenylyltransferase	WP_002807690.:	1470173..1471468	Glycogen Metabolism	COG0448
WP_002807690.1_1581	CUST_8379_PI442190825	MIT9292		WP_002807690.1_1581	CGGAACGCCTCTTGGAGTAGGGGAAGGAACTACAGTAAAAAGAGCAATTCTTGATAAGAA	glgC	Glucose-1-phosphate adenylyltransferase	WP_002807690.:	1470173..1471468	Glycogen Metabolism	COG0448
WP_002807690.1_1581	CUST_8380_PI442190825	MIT9292		WP_002807690.1_1581	TTTCTCCCCCCTTCTAAACTTGTAGATGCTCAAATTACTGATTCAATAGTCTGTGAAGGT	glgC	Glucose-1-phosphate adenylyltransferase	WP_002807690.:	1470173..1471468	Glycogen Metabolism	COG0448
WP_002806083.1_1582	CUST_8381_PI442190825	MIT9292		WP_002806083.1_1582	AGAACCAATAGACTTCCTCAGAATCTTGTTCAGGCAATGAGAGATTGTTTTGGCTCTCAT	edd	phosphogluconate dehydrogenase (NADP(+)-dependent, decarboxyla	WP_002806083.:	1471565..1472983	Pentose Phosphate/Entner-Dudorof	COG2084
WP_002806083.1_1582	CUST_8382_PI442190825	MIT9292		WP_002806083.1_1582	GATGGGATGAAACCTTTATTTGATGCTGTAGTCCTTGCCACAATTGCTAGCTATGCACAA	edd	phosphogluconate dehydrogenase (NADP(+)-dependent, decarboxyla	WP_002806083.:	1471565..1472983	Pentose Phosphate/Entner-Dudorof	COG2084
WP_002806083.1_1582	CUST_8383_PI442190825	MIT9292		WP_002806083.1_1582	TGCAAGAGTAATGAGCTCTTTAAAAGAGCAACGAAGTGAGATTGAAAAAACTATCCCATC	edd	phosphogluconate dehydrogenase (NADP(+)-dependent, decarboxyla	WP_002806083.:	1471565..1472983	Pentose Phosphate/Entner-Dudorof	COG2084
WP_002806083.1_1582	CUST_8384_PI442190825	MIT9292		WP_002806083.1_1582	GAAAGGTACTGGTTTATGGACTGTTGTAAGTGCTTTAGAACTGGGGGTATCAGTCCCAAC	edd	phosphogluconate dehydrogenase (NADP(+)-dependent, decarboxyla	WP_002806083.:	1471565..1472983	Pentose Phosphate/Entner-Dudorof	COG2084
WP_002806083.1_1582	CUST_8385_PI442190825	MIT9292		WP_002806083.1_1582	TACAAAAGATGAGATAACTGGAGATGATGTCGTGGAGAAAATATTAGATAAAGCTGGCCA	edd	phosphogluconate dehydrogenase (NADP(+)-dependent, decarboxyla	WP_002806083.:	1471565..1472983	Pentose Phosphate/Entner-Dudorof	COG2084
WP_002806083.1_1582	CUST_8386_PI442190825	MIT9292		WP_002806083.1_1582	TGCAGAAGCTTATGACCTTATGAAGAGAGTAAAAGGTATGAATGGAAAGCAGATGTCAGA	edd	phosphogluconate dehydrogenase (NADP(+)-dependent, decarboxyla	WP_002806083.:	1471565..1472983	Pentose Phosphate/Entner-Dudorof	COG2084
WP_002806386.1_1593	CUST_8442_PI442190825	MIT9292		WP_002806386.1_1593	TGCAAGATTTGCAAAGTGGAGAGCGGTTTTACAGATAACGAATGATGGTTGTCCTTCAAA	fda	Fructose-1,6-bisphosphate aldolase class I	WP_002806386.:	1480254..1481321	Glycolysis/Gluconeogenesis	COG0191
WP_002806386.1_1593	CUST_8443_PI442190825	MIT9292		WP_002806386.1_1593	ATGGAGCAGATTGCCCAACAAAGGCTGATCCTATGAAAGTTGCTGAGATGACTATAAGAA	fda	Fructose-1,6-bisphosphate aldolase class I	WP_002806386.:	1480254..1481321	Glycolysis/Gluconeogenesis	COG0191
WP_002806386.1_1593	CUST_8444_PI442190825	MIT9292		WP_002806386.1_1593	ATAGGAAAGCATTATGGAATGTTTCATTCTCCTATGGAAGAGCGCTACAGCACTCATGCT	fda	Fructose-1,6-bisphosphate aldolase class I	WP_002806386.:	1480254..1481321	Glycolysis/Gluconeogenesis	COG0191
WP_002806386.1_1593	CUST_8445_PI442190825	MIT9292		WP_002806386.1_1593	AAGGTTGATAAAGGTTTGAATCCACTTCCTGGAGGGGGAGATGTAGAAACATTTTGCTCT	fda	Fructose-1,6-bisphosphate aldolase class I	WP_002806386.:	1480254..1481321	Glycolysis/Gluconeogenesis	COG0191
WP_002806386.1_1593	CUST_8446_PI442190825	MIT9292		WP_002806386.1_1593	GGCCAAAAAGCCTTAATAGCAAGAGCTCAAGCAAACTCTGAAGCTTCAACAGGATCTTAT	fda	Fructose-1,6-bisphosphate aldolase class I	WP_002806386.:	1480254..1481321	Glycolysis/Gluconeogenesis	COG0191
WP_002806386.1_1593	CUST_8447_PI442190825	MIT9292		WP_002806386.1_1593	GTCTGTTCCGGGAATAGTTTTCTTATCAGGAGGATTAAGCGAAGAAGCGGCTTCTGTTTA	fda	Fructose-1,6-bisphosphate aldolase class I	WP_002806386.:	1480254..1481321	Glycolysis/Gluconeogenesis	COG0191
WP_002806339.1_1597	CUST_8465_PI442190825	MIT9292		WP_002806339.1_1597	TTCCAACCCCTGACGAGAGCTTTGTGATCATACATTTCAGAAAGGAGCAAGAGAGAAAT	prk	Phosphoribulokinase	WP_002806339.:	1483950..1484849	Carbon Fixation	COG3954
WP_002806339.1_1597	CUST_8466_PI442190825	MIT9292		WP_002806339.1_1597	GGGAAGATATTCCTAAGGGAACAGACGTACTTTTTTATGAAGGGTTACATGGCGGAGTGG	prk	Phosphoribulokinase	WP_002806339.:	1483950..1484849	Carbon Fixation	COG3954
WP_002806339.1_1597	CUST_8467_PI442190825	MIT9292		WP_002806339.1_1597	CTAGGAATGATTCACGATTCATTCATGTCTAGCCCAACCAGCATTGTTGTAAATGGTGGG	prk	Phosphoribulokinase	WP_002806339.:	1483950..1484849	Carbon Fixation	COG3954
WP_002806339.1_1597	CUST_8468_PI442190825	MIT9292		WP_002806339.1_1597	AAAAGCTATGGCAGAGGCTCTTTCTAAAGGTGAAAATTTTTCTCACTTTGGTCCAGAGGC	prk	Phosphoribulokinase	WP_002806339.:	1483950..1484849	Carbon Fixation	COG3954
WP_002806339.1_1597	CUST_8469_PI442190825	MIT9292		WP_002806339.1_1597	TGGTGAAACTGGGGGAGGGAAGAAAAGATATTATCTACATAGCCCTGAAGAAGCTGAAGA	prk	Phosphoribulokinase	WP_002806339.:	1483950..1484849	Carbon Fixation	COG3954
WP_002806339.1_1597	CUST_8470_PI442190825	MIT9292		WP_002806339.1_1597	GGTGATGGATACAATGTGGCATCTTATGCTGATTTACTTGTTGGAGTTGTTCCGATAACA	prk	Phosphoribulokinase	WP_002806339.:	1483950..1484849	Carbon Fixation	COG3954
WP_002805776.1_1673	CUST_8867_PI442190825	MIT9292		WP_002805776.1_1673	GAATTACCTGAGGGGGATTATACATGCGCTTTAGATCAGGCTGACGTTGTAAAAGAAGGT	pdhB	Pyruvate dehydrogenase E1 component beta subunit	WP_002805776.:	1548176..1549159	Glycolysis/Krebs cycle	COG0022
WP_002805776.1_1673	CUST_8868_PI442190825	MIT9292		WP_002805776.1_1673	TGGTATGGCTGTTGGTGCAGCAATGACTGGGTTAAGACCAATAGTAGAAGGAATGAATAT	pdhB	Pyruvate dehydrogenase E1 component beta subunit	WP_002805776.:	1548176..1549159	Glycolysis/Krebs cycle	COG0022
WP_002805776.1_1673	CUST_8869_PI442190825	MIT9292		WP_002805776.1_1673	GAGGAGTTGGTCGCCAACTTGGTGCTGAACATAGTCAAAGACTTGAAGCATATTTTCATG	pdhB	Pyruvate dehydrogenase E1 component beta subunit	WP_002805776.:	1548176..1549159	Glycolysis/Krebs cycle	COG0022
WP_002805776.1_1673	CUST_8870_PI442190825	MIT9292		WP_002805776.1_1673	GGTGCAGAATTAATTGCTTTGATAACAGAAGAGTGTTTCGATGATCTTGATGCCCGACCA	pdhB	Pyruvate dehydrogenase E1 component beta subunit	WP_002805776.:	1548176..1549159	Glycolysis/Krebs cycle	COG0022
WP_002805776.1_1673	CUST_8871_PI442190825	MIT9292		WP_002805776.1_1673	GGGAATGCTTAGATATACAAGCGGCGGAAATTATAAGATACCAGCAGTAGTTCGAGGTCC	pdhB	Pyruvate dehydrogenase E1 component beta subunit	WP_002805776.:	1548176..1549159	Glycolysis/Krebs cycle	COG0022
WP_002805776.1_1673	CUST_8872_PI442190825	MIT9292		WP_002805776.1_1673	TGTTGCATGCAGTACTCCTACAAATGCTAAAGGTTTAATGAAAGCAGCTATAAGAGATGA	pdhB	Pyruvate dehydrogenase E1 component beta subunit	WP_002805776.:	1548176..1549159	Glycolysis/Krebs cycle	COG0022
WP_002807107.1_1684	CUST_8923_PI442190825	MIT9292		WP_002807107.1_1684	TCCAAGCCCTAAAGCTAAAAGATTAGAATTCCGATCAGGAGACGCACTTGCAAATCCTTA	glnA	Glutamine synthetase/ glutamate--ammonialigase	WP_002807107.:	1556934..1558355	Nitrogen Metabolism	COG0174
WP_002807107.1_1684	CUST_8924_PI442190825	MIT9292		WP_002807107.1_1684	GGTTCCAGGATTCGAAGCACCTGTAAATCTAGTTTATTCTGAGGGTAATCGATCAGCTGC	glnA	Glutamine synthetase/ glutamate--ammonialigase	WP_002807107.:	1556934..1558355	Nitrogen Metabolism	COG0174
WP_002807107.1_1684	CUST_8925_PI442190825	MIT9292		WP_002807107.1_1684	GGAATGCATGTTCACCAAAGTTTATGGAAGAGTGGTCAGCCACTATTTTTTGGTGAAGGA	glnA	Glutamine synthetase/ glutamate--ammonialigase	WP_002807107.:	1556934..1558355	Nitrogen Metabolism	COG0174
WP_002807107.1_1684	CUST_8926_PI442190825	MIT9292		WP_002807107.1_1684	ATACGTTGTCAGGAATGTTGCTAAAAAATACGGGAAAACTGCAACATTTATGCCCAAGCC	glnA	Glutamine synthetase/ glutamate--ammonialigase	WP_002807107.:	1556934..1558355	Nitrogen Metabolism	COG0174
WP_002807107.1_1684	CUST_8927_PI442190825	MIT9292		WP_002807107.1_1684	TGGTATATCGGAGGCATACTTAAACATGCTCCATCCTTCTTAGCATTTACCAACCCAACT	glnA	Glutamine synthetase/ glutamate--ammonialigase	WP_002807107.:	1556934..1558355	Nitrogen Metabolism	COG0174
WP_002807107.1_1684	CUST_8928_PI442190825	MIT9292		WP_002807107.1_1684	GCGCAGGCCAACACGAACTTGGAATGAAATTCGATTCGTTAATAAATTCTGCAGATAACG	glnA	Glutamine synthetase/ glutamate--ammonialigase	WP_002807107.:	1556934..1558355	Nitrogen Metabolism	COG0174
WP_002807921.1_1692	CUST_8969_PI442190825	MIT9292		WP_002807921.1_1692	TCCTAAATCAAGGAGATTTAGTGGTTCAAACCGCAGGTACATTAACTGGAATTAGCGGCT	pykF	Pyruvate kinase	WP_002807921.:	1565575..1567365	Glycolysis	COG0469
WP_002807921.1_1692	CUST_8970_PI442190825	MIT9292		WP_002807921.1_1692	TTTGGCAACAACTACAGAGAGGAGTGTAGCTAGAAGATTGCAACTTGTTTGGGGAGTTAC	pykF	Pyruvate kinase	WP_002807921.:	1565575..1567365	Glycolysis	COG0469
WP_002807921.1_1692	CUST_8971_PI442190825	MIT9292		WP_002807921.1_1692	AGCTAGACAACTTGATGCAGGAGCTATAATCCCTTTAACAAAATCAGGTTCTACCGCTCG	pykF	Pyruvate kinase	WP_002807921.:	1565575..1567365	Glycolysis	COG0469
WP_002807921.1_1692	CUST_8972_PI442190825	MIT9292		WP_002807921.1_1692	GGCTATTGAAAGCCACTTACCCAGTACTATCCCAAATGCTATTAGTGCAGCAGTAAGT	pykF	Pyruvate kinase	WP_002807921.:	1565575..1567365	Glycolysis	COG0469
WP_002807921.1_1692	CUST_8973_PI442190825	MIT9292		WP_002807921.1_1692	AATGATGAACGAACAGCAAAAACTTTTAGTTTAGCTATGCAAATTGCTCAGGAGATGGGA	pykF	Pyruvate kinase	WP_002807921.:	1565575..1567365	Glycolysis	COG0469
WP_002807921.1_1692	CUST_8974_PI442190825	MIT9292		WP_002807921.1_1692	TGGCGACCTTATTACACTTCAGCTTAATGAAGGTGTTATTTACAAGGGACAAATTGAAGA	pykF	Pyruvate kinase	WP_002807921.:	1565575..1567365	Glycolysis	COG0469
WP_002807326.1_1714	CUST_9087_PI442190825	MIT9292		WP_002807326.1_1714	AAATGCATATGATCAACCTGGAGTTGAAGCTGGGAAGAAAGCAGCCGCAAATATTATTGA	pgi	Glucose-6-phosphate isomerase	WP_002807326.:	1586262..1587845	Glycolysis/Gluconeogenesis	COG0166
WP_002807326.1_1714	CUST_9088_PI442190825	MIT9292		WP_002807326.1_1714	AGAGGTGCATCATTAATGGATGAGGCCACACGTACAAGTGATTTTAAAAATAATCCCGCA	pgi	Glucose-6-phosphate isomerase	WP_002807326.:	1586262..1587845	Glycolysis/Gluconeogenesis	COG0166
WP_002807326.1_1714	CUST_9089_PI442190825	MIT9292		WP_002807326.1_1714	TGTTTTTCACTAGGGTCCTTAATCGCTTTATTTGAGAGGGCTGTATCCTTCTACGCTGAA	pgi	Glucose-6-phosphate isomerase	WP_002807326.:	1586262..1587845	Glycolysis/Gluconeogenesis	COG0166
WP_002807326.1_1714	CUST_9090_PI442190825	MIT9292		WP_002807326.1_1714	TCATCAGCATGGTATTTAACTGGAGATGGCGTTGGAAAGAGAGATATGGTCGTATTACCT	pgi	Glucose-6-phosphate isomerase	WP_002807326.:	1586262..1587845	Glycolysis/Gluconeogenesis	COG0166
WP_002807326.1_1714	CUST_9091_PI442190825	MIT9292		WP_002807326.1_1714	TCCGTTTTTGGTAATAAAGGATCTACAGATCAACATGCTTATGTTCAGCAACTGAGAGAT	pgi	Glucose-6-phosphate isomerase	WP_002807326.:	1586262..1587845	Glycolysis/Gluconeogenesis	COG0166
WP_002807326.1_1714	CUST_9092_PI442190825	MIT9292		WP_002807326.1_1714	ACTCCGTGAAATGTGTTTCGGTAATGATAATTATTTAGTTAAGGGCGATTGGTCAAATCC	pgi	Glucose-6-phosphate isomerase	WP_002807326.:	1586262..1587845	Glycolysis/Gluconeogenesis	COG0166
lcl2.1_WP_036918190.1	CUST_4079_PI442182269	MIT0603	LLII/III	lcl2.1_WP_036918190.1	GAGCGAATAAGTCTTACGGCTGAAGTGCTCAGTGCAGCCTCCAAAGTTATTTTTCTAGTA	pgi	Glucose-6-phosphate isomerase	WP_036918190.:	397885..398601	Glycolysis/Gluconeogenesis	COG0166
lcl4.1_WP_036919442.1	CUST_8877_PI442182269	MIT0603	LLII/III	lcl4.1_WP_036919442.1	AATCCAGTCAAGATGTTTCCTTATCTTGCTCAGATCTTGGTCAAGCCTTGTTGGATGACT	lldD	alpha-hydroxy-acidoxidizingprotein (L-lactate)	WP_036919442.:	273544..274716	Pyruvate metabolism	COG1304
lcl4.1_WP_036919442.1	CUST_8878_PI442182269	MIT0603	LLII/III	lcl4.1_WP_036919442.1	TTGATACCCCTGTCTCTGGATTACGCGAACGTGATTTACGTAATGGAACTAAGGAATTGC	lldD	alpha-hydroxy-acidoxidizingprotein (L-lactate)	WP_036919442.:	273544..274716	Pyruvate metabolism	COG1304
lcl4.1_WP_036919442.1	CUST_8879_PI442182269	MIT0603	LLII/III	lcl4.1_WP_036919442.1	CAGCTTTAGAGGCTTCAGTTGTAACTTGGGAGGATTTGAAATGGATTAAGGAAGCTTGGC	lldD	alpha-hydroxy-acidoxidizingprotein (L-lactate)	WP_036919442.:	273544..274716	Pyruvate metabolism	COG1304
lcl4.1_WP_036919442.1	CUST_8880_PI442182269	MIT0603	LLII/III	lcl4.1_WP_036919442.1	TTAGACAGTGTCGCTCCCACTATTCGTGTTCTTCCAGAAGTTGTAAAAGCTGTAAATGGG	lldD	alpha-hydroxy-acidoxidizingprotein (L-lactate)	WP_036919442.:	273544..274716	Pyruvate metabolism	COG1304
lcl4.1_WP_036919442.1	CUST_8881_PI442182269	MIT0603	LLII/III	lcl4.1_WP_036919442.1	TACTTGGAGGAAGAGATGTAGCATTAAAAACAATTGAGCGAGCCAAATCGGCAGGTTTTT	lldD	alpha-hydroxy-acidoxidizingprotein (L-lactate)	WP_036919442.:	273544..274716	Pyruvate metabolism	COG1304
lcl4.1_WP_036919442.1	CUST_8882_PI442182269	MIT0603	LLII/III	lcl4.1_WP_036919442.1	GGTGGAATTAGACGGGGAAGTGATGTTGTTAAAGCTTTATGTTTAGGAGCAAACGGTGTT	lldD	alpha-hydroxy-acidoxidizingprotein (L-lactate)	WP_036919442.:	273544..274716	Pyruvate metabolism	COG1304
WP_042849489.1_12	CUST_67_PI442190829	MIT0604	HLII	WP_042849489.1_12	CAGGAAGAGTGTATGGACATCTTCAGGCATTGTTAACTATGGTCAAAGGGGTACCACTTT	argH	Argininosuccinate lyase	WP_042849489.:	14513..15892	Amino Acid metabolism	COG0165
WP_042849489.1_12	CUST_68_PI442190829	MIT0604	HLII	WP_042849489.1_12	TGTCGTTAAATCAAGAAATAGCGAAGGCGGTACAGGTTTTGTTCAGGTAGAAAAAGAGCT	argH	Argininosuccinate lyase	WP_042849489.:	14513..15892	Amino Acid metabolism	COG0165
WP_042849489.1_12	CUST_69_PI442190829	MIT0604	HLII	WP_042849489.1_12	AGGAGCTGCAGCATTAGCAGGAACAAAAATAAAAATAGATAGGCATTTTACAGCTGCAGA	argH	Argininosuccinate lyase	WP_042849489.:	14513..15892	Amino Acid metabolism	COG0165
WP_042849489.1_12	CUST_70_PI442190829	MIT0604	HLII	WP_042849489.1_12	TCTAACGCTACCGATTTGGCAGATTACTTAGTTGGTAAAGATGTTCCTTTTAGGACTGCT	argH	Argininosuccinate lyase	WP_042849489.:	14513..15892	Amino Acid metabolism	COG0165

WP_042849489.1_12	CUST_71_PI442190829	MIT0604	HLII	WP_042849489.1_12	AATGTGCTACGGGAAGTAGTTTAATGCCTCAGAAAAAAAATCCTGATGTTCCAGAATTGA	argH	Argininosuccinate lyase	WP_042849489.:	14513..15892	Amino Acid metabolism	COG0165
WP_042849489.1_12	CUST_72_PI442190829	MIT0604	HLII	WP_042849489.1_12	GCAGTTAGCGATAGAGATTTTTGTATAGAATTTGTTTCTGCTTCTGCCTTGTCAATGTCA	argH	Argininosuccinate lyase	WP_042849489.:	14513..15892	Amino Acid metabolism	COG0165
WP_042849707.1_237	CUST_1295_PI442190829	MIT0604	HLII	WP_042849707.1_237	AAGAAGCAGGGGACTTATTATTAGAAGCAATTCAAAAAGCGGGATTTAAGCCTGGTGAGC	eno	Enolase	WP_042849707.:	227820..229112	Glycolysis/Gluconeogenesis	COG0148
WP_042849707.1_237	CUST_1296_PI442190829	MIT0604	HLII	WP_042849707.1_237	AAATCATTACTTGACCAAAAAGGTCTATCTACTGCTGTAGGCGATGAGGGTGGATTTGCA	eno	Enolase	WP_042849707.:	227820..229112	Glycolysis/Gluconeogenesis	COG0148
WP_042849707.1_237	CUST_1297_PI442190829	MIT0604	HLII	WP_042849707.1_237	TACAAGATCGGGTCAGATCAAGACAGGCTCTTTGAGCAGAAGTGAAAGGATTGCAAAATA	eno	Enolase	WP_042849707.:	227820..229112	Glycolysis/Gluconeogenesis	COG0148
WP_042849707.1_237	CUST_1298_PI442190829	MIT0604	HLII	WP_042849707.1_237	AGAGGACGGTTTAGCTGAGGATGATTGGGAGGGTTGGTCAGAATTAAACAAAGAATTAGG	eno	Enolase	WP_042849707.:	227820..229112	Glycolysis/Gluconeogenesis	COG0148
WP_042849707.1_237	CUST_1299_PI442190829	MIT0604	HLII	WP_042849707.1_237	GGCTTTTAAGAATTGAGGAGGAATTGGGAAATCAAGCAAGATTCGCTGGAGCTTTAGGTT	eno	Enolase	WP_042849707.:	227820..229112	Glycolysis/Gluconeogenesis	COG0148
WP_042849707.1_237	CUST_1300_PI442190829	MIT0604	HLII	WP_042849707.1_237	TCCGGTTTCACAAGTGTTATAAGTCATAGAAGTGGTGAGACTGAAGATACAACAATTGCA	eno	Enolase	WP_042849707.:	227820..229112	Glycolysis/Gluconeogenesis	COG0148
WP_042849722.1_254	CUST_1385_PI442190829	MIT0604	HLII	WP_042849722.1_254	TGGTCGGATTTTGTATCCGAAATCAAACTCAATTTTCCAAGAATTAATTTAGGCTCTGCC	eda	2-dehydro-3-deoxyphosphogluconate aldolase	WP_042849722.:	245716..246342	Entner-Dudoroff	COG0800
WP_042849722.1_254	CUST_1386_PI442190829	MIT0604	HLII	WP_042849722.1_254	ACTCAACTTTAAAAGTATTGATTTCATTGCTGCTGGAGGCCTATCAATCAATGATGTAAA	eda	2-dehydro-3-deoxyphosphogluconate aldolase	WP_042849722.:	245716..246342	Entner-Dudoroff	COG0800
WP_042849722.1_254	CUST_1387_PI442190829	MIT0604	HLII	WP_042849722.1_254	AGGATATAAAGCAATCGTGATTGGAGATAAAGGTATTATTAAAAAAAAATTTGACCCAAA	eda	2-dehydro-3-deoxyphosphogluconate aldolase	WP_042849722.:	245716..246342	Entner-Dudoroff	COG0800
WP_042849722.1_254	CUST_1388_PI442190829	MIT0604	HLII	WP_042849722.1_254	AATTCCAGGAATTAAAAATTTTAAAGATCTTGAGGAAGCGATAAATTTAAATTGCAACAT	eda	2-dehydro-3-deoxyphosphogluconate aldolase	WP_042849722.:	245716..246342	Entner-Dudoroff	COG0800
WP_042849722.1_254	CUST_1389_PI442190829	MIT0604	HLII	WP_042849722.1_254	AGGAAGCTTAGTAAAATTAGGATTAAAGAACATAGAAATAAGTTGGTCAAATAACGAAAA	eda	2-dehydro-3-deoxyphosphogluconate aldolase	WP_042849722.:	245716..246342	Entner-Dudoroff	COG0800
WP_042849722.1_254	CUST_1390_PI442190829	MIT0604	HLII	WP_042849722.1_254	AGCAATCAATAGAAGATTCGTTAAAAATTGGATTAAATTTTTCTATGATGAAATTTTGGG	eda	2-dehydro-3-deoxyphosphogluconate aldolase	WP_042849722.:	245716..246342	Entner-Dudoroff	COG0800
WP_042849815.1_351	CUST_1903_PI442190829	MIT0604	HLII	WP_042849815.1_351	AGAAATAAAATTCAAAAAAACTGATAAATCTGATAAAGCTAATAAAGATATCAAAAAGT	minE	septum site-determining protein MinE	WP_042849815.:	331757..332092	Cell Division	COG0851
WP_042849815.1_351	CUST_1904_PI442190829	MIT0604	HLII	WP_042849815.1_351	ACTGAGGATAGAATGACCGCATTAGTTGCCAATTTACCAATAAAAGAACCATTTCAGGA	minE	septum site-determining protein MinE	WP_042849815.:	331757..332092	Cell Division	COG0851
WP_042849815.1_351	CUST_1905_PI442190829	MIT0604	HLII	WP_042849815.1_351	AGTTCCTTAACAACTGATCTTTTAGATAAAATGAGGAAAGAGATTCTTGATGTTGTTGCT	minE	septum site-determining protein MinE	WP_042849815.:	331757..332092	Cell Division	COG0851
WP_042849815.1_351	CUST_1906_PI442190829	MIT0604	HLII	WP_042849815.1_351	AACTTCTAGTGCAAATACAGCAAGAGAAAGATTACAACTTGTACTTGCTCATGACAGGGT	minE	septum site-determining protein MinE	WP_042849815.:	331757..332092	Cell Division	COG0851
WP_042849816.1_352	CUST_1907_PI442190829	MIT0604	HLII	WP_042849816.1_352	CAAAGGAGGGGTTGGTAAAACAACTTTAACTGCAAACCTAGGGATAGCACTTGCCAATAG	minD	septum site-determining protein MinD	WP_042849816.:	332094..332909	Cell Division	COG2894
WP_042849816.1_352	CUST_1908_PI442190829	MIT0604	HLII	WP_042849816.1_352	TTTTGTGTTAGTAGATTGTCCCGCAGGTGTAGAAGATGGCTTTAAAAATGCTCTTGCAGC	minD	septum site-determining protein MinD	WP_042849816.:	332094..332909	Cell Division	COG2894
WP_042849816.1_352	CUST_1909_PI442190829	MIT0604	HLII	WP_042849816.1_352	ATCCGCAGTGCGGGATGCGGATAGAGTAATAGGGATTCTTAATACATCAGATATAGAGCC	minD	septum site-determining protein MinD	WP_042849816.:	332094..332909	Cell Division	COG2894
WP_042849816.1_352	CUST_1910_PI442190829	MIT0604	HLII	WP_042849816.1_352	TACCTGCTGGAGATCCAAGGATGTTGGATTGGATGAAGCCCGAGGATATGAAAAAAATTA	minD	septum site-determining protein MinD	WP_042849816.:	332094..332909	Cell Division	COG2894
WP_042849816.1_352	CUST_1911_PI442190829	MIT0604	HLII	WP_042849816.1_352	TCGCCCAAACATGATGGCTAGTCAAGAAATGTTATCTATCGAAGATGTTCAAGGAATCCT	minD	septum site-determining protein MinD	WP_042849816.:	332094..332909	Cell Division	COG2894
WP_042849816.1_352	CUST_1912_PI442190829	MIT0604	HLII	WP_042849816.1_352	AGTACAAATAGAGGAGAGCCACTGACACTAACAGATGGAAGATCTCCTGCAAAAAAATGT	minD	septum site-determining protein MinD	WP_042849816.:	332094..332909	Cell Division	COG2894
WP_042849817.1_353	CUST_1913_PI442190829	MIT0604	HLII	WP_042849817.1_353	AAATTACTAGGGATCGCATTCGCAGGGAAAAGTGGCAATAAAATGCCTCTATTGCATCA	minC	septum site-determining protein MinC	WP_042849817.:	333021..333683	Cell Division	COG0850
WP_042849817.1_353	CUST_1914_PI442190829	MIT0604	HLII	WP_042849817.1_353	CTAAACCCTTTACAGTTAAGAATTGCTGATGTGATAGCTATTGGGCCAAAGGATAAGCCT	minC	septum site-determining protein MinC	WP_042849817.:	333021..333683	Cell Division	COG0850
WP_042849817.1_353	CUST_1915_PI442190829	MIT0604	HLII	WP_042849817.1_353	TTCTTCACAAAGGAACAGTACGATCGGGTGAAAGAATATCTTCAAATGGAAACCTATGCA	minC	septum site-determining protein MinC	WP_042849817.:	333021..333683	Cell Division	COG0850
WP_042849817.1_353	CUST_1916_PI442190829	MIT0604	HLII	WP_042849817.1_353	TTCCATCAAGGGGCCTTTAGAAGCAAAAATTATTGCAATCAATGAATCAATAAGTTCTCA	minC	septum site-determining protein MinC	WP_042849817.:	333021..333683	Cell Division	COG0850
WP_042849817.1_353	CUST_1917_PI442190829	MIT0604	HLII	WP_042849817.1_353	ATTGGAGATGTTAATCCAGGAGCAATAGTTTCAGCTAATAAAAATATTTACGTTTGGGGC	minC	septum site-determining protein MinC	WP_042849817.:	333021..333683	Cell Division	COG0850
WP_042849817.1_353	CUST_1918_PI442190829	MIT0604	HLII	WP_042849817.1_353	CCAGAAATTGCTGTCATATATAAACAAACGATAATTATCAAACCACATCTAATCGAAAGT	minC	septum site-determining protein MinC	WP_042849817.:	333021..333683	Cell Division	COG0850
WP_042849909.1_459	CUST_2459_PI442190829	MIT0604	HLII	WP_042849909.1_459	TGCGGACGGAATGACTTTTGCTGGATTCTTTGCCGCTTATTTAACTTATAAAGCAGTCAA	cyoC	heme-copper oxidase subunit III	WP_042849909.:	425765..426367	Respiration	COG1845
WP_042849909.1_459	CUST_2460_PI442190829	MIT0604	HLII	WP_042849909.1_459	TGGCAATCAAGAATCAAGGGCGGAAGATTAACTAGTCAAAATATGTTCCCTCTGGAAGCT	cyoC	heme-copper oxidase subunit III	WP_042849909.:	425765..426367	Respiration	COG1845
WP_042849909.1_459	CUST_2461_PI442190829	MIT0604	HLII	WP_042849909.1_459	TGCAAGCACTTTTTATGCTCTTACTGGTTTTCATGGATTACATGTCACTTTAGGCACTTT	cyoC	heme-copper oxidase subunit III	WP_042849909.:	425765..426367	Respiration	COG1845
WP_042849909.1_459	CUST_2462_PI442190829	MIT0604	HLII	WP_042849909.1_459	AGTGCAACTTTCCATAAAGCAGGCAAAGCACTTTTAAAAGATAAAAACTCTGAATCCCAA	cyoC	heme-copper oxidase subunit III	WP_042849909.:	425765..426367	Respiration	COG1845
WP_042849909.1_459	CUST_2463_PI442190829	MIT0604	HLII	WP_042849909.1_459	TTCTGAAGTCAACGAAACACACGAAGATTTCAGAATGTTTGGTCTTATAACTTTCCTAAT	cyoC	heme-copper oxidase subunit III	WP_042849909.:	425765..426367	Respiration	COG1845
WP_042849909.1_459	CUST_2464_PI442190829	MIT0604	HLII	WP_042849909.1_459	TCCATTACCTGATGGTGCTATTTATGAATTAGAACTACCAATACCTACACTCAATACAAT	cyoC	heme-copper oxidase subunit III	WP_042849909.:	425765..426367	Respiration	COG1845
WP_042849910.1_460	CUST_2465_PI442190829	MIT0604	HLII	WP_042849910.1_460	AGAGTTGCAGAATATGATCCTCAATTCCAGTTCGTCAATCAAATTAGTAGCCTTGGGGCT	cyoB/coxA	Cytochrome c oxidase subunit I	WP_042849910.:	426374..427999	Respiration	COG0843
WP_042849910.1_460	CUST_2466_PI442190829	MIT0604	HLII	WP_042849910.1_460	TTCGTTTTTGGAGGTATTACAGGAGTTGCTTTGGCACAGGTACCTTTCGATATTCACGTA	cyoB/coxA	Cytochrome c oxidase subunit I	WP_042849910.:	426374..427999	Respiration	COG0843
WP_042849910.1_460	CUST_2467_PI442190829	MIT0604	HLII	WP_042849910.1_460	CTCCAGTTGAAAATTGGGAAGGACAAGCTCCACTAGTTGAAGAACCTTATGGTTATGGGA	cyoB/coxA	Cytochrome c oxidase subunit I	WP_042849910.:	426374..427999	Respiration	COG0843
WP_042849910.1_460	CUST_2468_PI442190829	MIT0604	HLII	WP_042849910.1_460	AAGATGCTGGAGATAACCCTTGGAATGCTCTTACACCTGAATGGTTAACATCTTCTCCAC	cyoB/coxA	Cytochrome c oxidase subunit I	WP_042849910.:	426374..427999	Respiration	COG0843
WP_042849910.1_460	CUST_2469_PI442190829	MIT0604	HLII	WP_042849910.1_460	ATGGGGATAGTAGTTTTAGGTTTAGTTGTTTGGGCGCATCACATGTTTACAAGTGGAACG	cyoB/coxA	Cytochrome c oxidase subunit I	WP_042849910.:	426374..427999	Respiration	COG0843
WP_042849910.1_460	CUST_2470_PI442190829	MIT0604	HLII	WP_042849910.1_460	ATTGGATTTAACTTGTGCTTTGCTCCTCAACATTGGCTTGGTTTAAATGGAATGCCAAGA	cyoB/coxA	Cytochrome c oxidase subunit I	WP_042849910.:	426374..427999	Respiration	COG0843
WP_042849911.1_461	CUST_2471_PI442190829	MIT0604	HLII	WP_042849911.1_461	GTGGCCCATATCATGGAGGGATGAGAGCCTCCATAATTGTTGAAGAAGAATCTGATTACA	ctaC/coxB	Cytochrome c oxidase subunit II	WP_042849911.:	427996..428799	Respiration	COG1622
WP_042849911.1_461	CUST_2472_PI442190829	MIT0604	HLII	WP_042849911.1_461	ATTCATGCTTTTTGGGTGCCAGAGTTCAGAATTAAACAGGATATTATTCCTGGACAGCCT	ctaC/coxB	Cytochrome c oxidase subunit II	WP_042849911.:	427996..428799	Respiration	COG1622
WP_042849911.1_461	CUST_2473_PI442190829	MIT0604	HLII	WP_042849911.1_461	TCTTTTTGGATTGGTTTTAATGTAAATTTGCTCCCAGCGGAAGCGAGTATTAATGCACCA	ctaC/coxB	Cytochrome c oxidase subunit II	WP_042849911.:	427996..428799	Respiration	COG1622
WP_042849911.1_461	CUST_2474_PI442190829	MIT0604	HLII	WP_042849911.1_461	AGCTACAACATCTACGATCGAATGGGAGGGATGAAAGAACTAAATCATAACCACGAAATG	ctaC/coxB	Cytochrome c oxidase subunit II	WP_042849911.:	427996..428799	Respiration	COG1622
WP_042849911.1_461	CUST_2475_PI442190829	MIT0604	HLII	WP_042849911.1_461	TCCCAAGGGCAATTTTATATCTGGAGAACTACATGTTCCTGTTGATCAAAAAGTATCAAT	ctaC/coxB	Cytochrome c oxidase subunit II	WP_042849911.:	427996..428799	Respiration	COG1622
WP_042849911.1_461	CUST_2476_PI442190829	MIT0604	HLII	WP_042849911.1_461	AGCTTATTTAAGTTTAGGAAAAGAAATGATCAGATAGGAGATGGTATAGCTCTAGAGGGA	ctaC/coxB	Cytochrome c oxidase subunit II	WP_042849911.:	427996..428799	Respiration	COG1622
WP_042849917.1_469	CUST_2513_PI442190829	MIT0604	HLII	WP_042849917.1_469	GCTGGCATTACGAGAGATGGACTATTGATGAGAATGAAATCGGAGCAATGGGATGATGTA	gdh	3-oxoacyl-acyl-carrier-protein reductase	WP_042849917.:	435415..436164	Entner-Dudoroff	COG1028
WP_042849917.1_469	CUST_2514_PI442190829	MIT0604	HLII	WP_042849917.1_469	AATCACTGGAGCTAGTAGAGGAATTGGTAAAGGAATTGCCTTTGAACTAAGCCGCTTAGG	gdh	3-oxoacyl-acyl-carrier-protein reductase	WP_042849917.:	435415..436164	Entner-Dudoroff	COG1028
WP_042849917.1_469	CUST_2515_PI442190829	MIT0604	HLII	WP_042849917.1_469	TGTTGTTGGAATAATTGGTAATCCAGGGCAAGCAAATTATTCTGCTGCTAAAGCTGGAGT	gdh	3-oxoacyl-acyl-carrier-protein reductase	WP_042849917.:	435415..436164	Entner-Dudoroff	COG1028
WP_042849917.1_469	CUST_2516_PI442190829	MIT0604	HLII	WP_042849917.1_469	TTCTTAGTTTCAAGCGATGCAGGAAAGTACATTACAGGGCAAACAATCAGTATTGACGGG	gdh	3-oxoacyl-acyl-carrier-protein reductase	WP_042849917.:	435415..436164	Entner-Dudoroff	COG1028
WP_042849917.1_469	CUST_2517_PI442190829	MIT0604	HLII	WP_042849917.1_469	ACTTGCGCTAAAGAATTTGCTTCAAGAGGTATAAACGTAAATGCAATAGCTCCAGGCTTC	gdh	3-oxoacyl-acyl-carrier-protein reductase	WP_042849917.:	435415..436164	Entner-Dudoroff	COG1028
WP_042849917.1_469	CUST_2518_PI442190829	MIT0604	HLII	WP_042849917.1_469	TTTCAAAAGAGGAATCTGTTAGTTCAGCTTTTGAAGAAATCATAAAAATTAATGGCACGA	gdh	3-oxoacyl-acyl-carrier-protein reductase	WP_042849917.:	435415..436164	Entner-Dudoroff	COG1028
WP_042851291.1_535	CUST_2871_PI442190829	MIT0604	HLII	WP_042851291.1_535	GTTGACGCAAGACTGAGCTTTGACTCCGAAGCTACGGTAGAAAAAGCAAGAAAATTGATC	tal	Transaldolase	WP_042851291.:	492474..493475	Pentose Phosphate	COG0176
WP_042851291.1_535	CUST_2872_PI442190829	MIT0604	HLII	WP_042851291.1_535	CGGTAACTTGTGCCAATGCAAAGATAACTCTAATTTCTCCGTTCGTTGGCCGTATATTGG	tal	Transaldolase	WP_042851291.:	492474..493475	Pentose Phosphate	COG0176
WP_042851291.1_535	CUST_2873_PI442190829	MIT0604	HLII	WP_042851291.1_535	TAAGATTGCTGCAACTTGGGAGGGAATTAAGGCAGCTGAAATTTTGGAAAAAGAGGGTAT	tal	Transaldolase	WP_042851291.:	492474..493475	Pentose Phosphate	COG0176
WP_042851291.1_535	CUST_2874_PI442190829	MIT0604	HLII	WP_042851291.1_535	AAACTGGTAAGACTAGTTTTGTTGGTGCTGAAGATCCTGGTGTTATTTCGGTTACGCAAA	tal	Transaldolase	WP_042851291.:	492474..493475	Pentose Phosphate	COG0176
WP_042851291.1_535	CUST_2875_PI442190829	MIT0604	HLII	WP_042851291.1_535	AGGTTGCGATCTTTTAACAATCGCACCAAAATTTCTTGAGGAACTGAAAAAAGAAAAGGG	tal	Transaldolase	WP_042851291.:	492474..493475	Pentose Phosphate	COG0176
WP_042851291.1_535	CUST_2876_PI442190829	MIT0604	HLII	WP_042851291.1_535	AAAGCTTAGTGAAGGTATTACTGGATTCAGTAAGGCTATAGAAGAATTGGAAGAGCTGCT	tal	Transaldolase	WP_042851291.:	492474..493475	Pentose Phosphate	COG0176
WP_011376108.1_567	CUST_3054_PI442190829	MIT0604	HLII	WP_011376108.1_567	ACCCTGATCATCATGTAAGAATCATCGGATACGATGCTTACACTCAAAGCCAAGGAACAG	rbcS	Ribulose bisphosphate carboxylase small subunit	WP_011376108.:	522365..522706	Carbon fixation	COG4451
WP_011376108.1_567	CUST_3055_PI442190829	MIT0604	HLII	WP_011376108.1_567	TGGAAGCTCCCATTCTTTGGGGAAAAAGACCTTAACTTGGTTGTAAGTGAATTAGAGGCA	rbcS	Ribulose bisphosphate carboxylase small subunit	WP_011376108.:	522365..522706	Carbon fixation	COG4451
WP_011376108.1_567	CUST_3056_PI442190829	MIT0604	HLII	WP_011376108.1_567	ATAATTGCTCAAGGCTGGAGTCCTGTTATTGAGCATGTTCATCCTAGTGGAAGTATGCAA	rbcS	Ribulose bisphosphate carboxylase small subunit	WP_011376108.:	522365..522706	Carbon fixation	COG4451
WP_011376108.1_567	CUST_3057_PI442190829	MIT0604	HLII	WP_011376108.1_567	TGGAAACATTCGGTTTCTTACCACCGATGACCCAGGAGGAAATATACGATCAAATTGCAT	rbcS	Ribulose bisphosphate carboxylase small subunit	WP_011376108.:	522365..522706	Carbon fixation	COG4451
WP_042850043.1_612	CUST_3293_PI442190829	MIT0604	HLII	WP_042850043.1_612	GTTCTAGCCAGCGAAGGAGGACACGTTGAATACTCTCCAAAGTCAAAATTAGAATGGGAA	glk	Glucokinase	WP_042850043.:	566823..567857	Glycolysis	COG0837
WP_042850043.1_612	CUST_3294_PI442190829	MIT0604	HLII	WP_042850043.1_612	TCCAAAATCGATCTCATTCTAAAGGCACTAACAATGATTTGCATGCCATAGTTGGAGCCG	glk	Glucokinase	WP_042850043.:	566823..567857	Glycolysis	COG0837
WP_042850043.1_612	CUST_3295_PI442190829	MIT0604	HLII	WP_042850043.1_612	TGAAGTTGAGAGTATTTGGTTAGGCGCTTATGCCTCTTTATTGGGAGATGTTGCTCTTCA	glk	Glucokinase	WP_042850043.:	566823..567857	Glycolysis	COG0837
WP_042850043.1_612	CUST_3296_PI442190829	MIT0604	HLII	WP_042850043.1_612	GGTTTATCAAGAATTGCCGAATGGAGACTAAGCAAACCTGATGCCCAAAACCATCCTTTA	glk	Glucokinase	WP_042850043.:	566823..567857	Glycolysis	COG0837
WP_042850043.1_612	CUST_3297_PI442190829	MIT0604	HLII	WP_042850043.1_612	GCTTTGGCGGGTTATGGATTTCTGGAGGAACCGCATCAAAGCATTTCAAAACTTTAAAT	glk	Glucokinase	WP_042850043.:	566823..567857	Glycolysis	COG0837
WP_042850043.1_612	CUST_3298_PI442190829	MIT0604	HLII	WP_042850043.1_612	TGAAAGTAATTTTAGATGAAGAGTTTGGACTTTTTAGTGCAGCCTGCAGAGCAAAAATGC	glk	Glucokinase	WP_042850043.:	566823..567857	Glycolysis	COG0837
WP_042850235.1_806	CUST_4298_PI442190829	MIT0604	HLII	WP_042850235.1_806	TTCAGTGAAAAGCCCACTGGAGAGAAGTTGAAGGCAATGGCAGTTGATACTTCAAAATTT	glgC	Glucose-1-phosphate adenylyltransferase	WP_042850235.:	741186..742481	Glycogen Metabolism	COG0448
WP_042850235.1_806	CUST_4299_PI442190829	MIT0604	HLII	WP_042850235.1_806	AGGACACTCTTGTTATGGGTGCCGATTTCTTTGAATCGCCTGAAGAGAGGATTGAATTAA	glgC	Glucose-1-phosphate adenylyltransferase	WP_042850235.:	741186..742481	Glycogen Metabolism	COG0448
WP_042850235.1_806	CUST_4300_PI442190829	MIT0604	HLII	WP_042850235.1_806	AGGAACACCTCTCGGAGTAGGGGAAGGAACCACTGTAAAAAGAGCAATTCTTGATAAGAA	glgC	Glucose-1-phosphate adenylyltransferase	WP_042850235.:	741186..742481	Glycogen Metabolism	COG0448
WP_042850235.1_806	CUST_4301_PI442190829	MIT0604	HLII	WP_042850235.1_806	TGTATTCGATGATTACTGGGAAGATATCGGTACCATTGGGGCATTCTTTGAGTCAAACCT	glgC	Glucose-1-phosphate adenylyltransferase	WP_042850235.:	741186..742481	Glycogen Metabolism	COG0448
WP_042850235.1_806	CUST_4302_PI442190829	MIT0604	HLII	WP_042850235.1_806	TTCCTAGTTATACAGATTTTGGTAAGGATATAATTCCTGAAGCCCTCAATAGGGGGGACA	glgC	Glucose-1-phosphate adenylyltransferase	WP_042850235.:	741186..742481	Glycogen Metabolism	COG0448
WP_042850235.1_806	CUST_4303_PI442190829	MIT0604	HLII	WP_042850235.1_806	AGAGTCAGCTACTGAAAAACCCTATCTAGCCTCTATGGGTATTTACGTTTTTAGCAGAAA	glgC	Glucose-1-phosphate adenylyltransferase	WP_042850235.:	741186..742481	Glycogen Metabolism	COG0448
WP_042850236.1_807	CUST_4304_PI442190829	MIT0604	HLII	WP_042850236.1_807	CCAATAGACTTCCTCAGAATCTTGTGCAGGCAATGAGAGACTGTTTTGGCTCTCATACAT	edd	phosphogluconate dehydrogenase (NADP(+)-dependent, decarboxyla	WP_042850236.:	742578..743996	Pentose Phosphate/Entner-Dudorof	COG2084
WP_042850236.1_807	CUST_4305_PI442190829	MIT0604	HLII	WP_042850236.1_807	GCTAAGGTAGTTTCTCTATCCACAAAGGCAGGTATACCAGTCCCATGTTTATCCAGTACT	edd	phosphogluconate dehydrogenase (NADP(+)-dependent, decarboxyla	WP_042850236.:	742578..743996	Pentose Phosphate/Entner-Dudorof	COG2084
WP_042850236.1_807	CUST_4306_PI442190829	MIT0604	HLII	WP_042850236.1_807	GCAGTATATAACTATGGTTTGAATATGCCGTCAATTGCACAAATATGGAAAGGTGGTTGC	edd	phosphogluconate dehydrogenase (NADP(+)-dependent, decarboxyla	WP_042850236.:	742578..743996	Pentose Phosphate/Entner-Dudorof	COG2084
WP_042850236.1_807	CUST_4307_PI442190829	MIT0604	HLII	WP_042850236.1_807	ATGAAACCTTTATTTGATGCTGTAGTCCTTGCCACAATTGCTAGCTATGCCCAAGGTATG	edd	phosphogluconate dehydrogenase (NADP(+)-dependent, decarboxyla	WP_042850236.:	742578..743996	Pentose Phosphate/Entner-Dudorof	COG2084
WP_042850236.1_807	CUST_4308_PI442190829	MIT0604	HLII	WP_042850236.1_807	TGTGAGTGCTCTAGAGCTGGGGGTATCAGTCCCAACTATTTATGCATCTTTAAATGCAAG	edd	phosphogluconate dehydrogenase (NADP(+)-dependent, decarboxyla	WP_042850236.:	742578..743996	Pentose Phosphate/Entner-Dudorof	COG2084
WP_042850236.1_807	CUST_4309_PI442190829	MIT0604	HLII	WP_042850236.1_807	TGCAGAAGCTTACGACCTTATGAAGAGAGTCAAAGGTATGAATGGAGAGCAGATGTCAGA	edd	phosphogluconate dehydrogenase (NADP(+)-dependent, decarboxyla	WP_042850236.:	742578..743996	Pentose Phosphate/Entner-Dudorof	COG2084
WP_042850244.1_819	CUST_4372_PI442190829	MIT0604	HLII	WP_042850244.1_819	GAGAATGCTTGGGGATTAGCAAGGTATGCAAGATCAGTTCAAGAATCTGGGTTAGTACCA	fda	Fructose-1,6-bisphosphate aldolase class I	WP_042850244.:	752482..753549	Glycolysis/Gluconeogenesis	COG0191
WP_042850244.1_819	CUST_4373_PI442190829	MIT0604	HLII	WP_042850244.1_819	GAAGGTTGCTGAAATGACAATTAGAACTATGGAAAGATGCGTTCCTGCATCTGTTCCTGG	fda	Fructose-1,6-bisphosphate aldolase class I	WP_042850244.:	752482..753549	Glycolysis/Gluconeogenesis	COG0191
WP_042850244.1_819	CUST_4374_PI442190829	MIT0604	HLII	WP_042850244.1_819	AAGGTCGATAAGGGTCTAAATCCACTTCCAGGAGGAGGAGATGTAGAAACTTTTTGCTCT	fda	Fructose-1,6-bisphosphate aldolase class I	WP_042850244.:	752482..753549	Glycolysis/Gluconeogenesis	COG0191
WP_042850244.1_819	CUST_4375_PI442190829	MIT0604	HLII	WP_042850244.1_819	TATGGAATGTTTCATTCTCCTATGGAAGAGCATTACAGCACTCATGCTTAAAGGCCTGGA	fda	Fructose-1,6-bisphosphate aldolase class I	WP_042850244.:	752482..753549	Glycolysis/Gluconeogenesis	COG0191
WP_042850244.1_819	CUST_4376_PI442190829	MIT0604	HLII	WP_042850244.1_819	TTAATAGCAAGAGCTCAAGCAAACTCTGAAGCTTCAAAAGGTGCCTATGTAGCAGGATCT	fda	Fructose-1,6-bisphosphate aldolase class I	WP_042850244.:	752482..753549	Glycolysis/Gluconeogenesis	COG0191
WP_042850244.1_819	CUST_4377_PI442190829	MIT0604	HLII	WP_042850244.1_819	GGCCTAGATGGATTAGTTGAAAGAGCCGCAAAATATTATGAACAAGGTGCCAGATTTGCA	fda	Fructose-1,6-bisphosphate aldolase class I	WP_042850244.:	752482..753549	Glycolysis/Gluconeogenesis	COG0191
WP_042850248.1_823	CUST_4396_PI442190829	MIT0604	HLII	WP_042850248.1_823	CGTACTTTTTTATGAAGGTTTGCATGGCGGGGTAGAAGGTGATGGATATAATGTGGCATC	prk	Phosphoribulokinase	WP_042850248.:	756168..757064	Carbon Fixation	COG3954
WP_042850248.1_823	CUST_4397_PI442190829	MIT0604	HLII	WP_042850248.1_823	TAGAAGGCGATAGTTACCACAGATTTGAGAGAATGCCTATGAAAAAAGCTATGGCGGACG	prk	Phosphoribulokinase	WP_042850248.:	756168..757064	Carbon Fixation	COG3954
WP_042850248.1_823	CUST_4398_PI442190829	MIT0604	HLII	WP_042850248.1_823	CTCCTGATGAGAGCTTTGTGATCATACATTTCAGAAAAGGGGGCAAGAGAGAAATGGGGTA	prk	Phosphoribulokinase	WP_042850248.:	756168..757064	Carbon Fixation	COG3954
WP_042850248.1_823	CUST_4399_PI442190829	MIT0604	HLII	WP_042850248.1_823	CAGAAGAACATAATACAAGACTTGGGACATCCCTAGAACCTGGGCAATTTACTCCATGGG	prk	Phosphoribulokinase	WP_042850248.:	756168..757064	Carbon Fixation	COG3954
WP_042850248.1_823	CUST_4400_PI442190829	MIT0604	HLII	WP_042850248.1_823	TAACGCAGAAAGAGGTTACTCAGCGGAAACCATTGTGGATACGATATTGAGAAGAATGCC	prk	Phosphoribulokinase	WP_042850248.:	756168..757064	Carbon Fixation	COG3954
WP_042850248.1_823	CUST_4401_PI442190829	MIT0604	HLII	WP_042850248.1_823	CATGTCAAGTCCAACAAGTATCGTTGTAAATGGTGGAAAAATGGGATTCGCAATGGAACT	prk	Phosphoribulokinase	WP_042850248.:	756168..757064	Carbon Fixation	COG3954
WP_042850322.1_899	CUST_4803_PI442190829	MIT0604	HLII	WP_042850322.1_899	TGAAGACTGGAGGTAATTGGTGCAGAATTAATCGCCTTGATAACAGAGGAGTGTTTCGATG	pdhB	Pyruvate dehydrogenase E1 component beta subunit	WP_042850322.:	820416..821399	Glycolysis/Krebs cycle	COG0022
WP_042850322.1_899	CUST_4804_PI442190829	MIT0604	HLII	WP_042850322.1_899	ACAACTTGTCTGAAGAATTGCCTGAGGGTGATTATACTTGTGCTTTAGATCAGGCTGACA	pdhB	Pyruvate dehydrogenase E1 component beta subunit	WP_042850322.:	820416..821399	Glycolysis/Krebs cycle	COG0022
WP_042850322.1_899	CUST_4805_PI442190829	MIT0604	HLII	WP_042850322.1_899	CGGAGGAAATTATAAAATACCAGCAGTAGTTAGAGGACCTGGAGGAGTTGGTCGTCAACT	pdhB	Pyruvate dehydrogenase E1 component beta subunit	WP_042850322.:	820416..821399	Glycolysis/Krebs cycle	COG0022
WP_042850322.1_899	CUST_4806_PI442190829	MIT0604	HLII	WP_042850322.1_899	AAGACTTGAAGCCTATTTTCATGCAGTTCCTGGTATAAAGATTGTTGCGTGCAGTACACC	pdhB	Pyruvate dehydrogenase E1 component beta subunit	WP_042850322.:	820416..821399	Glycolysis/Krebs cycle	COG0022
WP_042850322.1_899	CUST_4807_PI442190829	MIT0604	HLII	WP_042850322.1_899	GCAATGACTGGGTTAAGACCAATAGTAGAAGGAATGAATATGGGTTTTTTGCTATTAGCT	pdhB	Pyruvate dehydrogenase E1 component beta subunit	WP_042850322.:	820416..821399	Glycolysis/Krebs cycle	COG0022
WP_042850322.1_899	CUST_4808_PI442190829	MIT0604	HLII	WP_042850322.1_899	TGCTAAGGGTTTAATGAAAGCAGCTATAAGAGATGATAATCCGGTTCTATTTTTCGAACA	pdhB	Pyruvate dehydrogenase E1 component beta subunit	WP_042850322.:	820416..821399	Glycolysis/Krebs cycle	COG0022
WP_042850333.1_910	CUST_4860_PI442190829	MIT0604	HLII	WP_042850333.1_910	TGGTGAAGGGGCATATGCAAATTTATCTCAAACAGCAAGATGGTACATCGGAGGTATACT	glnA	Glutamine synthetase/ glutamate--ammonialigase	WP_042850333.:	829172..830593	Nitrogen Metabolism	COG0174
WP_042850333.1_910	CUST_4861_PI442190829	MIT0604	HLII	WP_042850333.1_910	AACTGCAACGTTTATGCCCAAGCCTGTATTTAACGATAATGGTACTGGAATGCATGTTCA	glnA	Glutamine synthetase/ glutamate--ammonialigase	WP_042850333.:	829172..830593	Nitrogen Metabolism	COG0174
WP_042850333.1_910	CUST_4862_PI442190829	MIT0604	HLII	WP_042850333.1_910	TAACTGGACCAAGCCCTAAAGCTAAAAGATTAGAATTCAGATCAGGTGACGCACTTGCAA	glnA	Glutamine synthetase/ glutamate--ammonialigase	WP_042850333.:	829172..830593	Nitrogen Metabolism	COG0174
WP_042850333.1_910	CUST_4863_PI442190829	MIT0604	HLII	WP_042850333.1_910	TCCAGGATTCGAAGCACCTGTAAATCTAGTTTATTCTGAGGGTAATAGATCAGCTGCCGT	glnA	Glutamine synthetase/ glutamate--ammonialigase	WP_042850333.:	829172..830593	Nitrogen Metabolism	COG0174
WP_042850333.1_910	CUST_4864_PI442190829	MIT0604	HLII	WP_042850333.1_910	TCACCATGAAGTTGCTGGTGCAGGTCAACACGAACTTGGAATGAAATTTGATTCGTTAAT	glnA	Glutamine synthetase/ glutamate--ammonialigase	WP_042850333.:	829172..830593	Nitrogen Metabolism	COG0174
WP_042850333.1_910	CUST_4865_PI442190829	MIT0604	HLII	WP_042850333.1_910	TGATCCTGGTGATGGAGTAGACGTAGATTTATTTGAACTTCCAGCTGATGAACTAGCAAA	glnA	Glutamine synthetase/ glutamate--ammonialigase	WP_042850333.:	829172..830593	Nitrogen Metabolism	COG0174
WP_042850341.1_918	CUST_4905_PI442190829	MIT0604	HLII	WP_042850341.1_918	TGCTATTAGTGCGGCAGTAAGTAACATAGCAAGACAACTTGATGCAGGAGCTATAATCCC	pykF	Pyruvate kinase	WP_042850341.:	837814..839607	Glycolysis	COG0469
WP_042850341.1_918	CUST_4906_PI442190829	MIT0604	HLII	WP_042850341.1_918	GATGGCAACCATCGCAAGAAGAATTGAAAGGGATTATCCACTTAAGGCTATTGAAAGCCA	pykF	Pyruvate kinase	WP_042850341.:	837814..839607	Glycolysis	COG0469
WP_042850341.1_918	CUST_4907_PI442190829	MIT0604	HLII	WP_042850341.1_918	GTAAGTAAGTTCAGACCACCAACCACTTCATCTTGGCAACTACTACAGAGAGGAGTGTAGCG	pykF	Pyruvate kinase	WP_042850341.:	837814..839607	Glycolysis	COG0469
WP_042850341.1_918	CUST_4908_PI442190829	MIT0604	HLII	WP_042850341.1_918	AGCTCAGGAGATGGGAATCCTAAATCAAGGAGATTTAGTAGTTCAAACCGCAGGTACATT	pykF	Pyruvate kinase	WP_042850341.:	837814..839607	Glycolysis	COG0469
WP_042850341.1_918	CUST_4909_PI442190829	MIT0604	HLII	WP_042850341.1_918	AGAAAGATTGTATCAAGAGGAATTTCAATAGGGGAAATCGGTGTTACGGGTAAAGCAAGA	pykF	Pyruvate kinase	WP_042850341.:	837814..839607	Glycolysis	COG0469
WP_042850341.1_918	CUST_4910_PI442190829	MIT0604	HLII	WP_042850341.1_918	TGGCGACCTTATTACACTCCAGCTTAATGAAGGTGTTATATACATGGGACAAATTGAAGA	pykF	Pyruvate kinase	WP_042850341.:	837814..839607	Glycolysis	COG0469
WP_042850366.1_940	CUST_5023_PI442190829	MIT0604	HLII	WP_042850366.1_940	CACTATTGTCATCCGCATGGTATTTAACTGGGGAGGAATTGGAAAGAGAGATATGGTCG	pgi	Glucose-6-phosphate isomerase	WP_042850366.:	858488..860071	Glycolysis/Gluconeogenesis	COG0166
WP_042850366.1_940	CUST_5024_PI442190829	MIT0604	HLII	WP_042850366.1_940	TTTCACTAGGTGCCTTAATCGCTTTATTTGAGAGGGCTGTATCCTTCTACGCTGAATTGG	pgi	Glucose-6-phosphate isomerase	WP_042850366.:	858488..860071	Glycolysis/Gluconeogenesis	COG0166
WP_042850366.1_940	CUST_5025_PI442190829	MIT0604	HLII	WP_042850366.1_940	ATGCTTATGATCAACCTGGAGTTGAAGCTGGAAAGAAAGCAGCCGCAAATATTATTGAGT	pgi	Glucose-6-phosphate isomerase	WP_042850366.:	858488..860071	Glycolysis/Gluconeogenesis	COG0166
WP_042850366.1_940	CUST_5026_PI442190829	MIT0604	HLII	WP_042850366.1_940	AGAGGTGCATCATTAATGGATGAGGCCACACGTATAAGTGATTTTAAAAATAATCCAGCA	pgi	Glucose-6-phosphate isomerase	WP_042850366.:	858488..860071	Glycolysis/Gluconeogenesis	COG0166
WP_042850366.1_940	CUST_5027_PI442190829	MIT0604	HLII	WP_042850366.1_940	TCAAGGTATTTCCGTTTTTGGTAATAAAGGATCTACAGATCAACATGCTTATGTTCAGCA	pgi	Glucose-6-phosphate isomerase	WP_042850366.:	858488..860071	Glycolysis/Gluconeogenesis	COG0166
WP_042850366.1_940	CUST_5028_PI442190829	MIT0604	HLII	WP_042850366.1_940	AGGAACCAGATCAGCACTCTCTAGTGAAAACAGACAAAGTATCACTATTACGTTAGAAAA	pgi	Glucose-6-phosphate isomerase	WP_042850366.:	858488..860071	Glycolysis/Gluconeogenesis	COG0166
WP_042850517.1_1100	CUST_5787_PI442190829	MIT0604	HLII	WP_042850517.1_1100	GTCATTTCTCATTCTGAAAAGTCCCTTGAGTATCCAGCTGGTGGTGGATCTATTATCGCG	rbsK	Sugar kinase, ribokinase family	WP_042850517.:	985792..986655	Pentose Phosphate	COG0524
WP_042850517.1_1100	CUST_5788_PI442190829	MIT0604	HLII	WP_042850517.1_1100	AACCGAGGGAGAGAAGGAGGCATATATTCCCAGGAGGAAGATATAAGGCTCTTAAAAA	rbsK	Sugar kinase, ribokinase family	WP_042850517.:	985792..986655	Pentose Phosphate	COG0524
WP_042850517.1_1100	CUST_5789_PI442190829	MIT0604	HLII	WP_042850517.1_1100	TCTTATGGATGTGGCGATTCTTTTGCTGCTGGGATTCTTTATGGAATGGCATCTAAATGG	rbsK	Sugar kinase, ribokinase family	WP_042850517.:	985792..986655	Pentose Phosphate	COG0524
WP_042850517.1_1100	CUST_5790_PI442190829	MIT0604	HLII	WP_042850517.1_1100	CTTGCTAAAGTAATGGGAAGAGACGCCAGTGAATTTTTCGGCCCATATGCAAATAGTGAT	rbsK	Sugar kinase, ribokinase family	WP_042850517.:	985792..986655	Pentose Phosphate	COG0524
WP_042850517.1_1100	CUST_5791_PI442190829	MIT0604	HLII	WP_042850517.1_1100	TCTCTCAAATATGGGAATTAAGTTGCATGTGGCTTGGCGTGATAAACCAACTAGAAGAGG	rbsK	Sugar kinase, ribokinase family	WP_042850517.:	985792..986655	Pentose Phosphate	COG0524
WP_042850517.1_1100	CUST_5792_PI442190829	MIT0604	HLII	WP_042850517.1_1100	AATTGACTCTCAAGGTGAAAGAGCAATAACAGTTATTGGAGAAAGGTTAGCTCCAACTCA	rbsK	Sugar kinase, ribokinase family	WP_042850517.:	985792..986655	Pentose Phosphate	COG0524
WP_042850518.1_1102	CUST_5798_PI442190829	MIT0604	HLII	WP_042850518.1_1102	TGGACCATTTATTTCAGGACGCATATAGATCTATTTGGTTATGCTTCAATAATCTGGCT	glcH	GPH family sugar transporter	WP_042850518.:	987065..988294	GlcH sugar transporter	COG2211
WP_042850518.1_1102	CUST_5799_PI442190829	MIT0604	HLII	WP_042850518.1_1102	GCTCCTTCAAATAAAAAAGGTTTTGCACTTGCTCTACTATCACAATGTTTCGCTATGGGT	glcH	GPH family sugar transporter	WP_042850518.:	987065..988294	GlcH sugar transporter	COG2211
WP_042850518.1_1102	CUST_5800_PI442190829	MIT0604	HLII	WP_042850518.1_1102	TGTTGTTTTTACAATGGCCTGTCGGTTCTTGGATATCTAAGAAAGGAAGATTATATGGCT	glcH	GPH family sugar transporter	WP_042850518.:	987065..988294	GlcH sugar transporter	COG2211
WP_042850518.1_1102	CUST_5801_PI442190829	MIT0604	HLII	WP_042850518.1_1102	GCTCTGATTCAAGTAACTTTGCCTCTGGATCTTGTTAAAGGTGGGATATATCGGAATGCA	glcH	GPH family sugar transporter	WP_042850518.:	987065..988294	GlcH sugar transporter	COG2211
WP_042850518.1_1102	CUST_5802_PI442190829	MIT0604	HLII	WP_042850518.1_1102	ACTCTCTTGTTAGAAGTTCGGAGGCTTTAGGAATATTTCTGGGGGTATTCTTAGGGGGTT	glcH	GPH family sugar transporter	WP_042850518.:	987065..988294	GlcH sugar transporter	COG2211
WP_042850518.1_1102	CUST_5803_PI442190829	MIT0604	HLII	WP_042850518.1_1102	TATATTGGCCTGCTGCCGATTTGGGGTCCCCTATTTTTGCCATCCTAT	glcH	GPH family sugar transporter	WP_042850518.:	987065..988294	GlcH sugar transporter	COG2211
WP_042850551.1_1129	CUST_5947_PI442190829	MIT0604	HLII	WP_042850551.1_1129	ACACTTGTGTAATACTTGGCTGTGAATCAGTTGAATGTATGAGACTTGAAGCAGGGGGAA	opcA	Glucose-6-P dehydrogenase subunit opcA	WP_042850551.:	1014295..1015596	Pentose Phosphate	COG3429
WP_042850551.1_1129	CUST_5948_PI442190829	MIT0604	HLII	WP_042850551.1_1129	AATTGGGTTAGGTTGAAAAATTGGAGGGAATCATTGGCAATGATTTTTGATCCGCCTTCG	opcA	Glucose-6-P dehydrogenase subunit opcA	WP_042850551.:	1014295..1015596	Pentose Phosphate	COG3429
WP_042850551.1_1129	CUST_5949_PI442190829	MIT0604	HLII	WP_042850551.1_1129	CTATTGATGAATTACCTTCATGGTTGTGGTGGAATGGAAGCTTGGATGAATCGCCTGAAA	opcA	Glucose-6-P dehydrogenase subunit opcA	WP_042850551.:	1014295..1015596	Pentose Phosphate	COG3429
WP_042850551.1_1129	CUST_5950_PI442190829	MIT0604	HLII	WP_042850551.1_1129	ACCCCTTATCTATCTTTGGGAAATCCAAGTATTCATTTAGGACAAGTTATTGGATTGAGGTTGA	opcA	Glucose-6-P dehydrogenase subunit opcA	WP_042850551.:	1014295..1015596	Pentose Phosphate	COG3429
WP_042850551.1_1129	CUST_5951_PI442190829	MIT0604	HLII	WP_042850551.1_1129	TGGATTAGCGATAAACTTGGTTGGTCTTTTCTAAGAGTTGAAAGGGATACAGAATCAACA	opcA	Glucose-6-P dehydrogenase subunit opcA	WP_042850551.:	1014295..1015596	Pentose Phosphate	COG3429
WP_042850551.1_1129	CUST_5952_PI442190829	MIT0604	HLII	WP_042850551.1_1129	TCATGATCTACGGTTAATAATTGATACTGCTCTTGGATCGCCTAACAAATGTTTAAAAGT	opcA	Glucose-6-P dehydrogenase subunit opcA	WP_042850551.:	1014295..1015596	Pentose Phosphate	COG3429
WP_042850552.1_1130	CUST_5953_PI442190829	MIT0604	HLII	WP_042850552.1_1130	CAGAAACTTATATTGCCACAAAAGTTTTTGTTGATAACTGGCGTTGGCAAGGCGTTCCTT	zwf	glucose-6-phosphate dehydrogenase zwf	WP_042850552.:	1015599..1017122	Pentose Phosphate	COG0364
WP_042850552.1_1130	CUST_5954_PI442190829	MIT0604	HLII	WP_042850552.1_1130	ACGAGGTCAATATGGAGAAGGAGGAAATATTTCAAGTCGACTCAAAGGGTATAGGCAGGA	zwf	glucose-6-phosphate dehydrogenase zwf	WP_042850552.:	1015599..1017122	Pentose Phosphate	COG0364
WP_042850552.1_1130	CUST_5955_PI442190829	MIT0604	HLII	WP_042850552.1_1130	CTATGGAACCGCCGGGAAAGTTCGAACCAGAAGCAATAAGAAATGAAAAAGCTAAGGTTC	zwf	glucose-6-phosphate dehydrogenase zwf	WP_042850552.:	1015599..1017122	Pentose Phosphate	COG0364
WP_042850552.1_1130	CUST_5956_PI442190829	MIT0604	HLII	WP_042850552.1_1130	AGAATTCAGCCAAACGAAGGAGCTACTTCAAATTTGAGGTAAAATCTCCTGGCTCTGGA	zwf	glucose-6-phosphate dehydrogenase zwf	WP_042850552.:	1015599..1017122	Pentose Phosphate	COG0364
WP_042850552.1_1130	CUST_5957_PI442190829	MIT0604	HLII	WP_042850552.1_1130	AGCTGATGCTCAATGCTTTCTGATCCAACTTTATTTACTCGAAGTGATGAAGTAGAGGCAGC	zwf	glucose-6-phosphate dehydrogenase zwf	WP_042850552.:	1015599..1017122	Pentose Phosphate	COG0364
WP_042850552.1_1130	CUST_5958_PI442190829	MIT0604	HLII	WP_042850552.1_1130	TGGGGACCTCCTGAGTCTGATCAATTACTTTCAAAAGATAATATTTTCTGGCGTAGACCT	zwf	glucose-6-phosphate dehydrogenase zwf	WP_042850552.:	1015599..1017122	Pentose Phosphate	COG0364
WP_042850621.1_1205	CUST_6366_PI442190829	MIT0604	HLII	WP_042850621.1_1205	GGGTGTTGGTTTCTTCCTAGGGGGTCTCTTATACAAGCTATTGGGCTTTAATAATGCAGT	glcH	GPH family sugar transporter	WP_042850621.:	1093943..1095199	GlcH sugar transporter	COG2211
WP_042850621.1_1205	CUST_6367_PI442190829	MIT0604	HLII	WP_042850621.1_1205	GGCTAATGCAGCAGGAAGATTATTTGGAACCCTACTATCGGGCTTATTATTTATGCTCGG	glcH	GPH family sugar transporter	WP_042850621.:	1093943..1095199	GlcH sugar transporter	COG2211
WP_042850621.1_1205	CUST_6368_PI442190829	MIT0604	HLII	WP_042850621.1_1205	GGGGGAAAAATAATAGCCCAACAAAACTACCGTTCAATTTTGGGGACTTATCTTGACGG	glcH	GPH family sugar transporter	WP_042850621.:	1093943..1095199	GlcH sugar transporter	COG2211
WP_042850621.1_1205	CUST_6369_PI442190829	MIT0604	HLII	WP_042850621.1_1205	AAGTGCAAAGGTTTTTTTTATTTGGAGCAAGAGATGTCTGGTTTGTAGTAGCTCTTCCAGT	glcH	GPH family sugar transporter	WP_042850621.:	1093943..1095199	GlcH sugar transporter	COG2211
WP_042850621.1_1205	CUST_6370_PI442190829	MIT0604	HLII	WP_042850621.1_1205	AGGGCTGTTCTTGGGTGGATGGATAATAATCTATGGTTTCTTTCAAGTATTAGCTCCATT	glcH	GPH family sugar transporter	WP_042850621.:	1093943..1095199	GlcH sugar transporter	COG2211
WP_042850621.1_1205	CUST_6371_PI442190829	MIT0604	HLII	WP_042850621.1_1205	TGGGTTTAGGTCTATGCTTCGCATTCTTTTTAACTTTATGTCTTCCTAAAGATTCAGGGA	glcH	GPH family sugar transporter	WP_042850621.:	1093943..1095199	GlcH sugar transporter	COG2211
WP_042850734.1_1335	CUST_6973_PI442190829	MIT0604	HLII	WP_042850734.1_1335	GCCAGCCTGGGTTTTTGATACAAGATCTATTCTGGATAGAAATCTTCTGACCTCCTTGGG	ugd	UDP-glucose 6-dehydrogenase/nucleotide sugar dehydrogenase	WP_042850734.:	1199600..1201006	Amino sugar and nucleotide sugar r	COG1004
WP_042850734.1_1335	CUST_6974_PI442190829	MIT0604	HLII	WP_042850734.1_1335	GACAAATTTTTATCGGCCAGTCCCGGTTTTGGAGGGAGTTGCTTCCAGAAAGATATTTTA	ugd	UDP-glucose 6-dehydrogenase/nucleotide sugar dehydrogenase	WP_042850734.:	1199600..1201006	Amino sugar and nucleotide sugar r	COG1004
WP_042850734.1_1335	CUST_6975_PI442190829	MIT0604	HLII	WP_042850734.1_1335	TGCGAGGCTACTGGAGCAAAGATTACGGAAGTCGTAAATGTTATTGGTTCTGATAAGAGG	ugd	UDP-glucose 6-dehydrogenase/nucleotide sugar dehydrogenase	WP_042850734.:	1199600..1201006	Amino sugar and nucleotide sugar r	COG1004
WP_042850734.1_1335	CUST_6976_PI442190829	MIT0604	HLII	WP_042850734.1_1335	TGATTTGAATGATGCATTAGAATCTTCAAATGCTGTAGTTGTTTTGACTGAATGGGATCA	ugd	UDP-glucose 6-dehydrogenase/nucleotide sugar dehydrogenase	WP_042850734.:	1199600..1201006	Amino sugar and nucleotide sugar r	COG1004
WP_042850734.1_1335	CUST_6977_PI442190829	MIT0604	HLII	WP_042850734.1_1335	TGGAGTAGTGAATTATCAAAATTGACTTCTAATGCTTTTCTTGCTCAGAGAATTAGCTCG	ugd	UDP-glucose 6-dehydrogenase/nucleotide sugar dehydrogenase	WP_042850734.:	1199600..1201006	Amino sugar and nucleotide sugar r	COG1004
WP_042850734.1_1335	CUST_6978_PI442190829	MIT0604	HLII	WP_042850734.1_1335	ATTGAAAGAGCTATGACTGAATTTAAATCTGACAATGAGTTTGAAGGAAAATGGCACTTT	ugd	UDP-glucose 6-dehydrogenase/nucleotide sugar dehydrogenase	WP_042850734.:	1199600..1201006	Amino sugar and nucleotide sugar r	COG1004
WP_042851346.1_1336	CUST_6979_PI442190829	MIT0604	HLII	WP_042851346.1_1336	TGCCAGCTTGAGGTATTTCAATCCAATAGGTGCACATGAATCTTCTTTGATAGGGGAGTT	galE	UDP-glucose 4-epimerase	WP_042851346.:	1201208..1202254	Galactose metabolism	COG1087
WP_042851346.1_1336	CUST_6980_PI442190829	MIT0604	HLII	WP_042851346.1_1336	GCGGCTCATAGGGATTTGAACCAAATGTGTGTGGATGGATGGAACTGGAAAATAAAAAAT	galE	UDP-glucose 4-epimerase	WP_042851346.:	1201208..1202254	Galactose metabolism	COG1087
WP_042851346.1_1336	CUST_6981_PI442190829	MIT0604	HLII	WP_042851346.1_1336	GAAGGGCTGGTGACGCAGCTGAAATTTATGCAGATGCAACACTTGCAAAAAAAGTTTTAG	galE	UDP-glucose 4-epimerase	WP_042851346.:	1201208..1202254	Galactose metabolism	COG1087
WP_042851346.1_1336	CUST_6982_PI442190829	MIT0604	HLII	WP_042851346.1_1336	TCTTGGAACAGGGAAAGGCACTTCGGTCATAGAGTTAATAAATGCATTCGAAAAAGCAAA	galE	UDP-glucose 4-epimerase	WP_042851346.:	1201208..1202254	Galactose metabolism	COG1087
WP_042851346.1_1336	CUST_6983_PI442190829	MIT0604	HLII	WP_042851346.1_1336	TGATCCCCCTTTTTCTGAAAATGCTGAATTGAATCCTGTTAATCCCTATGGAGAAACAAA	galE	UDP-glucose 4-epimerase	WP_042851346.:	1201208..1202254	Galactose metabolism	COG1087
WP_042851346.1_1336	CUST_6984_PI442190829	MIT0604	HLII	WP_042851346.1_1336	ACATTTTGCTGGTTTAAAAGCTGTTGCAGAATCAGTATTAGATCCAATTAGTTACTGGGA	galE	UDP-glucose 4-epimerase	WP_042851346.:	1201208..1202254	Galactose metabolism	COG1087
WP_081925755.1_1436	CUST_7455_PI442190829	MIT0604	HLII	WP_081925755.1_1436	TCTTGAAACTCAAATGCTTGATATTTCAAAATCACCCATGGAAGTTTCGCCTACTGCCCA	sdhC	succinate dehydrogenase sdhC	WP_081925755.:	1308149..1309903	Krebs cycle and related genes	COG2009
WP_081925755.1_1436	CUST_7456_PI442190829	MIT0604	HLII	WP_081925755.1_1436	GGTGGAAATTCTTTGGCAGAAATTCTTATTTTTGGCAAACGTGCTGGTATTGCATCCTCA	sdhC	succinate dehydrogenase sdhC	WP_081925755.:	1308149..1309903	Krebs cycle and related genes	COG2009
WP_081925755.1_1436	CUST_7457_PI442190829	MIT0604	HLII	WP_081925755.1_1436	TAAGTGCAAAAGCTACAATTCTTTCAGCACTCCAAAGAAATGAAAGTAGGGGGGCACATC	sdhC	succinate dehydrogenase sdhC	WP_081925755.:	1308149..1309903	Krebs cycle and related genes	COG2009
WP_081925755.1_1436	CUST_7458_PI442190829	MIT0604	HLII	WP_081925755.1_1436	TAATCCAGAAGATTTATCTACTTCAGTAAAGGGCCTTTTCGCAGCAGGAGAAGTAGCAGG	sdhC	succinate dehydrogenase sdhC	WP_081925755.:	1308149..1309903	Krebs cycle and related genes	COG2009
WP_081925755.1_1436	CUST_7459_PI442190829	MIT0604	HLII	WP_081925755.1_1436	TGCCTTTGCGCATGAAAACATTAATAAGTTCATCAAAAATGGAGATGAACTAGTCAGGCC	sdhC	succinate dehydrogenase sdhC	WP_081925755.:	1308149..1309903	Krebs cycle and related genes	COG2009
WP_081925755.1_1436	CUST_7460_PI442190829	MIT0604	HLII	WP_081925755.1_1436	GAAGTGATTGCCCATCACTTGACCCTTCATTTAAATATAATTGTCTAGTCAGAATGGACG	sdhC	succinate dehydrogenase sdhC	WP_081925755.:	1308149..1309903	Krebs cycle and related genes	COG2009
WP_042850832.1_1454	CUST_7557_PI442190829	MIT0604	HLII	WP_042850832.1_1454	GGAGGAGGGAAAAAGGTCGCTGTCTTAAATCTAAATGAATTTCTCAGCGATCATGCTTCA		polysaccharide biosynthesis/export family protein	WP_042850832.:	1324765..1326027	Other sugar transporters	COG1596
WP_042850832.1_1454	CUST_7558_PI442190829	MIT0604	HLII	WP_042850832.1_1454	ACTGGTTTGTCTCCACGTTTTGTAGAGGTAAATGTTTATGGAAGAGTTCAGAATCCAGGA		polysaccharide biosynthesis/export family protein	WP_042850832.:	1324765..1326027	Other sugar transporters	COG1596
WP_042850832.1_1454	CUST_7559_PI442190829	MIT0604	HLII	WP_042850832.1_1454	ACTCTGCTAAAGCACCAAGAGGTTCAAAAAGAAATCCTTTCATTAAAGAAGGTGACCTAA		polysaccharide biosynthesis/export family protein	WP_042850832.:	1324765..1326027	Other sugar transporters	COG1596
WP_042850832.1_1454	CUST_7560_PI442190829	MIT0604	HLII	WP_042850832.1_1454	AGGGACATTATCTGATGCAATAGATATAACTGGACCAATAAAACCATTATCAGGGAAGGT		polysaccharide biosynthesis/export family protein	WP_042850832.:	1324765..1326027	Other sugar transporters	COG1596
WP_042850832.1_1454	CUST_7561_PI442190829	MIT0604	HLII	WP_042850832.1_1454	ATTTCAGATGTTATTCGAAAAGCGGGAGGAATAACTTCATCAAGTGATTTGTCTAGAGTT		polysaccharide biosynthesis/export family protein	WP_042850832.:	1324765..1326027	Other sugar transporters	COG1596
WP_042850832.1_1454	CUST_7562_PI442190829	MIT0604	HLII	WP_042850832.1_1454	AGTGTTCTAGGGAAAACTAGTGGTGTAATACAAGAAATAACTGCTCCTCTTCAAGGAATA		polysaccharide biosynthesis/export family protein	WP_042850832.:	1324765..1326027	Other sugar transporters	COG1596
WP_042850841.1_1463	CUST_7610_PI442190829	MIT0604	HLII	WP_042850841.1_1463	ATTCTCTCGCAGATCCTGATGAATTGAGGTCTGAAAAAGAGAAGGAGTTTTGGGGGAAAA	pdhA	Pyruvate dehydrogenase E1 component alpha subunit	WP_042850841.1		Glycolysis/Krebs cycle	COG1071
WP_042850841.1_1463	CUST_7611_PI442190829	MIT0604	HLII	WP_042850841.1_1463	ACTGCAGCATTCTTTGGGGATGGGACTTGCAATAATGGGCAGTTTTTTGAATGTTTAAAT	pdhA	Pyruvate dehydrogenase E1 component alpha subunit	WP_042850841.1		Glycolysis/Krebs cycle	COG1071
WP_042850841.1_1463	CUST_7612_PI442190829	MIT0604	HLII	WP_042850841.1_1463	GATATGCATTTATTGGAGAGGGAATTCCAGTTGCACTAGGCGCAGCCTTTTCAAGTAAAT	pdhA	Pyruvate dehydrogenase E1 component alpha subunit	WP_042850841.1		Glycolysis/Krebs cycle	COG1071
WP_042850841.1_1463	CUST_7613_PI442190829	MIT0604	HLII	WP_042850841.1_1463	AAAATGCTATTGAAGCACCCGAACCCCCATCACAAGAATTAACTAAGTATATTTGGGCCG	pdhA	Pyruvate dehydrogenase E1 component alpha subunit	WP_042850841.1		Glycolysis/Krebs cycle	COG1071
WP_042850841.1_1463	CUST_7614_PI442190829	MIT0604	HLII	WP_042850841.1_1463	GTTGAAAATAACAAATGGGCTATCGGGATGGCTCACGATAGAGCTACTAGCAATCCTGAA	pdhA	Pyruvate dehydrogenase E1 component alpha subunit	WP_042850841.1		Glycolysis/Krebs cycle	COG1071
WP_042850841.1_1463	CUST_7615_PI442190829	MIT0604	HLII	WP_042850841.1_1463	GTTGAGCGAGCTAGAGCAGGAGAAGGGCCAACACTTTTAGAATGCTTGACTTATAGATAC	pdhA	Pyruvate dehydrogenase E1 component alpha subunit	WP_042850841.1		Glycolysis/Krebs cycle	COG1071
WP_042850863.1_1484	CUST_7732_PI442190829	MIT0604	HLII	WP_042850863.1_1484	TGATGTTTTAAGAATGGGAGTTCAAGGTATAAGTGAAGTAATCACACGCCCTGGCGAAGT	ftsZ	cell division protein FtsZ	WP_042850863.:	1357426..1358541	Cell Division	COG0206
WP_042850863.1_1484	CUST_7733_PI442190829	MIT0604	HLII	WP_042850863.1_1484	AGCTGCGATTAATAGTCCGTTATTAGAAGCAGGGAGAATTGATGGAGCAAAAGGTTGTCT	ftsZ	cell division protein FtsZ	WP_042850863.:	1357426..1358541	Cell Division	COG0206
WP_042850863.1_1484	CUST_7734_PI442190829	MIT0604	HLII	WP_042850863.1_1484	GACTCTTGACGATGTGACTGCAGTTGGTGAAGTTATTAGTGATGTCGTAGACAAGGATGC	ftsZ	cell division protein FtsZ	WP_042850863.:	1357426..1358541	Cell Division	COG0206
WP_042850863.1_1484	CUST_7735_PI442190829	MIT0604	HLII	WP_042850863.1_1484	TTGACTTTGCTGATGTCAGATCAGTTATGACAGAAGCTGGTACAGCACTTCTTGGAATGG	ftsZ	cell division protein FtsZ	WP_042850863.:	1357426..1358541	Cell Division	COG0206
WP_042850863.1_1484	CUST_7736_PI442190829	MIT0604	HLII	WP_042850863.1_1484	CAGCTCCAGTTGTTGCTGAAATAGCAAAGCAAAGCGGTGCATTAACTATTGGCATAGTAA	ftsZ	cell division protein FtsZ	WP_042850863.:	1357426..1358541	Cell Division	COG0206
WP_042850863.1_1484	CUST_7737_PI442190829	MIT0604	HLII	WP_042850863.1_1484	TGATCGTTTAAAAGAAGTTTCTGCAGGTGCCTCTATTCAAGAAGCATTCAGGAATGCTGA	ftsZ	cell division protein FtsZ	WP_042850863.:	1357426..1358541	Cell Division	COG0206
WP_042851365.1_1499	CUST_7809_PI442190829	MIT0604	HLII	WP_042851365.1_1499	TACTTCCTGATGCAATAGACGAAGACCCAGAGAAACCAGCAGGATTATATACTGCTTGGA	glcH	GPH family sugar transporter	WP_042851365.:	1370032..1371378	GlcH sugar transporter	COG2211
WP_042851365.1_1499	CUST_7810_PI442190829	MIT0604	HLII	WP_042851365.1_1499	TTTTTATAATCTGTCTTATTGGTATTGGAGCTTCAACCGCTTTTCTTATCCCTTGGTCAC	glcH	GPH family sugar transporter	WP_042851365.:	1370032..1371378	GlcH sugar transporter	COG2211
WP_042851365.1_1499	CUST_7811_PI442190829	MIT0604	HLII	WP_042851365.1_1499	ACAGGATTTCAGCGTTAAACTATGGAGCGATTATGTGGATTATTTCATGTACAGCAGCTT	glcH	GPH family sugar transporter	WP_042851365.:	1370032..1371378	GlcH sugar transporter	COG2211
WP_042851365.1_1499	CUST_7812_PI442190829	MIT0604	HLII	WP_042851365.1_1499	ATTGCGAAGTGGATCCCCATACCTTTCCAAATTAGCGCTTTAGTGGGTTTACAAATATGG	glcH	GPH family sugar transporter	WP_042851365.:	1370032..1371378	GlcH sugar transporter	COG2211
WP_042851365.1_1499	CUST_7813_PI442190829	MIT0604	HLII	WP_042851365.1_1499	TCTTCTCTGGTGCGCCTTACAATTAATGCAAACAGTAGCGTTAATCTATGTCGAGGATGT	glcH	GPH family sugar transporter	WP_042851365.:	1370032..1371378	GlcH sugar transporter	COG2211
WP_042851365.1_1499	CUST_7814_PI442190829	MIT0604	HLII	WP_042851365.1_1499	GCAACATTAATTTCTTGTTGGGGATTAGCTCCATTCGCAAAAAAGCAAGAAGGCCTTCA	glcH	GPH family sugar transporter	WP_042851365.:	1370032..1371378	GlcH sugar transporter	COG2211
WP_042850895.1_1518	CUST_7922_PI442190829	MIT0604	HLII	WP_042850895.1_1518	CTTGATGATATGTGCGGTGGAGGATATTTTCAAGATTCCATCATTCTCGCTACTGGAGCT	kaiC	circadian clock protein KaiC	WP_042850895.:	1389164..1390693	Circadian Rhythm	COG0467
WP_042850895.1_1518	CUST_7923_PI442190829	MIT0604	HLII	WP_042850895.1_1518	CGAACAAATATTTAGTGGTGCTCCTCACCGAGTTGTCCCTGATCAAAATGTCCAAAATGT	kaiC	circadian clock protein KaiC	WP_042850895.:	1389164..1390693	Circadian Rhythm	COG0467
WP_042850895.1_1518	CUST_7924_PI442190829	MIT0604	HLII	WP_042850895.1_1518	AGGTGAGATGGCACGAGCATTAAATGTCTTTAAAATGCGTGGATCATGGCATGATAAAAG	kaiC	circadian clock protein KaiC	WP_042850895.:	1389164..1390693	Circadian Rhythm	COG0467
WP_042850895.1_1518	CUST_7925_PI442190829	MIT0604	HLII	WP_042850895.1_1518	ACCAAAAAGAATGGCAATTGATTCTCTCTCTGCATTAGCCAGAGGTGTAAGTTTGAATGC	kaiC	circadian clock protein KaiC	WP_042850895.:	1389164..1390693	Circadian Rhythm	COG0467
WP_042850895.1_1518	CUST_7926_PI442190829	MIT0604	HLII	WP_042850895.1_1518	ACTTTTTGCATATGAAGAATCCAGAGCTCAATTACTTAGGAATGCGACTAGCTGGGGAAT	kaiC	circadian clock protein KaiC	WP_042850895.:	1389164..1390693	Circadian Rhythm	COG0467
WP_042850895.1_1518	CUST_7927_PI442190829	MIT0604	HLII	WP_042850895.1_1518	TGCTGTTACTGGTTATACAAAACAAGAGGAGATAGCAGGCTTTTTTACTAATACTGCAGA	kaiC	circadian clock protein KaiC	WP_042850895.:	1389164..1390693	Circadian Rhythm	COG0467
WP_025913788.1_1519	CUST_7928_PI442190829	MIT0604	HLII	WP_025913788.1_1519	TGGTTTAGATCTACTTTTGATGAATTAACTGAAACTGAATATAGTGGAGATAAGT	kaiB	Circadian clock protein KaiB	WP_025913788.:	1390766..1391083	Circadian Rhythm	COG4251
WP_025913788.1_1519	CUST_7929_PI442190829	MIT0604	HLII	WP_025913788.1_1519	CCTCCACCAGTTAGAAAAATAATAGGTGATCTTTCAGATAGAGAGAAAGTTTTAATTGGT	kaiB	Circadian clock protein KaiB	WP_025913788.:	1390766..1391083	Circadian Rhythm	COG4251
WP_025913788.1_1519	CUST_7930_PI442190829	MIT0604	HLII	WP_025913788.1_1519	TCGATGTACTTAAGCAACCACAACTTGCAGAAGAAGATAAAATTTTAGCCACTCCAACGT	kaiB	Circadian clock protein KaiB	WP_025913788.:	1390766..1391083	Circadian Rhythm	COG4251
WP_025913788.1_1519	CUST_7931_PI442190829	MIT0604	HLII	WP_025913788.1_1519	TGAGAGCTTTAAATACCTTAAGAGAAATTTTAGATAATGAATTCAAAGGAGTTTATGCTT	kaiB	Circadian clock protein KaiB	WP_025913788.:	1390766..1391083	Circadian Rhythm	COG4251
WP_025913788.1_1519	CUST_7932_PI442190829	MIT0604	HLII	WP_025913788.1_1519	TGGCAGCAAGGAAAACATATATTTTAAAACTCTATGTTGCTGGAAATACTCCTAATTCGA	kaiB	Circadian clock protein KaiB	WP_025913788.:	1390766..1391083	Circadian Rhythm	COG4251
WP_002806495.1_1619	CUST_8424_PI442190829	MIT0604	HLII	WP_002806495.1_1619	AATCCAGAAAAAATAAAGGCTCTCAAACAGGTATCGAAAGCCGAAGCAAGGATTCAAGCC	atpC	ATP synthase epsilon subunit	WP_002806495.:	1480978..1481382	Energy Metabolism	COG0355
WP_002806495.1_1619	CUST_8425_PI442190829	MIT0604	HLII	WP_002806495.1_1619	TGCTGAAATTGGTTCTGAAATTAATGTTCAAAATGCTGAACAAGATCTTAAAGAAGCAAA	atpC	ATP synthase epsilon subunit	WP_002806495.:	1480978..1481382	Energy Metabolism	COG0355
WP_002806495.1_1619	CUST_8426_PI442190829	MIT0604	HLII	WP_002806495.1_1619	TAAGGCTAAGAATGAATTCCCAATGGAAGTCAATAGCTCTAATGGAGGCTTCGCTGAAA	atpC	ATP synthase epsilon subunit	WP_002806495.:	1480978..1481382	Energy Metabolism	COG0355
WP_002806495.1_1619	CUST_8427_PI442190829	MIT0604	HLII	WP_002806495.1_1619	GGGAAGCTGAAGAAGTAATCCTTCCAAGTACTACTGGTCAGCTTGGTATACTACCAGGAC	atpC	ATP synthase epsilon subunit	WP_002806495.:	1480978..1481382	Energy Metabolism	COG0355
WP_011863563.1_1630	CUST_8485_PI442190829	MIT0604	HLII	WP_011863563.1_1630	CGCACAGTTTGATGAACTAGCTGCATTTTCCCAATTTGCATCTGATCTTGATGAAGCAAC	atpA	ATP synthase alpha subunit	WP_011863563.:	1490663..1492180	Energy Metabolism	COG0056
WP_011863563.1_1630	CUST_8486_PI442190829	MIT0604	HLII	WP_011863563.1_1630	GTAGATTACTAGAAAGAGCAGCAAAACTTTCTGATGCCATGGGAGGGGGTTCTATGACAG	atpA	ATP synthase alpha subunit	WP_011863563.:	1490663..1492180	Energy Metabolism	COG0056
WP_011863563.1_1630	CUST_8487_PI442190829	MIT0604	HLII	WP_011863563.1_1630	CAAGCCTACCGACAAATGTCTCTTCTTTTAAAAAGACCACCAGGAAGAGAGGCTTATCCA	atpA	ATP synthase alpha subunit	WP_011863563.:	1490663..1492180	Energy Metabolism	COG0056
WP_011863563.1_1630	CUST_8488_PI442190829	MIT0604	HLII	WP_011863563.1_1630	CAACCTCAATTCTCTCCACTAAACCTTGCAGAACAAGTTGCAGTTGTTATGCAGGAGTT	atpA	ATP synthase alpha subunit	WP_011863563.:	1490663..1492180	Energy Metabolism	COG0056
WP_011863563.1_1630	CUST_8489_PI442190829	MIT0604	HLII	WP_011863563.1_1630	ACTCAGGCAGGGGACGTTTCGGCTTACATTCCAACTAATGTTATTTCAATTCAGATGGA	atpA	ATP synthase alpha subunit	WP_011863563.:	1490663..1492180	Energy Metabolism	COG0056
WP_011863567.1_1635	CUST_8511_PI442190829	MIT0604	HLII	WP_011863567.1_1635	TGATCCTAAAGGCCTTCAAAACCTGCTTGAGTTCCTCTGGGATTACATAAGAGATCTTGC	atpI	ATP synthase subunit A	WP_011863567.:	1494202..1494927	Energy Metabolism	COG0356
WP_011863567.1_1635	CUST_8512_PI442190829	MIT0604	HLII	WP_011863567.1_1635	ATTTACAAGTGCAATTCAGGCATTGATTTTTGCAACACTAGCGGCCTACTACATTGGAGA	atpI	ATP synthase subunit A	WP_011863567.:	1494202..1494927	Energy Metabolism	COG0356

WP_011863567.1_1635	CUST_8513_PI442190829	MIT0604	HLII	WP_011863567.1_1635	TGGGGAGGAGCTTTAATTCCTTGGAGATTGATTAAGTTACCAAGTGGAGAATTAGGAGCA	atpI	ATP synthase subunit A	WP_011863567.:	1494202..1494927	Energy Metabolism	COG0356
WP_011863567.1_1635	CUST_8514_PI442190829	MIT0604	HLII	WP_011863567.1_1635	TGCAGGTTTAAGCAATAAGGGTTGGAGATACTTCGAATATTATGTTCACCCAACTCCGAT	atpI	ATP synthase subunit A	WP_011863567.:	1494202..1494927	Energy Metabolism	COG0356
WP_011863567.1_1635	CUST_8515_PI442190829	MIT0604	HLII	WP_011863567.1_1635	AGCATTAGAAGTTGGTCAACATCTTTATTGGCAAATTGGAAATATCAGACTACATGGGCA	atpI	ATP synthase subunit A	WP_011863567.:	1494202..1494927	Energy Metabolism	COG0356
WP_011863567.1_1635	CUST_8516_PI442190829	MIT0604	HLII	WP_011863567.1_1635	GTTGGTGTTCTAGTCTTTTTAGTTCCACTAATTCTCCCAATACCTGTTATGTTCCTAGGA	atpI	ATP synthase subunit A	WP_011863567.:	1494202..1494927	Energy Metabolism	COG0356
WP_042851007.1_1645	CUST_8567_PI442190829	MIT0604	HLII	WP_042851007.1_1645	AAGTTCTATCATGCCGGGTAAAGTGAATCCAACTCAATGCGAAGCCTTATCAATGGTTTG	fumC	class II fumarate hydratase	WP_042851007.:	1502608..1503993	Krebs cycle and related genes	COG0114
WP_042851007.1_1645	CUST_8568_PI442190829	MIT0604	HLII	WP_042851007.1_1645	CTTTGGGAGGAACTGCAGTTGGAACAGGGATTAATTGTCCAAAAGGATTTTGTGAAGAGT	fumC	class II fumarate hydratase	WP_042851007.:	1502608..1503993	Krebs cycle and related genes	COG0114
WP_042851007.1_1645	CUST_8569_PI442190829	MIT0604	HLII	WP_042851007.1_1645	AGCCATAGTGCCAAAAGTTGGTTATGAAAAAGCAGCTAAAATTGCAAACCTTGCCTTCAA	fumC	class II fumarate hydratase	WP_042851007.:	1502608..1503993	Krebs cycle and related genes	COG0114
WP_042851007.1_1645	CUST_8570_PI442190829	MIT0604	HLII	WP_042851007.1_1645	TGGGTCTTGAATATGCAGTCTCAATGGCAAATTCTAGCGGCACTTTACAGATGAATGAAT	fumC	class II fumarate hydratase	WP_042851007.:	1502608..1503993	Krebs cycle and related genes	COG0114
WP_042851007.1_1645	CUST_8571_PI442190829	MIT0604	HLII	WP_042851007.1_1645	TGGCCTCTCATGATCGTCTAGCTCAAGTAATGAGTCAGATAAAAATATTAGCAGGTGCAT	fumC	class II fumarate hydratase	WP_042851007.:	1502608..1503993	Krebs cycle and related genes	COG0114
WP_042851007.1_1645	CUST_8572_PI442190829	MIT0604	HLII	WP_042851007.1_1645	TCTATCTTCTGGTCCAAGATCAGGAATATATGAACTAATTATTCCTCAAAATGAACCTGG	fumC	class II fumarate hydratase	WP_042851007.:	1502608..1503993	Krebs cycle and related genes	COG0114
WP_042851053.1_1689	CUST_8808_PI442190829	MIT0604	HLII	WP_042851053.1_1689	GTGTTTGTGCGAAAATATCAGGTGAAATTGCAGAACTTCACGGCAATACTGGCTTCAATG	glsF	Ferredoxin-dependent glutamate synthase	WP_042851053.:	1551678..1556240	Nitrogen Metabolism	COG0069
WP_042851053.1_1689	CUST_8809_PI442190829	MIT0604	HLII	WP_042851053.1_1689	TCTAGGTTCCACAGGAAGAAATATTGGTGCGGGCATGACTGGTGGAATAGCTTTTATAAT	glsF	Ferredoxin-dependent glutamate synthase	WP_042851053.:	1551678..1556240	Nitrogen Metabolism	COG0069
WP_042851053.1_1689	CUST_8810_PI442190829	MIT0604	HLII	WP_042851053.1_1689	GGTAACAGAAGGGGCAGGTGATCATTGTTGTGAATACATGACTGGTGGGAAAGTAGTTAT	glsF	Ferredoxin-dependent glutamate synthase	WP_042851053.:	1551678..1556240	Nitrogen Metabolism	COG0069
WP_042851053.1_1689	CUST_8811_PI442190829	MIT0604	HLII	WP_042851053.1_1689	TGAAATCTCCTCCGAACAAGTCATCCTAGGAAATACTTGTCTTTATGGAGCAACAGGTGG	glsF	Ferredoxin-dependent glutamate synthase	WP_042851053.:	1551678..1556240	Nitrogen Metabolism	COG0069
WP_042851053.1_1689	CUST_8812_PI442190829	MIT0604	HLII	WP_042851053.1_1689	TGCAGGACAAAGCTTTGGTGCCTTTTTATTGAAGGGAATGAATGTTCAATTAATCGGAGA	glsF	Ferredoxin-dependent glutamate synthase	WP_042851053.:	1551678..1556240	Nitrogen Metabolism	COG0069
WP_042851053.1_1689	CUST_8813_PI442190829	MIT0604	HLII	WP_042851053.1_1689	TGGTCTCATTTTAAGAGTACTTTCAAATTGATTGTTCCCCCAAGTGAAGAAGAGATGCTT	glsF	Ferredoxin-dependent glutamate synthase	WP_042851053.:	1551678..1556240	Nitrogen Metabolism	COG0069
WP_011819135.1_1700	CUST_8863_PI442190829	MIT0604	HLII	WP_011819135.1_1700	TCTGATCCAACAAGTTCAGCAAGTCTAGCTGCTAATTCATTGCCAGGTAATCACTACTGG	psaB	Photosystem I P700 chlorophyll A apoprotein A2 PsaB	WP_011819135.1		Photosystem I	COG2885
WP_011819135.1_1700	CUST_8864_PI442190829	MIT0604	HLII	WP_011819135.1_1700	AAATTTACTAGCATGGGTAACTTTCTACTGGCATTGGAAACACCTAGCAATTTGGCAGGG	psaB	Photosystem I P700 chlorophyll A apoprotein A2 PsaB	WP_011819135.1		Photosystem I	COG2885
WP_011819135.1_1700	CUST_8865_PI442190829	MIT0604	HLII	WP_011819135.1_1700	GGGCTTGGATGTTCCTATTCGGTCACTTAGTTTGGGCTACTGGTTTCATGTTCCTAATAT	psaB	Photosystem I P700 chlorophyll A apoprotein A2 PsaB	WP_011819135.1		Photosystem I	COG2885
WP_011819135.1_1700	CUST_8866_PI442190829	MIT0604	HLII	WP_011819135.1_1700	GACGAGGAGGTACTTGTGATAGTTCATCTTGGGACGCTATGTACTTAGCTATGTTCTGGG	psaB	Photosystem I P700 chlorophyll A apoprotein A2 PsaB	WP_011819135.1		Photosystem I	COG2885
WP_011819135.1_1700	CUST_8867_PI442190829	MIT0604	HLII	WP_011819135.1_1700	CCAGTTGCACTTTCAATTGTTCAAGCTAGATTAGTTGGGCTAGCACATTTCACGATTGGA	psaB	Photosystem I P700 chlorophyll A apoprotein A2 PsaB	WP_011819135.1		Photosystem I	COG2885
WP_011819135.1_1700	CUST_8868_PI442190829	MIT0604	HLII	WP_011819135.1_1700	GTGCTTTCTTACCTATCGGTCCTGCAGATTTCTTAGTTCACCATGCTATCGCTTTAGGTC	psaB	Photosystem I P700 chlorophyll A apoprotein A2 PsaB	WP_011819135.1		Photosystem I	COG2885
WP_042851062.1_1701	CUST_8869_PI442190829	MIT0604	HLII	WP_042851062.1_1701	AGCTCACTTTATATGGGCTTTCAGTTTAATGTTCCTCTTCAGCGGAAGAGGATATTGGCA	psaA	Photosystem I P700 chlorophyll A apoprotein A1 PsaA	WP_042851062.1		Photosystem I	COG2885
WP_042851062.1_1701	CUST_8870_PI442190829	MIT0604	HLII	WP_042851062.1_1701	TTTCCACTTCTCTTGGAAAATGCAGAGTGATGTTTGGGGACTTACTGGTGGTAATTTTGC	psaA	Photosystem I P700 chlorophyll A apoprotein A1 PsaA	WP_042851062.1		Photosystem I	COG2885
WP_042851062.1_1701	CUST_8871_PI442190829	MIT0604	HLII	WP_042851062.1_1701	TAAATTAAAGGTTGCACCAACCATTCAACCTCGAGCTCTATCTATTACTCAGGGACGTGC	psaA	Photosystem I P700 chlorophyll A apoprotein A1 PsaA	WP_042851062.1		Photosystem I	COG2885
WP_042851062.1_1701	CUST_8872_PI442190829	MIT0604	HLII	WP_042851062.1_1701	GGAAGAGGTGGTACATGTCAAGTTTCTTCATGGGATCACGTTTTCTTAGCTCTCTTCTGG	psaA	Photosystem I P700 chlorophyll A apoprotein A1 PsaA	WP_042851062.1		Photosystem I	COG2885
WP_042851062.1_1701	CUST_8873_PI442190829	MIT0604	HLII	WP_042851062.1_1701	GTAGAAGTTGGTGGAAAGGTAGCTATAGCGCCAATTCCACTAGGGACAGCTGATTTAATG	psaA	Photosystem I P700 chlorophyll A apoprotein A1 PsaA	WP_042851062.1		Photosystem I	COG2885
WP_042851062.1_1701	CUST_8874_PI442190829	MIT0604	HLII	WP_042851062.1_1701	TGGGCTCAAGCTTCTCAAGTATTAACAAGCTATGGCCAATCAATAAGCATGTACGGTTTG	psaA	Photosystem I P700 chlorophyll A apoprotein A1 PsaA	WP_042851062.1		Photosystem I	COG2885
WP_042851139.1_1795	CUST_9386_PI442190829	MIT0604	HLII	WP_042851139.1_1795	ATTAGTTACATTCATGCAGCTGGATATCCAGCTGGTGAGATGAAACATGGTCCAATAGCT	glmS	Glutamine--fructose-6-phosphatetransaminase(isomerizing)	WP_042851139.:	1647103..1648998	Amino Acid metabolism	COG0449
WP_042851139.1_1795	CUST_9387_PI442190829	MIT0604	HLII	WP_042851139.1_1795	AGAAGCAAAAGCCAGAGATTCATTTTTGATTGGGATTGCCCCTGAACATAATGGAACTGA	glmS	Glutamine--fructose-6-phosphatetransaminase(isomerizing)	WP_042851139.:	1647103..1648998	Amino Acid metabolism	COG0449
WP_042851139.1_1795	CUST_9388_PI442190829	MIT0604	HLII	WP_042851139.1_1795	TTGAGTTACCACATAGCAGCTCATAGAGGATTGGACGTTGATCAACCAAGAAACTTAGCT	glmS	Glutamine--fructose-6-phosphatetransaminase(isomerizing)	WP_042851139.:	1647103..1648998	Amino Acid metabolism	COG0449
WP_042851139.1_1795	CUST_9389_PI442190829	MIT0604	HLII	WP_042851139.1_1795	GGAAGGCAGGTCTCAAATATAATTGATATCTGTGCAGGAATAGAAGTTGGAGTTGCAGCA	glmS	Glutamine--fructose-6-phosphatetransaminase(isomerizing)	WP_042851139.:	1647103..1648998	Amino Acid metabolism	COG0449
WP_042851139.1_1795	CUST_9390_PI442190829	MIT0604	HLII	WP_042851139.1_1795	ACCGGTAATTTCCATTGCTTCCCCTGGTGAAGTTTTCGATAAAGTTATAAGTAATGCTCA	glmS	Glutamine--fructose-6-phosphatetransaminase(isomerizing)	WP_042851139.:	1647103..1648998	Amino Acid metabolism	COG0449
WP_042851139.1_1795	CUST_9391_PI442190829	MIT0604	HLII	WP_042851139.1_1795	TTTTTAGGTAGGGGTATCAATTATCCCATAGCACTTGAAGGGGCGTTAAAACTCAAAGAA	glmS	Glutamine--fructose-6-phosphatetransaminase(isomerizing)	WP_042851139.:	1647103..1648998	Amino Acid metabolism	COG0449
WP_042851238.1_1892	CUST_9931_PI442190829	MIT0604	HLII	WP_042851238.1_1892	GTAGTAGGAATCATTGCAATTATCGTTCAGGGAGGACTAATTGGGCCTCTGGTAAAGCAA	glcH	GPH family sugar transporter	WP_042851238.:	1751241..1752512	GlcH sugar transporter	COG2211
WP_042851238.1_1892	CUST_9932_PI442190829	MIT0604	HLII	WP_042851238.1_1892	GTCTACAGAGTCTTGGGGGAGTTTTAGGAATTGCAATGGCAGGAAGGGTTTATGATAGTT	glcH	GPH family sugar transporter	WP_042851238.:	1751241..1752512	GlcH sugar transporter	COG2211
WP_042851238.1_1892	CUST_9933_PI442190829	MIT0604	HLII	WP_042851238.1_1892	TTATTCAGCTGTTTCATTTTTAGCCATTGGGGCAGGGTTAATTACACCCACCTTAAGAGC	glcH	GPH family sugar transporter	WP_042851238.:	1751241..1752512	GlcH sugar transporter	COG2211
WP_042851238.1_1892	CUST_9934_PI442190829	MIT0604	HLII	WP_042851238.1_1892	GCGAAATGCGATTAACACTTATGGGATCAGGCTTCATACTTGTTGCGTGTGCTCTTTTAA	glcH	GPH family sugar transporter	WP_042851238.:	1751241..1752512	GlcH sugar transporter	COG2211
WP_042851238.1_1892	CUST_9935_PI442190829	MIT0604	HLII	WP_042851238.1_1892	CCTCATATTTTTCCTTCAAGAATCCTTAAACTGGACTACCAAAGCATCAAGTGGAACTCT	glcH	GPH family sugar transporter	WP_042851238.:	1751241..1752512	GlcH sugar transporter	COG2211
WP_042851238.1_1892	CUST_9936_PI442190829	MIT0604	HLII	WP_042851238.1_1892	TCACCTTTTATAGCTGGTTCCGTTATCTTACTTTTCATGATATACCTTATTGCAGAGGGT	glcH	GPH family sugar transporter	WP_042851238.:	1751241..1752512	GlcH sugar transporter	COG2211
103.1_WP_079292450.:	CUST_39_PI442190811	HOT208_60m_808G21		103.1_WP_079292450.:	GTGTATGGGCATCTCCAGGCGTTGTTAACTATGGTCAAAGGGGTACCACTTTCTTATAAT	argH	Argininosuccinate lyase	WP_079292450.:	1019..2398	Amino Acid metabolism	COG0165
103.1_WP_079292450.:	CUST_40_PI442190811	HOT208_60m_808G21		103.1_WP_079292450.:	ACTGGAAGTAGCTTAATGCCGCAGAAAAAAAATCCAGATGTCCCAGAATTGATAAGAGGT	argH	Argininosuccinate lyase	WP_079292450.:	1019..2398	Amino Acid metabolism	COG0165
103.1_WP_079292450.:	CUST_41_PI442190811	HOT208_60m_808G21		103.1_WP_079292450.:	TGTCGTTAAATCAAGGAATAGCGAAGGTGGTACAGGTTTTGTTCAGGTAGAAAAAGAGGT	argH	Argininosuccinate lyase	WP_079292450.:	1019..2398	Amino Acid metabolism	COG0165
103.1_WP_079292450.:	CUST_42_PI442190811	HOT208_60m_808G21		103.1_WP_079292450.:	TGTTCCTTTTAGGACTGCCTATCAAGTTGTTGGCCAAATGGTTAAATATTGCTTAGAGAG	argH	Argininosuccinate lyase	WP_079292450.:	1019..2398	Amino Acid metabolism	COG0165
103.1_WP_079292450.:	CUST_43_PI442190811	HOT208_60m_808G21		103.1_WP_079292450.:	AGGCACTTTACAGCTTCAGAATTGGGTTTTACAAAGATTTATAAAAACAGTATCGATGCA	argH	Argininosuccinate lyase	WP_079292450.:	1019..2398	Amino Acid metabolism	COG0165
103.1_WP_079292450.:	CUST_44_PI442190811	HOT208_60m_808G21		103.1_WP_079292450.:	AAAGATTTTCAAGAGGATAAAGAGCCAATCTTTGATACTGCAGAAACAATATCCTCTTGT	argH	Argininosuccinate lyase	WP_079292450.:	1019..2398	Amino Acid metabolism	COG0165
106.1_WP_079292523.:	CUST_497_PI442190811	HOT208_60m_808G21		106.1_WP_079292523.:	TCCAGCAGCACTATTATCATCCGCATGGTATTTAACTGGGGATGGTATTGGAAAAAGAGA	pgi	Glucose-6-phosphate isomerase	WP_079292523.:	23262..24845	Glycolysis/Gluconeogenesis	COG0166
106.1_WP_079292523.:	CUST_498_PI442190811	HOT208_60m_808G21		106.1_WP_079292523.:	TTTCACTAGGTGCCTTAATCGCTTTATTTGAGAGGGCTGTATCCTTCTACGCTGAATTGG	pgi	Glucose-6-phosphate isomerase	WP_079292523.:	23262..24845	Glycolysis/Gluconeogenesis	COG0166
106.1_WP_079292523.:	CUST_499_PI442190811	HOT208_60m_808G21		106.1_WP_079292523.:	ATGCATATGATCAACCTGGAGTTGAAGCTGGAAAGAAAGCAGCCGCAAATATTATTGAGT	pgi	Glucose-6-phosphate isomerase	WP_079292523.:	23262..24845	Glycolysis/Gluconeogenesis	COG0166
106.1_WP_079292523.:	CUST_500_PI442190811	HOT208_60m_808G21		106.1_WP_079292523.:	TTCCGTTTTTGGTAATAAAGGATCTACAGATCAACATGCTTATGTTCAGCAACTGAGAGA	pgi	Glucose-6-phosphate isomerase	WP_079292523.:	23262..24845	Glycolysis/Gluconeogenesis	COG0166
106.1_WP_079292523.:	CUST_501_PI442190811	HOT208_60m_808G21		106.1_WP_079292523.:	ACTCCGTGAAATGTGCTTTGGTAATGATAATTATTTAGTTAAGGGCGATTGGTCAAATCC	pgi	Glucose-6-phosphate isomerase	WP_079292523.:	23262..24845	Glycolysis/Gluconeogenesis	COG0166
106.1_WP_079292523.:	CUST_502_PI442190811	HOT208_60m_808G21		106.1_WP_079292523.:	AAGGAACCAGATCGGCACTTTCTACTGAAAATAGACAAAGTATCACTATTACATTAGAAA	pgi	Glucose-6-phosphate isomerase	WP_079292523.:	23262..24845	Glycolysis/Gluconeogenesis	COG0166
106.1_WP_079292544.:	CUST_613_PI442190811	HOT208_60m_808G21		106.1_WP_079292544.:	AAATTCAGACCACCAACACCTATCTTGGCAACTACTACAGAGAGAAGTGTAGCGAGAAGA	pykF	Pyruvate kinase	WP_079292544.:	43734..45524	Glycolysis	COG0469
106.1_WP_079292544.:	CUST_614_PI442190811	HOT208_60m_808G21		106.1_WP_079292544.:	CCACTAAAAGCTATTGAGAGCCACTTACCTAGTACCATCCCAAATGCTATTAGTGCAGCA	pykF	Pyruvate kinase	WP_079292544.:	43734..45524	Glycolysis	COG0469
106.1_WP_079292544.:	CUST_615_PI442190811	HOT208_60m_808G21		106.1_WP_079292544.:	AGCAATGGCGACCTTATTACACTGCAGCTAAATGAAGGTGTTATATACATGGGGCAAATT	pykF	Pyruvate kinase	WP_079292544.:	43734..45524	Glycolysis	COG0469
106.1_WP_079292544.:	CUST_616_PI442190811	HOT208_60m_808G21		106.1_WP_079292544.:	ATATAGCTAGACAACTTGATGCCGGAGCTATCATCCCTTTAACAAAATCAGGCTCTACCG	pykF	Pyruvate kinase	WP_079292544.:	43734..45524	Glycolysis	COG0469
106.1_WP_079292544.:	CUST_617_PI442190811	HOT208_60m_808G21		106.1_WP_079292544.:	TGCTCAGGAAATGAGGATACTAAATCAAGGAGATTTAGTAGTTCAAACTGCAGGCACATT	pykF	Pyruvate kinase	WP_079292544.:	43734..45524	Glycolysis	COG0469
106.1_WP_079292544.:	CUST_618_PI442190811	HOT208_60m_808G21		106.1_WP_079292544.:	CGGCTTAGTAAGAAAAATTGTATCAAGAGGAATTTCAATAGGGGAAATCGGTGTAACAGG	pykF	Pyruvate kinase	WP_079292544.:	43734..45524	Glycolysis	COG0469
106.1_WP_079292551.:	CUST_658_PI442190811	HOT208_60m_808G21		106.1_WP_079292551.:	ATAACGGAACAGGAATGCATGTTCACCAAAGTTTATGGAAGAGTGGACAGCCTCTATTCT	glnA	Glutamine synthetase/ glutamate--ammonialigase	WP_079292551.:	52744..54165	Nitrogen Metabolism	COG0174
106.1_WP_079292551.:	CUST_659_PI442190811	HOT208_60m_808G21		106.1_WP_079292551.:	CCTAAGGCTAAAAGATTAGAATTCAGATCAGGCGATGCACTTGCAAACCCTTATTTGGCA	glnA	Glutamine synthetase/ glutamate--ammonialigase	WP_079292551.:	52744..54165	Nitrogen Metabolism	COG0174
106.1_WP_079292551.:	CUST_660_PI442190811	HOT208_60m_808G21		106.1_WP_079292551.:	TCTCAAACAGCAAGATGGTACATAGGAGGCATACTTAAACATGCACCTTCATTCCTAGCA	glnA	Glutamine synthetase/ glutamate--ammonialigase	WP_079292551.:	52744..54165	Nitrogen Metabolism	COG0174
106.1_WP_079292551.:	CUST_661_PI442190811	HOT208_60m_808G21		106.1_WP_079292551.:	TCCAGGATTCGAAGCACCTGTAAATCTTGTTTATTCTGAGGGTAATAGATCAGCTGCAGT	glnA	Glutamine synthetase/ glutamate--ammonialigase	WP_079292551.:	52744..54165	Nitrogen Metabolism	COG0174
106.1_WP_079292551.:	CUST_662_PI442190811	HOT208_60m_808G21		106.1_WP_079292551.:	GTGCCGGCCAACACGAGCTTGGAATGAAATTTGATTCGTTAATAAATGCTGCTGATAAC	glnA	Glutamine synthetase/ glutamate--ammonialigase	WP_079292551.:	52744..54165	Nitrogen Metabolism	COG0174
106.1_WP_079292551.:	CUST_663_PI442190811	HOT208_60m_808G21		106.1_WP_079292551.:	GAGGTACAACAACTAAGACAAAGGCCACATCCACATGAATTCTTCATGTACTACGATGCA	glnA	Glutamine synthetase/ glutamate--ammonialigase	WP_079292551.:	52744..54165	Nitrogen Metabolism	COG0174
107.1_WP_079292598.:	CUST_950_PI442190811	HOT208_60m_808G21		107.1_WP_079292598.:	AGTGTTAATTGGTTTAGATCTACTTTTTGATGAATTAACTGAAACTGAATATAGTGGAGA	kaiB	Circadian clock protein KaiB	WP_079292598.:	21275..21592	Circadian Rhythm	COG4251
107.1_WP_079292598.:	CUST_951_PI442190811	HOT208_60m_808G21		107.1_WP_079292598.:	AGCCACTCCAACGTTATCTAAAATTTTACCGCCCCCAGTTAGAAAAATAATAGGTGATCT	kaiB	Circadian clock protein KaiB	WP_079292598.:	21275..21592	Circadian Rhythm	COG4251
107.1_WP_079292598.:	CUST_952_PI442190811	HOT208_60m_808G21		107.1_WP_079292598.:	AGGAGTTTATGCTTTGAAGGTTATCGATGTACTTAAGCAACCGCAACTCGCAGAAGAAGA	kaiB	Circadian clock protein KaiB	WP_079292598.:	21275..21592	Circadian Rhythm	COG4251
107.1_WP_079292598.:	CUST_953_PI442190811	HOT208_60m_808G21		107.1_WP_079292598.:	TACTCCTAATTCGATGAGAGCTTTAAATACTTTAAGAGAAATTTTA	kaiB	Circadian clock protein KaiB	WP_079292598.:	21275..21592	Circadian Rhythm	COG4251
107.1_WP_079292598.:	CUST_954_PI442190811	HOT208_60m_808G21		107.1_WP_079292598.:	TGGCAGCGAGAAAAACATATATTTTAAAACTCTATGTTGCTGGAAATACTCCTAATTCGA	kaiB	Circadian clock protein KaiB	WP_079292598.:	21275..21592	Circadian Rhythm	COG4251
107.1_WP_079292599.:	CUST_955_PI442190811	HOT208_60m_808G21		107.1_WP_079292599.:	TGGCGATTGATTCTCTCTCTGCATTAGCCAGAGGTGTAAGTTTGAATGCATTTAGACAGT	kaiC	circadian clock protein KaiC	WP_079292599.:	21653..23182	Circadian Rhythm	COG0467
107.1_WP_079292599.:	CUST_956_PI442190811	HOT208_60m_808G21		107.1_WP_079292599.:	TACTTTTTGCATATGAAGAATCTAGGGCTCAATTGCTTAGGAATGCTACTAGCTGGGGCA	kaiC	circadian clock protein KaiC	WP_079292599.:	21653..23182	Circadian Rhythm	COG0467
107.1_WP_079292599.:	CUST_957_PI442190811	HOT208_60m_808G21		107.1_WP_079292599.:	TTCTCGCCACTGGAGCAACTGGTACAGGTAAAACAATGCTTGTATCGAAATTTGTTGAAG	kaiC	circadian clock protein KaiC	WP_079292599.:	21653..23182	Circadian Rhythm	COG0467
107.1_WP_079292599.:	CUST_958_PI442190811	HOT208_60m_808G21		107.1_WP_079292599.:	GGTGAGATGGCTCGAGCATTAAATGTTTTTAAAATGCGGGGATCATGGCATGATAAACGA	kaiC	circadian clock protein KaiC	WP_079292599.:	21653..23182	Circadian Rhythm	COG0467
107.1_WP_079292599.:	CUST_959_PI442190811	HOT208_60m_808G21		107.1_WP_079292599.:	TGCTGTTACCGGTTATACAAAACAAGAGGAAATAGCAGGCTTTTTTACTAATACTGCGGA	kaiC	circadian clock protein KaiC	WP_079292599.:	21653..23182	Circadian Rhythm	COG0467
107.1_WP_079292599.:	CUST_960_PI442190811	HOT208_60m_808G21		107.1_WP_079292599.:	TTAGTGGTGCCCCTCATAGAGTTGTGCCTGATCAAAATGTTCAAAATGTTTTTAAAGGAA	kaiC	circadian clock protein KaiC	WP_079292599.:	21653..23182	Circadian Rhythm	COG0467
107.1_WP_079292685.:	CUST_1068_PI442190811	HOT208_60m_808G21		107.1_WP_079292685.:	TGTAGAGGATGTACTGAATGTACCAACTTATATCGCGAAGTGGATCCCGATTCCTTTCCA	glcH	GPH family sugar transporter	WP_079292685.:	40971..42317	GlcH sugar transporter	COG2211
107.1_WP_079292685.:	CUST_1069_PI442190811	HOT208_60m_808G21		107.1_WP_079292685.:	GCTTCAACAGCTTTTCTTATCCCTTGGTCACTACTTCCTGATGCAATAGACGAAGATCCA	glcH	GPH family sugar transporter	WP_079292685.:	40971..42317	GlcH sugar transporter	COG2211
107.1_WP_079292685.:	CUST_1070_PI442190811	HOT208_60m_808G21		107.1_WP_079292685.:	GAAGCCGGCAGGACTATATACTGCTTGGATGGTACTTATTCAGAAGATTGGTATCGCATT	glcH	GPH family sugar transporter	WP_079292685.:	40971..42317	GlcH sugar transporter	COG2211
107.1_WP_079292685.:	CUST_1071_PI442190811	HOT208_60m_808G21		107.1_WP_079292685.:	AACAGGATTTCAGCTTTAAACTATGGAGGGATTATGTGGATTATTTCATGCACGGCAGCA	glcH	GPH family sugar transporter	WP_079292685.:	40971..42317	GlcH sugar transporter	COG2211
107.1_WP_079292685.:	CUST_1072_PI442190811	HOT208_60m_808G21		107.1_WP_079292685.:	AGTTATCACGCTTTATATTCTTCTCTGGTGCGCCTTACAATTGATGCAAACAGTAGCGTT	glcH	GPH family sugar transporter	WP_079292685.:	40971..42317	GlcH sugar transporter	COG2211
107.1_WP_079292685.:	CUST_1073_PI442190811	HOT208_60m_808G21		107.1_WP_079292685.:	AGGCCTTCAGGAAAAGTTGAAGCCATAACACTTCAATTCAAAAGGATCTTCAGAAATAA	glcH	GPH family sugar transporter	WP_079292685.:	40971..42317	GlcH sugar transporter	COG2211
107.1_WP_079292627.:	CUST_1149_PI442190811	HOT208_60m_808G21		107.1_WP_079292627.:	TAGCAGGAGCTCCTCTTCAAGAAGCATTTAGGAATGCTGATGATGTTTTAAGGATGGGAG	ftsZ	cell division protein FtsZ	WP_079292627.:	54377..55492	Cell Division	COG0206
107.1_WP_079292627.:	CUST_1150_PI442190811	HOT208_60m_808G21		107.1_WP_079292627.:	GTGGTCGATGAGGCAATGGAAGGGGAGATACAAGTTACTGTAATTGCTACAGGATTTGAA	ftsZ	cell division protein FtsZ	WP_079292627.:	54377..55492	Cell Division	COG0206
107.1_WP_079292627.:	CUST_1151_PI442190811	HOT208_60m_808G21		107.1_WP_079292627.:	TGCGACAAGCAGAGGAGGGGATAGCAAGACTAGCTGAAAACGTTGATACTCTTATAGTTA	ftsZ	cell division protein FtsZ	WP_079292627.:	54377..55492	Cell Division	COG0206
107.1_WP_079292627.:	CUST_1152_PI442190811	HOT208_60m_808G21		107.1_WP_079292627.:	CAGCACAGGCAGCCATGAATAGTCCTTTATTAGAAGCAGCAAGAATTGATGGAGCTAAAG	ftsZ	cell division protein FtsZ	WP_079292627.:	54377..55492	Cell Division	COG0206
107.1_WP_079292627.:	CUST_1153_PI442190811	HOT208_60m_808G21		107.1_WP_079292627.:	GTTGCAGAAGTAGCCAAACAAAGTGGGGCTTTAACCGTAGGTATAGTAACCAAACCATTT	ftsZ	cell division protein FtsZ	WP_079292627.:	54377..55492	Cell Division	COG0206
107.1_WP_079292627.:	CUST_1154_PI442190811	HOT208_60m_808G21		107.1_WP_079292627.:	GCTGCGTTATAAATATTACTGGAGGCAAAGACATGACTTTAGAAGACATGACCTCTGCAT	ftsZ	cell division protein FtsZ	WP_079292627.:	54377..55492	Cell Division	COG0206
107.1_WP_079292646.:	CUST_1272_PI442190811	HOT208_60m_808G21		107.1_WP_079292646.:	ATGGCTCATGATAGAGCTACTAGTAATCCTGAAATCTGGAGAAAAGCGTCTGCTTTTGGC	pdhA	Pyruvate dehydrogenase E1 component alpha subunit	WP_079292646.:	77610..78683	Glycolysis/Krebs cycle	COG1071
107.1_WP_079292646.:	CUST_1273_PI442190811	HOT208_60m_808G21		107.1_WP_079292646.:	TCCAGTTGCCTTAGGGGCAGCCTTTTCAAGTAAATACAAAAAAGAAGTTGCTGGGAATAG	pdhA	Pyruvate dehydrogenase E1 component alpha subunit	WP_079292646.:	77610..78683	Glycolysis/Krebs cycle	COG1071
107.1_WP_079292646.:	CUST_1274_PI442190811	HOT208_60m_808G21		107.1_WP_079292646.:	AAGTAATGAGTGAACTTTTTGGTAAATCCACTGGTTGTAGTAAAGGCCGAGGTGGATCCA	pdhA	Pyruvate dehydrogenase E1 component alpha subunit	WP_079292646.:	77610..78683	Glycolysis/Krebs cycle	COG1071
107.1_WP_079292646.:	CUST_1275_PI442190811	HOT208_60m_808G21		107.1_WP_079292646.:	GGGGATGGGACTTGCAATAATGGGCAGTTTTTTGAATGTTTAAATATGGCCCAGTTATGG	pdhA	Pyruvate dehydrogenase E1 component alpha subunit	WP_079292646.:	77610..78683	Glycolysis/Krebs cycle	COG1071
107.1_WP_079292646.:	CUST_1276_PI442190811	HOT208_60m_808G21		107.1_WP_079292646.:	GGGCAGGAGAAGGGCCCACACTTTTAGAATGTTTAACTTATAGGTATAGAGGGCATTCTC	pdhA	Pyruvate dehydrogenase E1 component alpha subunit	WP_079292646.:	77610..78683	Glycolysis/Krebs cycle	COG1071
107.1_WP_079292646.:	CUST_1277_PI442190811	HOT208_60m_808G21		107.1_WP_079292646.:	TGCACTTATTTTCAAGAGAGCATCACCTACTCGGAGGATATGCATTTATTGGAGAGGGAA	pdhA	Pyruvate dehydrogenase E1 component alpha subunit	WP_079292646.:	77610..78683	Glycolysis/Krebs cycle	COG1071
108.1_WP_011376108.:	CUST_1674_PI442190811	HOT208_60m_808G21		108.1_WP_011376108.:	AATCATCGGATACGATGCTTACACTCAAAGCCAAGGAACAGCTTTTGTAGTTTTCCAAGG	rbcS	Ribulose bisphosphate carboxylase small subunit	WP_011376108.:	32641..32982	Carbon fixation	COG4451
108.1_WP_011376108.:	CUST_1675_PI442190811	HOT208_60m_808G21		108.1_WP_011376108.:	AGACCTTAACTTGGTTGTAAGTGAACTAGAGGCATGTCATAGAGCATACCCTGATCACCA	rbcS	Ribulose bisphosphate carboxylase small subunit	WP_011376108.:	32641..32982	Carbon fixation	COG4451
108.1_WP_011376108.:	CUST_1676_PI442190811	HOT208_60m_808G21		108.1_WP_011376108.:	TCCCAGTGGAAGTATGCAAACTTATTGGTCTTATTGGAAGCTCCCATTTTTT	rbcS	Ribulose bisphosphate carboxylase small subunit	WP_011376108.:	32641..32982	Carbon fixation	COG4451
108.1_WP_011376108.:	CUST_1677_PI442190811	HOT208_60m_808G21		108.1_WP_011376108.:	AAATTGCATACATAATTGCTCAAGGCTGGAGTCCTGTTATTGAGCATGTTCATCCCAGTG	rbcS	Ribulose bisphosphate carboxylase small subunit	WP_011376108.:	32641..32982	Carbon fixation	COG4451
108.1_WP_011376108.:	CUST_1678_PI442190811	HOT208_60m_808G21		108.1_WP_011376108.:	CAGTTGCAACCCTGGAAACATTCGGTTTCTTACCACCGATGACCCAGGAAGAAATATAC	rbcS	Ribulose bisphosphate carboxylase small subunit	WP_011376108.:	32641..32982	Carbon fixation	COG4451
108.1_WP_002805854.:	CUST_1679_PI442190811	HOT208_60m_808G21		108.1_WP_002805854.:	CAATAGAGTCGCTTTAGAAGCTTGTGTAAAAGCACGTAATGCTGGTCGCGAAATCGAAAA	rbcL	Ribulose 1,5-bisphosphate carboxylase large subunit	WP_002805854.:	33076..34491	Carbon fixation	COG1850
108.1_WP_002805854.:	CUST_1681_PI442190811	HOT208_60m_808G21		108.1_WP_002805854.:	CGTATGGCATATGCCTGCACTCTTAGCAATTTTTGGAGATGATTCTTGTCTTCAGTTCGG	rbcL	Ribulose 1,5-bisphosphate carboxylase large subunit	WP_002805854.:	33076..34491	Carbon fixation	COG1850
108.1_WP_079292737.:	CUST_1854_PI442190811	HOT208_60m_808G21		108.1_WP_079292737.:	AGATTGCTGCAACTTGGGAGGGAATTAAGGCAGCTGAAATTTTGGAAAAAGAGGGTATTA	tal	Transaldolase	WP_079292737.:	61882..62883	Pentose Phosphate	COG0176
108.1_WP_079292737.:	CUST_1855_PI442190811	HOT208_60m_808G21		108.1_WP_079292737.:	GCGCGTATCTACAGAAGTTGATGCAAGACTGAGCTTTGATACCGAAGCTACTGTAGAAAA	tal	Transaldolase	WP_079292737.:	61882..62883	Pentose Phosphate	COG0176
108.1_WP_079292737.:	CUST_1856_PI442190811	HOT208_60m_808G21		108.1_WP_079292737.:	GCCAAGCGGTAACTTGTGCTAGTGCAAATATAACTCTAATTTCTCCTTTCGTAGGCCGTA	tal	Transaldolase	WP_079292737.:	61882..62883	Pentose Phosphate	COG0176
108.1_WP_079292737.:	CUST_1857_PI442190811	HOT208_60m_808G21		108.1_WP_079292737.:	TGGAAGATCAAATGGCAAGTGAAAAGCTGAGTGAAGGTATCACTGGATTCAGTAAGGCTA	tal	Transaldolase	WP_079292737.:	61882..62883	Pentose Phosphate	COG0176
108.1_WP_079292737.:	CUST_1858_PI442190811	HOT208_60m_808G21		108.1_WP_079292737.:	GGATTGGCATAAAGCAAAAACTGGTAAAACTAGTTTTGTTGGATCTGAAGACCCTGGTGT	tal	Transaldolase	WP_079292737.:	61882..62883	Pentose Phosphate	COG0176
108.1_WP_079292737.:	CUST_1859_PI442190811	HOT208_60m_808G21		108.1_WP_079292737.:	AGCAGGTTGCGATCTTTTAACAATCGCACCAAAATTTCTTGAGGAACTGAAAAAAGAAAA	tal	Transaldolase	WP_079292737.:	61882..62883	Pentose Phosphate	COG0176
112.1_546	CUST_2996_PI442190811	HOT208_60m_808G21		112.1_546	TGGATAACTGGCGTTGGCAAGGGGTTCCTTTTTATTTGAGAACAGGTAAAAGACTACCTA	zwf	glucose-6-phosphate dehydrogenase zwf		47792..49320	Pentose Phosphate	COG0364
112.1_546	CUST_2997_PI442190811	HOT208_60m_808G21		112.1_546	TAGCTGATGCAATGCTTTCTGACCCAACATTATTTACTCGAAGTGATGAGGTAGAGGCAG	zwf	glucose-6-phosphate dehydrogenase zwf		47792..49320	Pentose Phosphate	COG0364
112.1_546	CUST_2998_PI442190811	HOT208_60m_808G21		112.1_546	CGGGATATGCTTCAAAATCATATGACTCAAATGCTTGCAGTTACTGCAATGGAACCTCCT	zwf	glucose-6-phosphate dehydrogenase zwf		47792..49320	Pentose Phosphate	COG0364
112.1_546	CUST_2999_PI442190811	HOT208_60m_808G21		112.1_546	CAAAACTTGCTGACGAAAATGAACCGTGGAAGTGTTGCATAAGAGGTCAATATGGAGAGG	zwf	glucose-6-phosphate dehydrogenase zwf		47792..49320	Pentose Phosphate	COG0364
112.1_546	CUST_3000_PI442190811	HOT208_60m_808G21		112.1_546	TCCTGCCCCAAATCAACTTGTTCTTAGAATTCAGCCAAATGAAGGTGCTACTTTCAAATT	zwf	glucose-6-phosphate dehydrogenase zwf		47792..49320	Pentose Phosphate	COG0364
112.1_546	CUST_3001_PI442190811	HOT208_60m_808G21		112.1_546	TGAGGTAAAATCTCCTGGTTCTGGAATGAAATCAAGACCTGTTGTGAGATGGAATTTTCT	zwf	glucose-6-phosphate dehydrogenase zwf		47792..49320	Pentose Phosphate	COG0364
112.1_WP_079292920.:	CUST_3002_PI442190811	HOT208_60m_808G21		112.1_WP_079292920.:	TGGAAGCTTGGATGAGTCGCCTGAAATCTTCGAACATTTCACTGATCATGATCTAAGGTT	opcA	Glucose-6-P dehydrogenase subunit opcA	WP_079292920.:	49323..50624	Pentose Phosphate	COG3429
112.1_WP_079292920.:	CUST_3003_PI442190811	HOT208_60m_808G21		112.1_WP_079292920.:	TAACACTTGTGTAATACTTGGCTGTGAATCAGTGGAATGTATGAGACTTGAAGCAGGGGG	opcA	Glucose-6-P dehydrogenase subunit opcA	WP_079292920.:	49323..50624	Pentose Phosphate	COG3429
112.1_WP_079292920.:	CUST_3004_PI442190811	HOT208_60m_808G21		112.1_WP_079292920.:	TGAAAAATTGGAGGGAATCATTGGCAATGATTTTTGATCCGCCTTCGAGGAGACCTATTT	opcA	Glucose-6-P dehydrogenase subunit opcA	WP_079292920.:	49323..50624	Pentose Phosphate	COG3429
112.1_WP_079292920.:	CUST_3005_PI442190811	HOT208_60m_808G21		112.1_WP_079292920.:	TCGCGGGAGATCATATGATTCAAGCGTTGTTTTTGATTTCATGGATTAGTGATAAACTTG	opcA	Glucose-6-P dehydrogenase subunit opcA	WP_079292920.:	49323..50624	Pentose Phosphate	COG3429
112.1_WP_079292920.:	CUST_3006_PI442190811	HOT208_60m_808G21		112.1_WP_079292920.:	TCCCTTATCTTTGGGAAATCCAAGTATTCATTTAGGACAAGTTATTGGATTGAGGCTGAT	opcA	Glucose-6-P dehydrogenase subunit opcA	WP_079292920.:	49323..50624	Pentose Phosphate	COG3429
112.1_WP_079292920.:	CUST_3007_PI442190811	HOT208_60m_808G21		112.1_WP_079292920.:	ACATCAGAGTACGATGTTAGTAAATTATTGGGAAGTAGTAGAGGAAATACCAGTCCTCTT	opcA	Glucose-6-P dehydrogenase subunit opcA	WP_079292920.:	49323..50624	Pentose Phosphate	COG3429
113.1_WP_079292931.:	CUST_3054_PI442190811	HOT208_60m_808G21		113.1_WP_079292931.:	AGGCTCTGCCTCCATAGTTAATAAGCAATCAATAGAAGATTCTTTGAAAATTGGATTAAA	eda	2-dehydro-3-deoxyphosphogluconate aldolase	WP_079292931.:	7933..8559	Entner-Dudoroff	COG0800
113.1_WP_079292931.:	CUST_3055_PI442190811	HOT208_60m_808G21		113.1_WP_079292931.:	AGATAGTTCGATAAATATACTCAACTATAAAAATATTGATTTCATTGCGGCTGGAGGACT	eda	2-dehydro-3-deoxyphosphogluconate aldolase	WP_079292931.:	7933..8559	Entner-Dudoroff	COG0800
113.1_WP_079292931.:	CUST_3056_PI442190811	HOT208_60m_808G21		113.1_WP_079292931.:	AGTTGGTCTAACAACGAAAATTGGTTTGATTTTGTATCCGATATCAAAATTAAATATCCA	eda	2-dehydro-3-deoxyphosphogluconate aldolase	WP_079292931.:	7933..8559	Entner-Dudoroff	COG0800
113.1_WP_079292931.:	CUST_3057_PI442190811	HOT208_60m_808G21		113.1_WP_079292931.:	TTTTTCGATGATGAAATTTTGGGATAAAGATCTTTTCAATTATGCGCAGTCAAAAAATTA	eda	2-dehydro-3-deoxyphosphogluconate aldolase	WP_079292931.:	7933..8559	Entner-Dudoroff	COG0800
113.1_WP_079292931.:	CUST_3058_PI442190811	HOT208_60m_808G21		113.1_WP_079292931.:	GATCTTAAGGAAGCGATAGATTTAAACTGCAACATTATCAAAATTTACCCAATAAAAAGT	eda	2-dehydro-3-deoxyphosphogluconate aldolase	WP_079292931.:	7933..8559	Entner-Dudoroff	COG0800
113.1_WP_079292931.:	CUST_3059_PI442190811	HOT208_60m_808G21		113.1_WP_079292931.:	AGGGTATAAAGCAGTTGTAATTGGAGATAAAGCTATCAAAAATAAAAATTTTGATCCAAA	eda	2-dehydro-3-deoxyphosphogluconate aldolase	WP_079292931.:	7933..8559	Entner-Dudoroff	COG0800
113.1_WP_079292942.:	CUST_3145_PI442190811	HOT208_60m_808G21		113.1_WP_079292942.:	GAGACTGAAGATACAACAATCGCAGATTTATCTGTCGCCACAAGATCGGGTCAGATCAAG	eno	Enolase	WP_079292942.:	25158..26450	Glycolysis/Gluconeogenesis	COG0148
113.1_WP_079292942.:	CUST_3146_PI442190811	HOT208_60m_808G21		113.1_WP_079292942.:	AGAGGACGGTTTAGCTGAGGATGATTGGGAGGGTTGGTCAGAATTAAACAAAGAATTAGG	eno	Enolase	WP_079292942.:	25158..26450	Glycolysis/Gluconeogenesis	COG0148
113.1_WP_079292942.:	CUST_3147_PI442190811	HOT208_60m_808G21		113.1_WP_079292942.:	AAGCAGGGGACTTATTATTAGAAGCAATTCAAAAAGCAGGATTTAAGCCTGGTGAGCAGG	eno	Enolase	WP_079292942.:	25158..26450	Glycolysis/Gluconeogenesis	COG0148
113.1_WP_079292942.:	CUST_3148_PI442190811	HOT208_60m_808G21		113.1_WP_079292942.:	TGAGGAGGAACTAGGAAATCAAGCAAGATTTGCTGGAGCTCTAGGTTTAGGTCCAAAAAA	eno	Enolase	WP_079292942.:	25158..26450	Glycolysis/Gluconeogenesis	COG0148
113.1_WP_079292942.:	CUST_3149_PI442190811	HOT208_60m_808G21		113.1_WP_079292942.:	AAATCATTACTTGATCAAAAAGGTCTATCTACCGCTGTAGGCGATGAGGGTGGATTTGCC	eno	Enolase	WP_079292942.:	25158..26450	Glycolysis/Gluconeogenesis	COG0148
113.1_WP_079292942.:	CUST_3150_PI442190811	HOT208_60m_808G21		113.1_WP_079292942.:	GGAACATTAACTGAAACTTTGGAAGCTATTGAGTTAGCTAAAATGTCTGGTTTCACAAGT	eno	Enolase	WP_079292942.:	25158..26450	Glycolysis/Gluconeogenesis	COG0148
115.1_WP_079293008.:	CUST_3601_PI442190811	HOT208_60m_808G21		115.1_WP_079293008.:	TGCACAACGATATTATTTAGCAGTAGCGCAACTGTTTATGGTGATTCGCAAGAAATACCA	galE	UDP-glucose 4-epimerase	WP_079293008.:	74215..75264	Galactose metabolism	COG1087
115.1_WP_079293008.:	CUST_3602_PI442190811	HOT208_60m_808G21		115.1_WP_079293008.:	TCCTATTGGTTCCCATCCTTCAGGATTAATTGGGGAGAATCCAAATAGTAAGTCAGAAAA	galE	UDP-glucose 4-epimerase	WP_079293008.:	74215..75264	Galactose metabolism	COG1087
115.1_WP_079293008.:	CUST_3603_PI442190811	HOT208_60m_808G21		115.1_WP_079293008.:	TGGCCAACACCAGATGGGACTTGTTATCGAGATTATATACATATAGTTGATCTTGCAGAA	galE	UDP-glucose 4-epimerase	WP_079293008.:	74215..75264	Galactose metabolism	COG1087
115.1_WP_079293008.:	CUST_3604_PI442190811	HOT208_60m_808G21		115.1_WP_079293008.:	TGCAGTAATTCATTTTGCTGGCTTGAAGGCCATACAAGAATCTACATTAAAACCAATTCT	galE	UDP-glucose 4-epimerase	WP_079293008.:	74215..75264	Galactose metabolism	COG1087
115.1_WP_079293008.:	CUST_3605_PI442190811	HOT208_60m_808G21		115.1_WP_079293008.:	TTGGGAAAGGAAACTCATGCAGCGTTTTGGAACTTATAAATACTTTTGAAAGAGTTAACA	galE	UDP-glucose 4-epimerase	WP_079293008.:	74215..75264	Galactose metabolism	COG1087
115.1_WP_079293008.:	CUST_3606_PI442190811	HOT208_60m_808G21		115.1_WP_079293008.:	TGAATTTACAAGTCGAAGAGTTGGTGACATCGCAATTTATTATGCAGATACAACGAAAAT	galE	UDP-glucose 4-epimerase	WP_079293008.:	74215..75264	Galactose metabolism	COG1087
115.1_WP_079293009.:	CUST_3607_PI442190811	HOT208_60m_808G21		115.1_WP_079293009.:	AGCCCAACGACTTAGCTCGGTTAATACTATTTCAGCTATTTGTGAAACAACAGGAGCGAA	ugd	UDP-glucose 6-dehydrogenase/nucleotide sugar dehydrogenase	WP_079293009.:	75381..76793	Amino sugar and nucleotide sugar r	COG1004
115.1_WP_079293009.:	CUST_3608_PI442190811	HOT208_60m_808G21		115.1_WP_079293009.:	TGGATAAAAGGATAGGTGAGTATTTTCTCAATCATGGGCCTGGTTTTGGAGGTAGCTGTT	ugd	UDP-glucose 6-dehydrogenase/nucleotide sugar dehydrogenase	WP_079293009.:	75381..76793	Amino sugar and nucleotide sugar r	COG1004
115.1_WP_079293009.:	CUST_3609_PI442190811	HOT208_60m_808G21		115.1_WP_079293009.:	TGGAATTTGTTCTCTGAGTCAATGCGAAAACCAGCCTGGATTTTTGATTGTAGAGATGTT	ugd	UDP-glucose 6-dehydrogenase/nucleotide sugar dehydrogenase	WP_079293009.:	75381..76793	Amino sugar and nucleotide sugar r	COG1004
115.1_WP_079293009.:	CUST_3610_PI442190811	HOT208_60m_808G21		115.1_WP_079293009.:	TCAATTGAAGAATCTATGAAGAATGCAGATGCTGCAGTAATTTTGACAGAGTGGGAAGAA	ugd	UDP-glucose 6-dehydrogenase/nucleotide sugar dehydrogenase	WP_079293009.:	75381..76793	Amino sugar and nucleotide sugar r	COG1004
115.1_WP_079293009.:	CUST_3611_PI442190811	HOT208_60m_808G21		115.1_WP_079293009.:	ACTTCTGAAATTGATCATTTGAAGATTAACCTCTGGAAGTGCGGATATGCCAAATTTTCT	ugd	UDP-glucose 6-dehydrogenase/nucleotide sugar dehydrogenase	WP_079293009.:	75381..76793	Amino sugar and nucleotide sugar r	COG1004
115.1_WP_079293009.:	CUST_3612_PI442190811	HOT208_60m_808G21		115.1_WP_079293009.:	ATCCAAAAGTTAGTTTTGAAAGAATAGCTTTAGACTTGAACTTAGATGTTGCTGGTGAGG	ugd	UDP-glucose 6-dehydrogenase/nucleotide sugar dehydrogenase	WP_079293009.:	75381..76793	Amino sugar and nucleotide sugar r	COG1004
116.1_WP_025881193.:	CUST_3907_PI442190811	HOT208_60m_808G21		116.1_WP_025881193.:	CTTTTTTATGAAGGTTTGCATGGCGGGGTAGAAGGTGATGGATACAATGTGGCATCTTAT	prk	Phosphoribulokinase	WP_025881193.:	29647..30543	Carbon Fixation	COG3954
116.1_WP_025881193.:	CUST_3908_PI442190811	HOT208_60m_808G21		116.1_WP_025881193.:	TGCAGGAATATACCAACTCCTGATGAGAGCTTTGTGATCATACATTTCAGAAAAGGGGCA	prk	Phosphoribulokinase	WP_025881193.:	29647..30543	Carbon Fixation	COG3954
116.1_WP_025881193.:	CUST_3909_PI442190811	HOT208_60m_808G21		116.1_WP_025881193.:	AGAAGCAGAAGAACACAATACAAGACTTGGTACATCCTTAGAACCTGGGCAATTTACCCC	prk	Phosphoribulokinase	WP_025881193.:	29647..30543	Carbon Fixation	COG3954
116.1_WP_025881193.:	CUST_3910_PI442190811	HOT208_60m_808G21		116.1_WP_025881193.:	CTATGGCAGACGCTCTTTCAAAAGGTGAAAATTTCTCTCATTTTGGCCCAGAGGCTAATC	prk	Phosphoribulokinase	WP_025881193.:	29647..30543	Carbon Fixation	COG3954
116.1_WP_025881193.:	CUST_3911_PI442190811	HOT208_60m_808G21		116.1_WP_025881193.:	TGCAGAAAGAGGTTACTCAGCGGAAACCATTGTGGATACTATATTGAGAAGAATGCCTGA	prk	Phosphoribulokinase	WP_025881193.:	29647..30543	Carbon Fixation	COG3954
116.1_WP_025881193.:	CUST_3912_PI442190811	HOT208_60m_808G21		116.1_WP_025881193.:	TGTCAAGTCCAACAAGTATCGTTGTAAATGGAGGAAAAAATGGGTTTTGCAATGGAACTAA	prk	Phosphoribulokinase	WP_025881193.:	29647..30543	Carbon Fixation	COG3954
116.1_WP_079293060.:	CUST_3931_PI442190811	HOT208_60m_808G21		116.1_WP_079293060.:	AAGAACTATGGAAAGGTGCGTTCCTGCATCTGTTCCTGGAATAGTTTTCTTATCAGGAGG	fda	Fructose-1,6-bisphosphate aldolase class I	WP_079293060.:	33164..34231	Glycolysis/Gluconeogenesis	COG0191
116.1_WP_079293060.:	CUST_3932_PI442190811	HOT208_60m_808G21		116.1_WP_079293060.:	AAAGCATTAATAGCAAGAGCCCAAGCAAATTCTGAAGCTTCAAAAGGAGCCTATGTAGCA	fda	Fructose-1,6-bisphosphate aldolase class I	WP_079293060.:	33164..34231	Glycolysis/Gluconeogenesis	COG0191
116.1_WP_079293060.:	CUST_3933_PI442190811	HOT208_60m_808G21		116.1_WP_079293060.:	CCGTTCAAGAATCTGGTTTGGTACCAATTATTGAACCTGAAATCTTAATGGACGGCGACC	fda	Fructose-1,6-bisphosphate aldolase class I	WP_079293060.:	33164..34231	Glycolysis/Gluconeogenesis	COG0191
116.1_WP_079293060.:	CUST_3934_PI442190811	HOT208_60m_808G21		116.1_WP_079293060.:	TATGGAATGTTTCATTCTCCTATGGAAGAGCATTACAGCACTCATGCTTAAAGGCCTGGA	fda	Fructose-1,6-bisphosphate aldolase class I	WP_079293060.:	33164..34231	Glycolysis/Gluconeogenesis	COG0191
116.1_WP_079293060.:	CUST_3935_PI442190811	HOT208_60m_808G21		116.1_WP_079293060.:	TAAAGGTCTAAATCCACTTCCTGGAGGAGGAGATGTAGAAACTTTTTGCTCTGGCCTAGA	fda	Fructose-1,6-bisphosphate aldolase class I	WP_079293060.:	33164..34231	Glycolysis/Gluconeogenesis	COG0191
116.1_WP_079293060.:	CUST_3936_PI442190811	HOT208_60m_808G21		116.1_WP_079293060.:	CTTCTATGACTGTTAATGGAGCAGATTGTCCAACAAAGGCTGATCCCATGAAAGTTGCTG	fda	Fructose-1,6-bisphosphate aldolase class I	WP_079293060.:	33164..34231	Glycolysis/Gluconeogenesis	COG0191
116.1_WP_079293061.:	CUST_3937_PI442190811	HOT208_60m_808G21		116.1_WP_079293061.:	AAGGAAGCCAACAGGAGAAGTTCTTGCAATAAGCAGAATTGCTGAAATCCATAAAGCACT	cbbA	Fructose-1,6-bisphosphate aldolase class II	WP_079293061.:	34389..35462	Glycolysis/Gluconeogenesis	COG0191
116.1_WP_079293061.:	CUST_3939_PI442190811	HOT208_60m_808G21		116.1_WP_079293061.:	CAGCATTTGCTGACAAGGCAAACTTTGACCCAAGACATTTCAACAAACCTGCTAGAAAAT	cbbA	Fructose-1,6-bisphosphate aldolase class II	WP_079293061.:	34389..35462	Glycolysis/Gluconeogenesis	COG0191
116.1_WP_079293061.:	CUST_3940_PI442190811	HOT208_60m_808G21		116.1_WP_079293061.:	TGAGGGTGAACTTTCTACAGATATGCTTTTGACTGATCCTGAAGAAGCGGCAGATTTTGT	cbbA	Fructose-1,6-bisphosphate aldolase class II	WP_079293061.:	34389..35462	Glycolysis/Gluconeogenesis	COG0191
116.1_WP_079293061.:	CUST_3941_PI442190811	HOT208_60m_808G21		116.1_WP_079293061.:	AGTTTGTCTTGACAGATACAAGCAGTTCTGGTGCGAAGGTCAAGCAAGTAAGATCAAACA	cbbA	Fructose-1,6-bisphosphate aldolase class II	WP_079293061.:	34389..35462	Glycolysis/Gluconeogenesis	COG0191
116.1_WP_079293061.:	CUST_3942_PI442190811	HOT208_60m_808G21		116.1_WP_079293061.:	GTTAGGAAAGTCAACATTGATACCGATAACAGACTTGCTTTCACTGCCGCGGTTAGAGAG	cbbA	Fructose-1,6-bisphosphate aldolase class II	WP_079293061.:	34389..35462	Glycolysis/Gluconeogenesis	COG0191
116.1_WP_079293072.:	CUST_3998_PI442190811	HOT208_60m_808G21		116.1_WP_079293072.:	TAGACTTCCTCAGAACCTTGTTCAGGCCATGAGAGACTGTTTTGGCTCTCATACATATGA	edd	phosphogluconate dehydrogenase (NADP(+)-dependent, decarboxyla	WP_079293072.:	42732..44150	Pentose Phosphate/Entner-Dudorof	COG2084
116.1_WP_079293072.:	CUST_3999_PI442190811	HOT208_60m_808G21		116.1_WP_079293072.:	GCAGTATATAACTATGGTTTGAATATGCCGTCAATTGCTCAAATATGGAAAGGTGGTTGC	edd	phosphogluconate dehydrogenase (NADP(+)-dependent, decarboxyla	WP_079293072.:	42732..44150	Pentose Phosphate/Entner-Dudorof	COG2084
116.1_WP_079293072.:	CUST_4000_PI442190811	HOT208_60m_808G21		116.1_WP_079293072.:	TGATGCTGTAGTTCTTGCCACAATTGCTAGCTATGCTCAGGGTATGGACATTTTAAGAGA	edd	phosphogluconate dehydrogenase (NADP(+)-dependent, decarboxyla	WP_079293072.:	42732..44150	Pentose Phosphate/Entner-Dudorof	COG2084
116.1_WP_079293072.:	CUST_4001_PI442190811	HOT208_60m_808G21		116.1_WP_079293072.:	GGGTTATGGACTGTTGTAAGTGCTTTAGAACTGGGGGTATCAGTCCCAACTATTTATGCA	edd	phosphogluconate dehydrogenase (NADP(+)-dependent, decarboxyla	WP_079293072.:	42732..44150	Pentose Phosphate/Entner-Dudorof	COG2084
116.1_WP_079293072.:	CUST_4002_PI442190811	HOT208_60m_808G21		116.1_WP_079293072.:	GATGAGATAACTGGAGATGATGTCGTGGAAAAAAATATTAGATAAAGCTGGCCAGAAAGGT	edd	phosphogluconate dehydrogenase (NADP(+)-dependent, decarboxyla	WP_079293072.:	42732..44150	Pentose Phosphate/Entner-Dudorof	COG2084
116.1_WP_079293072.:	CUST_4003_PI442190811	HOT208_60m_808G21		116.1_WP_079293072.:	ACTTGCAGAAGCTTATGACCTTATGAAGAGAGTCAAAGGTATGAATGGACAGCAGATGTC	edd	phosphogluconate dehydrogenase (NADP(+)-dependent, decarboxyla	WP_079293072.:	42732..44150	Pentose Phosphate/Entner-Dudorof	COG2084
116.1_WP_079293073.:	CUST_4004_PI442190811	HOT208_60m_808G21		116.1_WP_079293073.:	TGTTATGGGAGCCGATTTCTTTGAATCTCCTGAAGAGAGGATTGAATTAAGGAAAGGGGG	glgC	Glucose-1-phosphate adenylyltransferase	WP_079293073.:	44246..45541	Glycogen Metabolism	COG0448
116.1_WP_079293073.:	CUST_4005_PI442190811	HOT208_60m_808G21		116.1_WP_079293073.:	ATGATTATTGGGAAGATATCGGCACCATTGGTGCATTCTTTGAGTCAAACCTTGCATTGA	glgC	Glucose-1-phosphate adenylyltransferase	WP_079293073.:	44246..45541	Glycogen Metabolism	COG0448
116.1_WP_079293073.:	CUST_4006_PI442190811	HOT208_60m_808G21		116.1_WP_079293073.:	GGAACGCCCCTTGGAGTAGGTGAAGGGACTACTGTAAAAAGAGCAATTCTTGATAAGAAT	glgC	Glucose-1-phosphate adenylyltransferase	WP_079293073.:	44246..45541	Glycogen Metabolism	COG0448
116.1_WP_079293073.:	CUST_4007_PI442190811	HOT208_60m_808G21		116.1_WP_079293073.:	AGTGAAAAGCCTACTGGAGAGAAGTTGAAGGCTATGGCAGTAGATACTTCAAAATTTGGA	glgC	Glucose-1-phosphate adenylyltransferase	WP_079293073.:	44246..45541	Glycogen Metabolism	COG0448
116.1_WP_079293073.:	CUST_4008_PI442190811	HOT208_60m_808G21		116.1_WP_079293073.:	AGGAGTCAGCTGCTGAAAAACCATATCTAGCCTCTATGGGTATTTACGTTTTTAGCAGAA	glgC	Glucose-1-phosphate adenylyltransferase	WP_079293073.:	44246..45541	Glycogen Metabolism	COG0448
116.1_WP_079293073.:	CUST_4009_PI442190811	HOT208_60m_808G21		116.1_WP_079293073.:	TGCATTGTGTTTTAGGTGTAAGAAGTAGGATTGAAAGTGACTCGGTTCTTGAGGATACTC	glgC	Glucose-1-phosphate adenylyltransferase	WP_079293073.:	44246..45541	Glycogen Metabolism	COG0448
126.1_WP_079293189.:	CUST_4759_PI442190811	HOT208_60m_808G21		126.1_WP_079293189.:	GGCAACCTATGCATTATTGGAGACGTTAATCCAGGGGCAATAGTTTCCGCAAAAAAAAAC	minC	septum site-determining protein MinC	WP_079293189.:	47992..48654	Cell Division	COG0850
126.1_WP_079293189.:	CUST_4760_PI442190811	HOT208_60m_808G21		126.1_WP_079293189.:	TAGGGATCGCATTCGCCGGAAAAACTGGAAATAAAAATGCCTCTATTGCATCACTTTACT	minC	septum site-determining protein MinC	WP_079293189.:	47992..48654	Cell Division	COG0850
126.1_WP_079293189.:	CUST_4761_PI442190811	HOT208_60m_808G21		126.1_WP_079293189.:	AGCCTTTACAATTACGAATTGCAGATGTAATAGCTATTGGACCAAAGGATAAGCCCAACA	minC	septum site-determining protein MinC	WP_079293189.:	47992..48654	Cell Division	COG0850
126.1_WP_079293189.:	CUST_4762_PI442190811	HOT208_60m_808G21		126.1_WP_079293189.:	TTAAGTCAAAAGACAAAGACGATGTTCTTTACAAAGGTACAGTTCGATCGGGAGACAGAA	minC	septum site-determining protein MinC	WP_079293189.:	47992..48654	Cell Division	COG0850
126.1_WP_079293189.:	CUST_4763_PI442190811	HOT208_60m_808G21		126.1_WP_079293189.:	AACAGTTTTCTTCCATAAAGGAACCACTGGAAGCAAAAATTTTCGCAATCAATGAGTCAA	minC	septum site-determining protein MinC	WP_079293189.:	47992..48654	Cell Division	COG0850
126.1_WP_079293189.:	CUST_4764_PI442190811	HOT208_60m_808G21		126.1_WP_079293189.:	ACCCAGAAATTGCGGTCTTAGATAAACAAACGATAATAATCAAACCACACTTAATCGAAA	minC	septum site-determining protein MinC	WP_079293189.:	47992..48654	Cell Division	COG0850
126.1_WP_079293190.:	CUST_4765_PI442190811	HOT208_60m_808G21		126.1_WP_079293190.:	AAGCTATCGTTGTTACTAACCCAGAATTATCCGCAGTGCGGGATGCAGATAGAGTAATTG	minD	septum site-determining protein MinD	WP_079293190.:	48766..49581	Cell Division	COG2894
126.1_WP_079293190.:	CUST_4766_PI442190811	HOT208_60m_808G21		126.1_WP_079293190.:	AAAGGAACCTAATCTTGCTCTTTTACCTGCTGGAGATCCAAGGATGTTGGATTGGATGAA	minD	septum site-determining protein MinD	WP_079293190.:	48766..49581	Cell Division	COG2894
126.1_WP_079293190.:	CUST_4767_PI442190811	HOT208_60m_808G21		126.1_WP_079293190.:	TGTTCAGGTAAAGGAGGAGTTGGTAAAACCACTTTAACTGCAAACCTAGGCATAGCACTT	minD	septum site-determining protein MinD	WP_079293190.:	48766..49581	Cell Division	COG2894
126.1_WP_079293190.:	CUST_4768_PI442190811	HOT208_60m_808G21		126.1_WP_079293190.:	TCGCATCATCTATACGGCTCAAGATGTTCTAGACAAGAATTGTCGTCTTGACCAAGCATT	minD	septum site-determining protein MinD	WP_079293190.:	48766..49581	Cell Division	COG2894
126.1_WP_079293190.:	CUST_4769_PI442190811	HOT208_60m_808G21		126.1_WP_079293190.:	TTGTCTTAGTAGATTGTCCTGCTGGTGTAGAAGATGGCTTTAAAAATGCTCTTGCAGCGT	minD	septum site-determining protein MinD	WP_079293190.:	48766..49581	Cell Division	COG2894
126.1_WP_079293190.:	CUST_4770_PI442190811	HOT208_60m_808G21		126.1_WP_079293190.:	TCGCCCTAACATGATGGCTAGTCAAGAAATGTTATCTATCGAAGATGTTCAAGGGATCCT	minD	septum site-determining protein MinD	WP_079293190.:	48766..49581	Cell Division	COG2894
126.1_WP_079293191.:	CUST_4771_PI442190811	HOT208_60m_808G21		126.1_WP_079293191.:	TCAGGAGAATTAAAGTTTAAAAAAGACGATAAGAATGATAAAACTCAAAAAGATGCCAAA	minE	septum site-determining protein MinE	WP_079293191.:	49583..49918	Cell Division	COG0851
126.1_WP_079293191.:	CUST_4772_PI442190811	HOT208_60m_808G21		126.1_WP_079293191.:	TGAGATTGACTTTGAAGAAGTGGCAGTAAGTTTAGAGACGGAAGACAGAATGACTGCTCT	minE	septum site-determining protein MinE	WP_079293191.:	49583..49918	Cell Division	COG0851
126.1_WP_079293191.:	CUST_4773_PI442190811	HOT208_60m_808G21		126.1_WP_079293191.:	AGTTCCTTAACAACTGATCTTTTAGATAAAATGAGGAAAGAAATACTCGATGTTGTTGCT	minE	septum site-determining protein MinE	WP_079293191.:	49583..49918	Cell Division	COG0851
126.1_WP_079293191.:	CUST_4774_PI442190811	HOT208_60m_808G21		126.1_WP_079293191.:	AGGCAGAGAAACGTCTAGTGCAAATACAGCTAGAGAAAGATTACAACTTGTACTTGCTCA	minE	septum site-determining protein MinE	WP_079293191.:	49583..49918	Cell Division	COG0851
136.1_WP_079293229.:	CUST_5095_PI442190811	HOT208_60m_808G21		136.1_WP_079293229.:	AAAAATCCAGAGAAAATTAAGGCCTTGAAAGAAGTTTCAAGGGCTGAGGCCAGGATTCAA	atpC	ATP synthase epsilon subunit	WP_079293229.:	301..705	Energy Metabolism	COG0355
136.1_WP_079293229.:	CUST_5096_PI442190811	HOT208_60m_808G21		136.1_WP_079293229.:	TGCTGAAATTGGTTCTGAAATTAATGTTCAAAATGCTGAACAAGATCTTAAAGAAGCAAA	atpC	ATP synthase epsilon subunit	WP_079293229.:	301..705	Energy Metabolism	COG0355
136.1_WP_079293229.:	CUST_5097_PI442190811	HOT208_60m_808G21		136.1_WP_079293229.:	TGGCGTTTTAAGACTCAGAATGAATTCCCAATGGAAATCAATAGCTCTTATGGGAGGCTT	atpC	ATP synthase epsilon subunit	WP_079293229.:	301..705	Energy Metabolism	COG0355
136.1_WP_079293229.:	CUST_5098_PI442190811	HOT208_60m_808G21		136.1_WP_079293229.:	CGAAGCTGAAGAAGTAATCCTTCCAAGTACTACTGGTCAACTTGGTATACTACCGGGACA	atpC	ATP synthase epsilon subunit	WP_079293229.:	301..705	Energy Metabolism	COG0355
143.1_954	CUST_5135_PI442190811	HOT208_60m_808G21		143.1_954	CTAAAGTAATGGGAAGAGACGCCAGTGAATTTTTCGGCCCATATGCAAATAGCGATGAAA	rbsK	Sugar kinase, ribokinase family		140..500	Pentose Phosphate	COG0524
143.1_954	CUST_5136_PI442190811	HOT208_60m_808G21		143.1_954	ATTCTTATGGCTGTGGCGATTCTTTTGCTGCTGGGATTCTTTATGGAATGGCATCTAAAT	rbsK	Sugar kinase, ribokinase family		140..500	Pentose Phosphate	COG0524
143.1_954	CUST_5137_PI442190811	HOT208_60m_808G21		143.1_954	AAACCGAGGGAGAGAAGGGAGGCATAATATTCCCAGGAGGAAGATATAAGGCTCTTAAAA	rbsK	Sugar kinase, ribokinase family		140..500	Pentose Phosphate	COG0524
143.1_954	CUST_5138_PI442190811	HOT208_60m_808G21		143.1_954	AGGTAGTAATCTTGATCCCGGAGAAGTTTTTTCTTTTTCTGAATTATCGTTAAAACCCAA	rbsK	Sugar kinase, ribokinase family		140..500	Pentose Phosphate	COG0524
154.1_WP_079293242.:	CUST_5298_PI442190811	HOT208_60m_808G21		154.1_WP_079293242.:	AAGAAATAGTGGTGCTACAGCGGTAACAGAAGGAGCAGGTGATCATTGTTGTGAATACAT	glsF	Ferredoxin-dependent glutamate synthase	WP_079293242.:	15487..20058	Nitrogen Metabolism	COG0069
154.1_WP_079293242.:	CUST_5299_PI442190811	HOT208_60m_808G21		154.1_WP_079293242.:	GGTAACACCTGTCTTTATGGAGCAACAGGCGGAAAATTATTTGCATTAGGAAAATCGGGA	glsF	Ferredoxin-dependent glutamate synthase	WP_079293242.:	15487..20058	Nitrogen Metabolism	COG0069
154.1_WP_079293242.:	CUST_5300_PI442190811	HOT208_60m_808G21		154.1_WP_079293242.:	GTTCCACAGGAAGGAATATTGGTGCGGGCATGACTGGTGGAATAGCTTTTATAATTGATG	glsF	Ferredoxin-dependent glutamate synthase	WP_079293242.:	15487..20058	Nitrogen Metabolism	COG0069
154.1_WP_079293242.:	CUST_5301_PI442190811	HOT208_60m_808G21		154.1_WP_079293242.:	AAATCGCAGAACTTCATGGCAACACTGGCTTCAATGGCGAACTCAACTTAAATTTCAAAG	glsF	Ferredoxin-dependent glutamate synthase	WP_079293242.:	15487..20058	Nitrogen Metabolism	COG0069
154.1_WP_079293242.:	CUST_5302_PI442190811	HOT208_60m_808G21		154.1_WP_079293242.:	TGCAGGACAAAGCTTTGGTGCCTTTTTATTGAAGGGAATGAATGTTCAATTAATTGGAGA	glsF	Ferredoxin-dependent glutamate synthase	WP_079293242.:	15487..20058	Nitrogen Metabolism	COG0069
154.1_WP_079293242.:	CUST_5303_PI442190811	HOT208_60m_808G21		154.1_WP_079293242.:	GAATGGAGGAATGCTCACAATAATTCCACCAAAAATAGAAAAATCTTCCTCCGAACAGGT	glsF	Ferredoxin-dependent glutamate synthase	WP_079293242.:	15487..20058	Nitrogen Metabolism	COG0069
154.1_WP_011819135.:	CUST_5354_PI442190811	HOT208_60m_808G21		154.1_WP_011819135.:	TATAATCCATTCGGGGTAAACTCTTTATCTCCTTGGGCTTGGATGTTCCTTTTCGGTCAC	psaB	Photosystem I P700 chlorophyll A apoprotein A2 PsaB	WP_011819135.:	26461..28689	Photosystem I	COG2885
154.1_WP_011819135.:	CUST_5355_PI442190811	HOT208_60m_808G21		154.1_WP_011819135.:	[illegible]	psaB	Photosystem I P700 chlorophyll A apoprotein A2 PsaB	WP_011819135.:	26461..28689	Photosystem I	COG2885
154.1_WP_011819135.:	CUST_5356_PI442190811	HOT208_60m_808G21		154.1_WP_011819135.:	[illegible]	psaB	Photosystem I P700 chlorophyll A apoprotein A2 PsaB	WP_011819135.:	26461..28689	Photosystem I	COG2885
154.1_WP_011819135.:	CUST_5357_PI442190811	HOT208_60m_808G21		154.1_WP_011819135.:	[illegible]	psaB	Photosystem I P700 chlorophyll A apoprotein A2 PsaB	WP_011819135.:	26461..28689	Photosystem I	COG2885
154.1_WP_011819135.:	CUST_5358_PI442190811	HOT208_60m_808G21		154.1_WP_011819135.:	[illegible]	psaB	Photosystem I P700 chlorophyll A apoprotein A2 PsaB	WP_011819135.:	26461..28689	Photosystem I	COG2885
154.1_WP_079293250.:	CUST_5360_PI442190811	HOT208_60m_808G21		154.1_WP_079293250.:	[illegible]	psaA	Photosystem I P700 chlorophyll A apoprotein A1 PsaA	WP_079293250.:	28717..31020	Photosystem I	COG2885
154.1_WP_079293250.:	CUST_5361_PI442190811	HOT208_60m_808G21		154.1_WP_079293250.:	[illegible]	psaA	Photosystem I P700 chlorophyll A apoprotein A1 PsaA	WP_079293250.:	28717..31020	Photosystem I	COG2885
154.1_WP_079293250.:	CUST_5362_PI442190811	HOT208_60m_808G21		154.1_WP_079293250.:	[illegible]	psaA	Photosystem I P700 chlorophyll A apoprotein A1 PsaA	WP_079293250.:	28717..31020	Photosystem I	COG2885
154.1_WP_079293250.:	CUST_5363_PI442190811	HOT208_60m_808G21		154.1_WP_079293250.:	[illegible]	psaA	Photosystem I P700 chlorophyll A apoprotein A1 PsaA	WP_079293250.:	28717..31020	Photosystem I	COG2885
154.1_WP_079293250.:	CUST_5364_PI442190811	HOT208_60m_808G21		154.1_WP_079293250.:	[illegible]	psaA	Photosystem I P700 chlorophyll A apoprotein A1 PsaA	WP_079293250.:	28717..31020	Photosystem I	COG2885
154.1_WP_079293250.:	CUST_5365_PI442190811	HOT208_60m_808G21		154.1_WP_079293250.:	[illegible]	psaA	Photosystem I P700 chlorophyll A apoprotein A1 PsaA	WP_079293250.:	28717..31020	Photosystem I	COG2885

163.1_WP_079293288.:	CUST_5627_PI442190811	HOT208_60m_808G21 163.1_WP_079293288.:	ATCACTGGAGCTAGCAGAGGAATTGGTAAAGAAATTGCTTTAGAACTAAGCCGATTGGGA	gdh	3-oxoacyl-acyl-carrier-protein reductase	WP_079293288.:	3..752	Entner-Dudoroff	COG1028
163.1_WP_079293288.:	CUST_5628_PI442190811	HOT208_60m_808G21 163.1_WP_079293288.:	CATCTGTTGTTGGCATAATTGGTAATCCTGGACAAGCAAATTATTCTGCAGCCAAAGCTG	gdh	3-oxoacyl-acyl-carrier-protein reductase	WP_079293288.:	3..752	Entner-Dudoroff	COG1028
163.1_WP_079293288.:	CUST_5629_PI442190811	HOT208_60m_808G21 163.1_WP_079293288.:	TTCAAGTGATGCAGGAAGTTACATTACAGGGCAAACAATCAGTATAGACGGGGGTATGAG	gdh	3-oxoacyl-acyl-carrier-protein reductase	WP_079293288.:	3..752	Entner-Dudoroff	COG1028
163.1_WP_079293288.:	CUST_5630_PI442190811	HOT208_60m_808G21 163.1_WP_079293288.:	TGCTGGAATTACTAGAGATGGACTATTGATGAGAATGAAATCGGAACAATGGGATGATGT	gdh	3-oxoacyl-acyl-carrier-protein reductase	WP_079293288.:	3..752	Entner-Dudoroff	COG1028
163.1_WP_079293288.:	CUST_5631_PI442190811	HOT208_60m_808G21 163.1_WP_079293288.:	ACCAAAACTTGTGCTAAAGAATTTGCCTCAAGAGGTATAAATGTTAATGCAATAGCTCCA	gdh	3-oxoacyl-acyl-carrier-protein reductase	WP_079293288.:	3..752	Entner-Dudoroff	COG1028
163.1_WP_079293288.:	CUST_5632_PI442190811	HOT208_60m_808G21 163.1_WP_079293288.:	TTTCAAAAGAGGATTCTGTCAGTTCAGCTTTTGAAGAAATAATCAAAATTAATGGCACCA	gdh	3-oxoacyl-acyl-carrier-protein reductase	WP_079293288.:	3..752	Entner-Dudoroff	COG1028
017.1_WP_079292342.:	CUST_5956_PI442190811	HOT208_60m_808G21 017.1_WP_079292342.:	AATTAGTTATATTCATGCAGCGGGATATCCCGCTGGTGAAATGAAACATGGTCCAATAGC	glmS	Glutamine--fructose-6-phosphatetransaminase(isomerizing)	WP_079292342.:	37075..38970	Amino Acid metabolism	COG0449
017.1_WP_079292342.:	CUST_5957_PI442190811	HOT208_60m_808G21 017.1_WP_079292342.:	GGAAGGCAGGTCTCAAATATAATTGATATATGTGCGGGAATAGAAGTTGGAGTTGCAGCA	glmS	Glutamine--fructose-6-phosphatetransaminase(isomerizing)	WP_079292342.:	37075..38970	Amino Acid metabolism	COG0449
017.1_WP_079292342.:	CUST_5958_PI442190811	HOT208_60m_808G21 017.1_WP_079292342.:	ATTGCCTCACCTGGAGAGGTTTTTGATAAAGTTATCAGTAATGCTCAAGAAGCAAAAGCT	glmS	Glutamine--fructose-6-phosphatetransaminase(isomerizing)	WP_079292342.:	37075..38970	Amino Acid metabolism	COG0449
017.1_WP_079292342.:	CUST_5959_PI442190811	HOT208_60m_808G21 017.1_WP_079292342.:	GCCTTTACAGTTATTGAGTTACCATATTGCAGCTCATAGAGGACTTGATGTGGATCAACC	glmS	Glutamine--fructose-6-phosphatetransaminase(isomerizing)	WP_079292342.:	37075..38970	Amino Acid metabolism	COG0449
017.1_WP_079292342.:	CUST_5960_PI442190811	HOT208_60m_808G21 017.1_WP_079292342.:	GGGATTGCTCCTGAATGTAATGGAACTGAAATCTTTGATTATTTGATGAAAGTTCCTTCC	glmS	Glutamine--fructose-6-phosphatetransaminase(isomerizing)	WP_079292342.:	37075..38970	Amino Acid metabolism	COG0449
017.1_WP_079292342.:	CUST_5961_PI442190811	HOT208_60m_808G21 017.1_WP_079292342.:	ACCGCCATTACTGGAAGATCTTTTAGAGAAACATAATAAATCTTCAGAAAAGCTAGCACA	glmS	Glutamine--fructose-6-phosphatetransaminase(isomerizing)	WP_079292342.:	37075..38970	Amino Acid metabolism	COG0449
169.1_1147	CUST_6127_PI442190811	HOT208_60m_808G21 169.1_1147	TCCATTACCAGATGGTGCTATTTATGAATTAGAACTACCAATACCTACTCTCAATACAAT	cyoC	heme-copper oxidase subunit III		355..506	Respiration	COG1845
169.1_1147	CUST_6128_PI442190811	HOT208_60m_808G21 169.1_1147	AATTGCGGACGGAATGACTTTTGCTGGATTCTTTGCTGCTTATTTAACTTATAAAGCAGT	cyoC	heme-copper oxidase subunit III		355..506	Respiration	COG1845
193.1_WP_079293338.:	CUST_6462_PI442190811	HOT208_60m_808G21 193.1_WP_079293338.:	AGGCCTTTAATTCCAGCTGATGTTAACTGGACAAAAACCACAGAAGAAACCTTTTCAGTT	ppc	Phosphoenolpyruvate carboxylase	WP_079293338.:	4761..6839	Krebs cycle and related genes	COG2352
193.1_WP_079293338.:	CUST_6463_PI442190811	HOT208_60m_808G21 193.1_WP_079293338.:	AGTGCGTCTGATTTGCTTGAAGTTCTCTTACTTGCAAAAGAAATGGGACTTCTTGATCAA	ppc	Phosphoenolpyruvate carboxylase	WP_079293338.:	4761..6839	Krebs cycle and related genes	COG2352
193.1_WP_079293338.:	CUST_6464_PI442190811	HOT208_60m_808G21 193.1_WP_079293338.:	CAGCCTATCAGGCAATTTTGGCTCAACCAAGCGGCACTTTAAAAGGACGAATAAAA	ppc	Phosphoenolpyruvate carboxylase	WP_079293338.:	4761..6839	Krebs cycle and related genes	COG2352
193.1_WP_079293338.:	CUST_6465_PI442190811	HOT208_60m_808G21 193.1_WP_079293338.:	TGCGAGTCTGTAAACACCCTACTAACTCAGGTTCATATTTTTGGATTTTCTCTAGCAAGT	ppc	Phosphoenolpyruvate carboxylase	WP_079293338.:	4761..6839	Krebs cycle and related genes	COG2352
193.1_WP_079293338.:	CUST_6466_PI442190811	HOT208_60m_808G21 193.1_WP_079293338.:	TTCCTCTTTTTGAAACTGTGGAAGACCTTAAAAGAGCACCAGAAGTAATGGAAAAGTTGT	ppc	Phosphoenolpyruvate carboxylase	WP_079293338.:	4761..6839	Krebs cycle and related genes	COG2352
209.1_1334	CUST_7027_PI442190811	HOT208_60m_808G21 209.1_1334	CTGGGCAATGCTTGGAACAAGTTTATTAGTTGTTTTGTCAACTCCTGTTTTAGCAGGCAC	cyoB/coxA	Cytochrome c oxidase subunit I		<1..453	Respiration	COG0843
209.1_1334	CUST_7028_PI442190811	HOT208_60m_808G21 209.1_1334	TGGTCCTTCCTGCCTTTGGTTTAGTTAGTGAAATTCTTCCTGTACATGCTAGAAAACCAC	cyoB/coxA	Cytochrome c oxidase subunit I		<1..453	Respiration	COG0843
209.1_1334	CUST_7029_PI442190811	HOT208_60m_808G21 209.1_1334	ATGGGGATAGTAGTTTTAGGTTTAGTTGTTTGGGCTCATCACATGTTTACGAGTGGAACG	cyoB/coxA	Cytochrome c oxidase subunit I		<1..453	Respiration	COG0843
209.1_1334	CUST_7030_PI442190811	HOT208_60m_808G21 209.1_1334	TGAGCGTTCTATTACTTGGAGGCAGTTCTATATTTGGTGGAATAAACTTTATCGCCACCA	cyoB/coxA	Cytochrome c oxidase subunit I		<1..453	Respiration	COG0843
209.1_1334	CUST_7031_PI442190811	HOT208_60m_808G21 209.1_1334	TTTAGGTGGCAATGTCGTAGTTTATCAGCATTTATTTTGGTTTTATTCTCAGCCAGCTGT	cyoB/coxA	Cytochrome c oxidase subunit I		<1..453	Respiration	COG0843
209.1_1334	CUST_7032_PI442190811	HOT208_60m_808G21 209.1_1334	GGCACTTTAATTCTACTTAGCTTCGATATCATAGCTAATACAGGTTTTTTCAATC	cyoB/coxA	Cytochrome c oxidase subunit I		<1..453	Respiration	COG0843
050.1_WP_079292432.:	CUST_7398_PI442190811	HOT208_60m_808G21 050.1_WP_079292432.:	CATGCTTTTTGGGTACCAGAGTTCAGAATTAAGCAGGATATTATTCCTGGACAACCGACT	ctaC/coxB	Cytochrome c oxidase subunit II	WP_079292432.:	138..813	Respiration	COG1622
050.1_WP_079292432.:	CUST_7399_PI442190811	HOT208_60m_808G21 050.1_WP_079292432.:	TCCCAAGGGCAATTTCATATCAGGAGAACTACACGTACCTGTTGATCAAAAAGTATCCAT	ctaC/coxB	Cytochrome c oxidase subunit II	WP_079292432.:	138..813	Respiration	COG1622
050.1_WP_079292432.:	CUST_7400_PI442190811	HOT208_60m_808G21 050.1_WP_079292432.:	TCTTTTTGGATTGGTTTTAATGTAAATTTGCTCCCAGCTGAAGCAAGTATTAATGCACCA	ctaC/coxB	Cytochrome c oxidase subunit II	WP_079292432.:	138..813	Respiration	COG1622
050.1_WP_079292432.:	CUST_7401_PI442190811	HOT208_60m_808G21 050.1_WP_079292432.:	AGGAAAAGGAATGATCAGGTAGGCGATGGTATAGCTTTAGAGGGAAATTTAAGCTTAGAA	ctaC/coxB	Cytochrome c oxidase subunit II	WP_079292432.:	138..813	Respiration	COG1622
050.1_WP_079292432.:	CUST_7402_PI442190811	HOT208_60m_808G21 050.1_WP_079292432.:	ACGATCGAATGGGAGGGATGAAAGAACTAAATCATAACCATGAAATGATGAGTTCTAACA	ctaC/coxB	Cytochrome c oxidase subunit II	WP_079292432.:	138..813	Respiration	COG1622
050.1_WP_079292432.:	CUST_7403_PI442190811	HOT208_60m_808G21 050.1_WP_079292432.:	TGGGCTGGAATTAGTCAAACTTCTGATAATGAAATCGCAATAAATAATTTATCAATTGAA	ctaC/coxB	Cytochrome c oxidase subunit II	WP_079292432.:	138..813	Respiration	COG1622
055.1_1414	CUST_7426_PI442190811	HOT208_60m_808G21 055.1_1414	AGAGCTTACAGCAGAAGTGAACTATTGAGGGGGGCACTTTTAACAATTAATGGGATAGCA	ppc	Phosphoenolpyruvate carboxylase		<1..911	Krebs cycle and related genes	COG2352
055.1_1414	CUST_7427_PI442190811	HOT208_60m_808G21 055.1_1414	TTTTGCAGGTTTCACTTCTAAGAAGACTAAGAGACCAGACAAGACAACCCCCAATAAGCG	ppc	Phosphoenolpyruvate carboxylase		<1..911	Krebs cycle and related genes	COG2352
055.1_1414	CUST_7428_PI442190811	HOT208_60m_808G21 055.1_1414	AGCTATTCCATGGGTATTCGGTTGGACACAAAGTAGATTTCTTTTGCCAAGTTGGTTTGG	ppc	Phosphoenolpyruvate carboxylase		<1..911	Krebs cycle and related genes	COG2352
055.1_1414	CUST_7431_PI442190811	HOT208_60m_808G21 055.1_1414	TGGCGTAGGCACTGCATTGTCATCTGAATTAAATTCAGATCCACAACAAATTGAATT	ppc	Phosphoenolpyruvate carboxylase		<1..911	Krebs cycle and related genes	COG2352
179.1_WP_025924170.:	CUST_715_PI442190807	HOT208_60m_813L03 179.1_WP_025924170.:	TCAGGAGAAATAAAATTCAAAAAAACTGATAAAACTGATAAAGCTAACAAAGATATCAAA	minE	septum site-determining protein MinE	WP_025924170.:	21071..21406	Cell Division	COG0851
179.1_WP_025924170.:	CUST_716_PI442190807	HOT208_60m_813L03 179.1_WP_025924170.:	AAGTTTGGAGACTGAGGATAGAATGACCGCACTAGTTGCCAATTTGCCAATCAAAAGAAC	minE	septum site-determining protein MinE	WP_025924170.:	21071..21406	Cell Division	COG0851
179.1_WP_025924170.:	CUST_717_PI442190807	HOT208_60m_813L03 179.1_WP_025924170.:	AGGAAAGAGATCCTTGATGTTGTTGCTAAATATGTTGAAATTGATTTTGAGGAGGTGGCA	minE	septum site-determining protein MinE	WP_025924170.:	21071..21406	Cell Division	COG0851
179.1_WP_025924170.:	CUST_718_PI442190807	HOT208_60m_813L03 179.1_WP_025924170.:	TGTACTTGCTCACGACAGAGTTGATATGAGTTCCTTAACAACTGATCT	minE	septum site-determining protein MinE	WP_025924170.:	21071..21406	Cell Division	COG0851
179.1_WP_025924170.:	CUST_719_PI442190807	HOT208_60m_813L03 179.1_WP_025924170.:	AGGCAGAGAAACGTCTAGTGCGAATACAGCTAGAGAAAGATTACAACTTGTACTTGCTCA	minE	septum site-determining protein MinE	WP_025924170.:	21071..21406	Cell Division	COG0851
179.1_WP_079296389.:	CUST_720_PI442190807	HOT208_60m_813L03 179.1_WP_079296389.:	ATGATGGCTAGTCAAGAAATGTTATCCATCGAGGATGTTCAAGGGATCCTTTCTTTGCCT	minD	septum site-determining protein MinD	WP_079296389.:	21408..22223	Cell Division	COG2894
179.1_WP_079296389.:	CUST_721_PI442190807	HOT208_60m_813L03 179.1_WP_079296389.:	CCTGCTGGTGTAGAAGATGGCTTTAAAAATGCTCTTGCAGCCTGTAAAGAAGCTATTGTT	minD	septum site-determining protein MinD	WP_079296389.:	21408..22223	Cell Division	COG2894
179.1_WP_079296389.:	CUST_722_PI442190807	HOT208_60m_813L03 179.1_WP_079296389.:	AAACAACTTTAACTGCAAACCTAGGAATAGCACTTGCTAATAGCGGAGCAACAACTGCTG	minD	septum site-determining protein MinD	WP_079296389.:	21408..22223	Cell Division	COG2894
179.1_WP_079296389.:	CUST_723_PI442190807	HOT208_60m_813L03 179.1_WP_079296389.:	TCAGCAGTGCGGGATGCAGATAGAGTAATAGGGATTCTTAATACTTCAGATATTGGGCCT	minD	septum site-determining protein MinD	WP_079296389.:	21408..22223	Cell Division	COG2894
179.1_WP_079296389.:	CUST_724_PI442190807	HOT208_60m_813L03 179.1_WP_079296389.:	CTCTTCTACCTGCTGGAGATCCAAGGATGTTGGATTGGATGAAACCCGAAGATATGAAAA	minD	septum site-determining protein MinD	WP_079296389.:	21408..22223	Cell Division	COG2894
179.1_WP_079296389.:	CUST_725_PI442190807	HOT208_60m_813L03 179.1_WP_079296389.:	TCGCATCATTTATACGGCTCAAGATGTTCTAGACAAAAATTGTCGTCTTGACCAAGCATT	minD	septum site-determining protein MinD	WP_079296389.:	21408..22223	Cell Division	COG2894
179.1_WP_079296391.:	CUST_726_PI442190807	HOT208_60m_813L03 179.1_WP_079296391.:	TGGGGCAAATTACTAGGGATCGCATTCGCAGGGAAAAGTGGAAATAAAAATGCATCTATT	minC	septum site-determining protein MinC	WP_079296391.:	22335..22997	Cell Division	COG0850
179.1_WP_079296391.:	CUST_727_PI442190807	HOT208_60m_813L03 179.1_WP_079296391.:	AAAGGTTTTCTTCCATCAAGGAACCTTTAGAAGCAAGAATTTTTGCTGTCAGCGAGTCAA	minC	septum site-determining protein MinC	WP_079296391.:	22335..22997	Cell Division	COG0850
179.1_WP_079296391.:	CUST_728_PI442190807	HOT208_60m_813L03 179.1_WP_079296391.:	TCCTTCACAAAGGAACAGTACGATCCGGTGAAAGAATATCTTCAAATGGAAACCTATGCA	minC	septum site-determining protein MinC	WP_079296391.:	22335..22997	Cell Division	COG0850
179.1_WP_079296391.:	CUST_729_PI442190807	HOT208_60m_813L03 179.1_WP_079296391.:	GCATCACTTTATCTAAACCCTTTACAGTTGAGAATTGCAGATATGATAGCTATAGGACCA	minC	septum site-determining protein MinC	WP_079296391.:	22335..22997	Cell Division	COG0850
179.1_WP_079296391.:	CUST_730_PI442190807	HOT208_60m_813L03 179.1_WP_079296391.:	AGCAAAATTAAAAACCCACCTTAACAAAATTAATGTTTGTTCTTTGCGCATTTACTCAAA	minC	septum site-determining protein MinC	WP_079296391.:	22335..22997	Cell Division	COG0850
179.1_WP_079296391.:	CUST_731_PI442190807	HOT208_60m_813L03 179.1_WP_079296391.:	TCCAGAGATTGCGGTAATAGATAAACAAACGATAATTATCAAACCACACTTAATCGAAAA	minC	septum site-determining protein MinC	WP_079296391.:	22335..22997	Cell Division	COG0850
181.1_WP_079296674.:	CUST_1575_PI442190807	HOT208_60m_813L03 181.1_WP_079296674.:	TACTAGTGCAATTCAGGCATTGATTTTTGCAACTTTAGCGGCCTACTACATTGGAGAAGC	atpI	ATP synthase subunit A	WP_079296674.:	527..1252	Energy Metabolism	COG0356
181.1_WP_079296674.:	CUST_1576_PI442190807	HOT208_60m_813L03 181.1_WP_079296674.:	TTGGGGAGGGGCTTTAATTCCTTGGAGATTGATTAAGTTACCAAGTGGAGAATTAGGAGC	atpI	ATP synthase subunit A	WP_079296674.:	527..1252	Energy Metabolism	COG0356
181.1_WP_079296674.:	CUST_1577_PI442190807	HOT208_60m_813L03 181.1_WP_079296674.:	TTGGCCGATGAACTTGTTGTTGGTGTTCTAGTCTTTTTAGTTCCGCTAATCCTCCCAATA	atpI	ATP synthase subunit A	WP_079296674.:	527..1252	Energy Metabolism	COG0356
181.1_WP_079296674.:	CUST_1578_PI442190807	HOT208_60m_813L03 181.1_WP_079296674.:	GCCTTCAAAACCTGCTTGAGTTCCTCTGGGATTACATAAGAGATCTTACTAGGACGCAAA	atpI	ATP synthase subunit A	WP_079296674.:	527..1252	Energy Metabolism	COG0356
181.1_WP_079296674.:	CUST_1579_PI442190807	HOT208_60m_813L03 181.1_WP_079296674.:	TGCTGGTTTAAGCAATAAGGGTTGGAGATACTTCGAATACTATGTTCACCCAACTCCGAT	atpI	ATP synthase subunit A	WP_079296674.:	527..1252	Energy Metabolism	COG0356
181.1_WP_079296674.:	CUST_1580_PI442190807	HOT208_60m_813L03 181.1_WP_079296674.:	TCCTTGCTAACAAATTTTGCAGCATTAGAAGTTGGTCAACATCTTTATTGGCAAATTGGA	atpI	ATP synthase subunit A	WP_079296674.:	527..1252	Energy Metabolism	COG0356
181.1_WP_079296690.:	CUST_1631_PI442190807	HOT208_60m_813L03 181.1_WP_079296690.:	TTGGTTACAGCCATAGTGCCAAAAGTTGGTTATGAAAAAGCAGCTGAAATTGCAAACCTT	fumC	class II fumarate hydratase	WP_079296690.:	8924..10309	Krebs cycle and related genes	COG0114
181.1_WP_079296690.:	CUST_1632_PI442190807	HOT208_60m_813L03 181.1_WP_079296690.:	ATGGTTTGTACTCAGATAATGGGTCTTGAATATGCAGTCTCAATGGCAAATTCTAGCGGT	fumC	class II fumarate hydratase	WP_079296690.:	8924..10309	Krebs cycle and related genes	COG0114
181.1_WP_079296690.:	CUST_1633_PI442190807	HOT208_60m_813L03 181.1_WP_079296690.:	AAATGAACCTGGAAGTTCTATTATGCCGGGTAAAGTAAATCCAACTCAATGCGAAGCCTT	fumC	class II fumarate hydratase	WP_079296690.:	8924..10309	Krebs cycle and related genes	COG0114
181.1_WP_079296690.:	CUST_1634_PI442190807	HOT208_60m_813L03 181.1_WP_079296690.:	TGGCCTCACATGATCGTCTTGCTCAAGTAATGAGTCAGATAAAAATATTAGCAGGTGCAT	fumC	class II fumarate hydratase	WP_079296690.:	8924..10309	Krebs cycle and related genes	COG0114
181.1_WP_079296690.:	CUST_1635_PI442190807	HOT208_60m_813L03 181.1_WP_079296690.:	ATGAATTGTATTTCTTACCTCTGGGAGGAACTGCGGTTGGTACTGGGATTAATTGTCCAC	fumC	class II fumarate hydratase	WP_079296690.:	8924..10309	Krebs cycle and related genes	COG0114
181.1_WP_079296690.:	CUST_1636_PI442190807	HOT208_60m_813L03 181.1_WP_079296690.:	TCCCCTTACTTTTGGGCAAGAAATATCAGGATGGTCAGAGCAACTTAAAGATGCTGAGAA	fumC	class II fumarate hydratase	WP_079296690.:	8924..10309	Krebs cycle and related genes	COG0114
181.1_WP_079296772.:	CUST_1883_PI442190807	HOT208_60m_813L03 181.1_WP_079296772.:	AGGAAGGAATATTGGTGCGGGCATGACTGGTGGAATAGCTTTTATAATCGATGAGAAGAA	glsF	Ferredoxin-dependent glutamate synthase	WP_079296772.:	60672..65243	Nitrogen Metabolism	COG0069
181.1_WP_079296772.:	CUST_1884_PI442190807	HOT208_60m_813L03 181.1_WP_079296772.:	AATCGGGAGAAAGATTTGCAGTAAGAAATAGTGGTGCTACTGCAGTAACAGAAGGAGCAG	glsF	Ferredoxin-dependent glutamate synthase	WP_079296772.:	60672..65243	Nitrogen Metabolism	COG0069
181.1_WP_079296772.:	CUST_1885_PI442190807	HOT208_60m_813L03 181.1_WP_079296772.:	CAGATAGAAGTGTTTGTGCGAAAATATCAGGTGAAATCGCAGAACTACACGGCAACACTG	glsF	Ferredoxin-dependent glutamate synthase	WP_079296772.:	60672..65243	Nitrogen Metabolism	COG0069
181.1_WP_079296772.:	CUST_1886_PI442190807	HOT208_60m_813L03 181.1_WP_079296772.:	CGAACAGGTTATTTTGGGCAACACATGTCTTTATGGAGCAACAGGTGGGAAATTATTTGC	glsF	Ferredoxin-dependent glutamate synthase	WP_079296772.:	60672..65243	Nitrogen Metabolism	COG0069
181.1_WP_079296772.:	CUST_1887_PI442190807	HOT208_60m_813L03 181.1_WP_079296772.:	CTTCAATGGCGAACTCAACTTAAATTTCAAAGGATATGCAGGTCAAAGCTTTGGTGCCTT	glsF	Ferredoxin-dependent glutamate synthase	WP_079296772.:	60672..65243	Nitrogen Metabolism	COG0069
181.1_WP_079296772.:	CUST_1888_PI442190807	HOT208_60m_813L03 181.1_WP_079296772.:	ACAAGAGTACTTTCAAATTAATAGTTCCCCCAAGCGAAGAAGAAATGCTTGGCATAAAGA	glsF	Ferredoxin-dependent glutamate synthase	WP_079296772.:	60672..65243	Nitrogen Metabolism	COG0069
181.1_WP_079296788.:	CUST_1939_PI442190807	HOT208_60m_813L03 181.1_WP_079296788.:	TCACTTAGTTTGGGCTACTGGCTTTATGTTCTTAATATCATGGCGTGGTTACTGGCAAGA	psaB	Photosystem I P700 chlorophyll A apoprotein A2 PsaB	WP_079296788.:	71646..73874	Photosystem I	COG2885
181.1_WP_079296788.:	CUST_1940_PI442190807	HOT208_60m_813L03 181.1_WP_079296788.:	GCATTAAGTGCTTTCTTACCTATCGGTCCTGCAGATTTCTTAGTCCATCATGCTATCGCT	psaB	Photosystem I P700 chlorophyll A apoprotein A2 PsaB	WP_079296788.:	71646..73874	Photosystem I	COG2885
181.1_WP_079296788.:	CUST_1941_PI442190807	HOT208_60m_813L03 181.1_WP_079296788.:	GCATTGGAAACACCTAGCAATATGGCAGGGTAATGTTGCGCAATTTAATGAATCAGGAAC	psaB	Photosystem I P700 chlorophyll A apoprotein A2 PsaB	WP_079296788.:	71646..73874	Photosystem I	COG2885
181.1_WP_079296788.:	CUST_1942_PI442190807	HOT208_60m_813L03 181.1_WP_079296788.:	CTCCAATAGCTAACCTTGTTGGCTGGAGAGATAAGCCAGTTGCACTTTCAATTGTTCAAG	psaB	Photosystem I P700 chlorophyll A apoprotein A2 PsaB	WP_079296788.:	71646..73874	Photosystem I	COG2885
181.1_WP_079296788.:	CUST_1943_PI442190807	HOT208_60m_813L03 181.1_WP_079296788.:	GGAGGTACTTGTGATAGTTCATCTTGGGACGCCATGTACTTAGCTATGTTTTGGGCATTA	psaB	Photosystem I P700 chlorophyll A apoprotein A2 PsaB	WP_079296788.:	71646..73874	Photosystem I	COG2885
181.1_WP_079296788.:	CUST_1944_PI442190807	HOT208_60m_813L03 181.1_WP_079296788.:	CGGATATAATCCATTCGGAGTTAACTCTTTATCTCCTTGGGCCTGGATGTTCCTATTCGG	psaB	Photosystem I P700 chlorophyll A apoprotein A2 PsaB	WP_079296788.:	71646..73874	Photosystem I	COG2885
181.1_WP_079296790.:	CUST_1945_PI442190807	HOT208_60m_813L03 181.1_WP_079296790.:	TTGGGGACTAACTGGAGGTAACTTCGCTCAAAGTGCCATTACTATTAATGGTTGGCTTAG	psaA	Photosystem I P700 chlorophyll A apoprotein A1 PsaA	WP_079296790.:	73902..76205	Photosystem I	COG2885
181.1_WP_079296790.:	CUST_1950_PI442190807	HOT208_60m_813L03 181.1_WP_079296790.:	GAAAGGTTGCTATCGCTCCAATCCCCTTAGGAACTGCTGACTTGATGATTCATCATATCC	psaA	Photosystem I P700 chlorophyll A apoprotein A1 PsaA	WP_079296790.:	73902..76205	Photosystem I	COG2885
182.1_WP_079296866.:	CUST_2245_PI442190807	HOT208_60m_813L03 182.1_WP_079296866.:	GAAGCAATTCAAAAAGCTGGGTTTAAGCCTGGTGAGCAGGTATCTTTAGCTTTAGATGCT	eno	Enolase	WP_079296866.:	43522..44814	Glycolysis/Gluconeogenesis	COG0148
182.1_WP_079296866.:	CUST_2246_PI442190807	HOT208_60m_813L03 182.1_WP_079296866.:	AATTATCCAATAGTTTCAATTGAGGACGGTTTAGCTGAGGATGATTGGGAGGGTTGGTCA	eno	Enolase	WP_079296866.:	43522..44814	Glycolysis/Gluconeogenesis	COG0148
182.1_WP_079296866.:	CUST_2247_PI442190807	HOT208_60m_813L03 182.1_WP_079296866.:	TCATTACTTGATCATAAAGGTCTATCTACTGCTGTAGGCGATGAGGGTGGATTTGCTCCT	eno	Enolase	WP_079296866.:	43522..44814	Glycolysis/Gluconeogenesis	COG0148
182.1_WP_079296866.:	CUST_2248_PI442190807	HOT208_60m_813L03 182.1_WP_079296866.:	AGGAGGAATTGGGTAATCAAGCAAGATTTGCTGGAGCTCTAGGTTTAGGTCCCAAAAATA	eno	Enolase	WP_079296866.:	43522..44814	Glycolysis/Gluconeogenesis	COG0148
182.1_WP_079296866.:	CUST_2249_PI442190807	HOT208_60m_813L03 182.1_WP_079296866.:	ACAGAAGTGGTGAGACTGAAGATACAACAATTGCAGATTTATCTGTCGCCACAAGATCGG	eno	Enolase	WP_079296866.:	43522..44814	Glycolysis/Gluconeogenesis	COG0148
182.1_WP_079296866.:	CUST_2250_PI442190807	HOT208_60m_813L03 182.1_WP_079296866.:	GGCAGATCAAGACCGGCTCTTTGAGTAGAAGTGAAAGGATTGCAAAATACAATAGGCTTT	eno	Enolase	WP_079296866.:	43522..44814	Glycolysis/Gluconeogenesis	COG0148
186.1_WP_079297028.:	CUST_2781_PI442190807	HOT208_60m_813L03 186.1_WP_079297028.:	GCCACAGGAAGTAGCTTAATGCCGCAGAAAAAAAATCCTGACGTTCCAGAATTGATAAGA	argH	Argininosuccinate lyase	WP_079297028.:	507..1886	Amino Acid metabolism	COG0165
186.1_WP_079297028.:	CUST_2782_PI442190807	HOT208_60m_813L03 186.1_WP_079297028.:	AGAGTGTATGGACATCTTCAGGCATTGTTAACAATGGTCAAAGGGGTACCACTTGCTTAT	argH	Argininosuccinate lyase	WP_079297028.:	507..1886	Amino Acid metabolism	COG0165
186.1_WP_079297028.:	CUST_2783_PI442190807	HOT208_60m_813L03 186.1_WP_079297028.:	TGTTCCTTTTAGGACTGCTTATCAAGTTGTTGGTCAAATGGTTAAATATTGCCTGGAGAG	argH	Argininosuccinate lyase	WP_079297028.:	507..1886	Amino Acid metabolism	COG0165
186.1_WP_079297028.:	CUST_2784_PI442190807	HOT208_60m_813L03 186.1_WP_079297028.:	TGTCGTTAAATCAAGAAATAGCGAAGGTGGTACAGGATTTGTTCAAGTAGAAAAAGAGGT	argH	Argininosuccinate lyase	WP_079297028.:	507..1886	Amino Acid metabolism	COG0165
186.1_WP_079297028.:	CUST_2786_PI442190807	HOT208_60m_813L03 186.1_WP_079297028.:	AGCAGATTCTGTAGAAAACGATTTTTCTAATGCTACTGATTTGGCAGATTACTTAGTCGA	argH	Argininosuccinate lyase	WP_079297028.:	507..1886	Amino Acid metabolism	COG0165
187.1_539	CUST_2805_PI442190807	HOT208_60m_813L03 187.1_539	ATTCGATGATTATTGGGAAGATATCGGCACCATTGGAGCATTCTTTGAGTCAAACCTTGC	glgC	Glucose-1-phosphate adenylyltransferase		<1..753	Glycogen Metabolism	COG0448
187.1_539	CUST_2806_PI442190807	HOT208_60m_813L03 187.1_539	CAGGATTGAAAGTGACTCGGTTCTTGAGGATACTCTTGTTATGGGTGCCGATTTCTTTGA	glgC	Glucose-1-phosphate adenylyltransferase		<1..753	Glycogen Metabolism	COG0448
187.1_539	CUST_2807_PI442190807	HOT208_60m_813L03 187.1_539	GGCGGAACACCTCTTGGAGTAGGTGAAGGGACTACTGTAAAAAGAGCAATTCTTGATAAG	glgC	Glucose-1-phosphate adenylyltransferase		<1..753	Glycogen Metabolism	COG0448
187.1_539	CUST_2808_PI442190807	HOT208_60m_813L03 187.1_539	GTAGATGCTCAAATTACTGATTCAATTGTTTGTGAAGGCACAATCTTGAAGTCGTGCAGT	glgC	Glucose-1-phosphate adenylyltransferase		<1..753	Glycogen Metabolism	COG0448
187.1_539	CUST_2809_PI442190807	HOT208_60m_813L03 187.1_539	TCGAGTAGAAGAAGCAGATAAGCCAGAATTAGGCTTCTATATCAGAAATGGAATTGTTGT	glgC	Glucose-1-phosphate adenylyltransferase		<1..753	Glycogen Metabolism	COG0448
187.1_539	CUST_2810_PI442190807	HOT208_60m_813L03 187.1_539	TGCATTGACTGAGCAACCAAAACCTCCATTTAGTTTTTATGATGAGAAATTTCCAAT	glgC	Glucose-1-phosphate adenylyltransferase		<1..753	Glycogen Metabolism	COG0448
187.1_WP_079297036.:	CUST_2811_PI442190807	HOT208_60m_813L03 187.1_WP_079297036.:	GTGCAGGCAATGAGAGACTGTTTTGGCTCTCATACATATGAAAGAACTGATAGGGAAGGT	edd	phosphogluconate dehydrogenase (NADP(+)-dependent, decarboxyla	WP_079297036.:	849..2267	Pentose Phosphate/Entner-Dudorof	COG2084
187.1_WP_079297036.:	CUST_2812_PI442190807	HOT208_60m_813L03 187.1_WP_079297036.:	AGTAGTTTCTCTATCCACAAAGGCAGGTATCCCAGTCCCATGTTTGTCCAGTACTTTAGA	edd	phosphogluconate dehydrogenase (NADP(+)-dependent, decarboxyla	WP_079297036.:	849..2267	Pentose Phosphate/Entner-Dudorof	COG2084
187.1_WP_079297036.:	CUST_2813_PI442190807	HOT208_60m_813L03 187.1_WP_079297036.:	TGAAACCTTTATTTGATGCTGTAGTCCTTGCAACAATTGCTAGCTATGCCCAAGGTATGG	edd	phosphogluconate dehydrogenase (NADP(+)-dependent, decarboxyla	WP_079297036.:	849..2267	Pentose Phosphate/Entner-Dudorof	COG2084
187.1_WP_079297036.:	CUST_2814_PI442190807	HOT208_60m_813L03 187.1_WP_079297036.:	AAGTGCTCTAGAACTGGGGGTATCAGTCCCAACTATTTATGCATCTTTAAATGCAAGAGT	edd	phosphogluconate dehydrogenase (NADP(+)-dependent, decarboxyla	WP_079297036.:	849..2267	Pentose Phosphate/Entner-Dudorof	COG2084
187.1_WP_079297036.:	CUST_2815_PI442190807	HOT208_60m_813L03 187.1_WP_079297036.:	TGATGTCGTGGAAAAAAATATTAGATAAAGCTGGCCAGAAAGGTACGGGGTTATGGACTGT	edd	phosphogluconate dehydrogenase (NADP(+)-dependent, decarboxyla	WP_079297036.:	849..2267	Pentose Phosphate/Entner-Dudorof	COG2084
187.1_WP_079297036.:	CUST_2816_PI442190807	HOT208_60m_813L03 187.1_WP_079297036.:	TGCAGAAGCTTATGACCTTATGAAGAGAGTTAAAGGTATGAGTGGACAGCAGATGTCAGA	edd	phosphogluconate dehydrogenase (NADP(+)-dependent, decarboxyla	WP_079297036.:	849..2267	Pentose Phosphate/Entner-Dudorof	COG2084
187.1_WP_011376344.:	CUST_2873_PI442190807	HOT208_60m_813L03 187.1_WP_011376344.:	ACGGCTCTCTAGAAGCAGATGCAAAAACACCCGCTAGTTACGAATATAATGTTGCAGTTA	cbbA	Fructose-1,6-bisphosphate aldolase class II	WP_011376344.:	9536..10609	Glycolysis/Gluconeogenesis	COG0191
187.1_WP_011376344.:	CUST_2874_PI442190807	HOT208_60m_813L03 187.1_WP_011376344.:	GGAGTTAGGAAAGTTAACATTGATACCGATAACAGACTTGCTTTCACTGCCGCGGTTAGA	cbbA	Fructose-1,6-bisphosphate aldolase class II	WP_011376344.:	9536..10609	Glycolysis/Gluconeogenesis	COG0191
187.1_WP_011376344.:	CUST_2875_PI442190807	HOT208_60m_813L03 187.1_WP_011376344.:	TGAAGTCCTTGCAATAAGCAGGATTGCTGAAATTCATAAAGCACTGCCAAATACCCATCT	cbbA	Fructose-1,6-bisphosphate aldolase class II	WP_011376344.:	9536..10609	Glycolysis/Gluconeogenesis	COG0191
187.1_WP_011376344.:	CUST_2878_PI442190807	HOT208_60m_813L03 187.1_WP_011376344.:	TGAAGCAAGTTTGTCTTGATAGATACAAGCAGTTCTGGTGCGAAGGTCAAGCAAGTAAGA	cbbA	Fructose-1,6-bisphosphate aldolase class II	WP_011376344.:	9536..10609	Glycolysis/Gluconeogenesis	COG0191
187.1_WP_079297055.:	CUST_2879_PI442190807	HOT208_60m_813L03 187.1_WP_079297055.:	ATTGAAAGTTGCTGAAATGACAATCAGAACTATGGAAAGATGCGTTCCTGCATCTGTCCC	fda	Fructose-1,6-bisphosphate aldolase class I	WP_079297055.:	10757..11824	Glycolysis/Gluconeogenesis	COG0191
187.1_WP_079297055.:	CUST_2880_PI442190807	HOT208_60m_813L03 187.1_WP_079297055.:	TGAACCTGAAATCTTAATGGACGGCGACCATACTATTGAGAAAACCGCTGAAGTTCAAGA	fda	Fructose-1,6-bisphosphate aldolase class I	WP_079297055.:	10757..11824	Glycolysis/Gluconeogenesis	COG0191
187.1_WP_079297055.:	CUST_2881_PI442190807	HOT208_60m_813L03 187.1_WP_079297055.:	TGAAAGAGCAGCAAAATATTATGAACAAGGTGCCAGATTTGCAAAGTGGAGAGCAGTTCT	fda	Fructose-1,6-bisphosphate aldolase class I	WP_079297055.:	10757..11824	Glycolysis/Gluconeogenesis	COG0191
187.1_WP_079297055.:	CUST_2882_PI442190807	HOT208_60m_813L03 187.1_WP_079297055.:	TAAAGGTCTAAATCCACTTCCAGGAGGAGGAGATGTAGAAACTTATTGCTCTGGCCTTGA	fda	Fructose-1,6-bisphosphate aldolase class I	WP_079297055.:	10757..11824	Glycolysis/Gluconeogenesis	COG0191
187.1_WP_079297055.:	CUST_2883_PI442190807	HOT208_60m_813L03 187.1_WP_079297055.:	CACTCATGCTTAAAAGCCTGGAAAGGAAGCGACGTTGAAGGAGGTCAAAAAGCATTAATA	fda	Fructose-1,6-bisphosphate aldolase class I	WP_079297055.:	10757..11824	Glycolysis/Gluconeogenesis	COG0191
187.1_WP_079297055.:	CUST_2884_PI442190807	HOT208_60m_813L03 187.1_WP_079297055.:	CTTCAAAAGGTGCCTATGTTGCAGGATCCCAACCTTCATCTGATGAGCAATTGTTTGTAG	fda	Fructose-1,6-bisphosphate aldolase class I	WP_079297055.:	10757..11824	Glycolysis/Gluconeogenesis	COG0191
187.1_WP_079297059.:	CUST_2901_PI442190807	HOT208_60m_813L03 187.1_WP_079297059.:	AAAAAAGCAATGGCAGACGCTCTTGCAAAAGGTGAAAATTTCTCTCATTTTGGTCCAGAG	prk	Phosphoribulokinase	WP_079297059.:	14442..15338	Carbon Fixation	COG3954
187.1_WP_079297059.:	CUST_2902_PI442190807	HOT208_60m_813L03 187.1_WP_079297059.:	CTCCTGATGAGAGCTTTGTGATCATACATTTCAGAAAAGGGGCAAGAGAGAAATGGGGTA	prk	Phosphoribulokinase	WP_079297059.:	14442..15338	Carbon Fixation	COG3954
187.1_WP_079297059.:	CUST_2903_PI442190807	HOT208_60m_813L03 187.1_WP_079297059.:	GAACAGACGTCCTTTTTTATGAAGGTTTGCATGGCGGGGTAGAAGGTAATGGATACAATG	prk	Phosphoribulokinase	WP_079297059.:	14442..15338	Carbon Fixation	COG3954
187.1_WP_079297059.:	CUST_2904_PI442190807	HOT208_60m_813L03 187.1_WP_079297059.:	CAGAAGAACATAATACAAGACTTGGGACATCCCTAGAACCTGGGCAATTTACTCCATGGG	prk	Phosphoribulokinase	WP_079297059.:	14442..15338	Carbon Fixation	COG3954
187.1_WP_079297059.:	CUST_2905_PI442190807	HOT208_60m_813L03 187.1_WP_079297059.:	TAACGCAGAAAGAGGTTACTCAGCGGAAACCATTGTGGATACGATATTGAGAAGAATGCC	prk	Phosphoribulokinase	WP_079297059.:	14442..15338	Carbon Fixation	COG3954
187.1_WP_079297059.:	CUST_2906_PI442190807	HOT208_60m_813L03 187.1_WP_079297059.:	TGTCAAGTCCAACAAGTATCGTTGTAAATGGTGGGAAAATGGGTTTCGCAATGGAACTAA	prk	Phosphoribulokinase	WP_079297059.:	14442..15338	Carbon Fixation	COG3954
188.1_WP_079297081.:	CUST_2972_PI442190807	HOT208_60m_813L03 188.1_WP_079297081.:	ACCTGGAGTTGAAGCTGGAAAGAAAGCAGCCGCAAATATTATTGAGTATCAGCAAAAAGT	pgi	Glucose-6-phosphate isomerase	WP_079297081.:	1214..2797	Glycolysis/Gluconeogenesis	COG0166
188.1_WP_079297081.:	CUST_2973_PI442190807	HOT208_60m_813L03 188.1_WP_079297081.:	TCCGTGAAATGTGTTTCGGAAATGATGATTATTTGGTTAGGGGCGATTGGTCAAATCCAA	pgi	Glucose-6-phosphate isomerase	WP_079297081.:	1214..2797	Glycolysis/Gluconeogenesis	COG0166
188.1_WP_079297081.:	CUST_2974_PI442190807	HOT208_60m_813L03 188.1_WP_079297081.:	CCGCAGCATTATTATCATCTGCATGGTATTTAACTGGAGATGGCGTTGGGAAGAGAGATA	pgi	Glucose-6-phosphate isomerase	WP_079297081.:	1214..2797	Glycolysis/Gluconeogenesis	COG0166
188.1_WP_079297081.:	CUST_2975_PI442190807	HOT208_60m_813L03 188.1_WP_079297081.:	TGTTTTTCACTAGGTGCCTTAATCGCATTATTTGAGAGAGCTGTATCCTTCTACGCTGAA	pgi	Glucose-6-phosphate isomerase	WP_079297081.:	1214..2797	Glycolysis/Gluconeogenesis	COG0166
188.1_WP_079297081.:	CUST_2976_PI442190807	HOT208_60m_813L03 188.1_WP_079297081.:	TGGTAACAAAGGATCTACAGATCAACATGCTTATGTTCAGCAACTGAGAGATGGGATTGA	pgi	Glucose-6-phosphate isomerase	WP_079297081.:	1214..2797	Glycolysis/Gluconeogenesis	COG0166
188.1_WP_079297081.:	CUST_2977_PI442190807	HOT208_60m_813L03 188.1_WP_079297081.:	AAGGAACAAGGTCCGCACTCTCTAGTGAGAACAGACAAAGTATAACTATCACATTAGAAA	pgi	Glucose-6-phosphate isomerase	WP_079297081.:	1214..2797	Glycolysis/Gluconeogenesis	COG0166
189.1_592	CUST_3112_PI442190807	HOT208_60m_813L03 189.1_592	GTGTGTTGGCCATAATCCTCGGTGGAGGAAAAGGTTCTAGACTTTACCCTCTAACAAAAA	glgC	Glucose-1-phosphate adenylyltransferase		24535..25319	Glycogen Metabolism	COG0448
189.1_592	CUST_3113_PI442190807	HOT208_60m_813L03 189.1_592	CATCATAGAGATAATGGTGCTGATTTAACCGTTGCAGCTTTGCCTGTTGATGAAGCTCAA	glgC	Glucose-1-phosphate adenylyltransferase		24535..25319	Glycogen Metabolism	COG0448
189.1_592	CUST_3114_PI442190807	HOT208_60m_813L03 189.1_592	GGCTAAACCTGCCGTGCCATTGGCAGGTAAGTATCGTTTGATAGATATTCCGATTAGTAA	glgC	Glucose-1-phosphate adenylyltransferase		24535..25319	Glycogen Metabolism	COG0448
189.1_592	CUST_3115_PI442190807	HOT208_60m_813L03 189.1_592	CACAACAGACTCCTGATAGTCCGAAGTGGTTTGAAGGTACTGCTGATGCTGTAAGAAAAT	glgC	Glucose-1-phosphate adenylyltransferase		24535..25319	Glycogen Metabolism	COG0448
189.1_592	CUST_3116_PI442190807	HOT208_60m_813L03 189.1_592	TAGGAAGAACCTATAATTTAAATGGCCCTTTCGGCCAAGGATTTGTTGAGGTTTTAGCGG	glgC	Glucose-1-phosphate adenylyltransferase		24535..25319	Glycogen Metabolism	COG0448
189.1_592	CUST_3117_PI442190807	HOT208_60m_813L03 189.1_592	TGGGATGTTGATGAATATTTAATATTGTCAGGTGATCAACTATACAGAATGGACTACAGT	glgC	Glucose-1-phosphate adenylyltransferase		24535..25319	Glycogen Metabolism	COG0448
190.1_WP_079297167.:	CUST_3276_PI442190807	HOT208_60m_813L03 190.1_WP_079297167.:	TTCTGACCCAACCCTATTTACTCGAAGTGATGAAGTAGAGGCAGCCTGGAAACTCTATAC	zwf	glucose-6-phosphate dehydrogenase zwf	WP_079297167.:	28124..29647	Pentose Phosphate	COG0364
190.1_WP_079297167.:	CUST_3277_PI442190807	HOT208_60m_813L03 190.1_WP_079297167.:	GCTGACGAAAATGAACCATGGAAATGTTGCATAAGAGGTCAATATGGGGAAGGAGGAAAT	zwf	glucose-6-phosphate dehydrogenase zwf	WP_079297167.:	28124..29647	Pentose Phosphate	COG0364
190.1_WP_079297167.:	CUST_3278_PI442190807	HOT208_60m_813L03 190.1_WP_079297167.:	GCTTCAAAATCATATGACTCAAATGCTTGCTGTTACTGCTATGGAACCTCCCGGAAAGTT	zwf	glucose-6-phosphate dehydrogenase zwf	WP_079297167.:	28124..29647	Pentose Phosphate	COG0364
190.1_WP_079297167.:	CUST_3279_PI442190807	HOT208_60m_813L03 190.1_WP_079297167.:	AAACTTTTATTGCGACAAAAGTTTTCGTTGATAACTGGCGTTGGCAAGGGGTTCCTTTTT	zwf	glucose-6-phosphate dehydrogenase zwf	WP_079297167.:	28124..29647	Pentose Phosphate	COG0364
190.1_WP_079297167.:	CUST_3280_PI442190807	HOT208_60m_813L03 190.1_WP_079297167.:	AGGTGCCACTTTCAAATTTGAGGTAAAATCTCCTGGTTCTGGAATGAAATCAAGACCTGT	zwf	glucose-6-phosphate dehydrogenase zwf	WP_079297167.:	28124..29647	Pentose Phosphate	COG0364
190.1_WP_079297167.:	CUST_3281_PI442190807	HOT208_60m_813L03 190.1_WP_079297167.:	TGAATCATTTGGAGAACCCTCAGATGAAGGCTATGTAAGATTATTAGCTGATGCAATGCT	zwf	glucose-6-phosphate dehydrogenase zwf	WP_079297167.:	28124..29647	Pentose Phosphate	COG0364
190.1_WP_079297169.:	CUST_3282_PI442190807	HOT208_60m_813L03 190.1_WP_079297169.:	ATGTGAATCAGTGGAATGTATGAGACTTGAGGCCGGGGGAATGGCTAATATGGAATTAAT	opcA	Glucose-6-P dehydrogenase subunit opcA	WP_079297169.:	29650..30951	Pentose Phosphate	COG3429
190.1_WP_079297169.:	CUST_3283_PI442190807	HOT208_60m_813L03 190.1_WP_079297169.:	GGCAATGATTTTTGATCCGCCCTCAAGGAGGCCAATTTTAGATCACATTACCGATATTGA	opcA	Glucose-6-P dehydrogenase subunit opcA	WP_079297169.:	29650..30951	Pentose Phosphate	COG3429
190.1_WP_079297169.:	CUST_3284_PI442190807	HOT208_60m_813L03 190.1_WP_079297169.:	TCAATTGATGAATTACCTTTATGGTTGTGGTGGAATGGAAGCTTGGATGAATCGCCTGAA	opcA	Glucose-6-P dehydrogenase subunit opcA	WP_079297169.:	29650..30951	Pentose Phosphate	COG3429
190.1_WP_079297169.:	CUST_3285_PI442190807	HOT208_60m_813L03 190.1_WP_079297169.:	TCCCTTATCTTTGGGAAATCCAAGTATTCATTTAGGACAAGTTATTGGATTGAGGCTGAT	opcA	Glucose-6-P dehydrogenase subunit opcA	WP_079297169.:	29650..30951	Pentose Phosphate	COG3429
190.1_WP_079297169.:	CUST_3286_PI442190807	HOT208_60m_813L03 190.1_WP_079297169.:	CATTGACATTGCGGGAGATCATATTATTCAAGCTTTGTTTTTGATTTCATGGATTAGCGA	opcA	Glucose-6-P dehydrogenase subunit opcA	WP_079297169.:	29650..30951	Pentose Phosphate	COG3429
190.1_WP_079297169.:	CUST_3287_PI442190807	HOT208_60m_813L03 190.1_WP_079297169.:	TGGTCTAAGGTTAATAATTGATACTGCTCTTGGATCGCCTCAAAGATGCTTAAAAGTTTT	opcA	Glucose-6-P dehydrogenase subunit opcA	WP_079297169.:	29650..30951	Pentose Phosphate	COG3429
195.1_WP_079297292.:	CUST_3656_PI442190807	HOT208_60m_813L03 195.1_WP_079297292.:	TGGTTTAGATCTACTTTTTGATGAATTAACTGAAACTGAATATAGTGGAGATACAT	kaiB	Circadian clock protein KaiB	WP_079297292.:	7536..7853	Circadian Rhythm	COG4251
195.1_WP_079297292.:	CUST_3657_PI442190807	HOT208_60m_813L03 195.1_WP_079297292.:	CCACCACCAGTGAGAAAAATAATAGGTGATCTCTCAGATAGAGAGAAAGTTTTAATTGGT	kaiB	Circadian clock protein KaiB	WP_079297292.:	7536..7853	Circadian Rhythm	COG4251
195.1_WP_079297292.:	CUST_3658_PI442190807	HOT208_60m_813L03 195.1_WP_079297292.:	GGTTATTGATGTTCTAAAGCAGCCACAACTTGCAGAAGAGGATAAGATTTTAGCCACCCC	kaiB	Circadian clock protein KaiB	WP_079297292.:	7536..7853	Circadian Rhythm	COG4251
195.1_WP_079297292.:	CUST_3659_PI442190807	HOT208_60m_813L03 195.1_WP_079297292.:	TGAGAGCTTTAAATACTTTAAGGGAAATTTTAGAAAATGAATTTAAAGGAGTTTATGCTT	kaiB	Circadian clock protein KaiB	WP_079297292.:	7536..7853	Circadian Rhythm	COG4251
195.1_WP_079297292.:	CUST_3660_PI442190807	HOT208_60m_813L03 195.1_WP_079297292.:	TGGCAGCAAGAAAAACATACATTTTAAAGCTCTATGTTGCTGGAAATACTCCTAATTCAA	kaiB	Circadian clock protein KaiB	WP_079297292.:	7536..7853	Circadian Rhythm	COG4251
195.1_WP_079297294.:	CUST_3661_PI442190807	HOT208_60m_813L03 195.1_WP_079297294.:	ATACTTTTTGCATATGAAGAATCTAGGGCTCAATTGCTTAGGAATGCGACTAGCTGGGGA	kaiC	circadian clock protein KaiC	WP_079297294.:	7922..9451	Circadian Rhythm	COG0467
195.1_WP_079297294.:	CUST_3662_PI442190807	HOT208_60m_813L03 195.1_WP_079297294.:	AAATAAAAGGTGAGATGGCACGAGCTTTAAATGTTTTTAAAATGCGGGGATCATGGCACG	kaiC	circadian clock protein KaiC	WP_079297294.:	7922..9451	Circadian Rhythm	COG0467
195.1_WP_079297294.:	CUST_3663_PI442190807	HOT208_60m_813L03 195.1_WP_079297294.:	ATTTTCAAGATTCCATCATTCTTGCCACTGGAGCTACTGGTACGGGTAAAACAATGCTTG	kaiC	circadian clock protein KaiC	WP_079297294.:	7922..9451	Circadian Rhythm	COG0467
195.1_WP_079297294.:	CUST_3664_PI442190807	HOT208_60m_813L03 195.1_WP_079297294.:	CTAATTTTGAGCAAATTTTTAGTGGCGCCCCTCACAGAGTTGTGCCTGATCAAAATGTTC	kaiC	circadian clock protein KaiC	WP_079297294.:	7922..9451	Circadian Rhythm	COG0467
195.1_WP_079297294.:	CUST_3665_PI442190807	HOT208_60m_813L03 195.1_WP_079297294.:	GCATTAGCCAGAGGTGTAAGTTTGAATGCATTTAGACAGTTTGTAATTGCTGTTACCGGT	kaiC	circadian clock protein KaiC	WP_079297294.:	7922..9451	Circadian Rhythm	COG0467
195.1_WP_079297294.:	CUST_3666_PI442190807	HOT208_60m_813L03 195.1_WP_079297294.:	TCCTCAAATATTAGGATTAGCTCTGGAGTTAAAGATCTTGATGATATGTGTGGTGGAGGA	kaiC	circadian clock protein KaiC	WP_079297294.:	7922..9451	Circadian Rhythm	COG0467
195.1_WP_079297319.:	CUST_3773_PI442190807	HOT208_60m_813L03 195.1_WP_079297319.:	CTGATGCAATAGACGAAGACCCAGAGAAACCAGCAGGATTATATACTGCTTGGATGGTAC	glcH	GPH family sugar transporter	WP_079297319.:	27247..28593	GlcH sugar transporter	COG2211
195.1_WP_079297319.:	CUST_3774_PI442190807	HOT208_60m_813L03 195.1_WP_079297319.:	TAATCTGTCTTATTGGTATTGGAGCTTCAACCGCTTTTCTTATCCCTTGGTCACTACTGC	glcH	GPH family sugar transporter	WP_079297319.:	27247..28593	GlcH sugar transporter	COG2211
195.1_WP_079297319.:	CUST_3775_PI442190807	HOT208_60m_813L03 195.1_WP_079297319.:	ACAGGATTTCAGCGTTAAACTATGGAGCGATTATGTGGATTATTTCATGTACGGCAGCTT	glcH	GPH family sugar transporter	WP_079297319.:	27247..28593	GlcH sugar transporter	COG2211
195.1_WP_079297319.:	CUST_3776_PI442190807	HOT208_60m_813L03 195.1_WP_079297319.:	TACTGAACGTACCAACATATATTGCGAAGTGGATCCCGATACCTTTCCAAATTAGCGCTT	glcH	GPH family sugar transporter	WP_079297319.:	27247..28593	GlcH sugar transporter	COG2211
195.1_WP_079297319.:	CUST_3777_PI442190807	HOT208_60m_813L03 195.1_WP_079297319.:	TTCTTCTCTGGTGTGCCTTACAATTAATGCAAACAGTAGCGTTAATCTATGTCGAGGATG	glcH	GPH family sugar transporter	WP_079297319.:	27247..28593	GlcH sugar transporter	COG2211
195.1_WP_079297319.:	CUST_3778_PI442190807	HOT208_60m_813L03 195.1_WP_079297319.:	GCAACATTAATTTCTTGTTGGGGATTAGCTCCATTCGCAAAAAAAGCAAGAAGGCCTTCA	glcH	GPH family sugar transporter	WP_079297319.:	27247..28593	GlcH sugar transporter	COG2211
195.1_WP_079297337.:	CUST_3852_PI442190807	HOT208_60m_813L03 195.1_WP_079297337.:	TATTGTTGGTGCAGTGGTTGATGAGGCAATGGAAGGGGAAATACAAGTAACTGTTATTGC	ftsZ	cell division protein FtsZ	WP_079297337.:	40089..41204	Cell Division	COG0206
195.1_WP_079297337.:	CUST_3853_PI442190807	HOT208_60m_813L03 195.1_WP_079297337.:	TCCCCTTTTAGAGGCAGCTAGAATTGATGGAGCTAAAGGCTGCGTCATTAATATTACTGG	ftsZ	cell division protein FtsZ	WP_079297337.:	40089..41204	Cell Division	COG0206
195.1_WP_079297337.:	CUST_3854_PI442190807	HOT208_60m_813L03 195.1_WP_079297337.:	AGTAACCAAGCCATTTTCTTTTGAAGGAAAAAGAAGAATGCGTCAAGCAGAGGAAGGGAT	ftsZ	cell division protein FtsZ	WP_079297337.:	40089..41204	Cell Division	COG0206
195.1_WP_079297337.:	CUST_3855_PI442190807	HOT208_60m_813L03 195.1_WP_079297337.:	TCAAGAAGCGTTTAGGAATGCTGATGATGTTCTAAGGATGGGTGTCAAAGGCATTAGTGA	ftsZ	cell division protein FtsZ	WP_079297337.:	40089..41204	Cell Division	COG0206
195.1_WP_079297337.:	CUST_3856_PI442190807	HOT208_60m_813L03 195.1_WP_079297337.:	CTTGCCCAGGATTAGTTAATGTTGATTTTGCAGACGTAAGATCGGTTATGACAGAAGCGG	ftsZ	cell division protein FtsZ	WP_079297337.:	40089..41204	Cell Division	COG0206
195.1_WP_079297337.:	CUST_3857_PI442190807	HOT208_60m_813L03 195.1_WP_079297337.:	GAATAGGTATAGGTTCAGGCAGATCGAGAGCTATAGAGGCAGCACAGGCAGCAATGAATA	ftsZ	cell division protein FtsZ	WP_079297337.:	40089..41204	Cell Division	COG0206
201.1_WP_079297472.:	CUST_4309_PI442190807	HOT208_60m_813L03 201.1_WP_079297472.:	CGGAGGGAGAGAGGGGAGGCATAATATTTCCAGGAGGAAGATATGAGGCTCTTAAAAATA	rbsK	Sugar kinase, ribokinase family	WP_079297472.:	25982..26851	Pentose Phosphate	COG0524
201.1_WP_079297472.:	CUST_4310_PI442190807	HOT208_60m_813L03 201.1_WP_079297472.:	AAGGTTGATTCTTATGGATGTGGTGATTCTTTTGCTGCTGGGATTCTTTATGGAATGGCA	rbsK	Sugar kinase, ribokinase family	WP_079297472.:	25982..26851	Pentose Phosphate	COG0524
201.1_WP_079297472.:	CUST_4311_PI442190807	HOT208_60m_813L03 201.1_WP_079297472.:	ATTACCAAAACCCGGAGTTATTTCTCATTCTGAAAAGTCCCTTGAGTATCCAGCTGGTGG	rbsK	Sugar kinase, ribokinase family	WP_079297472.:	25982..26851	Pentose Phosphate	COG0524
201.1_WP_079297472.:	CUST_4312_PI442190807	HOT208_60m_813L03 201.1_WP_079297472.:	CTCTCGAATATGGGAATTAAGTTGCATGTAGCTTGGCGTGATAAACCAACTAGAAGAGGA	rbsK	Sugar kinase, ribokinase family	WP_079297472.:	25982..26851	Pentose Phosphate	COG0524
201.1_WP_079297472.:	CUST_4313_PI442190807	HOT208_60m_813L03 201.1_WP_079297472.:	AGGCAGTAATATTGATCCCGGGGAAGCTTTTTCTTTTTCTGATTTATCGTTAAAACCCAA	rbsK	Sugar kinase, ribokinase family	WP_079297472.:	25982..26851	Pentose Phosphate	COG0524
201.1_WP_079297472.:	CUST_4314_PI442190807	HOT208_60m_813L03 201.1_WP_079297472.:	TGGACGGAGTTTTTATTACCGCATCTGATTCAGAGATTTTTAAAATGGCTAGATCAGCTT	rbsK	Sugar kinase, ribokinase family	WP_079297472.:	25982..26851	Pentose Phosphate	COG0524
022.1_WP_079295950.:	CUST_4761_PI442190807	HOT208_60m_813L03 022.1_WP_079295950.:	CAACTTGGTGCTGAGCACAGTCAAAGACTTGAAGCATATTTTCATGCAGTTCCTGGAATA	pdhB	Pyruvate dehydrogenase E1 component beta subunit	WP_079295950.:	238..1221	Glycolysis/Krebs cycle	COG0022
022.1_WP_079295950.:	CUST_4762_PI442190807	HOT208_60m_813L03 022.1_WP_079295950.:	GTGCAGAATTAATTGCCTTGATAACAGAAGAGTGTTTTGACGATCTTGATGCCCGACCAA	pdhB	Pyruvate dehydrogenase E1 component beta subunit	WP_079295950.:	238..1221	Glycolysis/Krebs cycle	COG0022
022.1_WP_079295950.:	CUST_4763_PI442190807	HOT208_60m_813L03 022.1_WP_079295950.:	ATTTGTCTGAAGAATTACCTGAGGGAGATTATACGTGCGCTTTAGATCAGGCTGACGTTG	pdhB	Pyruvate dehydrogenase E1 component beta subunit	WP_079295950.:	238..1221	Glycolysis/Krebs cycle	COG0022
022.1_WP_079295950.:	CUST_4764_PI442190807	HOT208_60m_813L03 022.1_WP_079295950.:	TATACAAGTGGCGGAAATTATAAAATACCAGCAGTAGTTCGAGGACCTGGAGGAGTTGGT	pdhB	Pyruvate dehydrogenase E1 component beta subunit	WP_079295950.:	238..1221	Glycolysis/Krebs cycle	COG0022
022.1_WP_079295950.:	CUST_4765_PI442190807	HOT208_60m_813L03 022.1_WP_079295950.:	GCAATGACTGGCTTAAGACCAATAGTAGAAGGAATGAATATGGGTTTTTTGCTTTTAGCT	pdhB	Pyruvate dehydrogenase E1 component beta subunit	WP_079295950.:	238..1221	Glycolysis/Krebs cycle	COG0022
022.1_WP_079295950.:	CUST_4766_PI442190807	HOT208_60m_813L03 022.1_WP_079295950.:	TCAGGATATTCCAACCCCTTATAATGGAAATCTTGAGAATTTGACGATAATCCAACCACA	pdhB	Pyruvate dehydrogenase E1 component beta subunit	WP_079295950.:	238..1221	Glycolysis/Krebs cycle	COG0022
022.1_WP_079295967.:	CUST_4819_PI442190807	HOT208_60m_813L03 022.1_WP_079295967.:	TAATGGAACTGGAATGCATGTTCACCAAAGTTTATGGAAGAGTGGGCAGCCACTATTTTT	glnA	Glutamine synthetase/ glutamate--ammonialigase	WP_079295967.:	8989..10410	Nitrogen Metabolism	COG0174
022.1_WP_079295967.:	CUST_4820_PI442190807	HOT208_60m_813L03 022.1_WP_079295967.:	CGCCGGTCAACACGAACTTGGAATGAAATTCGATTCGTTAATAAACTCTGCTGATAACGT	glnA	Glutamine synthetase/ glutamate--ammonialigase	WP_079295967.:	8989..10410	Nitrogen Metabolism	COG0174
022.1_WP_079295967.:	CUST_4821_PI442190807	HOT208_60m_813L03 022.1_WP_079295967.:	GGTGAAGGAGCATATGCAAATCTATCTCAAACAGCAAGATGGTACATCGGAGGCATACTA	glnA	Glutamine synthetase/ glutamate--ammonialigase	WP_079295967.:	8989..10410	Nitrogen Metabolism	COG0174
022.1_WP_079295967.:	CUST_4822_PI442190807	HOT208_60m_813L03 022.1_WP_079295967.:	AGATCAGGTGATGCACTTGCAAATCCTTACTTAGCATTTTCTGTAATGATGCTTGCTGGT	glnA	Glutamine synthetase/ glutamate--ammonialigase	WP_079295967.:	8989..10410	Nitrogen Metabolism	COG0174
022.1_WP_079295967.:	CUST_4823_PI442190807	HOT208_60m_813L03 022.1_WP_079295967.:	TGAGGGTAATAGATCGGCTGCAGTAAGAATACCTTTAACAGGTCCAAGCCCTAAAGCTAA	glnA	Glutamine synthetase/ glutamate--ammonialigase	WP_079295967.:	8989..10410	Nitrogen Metabolism	COG0174
022.1_WP_079295967.:	CUST_4824_PI442190807	HOT208_60m_813L03 022.1_WP_079295967.:	CGTTGTCAGAAATGTTGCTAAAAAAATATGGGAAAACTGCAACGTTTATGCCAAAGCCTGT	glnA	Glutamine synthetase/ glutamate--ammonialigase	WP_079295967.:	8989..10410	Nitrogen Metabolism	COG0174
022.1_WP_079295981.:	CUST_4865_PI442190807	HOT208_60m_813L03 022.1_WP_079295981.:	AAGCAATGGCGACCTCATTACACTGCAGCTAAATGAAGGTGTTATATACATGGGTCAAAT	pykF	Pyruvate kinase	WP_079295981.:	17628..19418	Glycolysis	COG0469
022.1_WP_079295981.:	CUST_4866_PI442190807	HOT208_60m_813L03 022.1_WP_079295981.:	TGCAAATTGCACAGGAAATGGGGATCCTAAATCAAGGAGATTTAGTAGTTCAAACGGCTG	pykF	Pyruvate kinase	WP_079295981.:	17628..19418	Glycolysis	COG0469
022.1_WP_079295981.:	CUST_4867_PI442190807	HOT208_60m_813L03 022.1_WP_079295981.:	CCAACACCTATCTTGGCAACTACTACTGAAAGGAGTGTAGCGAGAAGATTGCAACTTGTT	pykF	Pyruvate kinase	WP_079295981.:	17628..19418	Glycolysis	COG0469
022.1_WP_079295981.:	CUST_4868_PI442190807	HOT208_60m_813L03 022.1_WP_079295981.:	GCTAGACAACTTGACGCGGGAGCAATAATCCCTTTAACAAAATCAGGTTCTACTGCTAGA	pykF	Pyruvate kinase	WP_079295981.:	17628..19418	Glycolysis	COG0469
022.1_WP_079295981.:	CUST_4869_PI442190807	HOT208_60m_813L03 022.1_WP_079295981.:	CCACTTAAAGCTATTGAAAGCCACTTACCAAGTACGATCCCAAATGCTATTAGTGCAGCA	pykF	Pyruvate kinase	WP_079295981.:	17628..19418	Glycolysis	COG0469
022.1_WP_079295981.:	CUST_4870_PI442190807	HOT208_60m_813L03 022.1_WP_079295981.:	CGATTATCCTGTAGAGGCAGTAGAAACGATGGCAACCATAGCAAGAAGAATTGAAAGGGA	pykF	Pyruvate kinase	WP_079295981.:	17628..19418	Glycolysis	COG0469
225.1_954	CUST_4997_PI442190807	HOT208_60m_813L03 225.1_954	AGGCAGGTTTCAAATATAATTGATCTCTGCGTAGGTATAGAAGTTGGTGTCGCAGCAACA	glmS	Glutamine--fructose-6-phosphatetransaminase(isomerizing)		<1..461	Amino Acid metabolism	COG0449
225.1_954	CUST_4998_PI442190807	HOT208_60m_813L03 225.1_954	TCCTCCATTGCTGCCAAATACATTAACTATTGGAGTCACTCAATCTGGAGAAACTGCTGA	glmS	Glutamine--fructose-6-phosphatetransaminase(isomerizing)		<1..461	Amino Acid metabolism	COG0449
225.1_954	CUST_4999_PI442190807	HOT208_60m_813L03 225.1_954	CGCAGCGATAGATATGGAAATTAAAAGACGGTCTTCGATTGAAGATAAAAATTTTCAACC	glmS	Glutamine--fructose-6-phosphatetransaminase(isomerizing)		<1..461	Amino Acid metabolism	COG0449
225.1_954	CUST_5000_PI442190807	HOT208_60m_813L03 225.1_954	GCCTCCATTAATAGAAGATCTTTTAGAGCAACATAATAAATCTTCAGAAAAGCTAGCACA	glmS	Glutamine--fructose-6-phosphatetransaminase(isomerizing)		<1..461	Amino Acid metabolism	COG0449
225.1_954	CUST_5001_PI442190807	HOT208_60m_813L03 225.1_954	TGCTCAATTACTTTCTTTTTATGGATTAGCTATAAAATTTGCGGAAATTAAAGGTAGTCA	glmS	Glutamine--fructose-6-phosphatetransaminase(isomerizing)		<1..461	Amino Acid metabolism	COG0449
225.1_954	CUST_5002_PI442190807	HOT208_60m_813L03 225.1_954	AATTAAAGGTAGTCAAAGCTTAGATGAAATAAGTAATTTAATAAA	glmS	Glutamine--fructose-6-phosphatetransaminase(isomerizing)		<1..461	Amino Acid metabolism	COG0449
023.1_WP_079296043.:	CUST_5096_PI442190807	HOT208_60m_813L03 023.1_WP_079296043.:	TGGAGTTGTAAAAGTCCCTCTTGAAGCTGCACTATCAGATGCTATTGATTTGACTGGACC		polysaccharide biosynthesis/export family protein	WP_079296043.:	9878..11179	Other sugar transporters	COG1596
023.1_WP_079296043.:	CUST_5097_PI442190807	HOT208_60m_813L03 023.1_WP_079296043.:	TCTCGAGTTGAAATCATTAGAGATGTACCTCTTGGAAAAGGTGGAGGTAAGAAGAGAGCA		polysaccharide biosynthesis/export family protein	WP_079296043.:	9878..11179	Other sugar transporters	COG1596
023.1_WP_079296043.:	CUST_5098_PI442190807	HOT208_60m_813L03 023.1_WP_079296043.:	ATCGCAAAATCAAGTAAAAATATAACTACACTCTCTGATGCGATTAGAAAAGCTGGTGGT		polysaccharide biosynthesis/export family protein	WP_079296043.:	9878..11179	Other sugar transporters	COG1596
023.1_WP_079296043.:	CUST_5099_PI442190807	HOT208_60m_813L03 023.1_WP_079296043.:	CGATAAAACCGCTGTCTGGAAAAATTGTTTTAATAAGGTATGAAAAAGACGGAACAGTT		polysaccharide biosynthesis/export family protein	WP_079296043.:	9878..11179	Other sugar transporters	COG1596
023.1_WP_079296043.:	CUST_5100_PI442190807	HOT208_60m_813L03 023.1_WP_079296043.:	AAGTACAGGAGTGATTAGAGAAATTACAGCACCATTTATAGGACTTTATACAACCAAAGA		polysaccharide biosynthesis/export family protein	WP_079296043.:	9878..11179	Other sugar transporters	COG1596
023.1_WP_079296043.:	CUST_5101_PI442190807	HOT208_60m_813L03 023.1_WP_079296043.:	TACTTTCCAAAACTTAGTAAAGCTGATCCAAGTCAAATTCCTAGATCAATTTTATCTGGT		polysaccharide biosynthesis/export family protein	WP_079296043.:	9878..11179	Other sugar transporters	COG1596
242.1_WP_079297629.:	CUST_5423_PI442190807	HOT208_60m_813L03 242.1_WP_079297629.:	GACGCAGGAAGGTACATTACAGGGCAAACAATCAGTATTGACGGGGGTATGAGTATTTAA	gdh	3-oxoacyl-acyl-carrier-protein reductase	WP_079297629.:	18462..19211	Entner-Dudoroff	COG1028
242.1_WP_079297629.:	CUST_5424_PI442190807	HOT208_60m_813L03 242.1_WP_079297629.:	GGCAAGCAAATTATTCTGCTGCAAAAGCTGGAGTTATTGGATTTACCAAAACTTGCGCTA	gdh	3-oxoacyl-acyl-carrier-protein reductase	WP_079297629.:	18462..19211	Entner-Dudoroff	COG1028
242.1_WP_079297629.:	CUST_5425_PI442190807	HOT208_60m_813L03 242.1_WP_079297629.:	ATGCTGGGATTACGAGAGATGGACTATTGATGAGAATGAAATCGGAACAATGGGATGACG	gdh	3-oxoacyl-acyl-carrier-protein reductase	WP_079297629.:	18462..19211	Entner-Dudoroff	COG1028
242.1_WP_079297629.:	CUST_5426_PI442190807	HOT208_60m_813L03 242.1_WP_079297629.:	AGCTAGCAGAGGAATTGGTAAAGAAATTGCTTTAGAACTAAGCCGCTTAGGAGCAGAAGT	gdh	3-oxoacyl-acyl-carrier-protein reductase	WP_079297629.:	18462..19211	Entner-Dudoroff	COG1028
242.1_WP_079297629.:	CUST_5427_PI442190807	HOT208_60m_813L03 242.1_WP_079297629.:	TGCTTCAAGAGGTATAAATGTAAATGCAATAGCTCCAGGTTTCATAGAAACAGAGATGAC	gdh	3-oxoacyl-acyl-carrier-protein reductase	WP_079297629.:	18462..19211	Entner-Dudoroff	COG1028
242.1_WP_079297629.:	CUST_5428_PI442190807	HOT208_60m_813L03 242.1_WP_079297629.:	TTTCAAAAGAGGATTCTGTCAGTTTAGCTTTTGAAGAAATCATAAAAATTAATGGCACCA	gdh	3-oxoacyl-acyl-carrier-protein reductase	WP_079297629.:	18462..19211	Entner-Dudoroff	COG1028
242.1_WP_079297642.:	CUST_5466_PI442190807	HOT208_60m_813L03 242.1_WP_079297642.:	ATGTCATTCATGCTTTTTGGGTGCCAGAGTTCAGAATTAAACAGGATATCATTCCTGGGC	ctaC/coxB	Cytochrome c oxidase subunit II	WP_079297642.:	25826..26629	Respiration	COG1622
242.1_WP_079297642.:	CUST_5467_PI442190807	HOT208_60m_813L03 242.1_WP_079297642.:	GTGGCCCATATCATGGAGGGATGAGAGCCTCCATAATTGTTGAAGAAGAATCTGATTACA	ctaC/coxB	Cytochrome c oxidase subunit II	WP_079297642.:	25826..26629	Respiration	COG1622
242.1_WP_079297642.:	CUST_5468_PI442190807	HOT208_60m_813L03 242.1_WP_079297642.:	ACGATCGAATGGGAGGGATGAAAGAACTAAATCATAACCACGAAATGATGAGTTCGAACA	ctaC/coxB	Cytochrome c oxidase subunit II	WP_079297642.:	25826..26629	Respiration	COG1622
242.1_WP_079297642.:	CUST_5469_PI442190807	HOT208_60m_813L03 242.1_WP_079297642.:	TCTTTTTGGATTGGTTTTAATGTAAATTTGCTCCCAGCGGAAGCAAGTATTAATGCACCA	ctaC/coxB	Cytochrome c oxidase subunit II	WP_079297642.:	25826..26629	Respiration	COG1622
242.1_WP_079297642.:	CUST_5470_PI442190807	HOT208_60m_813L03 242.1_WP_079297642.:	ATCCCAAGGGCAATTTTATATCAGGAGAACTACACGTTCCAGTTGATCAAAAAGTATCAA	ctaC/coxB	Cytochrome c oxidase subunit II	WP_079297642.:	25826..26629	Respiration	COG1622
242.1_WP_079297642.:	CUST_5471_PI442190807	HOT208_60m_813L03 242.1_WP_079297642.:	ACCTACTATTCTAAATTTCACTCCTACAAAAGTAGGAAAATATCCGATAATTTGCGCAGA	ctaC/coxB	Cytochrome c oxidase subunit II	WP_079297642.:	25826..26629	Respiration	COG1622

242.1_WP_079297644.:	CUST_5472_PI442190807	HOT208_60m_813L03	242.1_WP_079297644.:	GCTTTGCTCCTCAACATTGGCTTGGTTTAAATGGAATGCCAAGAAGAGTTGCAGAATATG	cyoB/coxA	Cytochrome c oxidase subunit I	WP_079297644.:	26626..28251	Respiration	COG0843
242.1_WP_079297644.:	CUST_5473_PI442190807	HOT208_60m_813L03	242.1_WP_079297644.:	GAGATAACCCTTGGAATGCTCTTACACCTGAATGGTTAACATCTTCTCCACCTCCAGTTG	cyoB/coxA	Cytochrome c oxidase subunit I	WP_079297644.:	26626..28251	Respiration	COG0843
242.1_WP_079297644.:	CUST_5474_PI442190807	HOT208_60m_813L03	242.1_WP_079297644.:	CCCCTTGGATGAGATTATTCTTTACTATCGCCACAGCATTTATTGCTGTTCCGACAGGTA	cyoB/coxA	Cytochrome c oxidase subunit I	WP_079297644.:	26626..28251	Respiration	COG0843
242.1_WP_079297644.:	CUST_5475_PI442190807	HOT208_60m_813L03	242.1_WP_079297644.:	ATGGGAATTGTAGTTTTAGGTTTAGTTGTTTGGGCGCATCACATGTTTACGAGTGGAACG	cyoB/coxA	Cytochrome c oxidase subunit I	WP_079297644.:	26626..28251	Respiration	COG0843
242.1_WP_079297644.:	CUST_5476_PI442190807	HOT208_60m_813L03	242.1_WP_079297644.:	TGATCCTCAATTCCAGTTCGTTAATCAAATTAGTAGCCTTGGAGCCCTTTTGATGGCT	cyoB/coxA	Cytochrome c oxidase subunit I	WP_079297644.:	26626..28251	Respiration	COG0843
242.1_WP_079297644.:	CUST_5477_PI442190807	HOT208_60m_813L03	242.1_WP_079297644.:	GGTTGCGACATTATGGGGAGGTAAAATTTCCATCAATAGTGCAATTTTATTCTCTTGCGG	cyoB/coxA	Cytochrome c oxidase subunit I	WP_079297644.:	26626..28251	Respiration	COG0843
242.1_WP_079297646.:	CUST_5478_PI442190807	HOT208_60m_813L03	242.1_WP_079297646.:	TTGGTCTTATAACTTTCCTAATTGCGGACGGAATGACTTTTGCAGGATTCTTTGCTGCTT	cyoC	heme-copper oxidase subunit III	WP_079297646.:	28258..28860	Respiration	COG1845
242.1_WP_079297646.:	CUST_5479_PI442190807	HOT208_60m_813L03	242.1_WP_079297646.:	TGGCGGAAGATTAACTAGTCAAAATATGTTCCCATTGGAAGCTGTTGAATTGTACTGGCA	cyoC	heme-copper oxidase subunit III	WP_079297646.:	28258..28860	Respiration	COG1845
242.1_WP_079297646.:	CUST_5480_PI442190807	HOT208_60m_813L03	242.1_WP_079297646.:	AGTGCAACTTTCCATAAAGCAGGCAAAGCACTTTTAAAAGATAAAAACTCAGATTCCCAA	cyoC	heme-copper oxidase subunit III	WP_079297646.:	28258..28860	Respiration	COG1845
242.1_WP_079297646.:	CUST_5481_PI442190807	HOT208_60m_813L03	242.1_WP_079297646.:	TTTGCAAGTACTTTTTTTGCTCTTACAGGTTTTCATGGATTACATGTCACTTTAGGAACT	cyoC	heme-copper oxidase subunit III	WP_079297646.:	28258..28860	Respiration	COG1845
242.1_WP_079297646.:	CUST_5482_PI442190807	HOT208_60m_813L03	242.1_WP_079297646.:	AGCAGTAAATCCATTACCAGATGGTGCTATTTATGAATTAGAACTACCAATACCTACTCT	cyoC	heme-copper oxidase subunit III	WP_079297646.:	28258..28860	Respiration	COG1845
242.1_WP_079297646.:	CUST_5483_PI442190807	HOT208_60m_813L03	242.1_WP_079297646.:	GACAACACTAGACAGCTCAAAAGAAATTCAAAAAAATAATTCTGAAGTTAATGAAACACA	cyoC	heme-copper oxidase subunit III	WP_079297646.:	28258..28860	Respiration	COG1845
025.1_WP_079296061.:	CUST_5692_PI442190807	HOT208_60m_813L03	025.1_WP_079296061.:	TTCCATGGGTATTTGGCTGGACACAAAGTAGATTTCTTTTGCCAAGTTGGTTTGGAGTAG	ppc	Phosphoenolpyruvate carboxylase	WP_079296061.:	2460..5429	Krebs cycle and related genes	COG2352
025.1_WP_079296061.:	CUST_5693_PI442190807	HOT208_60m_813L03	025.1_WP_079296061.:	ATTAGGGTTTTTGCAGGTTTCACTTCTAAGAAGACTAAGAGACCAGTCAAGACAACCCCC	ppc	Phosphoenolpyruvate carboxylase	WP_079296061.:	2460..5429	Krebs cycle and related genes	COG2352
025.1_WP_079296061.:	CUST_5694_PI442190807	HOT208_60m_813L03	025.1_WP_079296061.:	TTGAGGGGAGCACTTTTAACTATTAATGGAATAGCAGCTGGCATGAGAAATACAGGTTGA	ppc	Phosphoenolpyruvate carboxylase	WP_079296061.:	2460..5429	Krebs cycle and related genes	COG2352
025.1_WP_079296061.:	CUST_5695_PI442190807	HOT208_60m_813L03	025.1_WP_079296061.:	AGAGTTCTGCATCAAAGATGGCCATTTTTTAGGATGCTTATATCTAAGGTGGAGATGACA	ppc	Phosphoenolpyruvate carboxylase	WP_079296061.:	2460..5429	Krebs cycle and related genes	COG2352
258.1_1120	CUST_5824_PI442190807	HOT208_60m_813L03	258.1_1120	CTGATCTGGAAGGATCTTATGCCTTAGCCGTATTATGGGCTGATGCTCCAACTTCATTAG	glmS	Glutamine--fructose-6-phosphatetransaminase(isomerizing)		3564..4321	Amino Acid metabolism	COG0449
258.1_1120	CUST_5825_PI442190807	HOT208_60m_813L03	258.1_1120	TAGGTCATACTAGATGGGCAACTCATGGAAAACCCGAGGTTAAAAATGCTCATCCTCATG	glmS	Glutamine--fructose-6-phosphatetransaminase(isomerizing)		3564..4321	Amino Acid metabolism	COG0449
258.1_1120	CUST_5826_PI442190807	HOT208_60m_813L03	258.1_1120	CCCTTGATTATTGGTTTGGGCGAGGGAGAATTTATTTGTGCAAGTGATACTCCAGCTATT	glmS	Glutamine--fructose-6-phosphatetransaminase(isomerizing)		3564..4321	Amino Acid metabolism	COG0449
258.1_1120	CUST_5827_PI442190807	HOT208_60m_813L03	258.1_1120	TGCCTATGGAGGATGAAGAAATTGCTTTATTGACTCCTCTCGGGATAGAAATATATGACA	glmS	Glutamine--fructose-6-phosphatetransaminase(isomerizing)		3564..4321	Amino Acid metabolism	COG0449
258.1_1120	CUST_5828_PI442190807	HOT208_60m_813L03	258.1_1120	TCGATGATTCAGGAACTATAGCGGTTGTTCAAAATGGCATTATTGAAAATTTCCAAGACT	glmS	Glutamine--fructose-6-phosphatetransaminase(isomerizing)		3564..4321	Amino Acid metabolism	COG0449
258.1_1120	CUST_5829_PI442190807	HOT208_60m_813L03	258.1_1120	TGTGTGGAATAGTTGCTGTAACAGGTTATAAAAAAGCTTTACCATTATTGATTAATGGCT	glmS	Glutamine--fructose-6-phosphatetransaminase(isomerizing)		3564..4321	Amino Acid metabolism	COG0449
030.1_WP_079296095.:	CUST_6385_PI442190807	HOT208_60m_813L03	030.1_WP_079296095.:	GCCGTGTTGGAGGTGCTGCTCAGACAAAAGCAATTAAGAAAATTGCAGGAACTTTAAAAT	atpA	ATP synthase alpha subunit	WP_079296095.:	4410..5787	Energy Metabolism	COG0056
030.1_WP_079296095.:	CUST_6386_PI442190807	HOT208_60m_813L03	030.1_WP_079296095.:	ACAAGTTGCAGTAGTTTACGCAGGAGTTAAAGGTCTTATTGATGAGGTGCCTGTTGAAGA	atpA	ATP synthase alpha subunit	WP_079296095.:	4410..5787	Energy Metabolism	COG0056
030.1_WP_079296095.:	CUST_6387_PI442190807	HOT208_60m_813L03	030.1_WP_079296095.:	TGAAACTCAGGCAGGGGACGTTTCGGCTTACATCCCAACTAATGTTATTTCAATTACAGA	atpA	ATP synthase alpha subunit	WP_079296095.:	4410..5787	Energy Metabolism	COG0056
030.1_WP_079296095.:	CUST_6388_PI442190807	HOT208_60m_813L03	030.1_WP_079296095.:	GATTACTAGAAAGAGCAGCAAAACTTTCTGATGCTATGGGAGGAGGCTCTATGACAGCTC	atpA	ATP synthase alpha subunit	WP_079296095.:	4410..5787	Energy Metabolism	COG0056
030.1_WP_079296095.:	CUST_6389_PI442190807	HOT208_60m_813L03	030.1_WP_079296095.:	ACTCGCACAGTTTGATGAACTAGCTGCTTTTTCTCAATTTGCATCTGATCTTGATGAAGC	atpA	ATP synthase alpha subunit	WP_079296095.:	4410..5787	Energy Metabolism	COG0056
030.1_WP_079296095.:	CUST_6390_PI442190807	HOT208_60m_813L03	030.1_WP_079296095.:	AGGTAAAAGACTAAGAGAGTTATTAAAGCAACCTCAATTCTCCCCGCTTAACCTTGCAGA	atpA	ATP synthase alpha subunit	WP_079296095.:	4410..5787	Energy Metabolism	COG0056
314.1_1288	CUST_6638_PI442190807	HOT208_60m_813L03	314.1_1288	CCTCATAAATCTTTAGGATATAAAGCAGTTGTAATTGAAGATAAAGCTATCAAAATTAA	eda	2-dehydro-3-deoxyphosphogluconate aldolase		<14..395	Entner-Dudoroff	COG0800
314.1_1288	CUST_6639_PI442190807	HOT208_60m_813L03	314.1_1288	AGATAGTTCGATAAATATACTCAACTATAAAAATATTGATTTCATTGCTGCTTGAGGACT	eda	2-dehydro-3-deoxyphosphogluconate aldolase		<14..395	Entner-Dudoroff	COG0800
314.1_1288	CUST_6640_PI442190807	HOT208_60m_813L03	314.1_1288	ATTCCTGGAATTAATAATTTAAAAGATCTTAAGGAAGCGATAGATTTAAATTCCAACATT	eda	2-dehydro-3-deoxyphosphogluconate aldolase		<14..395	Entner-Dudoroff	COG0800
314.1_1288	CUST_6641_PI442190807	HOT208_60m_813L03	314.1_1288	TGGATTAAATATTCGATGATGAAATTTTGGGATAAGGATCTTTTCAATTATGCGTAGTCA	eda	2-dehydro-3-deoxyphosphogluconate aldolase		<14..395	Entner-Dudoroff	COG0800
314.1_1288	CUST_6642_PI442190807	HOT208_60m_813L03	314.1_1288	TCCAAGAAATTATTTAGGCTCTACCTCCATAGTTAATAAGCAATCAATAGAAGATTCTTT	eda	2-dehydro-3-deoxyphosphogluconate aldolase		<14..395	Entner-Dudoroff	COG0800
036.1_1336	CUST_6889_PI442190807	HOT208_60m_813L03	036.1_1336	TGATGAGGGAGCAACTCTTTATATTCACGACCCCAAGGTATCCCCAAATCAAATTGCTAA	ugd	UDP-glucose 6-dehydrogenase/nucleotide sugar dehydrogenase		373..1789	Amino sugar and nucleotide sugar r	COG1004
036.1_1336	CUST_6890_PI442190807	HOT208_60m_813L03	036.1_1336	AGCTGCATTATGTGAAGCTACTGGTGCAGATGTCAGAGAAGTATCTAGAGCGATAGGATC	ugd	UDP-glucose 6-dehydrogenase/nucleotide sugar dehydrogenase		373..1789	Amino sugar and nucleotide sugar r	COG1004
036.1_1336	CUST_6891_PI442190807	HOT208_60m_813L03	036.1_1336	GACTTGAGGAGGTAGCTGACTTTTGGGGAAGCGTCGTGAATTTAAATAATTGGCATCAAC	ugd	UDP-glucose 6-dehydrogenase/nucleotide sugar dehydrogenase		373..1789	Amino sugar and nucleotide sugar r	COG1004
036.1_1336	CUST_6892_PI442190807	HOT208_60m_813L03	036.1_1336	TGATAATCGGATTGGATCTAAATTCTTAAATTCTGGCCCAGGATTTGGAGGGAGTTGCTT	ugd	UDP-glucose 6-dehydrogenase/nucleotide sugar dehydrogenase		373..1789	Amino sugar and nucleotide sugar r	COG1004
036.1_1336	CUST_6893_PI442190807	HOT208_60m_813L03	036.1_1336	TGGACAGAAATTGCGAAAGAAATGCGTGATCATGGATGGGTTTTTGATACAAGATCACTT	ugd	UDP-glucose 6-dehydrogenase/nucleotide sugar dehydrogenase		373..1789	Amino sugar and nucleotide sugar r	COG1004
036.1_1336	CUST_6894_PI442190807	HOT208_60m_813L03	036.1_1336	GGAGTAGTGAACTTGCTAAATTAACTGCAAATGCTTTTCTTGCTCAAAGGATCAGTTCCA	ugd	UDP-glucose 6-dehydrogenase/nucleotide sugar dehydrogenase		373..1789	Amino sugar and nucleotide sugar r	COG1004
038.1_WP_079296147.:	CUST_6937_PI442190807	HOT208_60m_813L03	038.1_WP_079296147.:	TTTGCAATTTGAGATATTTCAATCCAGTTGGAGCAGATCCATCGGGTTTGTTTGGAGAAA	galE	UDP-glucose 4-epimerase	WP_079296147.:	243..1295	Galactose metabolism	COG1087
038.1_WP_079296147.:	CUST_6938_PI442190807	HOT208_60m_813L03	038.1_WP_079296147.:	TGTACAATAAAATATGAGTTTGAGGATAGGCGGCCAGGGGATGTATCTTCTTTGATAGCT	galE	UDP-glucose 4-epimerase	WP_079296147.:	243..1295	Galactose metabolism	COG1087
038.1_WP_079296147.:	CUST_6939_PI442190807	HOT208_60m_813L03	038.1_WP_079296147.:	GACTGGCCTACTAAAGATGGCACAGCAATAAGAGATTATATTCATGTTTGCGACCTTTCT	galE	UDP-glucose 4-epimerase	WP_079296147.:	243..1295	Galactose metabolism	COG1087
038.1_WP_079296147.:	CUST_6940_PI442190807	HOT208_60m_813L03	038.1_WP_079296147.:	TCTAGCAGGATTGAAATCTGTTCCTGAATCTATTTCAGATCCTTTGAGGTATTGGGATGT	galE	UDP-glucose 4-epimerase	WP_079296147.:	243..1295	Galactose metabolism	COG1087
038.1_WP_079296147.:	CUST_6941_PI442190807	HOT208_60m_813L03	038.1_WP_079296147.:	ACCTTGGTACTGGTAAAGGCACTACCGTTCTTGAATTAATAAAAACATTCGAAAGAGTAA	galE	UDP-glucose 4-epimerase	WP_079296147.:	243..1295	Galactose metabolism	COG1087
038.1_WP_079296147.:	CUST_6942_PI442190807	HOT208_60m_813L03	038.1_WP_079296147.:	TCTCTTATAATCCAAGTAATCTTTTCCCTTATCTCAATTTGGTGGCAGGTGGAAAAAAGA	galE	UDP-glucose 4-epimerase	WP_079296147.:	243..1295	Galactose metabolism	COG1087
073.1_1446	CUST_7478_PI442190807	HOT208_60m_813L03	073.1_1446	CCATCCTTCTTCTGCATGTTTCGCTGTAGCTGGTCCTTTATCTAACAACAACGCAAAAAT	glk	Glucokinase		<1..310	Glycolysis	COG0837
073.1_1446	CUST_7479_PI442190807	HOT208_60m_813L03	073.1_1446	TCTTCTGATTGGGATTCTTTTGAAATGATCCTAGAAGATTTTTTTAAAAATGAATGCAAA	glk	Glucokinase		<1..310	Glycolysis	COG0837
073.1_1446	CUST_7480_PI442190807	HOT208_60m_813L03	073.1_1446	GTATTCGCAAGAGTAATAAATGATGATTCGCCTAAATTGTTATTCA	glk	Glucokinase		<1..310	Glycolysis	COG0837
073.1_1446	CUST_7481_PI442190807	HOT208_60m_813L03	073.1_1446	GAATTTTCTGGCTTGTGATTTAGGAGGTACCAAGGTTCTTTTGGGAGTATTCGCAAGAGT	glk	Glucokinase		<1..310	Glycolysis	COG0837
074.1_WP_079296276.:	CUST_7488_PI442190807	HOT208_60m_813L03	074.1_WP_079296276.:	AAACAGGTAAAACTAGTTTTGTTGGCGCTGAAGATCCTGGTGTTATTTCGGTTACGCAAA	tal	Transaldolase	WP_079296276.:	129..1130	Pentose Phosphate	COG0176
074.1_WP_079296276.:	CUST_7489_PI442190807	HOT208_60m_813L03	074.1_WP_079296276.:	AGATTGCAGCAACTTGGGAGGGAATTAAGGCAGCTGAAATTTTGGAAAAAGAGGGTATTA	tal	Transaldolase	WP_079296276.:	129..1130	Pentose Phosphate	COG0176
074.1_WP_079296276.:	CUST_7490_PI442190807	HOT208_60m_813L03	074.1_WP_079296276.:	TATTTCAGGGCGCGTATCTACAGAAGTTGATGCAAGACTAAGCTTTGACACTGAAGCTAC	tal	Transaldolase	WP_079296276.:	129..1130	Pentose Phosphate	COG0176
074.1_WP_079296276.:	CUST_7491_PI442190807	HOT208_60m_813L03	074.1_WP_079296276.:	CTTGTGCCAATGCAAAGATAACTCTGATTTCTCCGTTCGTTGGCCGTATATTAGATTGGC	tal	Transaldolase	WP_079296276.:	129..1130	Pentose Phosphate	COG0176
074.1_WP_079296276.:	CUST_7492_PI442190807	HOT208_60m_813L03	074.1_WP_079296276.:	AGCAGGTTGCGATCTTTTAACAATCGCACCAAAATTTCTTGAGGAACTGAAAAAAGAAAA	tal	Transaldolase	WP_079296276.:	129..1130	Pentose Phosphate	COG0176
074.1_WP_079296276.:	CUST_7493_PI442190807	HOT208_60m_813L03	074.1_WP_079296276.:	AAAGCTTAGTGAAGGTATTACTGGATTCAGTAAGGCTATAGAAGAATTGGAAGAGCTGCT	tal	Transaldolase	WP_079296276.:	129..1130	Pentose Phosphate	COG0176
102.1_WP_079322947.:	CUST_97_PI442190803	HOT208_60m_813G15	102.1_WP_079322947.:	TGTTATAGCAGGAGCTCCCCTTCAAGAAGCATTTCGGAATGCTGATGATGTTCTAAGAAT	ftsZ	cell division protein FtsZ	WP_079322947.:	8..1075	Cell Division	COG0206
102.1_WP_079322947.:	CUST_98_PI442190803	HOT208_60m_813G15	102.1_WP_079322947.:	GGTCGATGAGGCAATGGAAGGAGAGATACAAGTAACTGTGATCGCTACAGGATTTGAAAC	ftsZ	cell division protein FtsZ	WP_079322947.:	8..1075	Cell Division	COG0206
102.1_WP_079322947.:	CUST_99_PI442190803	HOT208_60m_813G15	102.1_WP_079322947.:	CAGCACAGGCAGCAATGAATAGTCCTTTATTGGAAGCAGCTAGAATTGATGGAGCTAAAG	ftsZ	cell division protein FtsZ	WP_079322947.:	8..1075	Cell Division	COG0206
102.1_WP_079322947.:	CUST_100_PI442190803	HOT208_60m_813G15	102.1_WP_079322947.:	CCCAGGATTAGTTAATGTTGATTTTGCTGACGTAAGATCAGTTATGACTGCAGCTGGCAC	ftsZ	cell division protein FtsZ	WP_079322947.:	8..1075	Cell Division	COG0206
102.1_WP_079322947.:	CUST_101_PI442190803	HOT208_60m_813G15	102.1_WP_079322947.:	CCCGTTGTTGCAGAAGTAGCCAAGCAAAGTGGAGCTTTAACAGTAGGTATAGTAACCAAA	ftsZ	cell division protein FtsZ	WP_079322947.:	8..1075	Cell Division	COG0206
102.1_WP_079322947.:	CUST_102_PI442190803	HOT208_60m_813G15	102.1_WP_079322947.:	GCGTCAAGCAGAGGAGGGGATCGCAAGACTAGCTGAAAACGTTGATACTCTTATAGTTAT	ftsZ	cell division protein FtsZ	WP_079322947.:	8..1075	Cell Division	COG0206
102.1_WP_079322949.:	CUST_176_PI442190803	HOT208_60m_813G15	102.1_WP_079322949.:	TGATGCGATAGACGAAGATCCAGAGAAACCAGCAGGATTATATACTGCTTGGATGGTACT	glcH	GPH family sugar transporter	WP_079322949.:	12589..13935	GlcH sugar transporter	COG2211
102.1_WP_079322949.:	CUST_177_PI442190803	HOT208_60m_813G15	102.1_WP_079322949.:	AATCTGTCTTATTGGTATTGGAGCTTCAACCGCTTTTCTTATCCCTTGGTCACTACTTCC	glcH	GPH family sugar transporter	WP_079322949.:	12589..13935	GlcH sugar transporter	COG2211
102.1_WP_079322949.:	CUST_178_PI442190803	HOT208_60m_813G15	102.1_WP_079322949.:	GCGAAGTGGATCCCGATACCTTTTCAAATTAGCGCTTTACTGGGTTTACAAATATGGACC	glcH	GPH family sugar transporter	WP_079322949.:	12589..13935	GlcH sugar transporter	COG2211
102.1_WP_079322949.:	CUST_179_PI442190803	HOT208_60m_813G15	102.1_WP_079322949.:	TTCTCTGGTGCGCCTTACAATTGATGCAAACGGTAGCGTTAATCTATGTAGAGGATGTAC	glcH	GPH family sugar transporter	WP_079322949.:	12589..13935	GlcH sugar transporter	COG2211
102.1_WP_079322949.:	CUST_180_PI442190803	HOT208_60m_813G15	102.1_WP_079322949.:	TCGCCAAAAAAGCGAGAAGGCCTTCAGGAAAAGTTGAAGCAATAACACTTCAATTCAAAA	glcH	GPH family sugar transporter	WP_079322949.:	12589..13935	GlcH sugar transporter	COG2211
102.1_WP_079322949.:	CUST_181_PI442190803	HOT208_60m_813G15	102.1_WP_079322949.:	TAAAATTAGTGGATGCATTGCAGTTGCTGCAACATTAATTTCTTGTTGGGGATTGGCTCC	glcH	GPH family sugar transporter	WP_079322949.:	12589..13935	GlcH sugar transporter	COG2211
110.1_WP_079323018.:	CUST_421_PI442190803	HOT208_60m_813G15	110.1_WP_079323018.:	TCACTTATTGGGAGGATATGCATTTATTGGAGAAGGAATTCCAGTTGCTCTAGGAGCAGC	pdhA	Pyruvate dehydrogenase E1 component alpha subunit	WP_079323018.:	<1..931	Glycolysis/Krebs cycle	COG1071
110.1_WP_079323018.:	CUST_422_PI442190803	HOT208_60m_813G15	110.1_WP_079323018.:	AAATTGATACGGAGATCTCAGAATCCGTTAAAAATGCTTTAGAAGCACCGGAACCTCCCT	pdhA	Pyruvate dehydrogenase E1 component alpha subunit	WP_079323018.:	<1..931	Glycolysis/Krebs cycle	COG1071
110.1_WP_079323018.:	CUST_423_PI442190803	HOT208_60m_813G15	110.1_WP_079323018.:	TGCAGATCCTGATGAATTAAGGTCTGAGAAAGAGAAAGAGTTTTGGGGGCAAAGAGATCC	pdhA	Pyruvate dehydrogenase E1 component alpha subunit	WP_079323018.:	<1..931	Glycolysis/Krebs cycle	COG1071
110.1_WP_079323018.:	CUST_424_PI442190803	HOT208_60m_813G15	110.1_WP_079323018.:	AGGTAATAGTAATAGTGATGCGGTTACTGCAGCATTCTTTGGGGATGGGACTTGCAATAA	pdhA	Pyruvate dehydrogenase E1 component alpha subunit	WP_079323018.:	<1..931	Glycolysis/Krebs cycle	COG1071
110.1_WP_079323018.:	CUST_425_PI442190803	HOT208_60m_813G15	110.1_WP_079323018.:	GAGGAAGTTGATGGAATGGATGTACTAGCAGTAAGAGGAGCAGCACAAAGAGCAATTGAA	pdhA	Pyruvate dehydrogenase E1 component alpha subunit	WP_079323018.:	<1..931	Glycolysis/Krebs cycle	COG1071
110.1_WP_079323018.:	CUST_426_PI442190803	HOT208_60m_813G15	110.1_WP_079323018.:	TGTTGAGAATAATAAATGGGCTATTGGTATGGCTCACGATAGAGCAACAAGCAATCCCGA	pdhA	Pyruvate dehydrogenase E1 component alpha subunit	WP_079323018.:	<1..931	Glycolysis/Krebs cycle	COG1071
112.1_WP_079323105.:	CUST_698_PI442190803	HOT208_60m_813G15	112.1_WP_079323105.:	ACAACTTGATGCAGGAGCTATTATCCCTTTAACTAAATCAGGTTCTACCGCCCGAAATGT	pykF	Pyruvate kinase	WP_079323105.:	1960..3753	Glycolysis	COG0469
112.1_WP_079323105.:	CUST_699_PI442190803	HOT208_60m_813G15	112.1_WP_079323105.:	TTAAGGCTATTGAAAGCCACTTACCCAGTACCATCCCAAATGCTATTAGTGCAGCAGTAA	pykF	Pyruvate kinase	WP_079323105.:	1960..3753	Glycolysis	COG0469
112.1_WP_079323105.:	CUST_700_PI442190803	HOT208_60m_813G15	112.1_WP_079323105.:	TACAGAAAGGAGTGTAGCGAGAAGATTGCAACTTGTTTGGGGAGTTACTCCCATAGTAGT	pykF	Pyruvate kinase	WP_079323105.:	1960..3753	Glycolysis	COG0469
112.1_WP_079323105.:	CUST_701_PI442190803	HOT208_60m_813G15	112.1_WP_079323105.:	TCAGGGAGATTTAGTAGTTCAAACTGCAGGTACATTAACTGGCATTAGCGGCTCTACAGA	pykF	Pyruvate kinase	WP_079323105.:	1960..3753	Glycolysis	COG0469
112.1_WP_079323105.:	CUST_702_PI442190803	HOT208_60m_813G15	112.1_WP_079323105.:	TGGCGACCTTATTACACTTCAGCTTAATGAGGGTGTTATTTACATGGGGCAAATTGAAGA	pykF	Pyruvate kinase	WP_079323105.:	1960..3753	Glycolysis	COG0469
112.1_WP_079323105.:	CUST_703_PI442190803	HOT208_60m_813G15	112.1_WP_079323105.:	ACGATGAAAGAACTGCAAAAACTTTTAGTTTAGCGATGCAAATTGCTCAGGAAATGGGAA	pykF	Pyruvate kinase	WP_079323105.:	1960..3753	Glycolysis	COG0469
112.1_WP_079323119.:	CUST_743_PI442190803	HOT208_60m_813G15	112.1_WP_079323119.:	TTATCTCAAACAGCAAGATGGTACATCGGAGGCATACTTAAACATGCGCCTTCATTCCTA	glnA	Glutamine synthetase/ glutamate--ammonialigase	WP_079323119.:	10972..12393	Nitrogen Metabolism	COG0174
112.1_WP_079323119.:	CUST_744_PI442190803	HOT208_60m_813G15	112.1_WP_079323119.:	CAGGTGACGCACTTGCAAATCCTTACTTAGCATTCTCTGTAATGATGCTTGCTGGTATTG	glnA	Glutamine synthetase/ glutamate--ammonialigase	WP_079323119.:	10972..12393	Nitrogen Metabolism	COG0174
112.1_WP_079323119.:	CUST_745_PI442190803	HOT208_60m_813G15	112.1_WP_079323119.:	AAACAGCAACATTTATGCCCAAGCCTGTTTTTAACGATAACGGAACAGGAATGCATGTTC	glnA	Glutamine synthetase/ glutamate--ammonialigase	WP_079323119.:	10972..12393	Nitrogen Metabolism	COG0174
112.1_WP_079323119.:	CUST_746_PI442190803	HOT208_60m_813G15	112.1_WP_079323119.:	GAAGTTGCTGGTGCAGGCCAACACGAGCTTGGAATGAAATTTGATTCATTAATAAATGCT	glnA	Glutamine synthetase/ glutamate--ammonialigase	WP_079323119.:	10972..12393	Nitrogen Metabolism	COG0174
112.1_WP_079323119.:	CUST_747_PI442190803	HOT208_60m_813G15	112.1_WP_079323119.:	TTCCAGGATTCGAAGCGCCTGTAAATCTAGTTTATTCTGAAGGTAATAGATCAGCTGCGG	glnA	Glutamine synthetase/ glutamate--ammonialigase	WP_079323119.:	10972..12393	Nitrogen Metabolism	COG0174
112.1_WP_079323119.:	CUST_748_PI442190803	HOT208_60m_813G15	112.1_WP_079323119.:	GAGGTACAACAACTAAGACAAAGGCCTCATCCCCATGAATTCTTCATGTATTACGATGCA	glnA	Glutamine synthetase/ glutamate--ammonialigase	WP_079323119.:	10972..12393	Nitrogen Metabolism	COG0174
112.1_WP_025891660.:	CUST_799_PI442190803	HOT208_60m_813G15	112.1_WP_025891660.:	GTGCAGAATTAATTGCCTTGATAACAGAAGAGTGTTTTGACGATCTTGATGCCCGACCAA	pdhB	Pyruvate dehydrogenase E1 component beta subunit	WP_025891660.:	20159..21142	Glycolysis/Krebs cycle	COG0022
112.1_WP_025891660.:	CUST_800_PI442190803	HOT208_60m_813G15	112.1_WP_025891660.:	GTTTTACGGGTATGGCTGTAGGTGCAGCAATGACTGGTTTAAGACCAATAGTAGAAGGAA	pdhB	Pyruvate dehydrogenase E1 component beta subunit	WP_025891660.:	20159..21142	Glycolysis/Krebs cycle	COG0022
112.1_WP_025891660.:	CUST_801_PI442190803	HOT208_60m_813G15	112.1_WP_025891660.:	ATTACCTGAGGGTGATTATACTTGCGCTTTAGATCAGGCTGACGTTGTAAAAGAAGGCAA	pdhB	Pyruvate dehydrogenase E1 component beta subunit	WP_025891660.:	20159..21142	Glycolysis/Krebs cycle	COG0022
112.1_WP_025891660.:	CUST_802_PI442190803	HOT208_60m_813G15	112.1_WP_025891660.:	GAAATTATAAAATACCAGCAGTAGTTCGAGGACCTGGAGGAGTTGGTCGTCAACTTGGTG	pdhB	Pyruvate dehydrogenase E1 component beta subunit	WP_025891660.:	20159..21142	Glycolysis/Krebs cycle	COG0022
112.1_WP_025891660.:	CUST_803_PI442190803	HOT208_60m_813G15	112.1_WP_025891660.:	TCATGCAGTTCCTGGTATAAAGATTGTTGCATGTAGTACACCCACAAATGCTAAAGGTTT	pdhB	Pyruvate dehydrogenase E1 component beta subunit	WP_025891660.:	20159..21142	Glycolysis/Krebs cycle	COG0022
112.1_WP_025891660.:	CUST_804_PI442190803	HOT208_60m_813G15	112.1_WP_025891660.:	TCAGGATATTCCAACTCCTTATAATGGAAATCTCGAGAATTTGACAATAATCCAACCACA	pdhB	Pyruvate dehydrogenase E1 component beta subunit	WP_025891660.:	20159..21142	Glycolysis/Krebs cycle	COG0022
114.1_WP_079323234.:	CUST_1136_PI442190803	HOT208_60m_813G15	114.1_WP_079323234.:	ATCCAGCAGCACTATTATCATCCGCATGGTATTTAACTGGGGATGGTATTGGAAAGAGAG	pgi	Glucose-6-phosphate isomerase	WP_079323234.:	26..1609	Glycolysis/Gluconeogenesis	COG0166
114.1_WP_079323234.:	CUST_1137_PI442190803	HOT208_60m_813G15	114.1_WP_079323234.:	TTTCACTAGGTGCCTTAATCGCTTTATTTGAGAGGGCTGTATCCTTCTACGCTGAATTGG	pgi	Glucose-6-phosphate isomerase	WP_079323234.:	26..1609	Glycolysis/Gluconeogenesis	COG0166
114.1_WP_079323234.:	CUST_1138_PI442190803	HOT208_60m_813G15	114.1_WP_079323234.:	ATGCATATGATCAACCTGGAGTTGAAGCTGGAAAGAAAGCAGCTGCAAATATTATTGAGT	pgi	Glucose-6-phosphate isomerase	WP_079323234.:	26..1609	Glycolysis/Gluconeogenesis	COG0166
114.1_WP_079323234.:	CUST_1139_PI442190803	HOT208_60m_813G15	114.1_WP_079323234.:	TCTGGTTTTTTACAAGGAACCAGATCAGCACTCTCTAGTGAAAACAGACAAAGTATCACT	pgi	Glucose-6-phosphate isomerase	WP_079323234.:	26..1609	Glycolysis/Gluconeogenesis	COG0166
114.1_WP_079323234.:	CUST_1140_PI442190803	HOT208_60m_813G15	114.1_WP_079323234.:	TTCCGTTTTTGGTAATAAAGGATCTACAGATCAACATGCATATGTTCAGCAACTGAGAGA	pgi	Glucose-6-phosphate isomerase	WP_079323234.:	26..1609	Glycolysis/Gluconeogenesis	COG0166
114.1_WP_079323234.:	CUST_1141_PI442190803	HOT208_60m_813G15	114.1_WP_079323234.:	ACTCCGTGAAATGTGTTTCGGTAATGATAATTATTTAGTTAAGGGCGATTGGTCAAATCC	pgi	Glucose-6-phosphate isomerase	WP_079323234.:	26..1609	Glycolysis/Gluconeogenesis	COG0166
117.1_WP_025927692.:	CUST_1321_PI442190803	HOT208_60m_813G15	117.1_WP_025927692.:	TTTCCACTTCTCTTGGAAAATGCAGAGTGATGTTTGGGGACTTACTGGTGGTAATTTTGC	psaA	Photosystem I P700 chlorophyll A apoprotein A1 PsaA	WP_025927692.:	16550..18853	Photosystem I	COG2885
117.1_WP_025927692.:	CUST_1322_PI442190803	HOT208_60m_813G15	117.1_WP_025927692.:	TGTACGGTTTGATGTTCTTGGGAGCTCACTTTATATGGGCGTTCAGTTTAATGTTCCTCT	psaA	Photosystem I P700 chlorophyll A apoprotein A1 PsaA	WP_025927692.:	16550..18853	Photosystem I	COG2885
117.1_WP_025927692.:	CUST_1323_PI442190803	HOT208_60m_813G15	117.1_WP_025927692.:	GGTTGGCTAAGAGATTTCCTCTGGGCTCAAGCTTCTCAAGTATTAACAAGCTATGGTCAA	psaA	Photosystem I P700 chlorophyll A apoprotein A1 PsaA	WP_025927692.:	16550..18853	Photosystem I	COG2885
117.1_WP_025927692.:	CUST_1324_PI442190803	HOT208_60m_813G15	117.1_WP_025927692.:	GGAAGAGGTGGTACATGTCAAGTTTCTTCATGGGATCACGTTTTCTTAGCTCTCTTCTGG	psaA	Photosystem I P700 chlorophyll A apoprotein A1 PsaA	WP_025927692.:	16550..18853	Photosystem I	COG2885
117.1_WP_025927692.:	CUST_1325_PI442190803	HOT208_60m_813G15	117.1_WP_025927692.:	GGACTTCCCTTTTAGCAGGTACTGCAGAAGCTTTACCTCACAAAGCTTTAAGTGAAGTCT	psaA	Photosystem I P700 chlorophyll A apoprotein A1 PsaA	WP_025927692.:	16550..18853	Photosystem I	COG2885
117.1_WP_025927692.:	CUST_1326_PI442190803	HOT208_60m_813G15	117.1_WP_025927692.:	CTATTCAACCTCGAGCTCTATCTATTACTCAGGGACGTGCAGTAGGTGTTACACACTTCC	psaA	Photosystem I P700 chlorophyll A apoprotein A1 PsaA	WP_025927692.:	16550..18853	Photosystem I	COG2885
117.1_WP_011819135.:	CUST_1327_PI442190803	HOT208_60m_813G15	117.1_WP_011819135.:	AAATTTACTAGCATGGGTAACTTTCTACTGGCATTGGAAACACCTAGCAATTTGGCAGGG	psaB	Photosystem I P700 chlorophyll A apoprotein A2 PsaB	WP_011819135.:	18881..21109	Photosystem I	COG2885
117.1_WP_011819135.:	CUST_1328_PI442190803	HOT208_60m_813G15	117.1_WP_011819135.:	GTGCTTTCTTACCTATCGGTCCTGCAGATTTCTTAGTTCACCATGCTATCGCTTTAGGTC	psaB	Photosystem I P700 chlorophyll A apoprotein A2 PsaB	WP_011819135.:	18881..21109	Photosystem I	COG2885
117.1_WP_011819135.:	CUST_1329_PI442190803	HOT208_60m_813G15	117.1_WP_011819135.:	CCTTGTTGGCTGGAGAGATAAGCCAGTTGCACTTTCAATTGTTCAAGCTAGATTAGTTGG	psaB	Photosystem I P700 chlorophyll A apoprotein A2 PsaB	WP_011819135.:	18881..21109	Photosystem I	COG2885
117.1_WP_011819135.:	CUST_1330_PI442190803	HOT208_60m_813G15	117.1_WP_011819135.:	TGGACGTGGAGGTACTTGTGATAGTTCTTCTTGGGACGCTATGTACTTAGCTATGTTCTG	psaB	Photosystem I P700 chlorophyll A apoprotein A2 PsaB	WP_011819135.:	18881..21109	Photosystem I	COG2885
117.1_WP_011819135.:	CUST_1331_PI442190803	HOT208_60m_813G15	117.1_WP_011819135.:	GCTTGGATGTTCCTATTCGGTCACTTAGTTTGGGCTACTGGCTTCATGTTCCTAATATCA	psaB	Photosystem I P700 chlorophyll A apoprotein A2 PsaB	WP_011819135.:	18881..21109	Photosystem I	COG2885
117.1_WP_011819135.:	CUST_1332_PI442190803	HOT208_60m_813G15	117.1_WP_011819135.:	TCAGCAAGTCTCGCTGCTAATTCATTGCCAGGTAATCACTACTGGATGGATTTGATCAAT	psaB	Photosystem I P700 chlorophyll A apoprotein A2 PsaB	WP_011819135.:	18881..21109	Photosystem I	COG2885
117.1_WP_079323265.:	CUST_1382_PI442190803	HOT208_60m_813G15	117.1_WP_079323265.:	CGGTGACAGAAGGAGCAGGTGATCATTGTTGTGAATACATGACTGGTGGAAAAGTAGTTA	glsF	Ferredoxin-dependent glutamate synthase	WP_079323265.:	27516..31095	Nitrogen Metabolism	COG0069
117.1_WP_079323265.:	CUST_1383_PI442190803	HOT208_60m_813G15	117.1_WP_079323265.:	AATACAGATAGAAGTGTTTGTGCGAAAATATCAGGCGAAATCGCAGAACTTCACGGCAAC	glsF	Ferredoxin-dependent glutamate synthase	WP_079323265.:	27516..31095	Nitrogen Metabolism	COG0069
117.1_WP_079323265.:	CUST_1384_PI442190803	HOT208_60m_813G15	117.1_WP_079323265.:	CTGGCTTCAATGGCCAACTCAACTTAAATTTCAAAGGATATGCAGGACAAAGCTTTGGTG	glsF	Ferredoxin-dependent glutamate synthase	WP_079323265.:	27516..31095	Nitrogen Metabolism	COG0069
117.1_WP_079323265.:	CUST_1385_PI442190803	HOT208_60m_813G15	117.1_WP_079323265.:	CTAGGTTCCACAGGAAGGAATATTGGAGCGGGCATGACTGGTGGAATAGCTTTTATAATC	glsF	Ferredoxin-dependent glutamate synthase	WP_079323265.:	27516..31095	Nitrogen Metabolism	COG0069
117.1_WP_079323265.:	CUST_1386_PI442190803	HOT208_60m_813G15	117.1_WP_079323265.:	TGGAGCAACAGGTGGAAAATTATTTGCATTAGGAAAATCGGGAGAAAGATTTGCGGTTAG	glsF	Ferredoxin-dependent glutamate synthase	WP_079323265.:	27516..31095	Nitrogen Metabolism	COG0069
117.1_WP_079323265.:	CUST_1387_PI442190803	HOT208_60m_813G15	117.1_WP_079323265.:	TCTCATTTTAAGAGTACTTTCAAATTGATTGTTCCCCCAAGCGAAGAAGAGATGCTTGGT	glsF	Ferredoxin-dependent glutamate synthase	WP_079323265.:	27516..31095	Nitrogen Metabolism	COG0069
159.1_332	CUST_1659_PI442190803	HOT208_60m_813G15	159.1_332	ATTACCAAAACCCGGAGTTATTTCTCATTCTGAAAAGTCCCTTGAGTATCCAGCTGGTGG	rbsK	Sugar kinase, ribokinase family		<1..423	Pentose Phosphate	COG0524
159.1_332	CUST_1660_PI442190803	HOT208_60m_813G15	159.1_332	AATTGACTCTCAAGGCGAAAGAGCAATAACAGTTATTGGAGAAAGGTTAGCTCCAACTCA	rbsK	Sugar kinase, ribokinase family		<1..423	Pentose Phosphate	COG0524
159.1_332	CUST_1661_PI442190803	HOT208_60m_813G15	159.1_332	CTCTCAAATATGGGAATTAAGTTGCATGTAGCTTGGCGTGATAAACCAACTAGAAGAGGA	rbsK	Sugar kinase, ribokinase family		<1..423	Pentose Phosphate	COG0524
159.1_332	CUST_1662_PI442190803	HOT208_60m_813G15	159.1_332	ACCAAATTCATTTTTTTACGTCAATAGGAAGTGATGATTCTGGAGATAAGTGTTTCAAGA	rbsK	Sugar kinase, ribokinase family		<1..423	Pentose Phosphate	COG0524
159.1_332	CUST_1663_PI442190803	HOT208_60m_813G15	159.1_332	AGGAAATTTAAATTTTGCCGTTGTTGGTCATATTGAGTGGATAAATTTTTTAAAGGTTGA	rbsK	Sugar kinase, ribokinase family		<1..423	Pentose Phosphate	COG0524
159.1_332	CUST_1664_PI442190803	HOT208_60m_813G15	159.1_332	ACTCATAAAGACAATTTAGATTGGAACATATTAAAAAAAATGAACGGAATTTTTA	rbsK	Sugar kinase, ribokinase family		<1..423	Pentose Phosphate	COG0524
170.1_348	CUST_1732_PI442190803	HOT208_60m_813G15	170.1_348	TTGCTAAAGTAATGGGAAGAGAAGCCAGTGAATTTTTCGGCCCATATGCAAATAGTGACG	rbsK	Sugar kinase, ribokinase family		288..691	Pentose Phosphate	COG0524
170.1_348	CUST_1733_PI442190803	HOT208_60m_813G15	170.1_348	AAGGTTGATTCTTATGGATGTGGCGATTCTTTTTCAGGTGGGATTCTTTAAGGAATGGCA	rbsK	Sugar kinase, ribokinase family		288..691	Pentose Phosphate	COG0524
170.1_348	CUST_1734_PI442190803	HOT208_60m_813G15	170.1_348	ACCGAGGGAGAGAAGGGAGGCATAATATTTCCAGGAGGAAGATATAAGGCTCTTAAAAAC	rbsK	Sugar kinase, ribokinase family		288..691	Pentose Phosphate	COG0524
170.1_348	CUST_1735_PI442190803	HOT208_60m_813G15	170.1_348	AGGCAGTAATCTTGATCCCGGAGAAGTTTTTTCTTTTTCTGAATTATCGATAAAACCCAA	rbsK	Sugar kinase, ribokinase family		288..691	Pentose Phosphate	COG0524
170.1_348	CUST_1736_PI442190803	HOT208_60m_813G15	170.1_348	AAAATGGCTAGATCAGCTTCAATACTGTGTACAACTCCAAGGGTAGGATTAAATACAATT	rbsK	Sugar kinase, ribokinase family		288..691	Pentose Phosphate	COG0524
020.1_WP_079321889.:	CUST_1852_PI442190803	HOT208_60m_813G15	020.1_WP_079321889.:	TGCTTGAGTTCCTCTGGGATTACATAAGAGATCTTGCTAGGACGCAAATAGGCGAAAAAG	atpI	ATP synthase subunit A	WP_079321889.:	27..563	Energy Metabolism	COG0356
020.1_WP_079321889.:	CUST_1853_PI442190803	HOT208_60m_813G15	020.1_WP_079321889.:	ATGTTTCTAGGATTGTTTACTAGTGCAATTCAGGCATTGATTTTTGCAACTTTGGCGGCC	atpI	ATP synthase subunit A	WP_079321889.:	27..563	Energy Metabolism	COG0356
020.1_WP_079321889.:	CUST_1854_PI442190803	HOT208_60m_813G15	020.1_WP_079321889.:	AGGGTTGGAGATACTTCGAATACTATGTTCACCCAACCCCGATTATGCTTCCTTTCAAAA	atpI	ATP synthase subunit A	WP_079321889.:	27..563	Energy Metabolism	COG0356
020.1_WP_079321889.:	CUST_1855_PI442190803	HOT208_60m_813G15	020.1_WP_079321889.:	ACCAAGTGGAGAATTAGGAGCACCTACTGCTGATATCAATACAACTATAGCCTTGGCTTT	atpI	ATP synthase subunit A	WP_079321889.:	27..563	Energy Metabolism	COG0356
020.1_WP_079321889.:	CUST_1856_PI442190803	HOT208_60m_813G15	020.1_WP_079321889.:	TGGATGCCATTTATAGGTACTTTATTTTTATTCGTCTTTGTTAGTAATTGGGGAGGAGCT	atpI	ATP synthase subunit A	WP_079321889.:	27..563	Energy Metabolism	COG0356
020.1_WP_079321889.:	CUST_1857_PI442190803	HOT208_60m_813G15	020.1_WP_079321889.:	ACTTTGGCGGCCTATTACATTGGAGAAGCTGTTGAGGAACATCATTAG	atpI	ATP synthase subunit A	WP_079321889.:	27..563	Energy Metabolism	COG0356
020.1_WP_079321866.:	CUST_1880_PI442190803	HOT208_60m_813G15	020.1_WP_079321866.:	TCTCAATTTGCATCTGATCTTGATGAAGCAACTCAGCAACAACTTGAACGAGGCAAAAGA	atpA	ATP synthase alpha subunit	WP_079321866.:	2585..4102	Energy Metabolism	COG0056
020.1_WP_079321866.:	CUST_1881_PI442190803	HOT208_60m_813G15	020.1_WP_079321866.:	TCTCCCAATTATTGAAACTCAGGCAGGGGACGTTTCTGCTTACATCCCAACTAATGTTAT	atpA	ATP synthase alpha subunit	WP_079321866.:	2585..4102	Energy Metabolism	COG0056
020.1_WP_079321866.:	CUST_1882_PI442190803	HOT208_60m_813G15	020.1_WP_079321866.:	AGTTATTAAAGCAACCTCAATTCTCTCCGCTGAACCTTGCAGAACAAGTTGCAGTTGTTT	atpA	ATP synthase alpha subunit	WP_079321866.:	2585..4102	Energy Metabolism	COG0056
020.1_WP_079321866.:	CUST_1883_PI442190803	HOT208_60m_813G15	020.1_WP_079321866.:	GTAGATTGTTAGAAAGAGCAGCAAAACTTTCTGATGCTATGGGAGGGGGCTCTATGACAG	atpA	ATP synthase alpha subunit	WP_079321866.:	2585..4102	Energy Metabolism	COG0056
020.1_WP_079321866.:	CUST_1884_PI442190803	HOT208_60m_813G15	020.1_WP_079321866.:	ACAAGCTCAGGCTTATAGACAAATGTCTCTTCTTTTAAAAAGACCACCAGGAAGAGAGGC	atpA	ATP synthase alpha subunit	WP_079321866.:	2585..4102	Energy Metabolism	COG0056
020.1_WP_079321887.:	CUST_1941_PI442190803	HOT208_60m_813G15	020.1_WP_079321887.:	CCAGAGAAGCTCAAAGCTTTGAAAGAGATTTCGAAGGCAGAAGTTAGAATTCAAGCTGCA	atpC	ATP synthase epsilon subunit	WP_079321887.:	13387..13791	Energy Metabolism	COG0355
020.1_WP_079321887.:	CUST_1942_PI442190803	HOT208_60m_813G15	020.1_WP_079321887.:	AATGCCGAAATTGGATCTGAAATAGATGTTCAAAATGCTGAACAAGACCTTAAAGAAGCT	atpC	ATP synthase epsilon subunit	WP_079321887.:	13387..13791	Energy Metabolism	COG0355
020.1_WP_079321887.:	CUST_1943_PI442190803	HOT208_60m_813G15	020.1_WP_079321887.:	TGGTGTTTTAAGGCTAAGAATGAACTCTCAGTGGAAATCGATAGCTTTAATGGGGGGCTT	atpC	ATP synthase epsilon subunit	WP_079321887.:	13387..13791	Energy Metabolism	COG0355
020.1_WP_079321887.:	CUST_1944_PI442190803	HOT208_60m_813G15	020.1_WP_079321887.:	TTCCTAGCACTACTGGCCAACTAGGTGTGTTACCAGGACATATTTCTTTAGTTACGGCTA	atpC	ATP synthase epsilon subunit	WP_079321887.:	13387..13791	Energy Metabolism	COG0355
020.1_WP_079321887.:	CUST_1945_PI442190803	HOT208_60m_813G15	020.1_WP_079321887.:	ATGGCAATTTCTCTTAAAGTTTTAGCTCCTAACAAAAATGTCTATCAAGGC	atpC	ATP synthase epsilon subunit	WP_079321887.:	13387..13791	Energy Metabolism	COG0355
193.1_WP_079323288.:	CUST_1954_PI442190803	HOT208_60m_813G15	193.1_WP_079323288.:	AGGCTTTTAAAAATTGAGGAGGAATTGGGAAATCAAGCAAGATTCGCTGGAGCTTTAGGT	eno	Enolase	WP_079323288.:	148..1440	Glycolysis/Gluconeogenesis	COG0148
193.1_WP_079323288.:	CUST_1955_PI442190803	HOT208_60m_813G15	193.1_WP_079323288.:	GTGAGACTGAAGATACAACAATTGCAGATTTATCTGTCGCCACAAGATCGGGTCAGATCA	eno	Enolase	WP_079323288.:	148..1440	Glycolysis/Gluconeogenesis	COG0148
193.1_WP_079323288.:	CUST_1956_PI442190803	HOT208_60m_813G15	193.1_WP_079323288.:	TCCAATAGTTTCAATAGAGGACGGTTTAGCTGAGGATGATTGGGAAGGTTGGTCAGAATT	eno	Enolase	WP_079323288.:	148..1440	Glycolysis/Gluconeogenesis	COG0148
193.1_WP_079323288.:	CUST_1957_PI442190803	HOT208_60m_813G15	193.1_WP_079323288.:	CCGGGTTTAAGCCTGGTGAGCAGGTATCTTTAGCTTTAGATGCTGCTAGTACTGAATTTT	eno	Enolase	WP_079323288.:	148..1440	Glycolysis/Gluconeogenesis	COG0148
193.1_WP_079323288.:	CUST_1958_PI442190803	HOT208_60m_813G15	193.1_WP_079323288.:	ATCAAAAAGGTTTATCTACTGCTGTAGGAGATGAGGGAGGATTTGCCCCGAATTTGTCAT	eno	Enolase	WP_079323288.:	148..1440	Glycolysis/Gluconeogenesis	COG0148
193.1_WP_079323288.:	CUST_1959_PI442190803	HOT208_60m_813G15	193.1_WP_079323288.:	GGTGCTCATGCACCAAATAGTCTTGATTTTCAGGAATTTATGCTTGTCCCACATGGAGTT	eno	Enolase	WP_079323288.:	148..1440	Glycolysis/Gluconeogenesis	COG0148
193.1_WP_079323300.:	CUST_2044_PI442190803	HOT208_60m_813G15	193.1_WP_079323300.:	TCGATTTTGTATCCGAAATCAAAATTAAATATCCAAGAATTAATTTAGGCTCTGCCTCCA	eda	2-dehydro-3-deoxyphosphogluconate aldolase	WP_079323300.:	18043..18669	Entner-Dudoroff	COG0800
193.1_WP_079323300.:	CUST_2045_PI442190803	HOT208_60m_813G15	193.1_WP_079323300.:	AGATAGTTCGATAGATATACGCAACTATAAAAATATTGATTTCATTGCTGCTGGAGGACT	eda	2-dehydro-3-deoxyphosphogluconate aldolase	WP_079323300.:	18043..18669	Entner-Dudoroff	COG0800
193.1_WP_079323300.:	CUST_2046_PI442190803	HOT208_60m_813G15	193.1_WP_079323300.:	AGGGTATAAAGCAGTCGTAATTGGAGATAAAGCAATCAAAAATAAAAAATTTGATCCAAA	eda	2-dehydro-3-deoxyphosphogluconate aldolase	WP_079323300.:	18043..18669	Entner-Dudoroff	COG0800
193.1_WP_079323300.:	CUST_2047_PI442190803	HOT208_60m_813G15	193.1_WP_079323300.:	TGGATTAAATTTTTCGATGATGAAATTTTGGGATAAAGATCTTTTCAATTATGCGAAGTC	eda	2-dehydro-3-deoxyphosphogluconate aldolase	WP_079323300.:	18043..18669	Entner-Dudoroff	COG0800
193.1_WP_079323300.:	CUST_2048_PI442190803	HOT208_60m_813G15	193.1_WP_079323300.:	GATCTTAAGGAGGCGATAGATTTAAATTGCAACATTATCAAAATTTACCCAATTAAAAGT	eda	2-dehydro-3-deoxyphosphogluconate aldolase	WP_079323300.:	18043..18669	Entner-Dudoroff	COG0800
193.1_WP_079323300.:	CUST_2049_PI442190803	HOT208_60m_813G15	193.1_WP_079323300.:	AGATTCTTTTTCGGAGTACCTGAAAATTGAATCTTTTTTTTTACTTATAAAACCTGAAGA	eda	2-dehydro-3-deoxyphosphogluconate aldolase	WP_079323300.:	18043..18669	Entner-Dudoroff	COG0800
193.1_WP_079323376.:	CUST_2558_PI442190803	HOT208_60m_813G15	193.1_WP_079323376.:	CAAAAAAACTGCTAAGACTGATAAAGCTAACAAAGATATCAAAAAGT	minE	septum site-determining protein MinE	WP_079323376.:	104086..104421	Cell Division	COG0851
193.1_WP_079323376.:	CUST_2559_PI442190803	HOT208_60m_813G15	193.1_WP_079323376.:	GTCGCCAATTTGCCAATCAAAAGAACTATTTCAGGAGAAATAAAATTCAAAAAAACTGCT	minE	septum site-determining protein MinE	WP_079323376.:	104086..104421	Cell Division	COG0851
193.1_WP_079323376.:	CUST_2560_PI442190803	HOT208_60m_813G15	193.1_WP_079323376.:	TGAAATTGATTTTGAGGAGGTGGCAGTAAGTTTGGAGACTGAGGATAGAATGACTGCACT	minE	septum site-determining protein MinE	WP_079323376.:	104086..104421	Cell Division	COG0851
193.1_WP_079323376.:	CUST_2561_PI442190803	HOT208_60m_813G15	193.1_WP_079323376.:	AGTTCCTTAACAACTGATCTTTTAGATAAAATGAGGAAAGAGATTCTTGATGTTGTTGCT	minE	septum site-determining protein MinE	WP_079323376.:	104086..104421	Cell Division	COG0851
193.1_WP_079323376.:	CUST_2562_PI442190803	HOT208_60m_813G15	193.1_WP_079323376.:	ACGTCTAGTGCGAATACAGCTAGAGAAAGATTACAACTTGTACTTGCTCATGACAGAGTT	minE	septum site-determining protein MinE	WP_079323376.:	104086..104421	Cell Division	COG0851
193.1_WP_079323377.:	CUST_2563_PI442190803	HOT208_60m_813G15	193.1_WP_079323377.:	GCTATTGTTGTTACTAACCCAGAATTATCTGCAGTGCGAGATGCGGATAGAGTAATCGGG	minD	septum site-determining protein MinD	WP_079323377.:	104423..105238	Cell Division	COG2894
193.1_WP_079323377.:	CUST_2564_PI442190803	HOT208_60m_813G15	193.1_WP_079323377.:	GGTAAAACAACTTTAACTGCAAACTTAGGCATAGCACTTGCTAATAGCGGAGCAACCACT	minD	septum site-determining protein MinD	WP_079323377.:	104423..105238	Cell Division	COG2894
193.1_WP_079323377.:	CUST_2565_PI442190803	HOT208_60m_813G15	193.1_WP_079323377.:	TTGGTTAGACATAAAAAGGAACCTAATCTTGCTCTTCTACCTGCTGGCGATCCAAGGATG	minD	septum site-determining protein MinD	WP_079323377.:	104423..105238	Cell Division	COG2894
193.1_WP_079323377.:	CUST_2566_PI442190803	HOT208_60m_813G15	193.1_WP_079323377.:	AAATAGAGGCGAGCCACTGACTCTTTCAGATGGTAGATCACCCGCAAAGAAATGTTATTT	minD	septum site-determining protein MinD	WP_079323377.:	104423..105238	Cell Division	COG2894
193.1_WP_079323377.:	CUST_2567_PI442190803	HOT208_60m_813G15	193.1_WP_079323377.:	TTTTGTCTTAGTAGATTGTCCTGCTGGCGTTGAAGATGGCTTTAAAAATGCTCTTGCAGC	minD	septum site-determining protein MinD	WP_079323377.:	104423..105238	Cell Division	COG2894
193.1_WP_079323377.:	CUST_2568_PI442190803	HOT208_60m_813G15	193.1_WP_079323377.:	AGAGTTCGCCCTAACATGATGGCTAGTCAAGAAATGTTATCCATCGAAGATGTTCAAGGA	minD	septum site-determining protein MinD	WP_079323377.:	104423..105238	Cell Division	COG2894
193.1_WP_079323378.:	CUST_2569_PI442190803	HOT208_60m_813G15	193.1_WP_079323378.:	ATTACTAGGGATTGCATTCGCAGGGAAAAGCGGAAATAAAAATGCCTCTATTGCATCACT	minC	septum site-determining protein MinC	WP_079323378.:	105350..106012	Cell Division	COG0850
193.1_WP_079323378.:	CUST_2570_PI442190803	HOT208_60m_813G15	193.1_WP_079323378.:	AGGAACAGTGCGATCCGGTGAAAGAATATCTTCAAACGGAAACCTATGCATTATTGGAGA	minC	septum site-determining protein MinC	WP_079323378.:	105350..106012	Cell Division	COG0850
193.1_WP_079323378.:	CUST_2571_PI442190803	HOT208_60m_813G15	193.1_WP_079323378.:	AGATGTGATAGCTATAGGACCAAAGGATAAGCCCAAAAATTGTTATCCAGAAATTGCGGT	minC	septum site-determining protein MinC	WP_079323378.:	105350..106012	Cell Division	COG0850
193.1_WP_079323378.:	CUST_2572_PI442190803	HOT208_60m_813G15	193.1_WP_079323378.:	TGAAACTTTCAAAAGATTTTCATCCATCAAGGAACCTTTAGAAGCAAAAATTTTCGCAGT	minC	septum site-determining protein MinC	WP_079323378.:	105350..106012	Cell Division	COG0850
193.1_WP_079323378.:	CUST_2573_PI442190803	HOT208_60m_813G15	193.1_WP_079323378.:	TGGAGATGTTAATCCAGGAGCAATAGTTTCCGCTAAGAAAAACATTTACGTTTG	minC	septum site-determining protein MinC	WP_079323378.:	105350..106012	Cell Division	COG0850
193.1_WP_079323378.:	CUST_2574_PI442190803	HOT208_60m_813G15	193.1_WP_079323378.:	ACAATAATCAGTGGGAAGTACTTAAAAATAGATTCAGCTTATATAAAAGAGCAAGAGATT	minC	septum site-determining protein MinC	WP_079323378.:	105350..106012	Cell Division	COG0850
193.1_WP_079323405.:	CUST_2761_PI442190803	HOT208_60m_813G15	193.1_WP_079323405.:	TGCGACTGTTGTGAACATAATGATTTAATCCCAAATGATTTTGAGAGTATTAAAAAAGAA	ppc	Phosphoenolpyruvate carboxylase	WP_079323405.:	141050..141427	Krebs cycle and related genes	COG2352
193.1_WP_079323405.:	CUST_2763_PI442190803	HOT208_60m_813G15	193.1_WP_079323405.:	TCAGGCTGATGGATTAAAAGGCGTCCTTAATAGGGATTTTGATAAATGGTCTAATTTTGA	ppc	Phosphoenolpyruvate carboxylase	WP_079323405.:	141050..141427	Krebs cycle and related genes	COG2352
193.1_WP_079323405.:	CUST_2764_PI442190803	HOT208_60m_813G15	193.1_WP_079323405.:	TGAATCAAAATTCTGTCAAAATAATTGGTATAAATGATGAACCAAGAAAAGATTCACATT	ppc	Phosphoenolpyruvate carboxylase	WP_079323405.:	141050..141427	Krebs cycle and related genes	COG2352
194.1_WP_079323488.:	CUST_3203_PI442190803	HOT208_60m_813G15	194.1_WP_079323488.:	CGTTGATAACTGGCGTTGGCAAGGGGTTCCTTTTTATTTGAGAACAGGTAAAAGACTACC	zwf	glucose-6-phosphate dehydrogenase zwf	WP_079323488.:	10380..11903	Pentose Phosphate	COG0364
194.1_WP_079323488.:	CUST_3204_PI442190803	HOT208_60m_813G15	194.1_WP_079323488.:	ATGCTTTCTGACCCAACCTTATTTACTCGAAGTGATGAAGTAGAGGCAGCTTGGAAACTT	zwf	glucose-6-phosphate dehydrogenase zwf	WP_079323488.:	10380..11903	Pentose Phosphate	COG0364
194.1_WP_079323488.:	CUST_3205_PI442190803	HOT208_60m_813G15	194.1_WP_079323488.:	CATATGACTCAAATGCTTGCTGTTACTGCAATGGAACCTCCTGGAAAGTTTGAACCAGAA	zwf	glucose-6-phosphate dehydrogenase zwf	WP_079323488.:	10380..11903	Pentose Phosphate	COG0364
194.1_WP_079323488.:	CUST_3206_PI442190803	HOT208_60m_813G15	194.1_WP_079323488.:	TTCAGCCAAATGAAGGTGCTACTTTCAAATTTGAGGTAAAATCCCCTGGTTCAGGAATGA	zwf	glucose-6-phosphate dehydrogenase zwf	WP_079323488.:	10380..11903	Pentose Phosphate	COG0364
194.1_WP_079323488.:	CUST_3207_PI442190803	HOT208_60m_813G15	194.1_WP_079323488.:	TGCTGACGAAAATGAACCATGGAATTGTTGCATAAGAGGTCAATATGGAGAGGGAGGAAA	zwf	glucose-6-phosphate dehydrogenase zwf	WP_079323488.:	10380..11903	Pentose Phosphate	COG0364
194.1_WP_079323488.:	CUST_3208_PI442190803	HOT208_60m_813G15	194.1_WP_079323488.:	GACCTGTTGAGATGGAATTTTCTTATGATGAATCCTTTGGAGAACCATCAGATGAAGGCT	zwf	glucose-6-phosphate dehydrogenase zwf	WP_079323488.:	10380..11903	Pentose Phosphate	COG0364
194.1_WP_079323489.:	CUST_3209_PI442190803	HOT208_60m_813G15	194.1_WP_079323489.:	AATACTTGGTTGTGAATCAGTGGAATGTATGAGACTTGAAGCAGGGGGAATGGCTGATAT	opcA	Glucose-6-P dehydrogenase subunit opcA	WP_079323489.:	11906..13207	Pentose Phosphate	COG3429
194.1_WP_079323489.:	CUST_3210_PI442190803	HOT208_60m_813G15	194.1_WP_079323489.:	ATTGGAGGGAATCATTGGCAATGATTTTTGATCCGCCTTCGAGGAGACCAATTTTAGATC	opcA	Glucose-6-P dehydrogenase subunit opcA	WP_079323489.:	11906..13207	Pentose Phosphate	COG3429
194.1_WP_079323489.:	CUST_3211_PI442190803	HOT208_60m_813G15	194.1_WP_079323489.:	ATGAATTACCTTCATGGTTGTGGTGGAATGGAAACTTGGATGAATCGCCTGAAATCTTCG	opcA	Glucose-6-P dehydrogenase subunit opcA	WP_079323489.:	11906..13207	Pentose Phosphate	COG3429
194.1_WP_079323489.:	CUST_3212_PI442190803	HOT208_60m_813G15	194.1_WP_079323489.:	TCCCTTATCTTTGGGAAATCCAAGTATTCATTTTGGACAAGTTATTGGATTGAGGCTGAT	opcA	Glucose-6-P dehydrogenase subunit opcA	WP_079323489.:	11906..13207	Pentose Phosphate	COG3429
194.1_WP_079323489.:	CUST_3213_PI442190803	HOT208_60m_813G15	194.1_WP_079323489.:	TGGTCTACGGTTAATAATTGATACTGCTCTTGGATCGCCTCAAAGATGTTTAAAGGTTTT	opcA	Glucose-6-P dehydrogenase subunit opcA	WP_079323489.:	11906..13207	Pentose Phosphate	COG3429
194.1_WP_079323489.:	CUST_3214_PI442190803	HOT208_60m_813G15	194.1_WP_079323489.:	CGCTGGAGATCATATGATTCAAGCTTTGTTTTTGATTTCATGGATTAGCGATAAACTTGG	opcA	Glucose-6-P dehydrogenase subunit opcA	WP_079323489.:	11906..13207	Pentose Phosphate	COG3429
197.1_WP_079323634.:	CUST_4065_PI442190803	HOT208_60m_813G15	197.1_WP_079323634.:	GTTACACTCCAGGGGTTGTTTTGGCTGGGAGAAGGATAAATAACTATATGGCAGATTGGA	ugd	UDP-glucose 6-dehydrogenase/nucleotide sugar dehydrogenase	WP_079323634.:	26163..27512	Amino sugar and nucleotide sugar r	COG1004
197.1_WP_079323634.:	CUST_4066_PI442190803	HOT208_60m_813G15	197.1_WP_079323634.:	ACTCTCGATGTACTGGAAGCCGCGAATACAAAATGGAATTTTTTAGATTTTAAGCCTGGA	ugd	UDP-glucose 6-dehydrogenase/nucleotide sugar dehydrogenase	WP_079323634.:	26163..27512	Amino sugar and nucleotide sugar r	COG1004
197.1_WP_079323634.:	CUST_4067_PI442190803	HOT208_60m_813G15	197.1_WP_079323634.:	TCAGATTCCTGATGATCAAAAATTTGATGCCATTATTGCTGCAGTTGCACATGATCAGTT	ugd	UDP-glucose 6-dehydrogenase/nucleotide sugar dehydrogenase	WP_079323634.:	26163..27512	Amino sugar and nucleotide sugar r	COG1004
197.1_WP_079323634.:	CUST_4068_PI442190803	HOT208_60m_813G15	197.1_WP_079323634.:	CGCTTCTTTCATTAAGGCTGGTACTCATCAAGCTAAAAGTATTAAAATTGCGGAAACTGC	ugd	UDP-glucose 6-dehydrogenase/nucleotide sugar dehydrogenase	WP_079323634.:	26163..27512	Amino sugar and nucleotide sugar r	COG1004
197.1_WP_079323634.:	CUST_4069_PI442190803	HOT208_60m_813G15	197.1_WP_079323634.:	GTTGGTGGACATTGTATTGGTGTTGACCCTTATTATTTAACTCACAGATCAACTTTAGCT	ugd	UDP-glucose 6-dehydrogenase/nucleotide sugar dehydrogenase	WP_079323634.:	26163..27512	Amino sugar and nucleotide sugar r	COG1004
197.1_WP_079323634.:	CUST_4070_PI442190803	HOT208_60m_813G15	197.1_WP_079323634.:	ACTAGGATTAACTTTTAAGGAGAATTGTCCTGATATGAGGAACTCTGGAGTTTTAAAATT	ugd	UDP-glucose 6-dehydrogenase/nucleotide sugar dehydrogenase	WP_079323634.:	26163..27512	Amino sugar and nucleotide sugar r	COG1004
197.1_WP_079323635.:	CUST_4071_PI442190803	HOT208_60m_813G15	197.1_WP_079323635.:	TCTTCTAGGAGAGCTGGTGATATTGAGTACTCTGTAGCGGACAATCAAAAGGCGAAAGAA	galE	UDP-glucose 4-epimerase	WP_079323635.:	27638..28705	Galactose metabolism	COG1087
197.1_WP_079323635.:	CUST_4072_PI442190803	HOT208_60m_813G15	197.1_WP_079323635.:	GCTCATACTTCAGGAGAATTAGGGGAAAGCCCCAAAAATACCCCAAACAATATAATGCCA	galE	UDP-glucose 4-epimerase	WP_079323635.:	27638..28705	Galactose metabolism	COG1087
197.1_WP_079323635.:	CUST_4073_PI442190803	HOT208_60m_813G15	197.1_WP_079323635.:	AATGGACAAATTTGAATGCAAAACACTTGTTTTTAGCAGCAGCGCTACGATTTATGGGAT	galE	UDP-glucose 4-epimerase	WP_079323635.:	27638..28705	Galactose metabolism	COG1087
197.1_WP_079323635.:	CUST_4074_PI442190803	HOT208_60m_813G15	197.1_WP_079323635.:	GGGACTCCAATCAGAGATTATATTCATGTAATGGATCTTGCTGATGGTCACTTAAAGGCA	galE	UDP-glucose 4-epimerase	WP_079323635.:	27638..28705	Galactose metabolism	COG1087
197.1_WP_079323635.:	CUST_4075_PI442190803	HOT208_60m_813G15	197.1_WP_079323635.:	AAAGATATGTGTAAAGACAGTTGGAACTGGGTATCTAAGAATCCAAATGGGTACTCAGAT	galE	UDP-glucose 4-epimerase	WP_079323635.:	27638..28705	Galactose metabolism	COG1087
197.1_WP_079323635.:	CUST_4076_PI442190803	HOT208_60m_813G15	197.1_WP_079323635.:	AAATTTAGGAACAGGTATTGGGAAAAGCGTTTTGCAATTAATTCATGTTTTTGAAGAGGT	galE	UDP-glucose 4-epimerase	WP_079323635.:	27638..28705	Galactose metabolism	COG1087
197.1_WP_079323636.:	CUST_4077_PI442190803	HOT208_60m_813G15	197.1_WP_079323636.:	GCAATATGCGAAGCAACTGGAGCCAATGTTAAAGAGGTATCAAGAGCAATTGGATCAGAT	ugd	UDP-glucose 6-dehydrogenase/nucleotide sugar dehydrogenase	WP_079323636.:	28708..30147	Amino sugar and nucleotide sugar r	COG1004
197.1_WP_079323636.:	CUST_4078_PI442190803	HOT208_60m_813G15	197.1_WP_079323636.:	AGAATCGGCTCAAAGTTTTTAAATACAGGCCCAGGTTTTGGTGGCAGTTGCTTTAAAAAG	ugd	UDP-glucose 6-dehydrogenase/nucleotide sugar dehydrogenase	WP_079323636.:	28708..30147	Amino sugar and nucleotide sugar r	COG1004
197.1_WP_079323636.:	CUST_4079_PI442190803	HOT208_60m_813G15	197.1_WP_079323636.:	TGGATTACACGAAGTGGCAGCCTTTTGGGAAAGTGTTGTAAATTTAAATAGTTGGCACCA	ugd	UDP-glucose 6-dehydrogenase/nucleotide sugar dehydrogenase	WP_079323636.:	28708..30147	Amino sugar and nucleotide sugar r	COG1004
197.1_WP_079323636.:	CUST_4080_PI442190803	HOT208_60m_813G15	197.1_WP_079323636.:	AGGTGGCAACTGGGATTTTGCGCAAGATTTATCTTGTATATTTAATAATGCCGATGCAGT	ugd	UDP-glucose 6-dehydrogenase/nucleotide sugar dehydrogenase	WP_079323636.:	28708..30147	Amino sugar and nucleotide sugar r	COG1004
197.1_WP_079323636.:	CUST_4081_PI442190803	HOT208_60m_813G15	197.1_WP_079323636.:	TTGGGAAGATGTCTCTAGGGTTATGAGATGTCCTGCTTGGATATTTGATACTCGTTCAAT	ugd	UDP-glucose 6-dehydrogenase/nucleotide sugar dehydrogenase	WP_079323636.:	28708..30147	Amino sugar and nucleotide sugar r	COG1004
197.1_WP_079323636.:	CUST_4082_PI442190803	HOT208_60m_813G15	197.1_WP_079323636.:	AATGACACAAGAGAGTCAGCAGCTATACAAATATGTCAAAATTTATTAGAGGAAGATGCA	ugd	UDP-glucose 6-dehydrogenase/nucleotide sugar dehydrogenase	WP_079323636.:	28708..30147	Amino sugar and nucleotide sugar r	COG1004
021.1_WP_079321891.:	CUST_4180_PI442190803	HOT208_60m_813G15	021.1_WP_079321891.:	GTGATTCGGTTCTTGAGGACACTCTAGTTATGGGTGCCGATTTCTTTGAATCTCCTGAAG	glgC	Glucose-1-phosphate adenylyltransferase	WP_079321891.:	1071..2366	Glycogen Metabolism	COG0448
021.1_WP_079321891.:	CUST_4181_PI442190803	HOT208_60m_813G15	021.1_WP_079321891.:	GATTGGAGCATTCTTTGAGTCAAACCTAGCACTCACTGAGCAACCAAAACCTCCATTTAG	glgC	Glucose-1-phosphate adenylyltransferase	WP_079321891.:	1071..2366	Glycogen Metabolism	COG0448
021.1_WP_079321891.:	CUST_4182_PI442190803	HOT208_60m_813G15	021.1_WP_079321891.:	TGAATTAAGAAAAGGAGGCGGAACGCCTCTTGGAGTAGGTGAAGGAACTACTGTAAAAAG	glgC	Glucose-1-phosphate adenylyltransferase	WP_079321891.:	1071..2366	Glycogen Metabolism	COG0448
021.1_WP_079321891.:	CUST_4183_PI442190803	HOT208_60m_813G15	021.1_WP_079321891.:	AGTGAAAAACCCACTGGTGAGAAGTTGAAAGCGATGGCAGTAGATACTTCAAAATTTGGA	glgC	Glucose-1-phosphate adenylyltransferase	WP_079321891.:	1071..2366	Glycogen Metabolism	COG0448
021.1_WP_079321891.:	CUST_4184_PI442190803	HOT208_60m_813G15	021.1_WP_079321891.:	TGAGTCAGCTGCCGAAAAACCTTACTTAGCCTCTATGGGTATTTATGTTTTTAGCAGAAA	glgC	Glucose-1-phosphate adenylyltransferase	WP_079321891.:	1071..2366	Glycogen Metabolism	COG0448
021.1_WP_079321891.:	CUST_4185_PI442190803	HOT208_60m_813G15	021.1_WP_079321891.:	TTTCCTGATTATACTGATTTTGGTAAGGACATTATTCCGGAAGCCCTTAATAGAGGCGAT	glgC	Glucose-1-phosphate adenylyltransferase	WP_079321891.:	1071..2366	Glycogen Metabolism	COG0448
021.1_WP_079321893.:	CUST_4186_PI442190803	HOT208_60m_813G15	021.1_WP_079321893.:	AGAACCAATAGACTTCCTCAGAATCTTGTTCAGGCAATGAGAGACTGTTTTGGCTCTCAT	edd	phosphogluconate dehydrogenase (NADP(+)-dependent, decarboxyla	WP_079321893.:	2461..3879	Pentose Phosphate/Entner-Dudorof	COG2084
021.1_WP_079321893.:	CUST_4187_PI442190803	HOT208_60m_813G15	021.1_WP_079321893.:	AGCTAAAGTAGTTTCTTTATCTACAAAGGCAGGTATACCAGTCCCATGTTTATCAAGTAC	edd	phosphogluconate dehydrogenase (NADP(+)-dependent, decarboxyla	WP_079321893.:	2461..3879	Pentose Phosphate/Entner-Dudorof	COG2084

021.1_WP_079321893.:	CUST_4188_PI442190803	HOT208_60m_813G15	021.1_WP_079321893.:	TGATTTGAATATGCCGTCAATTGCTCAAATATGGAAAGGTGGTTGCATAATTAGATCAAA	edd	phosphogluconate dehydrogenase (NADP(+)-dependent, decarboxyla	WP_079321893.:	2461..3879	Pentose Phosphate/Entner-Dudorof	COG2084
021.1_WP_079321893.:	CUST_4189_PI442190803	HOT208_60m_813G15	021.1_WP_079321893.:	AGTCCTTGCCACAATTGCTAGCTATGCCCAAGGTATGGATATTTTAAGAGAAGCATCTGA	edd	phosphogluconate dehydrogenase (NADP(+)-dependent, decarboxyla	WP_079321893.:	2461..3879	Pentose Phosphate/Entner-Dudorof	COG2084
021.1_WP_079321893.:	CUST_4190_PI442190803	HOT208_60m_813G15	021.1_WP_079321893.:	ATAAAGCTGGCCAGAAAGGTACAGGGTTATGGACTGTAGTAAGTGCTTTAGAATTGGGTG	edd	phosphogluconate dehydrogenase (NADP(+)-dependent, decarboxyla	WP_079321893.:	2461..3879	Pentose Phosphate/Entner-Dudorof	COG2084
021.1_WP_079321893.:	CUST_4191_PI442190803	HOT208_60m_813G15	021.1_WP_079321893.:	TACTCGCAGAAGCTTATGACCTTATGAAGAGAGTCAAGGGTATGAATGGACAGCAGATGT	edd	phosphogluconate dehydrogenase (NADP(+)-dependent, decarboxyla	WP_079321893.:	2461..3879	Pentose Phosphate/Entner-Dudorof	COG2084
021.1_WP_011376344.:	CUST_4250_PI442190803	HOT208_60m_813G15	021.1_WP_011376344.:	GCAGCATTTGCTGATAAAGCAAACTTTGACCCAAGACATTTCAACAAGCCTGCTAGAAAA	cbbA	Fructose-1,6-bisphosphate aldolase class II	WP_011376344.:	11143..12216	Glycolysis/Gluconeogenesis	COG0191
021.1_WP_011376344.:	CUST_4251_PI442190803	HOT208_60m_813G15	021.1_WP_011376344.:	AGCAAGTTTGTCTTGATAGATACAAGCAGTTCTGGTGCGAAGGTCAAGCAAGTAAGATCA	cbbA	Fructose-1,6-bisphosphate aldolase class II	WP_011376344.:	11143..12216	Glycolysis/Gluconeogenesis	COG0191
021.1_WP_011376344.:	CUST_4253_PI442190803	HOT208_60m_813G15	021.1_WP_011376344.:	AGGAGAAGTTCTCGCAATAAGCAGAATTGCCGAAATTCATAAAGCACTTCCAAATACCCA	cbbA	Fructose-1,6-bisphosphate aldolase class II	WP_011376344.:	11143..12216	Glycolysis/Gluconeogenesis	COG0191
021.1_WP_079321904.:	CUST_4254_PI442190803	HOT208_60m_813G15	021.1_WP_079321904.:	TCATGCTTAAAGGCCTGGAAAGGAAGCGACATTGAAGAAGGTCAAAAAGCATTAATAGCA	fda	Fructose-1,6-bisphosphate aldolase class I	WP_079321904.:	12365..13432	Glycolysis/Gluconeogenesis	COG0191
021.1_WP_079321904.:	CUST_4255_PI442190803	HOT208_60m_813G15	021.1_WP_079321904.:	GCTGACGTTCAAGAAGAGGTAATAAAGCAGGTTTATATTGCCTGCCAAGCAAATGGAGTT	fda	Fructose-1,6-bisphosphate aldolase class I	WP_079321904.:	12365..13432	Glycolysis/Gluconeogenesis	COG0191
021.1_WP_079321904.:	CUST_4256_PI442190803	HOT208_60m_813G15	021.1_WP_079321904.:	TCAGAACTATGGAAAGATGCGTTCCTGCATCTGTTCCTGGAATAGTTTTCTTGTCAGGAG	fda	Fructose-1,6-bisphosphate aldolase class I	WP_079321904.:	12365..13432	Glycolysis/Gluconeogenesis	COG0191
021.1_WP_079321904.:	CUST_4257_PI442190803	HOT208_60m_813G15	021.1_WP_079321904.:	AAGGTGCCTATATAGCAGGATCTCAACCTTCATCTGATGAGCAATTGTTTGTAGCTGGTT	fda	Fructose-1,6-bisphosphate aldolase class I	WP_079321904.:	12365..13432	Glycolysis/Gluconeogenesis	COG0191
021.1_WP_079321904.:	CUST_4258_PI442190803	HOT208_60m_813G15	021.1_WP_079321904.:	CTAGATGGGCTAGTTGAGAGAGCAGCAAAATATTATGAGCAAGGTGCCAGATTTGCAAAG	fda	Fructose-1,6-bisphosphate aldolase class I	WP_079321904.:	12365..13432	Glycolysis/Gluconeogenesis	COG0191
021.1_WP_079321904.:	CUST_4259_PI442190803	HOT208_60m_813G15	021.1_WP_079321904.:	ACCCTCTATGACTGTTAATGGAGCAGATTGTCTAACAAAGGCTGATCCTATGAAAGTGGC	fda	Fructose-1,6-bisphosphate aldolase class I	WP_079321904.:	12365..13432	Glycolysis/Gluconeogenesis	COG0191
021.1_WP_079321909.:	CUST_4276_PI442190803	HOT208_60m_813G15	021.1_WP_079321909.:	ATTCCTGAGGGAACAGACGTACTTTTTTATGAAGGTTTGCATGGCGGAGTAGAAGGTAAT	prk	Phosphoribulokinase	WP_079321909.:	16050..16946	Carbon Fixation	COG3954
021.1_WP_079321909.:	CUST_4277_PI442190803	HOT208_60m_813G15	021.1_WP_079321909.:	CTATGGCAGACGCTCTTTCAAAAGGCGAAAATTTCTCTCATTTTGGTCCAGAGGCTAATC	prk	Phosphoribulokinase	WP_079321909.:	16050..16946	Carbon Fixation	COG3954
021.1_WP_079321909.:	CUST_4278_PI442190803	HOT208_60m_813G15	021.1_WP_079321909.:	ACTCCTGACGAGAGCTTTGTAATCATACATTTCAGAAAAGGAGCAAGAGAGAAATGGGGG	prk	Phosphoribulokinase	WP_079321909.:	16050..16946	Carbon Fixation	COG3954
021.1_WP_079321909.:	CUST_4279_PI442190803	HOT208_60m_813G15	021.1_WP_079321909.:	TGCCCCCAATTCAGCAAAACCGATATTAATTTCCAAAGAATTCCCACAATTGACACTTCT	prk	Phosphoribulokinase	WP_079321909.:	16050..16946	Carbon Fixation	COG3954
021.1_WP_079321909.:	CUST_4280_PI442190803	HOT208_60m_813G15	021.1_WP_079321909.:	AGTCTTGAAGAAGCAGAAGAACATAATACAAGACTTGGGACATCATTAGAACCTGGGCAA	prk	Phosphoribulokinase	WP_079321909.:	16050..16946	Carbon Fixation	COG3954
021.1_WP_079321909.:	CUST_4281_PI442190803	HOT208_60m_813G15	021.1_WP_079321909.:	TGTCAAGTCCAACAAGTATCGTTGTAAATGGAGGAAAAATGGGTTTCGCAATGGAACTAA	prk	Phosphoribulokinase	WP_079321909.:	16050..16946	Carbon Fixation	COG3954
202.1_WP_079323652.:	CUST_4323_PI442190803	HOT208_60m_813G15	202.1_WP_079323652.:	CGCGAATCAATGGCGGAAGATTAACTAGTCAAAATATGTTCCCATTGGAGGCTGTTGAAT	cyoC	heme-copper oxidase subunit III	WP_079323652.:	2053..2655	Respiration	COG1845
202.1_WP_079323652.:	CUST_4324_PI442190803	HOT208_60m_813G15	202.1_WP_079323652.:	ATGTTTGGTCTCATAACTTTCCTAATTGCGGACGGAATGACTTTTGCTGGATTCTTTGCT	cyoC	heme-copper oxidase subunit III	WP_079323652.:	2053..2655	Respiration	COG1845
202.1_WP_079323652.:	CUST_4325_PI442190803	HOT208_60m_813G15	202.1_WP_079323652.:	ACACTCAATACAATTTTGTTACTTGTTAGTAGTGCAACTTTCCATAAAGCAGGCAAAGCA	cyoC	heme-copper oxidase subunit III	WP_079323652.:	2053..2655	Respiration	COG1845
202.1_WP_079323652.:	CUST_4326_PI442190803	HOT208_60m_813G15	202.1_WP_079323652.:	TGCAAGTACTTTTTATGCTCTTACTGGTTTTCATGGATTACATGTCACTTTAGGCACTTT	cyoC	heme-copper oxidase subunit III	WP_079323652.:	2053..2655	Respiration	COG1845
202.1_WP_079323652.:	CUST_4327_PI442190803	HOT208_60m_813G15	202.1_WP_079323652.:	TATAAAGCAGTAAATCCATTACCTGATGGTGCTATTTATGAATTAGAACTACCAATACCT	cyoC	heme-copper oxidase subunit III	WP_079323652.:	2053..2655	Respiration	COG1845
202.1_WP_079323652.:	CUST_4328_PI442190803	HOT208_60m_813G15	202.1_WP_079323652.:	AGCTCAAAAGAAATTCAAAAAAATAATTCTGAAGTTAATGAAACACATGAAGACTTCAGA	cyoC	heme-copper oxidase subunit III	WP_079323652.:	2053..2655	Respiration	COG1845
202.1_WP_079323653.:	CUST_4329_PI442190803	HOT208_60m_813G15	202.1_WP_079323653.:	TGTTTTTGGAGGTATTACAGGAGTTGCTTTGGCACAGGTACCTTTCGATATTCACGTACA	cyoB/coxA	Cytochrome c oxidase subunit I	WP_079323653.:	2661..4286	Respiration	COG0843
202.1_WP_079323653.:	CUST_4330_PI442190803	HOT208_60m_813G15	202.1_WP_079323653.:	TATTCCTTAGTGTGAGAAATGGGAAAGATGCTGGAGATAACCCTTGGAATGCCCTTACAC	cyoB/coxA	Cytochrome c oxidase subunit I	WP_079323653.:	2661..4286	Respiration	COG0843
202.1_WP_079323653.:	CUST_4331_PI442190803	HOT208_60m_813G15	202.1_WP_079323653.:	GGCTTGGTTTAAATGGAATGCCGAGAAGAGTTGCAGAATATGATCCTCAATTCCAGTTCG	cyoB/coxA	Cytochrome c oxidase subunit I	WP_079323653.:	2661..4286	Respiration	COG0843
202.1_WP_079323653.:	CUST_4332_PI442190803	HOT208_60m_813G15	202.1_WP_079323653.:	AATGGGAATAGTAGTTTTGGGTTTAGTTGTTTGGGCGCATCACATGTTTACGAGTGGAAC	cyoB/coxA	Cytochrome c oxidase subunit I	WP_079323653.:	2661..4286	Respiration	COG0843
202.1_WP_079323653.:	CUST_4333_PI442190803	HOT208_60m_813G15	202.1_WP_079323653.:	TAACATCTTCCCCTCCTCCAGTTGAAAATTGGGAAGGAGAAGCTCCATTAGTTGAAGAAC	cyoB/coxA	Cytochrome c oxidase subunit I	WP_079323653.:	2661..4286	Respiration	COG0843
202.1_WP_079323653.:	CUST_4334_PI442190803	HOT208_60m_813G15	202.1_WP_079323653.:	TGCCACGGCATTTATTGCAGTTCCAACAGGTATAAAATTTTTCAATTGGGTTGCAACACT	cyoB/coxA	Cytochrome c oxidase subunit I	WP_079323653.:	2661..4286	Respiration	COG0843
202.1_WP_079323654.:	CUST_4335_PI442190803	HOT208_60m_813G15	202.1_WP_079323654.:	ATCATGGAGGGATGCGAGCCTCGATAATTGTTGAAGAAGAATCTGATTACAACGAATGGT	ctaC/coxB	Cytochrome c oxidase subunit II	WP_079323654.:	4283..5086	Respiration	COG1622
202.1_WP_079323654.:	CUST_4336_PI442190803	HOT208_60m_813G15	202.1_WP_079323654.:	TCTTTTTGGATTGGTTTTAATGTAAATTTGCTCCCAGCGGAAGCAAGTATTAATGCACCA	ctaC/coxB	Cytochrome c oxidase subunit II	WP_079323654.:	4283..5086	Respiration	COG1622
202.1_WP_079323654.:	CUST_4337_PI442190803	HOT208_60m_813G15	202.1_WP_079323654.:	CATGCTTTTTGGGTACCAGAGTTCAGAATTAAACAGGATATTATTCCTGGACAACCGACT	ctaC/coxB	Cytochrome c oxidase subunit II	WP_079323654.:	4283..5086	Respiration	COG1622
202.1_WP_079323654.:	CUST_4338_PI442190803	HOT208_60m_813G15	202.1_WP_079323654.:	TCCCAAAGGCAATTTCATATCAGGAGAACTACACGTTCCTGTTGATCAAAAAGTATCAAT	ctaC/coxB	Cytochrome c oxidase subunit II	WP_079323654.:	4283..5086	Respiration	COG1622
202.1_WP_079323654.:	CUST_4339_PI442190803	HOT208_60m_813G15	202.1_WP_079323654.:	GGCGATGGCATAGCTTTAGAGGGAAATTTAAGCCTAGAAATTGTATGGACAATTATACCT	ctaC/coxB	Cytochrome c oxidase subunit II	WP_079323654.:	4283..5086	Respiration	COG1622
202.1_WP_079323654.:	CUST_4340_PI442190803	HOT208_60m_813G15	202.1_WP_079323654.:	ACAACATCTACGATCGAATGGGAGGGATGAAAGAACTAAATCATAACCATGAAATGATGA	ctaC/coxB	Cytochrome c oxidase subunit II	WP_079323654.:	4283..5086	Respiration	COG1622
207.1_WP_079323670.:	CUST_4441_PI442190803	HOT208_60m_813G15	207.1_WP_079323670.:	TGGTTTAGATCTACTGTTTGATGAATTAACTGAAACTGAATATAGTGGAGATTAA	kaiB	Circadian clock protein KaiB	WP_079323670.:	1992..2306	Circadian Rhythm	COG4251
207.1_WP_079323670.:	CUST_4442_PI442190803	HOT208_60m_813G15	207.1_WP_079323670.:	CCGCCACCAGTGAGAAAAATAATAGGTGATCTTTCAGATAGAGAGAAAGTTTTAATTGGT	kaiB	Circadian clock protein KaiB	WP_079323670.:	1992..2306	Circadian Rhythm	COG4251
207.1_WP_079323670.:	CUST_4443_PI442190803	HOT208_60m_813G15	207.1_WP_079323670.:	CAAGCAACCACAACTTGCAGAGGAAGATAAGATTTTAGCCACCCCAACCTTAGCTAAAAT	kaiB	Circadian clock protein KaiB	WP_079323670.:	1992..2306	Circadian Rhythm	COG4251
207.1_WP_079323670.:	CUST_4444_PI442190803	HOT208_60m_813G15	207.1_WP_079323670.:	TGAGGGCTTTAAATACTTTAAAAGAGATTTTAGAAAATGAATTTAAAGGAGTTTATGCTT	kaiB	Circadian clock protein KaiB	WP_079323670.:	1992..2306	Circadian Rhythm	COG4251
207.1_WP_079323670.:	CUST_4445_PI442190803	HOT208_60m_813G15	207.1_WP_079323670.:	TGGCAGCAAGAAAAACATACATTTTAAAACTATATGTCGCTGGAAATACCCCTAATTCAA	kaiB	Circadian clock protein KaiB	WP_079323670.:	1992..2306	Circadian Rhythm	COG4251
022.1_WP_079321949.:	CUST_4652_PI442190803	HOT208_60m_813G15	022.1_WP_079321949.:	TTGAGTTACCATATCGCAGCTCATAGAGGCCTTGACGTTGATCAACCAAGAAATTTAGCT	glmS	Glutamine--fructose-6-phosphatetransaminase(isomerizing)	WP_079321949.:	16292..18187	Amino Acid metabolism	COG0449
022.1_WP_079321949.:	CUST_4653_PI442190803	HOT208_60m_813G15	022.1_WP_079321949.:	TCGGAATTGCTCCTGAATGTAATGGAACTGAAATCTTTGATTATTTAATGAAAGTGCCTT	glmS	Glutamine--fructose-6-phosphatetransaminase(isomerizing)	WP_079321949.:	16292..18187	Amino Acid metabolism	COG0449
022.1_WP_079321949.:	CUST_4654_PI442190803	HOT208_60m_813G15	022.1_WP_079321949.:	ATTGCCTCTCCCGGTGAGGTTTTTGATAAAGTTATCAGTAATGCTCAAGAAGCAAAAGCT	glmS	Glutamine--fructose-6-phosphatetransaminase(isomerizing)	WP_079321949.:	16292..18187	Amino Acid metabolism	COG0449
022.1_WP_079321949.:	CUST_4655_PI442190803	HOT208_60m_813G15	022.1_WP_079321949.:	AAGTTACATTCATGCAGCTGGATATCCTGCTGGTGAAATGAAACATGGTCCAATAGCTTT	glmS	Glutamine--fructose-6-phosphatetransaminase(isomerizing)	WP_079321949.:	16292..18187	Amino Acid metabolism	COG0449
022.1_WP_079321949.:	CUST_4656_PI442190803	HOT208_60m_813G15	022.1_WP_079321949.:	TGGAAGGCAGGTTGCAAATATAATTGATATCTGTGCAGGAATAGAAGTTGGAGTTGCAGC	glmS	Glutamine--fructose-6-phosphatetransaminase(isomerizing)	WP_079321949.:	16292..18187	Amino Acid metabolism	COG0449
022.1_WP_079321949.:	CUST_4657_PI442190803	HOT208_60m_813G15	022.1_WP_079321949.:	ACTATTGGAGTCACTCAATCTGGAGAAACTGCTGATACAATTGCAGCTATCGATATGGAA	glmS	Glutamine--fructose-6-phosphatetransaminase(isomerizing)	WP_079321949.:	16292..18187	Amino Acid metabolism	COG0449
229.1_WP_079323719.:	CUST_5091_PI442190803	HOT208_60m_813G15	229.1_WP_079323719.:	ATGCAACTTAACTTTGCTTTTTAACTTCTGCCAAGCGGTAACTTGTGCCAATGCAAAGAT	tal	Transaldolase	WP_079323719.:	24531..25532	Pentose Phosphate	COG0176
229.1_WP_079323719.:	CUST_5092_PI442190803	HOT208_60m_813G15	229.1_WP_079323719.:	TGAAAATTATTTCAGGGCGCGTATCTACAGAAGTTGATGCAAGATTGAGCTTTGACACCG	tal	Transaldolase	WP_079323719.:	24531..25532	Pentose Phosphate	COG0176
229.1_WP_079323719.:	CUST_5093_PI442190803	HOT208_60m_813G15	229.1_WP_079323719.:	ATTAAGATTGCTGCAACTTGGGAGGGTATTAAGGCAGCTGAAATTTTGGAAAAAGAGGGT	tal	Transaldolase	WP_079323719.:	24531..25532	Pentose Phosphate	COG0176
229.1_WP_079323719.:	CUST_5094_PI442190803	HOT208_60m_813G15	229.1_WP_079323719.:	AGATTGGCATAAAGCAAAAACTGGTAAAACTAGTTTTGTTGGTGCTGAAGATCCTGGTGT	tal	Transaldolase	WP_079323719.:	24531..25532	Pentose Phosphate	COG0176
229.1_WP_079323719.:	CUST_5095_PI442190803	HOT208_60m_813G15	229.1_WP_079323719.:	AGCAGGTTGCGATCTTTTAACAATCGCACCAAAATTTCTTGAGGAACTGAAAAAAGAAAA	tal	Transaldolase	WP_079323719.:	24531..25532	Pentose Phosphate	COG0176
229.1_WP_079323719.:	CUST_5096_PI442190803	HOT208_60m_813G15	229.1_WP_079323719.:	TGGCAAGTGAAAAGCTTAGTGAAGGTATTACTGGATTCAGTAAGGCTATAGAAGAATTGG	tal	Transaldolase	WP_079323719.:	24531..25532	Pentose Phosphate	COG0176
229.1_WP_079323774.:	CUST_5450_PI442190803	HOT208_60m_813G15	229.1_WP_079323774.:	TGTCCAATACAGATTCATTATCAGGCAAAGTTGCTTTAATCACGGGAGCTAGCAGAGGAA	gdh	3-oxoacyl-acyl-carrier-protein reductase	WP_079323774.:	81705..82454	Entner-Dudorff	COG1028
229.1_WP_079323774.:	CUST_5451_PI442190803	HOT208_60m_813G15	229.1_WP_079323774.:	TCTGCAGCTAAAGCTGGAGTTATTGGGTTTACAAAAACTTGCGCTAAAGAATTTGCCTCA	gdh	3-oxoacyl-acyl-carrier-protein reductase	WP_079323774.:	81705..82454	Entner-Dudorff	COG1028
229.1_WP_079323774.:	CUST_5452_PI442190803	HOT208_60m_813G15	229.1_WP_079323774.:	TCAAGTGATGCAGGAAGTTACATTACAGGGCAAACAATTAGTATAGACGGGGGTATGAGT	gdh	3-oxoacyl-acyl-carrier-protein reductase	WP_079323774.:	81705..82454	Entner-Dudorff	COG1028
229.1_WP_079323774.:	CUST_5453_PI442190803	HOT208_60m_813G15	229.1_WP_079323774.:	TGCTGGAATTACTAGAGATGGACTATTGATGAGAATGAAATCGGAACAATGGGATGATGT	gdh	3-oxoacyl-acyl-carrier-protein reductase	WP_079323774.:	81705..82454	Entner-Dudorff	COG1028
229.1_WP_079323774.:	CUST_5454_PI442190803	HOT208_60m_813G15	229.1_WP_079323774.:	AGTGGTAGCATCGTAAATATTTCATCTGTTGTTGGAATAATTGGTAATCCTGGTCAAGCA	gdh	3-oxoacyl-acyl-carrier-protein reductase	WP_079323774.:	81705..82454	Entner-Dudorff	COG1028
229.1_WP_079323774.:	CUST_5455_PI442190803	HOT208_60m_813G15	229.1_WP_079323774.:	AAGCCGCTTAGGAGCAGAAGTTTTCATTAATTACTCTTCTTCTGATGAAAAAGCTGAAGA	gdh	3-oxoacyl-acyl-carrier-protein reductase	WP_079323774.:	81705..82454	Entner-Dudorff	COG1028
033.1_WP_079322057.:	CUST_5804_PI442190803	HOT208_60m_813G15	033.1_WP_079322057.:	TTTTGGTAGGGTTGAAACTCCTGGAGTTGTAAAACTTCCACTTGAAGCTGCACTATCAGA		polysaccharide biosynthesis/export family protein	WP_079322057.:	2884..4134	Other sugar transporters	COG1596
033.1_WP_079322057.:	CUST_5805_PI442190803	HOT208_60m_813G15	033.1_WP_079322057.:	GGAAAAACTACAGGCGTGCTTACAGAATTTACAGCTCCATTCGTTGGTATTTATTCAACT		polysaccharide biosynthesis/export family protein	WP_079322057.:	2884..4134	Other sugar transporters	COG1596
033.1_WP_079322057.:	CUST_5806_PI442190803	HOT208_60m_813G15	033.1_WP_079322057.:	GCGAAAGATGGGACAGTAATAAAGAAAAACATATCATATTCAGCAAGTGCTAAAAGAGGC		polysaccharide biosynthesis/export family protein	WP_079322057.:	2884..4134	Other sugar transporters	COG1596
033.1_WP_079322057.:	CUST_5807_PI442190803	HOT208_60m_813G15	033.1_WP_079322057.:	AGAGATATACCTCTTGGAAAAGGTGGAGGCAAAAAAAGAGCAATTATTGATTTTAATGCT		polysaccharide biosynthesis/export family protein	WP_079322057.:	2884..4134	Other sugar transporters	COG1596
033.1_WP_079322057.:	CUST_5808_PI442190803	HOT208_60m_813G15	033.1_WP_079322057.:	ACTACCATTTCCGATGTTATAAGGAATGCTGGTGGTATAACCTCTAAAACTGATTTATCT		polysaccharide biosynthesis/export family protein	WP_079322057.:	2884..4134	Other sugar transporters	COG1596
033.1_WP_079322057.:	CUST_5809_PI442190803	HOT208_60m_813G15	033.1_WP_079322057.:	ACGAATCAGATCCCACTAATGATATTCGTATATTTGATGGCGATACTTTATTCTTTCCAA		polysaccharide biosynthesis/export family protein	WP_079322057.:	2884..4134	Other sugar transporters	COG1596
091.1_WP_079322264.:	CUST_6680_PI442190803	HOT208_60m_813G15	091.1_WP_079322264.:	TGATCGAAGTTGAGAGGATTTGGTTAGGTGCTTATGCCTCTTTACTTGGAGATGTTGCTC	glk	Glucokinase	WP_079322264.:	8731..9765	Glycolysis	COG0837
091.1_WP_079322264.:	CUST_6681_PI442190803	HOT208_60m_813G15	091.1_WP_079322264.:	AAATGGATCTCATTCTGAAGGTGCTAATAATGAATTGCATGCCATTGTAGGAGCGGGAAC	glk	Glucokinase	WP_079322264.:	8731..9765	Glycolysis	COG0837
091.1_WP_079322264.:	CUST_6682_PI442190803	HOT208_60m_813G15	091.1_WP_079322264.:	GGCATAATTTCAGGCGGAAAGGTAAAAGTTTTAGCCAGTGAAGGTGGTCATGTTGAGTAC	glk	Glucokinase	WP_079322264.:	8731..9765	Glycolysis	COG0837
091.1_WP_079322264.:	CUST_6683_PI442190803	HOT208_60m_813G15	091.1_WP_079322264.:	TGTGCTTTGGAGGGTTATGGATTTCTGGAGGGACCGCATCAAAACATTTCAAAAACTTTA	glk	Glucokinase	WP_079322264.:	8731..9765	Glycolysis	COG0837
091.1_WP_079322264.:	CUST_6684_PI442190803	HOT208_60m_813G15	091.1_WP_079322264.:	GTTATTAGCGGCACTGGTTTATCAAGAATTGCTGAATGGAGACTAAGCAAACCTGATGCC	glk	Glucokinase	WP_079322264.:	8731..9765	Glycolysis	COG0837
091.1_WP_079322264.:	CUST_6685_PI442190803	HOT208_60m_813G15	091.1_WP_079322264.:	TGATGCGGCGAGAAAAGAACTGCCTGAAAAAAATTTGCAATCTTTCTAAAGAAGGGGATCA	glk	Glucokinase	WP_079322264.:	8731..9765	Glycolysis	COG0837
092.1_WP_079322384.:	CUST_7043_PI442190803	HOT208_60m_813G15	092.1_WP_079322384.:	ACTAATGTTGGTTACAGCCATAGTGCCAAAAGTTGGTTATGAAAAAGCAGCCGAAATTGC	fumC	class II fumarate hydratase	WP_079322384.:	7844..9229	Krebs cycle and related genes	COG0114
092.1_WP_079322384.:	CUST_7044_PI442190803	HOT208_60m_813G15	092.1_WP_079322384.:	TGGTTTGCACTCAGATAATGGGTCTTGAATATGCAGTTTCAATGGCAAATTCTAGCGGAA	fumC	class II fumarate hydratase	WP_079322384.:	7844..9229	Krebs cycle and related genes	COG0114
092.1_WP_079322384.:	CUST_7045_PI442190803	HOT208_60m_813G15	092.1_WP_079322384.:	CTCAAAATGAACCTGGAAGTTCTATCATGCCGGGTAAAGTGAATCCAACTCAATGCGAAG	fumC	class II fumarate hydratase	WP_079322384.:	7844..9229	Krebs cycle and related genes	COG0114
092.1_WP_079322384.:	CUST_7046_PI442190803	HOT208_60m_813G15	092.1_WP_079322384.:	TGGCCTCTCATGATCGTCTAGCTCAAGTAATGAGTCAGATAAAAATATTAGCAGGTGCAT	fumC	class II fumarate hydratase	WP_079322384.:	7844..9229	Krebs cycle and related genes	COG0114
092.1_WP_079322384.:	CUST_7047_PI442190803	HOT208_60m_813G15	092.1_WP_079322384.:	ACCTTTGGGAGGAACTGCAGTTGGAACAGGTATTAATTGTCCAAAAGGATTTTGTGAAGA	fumC	class II fumarate hydratase	WP_079322384.:	7844..9229	Krebs cycle and related genes	COG0114
092.1_WP_079322384.:	CUST_7048_PI442190803	HOT208_60m_813G15	092.1_WP_079322384.:	TCCCCTTACTTTTGGGCAAGAAATATCAGGATGGTCAGAGCAACTTAAAGATGCTGAGAA	fumC	class II fumarate hydratase	WP_079322384.:	7844..9229	Krebs cycle and related genes	COG0114
093.1_WP_079322533.:	CUST_7520_PI442190803	HOT208_60m_813G15	093.1_WP_079322533.:	ATGTGCCAGAATTAATAAGAGGTAAAACGGGAAGAGTTTATGGGCATCTCCAAGCTCTGT	argH	Argininosuccinate lyase	WP_079322533.:	70505..71884	Amino Acid metabolism	COG0165
093.1_WP_079322533.:	CUST_7521_PI442190803	HOT208_60m_813G15	093.1_WP_079322533.:	GTGAAGGCGGAACAGGTTTTACCCAAGTAGAAAAGGAGGTAAACAACTGGCAGAAAAAAT	argH	Argininosuccinate lyase	WP_079322533.:	70505..71884	Amino Acid metabolism	COG0165
093.1_WP_079322533.:	CUST_7522_PI442190803	HOT208_60m_813G15	093.1_WP_079322533.:	CAGCATTAGCTGGCACAAAAATAAAGATAGACCGGAACTTTACAGCCGCAGAATTAGGTT	argH	Argininosuccinate lyase	WP_079322533.:	70505..71884	Amino Acid metabolism	COG0165
093.1_WP_079322533.:	CUST_7523_PI442190803	HOT208_60m_813G15	093.1_WP_079322533.:	TAATGCCACTGATTTGGCAGATTACCTAGTTGGCAAACAGATCCCTTTTAGGACTGCTTA	argH	Argininosuccinate lyase	WP_079322533.:	70505..71884	Amino Acid metabolism	COG0165
093.1_WP_079322533.:	CUST_7524_PI442190803	HOT208_60m_813G15	093.1_WP_079322533.:	TGCAAAATTAACAGACAAATGTGCTACTGGGAGTAGCTTAATGCCGCAGAAAAAAAATCC	argH	Argininosuccinate lyase	WP_079322533.:	70505..71884	Amino Acid metabolism	COG0165
093.1_WP_079322533.:	CUST_7525_PI442190803	HOT208_60m_813G15	093.1_WP_079322533.:	CATCCCGATTTTGAAGAGGATGTTTTTGTCGATCTTGAACCTTTCAATGTAGTTAAGTCA	argH	Argininosuccinate lyase	WP_079322533.:	70505..71884	Amino Acid metabolism	COG0165
105.1_14	CUST_61_PI442190799	HOT208_60m_810P02	105.1_14	TGTAAAAAAATTATTTGGAACAGTAACAAGCAAAAAAAAATTGGAATACTAGGCTTCGCTT	ugd	UDP-glucose 6-dehydrogenase/nucleotide sugar dehydrogenase	frame=2	141..510	Amino sugar and nucleotide sugar r	COG1004
105.1_14	CUST_62_PI442190799	HOT208_60m_810P02	105.1_14	ACGGTTTAAAAGAGGTGGCAAATTACTGGGACGAAGTTCTAAAGATAAATCGATGGCAAC	ugd	UDP-glucose 6-dehydrogenase/nucleotide sugar dehydrogenase	frame=2	141..510	Amino sugar and nucleotide sugar r	COG1004
105.1_14	CUST_63_PI442190799	HOT208_60m_810P02	105.1_14	GGATTTGGAGGCAGCTGTTTTAAGAAAGATATTTTAAATTTGGTT	ugd	UDP-glucose 6-dehydrogenase/nucleotide sugar dehydrogenase	frame=2	141..510	Amino sugar and nucleotide sugar r	COG1004
105.1_14	CUST_64_PI442190799	HOT208_60m_810P02	105.1_14	GGTATGGACTATAGGATTGGTAGAAACTTTCTAAAATCAGGCCCTGGATTTGGAGGCAGC	ugd	UDP-glucose 6-dehydrogenase/nucleotide sugar dehydrogenase	frame=2	141..510	Amino sugar and nucleotide sugar r	COG1004
105.1_14	CUST_65_PI442190799	HOT208_60m_810P02	105.1_14	AGCTCAAAGGGTGAGTTCAATTAACTCTATCTCAGCATTATGTGAAAAAACTGGTGCAGA	ugd	UDP-glucose 6-dehydrogenase/nucleotide sugar dehydrogenase	frame=2	141..510	Amino sugar and nucleotide sugar r	COG1004
107.1_17	CUST_77_PI442190799	HOT208_60m_810P02	107.1_17	ATTAGCTGATGCAATGCTCTCTGACCCAACATTATTTACTCGAAGTGATGAGGTAGAGGC	opcA	Glucose-6-P dehydrogenase subunit opcA		<1..410	Pentose Phosphate	COG3429
107.1_17	CUST_78_PI442190799	HOT208_60m_810P02	107.1_17	GACCTGTTGAGATGGAATTTTCTTATGATGAATCATTTGGAGAACCCTCAGATGAAGGCT	opcA	Glucose-6-P dehydrogenase subunit opcA		<1..410	Pentose Phosphate	COG3429
107.1_17	CUST_79_PI442190799	HOT208_60m_810P02	107.1_17	TCAGTCAAATGAAGGTGCTACTTTCAAATTTGAGGTAAAATCTCCTGGTTCTGGAATGAA	opcA	Glucose-6-P dehydrogenase subunit opcA		<1..410	Pentose Phosphate	COG3429
107.1_17	CUST_80_PI442190799	HOT208_60m_810P02	107.1_17	TCCTTGGAAGTTACCTATTTATAATTATGAATCTATGACATGGGGACCTCCTGAGTCAGA	opcA	Glucose-6-P dehydrogenase subunit opcA		<1..410	Pentose Phosphate	COG3429
107.1_17	CUST_81_PI442190799	HOT208_60m_810P02	107.1_17	AAAGACGTTCCTGTTCATTTATTTGAATCAACAATAATAAATCCTGCCCCAAATCAACTT	opcA	Glucose-6-P dehydrogenase subunit opcA		<1..410	Pentose Phosphate	COG3429
107.1_17	CUST_82_PI442190799	HOT208_60m_810P02	107.1_17	TGATGAGGTAGAGGCAGCCTGGAAACTTTATACTCCATTAATAGA	opcA	Glucose-6-P dehydrogenase subunit opcA		<1..410	Pentose Phosphate	COG3429
110.1_WP_025945659.:	CUST_243_PI442190799	HOT208_60m_810P02	110.1_WP_025945659.:	TTTACCAATCAAAAGAACTATTTCAGGAGAAATAAAATTCAAAAAAACTGATAAAACTGA	minE	septum site-determining protein MinE	WP_025945659.:	22074..22409	Cell Division	COG0851
110.1_WP_025945659.:	CUST_244_PI442190799	HOT208_60m_810P02	110.1_WP_025945659.:	GATTTTGATGAGGTAGCAGTAAGTTTAGAGACTGAAGATAGAATGACTGCATTAGTTGCA	minE	septum site-determining protein MinE	WP_025945659.:	22074..22409	Cell Division	COG0851
110.1_WP_025945659.:	CUST_245_PI442190799	HOT208_60m_810P02	110.1_WP_025945659.:	AGTTCCTTAACAACTGATCTTTTAGATAAAATGAGAAAAGAGATTCTTGATGTTGTTGCT	minE	septum site-determining protein MinE	WP_025945659.:	22074..22409	Cell Division	COG0851
110.1_WP_025945659.:	CUST_246_PI442190799	HOT208_60m_810P02	110.1_WP_025945659.:	GGCAGAGAACCGGCTAGTGCAAATACAGCTAGAGAAAGATTACAACTTGTACTTGCTCAT	minE	septum site-determining protein MinE	WP_025945659.:	22074..22409	Cell Division	COG0851
110.1_WP_025945660.:	CUST_247_PI442190799	HOT208_60m_810P02	110.1_WP_025945660.:	AGCACTTGCTAACAGCGGAGCAACAACTGCTGTATTAGATGCTGATTTTGGTTTAAGAAA	minD	septum site-determining protein MinD	WP_025945660.:	22411..23226	Cell Division	COG2894
110.1_WP_025945660.:	CUST_248_PI442190799	HOT208_60m_810P02	110.1_WP_025945660.:	AGAAAATCGCATCATTTATACCGCCCAAGATGTCCTAGACAAGAATTGTCGTCTTGACCA	minD	septum site-determining protein MinD	WP_025945660.:	22411..23226	Cell Division	COG2894
110.1_WP_025945660.:	CUST_249_PI442190799	HOT208_60m_810P02	110.1_WP_025945660.:	AGATGGCTTTAAAAATGCTCTTGCAGCCTGCAAAGAAGCTATTGTTGTTACTAACCCAGA	minD	septum site-determining protein MinD	WP_025945660.:	22411..23226	Cell Division	COG2894
110.1_WP_025945660.:	CUST_250_PI442190799	HOT208_60m_810P02	110.1_WP_025945660.:	AAAGAACCTAATCTTGCTCTTCTACCTGCTGGAGATCCAAGGATGTTGGATTGGATGAAG	minD	septum site-determining protein MinD	WP_025945660.:	22411..23226	Cell Division	COG2894
110.1_WP_025945660.:	CUST_251_PI442190799	HOT208_60m_810P02	110.1_WP_025945660.:	ATTGTCCGCAGTGCGCGATGCGGATAGAGTAATAGGGATTCTTAATACTTCAGATATCGA	minD	septum site-determining protein MinD	WP_025945660.:	22411..23226	Cell Division	COG2894
110.1_WP_025945660.:	CUST_252_PI442190799	HOT208_60m_810P02	110.1_WP_025945660.:	TCGCCCTAACATGATGGCTAGTCAAGAAATGTTATCTATCGAAGATGTTCAAGGGATCCT	minD	septum site-determining protein MinD	WP_025945660.:	22411..23226	Cell Division	COG2894
110.1_WP_079336804.:	CUST_253_PI442190799	HOT208_60m_810P02	110.1_WP_079336804.:	AGATGTGATAGCTATTGGGCCAAAGGATAAGCCCAAAAATTGTTATCCAGAAATTGCGGT	minC	septum site-determining protein MinC	WP_079336804.:	23338..24000	Cell Division	COG0850
110.1_WP_079336804.:	CUST_254_PI442190799	HOT208_60m_810P02	110.1_WP_079336804.:	CTAGGAATCGCGTTCGCTGGGACAAGTGGAAATAAAAATGCCTCCATTTCATCACTTTAT	minC	septum site-determining protein MinC	WP_079336804.:	23338..24000	Cell Division	COG0850
110.1_WP_079336804.:	CUST_255_PI442190799	HOT208_60m_810P02	110.1_WP_079336804.:	TGGAGACGTTAATCCAGGAGCAATAGTTTCCGCTAAGAAAAATATTTACGTTTGGGGCAA	minC	septum site-determining protein MinC	WP_079336804.:	23338..24000	Cell Division	COG0850
110.1_WP_079336804.:	CUST_256_PI442190799	HOT208_60m_810P02	110.1_WP_079336804.:	TGTGCAATTCAAAAAAGAAAGACGATTTCCTTCACAAAGGAACAATACGATCGGGCGAAA	minC	septum site-determining protein MinC	WP_079336804.:	23338..24000	Cell Division	COG0850
110.1_WP_079336804.:	CUST_257_PI442190799	HOT208_60m_810P02	110.1_WP_079336804.:	TTCCATCAAGGAACCTTTAGAAGCAAAAATTTTCTCAGTCAATGAGTCAATAAGTTCTCA	minC	septum site-determining protein MinC	WP_079336804.:	23338..24000	Cell Division	COG0850
110.1_WP_079336804.:	CUST_258_PI442190799	HOT208_60m_810P02	110.1_WP_079336804.:	AGAGAAACAATTATTAGTGGAAAATCTTTAAAAATAGATTCAGCTTTTATGAAAGAGCAA	minC	septum site-determining protein MinC	WP_079336804.:	23338..24000	Cell Division	COG0850
110.1_WP_079336831.:	CUST_462_PI442190799	HOT208_60m_810P02	110.1_WP_079336831.:	TTGGGAAAGTATTTCAGTTCAGCAATGGATTTTCTCAAGGGCTTTGGAGGTTTTCAGAGG	ppc	Phosphoenolpyruvate carboxylase	WP_079336831.:	60554..60931	Krebs cycle and related genes	COG2352
110.1_WP_079336831.:	CUST_463_PI442190799	HOT208_60m_810P02	110.1_WP_079336831.:	TTGTAGATGAAATTGTATTGGCTAAGGCAAGAAGAGAAAGTGATGGAACATACTCTGCGT	ppc	Phosphoenolpyruvate carboxylase	WP_079336831.:	60554..60931	Krebs cycle and related genes	COG2352
110.1_WP_079336831.:	CUST_464_PI442190799	HOT208_60m_810P02	110.1_WP_079336831.:	TCAGGCTGATGGATTAAAAGGCATCCTTAATAGGGATTTTGATGAATGGTCTAATTTTGA	ppc	Phosphoenolpyruvate carboxylase	WP_079336831.:	60554..60931	Krebs cycle and related genes	COG2352
110.1_WP_079336831.:	CUST_465_PI442190799	HOT208_60m_810P02	110.1_WP_079336831.:	TGAATCAAAATTCTGTCAAGACAATTGGCATAAATGATGAACCAAGAAAAGATTCACACT	ppc	Phosphoenolpyruvate carboxylase	WP_079336831.:	60554..60931	Krebs cycle and related genes	COG2352
110.1_WP_079336831.:	CUST_466_PI442190799	HOT208_60m_810P02	110.1_WP_079336831.:	TCCCAAATGATTTTGAGAGCATTAGAAAAGAAAAATGCTTTGGTAAAAAAAGTGCTTACA	ppc	Phosphoenolpyruvate carboxylase	WP_079336831.:	60554..60931	Krebs cycle and related genes	COG2352
110.1_WP_079336831.:	CUST_467_PI442190799	HOT208_60m_810P02	110.1_WP_079336831.:	AGGTTTTCAGAGGTATAAAAATTGATATTAAATGCGATTGTTGTGAA	ppc	Phosphoenolpyruvate carboxylase	WP_079336831.:	60554..60931	Krebs cycle and related genes	COG2352
111.1_WP_079336909.:	CUST_981_PI442190799	HOT208_60m_810P02	111.1_WP_079336909.:	GTGCTTTGGCGGGTTATGGATTTCTGGAGGAACCGCATCAAAACATTTCAAAAACTTTAA	glk	Glucokinase	WP_079336909.:	49223..50257	Glycolysis	COG0837
111.1_WP_079336909.:	CUST_982_PI442190799	HOT208_60m_810P02	111.1_WP_079336909.:	ATATTCTGAAGTTACTAATAATGATTTGCATGCCATTGTTGGAGCGGGGACTGGTTTGGG	glk	Glucokinase	WP_079336909.:	49223..50257	Glycolysis	COG0837
111.1_WP_079336909.:	CUST_983_PI442190799	HOT208_60m_810P02	111.1_WP_079336909.:	GTTTATCAAGAATTGCCGAATGGAGACTAAGCAAACCTGATGCCCAAAACCATCGTCTAC	glk	Glucokinase	WP_079336909.:	49223..50257	Glycolysis	COG0837
111.1_WP_079336909.:	CUST_984_PI442190799	HOT208_60m_810P02	111.1_WP_079336909.:	AGTTGAGAGGATTTGGTTAAGCGCTTATGCCTCTTTATTGGGAGATGTTGCTCTTCAAGA	glk	Glucokinase	WP_079336909.:	49223..50257	Glycolysis	COG0837
111.1_WP_079336909.:	CUST_985_PI442190799	HOT208_60m_810P02	111.1_WP_079336909.:	CAGGGGAAAAGGTAAAAGTTTTAGCTAGTGAAGGTGGTCATGTTGAGTACTCACCAAAGT	glk	Glucokinase	WP_079336909.:	49223..50257	Glycolysis	COG0837
111.1_WP_079336909.:	CUST_986_PI442190799	HOT208_60m_810P02	111.1_WP_079336909.:	TGAAAGTAATTTTAGATGAAGAGTTTGGACTTTTTAGTGCAGCCTGCAGAGCAAAAATGC	glk	Glucokinase	WP_079336909.:	49223..50257	Glycolysis	COG0837
114.1_WP_011863425.:	CUST_1764_PI442190799	HOT208_60m_810P02	114.1_WP_011863425.:	GCAGCACAGGCAGCAATGAATAGTCCTTTATTAGAAGCAGCAAGAATTGATGGAGCTAAA	ftsZ	cell division protein FtsZ	WP_011863425.:	1347..2462	Cell Division	COG0206
114.1_WP_011863425.:	CUST_1765_PI442190799	HOT208_60m_810P02	114.1_WP_011863425.:	GTTGATTTTGCTGACGTTAGATCAGTAATGACTGAGGCTGGAACTGCACTTCTCGGAATA	ftsZ	cell division protein FtsZ	WP_011863425.:	1347..2462	Cell Division	COG0206
114.1_WP_011863425.:	CUST_1766_PI442190799	HOT208_60m_810P02	114.1_WP_011863425.:	CCTCTTCAAGAAGCATTTAGGAATGCTGATGATGTTTTAAGGATGGGGGTCAAAGGCATT	ftsZ	cell division protein FtsZ	WP_011863425.:	1347..2462	Cell Division	COG0206
114.1_WP_011863425.:	CUST_1767_PI442190799	HOT208_60m_810P02	114.1_WP_011863425.:	GGAGGGGATCGCAAGACTAGCTGAAAACGTTGATACTCTCATAGTTATTCCAAATGACCG	ftsZ	cell division protein FtsZ	WP_011863425.:	1347..2462	Cell Division	COG0206
114.1_WP_011863425.:	CUST_1768_PI442190799	HOT208_60m_810P02	114.1_WP_011863425.:	GCTCCCGTTGTTGCAGAAGTAGCTAAACAAAGTGGTGCTCTAACAGTAGGTATAGTAACC	ftsZ	cell division protein FtsZ	WP_011863425.:	1347..2462	Cell Division	COG0206
114.1_WP_011863425.:	CUST_1769_PI442190799	HOT208_60m_810P02	114.1_WP_011863425.:	TGATGTTGTTGATCAAGAAGCGAATATTATTGTTGGTGCAGTGGTCGATGAAGCAATGGA	ftsZ	cell division protein FtsZ	WP_011863425.:	1347..2462	Cell Division	COG0206
117.1_WP_079337153.:	CUST_2531_PI442190799	HOT208_60m_810P02	117.1_WP_079337153.:	CATTTCTCATTCTGAAAAGTCCATTGAGTATCCAGCTGGTGGGGGATCTATTATCGCGAA	rbsK	Sugar kinase, ribokinase family	WP_079337153.:	27179..28048	Pentose Phosphate	COG0524
117.1_WP_079337153.:	CUST_2532_PI442190799	HOT208_60m_810P02	117.1_WP_079337153.:	TTGCTAAAGTAATGGGAAGAGACGCCAGTGAATTTTTCGGCCCATATGCAAATAGTGATG	rbsK	Sugar kinase, ribokinase family	WP_079337153.:	27179..28048	Pentose Phosphate	COG0524
117.1_WP_079337153.:	CUST_2533_PI442190799	HOT208_60m_810P02	117.1_WP_079337153.:	TTCTTATGGATGTGGCGATTCTTTCGCTGCTGGGATTCTTTATGGAATGGCATCTAAATG	rbsK	Sugar kinase, ribokinase family	WP_079337153.:	27179..28048	Pentose Phosphate	COG0524
117.1_WP_079337153.:	CUST_2534_PI442190799	HOT208_60m_810P02	117.1_WP_079337153.:	AACCGAGGGAGAGAAGGGAGGAATTTTATTCCCTGGAGGAAGATATAAGGCTCTTAAAAA	rbsK	Sugar kinase, ribokinase family	WP_079337153.:	27179..28048	Pentose Phosphate	COG0524
117.1_WP_079337153.:	CUST_2535_PI442190799	HOT208_60m_810P02	117.1_WP_079337153.:	CTCTCAAATATGGGAATAAAGTTGCATGTAGCTTGGCGTGATAAACCAACTAGAAGAGGA	rbsK	Sugar kinase, ribokinase family	WP_079337153.:	27179..28048	Pentose Phosphate	COG0524
117.1_WP_079337153.:	CUST_2536_PI442190799	HOT208_60m_810P02	117.1_WP_079337153.:	TCAGAGATTTTTAAAATGGCTAGATCAGCCTCAATACTTTGTACAACACCAAGGGTAGGA	rbsK	Sugar kinase, ribokinase family	WP_079337153.:	27179..28048	Pentose Phosphate	COG0524
121.1_WP_079337190.:	CUST_2774_PI442190799	HOT208_60m_810P02	121.1_WP_079337190.:	GGATTAAATTTTTCGATGATGAAATTTTGGGATAAAGATCTTTTCAATTATGCGCAGTCA	eda	2-dehydro-3-deoxyphosphogluconate aldolase	WP_079337190.:	903..1529	Entner-Dudorff	COG0800
121.1_WP_079337190.:	CUST_2775_PI442190799	HOT208_60m_810P02	121.1_WP_079337190.:	TCGATTTTGTATCCTATATCAAAATTAAATATCCAAGAATTAATTTAGGCTCTGCCTCCA	eda	2-dehydro-3-deoxyphosphogluconate aldolase	WP_079337190.:	903..1529	Entner-Dudorff	COG0800
121.1_WP_079337190.:	CUST_2776_PI442190799	HOT208_60m_810P02	121.1_WP_079337190.:	AGGGTATAAAGCAGTCGTAATTGGAGATAAAGCTATCAAAAATAAAAAATTTGATCCAAA	eda	2-dehydro-3-deoxyphosphogluconate aldolase	WP_079337190.:	903..1529	Entner-Dudorff	COG0800
121.1_WP_079337190.:	CUST_2777_PI442190799	HOT208_60m_810P02	121.1_WP_079337190.:	AGTTCGATAAATATACTCAACTATAAAAATATTGATTTCATTGCTGCTGGAGGACTATCA	eda	2-dehydro-3-deoxyphosphogluconate aldolase	WP_079337190.:	903..1529	Entner-Dudorff	COG0800
121.1_WP_079337190.:	CUST_2778_PI442190799	HOT208_60m_810P02	121.1_WP_079337190.:	AGATTCTTTTTCGGAGTACCTGAAAACTGAATCTTTTTTTTTACTTATAAAACCTGAAGA	eda	2-dehydro-3-deoxyphosphogluconate aldolase	WP_079337190.:	903..1529	Entner-Dudorff	COG0800
121.1_WP_079337190.:	CUST_2779_PI442190799	HOT208_60m_810P02	121.1_WP_079337190.:	AGGATCTTAAGGAAGCGATAGATTTAAATTGCAACATTATCAAAATTTACCCAATAAAAA	eda	2-dehydro-3-deoxyphosphogluconate aldolase	WP_079337190.:	903..1529	Entner-Dudorff	COG0800
132.1_WP_079337330.:	CUST_3423_PI442190799	HOT208_60m_810P02	132.1_WP_079337330.:	ATTATTTCAGGGCGTGTATCTACAGAAGTTGATGCAAGACTGAGCTTTGACACTGAAGCT	tal	Transaldolase	WP_079337330.:	8396..9397	Pentose Phosphate	COG0176
132.1_WP_079337330.:	CUST_3424_PI442190799	HOT208_60m_810P02	132.1_WP_079337330.:	ACTTCTGCCAAGCTGTAACTTGTGCCAATGCAAAGATAACTCTAATTTCTCCCTTCGTTG	tal	Transaldolase	WP_079337330.:	8396..9397	Pentose Phosphate	COG0176
132.1_WP_079337330.:	CUST_3425_PI442190799	HOT208_60m_810P02	132.1_WP_079337330.:	GCAACTTGGGAGGGAATTAAGGCAGCTGAAATTTTGGAAAAAGAGGGTATTAAGTGCAAC	tal	Transaldolase	WP_079337330.:	8396..9397	Pentose Phosphate	COG0176
132.1_WP_079337330.:	CUST_3426_PI442190799	HOT208_60m_810P02	132.1_WP_079337330.:	TTGGATTGGCATAAAGCAAAAACTGGTAAAACTAGTTTTGTTGGTGCTGAAGACCCTGGT	tal	Transaldolase	WP_079337330.:	8396..9397	Pentose Phosphate	COG0176
132.1_WP_079337330.:	CUST_3427_PI442190799	HOT208_60m_810P02	132.1_WP_079337330.:	TTAGAAGATCAAATGGCAAGCGAAAAGCTTAGTGAAGGTATCACTGGATTCAGTAAGGCT	tal	Transaldolase	WP_079337330.:	8396..9397	Pentose Phosphate	COG0176
132.1_WP_079337330.:	CUST_3428_PI442190799	HOT208_60m_810P02	132.1_WP_079337330.:	AGAAGTAATGGGAGCGAGTTTTAGAAATCTCGATGAAATAAAAGAATTAGCAGGTTGCGA	tal	Transaldolase	WP_079337330.:	8396..9397	Pentose Phosphate	COG0176
132.1_WP_079337326.:	CUST_3787_PI442190799	HOT208_60m_810P02	132.1_WP_079337326.:	ATTGGTAATCCTGGTCAGGCAAATTATTCTGCAGCCAAAGCTGGAGTTATTGGATTTACC	gdh	3-oxoacyl-acyl-carrier-protein reductase	WP_079337326.:	65637..66386	Entner-Dudorff	COG1028
132.1_WP_079337326.:	CUST_3788_PI442190799	HOT208_60m_810P02	132.1_WP_079337326.:	AGCTAGTAGAGGAATTGGTAAAGAAATTGCTTTAGAACTAAGCCGATTGGGAGCAGTGGT	gdh	3-oxoacyl-acyl-carrier-protein reductase	WP_079337326.:	65637..66386	Entner-Dudorff	COG1028
132.1_WP_079337326.:	CUST_3789_PI442190799	HOT208_60m_810P02	132.1_WP_079337326.:	TCAAGTGATGCAGGAAAATACATTACAGGGCAAACAATCAGTATAGACGGGGGTATGAGT	gdh	3-oxoacyl-acyl-carrier-protein reductase	WP_079337326.:	65637..66386	Entner-Dudorff	COG1028
132.1_WP_079337326.:	CUST_3790_PI442190799	HOT208_60m_810P02	132.1_WP_079337326.:	TGCTGGAATTACTAGAGATGGACTATTGATGAGAATGAAATCGGAACAATGGGATGATGT	gdh	3-oxoacyl-acyl-carrier-protein reductase	WP_079337326.:	65637..66386	Entner-Dudorff	COG1028
132.1_WP_079337326.:	CUST_3791_PI442190799	HOT208_60m_810P02	132.1_WP_079337326.:	ACTTGCGCTAAAGAATTTGCTTCAAGAGGTATAAATGTTAATTCAATAGCTCCAGGTTTT	gdh	3-oxoacyl-acyl-carrier-protein reductase	WP_079337326.:	65637..66386	Entner-Dudorff	COG1028
132.1_WP_079337326.:	CUST_3792_PI442190799	HOT208_60m_810P02	132.1_WP_079337326.:	ATTCCTTTAGGGAAATTAGGAAGTTGTGCTCAAATTGCAAACTTAGTGTCATTCTTAGTT	gdh	3-oxoacyl-acyl-carrier-protein reductase	WP_079337326.:	65637..66386	Entner-Dudorff	COG1028
133.1_WP_079337341.:	CUST_3838_PI442190799	HOT208_60m_810P02	133.1_WP_079337341.:	AACTCTGAAGCTTCAAAAGGTGCCTATGTAGCACGATCTCAACCTTCATCTGATGAACAA	fda	Fructose-1,6-bisphosphate aldolase class I	WP_079337341.:	5193..6260	Glycolysis/Gluconeogenesis	COG0191
133.1_WP_079337341.:	CUST_3839_PI442190799	HOT208_60m_810P02	133.1_WP_079337341.:	GAGAATGCTTGGGGATTAGCAAGGTATGCAAGATCAGTTCAAGAATCTGGGTTAGTACCA	fda	Fructose-1,6-bisphosphate aldolase class I	WP_079337341.:	5193..6260	Glycolysis/Gluconeogenesis	COG0191
133.1_WP_079337341.:	CUST_3840_PI442190799	HOT208_60m_810P02	133.1_WP_079337341.:	GAAGGTTGCTGAAATGACAATTAGAACTATGGAAAGATGCGTTCCTGCATCTGTTCCTGG	fda	Fructose-1,6-bisphosphate aldolase class I	WP_079337341.:	5193..6260	Glycolysis/Gluconeogenesis	COG0191
133.1_WP_079337341.:	CUST_3841_PI442190799	HOT208_60m_810P02	133.1_WP_079337341.:	AAGGTCGATAAGGGTCTAAATCCACTTCCAGGAGGAGGAGATGTAGAAACTTTTTGCTCT	fda	Fructose-1,6-bisphosphate aldolase class I	WP_079337341.:	5193..6260	Glycolysis/Gluconeogenesis	COG0191
133.1_WP_079337341.:	CUST_3842_PI442190799	HOT208_60m_810P02	133.1_WP_079337341.:	TATGGAATGTTTCATTCTCCTATGGAAGAGCATTACAGCACTCATGCTTAAAGGCCTGGA	fda	Fructose-1,6-bisphosphate aldolase class I	WP_079337341.:	5193..6260	Glycolysis/Gluconeogenesis	COG0191
133.1_WP_079337341.:	CUST_3843_PI442190799	HOT208_60m_810P02	133.1_WP_079337341.:	TTAGTTGAAAGAGCCGCAAAATATTATGAACAAGGTGCCAGATTTGCAAAGTGGAGAGCA	fda	Fructose-1,6-bisphosphate aldolase class I	WP_079337341.:	5193..6260	Glycolysis/Gluconeogenesis	COG0191
133.1_WP_025881193.:	CUST_3861_PI442190799	HOT208_60m_810P02	133.1_WP_025881193.:	CTCCTGATGAGAGCTTTGTGATCATACATTTCAGAAAAGGGGCAAGAGAGAAATGGGGTA	prk	Phosphoribulokinase	WP_025881193.:	8879..9775	Carbon Fixation	COG3954
133.1_WP_025881193.:	CUST_3862_PI442190799	HOT208_60m_810P02	133.1_WP_025881193.:	CTATGGCAGACGCTCTTTCAAAAGGTGAAAATTTCTCTCATTTTGGTCCCGAGGCTAATC	prk	Phosphoribulokinase	WP_025881193.:	8879..9775	Carbon Fixation	COG3954
133.1_WP_025881193.:	CUST_3863_PI442190799	HOT208_60m_810P02	133.1_WP_025881193.:	GGGCAATTTACTCCATGGGAAGATATTCCTGAGGGGACAGACGTACTTTTTTATGAAGGT	prk	Phosphoribulokinase	WP_025881193.:	8879..9775	Carbon Fixation	COG3954
133.1_WP_025881193.:	CUST_3864_PI442190799	HOT208_60m_810P02	133.1_WP_025881193.:	GTGATGGATACAATGTGGCATCCTATGCTGATTTACTTGTTGGCGTTGTTCCGATAACAA	prk	Phosphoribulokinase	WP_025881193.:	8879..9775	Carbon Fixation	COG3954
133.1_WP_025881193.:	CUST_3865_PI442190799	HOT208_60m_810P02	133.1_WP_025881193.:	TAACGCAGAAAGAGGTTACTCAGCGGAAACCATTGTGGATACGATATTGAGAAGAATGCC	prk	Phosphoribulokinase	WP_025881193.:	8879..9775	Carbon Fixation	COG3954
133.1_WP_025881193.:	CUST_3866_PI442190799	HOT208_60m_810P02	133.1_WP_025881193.:	TGTCAAGTCCAACAAGTATCGTTGTAAATGGTGGGAAAATGGGATTCGCAATGGAACTAA	prk	Phosphoribulokinase	WP_025881193.:	8879..9775	Carbon Fixation	COG3954
134.1_WP_079337386.:	CUST_4002_PI442190799	HOT208_60m_810P02	134.1_WP_079337386.:	GGGATGGGACTTGCAACAATGGGCAGTTTTTTGAATGTTTAAATATGGCCCAATTATGGA	pdhA	Pyruvate dehydrogenase E1 component alpha subunit	WP_079337386.:	1186..2223	Glycolysis/Krebs cycle	COG1071
134.1_WP_079337386.:	CUST_4003_PI442190799	HOT208_60m_810P02	134.1_WP_079337386.:	TTGATGCAGAGATATCGGAGTCAGTTAAAAATGCTATTGAAGCTCCAGAACCCCCTTCAG	pdhA	Pyruvate dehydrogenase E1 component alpha subunit	WP_079337386.:	1186..2223	Glycolysis/Krebs cycle	COG1071
134.1_WP_079337386.:	CUST_4004_PI442190799	HOT208_60m_810P02	134.1_WP_079337386.:	CAGTTGCCTTAGGGGCGGCCTTTTCAAGTAGATACAAAAAGGAGGTTTCTGGTAATAGTA	pdhA	Pyruvate dehydrogenase E1 component alpha subunit	WP_079337386.:	1186..2223	Glycolysis/Krebs cycle	COG1071
134.1_WP_079337386.:	CUST_4005_PI442190799	HOT208_60m_810P02	134.1_WP_079337386.:	CAATTGAGCGCGCTAGAGCAGGAGAAGGTCCCACACTTTTAGAATGTTTGACTTATAGAT	pdhA	Pyruvate dehydrogenase E1 component alpha subunit	WP_079337386.:	1186..2223	Glycolysis/Krebs cycle	COG1071
134.1_WP_079337386.:	CUST_4006_PI442190799	HOT208_60m_810P02	134.1_WP_079337386.:	CTCACGATAGAGCCACTAGTAATCCTGAAATCTGGAGAAAAGCGTCTGCTTTTGGTATGC	pdhA	Pyruvate dehydrogenase E1 component alpha subunit	WP_079337386.:	1186..2223	Glycolysis/Krebs cycle	COG1071
134.1_WP_079337386.:	CUST_4007_PI442190799	HOT208_60m_810P02	134.1_WP_079337386.:	CGAGGTGGATCCATGCACTTATTTTCAAGAGAGCATCACTTACTCGGAGGATATGCATTT	pdhA	Pyruvate dehydrogenase E1 component alpha subunit	WP_079337386.:	1186..2223	Glycolysis/Krebs cycle	COG1071
143.1_782	CUST_4146_PI442190799	HOT208_60m_810P02	143.1_782	AAAGGTTAAAAATGGATATACACTTAACATTTATAAAGACAAATTAGAGCTATCTACAGC	pgi	Glucose-6-phosphate isomerase		<1..134	Glycolysis/Gluconeogenesis	COG0166
143.1_783	CUST_4147_PI442190799	HOT208_60m_810P02	143.1_783	ATATGAAAGAATTGATAAGGAAGGTAGTTTTCATACTGAATGGATGAAATGA	edd	phosphogluconate dehydrogenase (NADP(+)-dependent, decarboxylating)		122..420	Pentose Phosphate/Entner-Dudorof	COG2084
143.1_783	CUST_4148_PI442190799	HOT208_60m_810P02	143.1_783	AGACTTCCTCAGAACCTTGTTCAGGCAATGAGAGACTGTTTTGGTTCTCATACATATGAA	edd	phosphogluconate dehydrogenase (NADP(+)-dependent, decarboxylating)		122..420	Pentose Phosphate/Entner-Dudorof	COG2084
143.1_783	CUST_4149_PI442190799	HOT208_60m_810P02	143.1_783	GCTAGAGTTGTTTCTTTATCGACAAAAGCTGGTATACCAGTCCCATGTCTATCCAGTACC	edd	phosphogluconate dehydrogenase (NADP(+)-dependent, decarboxylating)		122..420	Pentose Phosphate/Entner-Dudorof	COG2084
143.1_783	CUST_4150_PI442190799	HOT208_60m_810P02	143.1_783	TCAAGATGCTTATAACAAAGACCCTAATCTAAAAAATTTAATTTTTGATGATTGGTTCAA	edd	phosphogluconate dehydrogenase (NADP(+)-dependent, decarboxylating)		122..420	Pentose Phosphate/Entner-Dudorof	COG2084
163.1_WP_079337619.:	CUST_4944_PI442190799	HOT208_60m_810P02	163.1_WP_079337619.:	CTACAAGATCGGGTCAGATCAAGACCGGCTCTTTGAGTAGAAGTGAAAGGATTGCAAAAT	eno	Enolase	WP_079337619.:	6908..8200	Glycolysis/Gluconeogenesis	COG0148
163.1_WP_079337619.:	CUST_4945_PI442190799	HOT208_60m_810P02	163.1_WP_079337619.:	AGAGGACGGTTTAGCTGAGGATGATTGGGAGGGTTGGTCAGAATTAAACAAAGAATTAGG	eno	Enolase	WP_079337619.:	6908..8200	Glycolysis/Gluconeogenesis	COG0148
163.1_WP_079337619.:	CUST_4946_PI442190799	HOT208_60m_810P02	163.1_WP_079337619.:	ACAATAGGCTTTTAAAAATTGAGGAGGAGTTGGGAAATCAAGCAAGATTCGCTGGAGCTT	eno	Enolase	WP_079337619.:	6908..8200	Glycolysis/Gluconeogenesis	COG0148
163.1_WP_079337619.:	CUST_4947_PI442190799	HOT208_60m_810P02	163.1_WP_079337619.:	CCGGGTTTAAGCCTGGTGAGCAGGTATCTTTAGCTTTAGATGCTGCTAGTACTGAATTTT	eno	Enolase	WP_079337619.:	6908..8200	Glycolysis/Gluconeogenesis	COG0148
163.1_WP_079337619.:	CUST_4948_PI442190799	HOT208_60m_810P02	163.1_WP_079337619.:	AATCATTACTTGATCAAAAAGGTCTATCTACTGCTGTAGGCGATGAGGGTGGATTTGCCC	eno	Enolase	WP_079337619.:	6908..8200	Glycolysis/Gluconeogenesis	COG0148
163.1_WP_079337619.:	CUST_4949_PI442190799	HOT208_60m_810P02	163.1_WP_079337619.:	GCCCCGAATTTGTCATCAAGCGAAGAGGCAGGGGACTTATTATTAGAAGCAATTCA	eno	Enolase	WP_079337619.:	6908..8200	Glycolysis/Gluconeogenesis	COG0148
164.1_WP_079337664.:	CUST_5106_PI442190799	HOT208_60m_810P02	164.1_WP_079337664.:	GGACATCTCCAGGCATTGTTAACGATGGTCAAAGGGGTACCACTTTCTTACAATAAGGAT	argH	Argininosuccinate lyase	WP_079337664.:	10962..12341	Amino Acid metabolism	COG0165
164.1_WP_079337664.:	CUST_5107_PI442190799	HOT208_60m_810P02	164.1_WP_079337664.:	TGTCGTTAAATCAAGAAATAGCGAAGGTGGCACAGGTTTTGTTCAGGTTGAAAAAGAGGT	argH	Argininosuccinate lyase	WP_079337664.:	10962..12341	Amino Acid metabolism	COG0165
164.1_WP_079337664.:	CUST_5108_PI442190799	HOT208_60m_810P02	164.1_WP_079337664.:	TGTTCCTTTTAGGACCGCTTATCAAGTTGTTGGTCAAATGGTCAAATATTGCCTTGAGAG	argH	Argininosuccinate lyase	WP_079337664.:	10962..12341	Amino Acid metabolism	COG0165
164.1_WP_079337664.:	CUST_5110_PI442190799	HOT208_60m_810P02	164.1_WP_079337664.:	TCTGATTCTGTAGAAAACGACTTTTCTAATGCTACTGATTTGGCAGATTACTTAGTTGGT	argH	Argininosuccinate lyase	WP_079337664.:	10962..12341	Amino Acid metabolism	COG0165
166.1_WP_079337672.:	CUST_5141_PI442190799	HOT208_60m_810P02	166.1_WP_079337672.:	GTACTTTAATCCTGTTGGAGCCCATAGTTCGGGGATATTAGGTGAAGACCAAAAGGGAAG	galE	UDP-glucose 4-epimerase	WP_079337672.:	1879..2925	Galactose metabolism	COG1087
166.1_WP_079337672.:	CUST_5142_PI442190799	HOT208_60m_810P02	166.1_WP_079337672.:	AGAAGCTGTTATACATTTTGCAGGTTTAAAGTCAGTAGAGGAATCAGTATCGGAACCTCT	galE	UDP-glucose 4-epimerase	WP_079337672.:	1879..2925	Galactose metabolism	COG1087
166.1_WP_079337672.:	CUST_5143_PI442190799	HOT208_60m_810P02	166.1_WP_079337672.:	GGTAAGTTAGACAAAGTTTTGATATTCGGGAATGATTGGCCTACAGAAGATGGAACTTGT	galE	UDP-glucose 4-epimerase	WP_079337672.:	1879..2925	Galactose metabolism	COG1087
166.1_WP_079337672.:	CUST_5144_PI442190799	HOT208_60m_810P02	166.1_WP_079337672.:	TCCAATTCCTTATGAATTTGCTAAACGTAGAAAAGGTGATGCTCCTTATCTAGTTGCAGA	galE	UDP-glucose 4-epimerase	WP_079337672.:	1879..2925	Galactose metabolism	COG1087
166.1_WP_079337672.:	CUST_5145_PI442190799	HOT208_60m_810P02	166.1_WP_079337672.:	TGGATTTATCTGAAGCACATTTTGCTGCATTAAAGTTTTTAAAAAAAAATGATCCTCAAT	galE	UDP-glucose 4-epimerase	WP_079337672.:	1879..2925	Galactose metabolism	COG1087
166.1_WP_079337672.:	CUST_5146_PI442190799	HOT208_60m_810P02	166.1_WP_079337672.:	TGTTCAACTTGTTAATTTAGTAGTAGTGCGACTATTTAGAGGCCTTTTAAAGATCAAAAA	galE	UDP-glucose 4-epimerase	WP_079337672.:	1879..2925	Galactose metabolism	COG1087
017.1_WP_025945934.:	CUST_5211_PI442190799	HOT208_60m_810P02	017.1_WP_025945934.:	GAACTTGGTGCTGAGCATAAAGAACTTGAAGCATATCTTATGACAGTTCGTGGCGGTATA	pdhB	Pyruvate dehydrogenase E1 component beta subunit	WP_025945934.:	1644..2627	Glycolysis/Krebs cycle	COG0022
017.1_WP_025945934.:	CUST_5212_PI442190799	HOT208_60m_810P02	017.1_WP_025945934.:	TATTGGCGCAGAATTAATTGCCTTGAATGCGGATGTTTCGATGATCTTGATGCCCCC	pdhB	Pyruvate dehydrogenase E1 component beta subunit	WP_025945934.:	1644..2627	Glycolysis/Krebs cycle	COG0022
017.1_WP_025945934.:	CUST_5213_PI442190799	HOT208_60m_810P02	017.1_WP_025945934.:	ACCTGAGGGTGATTATACTTGCGCCTTAGATCAGGCTGACGTTTGGCTGCTAAAGGGAAAGAG	pdhB	Pyruvate dehydrogenase E1 component beta subunit	WP_025945934.:	1644..2627	Glycolysis/Krebs cycle	COG0022
017.1_WP_025945934.:	CUST_5214_PI442190799	HOT208_60m_810P02	017.1_WP_025945934.:	TACAAGTGGCGGAAATTATAAATACCAGCAGTTAGAGGACGTTTAGTTATGGAATGACGCG	pdhB	Pyruvate dehydrogenase E1 component beta subunit	WP_025945934.:	1644..2627	Glycolysis/Krebs cycle	COG0022
017.1_WP_025945934.:	CUST_5215_PI442190799	HOT208_60m_810P02	017.1_WP_025945934.:	TGTTGCATGTAGTACACCTACAATGCCTAGCTAAGGTTTAATGAAAGCCAGCTATAAGAGATGA	pdhB	Pyruvate dehydrogenase E1 component beta subunit	WP_025945934.:	1644..2627	Glycolysis/Krebs cycle	COG0022
017.1_WP_025945934.:	CUST_5216_PI442190799	HOT208_60m_810P02	017.1_WP_025945934.:	TGAGACATCACTGCCTTAAAGCTGTTGAAGAATTAGAAAAAAAGGAATAGATGATTGAGT	pdhB	Pyruvate dehydrogenase E1 component beta subunit	WP_025945934.:	1644..2627	Glycolysis/Krebs cycle	COG0022
181.1_WP_025945861.:	CUST_5388_PI442190799	HOT208_60m_810P02	181.1_WP_025945861.:	ACTCGGTTCCTTGAGGATACTCTGTTATGTGGAAGCCGATTTCTTGAATCTCCTGAAGAGA	glgC	Glucose-1-phosphate adenylyltransferase	WP_025945861.:	17594..18889	Glycogen Metabolism	COG0448
181.1_WP_025945861.:	CUST_5389_PI442190799	HOT208_60m_810P02	181.1_WP_025945861.:	TCAGCCGCCGAAAAACCATATCTAGCCTCATGGGGATTTACGTTTTTAGCAGAAATACT	glgC	Glucose-1-phosphate adenylyltransferase	WP_025945861.:	17594..18889	Glycogen Metabolism	COG0448
181.1_WP_025945861.:	CUST_5390_PI442190799	HOT208_60m_810P02	181.1_WP_025945861.:	CGATGATTTACTGGGAAGATAGGCACAATTGGGGCATTCTTTGAGTCAAACCTTGCATT	glgC	Glucose-1-phosphate adenylyltransferase	WP_025945861.:	17594..18889	Glycogen Metabolism	COG0448
181.1_WP_025945861.:	CUST_5391_PI442190799	HOT208_60m_810P02	181.1_WP_025945861.:	AGTGAAAAGCCTACTGGAGAAGTTGAAGGCTATGGCGGGTAGATACTTCAAAATTTGGA	glgC	Glucose-1-phosphate adenylyltransferase	WP_025945861.:	17594..18889	Glycogen Metabolism	COG0448
181.1_WP_025945861.:	CUST_5392_PI442190799	HOT208_60m_810P02	181.1_WP_025945861.:	GCGGAACGCCTCTCGGAGTAGGTGAAGGAACTGCCTATCCTCTCAAATCTCTGATAAG	glgC	Glucose-1-phosphate adenylyltransferase	WP_025945861.:	17594..18889	Glycogen Metabolism	COG0448
181.1_WP_025945861.:	CUST_5393_PI442190799	HOT208_60m_810P02	181.1_WP_025945861.:	ACGGATTTTGGTAAGGACATAATTCAGAAGCGCTGAATGAAGGCGATACTCTTAAAAGT	glgC	Glucose-1-phosphate adenylyltransferase	WP_025945861.:	17594..18889	Glycogen Metabolism	COG0448
181.1_1027	CUST_5394_PI442190799	HOT208_60m_810P02	181.1_1027	GGATATGTGGACCAGGAGGCTCAGGTCATTTTGGAAAACTGTTCAACAAGGAATGGAA	edd	phosphogluconate dehydrogenase (NADP(+)-dependent, decarboxylating)		18985..19637	Pentose Phosphate/Entner-Dudorof	COG2084
181.1_1027	CUST_5395_PI442190799	HOT208_60m_810P02	181.1_1027	CGAAGAATTTCAAAGGTCGTGGCCTTGGGAGAATGATATAGAGGGAGCTGAAATCCTTCA	edd	phosphogluconate dehydrogenase (NADP(+)-dependent, decarboxylating)		18985..19637	Pentose Phosphate/Entner-Dudorof	COG2084
181.1_1027	CUST_5396_PI442190799	HOT208_60m_810P02	181.1_1027	AATACTCTTGAAAGTAAAAGTTTTGGATACATTGGATGGGAGTCTCTGGAGGGCCAAA	edd	phosphogluconate dehydrogenase (NADP(+)-dependent, decarboxylating)		18985..19637	Pentose Phosphate/Entner-Dudorof	COG2084
181.1_1027	CUST_5397_PI442190799	HOT208_60m_810P02	181.1_1027	TTTAGAGGGGCCAAGTATGATGCCCGGTGGTCATAACGGTTTTCATATGATCAATAGAAAG	edd	phosphogluconate dehydrogenase (NADP(+)-dependent, decarboxylating)		18985..19637	Pentose Phosphate/Entner-Dudorof	COG2084
181.1_1027	CUST_5398_PI442190799	HOT208_60m_810P02	181.1_1027	GCTAGAGGACCAGAAGAATTTAATGATGGTAAAAGCTGGGACGCGAGAGGACAGGCAAAA	edd	phosphogluconate dehydrogenase (NADP(+)-dependent, decarboxylating)		18985..19637	Pentose Phosphate/Entner-Dudorof	COG2084
181.1_1027	CUST_5399_PI442190799	HOT208_60m_810P02	181.1_1027	TGCAGAAGCTTATGACCTTATGAAGAGAGTCAAGGGTATGAATGGACAGCAGATGTCAGA	edd	phosphogluconate dehydrogenase (NADP(+)-dependent, decarboxylating)		18985..19637	Pentose Phosphate/Entner-Dudorof	COG2084
018.1_WP_079336562.:	CUST_5614_PI442190799	HOT208_60m_810P02	018.1_WP_079336562.:	AGTAGAAGTGAACTATTGAGGGGGCACTTTTAACTATTAATGGGATAGCAGCTGGCATG	ppc	Phosphoenolpyruvate carboxylase	WP_079336562.:	20278..23247	Krebs cycle and related genes	COG2352
018.1_WP_079336562.:	CUST_5615_PI442190799	HOT208_60m_810P02	018.1_WP_079336562.:	AGGGTTTTGCAGGTTTCACTTTAAGAAGATTAAGAGATCAGCAAGACAGCCTCCAAT	ppc	Phosphoenolpyruvate carboxylase	WP_079336562.:	20278..23247	Krebs cycle and related genes	COG2352

018.1_WP_079336562.:	CUST_5616_PI442190799	HOT208_60m_810P02 018.1_WP_079336562.:	AGGATGCTTATATCTAAGGTCGAAATGACATTATCGAAGGTGGATCTGGAAGTTGCTAGA	ppc	Phosphoenolpyruvate carboxylase	WP_079336562.:	20278..23247	Krebs cycle and related genes	COG2352
018.1_WP_025889445.:	CUST_5892_PI442190799	HOT208_60m_810P02 018.1_WP_025889445.:	ACATGTCAAGTTTCTTCATGGGACCACGTGTTCTTAGCCCTTTTCTGGATGTATAACTGT	psaA	Photosystem I P700 chlorophyll A apoprotein A1 PsaA	WP_025889445.:	59453..61756	Photosystem I	COG2885
018.1_WP_025889445.:	CUST_5893_PI442190799	HOT208_60m_810P02 018.1_WP_025889445.:	AAACTTAAAGTTGCTCCAACCATTCAACCAAGAGCTTTATCTATCACCCAGGGTAGAGCA	psaA	Photosystem I P700 chlorophyll A apoprotein A1 PsaA	WP_025889445.:	59453..61756	Photosystem I	COG2885
018.1_WP_025889445.:	CUST_5894_PI442190799	HOT208_60m_810P02 018.1_WP_025889445.:	GAAAATGCAGAGTGATGTTTGGGGACTTACTGGTGGTAACTTCGCACAAAGTTCCATTAC	psaA	Photosystem I P700 chlorophyll A apoprotein A1 PsaA	WP_025889445.:	59453..61756	Photosystem I	COG2885
018.1_WP_025889445.:	CUST_5895_PI442190799	HOT208_60m_810P02 018.1_WP_025889445.:	TGGAAGTTTAGTAGAAGTGGGTGGAAAAGTTGCTATAGCTCCAATTCCATTAGGGACAGC	psaA	Photosystem I P700 chlorophyll A apoprotein A1 PsaA	WP_025889445.:	59453..61756	Photosystem I	COG2885
018.1_WP_025889445.:	CUST_5896_PI442190799	HOT208_60m_810P02 018.1_WP_025889445.:	TTGATCCCTGATAAAGCTTCTTTAGGATTTAGATTCCCTTGTGATGGACCTGGAAGAGGC	psaA	Photosystem I P700 chlorophyll A apoprotein A1 PsaA	WP_025889445.:	59453..61756	Photosystem I	COG2885
018.1_WP_025889445.:	CUST_5897_PI442190799	HOT208_60m_810P02 018.1_WP_025889445.:	TGGTTGGTTAAGAGATTTTCTTTGGGCGCAAGCTTCTCAAGTATTAACAAGTTATGGTCA	psaA	Photosystem I P700 chlorophyll A apoprotein A1 PsaA	WP_025889445.:	59453..61756	Photosystem I	COG2885
018.1_WP_079336592.:	CUST_5898_PI442190799	HOT208_60m_810P02 018.1_WP_079336592.:	CCTATTCGGTCACTTAGTTTGGGCTACTGGTTTCATGTTCTTAATATCATGGCGTGGCTA	psaB	Photosystem I P700 chlorophyll A apoprotein A2 PsaB	WP_079336592.:	61784..64012	Photosystem I	COG2885
018.1_WP_079336592.:	CUST_5899_PI442190799	HOT208_60m_810P02 018.1_WP_079336592.:	AAATTTACTAGCATGGGTAACTTTCTACTGGCATTGGAAACACCTAGCAATTTGGCAGGG	psaB	Photosystem I P700 chlorophyll A apoprotein A2 PsaB	WP_079336592.:	61784..64012	Photosystem I	COG2885
018.1_WP_079336592.:	CUST_5900_PI442190799	HOT208_60m_810P02 018.1_WP_079336592.:	CTTTCTTACCTATCGGGCCAGCAGATTTCTTAGTTCACCATGCTATCGCTTTAGGTCTTC	psaB	Photosystem I P700 chlorophyll A apoprotein A2 PsaB	WP_079336592.:	61784..64012	Photosystem I	COG2885
018.1_WP_079336592.:	CUST_5901_PI442190799	HOT208_60m_810P02 018.1_WP_079336592.:	CCTTGTTGGCTGGAGAGATAAGCCAGTTGCACTTTCAATTGTTCAAGCTAGATTAGTTGG	psaB	Photosystem I P700 chlorophyll A apoprotein A2 PsaB	WP_079336592.:	61784..64012	Photosystem I	COG2885
018.1_WP_079336592.:	CUST_5902_PI442190799	HOT208_60m_810P02 018.1_WP_079336592.:	CCTACAAGTTCGGCAACTCTCGCAGCTAATTCATTACCAGGTAATCATTACTGGATGGAT	psaB	Photosystem I P700 chlorophyll A apoprotein A2 PsaB	WP_079336592.:	61784..64012	Photosystem I	COG2885
018.1_WP_079336592.:	CUST_5903_PI442190799	HOT208_60m_810P02 018.1_WP_079336592.:	TGGAGGTACTTGTGATAGTTCATCTTGGGACGCTATGTACTTAGCTATGTTCTGGGCATT	psaB	Photosystem I P700 chlorophyll A apoprotein A2 PsaB	WP_079336592.:	61784..64012	Photosystem I	COG2885
018.1_WP_079336601.:	CUST_5952_PI442190799	HOT208_60m_810P02 018.1_WP_079336601.:	AACGACTTCCTCCGAACAGGTTATTTTAGGAAATACCTGTCTTTATGGAGCAACTGGTGG	glsF	Ferredoxin-dependent glutamate synthase	WP_079336601.:	70414..74985	Nitrogen Metabolism	COG0069
018.1_WP_079336601.:	CUST_5953_PI442190799	HOT208_60m_810P02 018.1_WP_079336601.:	AAGAAATAGTGGTGCTACAGCGGTAACAGAAGGAGCAGGTGATCATTGTTGTGAATACAT	glsF	Ferredoxin-dependent glutamate synthase	WP_079336601.:	70414..74985	Nitrogen Metabolism	COG0069
018.1_WP_079336601.:	CUST_5954_PI442190799	HOT208_60m_810P02 018.1_WP_079336601.:	CACGGCAACACTGGCTTCAATGGTGAACTCAAATTAAATTTCAAAGGATTTGCAGGACAA	glsF	Ferredoxin-dependent glutamate synthase	WP_079336601.:	70414..74985	Nitrogen Metabolism	COG0069
018.1_WP_079336601.:	CUST_5955_PI442190799	HOT208_60m_810P02 018.1_WP_079336601.:	GTGGGAAAGTAGTTATTCTAGGTTCAACAGGAAGGAATATAGGTGCTGGCATGACTGGTG	glsF	Ferredoxin-dependent glutamate synthase	WP_079336601.:	70414..74985	Nitrogen Metabolism	COG0069
018.1_WP_079336601.:	CUST_5956_PI442190799	HOT208_60m_810P02 018.1_WP_079336601.:	TCTTATTACAAGAGTACTTTCAAATTGATTGTTCCCCCAAGCGAAGAAGAGATGCTTGGT	glsF	Ferredoxin-dependent glutamate synthase	WP_079336601.:	70414..74985	Nitrogen Metabolism	COG0069
018.1_WP_079336601.:	CUST_5957_PI442190799	HOT208_60m_810P02 018.1_WP_079336601.:	AGCTTTGGCGCCTTTTTATTGAAGGGAATGAATATTCAATTAATCGGAGAAGCAAATGAT	glsF	Ferredoxin-dependent glutamate synthase	WP_079336601.:	70414..74985	Nitrogen Metabolism	COG0069
018.1_WP_079336633.:	CUST_6204_PI442190799	HOT208_60m_810P02 018.1_WP_079336633.:	CCCTCAAAATGAACCTGGAAGTTCTATCATGCCAGGAAAAATAAATCCGACACAATGCGA	fumC	class II fumarate hydratase	WP_079336633.:	125361..126746	Krebs cycle and related genes	COG0114
018.1_WP_079336633.:	CUST_6205_PI442190799	HOT208_60m_810P02 018.1_WP_079336633.:	AGATAATGGGTCTTGAATATGCAGTTTCAATGGCAAATTCTAGCGGCACTTTGCAGATGA	fumC	class II fumarate hydratase	WP_079336633.:	125361..126746	Krebs cycle and related genes	COG0114
018.1_WP_079336633.:	CUST_6206_PI442190799	HOT208_60m_810P02 018.1_WP_079336633.:	TACCTCTGGGAGGAACTGCAGTTGGAACAGGGATTAATTGTCCTAAAGGATTTTGTGAAG	fumC	class II fumarate hydratase	WP_079336633.:	125361..126746	Krebs cycle and related genes	COG0114
018.1_WP_079336633.:	CUST_6207_PI442190799	HOT208_60m_810P02 018.1_WP_079336633.:	CCATTGTGCCAAAAGTTGGTTATGAAAAAGCAGCTGAAATTGCAAACCTTGCCTTCAAAG	fumC	class II fumarate hydratase	WP_079336633.:	125361..126746	Krebs cycle and related genes	COG0114
018.1_WP_079336633.:	CUST_6208_PI442190799	HOT208_60m_810P02 018.1_WP_079336633.:	TCATGGCCTCGCATGACCGTCTAGCTCAAGTAATGAGTCAGATAAAAATATTAGCAGGTG	fumC	class II fumarate hydratase	WP_079336633.:	125361..126746	Krebs cycle and related genes	COG0114
018.1_WP_079336633.:	CUST_6209_PI442190799	HOT208_60m_810P02 018.1_WP_079336633.:	TTTGGGCAAGAGATATCAGCATGGTCAGAGCAACTCAAAGATGCTGAGAATGCAATTATT	fumC	class II fumarate hydratase	WP_079336633.:	125361..126746	Krebs cycle and related genes	COG0114
018.1_WP_011863567.:	CUST_6260_PI442190799	HOT208_60m_810P02 018.1_WP_011863567.:	AAAGGCCTTCAAAACTTGCTTGAGTTCCTCTGGGATTACATAAGAGATCTTGCTAGGACG	atpI	ATP synthase subunit A	WP_011863567.:	134420..135145	Energy Metabolism	COG0356
018.1_WP_011863567.:	CUST_6261_PI442190799	HOT208_60m_810P02 018.1_WP_011863567.:	ATTGTTTACTAGTGCAATTCAGGCATTGATTTTCGCAACTTTGGCGGCCTATTACATTGG	atpI	ATP synthase subunit A	WP_011863567.:	134420..135145	Energy Metabolism	COG0356
018.1_WP_011863567.:	CUST_6262_PI442190799	HOT208_60m_810P02 018.1_WP_011863567.:	TGCAGGTTTAAGCAATAAGGGTTGGAGATACTTCGAATACTATGTTCACCCAACTCCGAT	atpI	ATP synthase subunit A	WP_011863567.:	134420..135145	Energy Metabolism	COG0356
018.1_WP_011863567.:	CUST_6263_PI442190799	HOT208_60m_810P02 018.1_WP_011863567.:	ACCAAGTGGAGAATTAGGAGCACCTACTGCTGATATCAATACAACTATAGCTTTGGCTTT	atpI	ATP synthase subunit A	WP_011863567.:	134420..135145	Energy Metabolism	COG0356
018.1_WP_011863567.:	CUST_6264_PI442190799	HOT208_60m_810P02 018.1_WP_011863567.:	AGCATTAGAAGTTGGTCAACATCTTTATTGGCAAATTGGAAATATCAGACTACATGGGCA	atpI	ATP synthase subunit A	WP_011863567.:	134420..135145	Energy Metabolism	COG0356
018.1_WP_011863567.:	CUST_6265_PI442190799	HOT208_60m_810P02 018.1_WP_011863567.:	TGGATGCCGTTTATAGGTACTTTATTTTTATTCGTCTTTGTTAGTAATTGGGGAGGAGCT	atpI	ATP synthase subunit A	WP_011863567.:	134420..135145	Energy Metabolism	COG0356
018.1_WP_011863563.:	CUST_6287_PI442190799	HOT208_60m_810P02 018.1_WP_011863563.:	AGTTATTAAAGCAACCTCAATTCTCTCCGCTGAACCTTGCAGAACAAGTTGCAGTTGTTT	atpA	ATP synthase alpha subunit	WP_011863563.:	137167..138684	Energy Metabolism	COG0056
018.1_WP_011863563.:	CUST_6288_PI442190799	HOT208_60m_810P02 018.1_WP_011863563.:	TCTCAATTTGCATCTGATCTTGATGAAGCAACTCAGCAACAACTTGAACGAGGCAAAAGA	atpA	ATP synthase alpha subunit	WP_011863563.:	137167..138684	Energy Metabolism	COG0056
018.1_WP_011863563.:	CUST_6290_PI442190799	HOT208_60m_810P02 018.1_WP_011863563.:	GCTCTCCCAATTATTGAAACTCAGGCAGGAGACGTTTCTGCTTACATCCCAACTAATGTT	atpA	ATP synthase alpha subunit	WP_011863563.:	137167..138684	Energy Metabolism	COG0056
018.1_WP_011863563.:	CUST_6291_PI442190799	HOT208_60m_810P02 018.1_WP_011863563.:	TACTTACACAGTAGATTGTTAGAAAGAGCAGCAAAACTTTCTGATGCTATGGAGGGGGGC	atpA	ATP synthase alpha subunit	WP_011863563.:	137167..138684	Energy Metabolism	COG0056
018.1_WP_011863563.:	CUST_6292_PI442190799	HOT208_60m_810P02 018.1_WP_011863563.:	TAGCACCTTATACCGGTGCAGCAATTGCTGAACATTTTATGTATCAGGGTAAAGCAACAC	atpA	ATP synthase alpha subunit	WP_011863563.:	137167..138684	Energy Metabolism	COG0056
018.1_WP_079336652.:	CUST_6349_PI442190799	HOT208_60m_810P02 018.1_WP_079336652.:	ATTTAAAGCCTTGAAAGAGGTTTCAAAGGCTGAGGCTAGGATTCAAGCCGCAAAAAACTA	atpC	ATP synthase epsilon subunit	WP_079336652.:	147966..148370	Energy Metabolism	COG0355
018.1_WP_079336652.:	CUST_6350_PI442190799	HOT208_60m_810P02 018.1_WP_079336652.:	TCTTAAAGAAGCAAAATTAGCAATTAGCAAATTTTCTGAAAATGA	atpC	ATP synthase epsilon subunit	WP_079336652.:	147966..148370	Energy Metabolism	COG0355
018.1_WP_079336652.:	CUST_6351_PI442190799	HOT208_60m_810P02 018.1_WP_079336652.:	TGCTGAAATTGGTTCTGAAATTAATGTTCAAAATGCTGAACAAGATCTTAAAGAAGCAAA	atpC	ATP synthase epsilon subunit	WP_079336652.:	147966..148370	Energy Metabolism	COG0355
018.1_WP_079336652.:	CUST_6352_PI442190799	HOT208_60m_810P02 018.1_WP_079336652.:	TGGCGTTTTAAGGCTAAGAATGAATTCTCAATGGAAATCAATAGCTCTTATGGGAGGCTT	atpC	ATP synthase epsilon subunit	WP_079336652.:	147966..148370	Energy Metabolism	COG0355
018.1_WP_079336652.:	CUST_6353_PI442190799	HOT208_60m_810P02 018.1_WP_079336652.:	AGGCGAAGCTGAAGAAGTAATACTTCCAAGTACTACTGGTCAACTTGGTATACTACCTGG	atpC	ATP synthase epsilon subunit	WP_079336652.:	147966..148370	Energy Metabolism	COG0355
194.1_1277	CUST_6744_PI442190799	HOT208_60m_810P02 194.1_1277	AGGGAATCATTGGCAATGATTTTTGATCCGCCTTCGAGGAGACCAATTTTGGATCATATT	zwf	glucose-6-phosphate dehydrogenase zwf		<1..734	Pentose Phosphate	COG0364
194.1_1277	CUST_6745_PI442190799	HOT208_60m_810P02 194.1_1277	TAACACTTGTGTAATACTTGGCTGTGAATCAGTGGAATGTATGAGACTTGAAGCAGGGGG	zwf	glucose-6-phosphate dehydrogenase zwf		<1..734	Pentose Phosphate	COG0364
194.1_1277	CUST_6746_PI442190799	HOT208_60m_810P02 194.1_1277	TGGAACGGAAGCTTGGATGAGTCACCCGAAATCTTCGAATATTTCACTGATTATGGTCAA	zwf	glucose-6-phosphate dehydrogenase zwf		<1..734	Pentose Phosphate	COG0364
194.1_1277	CUST_6747_PI442190799	HOT208_60m_810P02 194.1_1277	TAAGTGATACCCCAGCTATGCAACCTATATGTGGGGATTTATTGTTATTAGGGGGGGACT	zwf	glucose-6-phosphate dehydrogenase zwf		<1..734	Pentose Phosphate	COG0364
194.1_1277	CUST_6748_PI442190799	HOT208_60m_810P02 194.1_1277	CGCGGGAGATCATATGATTCAAGCATTGTTTTCGATTTCATGGATTAGCGATAAACTTGG	zwf	glucose-6-phosphate dehydrogenase zwf		<1..734	Pentose Phosphate	COG0364
194.1_1277	CUST_6749_PI442190799	HOT208_60m_810P02 194.1_1277	TCCCTTATCTTTGGGAAATCCAAGTATTCATTTAGGACAAGTTATTGGATTGAGGCTGAT	zwf	glucose-6-phosphate dehydrogenase zwf		<1..734	Pentose Phosphate	COG0364
032.1_WP_079336733.:	CUST_7292_PI442190799	HOT208_60m_810P02 032.1_WP_079336733.:	TAATGGAACAGGAATGCATGTTCACCAAAGTTTATGGAAGAGTGGGCAGCCACTATTTTT	glnA	Glutamine synthetase/ glutamate--ammonialigase	WP_079336733.:	823..1859	Nitrogen Metabolism	COG0174
032.1_WP_079336733.:	CUST_7293_PI442190799	HOT208_60m_810P02 032.1_WP_079336733.:	GCTTTGAAATACTTGGAATCTACTGGGATTGCTGATACAGCATTTTTTGGACCGGAACCA	glnA	Glutamine synthetase/ glutamate--ammonialigase	WP_079336733.:	823..1859	Nitrogen Metabolism	COG0174
032.1_WP_079336733.:	CUST_7294_PI442190799	HOT208_60m_810P02 032.1_WP_079336733.:	TGGTGCCGGCCAACACGAACTTGGAATGAAATTCGATTCGTTAATAAATTCTGCTGATAA	glnA	Glutamine synthetase/ glutamate--ammonialigase	WP_079336733.:	823..1859	Nitrogen Metabolism	COG0174
032.1_WP_079336733.:	CUST_7295_PI442190799	HOT208_60m_810P02 032.1_WP_079336733.:	TGCTCTATCCAGGAGCCTAGAAGTGGTGAACCTTATGATAGATGTCCAAGATCTTTAGCT	glnA	Glutamine synthetase/ glutamate--ammonialigase	WP_079336733.:	823..1859	Nitrogen Metabolism	COG0174
032.1_WP_079336733.:	CUST_7296_PI442190799	HOT208_60m_810P02 032.1_WP_079336733.:	GGTTCCAGGATTCGAAGCACCTGTAAATCTAGTCTATTCTGAGGGTAATAGATCAGCTGC	glnA	Glutamine synthetase/ glutamate--ammonialigase	WP_079336733.:	823..1859	Nitrogen Metabolism	COG0174
032.1_WP_079336733.:	CUST_7297_PI442190799	HOT208_60m_810P02 032.1_WP_079336733.:	ACTGCGCAAGACATCAGATCTGAAATGCTACTTTTAATGGGTGAATTAGGCATTCCTACA	glnA	Glutamine synthetase/ glutamate--ammonialigase	WP_079336733.:	823..1859	Nitrogen Metabolism	COG0174
039.1_WP_079336757.:	CUST_7475_PI442190799	HOT208_60m_810P02 039.1_WP_079336757.:	CTACAGCTCGAAATGTAAGTAAATTCAGACCACCAACACCTATCCTGGCAACCACTACAG	pykF	Pyruvate kinase	WP_079336757.:	<1..1283	Glycolysis	COG0469
039.1_WP_079336757.:	CUST_7476_PI442190799	HOT208_60m_810P02 039.1_WP_079336757.:	TGGGGATCCTCAATCAAGGAGATTTAGTAGTTCAAACTGCAGGCACATTAACTGGCATTA	pykF	Pyruvate kinase	WP_079336757.:	<1..1283	Glycolysis	COG0469
039.1_WP_079336757.:	CUST_7477_PI442190799	HOT208_60m_810P02 039.1_WP_079336757.:	AGCAATGGCGACCTTATTACACTGCAGCTAAATGAAGGTGTTATATACATGGGGCAAATT	pykF	Pyruvate kinase	WP_079336757.:	<1..1283	Glycolysis	COG0469
039.1_WP_079336757.:	CUST_7478_PI442190799	HOT208_60m_810P02 039.1_WP_079336757.:	ATCCCAAATGCTATTAGTGCAGCAGTAAGTAATATAGCTAGACAACTTGATGCTGGAGCT	pykF	Pyruvate kinase	WP_079336757.:	<1..1283	Glycolysis	COG0469
039.1_WP_079336757.:	CUST_7479_PI442190799	HOT208_60m_810P02 039.1_WP_079336757.:	AGAGAGAAGTGTAGCGAGAAGATTGCAACTTGTTTGGGGTGTTACTCCCATAGTAGTT	pykF	Pyruvate kinase	WP_079336757.:	<1..1283	Glycolysis	COG0469
039.1_WP_079336757.:	CUST_7480_PI442190799	HOT208_60m_810P02 039.1_WP_079336757.:	AGAAAGATTGTATCAAGAGGAATTTCAATAGGGGAAATCGGTGTTACAGGTAAAGCGAGA	pykF	Pyruvate kinase	WP_079336757.:	<1..1283	Glycolysis	COG0469
046.1_WP_079336769.:	CUST_7573_PI442190799	HOT208_60m_810P02 046.1_WP_079336769.:	AATGCGGGGATCATGGCATGATAAACGAATAAGAATTTGTTATTACGAACAGTGGCCC	kaiC	circadian clock protein KaiC	WP_079336769.:	437..1966	Circadian Rhythm	COG0467
046.1_WP_079336769.:	CUST_7574_PI442190799	HOT208_60m_810P02 046.1_WP_079336769.:	TATGTGTGGTGGAGGATATTTTCAAGATTCCATTATTCTCGCGACTGGAGCAACTGGTAC	kaiC	circadian clock protein KaiC	WP_079336769.:	437..1966	Circadian Rhythm	COG0467
046.1_WP_079336769.:	CUST_7575_PI442190799	HOT208_60m_810P02 046.1_WP_079336769.:	ATACTTTTTGCGTATGAAGAATCTAGGGCTCAATTGCTTAGGAATGCTACTAGCTGGGGT	kaiC	circadian clock protein KaiC	WP_079336769.:	437..1966	Circadian Rhythm	COG0467
046.1_WP_079336769.:	CUST_7576_PI442190799	HOT208_60m_810P02 046.1_WP_079336769.:	TGGCAATTGATTCTCTCTCTGCATTAGCCAGAGGTGTAAGTTTGAATGCATTTAGACAGT	kaiC	circadian clock protein KaiC	WP_079336769.:	437..1966	Circadian Rhythm	COG0467
046.1_WP_079336769.:	CUST_7577_PI442190799	HOT208_60m_810P02 046.1_WP_079336769.:	TTTAGTGGTGCCCCTCACAGAGTTATACCTGATCAAAATGTCCAAAATGTTTTTAAAGGA	kaiC	circadian clock protein KaiC	WP_079336769.:	437..1966	Circadian Rhythm	COG0467
046.1_WP_079336769.:	CUST_7578_PI442190799	HOT208_60m_810P02 046.1_WP_079336769.:	TACAGACACCATATTGTTACTTCAATATGTAGAAATAAAAGGTGAGATGGCACGAGCTTT	kaiC	circadian clock protein KaiC	WP_079336769.:	437..1966	Circadian Rhythm	COG0467
046.1_WP_079336770.:	CUST_7579_PI442190799	HOT208_60m_810P02 046.1_WP_079336770.:	AGTTTTAATTGGTTTAGATCTACTTTTTGATGAATTAACTGAAACTGAATATAGTGGAGA	kaiB	Circadian clock protein KaiB	WP_079336770.:	2038..2355	Circadian Rhythm	COG4251
046.1_WP_079336770.:	CUST_7580_PI442190799	HOT208_60m_810P02 046.1_WP_079336770.:	AGCTACTCCAACATTAGCTAAAATTTTACCGCCACCAGTTAGAAAAATAATAGGTGATCT	kaiB	Circadian clock protein KaiB	WP_079336770.:	2038..2355	Circadian Rhythm	COG4251
046.1_WP_079336770.:	CUST_7581_PI442190799	HOT208_60m_810P02 046.1_WP_079336770.:	AGGCGTTTATGCTTTGAAGGTTATCGATGTACTTAAGCAACCACAACTTGCAGAAGAAGA	kaiB	Circadian clock protein KaiB	WP_079336770.:	2038..2355	Circadian Rhythm	COG4251
046.1_WP_079336770.:	CUST_7582_PI442190799	HOT208_60m_810P02 046.1_WP_079336770.:	TACTCCTAATTCGATGAGAGCCTTAAATACCTTAAGAGAAATTTTA	kaiB	Circadian clock protein KaiB	WP_079336770.:	2038..2355	Circadian Rhythm	COG4251
046.1_WP_079336770.:	CUST_7583_PI442190799	HOT208_60m_810P02 046.1_WP_079336770.:	TGGCGGCAAGGAAAACATATATTTTAAAACTCTATGTTGCTGGAAATACTCCTAATTCGA	kaiB	Circadian clock protein KaiB	WP_079336770.:	2038..2355	Circadian Rhythm	COG4251
008.1_1501	CUST_7904_PI442190799	HOT208_60m_810P02 008.1_1501	CATTGGAGCTGGATATGTAGGGGGCCCTACGATGGCTGTAATTGCTTATAAATGTAGAAA	ugd	UDP-glucose 6-dehydrogenase/nucleotide sugar dehydrogenase		<1..435	Amino sugar and nucleotide sugar r	COG1004
008.1_1501	CUST_7905_PI442190799	HOT208_60m_810P02 008.1_1501	GTTTAGGGGCTGGCGAGGCAAGTGACTTGAAATATATTGAGAGTTCTGCAAGATCAATTG	ugd	UDP-glucose 6-dehydrogenase/nucleotide sugar dehydrogenase		<1..435	Amino sugar and nucleotide sugar r	COG1004
008.1_1501	CUST_7906_PI442190799	HOT208_60m_810P02 008.1_1501	TGCTGTTGGACATACAATAGTCGTTGAAAAAAGTACAATCCCAGTAAAAACAGCAGAAGT	ugd	UDP-glucose 6-dehydrogenase/nucleotide sugar dehydrogenase		<1..435	Amino sugar and nucleotide sugar r	COG1004
008.1_1501	CUST_7907_PI442190799	HOT208_60m_810P02 008.1_1501	AATCAATTTCCGAATCAGATATGGTTTTCATTTCTGTAAATACACCAACCAAACAGAGCG	ugd	UDP-glucose 6-dehydrogenase/nucleotide sugar dehydrogenase		<1..435	Amino sugar and nucleotide sugar r	COG1004
008.1_1501	CUST_7908_PI442190799	HOT208_60m_810P02 008.1_1501	TCTTGGAATAACGAGGACTTAACTAAATTGCCAGTCTATGAACCAGGATTAAAAGATTTA	ugd	UDP-glucose 6-dehydrogenase/nucleotide sugar dehydrogenase		<1..435	Amino sugar and nucleotide sugar r	COG1004
008.1_1501	CUST_7909_PI442190799	HOT208_60m_810P02 008.1_1501	TAAAAGATTTAATAAGAATATGCAGAGGTAAAAACTTATTTTTTAGCAC	ugd	UDP-glucose 6-dehydrogenase/nucleotide sugar dehydrogenase		<1..435	Amino sugar and nucleotide sugar r	COG1004
107.1_60	CUST_306_PI442190723	HOT208_60m_810B23 107.1_60	CAACGGACTCCTGATAGCCCTAAGTGGTTTGAAGGTACTGCTGATGCTGTAAGAAAATAC	glgC	Glucose-1-phosphate adenylyltransferase		<1..466	Glycogen Metabolism	COG0448
107.1_60	CUST_307_PI442190723	HOT208_60m_810B23 107.1_60	TCCTCGGAGGAGGAAAAGGATCTAGACTTTACCCTTTAACAAAAATGAGGGCAAAACCTG	glgC	Glucose-1-phosphate adenylyltransferase		<1..466	Glycogen Metabolism	COG0448
107.1_60	CUST_308_PI442190723	HOT208_60m_810B23 107.1_60	TGCATCTCTAAATAGACATATAGGAAGAACCTATAATTTAAATGGCCCTTTCGGTCAGGG	glgC	Glucose-1-phosphate adenylyltransferase		<1..466	Glycogen Metabolism	COG0448
107.1_60	CUST_309_PI442190723	HOT208_60m_810B23 107.1_60	CCTGCGGTGCCGTTAGCAGGTAAGTATCGTTTAATAGATATCCCAATAAGTAATTG	glgC	Glucose-1-phosphate adenylyltransferase		<1..466	Glycogen Metabolism	COG0448
107.1_60	CUST_310_PI442190723	HOT208_60m_810B23 107.1_60	TGGGATGTTGATGAATATTTAATATTGTCAGGTGATCAACTGTATAGAATGGACTACAGT	glgC	Glucose-1-phosphate adenylyltransferase		<1..466	Glycogen Metabolism	COG0448
107.1_60	CUST_311_PI442190723	HOT208_60m_810B23 107.1_60	ATAGAGATAATGGTGCTGATTTAACTGTTGCAGCTTTGCCTGTTGATGAAGCTCAAGCAG	glgC	Glucose-1-phosphate adenylyltransferase		<1..466	Glycogen Metabolism	COG0448
010.1_WP_079298148.:	CUST_469_PI442190723	HOT208_60m_810B23 010.1_WP_079298148.:	AGCAACTCCTACGTTAGCTAAAATTTTACCGCCACCAGTTAGAAAAATAATAGGTGACCT	kaiB	Circadian clock protein KaiB	WP_079298148.:	21349..21666	Circadian Rhythm	COG4251
010.1_WP_079298148.:	CUST_470_PI442190723	HOT208_60m_810B23 010.1_WP_079298148.:	CAAGGGAGTTTATGCTTTGAAGGTTATCGATGTACTTAAGCAACCACAACTTGCAGAAGA	kaiB	Circadian clock protein KaiB	WP_079298148.:	21349..21666	Circadian Rhythm	COG4251
010.1_WP_079298148.:	CUST_471_PI442190723	HOT208_60m_810B23 010.1_WP_079298148.:	AACTCTATGTTGCTGGAAATACTCCTAATTCGATGAGAGCTTTAAATACCTTAAGAGAAA	kaiB	Circadian clock protein KaiB	WP_079298148.:	21349..21666	Circadian Rhythm	COG4251
010.1_WP_079298150.:	CUST_472_PI442190723	HOT208_60m_810B23 010.1_WP_079298150.:	TTTTGCATATGAAGAATCCAGGGCTCAATTGCTTAGGAATGCTACTAGCTGGGGAATAGA	kaiC	circadian clock protein KaiC	WP_079298150.:	21737..23272	Circadian Rhythm	COG0467
010.1_WP_079298150.:	CUST_473_PI442190723	HOT208_60m_810B23 010.1_WP_079298150.:	TGATGATATGTGTGGTGGAGGTTATTTTCAAGATTCCATCATTCTCGCCACTGGAGCTAC	kaiC	circadian clock protein KaiC	WP_079298150.:	21737..23272	Circadian Rhythm	COG0467
010.1_WP_079298150.:	CUST_474_PI442190723	HOT208_60m_810B23 010.1_WP_079298150.:	CCAAAAAGAATGGCAATTGATTCTCTCTCTGCCTTAGCCAGAGGTGTAAGTTTGAATGCA	kaiC	circadian clock protein KaiC	WP_079298150.:	21737..23272	Circadian Rhythm	COG0467
010.1_WP_079298150.:	CUST_475_PI442190723	HOT208_60m_810B23 010.1_WP_079298150.:	AGGTGAGATGGCAAGAGCTTTAAATGTATTTAAAATGCGGGGATCATGGCATGATAAACG	kaiC	circadian clock protein KaiC	WP_079298150.:	21737..23272	Circadian Rhythm	COG0467
010.1_WP_079298150.:	CUST_476_PI442190723	HOT208_60m_810B23 010.1_WP_079298150.:	TGCTGTCACTGGTTATACAAAGCAAGAGGAGATAGCAGGCTTTTTACTAATACTGCAGA	kaiC	circadian clock protein KaiC	WP_079298150.:	21737..23272	Circadian Rhythm	COG0467
010.1_WP_079298150.:	CUST_477_PI442190723	HOT208_60m_810B23 010.1_WP_079298150.:	TGGAAAGTGATGGGTTGTTGAAAATTATTTGTGCGTATCCTGAATCAACTGGTTTAGAAG	kaiC	circadian clock protein KaiC	WP_079298150.:	21737..23272	Circadian Rhythm	COG0467
010.1_WP_079298216.:	CUST_585_PI442190723	HOT208_60m_810B23 010.1_WP_079298216.:	TACTTCCTGATGCAATAGACGAAGACCCAGAGAAACCAGCAGGATTATATACTGCTTGGA	glcH	GPH family sugar transporter	WP_079298216.:	41053..42399	GlcH sugar transporter	COG2211
010.1_WP_079298216.:	CUST_586_PI442190723	HOT208_60m_810B23 010.1_WP_079298216.:	TTTTTATAATCTGTCTAATTGGCATTGGAGCTTCAACCGCTTTTCTTATCCCTTGGTCAC	glcH	GPH family sugar transporter	WP_079298216.:	41053..42399	GlcH sugar transporter	COG2211
010.1_WP_079298216.:	CUST_587_PI442190723	HOT208_60m_810B23 010.1_WP_079298216.:	TAGGATTTCAGCATTAAACTATGGAGCGATTATGTGGATTTTTTCATGTACAGCAGCTTT	glcH	GPH family sugar transporter	WP_079298216.:	41053..42399	GlcH sugar transporter	COG2211
010.1_WP_079298216.:	CUST_588_PI442190723	HOT208_60m_810B23 010.1_WP_079298216.:	GCGAAGTGGATCCCAATACCTTTCCAAATAAGCGCTTTAGTAGGTTTACAAATATGGACC	glcH	GPH family sugar transporter	WP_079298216.:	41053..42399	GlcH sugar transporter	COG2211
010.1_WP_079298216.:	CUST_589_PI442190723	HOT208_60m_810B23 010.1_WP_079298216.:	TCTTCTCTGGTGCGCCTTACAATTAATGCAAACAGTAGCGTTAATCTATGTTGAGGATGT	glcH	GPH family sugar transporter	WP_079298216.:	41053..42399	GlcH sugar transporter	COG2211
010.1_WP_079298216.:	CUST_590_PI442190723	HOT208_60m_810B23 010.1_WP_079298216.:	GCAACATTAATTTCTTGTTGGGGATTAGCTCCATTCGCAAAAAAAGCAAGAAGGCCTTCA	glcH	GPH family sugar transporter	WP_079298216.:	41053..42399	GlcH sugar transporter	COG2211
010.1_WP_079298199.:	CUST_662_PI442190723	HOT208_60m_810B23 010.1_WP_079298199.:	AGTTAATGTTGATTTTGCAGACGTAAGATCAGTTATGACTGAAGCTGGCACTGCTCTCCT	ftsZ	cell division protein FtsZ	WP_079298199.:	53891..55006	Cell Division	COG0206
010.1_WP_079298199.:	CUST_663_PI442190723	HOT208_60m_810B23 010.1_WP_079298199.:	TTGTTGATCAAGAAGCAAATATTATTGTTGGTGCTGTGGTAGATGAGGCAATGGAAGGGG	ftsZ	cell division protein FtsZ	WP_079298199.:	53891..55006	Cell Division	COG0206
010.1_WP_079298199.:	CUST_664_PI442190723	HOT208_60m_810B23 010.1_WP_079298199.:	ATGCTGATGATGTTTTAAGGATGGGCGTCAAAGGCATAAGTGACATAATTACTTGCCCAG	ftsZ	cell division protein FtsZ	WP_079298199.:	53891..55006	Cell Division	COG0206
010.1_WP_079298199.:	CUST_665_PI442190723	HOT208_60m_810B23 010.1_WP_079298199.:	GCAAGCAGAGGAGGGGATCGCCAGATTAGCTGAAAACGTTGATACTCTTATAGTTATTCC	ftsZ	cell division protein FtsZ	WP_079298199.:	53891..55006	Cell Division	COG0206
010.1_WP_079298199.:	CUST_666_PI442190723	HOT208_60m_810B23 010.1_WP_079298199.:	AGCCAAACAAAGTGGGGCTCTAACAGTGGGTATAGTAACCAAACCATTTTCTTTTGAAGG	ftsZ	cell division protein FtsZ	WP_079298199.:	53891..55006	Cell Division	COG0206
010.1_WP_079298199.:	CUST_667_PI442190723	HOT208_60m_810B23 010.1_WP_079298199.:	ACAGGCAGCAATGAATAGTCCTTTGTTAGAAGCAGCTAGTATTGATGGAGCTAAAGGCTG	ftsZ	cell division protein FtsZ	WP_079298199.:	53891..55006	Cell Division	COG0206
109.1_129	CUST_691_PI442190723	HOT208_60m_810B23 109.1_129	GGCAATGGCAGTAGATACTTCAAAATTTGGATTAAGTAAGGAGTCAGCTGCCGACAAACC	glgC	Glucose-1-phosphate adenylyltransferase		<1..429	Glycogen Metabolism	COG0448
109.1_129	CUST_692_PI442190723	HOT208_60m_810B23 109.1_129	ACGGATTTTGGTAAGGACATAATTCCAGAAGCCCTGAATAGAGGCGATACTCTTAAAAGT	glgC	Glucose-1-phosphate adenylyltransferase		<1..429	Glycogen Metabolism	COG0448
109.1_129	CUST_693_PI442190723	HOT208_60m_810B23 109.1_129	TGGCCTAATGAGAACCGATGATGTAGGAAATATAAAAGAATTTAGTGAAAAGCCAACTGG	glgC	Glucose-1-phosphate adenylyltransferase		<1..429	Glycogen Metabolism	COG0448
109.1_129	CUST_694_PI442190723	HOT208_60m_810B23 109.1_129	TCTAGCCTCTATGGGTATTTACGTTTTTAGTAGAAATACTCTTTTCGATCTTCTAAATAA	glgC	Glucose-1-phosphate adenylyltransferase		<1..429	Glycogen Metabolism	COG0448
109.1_129	CUST_695_PI442190723	HOT208_60m_810B23 109.1_129	ATAGAGATAATGGTGCTGATTTAACTGTTGCAGCTTTGCCTGTTGATGAAGCTCAAGCAG	glgC	Glucose-1-phosphate adenylyltransferase		<1..429	Glycogen Metabolism	COG0448
109.1_129	CUST_696_PI442190723	HOT208_60m_810B23 109.1_129	ATTTAATATTGTCAGGTGATCAACTGTATAGAATGGACTACAGTTTATTTGTTCAACATC	glgC	Glucose-1-phosphate adenylyltransferase		<1..429	Glycogen Metabolism	COG0448
011.1_WP_025889299.:	CUST_935_PI442190723	HOT208_60m_810B23 011.1_WP_025889299.:	CCAGAGAAAATTAAAGCCTTAAAAGAGATTTCAAAGGCTGAGGCTAGGATTCAAGCTGCA	atpC	ATP synthase epsilon subunit	WP_025889299.:	33922..34326	Energy Metabolism	COG0355
011.1_WP_025889299.:	CUST_936_PI442190723	HOT208_60m_810B23 011.1_WP_025889299.:	TGCTGAAATTGGTTCTGAAATTAATGTTCAAAATGCTGAACAAGATCTTAAAGAAGCAAA	atpC	ATP synthase epsilon subunit	WP_025889299.:	33922..34326	Energy Metabolism	COG0355
011.1_WP_025889299.:	CUST_937_PI442190723	HOT208_60m_810B23 011.1_WP_025889299.:	TGAATTCCCAATGGAAATCAATAGCCCTTATGGGAGGCTTCGCTGAAATTGAATCAGATG	atpC	ATP synthase epsilon subunit	WP_025889299.:	33922..34326	Energy Metabolism	COG0355
011.1_WP_025889299.:	CUST_938_PI442190723	HOT208_60m_810B23 011.1_WP_025889299.:	TGTACTACCAGGACATATCTCTTTAGTTACTGCTATAGATATTGGCGT	atpC	ATP synthase epsilon subunit	WP_025889299.:	33922..34326	Energy Metabolism	COG0355
011.1_WP_025889299.:	CUST_939_PI442190723	HOT208_60m_810B23 011.1_WP_025889299.:	AGGCGAAGCTGAAGAAGTAATCCTTCCTAGTACTACTGGTCAAATTGGTGTACTACCAGG	atpC	ATP synthase epsilon subunit	WP_025889299.:	33922..34326	Energy Metabolism	COG0355
120.1_187	CUST_961_PI442190723	HOT208_60m_810B23 120.1_187	CCAGGTGGTACGAAGGCATCATATGATGCAATAGAGAGCTTATTAACAAAAATGGCTGCC	edd	phosphogluconate dehydrogenase (NADP(+)-dependent, decarboxylating)		<14..609	Pentose Phosphate/Entner-Dudorof	COG2084
120.1_187	CUST_962_PI442190723	HOT208_60m_810B23 120.1_187	TACTCTTGAAAGTAAAAGTTTTGGATACATTGGGATGGGAGTCTCTGGAGGTGCCAAAGG	edd	phosphogluconate dehydrogenase (NADP(+)-dependent, decarboxylating)		<14..609	Pentose Phosphate/Entner-Dudorof	COG2084
120.1_187	CUST_963_PI442190723	HOT208_60m_810B23 120.1_187	TATGTTGGCCCAGGCGGCTCAGGTCATTTTGTAAAAACTGTTCATAACGGAATTGAATAT	edd	phosphogluconate dehydrogenase (NADP(+)-dependent, decarboxylating)		<14..609	Pentose Phosphate/Entner-Dudorof	COG2084
120.1_187	CUST_964_PI442190723	HOT208_60m_810B23 120.1_187	GGGCCCGCAACAGATGCTGTCATTGATAATATTTCTGGATATCTCGAGGAAGGAGATTTA	edd	phosphogluconate dehydrogenase (NADP(+)-dependent, decarboxylating)		<14..609	Pentose Phosphate/Entner-Dudorof	COG2084
120.1_187	CUST_965_PI442190723	HOT208_60m_810B23 120.1_187	TGCAGAAGCTTATGACCTTATGAAGAGAGTCAAAGGTATGAATGGAAAGCAGATGTCAGA	edd	phosphogluconate dehydrogenase (NADP(+)-dependent, decarboxylating)		<14..609	Pentose Phosphate/Entner-Dudorof	COG2084
120.1_187	CUST_966_PI442190723	HOT208_60m_810B23 120.1_187	TGAGATAACAGAGATTTGTCTAAAAACAAAAGATGAGATAACTGGAGATGATGTCGTGGA	edd	phosphogluconate dehydrogenase (NADP(+)-dependent, decarboxylating)		<14..609	Pentose Phosphate/Entner-Dudorof	COG2084
140.1_284	CUST_1410_PI442190723	HOT208_60m_810B23 140.1_284	TGCGTTCCTGAATCTGTTCCTGAAATAGTTTTCTTATCATGAGGGCTAAGCGAAGAAGCT	fda/cbbA	fructose-1,6-bisphosphatealdolase		<1..446	Glycolysis/Gluconeogenesis	COG0191
140.1_284	CUST_1411_PI442190723	HOT208_60m_810B23 140.1_284	TATGGAATGTTTCATTCTCCTATGGAAGAGCATTACAGCACTCATGCTTAAAGGCCTGGA	fda/cbbA	fructose-1,6-bisphosphatealdolase		<1..446	Glycolysis/Gluconeogenesis	COG0191
140.1_284	CUST_1412_PI442190723	HOT208_60m_810B23 140.1_284	CGACGTTGAAGGAGGTCAAAAAGCATTAATAGCAAGAGCTCAAGCAAACTCTGAAGCTTC	fda/cbbA	fructose-1,6-bisphosphatealdolase		<1..446	Glycolysis/Gluconeogenesis	COG0191
140.1_284	CUST_1413_PI442190723	HOT208_60m_810B23 140.1_284	TCTATGACTGTTAATGGAGCAGATTGTCCAACAAAGGCTGATCCTATGAAGGTTGCTGAA	fda/cbbA	fructose-1,6-bisphosphatealdolase		<1..446	Glycolysis/Gluconeogenesis	COG0191
140.1_284	CUST_1414_PI442190723	HOT208_60m_810B23 140.1_284	TGCTGAAGTTCAAGAAGAGGTAATAAAGCAGGTCTATATTGCCTGTCAAGCAAATGGAGT	fda/cbbA	fructose-1,6-bisphosphatealdolase		<1..446	Glycolysis/Gluconeogenesis	COG0191
140.1_284	CUST_1415_PI442190723	HOT208_60m_810B23 140.1_284	TAAGCGAAGAAGCTGCTTCTGTTTATCTAAATAATATGAACACTCT	fda/cbbA	fructose-1,6-bisphosphatealdolase		<1..446	Glycolysis/Gluconeogenesis	COG0191
146.1_291	CUST_1447_PI442190723	HOT208_60m_810B23 146.1_291	CCAATTATTGAACCTGAAATCTTAATGGACGGCGACCATACTATTGAAAAAACTGCA	fda/cbbA	fructose-1,6-bisphosphatealdolase		<1..201	Glycolysis/Gluconeogenesis	COG0191
146.1_291	CUST_1448_PI442190723	HOT208_60m_810B23 146.1_291	GAGAATGCTTGGGGATTAGCAAGGTATGCAAGATCAGTTCAAGAATCTGGGTTAGTACCA	fda/cbbA	fructose-1,6-bisphosphatealdolase		<1..201	Glycolysis/Gluconeogenesis	COG0191
146.1_291	CUST_1449_PI442190723	HOT208_60m_810B23 146.1_291	TATGAACAAGGTGCCAGATTTGCAAAGTGGAGAGCAGTTCTGCAAATTACAAATGATGGT	fda/cbbA	fructose-1,6-bisphosphatealdolase		<1..201	Glycolysis/Gluconeogenesis	COG0191
197.1_384	CUST_1861_PI442190723	HOT208_60m_810B23 197.1_384	TTTGCCACAATTGCTAGCTATGCCCAAGGTATGGATATCTTAAGAGAAGCATCTGCAGTA	edd	phosphogluconate dehydrogenase (NADP(+)-dependent, decarboxylating)		<1..592	Pentose Phosphate/Entner-Dudorof	COG2084
197.1_384	CUST_1862_PI442190723	HOT208_60m_810B23 197.1_384	ATGGACTGTTGTAAGTGCTCTAGAACTGGGGGTATCAGTCCCAACTATTTATGCATCTCT	edd	phosphogluconate dehydrogenase (NADP(+)-dependent, decarboxylating)		<1..592	Pentose Phosphate/Entner-Dudorof	COG2084
197.1_384	CUST_1863_PI442190723	HOT208_60m_810B23 197.1_384	ACCAATAGACTTCCTCAGAATCTTGTTCAGGCAATGAGAGATTGTTTTGGCTCTCATACA	edd	phosphogluconate dehydrogenase (NADP(+)-dependent, decarboxylating)		<1..592	Pentose Phosphate/Entner-Dudorof	COG2084
197.1_384	CUST_1864_PI442190723	HOT208_60m_810B23 197.1_384	AACTATGGGTTGAATATGCCGTCAATTGCTCAAATATGGAAAGGTGGTTGCATAATTAGA	edd	phosphogluconate dehydrogenase (NADP(+)-dependent, decarboxylating)		<1..592	Pentose Phosphate/Entner-Dudorof	COG2084
197.1_384	CUST_1865_PI442190723	HOT208_60m_810B23 197.1_384	TTGGCTAAAGTAGTTTCTCTATCCACAAAGGCAGGTATACCAGTTCCATGTTTATCAAGT	edd	phosphogluconate dehydrogenase (NADP(+)-dependent, decarboxylating)		<1..592	Pentose Phosphate/Entner-Dudorof	COG2084
197.1_384	CUST_1866_PI442190723	HOT208_60m_810B23 197.1_384	TGCAAGAGTAATGAGTTCTTTAAAAGAGCAACGTAGTGAGATTGAAAAACTATTCCATC	edd	phosphogluconate dehydrogenase (NADP(+)-dependent, decarboxylating)		<1..592	Pentose Phosphate/Entner-Dudorof	COG2084
215.1_WP_079299910.:	CUST_2020_PI442190723	HOT208_60m_810B23 215.1_WP_079299910.:	GTTCTAGCCAGCGAAGGAGGACACGTTGAATACTCTCCAAAGTCAAAATTAGAATGGGAA	glk	Glucokinase	WP_079299910.:	5053..6087	Glycolysis	COG0837
215.1_WP_079299910.:	CUST_2021_PI442190723	HOT208_60m_810B23 215.1_WP_079299910.:	TTGGTTAGGCGCTTATGCCTCTTTATTGGGAGATGTTGCTCTTCAAGAATTATGCTTTGG	glk	Glucokinase	WP_079299910.:	5053..6087	Glycolysis	COG0837
215.1_WP_079299910.:	CUST_2022_PI442190723	HOT208_60m_810B23 215.1_WP_079299910.:	AATTATTAGCGGCACTGGTTTATCAAGAATTGCCGAATGGAGACTAAGCAAACCTGATGC	glk	Glucokinase	WP_079299910.:	5053..6087	Glycolysis	COG0837
215.1_WP_079299910.:	CUST_2023_PI442190723	HOT208_60m_810B23 215.1_WP_079299910.:	ATCCAAAATGGATCCCATTCAGAAGGTACTAACAATGATTTGCATGCCATTGTTGGAGCC	glk	Glucokinase	WP_079299910.:	5053..6087	Glycolysis	COG0837
215.1_WP_079299910.:	CUST_2024_PI442190723	HOT208_60m_810B23 215.1_WP_079299910.:	TGAAAGTAATTTTAGATGAAGAGTTTGGACTTTTTAGTGCAGCCTGCAGAGCAAAAATGC	glk	Glucokinase	WP_079299910.:	5053..6087	Glycolysis	COG0837
215.1_WP_079299910.:	CUST_2025_PI442190723	HOT208_60m_810B23 215.1_WP_079299910.:	GCGGGTTATGGATTTCTGGAGGAACCGCATCAAAACATTTCAAAAACTTTAAATCAGAT	glk	Glucokinase	WP_079299910.:	5053..6087	Glycolysis	COG0837
220.1_WP_079300035.:	CUST_2610_PI442190723	HOT208_60m_810B23 220.1_WP_079300035.:	TCCCCTTACTTTTGGGCAAGAAATATCAGGATGGTCAGAGCAACTTAAAGATGCTGAGAA	fumC	class II fumarate hydratase	WP_079300035.:	1525..2603	Krebs cycle and related genes	COG0114
220.1_WP_079300035.:	CUST_2611_PI442190723	HOT208_60m_810B23 220.1_WP_079300035.:	TCAAAATGAACCTGGAAGTTCTATTATGCCGGGTAAAGTGAATCCAACTCAATGCGAAGC	fumC	class II fumarate hydratase	WP_079300035.:	1525..2603	Krebs cycle and related genes	COG0114
220.1_WP_079300035.:	CUST_2612_PI442190723	HOT208_60m_810B23 220.1_WP_079300035.:	CATGGCCTCGCATGATCGTCTTGCTCAAGTAATGAGTCAGATAAAAATATTAGCAGGTGC	fumC	class II fumarate hydratase	WP_079300035.:	1525..2603	Krebs cycle and related genes	COG0114
220.1_WP_079300035.:	CUST_2613_PI442190723	HOT208_60m_810B23 220.1_WP_079300035.:	ACCTCTGGGAGGAACTGCAGTTGGTACAGGAATTAATTGTCCAAAAGGATTTTGTGAAGA	fumC	class II fumarate hydratase	WP_079300035.:	1525..2603	Krebs cycle and related genes	COG0114
220.1_WP_079300035.:	CUST_2614_PI442190723	HOT208_60m_810B23 220.1_WP_079300035.:	TGCAGCTTTAAAAACTAATTCAGAGCTTGGTAGCCATCAACCAATTCATCCGAATGATGA	fumC	class II fumarate hydratase	WP_079300035.:	1525..2603	Krebs cycle and related genes	COG0114
220.1_WP_079300035.:	CUST_2615_PI442190723	HOT208_60m_810B23 220.1_WP_079300035.:	AAAAAGTGAAGAATGGAAAGACCTTATAAAGATAGGAAGAACCCATTTTCAAGATGCAGT	fumC	class II fumarate hydratase	WP_079300035.:	1525..2603	Krebs cycle and related genes	COG0114
222.1_WP_079300045.:	CUST_2704_PI442190723	HOT208_60m_810B23 222.1_WP_079300045.:	GCCACAGGAAGTAGCTTAATGCCGCAGAAAAAAAATCCTGACGTTCCAGAATTGATAAGA	argH	Argininosuccinate lyase	WP_079300045.:	2186..3565	Amino Acid metabolism	COG0165
222.1_WP_079300045.:	CUST_2705_PI442190723	HOT208_60m_810B23 222.1_WP_079300045.:	GAGTGTATGGACATCTTCAGGCATTGTTAACGATGGTCAAAGGGGTACCGCTTTCTTATA	argH	Argininosuccinate lyase	WP_079300045.:	2186..3565	Amino Acid metabolism	COG0165
222.1_WP_079300045.:	CUST_2706_PI442190723	HOT208_60m_810B23 222.1_WP_079300045.:	TGTTCCTTTTAGGACCGCTTATCAAGTTGTTGGTCAAATGGTTAAATATTGCCTGGAGAG	argH	Argininosuccinate lyase	WP_079300045.:	2186..3565	Amino Acid metabolism	COG0165
222.1_WP_079300045.:	CUST_2707_PI442190723	HOT208_60m_810B23 222.1_WP_079300045.:	TGTCGTTAAATCAAGAAATAGCGAAGGTGGTACAGGTTTTGTTCAAGTAGAAAAAGAGGT	argH	Argininosuccinate lyase	WP_079300045.:	2186..3565	Amino Acid metabolism	COG0165
222.1_WP_079300045.:	CUST_2708_PI442190723	HOT208_60m_810B23 222.1_WP_079300045.:	CAGTATCGATGCAGTGAGTGATAGAGATTTTTGTATTGAGTTTGTTTCTGCATCTGCTAT	argH	Argininosuccinate lyase	WP_079300045.:	2186..3565	Amino Acid metabolism	COG0165
222.1_WP_079300045.:	CUST_2709_PI442190723	HOT208_60m_810B23 222.1_WP_079300045.:	TCTGATTCTGTAGAAAACGACTTTTCTAATGCTACTGATTTGGCAGATTACTTAGTTGAT	argH	Argininosuccinate lyase	WP_079300045.:	2186..3565	Amino Acid metabolism	COG0165
232.1_601	CUST_3017_PI442190723	HOT208_60m_810B23 232.1_601	CTAATACACTTCCAAAATGGCCACTAGCTCAGCCCATGAGATTTTTGGGTCATAATGGTG	glnA	Glutamine synthetase/ glutamate--ammonialigase		<1..969	Nitrogen Metabolism	COG0174
232.1_601	CUST_3018_PI442190723	HOT208_60m_810B23 232.1_601	CAAATCTTGATGCAACCCTTGAAATCAATATTCGTTCAGGTCAGCCCATCACTGACTCAT	glnA	Glutamine synthetase/ glutamate--ammonialigase		<1..969	Nitrogen Metabolism	COG0174
232.1_601	CUST_3019_PI442190723	HOT208_60m_810B23 232.1_601	GAGGAGATAGTGACTCAGGAGATGGAGCAGGCATTTTATGTTCAATTCCATGGGGATATC	glnA	Glutamine synthetase/ glutamate--ammonialigase		<1..969	Nitrogen Metabolism	COG0174
232.1_601	CUST_3020_PI442190723	HOT208_60m_810B23 232.1_601	TGGAGAACAGTACCTGTTAATAATGAAATCTTAGGTCCTTTAGCTAAAGCAAATGCTCCA	glnA	Glutamine synthetase/ glutamate--ammonialigase		<1..969	Nitrogen Metabolism	COG0174
232.1_601	CUST_3021_PI442190723	HOT208_60m_810B23 232.1_601	AGGCAATATTAATTGGGCTAAAGCTTCAGAAACACATATAGATGATTTTGGGGAGAGTT	glnA	Glutamine synthetase/ glutamate--ammonialigase		<1..969	Nitrogen Metabolism	COG0174
232.1_601	CUST_3022_PI442190723	HOT208_60m_810B23 232.1_601	CAAAAACGATGTTGGAGATTGTGAATTTTATTTTGCCTCACTAAGTTCTCAAACAGTTGT	glnA	Glutamine synthetase/ glutamate--ammonialigase		<1..969	Nitrogen Metabolism	COG0174
251.1_674	CUST_3351_PI442190723	HOT208_60m_810B23 251.1_674	ACAAAACAGCACTAATTATTTTGCTGATCTTTACGCAAAAGGTGATTTGGATCCAAAAGT	fda/cbbA	fructose-1,6-bisphosphatealdolase		<1..296	Glycolysis/Gluconeogenesis	COG0191
251.1_674	CUST_3352_PI442190723	HOT208_60m_810B23 251.1_674	AGCAAGTTTGTGTCTTGATAGATACAAGCAGTTCTGGTGCGAAGGTCAAGCAAGTAAGATCA	fda/cbbA	fructose-1,6-bisphosphatealdolase		<1..296	Glycolysis/Gluconeogenesis	COG0191
251.1_674	CUST_3353_PI442190723	HOT208_60m_810B23 251.1_674	AGCATTTGCTGATAAGGCAAACTTCGACCCAAGACATTTCAACAAGCCTGCTAGAAAATA	fda/cbbA	fructose-1,6-bisphosphatealdolase		<1..296	Glycolysis/Gluconeogenesis	COG0191
251.1_674	CUST_3354_PI442190723	HOT208_60m_810B23 251.1_674	TAGAAAAGTCAACATTGATACTGATAACAGACTTGCTTTCACTGCCGCGGTTAGAGAGGC	fda/cbbA	fructose-1,6-bisphosphatealdolase		<1..296	Glycolysis/Gluconeogenesis	COG0191
039.1_WP_079298496.:	CUST_3990_PI442190723	HOT208_60m_810B23 039.1_WP_079298496.:	TCGCACAGTTTGATGAACTAGCTGCCTTTTCTCAATTTGCATCTGATCTTGATGAAGCAA	atpA	ATP synthase alpha subunit	WP_079298496.:	966..2188	Energy Metabolism	COG0056
039.1_WP_079298496.:	CUST_3992_PI442190723	HOT208_60m_810B23 039.1_WP_079298496.:	TGAAACTCAGGCAGGGGACGTTTCTGCTTACATCCCAACTAACGTTATTTCAATTACAGA	atpA	ATP synthase alpha subunit	WP_079298496.:	966..2188	Energy Metabolism	COG0056
039.1_WP_079298496.:	CUST_3993_PI442190723	HOT208_60m_810B23 039.1_WP_079298496.:	TACTTACACAGTAGATTGTTAGAAAGAGCAGCAAAACTTTCTGATGCTATGGAGGGGGGC	atpA	ATP synthase alpha subunit	WP_079298496.:	966..2188	Energy Metabolism	COG0056
039.1_WP_079298496.:	CUST_3994_PI442190723	HOT208_60m_810B23 039.1_WP_079298496.:	ATTGGTCAGAAGTCAGCATCAGTAGCAAACATCGTAGAGGTATTAAGAGAGAGAGGAGCC	atpA	ATP synthase alpha subunit	WP_079298496.:	966..2188	Energy Metabolism	COG0056
039.1_WP_079298496.:	CUST_3995_PI442190723	HOT208_60m_810B23 039.1_WP_079298496.:	TTGAAGAAATGGCTCCTGGGATAATCAAGAGAAGATCAGTGCATGAACCAATGCAAACAG	atpA	ATP synthase alpha subunit	WP_079298496.:	966..2188	Energy Metabolism	COG0056
061.1_845	CUST_4171_PI442190723	HOT208_60m_810B23 061.1_845	TCGGAGTCAGTTAAAAATGCAATTGAAGCTCCTGAGCCTCCTTCAGAAGAATTAACCAAA	pdhA	Pyruvate dehydrogenase E1 component alpha subunit		<1..402	Glycolysis/Krebs cycle	COG1071
061.1_845	CUST_4172_PI442190723	HOT208_60m_810B23 061.1_845	AGATCCTATTAAGAAATTAGCTCAAGAAATTATTGACGGTAAATTCGCTACGGAAGAAGA	pdhA	Pyruvate dehydrogenase E1 component alpha subunit		<1..402	Glycolysis/Krebs cycle	COG1071
061.1_845	CUST_4173_PI442190723	HOT208_60m_810B23 061.1_845	GGCATTCTCTTGCAGATCCAGATGAATTAAGGTCTGAAAAAGAGAAGGAGTTTTGGGGGA	pdhA	Pyruvate dehydrogenase E1 component alpha subunit		<1..402	Glycolysis/Krebs cycle	COG1071
061.1_845	CUST_4174_PI442190723	HOT208_60m_810B23 061.1_845	CAAAGAGCAATTGAGCGAGCTAGGGCAGGAGAAGGTCCTACACTTTTAGAATGTTTAACT	pdhA	Pyruvate dehydrogenase E1 component alpha subunit		<1..402	Glycolysis/Krebs cycle	COG1071
061.1_845	CUST_4175_PI442190723	HOT208_60m_810B23 061.1_845	GAGAAAAGCATCTGCTTTTGGCATGCATGGCGAGGAAGTTGATGGAATGGATGTATTAG	pdhA	Pyruvate dehydrogenase E1 component alpha subunit		<1..402	Glycolysis/Krebs cycle	COG1071
006.1_WP_079298002.:	CUST_4630_PI442190723	HOT208_60m_810B23 006.1_WP_079298002.:	TGGGAAAATGTCTCAAAAGAAATGCTAAAGCCGTCATGGGTTTTTGACACCAGAGGTATT	ugd	UDP-glucose 6-dehydrogenase/nucleotide sugar dehydrogenase	WP_079298002.:	74699..76120	Amino sugar and nucleotide sugar r	COG1004
006.1_WP_079298002.:	CUST_4631_PI442190723	HOT208_60m_810B23 006.1_WP_079298002.:	GCCCTGAAACTGGATCTTGGGTTCACTCTCTTTCAATAAATGAATCCGTAAAGGATTCCC	ugd	UDP-glucose 6-dehydrogenase/nucleotide sugar dehydrogenase	WP_079298002.:	74699..76120	Amino sugar and nucleotide sugar r	COG1004
006.1_WP_079298002.:	CUST_4632_PI442190723	HOT208_60m_810B23 006.1_WP_079298002.:	TGGATTCAAGAATAGGGGATAAATTTTTAAATGCTGGTCCTGGTTTTGGAGGAAGTTGCT	ugd	UDP-glucose 6-dehydrogenase/nucleotide sugar dehydrogenase	WP_079298002.:	74699..76120	Amino sugar and nucleotide sugar r	COG1004
006.1_WP_079298002.:	CUST_4633_PI442190723	HOT208_60m_810B23 006.1_WP_079298002.:	ACAATCTTGATGAAGTAGCCAGCTATTGGAGAAAAGTTGTAGAGATCAATGAATGGCAAC	ugd	UDP-glucose 6-dehydrogenase/nucleotide sugar dehydrogenase	WP_079298002.:	74699..76120	Amino sugar and nucleotide sugar r	COG1004
006.1_WP_079298002.:	CUST_4634_PI442190723	HOT208_60m_810B23 006.1_WP_079298002.:	TCGTACAAATCTTTGGAGTTCAGAACTCTCTAAATTGACAGCTAATGCATTTTTAGCGCA	ugd	UDP-glucose 6-dehydrogenase/nucleotide sugar dehydrogenase	WP_079298002.:	74699..76120	Amino sugar and nucleotide sugar r	COG1004
006.1_WP_079298002.:	CUST_4635_PI442190723	HOT208_60m_810B23 006.1_WP_079298002.:	TGACACTAGGGAATCACCTGCAATAAATATATGTAAGGATCTTTTGTCAGAAGGTGCAAA	ugd	UDP-glucose 6-dehydrogenase/nucleotide sugar dehydrogenase	WP_079298002.:	74699..76120	Amino sugar and nucleotide sugar r	COG1004
006.1_WP_079298058.:	CUST_4648_PI442190723	HOT208_60m_810B23 006.1_WP_079298058.:	TCAGGGAGTCATACACTTTGCAGGTTTAAAGTCTGTTAAAGAATCCATTTCTGAGCCGCT	galE	UDP-glucose 4-epimerase	WP_079298058.:	79236..80297	Galactose metabolism	COG1087
006.1_WP_079298058.:	CUST_4649_PI442190723	HOT208_60m_810B23 006.1_WP_079298058.:	AATCCTATTGGAGCTCATCCATCTGGCATTATTGGAGAGGAACCAAAAGGAATTCCAAAT	galE	UDP-glucose 4-epimerase	WP_079298058.:	79236..80297	Galactose metabolism	COG1087
006.1_WP_079298058.:	CUST_4650_PI442190723	HOT208_60m_810B23 006.1_WP_079298058.:	AAAAATTCCTTACACATATGCATCTCGCAGAGCTGGAGATTATGCCAAAGTAGTTGCTGA	galE	UDP-glucose 4-epimerase	WP_079298058.:	79236..80297	Galactose metabolism	COG1087
006.1_WP_079298058.:	CUST_4651_PI442190723	HOT208_60m_810B23 006.1_WP_079298058.:	TCCTGATGGAACAGGTATAAGGGATTATATTCATGTTATGGATTTAGCAGATGGGCACGT	galE	UDP-glucose 4-epimerase	WP_079298058.:	79236..80297	Galactose metabolism	COG1087
006.1_WP_079298058.:	CUST_4652_PI442190723	HOT208_60m_810B23 006.1_WP_079298058.:	TGGGAACCAAAAAGAAATCTTGAACAAATGTGTCGAGATGGTTGGAAGTGGAAATTATCA	galE	UDP-glucose 4-epimerase	WP_079298058.:	79236..80297	Galactose metabolism	COG1087
006.1_WP_079298058.:	CUST_4653_PI442190723	HOT208_60m_810B23 006.1_WP_079298058.:	TGTTTTTAGCAGTAGTGCAACCATTTATGACTCTTCTGCGGGATCATTACTTGATGAATT	galE	UDP-glucose 4-epimerase	WP_079298058.:	79236..80297	Galactose metabolism	COG1087
006.1_WP_079298015.:	CUST_4714_PI442190723	HOT208_60m_810B23 006.1_WP_079298015.:	GTATTATCCAAGCGTGGGAGTAGGCGGCCACTGCATACCAATAGATCCTTTTATCTAAG	ugd	UDP-glucose 6-dehydrogenase/nucleotide sugar dehydrogenase	WP_079298015.:	91585..92895	Amino sugar and nucleotide sugar r	COG1004
006.1_WP_079298015.:	CUST_4715_PI442190723	HOT208_60m_810B23 006.1_WP_079298015.:	AAACGTAATTCAAACACTTATTCCTGTTTCAAGCACTAAAACTGCAGAAATGACCAAGCT	ugd	UDP-glucose 6-dehydrogenase/nucleotide sugar dehydrogenase	WP_079298015.:	91585..92895	Amino sugar and nucleotide sugar r	COG1004
006.1_WP_079298015.:	CUST_4716_PI442190723	HOT208_60m_810B23 006.1_WP_079298015.:	GGATATAGATATTTATGAAGTCATAAATGCAGCCGCAACAAAACCATTTGGATTTGTCCC	ugd	UDP-glucose 6-dehydrogenase/nucleotide sugar dehydrogenase	WP_079298015.:	91585..92895	Amino sugar and nucleotide sugar r	COG1004
006.1_WP_079298015.:	CUST_4717_PI442190723	HOT208_60m_810B23 006.1_WP_079298015.:	TGCTTTAATTGAACAAGAAAGTAATCTTATTATTGATACCACTGGGAGATTAAAGCCTTC	ugd	UDP-glucose 6-dehydrogenase/nucleotide sugar dehydrogenase	WP_079298015.:	91585..92895	Amino sugar and nucleotide sugar r	COG1004
006.1_WP_079298015.:	CUST_4718_PI442190723	HOT208_60m_810B23 006.1_WP_079298015.:	TCTAGCAGATAAGGTTAATGAATATATGCCCTTATATGTTTTAATAAATCCAAGGAGGT	ugd	UDP-glucose 6-dehydrogenase/nucleotide sugar dehydrogenase	WP_079298015.:	91585..92895	Amino sugar and nucleotide sugar r	COG1004
006.1_WP_079298015.:	CUST_4719_PI442190723	HOT208_60m_810B23 006.1_WP_079298015.:	AGTTCTTATTCTTGGAATTAGTTACAAAAAAAATATTGATGATGCCAGAGAATCTTGTTC	ugd	UDP-glucose 6-dehydrogenase/nucleotide sugar dehydrogenase	WP_079298015.:	91585..92895	Amino sugar and nucleotide sugar r	COG1004
006.1_WP_079298059.:	CUST_4744_PI442190723	HOT208_60m_810B23 006.1_WP_079298059.:	TAGAGGAGGAGAACAAGGAGCACAGCATTCTCAAGCTTTACATTCATGGTTTGCTCATAT	eno	Enolase	WP_079298059.:	96818..97807	Glycolysis/Gluconeogenesis	COG0148
006.1_WP_079298059.:	CUST_4745_PI442190723	HOT208_60m_810B23 006.1_WP_079298059.:	GCCTTCATCTCCTTTGGATGCAAGAGATCTTCTAATATCGTCTGTATTGTCTCCTGACCC	eno	Enolase	WP_079298059.:	96818..97807	Glycolysis/Gluconeogenesis	COG0148
006.1_WP_079298059.:	CUST_4746_PI442190723	HOT208_60m_810B23 006.1_WP_079298059.:	GTCCATCCAAGGATGGACTTTATGCTTTATGCAATTGATCAAATAGTTAATCAGGCTGCA	eno	Enolase	WP_079298059.:	96818..97807	Glycolysis/Gluconeogenesis	COG0148
006.1_WP_079298059.:	CUST_4747_PI442190723	HOT208_60m_810B23 006.1_WP_079298059.:	TGATGGAGGCTGGAGTTCATGTGGAATAGCATCAGAAATAATAAGTTTAGTTTGTCAGGA	eno	Enolase	WP_079298059.:	96818..97807	Glycolysis/Gluconeogenesis	COG0148
006.1_WP_079298059.:	CUST_4748_PI442190723	HOT208_60m_810B23 006.1_WP_079298059.:	TGTGGCTTTAGTACAAAATTAGCTTCTGAAGCTATCGATAAGTTTGTTGTGAAGACGTC	eno	Enolase	WP_079298059.:	96818..97807	Glycolysis/Gluconeogenesis	COG0148
006.1_WP_079298059.:	CUST_4749_PI442190723	HOT208_60m_810B23 006.1_WP_079298059.:	AGAAGAACAAATGATTGAACATATAGTTGAAAAGGATCTCTCAAATGAGGAACCAGTGGT	eno	Enolase	WP_079298059.:	96818..97807	Glycolysis/Gluconeogenesis	COG0148
073.1_993	CUST_4998_PI442190723	HOT208_60m_810B23 073.1_993	GAAACTTGGAAGGAAATTAAGTTCGAGTTTGACACTGTCGACAAGCTTGATGTTCAGGGT	rbcS/L	ribulose bisphosphate carboxylase		<16..252	Carbon fixation	COG1850/COG4451
073.1_993	CUST_4999_PI442190723	HOT208_60m_810B23 073.1_993	AGAGAGTAGAGACATTCTTATGGAAGCTGCTAAGCATAGTCCTGAATTAGCTA	rbcS/L	ribulose bisphosphate carboxylase		<16..252	Carbon fixation	COG1850/COG4451
073.1_993	CUST_5001_PI442190723	HOT208_60m_810B23 073.1_993	TTCGGTGGAGGAACACATGGTCATCCATGGGGTTCAGCTGCTGGAG	rbcS/L	ribulose bisphosphate carboxylase		<16..252	Carbon fixation	COG1850/COG4451
073.1_WP_011862568.:	CUST_5002_PI442190723	HOT208_60m_810B23 073.1_WP_011862568.:	TCGGATACGATGCTTACACTCAAAGTCAAGGAACAGCTTTTGTAGTATTCCAAGGACGTT	rbcS	Ribulose bisphosphate carboxylase small subunit	WP_011862568.:	342..683	Carbon fixation	COG4451
073.1_WP_011862568.:	CUST_5003_PI442190723	HOT208_60m_810B23 073.1_WP_011862568.:	GGAAAAAGATCTTAACTTGATCGTGAGCGAATTAGAGGCATGCCATAGAGCATACCCTGA	rbcS	Ribulose bisphosphate carboxylase small subunit	WP_011862568.:	342..683	Carbon fixation	COG4451
073.1_WP_011862568.:	CUST_5004_PI442190723	HOT208_60m_810B23 073.1_WP_011862568.:	TGAGCATGTTCATCCAAGTGGATGTATGCAAACTTATTGGTCTTATTGGAAACTCCCATT	rbcS	Ribulose bisphosphate carboxylase small subunit	WP_011862568.:	342..683	Carbon fixation	COG4451
073.1_WP_011862568.:	CUST_5005_PI442190723	HOT208_60m_810B23 073.1_WP_011862568.:	CCACCGATGACCCAGGAAGAAATATATGACCAAATTGCGTACATAATTGCTCAAGGTTGG	rbcS	Ribulose bisphosphate carboxylase small subunit	WP_011862568.:	342..683	Carbon fixation	COG4451
073.1_WP_011862568.:	CUST_5006_PI442190723	HOT208_60m_810B23 073.1_WP_011862568.:	TTCCAGAGCACAGTAAGCGACTATCAAACAGTTGCAACCCTGGAAACATTCGGTTTTTTA	rbcS	Ribulose bisphosphate carboxylase small subunit	WP_011862568.:	342..683	Carbon fixation	COG4451
075.1_WP_079298640.:	CUST_5451_PI442190723	HOT208_60m_810B23 075.1_WP_079298640.:	TCATCCTCATGGTATTTAACTGGGGAGGGCATTGGAAAGAGAGATATGGTCGTATTACCT	pgi	Glucose-6-phosphate isomerase	WP_079298640.:	54670..56253	Glycolysis/Gluconeogenesis	COG0166
075.1_WP_079298640.:	CUST_5452_PI442190723	HOT208_60m_810B23 075.1_WP_079298640.:	TTCACTAGGTGCCTTAATCGCTTTATTTGAGAGAGCTGTATCCTTCTACGCTGAATTGGT	pgi	Glucose-6-phosphate isomerase	WP_079298640.:	54670..56253	Glycolysis/Gluconeogenesis	COG0166
075.1_WP_079298640.:	CUST_5453_PI442190723	HOT208_60m_810B23 075.1_WP_079298640.:	GGTGCATCATTGATGGATGAGGCTACACGCATAAGTGATTTTAAAAATAATCCAGCAGCA	pgi	Glucose-6-phosphate isomerase	WP_079298640.:	54670..56253	Glycolysis/Gluconeogenesis	COG0166
075.1_WP_079298640.:	CUST_5454_PI442190723	HOT208_60m_810B23 075.1_WP_079298640.:	ATGCATATGATCAACCTGGAGTTGAAGCTGGAAAGAAAGCAGCTGCAAATATTATTGAGT	pgi	Glucose-6-phosphate isomerase	WP_079298640.:	54670..56253	Glycolysis/Gluconeogenesis	COG0166
075.1_WP_079298640.:	CUST_5455_PI442190723	HOT208_60m_810B23 075.1_WP_079298640.:	TTCCGTTTTTGGTAATAAAGGATCTACAGATCAACATGCTTATGTTCAGCAACTGAGAGA	pgi	Glucose-6-phosphate isomerase	WP_079298640.:	54670..56253	Glycolysis/Gluconeogenesis	COG0166
075.1_WP_079298640.:	CUST_5456_PI442190723	HOT208_60m_810B23 075.1_WP_079298640.:	ACTCCGTGAAATGTGTTTCGGTAATGATAATTATTTAGTTAAGGGCGATTGGTCAAATCC	pgi	Glucose-6-phosphate isomerase	WP_079298640.:	54670..56253	Glycolysis/Gluconeogenesis	COG0166
075.1_WP_079298674.:	CUST_5568_PI442190723	HOT208_60m_810B23 075.1_WP_079298674.:	AATCAGGTTCTACCGCTCGAAATGTAAGTAAATTCAGACCACCAACACCTATCTTGGCAA	pykF	Pyruvate kinase	WP_079298674.:	75144..76934	Glycolysis	COG0469

075.1_WP_079298674.:	CUST_5569_PI442190723	HOT208_60m_810B23	075.1_WP_079298674.:	CCACTTAAGGCTATAGAAAGCCACTTACCAAGTACGATCCCAAATGCTATTAGTGCAGCA	pykF	Pyruvate kinase	WP_079298674.:	75144..76934	Glycolysis	COG0469
075.1_WP_079298674.:	CUST_5570_PI442190723	HOT208_60m_810B23	075.1_WP_079298674.:	GCAAATTGCTCAGGAGATGGGAATCCTAAATCAAGGAGATTTAGTAGTTCAAACCGCAGG	pykF	Pyruvate kinase	WP_079298674.:	75144..76934	Glycolysis	COG0469
075.1_WP_079298674.:	CUST_5571_PI442190723	HOT208_60m_810B23	075.1_WP_079298674.:	AGCAACGGCGACCTTATTACACTGCAGCTAAATGAAGGTGTTATATATATGGGGCAAATT	pykF	Pyruvate kinase	WP_079298674.:	75144..76934	Glycolysis	COG0469
075.1_WP_079298674.:	CUST_5572_PI442190723	HOT208_60m_810B23	075.1_WP_079298674.:	AGAAAGATTGTATCAAGAGGAGTTTCAATAGGGGAGATCGGAGTTACAGGTAAAGCAAGA	pykF	Pyruvate kinase	WP_079298674.:	75144..76934	Glycolysis	COG0469
075.1_WP_079298674.:	CUST_5573_PI442190723	HOT208_60m_810B23	075.1_WP_079298674.:	ACAACAGAGAGAAGTGTAGCGAGAAGATTACAACTTGTTTGGGGAGTTACTCCAATAGTA	pykF	Pyruvate kinase	WP_079298674.:	75144..76934	Glycolysis	COG0469
075.1_WP_079298688.:	CUST_5614_PI442190723	HOT208_60m_810B23	075.1_WP_079298688.:	AGACGCACTGGCAAATCCTTACTTAGCATTTTCTGTAATGATGCTTGCTGGTATTGATGG	glnA	Glutamine synthetase/ glutamate--ammonialigase	WP_079298688.:	84155..85576	Nitrogen Metabolism	COG0174
075.1_WP_079298688.:	CUST_5615_PI442190723	HOT208_60m_810B23	075.1_WP_079298688.:	ACGTTTATGCCCAAGCCTGTATTTAACGATAATGGAACTGGAATGCATGTTCACCAAAGT	glnA	Glutamine synthetase/ glutamate--ammonialigase	WP_079298688.:	84155..85576	Nitrogen Metabolism	COG0174
075.1_WP_079298688.:	CUST_5616_PI442190723	HOT208_60m_810B23	075.1_WP_079298688.:	TGCTGGTGCCGGTCAACATGAGCTTGGAATGAAATTCGATTCATTAATAAATTCTGCTGA	glnA	Glutamine synthetase/ glutamate--ammonialigase	WP_079298688.:	84155..85576	Nitrogen Metabolism	COG0174
075.1_WP_079298688.:	CUST_5617_PI442190723	HOT208_60m_810B23	075.1_WP_079298688.:	TCTGAGGGTAATAGATCAGCTGCAGTAAGAATACCTTTAACAGGACCAAGCCCTAAAGCT	glnA	Glutamine synthetase/ glutamate--ammonialigase	WP_079298688.:	84155..85576	Nitrogen Metabolism	COG0174
075.1_WP_079298688.:	CUST_5618_PI442190723	HOT208_60m_810B23	075.1_WP_079298688.:	TGGTGAAGGAGCATATGCAAATTTATCTCAAACAGCAAGATGGTATATCGGAGGCATACT	glnA	Glutamine synthetase/ glutamate--ammonialigase	WP_079298688.:	84155..85576	Nitrogen Metabolism	COG0174
075.1_WP_079298688.:	CUST_5619_PI442190723	HOT208_60m_810B23	075.1_WP_079298688.:	GCATTTACTAACCCAACCACAAATAGTTATAAACGATTGGTTCCAGGATTCGAAGCTCCT	glnA	Glutamine synthetase/ glutamate--ammonialigase	WP_079298688.:	84155..85576	Nitrogen Metabolism	COG0174
076.1_WP_079298718.:	CUST_5666_PI442190723	HOT208_60m_810B23	076.1_WP_079298718.:	AAGTAGGTAAATATCCGATAATTTGCGCCGAATTATGTGGCCCATATCATGGAGGGATGA	ctaC/coxB	Cytochrome c oxidase subunit II	WP_079298718.:	358..1161	Respiration	COG1622
076.1_WP_079298718.:	CUST_5667_PI442190723	HOT208_60m_810B23	076.1_WP_079298718.:	GACGTCATTCATGCTTTTTGGGTGCCAGAGTTCAGAATTAAACAGGATATTATTCCGGGG	ctaC/coxB	Cytochrome c oxidase subunit II	WP_079298718.:	358..1161	Respiration	COG1622
076.1_WP_079298718.:	CUST_5668_PI442190723	HOT208_60m_810B23	076.1_WP_079298718.:	TCATTTTGGATTGGTTTTAATGTAAATTTGCTTCCCGCTGAAGCAAGTATCAATGCACCA	ctaC/coxB	Cytochrome c oxidase subunit II	WP_079298718.:	358..1161	Respiration	COG1622
076.1_WP_079298718.:	CUST_5669_PI442190723	HOT208_60m_810B23	076.1_WP_079298718.:	TCCCAAGGGCAATTTCATATCAGGAGAACTACACGTTCCTGTTGATCAAAAAGTATCAAT	ctaC/coxB	Cytochrome c oxidase subunit II	WP_079298718.:	358..1161	Respiration	COG1622
076.1_WP_079298718.:	CUST_5670_PI442190723	HOT208_60m_810B23	076.1_WP_079298718.:	AGCTACAACATCTACGATCGAATGGGAGGGATGAAAGAACTAAATCATAACCACGAAATG	ctaC/coxB	Cytochrome c oxidase subunit II	WP_079298718.:	358..1161	Respiration	COG1622
076.1_WP_079298718.:	CUST_5671_PI442190723	HOT208_60m_810B23	076.1_WP_079298718.:	ATTCAGGAAAAGAAATGATCAGATTGGAGATGGCATAGCTTTAGAGGGAAATTTAAGCTT	ctaC/coxB	Cytochrome c oxidase subunit II	WP_079298718.:	358..1161	Respiration	COG1622
076.1_WP_079298720.:	CUST_5672_PI442190723	HOT208_60m_810B23	076.1_WP_079298720.:	TTCCTTAGTGTCAGAAATGGGAAAAATGCAGGAGATAACCCTTGGAATGCTCTTACACCT	cyoB/coxA	Cytochrome c oxidase subunit I	WP_079298720.:	1158..2783	Respiration	COG0843
076.1_WP_079298720.:	CUST_5673_PI442190723	HOT208_60m_810B23	076.1_WP_079298720.:	GCTTTGCTCCTCAACATTGGCTTGGTTTAAATGGAATGCCAAGAAGAGTTGCAGAATATG	cyoB/coxA	Cytochrome c oxidase subunit I	WP_079298720.:	1158..2783	Respiration	COG0843
076.1_WP_079298720.:	CUST_5674_PI442190723	HOT208_60m_810B23	076.1_WP_079298720.:	TTTGGAGGTATTACAGGAGTTGCTTTGGCACAGGTTCCTTTCGATATTCACGTACATGAT	cyoB/coxA	Cytochrome c oxidase subunit I	WP_079298720.:	1158..2783	Respiration	COG0843
076.1_WP_079298720.:	CUST_5675_PI442190723	HOT208_60m_810B23	076.1_WP_079298720.:	GATAGTAGTTTTAGGTTTAGTTGTTTGGGCGCATCACATGTTTACAAGTGGAACGCCTCC	cyoB/coxA	Cytochrome c oxidase subunit I	WP_079298720.:	1158..2783	Respiration	COG0843
076.1_WP_079298720.:	CUST_5676_PI442190723	HOT208_60m_810B23	076.1_WP_079298720.:	GAATGGTTAACATCTTCTCCACCTCCAGTTGAAAATTGGGAAGGAGAGGCTCCATTAGTT	cyoB/coxA	Cytochrome c oxidase subunit I	WP_079298720.:	1158..2783	Respiration	COG0843
076.1_WP_079298720.:	CUST_5677_PI442190723	HOT208_60m_810B23	076.1_WP_079298720.:	TCGCCACAGCATTTATTGCTGTTCCGACAGGTATAAAATTTTTCAATTGGGTTGCAACTT	cyoB/coxA	Cytochrome c oxidase subunit I	WP_079298720.:	1158..2783	Respiration	COG0843
076.1_WP_025880735.:	CUST_5678_PI442190723	HOT208_60m_810B23	076.1_WP_025880735.:	TTGGTCTTATAACTTTCCTAATTGCGGACGGAATGACTTTTGCTGGATTCTTTGCTGCTT	cyoC	heme-copper oxidase subunit III	WP_025880735.:	2790..3392	Respiration	COG1845
076.1_WP_025880735.:	CUST_5679_PI442190723	HOT208_60m_810B23	076.1_WP_025880735.:	TGCTTGGCAATCGAGAATCAATGGCGGTAGATTAACCAGTCAAAATATGTTTCCATTGGA	cyoC	heme-copper oxidase subunit III	WP_025880735.:	2790..3392	Respiration	COG1845
076.1_WP_025880735.:	CUST_5680_PI442190723	HOT208_60m_810B23	076.1_WP_025880735.:	AGTGCAACTTTCCATAAAGCAGGCAAAGCACTTTTAAAAGATAAAAACTCAGATTCCCAA	cyoC	heme-copper oxidase subunit III	WP_025880735.:	2790..3392	Respiration	COG1845
076.1_WP_025880735.:	CUST_5681_PI442190723	HOT208_60m_810B23	076.1_WP_025880735.:	TGCAAGTACTTTTTATGCTCTTACTGGTTTTCATGGATTACATGTCACTTTAGGCACTTT	cyoC	heme-copper oxidase subunit III	WP_025880735.:	2790..3392	Respiration	COG1845
076.1_WP_025880735.:	CUST_5682_PI442190723	HOT208_60m_810B23	076.1_WP_025880735.:	AGCAGTAAACCCATTACCAGATGGTGCTATTTATGAATTAGAACTACCAATACCTACTCT	cyoC	heme-copper oxidase subunit III	WP_025880735.:	2790..3392	Respiration	COG1845
076.1_WP_025880735.:	CUST_5683_PI442190723	HOT208_60m_810B23	076.1_WP_025880735.:	TGGAAGCTGTTGAATTGTACTGGCATTTTGTAGATGGAATATGGGTTGTTTTATTT	cyoC	heme-copper oxidase subunit III	WP_025880735.:	2790..3392	Respiration	COG1845
077.1_WP_079299005.:	CUST_6553_PI442190723	HOT208_60m_810B23	077.1_WP_079299005.:	TCAGGAGAAATAAAATTCAAAAAAACTGAAAAAAATTGATAAAGTTGATAAATCTAACAAA	minE	septum site-determining protein MinE	WP_079299005.:	47483..47827	Cell Division	COG0851
077.1_WP_079299005.:	CUST_6554_PI442190723	HOT208_60m_810B23	077.1_WP_079299005.:	AGCAGTAAGTTTAGAGACTGAGGATAGAATGACTGCGTTAGTTGCCAATTTACCAATCAA	minE	septum site-determining protein MinE	WP_079299005.:	47483..47827	Cell Division	COG0851
077.1_WP_079299005.:	CUST_6555_PI442190723	HOT208_60m_810B23	077.1_WP_079299005.:	AGTTCCTTAACAACTGATCTTTTAGATAAAATGAGGAAAGAAATTCTTGATGTTGTTGCT	minE	septum site-determining protein MinE	WP_079299005.:	47483..47827	Cell Division	COG0851
077.1_WP_079299005.:	CUST_6556_PI442190723	HOT208_60m_810B23	077.1_WP_079299005.:	AGGCAGAGAAACGTCTAGTGCAAATACAGCTAGAGAAAGATTACAACTTGTACTTGCTCA	minE	septum site-determining protein MinE	WP_079299005.:	47483..47827	Cell Division	COG0851
077.1_WP_079299007.:	CUST_6557_PI442190723	HOT208_60m_810B23	077.1_WP_079299007.:	ATAGCACTTGCTAATAGCGGAGCTACAACTGCTGTATTAGATGCCGATTTTGGCTTAAGA	minD	septum site-determining protein MinD	WP_079299007.:	47829..48644	Cell Division	COG2894
077.1_WP_079299007.:	CUST_6558_PI442190723	HOT208_60m_810B23	077.1_WP_079299007.:	AGAGTTCGCCCTAACATGATGGCTAGTCAAGAAATGTTATCCATCGAAGATGTTCAAGGG	minD	septum site-determining protein MinD	WP_079299007.:	47829..48644	Cell Division	COG2894
077.1_WP_079299007.:	CUST_6559_PI442190723	HOT208_60m_810B23	077.1_WP_079299007.:	AGCACAAATAGAGGAGAGCCACTGACACTTTCAGATGGTAGATCTCCTGCAAAAAAATGT	minD	septum site-determining protein MinD	WP_079299007.:	47829..48644	Cell Division	COG2894
077.1_WP_079299007.:	CUST_6560_PI442190723	HOT208_60m_810B23	077.1_WP_079299007.:	ACTAACCCAGAATTATCCGCAGTGCGGGATGCGGATAGAGTAATAGGGATTCTTAATACT	minD	septum site-determining protein MinD	WP_079299007.:	47829..48644	Cell Division	COG2894
077.1_WP_079299007.:	CUST_6561_PI442190723	HOT208_60m_810B23	077.1_WP_079299007.:	ACTTAGTGCGAACTTTGATTTTGTCTTAGTAGATTGCCCTGCTGGTGTAGAAGATGGCTT	minD	septum site-determining protein MinD	WP_079299007.:	47829..48644	Cell Division	COG2894
077.1_WP_079299007.:	CUST_6562_PI442190723	HOT208_60m_810B23	077.1_WP_079299007.:	AAAGGAACCTAATCTTGCTCTTTTACCTGCTGGAGATCCAAGGATGTTGGATTGGATGAA	minD	septum site-determining protein MinD	WP_079299007.:	47829..48644	Cell Division	COG2894
077.1_WP_079299009.:	CUST_6563_PI442190723	HOT208_60m_810B23	077.1_WP_079299009.:	TAACCCAGGCGCAATAGTTTCCGCTGAGAAAAATATTTACGTTTGGGGCAAATTACTAGG	minC	septum site-determining protein MinC	WP_079299009.:	48756..49418	Cell Division	COG0850
077.1_WP_079299009.:	CUST_6564_PI442190723	HOT208_60m_810B23	077.1_WP_079299009.:	TGCAGATGTGGTAGCTATTGGACCAAAGGATAAGCCCAAAAATTGTTATCCAGAAGTTGC	minC	septum site-determining protein MinC	WP_079299009.:	48756..49418	Cell Division	COG0850
077.1_WP_079299009.:	CUST_6565_PI442190723	HOT208_60m_810B23	077.1_WP_079299009.:	CGCATTCGCAGGGAAAAGTGGAAATAAAAATGCCTCTATTGCATCACTTTATCTAAACCC	minC	septum site-determining protein MinC	WP_079299009.:	48756..49418	Cell Division	COG0850
077.1_WP_079299009.:	CUST_6566_PI442190723	HOT208_60m_810B23	077.1_WP_079299009.:	TTCTTCACAAAGGAACAGTAAGATCCGGTGAAAGAATATCTTCGAATGGAAACCTATGCA	minC	septum site-determining protein MinC	WP_079299009.:	48756..49418	Cell Division	COG0850
077.1_WP_079299009.:	CUST_6567_PI442190723	HOT208_60m_810B23	077.1_WP_079299009.:	TCCATAAAGGAACCTTTAGAAGCAAAAATTTTCGCAGTCAATGAGACAATAAGTTCTCAA	minC	septum site-determining protein MinC	WP_079299009.:	48756..49418	Cell Division	COG0850
077.1_WP_079299009.:	CUST_6568_PI442190723	HOT208_60m_810B23	077.1_WP_079299009.:	AGAGAAACAATTCTGAGTGGAAAATCTTTAAAAATAGATTCAGCTTTTATAGAAGAGCAA	minC	septum site-determining protein MinC	WP_079299009.:	48756..49418	Cell Division	COG0850
078.1_WP_079299073.:	CUST_6804_PI442190723	HOT208_60m_810B23	078.1_WP_079299073.:	ATTGAGTTACCATATTGCATCTCATAGGGGACTTGACGTGGATCAACCAAGAAATCTCGC	glmS	Glutamine--fructose-6-phosphatetransaminase(isomerizing)	WP_079299073.:	34349..36244	Amino Acid metabolism	COG0449
078.1_WP_079299073.:	CUST_6805_PI442190723	HOT208_60m_810B23	078.1_WP_079299073.:	ATACATGCAGCTGGATATCCTGCTGGAGAAATGAAACACGGTCCAATAGCTTTGTTAGAT	glmS	Glutamine--fructose-6-phosphatetransaminase(isomerizing)	WP_079299073.:	34349..36244	Amino Acid metabolism	COG0449
078.1_WP_079299073.:	CUST_6806_PI442190723	HOT208_60m_810B23	078.1_WP_079299073.:	TGTGCAGGAATAGAAGTTGGAGTTGCAGCAACAAAAACTTTTTTTGCTCAATTACTCGCT	glmS	Glutamine--fructose-6-phosphatetransaminase(isomerizing)	WP_079299073.:	34349..36244	Amino Acid metabolism	COG0449
078.1_WP_079299073.:	CUST_6807_PI442190723	HOT208_60m_810B23	078.1_WP_079299073.:	ATCGCCTCCCCTGGTGAGGTTTTTGATAAAGTTATTAGTAATGCTCAAGAAGCAAAAGCT	glmS	Glutamine--fructose-6-phosphatetransaminase(isomerizing)	WP_079299073.:	34349..36244	Amino Acid metabolism	COG0449
078.1_WP_079299073.:	CUST_6808_PI442190723	HOT208_60m_810B23	078.1_WP_079299073.:	ACCCAATCTTATTGCAATAACAAATAGAAAAGAGAGTTCCATAGGAAGGCAGGTCTCAAA	glmS	Glutamine--fructose-6-phosphatetransaminase(isomerizing)	WP_079299073.:	34349..36244	Amino Acid metabolism	COG0449
078.1_WP_079299073.:	CUST_6809_PI442190723	HOT208_60m_810B23	078.1_WP_079299073.:	TCCGCCATTACTGGAAGATCTCATAGAGAAACATAATAAATCTTCTGAAAAGCTAGCACA	glmS	Glutamine--fructose-6-phosphatetransaminase(isomerizing)	WP_079299073.:	34349..36244	Amino Acid metabolism	COG0449
007.1_WP_079298065.:	CUST_7522_PI442190723	HOT208_60m_810B23	007.1_WP_079298065.:	GTTGATGCAAGACTGAGCTTTGACACCGAAGCTACGATAGGAAAAGCAAGGAAATTGATC	tal	Transaldolase	WP_079298065.:	4013..5014	Pentose Phosphate	COG0176
007.1_WP_079298065.:	CUST_7523_PI442190723	HOT208_60m_810B23	007.1_WP_079298065.:	CAGTAACTTGTGCGAATGCAAAGATAACTCTAATTTCCCCGTTCGTTGGCCGTATATTGG	tal	Transaldolase	WP_079298065.:	4013..5014	Pentose Phosphate	COG0176
007.1_WP_079298065.:	CUST_7524_PI442190723	HOT208_60m_810B23	007.1_WP_079298065.:	TAAAACCAGTTTTGTTGGTGCTGAAGACCCTGGCGTTATTTCCGTTACGCAAATATACAA	tal	Transaldolase	WP_079298065.:	4013..5014	Pentose Phosphate	COG0176
007.1_WP_079298065.:	CUST_7525_PI442190723	HOT208_60m_810B23	007.1_WP_079298065.:	TGGGAGGGAATTAAGGCAGCTGAAATTTTGGAAAAAGAGGGGATTAAGTGCAACTTAACT	tal	Transaldolase	WP_079298065.:	4013..5014	Pentose Phosphate	COG0176
007.1_WP_079298065.:	CUST_7526_PI442190723	HOT208_60m_810B23	007.1_WP_079298065.:	TGGCGAGTGAAAAGCTTAGTGAAGGTATCACTGGATTCAGTAAGGCTATAGAAGAATTGG	tal	Transaldolase	WP_079298065.:	4013..5014	Pentose Phosphate	COG0176
007.1_WP_079298065.:	CUST_7527_PI442190723	HOT208_60m_810B23	007.1_WP_079298065.:	AGCCGGTTGCGATCTTTTAACAATCGCACCAAAATTTCTTGAGGAACTTAAAAAAGAAAA	tal	Transaldolase	WP_079298065.:	4013..5014	Pentose Phosphate	COG0176
080.1_WP_079299304.:	CUST_7729_PI442190723	HOT208_60m_810B23	080.1_WP_079299304.:	GTGCAGAATTAATTGCCTTGATAACAGAAGAGTGTTTTGACGATCTTGATGCCCGACCAA	pdhB	Pyruvate dehydrogenase E1 component beta subunit	WP_079299304.:	1157..2140	Glycolysis/Krebs cycle	COG0022
080.1_WP_079299304.:	CUST_7730_PI442190723	HOT208_60m_810B23	080.1_WP_079299304.:	TGCTGAGCACAGTCAAAGACTTGAAGCATATTTTCATGCAGTTCCTGGCATAAAGATTGT	pdhB	Pyruvate dehydrogenase E1 component beta subunit	WP_079299304.:	1157..2140	Glycolysis/Krebs cycle	COG0022
080.1_WP_079299304.:	CUST_7731_PI442190723	HOT208_60m_810B23	080.1_WP_079299304.:	CCTGAGGGAGATTATACTTGCGCTTTAGATCAGGCTGACGTTGTAAAAGAAGGGAAAGAT	pdhB	Pyruvate dehydrogenase E1 component beta subunit	WP_079299304.:	1157..2140	Glycolysis/Krebs cycle	COG0022
080.1_WP_079299304.:	CUST_7732_PI442190723	HOT208_60m_810B23	080.1_WP_079299304.:	TATACAAGTGGCGGAAATTATAAAATACCAGCAGTAGTTCGAGGACCTGGAGGAGTTGGT	pdhB	Pyruvate dehydrogenase E1 component beta subunit	WP_079299304.:	1157..2140	Glycolysis/Krebs cycle	COG0022
080.1_WP_079299304.:	CUST_7733_PI442190723	HOT208_60m_810B23	080.1_WP_079299304.:	AGTACACCCACAAATGCTAAGGGTTTAATGAAAGCAGCTATAAGAGATGATAATCCGGTT	pdhB	Pyruvate dehydrogenase E1 component beta subunit	WP_079299304.:	1157..2140	Glycolysis/Krebs cycle	COG0022
080.1_WP_079299304.:	CUST_7734_PI442190723	HOT208_60m_810B23	080.1_WP_079299304.:	TGAGACATCACTGCCTTAAAGCTGTTGAAGAATTAGAAAAAATAGGAATAGATGTTGAGT	pdhB	Pyruvate dehydrogenase E1 component beta subunit	WP_079299304.:	1157..2140	Glycolysis/Krebs cycle	COG0022
080.1_WP_079299430.:	CUST_8133_PI442190723	HOT208_60m_810B23	080.1_WP_079299430.:	TTTTTTATGAAGGTTTGCATGGCGGGGTAGAAGGTGATGGATACAATGTTGCCTCTTATG	prk	Phosphoribulokinase	WP_079299430.:	65516..66412	Carbon Fixation	COG3954
080.1_WP_079299430.:	CUST_8134_PI442190723	HOT208_60m_810B23	080.1_WP_079299430.:	CTCCTGATGAGAGCTTTGTGATCATACATTTCAGAAAAGGGGCAAGAGAGAAATGGGGTA	prk	Phosphoribulokinase	WP_079299430.:	65516..66412	Carbon Fixation	COG3954
080.1_WP_079299430.:	CUST_8135_PI442190723	HOT208_60m_810B23	080.1_WP_079299430.:	TGGCAGACGCTCTTTCAAAAGGTGAAAATTTCTCACATTTTGGTCCAGAGGCTAATCTTT	prk	Phosphoribulokinase	WP_079299430.:	65516..66412	Carbon Fixation	COG3954
080.1_WP_079299430.:	CUST_8136_PI442190723	HOT208_60m_810B23	080.1_WP_079299430.:	ATACAAGACTTGGAACATCCCTAGAACCTGGGCAATTTACTCCATGGGAAGATATTCCTG	prk	Phosphoribulokinase	WP_079299430.:	65516..66412	Carbon Fixation	COG3954
080.1_WP_079299430.:	CUST_8137_PI442190723	HOT208_60m_810B23	080.1_WP_079299430.:	AACGCAGAAAGAGGTTACTCAGCGGAAACCATTGTGGATACAATATTGAGAAGAATGCCT	prk	Phosphoribulokinase	WP_079299430.:	65516..66412	Carbon Fixation	COG3954
080.1_WP_079299430.:	CUST_8138_PI442190723	HOT208_60m_810B23	080.1_WP_079299430.:	TGTCAAGTCCAACAAGTATCGTTGTAAATGGAGGGAAAATGGGATTCGCAATGGAACTAA	prk	Phosphoribulokinase	WP_079299430.:	65516..66412	Carbon Fixation	COG3954
080.1_1578	CUST_8156_PI442190723	HOT208_60m_810B23	080.1_1578	AGGAATGCTTTTTACTACAGAAGGTCTTGGCAAATATATAAGTGGAGCAATCCTCTTCGA	fda/cbbA	fructose-1,6-bisphosphatealdolase		69031..69278	Glycolysis/Gluconeogenesis	COG0191
080.1_1578	CUST_8157_PI442190723	HOT208_60m_810B23	080.1_1578	AGCGGTAGATGAATCCACTAAAACAGTAGGTAAAAGACTTGCTGGAATTGGCGTTGAGAA	fda/cbbA	fructose-1,6-bisphosphatealdolase		69031..69278	Glycolysis/Gluconeogenesis	COG0191
080.1_1578	CUST_8158_PI442190723	HOT208_60m_810B23	080.1_1578	AATTACTACAAAAATGAGCTTAAAGAAAATGCTCAATTACTAGCTTCTAAAGGAAAAGGA	fda/cbbA	fructose-1,6-bisphosphatealdolase		69031..69278	Glycolysis/Gluconeogenesis	COG0191
085.1_WP_079299605.:	CUST_8752_PI442190723	HOT208_60m_810B23	085.1_WP_079299605.:	CGTTGATAACTGGCGTTGGCAAGGGGTTCCTTTTTATTTGAGAACAGGTAAAAGACTACC	zwf	glucose-6-phosphate dehydrogenase zwf	WP_079299605.:	24570..26093	Pentose Phosphate	COG0364
085.1_WP_079299605.:	CUST_8753_PI442190723	HOT208_60m_810B23	085.1_WP_079299605.:	TAGCTGATGCAATGCTTTCTGACCCAACCCTATTTACTCGAAGTGATGAAGTAGAAGCAG	zwf	glucose-6-phosphate dehydrogenase zwf	WP_079299605.:	24570..26093	Pentose Phosphate	COG0364
085.1_WP_079299605.:	CUST_8754_PI442190723	HOT208_60m_810B23	085.1_WP_079299605.:	TCTTCAAGCTTCAAAACTTGCTGACGAAAATGAACCGTGGAATTGTTGCATAAGAGGTCA	zwf	glucose-6-phosphate dehydrogenase zwf	WP_079299605.:	24570..26093	Pentose Phosphate	COG0364
085.1_WP_079299605.:	CUST_8755_PI442190723	HOT208_60m_810B23	085.1_WP_079299605.:	CTTGCTGTTACTGCTATGGAACCCCCTGGAAAGTTTGAACCAGAAGCAATAAGAAATGAA	zwf	glucose-6-phosphate dehydrogenase zwf	WP_079299605.:	24570..26093	Pentose Phosphate	COG0364
085.1_WP_079299605.:	CUST_8756_PI442190723	HOT208_60m_810B23	085.1_WP_079299605.:	TCAGCCAAATGAAGGTGCTACTTTCAAATTTGAGGTAAAATCTCCCGGTTCTGGAATGAA	zwf	glucose-6-phosphate dehydrogenase zwf	WP_079299605.:	24570..26093	Pentose Phosphate	COG0364
085.1_WP_079299605.:	CUST_8757_PI442190723	HOT208_60m_810B23	085.1_WP_079299605.:	TGGGGACCTCCTGAGTCTGATCAATTAATTTCAAAAGATAATATGTTCTGGCGTAGACCT	zwf	glucose-6-phosphate dehydrogenase zwf	WP_079299605.:	24570..26093	Pentose Phosphate	COG0364
085.1_WP_079299607.:	CUST_8758_PI442190723	HOT208_60m_810B23	085.1_WP_079299607.:	ATTGGAGGGAATCATTGGCAATGATTTTTGATCCGCCCTCGAGGAGACAAATTTTAGATC	opcA	Glucose-6-P dehydrogenase subunit opcA	WP_079299607.:	26096..27397	Pentose Phosphate	COG3429
085.1_WP_079299607.:	CUST_8759_PI442190723	HOT208_60m_810B23	085.1_WP_079299607.:	TGGTTGTGGTGGAATGGAAGCTTGGATGAATCGCCCGAAATCTTCGAATTTTTTACTAAT	opcA	Glucose-6-P dehydrogenase subunit opcA	WP_079299607.:	26096..27397	Pentose Phosphate	COG3429
085.1_WP_079299607.:	CUST_8760_PI442190723	HOT208_60m_810B23	085.1_WP_079299607.:	GCTGTGAATCAGTGGAGTGTATGAGACTTGAAGCAGGGGGAATGGCTAATATGGAATTAA	opcA	Glucose-6-P dehydrogenase subunit opcA	WP_079299607.:	26096..27397	Pentose Phosphate	COG3429
085.1_WP_079299607.:	CUST_8761_PI442190723	HOT208_60m_810B23	085.1_WP_079299607.:	TCAAGCTTTGTTTTTGATTTCATGGATTAGCGATAAGCTTGGTTGGTCATTTCTGAGAGT	opcA	Glucose-6-P dehydrogenase subunit opcA	WP_079299607.:	26096..27397	Pentose Phosphate	COG3429
085.1_WP_079299607.:	CUST_8762_PI442190723	HOT208_60m_810B23	085.1_WP_079299607.:	TCCCTTATCTTTGGGAAATCCAAGTATTCATTTAGGACAAGTTATTGGATTGAGGCTGAT	opcA	Glucose-6-P dehydrogenase subunit opcA	WP_079299607.:	26096..27397	Pentose Phosphate	COG3429
085.1_WP_079299607.:	CUST_8763_PI442190723	HOT208_60m_810B23	085.1_WP_079299607.:	ACATCAGAGTATGATGTTAGTAAATTGTTGGGAAGTAGTAGAGGTAATACCAGCCCTCTT	opcA	Glucose-6-P dehydrogenase subunit opcA	WP_079299607.:	26096..27397	Pentose Phosphate	COG3429
087.1_WP_079299767.:	CUST_9248_PI442190723	HOT208_60m_810B23	087.1_WP_079299767.:	AGTTGGTCCAACAACGAAAATTGGTTCGATTTTGTATCCGATATCAAAATTAAATATCCA	eda	2-dehydro-3-deoxyphosphogluconate aldolase	WP_079299767.:	10142..10768	Entner-Dudoroff	COG0800
087.1_WP_079299767.:	CUST_9249_PI442190723	HOT208_60m_810B23	087.1_WP_079299767.:	AGTTCGATAAACATACTCAATTATAAAAATGTTGATTTCATTGCTGCTGGAGGACTATCA	eda	2-dehydro-3-deoxyphosphogluconate aldolase	WP_079299767.:	10142..10768	Entner-Dudoroff	COG0800
087.1_WP_079299767.:	CUST_9250_PI442190723	HOT208_60m_810B23	087.1_WP_079299767.:	GGATTAAATTTTTCGATGATGAAATTTTGGGATAAAGATCTTTTCAATTATGCGCAGTCA	eda	2-dehydro-3-deoxyphosphogluconate aldolase	WP_079299767.:	10142..10768	Entner-Dudoroff	COG0800
087.1_WP_079299767.:	CUST_9251_PI442190723	HOT208_60m_810B23	087.1_WP_079299767.:	AGAATTAATTTAGGCTCTGCCTCCATAGTTAATAAGCAATCAATAGAAGATTCTTTAAAA	eda	2-dehydro-3-deoxyphosphogluconate aldolase	WP_079299767.:	10142..10768	Entner-Dudoroff	COG0800
087.1_WP_079299767.:	CUST_9252_PI442190723	HOT208_60m_810B23	087.1_WP_079299767.:	AGATTCTTTTTCAGAGTACCTGAAAATTGAGTCTTTTTTTTTACTTATAAAACCTGAAGA	eda	2-dehydro-3-deoxyphosphogluconate aldolase	WP_079299767.:	10142..10768	Entner-Dudoroff	COG0800
087.1_WP_079299767.:	CUST_9253_PI442190723	HOT208_60m_810B23	087.1_WP_079299767.:	AGGATATAAAGCAGTTGTAATTGGAGATAAAGCTATCAAAAATAAAAAATTTGATCCAAA	eda	2-dehydro-3-deoxyphosphogluconate aldolase	WP_079299767.:	10142..10768	Entner-Dudoroff	COG0800
109.1_13	CUST_64_PI442190698	HOT208_60m_808M21	109.1_13	CAACTTGGTGCTGAGCATAGTCAAAGACTTGAAGCATATTTTCATGCAGTTCCTGGCATA	pdhB	Pyruvate dehydrogenase E1 component beta subunit		<1..493	Glycolysis/Krebs cycle	COG0022
109.1_13	CUST_65_PI442190698	HOT208_60m_808M21	109.1_13	TACAAGTGGAGGAAATTATAAAATACCAGCAGTAGTTCGAGGACCTGGTGGAGTTGGTCG	pdhB	Pyruvate dehydrogenase E1 component beta subunit		<1..493	Glycolysis/Krebs cycle	COG0022
109.1_13	CUST_66_PI442190698	HOT208_60m_808M21	109.1_13	TTTGTCTGAAGAATTACCTGAGGGTGATTATACTTGCGCTTTAGATCAGGCTGACGTAGT	pdhB	Pyruvate dehydrogenase E1 component beta subunit		<1..493	Glycolysis/Krebs cycle	COG0022
109.1_13	CUST_67_PI442190698	HOT208_60m_808M21	109.1_13	TGTTGCATGTAGTACGCCGACAAATGCTAAAGGTTTAATGAAAGCAGCTATAAGAGATGA	pdhB	Pyruvate dehydrogenase E1 component beta subunit		<1..493	Glycolysis/Krebs cycle	COG0022
109.1_13	CUST_68_PI442190698	HOT208_60m_808M21	109.1_13	TGGTCCACTAGGCTTAAGACCAATAGTAGAAGGAATGAATATGGGTTTTTTGCTTTTAGC	pdhB	Pyruvate dehydrogenase E1 component beta subunit		<1..493	Glycolysis/Krebs cycle	COG0022
109.1_13	CUST_69_PI442190698	HOT208_60m_808M21	109.1_13	TGAGACATCACTGCCATAAAGCTGTTGAAGAATTAGAAAAAAAAGGAATAGATGTTGAGT	pdhB	Pyruvate dehydrogenase E1 component beta subunit		<1..493	Glycolysis/Krebs cycle	COG0022
124.1_WP_079338991.:	CUST_962_PI442190698	HOT208_60m_808M21	124.1_WP_079338991.:	TGGGGGAATCAGTTGAGAATCCTCTTAAATATTGGGAATACAACTGGCTTTCAGCCTTCA	galE	UDP-glucose 4-epimerase	WP_079338991.:	36823..37878	Galactose metabolism	COG1087
124.1_WP_079338991.:	CUST_963_PI442190698	HOT208_60m_808M21	124.1_WP_079338991.:	TGGCCAACTAAAGATGGAACCGGGATAAGAGATTATATACATGTCATGGACTTAGCGGAA	galE	UDP-glucose 4-epimerase	WP_079338991.:	36823..37878	Galactose metabolism	COG1087
124.1_WP_079338991.:	CUST_964_PI442190698	HOT208_60m_808M21	124.1_WP_079338991.:	AGGTACAGGAATAGGGACTTCTGTTATGGAACTTGTCGAGATTTTCAAGACAGTTAATGA	galE	UDP-glucose 4-epimerase	WP_079338991.:	36823..37878	Galactose metabolism	COG1087
124.1_WP_079338991.:	CUST_965_PI442190698	HOT208_60m_808M21	124.1_WP_079338991.:	ACGTGGCAAACCTAAGATATTTTAATCCAATAGGTGCTCATTCTTCAGGATTAATTGGAG	galE	UDP-glucose 4-epimerase	WP_079338991.:	36823..37878	Galactose metabolism	COG1087
124.1_WP_079338991.:	CUST_966_PI442190698	HOT208_60m_808M21	124.1_WP_079338991.:	GCTATGGTTGAATTTGAATGCCGAAATATTGTTTTTAGTAGTAGTGCCACAATTTATGGC	galE	UDP-glucose 4-epimerase	WP_079338991.:	36823..37878	Galactose metabolism	COG1087
124.1_WP_079338991.:	CUST_967_PI442190698	HOT208_60m_808M21	124.1_WP_079338991.:	ACTTTGGATGAGAAATCAGTAATTAACCCAATAAATCCATACGGTAATACAAAAGCAGCA	galE	UDP-glucose 4-epimerase	WP_079338991.:	36823..37878	Galactose metabolism	COG1087
126.1_WP_079339083.:	CUST_1262_PI442190698	HOT208_60m_808M21	126.1_WP_079339083.:	GGCGCGTATCTACAGAAGTTGATGCAAGATTGAGCTTTGATACCGAAGCTACAGTAGAAA	tal	Transaldolase	WP_079339083.:	21116..22117	Pentose Phosphate	COG0176
126.1_WP_079339083.:	CUST_1263_PI442190698	HOT208_60m_808M21	126.1_WP_079339083.:	AGATTGCTGCAACTTGGGAGGGAATTAAGGCAGCTGAAATTTTGGAAAAAGAGGGTATTA	tal	Transaldolase	WP_079339083.:	21116..22117	Pentose Phosphate	COG0176
126.1_WP_079339083.:	CUST_1264_PI442190698	HOT208_60m_808M21	126.1_WP_079339083.:	TGGAAGATCAAATGGCAAGTGAAAAGCTCAGTGAAGGTATCACTGGATTCAGTAAGGCTA	tal	Transaldolase	WP_079339083.:	21116..22117	Pentose Phosphate	COG0176
126.1_WP_079339083.:	CUST_1265_PI442190698	HOT208_60m_808M21	126.1_WP_079339083.:	TTCTGCCAAGCGGTAACTTGTGCTAATGCAAACATAACTCTAATTTCTCCTTTCGTAGGC	tal	Transaldolase	WP_079339083.:	21116..22117	Pentose Phosphate	COG0176
126.1_WP_079339083.:	CUST_1266_PI442190698	HOT208_60m_808M21	126.1_WP_079339083.:	GGATTGGCATAAAGCAAAAACTGGTAAAACTAGTTTTGTTGGATCTGAAGACCCTGGTGT	tal	Transaldolase	WP_079339083.:	21116..22117	Pentose Phosphate	COG0176
126.1_WP_079339083.:	CUST_1267_PI442190698	HOT208_60m_808M21	126.1_WP_079339083.:	AGCAGGTTGCGATCTTTTAACAATCGCACCAAAATTTCTTGAGGAACTGAAAAAAGAAAA	tal	Transaldolase	WP_079339083.:	21116..22117	Pentose Phosphate	COG0176
129.1_WP_079339271.:	CUST_1910_PI442190698	HOT208_60m_808M21	129.1_WP_079339271.:	CTCTAGGTGCCTTAATCGCTTTATTTGAGAGGGCTGTTTCCTTCTACGCTGAATTGGTAA	pgi	Glucose-6-phosphate isomerase	WP_079339271.:	14334..15917	Glycolysis/Gluconeogenesis	COG0166
129.1_WP_079339271.:	CUST_1911_PI442190698	HOT208_60m_808M21	129.1_WP_079339271.:	ATCCAGCAGCACTATTATCATCCGCATGGTATTTAACTGGGGATGGTATTGGAAAGAGAG	pgi	Glucose-6-phosphate isomerase	WP_079339271.:	14334..15917	Glycolysis/Gluconeogenesis	COG0166
129.1_WP_079339271.:	CUST_1912_PI442190698	HOT208_60m_808M21	129.1_WP_079339271.:	TGATCAACCTGGAGTTGAAGCTGGAAAGAAAGCAGCAGCAAATATTATAGAGTATCAGCA	pgi	Glucose-6-phosphate isomerase	WP_079339271.:	14334..15917	Glycolysis/Gluconeogenesis	COG0166
129.1_WP_079339271.:	CUST_1913_PI442190698	HOT208_60m_808M21	129.1_WP_079339271.:	ACTCCGTGAAATGTGTTTCGGTAATGATAATTATTTAGTTAAGGGCGATTGGTCAAATCC	pgi	Glucose-6-phosphate isomerase	WP_079339271.:	14334..15917	Glycolysis/Gluconeogenesis	COG0166
129.1_WP_079339271.:	CUST_1914_PI442190698	HOT208_60m_808M21	129.1_WP_079339271.:	TGGTAATAAAGGATCTACAGATCAACATGCTTATGTTCAGCAACTGAGAGATGGTATTGA	pgi	Glucose-6-phosphate isomerase	WP_079339271.:	14334..15917	Glycolysis/Gluconeogenesis	COG0166
129.1_WP_079339271.:	CUST_1915_PI442190698	HOT208_60m_808M21	129.1_WP_079339271.:	AGGAACCAGATCAGCACTTTCTAGTGAAAAAAGACAAAGTATCACTATCACCTTAGAAAA	pgi	Glucose-6-phosphate isomerase	WP_079339271.:	14334..15917	Glycolysis/Gluconeogenesis	COG0166
132.1_WP_079339413.:	CUST_2404_PI442190698	HOT208_60m_808M21	132.1_WP_079339413.:	TCTAGGTTCCACAGGAAGAAATATTGGTGCGGGCATGACTGGTGGAATAGCTTTTATAAT	glsF	Ferredoxin-dependent glutamate synthase	WP_079339413.:	14941..19506	Nitrogen Metabolism	COG0069
132.1_WP_079339413.:	CUST_2405_PI442190698	HOT208_60m_808M21	132.1_WP_079339413.:	TTTGCAGTAAGAAATAGTGGTGCTATAGCGGTAACAGAAGGAGCAGGTGATCATTGTTGT	glsF	Ferredoxin-dependent glutamate synthase	WP_079339413.:	14941..19506	Nitrogen Metabolism	COG0069
132.1_WP_079339413.:	CUST_2406_PI442190698	HOT208_60m_808M21	132.1_WP_079339413.:	GAAAATATCAGGCGAAATCGCAGAACTTCATGGCAATACTAGCTTCAATGGCGAACTCAA	glsF	Ferredoxin-dependent glutamate synthase	WP_079339413.:	14941..19506	Nitrogen Metabolism	COG0069
132.1_WP_079339413.:	CUST_2407_PI442190698	HOT208_60m_808M21	132.1_WP_079339413.:	ACTTCCTCCGAACAGGTTATTTTAGGAAACACCTGTCTTTATGGAGCAACAGGTGGAAAA	glsF	Ferredoxin-dependent glutamate synthase	WP_079339413.:	14941..19506	Nitrogen Metabolism	COG0069
132.1_WP_079339413.:	CUST_2408_PI442190698	HOT208_60m_808M21	132.1_WP_079339413.:	TGCAGGACAAAGCTTTGGGGCATTTTTATTGAAGGGAATGAATGTTCAATTAATCGGAGA	glsF	Ferredoxin-dependent glutamate synthase	WP_079339413.:	14941..19506	Nitrogen Metabolism	COG0069
132.1_WP_079339413.:	CUST_2409_PI442190698	HOT208_60m_808M21	132.1_WP_079339413.:	TGGTCTCATTTTAAGAGTATTTTCAAATTGATTGTTCCCCCAAGCGAAGAAGAGATGCTT	glsF	Ferredoxin-dependent glutamate synthase	WP_079339413.:	14941..19506	Nitrogen Metabolism	COG0069
132.1_WP_011819135.:	CUST_2458_PI442190698	HOT208_60m_808M21	132.1_WP_011819135.:	CCTCTTCGGTCACTTAGTTTGGGCTACTGGTTTCATGTTCTTAATATCATGGCGTGGTTA	psaB	Photosystem I P700 chlorophyll A apoprotein A2 PsaB	WP_011819135.:	25910..28138	Photosystem I	COG2885
132.1_WP_011819135.:	CUST_2459_PI442190698	HOT208_60m_808M21	132.1_WP_011819135.:	TGGACGTGGAGGTACTTGTGATAGTTCATCTTGGGACGCTATGTACTTAGCTATGTTCTG	psaB	Photosystem I P700 chlorophyll A apoprotein A2 PsaB	WP_011819135.:	25910..28138	Photosystem I	COG2885
132.1_WP_011819135.:	CUST_2460_PI442190698	HOT208_60m_808M21	132.1_WP_011819135.:	AAGCCAGTTGCGCTTTCAATTGTTCAAGCTAGATTAGTTGGACTAGCACATTTCACGATT	psaB	Photosystem I P700 chlorophyll A apoprotein A2 PsaB	WP_011819135.:	25910..28138	Photosystem I	COG2885
132.1_WP_011819135.:	CUST_2461_PI442190698	HOT208_60m_808M21	132.1_WP_011819135.:	AAATTTACTAGCATGGGTAACTTTCTACTGGCATTGGAAACACCTAGCAATTTGGCAGGG	psaB	Photosystem I P700 chlorophyll A apoprotein A2 PsaB	WP_011819135.:	25910..28138	Photosystem I	COG2885
132.1_WP_011819135.:	CUST_2462_PI442190698	HOT208_60m_808M21	132.1_WP_011819135.:	GTGCTTTCTTACCTATCGGTCCTGCAGATTTCTTAGTTCACCATGCTATCGCTTTAGGTC	psaB	Photosystem I P700 chlorophyll A apoprotein A2 PsaB	WP_011819135.:	25910..28138	Photosystem I	COG2885
132.1_WP_011819135.:	CUST_2463_PI442190698	HOT208_60m_808M21	132.1_WP_011819135.:	TGGCAAGAATTAATTGAAACGTTAGTTTGGGCACATCAGCGTACTCCAATTGCTAACCTT	psaB	Photosystem I P700 chlorophyll A apoprotein A2 PsaB	WP_011819135.:	25910..28138	Photosystem I	COG2885
132.1_WP_079339431.:	CUST_2464_PI442190698	HOT208_60m_808M21	132.1_WP_079339431.:	AGCTCACTTTATATGGGCTTTCAGTTTAATGTTCCTCTTCAGCGGAAGAGGATATTGGCA	psaA	Photosystem I P700 chlorophyll A apoprotein A1 PsaA	WP_079339431.:	28166..30469	Photosystem I	COG2885
132.1_WP_079339431.:	CUST_2465_PI442190698	HOT208_60m_808M21	132.1_WP_079339431.:	TTTCCACTTCTCTTGGAAAATGCAGAGTGATGTTTGGGGACTTACTGGTGGTAATTTTGC	psaA	Photosystem I P700 chlorophyll A apoprotein A1 PsaA	WP_079339431.:	28166..30469	Photosystem I	COG2885
132.1_WP_079339431.:	CUST_2466_PI442190698	HOT208_60m_808M21	132.1_WP_079339431.:	ATGGTTGGTTAAGAGATTTCCTCTGGGCTCAAGCTTCTCAAGTATTGACAAGCTATGGTC	psaA	Photosystem I P700 chlorophyll A apoprotein A1 PsaA	WP_079339431.:	28166..30469	Photosystem I	COG2885
132.1_WP_079339431.:	CUST_2467_PI442190698	HOT208_60m_808M21	132.1_WP_079339431.:	CCTCGAGCTTTATCTATTACTCAGGGACGTGCAGTAGGTGTTACACACTTTCTTGTAGGT	psaA	Photosystem I P700 chlorophyll A apoprotein A1 PsaA	WP_079339431.:	28166..30469	Photosystem I	COG2885
132.1_WP_079339431.:	CUST_2468_PI442190698	HOT208_60m_808M21	132.1_WP_079339431.:	AGAAGTTGGTGGAAAGGTAGCTATAGCGCCAATTCCATTAGGGACAGCTGATTTAATGAT	psaA	Photosystem I P700 chlorophyll A apoprotein A1 PsaA	WP_079339431.:	28166..30469	Photosystem I	COG2885
132.1_WP_079339431.:	CUST_2469_PI442190698	HOT208_60m_808M21	132.1_WP_079339431.:	GGAAGAGGTGGTACATGTCAAGTTTCTTCATGGGATCACGTTTTCTTAGCTCTCTTCTGG	psaA	Photosystem I P700 chlorophyll A apoprotein A1 PsaA	WP_079339431.:	28166..30469	Photosystem I	COG2885
133.1_WP_079339503.:	CUST_2720_PI442190698	HOT208_60m_808M21	133.1_WP_079339503.:	CGTTGATAACTGGCGTTGGCAAGGGGTTCCTTTTTATTTGAGAACAGGTAAAAGACTACC	zwf	glucose-6-phosphate dehydrogenase zwf	WP_079339503.:	46796..48319	Pentose Phosphate	COG0364
133.1_WP_079339503.:	CUST_2721_PI442190698	HOT208_60m_808M21	133.1_WP_079339503.:	ACAAATGCTTGCTGTTACTGCTATGGAACCTCCTGGAAAGTTTGAACCAGAAGCAATAAG	zwf	glucose-6-phosphate dehydrogenase zwf	WP_079339503.:	46796..48319	Pentose Phosphate	COG0364
133.1_WP_079339503.:	CUST_2722_PI442190698	HOT208_60m_808M21	133.1_WP_079339503.:	TCTTCAAGCTTCAAAACTTGCTGACGAAAATGAACCGTGGAATTGTTGCATAAGAGGTCA	zwf	glucose-6-phosphate dehydrogenase zwf	WP_079339503.:	46796..48319	Pentose Phosphate	COG0364
133.1_WP_079339503.:	CUST_2723_PI442190698	HOT208_60m_808M21	133.1_WP_079339503.:	ATGCTTTCTGATCCAACCCTATTTACTCGAAGTGATGAAGTAGAAGCAGCCTGGAAACTC	zwf	glucose-6-phosphate dehydrogenase zwf	WP_079339503.:	46796..48319	Pentose Phosphate	COG0364
133.1_WP_079339503.:	CUST_2724_PI442190698	HOT208_60m_808M21	133.1_WP_079339503.:	TCCTGCCCCAAATCAACTTATCCTTAGAATTCAGCCAAATGAAGGTGCTACTTTCAAATT	zwf	glucose-6-phosphate dehydrogenase zwf	WP_079339503.:	46796..48319	Pentose Phosphate	COG0364
133.1_WP_079339503.:	CUST_2725_PI442190698	HOT208_60m_808M21	133.1_WP_079339503.:	GACCTGTTGAGATGGAATTTTCTTATGATGAATCATTTGGAGAACCCTCAGATGAAGGCT	zwf	glucose-6-phosphate dehydrogenase zwf	WP_079339503.:	46796..48319	Pentose Phosphate	COG0364
133.1_WP_079339505.:	CUST_2726_PI442190698	HOT208_60m_808M21	133.1_WP_079339505.:	TGGTTGTGGTGGAATGGAAGCTTGGATGAATCGCCCGAAATCTTCGAATTTTTTACTAAT	opcA	Glucose-6-P dehydrogenase subunit opcA	WP_079339505.:	48322..49623	Pentose Phosphate	COG3429
133.1_WP_079339505.:	CUST_2727_PI442190698	HOT208_60m_808M21	133.1_WP_079339505.:	GCTGTGAATCAGTGGAGTGTATGAGACTTGAAGCAGGGGGAATGGCTAATATGGAATTAA	opcA	Glucose-6-P dehydrogenase subunit opcA	WP_079339505.:	48322..49623	Pentose Phosphate	COG3429
133.1_WP_079339505.:	CUST_2728_PI442190698	HOT208_60m_808M21	133.1_WP_079339505.:	TGGGTTAGATTGAAAAATTGGAGAGAATCATTGGCAATGATTTTTGATCCCCCCTCTAGG	opcA	Glucose-6-P dehydrogenase subunit opcA	WP_079339505.:	48322..49623	Pentose Phosphate	COG3429
133.1_WP_079339505.:	CUST_2729_PI442190698	HOT208_60m_808M21	133.1_WP_079339505.:	CGCAGGAGATCATATGATTCAAGCTTTGTTTTTGGTTTCATGGATTAGCGATAAGCTTGG	opcA	Glucose-6-P dehydrogenase subunit opcA	WP_079339505.:	48322..49623	Pentose Phosphate	COG3429
133.1_WP_079339505.:	CUST_2730_PI442190698	HOT208_60m_808M21	133.1_WP_079339505.:	TCCCTTATCTTTGGGAAATCCAAGTATTCATTTAGGACAAGTTATTGGATTGAGGCTGAT	opcA	Glucose-6-P dehydrogenase subunit opcA	WP_079339505.:	48322..49623	Pentose Phosphate	COG3429
133.1_WP_079339505.:	CUST_2731_PI442190698	HOT208_60m_808M21	133.1_WP_079339505.:	ACATCAGAGTATGATGTTAGTAAATTGTTGGGAAGTAGTAGAGGTAATACCAGCCCTCTT	opcA	Glucose-6-P dehydrogenase subunit opcA	WP_079339505.:	48322..49623	Pentose Phosphate	COG3429
133.1_WP_079339553.:	CUST_2890_PI442190698	HOT208_60m_808M21	133.1_WP_079339553.:	GTCATTTCTCATTCTGAAAAGTCCCTTGAGTATCCAGCTGGTGGTGGATCTATTATCGCG	rbsK	Sugar kinase, ribokinase family	WP_079339553.:	77257..78120	Pentose Phosphate	COG0524
133.1_WP_079339553.:	CUST_2891_PI442190698	HOT208_60m_808M21	133.1_WP_079339553.:	CACTATTAAAACCGAGGGAGAGAAGGGAGGCATACTATTCCCTGGAGGAAGATATAAGGC	rbsK	Sugar kinase, ribokinase family	WP_079339553.:	77257..78120	Pentose Phosphate	COG0524
133.1_WP_079339553.:	CUST_2892_PI442190698	HOT208_60m_808M21	133.1_WP_079339553.:	TGATTCTTATGGATGTGGCGATTCTTTTGCTGCGGGTATTCTTTATGGAATGGCATCAAA	rbsK	Sugar kinase, ribokinase family	WP_079339553.:	77257..78120	Pentose Phosphate	COG0524
133.1_WP_079339553.:	CUST_2893_PI442190698	HOT208_60m_808M21	133.1_WP_079339553.:	TCAGAGATTTTTAAAATGGCAAGATCAGCTTCCATACTGTGTACAACACCAAGGGTAGGA	rbsK	Sugar kinase, ribokinase family	WP_079339553.:	77257..78120	Pentose Phosphate	COG0524
133.1_WP_079339553.:	CUST_2894_PI442190698	HOT208_60m_808M21	133.1_WP_079339553.:	AATTGACTCTCGAGGTGAAAGAGCAATTACAGTTATTGGAGAAAGGTTAGCTCCAACTCA	rbsK	Sugar kinase, ribokinase family	WP_079339553.:	77257..78120	Pentose Phosphate	COG0524
133.1_WP_079339553.:	CUST_2895_PI442190698	HOT208_60m_808M21	133.1_WP_079339553.:	AGTTTAAATCTTGCTAAAGTAATGGGAAGAGACGCCAGTGAATTTTTCGGTCCATATGCA	rbsK	Sugar kinase, ribokinase family	WP_079339553.:	77257..78120	Pentose Phosphate	COG0524
135.1_WP_079339707.:	CUST_3335_PI442190698	HOT208_60m_808M21	135.1_WP_079339707.:	GCCACAGGAAGTAGCTTAATGCCGCAGAAAAAAAATCCTGACGTTCCAGAATTGATAAGA	argH	Argininosuccinate lyase	WP_079339707.:	7901..9280	Amino Acid metabolism	COG0165
135.1_WP_079339707.:	CUST_3337_PI442190698	HOT208_60m_808M21	135.1_WP_079339707.:	AGCGAAGGTGGCACAAGTTTTGTTCAGGTAGAAAAAGAGGTAAAAAATTGGCAAAAAAGA	argH	Argininosuccinate lyase	WP_079339707.:	7901..9280	Amino Acid metabolism	COG0165
135.1_WP_079339707.:	CUST_3338_PI442190698	HOT208_60m_808M21	135.1_WP_079339707.:	TGTTCCTTTTAGGACCGCTTATCAAGTTGTTGGTCAAATGGTTAAATATTGCCTCGAGAG	argH	Argininosuccinate lyase	WP_079339707.:	7901..9280	Amino Acid metabolism	COG0165
135.1_WP_079339707.:	CUST_3339_PI442190698	HOT208_60m_808M21	135.1_WP_079339707.:	ACAGTATTGACGCGGTAAGTGATAGAGATTTTTGTATAGAGTTTGTTTCTGCATCTGCTT	argH	Argininosuccinate lyase	WP_079339707.:	7901..9280	Amino Acid metabolism	COG0165
145.1_WP_079339783.:	CUST_3695_PI442190698	HOT208_60m_808M21	145.1_WP_079339783.:	TCAGGCTGATGGATTAAAAGGCATCCTTAATAGGGATTTTGATGAATGGTCAAATTTTGA	ppc	Phosphoenolpyruvate carboxylase	WP_079339783.:	1151..1528	Krebs cycle and related genes	COG2352
145.1_WP_079339783.:	CUST_3696_PI442190698	HOT208_60m_808M21	145.1_WP_079339783.:	TGAATCAAAATTCTGTCAAAACAATTGGTATGAATGATGAACCAAGAAAAGATTCATACT	ppc	Phosphoenolpyruvate carboxylase	WP_079339783.:	1151..1528	Krebs cycle and related genes	COG2352
145.1_WP_079339783.:	CUST_3697_PI442190698	HOT208_60m_808M21	145.1_WP_079339783.:	ATCGATATTAAATGCGACTGTTGTGAATATAATGATTTAATCCCAAATGATTTTGAGAGT	ppc	Phosphoenolpyruvate carboxylase	WP_079339783.:	1151..1528	Krebs cycle and related genes	COG2352
145.1_WP_079339783.:	CUST_3698_PI442190698	HOT208_60m_808M21	145.1_WP_079339783.:	TTGAGAGTATTAAAAAAGAAAAATGTTTTGGTAAAAAAAGTGCTTACATGAT	ppc	Phosphoenolpyruvate carboxylase	WP_079339783.:	1151..1528	Krebs cycle and related genes	COG2352
147.1_WP_079339949.:	CUST_4267_PI442190698	HOT208_60m_808M21	147.1_WP_079339949.:	ACAGCAGAAGTGAACTATTGAGGGGAGCACTTTTAACTATTAATGGTATAGCAGCTGGCA	ppc	Phosphoenolpyruvate carboxylase	WP_079339949.:	28464..31433	Krebs cycle and related genes	COG2352
147.1_WP_079339949.:	CUST_4268_PI442190698	HOT208_60m_808M21	147.1_WP_079339949.:	AGGGTTTTTGCAAGTTTCACTTCTAAGAAGATTAAGAGACCAGACAAGACAACCTCCAAT	ppc	Phosphoenolpyruvate carboxylase	WP_079339949.:	28464..31433	Krebs cycle and related genes	COG2352
147.1_WP_079339949.:	CUST_4269_PI442190698	HOT208_60m_808M21	147.1_WP_079339949.:	CCAAGTTGGTTTGGAGTCGGCACTGCATTGTCATCTGAATTAAATTCTGATCCTCAACAA	ppc	Phosphoenolpyruvate carboxylase	WP_079339949.:	28464..31433	Krebs cycle and related genes	COG2352
147.1_WP_079339949.:	CUST_4270_PI442190698	HOT208_60m_808M21	147.1_WP_079339949.:	GGTGCAAAAGATTTATCAAGTTTAAGAGCTATTCCATGGGTGTTTGGTTGGACACAAAGT	ppc	Phosphoenolpyruvate carboxylase	WP_079339949.:	28464..31433	Krebs cycle and related genes	COG2352
147.1_WP_079339949.:	CUST_4272_PI442190698	HOT208_60m_808M21	147.1_WP_079339949.:	AGTCTTCCTGAACTAGCCTTGTACAATCTTGAGACTGTTACTACAGCGGTAATTCAAAAT	ppc	Phosphoenolpyruvate carboxylase	WP_079339949.:	28464..31433	Krebs cycle and related genes	COG2352
152.1_WP_079340131.:	CUST_4987_PI442190698	HOT208_60m_808M21	152.1_WP_079340131.:	GGCAGCACAAGCAGCAATGAATAGTCCTTTATTAGAAGCAGCAAGAATTGATGGAGCTAA	ftsZ	cell division protein FtsZ	WP_079340131.:	11840..12955	Cell Division	COG0206
152.1_WP_079340131.:	CUST_4988_PI442190698	HOT208_60m_808M21	152.1_WP_079340131.:	TATCGCAGGAGCTCCTCTTCAAGAAGCATTTAGGAATGCTGATGATGTTCTAAGAATGGG	ftsZ	cell division protein FtsZ	WP_079340131.:	11840..12955	Cell Division	COG0206
152.1_WP_079340131.:	CUST_4989_PI442190698	HOT208_60m_808M21	152.1_WP_079340131.:	GGCTGCGTTATAAATATTACTGGAGGCAAGGACATGACTTTAGAAGACATGACCTCTGCA	ftsZ	cell division protein FtsZ	WP_079340131.:	11840..12955	Cell Division	COG0206
152.1_WP_079340131.:	CUST_4990_PI442190698	HOT208_60m_808M21	152.1_WP_079340131.:	ACTTGCCCAGGATTAGTTAATGTCGATTTTGCAGACGTTAGATCAGTTATGACGGAAGCT	ftsZ	cell division protein FtsZ	WP_079340131.:	11840..12955	Cell Division	COG0206
152.1_WP_079340131.:	CUST_4991_PI442190698	HOT208_60m_808M21	152.1_WP_079340131.:	GGAGGGGATCGCAAGACTAGCTGAAAACGTTGATACTCTGATAGTTATTCCAAATGACCG	ftsZ	cell division protein FtsZ	WP_079340131.:	11840..12955	Cell Division	COG0206
152.1_WP_079340131.:	CUST_4992_PI442190698	HOT208_60m_808M21	152.1_WP_079340131.:	TGTCGCAGAAGTAGCCAAACAAAGTGGGGCTTTAACAGTAGGTATAGTAACCAAACCATT	ftsZ	cell division protein FtsZ	WP_079340131.:	11840..12955	Cell Division	COG0206
156.1_1017	CUST_5302_PI442190698	HOT208_60m_808M21	156.1_1017	TCGATTTTGTATCCGATATCAAAATTAAATATCCAAGAATTAATTTAGGCTCTGCCTCCA	eda	2-dehydro-3-deoxyphosphogluconate aldolase		4349..4971	Entner-Dudoroff	COG0800
156.1_1017	CUST_5303_PI442190698	HOT208_60m_808M21	156.1_1017	GGATTAAATTTTTCGATGATGAAATTTTGGGATAAAGATCTTTTCAATTATGCGCAGTCA	eda	2-dehydro-3-deoxyphosphogluconate aldolase		4349..4971	Entner-Dudoroff	COG0800
156.1_1017	CUST_5304_PI442190698	HOT208_60m_808M21	156.1_1017	AGATAGTTCGATAACTATACTCAACTATAAAAATATTGATTTCATTGCTGCTGGAGGACT	eda	2-dehydro-3-deoxyphosphogluconate aldolase		4349..4971	Entner-Dudoroff	COG0800
156.1_1017	CUST_5305_PI442190698	HOT208_60m_808M21	156.1_1017	AGGGTATAAAGCAGTTGTAATTGGAGATAAAGCTATCAAAAATAAAAAATTTGATCCAAA	eda	2-dehydro-3-deoxyphosphogluconate aldolase		4349..4971	Entner-Dudoroff	COG0800
156.1_1017	CUST_5306_PI442190698	HOT208_60m_808M21	156.1_1017	GATCTTAAGGAAGCGATAGATTTAAATTGCAACATTATCAAAATTTACCCAATAAAAAGT	eda	2-dehydro-3-deoxyphosphogluconate aldolase		4349..4971	Entner-Dudoroff	COG0800
156.1_1017	CUST_5307_PI442190698	HOT208_60m_808M21	156.1_1017	ACTTAGTAAAATTAGGATTAAAGAATATTTAAATAAGTTGGTCTAACAACGAAAATTGGT	eda	2-dehydro-3-deoxyphosphogluconate aldolase		4349..4971	Entner-Dudoroff	COG0800
159.1_WP_079340246.:	CUST_5381_PI442190698	HOT208_60m_808M21	159.1_WP_079340246.:	TGGGGCAAATTACTAGGAATCGCATTCGCAGGGAAAAGTGGAAATAAAAATGCCTCTATT	minC	septum site-determining protein MinC	WP_079340246.:	6475..7137	Cell Division	COG0850
159.1_WP_079340246.:	CUST_5382_PI442190698	HOT208_60m_808M21	159.1_WP_079340246.:	ATGGAAACCTATGCATTATTGGAGACGTTAACCCAGGAGCAATAGTTTCCGCTAAGGAAA	minC	septum site-determining protein MinC	WP_079340246.:	6475..7137	Cell Division	COG0850
159.1_WP_079340246.:	CUST_5383_PI442190698	HOT208_60m_808M21	159.1_WP_079340246.:	AGATGTGATAGCTATTGGTCCAAAGGATAAGCCCAAAAATTGTTATCCAGAAGTTGCGGT	minC	septum site-determining protein MinC	WP_079340246.:	6475..7137	Cell Division	COG0850
159.1_WP_079340246.:	CUST_5384_PI442190698	HOT208_60m_808M21	159.1_WP_079340246.:	TCAATTCAAAAAAGAAAGACGATATTCTTCACAATGGAACAGTACGATCGGGTGAAAGAA	minC	septum site-determining protein MinC	WP_079340246.:	6475..7137	Cell Division	COG0850
159.1_WP_079340246.:	CUST_5385_PI442190698	HOT208_60m_808M21	159.1_WP_079340246.:	TTCCATCAAGGAACCTTTAGAAGCAAAAATTTTCGTAGTCAATGAGTCAATAAGTTCTCA	minC	septum site-determining protein MinC	WP_079340246.:	6475..7137	Cell Division	COG0850
159.1_WP_079340246.:	CUST_5386_PI442190698	HOT208_60m_808M21	159.1_WP_079340246.:	AGAGAAACAATCCTGAGTGGAAAATCTTTAAAAATAGATTCAGCTTTTATAGAAGAGCAA	minC	septum site-determining protein MinC	WP_079340246.:	6475..7137	Cell Division	COG0850
159.1_WP_079340248.:	CUST_5387_PI442190698	HOT208_60m_808M21	159.1_WP_079340248.:	ATTGGTTAGACATAAAAAGGAGCCTAATCTTGCCCTTTTACCTGCTGGAGATCCAAGGAT	minD	septum site-determining protein MinD	WP_079340248.:	7249..8064	Cell Division	COG2894
159.1_WP_079340248.:	CUST_5388_PI442190698	HOT208_60m_808M21	159.1_WP_079340248.:	CACAATATTAATTTGCTCAGGCAAAGGAGGGGTTGGCAAAACAACTTTAACTGCAAACCT	minD	septum site-determining protein MinD	WP_079340248.:	7249..8064	Cell Division	COG2894
159.1_WP_079340248.:	CUST_5389_PI442190698	HOT208_60m_808M21	159.1_WP_079340248.:	AGAGTTCGCCCTAACATGATGGCTAGTCAAGAAATGTTATCCATCGAAGATGTTCAAGGG	minD	septum site-determining protein MinD	WP_079340248.:	7249..8064	Cell Division	COG2894
159.1_WP_079340248.:	CUST_5390_PI442190698	HOT208_60m_808M21	159.1_WP_079340248.:	GCACTTGCTAATAGCGGAGCGACAACTGCTGTATTAGATGCTGATTTTGGCTTAAGAAAT	minD	septum site-determining protein MinD	WP_079340248.:	7249..8064	Cell Division	COG2894
159.1_WP_079340248.:	CUST_5391_PI442190698	HOT208_60m_808M21	159.1_WP_079340248.:	TTAGTAGATTGCCCTGCTGGTGTAGAAGATGGCTTTAAAAATGCTCTAGCAGCCTGTAAA	minD	septum site-determining protein MinD	WP_079340248.:	7249..8064	Cell Division	COG2894
159.1_WP_079340248.:	CUST_5392_PI442190698	HOT208_60m_808M21	159.1_WP_079340248.:	ACTAACCCAGAATTATCCGCAGTGCGGGATGCGGATAGAGTAATAGGGATTCTTAATACT	minD	septum site-determining protein MinD	WP_079340248.:	7249..8064	Cell Division	COG2894
159.1_WP_079340250.:	CUST_5393_PI442190698	HOT208_60m_808M21	159.1_WP_079340250.:	TCAGGAGAAATAAAATTCAAAAAAACTGATAAAACTGACAAGGCTGATAAATCTAACAAA	minE	septum site-determining protein MinE	WP_079340250.:	8066..8404	Cell Division	COG0851
159.1_WP_079340250.:	CUST_5394_PI442190698	HOT208_60m_808M21	159.1_WP_079340250.:	AGCAGTAAGTTTAGAGACTGAGGATAGAATGACTGCATTAGTTGCCAATTTACCAATCAA	minE	septum site-determining protein MinE	WP_079340250.:	8066..8404	Cell Division	COG0851
159.1_WP_079340250.:	CUST_5395_PI442190698	HOT208_60m_808M21	159.1_WP_079340250.:	AGTTCCTTAACAACTGATCTTTTAGATAAAATGAGGAAAGAAATTCTTGATGTTGTTGCT	minE	septum site-determining protein MinE	WP_079340250.:	8066..8404	Cell Division	COG0851
159.1_WP_079340250.:	CUST_5396_PI442190698	HOT208_60m_808M21	159.1_WP_079340250.:	AGGCAGAGAAACGTCTAGTGCAAATACAGCTAGAGAAAGATTACAACTTGTACTTGCTCA	minE	septum site-determining protein MinE	WP_079340250.:	8066..8404	Cell Division	COG0851
171.1_1083	CUST_5634_PI442190698	HOT208_60m_808M21	171.1_1083	ATGGTTTGCACTCAGATAATGGGTCTTGAATATGCAGTATCAATGGCAAATTCTAGCGGC	fumC	class II fumarate hydratase		746..1469	Krebs cycle and related genes	COG0114
171.1_1083	CUST_5635_PI442190698	HOT208_60m_808M21	171.1_1083	TTCCTCAAAATGAACCTGGAAGTTCTATCATGCCAGGCAAAGTAAATCCAACTCAATGCG	fumC	class II fumarate hydratase		746..1469	Krebs cycle and related genes	COG0114
171.1_1083	CUST_5636_PI442190698	HOT208_60m_808M21	171.1_1083	AGTTACAGCCATAGTGCCAAAAGTTGGTTATGAAAAAGCAGCTGAAATTGCAAACCTTGC	fumC	class II fumarate hydratase		746..1469	Krebs cycle and related genes	COG0114
171.1_1083	CUST_5637_PI442190698	HOT208_60m_808M21	171.1_1083	AAAAGAGGCAACACTTAAATTAGGTTATTTAAACGAAGATGAATTTGATGAAGCAATGAA	fumC	class II fumarate hydratase		746..1469	Krebs cycle and related genes	COG0114
171.1_1083	CUST_5638_PI442190698	HOT208_60m_808M21	171.1_1083	AGAAAACTTCAGAATAAAATTAGTTGATGGTATGGAGCCTAATCAAAAGAAAATGAAGTT	fumC	class II fumarate hydratase		746..1469	Krebs cycle and related genes	COG0114
171.1_1083	CUST_5639_PI442190698	HOT208_60m_808M21	171.1_1083	GGCACTTTACAGATGAATGAATATAAGCCTCTTATTGGATTTAATATTCTCACAAGT	fumC	class II fumarate hydratase		746..1469	Krebs cycle and related genes	COG0114
174.1_WP_079340310.:	CUST_5662_PI442190698	HOT208_60m_808M21	174.1_WP_079340310.:	TCATGCGCTAAGTGCGGGTGTTCCCTCATTTGAAGTAATGAGTGAACTTTTTGGTAAATC	pdhA	Pyruvate dehydrogenase E1 component alpha subunit	WP_079340310.:	714..1787	Glycolysis/Krebs cycle	COG1071
174.1_WP_079340310.:	CUST_5663_PI442190698	HOT208_60m_808M21	174.1_WP_079340310.:	ATGGCTCATGATAGAGCTACTAGTAATCCTGAAATCTGGAGAAAAGCGTCTGCTTTTGGC	pdhA	Pyruvate dehydrogenase E1 component alpha subunit	WP_079340310.:	714..1787	Glycolysis/Krebs cycle	COG1071
174.1_WP_079340310.:	CUST_5664_PI442190698	HOT208_60m_808M21	174.1_WP_079340310.:	AGATTGATGCAGAGATATCTGAGTCAGTTAAAAATGCTATTGAAGCACCCGAACCCCCTT	pdhA	Pyruvate dehydrogenase E1 component alpha subunit	WP_079340310.:	714..1787	Glycolysis/Krebs cycle	COG1071
174.1_WP_079340310.:	CUST_5665_PI442190698	HOT208_60m_808M21	174.1_WP_079340310.:	TCCAGTTGCCTTAGGGGCAGCCTTTTCAAGTAAATACAAAAAGGAAGTTGCTGGAAATAG	pdhA	Pyruvate dehydrogenase E1 component alpha subunit	WP_079340310.:	714..1787	Glycolysis/Krebs cycle	COG1071
174.1_WP_079340310.:	CUST_5666_PI442190698	HOT208_60m_808M21	174.1_WP_079340310.:	GAGGGCATTCTCTTGCAGATCCAGATGAATTGAGGTCTGAAAAAGAAAAGGAGTTTTGGG	pdhA	Pyruvate dehydrogenase E1 component alpha subunit	WP_079340310.:	714..1787	Glycolysis/Krebs cycle	COG1071
174.1_WP_079340310.:	CUST_5667_PI442190698	HOT208_60m_808M21	174.1_WP_079340310.:	AAAGAGCAATTGAGCGAGCTAGAGCAGGAGAAGGTCCCACACTTTTAGAATGTTTAACTT	pdhA	Pyruvate dehydrogenase E1 component alpha subunit	WP_079340310.:	714..1787	Glycolysis/Krebs cycle	COG1071
179.1_WP_025931480.:	CUST_5741_PI442190698	HOT208_60m_808M21	179.1_WP_025931480.:	ATCCCGGTCAAGCAAATTATTCTGCAGCTAAAGCTGGAGTTATTGGATTCACCAAAACTT	gdh	3-oxoacyl-acyl-carrier-protein reductase	WP_025931480.:	1702..2451	Entner-Dudoroff	COG1028
179.1_WP_025931480.:	CUST_5742_PI442190698	HOT208_60m_808M21	179.1_WP_025931480.:	AGCTAGCAGAGGAATTGGTAAAGAAATTGCTTTAGAACTAAGCCGCTTAGGAGCAGAAGT	gdh	3-oxoacyl-acyl-carrier-protein reductase	WP_025931480.:	1702..2451	Entner-Dudoroff	COG1028
179.1_WP_025931480.:	CUST_5743_PI442190698	HOT208_60m_808M21	179.1_WP_025931480.:	TTCAAGTGATGCAGGAAGTTACATTACAGGGCAAACAATCAGTATAGACGGGGGTATGAG	gdh	3-oxoacyl-acyl-carrier-protein reductase	WP_025931480.:	1702..2451	Entner-Dudoroff	COG1028
179.1_WP_025931480.:	CUST_5744_PI442190698	HOT208_60m_808M21	179.1_WP_025931480.:	TGCTGGTATTACTAGAGATGGACTATTGATGAGAATGAAATCGGAACAATGGGATGATGT	gdh	3-oxoacyl-acyl-carrier-protein reductase	WP_025931480.:	1702..2451	Entner-Dudoroff	COG1028
179.1_WP_025931480.:	CUST_5745_PI442190698	HOT208_60m_808M21	179.1_WP_025931480.:	TGCTTCAAGAGGTATAAACGTAAATGCAATAGCTCCAGGCTTTATAGAAACAGAAATGAC	gdh	3-oxoacyl-acyl-carrier-protein reductase	WP_025931480.:	1702..2451	Entner-Dudoroff	COG1028
179.1_WP_025931480.:	CUST_5746_PI442190698	HOT208_60m_808M21	179.1_WP_025931480.:	ACTCTTCTTCTGATGAGAAAGCTGAAGAAGTTGTAAATTCAATAAAAAATTCGGGAGGTA	gdh	3-oxoacyl-acyl-carrier-protein reductase	WP_025931480.:	1702..2451	Entner-Dudoroff	COG1028
019.1_WP_079338739.:	CUST_5870_PI442190698	HOT208_60m_808M21	019.1_WP_079338739.:	AATCTGTCTTATTGGCATAGGAGCTTCAACCGCTTTTCTTATCCCTTGGTCACTACTTCC	glcH	GPH family sugar transporter	WP_079338739.:	<1..1015	GlcH sugar transporter	COG2211
019.1_WP_079338739.:	CUST_5871_PI442190698	HOT208_60m_808M21	019.1_WP_079338739.:	TACTGAACGTACCAACATATATTGCGAAGTGGATCCCGATACCTTTCCAAATTAGCGCTT	glcH	GPH family sugar transporter	WP_079338739.:	<1..1015	GlcH sugar transporter	COG2211
019.1_WP_079338739.:	CUST_5872_PI442190698	HOT208_60m_808M21	019.1_WP_079338739.:	TGATGCAATAGACGAAGACCCAGAGAAACCAGCAGGATTATATACTGCTTGGATGGTACT	glcH	GPH family sugar transporter	WP_079338739.:	<1..1015	GlcH sugar transporter	COG2211

019.1_WP_079338739.:	CUST_5873_PI442190698	HOT208_60m_808M21019.1_WP_079338739.:	TTCTTCTATGGTGCGCCTTACAATTAATGCAAACAGTAGCGTTAATCTATGTCGAGGATG	glcH	GPH family sugar transporter		WP_079338739.: <1..1015	GlcH sugar transporter	COG2211
019.1_WP_079338739.:	CUST_5874_PI442190698	HOT208_60m_808M21019.1_WP_079338739.:	ACCTTCCTTATCAAAAATTTCAGGAGCTGGAAATAGTTTATTCCTCAATGCCAGCAACAT	glcH	GPH family sugar transporter		WP_079338739.: <1..1015	GlcH sugar transporter	COG2211
019.1_WP_079338739.:	CUST_5875_PI442190698	HOT208_60m_808M21019.1_WP_079338739.:	ACAGGATTTCAGCGTTAAACTATGGAGCAATTATGTGGATTATTTCATGTACAGCAGCTT	glcH	GPH family sugar transporter		WP_079338739.: <1..1015	GlcH sugar transporter	COG2211
273.1_WP_025881193.:	CUST_6868_PI442190698	HOT208_60m_808M21273.1_WP_025881193.:	ACGCAGAAAGAGGTTACTCAGCGGAAACCATTGTGGATACTATATTGAGAAGAATGCCTG	prk	Phosphoribulokinase		WP_025881193.: 20123..21019	Carbon Fixation	COG3954
273.1_WP_025881193.:	CUST_6869_PI442190698	HOT208_60m_808M21273.1_WP_025881193.:	TGGCAGACGCTCTTTCAAAAGGTGAAAATTTCTCACATTTTGGTCCAGAGGCTAATCTTT	prk	Phosphoribulokinase		WP_025881193.: 20123..21019	Carbon Fixation	COG3954
273.1_WP_025881193.:	CUST_6870_PI442190698	HOT208_60m_808M21273.1_WP_025881193.:	TCCCAACTCCTGATGAGAGCTTTGTGATCATACATTTCAGAAAAGGGGCAAGAGAGAAAT	prk	Phosphoribulokinase		WP_025881193.: 20123..21019	Carbon Fixation	COG3954
273.1_WP_025881193.:	CUST_6871_PI442190698	HOT208_60m_808M21273.1_WP_025881193.:	CAGAAGAACATAATACAAGACTTGGGACATCCCTAGAACCTGGGCAATTTACTCCATGGG	prk	Phosphoribulokinase		WP_025881193.: 20123..21019	Carbon Fixation	COG3954
273.1_WP_025881193.:	CUST_6872_PI442190698	HOT208_60m_808M21273.1_WP_025881193.:	GAACAGATGTACTTTTTTATGAAGGTTTGCATGGCGGGGTCGAAGGTGATGGATACAATG	prk	Phosphoribulokinase		WP_025881193.: 20123..21019	Carbon Fixation	COG3954
273.1_WP_025881193.:	CUST_6873_PI442190698	HOT208_60m_808M21273.1_WP_025881193.:	TGTCAAGTCCAACAAGTATCGTTGTAAATGGAGGGAAAATGGGATTCGCAATGGAACTAA	prk	Phosphoribulokinase		WP_025881193.: 20123..21019	Carbon Fixation	COG3954
273.1_WP_079340521.:	CUST_6891_PI442190698	HOT208_60m_808M21273.1_WP_079340521.:	GAAGCTTCAAAAGGTGCTTATGTAGCTGGATCCCAACCTTCATCTGATGAGCAATTGTTT	fda	Fructose-1,6-bisphosphate aldolase class I		WP_079340521.: 23638..24705	Glycolysis/Gluconeogenesis	COG0191
273.1_WP_079340521.:	CUST_6892_PI442190698	HOT208_60m_808M21273.1_WP_079340521.:	TGCTTAAAAGCTTGGAAAGGAAGCGACGTTGAAGGAGGTCAAAAAGCATTAATAGCAAGA	fda	Fructose-1,6-bisphosphate aldolase class I		WP_079340521.: 23638..24705	Glycolysis/Gluconeogenesis	COG0191
273.1_WP_079340521.:	CUST_6893_PI442190698	HOT208_60m_808M21273.1_WP_079340521.:	TGCTCTGGTCTAGATGGGTTAGTTGAAAGAGCAGCAAAATATTATGAACAAGGTGCCAGA	fda	Fructose-1,6-bisphosphate aldolase class I		WP_079340521.: 23638..24705	Glycolysis/Gluconeogenesis	COG0191
273.1_WP_079340521.:	CUST_6894_PI442190698	HOT208_60m_808M21273.1_WP_079340521.:	TTATTCCAGGTATAAAGGTCGATAAAGGCCTAAATCCACTTCCAGGAGGAGGGGATGTAG	fda	Fructose-1,6-bisphosphate aldolase class I		WP_079340521.: 23638..24705	Glycolysis/Gluconeogenesis	COG0191
273.1_WP_079340521.:	CUST_6895_PI442190698	HOT208_60m_808M21273.1_WP_079340521.:	AATGGAGCAGATTGTCCAACAAAGGCTGATCCTATGAAAGTTGCTGAAATGACAATCAGG	fda	Fructose-1,6-bisphosphate aldolase class I		WP_079340521.: 23638..24705	Glycolysis/Gluconeogenesis	COG0191
273.1_WP_079340521.:	CUST_6896_PI442190698	HOT208_60m_808M21273.1_WP_079340521.:	AGCAAGGTATGCAAGATCAGTTCAAGAATCTGGGTTGGTGCCAATTATTGAACCTGAAAT	fda	Fructose-1,6-bisphosphate aldolase class I		WP_079340521.: 23638..24705	Glycolysis/Gluconeogenesis	COG0191
273.1_WP_011376344.:	CUST_6897_PI442190698	HOT208_60m_808M21273.1_WP_011376344.:	ATGCACGGATCTAGTTCAGTGCCTCAGGAATGGTTGGATATCATTAACAAATATGGTGGT	cbbA	Fructose-1,6-bisphosphate aldolase class II		WP_011376344.: 24853..25926	Glycolysis/Gluconeogenesis	COG0191
273.1_WP_011376344.:	CUST_6898_PI442190698	HOT208_60m_808M21273.1_WP_011376344.:	ACTGATCCTGAAGAAGCGGCAGATTTTGTTGCCAAAACAAAAGTTGATGCATTAGCAATT	cbbA	Fructose-1,6-bisphosphate aldolase class II		WP_011376344.: 24853..25926	Glycolysis/Gluconeogenesis	COG0191
273.1_WP_011376344.:	CUST_6899_PI442190698	HOT208_60m_808M21273.1_WP_011376344.:	AGCCTACAGGAGAAGTTCTTGCAATAAGCAGAATTGCTGAGATCCATAAAGCTCTTCCAA	cbbA	Fructose-1,6-bisphosphate aldolase class II		WP_011376344.: 24853..25926	Glycolysis/Gluconeogenesis	COG0191
273.1_WP_011376344.:	CUST_6900_PI442190698	HOT208_60m_808M21273.1_WP_011376344.:	AGCAAGTTTGTCTTGATAGATACAAGCAGTTCTGGTGCGAAGGACAAGCAAGTAAGATCA	cbbA	Fructose-1,6-bisphosphate aldolase class II		WP_011376344.: 24853..25926	Glycolysis/Gluconeogenesis	COG0191
273.1_WP_011376344.:	CUST_6902_PI442190698	HOT208_60m_808M21273.1_WP_011376344.:	ATTCAGTTGGAGTTAGTGTTGAAGGAGAGTTGGGTTGCTTAGGTTCATTGGAAACAGGAA	cbbA	Fructose-1,6-bisphosphate aldolase class II		WP_011376344.: 24853..25926	Glycolysis/Gluconeogenesis	COG0191
278.1_WP_079340625.:	CUST_7209_PI442190698	HOT208_60m_808M21278.1_WP_079340625.:	CAGCCTTAGATGCAGCTAAGGGAACAGATGCAATAATTGTTTTGACCGAATGGGAAGAAT	ugd	UDP-glucose 6-dehydrogenase/nucleotide sugar dehydrogenase		WP_079340625.: 5045..6448	Amino sugar and nucleotide sugar r	COG1004
278.1_WP_079340625.:	CUST_7210_PI442190698	HOT208_60m_808M21278.1_WP_079340625.:	CTATTTCTGCACTCTGCGAGTTCACCGGGGCAAATATTAATGAAGTATCAGGTGCAATTG	ugd	UDP-glucose 6-dehydrogenase/nucleotide sugar dehydrogenase		WP_079340625.: 5045..6448	Amino sugar and nucleotide sugar r	COG1004
278.1_WP_079340625.:	CUST_7211_PI442190698	HOT208_60m_808M21278.1_WP_079340625.:	CTGATACAAGAATTGGTCAAAAATTTTTGAAACCTGGGCCTGGATTTGGGGGAAGTTGTT	ugd	UDP-glucose 6-dehydrogenase/nucleotide sugar dehydrogenase		WP_079340625.: 5045..6448	Amino sugar and nucleotide sugar r	COG1004
278.1_WP_079340625.:	CUST_7212_PI442190698	HOT208_60m_808M21278.1_WP_079340625.:	AGCACAAATTTGTGGAGCTCAGAACTTTCAAAGTTAGTTGCTAATGCTTTTTTGGCTCAA	ugd	UDP-glucose 6-dehydrogenase/nucleotide sugar dehydrogenase		WP_079340625.: 5045..6448	Amino sugar and nucleotide sugar r	COG1004
278.1_WP_079340625.:	CUST_7213_PI442190698	HOT208_60m_808M21278.1_WP_079340625.:	TGAGAAAGCCCGCATGGGTGTTTGATTCAAGAATTTGTCTAGATCAAAAACACTTAAAGA	ugd	UDP-glucose 6-dehydrogenase/nucleotide sugar dehydrogenase		WP_079340625.: 5045..6448	Amino sugar and nucleotide sugar r	COG1004
278.1_WP_079340625.:	CUST_7214_PI442190698	HOT208_60m_808M21278.1_WP_079340625.:	TTAGCTATTTATGGCTTTGCTTTTAAGGCGAATACTAATGATACAAGAGAATCTCCAAGT	ugd	UDP-glucose 6-dehydrogenase/nucleotide sugar dehydrogenase		WP_079340625.: 5045..6448	Amino sugar and nucleotide sugar r	COG1004
283.1_WP_079340654.:	CUST_7332_PI442190698	HOT208_60m_808M21283.1_WP_079340654.:	AATCAGGTTCTACCGCTCGAAATGTAAGTAAATTCAGACCACCAACACCTATCTTGGCAA	pykF	Pyruvate kinase		WP_079340654.: 4759..6549	Glycolysis	COG0469
283.1_WP_079340654.:	CUST_7333_PI442190698	HOT208_60m_808M21283.1_WP_079340654.:	GGAGATGGGGATTCTAAATCAAGGGGATTTAGTAGTCCAAACCGCAGGTACATTAACTGG	pykF	Pyruvate kinase		WP_079340654.: 4759..6549	Glycolysis	COG0469
283.1_WP_079340654.:	CUST_7334_PI442190698	HOT208_60m_808M21283.1_WP_079340654.:	TCCCAAATGCAATTAGTGCAGCAGTAAGTAATATAGCCAGACAACTTGATGCAGGAGCTA	pykF	Pyruvate kinase		WP_079340654.: 4759..6549	Glycolysis	COG0469
283.1_WP_079340654.:	CUST_7335_PI442190698	HOT208_60m_808M21283.1_WP_079340654.:	AGAAAGATTGTATCAAGAGGAATTTCAATAGGGGAAATCGGTGTTACGGGTAAAGCCAGA	pykF	Pyruvate kinase		WP_079340654.: 4759..6549	Glycolysis	COG0469
283.1_WP_079340654.:	CUST_7336_PI442190698	HOT208_60m_808M21283.1_WP_079340654.:	ACTGAGAGAACTGTAGCGAGAAGATTACAACTTGTTTGGGGAGTTACTCCAATAGTAGTT	pykF	Pyruvate kinase		WP_079340654.: 4759..6549	Glycolysis	COG0469
283.1_WP_079340654.:	CUST_7337_PI442190698	HOT208_60m_808M21283.1_WP_079340654.:	TGGCGACCTTATTACACTCCAGCTTAATGAAGGTGTTATATACATGGGACAAATTGAAGA	pykF	Pyruvate kinase		WP_079340654.: 4759..6549	Glycolysis	COG0469
283.1_WP_011818380.:	CUST_7379_PI442190698	HOT208_60m_808M21283.1_WP_011818380.:	AGGTACAACAACTAAGACAAAGGCCTCATCCACATGAATTCTTCATGTATTACGACGCGT	glnA	Glutamine synthetase/ glutamate--ammonialigase		WP_011818380.: 13769..15190	Nitrogen Metabolism	COG0174
283.1_WP_011818380.:	CUST_7380_PI442190698	HOT208_60m_808M21283.1_WP_011818380.:	CAGGTGACGCACTTGCAAATCCTTACTTAGCATTCTCTGTAATGATGCTTGCTGGTATTG	glnA	Glutamine synthetase/ glutamate--ammonialigase		WP_011818380.: 13769..15190	Nitrogen Metabolism	COG0174
283.1_WP_011818380.:	CUST_7381_PI442190698	HOT208_60m_808M21283.1_WP_011818380.:	GTTTATTCTGAGGGTAATAGATCAGCTGCGGTAAGAATACCTTTAACAGGACCAAGCCCC	glnA	Glutamine synthetase/ glutamate--ammonialigase		WP_011818380.: 13769..15190	Nitrogen Metabolism	COG0174
283.1_WP_011818380.:	CUST_7382_PI442190698	HOT208_60m_808M21283.1_WP_011818380.:	GCATTTACTAACCCAACCACTAATAGTTACAAACGATTGGTTCCAGGATTCGAAGCACCT	glnA	Glutamine synthetase/ glutamate--ammonialigase		WP_011818380.: 13769..15190	Nitrogen Metabolism	COG0174
283.1_WP_011818380.:	CUST_7383_PI442190698	HOT208_60m_808M21283.1_WP_011818380.:	AGTGGACAGCCACTATTCTTTGGTGAAGGATCATATGCAAATTTATCTCAAACAGCAAGA	glnA	Glutamine synthetase/ glutamate--ammonialigase		WP_011818380.: 13769..15190	Nitrogen Metabolism	COG0174
283.1_WP_011818380.:	CUST_7384_PI442190698	HOT208_60m_808M21283.1_WP_011818380.:	CATTTATGCCCAAACCTGTTTTTAACGACAACGGAACAGGAATGCATGTTCACCAAAGTT	glnA	Glutamine synthetase/ glutamate--ammonialigase		WP_011818380.: 13769..15190	Nitrogen Metabolism	COG0174
287.1_WP_079340747.:	CUST_7780_PI442190698	HOT208_60m_808M21287.1_WP_079340747.:	AATATCCGATAATTTGCGCAGAATTATGTGGTCCATATCATGGAGGAATGAGAGCCTCCA	ctaC/coxB	Cytochrome c oxidase subunit II		WP_079340747.: 3427..4230	Respiration	COG1622
287.1_WP_079340747.:	CUST_7781_PI442190698	HOT208_60m_808M21287.1_WP_079340747.:	TCCCAAGGGCAATTTCATATCCGGAGAACTACACGTTCCTGTTGATCAAAAAGTATCAAT	ctaC/coxB	Cytochrome c oxidase subunit II		WP_079340747.: 3427..4230	Respiration	COG1622
287.1_WP_079340747.:	CUST_7782_PI442190698	HOT208_60m_808M21287.1_WP_079340747.:	CATGCTTTTTGGGTACCAGAGTTCAGAATTAAACAGGATATTATTCCTGGACAGCCGACT	ctaC/coxB	Cytochrome c oxidase subunit II		WP_079340747.: 3427..4230	Respiration	COG1622
287.1_WP_079340747.:	CUST_7783_PI442190698	HOT208_60m_808M21287.1_WP_079340747.:	ACAGCTACAACATCTACGATCGAATGGGAGGGATGAAAGAACTAAATCATAACCACAAAA	ctaC/coxB	Cytochrome c oxidase subunit II		WP_079340747.: 3427..4230	Respiration	COG1622
287.1_WP_079340747.:	CUST_7784_PI442190698	HOT208_60m_808M21287.1_WP_079340747.:	GGCGATGGCATAGCTTTAGAGGGAAATTTAAGCTTAGAAATTGTATGGACAATTATCCCT	ctaC/coxB	Cytochrome c oxidase subunit II		WP_079340747.: 3427..4230	Respiration	COG1622
287.1_WP_079340747.:	CUST_7785_PI442190698	HOT208_60m_808M21287.1_WP_079340747.:	TCTTTTTGGATTGGTTTTAATGTAAATTTGCTCCCAGCTGAAGCAAGTATAAATGCACCA	ctaC/coxB	Cytochrome c oxidase subunit II		WP_079340747.: 3427..4230	Respiration	COG1622
287.1_WP_079340748.:	CUST_7786_PI442190698	HOT208_60m_808M21287.1_WP_079340748.:	TGTTTTTGGAGGTATTACAGGAGTTGCTTTGGCACAGGTACCTTTCGATATTCACGTACA	cyoB/coxA	Cytochrome c oxidase subunit I		WP_079340748.: 4227..5852	Respiration	COG0843
287.1_WP_079340748.:	CUST_7787_PI442190698	HOT208_60m_808M21287.1_WP_079340748.:	CTCCTCAACATTGGCTTGGTTTAAATGGAATGCCCAGAAGAGTTGCAGAATATGATCCTC	cyoB/coxA	Cytochrome c oxidase subunit I		WP_079340748.: 4227..5852	Respiration	COG0843
287.1_WP_079340748.:	CUST_7788_PI442190698	HOT208_60m_808M21287.1_WP_079340748.:	GAGATAACCCTTGGAATGCTCTTACACCTGAATGGTTAACATCTTCTCCACCTCCAGTTG	cyoB/coxA	Cytochrome c oxidase subunit I		WP_079340748.: 4227..5852	Respiration	COG0843
287.1_WP_079340748.:	CUST_7789_PI442190698	HOT208_60m_808M21287.1_WP_079340748.:	TATCGCCACAGCATTTATTGCTGTTCCGACGGGTATAAAATTTTTCAATTGGGTTGCAAC	cyoB/coxA	Cytochrome c oxidase subunit I		WP_079340748.: 4227..5852	Respiration	COG0843
287.1_WP_079340748.:	CUST_7790_PI442190698	HOT208_60m_808M21287.1_WP_079340748.:	TGGGGATAGTAGTTTTAGGTTTAGTTGTTTGGGCTCATCACATGTTTACGAGTGGAACGC	cyoB/coxA	Cytochrome c oxidase subunit I		WP_079340748.: 4227..5852	Respiration	COG0843
287.1_WP_079340748.:	CUST_7791_PI442190698	HOT208_60m_808M21287.1_WP_079340748.:	TGGGAAGGAGAAGCTCCATTAGTTGAAGAGCCCTATGGTTATGGTAAAGAAATTTCTGAA	cyoB/coxA	Cytochrome c oxidase subunit I		WP_079340748.: 4227..5852	Respiration	COG0843
287.1_WP_079340749.:	CUST_7792_PI442190698	HOT208_60m_808M21287.1_WP_079340749.:	TGGCAATCGAGAATCAATGGCGGAAGATTAACCAGTCAAAATATGTTTCCATTGGAAGCT	cyoC	heme-copper oxidase subunit III		WP_079340749.: 5859..6461	Respiration	COG1845
287.1_WP_079340749.:	CUST_7793_PI442190698	HOT208_60m_808M21287.1_WP_079340749.:	TGTTTGGTCTTATAACCTTCCTAATTGCGGACGGAATGACTTTTGCTGGATTCTTTGCTG	cyoC	heme-copper oxidase subunit III		WP_079340749.: 5859..6461	Respiration	COG1845
287.1_WP_079340749.:	CUST_7794_PI442190698	HOT208_60m_808M21287.1_WP_079340749.:	AGAACTCCCAATACCTACACTCAACACAATTTTGTTACTTGTTAGTAGTGCGACTTTCCA	cyoC	heme-copper oxidase subunit III		WP_079340749.: 5859..6461	Respiration	COG1845
287.1_WP_079340749.:	CUST_7795_PI442190698	HOT208_60m_808M21287.1_WP_079340749.:	TGCAAGTACTTTTTATGCTCTTACTGGTTTTCATGGATTACATGTCACTTTAGGCACTTT	cyoC	heme-copper oxidase subunit III		WP_079340749.: 5859..6461	Respiration	COG1845
287.1_WP_079340749.:	CUST_7796_PI442190698	HOT208_60m_808M21287.1_WP_079340749.:	AAAGCAGGTAAAGCACTTTTAAAAGATAAAAACTCTGATTCCCAAAAATGGCTATTCATT	cyoC	heme-copper oxidase subunit III		WP_079340749.: 5859..6461	Respiration	COG1845
287.1_WP_079340749.:	CUST_7797_PI442190698	HOT208_60m_808M21287.1_WP_079340749.:	AGCTCAAAAGAAATTCAAAAAAATAATTCTGAAGTTAATGAAACACATGAAGACTTCAGA	cyoC	heme-copper oxidase subunit III		WP_079340749.: 5859..6461	Respiration	COG1845
290.1_1548	CUST_7939_PI442190698	HOT208_60m_808M21290.1_1548	CGGATTTAGAAGGATCTTATGCTTTAGCAGTTTTATGGTCTGGTGCGCCAACTTCTTTGG	glmS	Glutamine--fructose-6-phosphatetransaminase(isomerizing)		<1..592	Amino Acid metabolism	COG0449
290.1_1548	CUST_7940_PI442190698	HOT208_60m_808M21290.1_1548	GCGCCCTTAATTATTGGTTTGGGTGAAGGAGAATTTATTTGTGCTAGTGATGTTCCAGCT	glmS	Glutamine--fructose-6-phosphatetransaminase(isomerizing)		<1..592	Amino Acid metabolism	COG0449
290.1_1548	CUST_7941_PI442190698	HOT208_60m_808M21290.1_1548	TGGAGGATGAAGAAATAGCTTTGCTGACTCCACTTGGAATTGAAATATATGACTCAAGCA	glmS	Glutamine--fructose-6-phosphatetransaminase(isomerizing)		<1..592	Amino Acid metabolism	COG0449
290.1_1548	CUST_7942_PI442190698	HOT208_60m_808M21290.1_1548	TTTCAAACACTACATGTTGAAAGAGATATATGATCAGCCACAGACTGCAAAAAACTGGTT	glmS	Glutamine--fructose-6-phosphatetransaminase(isomerizing)		<1..592	Amino Acid metabolism	COG0449
290.1_1548	CUST_7943_PI442190698	HOT208_60m_808M21290.1_1548	AGAGAAAGAGGGTATTATTTTTAATTCTGATACAGATACCGAGGTAATTCCTCATCTAGT	glmS	Glutamine--fructose-6-phosphatetransaminase(isomerizing)		<1..592	Amino Acid metabolism	COG0449
290.1_1548	CUST_7944_PI442190698	HOT208_60m_808M21290.1_1548	ATGAAAGACAGTATAGAAATCCAGTTCCTTTAAAAGTCTCAGAGCAAATAATGGATAAGA	glmS	Glutamine--fructose-6-phosphatetransaminase(isomerizing)		<1..592	Amino Acid metabolism	COG0449
303.1_WP_079340789.:	CUST_8110_PI442190698	HOT208_60m_808M21303.1_WP_079340789.:	ATTAGCTCTGGAGTCAAGGACCTTGATGACATGTGTGGTGGAGGTTATTTTCAAGATTCC	kaiC	circadian clock protein KaiC		WP_079340789.: 760..2298	Circadian Rhythm	COG0467
303.1_WP_079340789.:	CUST_8111_PI442190698	HOT208_60m_808M21303.1_WP_079340789.:	CCAAAAAGAATGGCAATTGATTCTCTCTCTGCCTTAGCCAGAGGTGTAAGTTTGAATGCA	kaiC	circadian clock protein KaiC		WP_079340789.: 760..2298	Circadian Rhythm	COG0467
303.1_WP_079340789.:	CUST_8112_PI442190698	HOT208_60m_808M21303.1_WP_079340789.:	GCCACTGGAGCTACTGGTACAGGTAAAACCATGCTTGTATCAAAATTTGTTGAAGATGCT	kaiC	circadian clock protein KaiC		WP_079340789.: 760..2298	Circadian Rhythm	COG0467
303.1_WP_079340789.:	CUST_8113_PI442190698	HOT208_60m_808M21303.1_WP_079340789.:	AGGTGAGATGGCAAGAGCTTTAAATGTATTTAAAATGCGGGGATCATGGCATGATAAACG	kaiC	circadian clock protein KaiC		WP_079340789.: 760..2298	Circadian Rhythm	COG0467
303.1_WP_079340789.:	CUST_8114_PI442190698	HOT208_60m_808M21303.1_WP_079340789.:	ACTTTTTGCATATGAAGAATCTAGGGCTCAATTGCTTAGGAATGCTACTAGCTGGGGAAT	kaiC	circadian clock protein KaiC		WP_079340789.: 760..2298	Circadian Rhythm	COG0467
303.1_WP_079340789.:	CUST_8115_PI442190698	HOT208_60m_808M21303.1_WP_079340789.:	TGCTGTCACTGGTTATACAAAACAAGAGGAGATAGCAGGCTTTTTTACTAATACTGCAGA	kaiC	circadian clock protein KaiC		WP_079340789.: 760..2298	Circadian Rhythm	COG0467
303.1_WP_079340790.:	CUST_8116_PI442190698	HOT208_60m_808M21303.1_WP_079340790.:	CGCCACCAGTTAGAAAAATAATAGGTGATCTTTCAGATAGAGAAAAAGTT	kaiB	Circadian clock protein KaiB		WP_079340790.: 2364..2681	Circadian Rhythm	COG4251
303.1_WP_079340790.:	CUST_8117_PI442190698	HOT208_60m_808M21303.1_WP_079340790.:	TGCAGAAGAAGATAAAATTTTAGCCACTCCTACGTTAGCTAAAATTTTACCGCCACCAGT	kaiB	Circadian clock protein KaiB		WP_079340790.: 2364..2681	Circadian Rhythm	COG4251
303.1_WP_079340790.:	CUST_8118_PI442190698	HOT208_60m_808M21303.1_WP_079340790.:	CCTAATTCGATGAGAGCTTTAAATACCTTAAGAGAAATTTTAGATAATGA	kaiB	Circadian clock protein KaiB		WP_079340790.: 2364..2681	Circadian Rhythm	COG4251
303.1_WP_079340790.:	CUST_8119_PI442190698	HOT208_60m_808M21303.1_WP_079340790.:	TGGCAGCAAGGAAAACATATATTTTAAAACTCTATGTTGCTGGAAATACTCCTAATTCGA	kaiB	Circadian clock protein KaiB		WP_079340790.: 2364..2681	Circadian Rhythm	COG4251
353.1_WP_079340851.:	CUST_8827_PI442190698	HOT208_60m_808M21353.1_WP_079340851.:	ATTGAGGAGGAATTAGGAAATCAAGCAAGATTCGCTGGAGCTTTAGGTTTAGGTCCCAAA	eno	Enolase		WP_079340851.: 42787..44079	Glycolysis/Gluconeogenesis	COG0148
353.1_WP_079340851.:	CUST_8828_PI442190698	HOT208_60m_808M21353.1_WP_079340851.:	GTGAGACTGAAGATACAACAATTGCAGATTTATCTGTCGCCACAAGATCGGGTCAGATCA	eno	Enolase		WP_079340851.: 42787..44079	Glycolysis/Gluconeogenesis	COG0148
353.1_WP_079340851.:	CUST_8829_PI442190698	HOT208_60m_808M21353.1_WP_079340851.:	AGAGGACGGTTTAGCTGAGGATGATTGGGAGGGTTGGTCAGAATTAAACAAAGAATTAGG	eno	Enolase		WP_079340851.: 42787..44079	Glycolysis/Gluconeogenesis	COG0148
353.1_WP_079340851.:	CUST_8830_PI442190698	HOT208_60m_808M21353.1_WP_079340851.:	AAGCAGGGGACTTATTATTAGAAGCAATTCAAAAAGCTGGATTTAAGCCTGGTGAGCAGG	eno	Enolase		WP_079340851.: 42787..44079	Glycolysis/Gluconeogenesis	COG0148
353.1_WP_079340851.:	CUST_8831_PI442190698	HOT208_60m_808M21353.1_WP_079340851.:	ATCATTACTTGATCAAAAAGGTTTATCTACTGCTGTAGGCGATGAGGGTGGATTTGCCCC	eno	Enolase		WP_079340851.: 42787..44079	Glycolysis/Gluconeogenesis	COG0148
353.1_WP_079340851.:	CUST_8832_PI442190698	HOT208_60m_808M21353.1_WP_079340851.:	TGGTGCTCATGCACCAAATAGTCTTGATTTTCAGGAATTTATGCTTGTTCCACATGGAGT	eno	Enolase		WP_079340851.: 42787..44079	Glycolysis/Gluconeogenesis	COG0148
366.1_1759	CUST_9032_PI442190698	HOT208_60m_808M21366.1_1759	TCCCCTTACTTTTGGGCAAGAAATATCAGGATGGTCAGAGCAACTTAAAGATGCTGAGAA	fumC	class II fumarate hydratase		<1..622	Krebs cycle and related genes	COG0114
366.1_1759	CUST_9033_PI442190698	HOT208_60m_808M21366.1_1759	TGCAGCATTAAAAACAAATTCAGAGCTTGGAAGTCATCAACCAATTCATCCTAATGATGA	fumC	class II fumarate hydratase		<1..622	Krebs cycle and related genes	COG0114
366.1_1759	CUST_9034_PI442190698	HOT208_60m_808M21366.1_1759	AAAAAGTGAAGAATGGAAAGACCTTATAAAGATAGGAAGAACCCATTTTCAAGATGCAGT	fumC	class II fumarate hydratase		<1..622	Krebs cycle and related genes	COG0114
366.1_1759	CUST_9035_PI442190698	HOT208_60m_808M21366.1_1759	AATCTCAGTCAACTAATGATACTTTTCCTGCTGCTATCCAAATATCAGTTGTTAATGAAA	fumC	class II fumarate hydratase		<1..622	Krebs cycle and related genes	COG0114
366.1_1759	CUST_9036_PI442190698	HOT208_60m_808M21366.1_1759	AATCAATTTCCGATGATACTAATCTAATGTTCTATAAATCAAAAAAATAATTTTTCTATCA	fumC	class II fumarate hydratase		<1..622	Krebs cycle and related genes	COG0114
366.1_1759	CUST_9037_PI442190698	HOT208_60m_808M21366.1_1759	TGCATTATTTAAAATTTCAAATGATATAAAAATTCTATCTTCTGGTCCAAGATCA	fumC	class II fumarate hydratase		<1..622	Krebs cycle and related genes	COG0114
378.1_1775	CUST_9114_PI442190698	HOT208_60m_808M21378.1_1775	GGCAGGTATTTTTAACATCTTGGATTTTATTAGGAGCGTTATTAGTTTTTATTTCT	atpI	ATP synthase subunit A		335..479	Energy Metabolism	COG0356
378.1_1775	CUST_9115_PI442190698	HOT208_60m_808M21378.1_1775	AGCATTAGAAGTTGGTCAACATCTTTATTGGCAAATTGGAAATATCAGACTACATGGGCA	atpI	ATP synthase subunit A		335..479	Energy Metabolism	COG0356
073.1_1827	CUST_9374_PI442190698	HOT208_60m_808M21073.1_1827	AGGCATGTACAGAAATACTTGATGGGCTTCATGATTCACAGTTTCCCTTAAAGGTTTGGC	fumC	class II fumarate hydratase		<1..315	Krebs cycle and related genes	COG0114
073.1_1827	CUST_9375_PI442190698	HOT208_60m_808M21073.1_1827	TGCACTCGCCCTCATAAAAAAAGCTGCTTCAATCGCTAATTTCAATTTAGGATTAATAGA	fumC	class II fumarate hydratase		<1..315	Krebs cycle and related genes	COG0114
073.1_1827	CUST_9376_PI442190698	HOT208_60m_808M21073.1_1827	GGAACAATAGAGGTTCCCATTGAAGCTTTGTGGGGTGCTCAAACACAAAGATCGATAAAA	fumC	class II fumarate hydratase		<1..315	Krebs cycle and related genes	COG0114
001.1_WP_079293414.:	CUST_1_PI442190703	HOT208_60m_805A16 001.1_WP_079293414.:	ACTTTTATCAGCCGCATGGTATTTAACTGGGGATGGAATTGGAAAGAGAGATATGGTCGT	pgi	Glucose-6-phosphate isomerase		WP_079293414.: 85..1177	Glycolysis/Gluconeogenesis	COG0166
001.1_WP_079293414.:	CUST_2_PI442190703	HOT208_60m_805A16 001.1_WP_079293414.:	AGAATATCTTTCTGGCTTTTTGCAAGGAACCAGATCAGCACTCTCTAGTGAAAGCAGACA	pgi	Glucose-6-phosphate isomerase		WP_079293414.: 85..1177	Glycolysis/Gluconeogenesis	COG0166
001.1_WP_079293414.:	CUST_3_PI442190703	HOT208_60m_805A16 001.1_WP_079293414.:	TTTCACTAGGTGCCTTAATCGCTTTATTTGAGAGGGCTGTATCCTTCTACGCTGAATTGG	pgi	Glucose-6-phosphate isomerase		WP_079293414.: 85..1177	Glycolysis/Gluconeogenesis	COG0166
001.1_WP_079293414.:	CUST_4_PI442190703	HOT208_60m_805A16 001.1_WP_079293414.:	ATGCATATGATCAACCTGGAGTTGAAGCTGGAAAGAAAGCAGCCGCAAATATTATTGAGT	pgi	Glucose-6-phosphate isomerase		WP_079293414.: 85..1177	Glycolysis/Gluconeogenesis	COG0166
001.1_WP_079293414.:	CUST_5_PI442190703	HOT208_60m_805A16 001.1_WP_079293414.:	AGGTGCATCATTAATGGATGATGCCACACGTATAAGTGATTTTAAAAATAATCCAGCAGC	pgi	Glucose-6-phosphate isomerase		WP_079293414.: 85..1177	Glycolysis/Gluconeogenesis	COG0166
001.1_WP_079293414.:	CUST_6_PI442190703	HOT208_60m_805A16 001.1_WP_079293414.:	TTCCGTATTTGGTAATAAAGGATCTACAGATCAACATGCTTATGTTCAGCAACTGAGAGA	pgi	Glucose-6-phosphate isomerase		WP_079293414.: 85..1177	Glycolysis/Gluconeogenesis	COG0166
104.1_11	CUST_53_PI442190703	HOT208_60m_805A16 104.1_11	CGAGGGAGAGAAGGGAGGCATACTATTCCCTGGAGGAAGATATAAGGCTCTTAAAAACAA	rbsK	Sugar kinase, ribokinase family		<10..723	Pentose Phosphate	COG0524
104.1_11	CUST_54_PI442190703	HOT208_60m_805A16 104.1_11	GTCATTTCTCATTCTGAAAAGTCCCTTGAGTATCCAGCTGGTGGTGGATCTATTATCGCG	rbsK	Sugar kinase, ribokinase family		<10..723	Pentose Phosphate	COG0524
104.1_11	CUST_55_PI442190703	HOT208_60m_805A16 104.1_11	CTCTCAAATATGGGAATTAAGTTGCATGTAGCTTGGCGTGATAAACCAACTAGAAGAGGA	rbsK	Sugar kinase, ribokinase family		<10..723	Pentose Phosphate	COG0524
104.1_11	CUST_56_PI442190703	HOT208_60m_805A16 104.1_11	AATTGACTCTCAAGGTGAAAGAGCAATAACAGTTATTGGAGAAAGGTTAGCTCCAACTCA	rbsK	Sugar kinase, ribokinase family		<10..723	Pentose Phosphate	COG0524
104.1_11	CUST_57_PI442190703	HOT208_60m_805A16 104.1_11	TCAGAGATTTTTAAAATGGCTAGATCAGCTTCAATACTGTGTACAACACCAAGGGTAGGA	rbsK	Sugar kinase, ribokinase family		<10..723	Pentose Phosphate	COG0524
104.1_11	CUST_58_PI442190703	HOT208_60m_805A16 104.1_11	GCCGTTGTTGGTCATGTTGAGTGGATAAATTTTTTAAAGGTTGATCAATTACCAAAACCC	rbsK	Sugar kinase, ribokinase family		<10..723	Pentose Phosphate	COG0524
124.1_WP_079293971.:	CUST_1219_PI442190703	HOT208_60m_805A16 124.1_WP_079293971.:	GGGGAAAAGGTAAAAGTTTTAGCTAGTGAAGGTGGTCATGTTGAGTACTCCCCAAAGTCA	glk	Glucokinase		WP_079293971.: 39094..40128	Glycolysis	COG0837
124.1_WP_079293971.:	CUST_1220_PI442190703	HOT208_60m_805A16 124.1_WP_079293971.:	CATGAATTGTGCTTTGGCGGGTTATGGATTTCTGGAGGAACAGCATCAAAACATTTCAAA	glk	Glucokinase		WP_079293971.: 39094..40128	Glycolysis	COG0837
124.1_WP_079293971.:	CUST_1221_PI442190703	HOT208_60m_805A16 124.1_WP_079293971.:	ACTATCCAAAATGGATTCCATGCTGAAGGAGCTAATAATGATTTGCATGCCGTTGTTGGA	glk	Glucokinase		WP_079293971.: 39094..40128	Glycolysis	COG0837
124.1_WP_079293971.:	CUST_1222_PI442190703	HOT208_60m_805A16 124.1_WP_079293971.:	AATTATTAGTGGCACTGGTTTATCAAGAATTGCCGAATGGAGACTAAGCAAACCTGACGC	glk	Glucokinase		WP_079293971.: 39094..40128	Glycolysis	COG0837
124.1_WP_079293971.:	CUST_1223_PI442190703	HOT208_60m_805A16 124.1_WP_079293971.:	AAAGAAGGGGATCAGCTAATGATTGAAGTTGAGAGGATTTGGTTAAGCGCTTATGCCTCT	glk	Glucokinase		WP_079293971.: 39094..40128	Glycolysis	COG0837
124.1_WP_079293971.:	CUST_1224_PI442190703	HOT208_60m_805A16 124.1_WP_079293971.:	TCCCTATGAAAGTAATTTTGGATGAAGAGTTTGGACTTTTTAGTGCAGCCTGTAGGGCAA	glk	Glucokinase		WP_079293971.: 39094..40128	Glycolysis	COG0837
126.1_WP_079294037.:	CUST_1593_PI442190703	HOT208_60m_805A16 126.1_WP_079294037.:	TTGAGTTACCATATTGCAGCTCATAGGGGACTTGACGTGGATCAACCAAGAAATTTAGCT	glmS	Glutamine--fructose-6-phosphatetransaminase(isomerizing)		WP_079294037.: 3905..5800	Amino Acid metabolism	COG0449
126.1_WP_079294037.:	CUST_1594_PI442190703	HOT208_60m_805A16 126.1_WP_079294037.:	GTTACATTCATGCAGCTGGATATCCTGCTGGTGAAATGAAGCATGGTCCAATAGCTTTAT	glmS	Glutamine--fructose-6-phosphatetransaminase(isomerizing)		WP_079294037.: 3905..5800	Amino Acid metabolism	COG0449
126.1_WP_079294037.:	CUST_1595_PI442190703	HOT208_60m_805A16 126.1_WP_079294037.:	GGAAGGCAGGTCTCAAATATAATTGATATCTGTGCAGGAATAGAAGTTGGAGTTGCAGCA	glmS	Glutamine--fructose-6-phosphatetransaminase(isomerizing)		WP_079294037.: 3905..5800	Amino Acid metabolism	COG0449
126.1_WP_079294037.:	CUST_1596_PI442190703	HOT208_60m_805A16 126.1_WP_079294037.:	TGCTCAAGAAGCAAAAGCTAGAGATGCATATTTGATTGGTATTGCTCCTGAATGTAAGGG	glmS	Glutamine--fructose-6-phosphatetransaminase(isomerizing)		WP_079294037.: 3905..5800	Amino Acid metabolism	COG0449
126.1_WP_079294037.:	CUST_1597_PI442190703	HOT208_60m_805A16 126.1_WP_079294037.:	TGTTATTTTTTTAGGAAGAGGGATAAATTACCCCATTGCCCTTGAAGGTGCTTTAAAACT	glmS	Glutamine--fructose-6-phosphatetransaminase(isomerizing)		WP_079294037.: 3905..5800	Amino Acid metabolism	COG0449
126.1_WP_079294037.:	CUST_1598_PI442190703	HOT208_60m_805A16 126.1_WP_079294037.:	GGCAATCAAAGTCCTGATGAAATAGGTAAATTAATAAACGAACTTAAAAAACTGCCGCCA	glmS	Glutamine--fructose-6-phosphatetransaminase(isomerizing)		WP_079294037.: 3905..5800	Amino Acid metabolism	COG0449
129.1_372	CUST_1964_PI442190703	HOT208_60m_805A16 129.1_372	AGTTCGATAAATATACTCAACTATAAAAATATTGATTTCATTGCTGCTGCAGGACTATCA	eda	2-dehydro-3-deoxyphosphogluconate aldolase		3496..3974	Entner-Dudoroff	COG0800
129.1_372	CUST_1965_PI442190703	HOT208_60m_805A16 129.1_372	AATTTTGGCTCTGCCTACATATTTAATAAGCAATCAATAGAAGATTCTTCAAAAATTGGA	eda	2-dehydro-3-deoxyphosphogluconate aldolase		3496..3974	Entner-Dudoroff	COG0800
129.1_372	CUST_1966_PI442190703	HOT208_60m_805A16 129.1_372	AGGGTATAAAGCAGTTGTAATTGGAGATAAAGCTATCAAAAATAAAAAATTTGATCCAAA	eda	2-dehydro-3-deoxyphosphogluconate aldolase		3496..3974	Entner-Dudoroff	COG0800
129.1_372	CUST_1967_PI442190703	HOT208_60m_805A16 129.1_372	AGACTATTTAAATAAGTTGGTCTAACAACGAAAATTAGTTCGATTTGTATCCGATATCAA	eda	2-dehydro-3-deoxyphosphogluconate aldolase		3496..3974	Entner-Dudoroff	COG0800
129.1_372	CUST_1968_PI442190703	HOT208_60m_805A16 129.1_372	AGAAGATTCTTTTTCTGAGTTCCTGAAATTGAGTCTTTTTTTATTTATAAAACCTGAAGA	eda	2-dehydro-3-deoxyphosphogluconate aldolase		3496..3974	Entner-Dudoroff	COG0800
129.1_372	CUST_1969_PI442190703	HOT208_60m_805A16 129.1_372	TCAAATACCTCTATAAGAAATTCATTTTTTGGAGAATTAGAAAACTTAGTAAAATTAGGA	eda	2-dehydro-3-deoxyphosphogluconate aldolase		3496..3974	Entner-Dudoroff	COG0800
134.1_WP_079294144.:	CUST_2248_PI442190703	HOT208_60m_805A16 134.1_WP_079294144.:	GGTCAAAAAGCATTAATAGCGAGAGCCCAAGCAAACTCTGAAGCTTCAAAAGGTTCCTAT	fda	Fructose-1,6-bisphosphate aldolase class I		WP_079294144.: 57..1055	Glycolysis/Gluconeogenesis	COG0191
134.1_WP_079294144.:	CUST_2249_PI442190703	HOT208_60m_805A16 134.1_WP_079294144.:	CCAGGTATAAAGGTCGATAAAGGCCTAAATCCACTTCCAGGAGGAGGAGATGTAGAAACT	fda	Fructose-1,6-bisphosphate aldolase class I		WP_079294144.: 57..1055	Glycolysis/Gluconeogenesis	COG0191
134.1_WP_079294144.:	CUST_2250_PI442190703	HOT208_60m_805A16 134.1_WP_079294144.:	GGGGCAGATTGTCCAACAAAAGCTGATCCTATGAAAGTTGCTGAAATGACAATCAGAACT	fda	Fructose-1,6-bisphosphate aldolase class I		WP_079294144.: 57..1055	Glycolysis/Gluconeogenesis	COG0191
134.1_WP_079294144.:	CUST_2251_PI442190703	HOT208_60m_805A16 134.1_WP_079294144.:	GTTTCATTCTCCTATGGAAGAGCATTACAGCACTCATGCTTAAAAGCCTGGAAAGGAAGC	fda	Fructose-1,6-bisphosphate aldolase class I		WP_079294144.: 57..1055	Glycolysis/Gluconeogenesis	COG0191
134.1_WP_079294144.:	CUST_2252_PI442190703	HOT208_60m_805A16 134.1_WP_079294144.:	TTAGTTGAAAGAGCCGCAAAATATTATGAACAAGGTGCCAGATTTGCAAAGTGGAGAGCA	fda	Fructose-1,6-bisphosphate aldolase class I		WP_079294144.: 57..1055	Glycolysis/Gluconeogenesis	COG0191
134.1_WP_079294144.:	CUST_2253_PI442190703	HOT208_60m_805A16 134.1_WP_079294144.:	GCATCTGTTCCTGGAATAGTTTTCTTATCAGGAGGGTTAAGCGAAGAAGCTGCTTCTGTT	fda	Fructose-1,6-bisphosphate aldolase class I		WP_079294144.: 57..1055	Glycolysis/Gluconeogenesis	COG0191
134.1_WP_011376344.:	CUST_2254_PI442190703	HOT208_60m_805A16 134.1_WP_011376344.:	TGGTGTGAAGGTCAAGCAAGTAAGATCAAGCAAAACAGCACCAATTACTTTGCTGATCTT	cbbA	Fructose-1,6-bisphosphate aldolase class II		WP_011376344.: 1203..2276	Glycolysis/Gluconeogenesis	COG0191
134.1_WP_011376344.:	CUST_2255_PI442190703	HOT208_60m_805A16 134.1_WP_011376344.:	ATGCACGGATCTAGTTCAGTGCCTCAGGAATGGTTGGATATCATTAACAAATATGGTGGT	cbbA	Fructose-1,6-bisphosphate aldolase class II		WP_011376344.: 1203..2276	Glycolysis/Gluconeogenesis	COG0191
134.1_WP_011376344.:	CUST_2256_PI442190703	HOT208_60m_805A16 134.1_WP_011376344.:	CAGCATTTGCTGACAAGGCAAACTTTGACCCAAGACATTTCAACAAACCTGCTAGAAAAT	cbbA	Fructose-1,6-bisphosphate aldolase class II		WP_011376344.: 1203..2276	Glycolysis/Gluconeogenesis	COG0191
134.1_WP_011376344.:	CUST_2257_PI442190703	HOT208_60m_805A16 134.1_WP_011376344.:	TACAAGGAAGCCTACGGGAGAAGTTCTTGCAATAAGCAGAATAGCTGAGATCCATAAAGC	cbbA	Fructose-1,6-bisphosphate aldolase class II		WP_011376344.: 1203..2276	Glycolysis/Gluconeogenesis	COG0191
139.1_WP_079294200.:	CUST_2689_PI442190703	HOT208_60m_805A16 139.1_WP_079294200.:	GGGCATTCTTTGAGTCAAACCTTGCATTGACTGAGCAACCAAAACCTCCATTTAGTTTTT	glgC	Glucose-1-phosphate adenylyltransferase		WP_079294200.: 3119..4414	Glycogen Metabolism	COG0448
139.1_WP_079294200.:	CUST_2690_PI442190703	HOT208_60m_805A16 139.1_WP_079294200.:	TGGATTGAGTAAGGAGTCCGCTGTTGAAAAACCATTTCTAGCCTCTATGGGAATTTACGT	glgC	Glucose-1-phosphate adenylyltransferase		WP_079294200.: 3119..4414	Glycogen Metabolism	COG0448
139.1_WP_079294200.:	CUST_2691_PI442190703	HOT208_60m_805A16 139.1_WP_079294200.:	TTAGGTGTAAGAAGCAGGATTGAAAGTGACTCGGTTCTTGAGGATACCCTTGTTATGGGA	glgC	Glucose-1-phosphate adenylyltransferase		WP_079294200.: 3119..4414	Glycogen Metabolism	COG0448
139.1_WP_079294200.:	CUST_2692_PI442190703	HOT208_60m_805A16 139.1_WP_079294200.:	CGGAACGCCTCTTGGAGTAGGTGAAGGGACTACTGTAAAAAGAGCAATTCTTGATAAGAA	glgC	Glucose-1-phosphate adenylyltransferase		WP_079294200.: 3119..4414	Glycogen Metabolism	COG0448
139.1_WP_079294200.:	CUST_2693_PI442190703	HOT208_60m_805A16 139.1_WP_079294200.:	AGAATTTAGTGAAAAGCCTACTGGAGAGAAGTTGAAGGCTATGGCAGTAGATACTTCAAA	glgC	Glucose-1-phosphate adenylyltransferase		WP_079294200.: 3119..4414	Glycogen Metabolism	COG0448
139.1_WP_079294200.:	CUST_2694_PI442190703	HOT208_60m_805A16 139.1_WP_079294200.:	GTTGATGCACAAATTACTGATTCAATTGTTTGTGAGGGTACAATCTTGAAGTCATGCAGT	glgC	Glucose-1-phosphate adenylyltransferase		WP_079294200.: 3119..4414	Glycogen Metabolism	COG0448
139.1_WP_079294201.:	CUST_2695_PI442190703	HOT208_60m_805A16 139.1_WP_079294201.:	AGAACAAATAGACTTCCTCAGAATCTTGTTCAGGCAATGAGAGACTGTTTTGGCTCTCAT	edd	phosphogluconate dehydrogenase (NADP(+)-dependent, decarboxyla		WP_079294201.: 4510..5928	Pentose Phosphate/Entner-Dudorof	COG2084
139.1_WP_079294201.:	CUST_2696_PI442190703	HOT208_60m_805A16 139.1_WP_079294201.:	TTGGCTAAAGTAGTTTCTTTATCCACAAAGGCAGGTATACCAGTCCCATGTTTATCCAGT	edd	phosphogluconate dehydrogenase (NADP(+)-dependent, decarboxyla		WP_079294201.: 4510..5928	Pentose Phosphate/Entner-Dudorof	COG2084
139.1_WP_079294201.:	CUST_2697_PI442190703	HOT208_60m_805A16 139.1_WP_079294201.:	GCAGTATACAACTATGGTTTAAATATGCCGTCAATTGCTCAAATATGGAAAGGTGGTTGC	edd	phosphogluconate dehydrogenase (NADP(+)-dependent, decarboxyla		WP_079294201.: 4510..5928	Pentose Phosphate/Entner-Dudorof	COG2084
139.1_WP_079294201.:	CUST_2698_PI442190703	HOT208_60m_805A16 139.1_WP_079294201.:	TGAAACCTTTATTTGATGCTGTAGTACTTGCCACAATTGCTAGCTATGCGCAAGGTATGG	edd	phosphogluconate dehydrogenase (NADP(+)-dependent, decarboxyla		WP_079294201.: 4510..5928	Pentose Phosphate/Entner-Dudorof	COG2084
139.1_WP_079294201.:	CUST_2699_PI442190703	HOT208_60m_805A16 139.1_WP_079294201.:	GTTGTGGACTGTTGTAAGTGCTCTAGAACTGGGGGTGTCAGTCCCAACTATTTATTCATC	edd	phosphogluconate dehydrogenase (NADP(+)-dependent, decarboxyla		WP_079294201.: 4510..5928	Pentose Phosphate/Entner-Dudorof	COG2084
139.1_WP_079294201.:	CUST_2700_PI442190703	HOT208_60m_805A16 139.1_WP_079294201.:	TGCAGAAGCTTATGACCTTATGAAGAGAGTCAAAGGTATGAATGGACAGCAGATGTCAGA	edd	phosphogluconate dehydrogenase (NADP(+)-dependent, decarboxyla		WP_079294201.: 4510..5928	Pentose Phosphate/Entner-Dudorof	COG2084
015.1_WP_079293479.:	CUST_3552_PI442190703	HOT208_60m_805A16 015.1_WP_079293479.:	CTTTTTAACTTCTGCCAAGCGGTGACTTGTGCCAATGCAAAAATAACTCTAATTTCCCCG	tal	Transaldolase		WP_079293479.: 4855..5856	Pentose Phosphate	COG0176
015.1_WP_079293479.:	CUST_3553_PI442190703	HOT208_60m_805A16 015.1_WP_079293479.:	ATTTTGATCAAGATTGCCGCAACTTGGGAGGGAATTAAGGCAGCTGAAATTTTGGAAAAA	tal	Transaldolase		WP_079293479.: 4855..5856	Pentose Phosphate	COG0176
015.1_WP_079293479.:	CUST_3554_PI442190703	HOT208_60m_805A16 015.1_WP_079293479.:	ATTGAAAATTATTTCAGGGCGCGTATCGACAGAAGTTGATGCAAGACTGAGCTTTGACAC	tal	Transaldolase		WP_079293479.: 4855..5856	Pentose Phosphate	COG0176
015.1_WP_079293479.:	CUST_3555_PI442190703	HOT208_60m_805A16 015.1_WP_079293479.:	TTGGATTGGCATAAAGCAAAAACTGGTAAAACTAGTTTTGTTGGTGCTGAAGACCCTGGT	tal	Transaldolase		WP_079293479.: 4855..5856	Pentose Phosphate	COG0176
015.1_WP_079293479.:	CUST_3556_PI442190703	HOT208_60m_805A16 015.1_WP_079293479.:	TGGCAAGTGAAAAGCTTAGTGAAGGTATCACTGGATTCAGTAAGGCTATAGAAGAATTGG	tal	Transaldolase		WP_079293479.: 4855..5856	Pentose Phosphate	COG0176
015.1_WP_079293479.:	CUST_3557_PI442190703	HOT208_60m_805A16 015.1_WP_079293479.:	AGCAGGTTGCGATCTTTTAACAATCGCACCAAAATTTCTTGAAGAACTGAAAAAAGAAAA	tal	Transaldolase		WP_079293479.: 4855..5856	Pentose Phosphate	COG0176
176.1_WP_079294308.:	CUST_4319_PI442190703	HOT208_60m_805A16 176.1_WP_079294308.:	TAGGAAAATCGGGAGAAAGATTTGCGGTTAGAAATAGTGGTGCTACAGCGGTAACAGAAG	glsF	Ferredoxin-dependent glutamate synthase		WP_079294308.: 26122..30693	Nitrogen Metabolism	COG0069
176.1_WP_079294308.:	CUST_4320_PI442190703	HOT208_60m_805A16 176.1_WP_079294308.:	ATACATGACTGGTGGGAAAGTTGTTATTCTAGGTTCCACAGGAAGGAATATTGGTGCTGG	glsF	Ferredoxin-dependent glutamate synthase		WP_079294308.: 26122..30693	Nitrogen Metabolism	COG0069
176.1_WP_079294308.:	CUST_4321_PI442190703	HOT208_60m_805A16 176.1_WP_079294308.:	GGCAACACTGGCTTCAATGGCGAACTAAACTTAAATTTCAAAGGATATGCAGGACAAAGC	glsF	Ferredoxin-dependent glutamate synthase		WP_079294308.: 26122..30693	Nitrogen Metabolism	COG0069
176.1_WP_079294308.:	CUST_4322_PI442190703	HOT208_60m_805A16 176.1_WP_079294308.:	ATCCTCCGAACAAGTCATCCTAGGAAATACTTGTCTTTATGGAGCAACAGGTGGAAAATT	glsF	Ferredoxin-dependent glutamate synthase		WP_079294308.: 26122..30693	Nitrogen Metabolism	COG0069
176.1_WP_079294308.:	CUST_4323_PI442190703	HOT208_60m_805A16 176.1_WP_079294308.:	TCTCATTTTAAGAGTACTTTCAAATTGATTGTTCCCCCAAGCGAAGAAGAGATGCTTGGT	glsF	Ferredoxin-dependent glutamate synthase		WP_079294308.: 26122..30693	Nitrogen Metabolism	COG0069
176.1_WP_079294308.:	CUST_4324_PI442190703	HOT208_60m_805A16 176.1_WP_079294308.:	TGAGATAAAAAATACAGATAGAAGTGTTTGTGCGAAAATATCAGGGGAAATCGCAGAACT	glsF	Ferredoxin-dependent glutamate synthase		WP_079294308.: 26122..30693	Nitrogen Metabolism	COG0069
176.1_WP_079294317.:	CUST_4375_PI442190703	HOT208_60m_805A16 176.1_WP_079294317.:	TGGACGTGGAGGTACTTGTGATAGTTCATCTTGGGACGCTATGTACTTAGCTATGTTCTG	psaB	Photosystem I P700 chlorophyll A apoprotein A2 PsaB		WP_079294317.: 37082..39310	Photosystem I	COG2885
176.1_WP_079294317.:	CUST_4376_PI442190703	HOT208_60m_805A16 176.1_WP_079294317.:	TCTTTATCTCCTTGGGCTTGGATGTTCCTATTTGGTCACTTAGTTTGGGCTACTGGTTTT	psaB	Photosystem I P700 chlorophyll A apoprotein A2 PsaB		WP_079294317.: 37082..39310	Photosystem I	COG2885
176.1_WP_079294317.:	CUST_4377_PI442190703	HOT208_60m_805A16 176.1_WP_079294317.:	TATAGGTCCTGCAGATTTCTTGGTTCACCATGCTATCGCTTTAGGTCTTCATACAACTGC	psaB	Photosystem I P700 chlorophyll A apoprotein A2 PsaB		WP_079294317.: 37082..39310	Photosystem I	COG2885
176.1_WP_079294317.:	CUST_4378_PI442190703	HOT208_60m_805A16 176.1_WP_079294317.:	GGTAACTTTCTACTGGCATTGGAAACACTTAGCAATTTGGCAGGGTAACGTAGCACAATT	psaB	Photosystem I P700 chlorophyll A apoprotein A2 PsaB		WP_079294317.: 37082..39310	Photosystem I	COG2885
176.1_WP_079294317.:	CUST_4379_PI442190703	HOT208_60m_805A16 176.1_WP_079294317.:	CCTACAAGTTCAGCAACTCTCGCAGCTAATTCATTACCAGGTAACCATTACTGGATGGAT	psaB	Photosystem I P700 chlorophyll A apoprotein A2 PsaB		WP_079294317.: 37082..39310	Photosystem I	COG2885
176.1_WP_079294317.:	CUST_4380_PI442190703	HOT208_60m_805A16 176.1_WP_079294317.:	GTGGTTACTGGCAAGAGTTAATTGAAACATTAGTCTGGGCACATCAGCGTACTCCAATTG	psaB	Photosystem I P700 chlorophyll A apoprotein A2 PsaB		WP_079294317.: 37082..39310	Photosystem I	COG2885
176.1_WP_079294318.:	CUST_4381_PI442190703	HOT208_60m_805A16 176.1_WP_079294318.:	TTTCCACTTCTCTTGGAAAATGCAGAGTGATGTTTGGGGACTTACTGGTGGTAATTTTGC	psaA	Photosystem I P700 chlorophyll A apoprotein A1 PsaA		WP_079294318.: 39338..41641	Photosystem I	COG2885
176.1_WP_079294318.:	CUST_4382_PI442190703	HOT208_60m_805A16 176.1_WP_079294318.:	TCAACCAAGAGCTTTATCTATTACTCAGGGTAGAGCAGTTGGTGTAACACACTTCCTGGT	psaA	Photosystem I P700 chlorophyll A apoprotein A1 PsaA		WP_079294318.: 39338..41641	Photosystem I	COG2885
176.1_WP_079294318.:	CUST_4383_PI442190703	HOT208_60m_805A16 176.1_WP_079294318.:	TCAAAGGAGTACTTTATGCAAGAAGTTCAAGGTTGATCCCTGACAAGGCTTCTTTGGGAT	psaA	Photosystem I P700 chlorophyll A apoprotein A1 PsaA		WP_079294318.: 39338..41641	Photosystem I	COG2885
176.1_WP_079294318.:	CUST_4384_PI442190703	HOT208_60m_805A16 176.1_WP_079294318.:	AGGTGGTACATGTCAAGTTTCTTCATGGGATCATGTGTTCTTGGCACTTTTCTGGATGTA	psaA	Photosystem I P700 chlorophyll A apoprotein A1 PsaA		WP_079294318.: 39338..41641	Photosystem I	COG2885
176.1_WP_079294318.:	CUST_4385_PI442190703	HOT208_60m_805A16 176.1_WP_079294318.:	TGGTTGGCTAAGAGATTTCCTTTGGGCCCAAGCTTCTCAAGTATTAACAAGTTATGGTCA	psaA	Photosystem I P700 chlorophyll A apoprotein A1 PsaA		WP_079294318.: 39338..41641	Photosystem I	COG2885
176.1_WP_079294318.:	CUST_4386_PI442190703	HOT208_60m_805A16 176.1_WP_079294318.:	TGGAAGTTTAGTTGAAGTTGGTGGAAAGGTTGCTATAGCTCCAATTCCATTAGGTACAGC	psaA	Photosystem I P700 chlorophyll A apoprotein A1 PsaA		WP_079294318.: 39338..41641	Photosystem I	COG2885
182.1_WP_079294335.:	CUST_4548_PI442190703	HOT208_60m_805A16 182.1_WP_079294335.:	TGGGCAATTTACTCCATGGGAAGATATTCCTGAGGGAACAGACGTACTTTTTTACGAAGG	prk	Phosphoribulokinase		WP_079294335.: 435..1331	Carbon Fixation	COG3954
182.1_WP_079294335.:	CUST_4549_PI442190703	HOT208_60m_805A16 182.1_WP_079294335.:	TGGCAGATGCTCTTGCAAAAGGTGAAAATTTCTCTCATTTTGGTCCAGAGGCTAATCTTT	prk	Phosphoribulokinase		WP_079294335.: 435..1331	Carbon Fixation	COG3954
182.1_WP_079294335.:	CUST_4550_PI442190703	HOT208_60m_805A16 182.1_WP_079294335.:	GGGGTAGAAGGTGATGGATACAATGTTGCCTATTATGCTGATTTGCTTGTTGGTGTTGTT	prk	Phosphoribulokinase		WP_079294335.: 435..1331	Carbon Fixation	COG3954
182.1_WP_079294335.:	CUST_4551_PI442190703	HOT208_60m_805A16 182.1_WP_079294335.:	ACTCCTGATGAGAGCTTTGTGATCATACATTTTAGAAAAGGGGCAAGAGAGAAATGGGGT	prk	Phosphoribulokinase		WP_079294335.: 435..1331	Carbon Fixation	COG3954
182.1_WP_079294335.:	CUST_4552_PI442190703	HOT208_60m_805A16 182.1_WP_079294335.:	CAGAACGAGGTTACTCAGCGGAAACCATTGTGGATACGATATTGAGAAGAATGCCTGATT	prk	Phosphoribulokinase		WP_079294335.: 435..1331	Carbon Fixation	COG3954
182.1_WP_079294335.:	CUST_4553_PI442190703	HOT208_60m_805A16 182.1_WP_079294335.:	TGTCAAGTCCAACAAGTATCGTTGTAAATGGTGGAAAAATGGGTTTCGCAATGGAACTAA	prk	Phosphoribulokinase		WP_079294335.: 435..1331	Carbon Fixation	COG3954
192.1_WP_079294345.:	CUST_4760_PI442190703	HOT208_60m_805A16 192.1_WP_079294345.:	ATATCGGAGGCATACTTAAGCATGCTCCATCATTCTTAGCGTTTACTAACCCAACCACAA	glnA	Glutamine synthetase/ glutamate--ammonialigase		WP_079294345.: 940..2165	Nitrogen Metabolism	COG0174
192.1_WP_079294345.:	CUST_4761_PI442190703	HOT208_60m_805A16 192.1_WP_079294345.:	TGAAATGCTTCTTTTAATGGGTGAATTAGGCATACCCACCGAAAAGCATCACCATGAAGT	glnA	Glutamine synthetase/ glutamate--ammonialigase		WP_079294345.: 940..2165	Nitrogen Metabolism	COG0174
192.1_WP_079294345.:	CUST_4762_PI442190703	HOT208_60m_805A16 192.1_WP_079294345.:	GTCAGGAATGTTGCTAAAAAATATGGGAAAACTGCAACGTTTATGCCCAAGCCTGTGTTT	glnA	Glutamine synthetase/ glutamate--ammonialigase		WP_079294345.: 940..2165	Nitrogen Metabolism	COG0174
192.1_WP_079294345.:	CUST_4763_PI442190703	HOT208_60m_805A16 192.1_WP_079294345.:	GGAATGCATGTTCACCAAAGTTTATGGAAGAGTGGTCAGCCACTATTTTTTGGTGAAGGA	glnA	Glutamine synthetase/ glutamate--ammonialigase		WP_079294345.: 940..2165	Nitrogen Metabolism	COG0174
192.1_WP_079294345.:	CUST_4764_PI442190703	HOT208_60m_805A16 192.1_WP_079294345.:	GCTGGTGCCGGTCAACACGAGCTTGGAATGAAATTCGATTCATTAATAAATTCTGCTGAT	glnA	Glutamine synthetase/ glutamate--ammonialigase		WP_079294345.: 940..2165	Nitrogen Metabolism	COG0174
192.1_WP_079294345.:	CUST_4765_PI442190703	HOT208_60m_805A16 192.1_WP_079294345.:	TCCAGGATTCGAAGCACCTGTAAATCTAGTTTATTCTGAGGGTAATAGATCAGCTGCAGT	glnA	Glutamine synthetase/ glutamate--ammonialigase		WP_079294345.: 940..2165	Nitrogen Metabolism	COG0174
023.1_WP_079293705.:	CUST_5388_PI442190703	HOT208_60m_805A16 023.1_WP_079293705.:	CGCGAATCAATGGCGGAAGATTAACTAGTCAAAATATGTTCCCATTGGAGGCTGTTGAAT	cyoC	heme-copper oxidase subunit III		WP_079293705.: 5363..5965	Respiration	COG1845
023.1_WP_079293705.:	CUST_5389_PI442190703	HOT208_60m_805A16 023.1_WP_079293705.:	TGTTTGGTCTTATAACTTTCCTAATTGCGGACGGGATGACTTTTGCTGGATTCTTTGCTG	cyoC	heme-copper oxidase subunit III		WP_079293705.: 5363..5965	Respiration	COG1845
023.1_WP_079293705.:	CUST_5390_PI442190703	HOT208_60m_805A16 023.1_WP_079293705.:	AGTGCAACTTTCCATAAAGCAGGCAAAGCACTTTTAAAAGACAAAAACGTTGATTCCCAA	cyoC	heme-copper oxidase subunit III		WP_079293705.: 5363..5965	Respiration	COG1845
023.1_WP_079293705.:	CUST_5391_PI442190703	HOT208_60m_805A16 023.1_WP_079293705.:	TTGCAAGTACTTTTTACGCTCTTACTGGTTTTCATGGATTACATGTCACTTTAGGCACTT	cyoC	heme-copper oxidase subunit III		WP_079293705.: 5363..5965	Respiration	COG1845
023.1_WP_079293705.:	CUST_5392_PI442190703	HOT208_60m_805A16 023.1_WP_079293705.:	AGCAGTAAATCCATTGCCTGATGGTGCTATTTATGAATTAGAACTTCCAATACCTACACT	cyoC	heme-copper oxidase subunit III		WP_079293705.: 5363..5965	Respiration	COG1845
023.1_WP_079293705.:	CUST_5393_PI442190703	HOT208_60m_805A16 023.1_WP_079293705.:	AGCTCAAAAGAAATTCAAAAAAATAATTCTGAAGTTAATGAAACACATGAAGACTTCAGA	cyoC	heme-copper oxidase subunit III		WP_079293705.: 5363..5965	Respiration	COG1845
023.1_WP_079293707.:	CUST_5394_PI442190703	HOT208_60m_805A16 023.1_WP_079293707.:	TGCTTTGCTCCTCAACATTGGCTTGGTTTAAATGGAATGCCTAGAAGAGTTGCAGAATAT	cyoB/coxA	Cytochrome c oxidase subunit I		WP_079293707.: 5972..7597	Respiration	COG0843
023.1_WP_079293707.:	CUST_5395_PI442190703	HOT208_60m_805A16 023.1_WP_079293707.:	GCGCATCACATGTTTACAAGTGGAACTCCACCTTGGATGAGATTGTTCTTTACTATTGCC	cyoB/coxA	Cytochrome c oxidase subunit I		WP_079293707.: 5972..7597	Respiration	COG0843
023.1_WP_079293707.:	CUST_5396_PI442190703	HOT208_60m_805A16 023.1_WP_079293707.:	CTCTTACACCTGAATGGTTAACATCTTCTCCACCTCCAGTGGAAAATTGGGAAGGAGAAG	cyoB/coxA	Cytochrome c oxidase subunit I		WP_079293707.: 5972..7597	Respiration	COG0843
023.1_WP_079293707.:	CUST_5397_PI442190703	HOT208_60m_805A16 023.1_WP_079293707.:	TGCTTTAGCACAGGTACCTTTCGATATTCACGTACATGATACCTATTTCGTTGTAGCCCA	cyoB/coxA	Cytochrome c oxidase subunit I		WP_079293707.: 5972..7597	Respiration	COG0843
023.1_WP_079293707.:	CUST_5398_PI442190703	HOT208_60m_805A16 023.1_WP_079293707.:	TGATCCTCAATTCCAGTTCGTTAATCAAATTAGTAGCCTTGGGGCTCTTTTGATGGCTA	cyoB/coxA	Cytochrome c oxidase subunit I		WP_079293707.: 5972..7597	Respiration	COG0843
023.1_WP_079293707.:	CUST_5399_PI442190703	HOT208_60m_805A16 023.1_WP_079293707.:	GGTAGCAACACTATGGGGAGGTAAAATTTCCATTAATAGTGCAATGTTATTCTCTTGCGG	cyoB/coxA	Cytochrome c oxidase subunit I		WP_079293707.: 5972..7597	Respiration	COG0843
023.1_WP_079293709.:	CUST_5400_PI442190703	HOT208_60m_805A16 023.1_WP_079293709.:	GGAAAATATCCGATAATTTGCGCAGAATTATGTGGCCCATATCATGGAGGAATGAGAGCC	ctaC/coxB	Cytochrome c oxidase subunit II		WP_079293709.: 7594..8397	Respiration	COG1622
023.1_WP_079293709.:	CUST_5401_PI442190703	HOT208_60m_805A16 023.1_WP_079293709.:	TCTTTTTGGATTGGTTTTAATGTAAATTTGCTCCCAGCAGAAGCAAGTATTAATGCCCCA	ctaC/coxB	Cytochrome c oxidase subunit II		WP_079293709.: 7594..8397	Respiration	COG1622
023.1_WP_079293709.:	CUST_5402_PI442190703	HOT208_60m_805A16 023.1_WP_079293709.:	TCCCAAGGGCAATTTCATATCAGGAGAACTACACGTTCCTGTTGATCAAAAAGTATCAAT	ctaC/coxB	Cytochrome c oxidase subunit II		WP_079293709.: 7594..8397	Respiration	COG1622
023.1_WP_079293709.:	CUST_5403_PI442190703	HOT208_60m_805A16 023.1_WP_079293709.:	AATGGAATCCAAAGATGTCATTCATGCTTTTTGGGTACCAGAATTCAGAATTAAACAGGA	ctaC/coxB	Cytochrome c oxidase subunit II		WP_079293709.: 7594..8397	Respiration	COG1622
023.1_WP_079293709.:	CUST_5404_PI442190703	HOT208_60m_805A16 023.1_WP_079293709.:	AGCTACAACATCTACGATCGAATGGGAGGGATGAAAGAACTAAATCATAATCATGAACTG	ctaC/coxB	Cytochrome c oxidase subunit II		WP_079293709.: 7594..8397	Respiration	COG1622
023.1_WP_079293709.:	CUST_5405_PI442190703	HOT208_60m_805A16 023.1_WP_079293709.:	AGGAAAAGAGATGATCAGATAGGCGATGGTATAGCTTTAGAGGGAAATTTAAGCTTAGAA	ctaC/coxB	Cytochrome c oxidase subunit II		WP_079293709.: 7594..8397	Respiration	COG1622
037.1_WP_079293758.:	CUST_5682_PI442190703	HOT208_60m_805A16 037.1_WP_079293758.:	TGGAAATGATTGGCCTACTTCCGATGGAACATGTCAAAGAGATTACTTGCATGTAGTCGA	galE	UDP-glucose 4-epimerase		WP_079293758.: 1371..2420	Galactose metabolism	COG1087
037.1_WP_079293758.:	CUST_5683_PI442190703	HOT208_60m_805A16 037.1_WP_079293758.:	TGGAAGCCTGAAAAGAGTATTGAGGATATGTGTAGGGATGGATGGAAATGGCAAAAAAGT	galE	UDP-glucose 4-epimerase		WP_079293758.: 1371..2420	Galactose metabolism	COG1087
037.1_WP_079293758.:	CUST_5684_PI442190703	HOT208_60m_805A16 037.1_WP_079293758.:	TCCAATAGGAGCCCATCCCTCTGGATTGATTGGTGAAAATTCATCATCTAAACCTGAAAA	galE	UDP-glucose 4-epimerase		WP_079293758.: 1371..2420	Galactose metabolism	COG1087
037.1_WP_079293758.:	CUST_5685_PI442190703	HOT208_60m_805A16 037.1_WP_079293758.:	CACAAATAGGCGATCTGGTGATGTAGGAATCTCTTTAGCAAATACTGAAAAAATGGTTGA	galE	UDP-glucose 4-epimerase		WP_079293758.: 1371..2420	Galactose metabolism	COG1087
037.1_WP_079293758.:	CUST_5686_PI442190703	HOT208_60m_805A16 037.1_WP_079293758.:	AGTAGTGCAACAGTTTATGGAAATTCAAGTGAAATTCCATTTAAAGAAACTTTAAAGACA	galE	UDP-glucose 4-epimerase		WP_079293758.: 1371..2420	Galactose metabolism	COG1087
037.1_WP_079293758.:	CUST_5687_PI442190703	HOT208_60m_805A16 037.1_WP_079293758.:	TGGGATTTTAATGTTGGTGGATCAATAAACATTTTAAAAGTAATGGAAAAAAACAATTGT	galE	UDP-glucose 4-epimerase		WP_079293758.: 1371..2420	Galactose metabolism	COG1087
037.1_WP_079293759.:	CUST_5688_PI442190703	HOT208_60m_805A16 037.1_WP_079293759.:	AGCTACAGGTGCAGATGTAAGAGAAGTATCCAGAGCAATTGGCACAGATAGTCGTATAGG	ugd	UDP-glucose 6-dehydrogenase/nucleotide sugar dehydrogenase		WP_079293759.: 2521..3906	Amino sugar and nucleotide sugar r	COG1004
037.1_WP_079293759.:	CUST_5689_PI442190703	HOT208_60m_805A16 037.1_WP_079293759.:	TGGATAGAAGCTGCAAAGCAAATGGCTAATCCTGCTTGGGTTTTTGATGCTAGATCAATT	ugd	UDP-glucose 6-dehydrogenase/nucleotide sugar dehydrogenase		WP_079293759.: 2521..3906	Amino sugar and nucleotide sugar r	COG1004
037.1_WP_079293759.:	CUST_5690_PI442190703	HOT208_60m_805A16 037.1_WP_079293759.:	GGAGCAGTGAACTGGCAAAACTTACAGCCAATGCTTTTTTAGCACAAAGAATTAGCTCAA	ugd	UDP-glucose 6-dehydrogenase/nucleotide sugar dehydrogenase		WP_079293759.: 2521..3906	Amino sugar and nucleotide sugar r	COG1004
037.1_WP_079293759.:	CUST_5691_PI442190703	HOT208_60m_805A16 037.1_WP_079293759.:	ACTCAATTGGGAATTGGGAATTCAGTAAAAGTCTTGATATTTTTGAAGGCGCGCATGCAA	ugd	UDP-glucose 6-dehydrogenase/nucleotide sugar dehydrogenase		WP_079293759.: 2521..3906	Amino sugar and nucleotide sugar r	COG1004
037.1_WP_079293759.:	CUST_5692_PI442190703	HOT208_60m_805A16 037.1_WP_079293759.:	TGGACTTAATGAAGTTGCAAATTTCTGGGAAACTGTCGTAAACCTCAATAATTGGCATCA	ugd	UDP-glucose 6-dehydrogenase/nucleotide sugar dehydrogenase		WP_079293759.: 2521..3906	Amino sugar and nucleotide sugar r	COG1004
037.1_WP_079293759.:	CUST_5693_PI442190703	HOT208_60m_805A16 037.1_WP_079293759.:	TGCAAGGATCTTATTGATGAAGGTGCAAGAATTTTTATACATGACCCCAAAGTAAGCGAA	ugd	UDP-glucose 6-dehydrogenase/nucleotide sugar dehydrogenase		WP_079293759.: 2521..3906	Amino sugar and nucleotide sugar r	COG1004
070.1_WP_079293792.:	CUST_6087_PI442190703	HOT208_60m_805A16 070.1_WP_079293792.:	CTGGACAAGCAAATTATTCTGCAGCCAAAGCTGGAGTTATTGGATTTACCAAAACTTGCG	gdh	3-oxoacyl-acyl-carrier-protein reductase	acyl-carrier-pro	WP_079293792.: <1..686	Entner-Dudoroff	COG1028
070.1_WP_079293792.:	CUST_6088_PI442190703	HOT208_60m_805A16 070.1_WP_079293792.:	ATCACTGGAGCTAGCAGAGGAATTGGTAAAGAAATTGCTTTAGAACTAAGCCGATTGGGA	gdh	3-oxoacyl-acyl-carrier-protein reductase	acyl-carrier-pro	WP_079293792.: <1..686	Entner-Dudoroff	COG1028
070.1_WP_079293792.:	CUST_6089_PI442190703	HOT208_60m_805A16 070.1_WP_079293792.:	TGGCACCATTGATATCCTCATTAACAATGCTGGAATAACTAGAGATGGACTATTGATGAG	gdh	3-oxoacyl-acyl-carrier-protein reductase	acyl-carrier-pro	WP_079293792.: <1..686	Entner-Dudoroff	COG1028
070.1_WP_079293792.:	CUST_6090_PI442190703	HOT208_60m_805A16 070.1_WP_079293792.:	TGCCTCAAGAGGTATAAATGTTAATGCAATAGCTCCAGGTTTTATAGAAACAGAGATGAC	gdh	3-oxoacyl-acyl-carrier-protein reductase	acyl-carrier-pro	WP_079293792.: <1..686	Entner-Dudoroff	COG1028
070.1_WP_079293792.:	CUST_6091_PI442190703	HOT208_60m_805A16 070.1_WP_079293792.:	TCCTTTAGGGAAATTGGGAAGTTGTACTCAAATTGCAAACTTAGTGTCATTCTTAGTTTC	gdh	3-oxoacyl-acyl-carrier-protein reductase	acyl-carrier-pro	WP_079293792.: <1..686	Entner-Dudoroff	COG1028

070.1_WP_079293792.:	CUST_6092_PI442190703	HOT208_60m_805A16	070.1_WP_079293792.:	ATGATGAAAAAAAGAAGTGGTAGCATCGTAAATATTTCATCTGTTGTTGGCATAATTGGT	gdh	3-oxoacyl-acyl-carrier-protein reductase	acyl-carrier-prot	WP_079293792.:	<1..686	Entner-Dudoroff	COG1028
103.1_8	CUST_35_PI442190707	HOT208_60m_826P21	103.1_8	AAAGAAATAGCAACTATTGCAGATATGCCTATGCCGCATCAACTCTGCGATCCACAAATT	psaA	Photosystem I P700 chlorophyll A apoprotein A1 PsaA			<1..1140	Photosystem I	COG2885
103.1_8	CUST_36_PI442190707	HOT208_60m_826P21	103.1_8	CCAATATTATTCCCTGCACCCAAAGGTCATCAAGGTCTTTTTGAGTTCATGGCAGAAAGT	psaA	Photosystem I P700 chlorophyll A apoprotein A1 PsaA			<1..1140	Photosystem I	COG2885
103.1_8	CUST_37_PI442190707	HOT208_60m_826P21	103.1_8	AAATGGAGTGGTTCCAAGATATTGAGTCGATGCTCAACCACCACATAGCAGGTTTAGTAG	psaA	Photosystem I P700 chlorophyll A apoprotein A1 PsaA			<1..1140	Photosystem I	COG2885
103.1_8	CUST_38_PI442190707	HOT208_60m_826P21	103.1_8	TCATATCGGAGCTCCTACTGCTGCACTTTTAGATGCAATTGATGCAGGTTCTCCTTTAAT	psaA	Photosystem I P700 chlorophyll A apoprotein A1 PsaA			<1..1140	Photosystem I	COG2885
103.1_8	CUST_39_PI442190707	HOT208_60m_826P21	103.1_8	GCGATGCTTGGATCAATAAGCATACTCGTATCTCATCACATGTATGCGATGCCTCCATAT	psaA	Photosystem I P700 chlorophyll A apoprotein A1 PsaA			<1..1140	Photosystem I	COG2885
103.1_8	CUST_40_PI442190707	HOT208_60m_826P21	103.1_8	GTTAGCTTTCTCAGACTTCCTTACTTTCAAAGGTGGACTTAACCCTGTGACAGGAAGCTT	psaA	Photosystem I P700 chlorophyll A apoprotein A1 PsaA			<1..1140	Photosystem I	COG2885
109.1_15	CUST_77_PI442190707	HOT208_60m_826P21	109.1_15	AGCCACTATTGGCCCTGCAACTCAATCTGAAGAGATAATTACAGATTTAATTAAAGCTGG	pykF	Pyruvate kinase			<1..501	Glycolysis	COG0469
109.1_15	CUST_78_PI442190707	HOT208_60m_826P21	109.1_15	TGGAGTGTATGGTAACTGTAGGAGGGGTTCTTTCAATCAATAAAGGTGTTAATTTTCCAG	pykF	Pyruvate kinase			<1..501	Glycolysis	COG0469
109.1_15	CUST_79_PI442190707	HOT208_60m_826P21	109.1_15	ACCTACGACAAACTTTCTCAAGAAGTTAGCGAAGGGAAAAGAATACTTTTAGATGATGGA	pykF	Pyruvate kinase			<1..501	Glycolysis	COG0469
109.1_15	CUST_80_PI442190707	HOT208_60m_826P21	109.1_15	GGAATACTACAAGATCTTCAAGGACCAAAAATAAGATTAGGACGCTTTAAAGATGGTCCA	pykF	Pyruvate kinase			<1..501	Glycolysis	COG0469
109.1_15	CUST_81_PI442190707	HOT208_60m_826P21	109.1_15	AAACTTCTCACATGGAGATCATAAAGATCATGCTGAGAGAATAAAAACCATAAGAGAAGT	pykF	Pyruvate kinase			<1..501	Glycolysis	COG0469
109.1_15	CUST_82_PI442190707	HOT208_60m_826P21	109.1_15	AGGTGATAAATTTTCATTAACATCGAATGAAGTCGAATGTACAAATACTATTGCAAATGT	pykF	Pyruvate kinase			<1..501	Glycolysis	COG0469
118.1_27	CUST_137_PI442190707	HOT208_60m_826P21	118.1_27	GCTGTAATGCTTTCAAATGAAACTGCAGTTGGCGATTATCCTGTAGAGGCAGTAGAAACG	pykF	Pyruvate kinase			<1..723	Glycolysis	COG0469
118.1_27	CUST_138_PI442190707	HOT208_60m_826P21	118.1_27	ATCCCAAATGCTATTAGTGCAGCAGTAAGCAATATAGCTAGACAACTTGATGCAGGAGCT	pykF	Pyruvate kinase			<1..723	Glycolysis	COG0469
118.1_27	CUST_139_PI442190707	HOT208_60m_826P21	118.1_27	AAATTCAGACCACCAACACCTATCTTGGCAACTACTACAGAGAGAAGTGTAGCGAGAAGA	pykF	Pyruvate kinase			<1..723	Glycolysis	COG0469
118.1_27	CUST_140_PI442190707	HOT208_60m_826P21	118.1_27	TATCCCCATAATTACAGCGACTCAAATGCTTGACTCTATGGCTTCGAATCCAAGACCAAC	pykF	Pyruvate kinase			<1..723	Glycolysis	COG0469
118.1_27	CUST_141_PI442190707	HOT208_60m_826P21	118.1_27	GGGTTATGGTTGCAAGAGGTGATTTAGGAGTAGAAATGCCTGCTGAAGAAGTTCCTCTTT	pykF	Pyruvate kinase			<1..723	Glycolysis	COG0469
118.1_27	CUST_142_PI442190707	HOT208_60m_826P21	118.1_27	TGGCAACTATAGCAAGAAGAATTGAAAGGGATTATCCACTTAAAGCTATTGAGAGCCACT	pykF	Pyruvate kinase			<1..723	Glycolysis	COG0469
015.1_WP_079292627.:	CUST_419_PI442190707	HOT208_60m_826P21	015.1_WP_079292627.:	TAGCAGGAGCTCCTCTTCAAGAAGCATTTAGGAATGCTGATGATGTTTTAAGGATGGGAG	ftsZ	cell division protein FtsZ		WP_079292627.:	5547..6662	Cell Division	COG0206
015.1_WP_079292627.:	CUST_420_PI442190707	HOT208_60m_826P21	015.1_WP_079292627.:	GTGGTCGATGAGGCAATGGAAGGGGAGATACAAGTTACTGTAATTGCTACAGGATTTGAA	ftsZ	cell division protein FtsZ		WP_079292627.:	5547..6662	Cell Division	COG0206
015.1_WP_079292627.:	CUST_421_PI442190707	HOT208_60m_826P21	015.1_WP_079292627.:	TGCGACAAGCAGAGGAGGGGATAGCAAGACTAGCTGAAAACGTTGATACTCTTATAGTTA	ftsZ	cell division protein FtsZ		WP_079292627.:	5547..6662	Cell Division	COG0206
015.1_WP_079292627.:	CUST_422_PI442190707	HOT208_60m_826P21	015.1_WP_079292627.:	CAGCACAGGCAGCCATGAATAGTCCTTTATTAGAAGCAGCAAGAATTGATGGAGCTAAAG	ftsZ	cell division protein FtsZ		WP_079292627.:	5547..6662	Cell Division	COG0206
015.1_WP_079292627.:	CUST_423_PI442190707	HOT208_60m_826P21	015.1_WP_079292627.:	CGTTGTTGCAGAAGTAGCCAAACAAAGTGGGGCTTTAACAGTAGGTATAGTAACCAAACC	ftsZ	cell division protein FtsZ		WP_079292627.:	5547..6662	Cell Division	COG0206
015.1_WP_079292627.:	CUST_424_PI442190707	HOT208_60m_826P21	015.1_WP_079292627.:	GCTGCGTTATAAATATTACTGGAGGCAAAGACATGACTTTAGAAGACATGACCTCTGCAT	ftsZ	cell division protein FtsZ		WP_079292627.:	5547..6662	Cell Division	COG0206
162.1_WP_032515541.:	CUST_1204_PI442190707	HOT208_60m_826P21	162.1_WP_032515541.:	GGCAATCGAGAATCAATGGCGGAAGATTAACTAGTCAAAATATGTTCCCGTTGGAAGCTG	cyoC	heme-copper oxidase subunit III		WP_032515541.:	53646..54248	Respiration	COG1845
162.1_WP_032515541.:	CUST_1205_PI442190707	HOT208_60m_826P21	162.1_WP_032515541.:	TTGGTCTTATAACTTTCCTAATTGCGGACGGAATGACTTTTGCTGGATTCTTTGCTGCTT	cyoC	heme-copper oxidase subunit III		WP_032515541.:	53646..54248	Respiration	COG1845
162.1_WP_032515541.:	CUST_1206_PI442190707	HOT208_60m_826P21	162.1_WP_032515541.:	TTGCAAGTACTTTTTATGCTCTCACTGGTTTTCATGGATTACATGTCACTTTAGGCACTT	cyoC	heme-copper oxidase subunit III		WP_032515541.:	53646..54248	Respiration	COG1845
162.1_WP_032515541.:	CUST_1207_PI442190707	HOT208_60m_826P21	162.1_WP_032515541.:	AGCAGTAAATCCATTACCAGATGGTGCTATTTATGAATTAGAACTACCGATACCTACACT	cyoC	heme-copper oxidase subunit III		WP_032515541.:	53646..54248	Respiration	COG1845
162.1_WP_032515541.:	CUST_1208_PI442190707	HOT208_60m_826P21	162.1_WP_032515541.:	AGTGCAACTTTCCATAAAGCAGGTAAAGCACTTTTAAAAGATAAAAACTCTGATTCCCAA	cyoC	heme-copper oxidase subunit III		WP_032515541.:	53646..54248	Respiration	COG1845
162.1_WP_032515541.:	CUST_1209_PI442190707	HOT208_60m_826P21	162.1_WP_032515541.:	AGCTCAAAAGAAATTCAAAAAAATAATTCTGAAGTTAATGAAACACATGAAGACTTCAGA	cyoC	heme-copper oxidase subunit III		WP_032515541.:	53646..54248	Respiration	COG1845
162.1_WP_079321285.:	CUST_1210_PI442190707	HOT208_60m_826P21	162.1_WP_079321285.:	TGTTTTTGGAGGTATTACAGGAGTTGCTTTGGCACAGGTACCTTTCGATATTCACGTACA	cyoB/coxA	Cytochrome c oxidase subunit I		WP_079321285.:	54255..55880	Respiration	COG0843
162.1_WP_079321285.:	CUST_1211_PI442190707	HOT208_60m_826P21	162.1_WP_079321285.:	ATTGGCTTGGTTTAAATGGAATGCCAAGAAGAGTTGCAGAATATGACCCTCAATTCCAGT	cyoB/coxA	Cytochrome c oxidase subunit I		WP_079321285.:	54255..55880	Respiration	COG0843
162.1_WP_079321285.:	CUST_1212_PI442190707	HOT208_60m_826P21	162.1_WP_079321285.:	GAGATAACCCTTGGAATGCTCTTACACCTGAATGGTTAACATCTTCTCCACCTCCAGTTG	cyoB/coxA	Cytochrome c oxidase subunit I		WP_079321285.:	54255..55880	Respiration	COG0843
162.1_WP_079321285.:	CUST_1213_PI442190707	HOT208_60m_826P21	162.1_WP_079321285.:	CCCCTTGGATGAGATTATTCTTTACTATCGCCACAGCATTTATTGCTGTTCCGACTGGTA	cyoB/coxA	Cytochrome c oxidase subunit I		WP_079321285.:	54255..55880	Respiration	COG0843
162.1_WP_079321285.:	CUST_1214_PI442190707	HOT208_60m_826P21	162.1_WP_079321285.:	TGGGGATAGTAGTTTTAGGTTTAGTTGTTTGGGCTCATCACATGTTTACGAGTGGAACGC	cyoB/coxA	Cytochrome c oxidase subunit I		WP_079321285.:	54255..55880	Respiration	COG0843
162.1_WP_079321285.:	CUST_1215_PI442190707	HOT208_60m_826P21	162.1_WP_079321285.:	GGTTGCAACATTATGGGGAGGTAAAATTTCCATCAATAGTGCAATGTTATTCTCTTGCGG	cyoB/coxA	Cytochrome c oxidase subunit I		WP_079321285.:	54255..55880	Respiration	COG0843
016.1_WP_079321136.:	CUST_1947_PI442190707	HOT208_60m_826P21	016.1_WP_079321136.:	GGCAACCTATGCATTATTGGAGACGTTAATCCAGGGGCAATAGTTTCCGCAAAAAAAAAC	minC	septum site-determining protein MinC		WP_079321136.:	20868..21414	Cell Division	COG0850
016.1_WP_079321136.:	CUST_1948_PI442190707	HOT208_60m_826P21	016.1_WP_079321136.:	TAGGGATCGCATTCGCCGGAAAAACTGGAAATAAAAATGCCTCTATTGCATCACTTTACT	minC	septum site-determining protein MinC		WP_079321136.:	20868..21414	Cell Division	COG0850
016.1_WP_079321136.:	CUST_1949_PI442190707	HOT208_60m_826P21	016.1_WP_079321136.:	TTAAGTCAAAAGACAAAGACGATGTTCTTTACAAAGGTACAGTTCGATCGGGAGACAGAA	minC	septum site-determining protein MinC		WP_079321136.:	20868..21414	Cell Division	COG0850
016.1_WP_079321136.:	CUST_1950_PI442190707	HOT208_60m_826P21	016.1_WP_079321136.:	AACAGTTTTCTTCCATAAAGGAACCACTGGAAGCAAAAATTTTCGCAATCAATGAGTCAA	minC	septum site-determining protein MinC		WP_079321136.:	20868..21414	Cell Division	COG0850
016.1_WP_079321136.:	CUST_1951_PI442190707	HOT208_60m_826P21	016.1_WP_079321136.:	ACGATATTAAGTGGAAAGTCTTTAAAAATAGATTCAACTTTTTATAAAGAGCGAGAGATT	minC	septum site-determining protein MinC		WP_079321136.:	20868..21414	Cell Division	COG0850
016.1_WP_079321136.:	CUST_1952_PI442190707	HOT208_60m_826P21	016.1_WP_079321136.:	TGGAAATCATTTTAAGTAGCACTAAAGGTAAATATTTAGAAATTTGTTCTATGTTTGATT	minC	septum site-determining protein MinC		WP_079321136.:	20868..21414	Cell Division	COG0850
176.1_WP_079294399.:	CUST_2891_PI442190707	HOT208_60m_826P21	176.1_WP_079294399.:	ATATCTTGTGAAAGGATCGTTAGCGGCACTGGTTTATCAAGAATTGCTGAGTGGAGACTT	glk	Glucokinase		WP_079294399.:	11644..12681	Glycolysis	COG0837
176.1_WP_079294399.:	CUST_2892_PI442190707	HOT208_60m_826P21	176.1_WP_079294399.:	ATCCAGAATGGATCACAATCTGAAGGTACTAACAATGATTTGCATGCCATTGTTGGAGCC	glk	Glucokinase		WP_079294399.:	11644..12681	Glycolysis	COG0837
176.1_WP_079294399.:	CUST_2893_PI442190707	HOT208_60m_826P21	176.1_WP_079294399.:	GAATTGTGCTTTGGCGGGTTATGGATTTCTGGAGGAACCGCATCAAAACATATCAAAAAC	glk	Glucokinase		WP_079294399.:	11644..12681	Glycolysis	COG0837
176.1_WP_079294399.:	CUST_2894_PI442190707	HOT208_60m_826P21	176.1_WP_079294399.:	AAAAGTTTTAGCCAGCGAAGGGGGACACGTTGAATACTCTCCAAAGTCAGAATTAGAGTG	glk	Glucokinase		WP_079294399.:	11644..12681	Glycolysis	COG0837
176.1_WP_079294399.:	CUST_2895_PI442190707	HOT208_60m_826P21	176.1_WP_079294399.:	AGAAGGGGATCAAATAATGGTTGAAGTTGAAAGGATCTGGTTAGGCGCTTATGCCTCTTT	glk	Glucokinase		WP_079294399.:	11644..12681	Glycolysis	COG0837
176.1_WP_079294399.:	CUST_2896_PI442190707	HOT208_60m_826P21	176.1_WP_079294399.:	ATGAAGAGTTTGGACTTTTTAGCGCAGCCTGCAGAGCAAAAATGCTTTTAAAAACTAAGT	glk	Glucokinase		WP_079294399.:	11644..12681	Glycolysis	COG0837
180.1_585	CUST_2983_PI442190707	HOT208_60m_826P21	180.1_585	GTTAAGAGTCTTAGATACTCCAATTGCCGAGAATAGTTTTACGGGTATGGCTGTGGGTGC	pdhB	Pyruvate dehydrogenase E1 component beta subunit			325..1093	Glycolysis/Krebs cycle	COG0022
180.1_585	CUST_2984_PI442190707	HOT208_60m_826P21	180.1_585	CAACTTGGTGCTGAGCACAGTCAAAGACTTGAAGCATATTTTCATGCAGTTCCTGGAATA	pdhB	Pyruvate dehydrogenase E1 component beta subunit			325..1093	Glycolysis/Krebs cycle	COG0022
180.1_585	CUST_2985_PI442190707	HOT208_60m_826P21	180.1_585	ATGGCAAAAGATGTAAATGTTTGTGTTATGGGGGGAGGATGTTGGTCAATATGGAGGTTC	pdhB	Pyruvate dehydrogenase E1 component beta subunit			325..1093	Glycolysis/Krebs cycle	COG0022
180.1_585	CUST_2986_PI442190707	HOT208_60m_826P21	180.1_585	ATTTGTCTGAAGAATTACCTGAGGGAGATTATACGTGCGCTTTAGATCAGGCTGACGTTG	pdhB	Pyruvate dehydrogenase E1 component beta subunit			325..1093	Glycolysis/Krebs cycle	COG0022
180.1_585	CUST_2987_PI442190707	HOT208_60m_826P21	180.1_585	TATACAAGTGGCGGAAATTATAAAATACCAGCAGTAGTTCGAGGACCTGGAGGAGTTGGT	pdhB	Pyruvate dehydrogenase E1 component beta subunit			325..1093	Glycolysis/Krebs cycle	COG0022
180.1_585	CUST_2988_PI442190707	HOT208_60m_826P21	180.1_585	GCAATGACTGGCTTAAGACCAATAGTAGAAGGAATGAATATGGGTTTTTTGCTTTTAGCT	pdhB	Pyruvate dehydrogenase E1 component beta subunit			325..1093	Glycolysis/Krebs cycle	COG0022
194.1_WP_079321393.:	CUST_3711_PI442190707	HOT208_60m_826P21	194.1_WP_079321393.:	AGAAGTACGCTCAAGAGTTAATATTTCTCCGTTAGGAGCTGCAGCATTAGCTGGAACAAA	argH	Argininosuccinate lyase		WP_079321393.:	996..1919	Amino Acid metabolism	COG0165
194.1_WP_079321393.:	CUST_3715_PI442190707	HOT208_60m_826P21	194.1_WP_079321393.:	AGGCACTTTACAGCTTCAGAATTGGGTTTTACAAAGATTTATAAAAACAGTATCGATGCA	argH	Argininosuccinate lyase		WP_079321393.:	996..1919	Amino Acid metabolism	COG0165
268.1_WP_011863563.:	CUST_4684_PI442190707	HOT208_60m_826P21	268.1_WP_011863563.:	TCGCACAGTTTGATGAACTAGCTGCCTTTTCTCAATTTGCATCTGATCTTGATGAAGCAA	atpA	ATP synthase alpha subunit		WP_011863563.:	30477..31994	Energy Metabolism	COG0056
268.1_WP_011863563.:	CUST_4685_PI442190707	HOT208_60m_826P21	268.1_WP_011863563.:	GCAAAACTTTCTGATGCAATGGGAGGGGGTTCTATGACAGCTCTTCCAATTATTGAAACT	atpA	ATP synthase alpha subunit		WP_011863563.:	30477..31994	Energy Metabolism	COG0056
268.1_WP_011863563.:	CUST_4686_PI442190707	HOT208_60m_826P21	268.1_WP_011863563.:	TGTCTCTTCTTTTAAAAAGACCACCAGGAAGAGAGGCTTATCCTGGAGACGTGTTCTACT	atpA	ATP synthase alpha subunit		WP_011863563.:	30477..31994	Energy Metabolism	COG0056
268.1_WP_011863563.:	CUST_4687_PI442190707	HOT208_60m_826P21	268.1_WP_011863563.:	CCGCAATTCTCTCCGCTAAATCTTGCAGAACAAGTTGCAGTTGTTTATGCAGGAGTTAAA	atpA	ATP synthase alpha subunit		WP_011863563.:	30477..31994	Energy Metabolism	COG0056
268.1_WP_011863563.:	CUST_4688_PI442190707	HOT208_60m_826P21	268.1_WP_011863563.:	CAGGCAGGAGATGTTTCTGCTTACATCCCCACCAATGTTATTTCAATTACAGATGGACAA	atpA	ATP synthase alpha subunit		WP_011863563.:	30477..31994	Energy Metabolism	COG0056
268.1_WP_011863567.:	CUST_4710_PI442190707	HOT208_60m_826P21	268.1_WP_011863567.:	CCAAGTGGAGAATTAGGAGCACCTACTGCGGATATCAATACAACAATAGCCTTGGCTTTA	atpI	ATP synthase subunit A		WP_011863567.:	34016..34741	Energy Metabolism	COG0356
268.1_WP_011863567.:	CUST_4711_PI442190707	HOT208_60m_826P21	268.1_WP_011863567.:	TTCCTAGGATTATTTACCAGTGCAATTCAGGCATTGATTTTCGCAACTTTAGCGGCCTAC	atpI	ATP synthase subunit A		WP_011863567.:	34016..34741	Energy Metabolism	COG0356
268.1_WP_011863567.:	CUST_4712_PI442190707	HOT208_60m_826P21	268.1_WP_011863567.:	AAGGCCTTCAAAACTTGCTTGAGTTCCTCTGGGATTACATAAGAGATCTTGCTAGAACGC	atpI	ATP synthase subunit A		WP_011863567.:	34016..34741	Energy Metabolism	COG0356
268.1_WP_011863567.:	CUST_4713_PI442190707	HOT208_60m_826P21	268.1_WP_011863567.:	AGCTTTAGAAGTTGGTCAACATCTTTATTGGCAAATTGGAAATATCAGACTTCATGGGCA	atpI	ATP synthase subunit A		WP_011863567.:	34016..34741	Energy Metabolism	COG0356
268.1_WP_011863567.:	CUST_4714_PI442190707	HOT208_60m_826P21	268.1_WP_011863567.:	TGCTTCCTTTCAAAATTCTAGAGGACTTCACAAAACCTTTATCACTCTCTTTCAGGCTAT	atpI	ATP synthase subunit A		WP_011863567.:	34016..34741	Energy Metabolism	COG0356
268.1_WP_011863567.:	CUST_4715_PI442190707	HOT208_60m_826P21	268.1_WP_011863567.:	TATGCAGGTTTAAGCAATAAGGGTTGGAGATACTTCGAATATTATGTTCATCCAACTCCT	atpI	ATP synthase subunit A		WP_011863567.:	34016..34741	Energy Metabolism	COG0356
027.1_WP_079293072.:	CUST_5364_PI442190707	HOT208_60m_826P21	027.1_WP_079293072.:	TAGACTTCCTCAGAACCTTGTTCAGGCCATGAGAGACTGTTTTGGCTCTCATACATATGA	edd	phosphogluconate dehydrogenase (NADP(+)-dependent, decarboxyla		WP_079293072.:	4316..5734	Pentose Phosphate/Entner-Dudorof	COG2084
027.1_WP_079293072.:	CUST_5365_PI442190707	HOT208_60m_826P21	027.1_WP_079293072.:	TGGTTTGAATATGCCGTCAATTGCTCAAATATGGAAAGGTGGTGCATAATTAGATCAAA	edd	phosphogluconate dehydrogenase (NADP(+)-dependent, decarboxyla		WP_079293072.:	4316..5734	Pentose Phosphate/Entner-Dudorof	COG2084
027.1_WP_079293072.:	CUST_5366_PI442190707	HOT208_60m_826P21	027.1_WP_079293072.:	TAGTCCTTGCCACAATTGCTAGCTATGCTCAGGGTATGGACATTTTAAGAGAAGCATCTG	edd	phosphogluconate dehydrogenase (NADP(+)-dependent, decarboxyla		WP_079293072.:	4316..5734	Pentose Phosphate/Entner-Dudorof	COG2084
027.1_WP_079293072.:	CUST_5367_PI442190707	HOT208_60m_826P21	027.1_WP_079293072.:	GGGTTATGGACTGTTGTAAGTGCTTTAGAACTGGGGGTATCAGTCCCAACTATTTATGCA	edd	phosphogluconate dehydrogenase (NADP(+)-dependent, decarboxyla		WP_079293072.:	4316..5734	Pentose Phosphate/Entner-Dudorof	COG2084
027.1_WP_079293072.:	CUST_5368_PI442190707	HOT208_60m_826P21	027.1_WP_079293072.:	GATGAGATAACTGGAGATGATGTCGTGGAAAAAATATTAGATAAAGCTGGCCAGAAAGGT	edd	phosphogluconate dehydrogenase (NADP(+)-dependent, decarboxyla		WP_079293072.:	4316..5734	Pentose Phosphate/Entner-Dudorof	COG2084
027.1_WP_079293072.:	CUST_5369_PI442190707	HOT208_60m_826P21	027.1_WP_079293072.:	ACTTGCAGAAGCTTATGACCTTATGAAGAGAGTCAAAGGTATGAATGGACAGCAGATGTC	edd	phosphogluconate dehydrogenase (NADP(+)-dependent, decarboxyla		WP_079293072.:	4316..5734	Pentose Phosphate/Entner-Dudorof	COG2084
316.1_1283	CUST_6569_PI442190707	HOT208_60m_826P21	316.1_1283	GCTGTGAATCAGTGGAGTGTATGAGACTTGAAGCAGGGGGAATGGCTAATATGGAATTAA	zwf	glucose-6-phosphate dehydrogenase zwf			<1..379	Pentose Phosphate	COG0364
316.1_1283	CUST_6570_PI442190707	HOT208_60m_826P21	316.1_1283	TCCCTTATCTTTGGGAAATCCAAGTATTCATTTAGGACAAGTTATTGGATTGAGGCTGAT	zwf	glucose-6-phosphate dehydrogenase zwf			<1..379	Pentose Phosphate	COG0364
316.1_1283	CUST_6571_PI442190707	HOT208_60m_826P21	316.1_1283	ACATCAGAGTACGATGTTAGTAAATTATTGGGAAGTAGTAGAGGAAATACCAGTCCTCTT	zwf	glucose-6-phosphate dehydrogenase zwf			<1..379	Pentose Phosphate	COG0364
316.1_1283	CUST_6572_PI442190707	HOT208_60m_826P21	316.1_1283	AGGGATATAGAATCAATAAAAATAGAGTTTGAAAGAATTAATGGCGTAATAATTTCTGCA	zwf	glucose-6-phosphate dehydrogenase zwf			<1..379	Pentose Phosphate	COG0364
316.1_1283	CUST_6573_PI442190707	HOT208_60m_826P21	316.1_1283	TCTTTTTGAAAATTCTATTAAAATTGCTCTTCAAATATTTAATGGTTTGAATAACT	zwf	glucose-6-phosphate dehydrogenase zwf			<1..379	Pentose Phosphate	COG0364
316.1_1283	CUST_6574_PI442190707	HOT208_60m_826P21	316.1_1283	GATTGAGGCTGATTTCAAAAATTAGTGAGGTTCAAAAAAATAACACT	zwf	glucose-6-phosphate dehydrogenase zwf			<1..379	Pentose Phosphate	COG0364
063.1_1523	CUST_7769_PI442190707	HOT208_60m_826P21	063.1_1523	AGCAATGGCGACCTTATTACACTGCAGCTAAATGAAGGTGTTATATACATGGGGCAAATT	pykF	Pyruvate kinase			1964..2413	Glycolysis	COG0469
063.1_1523	CUST_7770_PI442190707	HOT208_60m_826P21	063.1_1523	TCAAACTGCAGGCACATTAACTGGAATAAGTGGTTCTACAGATTTAATAAAAGTCGGCTT	pykF	Pyruvate kinase			1964..2413	Glycolysis	COG0469
063.1_1523	CUST_7771_PI442190707	HOT208_60m_826P21	063.1_1523	AGAAAAATTGTATCAAGAGGAATTTCAATAGGGGAAATCGGTGTAACAGGTAAAGCAAGA	pykF	Pyruvate kinase			1964..2413	Glycolysis	COG0469
063.1_1523	CUST_7772_PI442190707	HOT208_60m_826P21	063.1_1523	CCAATGAGTAAAAATATTGCGGGCATTGTTACGAATCAAAATGTAAATGATGTTTATGCT	pykF	Pyruvate kinase			1964..2413	Glycolysis	COG0469
063.1_1523	CUST_7773_PI442190707	HOT208_60m_826P21	063.1_1523	TCTAGATATATCATTAATTTGTCCAGGAGAAATATTATTTGTTCCTGAAGAATTAATGAA	pykF	Pyruvate kinase			1964..2413	Glycolysis	COG0469
063.1_1523	CUST_7774_PI442190707	HOT208_60m_826P21	063.1_1523	ACAAAAAAAATAAAAAGATTTCTACTATTTGTAATTTAGAAAATATGGATGATCATCAAA	pykF	Pyruvate kinase			1964..2413	Glycolysis	COG0469
073.1_1544	CUST_7872_PI442190707	HOT208_60m_826P21	073.1_1544	GCAAATCAGGTGGTACGCCTGAACCTAGAATTGCTATGGAAATTATACAAAGTCACTGCG	pgi	Glucose-6-phosphate isomerase			<1..769	Glycolysis/Gluconeogenesis	COG0166
073.1_1544	CUST_7873_PI442190707	HOT208_60m_826P21	073.1_1544	GGAGCATCATTAATGGATGAGGCCACACGCATAAGAGATTTTAAAAATAATCCAGCAGCA	pgi	Glucose-6-phosphate isomerase			<1..769	Glycolysis/Gluconeogenesis	COG0166
073.1_1544	CUST_7874_PI442190707	HOT208_60m_826P21	073.1_1544	GCATGATTGGGTTGGAGGAAGAACTAGTATTACAAGCTCTGTGGGATTACTTCCATTAGC	pgi	Glucose-6-phosphate isomerase			<1..769	Glycolysis/Gluconeogenesis	COG0166
073.1_1544	CUST_7875_PI442190707	HOT208_60m_826P21	073.1_1544	AGGAATTGGTGGAAGTGGTTTAGGACCATTACTTATTACAGAGTCACTACAGAAGTGCTC	pgi	Glucose-6-phosphate isomerase			<1..769	Glycolysis/Gluconeogenesis	COG0166
073.1_1544	CUST_7876_PI442190707	HOT208_60m_826P21	073.1_1544	TTGGCTTAGAAATCCATCAATTTCTCCATCTTCAAAAATAGGAGAGGAAATTAGCGCAGA	pgi	Glucose-6-phosphate isomerase			<1..769	Glycolysis/Gluconeogenesis	COG0166
073.1_1544	CUST_7877_PI442190707	HOT208_60m_826P21	073.1_1544	AAAAGAATTAGAAAATGGTGCATTCTCAAATATTGATGAAAATAGACAAGTTGGCCATTA	pgi	Glucose-6-phosphate isomerase			<1..769	Glycolysis/Gluconeogenesis	COG0166
001.1_1	CUST_1_PI442182261	HOT208_60m_813O14	001.1_1	GTAGGTGTAACACACTTCCTTGTCGGTGGTATTGCTACCACATGGGCCTTCTTC	psaA	Photosystem I P700 chlorophyll A apoprotein A1 PsaA			<1..434	Photosystem I	COG2885
001.1_1	CUST_2_PI442182261	HOT208_60m_813O14	001.1_1	AAACTTAAAGTTGCTCCAACCATTCAACCAAGAGCTTTATCTATCACCCAGGGTAGAGCG	psaA	Photosystem I P700 chlorophyll A apoprotein A1 PsaA			<1..434	Photosystem I	COG2885
001.1_1	CUST_3_PI442182261	HOT208_60m_813O14	001.1_1	AGCTCACTTCATATGGGCATTTAGTTTAATGTTCCTCTTTAGTGGAAGGGGATATTGGCA	psaA	Photosystem I P700 chlorophyll A apoprotein A1 PsaA			<1..434	Photosystem I	COG2885
001.1_1	CUST_4_PI442182261	HOT208_60m_813O14	001.1_1	TGGTTGGTTAAGAGATTACCTTTGGGCGCAAGCTTCTCAAGTACTAACAAGTTATGGTCA	psaA	Photosystem I P700 chlorophyll A apoprotein A1 PsaA			<1..434	Photosystem I	COG2885
001.1_1	CUST_5_PI442182261	HOT208_60m_813O14	001.1_1	TTTTCCACTTCTCTTGGAAAATGCAGAGTGATGTATGGGGTCTTACTGGTGGTAACTTCG	psaA	Photosystem I P700 chlorophyll A apoprotein A1 PsaA			<1..434	Photosystem I	COG2885
138.1_WP_079317390.:	CUST_332_PI442182261	HOT208_60m_813O14	138.1_WP_079317390.:	AAATTTAGACCACCAACACCTATATTGGCAACTACGACAGAGAGGAGTGTAGCGAGAAGA	pykF	Pyruvate kinase		WP_079317390.:	8778..10568	Glycolysis	COG0469
138.1_WP_079317390.:	CUST_333_PI442182261	HOT208_60m_813O14	138.1_WP_079317390.:	GCAAATTGCTCAGGAAATGGGGATCCTAAAGCAAGGAGATTTAGTAGTTCAAACAGCAGG	pykF	Pyruvate kinase		WP_079317390.:	8778..10568	Glycolysis	COG0469
138.1_WP_079317390.:	CUST_334_PI442182261	HOT208_60m_813O14	138.1_WP_079317390.:	AGCTAGACAACTTGATGCAGGAGCAATAATCCCTTTAACAAAATCAGGATCTACGGCTAG	pykF	Pyruvate kinase		WP_079317390.:	8778..10568	Glycolysis	COG0469
138.1_WP_079317390.:	CUST_335_PI442182261	HOT208_60m_813O14	138.1_WP_079317390.:	AAAGCTATTGATAGCCACTTACCAAGTACGATCCCAAATGCTATTAGTGCAGCAGTAAGT	pykF	Pyruvate kinase		WP_079317390.:	8778..10568	Glycolysis	COG0469
138.1_WP_079317390.:	CUST_336_PI442182261	HOT208_60m_813O14	138.1_WP_079317390.:	AGGAAGATTGTTTCAAGAGGAATTTCAATTGGGGAAATCGGTGTTACAGGTAAAGCAAGA	pykF	Pyruvate kinase		WP_079317390.:	8778..10568	Glycolysis	COG0469
138.1_WP_079317390.:	CUST_337_PI442182261	HOT208_60m_813O14	138.1_WP_079317390.:	TGGCGACCTAATTACATTGCAGCTTAATGAAGGTGTTATATACATGGGACAAATTGATGA	pykF	Pyruvate kinase		WP_079317390.:	8778..10568	Glycolysis	COG0469
138.1_WP_079317404.:	CUST_382_PI442182261	HOT208_60m_813O14	138.1_WP_079317404.:	AGATCAGGTGATGCACTTGCAAATCCTTACTTAGCATTTTCTGTAATGATGCTTGCTGGT	glnA	Glutamine synthetase/ glutamate--ammonialigase		WP_079317404.:	17786..19207	Nitrogen Metabolism	COG0174
138.1_WP_079317404.:	CUST_379_PI442182261	HOT208_60m_813O14	138.1_WP_079317404.:	AAACAGCAACATTTATGCCCAAGCCTGTTTTTAACGACAATGGAACTGGAATGCATGTAC	glnA	Glutamine synthetase/ glutamate--ammonialigase		WP_079317404.:	17786..19207	Nitrogen Metabolism	COG0174
138.1_WP_079317404.:	CUST_380_PI442182261	HOT208_60m_813O14	138.1_WP_079317404.:	GGTAACAGATCAGCAGCGGTAAGAATACCTTTAACAGGTCCAAGCCCTAAAGCTAAAAGA	glnA	Glutamine synthetase/ glutamate--ammonialigase		WP_079317404.:	17786..19207	Nitrogen Metabolism	COG0174
138.1_WP_079317404.:	CUST_381_PI442182261	HOT208_60m_813O14	138.1_WP_079317404.:	TCTCAAACAGCAAGATGGTATATCGGAGGCATACTTAAACATGCACCTTCATTCCTAGCA	glnA	Glutamine synthetase/ glutamate--ammonialigase		WP_079317404.:	17786..19207	Nitrogen Metabolism	COG0174
138.1_WP_079317404.:	CUST_383_PI442182261	HOT208_60m_813O14	138.1_WP_079317404.:	ACCAAAGTTTATGGAAGAGTGGACAGCCGCTATTCTTTGGTGAAGGATCATATGCAAATT	glnA	Glutamine synthetase/ glutamate--ammonialigase		WP_079317404.:	17786..19207	Nitrogen Metabolism	COG0174
138.1_WP_079317404.:	CUST_384_PI442182261	HOT208_60m_813O14	138.1_WP_079317404.:	ACTAACCCAACTACAAATAGTTATAAACGATTGGTTCCAGGATTCGAAGCACCCGTAAAT	glnA	Glutamine synthetase/ glutamate--ammonialigase		WP_079317404.:	17786..19207	Nitrogen Metabolism	COG0174
138.1_WP_025891660.:	CUST_437_PI442182261	HOT208_60m_813O14	138.1_WP_025891660.:	TGGGTGCAGCAATGACTGGCTTAAGACCAATAGTAGAAGGAATGAATATGGGTTTTTTGC	pdhB	Pyruvate dehydrogenase E1 component beta subunit		WP_025891660.:	26980..27963	Glycolysis/Krebs cycle	COG0022
138.1_WP_025891660.:	CUST_438_PI442182261	HOT208_60m_813O14	138.1_WP_025891660.:	CTTGAAGCATATTTTCATGCAGTCCCTGGCATAAAGATTGTTGCATGTAGTACACCCACA	pdhB	Pyruvate dehydrogenase E1 component beta subunit		WP_025891660.:	26980..27963	Glycolysis/Krebs cycle	COG0022
138.1_WP_025891660.:	CUST_439_PI442182261	HOT208_60m_813O14	138.1_WP_025891660.:	CCTGAGGGTGATTATACTTGCGCTTTAGATCAGGCTGACGTTGTAAAAGAAGGGAAAGAT	pdhB	Pyruvate dehydrogenase E1 component beta subunit		WP_025891660.:	26980..27963	Glycolysis/Krebs cycle	COG0022
138.1_WP_025891660.:	CUST_440_PI442182261	HOT208_60m_813O14	138.1_WP_025891660.:	GAAATTATAAAATACCAGCAGTAGTTCGAGGACCTGGAGGAGTTGGTCGTCAACTTGGTG	pdhB	Pyruvate dehydrogenase E1 component beta subunit		WP_025891660.:	26980..27963	Glycolysis/Krebs cycle	COG0022
138.1_WP_025891660.:	CUST_441_PI442182261	HOT208_60m_813O14	138.1_WP_025891660.:	GGTGCAGAATTAATTGCCTTGATAACAGAAGAGTGTTTTGATGATCTTGATGCCCGACCA	pdhB	Pyruvate dehydrogenase E1 component beta subunit		WP_025891660.:	26980..27963	Glycolysis/Krebs cycle	COG0022
138.1_WP_025891660.:	CUST_442_PI442182261	HOT208_60m_813O14	138.1_WP_025891660.:	TGAGACATCACTGCCTTAAGGCTGTAGAAGAATTAGAAAAAAAAGGAATAGATGTTGAGT	pdhB	Pyruvate dehydrogenase E1 component beta subunit		WP_025891660.:	26980..27963	Glycolysis/Krebs cycle	COG0022
142.1_WP_079317733.:	CUST_1587_PI442182261	HOT208_60m_813O14	142.1_WP_079317733.:	CGCGTATCTACAGAAGTTGATGCAAGACTGAGCTTTGACACTGAAGCTACGGTAGAAAAA	tal	Transaldolase		WP_079317733.:	35088..36089	Pentose Phosphate	COG0176
142.1_WP_079317733.:	CUST_1588_PI442182261	HOT208_60m_813O14	142.1_WP_079317733.:	AGATTGCTGCAACTTGGGAGGGAATTAAGGCAGCTGAAATTTTGGAAAAAGAGGGTATTA	tal	Transaldolase		WP_079317733.:	35088..36089	Pentose Phosphate	COG0176
142.1_WP_079317733.:	CUST_1589_PI442182261	HOT208_60m_813O14	142.1_WP_079317733.:	ACTTCTGCCAAGCGGTAACTTGTGCCAATGCAAAGATAACTCTAATTTCTCCTTTCGTTG	tal	Transaldolase		WP_079317733.:	35088..36089	Pentose Phosphate	COG0176
142.1_WP_079317733.:	CUST_1590_PI442182261	HOT208_60m_813O14	142.1_WP_079317733.:	AGAAGATCAAATGGCAAGTGAAAAACTTAGTGAGGGTATCACTGGATTCAGTAAGGCCAT	tal	Transaldolase		WP_079317733.:	35088..36089	Pentose Phosphate	COG0176
142.1_WP_079317733.:	CUST_1591_PI442182261	HOT208_60m_813O14	142.1_WP_079317733.:	TGTTGGTGCTGAAGACCCTGGTGTTATTTCGGTTACGCAAATATACAAGTACTTTAAAGA	tal	Transaldolase		WP_079317733.:	35088..36089	Pentose Phosphate	COG0176
142.1_WP_079317733.:	CUST_1592_PI442182261	HOT208_60m_813O14	142.1_WP_079317733.:	AGCAGGTTGCGATCTTTTAACAATTGCACCAAAATTTCTTGAGGAACTGAAAAAAGAAAA	tal	Transaldolase		WP_079317733.:	35088..36089	Pentose Phosphate	COG0176
143.1_WP_079317871.:	CUST_2197_PI442182261	HOT208_60m_813O14	143.1_WP_079317871.:	TCCCAGAATTAATTTAGGCTCTGCCTCCATAGTTAATAAGCAATCAATTGAAGATTCATT	eda	2-dehydro-3-deoxyphosphogluconate aldolase		WP_079317871.:	50121..50747	Entner-Dudoroff	COG0800
143.1_WP_079317871.:	CUST_2198_PI442182261	HOT208_60m_813O14	143.1_WP_079317871.:	AAATTTTTCGATGATGAAATTTTGGGATAAAGATCTTTTCAATTACGCGCAGTCAAGAAA	eda	2-dehydro-3-deoxyphosphogluconate aldolase		WP_079317871.:	50121..50747	Entner-Dudoroff	COG0800
143.1_WP_079317871.:	CUST_2199_PI442182261	HOT208_60m_813O14	143.1_WP_079317871.:	AGAATATTGAAATAAGTTGGTCTAATAACGAAAATTGGTTCGATTTTGTATCCGATATCA	eda	2-dehydro-3-deoxyphosphogluconate aldolase		WP_079317871.:	50121..50747	Entner-Dudoroff	COG0800
143.1_WP_079317871.:	CUST_2200_PI442182261	HOT208_60m_813O14	143.1_WP_079317871.:	AGATAGTTCGATAGATATACTCAACTATAAAAATATTAATTTCATTGCTGCTGGAGGACT	eda	2-dehydro-3-deoxyphosphogluconate aldolase		WP_079317871.:	50121..50747	Entner-Dudoroff	COG0800
143.1_WP_079317871.:	CUST_2201_PI442182261	HOT208_60m_813O14	143.1_WP_079317871.:	AGGGTATAAAGCAGTTGTAATTGGAGATAAAGCTATCAAAAATAAAAAATTTGATCCAAA	eda	2-dehydro-3-deoxyphosphogluconate aldolase		WP_079317871.:	50121..50747	Entner-Dudoroff	COG0800
143.1_WP_079317871.:	CUST_2202_PI442182261	HOT208_60m_813O14	143.1_WP_079317871.:	AAAGAAGATTCTTTTTCGGAGTACCTGAAAATTGAGTCTTTTTTTTTACTTATAAAACCT	eda	2-dehydro-3-deoxyphosphogluconate aldolase		WP_079317871.:	50121..50747	Entner-Dudoroff	COG0800
143.1_WP_079317893.:	CUST_2288_PI442182261	HOT208_60m_813O14	143.1_WP_079317893.:	AAGAAGCAGGGGACTTATTATTAGAAGCAATTCAAAAAGCCGGATTTAAGCCTGGTGAGC	eno	Enolase		WP_079317893.:	67345..68637	Glycolysis/Gluconeogenesis	COG0148
143.1_WP_079317893.:	CUST_2289_PI442182261	HOT208_60m_813O14	143.1_WP_079317893.:	ATTGAGGAGGAATTAGGAGATCAAGCAAGATTCGCTGGAGCTCTAGGTTTAGGTCCTAAA	eno	Enolase		WP_079317893.:	67345..68637	Glycolysis/Gluconeogenesis	COG0148
143.1_WP_079317893.:	CUST_2290_PI442182261	HOT208_60m_813O14	143.1_WP_079317893.:	CAATAGAGGACGGTTTAGCTGAGGATGATTGGGAGGGTTGGTCAGAATTAAACAAGGAAT	eno	Enolase		WP_079317893.:	67345..68637	Glycolysis/Gluconeogenesis	COG0148
143.1_WP_079317893.:	CUST_2291_PI442182261	HOT208_60m_813O14	143.1_WP_079317893.:	GTGAGACTGAAGATACAACAATTGCAGATTTATCTGTCGCCACAAGATCGGGTCAGATCA	eno	Enolase		WP_079317893.:	67345..68637	Glycolysis/Gluconeogenesis	COG0148
143.1_WP_079317893.:	CUST_2292_PI442182261	HOT208_60m_813O14	143.1_WP_079317893.:	TCATTGAAATCATTACTTGATCAAAAAGGCCTATCTACTGCTGTAGGCGATGAGGGTGGA	eno	Enolase		WP_079317893.:	67345..68637	Glycolysis/Gluconeogenesis	COG0148
143.1_WP_079317893.:	CUST_2293_PI442182261	HOT208_60m_813O14	143.1_WP_079317893.:	AAGTTCAGCTTGTAGGTGATGATTTATTCGTCACTAATACAGAAAGATTACGGAAAGGGA	eno	Enolase		WP_079317893.:	67345..68637	Glycolysis/Gluconeogenesis	COG0148
150.1_WP_025924170.:	CUST_3329_PI442182261	HOT208_60m_813O14	150.1_WP_025924170.:	TTTACCAATCAAAAGAACTATTTCAGGAGAAATAAAATTCAAAAAAACTGATAAAACTGA	minE	septum site-determining protein MinE		WP_025924170.:	4990..5325	Cell Division	COG0851
150.1_WP_025924170.:	CUST_3330_PI442182261	HOT208_60m_813O14	150.1_WP_025924170.:	GATTTTGAGGAAGTAGCAGTAAGTTTAGAGACTGAGGATAGAATGACTGCATTAGTTGCA	minE	septum site-determining protein MinE		WP_025924170.:	4990..5325	Cell Division	COG0851
150.1_WP_025924170.:	CUST_3331_PI442182261	HOT208_60m_813O14	150.1_WP_025924170.:	AGTTCCTTAACAACTGATCTTTTAGATAAAATGAGGAAAGAGATCCTTGATGTTGTTGCT	minE	septum site-determining protein MinE		WP_025924170.:	4990..5325	Cell Division	COG0851
150.1_WP_025924170.:	CUST_3332_PI442182261	HOT208_60m_813O14	150.1_WP_025924170.:	AGGCAGAGAGACGTCTAGTGCAAATACAGCGAGAGAAAGATTACAACTTGTACTTGCTCA	minE	septum site-determining protein MinE		WP_025924170.:	4990..5325	Cell Division	COG0851
150.1_WP_079318154.:	CUST_3333_PI442182261	HOT208_60m_813O14	150.1_WP_079318154.:	TAGAGGAGAGCCACTGACTCTTTCGGATGCTAGATCACCCGCAAAAAAATGTTATTTGAA	minD	septum site-determining protein MinD		WP_079318154.:	5328..6143	Cell Division	COG2894
150.1_WP_079318154.:	CUST_3334_PI442182261	HOT208_60m_813O14	150.1_WP_079318154.:	CACTTGCTAATAGCGGAGCAACCACTGCCGTATTAGATGCTGATTTTGGCTTAAGAAATT	minD	septum site-determining protein MinD		WP_079318154.:	5328..6143	Cell Division	COG2894
150.1_WP_079318154.:	CUST_3335_PI442182261	HOT208_60m_813O14	150.1_WP_079318154.:	TCTTGCAGCCTGTAAAGAAGCTATTGTTGTTACTAACCCAGAATTATCTGCAGTGCGAGA	minD	septum site-determining protein MinD		WP_079318154.:	5328..6143	Cell Division	COG2894
150.1_WP_079318154.:	CUST_3336_PI442182261	HOT208_60m_813O14	150.1_WP_079318154.:	AGCCTATCCAACTTGTAATCAACAGAGTTCGCCCTAACATGATGGCTAGTCAAGAAATGT	minD	septum site-determining protein MinD		WP_079318154.:	5328..6143	Cell Division	COG2894
150.1_WP_079318156.:	CUST_3340_PI442182261	HOT208_60m_813O14	150.1_WP_079318156.:	GCAAGACGATATTCTTCACAAAGGAACAGTACGATCGGGTGAAAGAATATCTTCAAACGG	minC	septum site-determining protein MinC		WP_079318156.:	6255..6917	Cell Division	COG0850
150.1_WP_079318154.:	CUST_3337_PI442182261	HOT208_60m_813O14	150.1_WP_079318154.:	TCTACCAGCTGGAGATCCAAGGATGTTGGATTGGATGAAGCCCGAAGATATGAAAAAAT	minD	septum site-determining protein MinD		WP_079318154.:	5328..6143	Cell Division	COG2894
150.1_WP_079318154.:	CUST_3338_PI442182261	HOT208_60m_813O14	150.1_WP_079318154.:	TCGCATCATTTATACGGCTCAAGATGTTCTAGACAAGAATTGTCGTCTTGACCAAGCATT	minD	septum site-determining protein MinD		WP_079318154.:	5328..6143	Cell Division	COG2894
150.1_WP_079318156.:	CUST_3339_PI442182261	HOT208_60m_813O14	150.1_WP_079318156.:	GCAATAGTTTCCGCTAAGAAAAACATTTACGTTTGGGGCAAATTACTGGGGATCGCATTC	minC	septum site-determining protein MinC		WP_079318156.:	6255..6917	Cell Division	COG0850
150.1_WP_079318156.:	CUST_3341_PI442182261	HOT208_60m_813O14	150.1_WP_079318156.:	AGATGTAATAGCTATAGGACCTAAGGATAAGCCCAAAAATTGTTACCCAGAAATTGCGGT	minC	septum site-determining protein MinC		WP_079318156.:	6255..6917	Cell Division	COG0850
150.1_WP_079318156.:	CUST_3342_PI442182261	HOT208_60m_813O14	150.1_WP_079318156.:	GCAGGGAAAAGCGGAAATAAAAATGCCTCTATTGCATCACTTTATCTAAATCCTCTACAG	minC	septum site-determining protein MinC		WP_079318156.:	6255..6917	Cell Division	COG0850
150.1_WP_079318156.:	CUST_3343_PI442182261	HOT208_60m_813O14	150.1_WP_079318156.:	TCCATCAAGGAACCTTTAGAAGCAAAAATTTTCGCAGTCAATGAGTCAATAAGTTCTCAT	minC	septum site-determining protein MinC		WP_079318156.:	6255..6917	Cell Division	COG0850
150.1_WP_079318156.:	CUST_3344_PI442182261	HOT208_60m_813O14	150.1_WP_079318156.:	ACAATAGTGAGTGGAAATTCAATAAAAATAGATTCAGCCTACATAAAAGAGCAAGAGATT	minC	septum site-determining protein MinC		WP_079318156.:	6255..6917	Cell Division	COG0850
151.1_WP_079318162.:	CUST_3369_PI442182261	HOT208_60m_813O14	151.1_WP_079318162.:	AACAATGGTTAAAGGGGTGCCCCTTGCATATAACAAGGATTTTCAAGAGGATAAAGAGCC	argH	Argininosuccinate lyase		WP_079318162.:	444..1823	Amino Acid metabolism	COG0165
151.1_WP_079318162.:	CUST_3370_PI442182261	HOT208_60m_813O14	151.1_WP_079318162.:	CAGATAAATGTGCTACTGGTAGTAGCTAATGCCGCAGAAAAAAAATCCCGACGTTCCAG	argH	Argininosuccinate lyase		WP_079318162.:	444..1823	Amino Acid metabolism	COG0165
151.1_WP_079318162.:	CUST_3371_PI442182261	HOT208_60m_813O14	151.1_WP_079318162.:	AGAACTAGTGAAGGTGGAACAGGTTTTATGCAAGTAGAGAATGAGGTGAAAAACTGGCAG	argH	Argininosuccinate lyase		WP_079318162.:	444..1823	Amino Acid metabolism	COG0165
151.1_WP_079318162.:	CUST_3372_PI442182261	HOT208_60m_813O14	151.1_WP_079318162.:	TGCAGATTATTTAGTTGGTAAAAAGGTTCCGTTTAGAACCGCTTATCAAGTTGTGGGTGA	argH	Argininosuccinate lyase		WP_079318162.:	444..1823	Amino Acid metabolism	COG0165
151.1_WP_079318162.:	CUST_3373_PI442182261	HOT208_60m_813O14	151.1_WP_079318162.:	TGCAGTGAGTGATAGAGATTTTTGTATAGAGTTCGTTTCTGCATCTGCTTTAGCAATGTC	argH	Argininosuccinate lyase		WP_079318162.:	444..1823	Amino Acid metabolism	COG0165
151.1_WP_079318162.:	CUST_3374_PI442182261	HOT208_60m_813O14	151.1_WP_079318162.:	CATCCCGAATTTGATGAAGATGTTTTTGTTGATCTTGAACCTCTTAATGTAGTTAGGTCA	argH	Argininosuccinate lyase		WP_079318162.:	444..1823	Amino Acid metabolism	COG0165
153.1_WP_079318180.:	CUST_3466_PI442182261	HOT208_60m_813O14	153.1_WP_079318180.:	GTGATTCGGTTCTAGAGGACACTCTTGTTATGGGAGCCGATTTCTTTGAATCACCTGAAG	glgC	Glucose-1-phosphate adenylyltransferase		WP_079318180.:	3324..4619	Glycogen Metabolism	COG0448
153.1_WP_079318180.:	CUST_3467_PI442182261	HOT208_60m_813O14	153.1_WP_079318180.:	CGATGATTATTGGGAAGATATCGGTACCATTGGGGCATTCTTTGAGTCAAACCTTGCATT	glgC	Glucose-1-phosphate adenylyltransferase		WP_079318180.:	3324..4619	Glycogen Metabolism	COG0448
153.1_WP_079318180.:	CUST_3468_PI442182261	HOT208_60m_813O14	153.1_WP_079318180.:	CGGAACGCCTCTTGGAGTAGGTGAAGGAACTACTGTAAAAAGAGCAATCCTTGATAAGAA	glgC	Glucose-1-phosphate adenylyltransferase		WP_079318180.:	3324..4619	Glycogen Metabolism	COG0448
153.1_WP_079318180.:	CUST_3469_PI442182261	HOT208_60m_813O14	153.1_WP_079318180.:	AGTGAAAAGCCTACTGGAGAGAAGTTGAAGGCAATGGCAGTAGATACTTCAAAATTTGGA	glgC	Glucose-1-phosphate adenylyltransferase		WP_079318180.:	3324..4619	Glycogen Metabolism	COG0448
153.1_WP_079318180.:	CUST_3470_PI442182261	HOT208_60m_813O14	153.1_WP_079318180.:	ACGGATTTTGGTAAGGACATAATTCCAGAAGCGCTGAATAGAGGCGATACTCTTAAAAGT	glgC	Glucose-1-phosphate adenylyltransferase		WP_079318180.:	3324..4619	Glycogen Metabolism	COG0448
153.1_WP_079318180.:	CUST_3471_PI442182261	HOT208_60m_813O14	153.1_WP_079318180.:	AGGAGTCAGCTGCTGAAAAACCATATCTAGCCTCTATGGGTATTTACGTTTTTAGCAGAA	glgC	Glucose-1-phosphate adenylyltransferase		WP_079318180.:	3324..4619	Glycogen Metabolism	COG0448
153.1_670	CUST_3472_PI442182261	HOT208_60m_813O14	153.1_670	CAAGCTAGAGAGACCTAGAAGAATTCTAATGATGGTAAAAGCTGGACCCGCAACTGATGC	edd	phosphogluconate dehydrogenase (NADP(+)-dependent, decarboxylating)			4715..5404	Pentose Phosphate/Entner-Dudorof	COG2084
153.1_670	CUST_3473_PI442182261	HOT208_60m_813O14	153.1_670	ATGTGTTGCATATGTTGGACCAGGAGGCTCAGGTCATTTTGTAAAACTGTTCATAACGG	edd	phosphogluconate dehydrogenase (NADP(+)-dependent, decarboxylating)			4715..5404	Pentose Phosphate/Entner-Dudorof	COG2084
153.1_670	CUST_3477_PI442182261	HOT208_60m_813O14	153.1_670	AGGCACATTTTGGTTTAATAGGTCTTGGTGTTATGGGCGAAAACTTAGTTCTTAACGCAG	edd	phosphogluconate dehydrogenase (NADP(+)-dependent, decarboxylating)			4715..5404	Pentose Phosphate/Entner-Dudorof	COG2084
153.1_670	CUST_3474_PI442182261	HOT208_60m_813O14	153.1_670	AAAAGTTTTGGATACATTGGTATGGGAGTTTCTGGAGGTGCTAAAGGAGCTCTAGAAGGC	edd	phosphogluconate dehydrogenase (NADP(+)-dependent, decarboxylating)			4715..5404	Pentose Phosphate/Entner-Dudorof	COG2084
153.1_670	CUST_3475_PI442182261	HOT208_60m_813O14	153.1_670	TGCAGAAGCTTATGACCTTATGAAGAGAGTCAAAGGCATGAATGGTAAGCAGATGTCAGA	edd	phosphogluconate dehydrogenase (NADP(+)-dependent, decarboxylating)			4715..5404	Pentose Phosphate/Entner-Dudorof	COG2084
153.1_670	CUST_3476_PI442182261	HOT208_60m_813O14	153.1_670	GCCTGGCGGGACTAAGGCTTCATATGATGCAATAGAAAACTTATTAACAAAAATGGCTGC	edd	phosphogluconate dehydrogenase (NADP(+)-dependent, decarboxylating)			4715..5404	Pentose Phosphate/Entner-Dudorof	COG2084
155.1_676	CUST_3502_PI442182261	HOT208_60m_813O14	155.1_676	TTTATGGAATGTTTCATTCTCCTATGGAAGAGCATTGCAGCACTCATGCTTAAAGGCCTG	fda/cbbA	fructose-1,6-bisphosphatealdolase			284..1348	Glycolysis/Gluconeogenesis	COG0191
155.1_676	CUST_3503_PI442182261	HOT208_60m_813O14	155.1_676	TGAAAGAGCAGCAAAATATTATGAACAAGGTGCCAGATTTGCAAAGTGGAGAGCAGTTCT	fda/cbbA	fructose-1,6-bisphosphatealdolase			284..1348	Glycolysis/Gluconeogenesis	COG0191
155.1_676	CUST_3504_PI442182261	HOT208_60m_813O14	155.1_676	GGTATTATTCCAGGTATAAAGGTCGATAAAGGCCTAAATCCACTTCCTGGAGGAGGAGGT	fda/cbbA	fructose-1,6-bisphosphatealdolase			284..1348	Glycolysis/Gluconeogenesis	COG0191
155.1_676	CUST_3505_PI442182261	HOT208_60m_813O14	155.1_676	CAAGAGCTCAAGCAAACTCTGAGCTTCAAAAGGTACCTATGTAGCAGGATCTCAACCTT	fda/cbbA	fructose-1,6-bisphosphatealdolase			284..1348	Glycolysis/Gluconeogenesis	COG0191
155.1_676	CUST_3506_PI442182261	HOT208_60m_813O14	155.1_676	ACAATGGAAAGATGCGTTCCTGCATCTGTTCCTGGAATAGTTTTCTTATCAGGAGGTTA	fda/cbbA	fructose-1,6-bisphosphatealdolase			284..1348	Glycolysis/Gluconeogenesis	COG0191
155.1_676	CUST_3507_PI442182261	HOT208_60m_813O14	155.1_676	TCAAGAGAATGCTTGGGGATTAGCAAGGTATGCAAGATCAGTTCAAGAATCTGGATTGGT	fda/cbbA	fructose-1,6-bisphosphatealdolase			284..1348	Glycolysis/Gluconeogenesis	COG0191
155.1_WP_025881193.:	CUST_3525_PI442182261	HOT208_60m_813O14	155.1_WP_025881193.:	ACTCCTGATGAGAGCTTTGTGATCATACATTTCAGAAAAGGTGCAAGAGAGAAATGGGGT	prk	Phosphoribulokinase		WP_025881193.:	3967..4863	Carbon Fixation	COG3954
155.1_WP_025881193.:	CUST_3529_PI442182261	HOT208_60m_813O14	155.1_WP_025881193.:	CATGTCAAGTCCAACAAGTATCGTTGTAAATGGTGGAAAAATGGGTTTCGCAATGGAACT	prk	Phosphoribulokinase		WP_025881193.:	3967..4863	Carbon Fixation	COG3954
155.1_WP_025881193.:	CUST_3526_PI442182261	HOT208_60m_813O14	155.1_WP_025881193.:	AACGCAGAAAGAGGTTACTCAGCGGAAACCATTGTGGATACGATATTAAGAAGAATGCCT	prk	Phosphoribulokinase		WP_025881193.:	3967..4863	Carbon Fixation	COG3954
155.1_WP_025881193.:	CUST_3527_PI442182261	HOT208_60m_813O14	155.1_WP_025881193.:	GAGCCTGGGCAATTTACTCCATGGGAAGATATTCCTGAGGGAACAGACGTACTTTTTTAT	prk	Phosphoribulokinase		WP_025881193.:	3967..4863	Carbon Fixation	COG3954
155.1_WP_025881193.:	CUST_3528_PI442182261	HOT208_60m_813O14	155.1_WP_025881193.:	GTGATGGATACAATGTGGCATCCTATGCTGATTTACTTGTTGGCGTTGTTCCGATAACAA	prk	Phosphoribulokinase		WP_025881193.:	3967..4863	Carbon Fixation	COG3954
155.1_WP_025881193.:	CUST_3530_PI442182261	HOT208_60m_813O14	155.1_WP_025881193.:	CCCGCAGTTGTAGAAGGTGATAGTTACCACAGATTTGAAAGAATGCCTATGAAAAAAGCG	prk	Phosphoribulokinase		WP_025881193.:	3967..4863	Carbon Fixation	COG3954
159.1_WP_079318323.:	CUST_3993_PI442182261	HOT208_60m_813O14	159.1_WP_079318323.:	CCATTGCAATTATTGAGTTATCATATAGCAGCCCATAGAGGACTTGACGTGGATCAGCCT	glmS	Glutamine--fructose-6-phosphatetransaminase(isomerizing)		WP_079318323.:	12639..14534	Amino Acid metabolism	COG0449
159.1_WP_079318323.:	CUST_3994_PI442182261	HOT208_60m_813O14	159.1_WP_079318323.:	[illegible]	glmS	Glutamine--fructose-6-phosphatetransaminase(isomerizing)		WP_079318323.:	12639..14534	Amino Acid metabolism	COG0449
159.1_WP_079318323.:	CUST_3995_PI442182261	HOT208_60m_813O14	159.1_WP_079318323.:	[illegible]	glmS	Glutamine--fructose-6-phosphatetransaminase(isomerizing)		WP_079318323.:	12639..14534	Amino Acid metabolism	COG0449
159.1_WP_079318323.:	CUST_3996_PI442182261	HOT208_60m_813O14	159.1_WP_079318323.:	[illegible]	glmS	Glutamine--fructose-6-phosphatetransaminase(isomerizing)		WP_079318323.:	12639..14534	Amino Acid metabolism	COG0449
159.1_WP_079318323.:	CUST_3997_PI442182261	HOT208_60m_813O14	159.1_WP_079318323.:	[illegible]	glmS	Glutamine--fructose-6-phosphatetransaminase(isomerizing)		WP_079318323.:	12639..14534	Amino Acid metabolism	COG0449
159.1_WP_079318323.:	CUST_3998_PI442182261	HOT208_60m_813O14	159.1_WP_079318323.:	[illegible]	glmS	Glutamine--fructose-6-phosphatetransaminase(isomerizing)		WP_079318323.:	12639..14534	Amino Acid metabolism	COG0449
182.1_873	CUST_4517_PI442182261	HOT208_60m_813O14	182.1_873	[illegible]	psaA	Photosystem I P700 chlorophyll A apoprotein A1 PsaA			<1..482	Photosystem I	COG2885
182.1_873	CUST_4519_PI442182261	HOT208_60m_813O14	182.1_873	[illegible]	psaA	Photosystem I P700 chlorophyll A apoprotein A1 PsaA			<1..482	Photosystem I	COG2885
182.1_873	CUST_4518_PI442182261	HOT208_60m_813O14	182.1_873	[illegible]	psaA	Photosystem I P700 chlorophyll A apoprotein A1 PsaA			<1..482	Photosystem I	COG2885
182.1_873	CUST_4520_PI442182261	HOT208_60m_813O14	182.1_873	[illegible]	psaA	Photosystem I P700 chlorophyll A apoprotein A1 PsaA			<1..482	Photosystem I	COG2885
182.1_873	CUST_4521_PI442182261	HOT208_60m_813O14	182.1_873	[illegible]	psaA	Photosystem I P700 chlorophyll A apoprotein A1 PsaA			<1..482	Photosystem I	COG2885
182.1_873	CUST_4522_PI442182261	HOT208_60m_813O14	182.1_873	[illegible]	psaA	Photosystem I P700 chlorophyll A apoprotein A1 PsaA			<1..482	Photosystem I	COG2885
019.1_WP_079316837.:	CUST_4669_PI442182261	HOT208_60m_813O14	019.1_WP_079316837.:	[illegible]	rbsK	Sugar kinase, ribokinase family		WP_079316837.:	8726..9595	Pentose Phosphate	COG0524
019.1_WP_079316837.:	CUST_4667_PI442182261	HOT208_60m_813O14	019.1_WP_079316837.:	[illegible]	rbsK	Sugar kinase, ribokinase family		WP_079316837.:	8726..9595	Pentose Phosphate	COG0524
019.1_WP_079316837.:	CUST_4670_PI442182261	HOT208_60m_813O14	019.1_WP_079316837.:	[illegible]	rbsK	Sugar kinase, ribokinase family		WP_079316837.:	8726..9595	Pentose Phosphate	COG0524
019.1_WP_079316837.:	CUST_4671_PI442182261	HOT208_60m_813O14	019.1_WP_079316837.:	[illegible]	rbsK	Sugar kinase, ribokinase family		WP_079316837.:	8726..9595	Pentose Phosphate	COG0524
019.1_WP_079316837.:	CUST_4672_PI442182261	HOT208_60m_813O14	019.1_WP_079316837.:	[illegible]	rbsK	Sugar kinase, ribokinase family		WP_079316837.:	8726..9595	Pentose Phosphate	COG0524
188.1_WP_079318522.:	CUST_4926_PI442182261	HOT208_60m_813O14	188.1_WP_079318522.:	[illegible]	ppc	Phosphoenolpyruvate carboxylase		WP_079318522.:	30098..30475	Krebs cycle and related genes	COG2352
188.1_WP_079318522.:	CUST_4930_PI442182261	HOT208_60m_813O14	188.1_WP_079318522.:	[illegible]	ppc	Phosphoenolpyruvate carboxylase		WP_079318522.:	30098..30475	Krebs cycle and related genes	COG2352
188.1_WP_079318522.:	CUST_4928_PI442182261	HOT208_60m_813O14	188.1_WP_079318522.:	[illegible]	ppc	Phosphoenolpyruvate carboxylase		WP_079318522.:	30098..30475	Krebs cycle and related genes	COG2352
188.1_WP_079318522.:	CUST_4931_PI442182261	HOT208_60m_813O14	188.1_WP_079318522.:	[illegible]	ppc	Phosphoenolpyruvate carboxylase		WP_079318522.:	30098..30475	Krebs cycle and related genes	COG2352
188.1_WP_079318675.:	CUST_5247_PI442182261	HOT208_60m_813O14	188.1_WP_079318675.:	[illegible]	glcH	GPH family sugar transporter		WP_079318675.:	82517..83863	GlcH sugar transporter	COG2211
188.1_WP_079318675.:	CUST_5244_PI442182261	HOT208_60m_813O14	188.1_WP_079318675.:	[illegible]	glcH	GPH family sugar transporter		WP_079318675.:	82517..83863	GlcH sugar transporter	COG2211
188.1_WP_079318675.:	CUST_5245_PI442182261	HOT208_60m_813O14	188.1_WP_079318675.:	[illegible]	glcH	GPH family sugar transporter		WP_079318675.:	82517..83863	GlcH sugar transporter	COG2211
188.1_WP_079318675.:	CUST_5246_PI442182261	HOT208_60m_813O14	188.1_WP_079318675.:	[illegible]	glcH	GPH family sugar transporter		WP_079318675.:	82517..83863	GlcH sugar transporter	COG2211
188.1_WP_079318675.:	CUST_5248_PI442182261	HOT208_60m_813O14	188.1_WP_079318675.:	[illegible]	glcH	GPH family sugar transporter		WP_079318675.:	82517..83863	GlcH sugar transporter	COG2211
188.1_WP_079318675.:	CUST_5249_PI442182261	HOT208_60m_813O14	188.1_WP_079318675.:	[illegible]	glcH	GPH family sugar transporter		WP_079318675.:	82517..83863	GlcH sugar transporter	COG2211
188.1_WP_079318605.:	CUST_5320_PI442182261	HOT208_60m_813O14	188.1_WP_079318605.:	[illegible]	ftsZ	cell division protein FtsZ		WP_079318605.:	95346..96461	Cell Division	COG0206
188.1_WP_079318605.:	CUST_5321_PI442182261	HOT208_60m_813O14	188.1_WP_079318605.:	[illegible]	ftsZ	cell division protein FtsZ		WP_079318605.:	95346..96461	Cell Division	COG0206
188.1_WP_079318605.:	CUST_5322_PI442182261	HOT208_60m_813O14	188.1_WP_079318605.:	[illegible]	ftsZ	cell division protein FtsZ		WP_079318605.:	95346..96461	Cell Division	COG0206
188.1_WP_079318605.:	CUST_5323_PI442182261	HOT208_60m_813O14	188.1_WP_079318605.:	[illegible]	ftsZ	cell division protein FtsZ		WP_079318605.:	95346..96461	Cell Division	COG0206
188.1_WP_079318605.:	CUST_5324_PI442182261	HOT208_60m_813O14	188.1_WP_079318605.:	[illegible]	ftsZ	cell division protein FtsZ		WP_079318605.:	95346..96461	Cell Division	COG0206

188.1_WP_079318605.:	CUST_5325_PI442182261	HOT208_60m_813O14	188.1_WP_079318605.:	CTATAGAGGCAGCCCAGGCGGCAATGAATAGTCCTTTATTAGAAGCAGCTAGAATTGATG	ftsZ	cell division protein FtsZ	WP_079318605.: 95346..96461	Cell Division	COG0206
188.1_WP_079318627.:	CUST_5443_PI442182261	HOT208_60m_813O14	188.1_WP_079318627.:	TAATAGTAGTGATTCAGTAACTGCAGCATTCTTTGGAGACGGGACTTGCAATAATGGGCA	pdhA	Pyruvate dehydrogenase E1 component alpha subunit	WP_079318627.: 118582..119655	Glycolysis/Krebs cycle	COG1071
188.1_WP_079318627.:	CUST_5444_PI442182261	HOT208_60m_813O14	188.1_WP_079318627.:	AGGGGGGTCCATGCACTTATTTTCAAGAGAGCATCACTTACTTGGAGGATATGCATTTAT	pdhA	Pyruvate dehydrogenase E1 component alpha subunit	WP_079318627.: 118582..119655	Glycolysis/Krebs cycle	COG1071
188.1_WP_079318627.:	CUST_5445_PI442182261	HOT208_60m_813O14	188.1_WP_079318627.:	AGAAGAAGTTGATGGAATGGATGTTTTAGCGGTTAGAGGAGCAGCACAAAGAGCAATAGA	pdhA	Pyruvate dehydrogenase E1 component alpha subunit	WP_079318627.: 118582..119655	Glycolysis/Krebs cycle	COG1071
188.1_WP_079318627.:	CUST_5446_PI442182261	HOT208_60m_813O14	188.1_WP_079318627.:	GTGAAGGGCCCACTCTTTTAGAATGCTTAACTTACCGATATAGGGGACATTCTCTTGCAG	pdhA	Pyruvate dehydrogenase E1 component alpha subunit	WP_079318627.: 118582..119655	Glycolysis/Krebs cycle	COG1071
188.1_WP_079318627.:	CUST_5447_PI442182261	HOT208_60m_813O14	188.1_WP_079318627.:	TAAATGGGCTATTGGTATGGCTCACGATAGAGCTACTAGCAATCCTGAGATCTGGAGAAA	pdhA	Pyruvate dehydrogenase E1 component alpha subunit	WP_079318627.: 118582..119655	Glycolysis/Krebs cycle	COG1071
188.1_WP_079318627.:	CUST_5448_PI442182261	HOT208_60m_813O14	188.1_WP_079318627.:	GGGAATTCCGGTTGCTTTAGGTGCAGCCTATTCAAGTAAATATAAAAAGGAAGTTGCTGG	pdhA	Pyruvate dehydrogenase E1 component alpha subunit	WP_079318627.: 118582..119655	Glycolysis/Krebs cycle	COG1071
021.1_WP_079316912.:	CUST_6344_PI442182261	HOT208_60m_813O14	021.1_WP_079316912.:	TCAAGTGATGCAGGAAGTTACATTACAGGACAAACAATCAGTATAGACGGGGGTATGAGT	gdh	3-oxoacyl-acyl-carrier-protein reductase	WP_079316912.: 1054..1803	Entner-Dudoroff	COG1028
021.1_WP_079316912.:	CUST_6342_PI442182261	HOT208_60m_813O14	021.1_WP_079316912.:	TGGTAATCCTGGTCAAGCAAATTATTCTGCAGCAAAAGCGGGAGTTATTGGATTTACCAA	gdh	3-oxoacyl-acyl-carrier-protein reductase	WP_079316912.: 1054..1803	Entner-Dudoroff	COG1028
021.1_WP_079316912.:	CUST_6343_PI442182261	HOT208_60m_813O14	021.1_WP_079316912.:	AGCAGGGGAATTGGTAAAGAAATTGCTTTAGAACTAAGCCGATTGGGAGCAGAAGTTTTT	gdh	3-oxoacyl-acyl-carrier-protein reductase	WP_079316912.: 1054..1803	Entner-Dudoroff	COG1028
021.1_WP_079316912.:	CUST_6345_PI442182261	HOT208_60m_813O14	021.1_WP_079316912.:	AACTTGCGCTAAAGAATTTGCATCAAGAGGTATAAATGTTAATGCGATAGCTCCAGGTTT	gdh	3-oxoacyl-acyl-carrier-protein reductase	WP_079316912.: 1054..1803	Entner-Dudoroff	COG1028
021.1_WP_079316912.:	CUST_6346_PI442182261	HOT208_60m_813O14	021.1_WP_079316912.:	TGCTGGAATTACTAGAGATGGACTATTGATGAGAATGAAATCGGAACAATGGGATGATGT	gdh	3-oxoacyl-acyl-carrier-protein reductase	WP_079316912.: 1054..1803	Entner-Dudoroff	COG1028
021.1_WP_079316912.:	CUST_6347_PI442182261	HOT208_60m_813O14	021.1_WP_079316912.:	TTTCAAAAGAGGATTCTGTCAGTTCAGCTTTTGAAGAAATAATCAAAATTAATGGCACCA	gdh	3-oxoacyl-acyl-carrier-protein reductase	WP_079316912.: 1054..1803	Entner-Dudoroff	COG1028
021.1_WP_079316924.:	CUST_6385_PI442182261	HOT208_60m_813O14	021.1_WP_079316924.:	GGAAAATATCCGATAATTTGCGCAGAATTATGTGGCCCATATCATGGAGGAATGAGAGCC	ctaC/coxB	Cytochrome c oxidase subunit II	WP_079316924.: 8418..9221	Respiration	COG1622
021.1_WP_079316924.:	CUST_6386_PI442182261	HOT208_60m_813O14	021.1_WP_079316924.:	TATTCATGCTTTTTGGGTGCCAGAGTTCAGAATTAAACAGGATGTTATTCCTGGGCAACC	ctaC/coxB	Cytochrome c oxidase subunit II	WP_079316924.: 8418..9221	Respiration	COG1622
021.1_WP_079316924.:	CUST_6387_PI442182261	HOT208_60m_813O14	021.1_WP_079316924.:	TCTTTTTGGATTGGTTTTAATGTAAATTTGCTGCCAGCGGAAGCAAGTATTAATGCACCA	ctaC/coxB	Cytochrome c oxidase subunit II	WP_079316924.: 8418..9221	Respiration	COG1622
021.1_WP_079316924.:	CUST_6388_PI442182261	HOT208_60m_813O14	021.1_WP_079316924.:	TCCCAAGGGCAATTTCATATCAGGAGAACTACACGTTCCTGTTGATCAAAAAGTATCAAT	ctaC/coxB	Cytochrome c oxidase subunit II	WP_079316924.: 8418..9221	Respiration	COG1622
021.1_WP_079316924.:	CUST_6389_PI442182261	HOT208_60m_813O14	021.1_WP_079316924.:	AGCTACAACATCTACGATCGAATGGGAGGGATGAAAGAACTAAATCATAACCACGAAATG	ctaC/coxB	Cytochrome c oxidase subunit II	WP_079316924.: 8418..9221	Respiration	COG1622
021.1_WP_079316924.:	CUST_6390_PI442182261	HOT208_60m_813O14	021.1_WP_079316924.:	AGTTCTAACACTGAAAAAATATGGGCTGGAATAAGTCAAACTTCCGATAATGAAATGGCA	ctaC/coxB	Cytochrome c oxidase subunit II	WP_079316924.: 8418..9221	Respiration	COG1622
021.1_WP_079316926.:	CUST_6391_PI442182261	HOT208_60m_813O14	021.1_WP_079316926.:	ACATCTTCTCCACCTCCAATTGAAAATTGGGAAGGAGAAGCTCCATTAGTTGAAGAACCT	cyoB/coxA	Cytochrome c oxidase subunit I	WP_079316926.: 9218..10831	Respiration	COG0843
021.1_WP_079316926.:	CUST_6395_PI442182261	HOT208_60m_813O14	021.1_WP_079316926.:	ATCACATGTTTACAAGTGGAACACCCCCTTGGATGAGATTGTTCTTTACAATTGCCACAG	cyoB/coxA	Cytochrome c oxidase subunit I	WP_079316926.: 9218..10831	Respiration	COG0843
021.1_WP_079316926.:	CUST_6392_PI442182261	HOT208_60m_813O14	021.1_WP_079316926.:	TTTCTTAGTGTGCGAAATGGAAAAGATGCTGGAGATAACCCTTGGAATGCTCTTACACCT	cyoB/coxA	Cytochrome c oxidase subunit I	WP_079316926.: 9218..10831	Respiration	COG0843
021.1_WP_079316926.:	CUST_6393_PI442182261	HOT208_60m_813O14	021.1_WP_079316926.:	GCTTTGCTCCTCAACATTGGCTTGGTTTAAATGGAATGCCAAGAAGAGTTGCAGAATATG	cyoB/coxA	Cytochrome c oxidase subunit I	WP_079316926.: 9218..10831	Respiration	COG0843
021.1_WP_079316926.:	CUST_6394_PI442182261	HOT208_60m_813O14	021.1_WP_079316926.:	TGCTTTGGCACAGGTACCTTTCGATATTCATGTACATGATACATATTTCGTTGTTGCCCA	cyoB/coxA	Cytochrome c oxidase subunit I	WP_079316926.: 9218..10831	Respiration	COG0843
021.1_WP_079316926.:	CUST_6396_PI442182261	HOT208_60m_813O14	021.1_WP_079316926.:	TGGTCCTTCCCGCCTTTGGTTTAGTAAGTGAAATACTTCCTATACATGCTAGAAAACCAC	cyoB/coxA	Cytochrome c oxidase subunit I	WP_079316926.: 9218..10831	Respiration	COG0843
021.1_WP_079316929.:	CUST_6397_PI442182261	HOT208_60m_813O14	021.1_WP_079316929.:	TCGAGAATCAAGGGCGGAAGATTAACTAGTCAAAATATGTTCCCCTTGGAAGCTGTTGAA	cyoC	heme-copper oxidase subunit III	WP_079316929.: 10848..11450	Respiration	COG1845
021.1_WP_079316929.:	CUST_6398_PI442182261	HOT208_60m_813O14	021.1_WP_079316929.:	AGTGCAACTTTCCACAAAGCAGGCAAAGCACTTTTAAAAGATAAAAACTCTGAATCCCAA	cyoC	heme-copper oxidase subunit III	WP_079316929.: 10848..11450	Respiration	COG1845
021.1_WP_079316929.:	CUST_6399_PI442182261	HOT208_60m_813O14	021.1_WP_079316929.:	TTTGGTCTTATAACTTTCCTAATTGCAGACGGAATGACTTTTGCTGGATTTTTTGCTGCA	cyoC	heme-copper oxidase subunit III	WP_079316929.: 10848..11450	Respiration	COG1845
021.1_WP_079316929.:	CUST_6400_PI442182261	HOT208_60m_813O14	021.1_WP_079316929.:	TGCAAGTACTTTTTATGCTCTTACTGGTTTTCATGGATTACATGTCACTTTAGGCACTTT	cyoC	heme-copper oxidase subunit III	WP_079316929.: 10848..11450	Respiration	COG1845
021.1_WP_079316929.:	CUST_6401_PI442182261	HOT208_60m_813O14	021.1_WP_079316929.:	AGCAGTAAATCCATTACCTGATGGTGCTATTTATGAATTAGAACTACCAATACCTACACT	cyoC	heme-copper oxidase subunit III	WP_079316929.: 10848..11450	Respiration	COG1845
021.1_WP_079316929.:	CUST_6402_PI442182261	HOT208_60m_813O14	021.1_WP_079316929.:	AGCTCAAAAGAAATTCAAAAAAATAATTCTGAAGTTAACGAAACACATGAAGACTTCAGA	cyoC	heme-copper oxidase subunit III	WP_079316929.: 10848..11450	Respiration	COG1845
021.1_WP_079316985.:	CUST_6582_PI442182261	HOT208_60m_813O14	021.1_WP_079316985.:	TGGCAATTGATTCCCTATCTGCATTAGCCAGAGGTGTAAGTTTGAATGCATTTAGACAGT	kaiC	circadian clock protein KaiC	WP_079316985.: 43050..44579	Circadian Rhythm	COG0467
021.1_WP_079316985.:	CUST_6580_PI442182261	HOT208_60m_813O14	021.1_WP_079316985.:	GCGCTACAGGGACAGGCAAGACAATGCTCGTATCAAAATTTGTTGAAGATGCTTATAACA	kaiC	circadian clock protein KaiC	WP_079316985.: 43050..44579	Circadian Rhythm	COG0467
021.1_WP_079316985.:	CUST_6581_PI442182261	HOT208_60m_813O14	021.1_WP_079316985.:	ACTTTTTGCATATGAGGAATCTAGGGCTCAATTACTGAGAAATGCCACTAGTTGGGGGAT	kaiC	circadian clock protein KaiC	WP_079316985.: 43050..44579	Circadian Rhythm	COG0467
021.1_WP_079316985.:	CUST_6583_PI442182261	HOT208_60m_813O14	021.1_WP_079316985.:	AGGTGAGATGGCTAGAGCTTTAAATGTTTTTAAAATGCGTGGATCATGGCATGATAAACG	kaiC	circadian clock protein KaiC	WP_079316985.: 43050..44579	Circadian Rhythm	COG0467
021.1_WP_079316985.:	CUST_6584_PI442182261	HOT208_60m_813O14	021.1_WP_079316985.:	TCCTCCAATATTAGGATTAGCTCTGGAGTTAAAGATCTTGATGATATGTGTGGTGGTGGA	kaiC	circadian clock protein KaiC	WP_079316985.: 43050..44579	Circadian Rhythm	COG0467
021.1_WP_079316985.:	CUST_6585_PI442182261	HOT208_60m_813O14	021.1_WP_079316985.:	TTTGCGCATATCCTGAATCAACAGGTTTAGAAGATCACTTGCAAATCATAAAAACGCAAA	kaiC	circadian clock protein KaiC	WP_079316985.: 43050..44579	Circadian Rhythm	COG0467
021.1_WP_079316987.:	CUST_6586_PI442182261	HOT208_60m_813O14	021.1_WP_079316987.:	CCCCACCGGTTAGAAAAATAATTGGTGACCTATCTGATAGAGAGAAAGTT	kaiB	Circadian clock protein KaiB	WP_079316987.: 44650..44967	Circadian Rhythm	COG4251
021.1_WP_079316987.:	CUST_6587_PI442182261	HOT208_60m_813O14	021.1_WP_079316987.:	TGCAGAGGAAGATAAGATTTTAGCTACCCCAACTTTAGCTAAAATATTACCCCCACCGGT	kaiB	Circadian clock protein KaiB	WP_079316987.: 44650..44967	Circadian Rhythm	COG4251
021.1_WP_079316987.:	CUST_6588_PI442182261	HOT208_60m_813O14	021.1_WP_079316987.:	AAATGAATTTAAAGGAGTATATGCGTTAAAGGTTATTGATGTACTTAAGCAACCACAACT	kaiB	Circadian clock protein KaiB	WP_079316987.: 44650..44967	Circadian Rhythm	COG4251
021.1_WP_079316987.:	CUST_6589_PI442182261	HOT208_60m_813O14	021.1_WP_079316987.:	AACTCTATGTCGCTGGAAATACTCCTAATTCAATGAGAGCCTTAAATACCTTAAAAGAAA	kaiB	Circadian clock protein KaiB	WP_079316987.: 44650..44967	Circadian Rhythm	COG4251
216.1_1305	CUST_6819_PI442182261	HOT208_60m_813O14	216.1_1305	GGTGCATACAAATTCACAAGGAAGCCTACAGGAGAAGTTCTTGCAATAAGCAGAATTGCT	fda/cbbA	fructose-1,6-bisphosphatealdolase	<1..978	Glycolysis/Gluconeogenesis	COG0191
216.1_1305	CUST_6820_PI442182261	HOT208_60m_813O14	216.1_1305	TATGAAGCAGGTTTGTCTTGATAGATACAAGCAGTTCTGGTGCGAAGGTCAAGCAAGTAA	fda/cbbA	fructose-1,6-bisphosphatealdolase	<1..978	Glycolysis/Gluconeogenesis	COG0191
216.1_1305	CUST_6824_PI442182261	HOT208_60m_813O14	216.1_1305	CCAAATACACACCTTGTAGTGCATGGATCTAGTTCAGTCCCTCAGGAATGGTTGGATATC	fda/cbbA	fructose-1,6-bisphosphatealdolase	<1..978	Glycolysis/Gluconeogenesis	COG0191
216.1_1305	CUST_6821_PI442182261	HOT208_60m_813O14	216.1_1305	GTTAGTGTTGAAGGAGAGTTGGGTTGCTTAGGTTCATTAGAGACAGAAAAGGTGAGGCAG	fda/cbbA	fructose-1,6-bisphosphatealdolase	<1..978	Glycolysis/Gluconeogenesis	COG0191
023.1_WP_079317100.:	CUST_7196_PI442182261	HOT208_60m_813O14	023.1_WP_079317100.:	ATTGGAGGGAATCATTGGCAATGATTTTTGATCCGCCTTCGAGGAGACCAATTTTAGATC	opcA	Glucose-6-P dehydrogenase subunit opcA	WP_079317100.: 11970..13271	Pentose Phosphate	COG3429
023.1_WP_079317100.:	CUST_7197_PI442182261	HOT208_60m_813O14	023.1_WP_079317100.:	GTGTAATACTTGGCTGTGAATCAGTGGAATGTATGAGACTTGAAGCAGGTGGAATGGCTA	opcA	Glucose-6-P dehydrogenase subunit opcA	WP_079317100.: 11970..13271	Pentose Phosphate	COG3429
023.1_WP_079317100.:	CUST_7198_PI442182261	HOT208_60m_813O14	023.1_WP_079317100.:	TTATCTATTGATGAATTACCTTCATGGTTGTGGTGGAATGGAAGCTTGGATGAGTCGCCT	opcA	Glucose-6-P dehydrogenase subunit opcA	WP_079317100.: 11970..13271	Pentose Phosphate	COG3429
023.1_WP_079317102.:	CUST_7202_PI442182261	HOT208_60m_813O14	023.1_WP_079317102.:	GACAAAAGTATTCGTTGATAACTGGCGTTGGCAAGGGGTTCCTTTCTATTTGAGAACAGG	opcA	Glucose-6-P dehydrogenase subunit opcA	WP_079317102.: 13274..14797	Pentose Phosphate	COG3429
023.1_WP_079317100.:	CUST_7199_PI442182261	HOT208_60m_813O14	023.1_WP_079317100.:	CGCGGGGGATCATATGATTCAAGCGTTGTTTTTGATTTCATGGATTAGCGATAAACTTGG	opcA	Glucose-6-P dehydrogenase subunit opcA	WP_079317100.: 11970..13271	Pentose Phosphate	COG3429
023.1_WP_079317100.:	CUST_7200_PI442182261	HOT208_60m_813O14	023.1_WP_079317100.:	TCCCTTATCTTTGGGAAATCCAAGTATTCATTTAGGACAAGTTATTGGACTGAGGCTGAT	opcA	Glucose-6-P dehydrogenase subunit opcA	WP_079317100.: 11970..13271	Pentose Phosphate	COG3429
023.1_WP_079317100.:	CUST_7201_PI442182261	HOT208_60m_813O14	023.1_WP_079317100.:	TCACTGATTATGGTCTAAGGTTAATAATTGATACTGCTCTTGGATCGCCTCAAAGATGTT	opcA	Glucose-6-P dehydrogenase subunit opcA	WP_079317100.: 11970..13271	Pentose Phosphate	COG3429
023.1_WP_079317102.:	CUST_7203_PI442182261	HOT208_60m_813O14	023.1_WP_079317102.:	ACTGCTATGGAACCTCCTGGGAAGTTTGAACCAGAAGCAATAAGAAATGAAAAGGCAAAG	opcA	Glucose-6-P dehydrogenase subunit opcA	WP_079317102.: 13274..14797	Pentose Phosphate	COG3429
023.1_WP_079317102.:	CUST_7204_PI442182261	HOT208_60m_813O14	023.1_WP_079317102.:	TAGCTGATGCAATGCTTTCTGACCCAACATTATTTACTCGAAGTGATGAGGTAGAGGCAG	opcA	Glucose-6-P dehydrogenase subunit opcA	WP_079317102.: 13274..14797	Pentose Phosphate	COG3429
023.1_WP_079317102.:	CUST_7205_PI442182261	HOT208_60m_813O14	023.1_WP_079317102.:	CTTGCTGATGAAAATGAACCTTGGAATTGTTGCATAAGAGGTCAATATGGAGAGGGAGGG	opcA	Glucose-6-P dehydrogenase subunit opcA	WP_079317102.: 13274..14797	Pentose Phosphate	COG3429
023.1_WP_079317102.:	CUST_7206_PI442182261	HOT208_60m_813O14	023.1_WP_079317102.:	TGGGGACCTCCTGAGTCAGATCAATTAATTTCAAAAGATAATATTTTCTGGCGCAGACCC	opcA	Glucose-6-P dehydrogenase subunit opcA	WP_079317102.: 13274..14797	Pentose Phosphate	COG3429
023.1_WP_079317102.:	CUST_7207_PI442182261	HOT208_60m_813O14	023.1_WP_079317102.:	TCAGCCAAATGAAGGTGCTACTTTCAAATTTGAGGTTAAATCTCCTGGTTCTGGAATGAA	opcA	Glucose-6-P dehydrogenase subunit opcA	WP_079317102.: 13274..14797	Pentose Phosphate	COG3429
247.1_WP_079318874.:	CUST_7357_PI442182261	HOT208_60m_813O14	247.1_WP_079318874.:	ACCAACTGGAGAATTAGGAGCACCTACTGCAGATATCAATACAACTATAGCTTTGGCTTT	atpI	ATP synthase subunit A	WP_079318874.: 168..670	Energy Metabolism	COG0356
247.1_WP_079318874.:	CUST_7355_PI442182261	HOT208_60m_813O14	247.1_WP_079318874.:	TCCAATACCCGTTATGTTCCTGGGATTATTTACTAGCGCAATTCAGGCATTGATTTTCGC	atpI	ATP synthase subunit A	WP_079318874.: 168..670	Energy Metabolism	COG0356
247.1_WP_079318874.:	CUST_7356_PI442182261	HOT208_60m_813O14	247.1_WP_079318874.:	TCGCTCTCTTTCAGGCTATTTGGAAATATCTTGGCTGATGAGCTTGTTGTTGGTGTTCTA	atpI	ATP synthase subunit A	WP_079318874.: 168..670	Energy Metabolism	COG0356
247.1_WP_079318874.:	CUST_7358_PI442182261	HOT208_60m_813O14	247.1_WP_079318874.:	TGTCCACCCAACTCCTATTATGCTTCCTTTCAAAATTCTAGAGGACTTTACGAAACCTTT	atpI	ATP synthase subunit A	WP_079318874.: 168..670	Energy Metabolism	COG0356
247.1_WP_079318874.:	CUST_7359_PI442182261	HOT208_60m_813O14	247.1_WP_079318874.:	TGGATGCCATTTATAGGTACTTTATTTTTATTTGTCTTTGTTAGTAACTGGGGAGGAGCT	atpI	ATP synthase subunit A	WP_079318874.: 168..670	Energy Metabolism	COG0356
247.1_WP_079318874.:	CUST_7360_PI442182261	HOT208_60m_813O14	247.1_WP_079318874.:	TTGGTTTCACTTTCTTATTTCTATGCAGGTTTAAGCAATAAGGGTTGGAGATATTTCGAA	atpI	ATP synthase subunit A	WP_079318874.: 168..670	Energy Metabolism	COG0356
028.1_1431	CUST_7477_PI442182261	HOT208_60m_813O14	028.1_1431	TATTCGGTCACTTGGTTTGGGTTACTGGTTTCATGTTCCTAATATCATGGCGTGGTTACT	psaB	Photosystem I P700 chlorophyll A apoprotein A2 PsaB	<1..873	Photosystem I	COG2885
028.1_1431	CUST_7478_PI442182261	HOT208_60m_813O14	028.1_1431	TGGACGTGGAGGTACTTGTGATAGTTCATCTTGGGACGCTATGTACTTAGCTATGTTCTG	psaB	Photosystem I P700 chlorophyll A apoprotein A2 PsaB	<1..873	Photosystem I	COG2885
028.1_1431	CUST_7482_PI442182261	HOT208_60m_813O14	028.1_1431	CATTTCACGATTGGAAACATACTTACATTTGGGGCATTCGTTATCGCATCCACTTCAGGT	psaB	Photosystem I P700 chlorophyll A apoprotein A2 PsaB	<1..873	Photosystem I	COG2885
028.1_1431	CUST_7479_PI442182261	HOT208_60m_813O14	028.1_1431	GATGCATTAAGTGCTTTCTTACCTATCGGGCCAGCAGATTTCTTAGTTCACCATGCAATC	psaB	Photosystem I P700 chlorophyll A apoprotein A2 PsaB	<1..873	Photosystem I	COG2885
028.1_1431	CUST_7480_PI442182261	HOT208_60m_813O14	028.1_1431	AAATTTACTAGCATGGGTAACTTTCTACTGGCATTGGAAACACCTAGCAATTTGGCAGGG	psaB	Photosystem I P700 chlorophyll A apoprotein A2 PsaB	<1..873	Photosystem I	COG2885
028.1_1431	CUST_7481_PI442182261	HOT208_60m_813O14	028.1_1431	AGTTAATTGAAACATTAGTTTGGGCACATCAGCGTACTCCAATTGCTAACCTTGTTGGCT	psaB	Photosystem I P700 chlorophyll A apoprotein A2 PsaB	<1..873	Photosystem I	COG2885
029.1_WP_079317152.:	CUST_7519_PI442182261	HOT208_60m_813O14	029.1_WP_079317152.:	TTGAGGGGGGCACTTTTAACTATTAATGGTATAGCAGCTGGCATGAGAAATACAGGTTGA	ppc	Phosphoenolpyruvate carboxylase	WP_079317152.: 5263..8232	Krebs cycle and related genes	COG2352
029.1_WP_079317152.:	CUST_7520_PI442182261	HOT208_60m_813O14	029.1_WP_079317152.:	TCTACAGGTTTCACTGCTAAGAAGACTAAGAGATCAGACAAGACAACCCCCAATTAGCGA	ppc	Phosphoenolpyruvate carboxylase	WP_079317152.: 5263..8232	Krebs cycle and related genes	COG2352
029.1_WP_079317152.:	CUST_7522_PI442182261	HOT208_60m_813O14	029.1_WP_079317152.:	AGACTTGACGCTACTCCAGAATGGAATCAATTAATGTCCAGGTTAGCAGAAACATCAAGA	ppc	Phosphoenolpyruvate carboxylase	WP_079317152.: 5263..8232	Krebs cycle and related genes	COG2352
031.1_1456	CUST_7622_PI442182261	HOT208_60m_813O14	031.1_1456	TTGAACTGGGGAGCATATGCCCAAAATCCTGATTCTCTAGATCAAGTATTTGGAACAGCT	psaB	Photosystem I P700 chlorophyll A apoprotein A2 PsaB	<1..1083	Photosystem I	COG2885
031.1_1456	CUST_7623_PI442182261	HOT208_60m_813O14	031.1_1456	ACAGGTCATTTGGTTCATGTTGCTATACCTGAATCAAGAGGACAACATGTAGGTTGGGAT	psaB	Photosystem I P700 chlorophyll A apoprotein A2 PsaB	<1..1083	Photosystem I	COG2885
031.1_1456	CUST_7624_PI442182261	HOT208_60m_813O14	031.1_1456	AGGTGGTCTTCATCCTCAAAGTGAAGCATTATGGCTTACTGACATTGCTCATCACCATAT	psaB	Photosystem I P700 chlorophyll A apoprotein A2 PsaB	<1..1083	Photosystem I	COG2885
031.1_1456	CUST_7625_PI442182261	HOT208_60m_813O14	031.1_1456	TAGCAAGAGTACTTGGAACCAAGGAAGCTTTGATAAGCCACCTTAGTTGGGTAACAATGT	psaB	Photosystem I P700 chlorophyll A apoprotein A2 PsaB	<1..1083	Photosystem I	COG2885
031.1_1456	CUST_7626_PI442182261	HOT208_60m_813O14	031.1_1456	CAACTTGGATTAGCATTAGCATCACTTGGTGTGGCAACTTCTCTTGTAGCCCAACATATG	psaB	Photosystem I P700 chlorophyll A apoprotein A2 PsaB	<1..1083	Photosystem I	COG2885
031.1_1456	CUST_7627_PI442182261	HOT208_60m_813O14	031.1_1456	TGCTCTATACAGTCATCATCAGTACATAGCAATGTTCTTGATGGTCGGTGCTTTTGCACA	psaB	Photosystem I P700 chlorophyll A apoprotein A2 PsaB	<1..1083	Photosystem I	COG2885
038.1_WP_079317207.:	CUST_7743_PI442182261	HOT208_60m_813O14	038.1_WP_079317207.:	AAAAGTTCCCAGAAAATAAAAGCTTTGAAAGAGATTTCGAAGGCCGAGGCGAGGATTCAA	atpC	ATP synthase epsilon subunit	WP_079317207.: 5583..5987	Energy Metabolism	COG0355
038.1_WP_079317207.:	CUST_7744_PI442182261	HOT208_60m_813O14	038.1_WP_079317207.:	TGTTCAAAATGCTGAACAAGATCTTAAAGAAGCAAAATTAGCAATTAGCAAATTTCCTGA	atpC	ATP synthase epsilon subunit	WP_079317207.: 5583..5987	Energy Metabolism	COG0355
038.1_WP_079317207.:	CUST_7747_PI442182261	HOT208_60m_813O14	038.1_WP_079317207.:	AATGTTTATCAAGGCGAAGCTGAAGAAGTAATCCTTCCTAGTACCACGGGACAAATTGGT	atpC	ATP synthase epsilon subunit	WP_079317207.: 5583..5987	Energy Metabolism	COG0355
038.1_WP_079317207.:	CUST_7745_PI442182261	HOT208_60m_813O14	038.1_WP_079317207.:	TGCTGAAATTGAATCAGATGAAGTCATTGTTTTGGTAAACAATGCAGAAATTGGATCTGA	atpC	ATP synthase epsilon subunit	WP_079317207.: 5583..5987	Energy Metabolism	COG0355
038.1_WP_079317207.:	CUST_7746_PI442182261	HOT208_60m_813O14	038.1_WP_079317207.:	TTGGCGTTTTAAGGCTAAGAATGAATTCTCAGTGGAAATCAATAGCTTTAATGGGTGGCT	atpC	ATP synthase epsilon subunit	WP_079317207.: 5583..5987	Energy Metabolism	COG0355
051.1_WP_079317266.:	CUST_7972_PI442182261	HOT208_60m_813O14	051.1_WP_079317266.:	GCTATTAATGTAGGTATTTCAGTTAGCCGTGTTGGAGGAGCCGCTCAAACAAAAGCAATT	atpA	ATP synthase alpha subunit	WP_079317266.: <1..1318	Energy Metabolism	COG0056
051.1_WP_079317266.:	CUST_7973_PI442182261	HOT208_60m_813O14	051.1_WP_079317266.:	ACCAGCTGCTTTACAGTATTTAGCACCTTATACTGGCGCAGCAATTGCTGAACATTTTAT	atpA	ATP synthase alpha subunit	WP_079317266.: <1..1318	Energy Metabolism	COG0056
051.1_WP_079317266.:	CUST_7974_PI442182261	HOT208_60m_813O14	051.1_WP_079317266.:	TAGCGATTGGTCAGAAGTCTGCATCAGTAGCAAACATCGTAGAAGTCTTAAGGGAGAGAG	atpA	ATP synthase alpha subunit	WP_079317266.: <1..1318	Energy Metabolism	COG0056
051.1_WP_079317266.:	CUST_7975_PI442182261	HOT208_60m_813O14	051.1_WP_079317266.:	AAAGACCACCAGGAAGAGAGGCTTATCCTGGAGACGTGTTTTACTTGCATAGTAGACTAC	atpA	ATP synthase alpha subunit	WP_079317266.: <1..1318	Energy Metabolism	COG0056
051.1_WP_079317266.:	CUST_7976_PI442182261	HOT208_60m_813O14	051.1_WP_079317266.:	ACTCAGGCAGGGGACGTTTCGGCTTACATTCCAACTAATGTTATTTCAATTACAGATGGA	atpA	ATP synthase alpha subunit	WP_079317266.: <1..1318	Energy Metabolism	COG0056
051.1_WP_079317266.:	CUST_7977_PI442182261	HOT208_60m_813O14	051.1_WP_079317266.:	AGAGAGTTACTAAAGCAACCTCAATTCTCTCCGCTAAACCTTGCAGAACAAGTTGCAGTT	atpA	ATP synthase alpha subunit	WP_079317266.: <1..1318	Energy Metabolism	COG0056
010.1_WP_079331852.:	CUST_13_PI442182265	HOT208_60m_813B04	010.1_WP_079331852.:	ACAGACTTGACGCTACTCCAGAATGGAATCAATTGATGTCTAGGTTGGCAGAAACATCAA	ppc	Phosphoenolpyruvate carboxylase	WP_079331852.: 2467..5436	Krebs cycle and related genes	COG2352
010.1_WP_079331852.:	CUST_14_PI442182265	HOT208_60m_813B04	010.1_WP_079331852.:	GATTTCTTTTGCCAAGTTGGTTTGGAGTGGGCACTGCATTGTCAACTGAATTAACTTCAG	ppc	Phosphoenolpyruvate carboxylase	WP_079331852.: 2467..5436	Krebs cycle and related genes	COG2352
010.1_WP_079331852.:	CUST_15_PI442182265	HOT208_60m_813B04	010.1_WP_079331852.:	GGGGTTTTTGCAGGTTTCACTTTTAAGAAGACTAAGAGACCAGACTAGACAACCCCCAAT	ppc	Phosphoenolpyruvate carboxylase	WP_079331852.: 2467..5436	Krebs cycle and related genes	COG2352
010.1_WP_079331852.:	CUST_17_PI442182265	HOT208_60m_813B04	010.1_WP_079331852.:	TTGAGAGGGGCACTTTTAACAATTAATGGGATAGCAGCAGGTATGAGAAATACAGGTTGA	ppc	Phosphoenolpyruvate carboxylase	WP_079331852.: 2467..5436	Krebs cycle and related genes	COG2352
010.1_WP_079331852.:	CUST_18_PI442182265	HOT208_60m_813B04	010.1_WP_079331852.:	AGAGTTCTGCATCAAAGATGGCCATTTTTCAGGATGCTTATATCGAAGGTAGAGATGACA	ppc	Phosphoenolpyruvate carboxylase	WP_079331852.: 2467..5436	Krebs cycle and related genes	COG2352
107.1_WP_079332013.:	CUST_284_PI442182265	HOT208_60m_813B04	107.1_WP_079332013.:	AAAAGGTTTTCTTCCATGAAGGAACCTTTAGAAGCAAAAATTTTTGCCGTCAGCGAGTCA	minC	septum site-determining protein MinC	WP_079332013.: 29490..30152	Cell Division	COG0850
107.1_WP_079332013.:	CUST_285_PI442182265	HOT208_60m_813B04	107.1_WP_079332013.:	TCTTCAAATGGAAACCTATGCATTATTGGAGACGTTAATCCAGGAGCAATAGTTTCCGCT	minC	septum site-determining protein MinC	WP_079332013.: 29490..30152	Cell Division	COG0850
107.1_WP_079332013.:	CUST_286_PI442182265	HOT208_60m_813B04	107.1_WP_079332013.:	TGTTTGGGGCAAATTACTAGGGATCGCATTTGCAGGTAAAAGTGGAAATAAAAATGCATC	minC	septum site-determining protein MinC	WP_079332013.: 29490..30152	Cell Division	COG0850
107.1_WP_079332013.:	CUST_287_PI442182265	HOT208_60m_813B04	107.1_WP_079332013.:	ACCCTTTACAGTTGAGAATTGCAGATGTGATAGCTATAGGACCAAAGGATAAACCCAAAA	minC	septum site-determining protein MinC	WP_079332013.: 29490..30152	Cell Division	COG0850
107.1_WP_079332013.:	CUST_288_PI442182265	HOT208_60m_813B04	107.1_WP_079332013.:	TCCTTAAATTCAAAAAAGAAAGAAGATCTTCTTCACAAAGGAACAGTACGCTCAGGTGAA	minC	septum site-determining protein MinC	WP_079332013.: 29490..30152	Cell Division	COG0850
107.1_WP_079332013.:	CUST_289_PI442182265	HOT208_60m_813B04	107.1_WP_079332013.:	TGTTTGTTCTTTGCGCATTTACTCAAATAATAGAAATACAGTATTAAGCGGGAAGTCTTT	minC	septum site-determining protein MinC	WP_079332013.: 29490..30152	Cell Division	COG0850
107.1_WP_079332015.:	CUST_290_PI442182265	HOT208_60m_813B04	107.1_WP_079332015.:	TCGGCAGTGCGGGATGCAGATAGAGTAATAGGGATTCTTAATACTTCAGATATTGAGCCT	minD	septum site-determining protein MinD	WP_079332015.: 30264..31079	Cell Division	COG2894
107.1_WP_079332015.:	CUST_291_PI442182265	HOT208_60m_813B04	107.1_WP_079332015.:	AGAGTTCGCCCTAACATGATGGCTAGTCAAGAAATGTTATCCATCGAAGATGTTCAAGGG	minD	septum site-determining protein MinD	WP_079332015.: 30264..31079	Cell Division	COG2894
107.1_WP_079332015.:	CUST_295_PI442182265	HOT208_60m_813B04	107.1_WP_079332015.:	AGTACAAATAGAGGAGAGCCACTTACACTTTCAGATGGTAGATCTCCTGCAAAAAAATGT	minD	septum site-determining protein MinD	WP_079332015.: 30264..31079	Cell Division	COG2894
107.1_WP_079332015.:	CUST_292_PI442182265	HOT208_60m_813B04	107.1_WP_079332015.:	CCTGCTGGTGTAGAAGATGGCTTTAAAAATGCTCTTGCAGCCTGTAAAGAAGCTATTGTT	minD	septum site-determining protein MinD	WP_079332015.: 30264..31079	Cell Division	COG2894
107.1_WP_079332015.:	CUST_293_PI442182265	HOT208_60m_813B04	107.1_WP_079332015.:	TTTAACTGCAAACCTAGGTATAGCGCTTGCTAATAGCGGAGCAACAACTGCTGTATTAGA	minD	septum site-determining protein MinD	WP_079332015.: 30264..31079	Cell Division	COG2894
107.1_WP_079332015.:	CUST_294_PI442182265	HOT208_60m_813B04	107.1_WP_079332015.:	AGCAGGCATTGGTTAGACATAAAAAGGAACCTAATCTTGCTCTTCTACCTGCTGGAGATC	minD	septum site-determining protein MinD	WP_079332015.: 30264..31079	Cell Division	COG2894
107.1_WP_025924170.:	CUST_296_PI442182265	HOT208_60m_813B04	107.1_WP_025924170.:	CAAAAAAACTGATAAAACTGATAAAGCTAACAAAGATATCAAAAAGTA	minE	septum site-determining protein MinE	WP_025924170.: 31081..31416	Cell Division	COG0851
107.1_WP_025924170.:	CUST_297_PI442182265	HOT208_60m_813B04	107.1_WP_025924170.:	AGTTGCCAATTTGCCAATCAAAAGAACTATTTCAGGGGAAATAAAATTCAAAAAAACTGA	minE	septum site-determining protein MinE	WP_025924170.: 31081..31416	Cell Division	COG0851
107.1_WP_025924170.:	CUST_298_PI442182265	HOT208_60m_813B04	107.1_WP_025924170.:	TGAAATTGATTTTGAGGAGGTGGCAGTAAGTTTGGAGACTGAGGATAGAATGACTGCACT	minE	septum site-determining protein MinE	WP_025924170.: 31081..31416	Cell Division	COG0851
107.1_WP_025924170.:	CUST_299_PI442182265	HOT208_60m_813B04	107.1_WP_025924170.:	AGTTCCTTAACAACTGATCTTTTGGATAAAATGAGGAAAGAGATTCTTGATGTTGTTGCT	minE	septum site-determining protein MinE	WP_025924170.: 31081..31416	Cell Division	COG0851
107.1_WP_025924170.:	CUST_300_PI442182265	HOT208_60m_813B04	107.1_WP_025924170.:	AGGCAGAGAAACGTCTAGTGCGAATACAGCTAGAGAAAGATTACAACTTGTACTTGCTCA	minE	septum site-determining protein MinE	WP_025924170.: 31081..31416	Cell Division	COG0851
108.1_WP_011818380.:	CUST_397_PI442182265	HOT208_60m_813B04	108.1_WP_011818380.:	GTTTATTCTGAGGGTAATAGATCAGCTGCGGTAAGAATACCTTTAACAGGACCAAGCCCC	glnA	Glutamine synthetase/ glutamate--ammonialigase	WP_011818380.: 5872..7293	Nitrogen Metabolism	COG0174
108.1_WP_011818380.:	CUST_395_PI442182265	HOT208_60m_813B04	108.1_WP_011818380.:	CATTTATGCCCAAACCTGTTTTTAACGACAACGGAACAGGAATGCATGTTCACCAAAGTT	glnA	Glutamine synthetase/ glutamate--ammonialigase	WP_011818380.: 5872..7293	Nitrogen Metabolism	COG0174
108.1_WP_011818380.:	CUST_396_PI442182265	HOT208_60m_813B04	108.1_WP_011818380.:	CAGGTGACGCACTTGCAAATCCTTACTTAGCATTCTCTGTAATGATGCTTGCTGGTATTG	glnA	Glutamine synthetase/ glutamate--ammonialigase	WP_011818380.: 5872..7293	Nitrogen Metabolism	COG0174
108.1_WP_011818380.:	CUST_398_PI442182265	HOT208_60m_813B04	108.1_WP_011818380.:	TGGTGAAGGATCCTATGCAAATTTATCTCAAACAGCAAGATGGTACATCGGAGGCATACT	glnA	Glutamine synthetase/ glutamate--ammonialigase	WP_011818380.: 5872..7293	Nitrogen Metabolism	COG0174
108.1_WP_011818380.:	CUST_399_PI442182265	HOT208_60m_813B04	108.1_WP_011818380.:	GTGCAGGCCAACACGAGCTTGGAATGAAATTTGATTCGCTAATAAATGCTGCTGATAAC	glnA	Glutamine synthetase/ glutamate--ammonialigase	WP_011818380.: 5872..7293	Nitrogen Metabolism	COG0174
108.1_WP_011818380.:	CUST_400_PI442182265	HOT208_60m_813B04	108.1_WP_011818380.:	AGGTACAACAACTAAGACAAAGGCCTCATCCACATGAATTCTTCATGTATTACGATGCGT	glnA	Glutamine synthetase/ glutamate--ammonialigase	WP_011818380.: 5872..7293	Nitrogen Metabolism	COG0174
108.1_WP_079332047.:	CUST_431_PI442182265	HOT208_60m_813B04	108.1_WP_079332047.:	GCTAGACAACTAGATGCGGGAGCTATAATCCCTTTAACTAAATCAGGTTCTACCGCTCGA	pykF	Pyruvate kinase	WP_079332047.: 11455..13245	Glycolysis	COG0469
108.1_WP_079332047.:	CUST_429_PI442182265	HOT208_60m_813B04	108.1_WP_079332047.:	CTTGGCAACTACTACAGAGAGAAGTGTAGCGAGAAGATTGCAACTTGTTTGGGGAGTTAC	pykF	Pyruvate kinase	WP_079332047.: 11455..13245	Glycolysis	COG0469
108.1_WP_079332047.:	CUST_430_PI442182265	HOT208_60m_813B04	108.1_WP_079332047.:	TAAGGCTATTGAAAGCCACTTACCCAGTACGATCCCAAATGCTATTAGTGCAGCAGTAAG	pykF	Pyruvate kinase	WP_079332047.: 11455..13245	Glycolysis	COG0469
108.1_WP_079332047.:	CUST_432_PI442182265	HOT208_60m_813B04	108.1_WP_079332047.:	TCAAGGAGATTTAGTAGTCCAAACCGCAGGTACATTAACTGGAATTAGCGGCTCTACAGA	pykF	Pyruvate kinase	WP_079332047.: 11455..13245	Glycolysis	COG0469
108.1_WP_079332047.:	CUST_433_PI442182265	HOT208_60m_813B04	108.1_WP_079332047.:	TCAAAGAACAGCAAAAACTTTTAGTTTAGCTATGCAAATTGCACAGGAGATGGGGATCCT	pykF	Pyruvate kinase	WP_079332047.: 11455..13245	Glycolysis	COG0469
108.1_WP_079332047.:	CUST_434_PI442182265	HOT208_60m_813B04	108.1_WP_079332047.:	AGCAATGGTGACCTTATTACACTTCAGCTAAATGAAGGTGTTATATACATGGGGCAAATT	pykF	Pyruvate kinase	WP_079332047.: 11455..13245	Glycolysis	COG0469
108.1_WP_079332080.:	CUST_550_PI442182265	HOT208_60m_813B04	108.1_WP_079332080.:	TTCCGTTTTTGGGAATAAAGGTTCTACAGATCAACATGCTTATGTTCAGCAACTGAGAGA	pgi	Glucose-6-phosphate isomerase	WP_079332080.: 32132..33715	Glycolysis/Gluconeogenesis	COG0166
108.1_WP_079332080.:	CUST_547_PI442182265	HOT208_60m_813B04	108.1_WP_079332080.:	ATGCATATGATCAACCTGGGGTTGAAGCTGGAAAGAAAGCAGCTGCAAATATTATTGAGT	pgi	Glucose-6-phosphate isomerase	WP_079332080.: 32132..33715	Glycolysis/Gluconeogenesis	COG0166
108.1_WP_079332080.:	CUST_548_PI442182265	HOT208_60m_813B04	108.1_WP_079332080.:	TCCAGCAGCACTATTATCATCCGGATGGTATTTAAGTGGGGATGGTATTGGAAAGAGAGA	pgi	Glucose-6-phosphate isomerase	WP_079332080.: 32132..33715	Glycolysis/Gluconeogenesis	COG0166
108.1_WP_079332080.:	CUST_549_PI442182265	HOT208_60m_813B04	108.1_WP_079332080.:	TGTTTTACACTAGGTGCCTTAATCGCATTATTTGAGAGGGCTGTATCCTTCTACGCTGAA	pgi	Glucose-6-phosphate isomerase	WP_079332080.: 32132..33715	Glycolysis/Gluconeogenesis	COG0166
108.1_WP_079332080.:	CUST_551_PI442182265	HOT208_60m_813B04	108.1_WP_079332080.:	AAGGTACGAGATCAGCACTCTCTAGTGAAAACAGACAAAGTATCACTATCACGTTAGAAA	pgi	Glucose-6-phosphate isomerase	WP_079332080.: 32132..33715	Glycolysis/Gluconeogenesis	COG0166
108.1_WP_079332080.:	CUST_552_PI442182265	HOT208_60m_813B04	108.1_WP_079332080.:	ACTCCGTGAAATGTGTTTCAGTAATGATAATTATTTAGTTAAGGGCGATTGGTCAAATCC	pgi	Glucose-6-phosphate isomerase	WP_079332080.: 32132..33715	Glycolysis/Gluconeogenesis	COG0166
110.1_340	CUST_1791_PI442182265	HOT208_60m_813B04	110.1_340	GTTGTAGATGAAATTGTATTAGCTAAGGCACGAAGAGAAAGTGATGGGACATATTCTGCA	ppc	Phosphoenolpyruvate carboxylase	65291..65394	Krebs cycle and related genes	COG2352
112.1_WP_079332493.:	CUST_1911_PI442182265	HOT208_60m_813B04	112.1_WP_079332493.:	CAGGATTGAGAGTGATTCAGTTCTTGAGGACACTCTTGTTATGGGTGCCGATTTCTTTGA	glgC	Glucose-1-phosphate adenylyltransferase	WP_079332493.: <1..1242	Glycogen Metabolism	COG0448
112.1_WP_079332493.:	CUST_1912_PI442182265	HOT208_60m_813B04	112.1_WP_079332493.:	GGGGAACGCCTCTAGGAGTAGGTGAAGGAACTACTGTAAAAAGAGCAATCCTTGATAAGA	glgC	Glucose-1-phosphate adenylyltransferase	WP_079332493.: <1..1242	Glycogen Metabolism	COG0448
112.1_WP_079332493.:	CUST_1913_PI442182265	HOT208_60m_813B04	112.1_WP_079332493.:	AGAATTCAGTGAAAAACCCACTGGAGAGAAGTTGAAGGCGATGGCAGTAGATACTTCAAA	glgC	Glucose-1-phosphate adenylyltransferase	WP_079332493.: <1..1242	Glycogen Metabolism	COG0448
112.1_WP_079332493.:	CUST_1914_PI442182265	HOT208_60m_813B04	112.1_WP_079332493.:	TATTGGGAAGATATCGGTACCATTGGGGCATTTTTTGAGTCAAACCTTGCATTGACTGAG	glgC	Glucose-1-phosphate adenylyltransferase	WP_079332493.: <1..1242	Glycogen Metabolism	COG0448
112.1_WP_079332493.:	CUST_1915_PI442182265	HOT208_60m_813B04	112.1_WP_079332493.:	AGAGTCTGCTGCCGAAAAACCTTATCTAGCCTCAATGGGTATTTACGTTTTTAGCAGAAA	glgC	Glucose-1-phosphate adenylyltransferase	WP_079332493.: <1..1242	Glycogen Metabolism	COG0448
112.1_WP_079332493.:	CUST_1916_PI442182265	HOT208_60m_813B04	112.1_WP_079332493.:	ACGGATTTTGGTAAGGACATAATTCCAGAAGCCCTCAATAGAGGCGATACTCTTAAAAGT	glgC	Glucose-1-phosphate adenylyltransferase	WP_079332493.: <1..1242	Glycogen Metabolism	COG0448
114.1_WP_011862568.:	CUST_2214_PI442182265	HOT208_60m_813B04	114.1_WP_011862568.:	CCCTGATCATCATGTAAGAATCATCGGATACGATGCTTACACTCAAAGTCAAGGGACAGC	rbcS	Ribulose bisphosphate carboxylase small subunit	WP_011862568.: 7805..8146	Carbon fixation	COG4451
114.1_WP_011862568.:	CUST_2215_PI442182265	HOT208_60m_813B04	114.1_WP_011862568.:	ACTCCCATTCTTTGGGGAAAAAGATCTTAACTTGATCGTGAGCGAATTAGAGGCATGTCA	rbcS	Ribulose bisphosphate carboxylase small subunit	WP_011862568.: 7805..8146	Carbon fixation	COG4451
114.1_WP_011862568.:	CUST_2216_PI442182265	HOT208_60m_813B04	114.1_WP_011862568.:	AGTCCAGTTATTGAGCATGTTCATCCAAGTGGATGTATGCAAACTTATTGGTCTTATTGG	rbcS	Ribulose bisphosphate carboxylase small subunit	WP_011862568.: 7805..8146	Carbon fixation	COG4451
114.1_WP_011862568.:	CUST_2217_PI442182265	HOT208_60m_813B04	114.1_WP_011862568.:	CCACCGATGACCCAGGAAGAAATATATGACCAAATTGCGTACATAATTGCTCAAGGTTGG	rbcS	Ribulose bisphosphate carboxylase small subunit	WP_011862568.: 7805..8146	Carbon fixation	COG4451
114.1_WP_011862568.:	CUST_2218_PI442182265	HOT208_60m_813B04	114.1_WP_011862568.:	TTCCAGAGCACAGTAAGCGACTATCAAACAGTTGCAACCCTGGAAACATTCGGTTTCTTA	rbcS	Ribulose bisphosphate carboxylase small subunit	WP_011862568.: 7805..8146	Carbon fixation	COG4451
114.1_WP_079332603.:	CUST_2395_PI442182265	HOT208_60m_813B04	114.1_WP_079332603.:	CAAGCGGTAACTTGTGCCAATGCAAAAATAACTCTAATTTCCCCGTTCGTTGGACGTATA	tal	Transaldolase	WP_079332603.: 37061..38062	Pentose Phosphate	COG0176
114.1_WP_079332603.:	CUST_2394_PI442182265	HOT208_60m_813B04	114.1_WP_079332603.:	TATCGACAGAAGTTGATGCAAGACTGAGCTTTGATACCGAAGCTACGGTAAAAAAAGCGA	tal	Transaldolase	WP_079332603.: 37061..38062	Pentose Phosphate	COG0176
114.1_WP_079332603.:	CUST_2396_PI442182265	HOT208_60m_813B04	114.1_WP_079332603.:	TTGGATTGGCATAAAGCAAAAACTGGTAAAACTAGTTTTGTTGGTGCTGAAGACCCTGGT	tal	Transaldolase	WP_079332603.: 37061..38062	Pentose Phosphate	COG0176
114.1_WP_079332603.:	CUST_2397_PI442182265	HOT208_60m_813B04	114.1_WP_079332603.:	GCTGCAACTTGGGAAGGAATTAAGGCAGCTGAAATTTTGGAAAAAGAAGGTATTAAGTGC	tal	Transaldolase	WP_079332603.: 37061..38062	Pentose Phosphate	COG0176
114.1_WP_079332603.:	CUST_2398_PI442182265	HOT208_60m_813B04	114.1_WP_079332603.:	TGGCAAGTGAAAAGCTTAGTGAAGGTATCACTGGATTCAGTAAGGCTATAGAAGAATTGG	tal	Transaldolase	WP_079332603.: 37061..38062	Pentose Phosphate	COG0176
114.1_WP_079332603.:	CUST_2399_PI442182265	HOT208_60m_813B04	114.1_WP_079332603.:	AGCAGGTTGCGATCTTTTAACAATCGCACCAAAATTTCTTGAGGAACTGAAAAAAGAAAA	tal	Transaldolase	WP_079332603.: 37061..38062	Pentose Phosphate	COG0176
114.1_WP_011817965.:	CUST_2752_PI442182265	HOT208_60m_813B04	114.1_WP_011817965.:	AGCTAGCAGAGGAATTGGTAAAGAAATTGCTTTAGAACTAAGCCGCTTAGGAGCAGAAGT	gdh	3-oxoacyl-acyl-carrier-protein reductase	WP_011817965.: 94296..95045	Entner-Dudoroff	COG1028
114.1_WP_011817965.:	CUST_2751_PI442182265	HOT208_60m_813B04	114.1_WP_011817965.:	CCTGGTCAAGCAAACTATTCTGCAGCTAAAGCTGGAGTTATTGGATTCACCAAAACTTGT	gdh	3-oxoacyl-acyl-carrier-protein reductase	WP_011817965.: 94296..95045	Entner-Dudoroff	COG1028
114.1_WP_011817965.:	CUST_2753_PI442182265	HOT208_60m_813B04	114.1_WP_011817965.:	TGCTGGTATTACTAGAGATGGACTATTGATGAGAATGAAATCGGAACAATGGGATGATGT	gdh	3-oxoacyl-acyl-carrier-protein reductase	WP_011817965.: 94296..95045	Entner-Dudoroff	COG1028
114.1_WP_011817965.:	CUST_2754_PI442182265	HOT208_60m_813B04	114.1_WP_011817965.:	TGCTTCAAGAGGTATAAACGTAAATGCAATAGCTCCAGGCTTTATAGAAACAGAAATGAC	gdh	3-oxoacyl-acyl-carrier-protein reductase	WP_011817965.: 94296..95045	Entner-Dudoroff	COG1028
114.1_WP_011817965.:	CUST_2755_PI442182265	HOT208_60m_813B04	114.1_WP_011817965.:	TCAAGTAATGCTGGAAGTTACATTACAGGGCAAACAATAAGTATAGATGGAGGTATGAGT	gdh	3-oxoacyl-acyl-carrier-protein reductase	WP_011817965.: 94296..95045	Entner-Dudoroff	COG1028
114.1_WP_011817965.:	CUST_2756_PI442182265	HOT208_60m_813B04	114.1_WP_011817965.:	AAGAGAGGATTCTGTCAGTTCAGCTTTTGAAGAAATCATAAAAATTAATGGCTCCATTGA	gdh	3-oxoacyl-acyl-carrier-protein reductase	WP_011817965.: 94296..95045	Entner-Dudoroff	COG1028
114.1_WP_079332714.:	CUST_2793_PI442182265	HOT208_60m_813B04	114.1_WP_079332714.:	GGAAAATATCCGATAATTTGCGCAGAATTATGTGGCCCATATCATGGAGGAATGAGAGCC	ctaC/coxB	Cytochrome c oxidase subunit II	WP_079332714.: 101643..102446	Respiration	COG1622
114.1_WP_079332714.:	CUST_2794_PI442182265	HOT208_60m_813B04	114.1_WP_079332714.:	CATGCTTTTTGGGTACCAGAGTTCAGAATTAAACAGGATATTATCCCTGGACAACCGACT	ctaC/coxB	Cytochrome c oxidase subunit II	WP_079332714.: 101643..102446	Respiration	COG1622
114.1_WP_079332714.:	CUST_2795_PI442182265	HOT208_60m_813B04	114.1_WP_079332714.:	TCCCAAGGGCAATTTCATATCAGGAGAACTACACGTTCCTGTTGATCAAAAAGTATCAAT	ctaC/coxB	Cytochrome c oxidase subunit II	WP_079332714.: 101643..102446	Respiration	COG1622
114.1_WP_079332714.:	CUST_2796_PI442182265	HOT208_60m_813B04	114.1_WP_079332714.:	TAGTTTTTGCTATATCTTTTTGGATTGGTTTTAATGTAAATTTGCTCCCAGCGGAAGCAA	ctaC/coxB	Cytochrome c oxidase subunit II	WP_079332714.: 101643..102446	Respiration	COG1622
114.1_WP_079332714.:	CUST_2797_PI442182265	HOT208_60m_813B04	114.1_WP_079332714.:	AGCTACAACATCTACGATCGAATGGGAGGAATGAAAGAACTAAATCATAACCATGAAATG	ctaC/coxB	Cytochrome c oxidase subunit II	WP_079332714.: 101643..102446	Respiration	COG1622
114.1_WP_079332714.:	CUST_2798_PI442182265	HOT208_60m_813B04	114.1_WP_079332714.:	AGCTTATTCAAATTTAGGAAAAGAGATGATCAGATAGGCGATGGTATAGCTTTAGAGGGA	ctaC/coxB	Cytochrome c oxidase subunit II	WP_079332714.: 101643..102446	Respiration	COG1622
114.1_WP_079332716.:	CUST_2799_PI442182265	HOT208_60m_813B04	114.1_WP_079332716.:	CATTGGCTTGGTTTAAATGGAATGCCGAGAAGAGTTGCAGAATATGATCCTCAGTTCCAG	cyoB/coxA	Cytochrome c oxidase subunit I	WP_079332716.: 102443..104068	Respiration	COG0843
114.1_WP_079332716.:	CUST_2803_PI442182265	HOT208_60m_813B04	114.1_WP_079332716.:	GTGAGAGATGGAAAAGATGCTGGAGATAACCCTTGGAATGCTCTTACACCTGAATGGTTA	cyoB/coxA	Cytochrome c oxidase subunit I	WP_079332716.: 102443..104068	Respiration	COG0843
114.1_WP_079332716.:	CUST_2800_PI442182265	HOT208_60m_813B04	114.1_WP_079332716.:	TCCACCTCCAGTTGAAAATTGGCAAGGAGAAGCTCCTTTAGTTGAAGAACCTTATGGTTA	cyoB/coxA	Cytochrome c oxidase subunit I	WP_079332716.: 102443..104068	Respiration	COG0843
114.1_WP_079332716.:	CUST_2801_PI442182265	HOT208_60m_813B04	114.1_WP_079332716.:	TGTTTTTGGAGGTATTACAGGAGTTGCATTGGCACAGGTACCTTTCGATATTCACGTACA	cyoB/coxA	Cytochrome c oxidase subunit I	WP_079332716.: 102443..104068	Respiration	COG0843
114.1_WP_079332716.:	CUST_2802_PI442182265	HOT208_60m_813B04	114.1_WP_079332716.:	TGCCACGGCATTTATTGCTGTTCCAACAGGTATAAAATTTTTCAATTGGGTTGCAACACT	cyoB/coxA	Cytochrome c oxidase subunit I	WP_079332716.: 102443..104068	Respiration	COG0843
114.1_WP_079332716.:	CUST_2804_PI442182265	HOT208_60m_813B04	114.1_WP_079332716.:	GAATAGTAGTTTTAGGTTTAGTTGTTTGGGCGCATCACATGTTTACGAGTGGAACTCCCC	cyoB/coxA	Cytochrome c oxidase subunit I	WP_079332716.: 102443..104068	Respiration	COG0843
114.1_WP_079332718.:	CUST_2805_PI442182265	HOT208_60m_813B04	114.1_WP_079332718.:	CGCGAATCAAGGGCGGAAGATTAACTAGTCAAAATATGTTCCCTTTGGAAGCTGTTGAAT	cyoC	heme-copper oxidase subunit III	WP_079332718.: 104075..104677	Respiration	COG1845
114.1_WP_079332718.:	CUST_2806_PI442182265	HOT208_60m_813B04	114.1_WP_079332718.:	TTGGTCTTATAACTTTCCTAATTGCGGACGGAATGACTTTTGCTGGATTCTTTGCTGCTT	cyoC	heme-copper oxidase subunit III	WP_079332718.: 104075..104677	Respiration	COG1845
114.1_WP_079332718.:	CUST_2807_PI442182265	HOT208_60m_813B04	114.1_WP_079332718.:	ACACTCAATACAATTTTGTTACTTGTTAGTAGTGCAACTTTCCATAAAGCAGGCAAAGCA	cyoC	heme-copper oxidase subunit III	WP_079332718.: 104075..104677	Respiration	COG1845
114.1_WP_079332718.:	CUST_2808_PI442182265	HOT208_60m_813B04	114.1_WP_079332718.:	TGCAAGTACTTTTTATGCTCTTACTGGTTTTCATGGATTGCATGTCACATTAGGAACTTT	cyoC	heme-copper oxidase subunit III	WP_079332718.: 104075..104677	Respiration	COG1845
114.1_WP_079332718.:	CUST_2809_PI442182265	HOT208_60m_813B04	114.1_WP_079332718.:	TACAAAGCAGTAAATCCATTACCTGATGGTGCTATTTATGAATTAGAACTCCCAATACCT	cyoC	heme-copper oxidase subunit III	WP_079332718.: 104075..104677	Respiration	COG1845
114.1_WP_079332718.:	CUST_2810_PI442182265	HOT208_60m_813B04	114.1_WP_079332718.:	AGCTCAAAAGAAATTCAAAAAAATAATTCTGAAGTCAACGAAACACATGAAGACTTCAGA	cyoC	heme-copper oxidase subunit III	WP_079332718.: 104075..104677	Respiration	COG1845
116.1_WP_079332776.:	CUST_2981_PI442182265	HOT208_60m_813B04	116.1_WP_079332776.:	AGATAGTTCGATAGATATACTCAACTATAAAAATATTGATTTCGTTGCTGCTGGGGGACT	eda	2-dehydro-3-deoxyphosphogluconate aldolase	WP_079332776.: 11550..12176	Entner-Dudoroff	COG0800
116.1_WP_079332776.:	CUST_2982_PI442182265	HOT208_60m_813B04	116.1_WP_079332776.:	TCGATTTTGTATCCGATATCAAAATTAAATATCCAAGAATTAATTTAGGCTCTGCCTCCA	eda	2-dehydro-3-deoxyphosphogluconate aldolase	WP_079332776.: 11550..12176	Entner-Dudoroff	COG0800
116.1_WP_079332776.:	CUST_2983_PI442182265	HOT208_60m_813B04	116.1_WP_079332776.:	GGATTAAATTTTTCGATGATGAAATTTTGGGATAAAGATCTTTTCAATTATGCGCAGTCA	eda	2-dehydro-3-deoxyphosphogluconate aldolase	WP_079332776.: 11550..12176	Entner-Dudoroff	COG0800
116.1_WP_079332776.:	CUST_2984_PI442182265	HOT208_60m_813B04	116.1_WP_079332776.:	GATCTTAAGGAGGCGATAGATTTACGTTGCAATATTATCAAAATTTACCCAATAAAAAGT	eda	2-dehydro-3-deoxyphosphogluconate aldolase	WP_079332776.: 11550..12176	Entner-Dudoroff	COG0800
116.1_WP_079332776.:	CUST_2985_PI442182265	HOT208_60m_813B04	116.1_WP_079332776.:	AGGGTATAAAGCAATCGTGATTGGAGATAAAGGTATTATTAAAAAAAAATTTGATCCAAA	eda	2-dehydro-3-deoxyphosphogluconate aldolase	WP_079332776.: 11550..12176	Entner-Dudoroff	COG0800
116.1_WP_079332776.:	CUST_2986_PI442182265	HOT208_60m_813B04	116.1_WP_079332776.:	ATGGACGTTAAGGGAGATTCTTTTTCTGATTTACTTTTAAAAGAATCTTTTTTTTTACTT	eda	2-dehydro-3-deoxyphosphogluconate aldolase	WP_079332776.: 11550..12176	Entner-Dudoroff	COG0800
122.1_WP_025881193.:	CUST_3513_PI442182265	HOT208_60m_813B04	122.1_WP_025881193.:	CTCCTGATGAGAGCTTTGTGATCATACATTTCAGAAAAGGGGCAAGAGAGAAATGGGGTA	prk	Phosphoribulokinase	WP_025881193.: 15227..16123	Carbon Fixation	COG3954
122.1_WP_025881193.:	CUST_3514_PI442182265	HOT208_60m_813B04	122.1_WP_025881193.:	GGGCAATTTACTCCATGGGAAGATATTCCTGAGGGGACAGACGTACTTTTTTATGAAGGT	prk	Phosphoribulokinase	WP_025881193.: 15227..16123	Carbon Fixation	COG3954
122.1_WP_025881193.:	CUST_3515_PI442182265	HOT208_60m_813B04	122.1_WP_025881193.:	TACCACAGATTTGAAAGAATGCCTATGAAAAAAGCGATGGCAGACGCTCTTTCAAAAGGT	prk	Phosphoribulokinase	WP_025881193.: 15227..16123	Carbon Fixation	COG3954
122.1_WP_025881193.:	CUST_3516_PI442182265	HOT208_60m_813B04	122.1_WP_025881193.:	GTGATGGATACAATGTGGCATCCTATGCTGATTTACTTGTTGGCGTTGTTCCGATAACAA	prk	Phosphoribulokinase	WP_025881193.: 15227..16123	Carbon Fixation	COG3954
122.1_WP_025881193.:	CUST_3517_PI442182265	HOT208_60m_813B04	122.1_WP_025881193.:	TAACGCAGAAAGAGGTTACTCAGCGGAAACCATTGTGGATACGATATTGAGAAGAATGCC	prk	Phosphoribulokinase	WP_025881193.: 15227..16123	Carbon Fixation	COG3954
122.1_WP_025881193.:	CUST_3518_PI442182265	HOT208_60m_813B04	122.1_WP_025881193.:	CATGTCAAGTCCAACAAGTATCGTTGTAAATGGTGGAAAAATGGGATTCGCAATGGAACT	prk	Phosphoribulokinase	WP_025881193.: 15227..16123	Carbon Fixation	COG3954
122.1_WP_079332918.:	CUST_3536_PI442182265	HOT208_60m_813B04	122.1_WP_079332918.:	GAAGGTTGCTGAAATGACAATTAGAACTATGGAAAGATGCGTTCCTGCATCTGTTCCTGG	fda	Fructose-1,6-bisphosphate aldolase class I	WP_079332918.: 18744..19811	Glycolysis/Gluconeogenesis	COG0191
122.1_WP_079332918.:	CUST_3537_PI442182265	HOT208_60m_813B04	122.1_WP_079332918.:	TATGGAATGTTTCATTCTCCTATGGAAGAGCATTACAGCACTCATGCTTAAAGGCCTGGA	fda	Fructose-1,6-bisphosphate aldolase class I	WP_079332918.: 18744..19811	Glycolysis/Gluconeogenesis	COG0191
122.1_WP_079332918.:	CUST_3538_PI442182265	HOT208_60m_813B04	122.1_WP_079332918.:	CAGGTATAAAGGTCGATAAGGGCCTAAATCCACTTCCAGGAGGAGGAGATGTAGAAACTT	fda	Fructose-1,6-bisphosphate aldolase class I	WP_079332918.: 18744..19811	Glycolysis/Gluconeogenesis	COG0191
122.1_WP_079332918.:	CUST_3539_PI442182265	HOT208_60m_813B04	122.1_WP_079332918.:	AACTGCTGAAGTTCAAGAAGAGGTAATAAAGCAGGTTTATAGTGCCTGCCAAGCAAATGG	fda	Fructose-1,6-bisphosphate aldolase class I	WP_079332918.: 18744..19811	Glycolysis/Gluconeogenesis	COG0191
122.1_WP_079332918.:	CUST_3540_PI442182265	HOT208_60m_813B04	122.1_WP_079332918.:	TCAAAAAGCATTAATAGCAAGAGCTCAAGCAAACTCCGAAGCTTCAAAAGGTGCCTATGT	fda	Fructose-1,6-bisphosphate aldolase class I	WP_079332918.: 18744..19811	Glycolysis/Gluconeogenesis	COG0191
122.1_WP_079332918.:	CUST_3541_PI442182265	HOT208_60m_813B04	122.1_WP_079332918.:	TTAGTTGAAAGAGCCGCAAAATATTATGAACAAGGTGCCAGATTTGCAAAGTGGAGAGCA	fda	Fructose-1,6-bisphosphate aldolase class I	WP_079332918.: 18744..19811	Glycolysis/Gluconeogenesis	COG0191
123.1_WP_079332924.:	CUST_3550_PI442182265	HOT208_60m_813B04	123.1_WP_079332924.:	GCAGCACAGGCAGCAATGAATAGTCCTTTATTAGAAGCAGCAAGAATTGATGGAGCTAAA	ftsZ	cell division protein FtsZ	WP_079332924.: 865..1980	Cell Division	COG0206
123.1_WP_079332924.:	CUST_3551_PI442182265	HOT208_60m_813B04	123.1_WP_079332924.:	CAAAGTGGGGCTCTTACAGTTGGCATAGTAACCAAACCATTTTCCTTTGAAGGGAAAAGA	ftsZ	cell division protein FtsZ	WP_079332924.: 865..1980	Cell Division	COG0206
123.1_WP_079332924.:	CUST_3552_PI442182265	HOT208_60m_813B04	123.1_WP_079332924.:	TTTGCAGACATTAGATCAGTTATGACTGAGGCTGGGACTGCACTTCTCGGAATAGGTATA	ftsZ	cell division protein FtsZ	WP_079332924.: 865..1980	Cell Division	COG0206
123.1_WP_079332924.:	CUST_3553_PI442182265	HOT208_60m_813B04	123.1_WP_079332924.:	GAATGCGTCAAGCAGATGAGGGGATCGCAAGACTAGCTGAAAACGTTGATACTCTTATAG	ftsZ	cell division protein FtsZ	WP_079332924.: 865..1980	Cell Division	COG0206
123.1_WP_079332924.:	CUST_3554_PI442182265	HOT208_60m_813B04	123.1_WP_079332924.:	AATGACCGCTTAAAAGACGTTATAGCAGGAGCTCCTCTTCAAGAAGCATTTAGGAATGCT	ftsZ	cell division protein FtsZ	WP_079332924.: 865..1980	Cell Division	COG0206
123.1_WP_079332924.:	CUST_3555_PI442182265	HOT208_60m_813B04	123.1_WP_079332924.:	ATTTATGATGTTGTTGATCCAGAAGCGAATATTATTGTTGGTGCAGTGGTCGATGAGGCC	ftsZ	cell division protein FtsZ	WP_079332924.: 865..1980	Cell Division	COG0206
123.1_WP_079332980.:	CUST_3675_PI442182265	HOT208_60m_813B04	123.1_WP_079332980.:	ACTGCAGCATTCTTTGGGGATGGGACTTGCAATAATGGGCAGTTTTTTGAATGTTTAAAT	pdhA	Pyruvate dehydrogenase E1 component alpha subunit	WP_079332980.: 24111..25148	Glycolysis/Krebs cycle	COG1071
123.1_WP_079332980.:	CUST_3676_PI442182265	HOT208_60m_813B04	123.1_WP_079332980.:	AATGCAATTGAAGCTCCCGAACCCCCTTCAGAAGAATTAACCAAATATATTTGGGCAGAA	pdhA	Pyruvate dehydrogenase E1 component alpha subunit	WP_079332980.: 24111..25148	Glycolysis/Krebs cycle	COG1071
123.1_WP_079332980.:	CUST_3680_PI442182265	HOT208_60m_813B04	123.1_WP_079332980.:	CGAGGAAGTTGATGGAATGGATGTATTAGCAGTTAGAGGAGCAGCACAAAGAGCAATTGA	pdhA	Pyruvate dehydrogenase E1 component alpha subunit	WP_079332980.: 24111..25148	Glycolysis/Krebs cycle	COG1071
123.1_WP_079332980.:	CUST_3677_PI442182265	HOT208_60m_813B04	123.1_WP_079332980.:	TTCAAGAGAGCATCACTTACTCGGAGGATATGCATTTATTGGAGAAGGAATTCCAGTGGC	pdhA	Pyruvate dehydrogenase E1 component alpha subunit	WP_079332980.: 24111..25148	Glycolysis/Krebs cycle	COG1071
123.1_WP_079332980.:	CUST_3678_PI442182265	HOT208_60m_813B04	123.1_WP_079332980.:	ATGGCTCACGATAGAGCTACTAGTAATCCTGAAATTTGGAGAAAAGCGTCTGCTTTTGGT	pdhA	Pyruvate dehydrogenase E1 component alpha subunit	WP_079332980.: 24111..25148	Glycolysis/Krebs cycle	COG1071
123.1_WP_079332980.:	CUST_3679_PI442182265	HOT208_60m_813B04	123.1_WP_079332980.:	GCGCGCTAGAGCAGGAGAAGGTCCAACACTTTTAGAATGTTTGACTTATAGATACAGAGG	pdhA	Pyruvate dehydrogenase E1 component alpha subunit	WP_079332980.: 24111..25148	Glycolysis/Krebs cycle	COG1071
129.1_WP_079333078.:	CUST_4047_PI442182265	HOT208_60m_813B04	129.1_WP_079333078.:	ATCTTGTGAAAGAATTATTAGCGGCACCGGTTTATCAAGAATTGCCGAATGGAGGCTAAC	glk	Glucokinase	WP_079333078.: 24969..26003	Glycolysis	COG0837
129.1_WP_079333078.:	CUST_4048_PI442182265	HOT208_60m_813B04	129.1_WP_079333078.:	TTCCAAAGAAGGGGATCAGATAATGATCGAAGTTGAGAGGATTTGGTTAGGGGCTTATGC	glk	Glucokinase	WP_079333078.: 24969..26003	Glycolysis	COG0837
129.1_WP_079333078.:	CUST_4051_PI442182265	HOT208_60m_813B04	129.1_WP_079333078.:	TTTATTGGGAGATGTTGCTCTTCAAGAATTATGCTTTGGCGGGTTATGGATTTCTGGAGG	glk	Glucokinase	WP_079333078.: 24969..26003	Glycolysis	COG0837
129.1_WP_079333078.:	CUST_4049_PI442182265	HOT208_60m_813B04	129.1_WP_079333078.:	ATATCAGGGAATAAAGTAAAAGTTCTTGCCAGCGAAGGCGGACATGTTGAATACTCTCCA	glk	Glucokinase	WP_079333078.: 24969..26003	Glycolysis	COG0837
129.1_WP_079333078.:	CUST_4050_PI442182265	HOT208_60m_813B04	129.1_WP_079333078.:	ACTATCCAAAATGGCTCCCATTCTGGAGGTAATAACAATGATTTGCATGCCATTGTTGGA	glk	Glucokinase	WP_079333078.: 24969..26003	Glycolysis	COG0837
129.1_WP_079333078.:	CUST_4052_PI442182265	HOT208_60m_813B04	129.1_WP_079333078.:	TGAAAGTAATTTTAGATGAAGAGTTTGGACTTTTTAGTGCAGCATGCAGAGCAAAAATGC	glk	Glucokinase	WP_079333078.: 24969..26003	Glycolysis	COG0837
130.1_WP_079333128.:	CUST_4215_PI442182265	HOT208_60m_813B04	130.1_WP_079333128.:	GAAGCAATTCAAAAAGCTGGGTTTAAGCCTGGTGAGCAGGTATCTTTAGCTTTAGATGCT	eno	Enolase	WP_079333128.: 22205..23497	Glycolysis/Gluconeogenesis	COG0148
130.1_WP_079333128.:	CUST_4216_PI442182265	HOT208_60m_813B04	130.1_WP_079333128.:	TCGGGTCAGATCAAGACCGGCTCTTTGAGTAGAAGTGAAAGGATTGCAAAATACAATAGG	eno	Enolase	WP_079333128.: 22205..23497	Glycolysis/Gluconeogenesis	COG0148
130.1_WP_079333128.:	CUST_4217_PI442182265	HOT208_60m_813B04	130.1_WP_079333128.:	GGAGGAGTTGGGAAATCAAGCAAGATTCGCAGGAGCTTTAGGTTTAGGACCAAAAAATAT	eno	Enolase	WP_079333128.: 22205..23497	Glycolysis/Gluconeogenesis	COG0148
130.1_WP_079333128.:	CUST_4218_PI442182265	HOT208_60m_813B04	130.1_WP_079333128.:	AAATCATTACTTGATCAAAAAGGTCTATCTACTGCTGTAGGCGATGAGGGTGGGTTTGCC	eno	Enolase	WP_079333128.: 22205..23497	Glycolysis/Gluconeogenesis	COG0148
130.1_WP_079333128.:	CUST_4219_PI442182265	HOT208_60m_813B04	130.1_WP_079333128.:	TCCAATAGTTTCAATAGAAGACGGTTTAGCTGAGGATGATTGGGAGGGTTGGTCAGAATT	eno	Enolase	WP_079333128.: 22205..23497	Glycolysis/Gluconeogenesis	COG0148

130.1_WP_079333128.:	CUST_4220_PI442182265	HOT208_60m_813B04	130.1_WP_079333128.:	AGTCATAGAAGTGGTGAGACTGAAGATACAACAATTGCAGATTTATCTGTCGCCACAAGA	eno	Enolase	WP_079333128.:	22205..23497	Glycolysis/Gluconeogenesis	COG0148
132.1_WP_079333152.:	CUST_4280_PI442182265	HOT208_60m_813B04	132.1_WP_079333152.:	GCGCTGGTAAAGCTAGTGATCTAAGATGGGTAGAAGAAAGTGCTAGGCAAGTAGCAAAAT	ugd	UDP-glucose 6-dehydrogenase/nucleotide sugar dehydrogenase	WP_079333152.:	<1..999	Amino sugar and nucleotide sugar r	COG1004
132.1_WP_079333152.:	CUST_4281_PI442182265	HOT208_60m_813B04	132.1_WP_079333152.:	TTCAAGAGCTATTGGCAGTGATAGCAGGATAGGTTCGAAATTTCTTGATTCAGGACCAGG	ugd	UDP-glucose 6-dehydrogenase/nucleotide sugar dehydrogenase	WP_079333152.:	<1..999	Amino sugar and nucleotide sugar r	COG1004
132.1_WP_079333152.:	CUST_4282_PI442182265	HOT208_60m_813B04	132.1_WP_079333152.:	GCTCAAAGAATAAGCTCAATCAATTCAATAGCTGCAATCTGTGAAGCTACAGGCGCAGAT	ugd	UDP-glucose 6-dehydrogenase/nucleotide sugar dehydrogenase	WP_079333152.:	<1..999	Amino sugar and nucleotide sugar r	COG1004
132.1_WP_079333152.:	CUST_4283_PI442182265	HOT208_60m_813B04	132.1_WP_079333152.:	TCCAGTGGACATACAATTGTTGTAGAAAAAAGCACTCTTCCTGTTAGAACAGCAGAAGTT	ugd	UDP-glucose 6-dehydrogenase/nucleotide sugar dehydrogenase	WP_079333152.:	<1..999	Amino sugar and nucleotide sugar r	COG1004
132.1_WP_079333152.:	CUST_4284_PI442182265	HOT208_60m_813B04	132.1_WP_079333152.:	TCTGCGGAACAAGATAATCAGCGAATTGGTATCAGTTTTAGTGTCTTATCAAACCCAGAA	ugd	UDP-glucose 6-dehydrogenase/nucleotide sugar dehydrogenase	WP_079333152.:	<1..999	Amino sugar and nucleotide sugar r	COG1004
132.1_WP_079333152.:	CUST_4285_PI442182265	HOT208_60m_813B04	132.1_WP_079333152.:	AGCCGAAGGTACAGCAATTAAAGATTTAGAAAATCCAGATAGGGTTTTAATTGGAGGAGA	ugd	UDP-glucose 6-dehydrogenase/nucleotide sugar dehydrogenase	WP_079333152.:	<1..999	Amino sugar and nucleotide sugar r	COG1004
146.1_854	CUST_4494_PI442182265	HOT208_60m_813B04	146.1_854	TCTTCCTTTTGCAGCTCTATCTACTGAAATTTCTGAAAAAACAGCAATAAGAACAAGACT	glcH	GPH family sugar transporter		43..450	GlcH sugar transporter	COG2211
146.1_854	CUST_4495_PI442182265	HOT208_60m_813B04	146.1_854	CTTTAGCTGCGATATGGTGGACACCCACTGGTTCAGTGCTAACCAAGACTATTTACTATG	glcH	GPH family sugar transporter		43..450	GlcH sugar transporter	COG2211
146.1_854	CUST_4496_PI442182265	HOT208_60m_813B04	146.1_854	CCGTTAATTGGATGGTTAAGTGATCGTACTAAATCAAGATGGGGGCCTAGAATCCCTTGG	glcH	GPH family sugar transporter		43..450	GlcH sugar transporter	COG2211
146.1_854	CUST_4497_PI442182265	HOT208_60m_813B04	146.1_854	TCAAATTCTTTATTTCTGCTGGTTTACCAGTAATAATTGCAGGATCATTATT	glcH	GPH family sugar transporter		43..450	GlcH sugar transporter	COG2211
146.1_854	CUST_4498_PI442182265	HOT208_60m_813B04	146.1_854	GCTGGGAGATGCAGGCACAGGTTTAGTCGCAACTCAATTTGGTTTTTTTCTTTTCAAATT	glcH	GPH family sugar transporter		43..450	GlcH sugar transporter	COG2211
020.1_930	CUST_4820_PI442182265	HOT208_60m_813B04	020.1_930	TGAAAATTGATATTAAATGCGATTGTTGTGAAAATAATACTTTAATCCCAAATGATTTTG	ppc	Phosphoenolpyruvate carboxylase		<5..265	Krebs cycle and related genes	COG2352
020.1_930	CUST_4822_PI442182265	HOT208_60m_813B04	020.1_930	TCAGGCTGATGGATTAAAAGGCATCCTTAATAGGGATTTTGATGAATGGTCTAATTTTGA	ppc	Phosphoenolpyruvate carboxylase		<5..265	Krebs cycle and related genes	COG2352
020.1_930	CUST_4823_PI442182265	HOT208_60m_813B04	020.1_930	TGAATCAAAATTCTGTCAAAACAATTGGTATTAATGATGAACCAAGAAAAGATTCACACT	ppc	Phosphoenolpyruvate carboxylase		<5..265	Krebs cycle and related genes	COG2352
239.1_WP_002807968.:	CUST_5400_PI442182265	HOT208_60m_813B04	239.1_WP_002807968.:	ACTTTTTGATGAATTAACTGAAACTGAATATAGTGGAGATAAATA	kaiB	Circadian clock protein KaiB	WP_002807968.:	50073..50390	Circadian Rhythm	COG4251
239.1_WP_002807968.:	CUST_5401_PI442182265	HOT208_60m_813B04	239.1_WP_002807968.:	AGGTGATCTTTCAGATAGAGAGAAAGTTCTAATTGGTTTAGATCTACTTTTTGATGAATT	kaiB	Circadian clock protein KaiB	WP_002807968.:	50073..50390	Circadian Rhythm	COG4251
239.1_WP_002807968.:	CUST_5402_PI442182265	HOT208_60m_813B04	239.1_WP_002807968.:	TGCAGAAGAAGATAAGATTTTAGCTACCCCAACCTTAGCTAAAATTTTACCGCCACCAGT	kaiB	Circadian clock protein KaiB	WP_002807968.:	50073..50390	Circadian Rhythm	COG4251
239.1_WP_002807968.:	CUST_5403_PI442182265	HOT208_60m_813B04	239.1_WP_002807968.:	AAATGAATTCAAAGGAGTTTATGCTTTGAAGGTTATTGATGTACTTAAGCAACCACAACT	kaiB	Circadian clock protein KaiB	WP_002807968.:	50073..50390	Circadian Rhythm	COG4251
239.1_WP_002807968.:	CUST_5404_PI442182265	HOT208_60m_813B04	239.1_WP_002807968.:	TGGCAGCAAGAAAAACATACATTTTAAAACTCTATGTTGCTGGAAATACACCAAATTCGA	kaiB	Circadian clock protein KaiB	WP_002807968.:	50073..50390	Circadian Rhythm	COG4251
239.1_WP_079333313.:	CUST_5405_PI442182265	HOT208_60m_813B04	239.1_WP_079333313.:	TTATTCTCGCCACTGGAGCTACTGGTACAGGCAAAACAATGCTTGTATCGAAATTTGTTG	kaiC	circadian clock protein KaiC	WP_079333313.:	50477..52006	Circadian Rhythm	COG0467
239.1_WP_079333313.:	CUST_5406_PI442182265	HOT208_60m_813B04	239.1_WP_079333313.:	GCCAGAGGTGTAAGTTTGAATGCATTTAGGCAGTTTGTCATTGCCGTTACTGGTTATACA	kaiC	circadian clock protein KaiC	WP_079333313.:	50477..52006	Circadian Rhythm	COG0467
239.1_WP_079333313.:	CUST_5407_PI442182265	HOT208_60m_813B04	239.1_WP_079333313.:	AAGGTGAGATGGCACGAGCTTTAAATGTTTTTAAAATGCGGGGATCATGGCATGATAAAC	kaiC	circadian clock protein KaiC	WP_079333313.:	50477..52006	Circadian Rhythm	COG0467
239.1_WP_079333313.:	CUST_5408_PI442182265	HOT208_60m_813B04	239.1_WP_079333313.:	ACTTTTTGCTTATGAAGAATCTAGGGCTCAATTACTTAGGAATGCGACTAGCTGGGGAAT	kaiC	circadian clock protein KaiC	WP_079333313.:	50477..52006	Circadian Rhythm	COG0467
239.1_WP_079333313.:	CUST_5409_PI442182265	HOT208_60m_813B04	239.1_WP_079333313.:	TTAGTGGTGCCCCTCATAGAGTTGTTCCTGATCAAAATGTTCAAAATGTTTTTAAAGGAA	kaiC	circadian clock protein KaiC	WP_079333313.:	50477..52006	Circadian Rhythm	COG0467
239.1_WP_079333313.:	CUST_5410_PI442182265	HOT208_60m_813B04	239.1_WP_079333313.:	TTAGCTCTGGAGTTAAAGATCTTGATGATATGTGTGGTGGAGGTTATTTTCAAGATTCCA	kaiC	circadian clock protein KaiC	WP_079333313.:	50477..52006	Circadian Rhythm	COG0467
247.1_1205	CUST_6133_PI442182265	HOT208_60m_813B04	247.1_1205	AAATACTTGATGGGCTTCACGATTCACAGTTTCCCTTAAAGGTTTGGCAAACAGGTAGTG	fumC	class II fumarate hydratase		<1..736	Krebs cycle and related genes	COG0114
247.1_1205	CUST_6134_PI442182265	HOT208_60m_813B04	247.1_1205	AAGAACCCATTTTCAAGATGCAGTTCCACTCACTTTTGGGCAAGAAATATCAGGATGGTC	fumC	class II fumarate hydratase		<1..736	Krebs cycle and related genes	COG0114
247.1_1205	CUST_6135_PI442182265	HOT208_60m_813B04	247.1_1205	TAGGATTGAAAAAGATAGTATGGGAACAATAGAGGTTCCCATGGAAGCTTTGTGGGGTGC	fumC	class II fumarate hydratase		<1..736	Krebs cycle and related genes	COG0114
247.1_1205	CUST_6136_PI442182265	HOT208_60m_813B04	247.1_1205	TACCTCTTGGAGGAACTGCGGTTGGTACAGAGATTAATTGTCCAAAAGGATTTTGTGAAG	fumC	class II fumarate hydratase		<1..736	Krebs cycle and related genes	COG0114
247.1_1205	CUST_6137_PI442182265	HOT208_60m_813B04	247.1_1205	TGCAGCTTTAAAAACTAATTCAGAGCTTGGTAGTCATCAACCAATTCATCCGAATGATGA	fumC	class II fumarate hydratase		<1..736	Krebs cycle and related genes	COG0114
247.1_1205	CUST_6138_PI442182265	HOT208_60m_813B04	247.1_1205	TCCAACAATCAGAGAACTTACTAAGGTCCTTGATAAAAAAAGTGAAGAATGGAAAGACCT	fumC	class II fumarate hydratase		<1..736	Krebs cycle and related genes	COG0114
250.1_WP_079333574.:	CUST_6231_PI442182265	HOT208_60m_813B04	250.1_WP_079333574.:	TGTCGTAAAATCAAGAAATAGCGAGGGTGGCACAGGTTTTGTACAGGTAGAAAAAGAGGT	argH	Argininosuccinate lyase	WP_079333574.:	8708..10087	Amino Acid metabolism	COG0165
250.1_WP_079333574.:	CUST_6230_PI442182265	HOT208_60m_813B04	250.1_WP_079333574.:	ATCCTGACGTTCCAGAATTGATTAGAGGTAAGACGGGAAGAGTGTATGGACATCTTCAGG	argH	Argininosuccinate lyase	WP_079333574.:	8708..10087	Amino Acid metabolism	COG0165
250.1_WP_079333574.:	CUST_6233_PI442182265	HOT208_60m_813B04	250.1_WP_079333574.:	AGGCAATTTACAGCGTCAGAATTGGGTTTTGAAGAGATTTATAAAAACAGCATTGATGCA	argH	Argininosuccinate lyase	WP_079333574.:	8708..10087	Amino Acid metabolism	COG0165
250.1_WP_079333574.:	CUST_6234_PI442182265	HOT208_60m_813B04	250.1_WP_079333574.:	TGGCAGATTACTTAGTTGGTAAAGATGTTCCTTTTAGAACCGCTTATCAAGTTGTTGGTG	argH	Argininosuccinate lyase	WP_079333574.:	8708..10087	Amino Acid metabolism	COG0165
250.1_WP_079333574.:	CUST_6235_PI442182265	HOT208_60m_813B04	250.1_WP_079333574.:	AAGGATTTTCAAGAGGATAAAGAGCCAATATTTGATACTGCAGAAACCGTATCTTCTTGT	argH	Argininosuccinate lyase	WP_079333574.:	8708..10087	Amino Acid metabolism	COG0165
251.1_WP_079333582.:	CUST_6258_PI442182265	HOT208_60m_813B04	251.1_WP_079333582.:	GAACCCTTCAGGTGATCCAGAAAATATTTTCCCTCATATTTGTCAAGTTGCCGCTGGTTT	galE	UDP-glucose 4-epimerase	WP_079333582.:	4558..5572	Galactose metabolism	COG1087
251.1_WP_079333582.:	CUST_6261_PI442182265	HOT208_60m_813B04	251.1_WP_079333582.:	AATGGAGAGTAGGATATTTAAGGTATTTTAATCCTATAGGCGCACATCCAAGCGGTTTGA	galE	UDP-glucose 4-epimerase	WP_079333582.:	4558..5572	Galactose metabolism	COG1087
251.1_WP_079333582.:	CUST_6259_PI442182265	HOT208_60m_813B04	251.1_WP_079333582.:	ACAAGCCGACGTGATGGAGATATTGAAAGGTCATTGGCTAAAACAGATAAGATGATCAAG	galE	UDP-glucose 4-epimerase	WP_079333582.:	4558..5572	Galactose metabolism	COG1087
251.1_WP_079333582.:	CUST_6260_PI442182265	HOT208_60m_813B04	251.1_WP_079333582.:	TGCCTATTGAAGGAGTAATTCACTGTGCTGGTTTAAAAGCAATAAAGGAGTCAATCTCCA	galE	UDP-glucose 4-epimerase	WP_079333582.:	4558..5572	Galactose metabolism	COG1087
251.1_WP_079333582.:	CUST_6262_PI442182265	HOT208_60m_813B04	251.1_WP_079333582.:	AACTTCAGATGGCACATGTCATAGGGATTATCTACATATAGTAGATTTAGCAACTGCGCA	galE	UDP-glucose 4-epimerase	WP_079333582.:	4558..5572	Galactose metabolism	COG1087
251.1_WP_079333582.:	CUST_6263_PI442182265	HOT208_60m_813B04	251.1_WP_079333582.:	GCAGCAGCGCAACAGTTTATGGTAATTTAAATCAGATTCCATTTGATGAATATGCAATCT	galE	UDP-glucose 4-epimerase	WP_079333582.:	4558..5572	Galactose metabolism	COG1087
264.1_1260	CUST_6425_PI442182265	HOT208_60m_813B04	264.1_1260	AATACTCTTGAAAGTAAAAGTTTTGGATACATTGGGATGGGAGTCTCTGGAGGGGCCAAA	edd	phosphogluconate dehydrogenase (NADP(+)-dependent, decarboxylating)		<1..616	Pentose Phosphate/Entner-Dudorof	COG2084
264.1_1260	CUST_6424_PI442182265	HOT208_60m_813B04	264.1_1260	CAATAGAAAGCTTGTTAACAAAAATGGCTGCCAAAGTTGAAGACGGACCATGTGTTGCAT	edd	phosphogluconate dehydrogenase (NADP(+)-dependent, decarboxylating)		<1..616	Pentose Phosphate/Entner-Dudorof	COG2084
264.1_1260	CUST_6426_PI442182265	HOT208_60m_813B04	264.1_1260	GACTTACTCAAAAACCGAAGAATTTTTACAAGGGCGTGGCCTTGGGAAGAACATAGAAGG	edd	phosphogluconate dehydrogenase (NADP(+)-dependent, decarboxylating)		<1..616	Pentose Phosphate/Entner-Dudorof	COG2084
264.1_1260	CUST_6427_PI442182265	HOT208_60m_813B04	264.1_1260	GGGCCCGCAACAGATGCTGTCATTGATAATATTTCTGGATATCTCGAGGAAGGAGATTTA	edd	phosphogluconate dehydrogenase (NADP(+)-dependent, decarboxylating)		<1..616	Pentose Phosphate/Entner-Dudorof	COG2084
264.1_1260	CUST_6428_PI442182265	HOT208_60m_813B04	264.1_1260	GCCCAGGCGGCTCAGGTCATTTTGTAAAAACTGTTCATAACGGAATTGAATATGGAATTG	edd	phosphogluconate dehydrogenase (NADP(+)-dependent, decarboxylating)		<1..616	Pentose Phosphate/Entner-Dudorof	COG2084
264.1_1260	CUST_6429_PI442182265	HOT208_60m_813B04	264.1_1260	ATGTCCAAGGCACATTTTGGTTTAATAGGTCTTGGTGTTATGGGCGAAAATTTAGTTCTT	edd	phosphogluconate dehydrogenase (NADP(+)-dependent, decarboxylating)		<1..616	Pentose Phosphate/Entner-Dudorof	COG2084
271.1_1268	CUST_6462_PI442182265	HOT208_60m_813B04	271.1_1268	GTCATCTAGCAATAATCGCTCTCTGGGTAGCTGGTAACTTATTTCATATCGCCTGGCAAG	psaB	Photosystem I P700 chlorophyll A apoprotein A2 PsaB		112..660	Photosystem I	COG2885
271.1_1268	CUST_6463_PI442182265	HOT208_60m_813B04	271.1_1268	ATTTTGGCTTGTTGGACTCTATTTGCAGGTTGGTTACACTTACAGCCGAAATTTAGACCC	psaB	Photosystem I P700 chlorophyll A apoprotein A2 PsaB		112..660	Photosystem I	COG2885
271.1_1268	CUST_6464_PI442182265	HOT208_60m_813B04	271.1_1268	GCAAGCTGGAGCTAGTGGTCCAGTAAATATAGCTTACTCAGGTCTATACCATTGGTGGTA	psaB	Photosystem I P700 chlorophyll A apoprotein A2 PsaB		112..660	Photosystem I	COG2885
271.1_1268	CUST_6467_PI442182265	HOT208_60m_813B04	271.1_1268	GTCCTATCGCTCATGCTATTTGGGATCCTCATTTTGGATCTGGGATCACAGAAGCAATG	psaB	Photosystem I P700 chlorophyll A apoprotein A2 PsaB		112..660	Photosystem I	COG2885
030.1_1315	CUST_6694_PI442182265	HOT208_60m_813B04	030.1_1315	TTGGTTACAGCCATAGTGCCAAAAGTTGGTTATGAAAAAGCAGCTGAAATTGCAAACCTT	fumC	class II fumarate hydratase		2006..2636	Krebs cycle and related genes	COG0114
030.1_1315	CUST_6695_PI442182265	HOT208_60m_813B04	030.1_1315	TCAAAATGAACCTGGAAGTTCTATTATGCCGGGTAAAGTGAATCCAACTCAATGCGAAGC	fumC	class II fumarate hydratase		2006..2636	Krebs cycle and related genes	COG0114
030.1_1315	CUST_6696_PI442182265	HOT208_60m_813B04	030.1_1315	ATCATGGCCTCGCACGATCGTCTTGCTCAAGTAATGAGTCAGTTAAAAATATTAGCAGGT	fumC	class II fumarate hydratase		2006..2636	Krebs cycle and related genes	COG0114
030.1_1315	CUST_6697_PI442182265	HOT208_60m_813B04	030.1_1315	GGTTTGTACTCAGATAATGGGTCTTGAATATGCAGTCTCAATGGCAAATTCTAGCGGCAC	fumC	class II fumarate hydratase		2006..2636	Krebs cycle and related genes	COG0114
030.1_1315	CUST_6698_PI442182265	HOT208_60m_813B04	030.1_1315	AGAAAACTTCAGAATAAAATTAGTTGATGGGATGGAACCTAATCAAAAGAGAATGAAGTT	fumC	class II fumarate hydratase		2006..2636	Krebs cycle and related genes	COG0114
030.1_1315	CUST_6699_PI442182265	HOT208_60m_813B04	030.1_1315	AAAAGAGGCAACAATTAAATTAGGTTATTTAAACGAAGATGAATTTGATGAAGCAATGAA	fumC	class II fumarate hydratase		2006..2636	Krebs cycle and related genes	COG0114
040.1_1349	CUST_6872_PI442182265	HOT208_60m_813B04	040.1_1349	GGCGATGCTGAAGAAGCAACAAGAAAAATCTTTTCAGCTCATTTTGGACATCTTGCAGTC	psaA	Photosystem I P700 chlorophyll A apoprotein A1 PsaA		124..807	Photosystem I	COG2885
040.1_1349	CUST_6873_PI442182265	HOT208_60m_813B04	040.1_1349	GTAATGGCTGCACTTATGCTTCATGCCGGAATTTTTCATTATCACAAAGCAGCTCCAAAA	psaA	Photosystem I P700 chlorophyll A apoprotein A1 PsaA		124..807	Photosystem I	COG2885
040.1_1349	CUST_6874_PI442182265	HOT208_60m_813B04	040.1_1349	GGTCCAAAAACTACAACTTGGATCTGGAATTTGCATGCTGACGCACATGATTTTGATGTG	psaA	Photosystem I P700 chlorophyll A apoprotein A1 PsaA		124..807	Photosystem I	COG2885
040.1_1349	CUST_6875_PI442182265	HOT208_60m_813B04	040.1_1349	TTCTAATTATTCAGGTTGGTTAGCTGATCCAACTCATGTCAAACCAGGAGCTCAGCAAGT	psaA	Photosystem I P700 chlorophyll A apoprotein A1 PsaA		124..807	Photosystem I	COG2885
040.1_1349	CUST_6876_PI442182265	HOT208_60m_813B04	040.1_1349	TGGAGCTAACTACAACGGAATTCAAATAAGTTCAGGAATTTTCCACATGTGGCGAGCATG	psaA	Photosystem I P700 chlorophyll A apoprotein A1 PsaA		124..807	Photosystem I	COG2885
040.1_1349	CUST_6877_PI442182265	HOT208_60m_813B04	040.1_1349	GTAGGATTAGGATCTTTAGCATGGGCTGGTCATTGTATTCATATTGGAGCTCCTACTGCA	psaA	Photosystem I P700 chlorophyll A apoprotein A1 PsaA		124..807	Photosystem I	COG2885
086.1_1436	CUST_7294_PI442182265	HOT208_60m_813B04	086.1_1436	CTAAAGTAATGGGAAGAGACGCCAGTGAATTTTTCGGCCCATATGCAAATAGCGATGAAA	rbsK	Sugar kinase, ribokinase family		270..539	Pentose Phosphate	COG0524
086.1_1436	CUST_7295_PI442182265	HOT208_60m_813B04	086.1_1436	GCTGTGGCGATTCTTTTGCTGCTGGGATTCTTTATGGAATGGCATCTAAATGGGATATAG	rbsK	Sugar kinase, ribokinase family		270..539	Pentose Phosphate	COG0524
086.1_1436	CUST_7296_PI442182265	HOT208_60m_813B04	086.1_1436	AACCGAGGGAGAGAAGGGAGGCATAATATTCCCAGGAGGAAGATATAAGGCTCTTAAAAA	rbsK	Sugar kinase, ribokinase family		270..539	Pentose Phosphate	COG0524
102.1_cds_OUL43850.1	CUST_55_PI442182257	HOT208_60m	102.1_cds_OUL43850.1	TCTCTGCGCAGGTATAGAAGTTGGAGTCGCAGCAACAAAAACTTTTTTTGCTCAATTACT	glmS	Glutamine--fructose-6-phosphatetransaminase(isomerizing)	OUL43850.1	259..1713	Amino Acid metabolism	COG0449
102.1_cds_OUL43850.1	CUST_56_PI442182257	HOT208_60m	102.1_cds_OUL43850.1	TCCTCCATTGCTGCCAAATACATTAACTATTGGAGTCACTCAATCTGGAGAAACTGCTGA	glmS	Glutamine--fructose-6-phosphatetransaminase(isomerizing)	OUL43850.1	259..1713	Amino Acid metabolism	COG0449
102.1_cds_OUL43850.1	CUST_57_PI442182257	HOT208_60m	102.1_cds_OUL43850.1	GCCACAGACCGCAAAAAACTGGTTGCAAAATTATTTAATTCAAAACTTAGAAGAGGGCCA	glmS	Glutamine--fructose-6-phosphatetransaminase(isomerizing)	OUL43850.1	259..1713	Amino Acid metabolism	COG0449
102.1_cds_OUL43850.1	CUST_58_PI442182257	HOT208_60m	102.1_cds_OUL43850.1	CAACCCAATCTTATTGCAATAACAAATAGAAAAGAGAGCTCGATAGGAAGGCAGGTTTCA	glmS	Glutamine--fructose-6-phosphatetransaminase(isomerizing)	OUL43850.1	259..1713	Amino Acid metabolism	COG0449
102.1_cds_OUL43850.1	CUST_59_PI442182257	HOT208_60m	102.1_cds_OUL43850.1	AGAACAATTTTCAGGTATCCCTACAAATGTCTTCTATGCAAGTGAATTTCGTTATTCACC	glmS	Glutamine--fructose-6-phosphatetransaminase(isomerizing)	OUL43850.1	259..1713	Amino Acid metabolism	COG0449
102.1_cds_OUL43850.1	CUST_60_PI442182257	HOT208_60m	102.1_cds_OUL43850.1	AAACAATGAAAGGCAATATCGTAATCCAGTTTCTCTAAAAGTCTCAGAGCAAATAATGGA	glmS	Glutamine--fructose-6-phosphatetransaminase(isomerizing)	OUL43850.1	259..1713	Amino Acid metabolism	COG0449
105.1_cds_OUL43845.1	CUST_72_PI442182257	HOT208_60m	105.1_cds_OUL43845.1	GTCCAACAAAGGCCGATCCCATGAAAGTTGCTGAAATGACAATAAGAACTATGGAAAGGT	fda	Fructose-1,6-bisphosphate aldolase class I	OUL43845.1	<1..456	Glycolysis/Gluconeogenesis	COG0191
105.1_cds_OUL43845.1	CUST_73_PI442182257	HOT208_60m	105.1_cds_OUL43845.1	AAAGCATTAATAGCAAGAGCCCAAGCAAATTCTGAAGCTTCAAAAGGAGCCTATGTAGCA	fda	Fructose-1,6-bisphosphate aldolase class I	OUL43845.1	<1..456	Glycolysis/Gluconeogenesis	COG0191
105.1_cds_OUL43845.1	CUST_74_PI442182257	HOT208_60m	105.1_cds_OUL43845.1	TATGGAATGTTTCATTCTCCTATGGAAGAGCATTACAGCACTCATGCTTAAAGGCCTGGA	fda	Fructose-1,6-bisphosphate aldolase class I	OUL43845.1	<1..456	Glycolysis/Gluconeogenesis	COG0191
105.1_cds_OUL43845.1	CUST_75_PI442182257	HOT208_60m	105.1_cds_OUL43845.1	CATCTGTTCCTGGAATAGTTTTCTTATCAGGAGGGTTAAGCGAAGAAGCTGCTTCTGTCT	fda	Fructose-1,6-bisphosphate aldolase class I	OUL43845.1	<1..456	Glycolysis/Gluconeogenesis	COG0191
105.1_cds_OUL43845.1	CUST_76_PI442182257	HOT208_60m	105.1_cds_OUL43845.1	TAGCAGGATCTCAACCTTCATCTGACGAGCAATTGTTTGTGGCAGGCTACACTTA	fda	Fructose-1,6-bisphosphate aldolase class I	OUL43845.1	<1..456	Glycolysis/Gluconeogenesis	COG0191
105.1_cds_OUL43845.1	CUST_77_PI442182257	HOT208_60m	105.1_cds_OUL43845.1	TGCCTGTCAAGCAAATGGAGTATTTCTAGAGGGAACTCTATTAAAACCTTCTATGACTGT	fda	Fructose-1,6-bisphosphate aldolase class I	OUL43845.1	<1..456	Glycolysis/Gluconeogenesis	COG0191
108.1_cds_OUL43842.1	CUST_95_PI442182257	HOT208_60m	108.1_cds_OUL43842.1	TAGTTAATCCTAATGATCTCTCCACTTCTGTTGAGGGCTTATTTGCTGCAGGCGAAGTTG	sdhA	Succinate dehydrogenase flavoprotein subunit	OUL43842.1	<1..839	Krebs cycle and related genes	COG1053
108.1_cds_OUL43842.1	CUST_94_PI442182257	HOT208_60m	108.1_cds_OUL43842.1	TGAAGAGATAGCAGGCACACTTGTAACTGAGGCTGTTAGAGGAGAAGGAGGAAAACTATT	sdhA	Succinate dehydrogenase flavoprotein subunit	OUL43842.1	<1..839	Krebs cycle and related genes	COG1053
108.1_cds_OUL43842.1	CUST_96_PI442182257	HOT208_60m	108.1_cds_OUL43842.1	CTTCATGGGGCAAATCGTCTTGGCGGCAATTCACTTGCTGAAATACTAATTTTTGGAAAG	sdhA	Succinate dehydrogenase flavoprotein subunit	OUL43842.1	<1..839	Krebs cycle and related genes	COG1053
108.1_cds_OUL43842.1	CUST_97_PI442182257	HOT208_60m	108.1_cds_OUL43842.1	TTTGGATGCTCAAATGCTTGATATCTCAAAAGAACCTATGGAAGTTGCTCCTACTGCTCA	sdhA	Succinate dehydrogenase flavoprotein subunit	OUL43842.1	<1..839	Krebs cycle and related genes	COG1053
108.1_cds_OUL43842.1	CUST_98_PI442182257	HOT208_60m	108.1_cds_OUL43842.1	TGGTAAGACCATTACAAAATCAATTGCGTTCAATAATGTGGGAATATTGTGGTGTTACCA	sdhA	Succinate dehydrogenase flavoprotein subunit	OUL43842.1	<1..839	Krebs cycle and related genes	COG1053
108.1_cds_OUL43842.1	CUST_99_PI442182257	HOT208_60m	108.1_cds_OUL43842.1	TCCTCAGTAGTGAGCGATGCGCATGAAAATATAAATAAATTCATCAAAAGTGGAGATGAA	sdhA	Succinate dehydrogenase flavoprotein subunit	OUL43842.1	<1..839	Krebs cycle and related genes	COG1053
132.1_cds_OUL43809.1	CUST_270_PI442182257	HOT208_60m	132.1_cds_OUL43809.1	GATGGCAAGTGACTTAAAATGGGTTGAGGCATCTGCGAGACAAGTAGCAAAATATGCTAG	ugd	UDP-glucose 6-dehydrogenase/nucleotide sugar dehydrogenase	OUL43809.1	<1..560	Amino sugar and nucleotide sugar r	COG1004
132.1_cds_OUL43809.1	CUST_271_PI442182257	HOT208_60m	132.1_cds_OUL43809.1	TTTGTTGCATTGGTGCAGGCTATGTAGGAGGTCCAACGATGTCAGTTATAGCAGATAAAT	ugd	UDP-glucose 6-dehydrogenase/nucleotide sugar dehydrogenase	OUL43809.1	<1..560	Amino sugar and nucleotide sugar r	COG1004
132.1_cds_OUL43809.1	CUST_272_PI442182257	HOT208_60m	132.1_cds_OUL43809.1	AGCAGATATGATTTTTATATCTGTCAACACCCCAACTAAAACCAAAGGAATTGGAGCAGG	ugd	UDP-glucose 6-dehydrogenase/nucleotide sugar dehydrogenase	OUL43809.1	<1..560	Amino sugar and nucleotide sugar r	COG1004
132.1_cds_OUL43809.1	CUST_273_PI442182257	HOT208_60m	132.1_cds_OUL43809.1	ACCAGGTTTAGAAGAAATAGTAAGAAGATGCAGAGGGAAGAACTTGCATTTCTCAACTGA	ugd	UDP-glucose 6-dehydrogenase/nucleotide sugar dehydrogenase	OUL43809.1	<1..560	Amino sugar and nucleotide sugar r	COG1004
132.1_cds_OUL43809.1	CUST_274_PI442182257	HOT208_60m	132.1_cds_OUL43809.1	AAGTACTCTCCCGGTAAGAACTGCAGAAGTAATTAAAACAATACTTTCTTCGTCTCAGTT	ugd	UDP-glucose 6-dehydrogenase/nucleotide sugar dehydrogenase	OUL43809.1	<1..560	Amino sugar and nucleotide sugar r	COG1004
132.1_cds_OUL43809.1	CUST_275_PI442182257	HOT208_60m	132.1_cds_OUL43809.1	ACAAAAATACTAGTTTTGATGTATTGTCAAATCCAGAATTTTTAGCCGAAGGAACAGCAA	ugd	UDP-glucose 6-dehydrogenase/nucleotide sugar dehydrogenase	OUL43809.1	<1..560	Amino sugar and nucleotide sugar r	COG1004
148.1_cds_OUL43793.1	CUST_379_PI442182257	HOT208_60m	148.1_cds_OUL43793.1	AGGTGAAGCGGAAGATGGTCATGGATTTGAAGGTGAACTTTCTACAGATATGCTTCTGAC	cbbA	Fructose-1,6-bisphosphate aldolase class II	OUL43793.1	<1..624	Glycolysis/Gluconeogenesis	COG0191
148.1_cds_OUL43793.1	CUST_380_PI442182257	HOT208_60m	148.1_cds_OUL43793.1	AGCAAGTTTGTCTTGATAGATACAAGCAGTTCTGGTGCGAAGGACAAGCAAGTAAGATCA	cbbA	Fructose-1,6-bisphosphate aldolase class II	OUL43793.1	<1..624	Glycolysis/Gluconeogenesis	COG0191
148.1_cds_OUL43793.1	CUST_381_PI442182257	HOT208_60m	148.1_cds_OUL43793.1	AGCATTTGCTGATAAGGCAAACTTCGACCCAAGACATTTCAACAAGCCTGCTAGAAAATA	cbbA	Fructose-1,6-bisphosphate aldolase class II	OUL43793.1	<1..624	Glycolysis/Gluconeogenesis	COG0191
148.1_cds_OUL43793.1	CUST_382_PI442182257	HOT208_60m	148.1_cds_OUL43793.1	TAGGAAAGTCAACATTGATACTGATAACAGACTTGCTTTTACTGCCGCGGTTAGAGAGGC	cbbA	Fructose-1,6-bisphosphate aldolase class II	OUL43793.1	<1..624	Glycolysis/Gluconeogenesis	COG0191
148.1_cds_OUL43793.1	CUST_383_PI442182257	HOT208_60m	148.1_cds_OUL43793.1	CCATAAAGCACTTCCAAATACCCATCTTGTAATGCATGGATCTAGTTCAGTGCCTCAGGA	cbbA	Fructose-1,6-bisphosphate aldolase class II	OUL43793.1	<1..624	Glycolysis/Gluconeogenesis	COG0191
148.1_cds_OUL43793.1	CUST_384_PI442182257	HOT208_60m	148.1_cds_OUL43793.1	TGATCCTGAAGAAGCTGCAGATTTTGTAGCTAAAACAAAAGTCGATGCTTTAGCTATTGC	cbbA	Fructose-1,6-bisphosphate aldolase class II	OUL43793.1	<1..624	Glycolysis/Gluconeogenesis	COG0191
201.1_cds_OUL43705.1	CUST_938_PI442182257	HOT208_60m	201.1_cds_OUL43705.1	TAAGGCTAAGAATGAATTCCCAGTGGAAATCAATAGCTCTTATGGGAGGCTTCGCTGAAA	atpC	ATP synthase epsilon subunit	OUL43705.1	9987..10391	Energy Metabolism	COG0355
201.1_cds_OUL43704.1	CUST_935_PI442182257	HOT208_60m	201.1_cds_OUL43704.1	ACAGTCGACAGGGCCAGAAAAATTGAAAAATTTCTCTCACAACCTTTCTTCGTAGCAGAA	atpB	ATP synthase beta subunit	OUL43704.1	8486..9946	Energy Metabolism	COG0055
201.1_cds_OUL43705.1	CUST_936_PI442182257	HOT208_60m	201.1_cds_OUL43705.1	CAGAGAAAATTAAAGCCTTGAAAGAGGTTTCAAAGGCTGAGGCTAGGATTCAAGCTGCAA	atpC	ATP synthase epsilon subunit	OUL43705.1	9987..10391	Energy Metabolism	COG0355
201.1_cds_OUL43705.1	CUST_937_PI442182257	HOT208_60m	201.1_cds_OUL43705.1	TGCTGAAATTGGTTCTGAAATTAATGTTCAAAATGCTGAACAAGATCTTAAAGAAGCAAA	atpC	ATP synthase epsilon subunit	OUL43705.1	9987..10391	Energy Metabolism	COG0355
201.1_cds_OUL43705.1	CUST_939_PI442182257	HOT208_60m	201.1_cds_OUL43705.1	AGGCGAAGCTGAAGAAGTAATCCTTCCTAGTACTACTGGTCAAATTGGTGTACTACCAGG	atpC	ATP synthase epsilon subunit	OUL43705.1	9987..10391	Energy Metabolism	COG0355
211.1_cds_OUL43516.1	CUST_1873_PI442182257	HOT208_60m	211.1_cds_OUL43516.1	TCTCGAGTTGAAATCATTAGAGATGTACCTCTTGGAAAAGGTGGAGGTAAGAAGAGAGCA		polysaccharide biosynthesis/export family protein	OUL43516.1	<1..718	Other sugar transporters	COG1596
211.1_cds_OUL43516.1	CUST_1872_PI442182257	HOT208_60m	211.1_cds_OUL43516.1	TGGAGTTGTAAAAGTCCCTCTTGAAGCTGCACTATCAGATGCTATTGATTTGACTGGACC		polysaccharide biosynthesis/export family protein	OUL43516.1	<1..718	Other sugar transporters	COG1596
211.1_cds_OUL43516.1	CUST_1874_PI442182257	HOT208_60m	211.1_cds_OUL43516.1	ATCACAAAATCAAGTAAAAATATAACTACACTCTCTGATGCGATTAGAAAAGCTGGTGGT		polysaccharide biosynthesis/export family protein	OUL43516.1	<1..718	Other sugar transporters	COG1596
211.1_cds_OUL43516.1	CUST_1875_PI442182257	HOT208_60m	211.1_cds_OUL43516.1	TCAGTGAGAAATAGGTTATTAGGAAAAAGTACAGGAGTGATTAGAGAAATTACAGCACCA		polysaccharide biosynthesis/export family protein	OUL43516.1	<1..718	Other sugar transporters	COG1596
211.1_cds_OUL43516.1	CUST_1876_PI442182257	HOT208_60m	211.1_cds_OUL43516.1	CGATAAAACCGCTGTCTGGAAAAATTGTTTTAATAAGGTATGAAAAAGACGGAACAGTT		polysaccharide biosynthesis/export family protein	OUL43516.1	<1..718	Other sugar transporters	COG1596
211.1_cds_OUL43516.1	CUST_1877_PI442182257	HOT208_60m	211.1_cds_OUL43516.1	TACTTTCCAAAACTTAGTAAAGCTGATCCAAGTCAAATTCCTAGATCAATTTTATCTGGT		polysaccharide biosynthesis/export family protein	OUL43516.1	<1..718	Other sugar transporters	COG1596
231.1_cds_OUL43319.1	CUST_3013_PI442182257	HOT208_60m	231.1_cds_OUL43319.1	TCCCAAATGATTTTGAGAGTATTAAAAAAGAAAAATGTTTTGGTAAAAAAAGTGCTTACA	ppc	Phosphoenolpyruvate carboxylase	OUL43319.1	5816..6193	Krebs cycle and related genes	COG2352
231.1_cds_OUL43319.1	CUST_3014_PI442182257	HOT208_60m	231.1_cds_OUL43319.1	AGAGCTTTGGAGGTTTTCAGAGGTAAGAAAATTGATATTAAATGCGACTGTTGTAAACAT	ppc	Phosphoenolpyruvate carboxylase	OUL43319.1	5816..6193	Krebs cycle and related genes	COG2352
231.1_cds_OUL43319.1	CUST_3015_PI442182257	HOT208_60m	231.1_cds_OUL43319.1	AGGGATTTTGATGATTGGTCGAATTTTGATGGTTGGGAAAGTATTTCAGTTCAGCAATGG	ppc	Phosphoenolpyruvate carboxylase	OUL43319.1	5816..6193	Krebs cycle and related genes	COG2352
231.1_cds_OUL43319.1	CUST_3016_PI442182257	HOT208_60m	231.1_cds_OUL43319.1	ACCAAGAAAAGATTCATACTTAGTATATGTAAATCAGGCTGATGGATTAAAAGGCATCCT	ppc	Phosphoenolpyruvate carboxylase	OUL43319.1	5816..6193	Krebs cycle and related genes	COG2352
243.1_cds_621	CUST_3150_PI442182257	HOT208_60m	243.1_cds_621	AGATAGTTCGATAAATATACTCAACTATAAAAATATTGATTTCATTGCTGCTTGAGGACT	eda	2-dehydro-3-deoxyphosphogluconate aldolase		2527..3104	Entner-Dudoroff	COG0800
243.1_cds_621	CUST_3148_PI442182257	HOT208_60m	243.1_cds_621	TGGATTAAATATTCGATGATGAAATTTTGGGATAAGGATCTTTTCAATTATGCGTAGTCA	eda	2-dehydro-3-deoxyphosphogluconate aldolase		2527..3104	Entner-Dudoroff	COG0800
243.1_cds_621	CUST_3149_PI442182257	HOT208_60m	243.1_cds_621	TCCAAGAAATTATTTAGGCTCTACCTCCATAGTTAATAAGCAATCAATAGAAGATTCTTT	eda	2-dehydro-3-deoxyphosphogluconate aldolase		2527..3104	Entner-Dudoroff	COG0800
243.1_cds_621	CUST_3151_PI442182257	HOT208_60m	243.1_cds_621	ATTCCTGGAATTAATAATTTAAAAGATCTTAAGGAAGCGATAGATTTAAATTCCAACATT	eda	2-dehydro-3-deoxyphosphogluconate aldolase		2527..3104	Entner-Dudoroff	COG0800
243.1_cds_621	CUST_3152_PI442182257	HOT208_60m	243.1_cds_621	TGGAGTAACTTAAAATTAAGTCTTTTTTTACTTTTAAAACCTGAGGATAATATTTCCACA	eda	2-dehydro-3-deoxyphosphogluconate aldolase		2527..3104	Entner-Dudoroff	COG0800
243.1_cds_621	CUST_3153_PI442182257	HOT208_60m	243.1_cds_621	CCTCATAAATCTTTAGGATATAAAGCAGTTGTAATTGAAGATAAAGCTATCAAAATTAA	eda	2-dehydro-3-deoxyphosphogluconate aldolase		2527..3104	Entner-Dudoroff	COG0800
024.1_cds_OUL44105.1	CUST_3460_PI442182257	HOT208_60m	024.1_cds_OUL44105.1	AAACAGGTAAAACTAGTTTTGTTGGCGCTGAAGATCCTGGTGTTATTTCGGTTACGCAAA	tal	Transaldolase	OUL44105.1	45175..46176	Pentose Phosphate	COG0176
024.1_cds_OUL44105.1	CUST_3461_PI442182257	HOT208_60m	024.1_cds_OUL44105.1	AGATTGCAGCAACTTGGGAGGGAATTAAGGCTGCTGAAATTTTGGAAAAAGAGGGTATTA	tal	Transaldolase	OUL44105.1	45175..46176	Pentose Phosphate	COG0176
024.1_cds_OUL44105.1	CUST_3462_PI442182257	HOT208_60m	024.1_cds_OUL44105.1	TATTTCAGGGCGCGTATCTACAGAAGTTGATGCAAGACTAAGCTTTGACACTGAAGCTAC	tal	Transaldolase	OUL44105.1	45175..46176	Pentose Phosphate	COG0176
024.1_cds_OUL44105.1	CUST_3463_PI442182257	HOT208_60m	024.1_cds_OUL44105.1	AATTTCTGCCAAGCGGTAACTTGTGCCAATGCAAAGATAACTCTAATTTCTCCGTTCGTT	tal	Transaldolase	OUL44105.1	45175..46176	Pentose Phosphate	COG0176
024.1_cds_OUL44105.1	CUST_3464_PI442182257	HOT208_60m	024.1_cds_OUL44105.1	AGCAGGTTGCGATCTTTTAACAATCGCACCAAAATTTCTTGAGGAACTGAAAAAAGAAAA	tal	Transaldolase	OUL44105.1	45175..46176	Pentose Phosphate	COG0176
024.1_cds_OUL44105.1	CUST_3465_PI442182257	HOT208_60m	024.1_cds_OUL44105.1	AAAGCTTAGTGAAGGTATTACTGGATTCAGTAAGGCTATAGAAGAATTGGAAGAGCTGCT	tal	Transaldolase	OUL44105.1	45175..46176	Pentose Phosphate	COG0176
302.1_cds_OUL43208.1	CUST_4230_PI442182257	HOT208_60m	302.1_cds_OUL43208.1	GGTGAATACTCTTGAAAGTAAAAGTTTTGGATACATCGGGATGGGAGTCTCAGGAGGTGC	edd	phosphogluconate dehydrogenase (NADP(+)-dependent, decarboxyla	OUL43208.1	<1..780	Pentose Phosphate/Entner-Dudorof	COG2084
302.1_cds_OUL43208.1	CUST_4231_PI442182257	HOT208_60m	302.1_cds_OUL43208.1	TATGTTGGCCCAGGCGGCTCAGGTCATTTTGTAAAAACTGTTCATAACGGAATTGAATAT	edd	phosphogluconate dehydrogenase (NADP(+)-dependent, decarboxyla	OUL43208.1	<1..780	Pentose Phosphate/Entner-Dudorof	COG2084
302.1_cds_OUL43208.1	CUST_4232_PI442182257	HOT208_60m	302.1_cds_OUL43208.1	AGGCTTCCTATGATGCCATAGAAAGCTTACTAACAAAAATGGCTGCCAAAGTTGAAGACG	edd	phosphogluconate dehydrogenase (NADP(+)-dependent, decarboxyla	OUL43208.1	<1..780	Pentose Phosphate/Entner-Dudorof	COG2084
302.1_cds_OUL43208.1	CUST_4233_PI442182257	HOT208_60m	302.1_cds_OUL43208.1	TGATGGTAAAAGTTGGGCCCGCAACAGATGCTGTCATTGAAAATATTTCTGGATATCTCG	edd	phosphogluconate dehydrogenase (NADP(+)-dependent, decarboxyla	OUL43208.1	<1..780	Pentose Phosphate/Entner-Dudorof	COG2084
302.1_cds_OUL43208.1	CUST_4234_PI442182257	HOT208_60m	302.1_cds_OUL43208.1	GGGTTATGGACTGTTGTAAGTGCTTTAGAACTGGGGGTATCAGTCCCAACTATTTATGCA	edd	phosphogluconate dehydrogenase (NADP(+)-dependent, decarboxyla	OUL43208.1	<1..780	Pentose Phosphate/Entner-Dudorof	COG2084
302.1_cds_OUL43208.1	CUST_4235_PI442182257	HOT208_60m	302.1_cds_OUL43208.1	ACTTGCAGAAGCTTATGACCTTATGAAGAGAGTCAAAGGTATGAATGGACAGCAGATGTC	edd	phosphogluconate dehydrogenase (NADP(+)-dependent, decarboxyla	OUL43208.1	<1..780	Pentose Phosphate/Entner-Dudorof	COG2084
335.1_cds_OUL43112.1	CUST_4687_PI442182257	HOT208_60m	335.1_cds_OUL43112.1	TTATTCCAAATGACCGATTAAAAGACGTTATAGCGGGAGCTCCCCTTCAAGAAGCGTTTA	ftsZ	cell division protein FtsZ	OUL43112.1	1052..2167	Cell Division	COG0206
335.1_cds_OUL43112.1	CUST_4686_PI442182257	HOT208_60m	335.1_cds_OUL43112.1	TATTGTTGGTGCAGTGGTTGATGAGGCAATGGAAGGGGAAATACAAGTAACTGTTATTGC	ftsZ	cell division protein FtsZ	OUL43112.1	1052..2167	Cell Division	COG0206
335.1_cds_OUL43112.1	CUST_4688_PI442182257	HOT208_60m	335.1_cds_OUL43112.1	TCCCCTTTTAGAGGCAGCTAGAATTGATGGAGCTAAAGGCTGCGTCATTAATATTACTGG	ftsZ	cell division protein FtsZ	OUL43112.1	1052..2167	Cell Division	COG0206
335.1_cds_OUL43112.1	CUST_4689_PI442182257	HOT208_60m	335.1_cds_OUL43112.1	AGTTAATGTTGATTTTGCAGACGTAAGATCGGTTATGACAGAAGCGGGAACTGCTCTTCT	ftsZ	cell division protein FtsZ	OUL43112.1	1052..2167	Cell Division	COG0206
335.1_cds_OUL43112.1	CUST_4690_PI442182257	HOT208_60m	335.1_cds_OUL43112.1	AGTAACCAAGCCATTTTCTTTTGAAGGAAAAAGAAGAATGCGTCAAGCAGAGGAAGGGAT	ftsZ	cell division protein FtsZ	OUL43112.1	1052..2167	Cell Division	COG0206
335.1_cds_OUL43112.1	CUST_4691_PI442182257	HOT208_60m	335.1_cds_OUL43112.1	GCTGATGATGTTCTAAGGATGGGTGTCAAAGGCATTAGTGACATAATTACTTGCCCAGGA	ftsZ	cell division protein FtsZ	OUL43112.1	1052..2167	Cell Division	COG0206
353.1_cds_995	CUST_5097_PI442182257	HOT208_60m	353.1_cds_995	TCAAAAAGTCATCCAGTAGTCGCTGTAACAGGATCTTCAGGAGCAGGAACAAGTACAGTA	prk	Phosphoribulokinase		1..478	Carbon Fixation	COG3954
353.1_cds_995	CUST_5096_PI442182257	HOT208_60m	353.1_cds_995	ACCCGCAGTTGTAGAAGGTGATAGTTACCACAGATTTGAAAGAATGCCCATGAAAAAAGC	prk	Phosphoribulokinase		1..478	Carbon Fixation	COG3954
353.1_cds_995	CUST_5098_PI442182257	HOT208_60m	353.1_cds_995	TTTTTTATGAAGGTTTGCATGGCGGGGTAGAAGGTGATGGATACAATGTTGCCTCTTATG	prk	Phosphoribulokinase		1..478	Carbon Fixation	COG3954
353.1_cds_995	CUST_5099_PI442182257	HOT208_60m	353.1_cds_995	CAGAAGAACATAATACAAGACTTGGGACATCCCTAGAACCTGGGCAATTTACTCCATGGG	prk	Phosphoribulokinase		1..478	Carbon Fixation	COG3954
353.1_cds_995	CUST_5100_PI442182257	HOT208_60m	353.1_cds_995	GCAGAAGCTCTTTCAAAAGGTGAAAATTTCCCTCATTTTGGCCCAGAGGCTAATCTTTTT	prk	Phosphoribulokinase		1..478	Carbon Fixation	COG3954
353.1_cds_995	CUST_5101_PI442182257	HOT208_60m	353.1_cds_995	TGACAAGCTAGAAGAACTTTTTAAAATCTATGGTGAAACTGGAGGAGGGAAAAAAAAGA	prk	Phosphoribulokinase		1..478	Carbon Fixation	COG3954
363.1_cds_OUL43066.1	CUST_5216_PI442182257	HOT208_60m	363.1_cds_OUL43066.1	TTGGTTACAGCCATAGTGCCAAAAGTTGGTTATGAAAAAGCAGCTGAAATTGCAAACCTT	fumC	class II fumarate hydratase	OUL43066.1	<1..367	Krebs cycle and related genes	COG0114
363.1_cds_OUL43066.1	CUST_5215_PI442182257	HOT208_60m	363.1_cds_OUL43066.1	AAAAGAGGCAACAATTAAATTAGGTTATTTAAACGAAGATGAATTTGATGAAGCAATGAA	fumC	class II fumarate hydratase	OUL43066.1	<1..367	Krebs cycle and related genes	COG0114
363.1_cds_OUL43066.1	CUST_5217_PI442182257	HOT208_60m	363.1_cds_OUL43066.1	AGAAAACTTCAGAATAAAATTAGTTGATGGGATGGAACCTAATCAAAAGAGAATGAAGTT	fumC	class II fumarate hydratase	OUL43066.1	<1..367	Krebs cycle and related genes	COG0114
363.1_cds_OUL43066.1	CUST_5218_PI442182257	HOT208_60m	363.1_cds_OUL43066.1	ATATAAACCTCTTATTGGATTTAATATTCTCACAAGTTTAAAATTACTAAAAAATGTA	fumC	class II fumarate hydratase	OUL43066.1	<1..367	Krebs cycle and related genes	COG0114
363.1_cds_OUL43066.1	CUST_5219_PI442182257	HOT208_60m	363.1_cds_OUL43066.1	TGGGTCTTGAATATGCAGTCTCAATGGCAAATTCTAGCGGCACTTTACAGATGAATGAAT	fumC	class II fumarate hydratase	OUL43066.1	<1..367	Krebs cycle and related genes	COG0114
041.1_cds_OUL43981.1	CUST_5372_PI442182257	HOT208_60m	041.1_cds_OUL43981.1	TATAAGATTGTGGCTAAGAACAGAGATTGATAATATTGAAATCTTAATAATTGATTTGC	argH	Argininosuccinate lyase	OUL43981.1	<1..406	Amino Acid metabolism	COG0165
041.1_cds_OUL43981.1	CUST_5374_PI442182257	HOT208_60m	041.1_cds_OUL43981.1	GTTGAGTATTTGGAGGGCAAATTTTCTCCTAGTCCACCTTCTGAAGATATTCACTATTGT	argH	Argininosuccinate lyase	OUL43981.1	<1..406	Amino Acid metabolism	COG0165
041.1_cds_OUL43981.1	CUST_5375_PI442182257	HOT208_60m	041.1_cds_OUL43981.1	TGTTCGATTGCTCATGCGAAAATGCTTAGCAAAACAAAAGTTTTATCCTCTTCTGAAGCT	argH	Argininosuccinate lyase	OUL43981.1	<1..406	Amino Acid metabolism	COG0165
041.1_cds_OUL43981.1	CUST_5376_PI442182257	HOT208_60m	041.1_cds_OUL43981.1	AATGCACTTGACCCTTTTATTGAAAAGTTTAATGCCTCAATCGGTTTTGATAGAAAGCTT	argH	Argininosuccinate lyase	OUL43981.1	<1..406	Amino Acid metabolism	COG0165
057.1_cds_OUL43917.1	CUST_5730_PI442182257	HOT208_60m	057.1_cds_OUL43917.1	TTTGCAATTTGAGATATTTCAATCCAGTTGGAGCAGATCCATCGGGTTTGTTTGGAGAAA	galE	UDP-glucose 4-epimerase	OUL43917.1	739..1506	Galactose metabolism	COG1087
057.1_cds_OUL43917.1	CUST_5731_PI442182257	HOT208_60m	057.1_cds_OUL43917.1	TGTACAATAAAATATGAGTTTGAGGATAGGCGGCCAGGGGATGTATCTTCTTTGATAGCT	galE	UDP-glucose 4-epimerase	OUL43917.1	739..1506	Galactose metabolism	COG1087
057.1_cds_OUL43917.1	CUST_5732_PI442182257	HOT208_60m	057.1_cds_OUL43917.1	GACTGGCCTACTAAAGATGGCACAGCAATAAGAGATTATATTCATGTTTGCGACCTTTCT	galE	UDP-glucose 4-epimerase	OUL43917.1	739..1506	Galactose metabolism	COG1087
057.1_cds_OUL43917.1	CUST_5733_PI442182257	HOT208_60m	057.1_cds_OUL43917.1	ACCTTGGTACTGGTAAAGGCACTACCGTTCTTGAATTAATAAAAACATTCGAAAGAGTAA	galE	UDP-glucose 4-epimerase	OUL43917.1	739..1506	Galactose metabolism	COG1087
057.1_cds_OUL43917.1	CUST_5734_PI442182257	HOT208_60m	057.1_cds_OUL43917.1	TCTCTTATAATCCAAGTAATCTTTTCCCTTATCTCAATTTGGTGGCAGGTGGAAAAAAGA	galE	UDP-glucose 4-epimerase	OUL43917.1	739..1506	Galactose metabolism	COG1087
057.1_cds_OUL43917.1	CUST_5735_PI442182257	HOT208_60m	057.1_cds_OUL43917.1	ATCTGTTCCTGAATCTATTTCAGATCCTTTGAGGTATTGGGATGTAAATGTAAATGGGAC	galE	UDP-glucose 4-epimerase	OUL43917.1	739..1506	Galactose metabolism	COG1087
120.1_37	CUST_187_PI442190719	HOT208_60m_824E10	120.1_37	TAATCCTGTCGGTGCGCATGCTTCAGGTCTAATAGGAGAAGATCCACAAGATATTCCTAA	galE	UDP-glucose 4-epimerase		<1..451	Galactose metabolism	COG1087
120.1_37	CUST_188_PI442190719	HOT208_60m_824E10	120.1_37	TGTCATACAATAGTATTTAGCAGCAGCGCAACAATTTATGGTTTGCCTGATTCAGTCCCA	galE	UDP-glucose 4-epimerase		<1..451	Galactose metabolism	COG1087
120.1_37	CUST_189_PI442190719	HOT208_60m_824E10	120.1_37	TGCTTCAGGAAAAAGAGAGAAAATAAGAATTTTCGGAGATGACTGGCCAACTGAAGATGG	galE	UDP-glucose 4-epimerase		<1..451	Galactose metabolism	COG1087
120.1_37	CUST_190_PI442190719	HOT208_60m_824E10	120.1_37	TCCACTTGAATATTGGGATGTGAATGTGAATGGTTCTAGAGTCTTGTTAAAAGTTATGGA	galE	UDP-glucose 4-epimerase		<1..451	Galactose metabolism	COG1087
120.1_37	CUST_191_PI442190719	HOT208_60m_824E10	120.1_37	TGAGAAATCAGCTGTTAAACCAATTAATCCATATGGAAGAACTAAATCTACTGTAGAATT	galE	UDP-glucose 4-epimerase		<1..451	Galactose metabolism	COG1087
120.1_37	CUST_192_PI442190719	HOT208_60m_824E10	120.1_37	TTTAAATGATTTATATTTAAGCGATAAAAATAAATGGAAAATAGCCAATTTAAGATATTT	galE	UDP-glucose 4-epimerase		<1..451	Galactose metabolism	COG1087
199.1_147	CUST_687_PI442190719	HOT208_60m_824E10	199.1_147	CCCCAAAGCTAAAAGATTAGAATTCAGATCAGGCGACGCACTTGCAAATCCTTACTTAGC	glnA	Glutamine synthetase/ glutamate--ammonialigase		<1..408	Nitrogen Metabolism	COG0174
199.1_147	CUST_688_PI442190719	HOT208_60m_824E10	199.1_147	GAGGTACAACAACCAAGACAAAGGCCACATCCACATGAATTCTTTATGTATTACGATGCA	glnA	Glutamine synthetase/ glutamate--ammonialigase		<1..408	Nitrogen Metabolism	COG0174
199.1_147	CUST_689_PI442190719	HOT208_60m_824E10	199.1_147	TGATCCTGGTGATGGAGTAGATGTAGATTTATTTGAACTTCCAGCTGAGGAACTTGCAAA	glnA	Glutamine synthetase/ glutamate--ammonialigase		<1..408	Nitrogen Metabolism	COG0174
199.1_147	CUST_690_PI442190719	HOT208_60m_824E10	199.1_147	TCTAAATGACTCACTTAATGTGCTAAAATCAGATAAGGATTATCTATTAGCTGGTGGAGT	glnA	Glutamine synthetase/ glutamate--ammonialigase		<1..408	Nitrogen Metabolism	COG0174
199.1_147	CUST_691_PI442190719	HOT208_60m_824E10	199.1_147	TCCTTACTTAGCATTCTCTGTAATGATGCTTGCTGGTATTGATGGAAT	glnA	Glutamine synthetase/ glutamate--ammonialigase		<1..408	Nitrogen Metabolism	COG0174
199.1_147	CUST_692_PI442190719	HOT208_60m_824E10	199.1_147	TTAGCTGGTGGAGTATTTACTGAAGATTTTATTGATAATTTTATTG	glnA	Glutamine synthetase/ glutamate--ammonialigase		<1..408	Nitrogen Metabolism	COG0174
228.1_WP_079342201.:	CUST_1224_PI442190719	HOT208_60m_824E10	228.1_WP_079342201.:	GGGGAAAAGGTAAAAGTTTTAGCTAGTGAAGGTGGTCATGTTGAGTACTCCCCAAAGTCA	glk	Glucokinase	WP_079342201.:	46..1032	Glycolysis	COG0837
228.1_WP_079342201.:	CUST_1225_PI442190719	HOT208_60m_824E10	228.1_WP_079342201.:	TCTTCAAGAATTGTGCTATGGCGGGTTATGGATTTCTGGAGGAACAGCATCAAAACATTT	glk	Glucokinase	WP_079342201.:	46..1032	Glycolysis	COG0837
228.1_WP_079342201.:	CUST_1226_PI442190719	HOT208_60m_824E10	228.1_WP_079342201.:	CAAAATGGAATCCATGCTGAAGGATCTAATAATGATTTGCATGCCATTGTTGGAGCTGGG	glk	Glucokinase	WP_079342201.:	46..1032	Glycolysis	COG0837
228.1_WP_079342201.:	CUST_1227_PI442190719	HOT208_60m_824E10	228.1_WP_079342201.:	CCTTTCTAAAGAAGGGGATCAGCTAATGATTGAAGTTGAGCGGATTTGGTTAGGCGCTTA	glk	Glucokinase	WP_079342201.:	46..1032	Glycolysis	COG0837
228.1_WP_079342201.:	CUST_1228_PI442190719	HOT208_60m_824E10	228.1_WP_079342201.:	ATTATTAGTGGAACTGGTTTATCAAGAATTGCCGAATGGAGACTAAGCAAACCTGACGCC	glk	Glucokinase	WP_079342201.:	46..1032	Glycolysis	COG0837
228.1_WP_079342201.:	CUST_1229_PI442190719	HOT208_60m_824E10	228.1_WP_079342201.:	TCCCTATGAAAGTAATTTTAGATGAAGAGTTTGGACTTTTTAGTGCAGCCTGTAGAGCAA	glk	Glucokinase	WP_079342201.:	46..1032	Glycolysis	COG0837
229.1_WP_079342228.:	CUST_1393_PI442190719	HOT208_60m_824E10	229.1_WP_079342228.:	TACTGAACGTACCAACATATATTGCGAAGTGGATCCCGATACCTTTCCAAATTAGCGCTT	glcH	GPH family sugar transporter	WP_079342228.:	3665..5011	GlcH sugar transporter	COG2211
229.1_WP_079342228.:	CUST_1394_PI442190719	HOT208_60m_824E10	229.1_WP_079342228.:	ACCCAGAGAAACCGGCAGGACTATATACTGCTTGGATGGTACTTATTCAGAAGATTGGTA	glcH	GPH family sugar transporter	WP_079342228.:	3665..5011	GlcH sugar transporter	COG2211
229.1_WP_079342228.:	CUST_1395_PI442190719	HOT208_60m_824E10	229.1_WP_079342228.:	TCTTATTGGTATTGGAGCTTCAACCGCTTTTCTTATCCCTTGGTCACTACTTCCTGATGC	glcH	GPH family sugar transporter	WP_079342228.:	3665..5011	GlcH sugar transporter	COG2211
229.1_WP_079342228.:	CUST_1396_PI442190719	HOT208_60m_824E10	229.1_WP_079342228.:	TTCTTCTCTGGTGCGCCTTACAATTAATGCAAACAGTAGCGTTAATCTATGTCGAGGATG	glcH	GPH family sugar transporter	WP_079342228.:	3665..5011	GlcH sugar transporter	COG2211
229.1_WP_079342228.:	CUST_1397_PI442190719	HOT208_60m_824E10	229.1_WP_079342228.:	ACAGGATTTCAGCGTTAAACTATGGAGCGATTATGTGGATTATTTCATGTACAGCAGCTT	glcH	GPH family sugar transporter	WP_079342228.:	3665..5011	GlcH sugar transporter	COG2211
229.1_WP_079342228.:	CUST_1398_PI442190719	HOT208_60m_824E10	229.1_WP_079342228.:	AGCAAGAAGGCCTTCAGGAAAAGTTGAAGCTATAACACTTCAATTCAAAAGGATCTTCAG	glcH	GPH family sugar transporter	WP_079342228.:	3665..5011	GlcH sugar transporter	COG2211
229.1_WP_079342218.:	CUST_1470_PI442190719	HOT208_60m_824E10	229.1_WP_079342218.:	GTGCAGTAGTCGATGAGGCAATGGAAGGGGAAATACAAGTTACTGTAATTGCTACAGGAT	ftsZ	cell division protein FtsZ	WP_079342218.:	16487..17602	Cell Division	COG0206
229.1_WP_079342218.:	CUST_1471_PI442190719	HOT208_60m_824E10	229.1_WP_079342218.:	GCTGATGATGTTCTAAGGATGGGAGTCAAAGGCATTAGTGACATAATTACTTGCCCTGGA	ftsZ	cell division protein FtsZ	WP_079342218.:	16487..17602	Cell Division	COG0206
229.1_WP_079342218.:	CUST_1472_PI442190719	HOT208_60m_824E10	229.1_WP_079342218.:	GAATGCGTCAAGCAGAGGAGGGGATCGCAAGACTAGCTGAAAATGTTGATACTCTTATAG	ftsZ	cell division protein FtsZ	WP_079342218.:	16487..17602	Cell Division	COG0206
229.1_WP_079342218.:	CUST_1473_PI442190719	HOT208_60m_824E10	229.1_WP_079342218.:	TGTTGCAGAAGTAGCCAAACAAAGTGGGGCTCTAACAGTAGGTATAGTAACCAAACCATT	ftsZ	cell division protein FtsZ	WP_079342218.:	16487..17602	Cell Division	COG0206
229.1_WP_079342218.:	CUST_1474_PI442190719	HOT208_60m_824E10	229.1_WP_079342218.:	AGTTAATGTTGATTTTGCAGATGTAAGATCAGTTATGACGGAAGCTGGCACTGCTCTCCT	ftsZ	cell division protein FtsZ	WP_079342218.:	16487..17602	Cell Division	COG0206
229.1_WP_079342218.:	CUST_1475_PI442190719	HOT208_60m_824E10	229.1_WP_079342218.:	GGCTGCCAGAATTGATGGAGCTAAAGGCTGTGTTATAAATATTACTGGTGGCAAAGACAT	ftsZ	cell division protein FtsZ	WP_079342218.:	16487..17602	Cell Division	COG0206
232.1_WP_011862567.:	CUST_1922_PI442190719	HOT208_60m_824E10	232.1_WP_011862567.:	TATTCACGTATGGCATATGCCTGCACTCCTAGCGATTTTTGGAGATGATTCTTGTCTTCA	rbcL	Ribulose 1,5-bisphosphate carboxylase large subunit	WP_011862567.:	17379..18794	Carbon fixation	COG1850
232.1_WP_011862567.:	CUST_1923_PI442190719	HOT208_60m_824E10	232.1_WP_011862567.:	CTAACAGAGTTGCTTTAGAAGCTTGTGTAAAAGCACGTAATGCTGGTCGCGAAATCGAAA	rbcL	Ribulose 1,5-bisphosphate carboxylase large subunit	WP_011862567.:	17379..18794	Carbon fixation	COG1850
234.1_WP_025937384.:	CUST_2250_PI442190719	HOT208_60m_824E10	234.1_WP_025937384.:	ATCAAAAGAACTATTTCAGGAGAAATAAAATTCAAAAAAACTGATAAAGCTAATAAAGAT	minE	septum site-determining protein MinE	WP_025937384.:	12290..12616	Cell Division	COG0851
234.1_WP_025937384.:	CUST_2251_PI442190719	HOT208_60m_824E10	234.1_WP_025937384.:	GAGGTAGCAGTAAGTTTAGAGACTGAGGATAGAATGACTGCATTAGTTGCCAATTTACCA	minE	septum site-determining protein MinE	WP_025937384.:	12290..12616	Cell Division	COG0851
234.1_WP_025937384.:	CUST_2252_PI442190719	HOT208_60m_824E10	234.1_WP_025937384.:	AGTTCTTTAACAACTGATCTTTTAGATAAAATGAGGAAAGAAATTCTTGATGTTGTTGCT	minE	septum site-determining protein MinE	WP_025937384.:	12290..12616	Cell Division	COG0851
234.1_WP_025937384.:	CUST_2253_PI442190719	HOT208_60m_824E10	234.1_WP_025937384.:	GCAGAGAACCGTCTAGTGCCAATACAGCAAGAGAAAGATTACAACTTGTACTGGCTCATG	minE	septum site-determining protein MinE	WP_025937384.:	12290..12616	Cell Division	COG0851
234.1_WP_025937385.:	CUST_2254_PI442190719	HOT208_60m_824E10	234.1_WP_025937385.:	GTCTCGATCAGGCATTGGTTAGACATAAAAAGGAGCCTAATCTTGCTCTTCTACCTGCTG	minD	septum site-determining protein MinD	WP_025937385.:	12618..13433	Cell Division	COG2894
234.1_WP_025937385.:	CUST_2255_PI442190719	HOT208_60m_824E10	234.1_WP_025937385.:	AAGGGAGGGGTTGGTAAAACAACTTTAACTGCAAACCTAGGAATTGCACTTGCTAATAGC	minD	septum site-determining protein MinD	WP_025937385.:	12618..13433	Cell Division	COG2894
234.1_WP_025937385.:	CUST_2256_PI442190719	HOT208_60m_824E10	234.1_WP_025937385.:	AAAATGCCCTTGCAGCCTGTAAAGAAGCTATTGTTGTTACTAACCCAGAATTATCCGCAG	minD	septum site-determining protein MinD	WP_025937385.:	12618..13433	Cell Division	COG2894
234.1_WP_025937385.:	CUST_2257_PI442190719	HOT208_60m_824E10	234.1_WP_025937385.:	TCGCCCTAATATGATGGCTAGTCAAGAAATGTTATCCATTGAAGATGTTCAAGGGATCCT	minD	septum site-determining protein MinD	WP_025937385.:	12618..13433	Cell Division	COG2894
234.1_WP_025937385.:	CUST_2258_PI442190719	HOT208_60m_824E10	234.1_WP_025937385.:	AGGAGAGCCACTGACACTTTCAGATAGTAGATCTCCTGCAAAAAGATGTTACTTGAATGT	minD	septum site-determining protein MinD	WP_025937385.:	12618..13433	Cell Division	COG2894
234.1_WP_025937385.:	CUST_2259_PI442190719	HOT208_60m_824E10	234.1_WP_025937385.:	GGAGATCCAAGGATGTTGGATTGGATGAAGCCCGAAGATATGAAAAAAATTAGTGAGCT	minD	septum site-determining protein MinD	WP_025937385.:	12618..13433	Cell Division	COG2894
234.1_WP_079342331.:	CUST_2260_PI442190719	HOT208_60m_824E10	234.1_WP_079342331.:	TCCGCTAAGAAAAATATTTACGTTTGGGGCAAATTACTAGGGATCGCATTTGCAGGGAAA	minC	septum site-determining protein MinC	WP_079342331.:	13545..14207	Cell Division	COG0850
234.1_WP_079342331.:	CUST_2261_PI442190719	HOT208_60m_824E10	234.1_WP_079342331.:	ACCCTTTACAGTTAAGAATTGCAGATGTGATAGCAATTGGACCAAAGGAAAAGCCCAAAA	minC	septum site-determining protein MinC	WP_079342331.:	13545..14207	Cell Division	COG0850
234.1_WP_079342331.:	CUST_2262_PI442190719	HOT208_60m_824E10	234.1_WP_079342331.:	TCGGGTGAAAGAATATCTTCAAACGGAAACCTATGTATTATTGGAGACGTCAATCCAGGA	minC	septum site-determining protein MinC	WP_079342331.:	13545..14207	Cell Division	COG0850
234.1_WP_079342331.:	CUST_2263_PI442190719	HOT208_60m_824E10	234.1_WP_079342331.:	TTCGATCAAGGAACCTTTAGAAGCAAAAATCTTCGCAGTCAATGAGTCAATAAGTTCTCA	minC	septum site-determining protein MinC	WP_079342331.:	13545..14207	Cell Division	COG0850
234.1_WP_079342331.:	CUST_2264_PI442190719	HOT208_60m_824E10	234.1_WP_079342331.:	GTGGAAGGTCCTTAAAAATAGATTCAGCTTACATAAAAGAGCAAGAGATCAAAAATCAGT	minC	septum site-determining protein MinC	WP_079342331.:	13545..14207	Cell Division	COG0850
234.1_WP_079342331.:	CUST_2265_PI442190719	HOT208_60m_824E10	234.1_WP_079342331.:	TCCAGAAATTGCAGTAATAGATAAACAAACAATAATTATCAAACCTCACCTAATCGAAAA	minC	septum site-determining protein MinC	WP_079342331.:	13545..14207	Cell Division	COG0850
237.1_547	CUST_2676_PI442190719	HOT208_60m_824E10	237.1_547	CCAAAAACTGGAGTTATTTCTCATTCTGAAAAGTCCCTTGAGTATCCAGCTGGTGGTGGA	rbsK	Sugar kinase, ribokinase family		<1..546	Pentose Phosphate	COG0524
237.1_547	CUST_2677_PI442190719	HOT208_60m_824E10	237.1_547	TCTCTCAAATATGGGAATTAAGTTGCATGTGGCTTGGCGTGATAAACCAACTAGAAGAGG	rbsK	Sugar kinase, ribokinase family		<1..546	Pentose Phosphate	COG0524
237.1_547	CUST_2678_PI442190719	HOT208_60m_824E10	237.1_547	AATTGACTCTCAAGGTGAAAGAGCAATAACAGTTATTGGAGAAAGGTTAGCTCCAACTCA	rbsK	Sugar kinase, ribokinase family		<1..546	Pentose Phosphate	COG0524
237.1_547	CUST_2679_PI442190719	HOT208_60m_824E10	237.1_547	AGGAAATTTAAATTTTGCCGTTATTGGTCATGTTGAGTGGATAAATTTTTTAAAGGTTGA	rbsK	Sugar kinase, ribokinase family		<1..546	Pentose Phosphate	COG0524
237.1_547	CUST_2680_PI442190719	HOT208_60m_824E10	237.1_547	AGACAATTTAGAATGGAACATTTTAAAAAAAATGGACGGAATTTTTATTACTGCATCTGA	rbsK	Sugar kinase, ribokinase family		<1..546	Pentose Phosphate	COG0524
237.1_547	CUST_2681_PI442190719	HOT208_60m_824E10	237.1_547	ACCAAATTCATTTTTTTACGTCATTAGGTAATGATGATTATGGAGATAAGTGTTTCAAGA	rbsK	Sugar kinase, ribokinase family		<1..546	Pentose Phosphate	COG0524

252.1_WP_079342464.:	CUST_3542_PI442190719	HOT208_60m_824E10		252.1_WP_079342464.:	AAATGGGCTATTGGTATGGCTCATGATAGAGCCACTAGCAATCCTGAAATCTGGAGAAAA	pdhA	Pyruvate dehydrogenase E1 component alpha subunit	WP_079342464.:	9910..10983	Glycolysis/Krebs cycle	COG1071
252.1_WP_079342464.:	CUST_3543_PI442190719	HOT208_60m_824E10		252.1_WP_079342464.:	ATAAAAAGGAAGTGGCTGGTGATAAAACTAGTGATGCGGTAACAGCAGCATTCTTTGGTG	pdhA	Pyruvate dehydrogenase E1 component alpha subunit	WP_079342464.:	9910..10983	Glycolysis/Krebs cycle	COG1071
252.1_WP_079342464.:	CUST_3544_PI442190719	HOT208_60m_824E10		252.1_WP_079342464.:	TCATGTTCATGCACTAAGTGCCGGTGTTCCCTCATTTGAAGTAATGAGTGAACTTTTTGG	pdhA	Pyruvate dehydrogenase E1 component alpha subunit	WP_079342464.:	9910..10983	Glycolysis/Krebs cycle	COG1071
252.1_WP_079342464.:	CUST_3545_PI442190719	HOT208_60m_824E10		252.1_WP_079342464.:	CGAGATATCTGAGTCAGTCAAAAATGCTATTGAAGCTCCCGAACCCCCTTCAGAAGAATT	pdhA	Pyruvate dehydrogenase E1 component alpha subunit	WP_079342464.:	9910..10983	Glycolysis/Krebs cycle	COG1071
252.1_WP_079342464.:	CUST_3546_PI442190719	HOT208_60m_824E10		252.1_WP_079342464.:	GGCATTCTCTTGCAGATCCAGATGAACTAAGGTCTGAAAAAGAGAAGGAGTTTTGGGGTA	pdhA	Pyruvate dehydrogenase E1 component alpha subunit	WP_079342464.:	9910..10983	Glycolysis/Krebs cycle	COG1071
252.1_WP_079342464.:	CUST_3547_PI442190719	HOT208_60m_824E10		252.1_WP_079342464.:	CGAGGAGGATCCATGCACTTATTCTCAAAAGAGCATCACTTACTCGGAGGATATGCATTT	pdhA	Pyruvate dehydrogenase E1 component alpha subunit	WP_079342464.:	9910..10983	Glycolysis/Krebs cycle	COG1071
252.1_716	CUST_3599_PI442190719	HOT208_60m_824E10		252.1_716	TCATACTCATCCTACTCTTAGTGAAGTAGTTGAGGTCGCATATAAACAGGCAGCTTCTCA	lpdA	dihydrolipoyl dehydrogenase/pyruvate dehydrogenasae E3 component		19514..19863	Glycolysis/Krebs cycle	COG1249
252.1_716	CUST_3600_PI442190719	HOT208_60m_824E10		252.1_716	TGGGTTACATGCAGCTGATTTAATTCAAGAAATTTCAAACGCTATTTCAAGGAACCAAGA	lpdA	dihydrolipoyl dehydrogenase/pyruvate dehydrogenasae E3 component		19514..19863	Glycolysis/Krebs cycle	COG1249
252.1_716	CUST_3601_PI442190719	HOT208_60m_824E10		252.1_716	AGGCCAATTCAAAAGCATTGGCTGAATTAGAGAGTGAGGGATTGCTAAAGTTGATTTTCA	lpdA	dihydrolipoyl dehydrogenase/pyruvate dehydrogenasae E3 component		19514..19863	Glycolysis/Krebs cycle	COG1249
252.1_716	CUST_3602_PI442190719	HOT208_60m_824E10		252.1_716	CACCCAGAGATAAGTTCAGTTGGTCTCTCTGAAGTTGAAGCTAAAGAGATATCTACAAAA	lpdA	dihydrolipoyl dehydrogenase/pyruvate dehydrogenasae E3 component		19514..19863	Glycolysis/Krebs cycle	COG1249
254.1_WP_079342502.:	CUST_3867_PI442190719	HOT208_60m_824E10		254.1_WP_079342502.:	AAGAAGCAGGGGACTTATTATTAGAAGCAATTCAAAAAGCCGGATTTAAGCCTGGTGAGC	eno	Enolase	WP_079342502.:	9118..10410	Glycolysis/Gluconeogenesis	COG0148
254.1_WP_079342502.:	CUST_3868_PI442190719	HOT208_60m_824E10		254.1_WP_079342502.:	AGAGGACGGTTTAGCTGAGGATGATTGGGAGGGTTGGTCAGAATTAAACAAAGAATTAGG	eno	Enolase	WP_079342502.:	9118..10410	Glycolysis/Gluconeogenesis	COG0148
254.1_WP_079342502.:	CUST_3869_PI442190719	HOT208_60m_824E10		254.1_WP_079342502.:	GTGAGACTGAAGATACAACAATTGCAGATTTATCTGTCGCCACAAGATCGGGTCAGATCA	eno	Enolase	WP_079342502.:	9118..10410	Glycolysis/Gluconeogenesis	COG0148
254.1_WP_079342502.:	CUST_3870_PI442190719	HOT208_60m_824E10		254.1_WP_079342502.:	GAGGAGGAATTAGGAAATCAAGCAAGATTCGCTGGAGCTCTAGGTTTAGGTCCCAAAAAT	eno	Enolase	WP_079342502.:	9118..10410	Glycolysis/Gluconeogenesis	COG0148
254.1_WP_079342502.:	CUST_3871_PI442190719	HOT208_60m_824E10		254.1_WP_079342502.:	AATCATTACTTGATCAAAAAGGTCTATCTACTGCTGTAGGCGATGAGGGTGGATTTGCCC	eno	Enolase	WP_079342502.:	9118..10410	Glycolysis/Gluconeogenesis	COG0148
254.1_WP_079342502.:	CUST_3872_PI442190719	HOT208_60m_824E10		254.1_WP_079342502.:	GGAACATTAACTGAAACTTTGGAAGCTATTGAGTTAGCTAAAATGTCTGGTTTCACAAGT	eno	Enolase	WP_079342502.:	9118..10410	Glycolysis/Gluconeogenesis	COG0148
256.1_WP_079342516.:	CUST_3976_PI442190719	HOT208_60m_824E10		256.1_WP_079342516.:	TGGCTTCAAAGGCAAACTCAACTTAAATTTCAAAGGATATGCAGGACAAAGCTTTGGTGC	glsF	Ferredoxin-dependent glutamate synthase	WP_079342516.:	3917..8488	Nitrogen Metabolism	COG0069
256.1_WP_079342516.:	CUST_3977_PI442190719	HOT208_60m_824E10		256.1_WP_079342516.:	GGGAAAGTAGTTATACTAGGTTCCATAGGAAGGAATATTGGTGCAGGTATGACCGGTGGA	glsF	Ferredoxin-dependent glutamate synthase	WP_079342516.:	3917..8488	Nitrogen Metabolism	COG0069
256.1_WP_079342516.:	CUST_3978_PI442190719	HOT208_60m_824E10		256.1_WP_079342516.:	TAGGAAAATCGGGAGAAAGATTTGCGGTTAGAAATAGTGGTGCTACAGCGGTAACAGAAG	glsF	Ferredoxin-dependent glutamate synthase	WP_079342516.:	3917..8488	Nitrogen Metabolism	COG0069
256.1_WP_079342516.:	CUST_3979_PI442190719	HOT208_60m_824E10		256.1_WP_079342516.:	TACAGATAGAAGTGTTTGTGCGAAAATATCAGGCGAAATCGCAGAACTTCATGGCACAC	glsF	Ferredoxin-dependent glutamate synthase	WP_079342516.:	3917..8488	Nitrogen Metabolism	COG0069
256.1_WP_079342516.:	CUST_3980_PI442190719	HOT208_60m_824E10		256.1_WP_079342516.:	TGAAATCTCCTCCGAACAAGTTATTTTAGGAAACACCTGTCTTTATGGAGCAACAGGTGG	glsF	Ferredoxin-dependent glutamate synthase	WP_079342516.:	3917..8488	Nitrogen Metabolism	COG0069
256.1_WP_079342516.:	CUST_3981_PI442190719	HOT208_60m_824E10		256.1_WP_079342516.:	TGGTCTCATTTCAAGAGTACTTTCAAATTAATTGTTCCCCCAAGCGAAGAAGAGATGCTT	glsF	Ferredoxin-dependent glutamate synthase	WP_079342516.:	3917..8488	Nitrogen Metabolism	COG0069
266.1_WP_079342549.:	CUST_4274_PI442190719	HOT208_60m_824E10		266.1_WP_079342549.:	CGCGTATCTACAGAAGTTGATGCAAGACTGAGCTTTGACACAGAAGCTACGGTAGAAAAA	tal	Transaldolase	WP_079342549.:	9926..10927	Pentose Phosphate	COG0176
266.1_WP_079342549.:	CUST_4275_PI442190719	HOT208_60m_824E10		266.1_WP_079342549.:	AGATTGCTGCAACTTGGGAGGGAATTAAGGCAGCTGAAATTTTGGAAAAAGAGGGTATTA	tal	Transaldolase	WP_079342549.:	9926..10927	Pentose Phosphate	COG0176
266.1_WP_079342549.:	CUST_4276_PI442190719	HOT208_60m_824E10		266.1_WP_079342549.:	CCTGTGCCAATGCAAAGATAACTCTAATTTCTCCGTTCGTTGGCCGTATATTAGATTGGC	tal	Transaldolase	WP_079342549.:	9926..10927	Pentose Phosphate	COG0176
266.1_WP_079342549.:	CUST_4277_PI442190719	HOT208_60m_824E10		266.1_WP_079342549.:	AAACTGGTAAAACTAGTTTTGTTGGTGCTGAAGACCCTGGTGTTATTTCGGTAACGCAAA	tal	Transaldolase	WP_079342549.:	9926..10927	Pentose Phosphate	COG0176
266.1_WP_079342549.:	CUST_4278_PI442190719	HOT208_60m_824E10		266.1_WP_079342549.:	AAAGCTTAGTGAAGGTATTACTGGATTCAGTAAGGCTATAGAAGAATTGGAAGAGCTGCT	tal	Transaldolase	WP_079342549.:	9926..10927	Pentose Phosphate	COG0176
266.1_WP_079342549.:	CUST_4279_PI442190719	HOT208_60m_824E10		266.1_WP_079342549.:	AGCAGGTTGCGATCTTTTAACAATAGCACCAAAATTTCTTGAGGAACTGAAAAAAGAAAA	tal	Transaldolase	WP_079342549.:	9926..10927	Pentose Phosphate	COG0176
299.1_934	CUST_4716_PI442190719	HOT208_60m_824E10		299.1_934	TGATACCGTATTACCCTTATGTGACGGAGTAATGGTTGCAAGAGGTGATTTGGGAGTAGA	pykF	Pyruvate kinase		<1..414	Glycolysis	COG0469
299.1_934	CUST_4717_PI442190719	HOT208_60m_824E10		299.1_934	GTTGCAAATGCAATTCTGGATGGTACAGATGCTGTAATGCTTTCAAACGAAACTGCAGTT	pykF	Pyruvate kinase		<1..414	Glycolysis	COG0469
299.1_934	CUST_4718_PI442190719	HOT208_60m_824E10		299.1_934	ATAATTACAGCGACTCAAATGCTTGATTCTATGGCTTCGAATCCAAGACCAACTAGGGCT	pykF	Pyruvate kinase		<1..414	Glycolysis	COG0469
299.1_934	CUST_4719_PI442190719	HOT208_60m_824E10		299.1_934	ACTTAAAGCTATTGAAAGCCACTTACCTAGTACGATCCCAAATGCCATTAGTGCAGCAGT	pykF	Pyruvate kinase		<1..414	Glycolysis	COG0469
299.1_934	CUST_4720_PI442190719	HOT208_60m_824E10		299.1_934	GCGATTATCCTGTAGAGGCAGTAGAAACAATGGCAACCATAGCAAGAAGAATTGAAAGGG	pykF	Pyruvate kinase		<1..414	Glycolysis	COG0469
299.1_934	CUST_4721_PI442190719	HOT208_60m_824E10		299.1_934	GATGCCTGCAGAAGAAGTTCCTCTTTTACAAAAAGAATTAATAAGAAAAGCTAATTCGCT	pykF	Pyruvate kinase		<1..414	Glycolysis	COG0469
314.1_974	CUST_4931_PI442190719	HOT208_60m_824E10		314.1_974	AATAGTTTGTGAAGGTACAATCTTGAAGTCATGCAGTATCTTACATTGTGTTTTAGGGGT	glgC	Glucose-1-phosphate adenylyltransferase		<1..337	Glycogen Metabolism	COG0448
314.1_974	CUST_4932_PI442190719	HOT208_60m_824E10		314.1_974	TTTCCAATCTACACAAGACCAAGATTTCTCCCCCCTTCTAAACTCGTAGATGCTCAAATT	glgC	Glucose-1-phosphate adenylyltransferase		<1..337	Glycogen Metabolism	COG0448
314.1_974	CUST_4933_PI442190719	HOT208_60m_824E10		314.1_974	CGATGATTATTGGGAAGATATTGGAACCATTGGGGCATTCTTTGAGTCCAATCTTGCCTT	glgC	Glucose-1-phosphate adenylyltransferase		<1..337	Glycogen Metabolism	COG0448
314.1_974	CUST_4934_PI442190719	HOT208_60m_824E10		314.1_974	ACGGATTTTGGTAAGGACATAATTCCTGAAGCACTTGATAGGGGAGATACTCTTAAAAGT	glgC	Glucose-1-phosphate adenylyltransferase		<1..337	Glycogen Metabolism	COG0448
319.1_981	CUST_4969_PI442190719	HOT208_60m_824E10		319.1_981	AGTTTAAATCTTGCTAAAGTAATGGGAAGAGACGCCAGTGAATTTTTCGGTCCATATGCA	rbsK	Sugar kinase, ribokinase family		<1..407	Pentose Phosphate	COG0524
319.1_981	CUST_4970_PI442190719	HOT208_60m_824E10		319.1_981	GTTGATTCTTATGGATGTGGAGATTCTTTTGCCGCGGGTATTCTTTATGGAATGGCATCA	rbsK	Sugar kinase, ribokinase family		<1..407	Pentose Phosphate	COG0524
319.1_981	CUST_4971_PI442190719	HOT208_60m_824E10		319.1_981	ACCGAGGGAGAGAAGGGAGGCATACTATTCCCTAGAGGAAGATATAAGGCTCTAAAAAAC	rbsK	Sugar kinase, ribokinase family		<1..407	Pentose Phosphate	COG0524
319.1_981	CUST_4972_PI442190719	HOT208_60m_824E10		319.1_981	TCAAATGTTCTTATAGATGGATTAATAGGCAGTAATCTTGATCCCGGGGAAGTTTTTTCT	rbsK	Sugar kinase, ribokinase family		<1..407	Pentose Phosphate	COG0524
319.1_981	CUST_4973_PI442190719	HOT208_60m_824E10		319.1_981	TCAGAGATTTTTAAAATGGCTAGATCAGCTTCAATACTGTGTACAACACCAAGGGTAGGA	rbsK	Sugar kinase, ribokinase family		<1..407	Pentose Phosphate	COG0524
320.1_982	CUST_4974_PI442190719	HOT208_60m_824E10		320.1_982	AATTTCTGCATTATGTGAGGCTACTGGCGCAGATATAAATCATGTTGCATTAGCAGTGGG	ugd	UDP-glucose 6-dehydrogenase/nucleotide sugar dehydrogenase		1..491	Amino sugar and nucleotide sugar r	COG1004
320.1_982	CUST_4975_PI442190719	HOT208_60m_824E10		320.1_982	TGGCCTAACAGAGGTCGCAAATTATTGGCAAACAGTTATAGATATAAATCAATGGCAGCA	ugd	UDP-glucose 6-dehydrogenase/nucleotide sugar dehydrogenase		1..491	Amino sugar and nucleotide sugar r	COG1004
320.1_982	CUST_4976_PI442190719	HOT208_60m_824E10		320.1_982	AGATCTAATTGAAGAAGGGGCAACCCTCCACATATATGATCCCAAAGTTGAATATGAAAA	ugd	UDP-glucose 6-dehydrogenase/nucleotide sugar dehydrogenase		1..491	Amino sugar and nucleotide sugar r	COG1004
320.1_982	CUST_4977_PI442190719	HOT208_60m_824E10		320.1_982	TCTGACAAAAGATTAGGAAAATATTTTCTAAATTCTGGACCAGGGTTTGGAGGTAGTTGT	ugd	UDP-glucose 6-dehydrogenase/nucleotide sugar dehydrogenase		1..491	Amino sugar and nucleotide sugar r	COG1004
320.1_982	CUST_4978_PI442190719	HOT208_60m_824E10		320.1_982	TCGTCCAAAAACTATTTGGAACTGTTTCTGGTAAAAGATTGCAATTTTAGGTTTTGCAT	ugd	UDP-glucose 6-dehydrogenase/nucleotide sugar dehydrogenase		1..491	Amino sugar and nucleotide sugar r	COG1004
320.1_982	CUST_4979_PI442190719	HOT208_60m_824E10		320.1_982	AAAAGATTTAGATTCTTTTGAAATATCTTCAAGAAAATTTAATGATAAAGTTGGAGGATG	ugd	UDP-glucose 6-dehydrogenase/nucleotide sugar dehydrogenase		1..491	Amino sugar and nucleotide sugar r	COG1004
325.1_986	CUST_4997_PI442190719	HOT208_60m_824E10		325.1_986	AGTACTGCAAGGTTCACAAATACTGTCCATATAAAAGATGGTGCAAAGAGTATCAGCCTT	cbbA	Fructose-1,6-bisphosphate aldolase class II		<1..304	Glycolysis/Gluconeogenesis	COG0191
325.1_986	CUST_4998_PI442190719	HOT208_60m_824E10		325.1_986	TGGATTATTATTTGATGGAGTTAAATTTGAAAGGGTTTGTGTTAGAACAAGTAGCGTAGT	cbbA	Fructose-1,6-bisphosphate aldolase class II		<1..304	Glycolysis/Gluconeogenesis	COG0191
325.1_986	CUST_4999_PI442190719	HOT208_60m_824E10		325.1_986	TATGAAGCGAATGAATTGGCATCGGGAGAAAATGTTGTTTTCGCTGGAAGTGGAATTACT	cbbA	Fructose-1,6-bisphosphate aldolase class II		<1..304	Glycolysis/Gluconeogenesis	COG0191
325.1_986	CUST_5000_PI442190719	HOT208_60m_824E10		325.1_986	CCAGCAATCGCTCAAACTTCTGAATGGGCTGATTACACAAAAGAAGGAAATATAAAACGT	cbbA	Fructose-1,6-bisphosphate aldolase class II		<1..304	Glycolysis/Gluconeogenesis	COG0191
328.1_WP_079342614.:	CUST_5187_PI442190719	HOT208_60m_824E10		328.1_WP_079342614.:	TTGGTTACAGCCATAGTGCCAAAAGTTGGTTATGAAAAAGCAGCTGAAATTGCAAACCTT	fumC	class II fumarate hydratase	WP_079342614.:	6111..7496	Krebs cycle and related genes	COG0114
328.1_WP_079342614.:	CUST_5188_PI442190719	HOT208_60m_824E10		328.1_WP_079342614.:	GGTTTGTACTCAGATAATGGGTCTTGAATATGCAGTCTCAATGGCAAATTCTAGCGGCAC	fumC	class II fumarate hydratase	WP_079342614.:	6111..7496	Krebs cycle and related genes	COG0114
328.1_WP_079342614.:	CUST_5189_PI442190719	HOT208_60m_824E10		328.1_WP_079342614.:	AAATGAACCTGGAAGTTCTATTATGCCGGGTAAAGTTAATCCAACTCAATGCGAAGCCTT	fumC	class II fumarate hydratase	WP_079342614.:	6111..7496	Krebs cycle and related genes	COG0114
328.1_WP_079342614.:	CUST_5190_PI442190719	HOT208_60m_824E10		328.1_WP_079342614.:	CATGGCCTCGCATGATCGTCTTGCTCAAGTAATGAGTCAAATAAAAATATTAGCAGGTGT	fumC	class II fumarate hydratase	WP_079342614.:	6111..7496	Krebs cycle and related genes	COG0114
328.1_WP_079342614.:	CUST_5191_PI442190719	HOT208_60m_824E10		328.1_WP_079342614.:	TCTGAATGAATTGTATTTCTTACCTCTGGGAGGAACTGCGGTTGGTACGGGGATTAATTG	fumC	class II fumarate hydratase	WP_079342614.:	6111..7496	Krebs cycle and related genes	COG0114
328.1_WP_079342614.:	CUST_5192_PI442190719	HOT208_60m_824E10		328.1_WP_079342614.:	TCCCCTTACTTTTGGGCAAGAAATATCAGGATGGTCAGAGCAACTTAAAGATGCTGAGAA	fumC	class II fumarate hydratase	WP_079342614.:	6111..7496	Krebs cycle and related genes	COG0114
035.1_WP_025972114.:	CUST_6077_PI442190719	HOT208_60m_824E10		035.1_WP_025972114.:	TATTGGTGCAGAATTAATTGCCTTGATAACAGAAGAGTGCTTTGATGACCTTGATGCCCG	pdhB	Pyruvate dehydrogenase E1 component beta subunit	WP_025972114.:	650..1633	Glycolysis/Krebs cycle	COG0022
035.1_WP_025972114.:	CUST_6078_PI442190719	HOT208_60m_824E10		035.1_WP_025972114.:	CAACTTGGTGCTGAGCATAGTCAAAGACTTGAAGCATATTTTCATGCAGTTCCTGGCATA	pdhB	Pyruvate dehydrogenase E1 component beta subunit	WP_025972114.:	650..1633	Glycolysis/Krebs cycle	COG0022
035.1_WP_025972114.:	CUST_6079_PI442190719	HOT208_60m_824E10		035.1_WP_025972114.:	TGGGTGCAGCAATGACTGGCTTAAGACCAATAGTAGAAGGAATGAATATGGGTTTTTTGC	pdhB	Pyruvate dehydrogenase E1 component beta subunit	WP_025972114.:	650..1633	Glycolysis/Krebs cycle	COG0022
035.1_WP_025972114.:	CUST_6080_PI442190719	HOT208_60m_824E10		035.1_WP_025972114.:	TTTGTCTGAAGAATTACCTGAGGGTGATTATACTTGCGCTTTAGATCAGGCTGACGTTGT	pdhB	Pyruvate dehydrogenase E1 component beta subunit	WP_025972114.:	650..1633	Glycolysis/Krebs cycle	COG0022
035.1_WP_025972114.:	CUST_6081_PI442190719	HOT208_60m_824E10		035.1_WP_025972114.:	ATACAAGTGGCGGAAATTATAAAATACCAGCAGTAGTTAGAGGACCTGGTGGAGTTGGTC	pdhB	Pyruvate dehydrogenase E1 component beta subunit	WP_025972114.:	650..1633	Glycolysis/Krebs cycle	COG0022
035.1_WP_025972114.:	CUST_6082_PI442190719	HOT208_60m_824E10		035.1_WP_025972114.:	TGTTGCATGTAGTACGCCTACAAATGCTAAAGGTTTAATGAAAGCAGCTATAAGAGATGA	pdhB	Pyruvate dehydrogenase E1 component beta subunit	WP_025972114.:	650..1633	Glycolysis/Krebs cycle	COG0022
037.1_WP_011863567.:	CUST_6330_PI442190719	HOT208_60m_824E10		037.1_WP_011863567.:	GTTCCTAGGATTATTTACTAGTGCAATTCAGGCATTGATTTTCGCAACTTTGGCGGCCTA	atpI	ATP synthase subunit A	WP_011863567.:	1583..2308	Energy Metabolism	COG0356
037.1_WP_011863567.:	CUST_6331_PI442190719	HOT208_60m_824E10		037.1_WP_011863567.:	AAAGGCCTTCAAAACCTGCTTGAGTTTCTCTGGGATTACATTAGAGATCTTGCTAGGACG	atpI	ATP synthase subunit A	WP_011863567.:	1583..2308	Energy Metabolism	COG0356
037.1_WP_011863567.:	CUST_6332_PI442190719	HOT208_60m_824E10		037.1_WP_011863567.:	ACCAAGTGGAGAATTAGGAGCACCTACTGCAGATATTAATACAACGATAGCCTTGGCTTT	atpI	ATP synthase subunit A	WP_011863567.:	1583..2308	Energy Metabolism	COG0356
037.1_WP_011863567.:	CUST_6333_PI442190719	HOT208_60m_824E10		037.1_WP_011863567.:	TGCAGGTTTAAGCAATAAGGGTTGGAGATACTTCGAATATTATGTTCACCCAACTCCGAT	atpI	ATP synthase subunit A	WP_011863567.:	1583..2308	Energy Metabolism	COG0356
037.1_WP_011863567.:	CUST_6334_PI442190719	HOT208_60m_824E10		037.1_WP_011863567.:	AGCATTAGAAGTTGGTCAACATCTTTATTGGCAAATTGGAAATATCAGACTTCATGGGCA	atpI	ATP synthase subunit A	WP_011863567.:	1583..2308	Energy Metabolism	COG0356
037.1_WP_011863567.:	CUST_6335_PI442190719	HOT208_60m_824E10		037.1_WP_011863567.:	ATGCTTCCTTTCAAAATTCTAGAGGACTTTACGAAACCTTTATCACTCTCTTTCAGGCTA	atpI	ATP synthase subunit A	WP_011863567.:	1583..2308	Energy Metabolism	COG0356
037.1_WP_002805169.:	CUST_6336_PI442190719	HOT208_60m_824E10		037.1_WP_002805169.:	TGGAGTCATTAACAATTACGGATTAGTTGTGGCTTTGGTACTACTTTTTGCGAATCCTT	atpE	ATP synthase subunit C	WP_002805169.:	2473..2718	Energy Metabolism	COG0636
037.1_WP_002805169.:	CUST_6337_PI442190719	HOT208_60m_824E10		037.1_WP_002805169.:	GCCCGTCAGCCAGAAGCTGAAGGTAAAATCAGAGGAACTCTTCTTTTATCTTTTGCTTTC	atpE	ATP synthase subunit C	WP_002805169.:	2473..2718	Energy Metabolism	COG0636
037.1_WP_002805169.:	CUST_6338_PI442190719	HOT208_60m_824E10		037.1_WP_002805169.:	TAGGTGCTATTGGCCCAGGTCTTGGACAAGGTAACGCAGCTCAAGG	atpE	ATP synthase subunit C	WP_002805169.:	2473..2718	Energy Metabolism	COG0636
037.1_WP_002805169.:	CUST_6339_PI442190719	HOT208_60m_824E10		037.1_WP_002805169.:	GATTACTTCCGCTGCATCAGTTGTAGCTGCTGGTTTAGCAGTTGGTCTAGGTGCTATTG	atpE	ATP synthase subunit C	WP_002805169.:	2473..2718	Energy Metabolism	COG0636
037.1_WP_079341916.:	CUST_6340_PI442190719	HOT208_60m_824E10		037.1_WP_079341916.:	AGACGTCTGCTGCTCGTGATCAACTTTCTAAACAGGCTGATGATCTAAGCGAACTTATTG	atpF	ATP synthase subunit B	WP_079341916.:	2787..3248	Energy Metabolism	COG0711
037.1_WP_079341916.:	CUST_6341_PI442190719	HOT208_60m_824E10		037.1_WP_079341916.:	AGGTTGAAAAATTAGAAGCTGATTTATTAGCTCAGCTTCAAAGTGCTCGTGCAGAAGCCC	atpF	ATP synthase subunit B	WP_079341916.:	2787..3248	Energy Metabolism	COG0711
037.1_WP_079341916.:	CUST_6342_PI442190719	HOT208_60m_824E10		037.1_WP_079341916.:	TGGTGCTACAGAAGGTGGATTATTTGATATAAATGCCACTTTGCCACTAATGGCGATACA	atpF	ATP synthase subunit B	WP_079341916.:	2787..3248	Energy Metabolism	COG0711
037.1_WP_079341916.:	CUST_6343_PI442190719	HOT208_60m_824E10		037.1_WP_079341916.:	AAGCGAAGCTGAAAATGAGTCTGACAAGCTTTATAAAGAAGCACTAGAACTTGCTAACAA	atpF	ATP synthase subunit B	WP_079341916.:	2787..3248	Energy Metabolism	COG0711
037.1_WP_079341916.:	CUST_6344_PI442190719	HOT208_60m_824E10		037.1_WP_079341916.:	AGTAGTTGCACTTACTTACATATTAAATTCTCTCTTTTTTAAGCCTGTTGGCAATGTTGT	atpF	ATP synthase subunit B	WP_079341916.:	2787..3248	Energy Metabolism	COG0711
037.1_WP_079341916.:	CUST_6345_PI442190719	HOT208_60m_824E10		037.1_WP_079341916.:	TAGAAAAAAGAGAAAAGTTTGTAAGTAATAATATTATTGAGGCCAAAAATAAACTTTCT	atpF	ATP synthase subunit B	WP_079341916.:	2787..3248	Energy Metabolism	COG0711
037.1_WP_025882323.:	CUST_6346_PI442190719	HOT208_60m_824E10		037.1_WP_025882323.:	ATCTCTGATGAGAGCTCTGAAGCATCCAGAGCAATTTCTCAACTTCGAAAAGAAGCTGTT	atpF	ATP synthase subunit B	WP_025882323.:	3245..3757	Energy Metabolism	COG0711
037.1_WP_025882323.:	CUST_6347_PI442190719	HOT208_60m_824E10		037.1_WP_025882323.:	TTTTTGCCTGGTTTCCTAGGTAAAATGCTTCAAAAAAGGAGAGAAGGAATCCTTCTTGAA	atpF	ATP synthase subunit B	WP_025882323.:	3245..3757	Energy Metabolism	COG0711
037.1_WP_025882323.:	CUST_6348_PI442190719	HOT208_60m_824E10		037.1_WP_025882323.:	ATCAGAATGGAAAGTGAGAAAAAAGCTATAGAGGAGATGGCACGAATTAAACAAAGTGCA	atpF	ATP synthase subunit B	WP_025882323.:	3245..3757	Energy Metabolism	COG0711
037.1_WP_025882323.:	CUST_6349_PI442190719	HOT208_60m_824E10		037.1_WP_025882323.:	ACTGGCAATTAAGAAAGCCTTAGATTCTCTCCCAAATCGACTTGATAAAACAACACAAGA	atpF	ATP synthase subunit B	WP_025882323.:	3245..3757	Energy Metabolism	COG0711
037.1_WP_025882323.:	CUST_6350_PI442190719	HOT208_60m_824E10		037.1_WP_025882323.:	TGCAACTCAAGCTTTAGAAAAAGCGAAGAAAGATTTATCTTCAGCAGAAGAAAAAGCTTC	atpF	ATP synthase subunit B	WP_025882323.:	3245..3757	Energy Metabolism	COG0711
037.1_WP_025882323.:	CUST_6351_PI442190719	HOT208_60m_824E10		037.1_WP_025882323.:	GCTACAGAAGGTTTTGGATTGAACTTCAATTTATTCGAAACTAATATCCTTAATTGGGCT	atpF	ATP synthase subunit B	WP_025882323.:	3245..3757	Energy Metabolism	COG0711
386.1_WP_079342747.:	CUST_6645_PI442190719	HOT208_60m_824E10		386.1_WP_079342747.:	GGATATTTTCAAGATTCCATTATTCTCGCCACTGGAGCAACCGGTACGGGTAAAACAATG	kaiC	circadian clock protein KaiC	WP_079342747.:	2802..4331	Circadian Rhythm	COG0467
386.1_WP_079342747.:	CUST_6646_PI442190719	HOT208_60m_824E10		386.1_WP_079342747.:	AAGGTGAGATGGCACGAGCTTTAAATGTTTTTAAAATGCGGGGATCATGGCATGATAAAC	kaiC	circadian clock protein KaiC	WP_079342747.:	2802..4331	Circadian Rhythm	COG0467
386.1_WP_079342747.:	CUST_6647_PI442190719	HOT208_60m_824E10		386.1_WP_079342747.:	AGGGATTCTTTTTCTAATTTTGAACAAATATTTAGTGGTGCGCCTCACAGAGTTGTGCCT	kaiC	circadian clock protein KaiC	WP_079342747.:	2802..4331	Circadian Rhythm	COG0467
386.1_WP_079342747.:	CUST_6648_PI442190719	HOT208_60m_824E10		386.1_WP_079342747.:	ACTTTTTGCATATGAAGAATCTAGGGCTCAATTGCTTAGAAATGCTACTAGCTGGGGTAT	kaiC	circadian clock protein KaiC	WP_079342747.:	2802..4331	Circadian Rhythm	COG0467
386.1_WP_079342747.:	CUST_6649_PI442190719	HOT208_60m_824E10		386.1_WP_079342747.:	TGGCAATTGATTCTCTCTCTGCATTAGCTAGAGGTGTAAGTTTGAATGCATTTAGACAGT	kaiC	circadian clock protein KaiC	WP_079342747.:	2802..4331	Circadian Rhythm	COG0467
386.1_WP_079342747.:	CUST_6650_PI442190719	HOT208_60m_824E10		386.1_WP_079342747.:	TGCTGTTACCGGTTATACAAAACAAGAAGAAATAGCAGGCTTTTTACTAATACTGCAGA	kaiC	circadian clock protein KaiC	WP_079342747.:	2802..4331	Circadian Rhythm	COG0467
386.1_WP_002807968.:	CUST_6651_PI442190719	HOT208_60m_824E10		386.1_WP_002807968.:	ACTTTTTGATGAATTAACTGAAACTGAATATAGTGGAGATAAATA	kaiB	Circadian clock protein KaiB	WP_002807968.:	4391..4708	Circadian Rhythm	COG4251
386.1_WP_002807968.:	CUST_6652_PI442190719	HOT208_60m_824E10		386.1_WP_002807968.:	AGGTGATCTTTCAGATAGAGAGAAAGTTTTAATTGGTTTAGATCTACTTTTTGATGAATT	kaiB	Circadian clock protein KaiB	WP_002807968.:	4391..4708	Circadian Rhythm	COG4251
386.1_WP_002807968.:	CUST_6653_PI442190719	HOT208_60m_824E10		386.1_WP_002807968.:	TGCAGAAGAAGATAAAATTCTTGCTACTCCAACGTTAGCTAAAATTTTACCGCCACCAGT	kaiB	Circadian clock protein KaiB	WP_002807968.:	4391..4708	Circadian Rhythm	COG4251
386.1_WP_002807968.:	CUST_6654_PI442190719	HOT208_60m_824E10		386.1_WP_002807968.:	AAATGAATTCAAAGGAGTTTATGCTTTAAAGGTTATCGATGTACTTAAGCAACCACAACT	kaiB	Circadian clock protein KaiB	WP_002807968.:	4391..4708	Circadian Rhythm	COG4251
386.1_WP_002807968.:	CUST_6655_PI442190719	HOT208_60m_824E10		386.1_WP_002807968.:	TGGCTGCAAGGAAAACATATATTTTAAAACTCTATGTTGCTGGAAATACTCCTAATTCGA	kaiB	Circadian clock protein KaiB	WP_002807968.:	4391..4708	Circadian Rhythm	COG4251
388.1_WP_079342764.:	CUST_6820_PI442190719	HOT208_60m_824E10		388.1_WP_079342764.:	ATCCCGGTCAAGCAAATTATTCTGCAGCTAAAGCTGGAGTTATTGGATTCACCAAAACTT	gdh	3-oxoacyl-acyl-carrier-protein reductase	WP_079342764.:	172..921	Entner-Dudoroff	COG1028
388.1_WP_079342764.:	CUST_6821_PI442190719	HOT208_60m_824E10		388.1_WP_079342764.:	TTCAAGTGATGCAGGAAGTTACATTACAGGGCAAACAATCAGTATAGACGGGGGTATGAG	gdh	3-oxoacyl-acyl-carrier-protein reductase	WP_079342764.:	172..921	Entner-Dudoroff	COG1028
388.1_WP_079342764.:	CUST_6822_PI442190719	HOT208_60m_824E10		388.1_WP_079342764.:	TGCTGGCATCACTAGAGATGGACTATTGATGAGAATGAAATCGGAACAATGGGATGATGT	gdh	3-oxoacyl-acyl-carrier-protein reductase	WP_079342764.:	172..921	Entner-Dudoroff	COG1028
388.1_WP_079342764.:	CUST_6823_PI442190719	HOT208_60m_824E10		388.1_WP_079342764.:	CAAGCAGAGGAATTGGTAAAGAAATTGCTTTAGAACTAAGCCGCTCAGGAGCAGAAGTTT	gdh	3-oxoacyl-acyl-carrier-protein reductase	WP_079342764.:	172..921	Entner-Dudoroff	COG1028
388.1_WP_079342764.:	CUST_6824_PI442190719	HOT208_60m_824E10		388.1_WP_079342764.:	TGCTTCAAGAGGTATAAACGTAAATGCAATAGCTCCAGGTTTTATAGAAACAGAAATGAC	gdh	3-oxoacyl-acyl-carrier-protein reductase	WP_079342764.:	172..921	Entner-Dudoroff	COG1028
388.1_WP_079342764.:	CUST_6825_PI442190719	HOT208_60m_824E10		388.1_WP_079342764.:	ACTCTTCTTCTGATGAAAAAGCTGAAGAAGTTGTAAGTTCAATAAAAAATTCGGGAGGTA	gdh	3-oxoacyl-acyl-carrier-protein reductase	WP_079342764.:	172..921	Entner-Dudoroff	COG1028
389.1_WP_025931179.:	CUST_6844_PI442190719	HOT208_60m_824E10		389.1_WP_025931179.:	TGTTCCTTTTAGGACCGCTTATCAAGTTGTTGGTCAAATGGTTAAATATTGCTTGGAGAG	argH	Argininosuccinate lyase	WP_025931179.:	1094..2473	Amino Acid metabolism	COG0165
389.1_WP_025931179.:	CUST_6845_PI442190719	HOT208_60m_824E10		389.1_WP_025931179.:	GTGAAGGTGGTACAGGTTTTGTTCAGGTAGAAAAAGAGGTAAATAATTGGCAAAAAAGGT	argH	Argininosuccinate lyase	WP_025931179.:	1094..2473	Amino Acid metabolism	COG0165
389.1_WP_025931179.:	CUST_6847_PI442190719	HOT208_60m_824E10		389.1_WP_025931179.:	AAATTTCATCCCGAATTTGATGAGGATGTTTTTGAGGATCTTAAACCTCTTAATGTCGTT	argH	Argininosuccinate lyase	WP_025931179.:	1094..2473	Amino Acid metabolism	COG0165
402.1_1363	CUST_6962_PI442190719	HOT208_60m_824E10		402.1_1363	TCAAAAAGCATTAATAGCACGAGCTCAAGCAAACTCTGAAGCTTCAAAAGGTGCCTATGT	fda/cbbA	fructose-1,6-bisphosphatealdolase		<3..492	Glycolysis/Gluconeogenesis	COG0191
402.1_1363	CUST_6963_PI442190719	HOT208_60m_824E10		402.1_1363	GAAGGTTGCTGAAATGACAATTAGAACTATGGAAAGATGCGTTCCTGCATCTGTTCCTGG	fda/cbbA	fructose-1,6-bisphosphatealdolase		<3..492	Glycolysis/Gluconeogenesis	COG0191
402.1_1363	CUST_6964_PI442190719	HOT208_60m_824E10		402.1_1363	TATGGAATGTTTCATTCTCCTATGGAAGAGCATTACAGCACTCATGCTTAAAGGCCTGGA	fda/cbbA	fructose-1,6-bisphosphatealdolase		<3..492	Glycolysis/Gluconeogenesis	COG0191
402.1_1363	CUST_6965_PI442190719	HOT208_60m_824E10		402.1_1363	AACTGCTGAAGTTCAAGAAGAGGTAATAAAGCAGGTTTATAGTGCCTGCCAAGCAAATGG	fda/cbbA	fructose-1,6-bisphosphatealdolase		<3..492	Glycolysis/Gluconeogenesis	COG0191
402.1_1363	CUST_6966_PI442190719	HOT208_60m_824E10		402.1_1363	AGGAACTCTATTAAAACCTTCTATGACTGTTAATGGAGCAGATTGTCCAACAAAGGCTGA	fda/cbbA	fructose-1,6-bisphosphatealdolase		<3..492	Glycolysis/Gluconeogenesis	COG0191
402.1_1363	CUST_6967_PI442190719	HOT208_60m_824E10		402.1_1363	GGAGGGTTAAGCGAAGAAGCTGCTTCTGTTTATCTAAATAATATGAACACTCTTGACAGG	fda/cbbA	fructose-1,6-bisphosphatealdolase		<3..492	Glycolysis/Gluconeogenesis	COG0191
047.1_1428	CUST_7299_PI442190719	HOT208_60m_824E10		047.1_1428	AATGGGAAATATTTCCTTGAGGCAGGGCCAGGATTTGGAGGAAGTTGCTTTAAAAAAGAT	ugd	UDP-glucose 6-dehydrogenase/nucleotide sugar dehydrogenase		18..643	Amino sugar and nucleotide sugar r	COG1004
047.1_1428	CUST_7300_PI442190719	HOT208_60m_824E10		047.1_1428	AGGGAGAAACCCAAAATCAGAATTTGAAATTAATCTTGAACAGGGTTCCTGGTCGCTAGA	ugd	UDP-glucose 6-dehydrogenase/nucleotide sugar dehydrogenase		18..643	Amino sugar and nucleotide sugar r	COG1004
047.1_1428	CUST_7301_PI442190719	HOT208_60m_824E10		047.1_1428	ACTTTGGCCTACCAGAAGTTGCGACATATTGGGAACAAGTTTTAAGTTTAAATGACTGGC	ugd	UDP-glucose 6-dehydrogenase/nucleotide sugar dehydrogenase		18..643	Amino sugar and nucleotide sugar r	COG1004
047.1_1428	CUST_7302_PI442190719	HOT208_60m_824E10		047.1_1428	ATTGGGATTCTTGGATTTGCATTTAAAGCGAATACAAACGATACAAGAGAGTCTTCCGCA	ugd	UDP-glucose 6-dehydrogenase/nucleotide sugar dehydrogenase		18..643	Amino sugar and nucleotide sugar r	COG1004
047.1_1428	CUST_7303_PI442190719	HOT208_60m_824E10		047.1_1428	CTGATGCAGTGTTAATTCTTACTGACTGGGAAGCGTACAAGAATTTTAATTGGAAGAAGT	ugd	UDP-glucose 6-dehydrogenase/nucleotide sugar dehydrogenase		18..643	Amino sugar and nucleotide sugar r	COG1004
047.1_1428	CUST_7304_PI442190719	HOT208_60m_824E10		047.1_1428	TCAGAAGGGGCAAACATTATTATTCATGATCCAAAAGTAAATGAAAGACAGATTGCTAAA	ugd	UDP-glucose 6-dehydrogenase/nucleotide sugar dehydrogenase		18..643	Amino sugar and nucleotide sugar r	COG1004
086.1_1512	CUST_7735_PI442190719	HOT208_60m_824E10		086.1_1512	TTCACTAGGTGCCTTAATCGCTTTATTTGAGAGGGCTGTATCCTTCTATGCTGAATTGGT	pgi	Glucose-6-phosphate isomerase		<1..434	Glycolysis/Gluconeogenesis	COG0166
086.1_1512	CUST_7736_PI442190719	HOT208_60m_824E10		086.1_1512	ATGCATATGATCAACCTGGAGTTGAAGCTGGAAAGAAAGCAGCTGCAAATATTATTGAGT	pgi	Glucose-6-phosphate isomerase		<1..434	Glycolysis/Gluconeogenesis	COG0166
086.1_1512	CUST_7737_PI442190719	HOT208_60m_824E10		086.1_1512	ACTCCGTGAAATGTGTTTCGGTAATGATAATTATTTAGTTAAGGGCGATTGGTCAAATCC	pgi	Glucose-6-phosphate isomerase		<1..434	Glycolysis/Gluconeogenesis	COG0166
086.1_1512	CUST_7738_PI442190719	HOT208_60m_824E10		086.1_1512	AGGAACCAGATCAGCACTCTCTAGTGAAAACAGACAAAGTATCACTATTACGTTAGAAAA	pgi	Glucose-6-phosphate isomerase		<1..434	Glycolysis/Gluconeogenesis	COG0166
086.1_1512	CUST_7739_PI442190719	HOT208_60m_824E10		086.1_1512	GGTGGAGAATATTCTATAAATGACATAACATCATTATTTGATAATTCATTTAGTGAACCT	pgi	Glucose-6-phosphate isomerase		<1..434	Glycolysis/Gluconeogenesis	COG0166
086.1_1512	CUST_7740_PI442190719	HOT208_60m_824E10		086.1_1512	ATTGGTCAAATCCAAATTCATTAGTTATTCAAAAAATAAATTCTTAA	pgi	Glucose-6-phosphate isomerase		<1..434	Glycolysis/Gluconeogenesis	COG0166
014.1_WP_079293075.:	CUST_584_PI442190711	HOT208_60m_824C06		014.1_WP_079293075.:	ATGAATTGGCATCGGGAGAAAATGTTGTTTTCGCTGGAAGTGGAATAACTGATGGGTTAT	cbbA	Fructose-1,6-bisphosphate aldolase class II	WP_079293075.:	5732..6733	Glycolysis/Gluconeogenesis	COG0191
014.1_WP_079293075.:	CUST_585_PI442190711	HOT208_60m_824C06		014.1_WP_079293075.:	TGCCTCTGATACGGGTGGTCTTTTCAATGCTCCTGATTTTTACATGAACAAATTAGCAGC	cbbA	Fructose-1,6-bisphosphate aldolase class II	WP_079293075.:	5732..6733	Glycolysis/Gluconeogenesis	COG0191
014.1_WP_079293075.:	CUST_586_PI442190711	HOT208_60m_824C06		014.1_WP_079293075.:	GACACTTTCAAGGACAACTAGTTTATGATCCAGCAATCGCTCAAACTTCTGAATGGGCTG	cbbA	Fructose-1,6-bisphosphate aldolase class II	WP_079293075.:	5732..6733	Glycolysis/Gluconeogenesis	COG0191
014.1_WP_079293075.:	CUST_587_PI442190711	HOT208_60m_824C06		014.1_WP_079293075.:	CCAGGGGTTGACTTCGCAGTAGATCCTTGTGAAGGAACTAATCTTTGTGCGAATAATCAA	cbbA	Fructose-1,6-bisphosphate aldolase class II	WP_079293075.:	5732..6733	Glycolysis/Gluconeogenesis	COG0191
014.1_WP_079293075.:	CUST_588_PI442190711	HOT208_60m_824C06		014.1_WP_079293075.:	TAAAGAGATTCGAGGATGTGGTGCAAAAGTACAACCGATTTCTGATGGTGATGTTCAAGC	cbbA	Fructose-1,6-bisphosphate aldolase class II	WP_079293075.:	5732..6733	Glycolysis/Gluconeogenesis	COG0191
014.1_WP_079293075.:	CUST_589_PI442190711	HOT208_60m_824C06		014.1_WP_079293075.:	GCGCCTCCTGCGGCCAAAGGTAAAGTTGATATTAGAAATTCAGCTACTGAAAACTTGA	cbbA	Fructose-1,6-bisphosphate aldolase class II	WP_079293075.:	5732..6733	Glycolysis/Gluconeogenesis	COG0191
142.1_WP_079294591.:	CUST_772_PI442190711	HOT208_60m_824C06		142.1_WP_079294591.:	CTAAAGTAATGGGAAGAGACGCCAGTGAATTTTTCGGCCCATATGCAAATAGCGATGAAA	rbsK	Sugar kinase, ribokinase family	WP_079294591.:	5775..6644	Pentose Phosphate	COG0524
142.1_WP_079294591.:	CUST_773_PI442190711	HOT208_60m_824C06		142.1_WP_079294591.:	GTCATTTCTCATTCTGAAAAGTCCCTTGAGTATCCAGCTGGTGGTGGATCTATTATCGCG	rbsK	Sugar kinase, ribokinase family	WP_079294591.:	5775..6644	Pentose Phosphate	COG0524
142.1_WP_079294591.:	CUST_774_PI442190711	HOT208_60m_824C06		142.1_WP_079294591.:	AACCGAGGGAGAGAAGGGAGGCATAATATTCCCAGGAGGAAGATATAAGGCTCTTAAAAA	rbsK	Sugar kinase, ribokinase family	WP_079294591.:	5775..6644	Pentose Phosphate	COG0524
142.1_WP_079294591.:	CUST_775_PI442190711	HOT208_60m_824C06		142.1_WP_079294591.:	ATTCTTATGGCTGTGGCGATTCTTTTGCTGCTGGGATTCTTTATGGAATGGCATCTAAAT	rbsK	Sugar kinase, ribokinase family	WP_079294591.:	5775..6644	Pentose Phosphate	COG0524
142.1_WP_079294591.:	CUST_776_PI442190711	HOT208_60m_824C06		142.1_WP_079294591.:	CTCTCAAATATGGGAATTAAGTTGCATGTAGCTTGGCGCGATAAACCTAGTAGAAGAGGA	rbsK	Sugar kinase, ribokinase family	WP_079294591.:	5775..6644	Pentose Phosphate	COG0524
142.1_WP_079294591.:	CUST_777_PI442190711	HOT208_60m_824C06		142.1_WP_079294591.:	AATTGACTCTCAAGGTGAAAGAGCAATAACAGTTATTGGAGAAAGGTTAGCTCCTACTCA	rbsK	Sugar kinase, ribokinase family	WP_079294591.:	5775..6644	Pentose Phosphate	COG0524
149.1_WP_079292544.:	CUST_2060_PI442190711	HOT208_60m_824C06		149.1_WP_079292544.:	AAATTCAGACCACCAACACCTATCTTGGCAACTACTACAGAGAGAAGTGTAGCGAGAAGA	pykF	Pyruvate kinase	WP_079292544.:	4470..6260	Glycolysis	COG0469
149.1_WP_079292544.:	CUST_2061_PI442190711	HOT208_60m_824C06		149.1_WP_079292544.:	AACTTGATGCAGGAGCTATCATCCCTTTAACAAAATCAGGCTCTACCGCTCGAAATGTAA	pykF	Pyruvate kinase	WP_079292544.:	4470..6260	Glycolysis	COG0469
149.1_WP_079292544.:	CUST_2062_PI442190711	HOT208_60m_824C06		149.1_WP_079292544.:	CCACTAAAAGCTATTGAGAGCCACTTACCTAGTACCATCCCAAATGCTATTAGTGCAGCA	pykF	Pyruvate kinase	WP_079292544.:	4470..6260	Glycolysis	COG0469
149.1_WP_079292544.:	CUST_2063_PI442190711	HOT208_60m_824C06		149.1_WP_079292544.:	AGCAATGGCGACCTTATTACACTGCAGCTAAATGAAGGTGTTATATACATGGGGCAAATT	pykF	Pyruvate kinase	WP_079292544.:	4470..6260	Glycolysis	COG0469
149.1_WP_079292544.:	CUST_2064_PI442190711	HOT208_60m_824C06		149.1_WP_079292544.:	TGCTCAGGAAATGAGGATACTAAATCAAGGAGATTTAGTAGTTCAAACTGCAGGCACATT	pykF	Pyruvate kinase	WP_079292544.:	4470..6260	Glycolysis	COG0469
149.1_WP_079292544.:	CUST_2065_PI442190711	HOT208_60m_824C06		149.1_WP_079292544.:	CGGCTTAGTAAGAAAAATTGTATCAAGAGGAATTTCAATAGGGGAAATCGGTGTAACAGG	pykF	Pyruvate kinase	WP_079292544.:	4470..6260	Glycolysis	COG0469
155.1_594	CUST_3014_PI442190711	HOT208_60m_824C06		155.1_594	GATTATTCATTGTAGGAGCAGGTGCGCATGCTGGAATTGCAATGGTTAGAGATTACGATC	psaA	Photosystem I P700 chlorophyll A apoprotein A1 PsaA		11687..13081	Photosystem I	COG2885
155.1_594	CUST_3015_PI442190711	HOT208_60m_824C06		155.1_594	GCGATGCTTGGATCAATAAGCATACTCGTATCTCATCACATGTATGCGATGCCTCCATAT	psaA	Photosystem I P700 chlorophyll A apoprotein A1 PsaA		11687..13081	Photosystem I	COG2885
155.1_594	CUST_3016_PI442190711	HOT208_60m_824C06		155.1_594	CCAATATTATTCCCTGCACCCAAAGGTCATCAAGGTCTTTTTGAGTTCATGGCAGAAAGT	psaA	Photosystem I P700 chlorophyll A apoprotein A1 PsaA		11687..13081	Photosystem I	COG2885
155.1_594	CUST_3017_PI442190711	HOT208_60m_824C06		155.1_594	AAAGAAATAGCAACTATTGCAGATATGCCTATGCCGCATCAACTCTGCGATCCACAAATT	psaA	Photosystem I P700 chlorophyll A apoprotein A1 PsaA		11687..13081	Photosystem I	COG2885
155.1_594	CUST_3018_PI442190711	HOT208_60m_824C06		155.1_594	TCATATCGGAGCTCCTACTGCTGCACTTTTAGATGCAATTGATGCAGGTTCTCCTTTAAT	psaA	Photosystem I P700 chlorophyll A apoprotein A1 PsaA		11687..13081	Photosystem I	COG2885
155.1_594	CUST_3019_PI442190711	HOT208_60m_824C06		155.1_594	AAATGGAGTGGTTCCAAGATATTGAGTCGATGCTCAACCACCACATAGCAGGTTTAGTAG	psaA	Photosystem I P700 chlorophyll A apoprotein A1 PsaA		11687..13081	Photosystem I	COG2885
165.1_728	CUST_3728_PI442190711	HOT208_60m_824C06		165.1_728	TTCCTTCAATAATTGTTTTAATAATAGGTTTATATAGTTATAACATCTACGATCGAATGG	ctaC/coxB	Cytochrome c oxidase subunit II		9026..9368	Respiration	COG1622
165.1_728	CUST_3729_PI442190711	HOT208_60m_824C06		165.1_728	AGGAAAAGGAATGATCAGGTAGGCGATGGTATAGCTTTAGAGGGAAATTTAAGCTTAGAA	ctaC/coxB	Cytochrome c oxidase subunit II		9026..9368	Respiration	COG1622
165.1_728	CUST_3730_PI442190711	HOT208_60m_824C06		165.1_728	TCTTTTCATCATTGGATTAATTATTTTTATAGGAATGACAATAGCAGTTATTTATAGCTT	ctaC/coxB	Cytochrome c oxidase subunit II		9026..9368	Respiration	COG1622
165.1_728	CUST_3731_PI442190711	HOT208_60m_824C06		165.1_728	TCTTTTTGGATTGGTTTTAATGTAAATTTGCTCCCAGCTGAAGCAAGTATTAATGCACCA	ctaC/coxB	Cytochrome c oxidase subunit II		9026..9368	Respiration	COG1622
172.1_745	CUST_3806_PI442190711	HOT208_60m_824C06		172.1_745	CCAGATTAAATGCCGTAGCTTTTTGGTTAATTCCTCCTTCAGGTTTGATGCTGGTAGCAA	cyoB/coxA	Cytochrome c oxidase subunit I		<16..567	Respiration	COG0843
172.1_745	CUST_3807_PI442190711	HOT208_60m_824C06		172.1_745	CTTCTATTTAATAGGAGGAACCTTAGCGAGCGCTATAAGAATTGAACTGGCCAGCCCAAT	cyoB/coxA	Cytochrome c oxidase subunit I		<16..567	Respiration	COG0843
172.1_745	CUST_3808_PI442190711	HOT208_60m_824C06		172.1_745	GGATGGACGGCTTATCCTCCTTTGAGCATAACTACTCCTCAATCGGGACAAATTATATGG	cyoB/coxA	Cytochrome c oxidase subunit I		<16..567	Respiration	COG0843
172.1_745	CUST_3809_PI442190711	HOT208_60m_824C06		172.1_745	TGAGCGTTCTATTACTTGGAGGCAGTTCTATATTTGGTGGAATAAACTTTATCGCGACCA	cyoB/coxA	Cytochrome c oxidase subunit I		<16..567	Respiration	COG0843
172.1_745	CUST_3810_PI442190711	HOT208_60m_824C06		172.1_745	ACGGTGCTTTTGGAAATTATTTAATTCCATTTTATGTAGGTGCGAGAGATATGGCATTCC	cyoB/coxA	Cytochrome c oxidase subunit I		<16..567	Respiration	COG0843
172.1_745	CUST_3811_PI442190711	HOT208_60m_824C06		172.1_745	TTCAACCTAAAGGCTGGCTTAGATACTTTAGTTTTAGCCTTGATCATAAAGTAATTGGGA	cyoB/coxA	Cytochrome c oxidase subunit I		<16..567	Respiration	COG0843
219.1_WP_079295017.:	CUST_4340_PI442190711	HOT208_60m_824C06		219.1_WP_079295017.:	TGGATAACTGGCGTTGGCAAGGGGTTCCTTTTTATTTGAGAACAGGTAAAAGACTACCTA	zwf	glucose-6-phosphate dehydrogenase zwf	WP_079295017.:	13009..14532	Pentose Phosphate	COG0364
219.1_WP_079295017.:	CUST_4341_PI442190711	HOT208_60m_824C06		219.1_WP_079295017.:	TAGCTGATGCAATGCTTTCTGACCCAACATTATTTACTCGAAGTGATGAGGTAGAGGCAG	zwf	glucose-6-phosphate dehydrogenase zwf	WP_079295017.:	13009..14532	Pentose Phosphate	COG0364
219.1_WP_079295017.:	CUST_4342_PI442190711	HOT208_60m_824C06		219.1_WP_079295017.:	CGGGATATGCTTCAAAATCATATGACTCAAATGCTTGCAGTTACTGCAATGGAACCTCCT	zwf	glucose-6-phosphate dehydrogenase zwf	WP_079295017.:	13009..14532	Pentose Phosphate	COG0364
219.1_WP_079295017.:	CUST_4343_PI442190711	HOT208_60m_824C06		219.1_WP_079295017.:	CAAAACTTGCTGACGAAAATGAACCGTGGAAGTGTTGCATAAGAGGTCAATATGGAGAGG	zwf	glucose-6-phosphate dehydrogenase zwf	WP_079295017.:	13009..14532	Pentose Phosphate	COG0364
219.1_WP_079295017.:	CUST_4344_PI442190711	HOT208_60m_824C06		219.1_WP_079295017.:	TCCTGCCCCAAATCAACTTGTTCTTAGAATTCAGCCAAATGAAGGTGCTACTTTCAAATT	zwf	glucose-6-phosphate dehydrogenase zwf	WP_079295017.:	13009..14532	Pentose Phosphate	COG0364
219.1_WP_079295017.:	CUST_4345_PI442190711	HOT208_60m_824C06		219.1_WP_079295017.:	GGGGGGGGATATTTCAAATCGACTCAAAGGATATAGGCAGGAAGATGGTGTTAATAGT	zwf	glucose-6-phosphate dehydrogenase zwf	WP_079295017.:	13009..14532	Pentose Phosphate	COG0364
228.1_930	CUST_4759_PI442190711	HOT208_60m_824C06		228.1_930	TCTTAATTGGAAACCATTAAAAAGTATTGATGATATGTGTAAGGATGGTTGGAATTGGCA	galE	UDP-glucose 4-epimerase		119..429	Galactose metabolism	COG1087
228.1_930	CUST_4760_PI442190711	HOT208_60m_824C06		228.1_930	TGAATTTACAAGTCGAAGAGTTGGTGACATCGCAATTTATTATGCAGATACAACGAAAAT	galE	UDP-glucose 4-epimerase		119..429	Galactose metabolism	COG1087
228.1_930	CUST_4761_PI442190711	HOT208_60m_824C06		228.1_930	TGGGAAAGGAAACTCATGCAGCGTTTTGGAACTTAGAAATACTTTGAAAGAGTTAACAA	galE	UDP-glucose 4-epimerase		119..429	Galactose metabolism	COG1087
004.1_1132	CUST_5827_PI442190711	HOT208_60m_824C06		004.1_1132	GGAAAATATCCGATAATTTGCGCAGAATTATGTGGGCCATATCATGGAGGAATGAGAGCC	ctaC/coxB	Cytochrome c oxidase subunit II		<1..463	Respiration	COG1622
004.1_1132	CUST_5828_PI442190711	HOT208_60m_824C06		004.1_1132	CATGCTTTTGGGTACCAGAGTTCAGAATTAAGCAGGATATTATTCCTGGACAACCGACT	ctaC/coxB	Cytochrome c oxidase subunit II		<1..463	Respiration	COG1622
004.1_1132	CUST_5829_PI442190711	HOT208_60m_824C06		004.1_1132	TCCCAAGGGCAATTTCATATCAGGAGAACTACACGTACCTGTTGATCAAAAAGTATCCAT	ctaC/coxB	Cytochrome c oxidase subunit II		<1..463	Respiration	COG1622
004.1_1132	CUST_5830_PI442190711	HOT208_60m_824C06		004.1_1132	ACGATCGAATGGGAGGGATGAAAGAACTAAATCATAACCATGAAATGATGAGTTCTAACA	ctaC/coxB	Cytochrome c oxidase subunit II		<1..463	Respiration	COG1622
004.1_1132	CUST_5831_PI442190711	HOT208_60m_824C06		004.1_1132	TGGGCTGGAATTAGTCAAACTTCTGATAATGAAATCGCAATAAATAATTATCAATTGAA	ctaC/coxB	Cytochrome c oxidase subunit II		<1..463	Respiration	COG1622
004.1_1132	CUST_5832_PI442190711	HOT208_60m_824C06		004.1_1132	GCCTCGATAATTGTTGAAGAAGAATCTGATTACAACGAATGGTTTAACAAAAATAAA	ctaC/coxB	Cytochrome c oxidase subunit II		<1..463	Respiration	COG1622
084.1_1245	CUST_6357_PI442190711	HOT208_60m_824C06		084.1_1245	AAAGAGGCAACAATTAAATTAGGTTATTTAAACGAAGATGAATTTGATGAAGCAATGAGT	fumC	class II fumarate hydratase		<1..257	Krebs cycle and related genes	COG0114
084.1_1245	CUST_6358_PI442190711	HOT208_60m_824C06		084.1_1245	AGCCATAGTGCCAAAAGTTGGTTATGAAAAAGCAGCTAAAATTGCAAACCTTGCCTTCAA	fumC	class II fumarate hydratase		<1..257	Krebs cycle and related genes	COG0114
084.1_1245	CUST_6359_PI442190711	HOT208_60m_824C06		084.1_1245	TGGGATGGAACCTAATCAAAAGAAAATGAAGTTAAACTTAGAAAATTCACTAATGTTGGT	fumC	class II fumarate hydratase		<1..257	Krebs cycle and related genes	COG0114
KGF98001	CUST_77_PI442172308	MIT9302	HLII	KGF98001	AGAGGAATTGGGAAATCAAGCAAGATTTGCTGGCGCTTTAGGTTTAGGTCCCAAAAATAT	eno	Enolase	ASM75997	14603..15895	Glycolysis/Gluconeogenesis	COG0148
KGF98001	CUST_78_PI442172308	MIT9302	HLII	KGF98001	GCTACAAGATCTGGTCAAATCAAGACCGGCTCGTTAAGTAGAAGTGAGAGGATTGCAAAA	eno	Enolase	ASM75997	14603..15895	Glycolysis/Gluconeogenesis	COG0148
KGF98001	CUST_79_PI442172308	MIT9302	HLII	KGF98001	TATCCAATAGTTTCAATAGAGGACGGTTTAGCAGAGGATGATTGGGAGGGTTGGTCAGAA	eno	Enolase	ASM75997	14603..15895	Glycolysis/Gluconeogenesis	COG0148
KGF98001	CUST_80_PI442172308	MIT9302	HLII	KGF98001	GAAGAAGCAGGGGACTTATTATTAGAAGCAATTCAAAAAGCCGGATTTACGCCTGGTGAG	eno	Enolase	ASM75997	14603..15895	Glycolysis/Gluconeogenesis	COG0148
KGF98001	CUST_81_PI442172308	MIT9302	HLII	KGF98001	AATCATTACTTGATCAAAAAGGTCTATCTACTGCTGTAGGCGATGAGGGTGGATTTGCCC	eno	Enolase	ASM75997	14603..15895	Glycolysis/Gluconeogenesis	COG0148
KGF98001	CUST_82_PI442172308	MIT9302	HLII	KGF98001	TGCACATGCACCAAATAGTCTTGATTTTCAGGAATTCATGCTTGTCCCACATGGAGTTAA	eno	Enolase	ASM75997	14603..15895	Glycolysis/Gluconeogenesis	COG0148
KGF98775	CUST_520_PI442172308	MIT9302	HLII	KGF98775	ATTAGGAGCTGCAGCATTGGCGGGCACAAAAATACAAATAGATAGGAACTTTACTGCTTC	argH	Argininosuccinate lyase	ASM75997	2693...4072	Amino Acid metabolism	COG0165
KGF98775	CUST_521_PI442172308	MIT9302	HLII	KGF98775	AAGCATTGCTAACCATGGTCAAAGGGGTACCACTTGCATATAACAAGGATTTTCAAGAGG	argH	Argininosuccinate lyase	ASM75997	2693...4072	Amino Acid metabolism	COG0165
KGF98775	CUST_522_PI442172308	MIT9302	HLII	KGF98775	TGGCAGATTACTTAGTTGCTAAGAAGGTTCCCTTTAGGACTGCTTATCAAGTTGTGGGTG	argH	Argininosuccinate lyase	ASM75997	2693...4072	Amino Acid metabolism	COG0165
KGF98775	CUST_523_PI442172308	MIT9302	HLII	KGF98775	TGCCGCAGAAAAAAAATCCAGATGTTCCAGAATTGATAAGAGGTAAAACGGGAAGAGTTT	argH	Argininosuccinate lyase	ASM75997	2693...4072	Amino Acid metabolism	COG0165
KGF98775	CUST_524_PI442172308	MIT9302	HLII	KGF98775	TGCGGATCTTGAACCTTTTAATGTTGTTAAGTCAAGAACTAGTGAAGGTGGAACAGGTTT	argH	Argininosuccinate lyase	ASM75997	2693...4072	Amino Acid metabolism	COG0165
KGF98775	CUST_525_PI442172308	MIT9302	HLII	KGF98775	TGCAGTGAGTGATAGAGATTTTTGTATAGAGTTTGCTTCTGCATCTGCTTTGTTAATGTC	argH	Argininosuccinate lyase	ASM75997	2693...4072	Amino Acid metabolism	COG0165
KGF99445	CUST_1429_PI442172308	MIT9302	HLII	KGF99445	ATTAAAGGTTGATTCTTATGGATGTGGCGATTCTTTCGCTGCGGGCATTCTTTATGGAAT	rbsK	Sugar kinase, ribokinase family	ASM75997	26403...27272	Pentose Phosphate	COG0524
KGF99445	CUST_1430_PI442172308	MIT9302	HLII	KGF99445	TGTGGCATGGCGTGATAAACCTACTAGAAGAGGGTTTAGTATGGTTGATTATCGAGAGGA	rbsK	Sugar kinase, ribokinase family	ASM75997	26403...27272	Pentose Phosphate	COG0524
KGF99445	CUST_1431_PI442172308	MIT9302	HLII	KGF99445	TCCAGCTGGTGCAGGCTCTGTTATCGCAAAAAGACTTTCTGAACTAACTTTAAACCAAAT	rbsK	Sugar kinase, ribokinase family	ASM75997	26403...27272	Pentose Phosphate	COG0524
KGF99445	CUST_1432_PI442172308	MIT9302	HLII	KGF99445	AACCGAAGGAGAGAAGGGAGGCATAATATTTCCAGGAGGAAGATATAAGGCTCTTAAAAA	rbsK	Sugar kinase, ribokinase family	ASM75997	26403...27272	Pentose Phosphate	COG0524
KGF99445	CUST_1433_PI442172308	MIT9302	HLII	KGF99445	TCAGAGATTTTTAAAATAGCTAGATCAGCTTCAATACTGTGTACAACACCAAGGGTGGGA	rbsK	Sugar kinase, ribokinase family	ASM75997	26403...27272	Pentose Phosphate	COG0524
KGF99445	CUST_1434_PI442172308	MIT9302	HLII	KGF99445	AGTTTAAATCTTGCTAAAGTAATGGGAAGAGACGCTAGTGAATTTTTAGGCCCATATGCA	rbsK	Sugar kinase, ribokinase family	ASM75997	26403...27272	Pentose Phosphate	COG0524
KGF96286	CUST_2127_PI442172308	MIT9302	HLII	KGF96286	ACCAATCAAAAGAACTATTTCAGGAGAAATAAAATTTAAAAAAACTGAAAAAACTGAAAA	minE	septum site-determining protein MinE	ASM75997	87550...87882	Cell Division	COG0851
KGF96287	CUST_2131_PI442172308	MIT9302	HLII	KGF96287	ACAAAAATTGTCGTCTAGACCAAGCATTAGTAAGGCATAAAAAGGAGCCCAATCTTGCCC	minD	septum site-determining protein MinD	ASM75997	87550...87882	Cell Division	COG2894
KGF96286	CUST_2128_PI442172308	MIT9302	HLII	KGF96286	TGATTTTGAAGAGGTAGCAGTAAGTTTAGAGACTGAGGATAGAATGACGGCATTAGTGGC	minE	septum site-determining protein MinE	ASM75997	87550...87882	Cell Division	COG0851
KGF96286	CUST_2129_PI442172308	MIT9302	HLII	KGF96286	AGTTCATTAACAACTGATCTTTTGGATAAAATGAGGAAAGAAATCCTCGATGTTGTTGCT	minE	septum site-determining protein MinE	ASM75997	87550...87882	Cell Division	COG0851
KGF96286	CUST_2130_PI442172308	MIT9302	HLII	KGF96286	AGGCAGAGAAACGTCTAGTGCAAATACAGCTAGAGAAAGATTACAACTTGTACTAGCTCA	minE	septum site-determining protein MinE	ASM75997	87550...87882	Cell Division	COG0851
KGF96287	CUST_2132_PI442172308	MIT9302	HLII	KGF96287	AGCTTGTAAAGAAGCTATTGTTGTTACCAACCCAGAATTATCTGCAGTACGAGATGCCGA	minD	septum site-determining protein MinD	ASM75997	87887...88702	Cell Division	COG2894
KGF96287	CUST_2133_PI442172308	MIT9302	HLII	KGF96287	CCAACAGCGGAGCGACCACTGCTGTATTAGATGCTGATTTTGGCTTAAGAAATTTAGATC	minD	septum site-determining protein MinD	ASM75997	87887...88702	Cell Division	COG2894
KGF96287	CUST_2134_PI442172308	MIT9302	HLII	KGF96287	AAATCGAGGCGAACCATTAACACTCACAGATGGCAGATCTCCAGCAAAAAAATGCTATTT	minD	septum site-determining protein MinD	ASM75997	87887...88702	Cell Division	COG2894
KGF96287	CUST_2135_PI442172308	MIT9302	HLII	KGF96287	TACCTGCTGGAGATCCCAGGATGTTGGATTGGATGAAGCCGGAAGATATGAAAAAAATTA	minD	septum site-determining protein MinD	ASM75997	87887...88702	Cell Division	COG2894
KGF96287	CUST_2136_PI442172308	MIT9302	HLII	KGF96287	GCCTATTCAGCTTGTAATCAATCGAGTACGTCCTAACATGATGGCTAGTCAAGAAATGCT	minD	septum site-determining protein MinD	ASM75997	87887...88702	Cell Division	COG2894
KGF96288	CUST_2137_PI442172308	MIT9302	HLII	KGF96288	TTCAGCCAAAAAGAATATTTATGTTTGGGGCAAACTACTAGGGATTGCCTTCGCAGGAAA	minC	septum site-determining protein MinC	ASM75997	88814...89473	Cell Division	COG0850
KGF96288	CUST_2138_PI442172308	MIT9302	HLII	KGF96288	ACGATCTCCTTCTAGAGGGGACAGTAAGGTCAGGAGATAGAATATCTTCGAATGGAAACC	minC	septum site-determining protein MinC	ASM75997	88814...89473	Cell Division	COG0850
KGF96288	CUST_2139_PI442172308	MIT9302	HLII	KGF96288	ACCCTTTACAGTTAAGAATTGCTGATGTAGTAGCTATTGGACCAAAGGATAAGCCCAAAA	minC	septum site-determining protein MinC	ASM75997	88814...89473	Cell Division	COG0850
KGF96288	CUST_2140_PI442172308	MIT9302	HLII	KGF96288	TTAGCCGGAAAGTCTTTAAAAATAGATTCATTTTTTTTGAAAGAGCACGAGGTCAAGGCT	minC	septum site-determining protein MinC	ASM75997	88814...89473	Cell Division	COG0850
KGF96288	CUST_2141_PI442172308	MIT9302	HLII	KGF96288	TCAAAGGCCATCTTGAAGTAAAATTTTTCGCAATAAATGAAACAATAACTTCCCATCAGT	minC	septum site-determining protein MinC	ASM75997	88814...89473	Cell Division	COG0850
KGF96288	CUST_2142_PI442172308	MIT9302	HLII	KGF96288	CCTGAAATTGCAGTAGTAGATAAACAAACCATAAATATCAAGCCTTACATAATAGAAACT	minC	septum site-determining protein MinC	ASM75997	88814...89473	Cell Division	COG0850
KGF97389	CUST_2720_PI442172308	MIT9302	HLII	KGF97389	TGTCACTTTAGGCACTTTAATGATTTTAATTATTGCTTGGCAATCGAGAATCAATGGCGG	ctaE/coxC	Cytochrome c oxidase subunit III	ASM75997	91317...91919	Respiration	COG1845

KGF97389	CUST_2718_PI442172308	MIT9302	HLII	KGF97389	TTGGTCTTATAACTTTCCTAATTGCAGACGGAATGACTTTTGCTGGATTCTTTGCTGCTT	ctaE/coxC	Cytochrome c oxidase subunit III	ASM75997	91317…91919	Respiration	COG1845
KGF97389	CUST_2719_PI442172308	MIT9302	HLII	KGF97389	TGTTCCCATTGGAAGCTGTTGAATTGTACTGGCACTTTGTAGATGGAATATGGGTTATTT	ctaE/coxC	Cytochrome c oxidase subunit III	ASM75997	91317…91919	Respiration	COG1845
KGF97389	CUST_2721_PI442172308	MIT9302	HLII	KGF97389	ACACTCAATACAATTTTGTTACTTGTTAGTAGTGCAACTTTCCATAAAGCAGGCAAAGCA	ctaE/coxC	Cytochrome c oxidase subunit III	ASM75997	91317…91919	Respiration	COG1845
KGF97389	CUST_2722_PI442172308	MIT9302	HLII	KGF97389	TATAAAGCAGTAAATCCATTACCTGATGGTGCTATTTATGAATTAGAACTACCAATACCT	ctaE/coxC	Cytochrome c oxidase subunit III	ASM75997	91317…91919	Respiration	COG1845
KGF97389	CUST_2723_PI442172308	MIT9302	HLII	KGF97389	ACCTTTTGGATTAACTGATAATTTATTTGCAAGTACTTTTTATGCTCTTACTGGTTTTCA	ctaE/coxC	Cytochrome c oxidase subunit III	ASM75997	91317…91919	Respiration	COG1845
KGF97390	CUST_2724_PI442172308	MIT9302	HLII	KGF97390	TGTTTTTGGAGGTATTACAGGAGTTGCTTTGGCACAGGTACCTTTCGATATTCACGTACA	cyoB/coxA	Cytochrome c oxidase subunit I	ASM75997	91926…93551	Respiration	COG0843
KGF97390	CUST_2725_PI442172308	MIT9302	HLII	KGF97390	TACTAGTGGAACGCCTCCTTGGATGAGATTGTTCTTTACTATCGCGACAGCATTTATTGC	cyoB/coxA	Cytochrome c oxidase subunit I	ASM75997	91926…93551	Respiration	COG0843
KGF97390	CUST_2726_PI442172308	MIT9302	HLII	KGF97390	GCTTTGCTCCTCAACATTGGCTTGGTTTAAATGGAATGCCAAGAAGAGTTGCAGAATATG	cyoB/coxA	Cytochrome c oxidase subunit I	ASM75997	91926…93551	Respiration	COG0843
KGF97390	CUST_2727_PI442172308	MIT9302	HLII	KGF97390	TTCTCCACCTCCAGTTGAAAATTGGGAAGGAGAAGCTCCATTAGTTGAAGAACCTTATGG	cyoB/coxA	Cytochrome c oxidase subunit I	ASM75997	91926…93551	Respiration	COG0843
KGF97390	CUST_2728_PI442172308	MIT9302	HLII	KGF97390	TTCCTTAGTGTGAGAAATGGCAAAGATGCTGGAGATAATCCATGGAATGCTCTTACACCT	cyoB/coxA	Cytochrome c oxidase subunit I	ASM75997	91926…93551	Respiration	COG0843
KGF97390	CUST_2729_PI442172308	MIT9302	HLII	KGF97390	TGGGTTGCAACATTATGGGGAGGTAGGATATCCATCAATAGTGCAATGTTATTCTCATGT	cyoB/coxA	Cytochrome c oxidase subunit I	ASM75997	91926…93551	Respiration	COG0843
KGF97391	CUST_2730_PI442172308	MIT9302	HLII	KGF97391	TATCCCATAATTTGCGCAGAATTATGTGGCCCATATCATGGTGGTATGAGAGCTTCCATA	ctaC/coxB	Cytochrome c oxidase subunit II	ASM75997	93548…94441	Respiration	COG1622
KGF97391	CUST_2731_PI442172308	MIT9302	HLII	KGF97391	TCCCAAGGGCAATTTTATATCAGGCGAACTACACGTTCCTGTTGATCAAAAAGTATCAAT	ctaC/coxB	Cytochrome c oxidase subunit II	ASM75997	93548…94441	Respiration	COG1622
KGF97391	CUST_2732_PI442172308	MIT9302	HLII	KGF97391	CATGCTTTTTGGGTACCAGAGTTCAGAATTAAACAGGATATTATTCCTGGACAACCCACT	ctaC/coxB	Cytochrome c oxidase subunit II	ASM75997	93548…94441	Respiration	COG1622
KGF97391	CUST_2733_PI442172308	MIT9302	HLII	KGF97391	AATGTAAATTTGCTCCCAGAGGAAGCGAGTATTAATGCACCAATTTACGATGAGCTTTTT	ctaC/coxB	Cytochrome c oxidase subunit II	ASM75997	93548…94441	Respiration	COG1622
KGF97391	CUST_2734_PI442172308	MIT9302	HLII	KGF97391	AGCTACAACATCTATGATCGAATGGGAGGGATGAAAGAACTAAATCATAACCACGAAATG	ctaC/coxB	Cytochrome c oxidase subunit II	ASM75997	93548…94441	Respiration	COG1622
KGF97391	CUST_2735_PI442172308	MIT9302	HLII	KGF97391	GGCGATGGTATAGCTTTAGAGGGAAATTTAAGCTTAGAAATTGTATGGACAATTATCCCT	ctaC/coxB	Cytochrome c oxidase subunit II	ASM75997	93548…94441	Respiration	COG1622
KGF97399	CUST_2772_PI442172308	MIT9302	HLII	KGF97399	AGCTAGCAGAGGAATTGGTAAAGAAATTGCTTTAGAACTAAGCCGCTTAGGAGCAGAAGT	gdh	3-oxoacyl-acyl-carrier-protein reductase	ASM75997	100929…101678	Entner-Dudoroff	COG1028
KGF97399	CUST_2773_PI442172308	MIT9302	HLII	KGF97399	CAAGCAAATTATTCTGCAGCGAAAGCTGGAGTTATTGGATTCACCAAACTTGCGCTAAA	gdh	3-oxoacyl-acyl-carrier-protein reductase	ASM75997	100929…101678	Entner-Dudoroff	COG1028
KGF97399	CUST_2777_PI442172308	MIT9302	HLII	KGF97399	ACTCTTCATCCGATGAAAAAGCTGAAGAAGTCGTAAATTCAATAAAAAATTCGGGAGGTA	gdh	3-oxoacyl-acyl-carrier-protein reductase	ASM75997	100929…101678	Entner-Dudoroff	COG1028
KGF97399	CUST_2774_PI442172308	MIT9302	HLII	KGF97399	TGCCGGCATTACTAGAGATGGACTATTAATGAGAATGAAATCGGAACAATGGGATGACGT	gdh	3-oxoacyl-acyl-carrier-protein reductase	ASM75997	100929…101678	Entner-Dudoroff	COG1028
KGF97399	CUST_2775_PI442172308	MIT9302	HLII	KGF97399	TGCTTCAAGAGGTATAAACGTAAATGCAATAGCTCCCGGTTTCATAGAAACAGAGATGAC	gdh	3-oxoacyl-acyl-carrier-protein reductase	ASM75997	100929…101678	Entner-Dudoroff	COG1028
KGF97399	CUST_2776_PI442172308	MIT9302	HLII	KGF97399	GGAAGTTGTACTCAAATTGCAACCTTAGTGTCATTCTTAGTTTCAAGTAATGCAGGAAGT	gdh	3-oxoacyl-acyl-carrier-protein reductase	ASM75997	100929…101678	Entner-Dudoroff	COG1028
KGF97464	CUST_3130_PI442172308	MIT9302	HLII	KGF97464	AGATCAAATGGCGAGCGAGAAGCTTAGTGAAGGGATAACAGGATTCAGTAAGGCTATAGA	tal	Transaldolase	ASM75997	157821…158825	Pentose Phosphate	COG0176
KGF97464	CUST_3131_PI442172308	MIT9302	HLII	KGF97464	GGCGTGTATCTACAGAAGTTGACGCAAGACTAAGCTTTGACACTGAAGCTACGGTAAAAA	tal	Transaldolase	ASM75997	157821…158825	Pentose Phosphate	COG0176
KGF97464	CUST_3132_PI442172308	MIT9302	HLII	KGF97464	CTTCTGCCAAGCGGTAACTTGTGCAAATTCAAAGATAACCTTAATCTCTCCGTTCGTTGG	tal	Transaldolase	ASM75997	157821…158825	Pentose Phosphate	COG0176
KGF97464	CUST_3133_PI442172308	MIT9302	HLII	KGF97464	AAGATTGCCGCTACTTGGGAAGGAATTAAAGCAGCTGAAATTTTGGAAAAAGAGGGTATT	tal	Transaldolase	ASM75997	157821…158825	Pentose Phosphate	COG0176
KGF97464	CUST_3134_PI442172308	MIT9302	HLII	KGF97464	GGATTGGCATAAAGCAAAAACTGGTAAAACTAGTTTTATAGGTGCTGAAGACCCTGGTGT	tal	Transaldolase	ASM75997	157821…158825	Pentose Phosphate	COG0176
KGF97464	CUST_3135_PI442172308	MIT9302	HLII	KGF97464	AATGGGAGCGAGTTTTAGAAATCTTGATGAAATAAAAGAATTAGCAGGTTGCGATCTTCT	tal	Transaldolase	ASM75997	157821…158825	Pentose Phosphate	COG0176
KGF97494	CUST_3304_PI442172308	MIT9302	HLII	KGF97494	TGCACTTTTAGCGATTTTTGGAGATGATTCTTGTCTTCAGTTTGGTGGAGGAACACATGG	rbcL	Ribulose 1,5-bisphosphate carboxylase large subunit	ASM75997	186223…187638	Carbon fixation	COG1850
KGF97494	CUST_3306_PI442172308	MIT9302	HLII	KGF97494	CTAACAGAGTCGCTTTAGAAGCTTGTGTTAAAGCACGTAATGCAGGTCGCGAAATTGAAA	rbcL	Ribulose 1,5-bisphosphate carboxylase large subunit	ASM75997	186223…187638	Carbon fixation	COG1850
KGF97494	CUST_3308_PI442172308	MIT9302	HLII	KGF97494	GCTCTTGAAACTTGGAAGGAAATCAAGTTTGAGTTTGATACCGTCGACAAGCTTGACGTT	rbcL	Ribulose 1,5-bisphosphate carboxylase large subunit	ASM75997	186223…187638	Carbon fixation	COG1850
KGF97495	CUST_3310_PI442172308	MIT9302	HLII	KGF97495	GTGAGAATCATCGGCTACGATGCTTATACTCAAAGCCAAGGAACAGCTTTTGTCGTTTTC	rbcS	Ribulose bisphosphate carboxylase small subunit	ASM75997	187733…188074	Carbon fixation	COG4451
KGF97495	CUST_3311_PI442172308	MIT9302	HLII	KGF97495	TGGAAACTCCCATTCTTTGGGGAAAAAGATCTTAACTTGGTTGTAAGTGAATTAGAGGCA	rbcS	Ribulose bisphosphate carboxylase small subunit	ASM75997	187733…188074	Carbon fixation	COG4451
KGF97495	CUST_3312_PI442172308	MIT9302	HLII	KGF97495	AAGGTTGGAGTCCTGTTATTGAGCATGTTCACCCTAGTGGAAGTATGCAAACTTATTGGT	rbcS	Ribulose bisphosphate carboxylase small subunit	ASM75997	187733…188074	Carbon fixation	COG4451
KGF97495	CUST_3313_PI442172308	MIT9302	HLII	KGF97495	GCAACCCTGGAAACATTCGGTTTCTTACCACCGATGACCCAGGAAGAAATATATGATCAA	rbcS	Ribulose bisphosphate carboxylase small subunit	ASM75997	187733…188074	Carbon fixation	COG4451
KGF97543	CUST_3564_PI442172308	MIT9302	HLII	KGF97543	ACCCTTTGGAGGGTTATGGATTTCTGGAGGGACTGCACCAAAACATTTCAAAAACTTTAA	glk	Glucokinase	ASM75997	233773…234810	Glycolysis	COG0837
KGF97543	CUST_3565_PI442172308	MIT9302	HLII	KGF97543	TGAGGGGGATCAGCTAATGATTGAAGTAGAGAGGATATGGTTAGGTGCTTATGCCTCTTT	glk	Glucokinase	ASM75997	233773…234810	Glycolysis	COG0837
KGF97543	CUST_3566_PI442172308	MIT9302	HLII	KGF97543	CAATCCAAAATGGAGCAACCTCTCAAGGTTCTAACAAAGATTTGCATGCCATTGTTGGTG	glk	Glucokinase	ASM75997	233773…234810	Glycolysis	COG0837
KGF97543	CUST_3567_PI442172308	MIT9302	HLII	KGF97543	CTTGTGAGAGAATTGTTAGCGGTCCTGGTTTATCAAGAATAGCCGAATGGAGACTTAGCA	glk	Glucokinase	ASM75997	233773…234810	Glycolysis	COG0837
KGF97543	CUST_3568_PI442172308	MIT9302	HLII	KGF97543	CAGGAAGTGAAGTAAAAGTTTTAGCTAGCGAAGGTGGCCATATTGAATACTCGCCAAAGT	glk	Glucokinase	ASM75997	233773…234810	Glycolysis	COG0837
KGF97543	CUST_3569_PI442172308	MIT9302	HLII	KGF97543	ACCTCTAAAAGTAATTTTAGATGAAGAGTTTGGACTTTTTAGTGCAGCCTGCAGAGCAAA	glk	Glucokinase	ASM75997	233773…234810	Glycolysis	COG0837
KGF96889	CUST_4810_PI442172308	MIT9302	HLII	KGF96889	TTAAATGCACCAGTTACGATAAGGTCCATTATTAATAGAGGTGGGTCTCAAGGCGCACAA	pdhB	Pyruvate dehydrogenase E1 component beta subuni	ASM75997	104166…105149	Glycolysis/Krebs cycle	COG0022
KGF96889	CUST_4808_PI442172308	MIT9302	HLII	KGF96889	AAATCTTGCATTAAAGGCCTCGACAAAACTCGATTCTGATGGAATAGGCGCTGAAGTTTT	pdhB	Pyruvate dehydrogenase E1 component beta subuni	ASM75997	104166…105149	Glycolysis/Krebs cycle	COG0022
KGF96889	CUST_4809_PI442172308	MIT9302	HLII	KGF96889	TACATAGTTGGTTCGCTCATATTCCAGGAATTCGAGTAGTCATGCCTTCTACAGTCGAGG	pdhB	Pyruvate dehydrogenase E1 component beta subuni	ASM75997	104166…105149	Glycolysis/Krebs cycle	COG0022
KGF96889	CUST_4811_PI442172308	MIT9302	HLII	KGF96889	AAATACTCTAATTGACCCTCCGAGAAGAATCACTCTCCCTGATTGTCCTGCTCCTTCAAG	pdhB	Pyruvate dehydrogenase E1 component beta subuni	ASM75997	104166…105149	Glycolysis/Krebs cycle	COG0022
KGF96889	CUST_4812_PI442172308	MIT9302	HLII	KGF96889	GCTGCGGTTGGAGCTTCTATTTGTGGGATGAAGCCAATAATAGTTCATCCTAGAATGGAT	pdhB	Pyruvate dehydrogenase E1 component beta subuni	ASM75997	104166…105149	Glycolysis/Krebs cycle	COG0022
KGF96889	CUST_4813_PI442172308	MIT9302	HLII	KGF96889	AGACTCAGGTTGGAAAACATGTGGAATGGCTTCTGAAGTAATAACTTCAATTGTTGAGCA	pdhB	Pyruvate dehydrogenase E1 component beta subuni	ASM75997	104166…105149	Glycolysis/Krebs cycle	COG0022
KGF96890	CUST_4814_PI442172308	MIT9302	HLII	KGF96890	TTTTTGGGATCTGTCCCAATAGTTACAGGAACAGTACCCTTAGCTCTAGGTGCAGGATTA	pdhA	Pyruvate dehydrogenase E1 component alpha subuni	ASM75997	105142…106083	Glycolysis/Krebs cycle	COG1071
KGF96890	CUST_4815_PI442172308	MIT9302	HLII	KGF96890	TTACATAAGCTATTCGCTGAAGTTTTGGGGAGAAATACAGGATTTTCCGCTGGTATGGGA	pdhA	Pyruvate dehydrogenase E1 component alpha subuni	ASM75997	105142…106083	Glycolysis/Krebs cycle	COG1071
KGF96890	CUST_4816_PI442172308	MIT9302	HLII	KGF96890	AGATGGTTCGGTCATGTTGATTGGAGGGAAGATATTGATGTAGGTGTTGCTAGGTCAAAA	pdhA	Pyruvate dehydrogenase E1 component alpha subuni	ASM75997	105142…106083	Glycolysis/Krebs cycle	COG1071
KGF96890	CUST_4817_PI442172308	MIT9302	HLII	KGF96890	TGGGAGATGGAGCTACTGAAGAAGGTGTATTTCATGAGTCACTAAATTTTGCAAGAGTTT	pdhA	Pyruvate dehydrogenase E1 component alpha subuni	ASM75997	105142…106083	Glycolysis/Krebs cycle	COG1071
KGF96890	CUST_4818_PI442172308	MIT9302	HLII	KGF96890	AAAAGTTGATGAGTTGATCGATGAAAATTGGGAAAAAGCTTTAAAAGATGATTTCCCTGA	pdhA	Pyruvate dehydrogenase E1 component alpha subuni	ASM75997	105142…106083	Glycolysis/Krebs cycle	COG1071
KGF96890	CUST_4819_PI442172308	MIT9302	HLII	KGF96890	AGATTTGCTAAAGCTCATGAAATAGAAACTAAACTAGTTGATGGTAATGATGTTTTGGAA	pdhA	Pyruvate dehydrogenase E1 component alpha subuni	ASM75997	105142…106083	Glycolysis/Krebs cycle	COG1071
KGF96900	CUST_4874_PI442172308	MIT9302	HLII	KGF96900	TCTGGTAGAAAAGAATGGCACCTAGCATCTTTAAGGTACTTTAATCCCATCGGAGCACAT	galE	UDP-glucose 4-epimerase	ASM75997	119047…120099	Galactose metabolism	COG1087
KGF96900	CUST_4875_PI442172308	MIT9302	HLII	KGF96900	GCATTGGGGTTACAAAAAGAATTAAAAGTGTTTGGTAATGACTGGCCTACTAAAGATGGG	galE	UDP-glucose 4-epimerase	ASM75997	119047…120099	Galactose metabolism	COG1087
KGF96900	CUST_4876_PI442172308	MIT9302	HLII	KGF96900	AAAATTGTTCCCAAATCATCAATAGAAGATATGTGTCGAGACGGCTGGAAATGGAAAAAA	galE	UDP-glucose 4-epimerase	ASM75997	119047…120099	Galactose metabolism	COG1087
KGF96900	CUST_4877_PI442172308	MIT9302	HLII	KGF96900	AAATGTTGGGACAGGTAAAGGTACCTCTGTATTGGAACTGATTAAAATCTTCGAAAGAGT	galE	UDP-glucose 4-epimerase	ASM75997	119047…120099	Galactose metabolism	COG1087
KGF96900	CUST_4878_PI442172308	MIT9302	HLII	KGF96900	TCAGGTTTGATAGGTGAATGTCCTATTGGGATACCAAATAATATTTTCCCTCTGATTACA	galE	UDP-glucose 4-epimerase	ASM75997	119047…120099	Galactose metabolism	COG1087
KGF96900	CUST_4879_PI442172308	MIT9302	HLII	KGF96900	AAAGGTGCCATACGTATATGTAAATAGAAGAATAGGAGATAAGTGTTCTTTAGTTGCTGA	galE	UDP-glucose 4-epimerase	ASM75997	119047…120099	Galactose metabolism	COG1087
KGF96901	CUST_4880_PI442172308	MIT9302	HLII	KGF96901	TGACGTAAAAGAGGTTGCTAGAGCTATTGGTACTGACAGTAGGATTGGCTCCAGATTTCT	ugd	UDP-glucose 6-dehydrogenase/nucleotide sugar del	ASM75997	120232…121671	Amino sugar and nucleotide sugar r	COG1004
KGF96901	CUST_4881_PI442172308	MIT9302	HLII	KGF96901	GAAATAGCGGCTTCTAACGCTGACGCAATAATCGTTTTAACTGAGTGGGATGAGTACTCT	ugd	UDP-glucose 6-dehydrogenase/nucleotide sugar del	ASM75997	120232…121671	Amino sugar and nucleotide sugar r	COG1004
KGF96901	CUST_4882_PI442172308	MIT9302	HLII	KGF96901	ACCTGCTTGGGTATTTGATGCGCGATCTGTCATTGATGAAAAAAAAGTATTAGCTGCAGA	ugd	UDP-glucose 6-dehydrogenase/nucleotide sugar del	ASM75997	120232…121671	Amino sugar and nucleotide sugar r	COG1004
KGF96901	CUST_4883_PI442172308	MIT9302	HLII	KGF96901	CTTAGCCCAAAGAGTAAGTTCTATTAATGCCATTGGAGCATTATGTGAAGCTACAGGCGC	ugd	UDP-glucose 6-dehydrogenase/nucleotide sugar del	ASM75997	120232…121671	Amino sugar and nucleotide sugar r	COG1004
KGF96901	CUST_4884_PI442172308	MIT9302	HLII	KGF96901	TGATGCTGGCCCAGGTTTTGGAGGTAGTTGCTTTAAAAAAGATATTCTCAATCTAGTCTA	ugd	UDP-glucose 6-dehydrogenase/nucleotide sugar del	ASM75997	120232…121671	Amino sugar and nucleotide sugar r	COG1004
KGF96901	CUST_4885_PI442172308	MIT9302	HLII	KGF96901	TGGAACTGTTACGGGCAAGAAAATTTGTATTTTAGGATTCGCATTCAAAGCTAACACAAA	ugd	UDP-glucose 6-dehydrogenase/nucleotide sugar del	ASM75997	120232…121671	Amino sugar and nucleotide sugar r	COG1004
KGF96969	CUST_5274_PI442172308	MIT9302	HLII	KGF96969	CATCATTTGTTGGGAGGATATGCATTTATTGGAGAAGGAATTCCAGTTGCTTTAGGGGCA	pdhA	Pyruvate dehydrogenase E1 component alpha subuni	ASM75997	191810…192883	Glycolysis/Krebs cycle	COG1071
KGF96969	CUST_5275_PI442172308	MIT9302	HLII	KGF96969	GTTGCTGGTAATAAAAGTAGTGATGCAGTAACTGCAGCATTCTTTGGAGATGGAACCTGC	pdhA	Pyruvate dehydrogenase E1 component alpha subuni	ASM75997	191810…192883	Glycolysis/Krebs cycle	COG1071
KGF96969	CUST_5276_PI442172308	MIT9302	HLII	KGF96969	AGGAAGTTGATGGAATGGATGTTTTAGCAGTAAGAGGAGCAGCAGAAAGAGCAATTGAGA	pdhA	Pyruvate dehydrogenase E1 component alpha subuni	ASM75997	191810…192883	Glycolysis/Krebs cycle	COG1071
KGF96969	CUST_5277_PI442172308	MIT9302	HLII	KGF96969	ATGCAGAGATATCAGAGTCCGTTAAAAATGCTTTAGAAGCACCTGAACCTCCTTCAGAGG	pdhA	Pyruvate dehydrogenase E1 component alpha subuni	ASM75997	191810…192883	Glycolysis/Krebs cycle	COG1071
KGF96969	CUST_5278_PI442172308	MIT9302	HLII	KGF96969	TTTGTTGTGGAGAATAATAAATGGGCGATAGGTATGGCTCACGATAGAGCAACCAGCAAT	pdhA	Pyruvate dehydrogenase E1 component alpha subuni	ASM75997	191810…192883	Glycolysis/Krebs cycle	COG1071
KGF96969	CUST_5279_PI442172308	MIT9302	HLII	KGF96969	AGGACATTCTCTCGCAGATCCTGATGAATTAAGGTCAGAGAAAGAGAAAGAGTTTTGGGG	pdhA	Pyruvate dehydrogenase E1 component alpha subuni	ASM75997	191810…192883	Glycolysis/Krebs cycle	COG1071
KGF96991	CUST_5399_PI442172308	MIT9302	HLII	KGF96991	GTGCAGTTGTTGATGAAGCAATGGAAGGGGAAATACAAGTTACTGTTATTGCTACCGGTT	ftsZ	cell division protein FtsZ	ASM75997	214956…216107	Cell Division	COG0206
KGF96991	CUST_5398_PI442172308	MIT9302	HLII	KGF96991	TGAAGGTAAAAGAAGGATGCGACAAGCAGAAGAAGGGATCGCTAGATTAGCTGAAAATGT	ftsZ	cell division protein FtsZ	ASM75997	214956…216107	Cell Division	COG0206
KGF96991	CUST_5400_PI442172308	MIT9302	HLII	KGF96991	GCAGGAGCACCCCTTCAAGAAGCATTTAGAAATGCTGATGATGTTTTAGGAATGGGAGTT	ftsZ	cell division protein FtsZ	ASM75997	214956…216107	Cell Division	COG0206
KGF96991	CUST_5401_PI442172308	MIT9302	HLII	KGF96991	CCCAGTGGTCGCAGAAGTAGCAAAACAAAGTGGTGCTTTAACAGTAGGAATAGTCACTAA	ftsZ	cell division protein FtsZ	ASM75997	214956…216107	Cell Division	COG0206
KGF96991	CUST_5402_PI442172308	MIT9302	HLII	KGF96991	TAGAGGCAGCACAAGCAGCAATGAATAGTCCTTTACTAGAAGCAGCTAGAATTGATGGAG	ftsZ	cell division protein FtsZ	ASM75997	214956…216107	Cell Division	COG0206
KGF96991	CUST_5403_PI442172308	MIT9302	HLII	KGF96991	AATGTTGATTTCGCAGATGTTAGATCAGTTATGACAGAAGCAGGAACTGCTCTTCTTGGT	ftsZ	cell division protein FtsZ	ASM75997	214956…216107	Cell Division	COG0206
KGF97005	CUST_5467_PI442172308	MIT9302	HLII	KGF97005	TCATGACCCAGAGAAGGCAGCAGGAATTATACTGCTTGGATGGTTTTATGCCAAAAATT	glcH	GPH family sugar transporter	ASM75997	227550…228923	GlcH sugar transporter	COG2211
KGF97005	CUST_5468_PI442172308	MIT9302	HLII	KGF97005	TTTAAGCGTAGGATTATTTGGTTATCTTCTCACTCTTACTGGGTTCAAGCAAGGGGCCTG	glcH	GPH family sugar transporter	ASM75997	227550…228923	GlcH sugar transporter	COG2211
KGF97005	CUST_5469_PI442172308	MIT9302	HLII	KGF97005	GTTGGTTTTGGAGCGTCAACAGCATATTTAATACCCTGGTCTTTATTGCCAGACGCTATT	glcH	GPH family sugar transporter	ASM75997	227550…228923	GlcH sugar transporter	COG2211
KGF97005	CUST_5470_PI442172308	MIT9302	HLII	KGF97005	ATAGGTGGACTTATATGGATTGGTGGTTGCTTGATTGCAATGGTATTACCTCCATTGTCA	glcH	GPH family sugar transporter	ASM75997	227550…228923	GlcH sugar transporter	COG2211
KGF97005	CUST_5471_PI442172308	MIT9302	HLII	KGF97005	TGCAACAAGTCATGAATGTCCCAAGAGGGATATCCTTTTGGATTATCATTCCTTTCCAAA	glcH	GPH family sugar transporter	ASM75997	227550…228923	GlcH sugar transporter	COG2211
KGF97024	CUST_5578_PI442172308	MIT9302	HLII	KGF97024	ACTTCGAACAGATATTTAGTGGTGCCCCTCATAGAGTTGTTCCTGATCAGAATGTTCAGA	kaiC	circadian clock protein KaiC	ASM75997	246718…248247	Circadian Rhythm	COG0467
KGF97024	CUST_5579_PI442172308	MIT9302	HLII	KGF97024	AGGAGAGATGGCTAGAGCATTAAATGTTTTTAAAATGCGAGGATCATGGCATGATAAGCG	kaiC	circadian clock protein KaiC	ASM75997	246718…248247	Circadian Rhythm	COG0467
KGF97024	CUST_5580_PI442172308	MIT9302	HLII	KGF97024	AACCAAAGAGAATGGCAATAGATTCTCTCTCAGCACTAGCCCGAGGTGTCAGTTTAAATG	kaiC	circadian clock protein KaiC	ASM75997	246718…248247	Circadian Rhythm	COG0467
KGF97024	CUST_5581_PI442172308	MIT9302	HLII	KGF97024	ACTTTTTGCATATGAGGAATCTAGGGCTCAATTACTTAGAAATGCCACTAGTTGGGGAAT	kaiC	circadian clock protein KaiC	ASM75997	246718…248247	Circadian Rhythm	COG0467
KGF97024	CUST_5582_PI442172308	MIT9302	HLII	KGF97024	GTGGTGGAGGATATTTTCAAGATTCCATTATCCTCGCTACTGGAGCTACTGGTACAGGTA	kaiC	circadian clock protein KaiC	ASM75997	246718…248247	Circadian Rhythm	COG0467
KGF97024	CUST_5583_PI442172308	MIT9302	HLII	KGF97024	AGTAGACGGTATTAGTGTGTTTGCTCTAGGCGCAATGAGACTAACTCAAGGATCTTCGAA	kaiC	circadian clock protein KaiC	ASM75997	246718…248247	Circadian Rhythm	COG0467
KGF97025	CUST_5584_PI442172308	MIT9302	HLII	KGF97025	CGGTTTAGATCTACTTTTTGATGAATTAACTGAAACTGAATATAGTGGAGATAAAT	kaiB	Circadian clock protein KaiB	ASM75997	248319…248636	Circadian Rhythm	COG4251
KGF97025	CUST_5585_PI442172308	MIT9302	HLII	KGF97025	CCTCCACCGGTTAGAAGAATAATTGGTGATCTTTCAGATAGAGAAAAAGTTTTAATCGGT	kaiB	Circadian clock protein KaiB	ASM75997	248319…248636	Circadian Rhythm	COG4251
KGF97025	CUST_5586_PI442172308	MIT9302	HLII	KGF97025	TACTTAAGCAACCTCAACTTGCTGAGGAAGATAAGATTTTGGCAACCCCAACCTTAGCTA	kaiB	Circadian clock protein KaiB	ASM75997	248319…248636	Circadian Rhythm	COG4251
KGF97025	CUST_5587_PI442172308	MIT9302	HLII	KGF97025	ACTTTAAGAGAAATTCTAGAAAATGAATTCAAAGGTGTTTATGCCTTGAAGGTTATCGAT	kaiB	Circadian clock protein KaiB	ASM75997	248319…248636	Circadian Rhythm	COG4251
KGF97025	CUST_5588_PI442172308	MIT9302	HLII	KGF97025	AACATACATCTTAAAACTCTATGTTGCTGGGAATACTCCTAATTCAATGAGGGCTTTGAA	kaiB	Circadian clock protein KaiB	ASM75997	248319…248636	Circadian Rhythm	COG4251
KGF97159	CUST_6093_PI442172308	MIT9302	HLII	KGF97159	CCAGAGAAAATTAAAGCTCTTCAAGAGATTTCAAAAGCTGAGGCAAGGATTCAAGCTGCA	atpC	ATP synthase epsilon subunit	ASM75997	329703…330107	Energy Metabolism	COG0355
KGF97159	CUST_6094_PI442172308	MIT9302	HLII	KGF97159	TTGGATCCGAGATTGATGTTCAAAATGCTGAACAAGAACTTAAAGAAGCAAAATTAGCAA	atpC	ATP synthase epsilon subunit	ASM75997	329703…330107	Energy Metabolism	COG0355
KGF97159	CUST_6095_PI442172308	MIT9302	HLII	KGF97159	TGGGAGGCTTCGCTGAAATTGAATCAGACGAAGTCATAGTTTTAGTAAACAATGCGGAAA	atpC	ATP synthase epsilon subunit	ASM75997	329703…330107	Energy Metabolism	COG0355
KGF97159	CUST_6096_PI442172308	MIT9302	HLII	KGF97159	AGGTGTGCTTCCGGGACACATTTCTTTAGTTACAGCTATAGATATTGGCGTTTTAAGGCT	atpC	ATP synthase epsilon subunit	ASM75997	329703…330107	Energy Metabolism	COG0355
KGF97159	CUST_6097_PI442172308	MIT9302	HLII	KGF97159	ACAAAAATGTTTATCAAGGCGAAGCTGAAGAAGTGATTCTTCCTAGCACTACTGGGCAAC	atpC	ATP synthase epsilon subunit	ASM75997	329703…330107	Energy Metabolism	COG0355
KGF97171	CUST_6159_PI442172308	MIT9302	HLII	KGF97171	AGTTAGTAGAGTTGGAGGAGCTGCTCAAACAAAGGCAATTAAGAAAATTGCTGGAACACT	atpA	ATP synthase alpha subunit	ASM75997	339396…340913	Energy Metabolism	COG0056
KGF97171	CUST_6156_PI442172308	MIT9302	HLII	KGF97171	GATGATATGGGCGGTGGTTCAATGACAGCATTACCAATTATTGAAACTCAAGCTGGTGAT	atpA	ATP synthase alpha subunit	ASM75997	339396…340913	Energy Metabolism	COG0056
KGF97171	CUST_6157_PI442172308	MIT9302	HLII	KGF97171	TTGCAGTTGTGTATGCAGGTGTTAAGGGTCTTATTGACGAAGTTCCAGTGGAAGATGTAA	atpA	ATP synthase alpha subunit	ASM75997	339396…340913	Energy Metabolism	COG0056
KGF97171	CUST_6160_PI442172308	MIT9302	HLII	KGF97171	GCAAGCTCAAGCTTATAGACAAATGTCTCTCCTTTTAAGAAGACCACCAGGAAGAGAAGC	atpA	ATP synthase alpha subunit	ASM75997	339396…340913	Energy Metabolism	COG0056
KGF97171	CUST_6161_PI442172308	MIT9302	HLII	KGF97171	GGCTTAGAAGCAACACTCAAAGAGGTAATAAAAGAAGTTAAATCTTCAATGCTTGCCACA	atpA	ATP synthase alpha subunit	ASM75997	339396…340913	Energy Metabolism	COG0056
KGF97176	CUST_6184_PI442172308	MIT9302	HLII	KGF97176	CTAGTGCAATTCAGGCATTAATTTTTGCAACTCTAGCCGCCTACTACATTGGAGAAGCTG	atpI	ATP synthase subunit A	ASM75997	342937…343662	Energy Metabolism	COG0356
KGF97176	CUST_6185_PI442172308	MIT9302	HLII	KGF97176	GGAGAGTTGGGAGCACCTACTGCAGATATTAATACAACAATAGCCTTGGCACTATTGGTT	atpI	ATP synthase subunit A	ASM75997	342937…343662	Energy Metabolism	COG0356
KGF97176	CUST_6186_PI442172308	MIT9302	HLII	KGF97176	AGCACTAGAAGTTGGTCAACATCTTTATTGGCAAATTGGAAATATCAGACTTCATGGGCA	atpI	ATP synthase subunit A	ASM75997	342937…343662	Energy Metabolism	COG0356
KGF97176	CUST_6187_PI442172308	MIT9302	HLII	KGF97176	GTATTTGTAAGCAACTGGGGAGGTGCTTTAATTCCTTGGAGATTGATTAAGTTACCAAGT	atpI	ATP synthase subunit A	ASM75997	342937…343662	Energy Metabolism	COG0356
KGF97176	CUST_6188_PI442172308	MIT9302	HLII	KGF97176	TGCTGGTTTAAGTAATAAGGGTTGGAGATACTTTGAATACTATGTTCATCCAACTCCGAT	atpI	ATP synthase subunit A	ASM75997	342937…343662	Energy Metabolism	COG0356
KGF97176	CUST_6189_PI442172308	MIT9302	HLII	KGF97176	AGGACTTCAAAACTTGCTTGAGTTTCTTTGGGATTATATAAGAGATCTTGCTAGGACTCA	atpI	ATP synthase subunit A	ASM75997	342937…343662	Energy Metabolism	COG0356
KGF97186	CUST_6241_PI442172308	MIT9302	HLII	KGF97186	TTGGTTACAGCCATAGTGCCAAAAGTTGGTTATGAAAAAGCAGCTGAAATTGCAAACCTT	fumC	Fumarate hydratase class II	ASM75997	351351…352736	Krebs cycle and related genes	COG0114
KGF97186	CUST_6242_PI442172308	MIT9302	HLII	KGF97186	TGGGTCTTGAATATGCAGTTTCAATGGCAAATTCTAGCGGCACTTTACAGATGAATGAAT	fumC	Fumarate hydratase class II	ASM75997	351351…352736	Krebs cycle and related genes	COG0114
KGF97186	CUST_6243_PI442172308	MIT9302	HLII	KGF97186	AGTTCTATTATGCCGGGTAAAGTGAATCCAACTCAATGCGAAGCCTTATCAATGGTTTGC	fumC	Fumarate hydratase class II	ASM75997	351351…352736	Krebs cycle and related genes	COG0114
KGF97186	CUST_6244_PI442172308	MIT9302	HLII	KGF97186	TGGCCTCGCATGATCGTCTAGCTCAAGTAATGTGTCAGATAAAAATATTAGCAGGTGCAT	fumC	Fumarate hydratase class II	ASM75997	351351…352736	Krebs cycle and related genes	COG0114
KGF97186	CUST_6245_PI442172308	MIT9302	HLII	KGF97186	GGGAGGAACTGCAGTTGGTACAGGGATTAATTGTCCACAGGATTTTGTGAAGAGTCTAT	fumC	Fumarate hydratase class II	ASM75997	351351…352736	Krebs cycle and related genes	COG0114
KGF97186	CUST_6246_PI442172308	MIT9302	HLII	KGF97186	TCCCCTTACTTTTGGGCAAGAAATATCAGGATGGTCAGAGCAACTTAAAGATGCTGAGAA	fumC	Fumarate hydratase class II	ASM75997	351351…352736	Krebs cycle and related genes	COG0114
KGF98185	CUST_6714_PI442172308	MIT9302	HLII	KGF98185	TTGAGAGGGGCTCTTCTAACTATTAACGGAATAGCAGCAGGTATGAGAAATACAGGTTGA	ppc	Phosphoenolpyruvate carboxylase	ASM75997	33747…36716	Krebs cycle and related genes	COG2352
KGF98185	CUST_6715_PI442172308	MIT9302	HLII	KGF98185	TGGGATTTTTGCAGGTTTCACTTTTAAGAAGACTTAGAGATCAAACAAGGCAACCTCCAA	ppc	Phosphoenolpyruvate carboxylase	ASM75997	33747…36716	Krebs cycle and related genes	COG2352
KGF98185	CUST_6716_PI442172308	MIT9302	HLII	KGF98185	TTTCAAGAGGTCACGCCAATAGAAGAAATAAGCAAATTACAGATATCTAGTAGGCCTGCT	ppc	Phosphoenolpyruvate carboxylase	ASM75997	33747…36716	Krebs cycle and related genes	COG2352
KGF98185	CUST_6717_PI442172308	MIT9302	HLII	KGF98185	AGAGTTTTGCATCAAAGATGGCCATTTTTTAGGATGCTCATATCTAAGGTAGAAATGACA	ppc	Phosphoenolpyruvate carboxylase	ASM75997	33747…36716	Krebs cycle and related genes	COG2352
KGF98217	CUST_6891_PI442172308	MIT9302	HLII	KGF98217	AGAGCTCGATAGGAAGGCAGGTTTCAAACATAATTGACATTTGTGCAGGAATTGAAGTGG	glmS	Glutamine--fructose-6-phosphatetransaminase(ison	ASM75997	67850…69763	Amino Acid metabolism	COG0449
KGF98217	CUST_6892_PI442172308	MIT9302	HLII	KGF98217	ATTCATGCAGCTGGATATCCAGCTGGTGAAATGAAACATGGTCCAATAGCTTTGTTAGAT	glmS	Glutamine--fructose-6-phosphatetransaminase(ison	ASM75997	67850…69763	Amino Acid metabolism	COG0449
KGF98217	CUST_6893_PI442172308	MIT9302	HLII	KGF98217	AGAAGCAAAAGCTAGAGATTCATATTTGATTGGGATCGCTCCTGAATGTAATGGGACTGA	glmS	Glutamine--fructose-6-phosphatetransaminase(ison	ASM75997	67850…69763	Amino Acid metabolism	COG0449
KGF98217	CUST_6894_PI442172308	MIT9302	HLII	KGF98217	AGCAGCTCATAGAGGACTTGACGTTGATCAACCAAGAAATTTAGCTAAAAGTGTAACTGT	glmS	Glutamine--fructose-6-phosphatetransaminase(ison	ASM75997	67850…69763	Amino Acid metabolism	COG0449
KGF98217	CUST_6895_PI442172308	MIT9302	HLII	KGF98217	ACCTGTGATTTCTATTGCCTCCCCGGATGAAGTTTTTGATAAAGTTATAAGTAATGCTCA	glmS	Glutamine--fructose-6-phosphatetransaminase(ison	ASM75997	67850…69763	Amino Acid metabolism	COG0449
KGF98217	CUST_6896_PI442172308	MIT9302	HLII	KGF98217	ATGAAAGTTCCTTCCTCTAACGAATGGGTTTCACCTTTATTTAACATAGTGCCTTTACAA	glmS	Glutamine--fructose-6-phosphatetransaminase(ison	ASM75997	67850…69763	Amino Acid metabolism	COG0449
KGF97860	CUST_7838_PI442172308	MIT9302	HLII	KGF97860	ATGAGCAATATAGATGGCCTTTTCGTTTTAGGAGAGGCTAACTATTCTGTTCATGGCGCT	sdhA	Succinate dehydrogenase flavoprotein subunit	ASM75997	23045…24970	Krebs cycle and related genes	COG1053
KGF97860	CUST_7839_PI442172308	MIT9302	HLII	KGF97860	AAAGGGAGGAATCATGTGGAGCCCATTTCAGAGAAGAATATCAAACGAAGGAAGGAGAAG	sdhA	Succinate dehydrogenase flavoprotein subunit	ASM75997	23045…24970	Krebs cycle and related genes	COG1053
KGF97860	CUST_7840_PI442172308	MIT9302	HLII	KGF97860	TGAACGAATCGTTGGTGAAGATCCAACAAAAGTACCTATGAAGATTTATCCTGCCCCTCA	sdhA	Succinate dehydrogenase flavoprotein subunit	ASM75997	23045…24970	Krebs cycle and related genes	COG1053
KGF97860	CUST_7841_PI442172308	MIT9302	HLII	KGF97860	CGGCTGGGTGCAAATGCTTTATTGCAATGCTTGTGTGATGGATACTTTATATCACCAAAA	sdhA	Succinate dehydrogenase flavoprotein subunit	ASM75997	23045…24970	Krebs cycle and related genes	COG1053
KGF97860	CUST_7842_PI442172308	MIT9302	HLII	KGF97860	GAAAAACCTGTTGATTATTTCCATAGAGCGCTAGGGGAAATAATGATTTCGAAATGCGGT	sdhA	Succinate dehydrogenase flavoprotein subunit	ASM75997	23045…24970	Krebs cycle and related genes	COG1053
KGF97860	CUST_7843_PI442172308	MIT9302	HLII	KGF97860	TCTGGTTTAAATGTAGAGCTTGAGAAAGCTTTAAGGTTAGAAGACTTTATTGAGCTCTCA	sdhA	Succinate dehydrogenase flavoprotein subunit	ASM75997	23045…24970	Krebs cycle and related genes	COG1053
KGF97861	CUST_7844_PI442172308	MIT9302	HLII	KGF97861	GTGCGTAGCAGGATGTAAAAATGCATCATCAAGTCTTTTTGTAGCTGCGAAACTAGCTCA	sdhB	Succinate dehydrogenase iron-sulphur protein subu	ASM75997	24967…25698	Krebs cycle and related genes	COG0479
KGF97861	CUST_7845_PI442172308	MIT9302	HLII	KGF97861	ACCATGGCGAGCAAAATCATTCCCAATTATCCAAGACTTGTCAGTGGATAGAAATGCATT	sdhB	Succinate dehydrogenase iron-sulphur protein subu	ASM75997	24967…25698	Krebs cycle and related genes	COG0479
KGF97861	CUST_7846_PI442172308	MIT9302	HLII	KGF97861	GACAATGTAGTAATAATCTTGAATGCGAGGCTGTTTGCCCCAAAGAAATATCCGCCGATT	sdhB	Succinate dehydrogenase iron-sulphur protein subu	ASM75997	24967…25698	Krebs cycle and related genes	COG0479
KGF97861	CUST_7847_PI442172308	MIT9302	HLII	KGF97861	TAGAGAGGGAATATGTGGAAGTTGTGGTTTTTTAGTAAATGGTCAACCTCATGGGCCCAA	sdhB	Succinate dehydrogenase iron-sulphur protein subu	ASM75997	24967…25698	Krebs cycle and related genes	COG0479
KGF97861	CUST_7848_PI442172308	MIT9302	HLII	KGF97861	GGGTGGCTATTTCTCTACTGAAACTGGTAACGCACCCGATGCCAATTTAATTCCAATAAG	sdhB	Succinate dehydrogenase iron-sulphur protein subu	ASM75997	24967…25698	Krebs cycle and related genes	COG0479
KGF97861	CUST_7849_PI442172308	MIT9302	HLII	KGF97861	AGGACAACTCCCTCAAGGGATTAATGATAAAAAAGAAAGGTCAAAAAAAATGCGAAAACA	sdhB	Succinate dehydrogenase iron-sulphur protein subu	ASM75997	24967…25698	Krebs cycle and related genes	COG0479
KGF96482	CUST_8116_PI442172308	MIT9302	HLII	KGF96482	AGATATGCTTCAAAATCATATGACTCAAATGCTTGCTGTTACGGCAATGGAACCTCCTGG	zwf	glucose-6-phosphate dehydrogenase zwf	ASM75997	47282…48805	Pentose Phosphate	COG0364
KGF96482	CUST_8113_PI442172308	MIT9302	HLII	KGF96482	GCAGCAAAAGTTTTTGTTGATAACTGGCGTTGGCAAGGGGTACCTTTTTATTTGAGAACA	zwf	glucose-6-phosphate dehydrogenase zwf	ASM75997	47282…48805	Pentose Phosphate	COG0364
KGF96482	CUST_8114_PI442172308	MIT9302	HLII	KGF96482	GGGGACCTCCTGAGTCTGATCAATTACTTTCAAAAGATAATATTTTCTGGCGCAGACCCT	zwf	glucose-6-phosphate dehydrogenase zwf	ASM75997	47282…48805	Pentose Phosphate	COG0364
KGF96482	CUST_8115_PI442172308	MIT9302	HLII	KGF96482	TGAAGGCGCTACTTTTAAATTCGAGGTAAAGTCACCTGGTTCCGGAATGAAATCTAGACC	zwf	glucose-6-phosphate dehydrogenase zwf	ASM75997	47282…48805	Pentose Phosphate	COG0364
KGF96482	CUST_8117_PI442172308	MIT9302	HLII	KGF96482	GCTTCAAAACTGGCTGACGAAAATGAACCGTGGCATTGTTGTATAAGAGGACAATATGGA	zwf	glucose-6-phosphate dehydrogenase zwf	ASM75997	47282…48805	Pentose Phosphate	COG0364
KGF96482	CUST_8118_PI442172308	MIT9302	HLII	KGF96482	GCTGATGCAATGCTTTCTGATCCAACCTTATTTACAAGAAGTGATGAGGTAGAAGCTGCC	zwf	glucose-6-phosphate dehydrogenase zwf	ASM75997	47282…48805	Pentose Phosphate	COG0364
KGF96483	CUST_8119_PI442172308	MIT9302	HLII	KGF96483	TGAAAAATTGGAGGGAATCATTGGCTATGATTTTTGATCCGCCATCGAGGAGACCAATTT	opcA	Glucose-6-P dehydrogenase subunit opcA	ASM75997	48808…50109	Pentose Phosphate	COG3429
KGF96483	CUST_8120_PI442172308	MIT9302	HLII	KGF96483	ACCTTCTTGGTTGTGGTGGAACGGAAGCTTGGATGAATCACCTGAAATTTTTGAATACTT	opcA	Glucose-6-P dehydrogenase subunit opcA	ASM75997	48808…50109	Pentose Phosphate	COG3429
KGF96483	CUST_8121_PI442172308	MIT9302	HLII	KGF96483	TTGTGTAATACTTGGATGTGAGTCAGTGGAATGTATGAGACTTGAAGCAGGAGGAATGGC	opcA	Glucose-6-P dehydrogenase subunit opcA	ASM75997	48808…50109	Pentose Phosphate	COG3429
KGF96483	CUST_8122_PI442172308	MIT9302	HLII	KGF96483	CGCAGGAGATCATATGATTCAGGCTTTGTTTTTGATCTCATGGATTAGCGATAAACTTGA	opcA	Glucose-6-P dehydrogenase subunit opcA	ASM75997	48808…50109	Pentose Phosphate	COG3429
KGF96483	CUST_8123_PI442172308	MIT9302	HLII	KGF96483	TCATGGTCAAAGGTTAATAATTGATACTGCTCTTGGATCGCCTAACAGATGTTTAAAAGT	opcA	Glucose-6-P dehydrogenase subunit opcA	ASM75997	48808…50109	Pentose Phosphate	COG3429
KGF96483	CUST_8124_PI442172308	MIT9302	HLII	KGF96483	TCCTTTACCTTTGGGTAATCCAAGTATTCATTTAGGACAAGTTATTGGATTGAGGTTGAT	opcA	Glucose-6-P dehydrogenase subunit opcA	ASM75997	48808…50109	Pentose Phosphate	COG3429
KGF98647	CUST_8467_PI442172308	MIT9302	HLII	KGF98647	ATTATTTGCATTAGGTAAATCGGGAGAAAGATTTGCAGTAAGGAATAGTGGCGCCACAGC	glsF	Ferredoxin-dependent glutamate synthase	ASM75997	18160…22734	Nitrogen Metabolism	COG0069
KGF98647	CUST_8468_PI442172308	MIT9302	HLII	KGF98647	GCGAAATCGCAGAACTTCATGGCAACACAGGATTCAATGGCGAAATCAACTTAAATTTCA	glsF	Ferredoxin-dependent glutamate synthase	ASM75997	18160…22734	Nitrogen Metabolism	COG0069
KGF98647	CUST_8469_PI442172308	MIT9302	HLII	KGF98647	AAGGAATATTGGTGCGGGCATGACTGGTGGAATAGCTTTCATACTCGATGAGAATAATGA	glsF	Ferredoxin-dependent glutamate synthase	ASM75997	18160…22734	Nitrogen Metabolism	COG0069
KGF98647	CUST_8470_PI442172308	MIT9302	HLII	KGF98647	AAAGCTCCTCTGAGCAAGTTATTTTAGGAAACACCTGTCTTTATGGAGCAACAGGAGGAA	glsF	Ferredoxin-dependent glutamate synthase	ASM75997	18160…22734	Nitrogen Metabolism	COG0069
KGF98647	CUST_8471_PI442172308	MIT9302	HLII	KGF98647	AACAGAGGGAGCAGGTGACCATTGTTGTGAATATATGACTGGTGGGAAAGTAGTTATTCT	glsF	Ferredoxin-dependent glutamate synthase	ASM75997	18160…22734	Nitrogen Metabolism	COG0069
KGF98647	CUST_8472_PI442172308	MIT9302	HLII	KGF98647	TCAAGAGTTCTTTCAAATTAATAGTTCCCCCAAGCGAGGAAGAGATGCTTGGCATAAAGA	glsF	Ferredoxin-dependent glutamate synthase	ASM75997	18160…22734	Nitrogen Metabolism	COG0069
KGF98658	CUST_8521_PI442172308	MIT9302	HLII	KGF98658	GTTAACTCCTTATCTCCTTGGGCTTGGATGTTCTTATTTGGCCATCTAGTTTGGGCTACT	psaB	Photosystem I P700 chlorophyll A apoprotein A2 Psa	ASM75997	29109…31337	Photosystem I	COG2885
KGF98658	CUST_8523_PI442172308	MIT9302	HLII	KGF98658	AGCAATGTTCTGGGCATTAAATCTAATTGCATGGGTAACTTTCTACTGGCATTGGAAGCA	psaB	Photosystem I P700 chlorophyll A apoprotein A2 Psa	ASM75997	29109…31337	Photosystem I	COG2885
KGF98658	CUST_8524_PI442172308	MIT9302	HLII	KGF98658	CTATTGGACCAGCAGATTTCTTAGTACATCACGCTATCGCACTTGGACTTCATACCACAG	psaB	Photosystem I P700 chlorophyll A apoprotein A2 Psa	ASM75997	29109…31337	Photosystem I	COG2885
KGF98658	CUST_8525_PI442172308	MIT9302	HLII	KGF98658	AAATTAATTCCTGATAAAAAGGACTTAGGTTATGCTTTCCCTTGCGACGGTCCAGGTCGT	psaB	Photosystem I P700 chlorophyll A apoprotein A2 Psa	ASM75997	29109…31337	Photosystem I	COG2885
KGF98658	CUST_8526_PI442172308	MIT9302	HLII	KGF98658	TCCTACGAGTTCTGCAACTATAGCTGCAAATTCTATGCCAGGAAATCACTATTGGATGGA	psaB	Photosystem I P700 chlorophyll A apoprotein A2 Psa	ASM75997	29109…31337	Photosystem I	COG2885
KGF98659	CUST_8527_PI442172308	MIT9302	HLII	KGF98659	TCAAGACTAATCCCTGATAAGGCTTCTCTTGGATTTAGATTCCCTTGTGATGGTCCTGGA	psaA	Photosystem I P700 chlorophyll A apoprotein A1 Psa	ASM75997	31365…33668	Photosystem I	COG2885
KGF98659	CUST_8528_PI442172308	MIT9302	HLII	KGF98659	TGCCCAATGGATCCAAAGTATTCAAGCTTCTGCTGTAGGTACTTCAATCTTAGCTGGTAC	psaA	Photosystem I P700 chlorophyll A apoprotein A1 Psa	ASM75997	31365…33668	Photosystem I	COG2885
KGF98659	CUST_8529_PI442172308	MIT9302	HLII	KGF98659	CAACAATACAACCAAGAGCACTTTCAATTACTCAAGGTAGAGCCGTTGGAGTAGCTCACT	psaA	Photosystem I P700 chlorophyll A apoprotein A1 Psa	ASM75997	31365…33668	Photosystem I	COG2885
KGF98659	CUST_8530_PI442172308	MIT9302	HLII	KGF98659	AAGCGATGTTTGGGGACTAACTGGAGGAAACTTCTCACAAAGTGGTATCACTATCAATGG	psaA	Photosystem I P700 chlorophyll A apoprotein A1 Psa	ASM75997	31365…33668	Photosystem I	COG2885
KGF98659	CUST_8531_PI442172308	MIT9302	HLII	KGF98659	AGGTGGAAAAGTTGCAATAGCTCCAATTCAACTTGGAACTGCTGATTTGATGATCCATCA	psaA	Photosystem I P700 chlorophyll A apoprotein A1 Psa	ASM75997	31365…33668	Photosystem I	COG2885
KGF98659	CUST_8532_PI442172308	MIT9302	HLII	KGF98659	TGGTGAAGCTATTAGCATGTATGGATTGATGTTCTTAGGAGCTCACTTCATATGGGCATT	psaA	Photosystem I P700 chlorophyll A apoprotein A1 Psa	ASM75997	31365…33668	Photosystem I	COG2885
KGF98458	CUST_9058_PI442172308	MIT9302	HLII	KGF98458	GAGAAGTTGAAGGCAATGGCAGTGGATACATCGAAATTTGGATTAACTAAGGAATCGGCC	glgC	Glucose-1-phosphate adenylyltransferase	ASM75997	92039…93334	Glycogen Metabolism	COG0448
KGF98458	CUST_9059_PI442172308	MIT9302	HLII	KGF98458	AGGAGGTGGAACACCTCTTGGAGTAGGTGAAGGAACTACTGTAAAAAGAGCAATTCTTGA	glgC	Glucose-1-phosphate adenylyltransferase	ASM75997	92039…93334	Glycogen Metabolism	COG0448
KGF98458	CUST_9060_PI442172308	MIT9302	HLII	KGF98458	GGTGTAAGAAGCAGGATTGAAAGTGATTCTGTTCTTGAAGACACTTTGGTTATGGGTGCA	glgC	Glucose-1-phosphate adenylyltransferase	ASM75997	92039…93334	Glycogen Metabolism	COG0448
KGF98458	CUST_9061_PI442172308	MIT9302	HLII	KGF98458	CTCCCCCCTTCTAAACTTGTAGATGCTCAAATTACTGATTCAATAGTTTGCGAAGGAACA	glgC	Glucose-1-phosphate adenylyltransferase	ASM75997	92039…93334	Glycogen Metabolism	COG0448
KGF98458	CUST_9062_PI442172308	MIT9302	HLII	KGF98458	GGAACCATTGGAGCATTCTTTGAATCAAATTTAGCATTAACAGAACAACCAAAACCTCCA	glgC	Glucose-1-phosphate adenylyltransferase	ASM75997	92039…93334	Glycogen Metabolism	COG0448
KGF98459	CUST_9066_PI442172308	MIT9302	HLII	KGF98459	TGGTTTGAATATGCCATCAATTGCACAAATATGGAAAGGAGGTTGCATAATCAGATCAAG	edd	6-Phosphogluconate dehydratase	ASM75997	93429…94847	Pentose Phosphate/Entner-Dudorof	COG2084
KGF98458	CUST_9063_PI442172308	MIT9302	HLII	KGF98458	AGGATAGGCGATAATGTTGTTATTATCAACAAAGATAGAGTTGAAGAAGCAGATAAGCCA	glgC	Glucose-1-phosphate adenylyltransferase	ASM75997	92039…93334	Glycogen Metabolism	COG0448
KGF98459	CUST_9064_PI442172308	MIT9302	HLII	KGF98459	AGAACAAATAGACTCCCTCAGAACTTAGTGCAGGCTATGAGAGACTGTTTTGGCTCTCAT	edd	6-Phosphogluconate dehydratase	ASM75997	93429…94847	Pentose Phosphate/Entner-Dudorof	COG2084
KGF98459	CUST_9065_PI442172308	MIT9302	HLII	KGF98459	TGGATAACTTAGCTAATGTAGTTTCTTTATCAACAAAAGCTGGTATACCCGTTCCATGTT	edd	6-Phosphogluconate dehydratase	ASM75997	93429…94847	Pentose Phosphate/Entner-Dudorof	COG2084
KGF98459	CUST_9067_PI442172308	MIT9302	HLII	KGF98459	TGATGCCGTAGTCCTTGCCACAATAGCAAGCTATGCTCAAGGAATGGATATTTTAAGAGA	edd	6-Phosphogluconate dehydratase	ASM75997	93429…94847	Pentose Phosphate/Entner-Dudorof	COG2084
KGF98459	CUST_9068_PI442172308	MIT9302	HLII	KGF98459	GATAAAGCTGGCCAGAAAGGTACTGGGTTATGGACTGTTGTAAGTGCTTTAGAACTAGGG	edd	6-Phosphogluconate dehydratase	ASM75997	93429…94847	Pentose Phosphate/Entner-Dudorof	COG2084
KGF98459	CUST_9069_PI442172308	MIT9302	HLII	KGF98459	TGCAGAAGCTTATGACCTTATGAAGAGAGTCAAAGGTATGAATGGACAGCAGATGTCAGA	edd	6-Phosphogluconate dehydratase	ASM75997	93429…94847	Pentose Phosphate/Entner-Dudorof	COG2084
KGF98471	CUST_9128_PI442172308	MIT9302	HLII	KGF98471	GATACAAGCAGTTCTGGTGCGAAGGTCAAGCAAGTAAGATCAAGCAAAACAGCACTAATT	cbbA	Fructose-1,6-bisphosphate aldolase class II	ASM75997	102115…103188	Glycolysis/Gluconeogenesis	COG0191
KGF98471	CUST_9130_PI442172308	MIT9302	HLII	KGF98471	TACATCTGTAATGATGGATGGTTCCTTAGAGGCAGATGCAAAAACACCAGCAAGTTACGA	cbbA	Fructose-1,6-bisphosphate aldolase class II	ASM75997	102115…103188	Glycolysis/Gluconeogenesis	COG0191
KGF98471	CUST_9133_PI442172308	MIT9302	HLII	KGF98471	GTCAGGAAAGTTAACATTGATACCGATAACAGACTTGCATTCACTGCCGCGGTTAGAGAG	cbbA	Fructose-1,6-bisphosphate aldolase class II	ASM75997	102115…103188	Glycolysis/Gluconeogenesis	COG0191
KGF98471	CUST_9131_PI442172308	MIT9302	HLII	KGF98471	CCATAAAGCACTTCCAAATACCCATCTTGTAATGCATGGATCTAGTTCAGTGCCTCAGGA	cbbA	Fructose-1,6-bisphosphate aldolase class II	ASM75997	102115…103188	Glycolysis/Gluconeogenesis	COG0191
KGF98471	CUST_9132_PI442172308	MIT9302	HLII	KGF98471	ACTTCGACCCAAGACATTTCAACAAGCCTGCTAGAAAGTACATGAAGCAAGTTTGTCTTG	cbbA	Fructose-1,6-bisphosphate aldolase class II	ASM75997	102115…103188	Glycolysis/Gluconeogenesis	COG0191
KGF98475	CUST_9150_PI442172308	MIT9302	HLII	KGF98475	GAAGCCGAAGAACATAATACAAGACTTGGGACATCCTTAGAACCAGGGCAATTTACTCCA	prk	Phosphoribulokinase	ASM75997	105796…106695	Carbon Fixation	COG3954
KGF98475	CUST_9151_PI442172308	MIT9302	HLII	KGF98475	GGGGCACGAGAAAAATGGGGAATTGATTTCCAATACTTGCTAGGAATGATTCACGATTCA	prk	Phosphoribulokinase	ASM75997	105796…106695	Carbon Fixation	COG3954
KGF98475	CUST_9152_PI442172308	MIT9302	HLII	KGF98475	GCCATGGCAGAAGCTCTATCAAAAGGTGAAAATTTTTCTCACTTTGGTCCAGAGGCTAAT	prk	Phosphoribulokinase	ASM75997	105796…106695	Carbon Fixation	COG3954
KGF98475	CUST_9153_PI442172308	MIT9302	HLII	KGF98475	TCCTGAAGGAACAGACGTACTTTTTTATGAAGGTTTACATGGCGGTGTAGAAGGTGAAGG	prk	Phosphoribulokinase	ASM75997	105796…106695	Carbon Fixation	COG3954
KGF98475	CUST_9154_PI442172308	MIT9302	HLII	KGF98475	AATGCAGAAAGAGGTTACTCAGCTGAAACCATTGTGGATACCATCTTGAGAAGAATGCCT	prk	Phosphoribulokinase	ASM75997	105796…106695	Carbon Fixation	COG3954
KGF98475	CUST_9155_PI442172308	MIT9302	HLII	KGF98475	ATGTCAAGTCCAACCAGTATTGTAGTTAATGGTGGGAAAATGGGTTTCGCAATGGAACTT	prk	Phosphoribulokinase	ASM75997	105796…106695	Carbon Fixation	COG3954
KGF98483	CUST_9199_PI442172308	MIT9302	HLII	KGF98483	AAGCGGTACAATTACACCTTACGACAAGAGTATAGTTATGGGTCCTATCAATGCGGAGTT		ABC-typesugartransportsystem	ASM75997	112594…113760	Other sugar transporters	
KGF98483	CUST_9198_PI442172308	MIT9302	HLII	KGF98483	TTCACCCTACAAGCAGTCTCGCAACAGCTTGCGGCATGCAATTATTAATAAATAGAATCG		ABC-typesugartransportsystem	ASM75997	112594…113760	Other sugar transporters	
KGF98483	CUST_9200_PI442172308	MIT9302	HLII	KGF98483	AGATGTTGCTGTAGTCGATGTTAATGATCTTGGCGGTGTGAAAGTCTTAGCTAGTTCAAA		ABC-typesugartransportsystem	ASM75997	112594…113760	Other sugar transporters	
KGF98483	CUST_9201_PI442172308	MIT9302	HLII	KGF98483	ACAGCAAAGGAATTGTAGAAAAAAATGATATTTTAACAATCGGTGAGAGTCCGCTAGCGA		ABC-typesugartransportsystem	ASM75997	112594…113760	Other sugar transporters	
KGF98483	CUST_9202_PI442172308	MIT9302	HLII	KGF98483	ACTCAAAAGAAACTTAATATCTAATCCAGCTGGCAATGGAGATGAAAAAACCCCTATAGT		ABC-typesugartransportsystem	ASM75997	112594…113760	Other sugar transporters	
KGF98483	CUST_9203_PI442172308	MIT9302	HLII	KGF98483	TGGTTAAAAGCATATTGGGAGAACTATGGACATTTTGGTATTTCACATAATAAGGATTGT		ABC-typesugartransportsystem	ASM75997	112594…113760	Other sugar transporters	
KGF98556	CUST_9565_PI442172308	MIT9302	HLII	KGF98556	TATGGCTGTTGGAGCAGCAATGACTGGGTTAAGACCAATAGTAGAAGGAATGAATATGGG	pdhB	Pyruvate dehydrogenase E1 component beta subuni	ASM75997	170331…171314	Glycolysis/Krebs cycle	COG0022
KGF98556	CUST_9566_PI442172308	MIT9302	HLII	KGF98556	GAGGAGTTGGTCGTCAACTTGGTGCTGAACACAGTCAAAGACTTGAAGCATATTTTCATG	pdhB	Pyruvate dehydrogenase E1 component beta subuni	ASM75997	170331…171314	Glycolysis/Krebs cycle	COG0022
KGF98556	CUST_9567_PI442172308	MIT9302	HLII	KGF98556	CAGAATTAATTGCCTTGATAACAGAAGAGTGCTTCGATGAACTTGATGCCCGGCCAATTA	pdhB	Pyruvate dehydrogenase E1 component beta subuni	ASM75997	170331…171314	Glycolysis/Krebs cycle	COG0022
KGF98556	CUST_9568_PI442172308	MIT9302	HLII	KGF98556	TGTTGCATGCAGTACACCCACAAATGCTAAAGGTTTAATGAAAGCGGCTATAAGAGATGA	pdhB	Pyruvate dehydrogenase E1 component beta subuni	ASM75997	170331…171314	Glycolysis/Krebs cycle	COG0022
KGF98556	CUST_9569_PI442172308	MIT9302	HLII	KGF98556	CTTGTCTGAAGAATTACCTGATGGTGACTATACTTGTGCTTTAGATCAGGCTGACGTGGT	pdhB	Pyruvate dehydrogenase E1 component beta subuni	ASM75997	170331…171314	Glycolysis/Krebs cycle	COG0022
KGF98556	CUST_9570_PI442172308	MIT9302	HLII	KGF98556	GGGTATGCTTAGATATACCAGTGGAGGAAATTATAAGATACCAGCAGTAGTTCGCGGACC	pdhB	Pyruvate dehydrogenase E1 component beta subuni	ASM75997	170331…171314	Glycolysis/Krebs cycle	COG0022
KGF98567	CUST_9623_PI442172308	MIT9302	HLII	KGF98567	GCCGGCCAACATGAGCTTGGAATGAAATTTGATTCTTTAATAAATGCTGCCGATAACGTT	glnA	Glutamine synthetase/ glutamate--ammonialigase	ASM75997	179091…180512	Nitrogen Metabolism	COG0174
KGF98567	CUST_9624_PI442172308	MIT9302	HLII	KGF98567	GAAGAGTGGACAGCCACTATTCTTTGGTGAAGGTGCCTATGCAAATTTATCTCAAACAGC	glnA	Glutamine synthetase/ glutamate--ammonialigase	ASM75997	179091…180512	Nitrogen Metabolism	COG0174
KGF98567	CUST_9625_PI442172308	MIT9302	HLII	KGF98567	AATAGATCTGCTGCAGTCAGAATACCATTAACTGGGCCAAGCCCAAAAGCTAAGAGATTA	glnA	Glutamine synthetase/ glutamate--ammonialigase	ASM75997	179091…180512	Nitrogen Metabolism	COG0174
KGF98567	CUST_9626_PI442172308	MIT9302	HLII	KGF98567	ACTCCCAGCCGATGAACTGGCAAAAATTGATACCGTACCTTCATCTCTAAATGATTCACT	glnA	Glutamine synthetase/ glutamate--ammonialigase	ASM75997	179091…180512	Nitrogen Metabolism	COG0174
KGF98567	CUST_9627_PI442172308	MIT9302	HLII	KGF98567	CAGGTGATGCACTAGCCAACCCATATCTAGCTTTCTCTGTAATGATGCTAGCTGGTATTG	glnA	Glutamine synthetase/ glutamate--ammonialigase	ASM75997	179091…180512	Nitrogen Metabolism	COG0174
KGF98567	CUST_9628_PI442172308	MIT9302	HLII	KGF98567	TGGGAAAACTGCAACATTTATGCCTAAACCTGTATTTAACGATAATGGAACTGGAATGCA	glnA	Glutamine synthetase/ glutamate--ammonialigase	ASM75997	179091…180512	Nitrogen Metabolism	COG0174
KGF98576	CUST_9669_PI442172308	MIT9302	HLII	KGF98576	TGGTGACCTAGTAACCTTGCAGCTTAATGAAGGGGTAATTTATATGGGACAAATTGAAGA	pykF	Pyruvate kinase	ASM75997	187739…189532	Glycolysis	COG0469
KGF98576	CUST_9670_PI442172308	MIT9302	HLII	KGF98576	AGAAAGATTCTTTCTAGAGGGATTTCGATAGGTGAAATTGGAGTTACAGGTAAAGCAAGA	pykF	Pyruvate kinase	ASM75997	187739…189532	Glycolysis	COG0469
KGF98576	CUST_9674_PI442172308	MIT9302	HLII	KGF98576	GCAAACCATGGCCACCATAGCAAGAAGAATTGAAAGGGATTATCCACTCAAAGCTATTGA	pykF	Pyruvate kinase	ASM75997	187739…189532	Glycolysis	COG0469
KGF98576	CUST_9671_PI442172308	MIT9302	HLII	KGF98576	TTGCTCAAGAAATGGGGATCTTGAATCAAGGTGATTTAGTGGTTCAAACTGCAGGTACAC	pykF	Pyruvate kinase	ASM75997	187739…189532	Glycolysis	COG0469

KGF98576	CUST_9672_PI442172308	MIT9302	HLII	KGF98576	TTTTGGCGACTACTACTGAGAGGAGTGTAGCTAGAAGACTTCAATTAGTTTGGGGCGTTA	pykF	Pyruvate kinase	ASM75997		187739...189532	Glycolysis	COG0469
KGF98576	CUST_9673_PI442172308	MIT9302	HLII	KGF98576	AGCTAGACAACTAGATGCAGGAGCAATAATTCCTTTAACAAAATCAGGCTCAACTGCTCG	pykF	Pyruvate kinase	ASM75997		187739...189532	Glycolysis	COG0469
KGF98597	CUST_9780_PI442172308	MIT9302	HLII	KGF98597	TCATCTGCATGGTATTTAACTGGTGATGGCGTTGGGAAGAGAGATATGGTTGTTTTACCT	pgi	Glucose-6-phosphate isomerase	ASM75997		208425...210008	Glycolysis/Gluconeogenesis	COG0166
KGF98597	CUST_9781_PI442172308	MIT9302	HLII	KGF98597	AGAGGTGCATCATTAATGGATGAGGCAACACGTACAAGCGATTTTAAAAATAATCCCGCA	pgi	Glucose-6-phosphate isomerase	ASM75997		208425...210008	Glycolysis/Gluconeogenesis	COG0166
KGF98597	CUST_9782_PI442172308	MIT9302	HLII	KGF98597	TTCATCCTCCGTGAAATGTGTTTCGGTAATGATGATTATTTAGTTAAGGGCGACTGGTCA	pgi	Glucose-6-phosphate isomerase	ASM75997		208425...210008	Glycolysis/Gluconeogenesis	COG0166
KGF98597	CUST_9783_PI442172308	MIT9302	HLII	KGF98597	AAATGCATATGATCAACCTGGTGTTGAAGCTGGAAAGAAAGCAGCTGCAAATATTATTGA	pgi	Glucose-6-phosphate isomerase	ASM75997		208425...210008	Glycolysis/Gluconeogenesis	COG0166
KGF98597	CUST_9784_PI442172308	MIT9302	HLII	KGF98597	AGGAACCAGATCCGCACTTTCTAGTGAAAACAGACAAAGTATCACTATCACATTAGAAGA	pgi	Glucose-6-phosphate isomerase	ASM75997		208425...210008	Glycolysis/Gluconeogenesis	COG0166
KGF98597	CUST_9785_PI442172308	MIT9302	HLII	KGF98597	TGTTTTTCACTAGGTTCCTTAATCGCTTTATTTGAGAGGGCTGTATCTTTCTATGCTGAA	pgi	Glucose-6-phosphate isomerase	ASM75997		208425...210008	Glycolysis/Gluconeogenesis	COG0166
ABM77194.1_438	CUST_2218_PI442172314	MIT9303	LLIV	ABM77194.1_438	GATATAACACCGGTGGATTCCCTGGTGAGTTTGTGTTTGCGCTTCATTCTTAGTGTC	glcH	GPH family sugar transporter		ABM77194.1	447714..449483	GlcH sugar transporter	COG2211
ABM77194.1_438	CUST_2219_PI442172314	MIT9303	LLIV	ABM77194.1_438	CTAGCTATCTCACCCACGATCTTTACCAGCGTTTTATAAGGCCGGATGCTGATCAGAGAG	glcH	GPH family sugar transporter		ABM77194.1	447714..449483	GlcH sugar transporter	COG2211
ABM77194.1_438	CUST_2220_PI442172314	MIT9303	LLIV	ABM77194.1_438	GGTCAATTCACCAGTGTTTTGCTGCTAGTTCTCGGGGTGATCACCGCATTAATTAG	glcH	GPH family sugar transporter		ABM77194.1	447714..449483	GlcH sugar transporter	COG2211
ABM77194.1_438	CUST_2221_PI442172314	MIT9303	LLIV	ABM77194.1_438	CTTGGTGACAATTCCTGATTACGGCACCAGGTTGATGATCACCACAGCGATTACTG	glcH	GPH family sugar transporter		ABM77194.1	447714..449483	GlcH sugar transporter	COG2211
ABM77194.1_438	CUST_2222_PI442172314	MIT9303	LLIV	ABM77194.1_438	ATTAGCGACAGCATCGGCACAGTGTTTCGTCTGGTGATTGCTATTGGTACC	glcH	GPH family sugar transporter		ABM77194.1	447714..449483	GlcH sugar transporter	COG2211
ABM77194.1_438	CUST_2223_PI442172314	MIT9303	LLIV	ABM77194.1_438	GTCTTGTCGTGGTCTCTCTGGTGGCGGCTTTCATGAGTACGGTCAGTAC	glcH	GPH family sugar transporter		ABM77194.1	447714..449483	GlcH sugar transporter	COG2211
ABM77690.1_934	CUST_4713_PI442172314	MIT9303	LLIV	ABM77690.1_934	GATCGACTGGGATGGCAATTAAAAGCCGCCCAGAAAATAAAAAGGAAGGCATTG	opcA	Glucose-6-P dehydrogenase subunit opcA		ABM77690.1	851724..853013	Pentose Phosphate	COG3429
ABM77690.1_934	CUST_4714_PI442172314	MIT9303	LLIV	ABM77690.1_934	CAATGCCCTTAGTCATGTTGATCAGCTCGAAATCGACGTTGAGGGTCATCACC	opcA	Glucose-6-P dehydrogenase subunit opcA		ABM77690.1	851724..853013	Pentose Phosphate	COG3429
ABM77690.1_934	CUST_4715_PI442172314	MIT9303	LLIV	ABM77690.1_934	CTGATTGAAGAAGTCGTCCCCATGCAGGTCGCGTCTGTAGAGATGGATGTAG	opcA	Glucose-6-P dehydrogenase subunit opcA		ABM77690.1	851724..853013	Pentose Phosphate	COG3429
ABM77690.1_934	CUST_4716_PI442172314	MIT9303	LLIV	ABM77690.1_934	GATCATCACGCCCTAACAGATGGGCTGGGAATCCTTCAACCTCTACTACCTG	opcA	Glucose-6-P dehydrogenase subunit opcA		ABM77690.1	851724..853013	Pentose Phosphate	COG3429
ABM77690.1_934	CUST_4717_PI442172314	MIT9303	LLIV	ABM77690.1_934	GAATGATCTGAATTGGTTAAGGCTGCGCACTTGGCGAGAACACCTCGCAATGGTGTTTG	opcA	Glucose-6-P dehydrogenase subunit opcA		ABM77690.1	851724..853013	Pentose Phosphate	COG3429
ABM77690.1_934	CUST_4718_PI442172314	MIT9303	LLIV	ABM77690.1_934	CTGTTCAGTTTCGTCTTATGCCTGTACCCATGGGGCAACCGAGCATTCATC	opcA	Glucose-6-P dehydrogenase subunit opcA		ABM77690.1	851724..853013	Pentose Phosphate	COG3429
ABM77691.1_935	CUST_4719_PI442172314	MIT9303	LLIV	ABM77691.1_935	CTCTACTGGAACTGATTGAAGACAGCCCATGGCAATTGCCCATTCATCCCTATGAATC	zwf	glucose-6-phosphate dehydrogenase zwf		ABM77691.1	853055..854578	Pentose Phosphate	COG0364
ABM77691.1_935	CUST_4720_PI442172314	MIT9303	LLIV	ABM77691.1_935	GATACGTCCGACTTTTGGCCGATGCCATGCTCAGTGACCCAACTTTATTTACC	zwf	glucose-6-phosphate dehydrogenase zwf		ABM77691.1	853055..854578	Pentose Phosphate	COG0364
ABM77691.1_935	CUST_4721_PI442172314	MIT9303	LLIV	ABM77691.1_935	CAGTAGAGATGGAATTCTCCTATGACGAATCCTTCGGTGAGCCATCCGATGAGGGATAC	zwf	glucose-6-phosphate dehydrogenase zwf		ABM77691.1	853055..854578	Pentose Phosphate	COG0364
ABM77691.1_935	CUST_4722_PI442172314	MIT9303	LLIV	ABM77691.1_935	CCTTTTACGTTCGTACTGGCAAACGACTGGCAAAGCGTCTTAGTGAAGTGGTG	zwf	glucose-6-phosphate dehydrogenase zwf		ABM77691.1	853055..854578	Pentose Phosphate	COG0364
ABM77691.1_935	CUST_4723_PI442172314	MIT9303	LLIV	ABM77691.1_935	GATCCCAGCAGCACCACCGAGACCTATGTAGCGATGAAACTTTTCATCGACAACTG	zwf	glucose-6-phosphate dehydrogenase zwf		ABM77691.1	853055..854578	Pentose Phosphate	COG0364
ABM77691.1_935	CUST_4724_PI442172314	MIT9303	LLIV	ABM77691.1_935	CAAATCACGGCCGCTGAAACCGTAGGTGTTGAAGAACGAGCCAACTACTACGAATCTTC	zwf	glucose-6-phosphate dehydrogenase zwf		ABM77691.1	853055..854578	Pentose Phosphate	COG0364
ABM78251.1_1495	CUST_7168_PI442172314	MIT9303	LLIV	ABM78251.1_1495	GCTGTATATGGCAACAAGGGTTCAACTGATCAACATGCTTATGTACAGCAATTACGCGAT	pgi	Glucose-6-phosphate isomerase		ABM78251.1	1309142..1310752	Glycolysis/Gluconeogenesis	COG0166
ABM78251.1_1495	CUST_7169_PI442172314	MIT9303	LLIV	ABM78251.1_1495	GTGACATGGTTGTTCTTCCATATAGAGATCGCCTTGAGGTCTTTAGTCGTTACCTGCAAC	pgi	Glucose-6-phosphate isomerase		ABM78251.1	1309142..1310752	Glycolysis/Gluconeogenesis	COG0166
ABM78251.1_1495	CUST_7170_PI442172314	MIT9303	LLIV	ABM78251.1_1495	TGTTGACAATTTCTTCGCCACATTTATTGAGGTGCTTGAGGATATTGAGAACATACCGGC	pgi	Glucose-6-phosphate isomerase		ABM78251.1	1309142..1310752	Glycolysis/Gluconeogenesis	COG0166
ABM78251.1_1495	CUST_7171_PI442172314	MIT9303	LLIV	ABM78251.1_1495	ATCCATCAAGTGATTGGAGAAGGGTCAGAAGAAGCCATCTTTTGGATCATGAGGCATTTA	pgi	Glucose-6-phosphate isomerase		ABM78251.1	1309142..1310752	Glycolysis/Gluconeogenesis	COG0166
ABM78251.1_1495	CUST_7172_PI442172314	MIT9303	LLIV	ABM78251.1_1495	CTGGTAATGGAGTCGCTAGGCAAGCGTTTAGATCGAGATGGCAATGTTGTTCATCAAG	pgi	Glucose-6-phosphate isomerase		ABM78251.1	1309142..1310752	Glycolysis/Gluconeogenesis	COG0166
ABM78251.1_1495	CUST_7173_PI442172314	MIT9303	LLIV	ABM78251.1_1495	CAATAAGCGTGGTTACGTTGCAGAAGGTGATTGGGGAATTCCGACTTCACTACGTTTCAG	pgi	Glucose-6-phosphate isomerase		ABM78251.1	1309142..1310752	Glycolysis/Gluconeogenesis	COG0166
ABM78386.1_1630	CUST_7865_PI442172314	MIT9303	LLIV	ABM78386.1_1630	ACTTCATTAACAACTGGATTGATATCAAATACGAAGAGGTGCAGCAATTGCGTCAACGGC	glnA	Glutamine synthetase/ glutamate--ammonialigase		ABM78386.1	1436303..1437724	Nitrogen Metabolism	COG0174
ABM78386.1_1630	CUST_7867_PI442172314	MIT9303	LLIV	ABM78386.1_1630	CTCTTCTTTGGAGAAGGTACCTATGCCAACTTGTCACAGACTGCTCGCTGGTATATC	glnA	Glutamine synthetase/ glutamate--ammonialigase		ABM78386.1	1436303..1437724	Nitrogen Metabolism	COG0174
ABM78431.1_1675	CUST_8089_PI442172314	MIT9303	LLIV	ABM78431.1_1675	GTTTACGCACCAAGGATCCTGACGATGGAAGTGACCTTGTTGAGAAAATTTTGGATAAGG	edd	6-Phosphogluconate dehydratase		ABM78431.1	1475770..1477188	Pentose Phosphate/Entner-Dudorof	COG2084
ABM78431.1_1675	CUST_8090_PI442172314	MIT9303	LLIV	ABM78431.1_1675	CTGATGTCTTTGCGCATTGGAATGCCACAGAAGAGCTCACTTCTTATCTGGTTGAAATC	edd	6-Phosphogluconate dehydratase		ABM78431.1	1475770..1477188	Pentose Phosphate/Entner-Dudorof	COG2084
ABM78431.1_1675	CUST_8091_PI442172314	MIT9303	LLIV	ABM78431.1_1675	CAGGATGCCTTTAATGCTGACCCTCAGCTTTCCAATTTGTTGATCGATCCTTGGTTTG	edd	6-Phosphogluconate dehydratase		ABM78431.1	1475770..1477188	Pentose Phosphate/Entner-Dudorof	COG2084
ABM78431.1_1675	CUST_8092_PI442172314	MIT9303	LLIV	ABM78431.1_1675	CTGAGCAGATTTTGCATGGGTCAGCAATCAGACCATTTGATCTTGGTAGTTCCGCAGATG	edd	6-Phosphogluconate dehydratase		ABM78431.1	1475770..1477188	Pentose Phosphate/Entner-Dudorof	COG2084
ABM78431.1_1675	CUST_8093_PI442172314	MIT9303	LLIV	ABM78431.1_1675	GATTGCTTCGGCTCACATACCTACCAACGGGTTGACAAAGAAGGAACCTTCCATACTGAG	edd	6-Phosphogluconate dehydratase		ABM78431.1	1475770..1477188	Pentose Phosphate/Entner-Dudorof	COG2084
ABM78431.1_1675	CUST_8094_PI442172314	MIT9303	LLIV	ABM78431.1_1675	GTATCCCAGTGCCTTGCTTCAGCAGCACTCTCGATTACATCAATAGCTATCGCAC	edd	6-Phosphogluconate dehydratase		ABM78431.1	1475770..1477188	Pentose Phosphate/Entner-Dudorof	COG2084
ABM78601.1_1845	CUST_8927_PI442172314	MIT9303	LLIV	ABM78601.1_1845	GAAGCATTTGGAACTGATTAATGACTTTGGCGTGCTGATTTACGGCCTACCCTTTCTCAA	glk	Glucokinase		ABM78601.1	1621167..1622228	Glycolysis	COG0837
ABM78601.1_1845	CUST_8928_PI442172314	MIT9303	LLIV	ABM78601.1_1845	GATTCAATTCTTCAAGAAGCCTTACAGCTCTGGCTAGCTGCTTACGGTTCTGCTG	glk	Glucokinase		ABM78601.1	1621167..1622228	Glycolysis	COG0837
ABM78601.1_1845	CUST_8929_PI442172314	MIT9303	LLIV	ABM78601.1_1845	GAATTTGCTCCTCGCAGTGAATGTGAGTGGCAACTTTGTGAGTGGCTCAAG	glk	Glucokinase		ABM78601.1	1621167..1622228	Glycolysis	COG0837
ABM78601.1_1845	CUST_8930_PI442172314	MIT9303	LLIV	ABM78601.1_1845	GTGCTGATGATCATTCCGACCACTTGGATCTGCCTGCTCTTGCTAGTCAAG	glk	Glucokinase		ABM78601.1	1621167..1622228	Glycolysis	COG0837
ABM78601.1_1845	CUST_8931_PI442172314	MIT9303	LLIV	ABM78601.1_1845	CTTCAAGGTCTTCGCTCAAGCACCTTTCTTGAAGCCTTCCGCAACAAGG	glk	Glucokinase		ABM78601.1	1621167..1622228	Glycolysis	COG0837
ABM78601.1_1845	CUST_8932_PI442172314	MIT9303	LLIV	ABM78601.1_1845	CTTCCGTCCGTTTCTAGAGCAATTGCCAGTGATGGCAGTGATCGATCCC	glk	Glucokinase		ABM78601.1	1621167..1622228	Glycolysis	COG0837
ABM79091.1_2335	CUST_11370_PI442172314	MIT9303	LLIV	ABM79091.1_2335	CCTGCTGGATGAGCAAGGAATTGTGAGCGATCTTGTGAACAAAGAGATTGTTGAGATTTT	glsF	Ferredoxin-dependent glutamate synthase		ABM79091.1	2073971..2078554	Nitrogen Metabolism	COG0069
ABM79091.1_2335	CUST_11371_PI442172314	MIT9303	LLIV	ABM79091.1_2335	CTTGTTCCACCAAGTGAAAAGGTCAATGTTGGTTTAACTTTGCTTGAGAAAGTTGCAGCC	glsF	Ferredoxin-dependent glutamate synthase		ABM79091.1	2073971..2078554	Nitrogen Metabolism	COG0069
ABM79091.1_2335	CUST_11372_PI442172314	MIT9303	LLIV	ABM79091.1_2335	CAGTACAAAAGCCAAGGCGATTTTGGCCAACTGGATCAGTTTGAAAGCACTGTTTAAG	glsF	Ferredoxin-dependent glutamate synthase		ABM79091.1	2073971..2078554	Nitrogen Metabolism	COG0069
ABM79091.1_2335	CUST_11373_PI442172314	MIT9303	LLIV	ABM79091.1_2335	ATTTTCTCGCTAACCACTGTTGAGCAGGAAGCCATTCTCAAAACACTGCTCGAAG	glsF	Ferredoxin-dependent glutamate synthase		ABM79091.1	2073971..2078554	Nitrogen Metabolism	COG0069
ABM79091.1_2335	CUST_11374_PI442172314	MIT9303	LLIV	ABM79091.1_2335	GTGAATGTGCGCTTAGTGGGTGAGGCCAATGATTATGTCGGTAAAGGCATGAATG	glsF	Ferredoxin-dependent glutamate synthase		ABM79091.1	2073971..2078554	Nitrogen Metabolism	COG0069
ABM79091.1_2335	CUST_11375_PI442172314	MIT9303	LLIV	ABM79091.1_2335	GTAATAGCGGTGTGAAGGCTGTTGTTGAAGGTGCGGGGGATCATTGCTGTGAATACATG	glsF	Ferredoxin-dependent glutamate synthase		ABM79091.1	2073971..2078554	Nitrogen Metabolism	COG0069
ABM79270.1_2514	CUST_12212_PI442172314	MIT9303	LLIV	ABM79270.1_2514	GGGTTTGGTGGGAGTTGTTTTCAGAAAGACATCCTCAATCTGGTTTATCTCTGTCGTCAT	ugd	UDP-glucose 6-dehydrogenase/nucleotide sugar dehydrogenase		ABM79270.1	2232863..2234311	Amino sugar and nucleotide sugar r	COG1004
ABM79270.1_2514	CUST_12213_PI442172314	MIT9303	LLIV	ABM79270.1_2514	CTCAGCTGGCTATTTATGATCCGAAGGTGGATCCGCAACAGATTGCGTTTGATTTG	ugd	UDP-glucose 6-dehydrogenase/nucleotide sugar dehydrogenase		ABM79270.1	2232863..2234311	Amino sugar and nucleotide sugar r	COG1004
ABM79270.1_2514	CUST_12214_PI442172314	MIT9303	LLIV	ABM79270.1_2514	CATTTCTCGTTTGGTGGTTCAAAAGCTTTTTGGAACGGTTACAGGTAAGCGGCTG	ugd	UDP-glucose 6-dehydrogenase/nucleotide sugar dehydrogenase		ABM79270.1	2232863..2234311	Amino sugar and nucleotide sugar r	COG1004
ABM79270.1_2514	CUST_12215_PI442172314	MIT9303	LLIV	ABM79270.1_2514	CTGTTGTCGATCCAGAAGCTGTGAAATCGTCTGGTTTGATGCTTTGGCGAGTTGGAGATG	ugd	UDP-glucose 6-dehydrogenase/nucleotide sugar dehydrogenase		ABM79270.1	2232863..2234311	Amino sugar and nucleotide sugar r	COG1004
ABM79270.1_2514	CUST_12216_PI442172314	MIT9303	LLIV	ABM79270.1_2514	ATTTTGGTTTGCCGGATGTGGCGGACTATTGGGAAAACGTGGTAGCTCTCAATACTTG	ugd	UDP-glucose 6-dehydrogenase/nucleotide sugar dehydrogenase		ABM79270.1	2232863..2234311	Amino sugar and nucleotide sugar r	COG1004
ABM79270.1_2514	CUST_12217_PI442172314	MIT9303	LLIV	ABM79270.1_2514	CATGAGTTTAGACAGCTTGATTGGGTCGCTTTGGCTCCTTTGATGCGTCAAC	ugd	UDP-glucose 6-dehydrogenase/nucleotide sugar dehydrogenase		ABM79270.1	2232863..2234311	Amino sugar and nucleotide sugar r	COG1004
ABM79275.1_2519	CUST_12231_PI442172314	MIT9303	LLIV	ABM79275.1_2519	CTCAAGGCAGTTCATGAATCAGTTCAGTTACCACTGAAATACTGGGATGTGAATGTGGCT	galE	UDP-glucose 4-epimerase		ABM79275.1	2236629..2237675	Galactose metabolism	COG1087
ABM79275.1_2519	CUST_12232_PI442172314	MIT9303	LLIV	ABM79275.1_2519	CAAATCCCAATCCCTGAAACGACTAGGGTGCAACCGATCAATCCATATGGTCAAAG	galE	UDP-glucose 4-epimerase		ABM79275.1	2236629..2237675	Galactose metabolism	COG1087
ABM79275.1_2519	CUST_12233_PI442172314	MIT9303	LLIV	ABM79275.1_2519	GAAGATCCCAAGGGAACACCCAACAACCTTTTCCCTTTTGTGAGCCAAGTAGCAGTAG	galE	UDP-glucose 4-epimerase		ABM79275.1	2236629..2237675	Galactose metabolism	COG1087
ABM79275.1_2519	CUST_12234_PI442172314	MIT9303	LLIV	ABM79275.1_2519	GACAGTTGGAATTGGCAGAAGGCCAATCCACAGGGCTATAGCCAGAAACAACAATG	galE	UDP-glucose 4-epimerase		ABM79275.1	2236629..2237675	Galactose metabolism	COG1087
ABM79275.1_2519	CUST_12235_PI442172314	MIT9303	LLIV	ABM79275.1_2519	CATAACTGCCGCACGATTGTTTTCAGCAGCAGTGCAACGCTTTATGGCTATC	galE	UDP-glucose 4-epimerase		ABM79275.1	2236629..2237675	Galactose metabolism	COG1087
ABM79275.1_2519	CUST_12236_PI442172314	MIT9303	LLIV	ABM79275.1_2519	CACTGGAGGTCCTGCAACGAGAGGAACCGCAACTCCTCACTCTTAATCTC	galE	UDP-glucose 4-epimerase		ABM79275.1	2236629..2237675	Galactose metabolism	COG1087
ABM79334.1_2578	CUST_12516_PI442172314	MIT9303	LLIV	ABM79334.1_2578	ATCAACTATCCCATTGCCCTTGAGGGAGCTCTCAAACTTAAGGAGATCAGCTATATCCAT	glmS	Glutamine--fructose-6-phosphatetransaminase(isomerizing)		ABM79334.1	2297138..2299042	Amino Acid metabolism	COG0449
ABM79334.1_2578	CUST_12517_PI442172314	MIT9303	LLIV	ABM79334.1_2578	GATCAACGCTGCGAAGTTTTAGCTAACCGCTTCGCTGATACTCAGGATGTGATCTTTTTG	glmS	Glutamine--fructose-6-phosphatetransaminase(isomerizing)		ABM79334.1	2297138..2299042	Amino Acid metabolism	COG0449
ABM79334.1_2578	CUST_12518_PI442172314	MIT9303	LLIV	ABM79334.1_2578	GTCGTAGTCACCGTTCAGTGACTGAGATTGCCAACCTAAAAGACGAACTAAGGGCTTTAC	glmS	Glutamine--fructose-6-phosphatetransaminase(isomerizing)		ABM79334.1	2297138..2299042	Amino Acid metabolism	COG0449
ABM79334.1_2578	CUST_12519_PI442172314	MIT9303	LLIV	ABM79334.1_2578	CGATACAGCTACTGAGCTATCACATTGCTGCTCATAGGGGTCTTGATGTGGATCAG	glmS	Glutamine--fructose-6-phosphatetransaminase(isomerizing)		ABM79334.1	2297138..2299042	Amino Acid metabolism	COG0449
ABM79334.1_2578	CUST_12520_PI442172314	MIT9303	LLIV	ABM79334.1_2578	CAGAAAGCTCCCTGACTCGTCAGGTTCCATACATTCTCGATATCGGTGCAGGGATTGAAG	glmS	Glutamine--fructose-6-phosphatetransaminase(isomerizing)		ABM79334.1	2297138..2299042	Amino Acid metabolism	COG0449
ABM79334.1_2578	CUST_12521_PI442172314	MIT9303	LLIV	ABM79334.1_2578	CTACAAAGACCTTCTTGGGGCAGTTGTTGGCTTTCTATGGGCTTGCTTTGGCATTTG	glmS	Glutamine--fructose-6-phosphatetransaminase(isomerizing)		ABM79334.1	2297138..2299042	Amino Acid metabolism	COG0449
04.1_WP_036904462.1_	CUST_850_PI442190820	PAC1	LLI	04.1_WP_036904462.1_	TATCTATCGCTGTCCCGGGGATTGTTTATGAAAAGGTACTTAGTAACTCGCAAGAGGCTA	glmS	Glutamine--fructose-6-phosphatetransaminase(isomerizing)		WP_036904462.	50837..52741	Amino Acid metabolism	COG0449
04.1_WP_036904478.1_	CUST_907_PI442190820	PAC1	LLI	04.1_WP_036904478.1_	TAGATGTGTTGCTAGATGGAGGTATTCGCAGGGGTAGTGATGTTGTTAAAGCATTGTGTC	lldD	alpha-hydroxy-acidoxidizingprotein (L-lactate)		WP_036904478.	63749..64933	Pyruvate metabolism	COG1304
04.1_WP_036904478.1_	CUST_908_PI442190820	PAC1	LLI	04.1_WP_036904478.1_	ATGGAGGCTTAATGAGTTTTCCTAATGTTCAACTAGATGATGGCCCTATGGGTTACACGG	lldD	alpha-hydroxy-acidoxidizingprotein (L-lactate)		WP_036904478.	63749..64933	Pyruvate metabolism	COG1304
04.1_WP_036904478.1_	CUST_909_PI442190820	PAC1	LLI	04.1_WP_036904478.1_	CACCCGTATCTGGTTTGAGGGAAAGAGATATGCGATCAGGAACTCAACAGCTTTTATCAA	lldD	alpha-hydroxy-acidoxidizingprotein (L-lactate)		WP_036904478.	63749..64933	Pyruvate metabolism	COG1304
04.1_WP_036904478.1_	CUST_910_PI442190820	PAC1	LLI	04.1_WP_036904478.1_	TATTGTTAAGGGTATACATATTGGTGATGACGCAAAAAAAGCGGTAGAGCTAGGGGCTGA	lldD	alpha-hydroxy-acidoxidizingprotein (L-lactate)		WP_036904478.	63749..64933	Pyruvate metabolism	COG1304
04.1_WP_036904478.1_	CUST_911_PI442190820	PAC1	LLI	04.1_WP_036904478.1_	GCAGGAGGGAAAGGCGTTGCCAGAGCTATAGAAATTCTTCAAACAGATATAGTGAGGAC	lldD	alpha-hydroxy-acidoxidizingprotein (L-lactate)		WP_036904478.	63749..64933	Pyruvate metabolism	COG1304
04.1_WP_036904478.1_	CUST_912_PI442190820	PAC1	LLI	04.1_WP_036904478.1_	AGAAGTCGCGTTAAAAACAATTGCTAGAGCTAAAGAAGCTGGATTCTCAGCAATAGTTGT	lldD	alpha-hydroxy-acidoxidizingprotein (L-lactate)		WP_036904478.	63749..64933	Pyruvate metabolism	COG1304
04.1_WP_036904517.1_	CUST_1095_PI442190820	PAC1	LLI	04.1_WP_036904517.1_	CTTTAGCTACTGAATTAAAGGCTGACCCCGACCAAATGGAGATGTTGCGAATGTTGAATC	ppc	Phosphoenolpyruvate carboxylase		WP_036904517.	94951..97935	Krebs cycle and related genes	COG2352
04.1_WP_036904517.1_	CUST_1096_PI442190820	PAC1	LLI	04.1_WP_036904517.1_	TCAAAACTATTGAGTGCAGATCCTGCTTTGCAGTTGTCTGTCAACCTGAGAAATAGGACT	ppc	Phosphoenolpyruvate carboxylase		WP_036904517.	94951..97935	Krebs cycle and related genes	COG2352
04.1_WP_036904517.1_	CUST_1097_PI442190820	PAC1	LLI	04.1_WP_036904517.1_	CAGCTTGGTTACCAATAAATTGGATGCTACGCCAAGTTGGAATGAATTGATGACCAGACT	ppc	Phosphoenolpyruvate carboxylase		WP_036904517.	94951..97935	Krebs cycle and related genes	COG2352
04.1_WP_036904517.1_	CUST_1098_PI442190820	PAC1	LLI	04.1_WP_036904517.1_	AATTATTGCGTGGTGCATTGTTGACTATCAATGGTATCGCTGCAGGTATGAGAAACACAG	ppc	Phosphoenolpyruvate carboxylase		WP_036904517.	94951..97935	Krebs cycle and related genes	COG2352
04.1_WP_036904517.1_	CUST_1099_PI442190820	PAC1	LLI	04.1_WP_036904517.1_	AAAGACTTATCAAGTCTTCGAGCTATCCCATGGGTCTTTGGTTGGACTCAAAGTCGTTTC	ppc	Phosphoenolpyruvate carboxylase		WP_036904517.	94951..97935	Krebs cycle and related genes	COG2352
04.1_WP_036904517.1_	CUST_1100_PI442190820	PAC1	LLI	04.1_WP_036904517.1_	TTGGGATTTTTACAAGTTGCTCTCCTCAAGCGATTAAGAGATCAGAATCGTCAACCACCT	ppc	Phosphoenolpyruvate carboxylase		WP_036904517.	94951..97935	Krebs cycle and related genes	COG2352
05.1_WP_036904623.1_	CUST_1458_PI442190820	PAC1	LLI	05.1_WP_036904623.1_	CAATTGAACAGGCCGGATTTAAACCAGGAGAGCAAGTCTCTCTAGCTTTAGATGTTGCTA	eno	Enolase		WP_036904623.	16007..17308	Glycolysis/Gluconeogenesis	COG0148
05.1_WP_036904623.1_	CUST_1459_PI442190820	PAC1	LLI	05.1_WP_036904623.1_	AAGGTTTATCAACTGCTGTTGGTGATGAAGGGGGATTCGCTCCAAATCTTGAGAGTAATA	eno	Enolase		WP_036904623.	16007..17308	Glycolysis/Gluconeogenesis	COG0148
05.1_WP_036904623.1_	CUST_1460_PI442190820	PAC1	LLI	05.1_WP_036904623.1_	TTCCAATTGTTTCTATTGAAGATGGATTAGCCGAAGATGATTGGGATGGTTGGAGCTTGC	eno	Enolase		WP_036904623.	16007..17308	Glycolysis/Gluconeogenesis	COG0148
05.1_WP_036904623.1_	CUST_1461_PI442190820	PAC1	LLI	05.1_WP_036904623.1_	TACGGAGTTTTATGAGGAGGGGCAATATTGTTATGGTGGAAATTCTTATTCCAGCGAGCA	eno	Enolase		WP_036904623.	16007..17308	Glycolysis/Gluconeogenesis	COG0148
05.1_WP_036904623.1_	CUST_1462_PI442190820	PAC1	LLI	05.1_WP_036904623.1_	TACTTCGTATTGAGGATGAGTTAGGCAGTCAAGCAACTTATGCTGGACTTGCTTGGATTGG	eno	Enolase		WP_036904623.	16007..17308	Glycolysis/Gluconeogenesis	COG0148
05.1_WP_036904623.1_	CUST_1463_PI442190820	PAC1	LLI	05.1_WP_036904623.1_	AGCAACTAAATCTGGTCAAATCAAAACAGGTTCATTGAGTCGTAGTGAAAGAGTCGCGAA	eno	Enolase		WP_036904623.	16007..17308	Glycolysis/Gluconeogenesis	COG0148
10.1_WP_036905297.1_	CUST_3175_PI442190820	PAC1	LLI	10.1_WP_036905297.1_	CCTTTATTGGTGAAGGTATTCCAGTTGCCCTTGGAGCAGCTTTCAGCAGTAAATACAAAA	pdhA	Pyruvate dehydrogenase E1 component alpha subunit		WP_036905297.	61822..62910	Glycolysis/Krebs cycle	COG1071
10.1_WP_036905297.1_	CUST_3176_PI442190820	PAC1	LLI	10.1_WP_036905297.1_	AAGAAATTGATCTAGAAGTTAACGATGCTGTTGAATTTGCCTTAAACGCCCCAGAGCCTG	pdhA	Pyruvate dehydrogenase E1 component alpha subunit		WP_036905297.	61822..62910	Glycolysis/Krebs cycle	COG1071
10.1_WP_036905297.1_	CUST_3177_PI442190820	PAC1	LLI	10.1_WP_036905297.1_	GCTGCAGTAAAGGCAGAGGTGGATCTATGCATCTGTTCTCTAAAGAACATCACCTACTTG	pdhA	Pyruvate dehydrogenase E1 component alpha subunit		WP_036905297.	61822..62910	Glycolysis/Krebs cycle	COG1071
10.1_WP_036905297.1_	CUST_3178_PI442190820	PAC1	LLI	10.1_WP_036905297.1_	CCGATCATATTTGTAGTCGAAAATAATAAATGGGCAATTGGGATGGCCCATGACAGAGCA	pdhA	Pyruvate dehydrogenase E1 component alpha subunit		WP_036905297.	61822..62910	Glycolysis/Krebs cycle	COG1071
10.1_WP_036905297.1_	CUST_3179_PI442190820	PAC1	LLI	10.1_WP_036905297.1_	GAAGGCCCTACTTTGATAGAATGTCTCACTTACAGATTCAGGGGGCATTCATTAGCTGAC	pdhA	Pyruvate dehydrogenase E1 component alpha subunit		WP_036905297.	61822..62910	Glycolysis/Krebs cycle	COG1071
10.1_WP_036905297.1_	CUST_3180_PI442190820	PAC1	LLI	10.1_WP_036905297.1_	CTAGTGAGACAGAAATATGGAGAAAAGCTTCAGCGTTTGGCATGCACGGAGAAGAGATTG	pdhA	Pyruvate dehydrogenase E1 component alpha subunit		WP_036905297.	61822..62910	Glycolysis/Krebs cycle	COG1071
10.1_WP_052038648.1_	CUST_3292_PI442190820	PAC1	LLI	10.1_WP_052038648.1_	ATCGTTGGTACAGTTGTTGACGAAAAACTTGAGGGAGAGATTCAGGTAACCGTTATTGCT	ftsZ	cell division protein FtsZ		WP_052038648.	84298..85401	Cell Division	COG0206
10.1_WP_052038648.1_	CUST_3293_PI442190820	PAC1	LLI	10.1_WP_052038648.1_	GAATGTAGATTTTGCTGATGTGCGTTCTGTTATGACTGAAGCTGGTACTGCGCTTTTAGG	ftsZ	cell division protein FtsZ		WP_052038648.	84298..85401	Cell Division	COG0206
10.1_WP_052038648.1_	CUST_3294_PI442190820	PAC1	LLI	10.1_WP_052038648.1_	AGCGATCAATAGTCCTCTTTTAGAAGCAGCTCGAATAGATGGTGCAAAAGGATGCGTAAT	ftsZ	cell division protein FtsZ		WP_052038648.	84298..85401	Cell Division	COG0206
10.1_WP_052038648.1_	CUST_3295_PI442190820	PAC1	LLI	10.1_WP_052038648.1_	TCGTTTAAGACAAGCTGACGAAGGTATCGCAAGGCTTGCAGAGAATGTTGATACCTTAAT	ftsZ	cell division protein FtsZ		WP_052038648.	84298..85401	Cell Division	COG0206
10.1_WP_052038648.1_	CUST_3296_PI442190820	PAC1	LLI	10.1_WP_052038648.1_	GATTAAAAGACGTAATTTCAGGAGCCCCTTTGCAAGAAGCCTTCAGAAGTGCTGATGATG	ftsZ	cell division protein FtsZ		WP_052038648.	84298..85401	Cell Division	COG0206
10.1_WP_052038648.1_	CUST_3297_PI442190820	PAC1	LLI	10.1_WP_052038648.1_	AATAGAGCAAGACTTTCACCTAAATCACTTTACGAACAACCAGAAGAGAGGGAAGCAGGG	ftsZ	cell division protein FtsZ		WP_052038648.	84298..85401	Cell Division	COG0206
10.1_WP_036905408.1_	CUST_3502_PI442190820	PAC1	LLI	10.1_WP_036905408.1_	GAGGTGAAATGGCAAGAGCAATAAATGTTTTTAAGATGCGCGGATCTTGGCATGACAAAA	kaiC	circadian clock protein KaiC		WP_036905408.	120513..122015	Circadian Rhythm	COG0467
10.1_WP_036905408.1_	CUST_3503_PI442190820	PAC1	LLI	10.1_WP_036905408.1_	GAGCACAACTTCTTCGAAATGCAACTAGCTGGGGGATTGATTTTGAAAAAATGGAAGCTG	kaiC	circadian clock protein KaiC		WP_036905408.	120513..122015	Circadian Rhythm	COG0467
10.1_WP_036905408.1_	CUST_3504_PI442190820	PAC1	LLI	10.1_WP_036905408.1_	TTAGTGGTGCCCCTCATCGTATTAATCCTGAAGAACAGATACCTGGGGTTTTTAAAAGCA	kaiC	circadian clock protein KaiC		WP_036905408.	120513..122015	Circadian Rhythm	COG0467
10.1_WP_036905408.1_	CUST_3505_PI442190820	PAC1	LLI	10.1_WP_036905408.1_	ATATAAACCATCCAGAATGGCGATAGATTCTCTATCCGCACTGGCTAGAGGTGTAAGCTT	kaiC	circadian clock protein KaiC		WP_036905408.	120513..122015	Circadian Rhythm	COG0467
10.1_WP_036905408.1_	CUST_3506_PI442190820	PAC1	LLI	10.1_WP_036905408.1_	TGTGTGGTGGAGGATATTTTCAAGATTCAATTATTCTTGCAACTGGTGCAACTGGTACCG	kaiC	circadian clock protein KaiC		WP_036905408.	120513..122015	Circadian Rhythm	COG0467
10.1_WP_036905408.1_	CUST_3507_PI442190820	PAC1	LLI	10.1_WP_036905408.1_	ACTGCAGAAGAATTCATGGGAAGCCACTCAATCACTGATTCCCATATTTCAACGATTACT	kaiC	circadian clock protein KaiC		WP_036905408.	120513..122015	Circadian Rhythm	COG0467
10.1_WP_036905412.1_	CUST_3508_PI442190820	PAC1	LLI	10.1_WP_036905412.1_	TGGACCTTTTGTATGATGAATTATCAGATAAAGAAATGTTTTACTCTTCAGA	kaiB	Circadian clock protein KaiB		WP_036905412.	122146..122469	Circadian Rhythm	COG4251
10.1_WP_036905412.1_	CUST_3509_PI442190820	PAC1	LLI	10.1_WP_036905412.1_	GTTCGAAGAATAATTGGTGACCTATCAGATAGAGAAAAGGTGTTAATTGGATTGGACCTT	kaiB	Circadian clock protein KaiB		WP_036905412.	122146..122469	Circadian Rhythm	COG4251
10.1_WP_036905412.1_	CUST_3510_PI442190820	PAC1	LLI	10.1_WP_036905412.1_	TTAGCCGAAGAAGATAAAATACTTGCCACACCAACCTTGTCTAAGATTTTACCCCCACCT	kaiB	Circadian clock protein KaiB		WP_036905412.	122146..122469	Circadian Rhythm	COG4251
10.1_WP_036905412.1_	CUST_3511_PI442190820	PAC1	LLI	10.1_WP_036905412.1_	GAAAGTGAATTCAAAGGTGTCTATGCTCTAAAGGTTATTGATGTTTTAAAAAGCCCT	kaiB	Circadian clock protein KaiB		WP_036905412.	122146..122469	Circadian Rhythm	COG4251
10.1_WP_036905412.1_	CUST_3512_PI442190820	PAC1	LLI	10.1_WP_036905412.1_	GTGGCAGGGAATACACCAAACTCTATGAGGGCTTTGAACACGTTGAGAGAAATCTTAGAA	kaiB	Circadian clock protein KaiB		WP_036905412.	122146..122469	Circadian Rhythm	COG4251
10.1_WP_036905527.1_	CUST_3751_PI442190820	PAC1	LLI	10.1_WP_036905527.1_	AGGGGAATTCTCCTGAAAAAATCAAAGCACAAGAAACATTGAACAAAGCAAAAGCTTGGT	atpC	ATP synthase epsilon subunit		WP_036905527.	161459..161866	Energy Metabolism	COG0355
10.1_WP_036905527.1_	CUST_3752_PI442190820	PAC1	LLI	10.1_WP_036905527.1_	AAAGCTGAAATGGGGAAAAATATTGACTCTGGCAAAGCTGAAGCTGAATTAGAACAAGCT	atpC	ATP synthase epsilon subunit		WP_036905527.	161459..161866	Energy Metabolism	COG0355
10.1_WP_036905527.1_	CUST_3753_PI442190820	PAC1	LLI	10.1_WP_036905527.1_	ATTGGGACTCAATAGCCCTAACTGGAGGTTTTGCAGAAGTTGAATCTAACGAAGTTACCG	atpC	ATP synthase epsilon subunit		WP_036905527.	161459..161866	Energy Metabolism	COG0355
10.1_WP_036905527.1_	CUST_3754_PI442190820	PAC1	LLI	10.1_WP_036905527.1_	ACCTGGTCACATTTCAATGGTTACTGCTATTGATTTTGGTGTATTGAGGGTT	atpC	ATP synthase epsilon subunit		WP_036905527.	161459..161866	Energy Metabolism	COG0355
10.1_WP_036905527.1_	CUST_3755_PI442190820	PAC1	LLI	10.1_WP_036905527.1_	ATACAGCTGATGAAATAATACTTCCAAGCACTACAGGGCTTTTGGGGGTATTACCTGGTC	atpC	ATP synthase epsilon subunit		WP_036905527.	161459..161866	Energy Metabolism	COG0355
10.1_WP_036905549.1_	CUST_3807_PI442190820	PAC1	LLI	10.1_WP_036905549.1_	GGAGATGTTTTTTATTGCCATAGTCGCTTACTTGAGAGGGCTGCAAAACTTTCTGATGCT	atpA	ATP synthase alpha subunit		WP_036905549.	170997..172511	Energy Metabolism	COG0056
10.1_WP_036905549.1_	CUST_3808_PI442190820	PAC1	LLI	10.1_WP_036905549.1_	GAGCTGCGCAAACAAAAGCTATTAAGAAAATTGCCGGTACGTTAAAACTTGAACTGGCTC	atpA	ATP synthase alpha subunit		WP_036905549.	170997..172511	Energy Metabolism	COG0056
10.1_WP_036905549.1_	CUST_3809_PI442190820	PAC1	LLI	10.1_WP_036905549.1_	CACTTGTTATTTATGACGACTTAACCAAGCAAGCTCAGGCTTACAGGCAGATGTCTTTGC	atpA	ATP synthase alpha subunit		WP_036905549.	170997..172511	Energy Metabolism	COG0056
10.1_WP_036905549.1_	CUST_3810_PI442190820	PAC1	LLI	10.1_WP_036905549.1_	TGAACTTGCGGCATTCTCTCAATTTGCTTCAGATCTTGATGAGGCTACTCAAAAGCAATT	atpA	ATP synthase alpha subunit		WP_036905549.	170997..172511	Energy Metabolism	COG0056
10.1_WP_036905549.1_	CUST_3811_PI442190820	PAC1	LLI	10.1_WP_036905549.1_	AGTGAAGCATCTGAATCAATGCTTAAAGACGCTATTAGCGAGGTTAAGTCCTCCATGCTT	atpA	ATP synthase alpha subunit		WP_036905549.	170997..172511	Energy Metabolism	COG0056
10.1_WP_036905549.1_	CUST_3812_PI442190820	PAC1	LLI	10.1_WP_036905549.1_	AGTTAAAGGATTGATTGATGAGGTTCCCGAGGAGGAAGTGGTTAAGTTTGCTCGTGAATT	atpA	ATP synthase alpha subunit		WP_036905549.	170997..172511	Energy Metabolism	COG0056
10.1_WP_036905574.1_	CUST_3894_PI442190820	PAC1	LLI	10.1_WP_036905574.1_	CAGCCAATGAACCTGGTAGCTCAATAATGCCAGGGAAAATTAATCCTACTCAATGCGAAG	fumC	class II fumarate hydratase		WP_036905574.	183366..184760	Krebs cycle and related genes	COG0114
10.1_WP_036905574.1_	CUST_3895_PI442190820	PAC1	LLI	10.1_WP_036905574.1_	ATCTCTGCTTGGGCTGCTCAATTAGAGACTGCTTTAAAACGCATTGAAATCAACATCGAG	fumC	class II fumarate hydratase		WP_036905574.	183366..184760	Krebs cycle and related genes	COG0114
10.1_WP_036905574.1_	CUST_3896_PI442190820	PAC1	LLI	10.1_WP_036905574.1_	GATCGCAGGAAGTGGGGGACATCTACAAATGAATGTCTATAAACCACTGATTGGCTACAA	fumC	class II fumarate hydratase		WP_036905574.	183366..184760	Krebs cycle and related genes	COG0114
10.1_WP_036905574.1_	CUST_3897_PI442190820	PAC1	LLI	10.1_WP_036905574.1_	ACTTTATCCACTTCCACTTGGAGGAACCGCTATTGGTACAGGGATTAATACTCCTAAGGA	fumC	class II fumarate hydratase		WP_036905574.	183366..184760	Krebs cycle and related genes	COG0114
10.1_WP_036905574.1_	CUST_3898_PI442190820	PAC1	LLI	10.1_WP_036905574.1_	AACCGCCTTATCACCATCAATAGGATACGAAAAAGCAAGTAAAATTGCTCAACTAGCCCA	fumC	class II fumarate hydratase		WP_036905574.	183366..184760	Krebs cycle and related genes	COG0114
10.1_WP_036905574.1_	CUST_3899_PI442190820	PAC1	LLI	10.1_WP_036905574.1_	GCCGTCACTTTTCTCAAAATTGTTAATGATCTTCGACTTTTATCTTGTGGGCCAAGAGCT	fumC	class II fumarate hydratase		WP_036905574.	183366..184760	Krebs cycle and related genes	COG0114
10.1_WP_036905728.1_	CUST_4333_PI442190820	PAC1	LLI	10.1_WP_036905728.1_	GAGGAGTAGTTGTTGTGCTTGGGAAAACTGGAAGAAATATAGGAGCAGGAATGACTGGAG	glsF	Ferredoxin-dependent glutamate synthase		WP_036905728.	265272..269861	Nitrogen Metabolism	COG0069
10.1_WP_036905728.1_	CUST_4334_PI442190820	PAC1	LLI	10.1_WP_036905728.1_	CATCTAACACTCAGGTGATACTTGGCAACACATGTCTATATGGAGCAACTGGTGGGAAAT	glsF	Ferredoxin-dependent glutamate synthase		WP_036905728.	265272..269861	Nitrogen Metabolism	COG0069
10.1_WP_036905728.1_	CUST_4335_PI442190820	PAC1	LLI	10.1_WP_036905728.1_	AGATTCTCGCAGACTGGTCTTCATGGAAGGAATTATTTAAAGCAGTAGTTCCTCCAAGCG	glsF	Ferredoxin-dependent glutamate synthase		WP_036905728.	265272..269861	Nitrogen Metabolism	COG0069
10.1_WP_036905728.1_	CUST_4336_PI442190820	PAC1	LLI	10.1_WP_036905728.1_	CCTACTCAAAGATGAAGAGGTTGCTAATGCACTCCAAACTCAGGGAACCATTACGAAAGA	glsF	Ferredoxin-dependent glutamate synthase		WP_036905728.	265272..269861	Nitrogen Metabolism	COG0069
10.1_WP_036905728.1_	CUST_4337_PI442190820	PAC1	LLI	10.1_WP_036905728.1_	CAATAGTGGAGCTCATGCTGTTATTGAAGGAGCTGGAGACCATTGTTGCGAATACATGAC	glsF	Ferredoxin-dependent glutamate synthase		WP_036905728.	265272..269861	Nitrogen Metabolism	COG0069
10.1_WP_036905728.1_	CUST_4338_PI442190820	PAC1	LLI	10.1_WP_036905728.1_	GGTTTTAATGGGAATTTAAATTTGATATTTAAAGGTTCAGCGGGGCAAAGCTTTGGTGCA	glsF	Ferredoxin-dependent glutamate synthase		WP_036905728.	265272..269861	Nitrogen Metabolism	COG0069
10.1_WP_011295427.1_	CUST_4361_PI442190820	PAC1	LLI	10.1_WP_011295427.1_	CCATGCCAGGTAATCACTACTGGATGGACATGATCAATAGACCAGATGCACTAACCAATT	psaB	Photosystem I P700 chlorophyll A apoprotein A2 PsaB		WP_011295427.	275940..278168	Photosystem I	COG2885
10.1_WP_011295427.1_	CUST_4362_PI442190820	PAC1	LLI	10.1_WP_011295427.1_	TGTAAATGCTTTATCTCCATGGGCTTGGATGTTCTTATTTGGTCATCTCATTTGGGCCAC	psaB	Photosystem I P700 chlorophyll A apoprotein A2 PsaB		WP_011295427.	275940..278168	Photosystem I	COG2885
10.1_WP_011295427.1_	CUST_4363_PI442190820	PAC1	LLI	10.1_WP_011295427.1_	CCAGGTCGTGGTGGTACTTGTGATAGTTCTTCTTGGGACGCTACTTATTTAGCAATGTTC	psaB	Photosystem I P700 chlorophyll A apoprotein A2 PsaB		WP_011295427.	275940..278168	Photosystem I	COG2885
10.1_WP_011295427.1_	CUST_4364_PI442190820	PAC1	LLI	10.1_WP_011295427.1_	TGGCATTGGAAACATCTAGCAATTTGGATGGGTAATACCGCTCAATTCAATGAATCTGGT	psaB	Photosystem I P700 chlorophyll A apoprotein A2 PsaB		WP_011295427.	275940..278168	Photosystem I	COG2885
10.1_WP_011295427.1_	CUST_4365_PI442190820	PAC1	LLI	10.1_WP_011295427.1_	TATTGGACCTGCAGATTTCTTAGTTCACCATGCGATTGCACTAGGACTACATACAACTGC	psaB	Photosystem I P700 chlorophyll A apoprotein A2 PsaB		WP_011295427.	275940..278168	Photosystem I	COG2885
10.1_WP_011295427.1_	CUST_4366_PI442190820	PAC1	LLI	10.1_WP_011295427.1_	GTTCCTCATCTCTTGGAGAGGATATTGGCAAGAACTTATCGAAACTCTTGTTTGGGCTCA	psaB	Photosystem I P700 chlorophyll A apoprotein A2 PsaB		WP_011295427.	275940..278168	Photosystem I	COG2885
11.1_WP_036906074.1_	CUST_5053_PI442190820	PAC1	LLI	11.1_WP_036906074.1_	CCGACCGATGTGCTACTGGAAGTAGTCTTATGCCTCAAAAAAGAATCCTGATGTACCAG	argH	Argininosuccinate lyase		WP_036906074.	127034..128425	Amino Acid metabolism	COG0165
11.1_WP_036906074.1_	CUST_5054_PI442190820	PAC1	LLI	11.1_WP_036906074.1_	CCTTTTCGAGAGGCTTATCAATTGGTTGGACGAATTGTAAAAACTTCTTTGGAGGAGGGG	argH	Argininosuccinate lyase		WP_036906074.	127034..128425	Amino Acid metabolism	COG0165
11.1_WP_036906074.1_	CUST_5055_PI442190820	PAC1	LLI	11.1_WP_036906074.1_	AGCTTGTTCGAGGCAAGTCAGGAAGAGTATTTGGACATTTACAAGCTATGCTGACTATGA	argH	Argininosuccinate lyase		WP_036906074.	127034..128425	Amino Acid metabolism	COG0165
11.1_WP_036906074.1_	CUST_5056_PI442190820	PAC1	LLI	11.1_WP_036906074.1_	GCTGGTGGAACTGGATTTGAGAGAGTTCAAGAACAGCTTCTTTCTTGGCGAGAAAAATTA	argH	Argininosuccinate lyase		WP_036906074.	127034..128425	Amino Acid metabolism	COG0165
11.1_WP_036906074.1_	CUST_5057_PI442190820	PAC1	LLI	11.1_WP_036906074.1_	AGCTGTTTCCTCAGATTTTTCAAATGCTACTGATGTTGCTGATTATTTAGTGGCTAAGGA	argH	Argininosuccinate lyase		WP_036906074.	127034..128425	Amino Acid metabolism	COG0165
11.1_WP_036906074.1_	CUST_5058_PI442190820	PAC1	LLI	11.1_WP_036906074.1_	TGCTGTAAGTGATAGAGACTTTGTCGTAGAATTTTTAGGAGCCTCATCGTTAATTATGGC	argH	Argininosuccinate lyase		WP_036906074.	127034..128425	Amino Acid metabolism	COG0165
12.1_WP_036906169.1_	CUST_5358_PI442190820	PAC1	LLI	12.1_WP_036906169.1_	CCTAAAAAGTGGATATGATGCTTTGGTCATCGGAAGAGAATTGCGAAATCAAACACCTGA	eda	2-dehydro-3-deoxyphosphogluconate aldolase		WP_036906169.	1256..1891	Entner-Dudoroff	COG0800
12.1_WP_036906169.1_	CUST_5359_PI442190820	PAC1	LLI	12.1_WP_036906169.1_	ATTGTCTAATTTCGGTATAAAAAATATTGAAATAGGCTGGGATCCCAATCCAGAATGGGT	eda	2-dehydro-3-deoxyphosphogluconate aldolase		WP_036906169.	1256..1891	Entner-Dudoroff	COG0800
12.1_WP_036906169.1_	CUST_5360_PI442190820	PAC1	LLI	12.1_WP_036906169.1_	ATTGGTGTTGCATCAATTTCTTCAAGGCAATCTTTAGAATCAATTCTCTCATTAGATGTT	eda	2-dehydro-3-deoxyphosphogluconate aldolase		WP_036906169.	1256..1891	Entner-Dudoroff	COG0800
12.1_WP_036906169.1_	CUST_5361_PI442190820	PAC1	LLI	12.1_WP_036906169.1_	AAGAGGCAATCGATCTAGGATATAAAATTGTAAAAATCTTTCCAGCATCAAAATTAGGAA	eda	2-dehydro-3-deoxyphosphogluconate aldolase		WP_036906169.	1256..1891	Entner-Dudoroff	COG0800
12.1_WP_036906169.1_	CUST_5362_PI442190820	PAC1	LLI	12.1_WP_036906169.1_	TGGAATCTCAGCAAAATAATTTAATAAAATCTCTTAGGATACAGCCGCTTATAATTGTAA	eda	2-dehydro-3-deoxyphosphogluconate aldolase		WP_036906169.	1256..1891	Entner-Dudoroff	COG0800
12.1_WP_036906169.1_	CUST_5363_PI442190820	PAC1	LLI	12.1_WP_036906169.1_	TACAAGCCATTGAATACAATCAATTAGTTATTCCAGGGATATCAAATATTGAAAATTTCA	eda	2-dehydro-3-deoxyphosphogluconate aldolase		WP_036906169.	1256..1891	Entner-Dudoroff	COG0800
12.1_WP_036906351.1_	CUST_5954_PI442190820	PAC1	LLI	12.1_WP_036906351.1_	CCAACTTAAAGAGCCTAAGAATCAGTCAGAAGTAGATTCTCCAGAAACTGAAGGCACAGA	minE	septum site-determining protein MinE		WP_036906351.	104603..104944	Cell Division	COG0851
12.1_WP_036906351.1_	CUST_5955_PI442190820	PAC1	LLI	12.1_WP_036906351.1_	GAGACAGAGGATCGCATGACCGCATTAGTTGCGAACCTACCAATAAAAGAACTATGACT	minE	septum site-determining protein MinE		WP_036906351.	104603..104944	Cell Division	COG0851
12.1_WP_036906351.1_	CUST_5956_PI442190820	PAC1	LLI	12.1_WP_036906351.1_	ATACTTGAGGTGGTCGCAAAGTATGTAGAGATAGATGTGGATGAAGGAGCAGTAAGTCTT	minE	septum site-determining protein MinE		WP_036906351.	104603..104944	Cell Division	COG0851
12.1_WP_036906351.1_	CUST_5957_PI442190820	PAC1	LLI	12.1_WP_036906351.1_	GCGAGAGAGAGGTTGCAACTTGTTCTAGCTCATGATCGATCAGATCTCAGTACAGAACTT	minE	septum site-determining protein MinE		WP_036906351.	104603..104944	Cell Division	COG0851
12.1_WP_036906351.1_	CUST_5958_PI442190820	PAC1	LLI	12.1_WP_036906351.1_	ATTACACTTAGAGACATTATCAACAAGTTACTACGTAGACAGCCCGCTAGTGCTAGTACC	minE	septum site-determining protein MinE		WP_036906351.	104603..104944	Cell Division	COG0851
12.1_WP_036906315.1_	CUST_5959_PI442190820	PAC1	LLI	12.1_WP_036906315.1_	CCAATCTATCTTTACTTCCTGCGGGGAATCCAAGAATGCTTGATTGGTTAAAACCCGATG	minD	septum site-determining protein MinD		WP_036906315.	104955..105770	Cell Division	COG2894
12.1_WP_036906315.1_	CUST_5960_PI442190820	PAC1	LLI	12.1_WP_036906315.1_	TTACGTTTTAATTGACTGCCCTGCAGGAGTTGAAGATGGATTTAAGAATGCTATGGCAGC	minD	septum site-determining protein MinD		WP_036906315.	104955..105770	Cell Division	COG2894
12.1_WP_036906315.1_	CUST_5961_PI442190820	PAC1	LLI	12.1_WP_036906315.1_	TACCAACCCTGAGGTATCAGCAGTAAGGGATGCAGACAGAGTAATTGGTCTACTTAACAC	minD	septum site-determining protein MinD		WP_036906315.	104955..105770	Cell Division	COG2894
12.1_WP_036906315.1_	CUST_5962_PI442190820	PAC1	LLI	12.1_WP_036906315.1_	AGTACAAACCGAGGAGAGCCACTAACTTTAAATAGCGTTAATTCCCCAGCAGCTAAATGT	minD	septum site-determining protein MinD		WP_036906315.	104955..105770	Cell Division	COG2894
12.1_WP_036906315.1_	CUST_5963_PI442190820	PAC1	LLI	12.1_WP_036906315.1_	ATCGAATTGTATATACGGCTCAAGAAGTTCTAGAGGAGGAATGCAGACTTGATCAGGCCT	minD	septum site-determining protein MinD		WP_036906315.	104955..105770	Cell Division	COG2894
12.1_WP_036906315.1_	CUST_5964_PI442190820	PAC1	LLI	12.1_WP_036906315.1_	TAGAGTTAGGCCAAAGATGATGGCCAACCAAGAAATGCTTTCTATCGATGATGTGACTGA	minD	septum site-determining protein MinD		WP_036906315.	104955..105770	Cell Division	COG2894
12.1_WP_036906316.1_	CUST_5965_PI442190820	PAC1	LLI	12.1_WP_036906316.1_	GGCAGCAAGGGAAACTCTAAAGCAAAAATATCCGCACTTCAACTTAGTCCTGTTCAACTT	minC	septum site-determining protein MinC		WP_036906316.	105933..106577	Cell Division	COG0850
12.1_WP_036906316.1_	CUST_5966_PI442190820	PAC1	LLI	12.1_WP_036906316.1_	GGAGAATATCTTGAAAGTCCTGGGGATTTGTTAATCCTTGGAGATGTTAATCCAGGGGCA	minC	septum site-determining protein MinC		WP_036906316.	105933..106577	Cell Division	COG0850
12.1_WP_036906316.1_	CUST_5967_PI442190820	PAC1	LLI	12.1_WP_036906316.1_	GGTCCTAAGGAGAAGCCTCAATTTGGTCTTGCAGAACAAGCAAGAATCGATTCTGAAGAA	minC	septum site-determining protein MinC		WP_036906316.	105933..106577	Cell Division	COG0850
12.1_WP_036906316.1_	CUST_5968_PI442190820	PAC1	LLI	12.1_WP_036906316.1_	AATGTTAGCGCAGAGGGAAATATAATTATTTGGGGAAGATTACTTGGGATTGCTCATGCA	minC	septum site-determining protein MinC		WP_036906316.	105933..106577	Cell Division	COG0850
12.1_WP_036906316.1_	CUST_5969_PI442190820	PAC1	LLI	12.1_WP_036906316.1_	TGCCCCAGAGATAAACAAGAAAAATCCAACTTTTTCAAAAACTCACTTTCATCAAGGAAC	minC	septum site-determining protein MinC		WP_036906316.	105933..106577	Cell Division	COG0850
12.1_WP_036906316.1_	CUST_5970_PI442190820	PAC1	LLI	12.1_WP_036906316.1_	AGATTATTTGTTTTTGCTCATCATCTCCAAAGACGATTGTAAGTTCTCAATCACTAGGTT	minC	septum site-determining protein MinC		WP_036906316.	105933..106577	Cell Division	COG0850
14.1_WP_036906602.1_	CUST_6618_PI442190820	PAC1	LLI	14.1_WP_036906602.1_	ATCAGATGAAGGTTATGTAAGACTCCTTGCCGATGCAATGCTTGGAGATCCTACATTGTT	zwf	glucose-6-phosphate dehydrogenase zwf		WP_036906602.	74116..75639	Pentose Phosphate	COG0364
14.1_WP_036906602.1_	CUST_6619_PI442190820	PAC1	LLI	14.1_WP_036906602.1_	CTAGGTTACAGAGATGAACCAGGTGTTGATCCAAACAGCACAACCGAAACATATGTAGCT	zwf	glucose-6-phosphate dehydrogenase zwf		WP_036906602.	74116..75639	Pentose Phosphate	COG0364
14.1_WP_036906602.1_	CUST_6620_PI442190820	PAC1	LLI	14.1_WP_036906602.1_	ACCTGGGCATTTTGATCCAGAAGCCATAAGAAATGAAAAAGCTAAAGTTCTTCAGGCAGT	zwf	glucose-6-phosphate dehydrogenase zwf		WP_036906602.	74116..75639	Pentose Phosphate	COG0364
14.1_WP_036906602.1_	CUST_6621_PI442190820	PAC1	LLI	14.1_WP_036906602.1_	TCCCCAGGGTCTGGAATGAGAAGTAGACCTGTAAATATGGAGTTTTCCTATGACGAATCC	zwf	glucose-6-phosphate dehydrogenase zwf		WP_036906602.	74116..75639	Pentose Phosphate	COG0364
14.1_WP_036906602.1_	CUST_6622_PI442190820	PAC1	LLI	14.1_WP_036906602.1_	TTCTCCTTGGGAATTACCTATTTATCCATATGAATCAAGAACATGGGGGCCGACCGAATC	zwf	glucose-6-phosphate dehydrogenase zwf		WP_036906602.	74116..75639	Pentose Phosphate	COG0364
14.1_WP_036906602.1_	CUST_6623_PI442190820	PAC1	LLI	14.1_WP_036906602.1_	TGCCCAACTTCAAACCAATTGATTCTTAGAATTCAACCCAACGAAGGGGCTGAATTTAGT	zwf	glucose-6-phosphate dehydrogenase zwf		WP_036906602.	74116..75639	Pentose Phosphate	COG0364
14.1_WP_036906603.1_	CUST_6624_PI442190820	PAC1	LLI	14.1_WP_036906603.1_	ATTGCTTCAAAGAATCCAATCTGGACAAGCTGTTAACGACCTAAATTGGCTTCGCCTTAG	opcA	Glucose-6-P dehydrogenase subunit opcA		WP_036906603.	75677..76984	Pentose Phosphate	COG3429
14.1_WP_036906603.1_	CUST_6625_PI442190820	PAC1	LLI	14.1_WP_036906603.1_	GAGATTAGAAGCGGGAGGGATGGCAAGAATGGAACTTATTGAACAAGTCGTTCCTATTCA	opcA	Glucose-6-P dehydrogenase subunit opcA		WP_036906603.	75677..76984	Pentose Phosphate	COG3429
14.1_WP_036906603.1_	CUST_6626_PI442190820	PAC1	LLI	14.1_WP_036906603.1_	GATATAGACATTGAGGGCTCACAAACAGTTCAAGGTCTTTTACTTGCTGCATGGATTGCC	opcA	Glucose-6-P dehydrogenase subunit opcA		WP_036906603.	75677..76984	Pentose Phosphate	COG3429
14.1_WP_036906603.1_	CUST_6627_PI442190820	PAC1	LLI	14.1_WP_036906603.1_	TATCAATGATGGGCTTGAAATTGTAGAGAATCTTGTTCCCGAGGAACTTCCATCTTGGCT	opcA	Glucose-6-P dehydrogenase subunit opcA		WP_036906603.	75677..76984	Pentose Phosphate	COG3429
14.1_WP_036906603.1_	CUST_6628_PI442190820	PAC1	LLI	14.1_WP_036906603.1_	TCGTTTACTTCCAGTAGTAGAGGCAATACGAGTCCTTTACTTGAGAGTGCGACACCAAT	opcA	Glucose-6-P dehydrogenase subunit opcA		WP_036906603.	75677..76984	Pentose Phosphate	COG3429
14.1_WP_036906603.1_	CUST_6629_PI442190820	PAC1	LLI	14.1_WP_036906603.1_	GGAATGGAAGAATTGATGAGGCTCCTGAATTCCTCAATGCACTATCTCTCCCAAATCGAA	opcA	Glucose-6-P dehydrogenase subunit opcA		WP_036906603.	75677..76984	Pentose Phosphate	COG3429
14.1_WP_036906651.1_	CUST_6800_PI442190820	PAC1	LLI	14.1_WP_036906651.1_	CATGTCGAATGGGTAACATTCTTAAAGGTTGATCAGCTCCCATTAGCGGGACAAATTTCG	rbsK	Sugar kinase, ribokinase family		WP_036906651.	108618..109460	Pentose Phosphate	COG0524
14.1_WP_036906651.1_	CUST_6801_PI442190820	PAC1	LLI	14.1_WP_036906651.1_	ACTGACAAAGAAGGAATAAGATTCGCTAGGAAAGCTAGATTCCTCTCTGCAACTCCCAGA	rbsK	Sugar kinase, ribokinase family		WP_036906651.	108618..109460	Pentose Phosphate	COG0524
14.1_WP_036906651.1_	CUST_6802_PI442190820	PAC1	LLI	14.1_WP_036906651.1_	CGCAGCTGTTCAGATGGCAAGGCTAATAAATAACCCTGTTGACTTAATCACTTCATTGGG	rbsK	Sugar kinase, ribokinase family		WP_036906651.	108618..109460	Pentose Phosphate	COG0524
14.1_WP_036906651.1_	CUST_6803_PI442190820	PAC1	LLI	14.1_WP_036906651.1_	TGGCGTGAGAAACCAACTAGAAAAGGTATTAGCCTAATCAGCAAAGATGGAGAAAGAGCA	rbsK	Sugar kinase, ribokinase family		WP_036906651.	108618..109460	Pentose Phosphate	COG0524
14.1_WP_036906651.1_	CUST_6804_PI442190820	PAC1	LLI	14.1_WP_036906651.1_	GGGGGCTGTGACTACTGCTCTCTCAGCAAAGCTAAATTTAGAGCAAGCGATTAATATAGG	rbsK	Sugar kinase, ribokinase family		WP_036906651.	108618..109460	Pentose Phosphate	COG0524
14.1_WP_036906651.1_	CUST_6805_PI442190820	PAC1	LLI	14.1_WP_036906651.1_	CAAACCCATTACGCCTAGCTCTAAAGAAATAGATACCTATGGTTGTGGAGACAGTTTCGC	rbsK	Sugar kinase, ribokinase family		WP_036906651.	108618..109460	Pentose Phosphate	COG0524
15.1_WP_036906802.1_	CUST_7233_PI442190820	PAC1	LLI	15.1_WP_036906802.1_	AAAAAACGAATCAAAACAATGCCAAAATTGGCTCCTCATAACAGGTGTATTGGGAGTGGC	cyoC	heme-copper oxidase subunit III		WP_036906802.	51800..52405	Respiration	COG1845
15.1_WP_036906802.1_	CUST_7234_PI442190820	PAC1	LLI	15.1_WP_036906802.1_	TAGAGCTCTATTGGCACTTCGTCGATGGGATTTGGGTCGTTTTATTCATCATCCTTTACC	cyoC	heme-copper oxidase subunit III		WP_036906802.	51800..52405	Respiration	COG1845
15.1_WP_036906802.1_	CUST_7235_PI442190820	PAC1	LLI	15.1_WP_036906802.1_	TGCGAGTACTTTTTATGCATTAACTGGCTTTCATGGGCTTCACGTAACACTTGGTTCAAT	cyoC	heme-copper oxidase subunit III		WP_036906802.	51800..52405	Respiration	COG1845
15.1_WP_036906802.1_	CUST_7236_PI442190820	PAC1	LLI	15.1_WP_036906802.1_	TAATGATTATTTGGTGGCAAACACGTGTCCCATCTGGCCGAGTGAATAATGAAAACAAGT	cyoC	heme-copper oxidase subunit III		WP_036906802.	51800..52405	Respiration	COG1845
15.1_WP_036906802.1_	CUST_7237_PI442190820	PAC1	LLI	15.1_WP_036906802.1_	TTTGTTTGGCTTAATAGCTTTTTTAATCGCAGACGGGATGACCTTTGCAGGTTTTTTCGC	cyoC	heme-copper oxidase subunit III		WP_036906802.	51800..52405	Respiration	COG1845
15.1_WP_036906802.1_	CUST_7238_PI442190820	PAC1	LLI	15.1_WP_036906802.1_	AGTTCCAAAAGAGCAATCCTCAGGAAGCAAGAATGAGCTTTCAACTGAAGAACATGAAGA	cyoC	heme-copper oxidase subunit III		WP_036906802.	51800..52405	Respiration	COG1845
15.1_WP_036906805.1_	CUST_7239_PI442190820	PAC1	LLI	15.1_WP_036906805.1_	AGGCTTTAATCTTTGTTTTGCTCCTCAACATTGGCTAGGTTTAAACGGAATGCCTAGACG	cyoB/coxA	Cytochrome c oxidase subunit I		WP_036906805.	52402..54042	Respiration	COG0843
15.1_WP_036906805.1_	CUST_7240_PI442190820	PAC1	LLI	15.1_WP_036906805.1_	TGCGACCTTATGGGGAGGAAAAATATCACTTAATGCTGCAATGTTATTTTCCTGCGGATT	cyoB/coxA	Cytochrome c oxidase subunit I		WP_036906805.	52402..54042	Respiration	COG0843
15.1_WP_036906805.1_	CUST_7241_PI442190820	PAC1	LLI	15.1_WP_036906805.1_	CAAGCGGTACTCCTCCTTGGATGCGCTTATTTTTTACAATCGCTACTGCATTTATAGCTG	cyoB/coxA	Cytochrome c oxidase subunit I		WP_036906805.	52402..54042	Respiration	COG0843
15.1_WP_036906805.1_	CUST_7242_PI442190820	PAC1	LLI	15.1_WP_036906805.1_	GGAGTTGCCCTAGCGCAAGTACCTTTTGATGTTCATGTACACGACACTTATTTTGTTGTT	cyoB/coxA	Cytochrome c oxidase subunit I		WP_036906805.	52402..54042	Respiration	COG0843
15.1_WP_036906805.1_	CUST_7243_PI442190820	PAC1	LLI	15.1_WP_036906805.1_	AGCCCCACTCGTTCTTGAACCTTATGGTTATGGGAAAAAAGATTCAAATGAGACTCAGGA	cyoB/coxA	Cytochrome c oxidase subunit I		WP_036906805.	52402..54042	Respiration	COG0843
15.1_WP_036906805.1_	CUST_7244_PI442190820	PAC1	LLI	15.1_WP_036906805.1_	TGGCGGTTCTGTCTTTGTTATCTTCTCCTCGATTTATCACTGGTTCCCGAAATTTACTGG	cyoB/coxA	Cytochrome c oxidase subunit I		WP_036906805.	52402..54042	Respiration	COG0843
15.1_WP_036906807.1_	CUST_7245_PI442190820	PAC1	LLI	15.1_WP_036906807.1_	GCTACGATATTTATGATCGAATGGGTGGTATGCAACCTTTAATGCATGATCACAGTGGGC	ctaC/coxB	Cytochrome c oxidase subunit II		WP_036906807.	54047..54859	Respiration	COG1622
15.1_WP_036906807.1_	CUST_7246_PI442190820	PAC1	LLI	15.1_WP_036906807.1_	TAATTTCCCAATTGTTTGCGCAGAATTGTGTGGTCCTTATCATGGTGGGATGAGATCAAA	ctaC/coxB	Cytochrome c oxidase subunit II		WP_036906807.	54047..54859	Respiration	COG1622
15.1_WP_036906807.1_	CUST_7247_PI442190820	PAC1	LLI	15.1_WP_036906807.1_	TAGTCTTATCCAATTTCGTCGTAGACCTGGAGAAACTGGAGATGGCATTGACTTAGAGGG	ctaC/coxB	Cytochrome c oxidase subunit II		WP_036906807.	54047..54859	Respiration	COG1622
15.1_WP_036906807.1_	CUST_7248_PI442190820	PAC1	LLI	15.1_WP_036906807.1_	GAGCATGAATGCTCCTGTTTACGATGAACTCTATCGAGTCCTGTTCATTATTGGCGTCAT	ctaC/coxB	Cytochrome c oxidase subunit II		WP_036906807.	54047..54859	Respiration	COG1622
15.1_WP_036906807.1_	CUST_7249_PI442190820	PAC1	LLI	15.1_WP_036906807.1_	TACATGCTTTTTGGGTTCCAGAATTCAGACTTAAACAAGATGTCATTCCTGGCCAACCAA	ctaC/coxB	Cytochrome c oxidase subunit II		WP_036906807.	54047..54859	Respiration	COG1622
15.1_WP_036906807.1_	CUST_7250_PI442190820	PAC1	LLI	15.1_WP_036906807.1_	CGGCCATCTTAACTCTTACAACAAGTTTAATTCTTGGAATAGCTGGAGTTTGGGTTGCGT	ctaC/coxB	Cytochrome c oxidase subunit II		WP_036906807.	54047..54859	Respiration	COG1622
15.1_WP_036906838.1_	CUST_7382_PI442190820	PAC1	LLI	15.1_WP_036906838.1_	AATTTGCGAGCAGAGGAATTACTGTGAATGCAGTTGCCCCTGGTTTTATTTCAACTGATA	gdh	3-oxoacyl-acyl-carrier-protein reductase		WP_036906838.	77077..77829	Entner-Dudoroff	COG1028
15.1_WP_036906838.1_	CUST_7383_PI442190820	PAC1	LLI	15.1_WP_036906838.1_	CATTATCAAAATTACTTGAAGGTCAAACTGCAATTGTAACTGGCGCAAGCAGGGGGATTG	gdh	3-oxoacyl-acyl-carrier-protein reductase		WP_036906838.	77077..77829	Entner-Dudoroff	COG1028
15.1_WP_036906838.1_	CUST_7384_PI442190820	PAC1	LLI	15.1_WP_036906838.1_	CGCAGGGATAACTAAAGATGGCCTTTTAATGAGAATGAAAACGGACGATTGGCAGAAAGT	gdh	3-oxoacyl-acyl-carrier-protein reductase		WP_036906838.	77077..77829	Entner-Dudoroff	COG1028
15.1_WP_036906838.1_	CUST_7385_PI442190820	PAC1	LLI	15.1_WP_036906838.1_	TAAACTCCTTTGGAGGGAAGGCATACCCTCTTCAAGCTGATATTTCTAATGAAAGCTCGG	gdh	3-oxoacyl-acyl-carrier-protein reductase		WP_036906838.	77077..77829	Entner-Dudoroff	COG1028
15.1_WP_036906838.1_	CUST_7386_PI442190820	PAC1	LLI	15.1_WP_036906838.1_	AAGCAAAAAAAAGGAAGAATTGTCAACATAACCTCTGTAGTTGGGTTGATGGGCAACCCA	gdh	3-oxoacyl-acyl-carrier-protein reductase		WP_036906838.	77077..77829	Entner-Dudoroff	COG1028
15.1_WP_036906838.1_	CUST_7387_PI442190820	PAC1	LLI	15.1_WP_036906838.1_	AAAGATCTGAATAGTGAGTCAATCCTTTCTGCTATCCCACTTGGACGATTCGGTAACCCT	gdh	3-oxoacyl-acyl-carrier-protein reductase		WP_036906838.	77077..77829	Entner-Dudoroff	COG1028

15.1_WP_036906935.1_	CUST_7748_PI442190820	PAC1	LLI	15.1_WP_036906935.1_	TGCAGATGGTATTTCTAATGATCGTGTTTTAATCAAAATCGCCTCTACGTGGGAGGGGAT	tal	Transaldolase	WP_036906935.:	136089..137087	Pentose Phosphate	COG0176
15.1_WP_036906935.1_	CUST_7749_PI442190820	PAC1	LLI	15.1_WP_036906935.1_	GAACTAATGATGGCTGGAGATAAAATGGCTACGGAAAAACTTGACGATGGAATTAGCGGC	tal	Transaldolase	WP_036906935.:	136089..137087	Pentose Phosphate	COG0176
15.1_WP_036906935.1_	CUST_7750_PI442190820	PAC1	LLI	15.1_WP_036906935.1_	GGTCATGGGAGCAAGTTTCAGAAATATAGAGGAAATAACCGAGCTTGCAGGATGTGATCT	tal	Transaldolase	WP_036906935.:	136089..137087	Pentose Phosphate	COG0176
15.1_WP_036906935.1_	CUST_7751_PI442190820	PAC1	LLI	15.1_WP_036906935.1_	TTGCAACTTAACTTTGTTGTTTAATTTTTATCAAGCCGTTGCTTGTGCTGAGGCAGGTGT	tal	Transaldolase	WP_036906935.:	136089..137087	Pentose Phosphate	COG0176
15.1_WP_036906935.1_	CUST_7752_PI442190820	PAC1	LLI	15.1_WP_036906935.1_	TTAATTCCTGGCCGTGTTTCAACAGAAGTTGATGCAAGACTAAGTTTTGACACTGATGCG	tal	Transaldolase	WP_036906935.:	136089..137087	Pentose Phosphate	COG0176
15.1_WP_036906935.1_	CUST_7753_PI442190820	PAC1	LLI	15.1_WP_036906935.1_	TTTAGATTGGTATAAATCTGCTACAGGAAGGGATTCTTATCCAGCGACTGAGGACCCAGG	tal	Transaldolase	WP_036906935.:	136089..137087	Pentose Phosphate	COG0176
15.1_WP_036906976.1_	CUST_7911_PI442190820	PAC1	LLI	15.1_WP_036906976.1_	GCAGGTAGAGAAATCGAAAAAGAAAGTCGCGATATCCTTCTTGAAGCTGCAAAGCATAGC	rbcL	Ribulose 1,5-bisphosphate carboxylase large subunit	WP_036906976.:	163282..164694	Carbon fixation	COG1850
15.1_WP_036906976.1_	CUST_7912_PI442190820	PAC1	LLI	15.1_WP_036906976.1_	GAGATCGTCAAACAACTCTTGGTTATATCGACAACCTTCGTGAATCCTTTGTCCCTGAGG	rbcL	Ribulose 1,5-bisphosphate carboxylase large subunit	WP_036906976.:	163282..164694	Carbon fixation	COG1850
15.1_WP_036906976.1_	CUST_7913_PI442190820	PAC1	LLI	15.1_WP_036906976.1_	GAGCTGGCAATTGCTCTTGAGACATGGAAGGAGATTAAGTTCGAATTCGATACAGTCGAT	rbcL	Ribulose 1,5-bisphosphate carboxylase large subunit	WP_036906976.:	163282..164694	Carbon fixation	COG1850
15.1_WP_036906976.1_	CUST_7914_PI442190820	PAC1	LLI	15.1_WP_036906976.1_	GCGTGCTGAGTTTGCTAAAGAACTTGATATGCCCATCATCATGCATGACTACATTACTGG	rbcL	Ribulose 1,5-bisphosphate carboxylase large subunit	WP_036906976.:	163282..164694	Carbon fixation	COG1850
15.1_WP_036906976.1_	CUST_7916_PI442190820	PAC1	LLI	15.1_WP_036906976.1_	GTTTACGCCTATCAGGCGGAGACCAACTTCATACAGGAACAGTTGTTGGAAAACTAGAAG	rbcL	Ribulose 1,5-bisphosphate carboxylase large subunit	WP_036906976.:	163282..164694	Carbon fixation	COG1850
15.1_WP_036906978.1_	CUST_7917_PI442190820	PAC1	LLI	15.1_WP_036906978.1_	GAAGCTTGTCATAGAGCATATCCTGACCACCATGTTCGTATCGTTGGATATGACGCATAC	rbcS	Ribulose bisphosphate carboxylase small subunit	WP_036906978.:	164795..165136	Carbon fixation	COG4451
15.1_WP_036906978.1_	CUST_7918_PI442190820	PAC1	LLI	15.1_WP_036906978.1_	GAGCCCAGTTATTGAGCATGTTCATCCAAGTGGTTCAATGCAGACCTATTGGTCTTATTG	rbcS	Ribulose bisphosphate carboxylase small subunit	WP_036906978.:	164795..165136	Carbon fixation	COG4451
15.1_WP_036906978.1_	CUST_7919_PI442190820	PAC1	LLI	15.1_WP_036906978.1_	TTCTTACCGCCGATGACCCAGGACGAAATTTACGATCAAATCGCTTATATCATTGCTCAG	rbcS	Ribulose bisphosphate carboxylase small subunit	WP_036906978.:	164795..165136	Carbon fixation	COG4451
15.1_WP_036906978.1_	CUST_7920_PI442190820	PAC1	LLI	15.1_WP_036906978.1_	CGCATACACGCAAAGTCAAGGTACTTGCTTTGTAGTTTTCCAAGGCCGCTAG	rbcS	Ribulose bisphosphate carboxylase small subunit	WP_036906978.:	164795..165136	Carbon fixation	COG4451
15.1_WP_036906978.1_	CUST_7921_PI442190820	PAC1	LLI	15.1_WP_036906978.1_	ATGCCTTTCCAGAGCACAGTAGGTGACTATCAGACAGTCGCCACCCTGGAAACATTC	rbcS	Ribulose bisphosphate carboxylase small subunit	WP_036906978.:	164795..165136	Carbon fixation	COG4451
15.1_WP_036906978.1_	CUST_7922_PI442190820	PAC1	LLI	15.1_WP_036906978.1_	GTCTTATTGGAAATTACCTTTCTTCGGTGAAAAGGATTTGAATCTTGTAGT	rbcS	Ribulose bisphosphate carboxylase small subunit	WP_036906978.:	164795..165136	Carbon fixation	COG4451
15.1_WP_036907043.1_	CUST_8166_PI442190820	PAC1	LLI	15.1_WP_036907043.1_	GGGTTATGGATTTCAGGTGGAACAGCCGCAAAAAACCTCGATGGAATAAACTCTTCTAAC	glk	Glucokinase	WP_036907043.:	211127..212170	Glycolysis	COG0837
15.1_WP_036907043.1_	CUST_8167_PI442190820	PAC1	LLI	15.1_WP_036907043.1_	CAAATTAATGACTGAAGCTTTGCAACTATGGCTAAATGCTTATGGGTCTGCAGCTGGAGA	glk	Glucokinase	WP_036907043.:	211127..212170	Glycolysis	COG0837
15.1_WP_036907043.1_	CUST_8168_PI442190820	PAC1	LLI	15.1_WP_036907043.1_	CCCTAAAAGCATTTCTATATTTCCAAGTGAAGGAGGCCATCGAGAATTTGCCCCAAGAAC	glk	Glucokinase	WP_036907043.:	211127..212170	Glycolysis	COG0837
15.1_WP_036907043.1_	CUST_8169_PI442190820	PAC1	LLI	15.1_WP_036907043.1_	CATTGATTGTTCTTAAAGATCCAGAAGCGACATTATTCAGTTCAGCTTGCAGAGCACGCT	glk	Glucokinase	WP_036907043.:	211127..212170	Glycolysis	COG0837
15.1_WP_036907043.1_	CUST_8170_PI442190820	PAC1	LLI	15.1_WP_036907043.1_	TGGCATGATTGCCAGATGGAAATTGGATGATCCAATAAATGAAAGCCATCCACTTCAGAA	glk	Glucokinase	WP_036907043.:	211127..212170	Glycolysis	COG0837
15.1_WP_036907043.1_	CUST_8171_PI442190820	PAC1	LLI	15.1_WP_036907043.1_	ACAATCAAAAATTAATTGCAATTATCGGAGCTGGTACTGGCTTAGGAATGTCCAGAGGCT	glk	Glucokinase	WP_036907043.:	211127..212170	Glycolysis	COG0837
15.1_WP_036907078.1_	CUST_8343_PI442190820	PAC1	LLI	15.1_WP_036907078.1_	TCAAGGGATCTGGAAAATGGCCAGTTCTATTCCAGAAGCATTGAAAAATGTTGATGCAGT	ugd	UDP-glucose 6-dehydrogenase/nucleotide sugar dehydrogenase	WP_036907078.:	243882..245285	Amino sugar and nucleotide sugar r	COG1004
15.1_WP_036907078.1_	CUST_8344_PI442190820	PAC1	LLI	15.1_WP_036907078.1_	TCAATTTCTTAGTGCAGGTCCTGGCTTTGGTGGGAGCTGCTTAAAAAGGATATTTTGAA	ugd	UDP-glucose 6-dehydrogenase/nucleotide sugar dehydrogenase	WP_036907078.:	243882..245285	Amino sugar and nucleotide sugar r	COG1004
15.1_WP_036907078.1_	CUST_8345_PI442190820	PAC1	LLI	15.1_WP_036907078.1_	GGCAAACACAAACGATACCAGAGAATCACCTGCGATAAGAATTTCCAGTGACTTACTCGA	ugd	UDP-glucose 6-dehydrogenase/nucleotide sugar dehydrogenase	WP_036907078.:	243882..245285	Amino sugar and nucleotide sugar r	COG1004
15.1_WP_036907078.1_	CUST_8346_PI442190820	PAC1	LLI	15.1_WP_036907078.1_	GTGAGGCTACTGGAGCTGATGTTCAGGAAGTTGCTAAAGCAATAGGAACAGATAAAAGGA	ugd	UDP-glucose 6-dehydrogenase/nucleotide sugar dehydrogenase	WP_036907078.:	243882..245285	Amino sugar and nucleotide sugar r	COG1004
15.1_WP_036907078.1_	CUST_8347_PI442190820	PAC1	LLI	15.1_WP_036907078.1_	TGGATTACCAGAGGTTGCTAATTACTGGAATCAAGTTGTCGTGCTAAATACTTGGCAGCA	ugd	UDP-glucose 6-dehydrogenase/nucleotide sugar dehydrogenase	WP_036907078.:	243882..245285	Amino sugar and nucleotide sugar r	COG1004
15.1_WP_036907078.1_	CUST_8348_PI442190820	PAC1	LLI	15.1_WP_036907078.1_	TGTTAATCGACAAGAAGTTGAAGATACAGGTGTAAATCTTTGGAAGCTGGGTCAGGGGAA	ugd	UDP-glucose 6-dehydrogenase/nucleotide sugar dehydrogenase	WP_036907078.:	243882..245285	Amino sugar and nucleotide sugar r	COG1004
15.1_WP_036907187.1_	CUST_8775_PI442190820	PAC1	LLI	15.1_WP_036907187.1_	GATTCGAGGCACCAGTTAATTTAGTTTACTCTCAGGGCAATCGATCAGCTGCTGTAAGAA	glnA	Glutamine synthetase/ glutamate--ammonialigase	WP_036907187.:	318986..320407	Nitrogen Metabolism	COG0174
15.1_WP_036907187.1_	CUST_8776_PI442190820	PAC1	LLI	15.1_WP_036907187.1_	CAGTCTTCAATGACAATGGGACAGGAATGCATGTTCATCAAAGTCTTTGGAAAGGAGGTC	glnA	Glutamine synthetase/ glutamate--ammonialigase	WP_036907187.:	318986..320407	Nitrogen Metabolism	COG0174
15.1_WP_036907187.1_	CUST_8777_PI442190820	PAC1	LLI	15.1_WP_036907187.1_	ACATGCACCGTCTTTCCTTGCTTTTACCAATCCAACTACCAACAGTTACAAAAGATTGGT	glnA	Glutamine synthetase/ glutamate--ammonialigase	WP_036907187.:	318986..320407	Nitrogen Metabolism	COG0174
15.1_WP_036907187.1_	CUST_8778_PI442190820	PAC1	LLI	15.1_WP_036907187.1_	ACCAAGTCCAAAAGCAAAGAGACTTGAATTCAGATCAGGTGACGCATTAGCTAACCCTTA	glnA	Glutamine synthetase/ glutamate--ammonialigase	WP_036907187.:	318986..320407	Nitrogen Metabolism	COG0174
15.1_WP_036907187.1_	CUST_8779_PI442190820	PAC1	LLI	15.1_WP_036907187.1_	ATCAAATTGATCCTGGAGATGGAGTTGACGTTGACCTTTTTGAGCTTCCTTCAGAGGAAT	glnA	Glutamine synthetase/ glutamate--ammonialigase	WP_036907187.:	318986..320407	Nitrogen Metabolism	COG0174
15.1_WP_036907187.1_	CUST_8780_PI442190820	PAC1	LLI	15.1_WP_036907187.1_	AGACACTGTCCCCTCTTCATTGAACGATGCATTAGAGGCATTAAAAAATGACAGCCAGTA	glnA	Glutamine synthetase/ glutamate--ammonialigase	WP_036907187.:	318986..320407	Nitrogen Metabolism	COG0174
15.1_WP_036907250.1_	CUST_8969_PI442190820	PAC1	LLI	15.1_WP_036907250.1_	GTGTTGGGAGTTAGGAGTCGGATTGAAAGCGACGTTGTTCTCAATGAGACCTTAGTTATG	glgC	Glucose-1-phosphate adenylyltransferase	WP_036907250.:	357607..358902	Glycogen Metabolism	COG0448
15.1_WP_036907250.1_	CUST_8970_PI442190820	PAC1	LLI	15.1_WP_036907250.1_	TTTTACGAATCTTATGAAGAGAGAATAGCGCTAAGAAATGGTGGTGGTATCCCCTTGGGC	glgC	Glucose-1-phosphate adenylyltransferase	WP_036907250.:	357607..358902	Glycogen Metabolism	COG0448
15.1_WP_036907250.1_	CUST_8971_PI442190820	PAC1	LLI	15.1_WP_036907250.1_	GGAACTATTGGTGCATTCTTTGAATCAAATTTAGCCTTAACTCAGCAACCTACCCCTCCG	glgC	Glucose-1-phosphate adenylyltransferase	WP_036907250.:	357607..358902	Glycogen Metabolism	COG0448
15.1_WP_036907250.1_	CUST_8972_PI442190820	PAC1	LLI	15.1_WP_036907250.1_	ACCTACTGGTGATTCATTGAAAGCAATGGCTGTAGATACATCAAGGTTTGGATTGGAAGC	glgC	Glucose-1-phosphate adenylyltransferase	WP_036907250.:	357607..358902	Glycogen Metabolism	COG0448
15.1_WP_036907250.1_	CUST_8973_PI442190820	PAC1	LLI	15.1_WP_036907250.1_	GCACAACTGTTAAAAGAGCCATTCTCGATAAAAATGCTCGCATTGGTGACAATGTGACGA	glgC	Glucose-1-phosphate adenylyltransferase	WP_036907250.:	357607..358902	Glycogen Metabolism	COG0448
15.1_WP_036907250.1_	CUST_8974_PI442190820	PAC1	LLI	15.1_WP_036907250.1_	TCCTTCTTATACCGATTTTGGAAAAGAAATTATTCCGGAGGCTTTAGGTAGAGGTGACAA	glgC	Glucose-1-phosphate adenylyltransferase	WP_036907250.:	357607..358902	Glycogen Metabolism	COG0448
15.1_WP_036907253.1_	CUST_8975_PI442190820	PAC1	LLI	15.1_WP_036907253.1_	GGATGCCGTTGTATTGGCTACAATAGCTAGCTATGCCCAAGGTATGGATATTTTACGACT	edd	phosphogluconate dehydrogenase (NADP(+)-dependent, decarboxyla	WP_036907253.:	359060..360478	Pentose Phosphate/Entner-Dudorof	COG2084
15.1_WP_036907253.1_	CUST_8976_PI442190820	PAC1	LLI	15.1_WP_036907253.1_	AATGGGAACAAACCTTCTTTTGTTGATTTTGGAAAGCCTTCAGATGGTATGCCTCTCCTT	edd	phosphogluconate dehydrogenase (NADP(+)-dependent, decarboxyla	WP_036907253.:	359060..360478	Pentose Phosphate/Entner-Dudorof	COG2084
15.1_WP_036907253.1_	CUST_8977_PI442190820	PAC1	LLI	15.1_WP_036907253.1_	TCCTCAAAACCTAGTTCAAGCAATGAGAGACTGTTTTGGTTCTCATACATATGAGCGCGT	edd	phosphogluconate dehydrogenase (NADP(+)-dependent, decarboxyla	WP_036907253.:	359060..360478	Pentose Phosphate/Entner-Dudorof	COG2084
15.1_WP_036907253.1_	CUST_8978_PI442190820	PAC1	LLI	15.1_WP_036907253.1_	CCTCCATCGCTCAAATATGGAAAGGTGGTTGCATAATAAGGTCTACCCTTTTGAGTCGTA	edd	phosphogluconate dehydrogenase (NADP(+)-dependent, decarboxyla	WP_036907253.:	359060..360478	Pentose Phosphate/Entner-Dudorof	COG2084
15.1_WP_036907253.1_	CUST_8979_PI442190820	PAC1	LLI	15.1_WP_036907253.1_	GTTATTGATAAATGGTTTACCGATCAGGTAAACAATCGTCTCTCGGGCCTGACACAGGTC	edd	phosphogluconate dehydrogenase (NADP(+)-dependent, decarboxyla	WP_036907253.:	359060..360478	Pentose Phosphate/Entner-Dudorof	COG2084
15.1_WP_036907253.1_	CUST_8980_PI442190820	PAC1	LLI	15.1_WP_036907253.1_	TAATGGATAAAGCGGGTCAAAAAGGTACTGGATTATGGACAGTCGTTAGTGCCTTAGAGC	edd	phosphogluconate dehydrogenase (NADP(+)-dependent, decarboxyla	WP_036907253.:	359060..360478	Pentose Phosphate/Entner-Dudorof	COG2084
15.1_WP_036907281.1_	CUST_9040_PI442190820	PAC1	LLI	15.1_WP_036907281.1_	TAGATGGACTAGTTGAAAGAGCTGCTGATTACTACGAGCAAGGTGCAAGATTCGCAAAAT	fda	Fructose-1,6-bisphosphate aldolase class I	WP_036907281.:	367919..368986	Glycolysis/Gluconeogenesis	COG0191
15.1_WP_036907281.1_	CUST_9041_PI442190820	PAC1	LLI	15.1_WP_036907281.1_	AACCCGAAATCCTAATGGATGGTTCACATTCAATTGAAAAGACTGCCGCAGTACAAGAAG	fda	Fructose-1,6-bisphosphate aldolase class I	WP_036907281.:	367919..368986	Glycolysis/Gluconeogenesis	COG0191
15.1_WP_036907281.1_	CUST_9042_PI442190820	PAC1	LLI	15.1_WP_036907281.1_	CATATGGTCGTGCTTTGCAACACTCATGTCTAAAAGCATGGAAAGGCTCTAATACTGCTG	fda	Fructose-1,6-bisphosphate aldolase class I	WP_036907281.:	367919..368986	Glycolysis/Gluconeogenesis	COG0191
15.1_WP_036907281.1_	CUST_9043_PI442190820	PAC1	LLI	15.1_WP_036907281.1_	CTCAAGCAAACTCTGAAGCATCAAAAGGATTGTATGTTGCTGGTTCTCAGCCTTCTTCTG	fda	Fructose-1,6-bisphosphate aldolase class I	WP_036907281.:	367919..368986	Glycolysis/Gluconeogenesis	COG0191
15.1_WP_036907281.1_	CUST_9044_PI442190820	PAC1	LLI	15.1_WP_036907281.1_	ACGGAGTACTTCTAGAAGGAACTCTTCTAAAGCCATCAATGACAGTACAAGGTGCTGACA	fda	Fructose-1,6-bisphosphate aldolase class I	WP_036907281.:	367919..368986	Glycolysis/Gluconeogenesis	COG0191
15.1_WP_036907281.1_	CUST_9045_PI442190820	PAC1	LLI	15.1_WP_036907281.1_	TCCCAGGTATTACTTTCCTTTCAGGTGGTTTGAGCGAGGAAGCAGCATCAGTTTATTTAA	fda	Fructose-1,6-bisphosphate aldolase class I	WP_036907281.:	367919..368986	Glycolysis/Gluconeogenesis	COG0191
15.1_WP_036907900.1_	CUST_9069_PI442190820	PAC1	LLI	15.1_WP_036907900.1_	ACAAGTATTGTTGTTAACGGCGGGAAAATGGGTTTTGCGATGGAACTTATTCTTACACCT	prk	Phosphoribulokinase	WP_036907900.:	372130..373032	Carbon Fixation	COG3954
15.1_WP_036907900.1_	CUST_9070_PI442190820	PAC1	LLI	15.1_WP_036907900.1_	GAGAGGATACTCAGCCGAGACGATCGTTGATACAATTCTGAGAAGAATGCCTGACTACAT	prk	Phosphoribulokinase	WP_036907900.:	372130..373032	Carbon Fixation	COG3954
15.1_WP_036907900.1_	CUST_9071_PI442190820	PAC1	LLI	15.1_WP_036907900.1_	ACGTCAAACCCATTTATCTGCAGAAACATTCCCACACCTGACGAAAGCTTTGTAATCATT	prk	Phosphoribulokinase	WP_036907900.:	372130..373032	Carbon Fixation	COG3954
15.1_WP_036907900.1_	CUST_9072_PI442190820	PAC1	LLI	15.1_WP_036907900.1_	CCAGGTCAATTCACTCCTTGGGAAGATATTCCATCAGGAACCGACCTTTTGTTCTATGAA	prk	Phosphoribulokinase	WP_036907900.:	372130..373032	Carbon Fixation	COG3954
15.1_WP_036907900.1_	CUST_9073_PI442190820	PAC1	LLI	15.1_WP_036907900.1_	TTATCACCGTTTCGAAAGAATGCCTATGAAAAAGGCAATGTCTGAGGCACTTTCAAGAGG	prk	Phosphoribulokinase	WP_036907900.:	372130..373032	Carbon Fixation	COG3954
15.1_WP_036907900.1_	CUST_9074_PI442190820	PAC1	LLI	15.1_WP_036907900.1_	GAGGTCAGAAAAGATATTATTTACATAGCCCAGAAGAAGCCGAGGAGCACAACACTCGTT	prk	Phosphoribulokinase	WP_036907900.:	372130..373032	Carbon Fixation	COG3954
15.1_WP_036907349.1_	CUST_9226_PI442190820	PAC1	LLI	15.1_WP_036907349.1_	TGAAAGTCGAAGAGATGGTGATGTGGCAGTGTCTTACGCAGATATATCAAAGCCAAGAG	galE	UDP-glucose 4-epimerase	WP_036907349.:	396836..397882	Galactose metabolism	COG1087
15.1_WP_036907349.1_	CUST_9227_PI442190820	PAC1	LLI	15.1_WP_036907349.1_	ATTTATGGAAAATTTGTTCCTTGCGTTATTTTAATCCTGTTGGTGCGCATCCTTCGGCC	galE	UDP-glucose 4-epimerase	WP_036907349.:	396836..397882	Galactose metabolism	COG1087
15.1_WP_036907349.1_	CUST_9228_PI442190820	PAC1	LLI	15.1_WP_036907349.1_	AGTTGGACTCCTAAGAGATCCTTAGAACAGATGTGCCTAGATGGATGGAATTGGCAAATG	galE	UDP-glucose 4-epimerase	WP_036907349.:	396836..397882	Galactose metabolism	COG1087
15.1_WP_036907349.1_	CUST_9229_PI442190820	PAC1	LLI	15.1_WP_036907349.1_	AAAGACTTTAAGCATTTATGAGATGATTGGGAAACAAAGGATGGTTCAGGGATTCGTGA	galE	UDP-glucose 4-epimerase	WP_036907349.:	396836..397882	Galactose metabolism	COG1087
15.1_WP_036907349.1_	CUST_9230_PI442190820	PAC1	LLI	15.1_WP_036907349.1_	TGTCCCTATCTTAGAAGAACAAAAGATTTCACCAATAACACCCTATGGTCAAACCAAAGT	galE	UDP-glucose 4-epimerase	WP_036907349.:	396836..397882	Galactose metabolism	COG1087
15.1_WP_036907349.1_	CUST_9231_PI442190820	PAC1	LLI	15.1_WP_036907349.1_	TCTTGCTGGTGTTAAGTCAGTAGTCGAATCTTTGACTAATCCTCTTTATTATTGGGATGT	galE	UDP-glucose 4-epimerase	WP_036907349.:	396836..397882	Galactose metabolism	COG1087
15.1_WP_036907482.1_	CUST_9535_PI442190820	PAC1	LLI	15.1_WP_036907482.1_	CGAGAATGCGTCACCATTGTTTAAAAGCAGTCGAGCTTCTTGAAGGTAAAGGAATCGATG	pdhB	Pyruvate dehydrogenase E1 component beta subunit	WP_036907482.:	447976..448965	Glycolysis/Krebs cycle	COG0022
15.1_WP_036907482.1_	CUST_9536_PI442190820	PAC1	LLI	15.1_WP_036907482.1_	GGTTTAAGGCCAATTGTGGAAGGGATGAATATGGGTTTTTGTTGCTGGCGTTTAATCAA	pdhB	Pyruvate dehydrogenase E1 component beta subunit	WP_036907482.:	447976..448965	Glycolysis/Krebs cycle	COG0022
15.1_WP_036907482.1_	CUST_9537_PI442190820	PAC1	LLI	15.1_WP_036907482.1_	AATATGGGCATGCTTAGATACACGAGCGGCGGAAATTTTACTATCCCAACAGTTGTTAGA	pdhB	Pyruvate dehydrogenase E1 component beta subunit	WP_036907482.:	447976..448965	Glycolysis/Krebs cycle	COG0022
15.1_WP_036907482.1_	CUST_9538_PI442190820	PAC1	LLI	15.1_WP_036907482.1_	GGCGCTGAACACAGTCAAAGACTTGAAGCCTATTTTCATGCTGTTCCTGGTATCAAAATT	pdhB	Pyruvate dehydrogenase E1 component beta subunit	WP_036907482.:	447976..448965	Glycolysis/Krebs cycle	COG0022
15.1_WP_036907482.1_	CUST_9539_PI442190820	PAC1	LLI	15.1_WP_036907482.1_	ATTGCTTCGATGATTTAGATTCTCCTCCCGTTCGTTTAAGCAGTCAGGATATTCCGACTC	pdhB	Pyruvate dehydrogenase E1 component beta subunit	WP_036907482.:	447976..448965	Glycolysis/Krebs cycle	COG0022
15.1_WP_036907482.1_	CUST_9540_PI442190820	PAC1	LLI	15.1_WP_036907482.1_	TGTGGCTTGTAGTACTCCTACAAATGCTAAAGGTCTAATGAAAGCTGCGATAAGGGACA	pdhB	Pyruvate dehydrogenase E1 component beta subunit	WP_036907482.:	447976..448965	Glycolysis/Krebs cycle	COG0022
15.1_WP_036907510.1_	CUST_9600_PI442190820	PAC1	LLI	15.1_WP_036907510.1_	TTATCCCATTGAGGCTGTGGCGACTATGGCTACTATTGCAAAAGAATCGAACGAGATTA	pykF	Pyruvate kinase	WP_036907510.:	460363..461820	Glycolysis	COG0469
15.1_WP_036907510.1_	CUST_9601_PI442190820	PAC1	LLI	15.1_WP_036907510.1_	AAATCCTCAAGCAAGGAGATCTTGTTGTTGAGACAGCTGGAACACTATCAGGTGTAAGTG	pykF	Pyruvate kinase	WP_036907510.:	460363..461820	Glycolysis	COG0469
15.1_WP_036907510.1_	CUST_9602_PI442190820	PAC1	LLI	15.1_WP_036907510.1_	AAAGTGGTGCTACTGCTAAAAACGTAAGCAAGTTTAGACCTCCCACTCCAGTATTAGCAG	pykF	Pyruvate kinase	WP_036907510.:	460363..461820	Glycolysis	COG0469
15.1_WP_036907510.1_	CUST_9603_PI442190820	PAC1	LLI	15.1_WP_036907510.1_	ATGCGATTAGTGCGGCTGTAAGCAGTATTGCTAGACAAATAAACGCTGCAGCAATAATAC	pykF	Pyruvate kinase	WP_036907510.:	460363..461820	Glycolysis	COG0469
15.1_WP_036907510.1_	CUST_9604_PI442190820	PAC1	LLI	15.1_WP_036907510.1_	TCAAAAAGGCAAACAGCCTTGGCATACCAATAATTACAGCCACACAAATGCTTGATTCAA	pykF	Pyruvate kinase	WP_036907510.:	460363..461820	Glycolysis	COG0469
15.1_WP_036907510.1_	CUST_9605_PI442190820	PAC1	LLI	15.1_WP_036907510.1_	GACGCTGTTTTAAAATTATGTGATGGAGTCATGGTGGCTAGAGGTGATCTCGGTGTAGAG	pykF	Pyruvate kinase	WP_036907510.:	460363..461820	Glycolysis	COG0469
15.1_WP_036907556.1_	CUST_9739_PI442190820	PAC1	LLI	15.1_WP_036907556.1_	AGCTGTGAGTCTCTATGCCGAATTGATTAATGTTAATGCCTACAATCAACCGGGAGTTGA	pgi	Glucose-6-phosphate isomerase	WP_036907556.:	484531..486129	Glycolysis/Gluconeogenesis	COG0166
15.1_WP_036907556.1_	CUST_9740_PI442190820	PAC1	LLI	15.1_WP_036907556.1_	GTCTTTAGCTTGGTTCAAATCCGGAAATGGAAAAGGGTTGAGGGATATGGTTGTTCTTCC	pgi	Glucose-6-phosphate isomerase	WP_036907556.:	484531..486129	Glycolysis/Gluconeogenesis	COG0166
15.1_WP_036907556.1_	CUST_9741_PI442190820	PAC1	LLI	15.1_WP_036907556.1_	AAGACCAGGTGACTATTTGTCTGGATTTCTTCAAGGAACTCGTTCAGCATTGACTGAAGG	pgi	Glucose-6-phosphate isomerase	WP_036907556.:	484531..486129	Glycolysis/Gluconeogenesis	COG0166
15.1_WP_036907556.1_	CUST_9742_PI442190820	PAC1	LLI	15.1_WP_036907556.1_	AGGTATCGCAGTCTATGGAAACAAAGGTTCTACAGATCAGCATGCATATGTGCAGCAATT	pgi	Glucose-6-phosphate isomerase	WP_036907556.:	484531..486129	Glycolysis/Gluconeogenesis	COG0166
15.1_WP_036907556.1_	CUST_9743_PI442190820	PAC1	LLI	15.1_WP_036907556.1_	TCTTCAACAACTTGTGATGGAATCTTTGGGCAAGAAGTTTGATAGAGATGAAAATCAAGT	pgi	Glucose-6-phosphate isomerase	WP_036907556.:	484531..486129	Glycolysis/Gluconeogenesis	COG0166
15.1_WP_036907556.1_	CUST_9744_PI442190820	PAC1	LLI	15.1_WP_036907556.1_	AATCACTTTTAAGTCATTTGATGAATCAAGTCTTGGTGCTTTGATTGCATTATTTGAGCG	pgi	Glucose-6-phosphate isomerase	WP_036907556.:	484531..486129	Glycolysis/Gluconeogenesis	COG0166
WP_011293972.1_12	CUST_67_PI442190932	NATL2	LLI	WP_011293972.1_12	CCGACCGATGTGCTACTGGAAGTAGTCTTATGCCTCAAAAAAAGAATCCTGATGTACCAG	argH	Argininosuccinate lyase	WP_011293972.:	14786..16177	Amino Acid metabolism	COG0165
WP_011293972.1_12	CUST_68_PI442190932	NATL2	LLI	WP_011293972.1_12	TTCAAATGCCACTGATGTAGCTGATTATTTAGTGGCTAAGGACATACCTTTCCGAGAGGC	argH	Argininosuccinate lyase	WP_011293972.:	14786..16177	Amino Acid metabolism	COG0165
WP_011293972.1_12	CUST_69_PI442190932	NATL2	LLI	WP_011293972.1_12	AAAAGATATTTATGAAAAGCTTTTGCCTTCGAGTGTGGTCGAGTCTCGTTTGAGTGCTGG	argH	Argininosuccinate lyase	WP_011293972.:	14786..16177	Amino Acid metabolism	COG0165
WP_011293972.1_12	CUST_70_PI442190932	NATL2	LLI	WP_011293972.1_12	AGCTTGTTCGAGGCAAGTCAGGAAGAGTATTTGGACATTTACAAGCTATGCTGACTATGA	argH	Argininosuccinate lyase	WP_011293972.:	14786..16177	Amino Acid metabolism	COG0165
WP_011293972.1_12	CUST_71_PI442190932	NATL2	LLI	WP_011293972.1_12	GCTGGTGGAACTGGATTTGAGAGAGTTCAAGAACAGCTTCTTTCTTGGCGAGAAA	argH	Argininosuccinate lyase	WP_011293972.:	14786..16177	Amino Acid metabolism	COG0165
WP_011293972.1_12	CUST_72_PI442190932	NATL2	LLI	WP_011293972.1_12	TGCTGTAAGTGATAGAGACTTTGTTGTAGAATTTTTAGGAGCCTCGTCGTTAATTATGGC	argH	Argininosuccinate lyase	WP_011293972.:	14786..16177	Amino Acid metabolism	COG0165
WP_011294208.1_248	CUST_1360_PI442190932	NATL2	LLI	WP_011294208.1_248	GGTGATGAAGGTGGATTCGCTCCAAATCTTGAGAGTAATAAAGCAGCAGGCGATCTATTA	eno	Enolase	WP_011294208.:	245573..246874	Glycolysis/Gluconeogenesis	COG0148
WP_011294208.1_248	CUST_1361_PI442190932	NATL2	LLI	WP_011294208.1_248	TAGCAACTAAATCTGGTCAAATCAAAACAGGTTCATTGAGTCGGAGTGAAAGAGTCGCGA	eno	Enolase	WP_011294208.:	245573..246874	Glycolysis/Gluconeogenesis	COG0148
WP_011294208.1_248	CUST_1362_PI442190932	NATL2	LLI	WP_011294208.1_248	ATGATTGGGATGGTTGGAGATTGCTTACGAAAAAGCTCGGCAAAAATGTGCAATTAGTTG	eno	Enolase	WP_011294208.:	245573..246874	Glycolysis/Gluconeogenesis	COG0148
WP_011294208.1_248	CUST_1363_PI442190932	NATL2	LLI	WP_011294208.1_248	CTTTAGATGTTGCGAGCACGGAGTTTTATGCGGAGGGACAATATTGTTATGGTGGAAATT	eno	Enolase	WP_011294208.:	245573..246874	Glycolysis/Gluconeogenesis	COG0148
WP_011294208.1_248	CUST_1364_PI442190932	NATL2	LLI	WP_011294208.1_248	TACTTCGTATTGAGGATGAGTTAGGCAGTCAAGCAACTTATGCTGGACTTGTTGGATTGG	eno	Enolase	WP_011294208.:	245573..246874	Glycolysis/Gluconeogenesis	COG0148
WP_011294208.1_248	CUST_1365_PI442190932	NATL2	LLI	WP_011294208.1_248	TTCCAGCGAGCAAATGGTTGAGGAATTGGCTGGATTAGTTAATGCATTTCCAATTGTTTC	eno	Enolase	WP_011294208.:	245573..246874	Glycolysis/Gluconeogenesis	COG0148
WP_011294227.1_267	CUST_1462_PI442190932	NATL2	LLI	WP_011294227.1_267	CCTACAAAGTGGATATGATGCTTTGGTGATAGGAAGAGAATTGCGAAATCAAACACCTGA	eda	2-dehydro-3-deoxyphosphogluconate aldolase	WP_011294227.:	265219..265854	Entner-Dudoroff	COG0800
WP_011294227.1_267	CUST_1463_PI442190932	NATL2	LLI	WP_011294227.1_267	ATTGGTGTTGCATCAATTTCTTCAAGGCAATCTTTAGAATCACTTCTCTCATTAGATGTT	eda	2-dehydro-3-deoxyphosphogluconate aldolase	WP_011294227.:	265219..265854	Entner-Dudoroff	COG0800
WP_011294227.1_267	CUST_1464_PI442190932	NATL2	LLI	WP_011294227.1_267	ATTGTCTAATTTCGGTATAAAAAATATTGAAATAGGGTGGGATTCCAATCCAGAATGGGT	eda	2-dehydro-3-deoxyphosphogluconate aldolase	WP_011294227.:	265219..265854	Entner-Dudoroff	COG0800
WP_011294227.1_267	CUST_1465_PI442190932	NATL2	LLI	WP_011294227.1_267	TGGAAGCTAAGCAAAACTATTTAAAAAAAACTCTTAGGATACAGCCGCTTATAGTTGTAA	eda	2-dehydro-3-deoxyphosphogluconate aldolase	WP_011294227.:	265219..265854	Entner-Dudoroff	COG0800
WP_011294227.1_267	CUST_1466_PI442190932	NATL2	LLI	WP_011294227.1_267	CAATTAGTTATTCCAGGGATATCAAATATTGAAAATTTCAAAGAGGCAATCGATCTAGGA	eda	2-dehydro-3-deoxyphosphogluconate aldolase	WP_011294227.:	265219..265854	Entner-Dudoroff	COG0800
WP_011294227.1_267	CUST_1467_PI442190932	NATL2	LLI	WP_011294227.1_267	AAAAGACTTTAAAAAAAAGGATATTTTTTTATTGGTGCAGGTGGAATTAAAAGTAAGCA	eda	2-dehydro-3-deoxyphosphogluconate aldolase	WP_011294227.:	265219..265854	Entner-Dudoroff	COG0800
WP_041711143.1_371	CUST_2028_PI442190932	NATL2	LLI	WP_041711143.1_371	GAGCCTAAGAATCAGTCAGAAGTAGATTCTCCAGAAACTGAAGGCAAAGATCAAAACTCT	minE	septum site-determining protein MinE	WP_041711143.:	362931..363272	Cell Division	COG0851
WP_041711143.1_371	CUST_2029_PI442190932	NATL2	LLI	WP_041711143.1_371	ATCGCATGACCGCATTAGTTGCAAACCTACCAATAAAAAGAACTATGACTGGGCAAATCC	minE	septum site-determining protein MinE	WP_041711143.:	362931..363272	Cell Division	COG0851
WP_041711143.1_371	CUST_2030_PI442190932	NATL2	LLI	WP_041711143.1_371	AGGTGGTCGCAAAGTATGTAGAGATAGATGTGGATGAAGGAGCAGTAAGTCTTGAGACAG	minE	septum site-determining protein MinE	WP_041711143.:	362931..363272	Cell Division	COG0851
WP_041711143.1_371	CUST_2031_PI442190932	NATL2	LLI	WP_041711143.1_371	GCGGTTGCAACTTGTTCTGGCTCATGATCGATCAGATCTCAGTACAGAACTTTTAGATCA	minE	septum site-determining protein MinE	WP_041711143.:	362931..363272	Cell Division	COG0851
WP_041711143.1_371	CUST_2032_PI442190932	NATL2	LLI	WP_041711143.1_371	GACACTTAGAGACATTATCAACAAGTTACTACGTAGACAGCCTGCTAGCGCTAGTACCGC	minE	septum site-determining protein MinE	WP_041711143.:	362931..363272	Cell Division	COG0851
WP_011294332.1_372	CUST_2033_PI442190932	NATL2	LLI	WP_011294332.1_372	CCAATCTATCTTTACTTCCTGCGGGGAATCCAAGAATGCTTGATTGGTTAAAACCCGATG	minD	septum site-determining protein MinD	WP_011294332.:	363283..364098	Cell Division	COG2894
WP_011294332.1_372	CUST_2034_PI442190932	NATL2	LLI	WP_011294332.1_372	GCCTTGTGCTAGAGGATGAACAAGTAATTGTAAGTACAAACCGAGGTGAGCCACTAACTT	minD	septum site-determining protein MinD	WP_011294332.:	363283..364098	Cell Division	COG2894
WP_011294332.1_372	CUST_2035_PI442190932	NATL2	LLI	WP_011294332.1_372	ATCGAATTGTATATACGGCTCAAGAAGTTCTAGAGGAGGAATGCAGACTTGATCAGGCCT	minD	septum site-determining protein MinD	WP_011294332.:	363283..364098	Cell Division	COG2894
WP_011294332.1_372	CUST_2036_PI442190932	NATL2	LLI	WP_011294332.1_372	TCTCAAGAAGCAATTGTAGTTACCAACCCTGAGGTATCAGCAGTAAGAGATGCAGACAGA	minD	septum site-determining protein MinD	WP_011294332.:	363283..364098	Cell Division	COG2894
WP_011294332.1_372	CUST_2037_PI442190932	NATL2	LLI	WP_011294332.1_372	AACCAGTCCAATTAGTTCTTAATCGAGTTAGGCCAAAGATGATGGCCAACCAAGAAATGC	minD	septum site-determining protein MinD	WP_011294332.:	363283..364098	Cell Division	COG2894
WP_011294332.1_372	CUST_2038_PI442190932	NATL2	LLI	WP_011294332.1_372	ACAAGGAGAAGATATTCCACTTATTGATCCAGCAGAGGAGATTTCTGGATTTGGTGCGAA	minD	septum site-determining protein MinD	WP_011294332.:	363283..364098	Cell Division	COG2894
WP_011294333.1_373	CUST_2039_PI442190932	NATL2	LLI	WP_011294333.1_373	TGGGGAAGATTACTTGGGATTGCTCATGCAGGCAACAAAGGAAACTCTAAAGCAAAAATA	minC	septum site-determining protein MinC	WP_011294333.:	364262..364906	Cell Division	COG0850
WP_011294333.1_373	CUST_2040_PI442190932	NATL2	LLI	WP_011294333.1_373	TAAAGTTGCTAGAGGTCCTAAGGAGAAGCCTCAATTTGGTCTTGCAGAACAAGCAAGAAT	minC	septum site-determining protein MinC	WP_011294333.:	364262..364906	Cell Division	COG0850
WP_011294333.1_373	CUST_2041_PI442190932	NATL2	LLI	WP_011294333.1_373	ATTGAAAGTCCTGGGGATTTGTTAATCCTTGGAGATGTTAATCCAGGGGCAAATGTTAGC	minC	septum site-determining protein MinC	WP_011294333.:	364262..364906	Cell Division	COG0850
WP_011294333.1_373	CUST_2042_PI442190932	NATL2	LLI	WP_011294333.1_373	ACAAGAGAAATCCAACTTTTTAAAAACTCACTTTCATCAAGGAACTGTCAGGTCAGGAG	minC	septum site-determining protein MinC	WP_011294333.:	364262..364906	Cell Division	COG0850
WP_011294333.1_373	CUST_2043_PI442190932	NATL2	LLI	WP_011294333.1_373	AGATTATTTGTTTTTGCTCATCATCTCCAAAGACGATTGTAAGCTCTCAATCACTAGGTT	minC	septum site-determining protein MinC	WP_011294333.:	364262..364906	Cell Division	COG0850
WP_011294333.1_373	CUST_2044_PI442190932	NATL2	LLI	WP_011294333.1_373	TGCTGAAAATATCGATCTTTCATGTAAAGAGATATCAGAAATCATTGAAATTGCGAATCA	minC	septum site-determining protein MinC	WP_011294333.:	364262..364906	Cell Division	COG0850
WP_011294408.1_454	CUST_2463_PI442190932	NATL2	LLI	WP_011294408.1_454	AGAAAAAAACGAATCAAAGCTCTGCCAAAAATGGCTCCTCATAACAGGTGTATTGGGAGT	cyoC	heme-copper oxidase subunit III	WP_011294408.:	440981..441586	Respiration	COG1845
WP_011294408.1_454	CUST_2464_PI442190932	NATL2	LLI	WP_011294408.1_454	TGCGAGTACTTTTTATGCATTAACTGGCTTTCATGGGCTTCACGTAACACTTGGTTCAAT	cyoC	heme-copper oxidase subunit III	WP_011294408.:	440981..441586	Respiration	COG1845
WP_011294408.1_454	CUST_2465_PI442190932	NATL2	LLI	WP_011294408.1_454	GGTTTTTTCGCGGCATATCTCACTTTCAAAGCAGTCAATCCTATTGGTCCTGATGCTATT	cyoC	heme-copper oxidase subunit III	WP_011294408.:	440981..441586	Respiration	COG1845
WP_011294408.1_454	CUST_2466_PI442190932	NATL2	LLI	WP_011294408.1_454	GCCGAGTGAATAATCAAAACAAGTTTCCATTTGAGGCTGTAGAGCTCTATTGGCACTTCG	cyoC	heme-copper oxidase subunit III	WP_011294408.:	440981..441586	Respiration	COG1845
WP_011294408.1_454	CUST_2467_PI442190932	NATL2	LLI	WP_011294408.1_454	AGTTCCAAAAGAGCAATCCTCAGGAAGCAAGAATGAGCTATCAACTGAAGAACATGAAGA	cyoC	heme-copper oxidase subunit III	WP_011294408.:	440981..441586	Respiration	COG1845
WP_011294408.1_454	CUST_2468_PI442190932	NATL2	LLI	WP_011294408.1_454	ACCAACTTTAAATACAATCTTGCTCTTAGTTAGTAGCTTTACTTTTCATCGGGCTGGGAA	cyoC	heme-copper oxidase subunit III	WP_011294408.:	440981..441586	Respiration	COG1845
WP_011294409.1_455	CUST_2469_PI442190932	NATL2	LLI	WP_011294409.1_455	GGGACGGAGAAGCCCCACTCGTTCTTGAACCTTATGGTTATGGAAAAAAGATTCAAATG	cyoB/coxA	Cytochrome c oxidase subunit I	WP_011294409.:	441583..443223	Respiration	COG0843
WP_011294409.1_455	CUST_2470_PI442190932	NATL2	LLI	WP_011294409.1_455	TTAATCTTTGTTTTGCTCCTCAACATTGGCTAGGTTTAAACGGAATGCCTCGACGAGTTG	cyoB/coxA	Cytochrome c oxidase subunit I	WP_011294409.:	441583..443223	Respiration	COG0843
WP_011294409.1_455	CUST_2471_PI442190932	NATL2	LLI	WP_011294409.1_455	GGGAGTTGCACTAGCGCAAGTACCTTTTGATGTTCATGTACACGACACTTATTTTGTTGT	cyoB/coxA	Cytochrome c oxidase subunit I	WP_011294409.:	441583..443223	Respiration	COG0843
WP_011294409.1_455	CUST_2472_PI442190932	NATL2	LLI	WP_011294409.1_455	AGCTGTTCCCACAGGTATTAAATTTTTTAATTGGGTTGCGACCTTATGGGGAGGGAAAAT	cyoB/coxA	Cytochrome c oxidase subunit I	WP_011294409.:	441583..443223	Respiration	COG0843
WP_011294409.1_455	CUST_2473_PI442190932	NATL2	LLI	WP_011294409.1_455	GTGTTAGGTCTAGTTGTTTGGGCTCATCATATGTTTACAAGCGGTACTCCTCCTTGGATG	cyoB/coxA	Cytochrome c oxidase subunit I	WP_011294409.:	441583..443223	Respiration	COG0843
WP_011294409.1_455	CUST_2474_PI442190932	NATL2	LLI	WP_011294409.1_455	TGGTTTTACTCGCATCCTGCTGTTTATATCATGGTCTTACCTGCCTTTGGTTTAGTCAGT	cyoB/coxA	Cytochrome c oxidase subunit I	WP_011294409.:	441583..443223	Respiration	COG0843
WP_011294410.1_456	CUST_2475_PI442190932	NATL2	LLI	WP_011294410.1_456	GTCTTATCCAATTTCGTCGTAGACCTGGAGAAACTGGAGATGGCGTTAACTTAGAGGGTA	ctaC/coxB	Cytochrome c oxidase subunit II	WP_011294410.:	443228..444040	Respiration	COG1622
WP_011294410.1_456	CUST_2476_PI442190932	NATL2	LLI	WP_011294410.1_456	GCTACGATATTTATGATCGAATGGGTGGTATGCAACCTTTAATGCATGATCACAGTGGGC	ctaC/coxB	Cytochrome c oxidase subunit II	WP_011294410.:	443228..444040	Respiration	COG1622
WP_011294410.1_456	CUST_2477_PI442190932	NATL2	LLI	WP_011294410.1_456	TAATTTCCCAATTGTTTGCGCAGAATTGTGTGGTCCTTATCATGGTGGGATGAGATCAAA	ctaC/coxB	Cytochrome c oxidase subunit II	WP_011294410.:	443228..444040	Respiration	COG1622
WP_011294410.1_456	CUST_2478_PI442190932	NATL2	LLI	WP_011294410.1_456	CATGAATGCTCCTGTTTACGATGAACTCTATCGAGTCCTGTTCATTATTGGCGCCATACT	ctaC/coxB	Cytochrome c oxidase subunit II	WP_011294410.:	443228..444040	Respiration	COG1622
WP_011294410.1_456	CUST_2479_PI442190932	NATL2	LLI	WP_011294410.1_456	CGGCCATCTTAACTCTTACAACAAGTTTAATTCTTGGAATAGCTGGAGTTTGGGTTGCGT	ctaC/coxB	Cytochrome c oxidase subunit II	WP_011294410.:	443228..444040	Respiration	COG1622
WP_011294410.1_456	CUST_2480_PI442190932	NATL2	LLI	WP_011294410.1_456	TGTAATACATGCTTTTTGGGTGCCTCAATTCAGACTTAAACAAGATGTCATTCCTGGCCA	ctaC/coxB	Cytochrome c oxidase subunit II	WP_011294410.:	443228..444040	Respiration	COG1622
WP_011294418.1_463	CUST_2517_PI442190932	NATL2	LLI	WP_011294418.1_463	TTTTTAGCAGCGGATCCTTCGGCTTCTTACATAACAGGTCAGGTAATTCAAGTTGATGGA	gdh	3-oxoacyl-acyl-carrier-protein reductase	WP_011294418.:	450705..451457	Entner-Dudoroff	COG1028
WP_011294418.1_463	CUST_2518_PI442190932	NATL2	LLI	WP_011294418.1_463	AGAATTTGCCAGCAGAGGAATTACTGTAAATGCAGTTGCCCCTGGTTTTATTTCAACTGA	gdh	3-oxoacyl-acyl-carrier-protein reductase	WP_011294418.:	450705..451457	Entner-Dudoroff	COG1028
WP_011294418.1_463	CUST_2519_PI442190932	NATL2	LLI	WP_011294418.1_463	TTGTAACTGGCGCAAGCAGAGGTATTGGTAAAGCTATTGCAATTTTTCTAGCGAAGGAAG	gdh	3-oxoacyl-acyl-carrier-protein reductase	WP_011294418.:	450705..451457	Entner-Dudoroff	COG1028
WP_011294418.1_463	CUST_2520_PI442190932	NATL2	LLI	WP_011294418.1_463	TCTTAACTTGAGTGGTGTTTTTTATTGCACAAGAGCGGTATCAAGGCAGATGTTGAAGCA	gdh	3-oxoacyl-acyl-carrier-protein reductase	WP_011294418.:	450705..451457	Entner-Dudoroff	COG1028
WP_011294418.1_463	CUST_2521_PI442190932	NATL2	LLI	WP_011294418.1_463	ATGACAAAAGATCTGAATAGTGAATCAATCCTTTCTGCTATCCCGCTTGGACGATTCGGC	gdh	3-oxoacyl-acyl-carrier-protein reductase	WP_011294418.:	450705..451457	Entner-Dudoroff	COG1028
WP_011294418.1_463	CUST_2522_PI442190932	NATL2	LLI	WP_011294418.1_463	AATTATCAACATAACTTCTGTCGTTGGGTTGATGGGCAACCCAGGGCAAGCAAATTATTC	gdh	3-oxoacyl-acyl-carrier-protein reductase	WP_011294418.:	450705..451457	Entner-Dudoroff	COG1028
WP_011294483.1_529	CUST_2883_PI442190932	NATL2	LLI	WP_011294483.1_529	GGCCGATGTGGTTAAGGAAGCTTTGGATGAACTGTGTGTTGTCTTGGTAAAGAGATTTT	tal	Transaldolase	WP_011294483.:	509733..510731	Pentose Phosphate	COG0176
WP_011294483.1_529	CUST_2884_PI442190932	NATL2	LLI	WP_011294483.1_529	CCGTGTTTCAACAGAGGTTGATGCAAGACTAAGCTTTGATACTGATGCGACCATTGAAAA	tal	Transaldolase	WP_011294483.:	509733..510731	Pentose Phosphate	COG0176
WP_011294483.1_529	CUST_2885_PI442190932	NATL2	LLI	WP_011294483.1_529	GGTCATGGGAGCAAGTTTCAGAAATATAGAGGAAATAACCGAGCTTGCAGGATGTGATCT	tal	Transaldolase	WP_011294483.:	509733..510731	Pentose Phosphate	COG0176
WP_011294483.1_529	CUST_2886_PI442190932	NATL2	LLI	WP_011294483.1_529	CTTGTGCTGAGGCAGGTGTAACGCTTATTTCACCCTTTGTTGGAAGAATTTTAGATTGGT	tal	Transaldolase	WP_011294483.:	509733..510731	Pentose Phosphate	COG0176
WP_011294483.1_529	CUST_2887_PI442190932	NATL2	LLI	WP_011294483.1_529	GAACTAATGATGGCTGGAGATAAAATGGCTACGGAAAAACTTGACGATGGAATTAGCGGT	tal	Transaldolase	WP_011294483.:	509733..510731	Pentose Phosphate	COG0176
WP_011294483.1_529	CUST_2888_PI442190932	NATL2	LLI	WP_011294483.1_529	GGAAGGGATTCTTATCCAGCGACTGAGGACCCAGGGGTAGTTTCGGTTACTAAAATATTT	tal	Transaldolase	WP_011294483.:	509733..510731	Pentose Phosphate	COG0176
WP_011293566.1_604	CUST_3300_PI442190932	NATL2	LLI	WP_011293566.1_604	GTGACAAATCAGATTCCACAGATTTACCCGCACTTGTTTGGGAAAAAGCAAAAAACGGAG	glk	Glucokinase	WP_011293566.:	584817..585842	Glycolysis	COG0837
WP_011293566.1_604	CUST_3301_PI442190932	NATL2	LLI	WP_011293566.1_604	ATTGTTAGTGGTACTGGCCTTGGCATGATTGCCAGATGGAAATTGGATGATCCAATAAAT	glk	Glucokinase	WP_011293566.:	584817..585842	Glycolysis	COG0837
WP_011293566.1_604	CUST_3302_PI442190932	NATL2	LLI	WP_011293566.1_604	CATTGGTTGTTCTTAAAGATAAAGAAGCGACATTATTCAGCTCAGCTTGCAGAGCACGCT	glk	Glucokinase	WP_011293566.:	584817..585842	Glycolysis	COG0837
WP_011293566.1_604	CUST_3303_PI442190932	NATL2	LLI	WP_011293566.1_604	GAATGTCCAGAGGCTTGATAACCCCTAAAAGCATCTCTATATTTCCAAGTGAAGGAGGGC	glk	Glucokinase	WP_011293566.:	584817..585842	Glycolysis	COG0837
WP_011293566.1_604	CUST_3304_PI442190932	NATL2	LLI	WP_011293566.1_604	ATGGATAGCAGGTGGAACAGCAGCAAAAAACCTCGATGGAATAAACTCTTCTAACTTCCT	glk	Glucokinase	WP_011293566.:	584817..585842	Glycolysis	COG0837
WP_011293566.1_604	CUST_3305_PI442190932	NATL2	LLI	WP_011293566.1_604	CAACTATGGCTAAATGCTTATGGGTCTGCAGCTGGAGACCTTGCTTTACAAGAACTTTG	glk	Glucokinase	WP_011293566.:	584817..585842	Glycolysis	COG0837
WP_011293596.1_634	CUST_3477_PI442190932	NATL2	LLI	WP_011293596.1_634	TAGGTAATCAATTTCTTAGTGCAGGACCTGGGTTTGGTGGGAGCTGCTTTAAAAAGGATA	ugd	UDP-glucose 6-dehydrogenase/nucleotide sugar dehydrogenase	WP_011293596.:	617562..618965	Amino sugar and nucleotide sugar r	COG1004
WP_011293596.1_634	CUST_3478_PI442190932	NATL2	LLI	WP_011293596.1_634	TTTAAGGCAAACACCAACGATACCCGAGAATCACCTGCGATAAGAATTTCCAGTGACTTA	ugd	UDP-glucose 6-dehydrogenase/nucleotide sugar dehydrogenase	WP_011293596.:	617562..618965	Amino sugar and nucleotide sugar r	COG1004
WP_011293596.1_634	CUST_3479_PI442190932	NATL2	LLI	WP_011293596.1_634	TGGATTACCAGAGGTTGCTAATTACTGGAATCAAGTTGTCGTGCTCAATACTTGGCAACA	ugd	UDP-glucose 6-dehydrogenase/nucleotide sugar dehydrogenase	WP_011293596.:	617562..618965	Amino sugar and nucleotide sugar r	COG1004
WP_011293596.1_634	CUST_3480_PI442190932	NATL2	LLI	WP_011293596.1_634	TGTTAATCGACAAGAAGTTGAAGATACAGGTGTAAATCTTTGGAAGCTGGGTGAGGGGAA	ugd	UDP-glucose 6-dehydrogenase/nucleotide sugar dehydrogenase	WP_011293596.:	617562..618965	Amino sugar and nucleotide sugar r	COG1004
WP_011293596.1_634	CUST_3481_PI442190932	NATL2	LLI	WP_011293596.1_634	GTGAGGCTACTGGAGCTGATGTTCAGGAAGTTGCTAAAGCAATAGGAACAGATAAAAGAA	ugd	UDP-glucose 6-dehydrogenase/nucleotide sugar dehydrogenase	WP_011293596.:	617562..618965	Amino sugar and nucleotide sugar r	COG1004
WP_011293596.1_634	CUST_3482_PI442190932	NATL2	LLI	WP_011293596.1_634	TCAAGGAATTTGGAAAATGGCCAGTTCTATTCCAGAAGCATTGAAAAATGTTGATGCAGT	ugd	UDP-glucose 6-dehydrogenase/nucleotide sugar dehydrogenase	WP_011293596.:	617562..618965	Amino sugar and nucleotide sugar r	COG1004
WP_011293675.1_716	CUST_3911_PI442190932	NATL2	LLI	WP_011293675.1_716	AAGTTCAACAGCTAAGACAAAGGCCTCATCCACATGAATTTACTGTGTACTACGACGCTT	glnA	Glutamine synthetase/ glutamate--ammonialigase	WP_011293675.:	692772..694193	Nitrogen Metabolism	COG0174
WP_011293675.1_716	CUST_3912_PI442190932	NATL2	LLI	WP_011293675.1_716	TTTAGTTTACTCACAGGGCAATCGATCAGCTGCTGTAAGAATACCTTTGACTGGACCAAG	glnA	Glutamine synthetase/ glutamate--ammonialigase	WP_011293675.:	692772..694193	Nitrogen Metabolism	COG0174
WP_011293675.1_716	CUST_3913_PI442190932	NATL2	LLI	WP_011293675.1_716	CAGTCTTCAATGACAATGGGACAGGAATGCATGTTCATCAAAGTCTTTGGAAAGGAGGTC	glnA	Glutamine synthetase/ glutamate--ammonialigase	WP_011293675.:	692772..694193	Nitrogen Metabolism	COG0174
WP_011293675.1_716	CUST_3914_PI442190932	NATL2	LLI	WP_011293675.1_716	CCTGGTCAACATGAATTAGGAATGAAATTTGCTCCCCTAATCAACGCAGCTGACAATGTA	glnA	Glutamine synthetase/ glutamate--ammonialigase	WP_011293675.:	692772..694193	Nitrogen Metabolism	COG0174
WP_011293675.1_716	CUST_3915_PI442190932	NATL2	LLI	WP_011293675.1_716	ATCAAATTGATCCTGGAGATGGAGTTGACGTTGACCTTTTTGAGCTTCCTTCAGAGGAAT	glnA	Glutamine synthetase/ glutamate--ammonialigase	WP_011293675.:	692772..694193	Nitrogen Metabolism	COG0174
WP_011293675.1_716	CUST_3916_PI442190932	NATL2	LLI	WP_011293675.1_716	CGCATTAGCTAACCCTTACATTGCTTTTTCAGCAATGATGATGGCAGGTATCGATGGAAT	glnA	Glutamine synthetase/ glutamate--ammonialigase	WP_011293675.:	692772..694193	Nitrogen Metabolism	COG0174
WP_011293709.1_749	CUST_4104_PI442190932	NATL2	LLI	WP_011293709.1_749	ATTGGTGCATTCTTTGAATCAAATTTAGCCTTAACTCAGCAACCCACCCCTCCATTTAGT	glgC	Glucose-1-phosphate adenylyltransferase	WP_011293709.:	731382..732677	Glycogen Metabolism	COG0448
WP_011293709.1_749	CUST_4105_PI442190932	NATL2	LLI	WP_011293709.1_749	TTCATCATTGTGTGTTGGGAGTTAGGAGTCGGATTGAAAGCGACGTTGTTCTAAATGAGA	glgC	Glucose-1-phosphate adenylyltransferase	WP_011293709.:	731382..732677	Glycogen Metabolism	COG0448
WP_011293709.1_749	CUST_4106_PI442190932	NATL2	LLI	WP_011293709.1_749	TTTTTACGAATCTTATGAAGAGAGAATAGCGCTAAGAAATGGCGGTGGTATCCCCTTGGG	glgC	Glucose-1-phosphate adenylyltransferase	WP_011293709.:	731382..732677	Glycogen Metabolism	COG0448
WP_011293709.1_749	CUST_4107_PI442190932	NATL2	LLI	WP_011293709.1_749	GAGCCATTCTCGATAAAAATGCTCGCATTGGTGACAATGTGACGATAGTCAACAAAGACA	glgC	Glucose-1-phosphate adenylyltransferase	WP_011293709.:	731382..732677	Glycogen Metabolism	COG0448
WP_011293709.1_749	CUST_4108_PI442190932	NATL2	LLI	WP_011293709.1_749	ACCTACTGGTGATTCATTGAAAGCAATGGCTGTAGATACATCAAGGTTTGGATTGGAAGC	glgC	Glucose-1-phosphate adenylyltransferase	WP_011293709.:	731382..732677	Glycogen Metabolism	COG0448
WP_011293709.1_749	CUST_4109_PI442190932	NATL2	LLI	WP_011293709.1_749	GGAAGAAGCTGATCGCGCAGATCAAGGTTTTTATATTCGTAATGGAATAGTTGTTATCGT	glgC	Glucose-1-phosphate adenylyltransferase	WP_011293709.:	731382..732677	Glycogen Metabolism	COG0448
WP_011293710.1_750	CUST_4110_PI442190932	NATL2	LLI	WP_011293710.1_750	AATGGGAACAAACCTTCTTTTGTTGATTTTGGAAAGCCTTCAGATGGTATGCCTCTCCTT	edd	phosphogluconate dehydrogenase (NADP(+)-dependent, decarboxyla	WP_011293710.:	732836..734254	Pentose Phosphate/Entner-Dudorof	COG2084
WP_011293710.1_750	CUST_4111_PI442190932	NATL2	LLI	WP_011293710.1_750	ATAAAGCGGGTCAAAAAGGTACTGGATTATGGACAGTCGTTAGTGCATTAGAGCTTGGAG	edd	phosphogluconate dehydrogenase (NADP(+)-dependent, decarboxyla	WP_011293710.:	732836..734254	Pentose Phosphate/Entner-Dudorof	COG2084
WP_011293710.1_750	CUST_4112_PI442190932	NATL2	LLI	WP_011293710.1_750	GAGAGACTGTTTTGGCTCTCATACATATGAGCGCGTAGACAAGGCTGGATCATTTCATAC	edd	phosphogluconate dehydrogenase (NADP(+)-dependent, decarboxyla	WP_011293710.:	732836..734254	Pentose Phosphate/Entner-Dudorof	COG2084
WP_011293710.1_750	CUST_4113_PI442190932	NATL2	LLI	WP_011293710.1_750	TGTATTGGCTACGATAGCTAGCTATGCCCAAGGTATGGATATTTTACGACTCGCTTCTGA	edd	phosphogluconate dehydrogenase (NADP(+)-dependent, decarboxyla	WP_011293710.:	732836..734254	Pentose Phosphate/Entner-Dudorof	COG2084
WP_011293710.1_750	CUST_4114_PI442190932	NATL2	LLI	WP_011293710.1_750	ATTATTGATAAATGGTTTACCGATCAGGTAAACAATCGTCTCTCGGGCCTGACACAGGTC	edd	phosphogluconate dehydrogenase (NADP(+)-dependent, decarboxyla	WP_011293710.:	732836..734254	Pentose Phosphate/Entner-Dudorof	COG2084
WP_011293710.1_750	CUST_4115_PI442190932	NATL2	LLI	WP_011293710.1_750	CCTCCATCGCTCAAATATGGAAAGGTGGTTGCATAATAAGGTCTACCCTTTTGAGTCGTA	edd	phosphogluconate dehydrogenase (NADP(+)-dependent, decarboxyla	WP_011293710.:	732836..734254	Pentose Phosphate/Entner-Dudorof	COG2084
WP_011293722.1_762	CUST_4174_PI442190932	NATL2	LLI	WP_011293722.1_762	AATACGTACAATGGAACGATGCGTACCAGCAAGTGTACCTGGTATTACTTTCCTTTCAGG	fda	Fructose-1,6-bisphosphate aldolase class I	WP_011293722.:	741708..742775	Glycolysis/Gluconeogenesis	COG0191
WP_011293722.1_762	CUST_4175_PI442190932	NATL2	LLI	WP_011293722.1_762	TGATGTTGAGACCTTCTGCTCAGGATTAGATGGACTTGTTGAAAGAGCTGCTGACTACTA	fda	Fructose-1,6-bisphosphate aldolase class I	WP_011293722.:	741708..742775	Glycolysis/Gluconeogenesis	COG0191
WP_011293722.1_762	CUST_4176_PI442190932	NATL2	LLI	WP_011293722.1_762	TTGAACCAGAAATCCTAATGGACGGTTCACATTCAATTGAAAAGACTGCAGCAGTCCAAG	fda	Fructose-1,6-bisphosphate aldolase class I	WP_011293722.:	741708..742775	Glycolysis/Gluconeogenesis	COG0191
WP_011293722.1_762	CUST_4177_PI442190932	NATL2	LLI	WP_011293722.1_762	TCTGAGGCATCAAAAGGATTGTATGTTGCAGGTTCTCAGCCTTCTTCTGATGAACAGTTA	fda	Fructose-1,6-bisphosphate aldolase class I	WP_011293722.:	741708..742775	Glycolysis/Gluconeogenesis	COG0191
WP_011293722.1_762	CUST_4178_PI442190932	NATL2	LLI	WP_011293722.1_762	AAAGTTAGGCATTATCCCAGGAATCAAGGTTGATAAAGGTCTAAGACCATTAGCCGGTGG	fda	Fructose-1,6-bisphosphate aldolase class I	WP_011293722.:	741708..742775	Glycolysis/Gluconeogenesis	COG0191
WP_011293722.1_762	CUST_4179_PI442190932	NATL2	LLI	WP_011293722.1_762	AGCAAGGTGCAAGATTCGCAAAATGGAGAGCAGTACTTCAAATAACAGATGATGGTTGTC	fda	Fructose-1,6-bisphosphate aldolase class I	WP_011293722.:	741708..742775	Glycolysis/Gluconeogenesis	COG0191
WP_011293727.1_767	CUST_4202_PI442190932	NATL2	LLI	WP_011293727.1_767	AAAATTAGATCCAGGTCAATTCACTCCCTGGGAAGATATTCCAACAGGAACCGACCTTTT	prk	Phosphoribulokinase	WP_011293727.:	745919..746821	Carbon Fixation	COG3954
WP_011293727.1_767	CUST_4203_PI442190932	NATL2	LLI	WP_011293727.1_767	GGGGTCAGAAAAGATATTATTTACATAGCCCAGAAGAAGCCGAGGAACACAACACTCGTT	prk	Phosphoribulokinase	WP_011293727.:	745919..746821	Carbon Fixation	COG3954
WP_011293727.1_767	CUST_4204_PI442190932	NATL2	LLI	WP_011293727.1_767	ATGTGGCCTCATTCGCTGATTTACTTGTTGGTGTAGTACCAATTACCAATCTCGAATGGA	prk	Phosphoribulokinase	WP_011293727.:	745919..746821	Carbon Fixation	COG3954
WP_011293727.1_767	CUST_4205_PI442190932	NATL2	LLI	WP_011293727.1_767	AAAAGGCAATGTCTGAGGCACTTTCAAGAGGTGAAAACTTTTCTCATTTTGGACCAGAGG	prk	Phosphoribulokinase	WP_011293727.:	745919..746821	Carbon Fixation	COG3954
WP_011293727.1_767	CUST_4206_PI442190932	NATL2	LLI	WP_011293727.1_767	TAACTTCCAGAGAGTACCGACCATTGATACGTCCAACCCATTTATCTGCAGAAACATTCC	prk	Phosphoribulokinase	WP_011293727.:	745919..746821	Carbon Fixation	COG3954
WP_011293727.1_767	CUST_4207_PI442190932	NATL2	LLI	WP_011293727.1_767	TAACGCAGAAAGGGGATACTCAGCCGAAACTATCGTAGATACGATTCTGAGAAGAATGCC	prk	Phosphoribulokinase	WP_011293727.:	745919..746821	Carbon Fixation	COG3954
WP_011293755.1_794	CUST_4356_PI442190932	NATL2	LLI	WP_011293755.1_794	CGCATCCATCAGGATTGATTGGTGAAGATCCCATTGGGATTCCCAATAATCTTTTTCCTT	galE	UDP-glucose 4-epimerase	WP_011293755.:	770645..771688	Galactose metabolism	COG1087
WP_011293755.1_794	CUST_4357_PI442190932	NATL2	LLI	WP_011293755.1_794	CCACTATTTATGGTTTAAGCGATTCCGTTCCTATGGCAGAAGATCACAGGATCTCACCAA	galE	UDP-glucose 4-epimerase	WP_011293755.:	770645..771688	Galactose metabolism	COG1087
WP_011293755.1_794	CUST_4358_PI442190932	NATL2	LLI	WP_011293755.1_794	TGGCTCAGGTATCCGTGATTATATTCATATTATGGATTTAGCTGAGGGCCATTTAGCCTC	galE	UDP-glucose 4-epimerase	WP_011293755.:	770645..771688	Galactose metabolism	COG1087
WP_011293755.1_794	CUST_4359_PI442190932	NATL2	LLI	WP_011293755.1_794	TTGACTACTGGTTGCCAAATTCCTTTTTCTATTCAAAACAGAAGAGATGGTGATGTCGCT	galE	UDP-glucose 4-epimerase	WP_011293755.:	770645..771688	Galactose metabolism	COG1087
WP_011293755.1_794	CUST_4360_PI442190932	NATL2	LLI	WP_011293755.1_794	AGGTTGGACTCCAAAAAGAACATTAGAACAAATTTGCTTAGATGGATGGAACTGGCAAAT	galE	UDP-glucose 4-epimerase	WP_011293755.:	770645..771688	Galactose metabolism	COG1087
WP_011293755.1_794	CUST_4361_PI442190932	NATL2	LLI	WP_011293755.1_794	AGCTGGCTTGAAATCTGTAGCTGAATCTCTTACTAATCCTATTCACTATTGGGATGTGAA	galE	UDP-glucose 4-epimerase	WP_011293755.:	770645..771688	Galactose metabolism	COG1087
WP_011293815.1_852	CUST_4670_PI442190932	NATL2	LLI	WP_011293815.1_852	CAATTAGGCGCTGAACACAGTCAAAGACTTGAAGCCTATTTTCATGCAGTTCCTGGTATC	pdhB	Pyruvate dehydrogenase E1 component beta subunit	WP_011293815.:	822023..823012	Glycolysis/Krebs cycle	COG0022
WP_011293815.1_852	CUST_4671_PI442190932	NATL2	LLI	WP_011293815.1_852	TTTGACTTATTCGAGAATGCGTCACCACTGTTTAAAAGCAGTCGAACTTCTCGAGGCTAA	pdhB	Pyruvate dehydrogenase E1 component beta subunit	WP_011293815.:	822023..823012	Glycolysis/Krebs cycle	COG0022
WP_011293815.1_852	CUST_4672_PI442190932	NATL2	LLI	WP_011293815.1_852	TGGGAATGCTTAGATATACGAGCGGGGGAAATTTTACTATTCCAACAGTTGTAAGAGGCC	pdhB	Pyruvate dehydrogenase E1 component beta subunit	WP_011293815.:	822023..823012	Glycolysis/Krebs cycle	COG0022
WP_011293815.1_852	CUST_4673_PI442190932	NATL2	LLI	WP_011293815.1_852	GTTCTTCTATATAACCTCACAGAAGAGCTGCCTGAGGGTGATTATGTCTGTGCCCTAGAT	pdhB	Pyruvate dehydrogenase E1 component beta subunit	WP_011293815.:	822023..823012	Glycolysis/Krebs cycle	COG0022
WP_011293815.1_852	CUST_4674_PI442190932	NATL2	LLI	WP_011293815.1_852	TGCTTCGATGATTTAGATTCTCCTCCTGTTCGTTTAAGCAGTCAGGATATTCCGACTCCT	pdhB	Pyruvate dehydrogenase E1 component beta subunit	WP_011293815.:	822023..823012	Glycolysis/Krebs cycle	COG0022
WP_011293815.1_852	CUST_4675_PI442190932	NATL2	LLI	WP_011293815.1_852	TGTCGCTTGTAGTACTCCTACAAATGCTAAAGGTCTAATGAAAGCTGCTATAAGGGACAA	pdhB	Pyruvate dehydrogenase E1 component beta subunit	WP_011293815.:	822023..823012	Glycolysis/Krebs cycle	COG0022
WP_011293829.1_866	CUST_4737_PI442190932	NATL2	LLI	WP_011293829.1_866	TTAACAAAAAGTGGTGCTACCGCTAAAAACGTAAGCAAGTTTAGACCTCCCACTCCAGTA	pykF	Pyruvate kinase	WP_011293829.:	834406..835863	Glycolysis	COG0469
WP_011293829.1_866	CUST_4738_PI442190932	NATL2	LLI	WP_011293829.1_866	TAAAATTATGTGATGGAGTCATGGTGGCTAGAGGAGATCTAGGTGTAGAGATGCCAGCAG	pykF	Pyruvate kinase	WP_011293829.:	834406..835863	Glycolysis	COG0469
WP_011293829.1_866	CUST_4739_PI442190932	NATL2	LLI	WP_011293829.1_866	ATGCGATTAGTGCGGCTGTAAGCAGTATTGCTAGACAAATAAACGCTGCAGCAATAATAC	pykF	Pyruvate kinase	WP_011293829.:	834406..835863	Glycolysis	COG0469
WP_011293829.1_866	CUST_4740_PI442190932	NATL2	LLI	WP_011293829.1_866	CATTGAGTCTGTGGCGACTATGGCTACTATTGCGAAAAGAATCGAACGAGATTATCCACA	pykF	Pyruvate kinase	WP_011293829.:	834406..835863	Glycolysis	COG0469
WP_011293829.1_866	CUST_4741_PI442190932	NATL2	LLI	WP_011293829.1_866	TCAAAAAGGCAAACAGCCTTGGCATACCAATTATTACAGCCACACAAATGCTTGATTCAA	pykF	Pyruvate kinase	WP_011293829.:	834406..835863	Glycolysis	COG0469
WP_011293829.1_866	CUST_4742_PI442190932	NATL2	LLI	WP_011293829.1_866	ATGAAAATCCTCAAGCAAGGAGATCTTGTTGTTGAGACAGCTGGAACACTTTCAGGTGTA	pykF	Pyruvate kinase	WP_011293829.:	834406..835863	Glycolysis	COG0469
WP_011293854.1_891	CUST_4876_PI442190932	NATL2	LLI	WP_011293854.1_891	GAATCAAGTCTTGGCGCTTTAATTGCATTGTTTGAGCGAGCTGTAAGTCTCTATGCTGAA	pgi	Glucose-6-phosphate isomerase	WP_011293854.:	858576..860174	Glycolysis/Gluconeogenesis	COG0166
WP_011293854.1_891	CUST_4877_PI442190932	NATL2	LLI	WP_011293854.1_891	AAGACCAGGTGACTATTTGTCTGGATTTCTTCAAGGAACTCGTTCAGCATTGACTGAAGG	pgi	Glucose-6-phosphate isomerase	WP_011293854.:	858576..860174	Glycolysis/Gluconeogenesis	COG0166
WP_011293854.1_891	CUST_4878_PI442190932	NATL2	LLI	WP_011293854.1_891	CCAGCAGCACTTTTGTCCTTAGCTTGGTTTAAATCTGGAAATGGAAAAGGGTTAAGGGAT	pgi	Glucose-6-phosphate isomerase	WP_011293854.:	858576..860174	Glycolysis/Gluconeogenesis	COG0166
WP_011293854.1_891	CUST_4879_PI442190932	NATL2	LLI	WP_011293854.1_891	TGATTAATGTTAATGCCTACAATCAACCGGGAGTTGAAGCAGGTAAAAAAGCGGCTACAA	pgi	Glucose-6-phosphate isomerase	WP_011293854.:	858576..860174	Glycolysis/Gluconeogenesis	COG0166

WP_011293854.1_891	CUST_4880_PI442190932	NATL2	LLI	WP_011293854.1_891	TCAAGGTATCGCAGTTTATGGAAACAAAGGTTCTACAGATCAGCATGCATATGTCCAGCA	pgi	Glucose-6-phosphate isomerase	WP_011293854.:	858576..860174	Glycolysis/Gluconeogenesis	COG0166
WP_011293854.1_891	CUST_4881_PI442190932	NATL2	LLI	WP_011293854.1_891	TGGAAAGCAAAGAACATTAAATGAAATCAATGATGCGTTATCTTCTGATTCAACAGAGTC	pgi	Glucose-6-phosphate isomerase	WP_011293854.:	858576..860174	Glycolysis/Gluconeogenesis	COG0166
WP_011294765.1_1293	CUST_6605_PI442190932	NATL2	LLI	WP_011294765.1_1293	CTTTTTGATGCTGCAGGTGGGTGCCCAACTTCAAACCAATTAATTCTTAGAATTCAACCC	zwf	glucose-6-phosphate dehydrogenase zwf	WP_011294765.:	1171202..1172725	Pentose Phosphate	COG0364
WP_011294765.1_1293	CUST_6606_PI442190932	NATL2	LLI	WP_011294765.1_1293	CCGAAACATATGTAGCTATGAAGCTTTTCATAGATAATTGGAGATGGCAGGGGGTCCCTT	zwf	glucose-6-phosphate dehydrogenase zwf	WP_011294765.:	1171202..1172725	Pentose Phosphate	COG0364
WP_011294765.1_1293	CUST_6607_PI442190932	NATL2	LLI	WP_011294765.1_1293	CTCCTTGGGAATTACCTATTTATCCTTATGAATCAAGGACATGGGGCCGACTGAATCAG	zwf	glucose-6-phosphate dehydrogenase zwf	WP_011294765.:	1171202..1172725	Pentose Phosphate	COG0364
WP_011294765.1_1293	CUST_6608_PI442190932	NATL2	LLI	WP_011294765.1_1293	CCAGAAGCCATAAGGAATGAAAAAGCTAAGGTTCTTCAGGCAGTTAAGCTTGCGAATGAA	zwf	glucose-6-phosphate dehydrogenase zwf	WP_011294765.:	1171202..1172725	Pentose Phosphate	COG0364
WP_011294765.1_1293	CUST_6609_PI442190932	NATL2	LLI	WP_011294765.1_1293	GAGGTCAATACTCAAAGGGAGGTAGCGATGAAGAACCACTCCTAGGTTACAGAGATGAAC	zwf	glucose-6-phosphate dehydrogenase zwf	WP_011294765.:	1171202..1172725	Pentose Phosphate	COG0364
WP_011294765.1_1293	CUST_6610_PI442190932	NATL2	LLI	WP_011294765.1_1293	CTATGATGAATCCTTTGGAGAGCCATCAGATGAAGGTTATGTGAGACTCCTTGCTGATGC	zwf	glucose-6-phosphate dehydrogenase zwf	WP_011294765.:	1171202..1172725	Pentose Phosphate	COG0364
WP_011294766.1_1294	CUST_6611_PI442190932	NATL2	LLI	WP_011294766.1_1294	TACTTTCTAGTAGTAGAGGCAATACGAGTCCTTTGCTTGAGAGTGCGACACCAATAGCAA	opcA	Glucose-6-P dehydrogenase subunit opcA	WP_011294766.:	1172764..1174071	Pentose Phosphate	COG3429
WP_011294766.1_1294	CUST_6612_PI442190932	NATL2	LLI	WP_011294766.1_1294	ATTGCTTCAAAGAATCCAATCTGGACAAGCTGTTAACGACCTAAATTGGCTTCGCCTTAG	opcA	Glucose-6-P dehydrogenase subunit opcA	WP_011294766.:	1172764..1174071	Pentose Phosphate	COG3429
WP_011294766.1_1294	CUST_6613_PI442190932	NATL2	LLI	WP_011294766.1_1294	GAGATTAGAAGCGGGAGGGATGGCAAGAATGGAACTTATTGAACAAGTCGTTCCTATTCA	opcA	Glucose-6-P dehydrogenase subunit opcA	WP_011294766.:	1172764..1174071	Pentose Phosphate	COG3429
WP_011294766.1_1294	CUST_6614_PI442190932	NATL2	LLI	WP_011294766.1_1294	TATCAATGATGGGCTTGAAATTGTAGAGAATCTTGTTCCCGAGGAACTTCCATCTTGGCT	opcA	Glucose-6-P dehydrogenase subunit opcA	WP_011294766.:	1172764..1174071	Pentose Phosphate	COG3429
WP_011294766.1_1294	CUST_6615_PI442190932	NATL2	LLI	WP_011294766.1_1294	CTTGCTGCATGGATTGCCGATCGTCTCAACTGGAAACTAGAAAGTAGTATCTCCTCAAAG	opcA	Glucose-6-P dehydrogenase subunit opcA	WP_011294766.:	1172764..1174071	Pentose Phosphate	COG3429
WP_011294766.1_1294	CUST_6616_PI442190932	NATL2	LLI	WP_011294766.1_1294	AGGTTGGAGAGAAACCTTAGCGATGGTATTTGATCCTCTTCAAAGAAGAAATGCCCTTGA	opcA	Glucose-6-P dehydrogenase subunit opcA	WP_011294766.:	1172764..1174071	Pentose Phosphate	COG3429
WP_011294798.1_1326	CUST_6788_PI442190932	NATL2	LLI	WP_011294798.1_1326	TAGCTTGGCGTGAGAAACCAACTAGAAAAGGGATTAGCCTAATCAGCAAAGATGGAGAAA	rbsK	Sugar kinase, ribokinase family	WP_011294798.:	1205730..1206572	Pentose Phosphate	COG0524
WP_011294798.1_1326	CUST_6789_PI442190932	NATL2	LLI	WP_011294798.1_1326	CCCTCTCAGCAAAGCTAAATTTAGAGCAAGCGATTAATATAGGTGCATATTGCGGTGCTG	rbsK	Sugar kinase, ribokinase family	WP_011294798.:	1205730..1206572	Pentose Phosphate	COG0524
WP_011294798.1_1326	CUST_6790_PI442190932	NATL2	LLI	WP_011294798.1_1326	TGCGACTCCCCGAACAGGTCAACAAACCCTAAAGAATTCAAAAGTAAAACTCAATGCATT	rbsK	Sugar kinase, ribokinase family	WP_011294798.:	1205730..1206572	Pentose Phosphate	COG0524
WP_011294798.1_1326	CUST_6791_PI442190932	NATL2	LLI	WP_011294798.1_1326	TTAGCGGTGATCGGTCATGTTGAATGGGTAACATTCTTAAAGGTTGATCAGCTCCCATTA	rbsK	Sugar kinase, ribokinase family	WP_011294798.:	1205730..1206572	Pentose Phosphate	COG0524
WP_011294798.1_1326	CUST_6792_PI442190932	NATL2	LLI	WP_011294798.1_1326	CAGCTGTTCAGATGGCAAGGCTAATAAATAACCCTGTTGACTTAATCACATCATTGGGCA	rbsK	Sugar kinase, ribokinase family	WP_011294798.:	1205730..1206572	Pentose Phosphate	COG0524
WP_011294798.1_1326	CUST_6793_PI442190932	NATL2	LLI	WP_011294798.1_1326	AAACCCATTACTCCTAGTTCTGAAGAAATAGATACCTATGGTTGTGGGGACAGTTTCGCG	rbsK	Sugar kinase, ribokinase family	WP_011294798.:	1205730..1206572	Pentose Phosphate	COG0524
WP_041711031.1_1475	CUST_7563_PI442190932	NATL2	LLI	WP_041711031.1_1475	AATTTGCTTTAAACGCCCCAGAGCCTGACCCTAGTGAGTTAACAAAATATATTTGGGCAG	pdhA	Pyruvate dehydrogenase E1 component alpha subunit	WP_041711031.:	1358583..1359671	Glycolysis/Krebs cycle	COG1071
WP_041711031.1_1475	CUST_7564_PI442190932	NATL2	LLI	WP_041711031.1_1475	ACTAGTGAGACAGAAATATGGAGAAAAGCTTCAGCATTTGGTATGCCAGGCGAAGAGATT	pdhA	Pyruvate dehydrogenase E1 component alpha subunit	WP_041711031.:	1358583..1359671	Glycolysis/Krebs cycle	COG1071
WP_041711031.1_1475	CUST_7565_PI442190932	NATL2	LLI	WP_041711031.1_1475	ACCGATCATATTTGTAGTCGAAAATAATAAATGGGCAATTGGGATGGCCCATGACAGAGC	pdhA	Pyruvate dehydrogenase E1 component alpha subunit	WP_041711031.:	1358583..1359671	Glycolysis/Krebs cycle	COG1071
WP_041711031.1_1475	CUST_7566_PI442190932	NATL2	LLI	WP_041711031.1_1475	GCTGCAGTAAAGGCAGAGGTGGATCTATGCATCTGTTCTCTAAAGAACATCACCTACTTG	pdhA	Pyruvate dehydrogenase E1 component alpha subunit	WP_041711031.:	1358583..1359671	Glycolysis/Krebs cycle	COG1071
WP_041711031.1_1475	CUST_7567_PI442190932	NATL2	LLI	WP_041711031.1_1475	GCCTTTATTGGTGAAGGTATTCCAGTTGCCCTTGGAGCAGCTTTCAGCAGTAAATATAAA	pdhA	Pyruvate dehydrogenase E1 component alpha subunit	WP_041711031.:	1358583..1359671	Glycolysis/Krebs cycle	COG1071
WP_041711031.1_1475	CUST_7568_PI442190932	NATL2	LLI	WP_041711031.1_1475	ACCCAGACGAGCTTAGATCTGAGAGGGAAAAGGAATTCTGGGCTAAAAGAGATCCAATTA	pdhA	Pyruvate dehydrogenase E1 component alpha subunit	WP_041711031.:	1358583..1359671	Glycolysis/Krebs cycle	COG1071
WP_049752697.1_1495	CUST_7680_PI442190932	NATL2	LLI	WP_049752697.1_1495	ATCGTTGGTACAGTTGTTGACGAAAAACTTGAGGGAGAGATTCAGGTAACCGTTATTGCT	ftsZ	cell division protein FtsZ	WP_049752697.:	1381059..1382162	Cell Division	COG0206
WP_049752697.1_1495	CUST_7681_PI442190932	NATL2	LLI	WP_049752697.1_1495	GTTATCCCAAACGACAGATTAAAAGACGTAATTTCAGGAGCCCCTTTGCAAGAAGCCTTC	ftsZ	cell division protein FtsZ	WP_049752697.:	1381059..1382162	Cell Division	COG0206
WP_049752697.1_1495	CUST_7682_PI442190932	NATL2	LLI	WP_049752697.1_1495	TTACTTGTCCTGGTTTGGTGAATGTGGATTTTGCTGATGTGCGTTCTGTTATGACTGAAG	ftsZ	cell division protein FtsZ	WP_049752697.:	1381059..1382162	Cell Division	COG0206
WP_049752697.1_1495	CUST_7683_PI442190932	NATL2	LLI	WP_049752697.1_1495	TAAGCGTCGTTTAAGACAAGCTGACGAAGGTATAGCAAGGCTTGCAGAGAATGTTGATAC	ftsZ	cell division protein FtsZ	WP_049752697.:	1381059..1382162	Cell Division	COG0206
WP_049752697.1_1495	CUST_7684_PI442190932	NATL2	LLI	WP_049752697.1_1495	AATAGAGCAAGACTTTCACCTAAATCACTTTACGAACAACCAGAAGAGAGGGAAGCAGGG	ftsZ	cell division protein FtsZ	WP_049752697.:	1381059..1382162	Cell Division	COG0206
WP_049752697.1_1495	CUST_7685_PI442190932	NATL2	LLI	WP_049752697.1_1495	GCCCCTGTAGTAGCTCAAGTTGCGAAAGAAAGTGGAGCTTTAACAGTAGGAATAGTTACA	ftsZ	cell division protein FtsZ	WP_049752697.:	1381059..1382162	Cell Division	COG0206
WP_011295242.1_1514	CUST_7777_PI442190932	NATL2	LLI	WP_011295242.1_1514	TGGGATTAATACCTTCTTTACTAGTAATTGCTGGATTAATAACTATGAAACCGTGGCGAA	glcH	GPH family sugar transporter	WP_011295242.:	1397587..1398942	GlcH sugar transporter	COG2211
WP_011295242.1_1514	CUST_7779_PI442190932	NATL2	LLI	WP_011295242.1_1514	TGGAAGAATATCAGCACTATTCAAAGGTGGGAAAATATGGGTATTAGCCTGTTTTTTAGT	glcH	GPH family sugar transporter	WP_011295242.:	1397587..1398942	GlcH sugar transporter	COG2211
WP_011295242.1_1514	CUST_7781_PI442190932	NATL2	LLI	WP_011295242.1_1514	AGCCAGAAAGCCCACTTCTCAATCTGAACCTTTTAATCAGCAGCTAAAAAGAGTTTTAA	glcH	GPH family sugar transporter	WP_011295242.:	1397587..1398942	GlcH sugar transporter	COG2211
WP_011295242.1_1514	CUST_7782_PI442190932	NATL2	LLI	WP_011295242.1_1514	GTTATTGCTACTTTTACCACGTTAATTGCTTGCTGGGGACTAGCCCCATTTGCAAAAAAA	glcH	GPH family sugar transporter	WP_011295242.:	1397587..1398942	GlcH sugar transporter	COG2211
WP_011295261.1_1533	CUST_7889_PI442190932	NATL2	LLI	WP_011295261.1_1533	AATGGCGATAGATTCCCTATCCGCATTGGCTAGAGGTGTAAGCTTAAATGCATTTAGACA	kaiC	circadian clock protein KaiC	WP_011295261.:	1417273..1418775	Circadian Rhythm	COG0467
WP_011295261.1_1533	CUST_7890_PI442190932	NATL2	LLI	WP_011295261.1_1533	GCCCCTCATCGTATTAATCCTGAAGAACAGATGCCTGGCGTTTTTAAAAGCATTAATCCA	kaiC	circadian clock protein KaiC	WP_011295261.:	1417273..1418775	Circadian Rhythm	COG0467
WP_011295261.1_1533	CUST_7891_PI442190932	NATL2	LLI	WP_011295261.1_1533	CCTTTTTGCTTATGAAGAATCAAGAGCACAACTTCTTCGAAATGCAACTAGCTGGGGGAT	kaiC	circadian clock protein KaiC	WP_011295261.:	1417273..1418775	Circadian Rhythm	COG0467
WP_011295261.1_1533	CUST_7892_PI442190932	NATL2	LLI	WP_011295261.1_1533	ACGGGTGCAACTGGTACCGGAAAAACAATGCTAGTTTCTAAGTTTGTTGAAGATGCATAC	kaiC	circadian clock protein KaiC	WP_011295261.:	1417273..1418775	Circadian Rhythm	COG0467
WP_011295261.1_1533	CUST_7893_PI442190932	NATL2	LLI	WP_011295261.1_1533	AAATAAGAGGTGAAATGGCAAGAGCAATAAATGTTTTCAAGATGCGCGGATCATGGCATG	kaiC	circadian clock protein KaiC	WP_011295261.:	1417273..1418775	Circadian Rhythm	COG0467
WP_011295261.1_1533	CUST_7894_PI442190932	NATL2	LLI	WP_011295261.1_1533	TCATCCAATATTCGAATAAGTTCTGGTGTTCAGGATTTGGATGAAATGTGTGGCGGAGGA	kaiC	circadian clock protein KaiC	WP_011295261.:	1417273..1418775	Circadian Rhythm	COG0467
WP_011295262.1_1534	CUST_7895_PI442190932	NATL2	LLI	WP_011295262.1_1534	TGGACCTTTTGTATGATGAATTATCTGATAATGAAATGTTTTACTCTTCAGA	kaiB	Circadian clock protein KaiB	WP_011295262.:	1418906..1419229	Circadian Rhythm	COG4251
WP_011295262.1_1534	CUST_7896_PI442190932	NATL2	LLI	WP_011295262.1_1534	GTTCGAAGAATCATTGGTGACCTATCAGATAGAGAAAAGGTCTTAATTGGACTGGACCTT	kaiB	Circadian clock protein KaiB	WP_011295262.:	1418906..1419229	Circadian Rhythm	COG4251
WP_011295262.1_1534	CUST_7897_PI442190932	NATL2	LLI	WP_011295262.1_1534	TTAGCCGAAGAAGATAAAATACTTGCCACACCAACCTTGTCTAAGATTTTACCCCCACCT	kaiB	Circadian clock protein KaiB	WP_011295262.:	1418906..1419229	Circadian Rhythm	COG4251
WP_011295262.1_1534	CUST_7898_PI442190932	NATL2	LLI	WP_011295262.1_1534	GAAAGTGAATTCAAAGGTGTCTATGCTCTGAAGGTTATTGATGTTTTAAAAAGCCCT	kaiB	Circadian clock protein KaiB	WP_011295262.:	1418906..1419229	Circadian Rhythm	COG4251
WP_011295262.1_1534	CUST_7899_PI442190932	NATL2	LLI	WP_011295262.1_1534	GTGGCTGGGAATACACCAAACTCTATGAGGGCTTTGAACACGTTGAGAGAAATCTTAGAA	kaiB	Circadian clock protein KaiB	WP_011295262.:	1418906..1419229	Circadian Rhythm	COG4251
WP_011295317.1_1597	CUST_8176_PI442190932	NATL2	LLI	WP_011295317.1_1597	TCTCCTGAAAAAATCAAAGCACAAGAAACATTGAACAAAGCAAAAGCTTGGTTTCAAGCA	atpC	ATP synthase epsilon subunit	WP_011295317.:	1465040..1465447	Energy Metabolism	COG0355
WP_011295317.1_1597	CUST_8177_PI442190932	NATL2	LLI	WP_011295317.1_1597	AAAGCTGAAATGGGGAAAAATATTGACTCTGGCAAAGCTGAAGCTGAATTAGAAAAAGCT	atpC	ATP synthase epsilon subunit	WP_011295317.:	1465040..1465447	Energy Metabolism	COG0355
WP_011295317.1_1597	CUST_8178_PI442190932	NATL2	LLI	WP_011295317.1_1597	TGGGAATTGGGACTCAATAGCCTTAACTGGAGGTTTTGCAGAAGTTGAATCTAACGAAGT	atpC	ATP synthase epsilon subunit	WP_011295317.:	1465040..1465447	Energy Metabolism	COG0355
WP_011295317.1_1597	CUST_8179_PI442190932	NATL2	LLI	WP_011295317.1_1597	ACCTGGTCACATTTCAATGGTTACTGCTATTGATTTTGGTGTATTGAGGGTTTTAAAAAA	atpC	ATP synthase epsilon subunit	WP_011295317.:	1465040..1465447	Energy Metabolism	COG0355
WP_011295317.1_1597	CUST_8180_PI442190932	NATL2	LLI	WP_011295317.1_1597	ATTTGACGATACTGCTGATGAAATAATACTTCCGAGCACTACAGGGCTTTTGGGGGTATT	atpC	ATP synthase epsilon subunit	WP_011295317.:	1465040..1465447	Energy Metabolism	COG0355
WP_011295327.1_1607	CUST_8232_PI442190932	NATL2	LLI	WP_011295327.1_1607	AACTCTGGATTAAGACCTGCAATTAACGTTGGTATATCGGTTAGTCGTGTAGGAGGAGCT	atpA	ATP synthase alpha subunit	WP_011295327.:	1474578..1476092	Energy Metabolism	COG0056
WP_011295327.1_1607	CUST_8233_PI442190932	NATL2	LLI	WP_011295327.1_1607	GCTCAATTTGATGAACTTGCGGCATTCTCTCAATTTGCTTCAGATCTTGATGAGGCTACT	atpA	ATP synthase alpha subunit	WP_011295327.:	1474578..1476092	Energy Metabolism	COG0056
WP_011295327.1_1607	CUST_8234_PI442190932	NATL2	LLI	WP_011295327.1_1607	AAAGCAACTAGGTAGAGGTAAAAGGCTAAGAGAACTTCTCAAGCAGCCTCAATTTGACCC	atpA	ATP synthase alpha subunit	WP_011295327.:	1474578..1476092	Energy Metabolism	COG0056
WP_011295327.1_1607	CUST_8235_PI442190932	NATL2	LLI	WP_011295327.1_1607	AAACTTTCTGATGCTATGGGTGCAGGATCAATGACATCCTTGCCTATTATTGAAACGCAG	atpA	ATP synthase alpha subunit	WP_011295327.:	1474578..1476092	Energy Metabolism	COG0056
WP_011295327.1_1607	CUST_8236_PI442190932	NATL2	LLI	WP_011295327.1_1607	AGTGAAGCATCTGAATCAATGCTTAAAGACGCTATTAGCGAGGTTAAGTCCTCCATGCTT	atpA	ATP synthase alpha subunit	WP_011295327.:	1474578..1476092	Energy Metabolism	COG0056
WP_011295327.1_1607	CUST_8237_PI442190932	NATL2	LLI	WP_011295327.1_1607	AGGCAAGGCGACACTAGTTATATACGATGATCTAACTAAGCAAGCTCAGGCTTATAGGCA	atpA	ATP synthase alpha subunit	WP_011295327.:	1474578..1476092	Energy Metabolism	COG0056
WP_011295342.1_1623	CUST_8319_PI442190932	NATL2	LLI	WP_011295342.1_1623	CAGCCAATGAACCTGGTAGCTCAATAATGCCAGGGAAAATTAATCCTACTCAATGCGAAG	fumC	class II fumarate hydratase	WP_011295342.:	1486955..1488349	Krebs cycle and related genes	COG0114
WP_011295342.1_1623	CUST_8320_PI442190932	NATL2	LLI	WP_011295342.1_1623	CGATCGCAGGAAGTGGAGGACATCTACAAATGAATGTCTATAAACCACTGATTGGCTACA	fumC	class II fumarate hydratase	WP_011295342.:	1486955..1488349	Krebs cycle and related genes	COG0114
WP_011295342.1_1623	CUST_8321_PI442190932	NATL2	LLI	WP_011295342.1_1623	TTATCCGCTTCCACTTGGAGGAACCGCTATTGGTACAGGGATTAATACTCCTAAGGATTT	fumC	class II fumarate hydratase	WP_011295342.:	1486955..1488349	Krebs cycle and related genes	COG0114
WP_011295342.1_1623	CUST_8322_PI442190932	NATL2	LLI	WP_011295342.1_1623	AACCGCCTTATCTCCATCAATAGGATACGAAAAAGCAAGTAAAATTGCTCAACTAGCCCA	fumC	class II fumarate hydratase	WP_011295342.:	1486955..1488349	Krebs cycle and related genes	COG0114
WP_011295342.1_1623	CUST_8323_PI442190932	NATL2	LLI	WP_011295342.1_1623	GCCGTCACTTTTCTCAAAATTGTTAATGATCTTCGACTTTTATCTTGTGGACCAAGAGCT	fumC	class II fumarate hydratase	WP_011295342.:	1486955..1488349	Krebs cycle and related genes	COG0114
WP_011295342.1_1623	CUST_8324_PI442190932	NATL2	LLI	WP_011295342.1_1623	ACCTTTTAAAACTGCAGCTAATAAATTTGCCATCATGGCTAGTCATGATGGTCTTGTAAA	fumC	class II fumarate hydratase	WP_011295342.:	1486955..1488349	Krebs cycle and related genes	COG0114
WP_011295422.1_1705	CUST_8758_PI442190932	NATL2	LLI	WP_011295422.1_1705	TTATTGAAGGAGCTGGAGACCATTGTTGCGAATATATGACTGGAGGAGTAGTTGTTGTGC	glsF	Ferredoxin-dependent glutamate synthase	WP_011295422.:	1568831..1573420	Nitrogen Metabolism	COG0069
WP_011295422.1_1705	CUST_8759_PI442190932	NATL2	LLI	WP_011295422.1_1705	CACATCTAACACTCAGGTGATACTTGGCAACACATGTCTATATGGAGCAACTGGTGGAAA	glsF	Ferredoxin-dependent glutamate synthase	WP_011295422.:	1568831..1573420	Nitrogen Metabolism	COG0069
WP_011295422.1_1705	CUST_8760_PI442190932	NATL2	LLI	WP_011295422.1_1705	AGATTCTCGCAGACTGGTCTTCATGGAAGGAATTATTTAAAGCAGTAGTTCCTCCAAGCG	glsF	Ferredoxin-dependent glutamate synthase	WP_011295422.:	1568831..1573420	Nitrogen Metabolism	COG0069
WP_011295422.1_1705	CUST_8761_PI442190932	NATL2	LLI	WP_011295422.1_1705	GGTTTTAATGGGAATTTAAATTTGATATTTAAAGGTTCAGCGGGGCAAAGCTTTGGTGCA	glsF	Ferredoxin-dependent glutamate synthase	WP_011295422.:	1568831..1573420	Nitrogen Metabolism	COG0069
WP_011295422.1_1705	CUST_8762_PI442190932	NATL2	LLI	WP_011295422.1_1705	TGGAGAAGCTAATGATTATGTAGGAAAAGGCATTAACGGAGGTTCCATTACGATTGTTCC	glsF	Ferredoxin-dependent glutamate synthase	WP_011295422.:	1568831..1573420	Nitrogen Metabolism	COG0069
WP_011295422.1_1705	CUST_8763_PI442190932	NATL2	LLI	WP_011295422.1_1705	TGGAAAAACTGGAAGAAATATAGGAGCAGGAATGACTGGAGGCATAGCTTTTATTCTTGA	glsF	Ferredoxin-dependent glutamate synthase	WP_011295422.:	1568831..1573420	Nitrogen Metabolism	COG0069
WP_011295426.1_1709	CUST_8781_PI442190932	NATL2	LLI	WP_011295426.1_1709	CATGCCAAGTTTCATCTTGGGATCATGTTTTCCTTGGATTGTTCTGGATGTATAACGGCT	psaA	Photosystem I P700 chlorophyll A apoprotein A1 PsaA	WP_011295426.:	1577169..1579475	Photosystem I	COG2885
WP_011295426.1_1709	CUST_8782_PI442190932	NATL2	LLI	WP_011295426.1_1709	AGTGGATTCAAAATATTCATAATTCAGCAGCTGGTTCTACCACTCTTGCTGGTGCAAACG	psaA	Photosystem I P700 chlorophyll A apoprotein A1 PsaA	WP_011295426.:	1577169..1579475	Photosystem I	COG2885
WP_011295426.1_1709	CUST_8783_PI442190932	NATL2	LLI	WP_011295426.1_1709	ACTAATTCCTGACAAAGCGAATCTTGGATTTAGATTCCCATGTGATGGACCAGGAAGAGG	psaA	Photosystem I P700 chlorophyll A apoprotein A1 PsaA	WP_011295426.:	1577169..1579475	Photosystem I	COG2885
WP_011295426.1_1709	CUST_8784_PI442190932	NATL2	LLI	WP_011295426.1_1709	TCCTATGGGCTCAGTCATCTCAGGTCCTAACAAGTTATGGTCAACCTATAAGCATGTACG	psaA	Photosystem I P700 chlorophyll A apoprotein A1 PsaA	WP_011295426.:	1577169..1579475	Photosystem I	COG2885
WP_011295426.1_1709	CUST_8785_PI442190932	NATL2	LLI	WP_011295426.1_1709	TGGAAGATGCAAAGTGATGTATGGGGTCTAACAGGAGGAAACTTTGCTCAAAGTTCCATA	psaA	Photosystem I P700 chlorophyll A apoprotein A1 PsaA	WP_011295426.:	1577169..1579475	Photosystem I	COG2885
WP_011295426.1_1709	CUST_8786_PI442190932	NATL2	LLI	WP_011295426.1_1709	GGTTCCGTAAGTCAAGTTGGAGGAAAAATTGGAATCGCTCCTATACCTTTAGGAACTGCT	psaA	Photosystem I P700 chlorophyll A apoprotein A1 PsaA	WP_011295426.:	1577169..1579475	Photosystem I	COG2885
WP_011295427.1_1710	CUST_8787_PI442190932	NATL2	LLI	WP_011295427.1_1710	CCATGCCAGGTAATCACTACTGGATGGACATGATCAATAGACCAGATGCACTAACCAATT	psaB	Photosystem I P700 chlorophyll A apoprotein A2 PsaB	WP_011295427.:	1579500..1581728	Photosystem I	COG2885
WP_011295427.1_1710	CUST_8788_PI442190932	NATL2	LLI	WP_011295427.1_1710	TGGTGTAAATGCTTTATCTCCATGGGCTTGGATGTTCTTATTTGGTCATCTCATTTGGGC	psaB	Photosystem I P700 chlorophyll A apoprotein A2 PsaB	WP_011295427.:	1579500..1581728	Photosystem I	COG2885
WP_011295427.1_1710	CUST_8789_PI442190932	NATL2	LLI	WP_011295427.1_1710	GTTGGAGAGATAAGCCTGTTGCATTATCCATTGTCCAAGCACGATTAGTTGGTTTAACCC	psaB	Photosystem I P700 chlorophyll A apoprotein A2 PsaB	WP_011295427.:	1579500..1581728	Photosystem I	COG2885
WP_011295427.1_1710	CUST_8790_PI442190932	NATL2	LLI	WP_011295427.1_1710	CCAGGTCGTGGTGGTACTTGTGATAGTTCTTCTTGGGACGCTACTTATTTAGCAATGTTC	psaB	Photosystem I P700 chlorophyll A apoprotein A2 PsaB	WP_011295427.:	1579500..1581728	Photosystem I	COG2885
WP_011295427.1_1710	CUST_8791_PI442190932	NATL2	LLI	WP_011295427.1_1710	TGGCATTGGAAACATCTAGCAATTTGGATGGGTAATACCGCTCAATTCAATGAATCTGGT	psaB	Photosystem I P700 chlorophyll A apoprotein A2 PsaB	WP_011295427.:	1579500..1581728	Photosystem I	COG2885
WP_011295427.1_1710	CUST_8792_PI442190932	NATL2	LLI	WP_011295427.1_1710	TATTGGACCTGCAGATTTCTTAGTTCACCATGCGATTGCACTAGGACTACATACAACTGC	psaB	Photosystem I P700 chlorophyll A apoprotein A2 PsaB	WP_011295427.:	1579500..1581728	Photosystem I	COG2885
WP_011295491.1_1778	CUST_9150_PI442190932	NATL2	LLI	WP_011295491.1_1778	ACCCTGCTTTGCAGTTGTCTGTCAACCTGAGAAATAGGACTATTGTCCCTTTAGGATTTT	ppc	Phosphoenolpyruvate carboxylase	WP_011295491.:	1632922..1635906	Krebs cycle and related genes	COG2352
WP_011295491.1_1778	CUST_9151_PI442190932	NATL2	LLI	WP_011295491.1_1778	ATAGTCGTAGTGAATTATTGCGTGGTGCATTGTTGACTATCAATGGTATCGCTGCAGGTA	ppc	Phosphoenolpyruvate carboxylase	WP_011295491.:	1632922..1635906	Krebs cycle and related genes	COG2352
WP_011295491.1_1778	CUST_9152_PI442190932	NATL2	LLI	WP_011295491.1_1778	CTTTAGCTACTGAATTAAAGGCTGACCCCGACCAAATGGAGATGTTGCGAATGTTGAATC	ppc	Phosphoenolpyruvate carboxylase	WP_011295491.:	1632922..1635906	Krebs cycle and related genes	COG2352
WP_011295491.1_1778	CUST_9153_PI442190932	NATL2	LLI	WP_011295491.1_1778	AAAGACTTATCAAGTCTTCGAGCTATCCCATGGGTCTTTGGTTGGACTCAAAGTCGTTTC	ppc	Phosphoenolpyruvate carboxylase	WP_011295491.:	1632922..1635906	Krebs cycle and related genes	COG2352
WP_011295491.1_1778	CUST_9154_PI442190932	NATL2	LLI	WP_011295491.1_1778	TTGGTTACCAATAAATTGGATGCTACGCCAAGTTGGAATGAATTGATGACCAGACTTGCA	ppc	Phosphoenolpyruvate carboxylase	WP_011295491.:	1632922..1635906	Krebs cycle and related genes	COG2352
WP_011295491.1_1778	CUST_9155_PI442190932	NATL2	LLI	WP_011295491.1_1778	TGGGTGGAAGTGATGATCGGGATGCTTTCGCTAGAATTTTCGATATTATCTCAAGCGAAT	ppc	Phosphoenolpyruvate carboxylase	WP_011295491.:	1632922..1635906	Krebs cycle and related genes	COG2352
WP_011295516.1_1803	CUST_9293_PI442190932	NATL2	LLI	WP_011295516.1_1803	CCCGAAATTTTAGCTGCAGTTGATGGGAAAATAGATGTGTTGCTAGATGGAGGTATTCGC	lldD	alpha-hydroxy-acidoxidizingprotein (L-lactate)	WP_011295516.:	1659756..1660940	Pyruvate metabolism	COG1304
WP_011295516.1_1803	CUST_9294_PI442190932	NATL2	LLI	WP_011295516.1_1803	AGGCTTAATGAGTTTTCCTAATGTTCAACTAGATGATGGCCCTATGGGATACACGGCAAT	lldD	alpha-hydroxy-acidoxidizingprotein (L-lactate)	WP_011295516.:	1659756..1660940	Pyruvate metabolism	COG1304
WP_011295516.1_1803	CUST_9295_PI442190932	NATL2	LLI	WP_011295516.1_1803	AATTATTGTTAAGGGTATACATATTGGTGATGACGCAAAAAAAGCGGCAGAGCTAGGGGC	lldD	alpha-hydroxy-acidoxidizingprotein (L-lactate)	WP_011295516.:	1659756..1660940	Pyruvate metabolism	COG1304
WP_011295516.1_1803	CUST_9296_PI442190932	NATL2	LLI	WP_011295516.1_1803	TGCAGGAGGGAAAGGCGTTGCCAGAGCTATAGAAATTCTTCAAACAGATATAGTGAGAAC	lldD	alpha-hydroxy-acidoxidizingprotein (L-lactate)	WP_011295516.:	1659756..1660940	Pyruvate metabolism	COG1304
WP_011295516.1_1803	CUST_9297_PI442190932	NATL2	LLI	WP_011295516.1_1803	GTGATGTTGTGAAAGCATTATGTCTTGGCGCGAAAGGAGTTTTGATCGGTAGAGCATATG	lldD	alpha-hydroxy-acidoxidizingprotein (L-lactate)	WP_011295516.:	1659756..1660940	Pyruvate metabolism	COG1304
WP_011295516.1_1803	CUST_9298_PI442190932	NATL2	LLI	WP_011295516.1_1803	TGATACACCTGTATCTGGTTTGAGGGAAAGAGATATGCGATCAGGAACTCAACAGCTTTT	lldD	alpha-hydroxy-acidoxidizingprotein (L-lactate)	WP_011295516.:	1659756..1660940	Pyruvate metabolism	COG1304
WP_011822813.1_12	CUST_67_PI442190912	NATL1	LLI	WP_011822813.1_12	CCTGATGTACCTGAACTTGTTCGAGGCAAGTCAGGAAGAGTATTTGGACATTTACAAGCT	argH	Argininosuccinate lyase	WP_011822813.:	14783..16174	Amino Acid metabolism	COG0165
WP_011822813.1_12	CUST_68_PI442190912	NATL1	LLI	WP_011822813.1_12	TTTGTGGGCATCTGAAGAATTTGCCTTTATTCAATTAACCGACCGATGTGCTACTGGAAG	argH	Argininosuccinate lyase	WP_011822813.:	14783..16174	Amino Acid metabolism	COG0165
WP_011822813.1_12	CUST_69_PI442190912	NATL1	LLI	WP_011822813.1_12	TTTCAAATGCGACTGATGTCGCTGATTATTTAGTGGCTAAGGACATACCTTTCCGAGAGG	argH	Argininosuccinate lyase	WP_011822813.:	14783..16174	Amino Acid metabolism	COG0165
WP_011822813.1_12	CUST_70_PI442190912	NATL1	LLI	WP_011822813.1_12	GCTGGTGGAACTGGATTTGAGAGAGTTCAAGAACAGCTTCTTTCTTGGCGAGAAAAATTA	argH	Argininosuccinate lyase	WP_011822813.:	14783..16174	Amino Acid metabolism	COG0165
WP_011822813.1_12	CUST_71_PI442190912	NATL1	LLI	WP_011822813.1_12	AGTTGGGCGAATTGTAAAAACTTCCTTGGAGGAGGGGATTTTATTAAAAGATTTTCCTTT	argH	Argininosuccinate lyase	WP_011822813.:	14783..16174	Amino Acid metabolism	COG0165
WP_011822813.1_12	CUST_72_PI442190912	NATL1	LLI	WP_011822813.1_12	TGCTGTAAGTGATAGAGACTTTGTCGTAGAATTTTTAGGAGCTTCATCGTTAATTATGGC	argH	Argininosuccinate lyase	WP_011822813.:	14783..16174	Amino Acid metabolism	COG0165
WP_011823063.1_260	CUST_1413_PI442190912	NATL1	LLI	WP_011823063.1_260	AGTGTGCAATTAGTTGGAGATGATTTATTTGTTACCAACACACTGCGTTTGCAACGAGGA	eno	Enolase	WP_011823063.:	263364..264665	Glycolysis/Gluconeogenesis	COG0148
WP_011823063.1_260	CUST_1414_PI442190912	NATL1	LLI	WP_011823063.1_260	GGAGAGCAAGTATCTCTAGCTTTAGATGTTGCGAGTACGGAGTTTTATGAGGAGGGACAA	eno	Enolase	WP_011823063.:	263364..264665	Glycolysis/Gluconeogenesis	COG0148
WP_011823063.1_260	CUST_1415_PI442190912	NATL1	LLI	WP_011823063.1_260	TTGAAGATGGATTAGCTGAAGATGATTGGGATGGTTGGAGCTTGCTTACGAAAAAGCTTG	eno	Enolase	WP_011823063.:	263364..264665	Glycolysis/Gluconeogenesis	COG0148
WP_011823063.1_260	CUST_1416_PI442190912	NATL1	LLI	WP_011823063.1_260	AAGGTTTATCAACTGCTGTTGGTGATGAAGGGGGATTCGCTCCAAATCTTGAGAGTAATA	eno	Enolase	WP_011823063.:	263364..264665	Glycolysis/Gluconeogenesis	COG0148
WP_011823063.1_260	CUST_1417_PI442190912	NATL1	LLI	WP_011823063.1_260	TAATCAACTACTGCGTATTGAGGATGAGTTAGGAAGTCAAGCAACATATGCTGGGCTTGT	eno	Enolase	WP_011823063.:	263364..264665	Glycolysis/Gluconeogenesis	COG0148
WP_011823063.1_260	CUST_1418_PI442190912	NATL1	LLI	WP_011823063.1_260	TGTTATGGTGGAAATTCTTATTCCAGCGAGCAAATGGTTGAGGAATTGGCTGGATTAGTT	eno	Enolase	WP_011823063.:	263364..264665	Glycolysis/Gluconeogenesis	COG0148
WP_011823080.1_279	CUST_1515_PI442190912	NATL1	LLI	WP_011823080.1_279	TGGATATGATGCTTTGGTCATCGGAAGAGAATTGCGAAATCAAACACCTGATAAAGACCT	eda	2-dehydro-3-deoxyphosphogluconate aldolase	WP_011823080.:	283012..283647	Entner-Dudoroff	COG0800
WP_011823080.1_279	CUST_1516_PI442190912	NATL1	LLI	WP_011823080.1_279	ATTGGTGTTGCATCAATTTCTTCAAGGCAATCTTTAGAATCAATTCTCTCATTAGATGTT	eda	2-dehydro-3-deoxyphosphogluconate aldolase	WP_011823080.:	283012..283647	Entner-Dudoroff	COG0800
WP_011823080.1_279	CUST_1517_PI442190912	NATL1	LLI	WP_011823080.1_279	CAATTAGTTATTCCGGGGATATCAAATATTGAAAATTTCAAAGAGGCAATCGATCTAGGA	eda	2-dehydro-3-deoxyphosphogluconate aldolase	WP_011823080.:	283012..283647	Entner-Dudoroff	COG0800
WP_011823080.1_279	CUST_1518_PI442190912	NATL1	LLI	WP_011823080.1_279	ATTGTCTAATTTTGGTATAAAAAATATTGAAATAGGGTGGGATTCTAATCCAGAATGGGT	eda	2-dehydro-3-deoxyphosphogluconate aldolase	WP_011823080.:	283012..283647	Entner-Dudoroff	COG0800
WP_011823080.1_279	CUST_1519_PI442190912	NATL1	LLI	WP_011823080.1_279	TGGAAGCTAAGCAAAATAATTTAATAAAATCTCTTAGGATACAGCCGCTTATAGTTGTAA	eda	2-dehydro-3-deoxyphosphogluconate aldolase	WP_011823080.:	283012..283647	Entner-Dudoroff	COG0800
WP_011823080.1_279	CUST_1520_PI442190912	NATL1	LLI	WP_011823080.1_279	AAAGGATATTTTTTTTATTGGTGCAGGTGGAATTAAAAGTAAGAATTTAAAAAAATTCCT	eda	2-dehydro-3-deoxyphosphogluconate aldolase	WP_011823080.:	283012..283647	Entner-Dudoroff	COG0800
WP_041700668.1_381	CUST_2070_PI442190912	NATL1	LLI	WP_041700668.1_381	CCAACTTAAAGAGCCTAAGAATCAGTCAGAATTAGATTCTCCAGAAACTGAAGGCACAGA	minE	septum site-determining protein MinE	WP_041700668.:	377904..378245	Cell Division	COG0851
WP_041700668.1_381	CUST_2071_PI442190912	NATL1	LLI	WP_041700668.1_381	CAGAGGATCGCATGACCGCATTAGTTGCAAACCTACCAATAAAAGAACTATTACTGGGC	minE	septum site-determining protein MinE	WP_041700668.:	377904..378245	Cell Division	COG0851
WP_041700668.1_381	CUST_2072_PI442190912	NATL1	LLI	WP_041700668.1_381	ACTTGAGGTGGTCGCAAAGTATGTAGAGATAGATGTGGATGAAGGAGCAGTAAGTCTTGA	minE	septum site-determining protein MinE	WP_041700668.:	377904..378245	Cell Division	COG0851
WP_041700668.1_381	CUST_2073_PI442190912	NATL1	LLI	WP_041700668.1_381	GCGAGAGAGAGGTTGCAACTTGTTCTAGCTCATGATCGATCAGATCTCAGTACAGAACTT	minE	septum site-determining protein MinE	WP_041700668.:	377904..378245	Cell Division	COG0851
WP_041700668.1_381	CUST_2074_PI442190912	NATL1	LLI	WP_041700668.1_381	ATGACACTTAGAGACATTATCAACAAGTTACTACGTAGACAGCCTGCTAGCGCTAGTACC	minE	septum site-determining protein MinE	WP_041700668.:	377904..378245	Cell Division	COG0851
WP_011823170.1_382	CUST_2075_PI442190912	NATL1	LLI	WP_011823170.1_382	CCAATCTATCTTTACTTCCTGCGGGGAATCCAAGAATGCTTGATTGGTTAAAACCCGATG	minD	septum site-determining protein MinD	WP_011823170.:	378256..379071	Cell Division	COG2894
WP_011823170.1_382	CUST_2076_PI442190912	NATL1	LLI	WP_011823170.1_382	GCCTTGTGCTAGAGGATGAACAAGTAATTGTAAGTACAAACCGAGGTGAGCCACTAACTT	minD	septum site-determining protein MinD	WP_011823170.:	378256..379071	Cell Division	COG2894
WP_011823170.1_382	CUST_2077_PI442190912	NATL1	LLI	WP_011823170.1_382	TCTCAAGAAGCAATTGTAGTTACCAACCCTGAGGTATCAGCAGTAAGAGATGCAGACAGA	minD	septum site-determining protein MinD	WP_011823170.:	378256..379071	Cell Division	COG2894
WP_011823170.1_382	CUST_2078_PI442190912	NATL1	LLI	WP_011823170.1_382	CAAGAAGTTCTAGAGGAAGAATGCAGACTTGATCAGGCCTTAGTGAAGCACAAGCAAGAA	minD	septum site-determining protein MinD	WP_011823170.:	378256..379071	Cell Division	COG2894
WP_011823170.1_382	CUST_2079_PI442190912	NATL1	LLI	WP_011823170.1_382	AACCAGTCCAATTAGTTCTTAATCGAGTTAGGCCAAAGATGATGGCCAACCAAGAAATGC	minD	septum site-determining protein MinD	WP_011823170.:	378256..379071	Cell Division	COG2894
WP_011823170.1_382	CUST_2080_PI442190912	NATL1	LLI	WP_011823170.1_382	ACAAGGAGAAGATATTCCACTTATTGATCCAGCAGAGGAGATTTCTGGATTTGGTGCGAA	minD	septum site-determining protein MinD	WP_011823170.:	378256..379071	Cell Division	COG2894
WP_011823171.1_383	CUST_2081_PI442190912	NATL1	LLI	WP_011823171.1_383	TAAAAAAGTTGCTAGAGGTCCAAAGGAGAAGCCTCAATTTGGTCTTGCAGAACAAGCAAG	minC	septum site-determining protein MinC	WP_011823171.:	379235..379879	Cell Division	COG0850
WP_011823171.1_383	CUST_2082_PI442190912	NATL1	LLI	WP_011823171.1_383	AGGCAACAAAGGAAACTCTAAAGCAAAAATATCCGCACTTCAACTTAGCCCTGTTCAACT	minC	septum site-determining protein MinC	WP_011823171.:	379235..379879	Cell Division	COG0850
WP_011823171.1_383	CUST_2083_PI442190912	NATL1	LLI	WP_011823171.1_383	CCTTGGAGATGTTAATCCAGGGGCAAATGTTAGCGCAGAGGGAAATATAATTATTTGGGG	minC	septum site-determining protein MinC	WP_011823171.:	379235..379879	Cell Division	COG0850
WP_011823171.1_383	CUST_2084_PI442190912	NATL1	LLI	WP_011823171.1_383	CTCACTTTCATCAAGGAACTGTCAGGTCAGGAGAATATATTGAAAGTCCTGGGGATTTGT	minC	septum site-determining protein MinC	WP_011823171.:	379235..379879	Cell Division	COG0850
WP_011823171.1_383	CUST_2085_PI442190912	NATL1	LLI	WP_011823171.1_383	AGATTATTTGTTTTTGCTCATCATCTCCAAAGACGATTGTAAGTTCTCAATCACTAGGTT	minC	septum site-determining protein MinC	WP_011823171.:	379235..379879	Cell Division	COG0850
WP_011823171.1_383	CUST_2086_PI442190912	NATL1	LLI	WP_011823171.1_383	TGCTCAAAATATCGATCTTTCATGTAAAGAGATATTAGAAATAATTGAAATTGCGAATCA	minC	septum site-determining protein MinC	WP_011823171.:	379235..379879	Cell Division	COG0850
WP_011823243.1_462	CUST_2486_PI442190912	NATL1	LLI	WP_011823243.1_462	CTCAGGAAGCAAGAATGAGCTCTCAGCTGAAGAACATGAAGATTTTCGTTTGTTTGGCTT	cyoC	heme-copper oxidase subunit III	WP_011823243.:	453350..453955	Respiration	COG1845
WP_011823243.1_462	CUST_2487_PI442190912	NATL1	LLI	WP_011823243.1_462	GCTGTAGAGCTCTATTGGCACTTCGTAGATGGGATTTGGGTCGTTTTATTCATCATCCTT	cyoC	heme-copper oxidase subunit III	WP_011823243.:	453350..453955	Respiration	COG1845
WP_011823243.1_462	CUST_2488_PI442190912	NATL1	LLI	WP_011823243.1_462	GGTTTTTTCGCGGCATATCTCACTTTCAAAGCAGTCAATCCTATTGGTCCTGATGCTATC	cyoC	heme-copper oxidase subunit III	WP_011823243.:	453350..453955	Respiration	COG1845
WP_011823243.1_462	CUST_2489_PI442190912	NATL1	LLI	WP_011823243.1_462	TAATGATTATTTGGTGGCAAACACGTGTCCCATCTGGCCGAGTGAATAATCAAAACAAGT	cyoC	heme-copper oxidase subunit III	WP_011823243.:	453350..453955	Respiration	COG1845
WP_011823243.1_462	CUST_2490_PI442190912	NATL1	LLI	WP_011823243.1_462	ACTGACAACCTTTTTGCCAGTACTTTTTATGCATTAACTGGCTTTCATGGGCTTCACGTA	cyoC	heme-copper oxidase subunit III	WP_011823243.:	453350..453955	Respiration	COG1845
WP_011823243.1_462	CUST_2491_PI442190912	NATL1	LLI	WP_011823243.1_462	TTCATCGGGCTGGGAAATTCCTAGAAAAAAACGAATCAAAACAATGCCAAAAATGGCTCC	cyoC	heme-copper oxidase subunit III	WP_011823243.:	453350..453955	Respiration	COG1845
WP_011823244.1_463	CUST_2492_PI442190912	NATL1	LLI	WP_011823244.1_463	TTGAGAATTGGGACGGAGAAGCCCCACTAGTTCTTGAACCTTATGGTTATGGAGAAAAAG	cyoB/coxA	Cytochrome c oxidase subunit I	WP_011823244.:	453952..455592	Respiration	COG0843
WP_011823244.1_463	CUST_2493_PI442190912	NATL1	LLI	WP_011823244.1_463	TGCGACCTTATGGGGAGGAAAAATATCACTTAATGCTGCAATGTTATTTTCCTGCGGATT	cyoB/coxA	Cytochrome c oxidase subunit I	WP_011823244.:	453952..455592	Respiration	COG0843
WP_011823244.1_463	CUST_2494_PI442190912	NATL1	LLI	WP_011823244.1_463	CAAGCGGTACTCCTCCTTGGATGCGCTTATTTTTTACAATCGCTACTGCATTTATAGCTG	cyoB/coxA	Cytochrome c oxidase subunit I	WP_011823244.:	453952..455592	Respiration	COG0843
WP_011823244.1_463	CUST_2495_PI442190912	NATL1	LLI	WP_011823244.1_463	TTAATCTTTGTTTTGCTCCTCAACATTGGCTAGGTTTAAACGGAATGCCTCGACGAGTTG	cyoB/coxA	Cytochrome c oxidase subunit I	WP_011823244.:	453952..455592	Respiration	COG0843
WP_011823244.1_463	CUST_2496_PI442190912	NATL1	LLI	WP_011823244.1_463	GGAGTTGCCCTAGCGCAAGTACCTTTTGATGTTCATGTACACGACACTTATTTTGTTGTT	cyoB/coxA	Cytochrome c oxidase subunit I	WP_011823244.:	453952..455592	Respiration	COG0843
WP_011823244.1_463	CUST_2497_PI442190912	NATL1	LLI	WP_011823244.1_463	TTATGGAATATTCTTCAAAGCATCCTCTTTGGGGAAGAGGCTGGTGATAACCCTTGGAAT	cyoB/coxA	Cytochrome c oxidase subunit I	WP_011823244.:	453952..455592	Respiration	COG0843
WP_011823245.1_464	CUST_2498_PI442190912	NATL1	LLI	WP_011823245.1_464	ATGAATGCTCCTGTTTACGATGAACTCTATCGAGTCCTGTTCATTATTGGCGTCATGCTT	ctaC/coxB	Cytochrome c oxidase subunit II	WP_011823245.:	455597..456409	Respiration	COG1622
WP_011823245.1_464	CUST_2499_PI442190912	NATL1	LLI	WP_011823245.1_464	GCTACGATATTTATGATCGAATGGGTGGTATGCAACCTTTAATGCATGATCACAGTGGGC	ctaC/coxB	Cytochrome c oxidase subunit II	WP_011823245.:	455597..456409	Respiration	COG1622
WP_011823245.1_464	CUST_2500_PI442190912	NATL1	LLI	WP_011823245.1_464	TAATTTCCCAATTGTTTGCGCAGAATTGTGTGGTCCTTATCATGGTGGGATGAGATCAAA	ctaC/coxB	Cytochrome c oxidase subunit II	WP_011823245.:	455597..456409	Respiration	COG1622
WP_011823245.1_464	CUST_2501_PI442190912	NATL1	LLI	WP_011823245.1_464	TAGTCTTATCCAATTTCGTCGTAGACCTGGAGAAACTGGAGATGGCATTGACTTAGAGGG	ctaC/coxB	Cytochrome c oxidase subunit II	WP_011823245.:	455597..456409	Respiration	COG1622
WP_011823245.1_464	CUST_2502_PI442190912	NATL1	LLI	WP_011823245.1_464	GCCGAGAGAGTTTGGGGAGGGATTGGTTCTGGTCCAATTGAGTCTTCATCTAAAAATAAT	ctaC/coxB	Cytochrome c oxidase subunit II	WP_011823245.:	455597..456409	Respiration	COG1622
WP_011823245.1_464	CUST_2503_PI442190912	NATL1	LLI	WP_011823245.1_464	TGTAATACATGCTTTTTGGGTGCCTCAATTCAGACTTAAACAAGATGTCATTCCTGGCCA	ctaC/coxB	Cytochrome c oxidase subunit II	WP_011823245.:	455597..456409	Respiration	COG1622
WP_011823253.1_471	CUST_2540_PI442190912	NATL1	LLI	WP_011823253.1_471	AAAAAGGAAGAATTATCAACATAACTTCTGTAGTTGGGTTGATGGGCAACCCAGGGCAAG	gdh	3-oxoacyl-acyl-carrier-protein reductase	WP_011823253.:	463082..463834	Entner-Dudoroff	COG1028
WP_011823253.1_471	CUST_2541_PI442190912	NATL1	LLI	WP_011823253.1_471	CTTCGGCGTCTTACATAACAGGTCAGGTAATTCAAGTTGATGGTGGGATGGTTATGAGTT	gdh	3-oxoacyl-acyl-carrier-protein reductase	WP_011823253.:	463082..463834	Entner-Dudoroff	COG1028
WP_011823253.1_471	CUST_2542_PI442190912	NATL1	LLI	WP_011823253.1_471	TTGTAACTGGCGCAAGCAGAGGTATTGGTAAAGCTATTGCAATTTTTCTAGCGAAGGAAG	gdh	3-oxoacyl-acyl-carrier-protein reductase	WP_011823253.:	463082..463834	Entner-Dudoroff	COG1028
WP_011823253.1_471	CUST_2543_PI442190912	NATL1	LLI	WP_011823253.1_471	CGCTGGGATAACTAAAGATGGCCTTTTAATGAGAATGAAAACGGACGATTGGCAGAAAGT	gdh	3-oxoacyl-acyl-carrier-protein reductase	WP_011823253.:	463082..463834	Entner-Dudoroff	COG1028
WP_011823253.1_471	CUST_2544_PI442190912	NATL1	LLI	WP_011823253.1_471	AAACTCCTTTGGAGGGAAGGCATACCCTCTTCAAGCTGATATTTCTAATGAAAACTCGGT	gdh	3-oxoacyl-acyl-carrier-protein reductase	WP_011823253.:	463082..463834	Entner-Dudoroff	COG1028
WP_011823253.1_471	CUST_2545_PI442190912	NATL1	LLI	WP_011823253.1_471	GTAGTAGGTCTCACACAAAGCGCTGCAAAAGAATTTGCCAGCAGAGGAATTACTGTAAAT	gdh	3-oxoacyl-acyl-carrier-protein reductase	WP_011823253.:	463082..463834	Entner-Dudoroff	COG1028
WP_011823313.1_537	CUST_2906_PI442190912	NATL1	LLI	WP_011823313.1_537	GGTCATGGGAGCAAGTTTCAGAAATATAGAGGAAATAACCGAGCTTGCAGGATGTGATCT	tal	Transaldolase	WP_011823313.:	522100..523098	Pentose Phosphate	COG0176
WP_011823313.1_537	CUST_2907_PI442190912	NATL1	LLI	WP_011823313.1_537	GCCGTGTTTCAACAGAGGTTGATGCAAGACTAAGTTTTGATACTGATGCGACCATTGAAA	tal	Transaldolase	WP_011823313.:	522100..523098	Pentose Phosphate	COG0176
WP_011823313.1_537	CUST_2908_PI442190912	NATL1	LLI	WP_011823313.1_537	CTTGTGCTGAGGCAGGTGTAACGCTTATTTCACCCTTTGTTGGAAGAATTTTAGATTGGT	tal	Transaldolase	WP_011823313.:	522100..523098	Pentose Phosphate	COG0176
WP_011823313.1_537	CUST_2909_PI442190912	NATL1	LLI	WP_011823313.1_537	GCCAAGGTTGATGTGGTTAAGGAAGCTTTGGATGAACTTTGTGTTGTCTTCGGTAAAGAG	tal	Transaldolase	WP_011823313.:	522100..523098	Pentose Phosphate	COG0176
WP_011823313.1_537	CUST_2910_PI442190912	NATL1	LLI	WP_011823313.1_537	GGAAGGGATTCTTATCCAGCGACTGAGGACCCAGGGGTAGTTTCGGTTACTAAAATATTT	tal	Transaldolase	WP_011823313.:	522100..523098	Pentose Phosphate	COG0176
WP_011823313.1_537	CUST_2911_PI442190912	NATL1	LLI	WP_011823313.1_537	TGATCGTGTTTTAATCAAAATTGCCTCTACGTGGGAGGGAATAAAAGCTGCTGAGGTATT	tal	Transaldolase	WP_011823313.:	522100..523098	Pentose Phosphate	COG0176
WP_011294510.1_565	CUST_3071_PI442190912	NATL1	LLI	WP_011294510.1_565	TAGCCCTGAGTTGGCAATTGCTCTTGAGACATGGAAGGAGATTAAGTTCGAATTCGATAC	rbcL	Ribulose 1,5-bisphosphate carboxylase large subunit	WP_011294510.:	549278..550690	Carbon fixation	COG1850
WP_011294510.1_565	CUST_3073_PI442190912	NATL1	LLI	WP_011294510.1_565	GTGGTATTCATGTTTGGCATATGCCAGCATTGCTTGCAATCTTTGGAGATGATTCATGTC	rbcL	Ribulose 1,5-bisphosphate carboxylase large subunit	WP_011294510.:	549278..550690	Carbon fixation	COG1850
WP_011294510.1_565	CUST_3074_PI442190912	NATL1	LLI	WP_011294510.1_565	CTCTTGGTTATATCGATAACCTACGTGAATCCTTTGTTCCTGAAGACCGTACTCGCGGTA	rbcL	Ribulose 1,5-bisphosphate carboxylase large subunit	WP_011294510.:	549278..550690	Carbon fixation	COG1850
WP_011294511.1_566	CUST_3077_PI442190912	NATL1	LLI	WP_011294511.1_566	TATCCTGACCATCACGTTCGTATCGTTGGATATGACGCGTATACACAAAGTCAAGGTACT	rbcS	Ribulose bisphosphate carboxylase small subunit	WP_011294511.:	550791..551132	Carbon fixation	COG4451
WP_011294511.1_566	CUST_3078_PI442190912	NATL1	LLI	WP_011294511.1_566	TTCTTTGGCGAGAAAGATTTAAATTTGGTTGTTAGCGAGTTAGAAGCT	rbcS	Ribulose bisphosphate carboxylase small subunit	WP_011294511.:	550791..551132	Carbon fixation	COG4451
WP_011294511.1_566	CUST_3079_PI442190912	NATL1	LLI	WP_011294511.1_566	CAGCCAAGTGGTTCAATGCAGACTTATTGGTCTTATTGGAAGTTACCTTTCTTTGGCGAG	rbcS	Ribulose bisphosphate carboxylase small subunit	WP_011294511.:	550791..551132	Carbon fixation	COG4451
WP_011294511.1_566	CUST_3080_PI442190912	NATL1	LLI	WP_011294511.1_566	GACGAAATCTACGATCAAATCGCTTATATCATTGCTCAGGGTTGGAGCCCGGTTATTGAG	rbcS	Ribulose bisphosphate carboxylase small subunit	WP_011294511.:	550791..551132	Carbon fixation	COG4451
WP_011294511.1_566	CUST_3081_PI442190912	NATL1	LLI	WP_011294511.1_566	CTTTCCAGAGCACAGTAGGTGACTATCAAACAGTCGCCACCCTGGAAACATTC	rbcS	Ribulose bisphosphate carboxylase small subunit	WP_011294511.:	550791..551132	Carbon fixation	COG4451
WP_011823376.1_612	CUST_3325_PI442190912	NATL1	LLI	WP_011823376.1_612	GGGTTATGGATTTCAGGTGGAACAGCCGCAAAAAACCTCGATGGAATAAACTCTTCTAAC	glk	Glucokinase	WP_011823376.:	597149..598192	Glycolysis	COG0837
WP_011823376.1_612	CUST_3326_PI442190912	NATL1	LLI	WP_011823376.1_612	GTGACAAATCAGATTCCACAGATTTACCCGCACTTGTTTGGGAAAAAGCAAAAAACGGAG	glk	Glucokinase	WP_011823376.:	597149..598192	Glycolysis	COG0837
WP_011823376.1_612	CUST_3327_PI442190912	NATL1	LLI	WP_011823376.1_612	ATTGTTAGTGGTACTGGCCTTGGCATGATTGCCAGATGGAAATTGGATGATCCAATAAAT	glk	Glucokinase	WP_011823376.:	597149..598192	Glycolysis	COG0837
WP_011823376.1_612	CUST_3328_PI442190912	NATL1	LLI	WP_011823376.1_612	ATGTCCAGAGGCTTGATAACCCCTAAAAGCATTTCTATATTTCCAAGTGAAGGAGGGCAT	glk	Glucokinase	WP_011823376.:	597149..598192	Glycolysis	COG0837
WP_011823376.1_612	CUST_3329_PI442190912	NATL1	LLI	WP_011823376.1_612	CATTGATTGTTCTTAAAGATCCAGAAGCGACATTATTCAGTTCAGCTTGCAGAGCACGCT	glk	Glucokinase	WP_011823376.:	597149..598192	Glycolysis	COG0837
WP_011823376.1_612	CUST_3330_PI442190912	NATL1	LLI	WP_011823376.1_612	CAAATTAATGACTGAAGCATTGCAACTATGGCTAAATGCTTATGGGTCTGCAGCTGGAGA	glk	Glucokinase	WP_011823376.:	597149..598192	Glycolysis	COG0837
WP_011823404.1_642	CUST_3502_PI442190912	NATL1	LLI	WP_011823404.1_642	TTTAAGGCAAACACCAACGATACCCGAGAATCACCTGCGATAAGAATTTCCAGTGACTTA	ugd	UDP-glucose 6-dehydrogenase/nucleotide sugar dehydrogenase	WP_011823404.:	629904..631307	Amino sugar and nucleotide sugar r	COG1004
WP_011823404.1_642	CUST_3503_PI442190912	NATL1	LLI	WP_011823404.1_642	GTGAGGCTACTGGAGCTGATGTTCAGGAAGTTGCTAAAGCAATAGGAACAGATAAAAGGA	ugd	UDP-glucose 6-dehydrogenase/nucleotide sugar dehydrogenase	WP_011823404.:	629904..631307	Amino sugar and nucleotide sugar r	COG1004
WP_011823404.1_642	CUST_3504_PI442190912	NATL1	LLI	WP_011823404.1_642	TCAATTTCTTAGTGCAGGTCCTGGGTTTGGTGGGAGCTGCTTTAAAAAGGATATTTTGAA	ugd	UDP-glucose 6-dehydrogenase/nucleotide sugar dehydrogenase	WP_011823404.:	629904..631307	Amino sugar and nucleotide sugar r	COG1004
WP_011823404.1_642	CUST_3505_PI442190912	NATL1	LLI	WP_011823404.1_642	GGATTACCAGAGGTTGCTAATTACTGGAATCAAGTTGTTGTGCTCAATACTTGGCAGCAA	ugd	UDP-glucose 6-dehydrogenase/nucleotide sugar dehydrogenase	WP_011823404.:	629904..631307	Amino sugar and nucleotide sugar r	COG1004
WP_011823404.1_642	CUST_3506_PI442190912	NATL1	LLI	WP_011823404.1_642	TGTTAATCGACAAGAAGTTGAAGATACAGGTGTAAATCTTTGGAAGCTGGGTGAGGGGAA	ugd	UDP-glucose 6-dehydrogenase/nucleotide sugar dehydrogenase	WP_011823404.:	629904..631307	Amino sugar and nucleotide sugar r	COG1004
WP_011823404.1_642	CUST_3507_PI442190912	NATL1	LLI	WP_011823404.1_642	TCAAGGGATTTGGAAAATGGCCACTTCTATTCCAGAAGCATTGAAAAATGTTGATGCAGT	ugd	UDP-glucose 6-dehydrogenase/nucleotide sugar dehydrogenase	WP_011823404.:	629904..631307	Amino sugar and nucleotide sugar r	COG1004
WP_011823483.1_723	CUST_3934_PI442190912	NATL1	LLI	WP_011823483.1_723	CAAAAATCAAATTGATCCTGGAGATGGGGTTGACGTTGACCTTTTTGAGCTTCCTTCAGA	glnA	Glutamine synthetase/ glutamate--ammonialigase	WP_011823483.:	705143..706564	Nitrogen Metabolism	COG0174
WP_011823483.1_723	CUST_3935_PI442190912	NATL1	LLI	WP_011823483.1_723	GTGGAATACTCAAACATGCACCGTCTTTCCTTGCTTTTACCAATCCAACTACCAACAGTT	glnA	Glutamine synthetase/ glutamate--ammonialigase	WP_011823483.:	705143..706564	Nitrogen Metabolism	COG0174
WP_011823483.1_723	CUST_3936_PI442190912	NATL1	LLI	WP_011823483.1_723	TTTAGTTTACTCACAGGGCAATCGATCAGCTGCTGTAAGAATACCTTTGACTGGACCAAG	glnA	Glutamine synthetase/ glutamate--ammonialigase	WP_011823483.:	705143..706564	Nitrogen Metabolism	COG0174
WP_011823483.1_723	CUST_3937_PI442190912	NATL1	LLI	WP_011823483.1_723	CAGTCTTCAATGACAATGGGACAGGAATGCATGTTCATCAAAGTCTTTGGAAAGGAGGTC	glnA	Glutamine synthetase/ glutamate--ammonialigase	WP_011823483.:	705143..706564	Nitrogen Metabolism	COG0174
WP_011823483.1_723	CUST_3938_PI442190912	NATL1	LLI	WP_011823483.1_723	AGCTGGTCAACATGAATTAGGAATGAAATTTGCTCCCTTAATCAACGCAGCTGACAATGT	glnA	Glutamine synthetase/ glutamate--ammonialigase	WP_011823483.:	705143..706564	Nitrogen Metabolism	COG0174
WP_011823483.1_723	CUST_3939_PI442190912	NATL1	LLI	WP_011823483.1_723	TTAGATCAGGTGACGCATTAGCTAACCCTTACATTGCTTTTTCAGCAATGATGATGGCAG	glnA	Glutamine synthetase/ glutamate--ammonialigase	WP_011823483.:	705143..706564	Nitrogen Metabolism	COG0174
WP_011823513.1_756	CUST_4127_PI442190912	NATL1	LLI	WP_011823513.1_756	TGGTATTCCCTTGGGAGTGGGACAAGGCACAACTGTTAAAAGAGCTATTCTCGATAAAAA	glgC	Glucose-1-phosphate adenylyltransferase	WP_011823513.:	743772..745067	Glycogen Metabolism	COG0448
WP_011823513.1_756	CUST_4128_PI442190912	NATL1	LLI	WP_011823513.1_756	GGTGACAACGTGACGATAGTCAATAAAGACAATGTGGAAGAAGCTGATCGAGCAGATCAA	glgC	Glucose-1-phosphate adenylyltransferase	WP_011823513.:	743772..745067	Glycogen Metabolism	COG0448
WP_011823513.1_756	CUST_4129_PI442190912	NATL1	LLI	WP_011823513.1_756	CGTGAAAAACCAACTGGTGACTCATTGAAAGCAATGGCAGTAGATACATCAAGGTTTGGA	glgC	Glucose-1-phosphate adenylyltransferase	WP_011823513.:	743772..745067	Glycogen Metabolism	COG0448
WP_011823513.1_756	CUST_4130_PI442190912	NATL1	LLI	WP_011823513.1_756	TCATTGTGTGTTGGGAGTTAGGAGTCGGATTGAAAGTGACGTTGTTCTAAATGAAACCCT	glgC	Glucose-1-phosphate adenylyltransferase	WP_011823513.:	743772..745067	Glycogen Metabolism	COG0448
WP_011823513.1_756	CUST_4131_PI442190912	NATL1	LLI	WP_011823513.1_756	GGAACTATGGTGCATTCTTTGAATCAAATTTAGCCTTAACTCAGCAACCTACCCCTTTCCG	glgC	Glucose-1-phosphate adenylyltransferase	WP_011823513.:	743772..745067	Glycogen Metabolism	COG0448
WP_011823513.1_756	CUST_4132_PI442190912	NATL1	LLI	WP_011823513.1_756	GGCTTTTATATCCGTAATGGAATAGTTGTTATCGTCAAAAACGCCACAATTCCAGATGGA	glgC	Glucose-1-phosphate adenylyltransferase	WP_011823513.:	743772..745067	Glycogen Metabolism	COG0448
WP_011293710.1_757	CUST_4133_PI442190912	NATL1	LLI	WP_011293710.1_757	GAGAGACTGTTTTGGCTCTCATACATATGAGCGCGTAGACAAGGCTGGATCATTTCATAC	edd	phosphogluconate dehydrogenase (NADP(+)-dependent, decarboxyla	WP_011293710.:	745227..746645	Pentose Phosphate/Entner-Dudorof	COG2084
WP_011293710.1_757	CUST_4134_PI442190912	NATL1	LLI	WP_011293710.1_757	TATCGATAAATGGTTTACCGATCAGGTAAATAATCGTCTCTCTGGCCTGACACAGGTCGT	edd	phosphogluconate dehydrogenase (NADP(+)-dependent, decarboxyla	WP_011293710.:	745227..746645	Pentose Phosphate/Entner-Dudorof	COG2084
WP_011293710.1_757	CUST_4135_PI442190912	NATL1	LLI	WP_011293710.1_757	TATGCCCTCCATTGCTCAAATATGGAAAGGTGGTTGCATAATAAGGTCAACCCTTTGAG	edd	phosphogluconate dehydrogenase (NADP(+)-dependent, decarboxyla	WP_011293710.:	745227..746645	Pentose Phosphate/Entner-Dudorof	COG2084
WP_011293710.1_757	CUST_4136_PI442190912	NATL1	LLI	WP_011293710.1_757	GGATGCCGTTGTATTGGCTACAATAGCTAGCTATGCCCAAGGTATGGATATTTTACGACT	edd	phosphogluconate dehydrogenase (NADP(+)-dependent, decarboxyla	WP_011293710.:	745227..746645	Pentose Phosphate/Entner-Dudorof	COG2084
WP_011293710.1_757	CUST_4137_PI442190912	NATL1	LLI	WP_011293710.1_757	ATAAAGCGGGTCAAAAAGGTACTGGATTATGGACAGTCGTTAGTGCATTAGAGCTTGGAG	edd	phosphogluconate dehydrogenase (NADP(+)-dependent, decarboxyla	WP_011293710.:	745227..746645	Pentose Phosphate/Entner-Dudorof	COG2084
WP_011293710.1_757	CUST_4138_PI442190912	NATL1	LLI	WP_011293710.1_757	ATGAAGAGAGTTTGTGGAATGAGTGGTGATGAGATGGCATCTGTTATGGGCTATTGGAAT	edd	phosphogluconate dehydrogenase (NADP(+)-dependent, decarboxyla	WP_011293710.:	745227..746645	Pentose Phosphate/Entner-Dudorof	COG2084
WP_011823524.1_769	CUST_4197_PI442190912	NATL1	LLI	WP_011823524.1_769	GGTCTAAGACCATTAGCCGGTGGACATGATGTAGAAACTTTTTGTTCAGGTTTAGACGGT	fda	Fructose-1,6-bisphosphate aldolase class I	WP_011823524.:	754079..755146	Glycolysis/Gluconeogenesis	COG0191
WP_011823524.1_769	CUST_4198_PI442190912	NATL1	LLI	WP_011823524.1_769	TGTCTAAAAGCATGGAAAGGCTCGAACACTTCTGATGGACAAAAAGCACTCATAGCTAGA	fda	Fructose-1,6-bisphosphate aldolase class I	WP_011823524.:	754079..755146	Glycolysis/Gluconeogenesis	COG0191
WP_011823524.1_769	CUST_4199_PI442190912	NATL1	LLI	WP_011823524.1_769	CCCTGGTATTACTTTCCTTTCAGGTGGATTGAGCGAGGAAGCTGCATCAGTTTATCTAAA	fda	Fructose-1,6-bisphosphate aldolase class I	WP_011823524.:	754079..755146	Glycolysis/Gluconeogenesis	COG0191
WP_011823524.1_769	CUST_4200_PI442190912	NATL1	LLI	WP_011823524.1_769	TGCAAGATTTGCCAAGTGGAGAGCAGTACTTCAAATAACAGACGATGGTTGTCCTTCTAA	fda	Fructose-1,6-bisphosphate aldolase class I	WP_011823524.:	754079..755146	Glycolysis/Gluconeogenesis	COG0191
WP_011823524.1_769	CUST_4201_PI442190912	NATL1	LLI	WP_011823524.1_769	TTAGCTTGCCAGGTAAATGGAGTACTTCTAGAGGGAACTCTTCTGAAGCCATCAATGACT	fda	Fructose-1,6-bisphosphate aldolase class I	WP_011823524.:	754079..755146	Glycolysis/Gluconeogenesis	COG0191
WP_011823524.1_769	CUST_4202_PI442190912	NATL1	LLI	WP_011823524.1_769	TAGAGAAAATGCTTGGGGTTTAGCAAGATACGCTAGATCAGTTCAAGAATCTGGCTTGGT	fda	Fructose-1,6-bisphosphate aldolase class I	WP_011823524.:	754079..755146	Glycolysis/Gluconeogenesis	COG0191
WP_011823529.1_774	CUST_4224_PI442190912	NATL1	LLI	WP_011823529.1_774	AAAACTGGCGGAGGTCAGAAAAGATATTATTTACATAGCCCAGAAGAAGCCGAGGAACAT	prk	Phosphoribulokinase	WP_011823529.:	758288..759190	Carbon Fixation	COG3954
WP_011823529.1_774	CUST_4225_PI442190912	NATL1	LLI	WP_011823529.1_774	AGGAACCGACCTTTTGTTTTACGAGGGACTACACGGTGGAGTAGTAGGAAAAGATTATGA	prk	Phosphoribulokinase	WP_011823529.:	758288..759190	Carbon Fixation	COG3954
WP_011823529.1_774	CUST_4226_PI442190912	NATL1	LLI	WP_011823529.1_774	AAAAGGCAATGTCTGAGGCACTTTCAAGAGGTGAAAACTTTTCTCATTTTGGACCAGAGG	prk	Phosphoribulokinase	WP_011823529.:	758288..759190	Carbon Fixation	COG3954
WP_011823529.1_774	CUST_4227_PI442190912	NATL1	LLI	WP_011823529.1_774	TAACGCAGAAAGGGGATACTCAGCCGAAACTATCGTAGATACGATTCTGAGAAGAATGCC	prk	Phosphoribulokinase	WP_011823529.:	758288..759190	Carbon Fixation	COG3954
WP_011823529.1_774	CUST_4228_PI442190912	NATL1	LLI	WP_011823529.1_774	CATGTCAAGTCCTACAAGTATTGTTGTTAACGGAGGGAAAATGGGATTTGCTATGGAGCT	prk	Phosphoribulokinase	WP_011823529.:	758288..759190	Carbon Fixation	COG3954
WP_011823529.1_774	CUST_4229_PI442190912	NATL1	LLI	WP_011823529.1_774	TGCCCCCAATTCAGTCGAACAGATATCAACTTTCAGAGAGTACCTACAATTGATACGTCA	prk	Phosphoribulokinase	WP_011823529.:	758288..759190	Carbon Fixation	COG3954
WP_011823556.1_802	CUST_4381_PI442190912	NATL1	LLI	WP_011823556.1_802	AATTTATGGAAAATTTGTTCCTTGCGTTATTTTAATCCTGTTGGTGCGCACCCTTCTGGC	galE	UDP-glucose 4-epimerase	WP_011823556.:	783035..784081	Galactose metabolism	COG1087
WP_011823556.1_802	CUST_4382_PI442190912	NATL1	LLI	WP_011823556.1_802	TGAAAGTCGAAGAGATGGTGATGTGGCAGTCTCTTACGCAGATATATCAAAAGCCAAGAG	galE	UDP-glucose 4-epimerase	WP_011823556.:	783035..784081	Galactose metabolism	COG1087
WP_011823556.1_802	CUST_4383_PI442190912	NATL1	LLI	WP_011823556.1_802	AGTTGGACTCCTAAGAGATCCTTAGAACAGATTTGCCTAGATGGATGGAATTGGCAAATT	galE	UDP-glucose 4-epimerase	WP_011823556.:	783035..784081	Galactose metabolism	COG1087

WP_011823556.1_802	CUST_4384_PI442190912	NATL1	LLI	WP_011823556.1_802	GGCTCTGGTAAAGGGTATTCTGTATTTCAGATTATTCGGCAATTTGAGTTGTCTACAGGG	galE	UDP-glucose 4-epimerase	WP_011823556.: 783035..784081	Galactose metabolism	COG1087
WP_011823556.1_802	CUST_4385_PI442190912	NATL1	LLI	WP_011823556.1_802	AAACATTTATGGAGATGATTGGGAAACAAAGGATGGTTCAGGGATTCGTGATTATGTTCA	galE	UDP-glucose 4-epimerase	WP_011823556.: 783035..784081	Galactose metabolism	COG1087
WP_011823556.1_802	CUST_4386_PI442190912	NATL1	LLI	WP_011823556.1_802	TTGGCGAGGATCCAAGAGGAATTCCTAATAATCTGTTCCCTTTTATAACTCAAGTAGCAA	galE	UDP-glucose 4-epimerase	WP_011823556.: 783035..784081	Galactose metabolism	COG1087
WP_011823581.1_826	CUST_4525_PI442190912	NATL1	LLI	WP_011823581.1_826	CATTAAGAGCAATAACTGAACCATTTGCGGGCATTTTTGCCACCAAAGAGTTAATGGAGG		polysaccharide biosynthesis/export family protein	WP_011823581.: 816931..818178	Other sugar transporters	COG1596
WP_011823581.1_826	CUST_4526_PI442190912	NATL1	LLI	WP_011823581.1_826	TCAATACTGTCCGGTCTATCTCCCAGGTTTATAAATGTATCAGTTGGGGGGCGAATAGAA		polysaccharide biosynthesis/export family protein	WP_011823581.: 816931..818178	Other sugar transporters	COG1596
WP_011823581.1_826	CUST_4527_PI442190912	NATL1	LLI	WP_011823581.1_826	AGAAATTACCAGAGAAATACCTCTTGGGAACGGAGGTGGCAAAAAACGAGCGATAGTTAA		polysaccharide biosynthesis/export family protein	WP_011823581.: 816931..818178	Other sugar transporters	COG1596
WP_011823581.1_826	CUST_4528_PI442190912	NATL1	LLI	WP_011823581.1_826	TGAAGGAAGTCTTTCTGATGCAATGAATTTAACAGGTCCAAGGAAGCCTTTGTCAGGAGA		polysaccharide biosynthesis/export family protein	WP_011823581.: 816931..818178	Other sugar transporters	COG1596
WP_011823581.1_826	CUST_4529_PI442190912	NATL1	LLI	WP_011823581.1_826	TTCTTCAAGTGCCCCTCCAGGATCTCCAAAGAATCCATACTTATTAGCTGGTGATTCAAT		polysaccharide biosynthesis/export family protein	WP_011823581.: 816931..818178	Other sugar transporters	COG1596
WP_011823581.1_826	CUST_4530_PI442190912	NATL1	LLI	WP_011823581.1_826	TGCAGACGCCTCATCAGATATGAGACTATTTGATGGAGACGATATCTTTATTCCTCGTCT		polysaccharide biosynthesis/export family protein	WP_011823581.: 816931..818178	Other sugar transporters	COG1596
WP_011823642.1_892	CUST_4877_PI442190912	NATL1	LLI	WP_011823642.1_892	GTCTGTGCCCTAGATCAAGCCGATTTAGTAAAGCAGGGAAGCGACATTACTATTTTGACT	pdhB	Pyruvate dehydrogenase E1 component beta subunit	WP_011823642.: 875727..876716	Glycolysis/Krebs cycle	COG0022
WP_011823642.1_892	CUST_4878_PI442190912	NATL1	LLI	WP_011823642.1_892	CAATTAGGCGCTGAACACAGTCAAAGACTTGAAGCCTATTTTCATGCAGTTCCTGGTATC	pdhB	Pyruvate dehydrogenase E1 component beta subunit	WP_011823642.: 875727..876716	Glycolysis/Krebs cycle	COG0022
WP_011823642.1_892	CUST_4879_PI442190912	NATL1	LLI	WP_011823642.1_892	TGGGAATGCTTAGATATACGAGCGGGGGAAATTTTACTATTCCAACAGTTGTAAGAGGCC	pdhB	Pyruvate dehydrogenase E1 component beta subunit	WP_011823642.: 875727..876716	Glycolysis/Krebs cycle	COG0022
WP_011823642.1_892	CUST_4880_PI442190912	NATL1	LLI	WP_011823642.1_892	TGCTTCGATGATTTAGATTCTCCTCCTGTTCGTTTAAGCAGTCAGGATATTCCGACTCCT	pdhB	Pyruvate dehydrogenase E1 component beta subunit	WP_011823642.: 875727..876716	Glycolysis/Krebs cycle	COG0022
WP_011823642.1_892	CUST_4881_PI442190912	NATL1	LLI	WP_011823642.1_892	TATTCGAGAATGCGTCACCACTGTTTAAAAGCAGTCGAACTTCTTGAGGCTAAGGGAATT	pdhB	Pyruvate dehydrogenase E1 component beta subunit	WP_011823642.: 875727..876716	Glycolysis/Krebs cycle	COG0022
WP_011823642.1_892	CUST_4882_PI442190912	NATL1	LLI	WP_011823642.1_892	TGACAGGTTTAAGACCAATTGTGGAAGGGATGAATATGGGTTTTTTGTTGCTTGCGTTT	pdhB	Pyruvate dehydrogenase E1 component beta subunit	WP_011823642.: 875727..876716	Glycolysis/Krebs cycle	COG0022
WP_011823654.1_906	CUST_4944_PI442190912	NATL1	LLI	WP_011823654.1_906	TCCCACTCCAGTATTAGCAGTAACTAATGAAAAGAGTGTTGCCAGCACACTTCAACTTGT	pykF	Pyruvate kinase	WP_011823654.: 888112..889569	Glycolysis	COG0469
WP_011823654.1_906	CUST_4945_PI442190912	NATL1	LLI	WP_011823654.1_906	ACACTATCAGGGGTAAGTGGGTCAACAGACTTGGTAAAGGTTGGGATAGTTAGTTCAGAA	pykF	Pyruvate kinase	WP_011823654.: 888112..889569	Glycolysis	COG0469
WP_011823654.1_906	CUST_4946_PI442190912	NATL1	LLI	WP_011823654.1_906	TCATCTTCCAAGCACTATTCCAAATGCTATTAGTGCGGCTGTAAGCAGTATTGCTAGACA	pykF	Pyruvate kinase	WP_011823654.: 888112..889569	Glycolysis	COG0469
WP_011823654.1_906	CUST_4947_PI442190912	NATL1	LLI	WP_011823654.1_906	ACGCTGTCTTAAAATTATGTGATGGAGTCATGGTTGCTAGAGGTGATCTCGGTGTAGAGA	pykF	Pyruvate kinase	WP_011823654.: 888112..889569	Glycolysis	COG0469
WP_011823654.1_906	CUST_4948_PI442190912	NATL1	LLI	WP_011823654.1_906	TCAAAAAGGCAAACAGCCTTGGCATACCAATAATTACAGCCACACAAATGCTTGATTCAA	pykF	Pyruvate kinase	WP_011823654.: 888112..889569	Glycolysis	COG0469
WP_011823654.1_906	CUST_4949_PI442190912	NATL1	LLI	WP_011823654.1_906	CTGTGGCGACTATGGCTACTATTGCGAAAAGAATCGAACGAGATTATCCACAAAAAGCAT	pykF	Pyruvate kinase	WP_011823654.: 888112..889569	Glycolysis	COG0469
WP_011823677.1_931	CUST_5082_PI442190912	NATL1	LLI	WP_011823677.1_931	GAATTGATTAATGTTAATGCCTACAATCAACCGGGAGTTGAAGCAGGTAAAGAAGCGGCT	pgi	Glucose-6-phosphate isomerase	WP_011823677.: 912282..913880	Glycolysis/Gluconeogenesis	COG0166
WP_011823677.1_931	CUST_5083_PI442190912	NATL1	LLI	WP_011823677.1_931	AAGACCAGGTGACTATTTGTCTGGATTTCTTCAAGGAACTCGTTCAGCATTGACTGAAGG	pgi	Glucose-6-phosphate isomerase	WP_011823677.: 912282..913880	Glycolysis/Gluconeogenesis	COG0166
WP_011823677.1_931	CUST_5084_PI442190912	NATL1	LLI	WP_011823677.1_931	AGCAGCACTTTTGTCTTTAGCTTGGTTCAAATCCGGAAATGGAAAAGGGTTAAGGGATAT	pgi	Glucose-6-phosphate isomerase	WP_011823677.: 912282..913880	Glycolysis/Gluconeogenesis	COG0166
WP_011823677.1_931	CUST_5085_PI442190912	NATL1	LLI	WP_011823677.1_931	AGGTATCGCAGTCTATGGAAACAAAGGATCTACAGATCAGCATGCATATGTGCAGCAATT	pgi	Glucose-6-phosphate isomerase	WP_011823677.: 912282..913880	Glycolysis/Gluconeogenesis	COG0166
WP_011823677.1_931	CUST_5086_PI442190912	NATL1	LLI	WP_011823677.1_931	TGATGAATCAAGTCTTGGTGCTTTAATTGCATTGTTTGAGCGAGCTGTAAGTCTCTATGC	pgi	Glucose-6-phosphate isomerase	WP_011823677.: 912282..913880	Glycolysis/Gluconeogenesis	COG0166
WP_011823677.1_931	CUST_5087_PI442190912	NATL1	LLI	WP_011823677.1_931	TCTTCAACAACTTGTGATGGAATCTTTAGGCAAGAAGTTTGATAGAGATGAAAATCAAGT	pgi	Glucose-6-phosphate isomerase	WP_011823677.: 912282..913880	Glycolysis/Gluconeogenesis	COG0166
WP_011824089.1_1334	CUST_6828_PI442190912	NATL1	LLI	WP_011824089.1_1334	CCGAAACATATGTAGCTATGAAGCTTTTCATAGATAATTGGAGATGGCAGGGGGTCCCTT	zwf	glucose-6-phosphate dehydrogenase zwf	WP_011824089.: 1222696..1224219	Pentose Phosphate	COG0364
WP_011824089.1_1334	CUST_6829_PI442190912	NATL1	LLI	WP_011824089.1_1334	GGAATGTTGCGTTAGAGGTCAATATTCAAAGGGAGGTAGCGATGAAGAACCACTCTTAGG	zwf	glucose-6-phosphate dehydrogenase zwf	WP_011824089.: 1222696..1224219	Pentose Phosphate	COG0364
WP_011824089.1_1334	CUST_6830_PI442190912	NATL1	LLI	WP_011824089.1_1334	TGATGAATCCTTTGGAGAACCATCAGATGAAGGTTATGTGAGACTCCTTGCTGATGCAAT	zwf	glucose-6-phosphate dehydrogenase zwf	WP_011824089.: 1222696..1224219	Pentose Phosphate	COG0364
WP_011824089.1_1334	CUST_6831_PI442190912	NATL1	LLI	WP_011824089.1_1334	ACCTGGGCATTTTGATCCAGAAGCCATAAGAAATGAAAAAGCTAAAGTTCTTCAGGCAGT	zwf	glucose-6-phosphate dehydrogenase zwf	WP_011824089.: 1222696..1224219	Pentose Phosphate	COG0364
WP_011824089.1_1334	CUST_6832_PI442190912	NATL1	LLI	WP_011824089.1_1334	ACCGGAAAACGTTTAGCTAAAAGGCTTAGTGAGGTTGTTCTCACATTTAGAGAAGCACCT	zwf	glucose-6-phosphate dehydrogenase zwf	WP_011824089.: 1222696..1224219	Pentose Phosphate	COG0364
WP_011824089.1_1334	CUST_6833_PI442190912	NATL1	LLI	WP_011824089.1_1334	ATCCAACATTGTTTACTCGAAGCGATGAAGTAGAGGCTGCTTGGCGTTTATATACTCCTC	zwf	glucose-6-phosphate dehydrogenase zwf	WP_011824089.: 1222696..1224219	Pentose Phosphate	COG0364
WP_011824090.1_1335	CUST_6834_PI442190912	NATL1	LLI	WP_011824090.1_1335	GTGGAATGGAAGAATAGATGAGGCTCCTGAATTCCTCAATGCACTATCTCTCCCAAATCG	opcA	Glucose-6-P dehydrogenase subunit opcA	WP_011824090.: 1224258..1225565	Pentose Phosphate	COG3429
WP_011824090.1_1335	CUST_6835_PI442190912	NATL1	LLI	WP_011824090.1_1335	CGATCGTCTCAACTGGAAACTCGATGGTCGGATCTCCTCAAAAAAAGATCAACTGAAAGT	opcA	Glucose-6-P dehydrogenase subunit opcA	WP_011824090.: 1224258..1225565	Pentose Phosphate	COG3429
WP_011824090.1_1335	CUST_6836_PI442190912	NATL1	LLI	WP_011824090.1_1335	ATTGCTTCAAAGAATCCAATCTGGACAAGCTGTTAACGACCTAAATTGGCTTCGCCTTAG	opcA	Glucose-6-P dehydrogenase subunit opcA	WP_011824090.: 1224258..1225565	Pentose Phosphate	COG3429
WP_011824090.1_1335	CUST_6837_PI442190912	NATL1	LLI	WP_011824090.1_1335	GAGATTAGAAGCGGGAGGGATGGCAAGAATGGAACTTATTGAACAAGTCGTTCCTATTCA	opcA	Glucose-6-P dehydrogenase subunit opcA	WP_011824090.: 1224258..1225565	Pentose Phosphate	COG3429
WP_011824090.1_1335	CUST_6838_PI442190912	NATL1	LLI	WP_011824090.1_1335	TGGTAAGCCAAGCATCAATCCTGGTCAAATAGTTGGTCTGAGATTAATTGCAAAAGCCAA	opcA	Glucose-6-P dehydrogenase subunit opcA	WP_011824090.: 1224258..1225565	Pentose Phosphate	COG3429
WP_011824090.1_1335	CUST_6839_PI442190912	NATL1	LLI	WP_011824090.1_1335	TATCAATGATGGGCTTGAAATTGTAGAGAATCTTGTTCCCGAGGAACTTCCATCTTGGCT	opcA	Glucose-6-P dehydrogenase subunit opcA	WP_011824090.: 1224258..1225565	Pentose Phosphate	COG3429
WP_011824120.1_1367	CUST_7012_PI442190912	NATL1	LLI	WP_011824120.1_1367	CATTACCGCTACTGACAAAGAAGGAATAAGATTCGCTAGGAAAGCCAGATTCCTCTCTGC	rbsK	Sugar kinase, ribokinase family	WP_011824120.: 1257194..1258036	Pentose Phosphate	COG0524
WP_011824120.1_1367	CUST_7013_PI442190912	NATL1	LLI	WP_011824120.1_1367	TTAGCGGTGATCGGTCATGTTGAATGGGTAACATTCTTAAAGGTTGATCAGCTCCCATTA	rbsK	Sugar kinase, ribokinase family	WP_011824120.: 1257194..1258036	Pentose Phosphate	COG0524
WP_011824120.1_1367	CUST_7014_PI442190912	NATL1	LLI	WP_011824120.1_1367	TGCTCTCTCAGCAAAGCTAAATTTAGAGCAAGCGATTAATATAGGTGCATATTGCGGTGC	rbsK	Sugar kinase, ribokinase family	WP_011824120.: 1257194..1258036	Pentose Phosphate	COG0524
WP_011824120.1_1367	CUST_7015_PI442190912	NATL1	LLI	WP_011824120.1_1367	TAGAAGTAGCTTGGCGAGAGAAACCCACCAGAAAAGGTATTAGCCTAATCAGCAAAGATG	rbsK	Sugar kinase, ribokinase family	WP_011824120.: 1257194..1258036	Pentose Phosphate	COG0524
WP_011824120.1_1367	CUST_7016_PI442190912	NATL1	LLI	WP_011824120.1_1367	CCCCGAACAGGTCAACAAACCCTAAAGGATTCAAAAGTAAAACTCAATGCATTGATTGGC	rbsK	Sugar kinase, ribokinase family	WP_011824120.: 1257194..1258036	Pentose Phosphate	COG0524
WP_011824120.1_1367	CUST_7017_PI442190912	NATL1	LLI	WP_011824120.1_1367	TACAAGCCCATTACGCCTAGTTCTAAAGAAATAGACACCTATGGTTGTGGGGACAGTTTC	rbsK	Sugar kinase, ribokinase family	WP_011824120.: 1257194..1258036	Pentose Phosphate	COG0524
WP_041700622.1_1517	CUST_7781_PI442190912	NATL1	LLI	WP_041700622.1_1517	CCTTTATTGGTGAAGGTATTCCAGTTGCCCTTGGAGCAGCTTTCAGCAGTAAATACAAAA	pdhA	Pyruvate dehydrogenase E1 component alpha subunit	WP_041700622.: 1393089..1394177	Glycolysis/Krebs cycle	COG1071
WP_041700622.1_1517	CUST_7782_PI442190912	NATL1	LLI	WP_041700622.1_1517	AATTTGCTTTAAACGCCCCAGAGCCTGACCCTAGTGAGTTAACAAAATATATTTGGGCAG	pdhA	Pyruvate dehydrogenase E1 component alpha subunit	WP_041700622.: 1393089..1394177	Glycolysis/Krebs cycle	COG1071
WP_041700622.1_1517	CUST_7783_PI442190912	NATL1	LLI	WP_041700622.1_1517	GTAGTCGAAAATAATAAATGGGCTATTGGGATGGCCCATGACAGAGCAACTAGTGAGACA	pdhA	Pyruvate dehydrogenase E1 component alpha subunit	WP_041700622.: 1393089..1394177	Glycolysis/Krebs cycle	COG1071
WP_041700622.1_1517	CUST_7784_PI442190912	NATL1	LLI	WP_041700622.1_1517	CTGACCCAGACGAGCTTAGATCTGAAAAGGAAAAGGAATTCTGGGCTAAAAGAGATCCAA	pdhA	Pyruvate dehydrogenase E1 component alpha subunit	WP_041700622.: 1393089..1394177	Glycolysis/Krebs cycle	COG1071
WP_041700622.1_1517	CUST_7785_PI442190912	NATL1	LLI	WP_041700622.1_1517	GTTGCAGCAAAGGCAGAGGTGGATCTATGCATCTGTTCTCTAAAGAACATCACCTACTTG	pdhA	Pyruvate dehydrogenase E1 component alpha subunit	WP_041700622.: 1393089..1394177	Glycolysis/Krebs cycle	COG1071
WP_041700622.1_1517	CUST_7786_PI442190912	NATL1	LLI	WP_041700622.1_1517	AGAAATATGGAGAAAAGCTTCAGCATTTGGCATGCACGGAGAAGAGATTGACGGTATG	pdhA	Pyruvate dehydrogenase E1 component alpha subunit	WP_041700622.: 1393089..1394177	Glycolysis/Krebs cycle	COG1071
WP_049752910.1_1537	CUST_7898_PI442190912	NATL1	LLI	WP_049752910.1_1537	TCTGGAAGATCAAGAGCTTTGGAAGCAGCTCAAGCAGCAATCAATAGTCCTCTTTTAGAA	ftsZ	cell division protein FtsZ	WP_049752910.: 1415566..1416669	Cell Division	COG0206
WP_049752910.1_1537	CUST_7899_PI442190912	NATL1	LLI	WP_049752910.1_1537	GGTAAATGTAGATTTTGCTGATGTGCGTTCTGTTATGACTGAAGCTGGTACTGCGCTTTT	ftsZ	cell division protein FtsZ	WP_049752910.: 1415566..1416669	Cell Division	COG0206
WP_049752910.1_1537	CUST_7900_PI442190912	NATL1	LLI	WP_049752910.1_1537	ATCGTTGGTACAGTTGTTGACGAAAAACTTGAGGGAGAGATTCAGGTAACCGTTATTGCT	ftsZ	cell division protein FtsZ	WP_049752910.: 1415566..1416669	Cell Division	COG0206
WP_049752910.1_1537	CUST_7901_PI442190912	NATL1	LLI	WP_049752910.1_1537	TGAAAGGACTAGAGCAAGACTTTCACCTAAATCCCTTTACGAACAACCAGAAGAGAGGGA	ftsZ	cell division protein FtsZ	WP_049752910.: 1415566..1416669	Cell Division	COG0206
WP_049752910.1_1537	CUST_7902_PI442190912	NATL1	LLI	WP_049752910.1_1537	AGACGTAATTTCAGGAGCTCCTTTGCAAGAAGCCTTCAGAAGTGCTGATGATGTTCTTAT	ftsZ	cell division protein FtsZ	WP_049752910.: 1415566..1416669	Cell Division	COG0206
WP_049752910.1_1537	CUST_7903_PI442190912	NATL1	LLI	WP_049752910.1_1537	CGTCGTTTAAGACAAGCTGACGAAGGTATCGCAAGGCTTGCAGAGAATGTTGATACTTTA	ftsZ	cell division protein FtsZ	WP_049752910.: 1415566..1416669	Cell Division	COG0206
WP_041700719.1_1556	CUST_8001_PI442190912	NATL1	LLI	WP_041700719.1_1556	GCCCCCAATAACTAAAGGAGTCAGTATCAATTCTTTATTAGCCTTTGGTGATATTGAAGG	glcH	GPH family sugar transporter	WP_041700719.: 1432092..1433447	GlcH sugar transporter	COG2211
WP_011824320.1_1575	CUST_8108_PI442190912	NATL1	LLI	WP_011824320.1_1575	GAGGTGAAATGGCAAGAGCAATAAATGTTTTTAAGATGCGCGGATCTTGGCATGACAAAA	kaiC	circadian clock protein KaiC	WP_011824320.: 1451777..1453279	Circadian Rhythm	COG0467
WP_011824320.1_1575	CUST_8109_PI442190912	NATL1	LLI	WP_011824320.1_1575	GAGCACAACTTCTTCGAAATGCAACTAGCTGGGGGATTGATTTTGAAAAAATGGAAGCTG	kaiC	circadian clock protein KaiC	WP_011824320.: 1451777..1453279	Circadian Rhythm	COG0467
WP_011824320.1_1575	CUST_8110_PI442190912	NATL1	LLI	WP_011824320.1_1575	TCGAATAAGTTCTGGTGTTCCCGCTTTGGATGAAATGTGTGGAGGAGGATATTTTCAAGA	kaiC	circadian clock protein KaiC	WP_011824320.: 1451777..1453279	Circadian Rhythm	COG0467
WP_011824320.1_1575	CUST_8111_PI442190912	NATL1	LLI	WP_011824320.1_1575	GAACAGATATTTAGTGGTGCCCCTCATCGAATTAATCCTGAAGAACAGATACCTGGCGTT	kaiC	circadian clock protein KaiC	WP_011824320.: 1451777..1453279	Circadian Rhythm	COG0467
WP_011824320.1_1575	CUST_8112_PI442190912	NATL1	LLI	WP_011824320.1_1575	CATCCAGAATGGCTATAGATTCTCTATCCGCATTGGCTAGAGGTGTCAGCTTAAATGCAT	kaiC	circadian clock protein KaiC	WP_011824320.: 1451777..1453279	Circadian Rhythm	COG0467
WP_011824320.1_1575	CUST_8113_PI442190912	NATL1	LLI	WP_011824320.1_1575	TTGCAACTGGTGCAACTGGTACCGGAAAAACAATGCTAGTTTCAAAGTTTGTTGAAGATG	kaiC	circadian clock protein KaiC	WP_011824320.: 1451777..1453279	Circadian Rhythm	COG0467
WP_011824321.1_1576	CUST_8114_PI442190912	NATL1	LLI	WP_011824321.1_1576	CCTTTTGTATGATGAATTAACTGATAATGAAATGTTTTACTCTTCAGATAAA	kaiB	Circadian clock protein KaiB	WP_011824321.: 1453410..1453733	Circadian Rhythm	COG4251
WP_011824321.1_1576	CUST_8115_PI442190912	NATL1	LLI	WP_011824321.1_1576	GAAGAATCATTGGTGACCTATCAGATAGAGAAAAGGTGTTAATTGGACTGGACCTTTTGT	kaiB	Circadian clock protein KaiB	WP_011824321.: 1453410..1453733	Circadian Rhythm	COG4251
WP_011824321.1_1576	CUST_8116_PI442190912	NATL1	LLI	WP_011824321.1_1576	CCGAAGAAGATAAAATACTTGCCACACCAACCTTGTCAAAGATTTTACCCCCACCTGTTC	kaiB	Circadian clock protein KaiB	WP_011824321.: 1453410..1453733	Circadian Rhythm	COG4251
WP_011824321.1_1576	CUST_8117_PI442190912	NATL1	LLI	WP_011824321.1_1576	AGTGAATTCAAAGGTGTCTATGCTCTGAAGGTTATTGATGTTTTAAAAAGCCCTCAATTA	kaiB	Circadian clock protein KaiB	WP_011824321.: 1453410..1453733	Circadian Rhythm	COG4251
WP_011824321.1_1576	CUST_8118_PI442190912	NATL1	LLI	WP_011824321.1_1576	GTGGCTGGGAATACACCAAACTCTATGAGGGCTTTGAACACGTTGAGAGAAATCTTAGAA	kaiB	Circadian clock protein KaiB	WP_011824321.: 1453410..1453733	Circadian Rhythm	COG4251
WP_011824381.1_1638	CUST_8398_PI442190912	NATL1	LLI	WP_011824381.1_1638	GAAATAATACTTCCGAGCACTACAGGGCTTTTGGGGGTTTTACCTGGTCACATTTCAATG	atpC	ATP synthase epsilon subunit	WP_011824381.: 1501644..1502051	Energy Metabolism	COG0355
WP_011824381.1_1638	CUST_8399_PI442190912	NATL1	LLI	WP_011824381.1_1638	ATTGGGACTCAATAGCCCTAACTGGAGGTTTTGCAGAAGTTGAATCTAACGAAGTTACCG	atpC	ATP synthase epsilon subunit	WP_011824381.: 1501644..1502051	Energy Metabolism	COG0355
WP_011824381.1_1638	CUST_8400_PI442190912	NATL1	LLI	WP_011824381.1_1638	ATGACTCTTACTCTACGAGTTCTTGCTCCTGATCAGAGCGTATTTGACGATACTGCTGAT	atpC	ATP synthase epsilon subunit	WP_011824381.: 1501644..1502051	Energy Metabolism	COG0355
WP_011824381.1_1638	CUST_8401_PI442190912	NATL1	LLI	WP_011824381.1_1638	AAAGCTGAAATGGGGAAAAATATTGACTCTGGCAAAGCTGAAGCTGAATTAGAACAAGCT	atpC	ATP synthase epsilon subunit	WP_011824381.: 1501644..1502051	Energy Metabolism	COG0355
WP_011824381.1_1638	CUST_8402_PI442190912	NATL1	LLI	WP_011824381.1_1638	TCAAAGCACAAGAAACATTGAACAAAGCAAAAGCTTGGTTTCAAGCATCAAAATCAGACT	atpC	ATP synthase epsilon subunit	WP_011824381.: 1501644..1502051	Energy Metabolism	COG0355
WP_011824381.1_1638	CUST_8403_PI442190912	NATL1	LLI	WP_011824381.1_1638	TAGAACAAGCTAAAAATCAACTAAGCCAAAACAAGGATCAGGGGAATTC	atpC	ATP synthase epsilon subunit	WP_011824381.: 1501644..1502051	Energy Metabolism	COG0355
WP_011824391.1_1648	CUST_8455_PI442190912	NATL1	LLI	WP_011824391.1_1648	GAGCTGCGCAAACAAAAGCTATTAAGAAAATTGCCGGTACGTTAAAACTTGAACTGGCTC	atpA	ATP synthase alpha subunit	WP_011824391.: 1511187..1512701	Energy Metabolism	COG0056
WP_011824391.1_1648	CUST_8456_PI442190912	NATL1	LLI	WP_011824391.1_1648	TGAACTTGCGGCATTCTCTCAATTTGCTTCAGATCTTGATGAGGCTACTCAAAAGCAATT	atpA	ATP synthase alpha subunit	WP_011824391.: 1511187..1512701	Energy Metabolism	COG0056
WP_011824391.1_1648	CUST_8457_PI442190912	NATL1	LLI	WP_011824391.1_1648	CGATGATCTAACTAAGCAAGCTCAGGCTTACAGGCAAATGTCATTACTCTTACGTCGTCC	atpA	ATP synthase alpha subunit	WP_011824391.: 1511187..1512701	Energy Metabolism	COG0056
WP_011824391.1_1648	CUST_8458_PI442190912	NATL1	LLI	WP_011824391.1_1648	TGAAACGCAGGCTGGTGATGTTTCGGCTTATATCCCAACTAACGTCATATCAATTACTGA	atpA	ATP synthase alpha subunit	WP_011824391.: 1511187..1512701	Energy Metabolism	COG0056
WP_011824391.1_1648	CUST_8459_PI442190912	NATL1	LLI	WP_011824391.1_1648	CTAGGCCGTGAAGCATATCCAGGAGATGTTTTCTATTGTCATAGTCGTTTGCTTGAGAGG	atpA	ATP synthase alpha subunit	WP_011824391.: 1511187..1512701	Energy Metabolism	COG0056
WP_011824391.1_1648	CUST_8460_PI442190912	NATL1	LLI	WP_011824391.1_1648	AGTGAAGCATCTGAATCAATGCTTAAAGACGCTATTAGCGAGGTTAAGTCCTCCATGCTT	atpA	ATP synthase alpha subunit	WP_011824391.: 1511187..1512701	Energy Metabolism	COG0056
WP_011295331.1_1653	CUST_8483_PI442190912	NATL1	LLI	WP_011295331.1_1653	TTGAGCAATAAAGGTTTGCGTTACTTCGAGTATTACGTTCACCCAACGCCAATTATGCTC	atpI	ATP synthase subunit A	WP_011295331.: 1514831..1515556	Energy Metabolism	COG0356
WP_011295331.1_1653	CUST_8484_PI442190912	NATL1	LLI	WP_011295331.1_1653	ACTAAAAAAATGGAACGTGATCCAAGGGGTGTCCAGAACCTTTTGGAATTTCTTTGGGAT	atpI	ATP synthase subunit A	WP_011295331.: 1514831..1515556	Energy Metabolism	COG0356
WP_011295331.1_1653	CUST_8485_PI442190912	NATL1	LLI	WP_011295331.1_1653	CTTGGGGCACCTACAGCTGACATAAATACAACTGTTGCATTAGCACTACTGGTATCTCTC	atpI	ATP synthase subunit A	WP_011295331.: 1514831..1515556	Energy Metabolism	COG0356
WP_011295331.1_1653	CUST_8486_PI442190912	NATL1	LLI	WP_011295331.1_1653	ATAGAGATTGGATGCCTTTCATCGGAACCCTCTTTCTGTTCATTTTTGTGAGCAATTGGG	atpI	ATP synthase subunit A	WP_011295331.: 1514831..1515556	Energy Metabolism	COG0356
WP_011295331.1_1653	CUST_8487_PI442190912	NATL1	LLI	WP_011295331.1_1653	AGCGGTGCTCGTTTTTCTAGTACCTCTTGTTCTTCCAGTTCCAGTTATGTTTTTAGGCTT	atpI	ATP synthase subunit A	WP_011295331.: 1514831..1515556	Energy Metabolism	COG0356
WP_011295331.1_1653	CUST_8488_PI442190912	NATL1	LLI	WP_011295331.1_1653	CTATTCAAGCTCTGATTTTTGCAACTCTTGCTGCTTACTACATCGGGGAAGCAGTTGAAG	atpI	ATP synthase subunit A	WP_011295331.: 1514831..1515556	Energy Metabolism	COG0356
WP_011824403.1_1664	CUST_8543_PI442190912	NATL1	LLI	WP_011824403.1_1664	CTTCGACTTTTATCTTGTGGACCAAGAGCTGGATTATCAGAACTACAGCTTCCAGCCAAT	fumC	class II fumarate hydratase	WP_011824403.: 1523566..1524960	Krebs cycle and related genes	COG0114
WP_011824403.1_1664	CUST_8544_PI442190912	NATL1	LLI	WP_011824403.1_1664	TAATCCTACTCAATGCGAAGCCATGGCAATGGTATGTACGCAAATAATTGGCATGGATAC	fumC	class II fumarate hydratase	WP_011824403.: 1523566..1524960	Krebs cycle and related genes	COG0114
WP_011824403.1_1664	CUST_8545_PI442190912	NATL1	LLI	WP_011824403.1_1664	ATTGAAATCAACATCGAGGAACTTTATCCACTTCCACTTGGAGGAACCGCTATTGGTACA	fumC	class II fumarate hydratase	WP_011824403.: 1523566..1524960	Krebs cycle and related genes	COG0114
WP_011824403.1_1664	CUST_8546_PI442190912	NATL1	LLI	WP_011824403.1_1664	CGCCTTATCGCCATCAATAGGATACGAAAAAGCAAGTAAAATTGCTCAACTAGCCCATGA	fumC	class II fumarate hydratase	WP_011824403.: 1523566..1524960	Krebs cycle and related genes	COG0114
WP_011824403.1_1664	CUST_8547_PI442190912	NATL1	LLI	WP_011824403.1_1664	AGCAGGAAGTGGAGGACATCTACAAATGAATGTCTATAAACCACTGATTGGCTACAACAT	fumC	class II fumarate hydratase	WP_011824403.: 1523566..1524960	Krebs cycle and related genes	COG0114
WP_011824403.1_1664	CUST_8548_PI442190912	NATL1	LLI	WP_011824403.1_1664	ACCTTTTGAAACTGCAGCTAATAAATTTGCCATCATGGCTAGTCATGATGGTCTTGTAAA	fumC	class II fumarate hydratase	WP_011824403.: 1523566..1524960	Krebs cycle and related genes	COG0114
WP_041700632.1_1746	CUST_8980_PI442190912	NATL1	LLI	WP_041700632.1_1746	TTATTGAAGGAGCTGGAGACCATTGTTGCGAATATATGACTGGAGGAGTAGTTGTTGTGC	glsF	Ferredoxin-dependent glutamate synthase	WP_041700632.: 1605452..1610041	Nitrogen Metabolism	COG0069
WP_041700632.1_1746	CUST_8981_PI442190912	NATL1	LLI	WP_041700632.1_1746	CACATCTAACACTCAGGTGATACTTGGCAACACATGTCTATATGGAGCAACTGGTGGAAA	glsF	Ferredoxin-dependent glutamate synthase	WP_041700632.: 1605452..1610041	Nitrogen Metabolism	COG0069
WP_041700632.1_1746	CUST_8982_PI442190912	NATL1	LLI	WP_041700632.1_1746	GCAGACTGGTCTTCATGGAAGGAATTATTTAAAGCAGTAGTTCCGCCAAGCGAAAAAAGT	glsF	Ferredoxin-dependent glutamate synthase	WP_041700632.: 1605452..1610041	Nitrogen Metabolism	COG0069
WP_041700632.1_1746	CUST_8983_PI442190912	NATL1	LLI	WP_041700632.1_1746	GGTTTTAATGGGAATTTAAATTTGATATTTAAAGGTTCAGCGGGGCAAAGCTTTGGTGCA	glsF	Ferredoxin-dependent glutamate synthase	WP_041700632.: 1605452..1610041	Nitrogen Metabolism	COG0069
WP_041700632.1_1746	CUST_8984_PI442190912	NATL1	LLI	WP_041700632.1_1746	TGGAGAAGCTAATGATTATGTAGGAAAAGGCATTAACGGAGGTTCCATTACGATTGTTCC	glsF	Ferredoxin-dependent glutamate synthase	WP_041700632.: 1605452..1610041	Nitrogen Metabolism	COG0069
WP_041700632.1_1746	CUST_8985_PI442190912	NATL1	LLI	WP_041700632.1_1746	TGGAAAAACTGGAAGAAATATAGGAGCAGGAATGACTGGAGGCATAGCTTTTATTCTTGA	glsF	Ferredoxin-dependent glutamate synthase	WP_041700632.: 1605452..1610041	Nitrogen Metabolism	COG0069
WP_011824482.1_1750	CUST_9003_PI442190912	NATL1	LLI	WP_011824482.1_1750	CATGCCAAGTTTCATCTTGGGATCATGTTTTCCTTGGATTGTTCTGGATGTATAACGGCT	psaA	Photosystem I P700 chlorophyll A apoprotein A1 PsaA	WP_011824482.: 1613778..1616084	Photosystem I	COG2885
WP_011824482.1_1750	CUST_9004_PI442190912	NATL1	LLI	WP_011824482.1_1750	AGTGGATTCAAAATATTCATAATTCAGCAGCTGGTTCTACCACTCTTGCTGGTGCAAACG	psaA	Photosystem I P700 chlorophyll A apoprotein A1 PsaA	WP_011824482.: 1613778..1616084	Photosystem I	COG2885
WP_011824482.1_1750	CUST_9005_PI442190912	NATL1	LLI	WP_011824482.1_1750	TCCTATGGGCTCAGTCATCTCAGGTCCTAACAAGTTATGGTCAACCTATAAGCATGTACG	psaA	Photosystem I P700 chlorophyll A apoprotein A1 PsaA	WP_011824482.: 1613778..1616084	Photosystem I	COG2885
WP_011824482.1_1750	CUST_9006_PI442190912	NATL1	LLI	WP_011824482.1_1750	TGGAAGATGCAAAGTGATGTATGGGGTCTAACAGGAGGAAACTTTGCTCAAAGTTCCATA	psaA	Photosystem I P700 chlorophyll A apoprotein A1 PsaA	WP_011824482.: 1613778..1616084	Photosystem I	COG2885
WP_011824482.1_1750	CUST_9007_PI442190912	NATL1	LLI	WP_011824482.1_1750	GGTTCCGTAAGTCAAGTTGGAGGAAAAATTGGAATCGCTCCTATACCTTTAGGAACTGCT	psaA	Photosystem I P700 chlorophyll A apoprotein A1 PsaA	WP_011824482.: 1613778..1616084	Photosystem I	COG2885
WP_011824482.1_1750	CUST_9008_PI442190912	NATL1	LLI	WP_011824482.1_1750	TTGAGTCAATCATTTGGGCTCATAATAAACTTAACTTGGCTCCAACCATCCAACCAAGGG	psaA	Photosystem I P700 chlorophyll A apoprotein A1 PsaA	WP_011824482.: 1613778..1616084	Photosystem I	COG2885
WP_011295427.1_1751	CUST_9009_PI442190912	NATL1	LLI	WP_011295427.1_1751	CCATGCCAGGTAATCACTACTGGATGGACATGATCAATAGACCAGATGCACTAACCAATT	psaB	Photosystem I P700 chlorophyll A apoprotein A2 PsaB	WP_011295427.: 1616109..1618337	Photosystem I	COG2885
WP_011295427.1_1751	CUST_9010_PI442190912	NATL1	LLI	WP_011295427.1_1751	TGGTGTAAATGCTTTATCTCCATGGGCTTGGATGTTCTTATTTGGTCATCTCATTTGGGC	psaB	Photosystem I P700 chlorophyll A apoprotein A2 PsaB	WP_011295427.: 1616109..1618337	Photosystem I	COG2885
WP_011295427.1_1751	CUST_9011_PI442190912	NATL1	LLI	WP_011295427.1_1751	CCAGGTCGTGGTGGTACTTGTGATAGTTCTTCTTGGGACGCTACTTATTTAGCAATGTTC	psaB	Photosystem I P700 chlorophyll A apoprotein A2 PsaB	WP_011295427.: 1616109..1618337	Photosystem I	COG2885
WP_011295427.1_1751	CUST_9012_PI442190912	NATL1	LLI	WP_011295427.1_1751	TGGCATTGGAAACATCTAGCAATTTGGATGGGTAATACCGCTCAATTCAATGAATCTGGT	psaB	Photosystem I P700 chlorophyll A apoprotein A2 PsaB	WP_011295427.: 1616109..1618337	Photosystem I	COG2885
WP_011295427.1_1751	CUST_9013_PI442190912	NATL1	LLI	WP_011295427.1_1751	TATTGGACCTGCAGATTTCTTAGTTCACCATGCGATTGCACTAGGACTACATACAACTGC	psaB	Photosystem I P700 chlorophyll A apoprotein A2 PsaB	WP_011295427.: 1616109..1618337	Photosystem I	COG2885
WP_011295427.1_1751	CUST_9014_PI442190912	NATL1	LLI	WP_011295427.1_1751	GTTCCTCATCTCTTGGAGAGGATATTGGCAAGAACTTATCGAAACTCTTGTTTGGGCTCA	psaB	Photosystem I P700 chlorophyll A apoprotein A2 PsaB	WP_011295427.: 1616109..1618337	Photosystem I	COG2885
WP_011824529.1_1814	CUST_9343_PI442190912	NATL1	LLI	WP_011824529.1_1814	ACCCTGCTTTGCAGTTGTCTGTCAACCTGAGAAATAGGACTATTGTCCCTTTAGGATTTT	ppc	Phosphoenolpyruvate carboxylase	WP_011824529.: 1663493..1666477	Krebs cycle and related genes	COG2352
WP_011824529.1_1814	CUST_9344_PI442190912	NATL1	LLI	WP_011824529.1_1814	CTTTAGCTACTGAATTAAAGGCTGACCCCGACCAAATGGAGATGTTGCGAATGTTGAATC	ppc	Phosphoenolpyruvate carboxylase	WP_011824529.: 1663493..1666477	Krebs cycle and related genes	COG2352
WP_011824529.1_1814	CUST_9345_PI442190912	NATL1	LLI	WP_011824529.1_1814	AATTATTGCGTGGTGCATTGTTGACTATCAATGGTATCGCTGCAGGTATGAGAAACACAG	ppc	Phosphoenolpyruvate carboxylase	WP_011824529.: 1663493..1666477	Krebs cycle and related genes	COG2352
WP_011824529.1_1814	CUST_9346_PI442190912	NATL1	LLI	WP_011824529.1_1814	AAAGACTTATCAAGTCTTCGAGCTATCCCATGGGTCTTTGGTTGGACTCAAAGTCGTTTC	ppc	Phosphoenolpyruvate carboxylase	WP_011824529.: 1663493..1666477	Krebs cycle and related genes	COG2352
WP_011824529.1_1814	CUST_9347_PI442190912	NATL1	LLI	WP_011824529.1_1814	TTGGTTACCAATAAATTGGATGCTACGCCAAGTTGGAATGAATTGATGACCAGACTTGCA	ppc	Phosphoenolpyruvate carboxylase	WP_011824529.: 1663493..1666477	Krebs cycle and related genes	COG2352
WP_011824529.1_1814	CUST_9348_PI442190912	NATL1	LLI	WP_011824529.1_1814	TGGTTAGTTTGGGTGGAAGTGATGATCGGGATGCTTTCGCTAGCATTTTCGATATTATCT	ppc	Phosphoenolpyruvate carboxylase	WP_011824529.: 1663493..1666477	Krebs cycle and related genes	COG2352
WP_011824553.1_1839	CUST_9485_PI442190912	NATL1	LLI	WP_011824553.1_1839	CCCGAAATTTTAGCTGCAGTTGATGGGAAAATAGATGTGTTGCTAGATGGAGGTATTCGC	lldD	alpha-hydroxy-acidoxidizingprotein (L-lactate)	WP_011824553.: 1690320..1691516	Pyruvate metabolism	COG1304
WP_011824553.1_1839	CUST_9486_PI442190912	NATL1	LLI	WP_011824553.1_1839	AGGCTTAATGAGTTTTCCTAATGTTCAACTAGATGATGGCCCTATGGGATACACGGCAAT	lldD	alpha-hydroxy-acidoxidizingprotein (L-lactate)	WP_011824553.: 1690320..1691516	Pyruvate metabolism	COG1304
WP_011824553.1_1839	CUST_9487_PI442190912	NATL1	LLI	WP_011824553.1_1839	GCGTGGGGTGGAAAAATTATTGTTAAGGGTATACATATTGGCGATGACGCAAAAAAAGCG	lldD	alpha-hydroxy-acidoxidizingprotein (L-lactate)	WP_011824553.: 1690320..1691516	Pyruvate metabolism	COG1304
WP_011824553.1_1839	CUST_9488_PI442190912	NATL1	LLI	WP_011824553.1_1839	TCTGGTTTGAGGGAAAGAGATATGCGATCAGGAACCCAACAGCTTTTATCAATGAATCCT	lldD	alpha-hydroxy-acidoxidizingprotein (L-lactate)	WP_011824553.: 1690320..1691516	Pyruvate metabolism	COG1304
WP_011824553.1_1839	CUST_9489_PI442190912	NATL1	LLI	WP_011824553.1_1839	TGCAGGAGGGAAAGGCGTTGCCAGAGCTATAGAAATTCTTCAAACAGATATAGTGAGAAC	lldD	alpha-hydroxy-acidoxidizingprotein (L-lactate)	WP_011824553.: 1690320..1691516	Pyruvate metabolism	COG1304
WP_011824553.1_1839	CUST_9490_PI442190912	NATL1	LLI	WP_011824553.1_1839	AAAGCATTATGTCTTGGAGCGAAAGGAGTTTTGATCGGTAGAGCATATGCGTATGGACTT	lldD	alpha-hydroxy-acidoxidizingprotein (L-lactate)	WP_011824553.: 1690320..1691516	Pyruvate metabolism	COG1304
WP_011819337.1_12	CUST_67_PI442190949	MIT9515	HLI	WP_011819337.1_12	TCCCATGAACGTAGTGAAGTCTAGAAATAGTGTAGGAGGCACAGGATTTGAACAAGTAGA	argH	Argininosuccinate lyase	WP_011819337.: 14522..15901	Amino Acid metabolism	COG0165
WP_011819337.1_12	CUST_68_PI442190949	MIT9515	HLI	WP_011819337.1_12	TGCAACTGATTTGGCAGATTATCTAGTTTCTAAGCAGGTTCCTTTTAGGAAGGCTTATCA	argH	Argininosuccinate lyase	WP_011819337.: 14522..15901	Amino Acid metabolism	COG0165
WP_011819337.1_12	CUST_69_PI442190949	MIT9515	HLI	WP_011819337.1_12	TGGTAGGGTTTATGGAAATTTGCACACATTATTAACACTTATTAAAGGTGTACCTCTTGC	argH	Argininosuccinate lyase	WP_011819337.: 14522..15901	Amino Acid metabolism	COG0165
WP_011819337.1_12	CUST_70_PI442190949	MIT9515	HLI	WP_011819337.1_12	ACGGATAAGTGTGCTACTGGTAGTAGTTTGATGCCTCAGAAAAAAAATCCAGACGTTCCA	argH	Argininosuccinate lyase	WP_011819337.: 14522..15901	Amino Acid metabolism	COG0165
WP_011819337.1_12	CUST_71_PI442190949	MIT9515	HLI	WP_011819337.1_12	AGTATTGATGCTGTAAGTGATAGAGATTTTTGCATCGAATTTGCTTCTGCTTCTGCTTTA	argH	Argininosuccinate lyase	WP_011819337.: 14522..15901	Amino Acid metabolism	COG0165
WP_011819337.1_12	CUST_72_PI442190949	MIT9515	HLI	WP_011819337.1_12	AAGGCTTAGAGAAGTTAGAGCTAGGGTAAATATTTCGCCATTAGGTGCTGCAGCTTTAGC	argH	Argininosuccinate lyase	WP_011819337.: 14522..15901	Amino Acid metabolism	COG0165
WP_011819560.1_230	CUST_1249_PI442190949	MIT9515	HLI	WP_011819560.1_230	GCTTGATAAAAAAGGATTATCTACCGCTGTTGGAGATGAAGGTGGTTTTGCCCCAGAATT	eno	Enolase	WP_011819560.: 218871..220163	Glycolysis/Gluconeogenesis	COG0148
WP_011819560.1_230	CUST_1250_PI442190949	MIT9515	HLI	WP_011819560.1_230	CAGAAAGCCGGATTTATTCCTGGTAAACAAGTATCATTAGCTCTAGACGTTGCGAGCACT	eno	Enolase	WP_011819560.: 218871..220163	Glycolysis/Gluconeogenesis	COG0148
WP_011819560.1_230	CUST_1251_PI442190949	MIT9515	HLI	WP_011819560.1_230	TAGACTCCTAAGGATTGAGGAAGAGTTAGGGAACCAAGCTAGATTTGCTGGGGATTTAGG	eno	Enolase	WP_011819560.: 218871..220163	Glycolysis/Gluconeogenesis	COG0148
WP_011819560.1_230	CUST_1252_PI442190949	MIT9515	HLI	WP_011819560.1_230	TGTAGCAACTAGAGCTGGTCAAATTAAAACGGGCTCTTTGAGCAGAAGTGAAAGAATTGC	eno	Enolase	WP_011819560.: 218871..220163	Glycolysis/Gluconeogenesis	COG0148
WP_011819560.1_230	CUST_1253_PI442190949	MIT9515	HLI	WP_011819560.1_230	TTATGCTCGTACCTCACGGGGTGAAAACTTTTAGTGAAGCACTAAGAATGGGTACTGAAA	eno	Enolase	WP_011819560.: 218871..220163	Glycolysis/Gluconeogenesis	COG0148
WP_011819560.1_230	CUST_1254_PI442190949	MIT9515	HLI	WP_011819560.1_230	GTCTCTATAGAAGATGGATTGGGAGAAGATGATTGGGAGGGTTGGGCAGCCTTAAATAAG	eno	Enolase	WP_011819560.: 218871..220163	Glycolysis/Gluconeogenesis	COG0148
WP_011819578.1_248	CUST_1339_PI442190949	MIT9515	HLI	WP_011819578.1_248	TCGCAATATAAAAATATAGACTTTATCGCAGCCGGGGGTTTATCAATAGTTGATGTGCCT	eda	2-dehydro-3-deoxyphosphogluconate aldolase	WP_011819578.: 238417..239088	Entner-Dudoroff	COG0800
WP_011819578.1_248	CUST_1340_PI442190949	MIT9515	HLI	WP_011819578.1_248	TGGTTTGATTATGTTTCCAATCTCAAATTAAAGTTCCCAAAACTTAATTTAGGCTCTGCT	eda	2-dehydro-3-deoxyphosphogluconate aldolase	WP_011819578.: 238417..239088	Entner-Dudoroff	COG0800
WP_011819578.1_248	CUST_1341_PI442190949	MIT9515	HLI	WP_011819578.1_248	TGGGATAAGGATCTTTTAAATTATTCAAGATCAAAAAACCATCTATTGATTCCCGGCATT	eda	2-dehydro-3-deoxyphosphogluconate aldolase	WP_011819578.: 238417..239088	Entner-Dudoroff	COG0800
WP_011819578.1_248	CUST_1342_PI442190949	MIT9515	HLI	WP_011819578.1_248	AAAACAACTAGAAAAGTTAATAAAAATTGGTTTTTTGAACATTGAAATTAGTTGGTCTGA	eda	2-dehydro-3-deoxyphosphogluconate aldolase	WP_011819578.: 238417..239088	Entner-Dudoroff	COG0800
WP_011819578.1_248	CUST_1343_PI442190949	MIT9515	HLI	WP_011819578.1_248	AGATCTTAAAGAAGCAATAGATTTGCATTGTAAAATTATTAAAATTTATCCAGTAAAAGA	eda	2-dehydro-3-deoxyphosphogluconate aldolase	WP_011819578.: 238417..239088	Entner-Dudoroff	COG0800
WP_011819578.1_248	CUST_1344_PI442190949	MIT9515	HLI	WP_011819578.1_248	AAAGATTTTTTCTCCAAAAATTTAGAGTCAAAATCTTTTTTTTTATTGATAAAACCTACT	eda	2-dehydro-3-deoxyphosphogluconate aldolase	WP_011819578.: 238417..239088	Entner-Dudoroff	COG0800
WP_041710549.1_346	CUST_1855_PI442190949	MIT9515	HLI	WP_041710549.1_346	AAAAGAACTCTCACTGGAGAAATAGAATTCAAAAAAAATCACGATAACCCAAAAAAGAAA	minE	septum site-determining protein MinE	WP_041710549.: 324592..324912	Cell Division	COG0851
WP_041710549.1_346	CUST_1856_PI442190949	MIT9515	HLI	WP_041710549.1_346	GTTGCTGTCAGTCTAGAAACAGAGGATAGAATGACTGCATTGGTTGCTAATCTTCCTATA	minE	septum site-determining protein MinE	WP_041710549.: 324592..324912	Cell Division	COG0851
WP_041710549.1_346	CUST_1857_PI442190949	MIT9515	HLI	WP_041710549.1_346	TATTGGATAAGATGAGAAAAGAAATCCTTGATGTAGTTGCTAAATATGTTGA	minE	septum site-determining protein MinE	WP_041710549.: 324592..324912	Cell Division	COG0851
WP_041710549.1_346	CUST_1858_PI442190949	MIT9515	HLI	WP_041710549.1_346	GCAACTAGTTTTAGCTCATGACAGAGTTGATATGAGCTCATTGACCACTGATCTATTGGA	minE	septum site-determining protein MinE	WP_041710549.: 324592..324912	Cell Division	COG0851
WP_041710549.1_346	CUST_1859_PI442190949	MIT9515	HLI	WP_041710549.1_346	CCTTATAAACAAATTGCTAGGCAGAGAAACGTCCAGTGCCAATACAGCAAGGGAAAGATT	minE	septum site-determining protein MinE	WP_041710549.: 324592..324912	Cell Division	COG0851
WP_011819670.1_347	CUST_1860_PI442190949	MIT9515	HLI	WP_011819670.1_347	GTCCTGCCGGAGTTGAAGATGGTTTTAAAAATGCTCTGTCAGCCTGTAAAGAAGCAATAG	minD	septum site-determining protein MinD	WP_011819670.: 324916..325731	Cell Division	COG2894
WP_011819670.1_347	CUST_1861_PI442190949	MIT9515	HLI	WP_011819670.1_347	ATTGCTCTAGCAAATAGTGGTGCAACAACAGCTGTTCTAGATGCTGATTTTGGATTGAGG	minD	septum site-determining protein MinD	WP_011819670.: 324916..325731	Cell Division	COG2894
WP_011819670.1_347	CUST_1862_PI442190949	MIT9515	HLI	WP_011819670.1_347	GATGTTCTCGACAAAAATTGTCGCCTTGACCAAGCCTTAGTTAGACATAAAAAGGAGCCT	minD	septum site-determining protein MinD	WP_011819670.: 324916..325731	Cell Division	COG2894
WP_011819670.1_347	CUST_1863_PI442190949	MIT9515	HLI	WP_011819670.1_347	CCAGCTGGTGATCCTAGAATGCTTGATTGGATGAAACCTGAAGATATGAAACAAATCAGT	minD	septum site-determining protein MinD	WP_011819670.: 324916..325731	Cell Division	COG2894
WP_011819670.1_347	CUST_1864_PI442190949	MIT9515	HLI	WP_011819670.1_347	AGATGTTCAGAGTATCCTTTCTTTGCCCTTACTTGGTATTGTTTTAGAGGATGAGCAAGT	minD	septum site-determining protein MinD	WP_011819670.: 324916..325731	Cell Division	COG2894
WP_011819670.1_347	CUST_1865_PI442190949	MIT9515	HLI	WP_011819670.1_347	CTCAAAGGCTAACGGGAAAAGATATTCCTATTATCGATCCAAAAAATGAAGGACAAAGCA	minD	septum site-determining protein MinD	WP_011819670.: 324916..325731	Cell Division	COG2894
WP_011819671.1_348	CUST_1866_PI442190949	MIT9515	HLI	WP_011819671.1_348	AGCCATAGGGCCAAAAGAGAAACCAAAGCATCAATATCCTGAAATTGCAATTTTGGAAGC	minC	septum site-determining protein MinC	WP_011819671.: 325841..326509	Cell Division	COG0850
WP_011819671.1_348	CUST_1867_PI442190949	MIT9515	HLI	WP_011819671.1_348	GGAAAATTATTAGGTACCGCTTTCGCTGGCGAAAATGGTAATAAAAACGCCTCAATTGCC	minC	septum site-determining protein MinC	WP_011819671.: 325841..326509	Cell Division	COG0850
WP_011819671.1_348	CUST_1868_PI442190949	MIT9515	HLI	WP_011819671.1_348	TCGGAGTTAAGTAGCATCAAAGAACCAGTCGAAGCGCATCTTTTTACAGCAAATGAAAGA	minC	septum site-determining protein MinC	WP_011819671.: 325841..326509	Cell Division	COG0850
WP_011819671.1_348	CUST_1869_PI442190949	MIT9515	HLI	WP_011819671.1_348	GGGGACAGAATATCTTCAAATGGTGATCTTTTTATTATGGGAGATGTCAACCCTGGAGCT	minC	septum site-determining protein MinC	WP_011819671.: 325841..326509	Cell Division	COG0850
WP_011819671.1_348	CUST_1870_PI442190949	MIT9515	HLI	WP_011819671.1_348	TTTTGAGAAACTCCAATAACGATTTTATAAAATCAATATCTTTCGCTAAATTTGAGGCTT	minC	septum site-determining protein MinC	WP_011819671.: 325841..326509	Cell Division	COG0850
WP_011819671.1_348	CUST_1871_PI442190949	MIT9515	HLI	WP_011819671.1_348	ACTTCTAATGAATTAGCAAAACTGAAATTCCACCTAAAACAACTAAATATCAAGTTTTCA	minC	septum site-determining protein MinC	WP_011819671.: 325841..326509	Cell Division	COG0850
WP_011819738.1_408	CUST_2162_PI442190949	MIT9515	HLI	WP_011819738.1_408	TCAAAATCCAGTGATCCCATATCACGATTATACTGCCGCCAAAGGGGGTTTATTATCACT	gdh	3-oxoacyl-acyl-carrier-protein reductase	WP_011819738.: 377373..378137	Entner-Dudoroff	COG1028
WP_011819738.1_408	CUST_2163_PI442190949	MIT9515	HLI	WP_011819738.1_408	CCCCTTGGTCAAGGGCAGTTACTGGGCAAAATTTAGTAGTTGATGGAGGATTAGTTCTAA	gdh	3-oxoacyl-acyl-carrier-protein reductase	WP_011819738.: 377373..378137	Entner-Dudoroff	COG1028
WP_011819738.1_408	CUST_2164_PI442190949	MIT9515	HLI	WP_011819738.1_408	TGGAAGAGGCTTAGGTTCCGAAATAGCAAAATCTTTTCATCGAGAGGGAGCAAAAGTAGT	gdh	3-oxoacyl-acyl-carrier-protein reductase	WP_011819738.: 377373..378137	Entner-Dudoroff	COG1028
WP_011819738.1_408	CUST_2165_PI442190949	MIT9515	HLI	WP_011819738.1_408	ACTGATGCTAGCAAAGGAACTCCCGATAGTGTTTTTGAGTATATTTCAAGCATCACACCT	gdh	3-oxoacyl-acyl-carrier-protein reductase	WP_011819738.: 377373..378137	Entner-Dudoroff	COG1028
WP_011819738.1_408	CUST_2166_PI442190949	MIT9515	HLI	WP_011819738.1_408	ACGAGAACTGCGGCAATAGATCTAGGACAATTTAATATCACAGTAAATATGGTTTCAGGA	gdh	3-oxoacyl-acyl-carrier-protein reductase	WP_011819738.: 377373..378137	Entner-Dudoroff	COG1028
WP_011819738.1_408	CUST_2167_PI442190949	MIT9515	HLI	WP_011819738.1_408	AGTGGCAAGATTTCCAAAACCAAATTGAAACTTCTCAGAAAGGATTTTTAAATCTGCTGA	gdh	3-oxoacyl-acyl-carrier-protein reductase	WP_011819738.: 377373..378137	Entner-Dudoroff	COG1028
WP_011819823.1_492	CUST_2604_PI442190949	MIT9515	HLI	WP_011819823.1_492	CTGCCTACCTCACATATAAAGCAGTCAATCCTTTGCCGGATGGTGCTATCTACGAATTAG	cyoC	heme-copper oxidase subunit III	WP_011819823.: 453407..454009	Respiration	COG1845
WP_011819823.1_492	CUST_2605_PI442190949	MIT9515	HLI	WP_011819823.1_492	TGGCAAGCAAGATCAAAGGGAGGAAGAATTACCAATCAAAATATGTTTCCATTAGAAGCT	cyoC	heme-copper oxidase subunit III	WP_011819823.: 453407..454009	Respiration	COG1845
WP_011819823.1_492	CUST_2606_PI442190949	MIT9515	HLI	WP_011819823.1_492	TGCAAGTACTTTTTATGCTTTAACAGGCTTTCATGGTCTCCATGTAACTTTAGGAACGTT	cyoC	heme-copper oxidase subunit III	WP_011819823.: 453407..454009	Respiration	COG1845
WP_011819823.1_492	CUST_2607_PI442190949	MIT9515	HLI	WP_011819823.1_492	AGTGCAACCTTTCATAAAGCAGGTAAAGCGCTTCTTAAAAATAAAAATTCCGATACCCAG	cyoC	heme-copper oxidase subunit III	WP_011819823.: 453407..454009	Respiration	COG1845
WP_011819823.1_492	CUST_2608_PI442190949	MIT9515	HLI	WP_011819823.1_492	TGAGAGTGAGCTAAGGGAACATCATGAAGACTTCAGGATGTTTGGATTAATCACTTTTTT	cyoC	heme-copper oxidase subunit III	WP_011819823.: 453407..454009	Respiration	COG1845
WP_011819823.1_492	CUST_2609_PI442190949	MIT9515	HLI	WP_011819823.1_492	AAATGGTTATTAGTTACTGCTCTTCTTGGGATAGTTTTTTTAATATGTCAATTATTCGAA	cyoC	heme-copper oxidase subunit III	WP_011819823.: 453407..454009	Respiration	COG1845
WP_011819824.1_493	CUST_2610_PI442190949	MIT9515	HLI	WP_011819824.1_493	TAGCAGTTCCAACTGGAATTAAATTTTTTAATTGGGTTGCAACATTGTGGGGCGGGAAAA	cyoB/coxA	Cytochrome c oxidase subunit I	WP_011819824.: 454025..455644	Respiration	COG0843
WP_011819824.1_493	CUST_2611_PI442190949	MIT9515	HLI	WP_011819824.1_493	GGTCTAGTTGTTTGGGCTCATCATATGTTTACAAGTGGAACCCCACCTTGGATGAGATTA	cyoB/coxA	Cytochrome c oxidase subunit I	WP_011819824.: 454025..455644	Respiration	COG0843
WP_011819824.1_493	CUST_2612_PI442190949	MIT9515	HLI	WP_011819824.1_493	TGGTAAGGATTCTGGGGATAATCCTTGGGATGCTTTAACTCCTGAATGGTTAACAACCTC	cyoB/coxA	Cytochrome c oxidase subunit I	WP_011819824.: 454025..455644	Respiration	COG0843
WP_011819824.1_493	CUST_2613_PI442190949	MIT9515	HLI	WP_011819824.1_493	ACGGAGGGACCGTTTTTATTATCTTCTCATCCATATACCATTGGTTCCCAAAGGTGACAG	cyoB/coxA	Cytochrome c oxidase subunit I	WP_011819824.: 454025..455644	Respiration	COG0843
WP_011819824.1_493	CUST_2614_PI442190949	MIT9515	HLI	WP_011819824.1_493	CTCCACCCGTTGAGAATTGGGAAGGAGAAGCACCTTTAGTAGAAGAACCTTATGGTTATG	cyoB/coxA	Cytochrome c oxidase subunit I	WP_011819824.: 454025..455644	Respiration	COG0843
WP_011819824.1_493	CUST_2615_PI442190949	MIT9515	HLI	WP_011819824.1_493	CAACATTGGCTTGGTCTTAATGGAATGCCTAGAAGGGTGGCAGAATATGATCCTCAATTT	cyoB/coxA	Cytochrome c oxidase subunit I	WP_011819824.: 454025..455644	Respiration	COG0843
WP_011819825.1_494	CUST_2616_PI442190949	MIT9515	HLI	WP_011819825.1_494	TGCTTTTTGGGTTCCGGAATTCAGAATAAAACAAGATATCATTCCAGGCCAACCTACGAT	ctaC/coxB	Cytochrome c oxidase subunit II	WP_011819825.: 455641..456444	Respiration	COG1622
WP_011819825.1_494	CUST_2617_PI442190949	MIT9515	HLI	WP_011819825.1_494	GGGAAATATCCAATAATTTGTGCTGAATTATGTGGTCCTTATCACGGAGGCATGAGAGCA	ctaC/coxB	Cytochrome c oxidase subunit II	WP_011819825.: 455641..456444	Respiration	COG1622
WP_011819825.1_494	CUST_2618_PI442190949	MIT9515	HLI	WP_011819825.1_494	TGGGGAGCTTCATGTTCCAGTTGATCATAAGGTCTCTATGAAGATGGAATCGAAAGATGT	ctaC/coxB	Cytochrome c oxidase subunit II	WP_011819825.: 455641..456444	Respiration	COG1622
WP_011819825.1_494	CUST_2619_PI442190949	MIT9515	HLI	WP_011819825.1_494	CACAGCCATGAAATGATGAAATCAAATACAGAAAAGATATGGGCTGGAATTAGCCAAACA	ctaC/coxB	Cytochrome c oxidase subunit II	WP_011819825.: 455641..456444	Respiration	COG1622
WP_011819825.1_494	CUST_2620_PI442190949	MIT9515	HLI	WP_011819825.1_494	CGGAGATGGGATAGCTTTAGAAGGTAATTTAAGCCTTGAAATTGTATGGACAATTATCCC	ctaC/coxB	Cytochrome c oxidase subunit II	WP_011819825.: 455641..456444	Respiration	COG1622
WP_011819825.1_494	CUST_2621_PI442190949	MIT9515	HLI	WP_011819825.1_494	TCTTTTTGGATCGGATTTAATGTTAATTTACTTCCTGTTGAAGCAAGTATAAATGCACCA	ctaC/coxB	Cytochrome c oxidase subunit II	WP_011819825.: 455641..456444	Respiration	COG1622
WP_011819899.1_568	CUST_3021_PI442190949	MIT9515	HLI	WP_011819899.1_568	AGCTGTAGCCTGTGCGAATGCAAAAATCACCTTAATCTCCCCTTTTGTTGGAAGAATATT	tal	Transaldolase	WP_011819899.: 521181..522182	Pentose Phosphate	COG0176
WP_011819899.1_568	CUST_3022_PI442190949	MIT9515	HLI	WP_011819899.1_568	AAGATCGCTTCAACTTGGGAAGGAATTAAAGCAGCTGAAATCTTGGAAAAGGAGGGTATT	tal	Transaldolase	WP_011819899.: 521181..522182	Pentose Phosphate	COG0176
WP_011819899.1_568	CUST_3023_PI442190949	MIT9515	HLI	WP_011819899.1_568	AGATTGGCATAAAGTAAAAACAGGCAAAGATAATTTCTCAGGGAAGGAAGATCCAGGTGT	tal	Transaldolase	WP_011819899.: 521181..522182	Pentose Phosphate	COG0176
WP_011819899.1_568	CUST_3024_PI442190949	MIT9515	HLI	WP_011819899.1_568	AGCAGGGTGTGACCTTCTAACTATTGCGCCAAAGTTTTTAGATGAACTTAGTAAAGAAGA	tal	Transaldolase	WP_011819899.: 521181..522182	Pentose Phosphate	COG0176
WP_011819899.1_568	CUST_3025_PI442190949	MIT9515	HLI	WP_011819899.1_568	TCAGGAAGAGTATCTACAGAAGTTGATGCAAGACTTAGTTTTGATACTGATGCCACAGTT	tal	Transaldolase	WP_011819899.: 521181..522182	Pentose Phosphate	COG0176
WP_011819899.1_568	CUST_3026_PI442190949	MIT9515	HLI	WP_011819899.1_568	AGAAGATCAAATGGCTAGTGAGAAGCTAAGTGAAGGAATAACAGGTTTCAGTAAAGCAAT	tal	Transaldolase	WP_011819899.: 521181..522182	Pentose Phosphate	COG0176
WP_002805854.1_599	CUST_3197_PI442190949	MIT9515	HLI	WP_002805854.1_599	GGTGGTATTCACGTATGGCATATGCCTGCACTTTTAGCGATTTTTGGAGATGATTCTTGT	rbcL	Ribulose 1,5-bisphosphate carboxylase large subunit	WP_002805854.: 549602..551017	Carbon fixation	COG1850
WP_002805854.1_599	CUST_3198_PI442190949	MIT9515	HLI	WP_002805854.1_599	TCACTACCTAAACTGTACTGCAAACACTCCTGAAGAGCTCTATGAACGTGCTGAATTTGC	rbcL	Ribulose 1,5-bisphosphate carboxylase large subunit	WP_002805854.: 549602..551017	Carbon fixation	COG1850
WP_002805854.1_599	CUST_3199_PI442190949	MIT9515	HLI	WP_002805854.1_599	GACATTCTTATGGAAGCTGCTAAGCACAGTCCTGAATTGGCTATTGCTCTTGAAACTTGG	rbcL	Ribulose 1,5-bisphosphate carboxylase large subunit	WP_002805854.: 549602..551017	Carbon fixation	COG1850
WP_011376108.1_600	CUST_3203_PI442190949	MIT9515	HLI	WP_011376108.1_600	ACCCTGATCACCATGTAAGAATAATCGGATACGATGCTTACACTCAAAGCCAAGGAACAG	rbcS	Ribulose bisphosphate carboxylase small subunit	WP_011376108.: 551111..551452	Carbon fixation	COG4451
WP_011376108.1_600	CUST_3204_PI442190949	MIT9515	HLI	WP_011376108.1_600	AGTGGCAGTATGCAAACTTATTGGTCTTATTGGAAGCTCCCATTCTTTGGGGAAAAAGAC	rbcS	Ribulose bisphosphate carboxylase small subunit	WP_011376108.: 551111..551452	Carbon fixation	COG4451
WP_011376108.1_600	CUST_3205_PI442190949	MIT9515	HLI	WP_011376108.1_600	CGATCAAATTGCATACATAATTGCTCAAGGCTGGAGTCCTGTTATTGAGCA	rbcS	Ribulose bisphosphate carboxylase small subunit	WP_011376108.: 551111..551452	Carbon fixation	COG4451
WP_011376108.1_600	CUST_3206_PI442190949	MIT9515	HLI	WP_011376108.1_600	AACCCTGGAAACATTCGGTTTCTTACCACCGATGACCCAGGAAGAAATATACGATCAAAT	rbcS	Ribulose bisphosphate carboxylase small subunit	WP_011376108.: 551111..551452	Carbon fixation	COG4451
WP_011819975.1_646	CUST_3445_PI442190949	MIT9515	HLI	WP_011819975.1_646	CGCAATAGTTGGAGCAGGTACAGGTCTAGGTATTGCGAGGGGCTTTATTAATGGAAATAA	glk	Glucokinase	WP_011819975.: 596236..597273	Glycolysis	COG0837
WP_011819975.1_646	CUST_3446_PI442190949	MIT9515	HLI	WP_011819975.1_646	ACTATGCTTTGGGGGATTATGGATTGCAGGAGGTACGGCTACAAAACATTTCAAAAACTT	glk	Glucokinase	WP_011819975.: 596236..597273	Glycolysis	COG0837
WP_011819975.1_646	CUST_3447_PI442190949	MIT9515	HLI	WP_011819975.1_646	GCGAAGGAGGTCATGTGGAGTTCTCCCCAAGGTCAAAAGAAGAATGGGAATTAAAGATAT	glk	Glucokinase	WP_011819975.: 596236..597273	Glycolysis	COG0837
WP_011819975.1_646	CUST_3448_PI442190949	MIT9515	HLI	WP_011819975.1_646	ATTGTTAGTGGTGAAGGTCTCTCAAGAATAGCTGAGTGGAGATTAAGCAAACCTGATGCT	glk	Glucokinase	WP_011819975.: 596236..597273	Glycolysis	COG0837
WP_011819975.1_646	CUST_3449_PI442190949	MIT9515	HLI	WP_011819975.1_646	GATGAAGAGTTTGGACTTTATAGTGCAGCCTGCAGAGCAAAAATGCTATTAAAAACAACA	glk	Glucokinase	WP_011819975.: 596236..597273	Glycolysis	COG0837
WP_011819975.1_646	CUST_3450_PI442190949	MIT9515	HLI	WP_011819975.1_646	TGAAGGGGATATTTTAATGATGGAAATTGAAAGGATATGGTTAGAAGCTTATGCCTCTTT	glk	Glucokinase	WP_011819975.: 596236..597273	Glycolysis	COG0837
WP_011820110.1_777	CUST_4162_PI442190949	MIT9515	HLI	WP_011820110.1_777	TTTACTCCGTGGGAAGATATTCCGAACGGAACAGATGTTCTCTTCTACGAGGGTTTACAT	prk	Phosphoribulokinase	WP_011820110.: 718636..719535	Carbon Fixation	COG3954
WP_011820110.1_777	CUST_4163_PI442190949	MIT9515	HLI	WP_011820110.1_777	TCACAGGTTTGAAAGAATGCCTATGAAAAAAGCTATGGCTGAAGCCCTTTCAAAAGGAGA	prk	Phosphoribulokinase	WP_011820110.: 718636..719535	Carbon Fixation	COG3954
WP_011820110.1_777	CUST_4164_PI442190949	MIT9515	HLI	WP_011820110.1_777	TTTGCTGACTTGCTTGTGGGTGTTGTACCCATAACCAACCTAGAGTGGATACAGAAAATT	prk	Phosphoribulokinase	WP_011820110.: 718636..719535	Carbon Fixation	COG3954
WP_011820110.1_777	CUST_4165_PI442190949	MIT9515	HLI	WP_011820110.1_777	TGGGGAAACTGGTGGAGGGAAAAAAAGATATTATCTTCACAGTACTGAAGAAGCTCAGGA	prk	Phosphoribulokinase	WP_011820110.: 718636..719535	Carbon Fixation	COG3954
WP_011820110.1_777	CUST_4166_PI442190949	MIT9515	HLI	WP_011820110.1_777	TGTCAAGTCCAACAAGTATTGTTGTTAATGGAGGCAAAATGGCTTTTGCAATGGAATTGA	prk	Phosphoribulokinase	WP_011820110.: 718636..719535	Carbon Fixation	COG3954
WP_011820110.1_777	CUST_4167_PI442190949	MIT9515	HLI	WP_011820110.1_777	TTTCTCACTTTGGTCCGGAAGCAAATCATTTTGGACAGTTGGAGGAACTTTTTAAAGTTT	prk	Phosphoribulokinase	WP_011820110.: 718636..719535	Carbon Fixation	COG3954
WP_011820114.1_781	CUST_4184_PI442190949	MIT9515	HLI	WP_011820114.1_781	CGTTCTTGATTGACGTGCTTTACAGCATTCTTGCTTAAAAGCATGGAAAGGTAGCGAA	fda	Fructose-1,6-bisphosphate aldolase class I	WP_011820114.: 722197..723264	Glycolysis/Gluconeogenesis	COG0191
WP_011820114.1_781	CUST_4185_PI442190949	MIT9515	HLI	WP_011820114.1_781	AAGGCGCAAGATTTCAAAAGTGAGAGCAGTTCAAAATCAGAAGGGGTTTTGGGTTGTCCTT	fda	Fructose-1,6-bisphosphate aldolase class I	WP_011820114.: 722197..723264	Glycolysis/Gluconeogenesis	COG0191
WP_011820114.1_781	CUST_4186_PI442190949	MIT9515	HLI	WP_011820114.1_781	[illegible]	fda	Fructose-1,6-bisphosphate aldolase class I	WP_011820114.: 722197..723264	Glycolysis/Gluconeogenesis	COG0191
WP_011820114.1_781	CUST_4187_PI442190949	MIT9515	HLI	WP_011820114.1_781	[illegible]	fda	Fructose-1,6-bisphosphate aldolase class I	WP_011820114.: 722197..723264	Glycolysis/Gluconeogenesis	COG0191
WP_011820114.1_781	CUST_4188_PI442190949	MIT9515	HLI	WP_011820114.1_781	[illegible]	fda	Fructose-1,6-bisphosphate aldolase class I	WP_011820114.: 722197..723264	Glycolysis/Gluconeogenesis	COG0191
WP_011820114.1_781	CUST_4189_PI442190949	MIT9515	HLI	WP_011820114.1_781	[illegible]	fda	Fructose-1,6-bisphosphate aldolase class I	WP_011820114.: 722197..723264	Glycolysis/Gluconeogenesis	COG0191

WP_011820114.1_781	CUST_4190_PI442190949	MIT9515	HLI	WP_011820114.1_781	TGGAGTTTTCCTTGAGGGAACACTCTTAAAACCTTCGATGACTGTTAATGGAGCTGAGTG	fda	Fructose-1,6-bisphosphate aldolase class I	WP_011820114. 722197..723264	Glycolysis/Gluconeogenesis	COG0191
WP_011820115.1_782	CUST_4191_PI442190949	MIT9515	HLI	WP_011820115.1_782	ATGGTTAGATATCATAAATAAATACGGGGGAGAGATTCCGCAAACATATGGCGTGCCTGT	cbbA	Fructose-1,6-bisphosphate aldolase class II	WP_011820115. 723412..724485	Glycolysis/Gluconeogenesis	COG0191
WP_011820115.1_782	CUST_4192_PI442190949	MIT9515	HLI	WP_011820115.1_782	AGATACAGACAATTCTGGTGTGAAGGTCAAGCAGGTAGGATCAAGCAAAGCAGTACAAAT	cbbA	Fructose-1,6-bisphosphate aldolase class II	WP_011820115. 723412..724485	Glycolysis/Gluconeogenesis	COG0191
WP_011820115.1_782	CUST_4193_PI442190949	MIT9515	HLI	WP_011820115.1_782	TAGAGAAGCGGCTTTTTCAGACACAGCAAACTTCGATCCAAGACATTTCAATAAACCTGC	cbbA	Fructose-1,6-bisphosphate aldolase class II	WP_011820115. 723412..724485	Glycolysis/Gluconeogenesis	COG0191
WP_011820115.1_782	CUST_4194_PI442190949	MIT9515	HLI	WP_011820115.1_782	TTGAAGGTGAGCTTTCAACAGATATGCTTTTAACTGATCCGGAAGAAGCCGCAGATTTTG	cbbA	Fructose-1,6-bisphosphate aldolase class II	WP_011820115. 723412..724485	Glycolysis/Gluconeogenesis	COG0191
WP_011820115.1_782	CUST_4196_PI442190949	MIT9515	HLI	WP_011820115.1_782	GGGAGAACTAGGATGTTTAGGTTCATTAGAAACTGGAAAAGGCGAAGCTGAAGATGGACA	cbbA	Fructose-1,6-bisphosphate aldolase class II	WP_011820115. 723412..724485	Glycolysis/Gluconeogenesis	COG0191
WP_011820127.1_794	CUST_4254_PI442190949	MIT9515	HLI	WP_011820127.1_794	AGATAATTTAGCTAATGTTGTTTCTTCCTCAACAAAAGCTGGCATCCCTGTGCCCTGTTT	edd	phosphogluconate dehydrogenase (NADP(+)-dependent, decarboxyla	WP_011820127. 731774..733192	Pentose Phosphate/Entner-Dudorof	COG2084
WP_011820127.1_794	CUST_4255_PI442190949	MIT9515	HLI	WP_011820127.1_794	GTGGTTCTTGCTACTATCGCTAGTTACGCTCAAGGCATGGACATATTAAGTGAAGCATCA	edd	phosphogluconate dehydrogenase (NADP(+)-dependent, decarboxyla	WP_011820127. 731774..733192	Pentose Phosphate/Entner-Dudorof	COG2084
WP_011820127.1_794	CUST_4256_PI442190949	MIT9515	HLI	WP_011820127.1_794	ATGTTGTTGAGAAAATATTGGATAAAGCTGGCCAAAAAGGTACTGGCTTATGGACGGTGG	edd	phosphogluconate dehydrogenase (NADP(+)-dependent, decarboxyla	WP_011820127. 731774..733192	Pentose Phosphate/Entner-Dudorof	COG2084
WP_011820127.1_794	CUST_4257_PI442190949	MIT9515	HLI	WP_011820127.1_794	AAACAGACTTCCACAAAACCTGGTGCAGGCTATGAGAGATTGTTTTGGCTCTCATACTTA	edd	phosphogluconate dehydrogenase (NADP(+)-dependent, decarboxyla	WP_011820127. 731774..733192	Pentose Phosphate/Entner-Dudorof	COG2084
WP_011820127.1_794	CUST_4258_PI442190949	MIT9515	HLI	WP_011820127.1_794	AGTGCGTTAGAACTTGGTGTGTCAGTTCCAACTATTTATGCCTCTTTAAATGCAAGGGTT	edd	phosphogluconate dehydrogenase (NADP(+)-dependent, decarboxyla	WP_011820127. 731774..733192	Pentose Phosphate/Entner-Dudorof	COG2084
WP_011820127.1_794	CUST_4259_PI442190949	MIT9515	HLI	WP_011820127.1_794	TGATTTGAATATGCCGTCAATTGCTCAAATATGGAAAGGTGGGTGCATTATAAGATCAAA	edd	phosphogluconate dehydrogenase (NADP(+)-dependent, decarboxyla	WP_011820127. 731774..733192	Pentose Phosphate/Entner-Dudorof	COG2084
WP_011820128.1_795	CUST_4260_PI442190949	MIT9515	HLI	WP_011820128.1_795	AGACAATGTAGTCATAGTGAACAAGGATAGAGTAGAAGAAGCAGACAAACCTGAGTTGGG	glgC	Glucose-1-phosphate adenylyltransferase	WP_011820128. 733291..734586	Glycogen Metabolism	COG0448
WP_011820128.1_795	CUST_4261_PI442190949	MIT9515	HLI	WP_011820128.1_795	GAAAAGGTGGTGGTACACCTCTTGGTGTAGGTGTAGGCTCAACTATAAAAAGAGCAATCC	glgC	Glucose-1-phosphate adenylyltransferase	WP_011820128. 733291..734586	Glycogen Metabolism	COG0448
WP_011820128.1_795	CUST_4262_PI442190949	MIT9515	HLI	WP_011820128.1_795	CTGTCATTGAAGATGCCTTAGTAATGGGGGCTGATTTCTTTGAATCGCAAGAAGAAAGGG	glgC	Glucose-1-phosphate adenylyltransferase	WP_011820128. 733291..734586	Glycogen Metabolism	COG0448
WP_011820128.1_795	CUST_4263_PI442190949	MIT9515	HLI	WP_011820128.1_795	CGGTACTATTGGAGCATTTTTCGAATCGAATTTAGCATTAACACGTCAACCTAAACCTCC	glgC	Glucose-1-phosphate adenylyltransferase	WP_011820128. 733291..734586	Glycogen Metabolism	COG0448
WP_011820128.1_795	CUST_4264_PI442190949	MIT9515	HLI	WP_011820128.1_795	TCTAAGTTTGGATTAACGAAGGAATCAGCATTAGAGAAACCCTATCTTGCCTCGATGGGT	glgC	Glucose-1-phosphate adenylyltransferase	WP_011820128. 733291..734586	Glycogen Metabolism	COG0448
WP_011820128.1_795	CUST_4265_PI442190949	MIT9515	HLI	WP_011820128.1_795	TGATGAAGCTCAGGCTGAAGGCTTTGGTCTGATGAGAACAGACGATTTGGGTAATATTAA	glgC	Glucose-1-phosphate adenylyltransferase	WP_011820128. 733291..734586	Glycogen Metabolism	COG0448
WP_011820281.1_934	CUST_4945_PI442190949	MIT9515	HLI	WP_011820281.1_934	TCCAGCTGCATTACTTTCTTCGGCTTGGTTTTTTTCTGGAAATGGAATAGGGAAAAGAGA	pgi	Glucose-6-phosphate isomerase	WP_011820281. 868110..869693	Glycolysis/Gluconeogenesis	COG0166
WP_011820281.1_934	CUST_4946_PI442190949	MIT9515	HLI	WP_011820281.1_934	AATGCTTATGATCAACCTGGAGTTGAAGCAGGTAAAAAAGCAGCAGCAGAAATACTTGAA	pgi	Glucose-6-phosphate isomerase	WP_011820281. 868110..869693	Glycolysis/Gluconeogenesis	COG0166
WP_011820281.1_934	CUST_4947_PI442190949	MIT9515	HLI	WP_011820281.1_934	TGTTTAACTCTCGGAGCCTTAATTGCATTATTTGAGCGAGCTGTTTCTTTTTACGCAGAA	pgi	Glucose-6-phosphate isomerase	WP_011820281. 868110..869693	Glycolysis/Gluconeogenesis	COG0166
WP_011820281.1_934	CUST_4948_PI442190949	MIT9515	HLI	WP_011820281.1_934	TGGTAACAAAGGCTCCACTGATCAACATGCATATGTTCAACAGTTAAGAGATGGAATTGA	pgi	Glucose-6-phosphate isomerase	WP_011820281. 868110..869693	Glycolysis/Gluconeogenesis	COG0166
WP_011820281.1_934	CUST_4949_PI442190949	MIT9515	HLI	WP_011820281.1_934	ATACGTCAGATGTGCTTTGGCAATGATGATTATCTAATAAAAGGAGATTGGTCAAACCCT	pgi	Glucose-6-phosphate isomerase	WP_011820281. 868110..869693	Glycolysis/Gluconeogenesis	COG0166
WP_011820281.1_934	CUST_4950_PI442190949	MIT9515	HLI	WP_011820281.1_934	TCTGGATTTTTGCAAGGTACAAGATCAGCATTATCTAACGAAAACAGACAAAGCATAACA	pgi	Glucose-6-phosphate isomerase	WP_011820281. 868110..869693	Glycolysis/Gluconeogenesis	COG0166
WP_011820303.1_956	CUST_5062_PI442190949	MIT9515	HLI	WP_011820303.1_956	AAGCAATATTGCGAGGCAGCTTGACGCTGGAGCAATAATTCCTTTAACTAAATCAGGATC	pykF	Pyruvate kinase	WP_011820303. 888621..890411	Glycolysis	COG0469
WP_011820303.1_956	CUST_5063_PI442190949	MIT9515	HLI	WP_011820303.1_956	GCACGTAATGTAAGTAAGTTCAGACCACCTACTCCTATCCTTGCCACGACTACTGAGAAG	pykF	Pyruvate kinase	WP_011820303. 888621..890411	Glycolysis	COG0469
WP_011820303.1_956	CUST_5064_PI442190949	MIT9515	HLI	WP_011820303.1_956	ACAAACTGCTGGAACTTTGACAGGAATAAGTGGATCTACTGACCTTATAAAAGTTGGCCT	pykF	Pyruvate kinase	WP_011820303. 888621..890411	Glycolysis	COG0469
WP_011820303.1_956	CUST_5065_PI442190949	MIT9515	HLI	WP_011820303.1_956	TGTCGCAAGAAGATTACAACTTGTGTGGGGAGTGACACCATTATTAGTAAAATCTGACGA	pykF	Pyruvate kinase	WP_011820303. 888621..890411	Glycolysis	COG0469
WP_011820303.1_956	CUST_5066_PI442190949	MIT9515	HLI	WP_011820303.1_956	CCATTAAAAGCAATCGAGAGTAATCTGCCTAGTACTATCCCAAATGCAATAAGTGCTGCA	pykF	Pyruvate kinase	WP_011820303. 888621..890411	Glycolysis	COG0469
WP_011820303.1_956	CUST_5067_PI442190949	MIT9515	HLI	WP_011820303.1_956	AGCAAGAACATTTAGTATTGCAATGCAAATAGCTCAAGAAATGGGGATTCTTAAGCAAGG	pykF	Pyruvate kinase	WP_011820303. 888621..890411	Glycolysis	COG0469
WP_011820311.1_965	CUST_5111_PI442190949	MIT9515	HLI	WP_011820311.1_965	AGGTTTTGAAGCGCCAGTAAATTTAGTTTATTCTGAAGGAAATCGCTCGGCAGCTGTAAG	glnA	Glutamine synthetase/ glutamate--ammonialigase	WP_011820311. 897619..899040	Nitrogen Metabolism	COG0174
WP_011820311.1_965	CUST_5112_PI442190949	MIT9515	HLI	WP_011820311.1_965	CCTGGTGATGGAGTAGATGTGGATCTATTTGAATTACCAGCCGAAGAGCTCGCAAAAATA	glnA	Glutamine synthetase/ glutamate--ammonialigase	WP_011820311. 897619..899040	Nitrogen Metabolism	COG0174
WP_011820311.1_965	CUST_5113_PI442190949	MIT9515	HLI	WP_011820311.1_965	ATGTTGTAAGAAACGTAGCCAAAAAATACGGCAAAACTGCCACCTTTATGCCAAAACCTG	glnA	Glutamine synthetase/ glutamate--ammonialigase	WP_011820311. 897619..899040	Nitrogen Metabolism	COG0174
WP_011820311.1_965	CUST_5114_PI442190949	MIT9515	HLI	WP_011820311.1_965	CACCATGAAGTAGCCGGAGCAGGACAACATGAACTTGGAATGAAATTTGATTCCCTAATT	glnA	Glutamine synthetase/ glutamate--ammonialigase	WP_011820311. 897619..899040	Nitrogen Metabolism	COG0174
WP_011820311.1_965	CUST_5115_PI442190949	MIT9515	HLI	WP_011820311.1_965	CAAACAGCAAGATGGTATATCGGAGGTATTTTGAAGCACGCTCCATCATTCCTTGCATTT	glnA	Glutamine synthetase/ glutamate--ammonialigase	WP_011820311. 897619..899040	Nitrogen Metabolism	COG0174
WP_011820311.1_965	CUST_5116_PI442190949	MIT9515	HLI	WP_011820311.1_965	GATCAGGAGATGCACTTGCTAACCCTTATTTAGCATTTTCTGTAATGATGTTAGCTGGCA	glnA	Glutamine synthetase/ glutamate--ammonialigase	WP_011820311. 897619..899040	Nitrogen Metabolism	COG0174
WP_011820321.1_976	CUST_5169_PI442190949	MIT9515	HLI	WP_011820321.1_976	TGGGATCAAGATAGTTGCTTGTAGTACCCCCACAAATGCAAAAGGATTAATGAAAGCTGC	pdhB	Pyruvate dehydrogenase E1 component beta subunit	WP_011820321. 906847..907830	Glycolysis/Krebs cycle	COG0022
WP_011820321.1_976	CUST_5170_PI442190949	MIT9515	HLI	WP_011820321.1_976	TTGGTGCTGCAATGACAGGATTAAGACCGATAGTGGAAGGTATGAACATGGGATTTCTAC	pdhB	Pyruvate dehydrogenase E1 component beta subunit	WP_011820321. 906847..907830	Glycolysis/Krebs cycle	COG0022
WP_011820321.1_976	CUST_5171_PI442190949	MIT9515	HLI	WP_011820321.1_976	ATATGGGCATGCTCAGATATACAAGTGGGGGCAATTATAAAATACCTGCAGTTGTTCGTG	pdhB	Pyruvate dehydrogenase E1 component beta subunit	WP_011820321. 906847..907830	Glycolysis/Krebs cycle	COG0022
WP_011820321.1_976	CUST_5172_PI442190949	MIT9515	HLI	WP_011820321.1_976	TTGGGAGACAACTAGGCGCTGAACATAGTCAAAGATTAGAGGCTTACTTTCACGCAGTTC	pdhB	Pyruvate dehydrogenase E1 component beta subunit	WP_011820321. 906847..907830	Glycolysis/Krebs cycle	COG0022
WP_011820321.1_976	CUST_5173_PI442190949	MIT9515	HLI	WP_011820321.1_976	TGCCTGAGGGAGATTATATCTGTTCTTTAGATCAAGCTGACATAGTCAAAGAGGGGAAAG	pdhB	Pyruvate dehydrogenase E1 component beta subunit	WP_011820321. 906847..907830	Glycolysis/Krebs cycle	COG0022
WP_011820321.1_976	CUST_5174_PI442190949	MIT9515	HLI	WP_011820321.1_976	ACTGGAGGAATTGGAGCAGAATTGATTGCTTTGATTACAGAAGAATGTTTTGATGATCTT	pdhB	Pyruvate dehydrogenase E1 component beta subunit	WP_011820321. 906847..907830	Glycolysis/Krebs cycle	COG0022
WP_011820442.1_1084	CUST_5682_PI442190949	MIT9515	HLI	WP_011820442.1_1084	TGTTGCATGGCGTGATAAACCAACAAGAAAAGGTTTTAGTGTTATTGACTCTGAAGGCGA	rbsK	Sugar kinase, ribokinase family	WP_011820442. 987956..988825	Pentose Phosphate	COG0524
WP_011820442.1_1084	CUST_5683_PI442190949	MIT9515	HLI	WP_011820442.1_1084	GCCTAAGCCAGGTTTAATCTCTCATTCAAGAAAATCTCTAGAATATCCTGCGGGTGGAGG	rbsK	Sugar kinase, ribokinase family	WP_011820442. 987956..988825	Pentose Phosphate	COG0524
WP_011820442.1_1084	CUST_5684_PI442190949	MIT9515	HLI	WP_011820442.1_1084	GGATGTGGAGATTCTTTCGCCGCTGGTATCCTTTATGGATTATCATCAGGATGGAACATA	rbsK	Sugar kinase, ribokinase family	WP_011820442. 987956..988825	Pentose Phosphate	COG0524
WP_011820442.1_1084	CUST_5685_PI442190949	MIT9515	HLI	WP_011820442.1_1084	AACGGAAGGAGAGAATGGTGGGATAGTATTCCCAGGAGGTAGATATAAAGCTATTGAAAA	rbsK	Sugar kinase, ribokinase family	WP_011820442. 987956..988825	Pentose Phosphate	COG0524
WP_011820442.1_1084	CUST_5686_PI442190949	MIT9515	HLI	WP_011820442.1_1084	ATTGTGAAATCGTTGTGTACTACTCCGAGAGCAGGTTTAGATATTATTAATGACTCTGGT	rbsK	Sugar kinase, ribokinase family	WP_011820442. 987956..988825	Pentose Phosphate	COG0524
WP_011820442.1_1084	CUST_5687_PI442190949	MIT9515	HLI	WP_011820442.1_1084	TGCTAAAAGACTTCGTGAACTAACCAAGAGCGAAGTTCATTTTTTTACATCTTTAGGAGA	rbsK	Sugar kinase, ribokinase family	WP_011820442. 987956..988825	Pentose Phosphate	COG0524
WP_011820470.1_1112	CUST_5839_PI442190949	MIT9515	HLI	WP_011820470.1_1112	TGAGACTTGAAGCCGGAGGTATGGCTGATATGCAATTAATAGAACAAGTTGTCCCAAATG	opcA	Glucose-6-P dehydrogenase subunit opcA	WP_011820470. 1016594..1017895	Pentose Phosphate	COG3429
WP_011820470.1_1112	CUST_5840_PI442190949	MIT9515	HLI	WP_011820470.1_1112	AGACTTAAAAGTTGGCGTGAATCATTAGCAATGATTTTTGATCCTCCATCGAGGAGACCT	opcA	Glucose-6-P dehydrogenase subunit opcA	WP_011820470. 1016594..1017895	Pentose Phosphate	COG3429
WP_011820470.1_1112	CUST_5841_PI442190949	MIT9515	HLI	WP_011820470.1_1112	TCCATAAAAGAATTACCTACTTGGTTATGGTGGAATGGAAGCCTAGATGAATCCCCAGAA	opcA	Glucose-6-P dehydrogenase subunit opcA	WP_011820470. 1016594..1017895	Pentose Phosphate	COG3429
WP_011820470.1_1112	CUST_5842_PI442190949	MIT9515	HLI	WP_011820470.1_1112	GGGGAGTAGTAGAGGAAATACTAGCCCACTTTTTGAAAATGCTATTAAGGTTGCAGTTCA	opcA	Glucose-6-P dehydrogenase subunit opcA	WP_011820470. 1016594..1017895	Pentose Phosphate	COG3429
WP_011820470.1_1112	CUST_5843_PI442190949	MIT9515	HLI	WP_011820470.1_1112	ACCCTTTACCTTTAGGTAATCCAAGTATTCATTCGGGACAAGTCATTGGATTAAGGTTGA	opcA	Glucose-6-P dehydrogenase subunit opcA	WP_011820470. 1016594..1017895	Pentose Phosphate	COG3429
WP_011820470.1_1112	CUST_5844_PI442190949	MIT9515	HLI	WP_011820470.1_1112	ACTGCAAATGGCTCGCCTAATAGATGCTTAAAGATTCTTGATCAATCAATAAGGTCAAAT	opcA	Glucose-6-P dehydrogenase subunit opcA	WP_011820470. 1016594..1017895	Pentose Phosphate	COG3429
WP_011820471.1_1113	CUST_5845_PI442190949	MIT9515	HLI	WP_011820471.1_1113	TTCTTCAAGCGTCAAAACTTGCTGATGAGAATCAACCATGGAATTGCTGTATAAGAGGGC	zwf	glucose-6-phosphate dehydrogenase zwf	WP_011820471. 1017903..1019426	Pentose Phosphate	COG0364
WP_011820471.1_1113	CUST_5846_PI442190949	MIT9515	HLI	WP_011820471.1_1113	ATATGCTCCAAAATCATTTAACTCAAATGCTTGCTGTTACTGCAATGGAGCCACCAGGTA	zwf	glucose-6-phosphate dehydrogenase zwf	WP_011820471. 1017903..1019426	Pentose Phosphate	COG0364
WP_011820471.1_1113	CUST_5847_PI442190949	MIT9515	HLI	WP_011820471.1_1113	AGTCAAATCACCCGGTTCAGGGATGAAATCAAGACCAGTTGAGATGGAATTTTCTTACGA	zwf	glucose-6-phosphate dehydrogenase zwf	WP_011820471. 1017903..1019426	Pentose Phosphate	COG0364
WP_011820471.1_1113	CUST_5848_PI442190949	MIT9515	HLI	WP_011820471.1_1113	GGGGGCCTCCTGAATCAGATCAATTACTTTCTAAAGATAATATTTTCTGGCGCAGACCCT	zwf	glucose-6-phosphate dehydrogenase zwf	WP_011820471. 1017903..1019426	Pentose Phosphate	COG0364
WP_011820471.1_1113	CUST_5849_PI442190949	MIT9515	HLI	WP_011820471.1_1113	GGCTGATGCTATGCTTTCTGATCCAACATTATTCACAAGAAGTGATGAGGTAGAAGCTGC	zwf	glucose-6-phosphate dehydrogenase zwf	WP_011820471. 1017903..1019426	Pentose Phosphate	COG0364
WP_011820471.1_1113	CUST_5850_PI442190949	MIT9515	HLI	WP_011820471.1_1113	TGGCGTTGGCAAGGAGTTCCTTTTTACTTAAGAACAGGTAAAAGGCTACCTAAAAGACTT	zwf	glucose-6-phosphate dehydrogenase zwf	WP_011820471. 1017903..1019426	Pentose Phosphate	COG0364
WP_011820569.1_1203	CUST_6299_PI442190949	MIT9515	HLI	WP_011820569.1_1203	ACGATAAAAGGCTAAGAAATGGTAATAGAGGTTGGGCGACAGTTAAACCTGCTCCTGATC	ctaC/coxB	Cytochrome c oxidase subunit II	WP_011820569. 1108682..1109617	Respiration	COG1622
WP_011820569.1_1203	CUST_6300_PI442190949	MIT9515	HLI	WP_011820569.1_1203	GCATTTGAAACCACTATTGGTACTTTTATCTTTCTGGGCTGTACAGGAACAATGGCTTGG	ctaC/coxB	Cytochrome c oxidase subunit II	WP_011820569. 1108682..1109617	Respiration	COG1622
WP_011820569.1_1203	CUST_6301_PI442190949	MIT9515	HLI	WP_011820569.1_1203	TGGACCTATTGACGTGATTTCAAGGCAATGGAATTGGGAGTTTGTTTATCCAAATGGAAT	ctaC/coxB	Cytochrome c oxidase subunit II	WP_011820569. 1108682..1109617	Respiration	COG1622
WP_011820569.1_1203	CUST_6302_PI442190949	MIT9515	HLI	WP_011820569.1_1203	CGCTTACACCTACTAAAGAAGGTGTATATAGATTGAGAGACGCAATGTTTAGTGGTGCCT	ctaC/coxB	Cytochrome c oxidase subunit II	WP_011820569. 1108682..1109617	Respiration	COG1622
WP_011820569.1_1203	CUST_6303_PI442190949	MIT9515	HLI	WP_011820569.1_1203	GGATTAATGAGACTGTAAAAAAAGAACCTCAAAATGGATTAAATCCTGCTGGCCGTTTGT	ctaC/coxB	Cytochrome c oxidase subunit II	WP_011820569. 1108682..1109617	Respiration	COG1622
WP_011820569.1_1203	CUST_6304_PI442190949	MIT9515	HLI	WP_011820569.1_1203	AGAGCTCCTAAGTATGACGAAAGCAATGGTGAACCTTTAGAGGGAAATTTAAAACTTGAA	ctaC/coxB	Cytochrome c oxidase subunit II	WP_011820569. 1108682..1109617	Respiration	COG1622
WP_011820570.1_1204	CUST_6305_PI442190949	MIT9515	HLI	WP_011820570.1_1204	CCCCTCTCCTCCGCCTCACGAAAATTTTGAAGAAGATATTCCAACGGTTTTAAATGAACC	cyoB/coxA	Cytochrome c oxidase subunit I	WP_011820570. 1109626..1111317	Respiration	COG0843
WP_011820570.1_1204	CUST_6306_PI442190949	MIT9515	HLI	WP_011820570.1_1204	GGAATCACTGGCGTAATGCTTGCAACAGTGCCAGTAGACATTCATGTTGGAAATACATAT	cyoB/coxA	Cytochrome c oxidase subunit I	WP_011820570. 1109626..1111317	Respiration	COG0843
WP_011820570.1_1204	CUST_6307_PI442190949	MIT9515	HLI	WP_011820570.1_1204	GGGCTGGTTTATTAGGAATGCCGAGAAGAGTTGCCTCTTATGATCCTGAATTTGCAATTT	cyoB/coxA	Cytochrome c oxidase subunit I	WP_011820570. 1109626..1111317	Respiration	COG0843
WP_011820570.1_1204	CUST_6308_PI442190949	MIT9515	HLI	WP_011820570.1_1204	CTACATTATGGGGCGGAAATATTAGGTTAAGTACACCCATGCTTTTTTGTTTGGGGGGAT	cyoB/coxA	Cytochrome c oxidase subunit I	WP_011820570. 1109626..1111317	Respiration	COG0843
WP_011820570.1_1204	CUST_6309_PI442190949	MIT9515	HLI	WP_011820570.1_1204	TATGGTTAGTTCATGGGTTAGAGGTAAATCAGCTCCCCCAAATCCTTGGAAAGCTATAGG	cyoB/coxA	Cytochrome c oxidase subunit I	WP_011820570. 1109626..1111317	Respiration	COG0843
WP_011820570.1_1204	CUST_6310_PI442190949	MIT9515	HLI	WP_011820570.1_1204	TCTTTCATTAGTTGTTTGGGTTCATCACATGTTCTATACAGGCACACCAATGTGGATGAG	cyoB/coxA	Cytochrome c oxidase subunit I	WP_011820570. 1109626..1111317	Respiration	COG0843
WP_011820725.1_1355	CUST_7109_PI442190949	MIT9515	HLI	WP_011820725.1_1355	AGACTCAAAAATATCAGTATCTAAATCAGCTTTAGGAGCTGCTGAAGGCGCAGATGCAGT	ugd	UDP-glucose 6-dehydrogenase/nucleotide sugar dehydrogenase	WP_011820725. 1258958..1260355	Amino sugar and nucleotide sugar r	COG1004
WP_011820725.1_1355	CUST_7110_PI442190949	MIT9515	HLI	WP_011820725.1_1355	TGATACTAGAATTGGAAATAAGTTTCTTAATGCAGGTCCAGGCTTTGGAGGGAGCTGCTT	ugd	UDP-glucose 6-dehydrogenase/nucleotide sugar dehydrogenase	WP_011820725. 1258958..1260355	Amino sugar and nucleotide sugar r	COG1004
WP_011820725.1_1355	CUST_7111_PI442190949	MIT9515	HLI	WP_011820725.1_1355	TTGGCCCAGAGAATATCTTCTGTTAATTCTATTTCTGCTCTATGCGAATCAACTGGAGCT	ugd	UDP-glucose 6-dehydrogenase/nucleotide sugar dehydrogenase	WP_011820725. 1258958..1260355	Amino sugar and nucleotide sugar r	COG1004
WP_011820725.1_1355	CUST_7112_PI442190949	MIT9515	HLI	WP_011820725.1_1355	TGGACTCAATGAGGTTGCTGCATATTGGGAGCAAATAGTTCAAATTAATTTATGGCAACA	ugd	UDP-glucose 6-dehydrogenase/nucleotide sugar dehydrogenase	WP_011820725. 1258958..1260355	Amino sugar and nucleotide sugar r	COG1004
WP_011820725.1_1355	CUST_7113_PI442190949	MIT9515	HLI	WP_011820725.1_1355	AAGTCATGAGAAAACCTGCATGGGTTTTTGATACTCGAATTTGCTTAAATCGAGAAGAAA	ugd	UDP-glucose 6-dehydrogenase/nucleotide sugar dehydrogenase	WP_011820725. 1258958..1260355	Amino sugar and nucleotide sugar r	COG1004
WP_011820725.1_1355	CUST_7114_PI442190949	MIT9515	HLI	WP_011820725.1_1355	TGATACGAGAGAGTCTCCTAGTATTAATATCTCTAAAGAGTTTTTAAAGGAAGGAGCAGA	ugd	UDP-glucose 6-dehydrogenase/nucleotide sugar dehydrogenase	WP_011820725. 1258958..1260355	Amino sugar and nucleotide sugar r	COG1004
WP_011820753.1_1381	CUST_7258_PI442190949	MIT9515	HLI	WP_011820753.1_1381	AGAGCTAGAGCAGGTGAGGGTCCTACTCTACTCGAATGCTTAACTTACAGATTTAGAGGT	pdhA	Pyruvate dehydrogenase E1 component alpha subunit	WP_011820753. 1284254..1285291	Glycolysis/Krebs cycle	COG1071
WP_011820753.1_1381	CUST_7259_PI442190949	MIT9515	HLI	WP_011820753.1_1381	CATCATCTCTTAGGAGGATATGCTTTTATTGGTGAGGGCATTCCAGTTGCTTTAGGGTCA	pdhA	Pyruvate dehydrogenase E1 component alpha subunit	WP_011820753. 1284254..1285291	Glycolysis/Krebs cycle	COG1071
WP_011820753.1_1381	CUST_7260_PI442190949	MIT9515	HLI	WP_011820753.1_1381	ATATAAAAAAGTAGTTGCAGGTGACAGCCAAAGCGACTCTGTAACAGCTGCTTTCTTTGG	pdhA	Pyruvate dehydrogenase E1 component alpha subunit	WP_011820753. 1284254..1285291	Glycolysis/Krebs cycle	COG1071
WP_011820753.1_1381	CUST_7261_PI442190949	MIT9515	HLI	WP_011820753.1_1381	CATGTTCATGCTCTAAGTGCAGGTGTCCCTTCTTTTGAAGTGATGAGTGAATTATTCGGC	pdhA	Pyruvate dehydrogenase E1 component alpha subunit	WP_011820753. 1284254..1285291	Glycolysis/Krebs cycle	COG1071
WP_011820753.1_1381	CUST_7262_PI442190949	MIT9515	HLI	WP_011820753.1_1381	TTTGTAGTTGAAAACAATAAATGGGCTATAGGCATGGCTCATGACAGAGCTACAAGCGAC	pdhA	Pyruvate dehydrogenase E1 component alpha subunit	WP_011820753. 1284254..1285291	Glycolysis/Krebs cycle	COG1071
WP_011820753.1_1381	CUST_7263_PI442190949	MIT9515	HLI	WP_011820753.1_1381	GAAATATGGAGAAAAGCATCGGCTTTCGGGATGCATGGTGAAGAAGTTGATGGAATGGAC	pdhA	Pyruvate dehydrogenase E1 component alpha subunit	WP_011820753. 1284254..1285291	Glycolysis/Krebs cycle	COG1071
WP_011820774.1_1402	CUST_7381_PI442190949	MIT9515	HLI	WP_011820774.1_1402	GAAGGCAAAAGGAGAATGAGGCAAGCAGAAGAAGGTATTGCAAGACTAGCTGAAAATGTT	ftsZ	cell division protein FtsZ	WP_011820774. 1307400..1308515	Cell Division	COG0206
WP_011820774.1_1402	CUST_7382_PI442190949	MIT9515	HLI	WP_011820774.1_1402	GTTACGGGAGGTGCTTCGATTCAAGAAGCCTTCAGAAACGCTGATGATGTTTTAAGAATG	ftsZ	cell division protein FtsZ	WP_011820774. 1307400..1308515	Cell Division	COG0206
WP_011820774.1_1402	CUST_7383_PI442190949	MIT9515	HLI	WP_011820774.1_1402	AATCAATGGAAGGGGAGGTACAAGTAACTGTTATTGCAACAGGTTTCGAAACCAACCAAC	ftsZ	cell division protein FtsZ	WP_011820774. 1307400..1308515	Cell Division	COG0206
WP_011820774.1_1402	CUST_7384_PI442190949	MIT9515	HLI	WP_011820774.1_1402	AATGTTGATTTTGCTGATGTTCGATCTGTAATGACTGAAGCTGGGACAGCTCTTCTAGGT	ftsZ	cell division protein FtsZ	WP_011820774. 1307400..1308515	Cell Division	COG0206
WP_011820774.1_1402	CUST_7385_PI442190949	MIT9515	HLI	WP_011820774.1_1402	GGCAGGAAGAATTGATGGGGCCAAAGGTTGTGTTATTAACATCACTGGAGGTAAAGATTT	ftsZ	cell division protein FtsZ	WP_011820774. 1307400..1308515	Cell Division	COG0206
WP_011820774.1_1402	CUST_7386_PI442190949	MIT9515	HLI	WP_011820774.1_1402	GCTGAGGTTGCAAAACAAAGTGGTGCCTTAACTGTAGGAATAGTTACAAAGCCGTTTTCT	ftsZ	cell division protein FtsZ	WP_011820774. 1307400..1308515	Cell Division	COG0206
WP_011820787.1_1418	CUST_7461_PI442190949	MIT9515	HLI	WP_011820787.1_1418	AGATGAAGATCCAGAAAAACCAGCAGGACTATATACCGCTTGGATGGTTCTTATTCAGAA	glcH	GPH family sugar transporter	WP_011820787. 1320039..1321388	GlcH sugar transporter	COG2211
WP_011820787.1_1418	CUST_7462_PI442190949	MIT9515	HLI	WP_011820787.1_1418	AATTGGTATAGGTGCTTCAACTGCTTACCTAATTCCATGGTCACTTCTTCCTGATGCGAT	glcH	GPH family sugar transporter	WP_011820787. 1320039..1321388	GlcH sugar transporter	COG2211
WP_011820787.1_1418	CUST_7463_PI442190949	MIT9515	HLI	WP_011820787.1_1418	ACTGAATGTGCCTTCAGAAATTGCTAACTGGGTTCCAATACCCTTCCAAATAAGTGCATT	glcH	GPH family sugar transporter	WP_011820787. 1320039..1321388	GlcH sugar transporter	COG2211
WP_011820787.1_1418	CUST_7464_PI442190949	MIT9515	HLI	WP_011820787.1_1418	TTTACTTTGGTGCGCCTTACAGTTAATGCAAACAGTTGCCTTAATTTATGTAGAAGATGT	glcH	GPH family sugar transporter	WP_011820787. 1320039..1321388	GlcH sugar transporter	COG2211
WP_011820787.1_1418	CUST_7465_PI442190949	MIT9515	HLI	WP_011820787.1_1418	CTCCATATGCCAAAAAAGCTAGGAAACCATCTGGGAAGGTCGAACCAATTAAACTCCAAT	glcH	GPH family sugar transporter	WP_011820787. 1320039..1321388	GlcH sugar transporter	COG2211
WP_011820787.1_1418	CUST_7466_PI442190949	MIT9515	HLI	WP_011820787.1_1418	AATGGGTAAAATCAGTGGGTTTATAGCGGTAACAACAACCTTAATATCTTGTTGGGGATT	glcH	GPH family sugar transporter	WP_011820787. 1320039..1321388	GlcH sugar transporter	COG2211
WP_011820806.1_1437	CUST_7572_PI442190949	MIT9515	HLI	WP_011820806.1_1437	AGTTTGTGATTGGAGTTACGGGGTATTCAAAGCAAGAGGAGATAGCAGGTTTCTTCACAA	kaiC	circadian clock protein KaiC	WP_011820806. 1339194..1340723	Circadian Rhythm	COG0467
WP_011820806.1_1437	CUST_7573_PI442190949	MIT9515	HLI	WP_011820806.1_1437	TACTGGTGCCACTGGCACAGGTAAAACTATGCTGGTATCAAAGTTTATTGAGGATGCTTA	kaiC	circadian clock protein KaiC	WP_011820806. 1339194..1340723	Circadian Rhythm	COG0467
WP_011820806.1_1437	CUST_7574_PI442190949	MIT9515	HLI	WP_011820806.1_1437	AAGAGATTGGCAATCGACTCCCTCTCAGCATTAGCTAGAGGTGTAAGTTTGAATGCTTTT	kaiC	circadian clock protein KaiC	WP_011820806. 1339194..1340723	Circadian Rhythm	COG0467
WP_011820806.1_1437	CUST_7575_PI442190949	MIT9515	HLI	WP_011820806.1_1437	AGAGATTAAAGGAGAAATGGCAAGAGCAATAAATGTATTTAAAATGAGAGGCTCATGGCA	kaiC	circadian clock protein KaiC	WP_011820806. 1339194..1340723	Circadian Rhythm	COG0467
WP_011820806.1_1437	CUST_7576_PI442190949	MIT9515	HLI	WP_011820806.1_1437	AGGTCCTGAAATAAAAGATTCATTTTCAAATTTTGAACAAATATTTAGCGGTGCCCCTCA	kaiC	circadian clock protein KaiC	WP_011820806. 1339194..1340723	Circadian Rhythm	COG0467
WP_011820806.1_1437	CUST_7577_PI442190949	MIT9515	HLI	WP_011820806.1_1437	ACTGCTGAGGAATTTATGGGAAGTCATTCCATTACAGACTCTCACATATCAACTATTACA	kaiC	circadian clock protein KaiC	WP_011820806. 1339194..1340723	Circadian Rhythm	COG0467
WP_011820807.1_1438	CUST_7578_PI442190949	MIT9515	HLI	WP_011820807.1_1438	GGTTTTAATAGGTTTAGATTTACTTTTTGATGAATTATCAGAGAGTGAATTGAATGGGGG	kaiB	Circadian clock protein KaiB	WP_011820807. 1340785..1341111	Circadian Rhythm	COG4251
WP_011820807.1_1438	CUST_7579_PI442190949	MIT9515	HLI	WP_011820807.1_1438	GGCTACTCCAACTCTTGCCAAGATTTTACCTCCGCCTGTTAGAAGAATAATTGGTGATCT	kaiB	Circadian clock protein KaiB	WP_011820807. 1340785..1341111	Circadian Rhythm	COG4251
WP_011820807.1_1438	CUST_7580_PI442190949	MIT9515	HLI	WP_011820807.1_1438	AGGAGTTTATGCTTTAAAAGTAATAGATGTCCTTAAACAACCACAACTTGCAGAAGAAGA	kaiB	Circadian clock protein KaiB	WP_011820807. 1340785..1341111	Circadian Rhythm	COG4251
WP_011820807.1_1438	CUST_7581_PI442190949	MIT9515	HLI	WP_011820807.1_1438	TACTCCTAATTCAATGAGAGCTCTAAATACTCTTAAAGAAATTCTA	kaiB	Circadian clock protein KaiB	WP_011820807. 1340785..1341111	Circadian Rhythm	COG4251
WP_011820807.1_1438	CUST_7582_PI442190949	MIT9515	HLI	WP_011820807.1_1438	TGGCGGCAAGAAAAACTTATATTCTAAAACTTTACGTAGCAGGTAATACTCCTAATTCAA	kaiB	Circadian clock protein KaiB	WP_011820807. 1340785..1341111	Circadian Rhythm	COG4251
WP_011820920.1_1524	CUST_7980_PI442190949	MIT9515	HLI	WP_011820920.1_1524	AGTCCAGAAAAGATTAAAGCTCTTAATGAAATTTCAAAGGCTGAGGCAAGATTTCAAGCA	atpC	ATP synthase epsilon subunit	WP_011820920. 1414805..1415209	Energy Metabolism	COG0355
WP_011820920.1_1524	CUST_7981_PI442190949	MIT9515	HLI	WP_011820920.1_1524	AGTGCTGAAATTGGTTCTGAAATTAATACCCAAAATGCTGAAGAAGCTCTTAAAAAAGCA	atpC	ATP synthase epsilon subunit	WP_011820920. 1414805..1415209	Energy Metabolism	COG0355
WP_011820920.1_1524	CUST_7982_PI442190949	MIT9515	HLI	WP_011820920.1_1524	TTCAAAATGGACATCAATAGCTTTGATGGGTGGCTTTGCTGAAATTGAATCTGATGAAGT	atpC	ATP synthase epsilon subunit	WP_011820920. 1414805..1415209	Energy Metabolism	COG0355
WP_011820920.1_1524	CUST_7983_PI442190949	MIT9515	HLI	WP_011820920.1_1524	TCCGGGTCACATATCCTTGGTTACGGCAATTGATATTGGTGTGTTAAGGCTAAGAATGAA	atpC	ATP synthase epsilon subunit	WP_011820920. 1414805..1415209	Energy Metabolism	COG0355
WP_011820920.1_1524	CUST_7984_PI442190949	MIT9515	HLI	WP_011820920.1_1524	AAATGTTTACGAAGGTGATGCTGAAGAGGTAATTCTTCCAAGTACGACCGGTCAGATTGG	atpC	ATP synthase epsilon subunit	WP_011820920. 1414805..1415209	Energy Metabolism	COG0355
WP_011820932.1_1535	CUST_8040_PI442190949	MIT9515	HLI	WP_011820932.1_1535	AATTGCTTAAACAAGCACAATTCTCACCATTAAACCTTGCTGAACAAGTGGCGGTTGTTT	atpA	ATP synthase alpha subunit	WP_011820932. 1424492..1426009	Energy Metabolism	COG0056
WP_011820932.1_1535	CUST_8041_PI442190949	MIT9515	HLI	WP_011820932.1_1535	GTTAGTAGAGTTGGTGGGGCTGCGCAAACAAAAGCAATTAAGAAAATAGCTGGAACACTA	atpA	ATP synthase alpha subunit	WP_011820932. 1424492..1426009	Energy Metabolism	COG0056
WP_011820932.1_1535	CUST_8042_PI442190949	MIT9515	HLI	WP_011820932.1_1535	TTATCTGACGATATGGGTGGTGGATCAATGACAGCGCTACCAATTATTGAAACTCAAGCT	atpA	ATP synthase alpha subunit	WP_011820932. 1424492..1426009	Energy Metabolism	COG0056
WP_011820932.1_1535	CUST_8043_PI442190949	MIT9515	HLI	WP_011820932.1_1535	AGCATTTTCTCAATTCGCCTCTGATCTTGATGAGGCAACTCAACAACAACTTGAAAGAGG	atpA	ATP synthase alpha subunit	WP_011820932. 1424492..1426009	Energy Metabolism	COG0056
WP_011820932.1_1535	CUST_8044_PI442190949	MIT9515	HLI	WP_011820932.1_1535	TTTAACAAAGCAAGCGCAAGCATACAGACAAATGTCTCTTCTTTTGAGAAGACCACCAGG	atpA	ATP synthase alpha subunit	WP_011820932. 1424492..1426009	Energy Metabolism	COG0056
WP_011820932.1_1535	CUST_8045_PI442190949	MIT9515	HLI	WP_011820932.1_1535	TGCAGGTGTTAAGGGTCTTATTGACGAAGTACCTGTTGAAGATGTAACCAAATTTGCTGC	atpA	ATP synthase alpha subunit	WP_011820932. 1424492..1426009	Energy Metabolism	COG0056
WP_041710653.1_1540	CUST_8068_PI442190949	MIT9515	HLI	WP_041710653.1_1540	CAAGTGGAGAATTAGGAGCTCCGACAGCTGATATTAATACAACTATTGCACTGGCCTTGT	atpI	ATP synthase subunit A	WP_041710653. 1428031..1428756	Energy Metabolism	COG0356
WP_041710653.1_1540	CUST_8069_PI442190949	MIT9515	HLI	WP_041710653.1_1540	CAATCCAAGCATTAATCTTCGCTACACTTGCTGCTTACTACATAGGAGAAGCGGTAGAGG	atpI	ATP synthase subunit A	WP_041710653. 1428031..1428756	Energy Metabolism	COG0356
WP_041710653.1_1540	CUST_8070_PI442190949	MIT9515	HLI	WP_041710653.1_1540	TTTTGGGAATATTCTTGCTGATGAACTTGTAGTTGGTGTTTTGGTTTTCCTGGTTCCCCT	atpI	ATP synthase subunit A	WP_041710653. 1428031..1428756	Energy Metabolism	COG0356
WP_041710653.1_1540	CUST_8071_PI442190949	MIT9515	HLI	WP_041710653.1_1540	GGATGCCTTTTATCGGAACTCTATTCTTATTTGTGTTTGTTAGTAATTGGGGAGGGGCGC	atpI	ATP synthase subunit A	WP_041710653. 1428031..1428756	Energy Metabolism	COG0356
WP_041710653.1_1540	CUST_8072_PI442190949	MIT9515	HLI	WP_041710653.1_1540	TGCTGGTTTAAGCAATAAGGGCTGGAGATACTTCGAATATTATGTTCATCCAACACCGAT	atpI	ATP synthase subunit A	WP_041710653. 1428031..1428756	Energy Metabolism	COG0356
WP_041710653.1_1540	CUST_8073_PI442190949	MIT9515	HLI	WP_041710653.1_1540	TCCTTGTCGACAAATTTTGCAGCATTAGAAGTTGGTCAACATCTATATTGGCAAATTGGA	atpI	ATP synthase subunit A	WP_041710653. 1428031..1428756	Energy Metabolism	COG0356
WP_011820947.1_1550	CUST_8124_PI442190949	MIT9515	HLI	WP_011820947.1_1550	CAGCTCAATCATGCCTGGGAAAGTAAATCCTACCCAATGTGAAGCATTATCCATGGTATG	fumC	class II fumarate hydratase	WP_011820947. 1436452..1437837	Krebs cycle and related genes	COG0114
WP_011820947.1_1550	CUST_8125_PI442190949	MIT9515	HLI	WP_011820947.1_1550	TGCCACTAGGTGGTACTGCAGTTGGTACAGGAATTAATTGTCCAAAAGATTTTCCAGAAG	fumC	class II fumarate hydratase	WP_011820947. 1436452..1437837	Krebs cycle and related genes	COG0114
WP_011820947.1_1550	CUST_8126_PI442190949	MIT9515	HLI	WP_011820947.1_1550	TGGGTTTTGAATATGCAGTATCAATAGCCAATGCCAGCGGTACTTTACAAATGAACGAAT	fumC	class II fumarate hydratase	WP_011820947. 1436452..1437837	Krebs cycle and related genes	COG0114
WP_011820947.1_1550	CUST_8127_PI442190949	MIT9515	HLI	WP_011820947.1_1550	TGGCATCTCATGATCGATTAGCAAACGTAATGGGTCAAATAAAAATACTTGCGAGTGCAT	fumC	class II fumarate hydratase	WP_011820947. 1436452..1437837	Krebs cycle and related genes	COG0114
WP_011820947.1_1550	CUST_8128_PI442190949	MIT9515	HLI	WP_011820947.1_1550	ACAGCATTAGTACCCAAAATAGGTTACGAAAAAGCAGCAGAAATTGCGAATCTTGCTTTT	fumC	class II fumarate hydratase	WP_011820947. 1436452..1437837	Krebs cycle and related genes	COG0114
WP_011820947.1_1550	CUST_8129_PI442190949	MIT9515	HLI	WP_011820947.1_1550	ATTATCCTCAGGCCCTAGATCAGGTATTTATGAGTTAATAATTCCTCAGAATGAACCTGG	fumC	class II fumarate hydratase	WP_011820947. 1436452..1437837	Krebs cycle and related genes	COG0114
WP_011820991.1_1596	CUST_8377_PI442190949	MIT9515	HLI	WP_011820991.1_1596	ACTGGGAGAAATATTGGAGCGGGAATGACCGGTGGAATAGCTTACATACTTGATGAAAAT	glsF	Ferredoxin-dependent glutamate synthase	WP_011820991. 1488374..1492948	Nitrogen Metabolism	COG0069
WP_011820991.1_1596	CUST_8378_PI442190949	MIT9515	HLI	WP_011820991.1_1596	ACATGTCTTTATGGTGCGACAGGTGGTAAATTGTTTGCTCTAGGTAAATCCGGAGAAAGA	glsF	Ferredoxin-dependent glutamate synthase	WP_011820991. 1488374..1492948	Nitrogen Metabolism	COG0069
WP_011820991.1_1596	CUST_8379_PI442190949	MIT9515	HLI	WP_011820991.1_1596	CTTAATTTAAATTTTTATGGACACGCGGGGCAGAGCTTCGGTGCTTTTCTTTTAAAGGGG	glsF	Ferredoxin-dependent glutamate synthase	WP_011820991. 1488374..1492948	Nitrogen Metabolism	COG0069
WP_011820991.1_1596	CUST_8380_PI442190949	MIT9515	HLI	WP_011820991.1_1596	GGAGCTATTGCAGTAACAGAGGGTTCTGGTGATCATTGTTGTGAATATATGACTGGTGGT	glsF	Ferredoxin-dependent glutamate synthase	WP_011820991. 1488374..1492948	Nitrogen Metabolism	COG0069
WP_011820991.1_1596	CUST_8381_PI442190949	MIT9515	HLI	WP_011820991.1_1596	AGAAGTGTTTGTGCAAAAATTTCGGGAGAAATCGCTGAGCTTTTTGGAAACAATGGTTTT	glsF	Ferredoxin-dependent glutamate synthase	WP_011820991. 1488374..1492948	Nitrogen Metabolism	COG0069
WP_011820991.1_1596	CUST_8382_PI442190949	MIT9515	HLI	WP_011820991.1_1596	GAATGGAGGTTTACTTACTATTGTTCCACCCAAAGTAGACGAGAAATCTTCGGAGCAAGT	glsF	Ferredoxin-dependent glutamate synthase	WP_011820991. 1488374..1492948	Nitrogen Metabolism	COG0069
WP_011821001.1_1607	CUST_8433_PI442190949	MIT9515	HLI	WP_011821001.1_1607	TTTTTACCCATTGGACCAGCAGATTTCTTGGTTCATCATGCTATTGCTCTTGGACTTCAC	psaB	Photosystem I P700 chlorophyll A apoprotein A2 PsaB	WP_011821001. 1499343..1501571	Photosystem I	COG2885
WP_011821001.1_1607	CUST_8434_PI442190949	MIT9515	HLI	WP_011821001.1_1607	GTTAATTCACTTTCTGTTTGGGCTTGGATGTTCTTATTCGGCCATTTAGTATGGGCCACT	psaB	Photosystem I P700 chlorophyll A apoprotein A2 PsaB	WP_011821001. 1499343..1501571	Photosystem I	COG2885
WP_011821001.1_1607	CUST_8435_PI442190949	MIT9515	HLI	WP_011821001.1_1607	AGCTTATTCCAGACAAGAAAGATTTAGGTTATGCATTCCCATGTGATGGACCAGGGAGAG	psaB	Photosystem I P700 chlorophyll A apoprotein A2 PsaB	WP_011821001. 1499343..1501571	Photosystem I	COG2885
WP_011821001.1_1607	CUST_8436_PI442190949	MIT9515	HLI	WP_011821001.1_1607	GGGACGCCATGTATCTAGCAATGTTCTGGGCATTAAACTTGATAGCATGGGTAACTTTTT	psaB	Photosystem I P700 chlorophyll A apoprotein A2 PsaB	WP_011821001. 1499343..1501571	Photosystem I	COG2885
WP_011821001.1_1607	CUST_8438_PI442190949	MIT9515	HLI	WP_011821001.1_1607	GTGCAGCTAGTGTCGCTGCAAATTCTTTACCGGGAAATCATTACTGGATGGATTTGATTA	psaB	Photosystem I P700 chlorophyll A apoprotein A2 PsaB	WP_011821001. 1499343..1501571	Photosystem I	COG2885
WP_011821002.1_1608	CUST_8439_PI442190949	MIT9515	HLI	WP_011821002.1_1608	TTGGAGGAAAAGTTGCGATGACTGGTATCCCTCTAGGAACTGCTGATTTAATGATTCACC	psaA	Photosystem I P700 chlorophyll A apoprotein A1 PsaA	WP_011821002. 1501600..1503882	Photosystem I	COG2885
WP_011821002.1_1608	CUST_8440_PI442190949	MIT9515	HLI	WP_011821002.1_1608	CAAGAGCACTATCAATTACTCAAGGTCGTGCAGTTGGTGTAACTCACTTCTTAGTGGGTG	psaA	Photosystem I P700 chlorophyll A apoprotein A1 PsaA	WP_011821002. 1501600..1503882	Photosystem I	COG2885
WP_011821002.1_1608	CUST_8441_PI442190949	MIT9515	HLI	WP_011821002.1_1608	AATTCCTGATAAAGCATCACTTGGATTTAGATTCCCATGTGATGGTCCTGGAAGAGGAGG	psaA	Photosystem I P700 chlorophyll A apoprotein A1 PsaA	WP_011821002. 1501600..1503882	Photosystem I	COG2885
WP_011821002.1_1608	CUST_8442_PI442190949	MIT9515	HLI	WP_011821002.1_1608	TCTTGGAAAATGCAAAGTGATGTTTGGGGTCTTACAGGAGGTAATTTTGCTCAAAGTGCA	psaA	Photosystem I P700 chlorophyll A apoprotein A1 PsaA	WP_011821002. 1501600..1503882	Photosystem I	COG2885
WP_011821002.1_1608	CUST_8443_PI442190949	MIT9515	HLI	WP_011821002.1_1608	TATGGGCACAAGCAGCACAGGTATTGACAAGTTACGGTCAATCAATAAGTATGTATGGCT	psaA	Photosystem I P700 chlorophyll A apoprotein A1 PsaA	WP_011821002. 1501600..1503882	Photosystem I	COG2885
WP_011821002.1_1608	CUST_8444_PI442190949	MIT9515	HLI	WP_011821002.1_1608	ACATGTCAGGTTTCTTCCTGGGATCATGTCTTCTTGGCACTTTTCTGGATGTATAATTGT	psaA	Photosystem I P700 chlorophyll A apoprotein A1 PsaA	WP_011821002. 1501600..1503882	Photosystem I	COG2885
WP_011821055.1_1667	CUST_8735_PI442190949	MIT9515	HLI	WP_011821055.1_1667	CTTGATGCAACGCCAGAATGGAACGATTTAATGACTAGGTTGGCAGAAACTTCGAGAATT	ppc	Phosphoenolpyruvate carboxylase	WP_011821055. 1547001..1549970	Krebs cycle and related genes	COG2352
WP_011821055.1_1667	CUST_8736_PI442190949	MIT9515	HLI	WP_011821055.1_1667	ATACCATGGGTTTTTGGATGGACACAGAGTAGATTTCTTTTGCCAAGTTGGTTTGGAGTG	ppc	Phosphoenolpyruvate carboxylase	WP_011821055. 1547001..1549970	Krebs cycle and related genes	COG2352
WP_011821055.1_1667	CUST_8737_PI442190949	MIT9515	HLI	WP_011821055.1_1667	CTTATAGTCGTAGTGAACTTTTAAGGGGCGCACTTTTAACAATTAACGGAATCGCGGCAG	ppc	Phosphoenolpyruvate carboxylase	WP_011821055. 1547001..1549970	Krebs cycle and related genes	COG2352
WP_011821055.1_1667	CUST_8738_PI442190949	MIT9515	HLI	WP_011821055.1_1667	TGGGATTTTTGCAGGTTTCACTTTTAAGAAGACTGAGAGATCAAACAAGACAACCTCCAA	ppc	Phosphoenolpyruvate carboxylase	WP_011821055. 1547001..1549970	Krebs cycle and related genes	COG2352
WP_011821055.1_1667	CUST_8739_PI442190949	MIT9515	HLI	WP_011821055.1_1667	CAAGAGGTAACTCCCATCGAAGAAATTAGTAAATTGCAGATATCCAGTAGACCTGCAAGA	ppc	Phosphoenolpyruvate carboxylase	WP_011821055. 1547001..1549970	Krebs cycle and related genes	COG2352
WP_011821055.1_1667	CUST_8740_PI442190949	MIT9515	HLI	WP_011821055.1_1667	AGTCGCGAAATATTATGTAGATACACTTGGAAGTAAAGAAAATGCAAAGTCATTTGGTGA	ppc	Phosphoenolpyruvate carboxylase	WP_011821055. 1547001..1549970	Krebs cycle and related genes	COG2352
WP_011821087.1_1698	CUST_8914_PI442190949	MIT9515	HLI	WP_011821087.1_1698	CTATAGTCCCTCTCCAATTATTGAGTTACCACATAGCTGCTCATCGAGGCCTCGATGTAG	glmS	Glutamine--fructose-6-phosphatetransaminase(isomerizing)	WP_011821087. 1580020..1581915	Amino Acid metabolism	COG0449
WP_011821087.1_1698	CUST_8915_PI442190949	MIT9515	HLI	WP_011821087.1_1698	AAGAAGCTAAAGCGAGAGATGCATATTTAATTGGTGTTGCCCCCAAATGTAAGGGAACTG	glmS	Glutamine--fructose-6-phosphatetransaminase(isomerizing)	WP_011821087. 1580020..1581915	Amino Acid metabolism	COG0449
WP_011821087.1_1698	CUST_8916_PI442190949	MIT9515	HLI	WP_011821087.1_1698	TTGATATCGGTGCTGGAATTGAAATTGGAGTTGCTGCTACAAAAACTTTCTTTGCGCAAC	glmS	Glutamine--fructose-6-phosphatetransaminase(isomerizing)	WP_011821087. 1580020..1581915	Amino Acid metabolism	COG0449
WP_011821087.1_1698	CUST_8917_PI442190949	MIT9515	HLI	WP_011821087.1_1698	TGGAAATTCAAAGAAGAGCCTCAATGGGAGATGAAAGTTACAAACCAAATTTAGTTGCGA	glmS	Glutamine--fructose-6-phosphatetransaminase(isomerizing)	WP_011821087. 1580020..1581915	Amino Acid metabolism	COG0449
WP_011821087.1_1698	CUST_8918_PI442190949	MIT9515	HLI	WP_011821087.1_1698	AAATCTTTGATTATTTAATGGCCATCCCTTCAACAAATGAGTGGATTTCACCATTGCTTG	glmS	Glutamine--fructose-6-phosphatetransaminase(isomerizing)	WP_011821087. 1580020..1581915	Amino Acid metabolism	COG0449
WP_011821087.1_1698	CUST_8919_PI442190949	MIT9515	HLI	WP_011821087.1_1698	TTTTGGGAAGAGGAATAAATTATCCTATTGCACTTGAGGGGGCTTTAAAGTTAAAGGAAA	glmS	Glutamine--fructose-6-phosphatetransaminase(isomerizing)	WP_011821087. 1580020..1581915	Amino Acid metabolism	COG0449
WP_011821124.1_1735	CUST_9116_PI442190949	MIT9515	HLI	WP_011821124.1_1735	CATCTTGTGCGACATATGGGATACCTCTAGAAGCCGAAATCCCCATAATTGAAAGGACTC	galE	UDP-glucose 4-epimerase	WP_011821124. 1615832..1616878	Galactose metabolism	COG1087
WP_011821124.1_1735	CUST_9117_PI442190949	MIT9515	HLI	WP_011821124.1_1735	GCGCGGACATTAATGGGGACATTGGTGAAGATCATAATCCAGAAACACATTTGATTCCTT	galE	UDP-glucose 4-epimerase	WP_011821124. 1615832..1616878	Galactose metabolism	COG1087
WP_011821124.1_1735	CUST_9118_PI442190949	MIT9515	HLI	WP_011821124.1_1735	TACTGGGAAAGAAATTAGAATATTACAATCCAAAAGACGGCAAGGCGATCCCCCTGTACT	galE	UDP-glucose 4-epimerase	WP_011821124. 1615832..1616878	Galactose metabolism	COG1087
WP_011821124.1_1735	CUST_9119_PI442190949	MIT9515	HLI	WP_011821124.1_1735	CAGCCTCTTAGAGGCTTTGTTGGAAGATTCCAAAAAAAGAAACTCTCAACCAATCCCGAT	galE	UDP-glucose 4-epimerase	WP_011821124. 1615832..1616878	Galactose metabolism	COG1087
WP_011821124.1_1735	CUST_9120_PI442190949	MIT9515	HLI	WP_011821124.1_1735	TGTTATGGAAACCAGAATTCCAAGATTTAGAAAGCATCATACGGACTGCTTGGAATTGGT	galE	UDP-glucose 4-epimerase	WP_011821124. 1615832..1616878	Galactose metabolism	COG1087
WP_011821124.1_1735	CUST_9121_PI442190949	MIT9515	HLI	WP_011821124.1_1735	TTGATTATCACAAAGCTTATGAGCTGCCTGTATCCATACTTCGTTACTTCAATGCTGCAG	galE	UDP-glucose 4-epimerase	WP_011821124. 1615832..1616878	Galactose metabolism	COG1087
WP_011129392.1_13	CUST_73_PI442190945	MIT9313	LLIV	WP_011129392.1_13	AAGGGATTGCCATTGGCTTACAACAAGGATTTTCAGGAGGATAAGGAAGCTCTTTTTGAC	argH	Argininosuccinate lyase	WP_011129392.: 15697..17109	Amino Acid metabolism	COG0165
WP_011129392.1_13	CUST_74_PI442190945	MIT9313	LLIV	WP_011129392.1_13	GATCTAAGCCTTGAACAATGGCAGGAATTTCACTCTGCTATTGCTGAGGATTTGCATGAA	argH	Argininosuccinate lyase	WP_011129392.: 15697..17109	Amino Acid metabolism	COG0165
WP_011129392.1_13	CUST_75_PI442190945	MIT9313	LLIV	WP_011129392.1_13	CACTACTAAAGATTGTGTGGAAGCCATGTCGATTTTGATGGAGGAAGGGTTGGAGTTTTG	argH	Argininosuccinate lyase	WP_011129392.: 15697..17109	Amino Acid metabolism	COG0165
WP_011129392.1_13	CUST_76_PI442190945	MIT9313	LLIV	WP_011129392.1_13	CCTATTGATCGCCAAAATACAGCAGCAGCACTTGGCTTTGAGTGTATTTACGCGAATAG	argH	Argininosuccinate lyase	WP_011129392.: 15697..17109	Amino Acid metabolism	COG0165
WP_011129392.1_13	CUST_77_PI442190945	MIT9313	LLIV	WP_011129392.1_13	CTTGATGCCTCAGAAGAAGAATCCTGATGTGCCTGAATTGGTACGAGGCAAATGTG	argH	Argininosuccinate lyase	WP_011129392.: 15697..17109	Amino Acid metabolism	COG0165
WP_011129392.1_13	CUST_78_PI442190945	MIT9313	LLIV	WP_011129392.1_13	GTTGAATCCGACTTTTCCAATGCCACTGATGTGGCTGACTACTTGGTTGCTAAAGGAGTG	argH	Argininosuccinate lyase	WP_011129392.: 15697..17109	Amino Acid metabolism	COG0165
WP_011129465.1_88	CUST_484_PI442190945	MIT9313	LLIV	WP_011129465.1_88	CATCAGGAGGAACCGTTTATGGCATTCCTAAACAGGTACCAATTGCTGAGAATCATCCTA	galE	UDP-glucose 4-epimerase	WP_011129465.: 93599..94525	Galactose metabolism	COG1087
WP_011129465.1_88	CUST_485_PI442190945	MIT9313	LLIV	WP_011129465.1_88	GAGATGGCAGCACCATCCGCGATTTTCTTTATATTACCGATGTTGTTCAAGCTCTTCTTG	galE	UDP-glucose 4-epimerase	WP_011129465.: 93599..94525	Galactose metabolism	COG1087
WP_011129465.1_88	CUST_486_PI442190945	MIT9313	LLIV	WP_011129465.1_88	AGGTTTGAGTCTTTGTGAACTAGTTAAATTGATTGAGAATGAGCTCGGCCGTCCACTTCA	galE	UDP-glucose 4-epimerase	WP_011129465.: 93599..94525	Galactose metabolism	COG1087
WP_011129465.1_88	CUST_487_PI442190945	MIT9313	LLIV	WP_011129465.1_88	GATTCTTGTCAAGGTGTGGTTCCTGTTTTTCTAAATCGTGCTTTGCGTTCAGAACCTCTC	galE	UDP-glucose 4-epimerase	WP_011129465.: 93599..94525	Galactose metabolism	COG1087
WP_011129465.1_88	CUST_488_PI442190945	MIT9313	LLIV	WP_011129465.1_88	GTCCGAAGGTCTGCGCTAATGATGGGATTCATCGATTTCATCAAAGCATTTTAGGACATT	galE	UDP-glucose 4-epimerase	WP_011129465.: 93599..94525	Galactose metabolism	COG1087
WP_011129465.1_88	CUST_489_PI442190945	MIT9313	LLIV	WP_011129465.1_88	ATATGGATTAAATAGTACGGTAGTCCGCTTAGCGAATCCTTATGGTGAGCGTCAACGTCT	galE	UDP-glucose 4-epimerase	WP_011129465.: 93599..94525	Galactose metabolism	COG1087
WP_041384855.1_131	CUST_741_PI442190945	MIT9313	LLIV	WP_041384855.1_131	CTTTCATTCACTTGGCCCTGAGCTTGGCCAAGCTGATTGTTAATCGACGAACTGGTAATC	sdhA	Succinate dehydrogenase flavoprotein subunit	WP_041384855.: 150083..150715	Krebs cycle and related genes	COG1053
WP_041384855.1_131	CUST_742_PI442190945	MIT9313	LLIV	WP_041384855.1_131	CAGTTTGGGATTGCTTGATCCAGTTAATGGTTTTCGCATTCGGCGCGTCTTTC	sdhA	Succinate dehydrogenase flavoprotein subunit	WP_041384855.: 150083..150715	Krebs cycle and related genes	COG1053
WP_041384855.1_131	CUST_743_PI442190945	MIT9313	LLIV	WP_041384855.1_131	GTGATCGTCTTGCTGTTTTAGCAGCACGTAGTCAGGCGATTGGTGGTTTGATC	sdhA	Succinate dehydrogenase flavoprotein subunit	WP_041384855.: 150083..150715	Krebs cycle and related genes	COG1053
WP_041384855.1_131	CUST_744_PI442190945	MIT9313	LLIV	WP_041384855.1_131	CTTTGAGATTTATGCAGCGGCCTTGCATCAGTCTGCTTGGTTGCCGTTTGCAGAATTTG	sdhA	Succinate dehydrogenase flavoprotein subunit	WP_041384855.: 150083..150715	Krebs cycle and related genes	COG1053
WP_041384855.1_131	CUST_745_PI442190945	MIT9313	LLIV	WP_041384855.1_131	CTACAGGGTTGCTGTTGGTGCTTTTCCTGATGCTCCATTTGGGTGGAGTTGTGATG	sdhA	Succinate dehydrogenase flavoprotein subunit	WP_041384855.: 150083..150715	Krebs cycle and related genes	COG1053
WP_041384855.1_131	CUST_746_PI442190945	MIT9313	LLIV	WP_041384855.1_131	CTTTGTTGGCTCAGCCTGCAAACGTGGCTCTCTATTTATTGGCCGCTG	sdhA	Succinate dehydrogenase flavoprotein subunit	WP_041384855.: 150083..150715	Krebs cycle and related genes	COG1053
WP_011129511.1_132	CUST_747_PI442190945	MIT9313	LLIV	WP_011129511.1_132	CATTACACGATGGGTGGTCTCTGGGTTGATTACTACCTGATGAGTTCGATTCCTGGATTG	sdhA	Succinate dehydrogenase flavoprotein subunit	WP_011129511.: 150715..152631	Krebs cycle and related genes	COG1053
WP_011129511.1_132	CUST_748_PI442190945	MIT9313	LLIV	WP_011129511.1_132	CTGATTCTTTTCATCGTGAGCTTGGAGCTGTGATGATTGATCGCTGCGGTATCAG	sdhA	Succinate dehydrogenase flavoprotein subunit	WP_011129511.: 150715..152631	Krebs cycle and related genes	COG1053
WP_011129511.1_132	CUST_749_PI442190945	MIT9313	LLIV	WP_011129511.1_132	GTTCAGGGACCTAATCCTGAACTCGAGAAGGGGTTGAGGGTTGCTGATTTCTTTGGAATG	sdhA	Succinate dehydrogenase flavoprotein subunit	WP_011129511.: 150715..152631	Krebs cycle and related genes	COG1053
WP_011129511.1_132	CUST_750_PI442190945	MIT9313	LLIV	WP_011129511.1_132	CATGCTGAGCCGCTCAACTTCAAGAGTTTGAAGCCCAGTGAACGTAGTTATCGATG	sdhA	Succinate dehydrogenase flavoprotein subunit	WP_011129511.: 150715..152631	Krebs cycle and related genes	COG1053
WP_011129511.1_132	CUST_751_PI442190945	MIT9313	LLIV	WP_011129511.1_132	GAAGAGCATCAGACAGAAGAAGGCGAAGCTCGGCGTGATGATGAGAACTTTG	sdhA	Succinate dehydrogenase flavoprotein subunit	WP_011129511.: 150715..152631	Krebs cycle and related genes	COG1053
WP_011129511.1_132	CUST_752_PI442190945	MIT9313	LLIV	WP_011129511.1_132	CTAATCGTCTCGGCGCTAGTGCTCTGATGCAAGGTTTGGCTGATGGCTACTTCATTGTG	sdhA	Succinate dehydrogenase flavoprotein subunit	WP_011129511.: 150715..152631	Krebs cycle and related genes	COG1053
WP_011129512.1_133	CUST_753_PI442190945	MIT9313	LLIV	WP_011129512.1_133	TGTCTTTTGATCACGACTGTCGTGAGGGTATTTGTGGTAGCTGTGGCTTTCTTGTTAATG	sdhB	Succinate dehydrogenase iron-sulphur protein subunit	WP_011129512.: 152628..153365	Krebs cycle and related genes	COG0479
WP_011129512.1_133	CUST_754_PI442190945	MIT9313	LLIV	WP_011129512.1_133	GAGCTTTTCCTTTCATTCAAGATTTAGTGGTGGATCGTGCGGCTTTTGATCGGTTGATTG	sdhB	Succinate dehydrogenase iron-sulphur protein subunit	WP_011129512.: 152628..153365	Krebs cycle and related genes	COG0479
WP_011129512.1_133	CUST_755_PI442190945	MIT9313	LLIV	WP_011129512.1_133	GATTTCTGCCGACATGTCGCTATTGGAGGCTCTTGATCGTCTCAATGAACAGCTGATTG	sdhB	Succinate dehydrogenase iron-sulphur protein subunit	WP_011129512.: 152628..153365	Krebs cycle and related genes	COG0479
WP_011129512.1_133	CUST_756_PI442190945	MIT9313	LLIV	WP_011129512.1_133	CAATGACTTGCTGATAGGGAAGGATCAAGCTGCCTCTTCCTTTGAGACGGCTACTTG	sdhB	Succinate dehydrogenase iron-sulphur protein subunit	WP_011129512.: 152628..153365	Krebs cycle and related genes	COG0479
WP_011129512.1_133	CUST_757_PI442190945	MIT9313	LLIV	WP_011129512.1_133	[illegible]	sdhB	Succinate dehydrogenase iron-sulphur protein subunit	WP_011129512.: 152628..153365	Krebs cycle and related genes	COG0479
WP_011129512.1_133	CUST_758_PI442190945	MIT9313	LLIV	WP_011129512.1_133	[illegible]	sdhB	Succinate dehydrogenase iron-sulphur protein subunit	WP_011129512.: 152628..153365	Krebs cycle and related genes	COG0479
WP_011129524.1_145	CUST_825_PI442190945	MIT9313	LLIV	WP_011129524.1_145	[illegible]		sugar ABC transporter substrate-binding protein	WP_011129524.: 163809..165113	Other sugar transporters	
WP_011129524.1_145	CUST_826_PI442190945	MIT9313	LLIV	WP_011129524.1_145	[illegible]		sugar ABC transporter substrate-binding protein	WP_011129524.: 163809..165113	Other sugar transporters	
WP_011129524.1_145	CUST_827_PI442190945	MIT9313	LLIV	WP_011129524.1_145	[illegible]		sugar ABC transporter substrate-binding protein	WP_011129524.: 163809..165113	Other sugar transporters	
WP_011129524.1_145	CUST_828_PI442190945	MIT9313	LLIV	WP_011129524.1_145	[illegible]		sugar ABC transporter substrate-binding protein	WP_011129524.: 163809..165113	Other sugar transporters	
WP_011129524.1_145	CUST_829_PI442190945	MIT9313	LLIV	WP_011129524.1_145	[illegible]		sugar ABC transporter substrate-binding protein	WP_011129524.: 163809..165113	Other sugar transporters	
WP_011129524.1_145	CUST_830_PI442190945	MIT9313	LLIV	WP_011129524.1_145	[illegible]		sugar ABC transporter substrate-binding protein	WP_011129524.: 163809..165113	Other sugar transporters	
WP_011129698.1_333	CUST_1811_PI442190945	MIT9313	LLIV	WP_011129698.1_333	[illegible]	ftsZ	cell division protein FtsZ	WP_011129698.: 365392..366555	Cell Division	COG0206
WP_011129698.1_333	CUST_1812_PI442190945	MIT9313	LLIV	WP_011129698.1_333	[illegible]	ftsZ	cell division protein FtsZ	WP_011129698.: 365392..366555	Cell Division	COG0206
WP_011129698.1_333	CUST_1813_PI442190945	MIT9313	LLIV	WP_011129698.1_333	[illegible]	ftsZ	cell division protein FtsZ	WP_011129698.: 365392..366555	Cell Division	COG0206
WP_011129698.1_333	CUST_1814_PI442190945	MIT9313	LLIV	WP_011129698.1_333	[illegible]	ftsZ	cell division protein FtsZ	WP_011129698.: 365392..366555	Cell Division	COG0206
WP_011129698.1_333	CUST_1815_PI442190945	MIT9313	LLIV	WP_011129698.1_333	[illegible]	ftsZ	cell division protein FtsZ	WP_011129698.: 365392..366555	Cell Division	COG0206
WP_011129698.1_333	CUST_1816_PI442190945	MIT9313	LLIV	WP_011129698.1_333	[illegible]	ftsZ	cell division protein FtsZ	WP_011129698.: 365392..366555	Cell Division	COG0206
WP_011129726.1_360	CUST_1966_PI442190945	MIT9313	LLIV	WP_011129726.1_360	[illegible]	pdhA	Pyruvate dehydrogenase E1 component alpha subunit	WP_011129726.: 395091..396182	Glycolysis/Krebs cycle	COG1071
WP_011129726.1_360	CUST_1967_PI442190945	MIT9313	LLIV	WP_011129726.1_360	[illegible]	pdhA	Pyruvate dehydrogenase E1 component alpha subunit	WP_011129726.: 395091..396182	Glycolysis/Krebs cycle	COG1071
WP_011129726.1_360	CUST_1968_PI442190945	MIT9313	LLIV	WP_011129726.1_360	[illegible]	pdhA	Pyruvate dehydrogenase E1 component alpha subunit	WP_011129726.: 395091..396182	Glycolysis/Krebs cycle	COG1071
WP_011129726.1_360	CUST_1969_PI442190945	MIT9313	LLIV	WP_011129726.1_360	[illegible]	pdhA	Pyruvate dehydrogenase E1 component alpha subunit	WP_011129726.: 395091..396182	Glycolysis/Krebs cycle	COG1071
WP_011129726.1_360	CUST_1970_PI442190945	MIT9313	LLIV	WP_011129726.1_360	[illegible]	pdhA	Pyruvate dehydrogenase E1 component alpha subunit	WP_011129726.: 395091..396182	Glycolysis/Krebs cycle	COG1071

WP_011129726.1_360	CUST_1971_PI442190945	MIT9313	LLIV	WP_011129726.1_360	CATGCACCTCTTTTCGCAAGAGCATCACCTCCTAGGAGGATTTGCCTTCATC	pdhA	Pyruvate dehydrogenase E1 component alpha subunit	WP_011129726.: 395091..396182	Glycolysis/Krebs cycle	COG1071
WP_011129804.1_440	CUST_2419_PI442190945	MIT9313	LLIV	WP_011129804.1_440	TGAAGCATTTGGAACTGATTAATGACTTTGGCGTATTGATCTACGGCCTACCCTTTCTCA	glk	Glucokinase	WP_011129804.: 476670..477731	Glycolysis	COG0837
WP_011129804.1_440	CUST_2420_PI442190945	MIT9313	LLIV	WP_011129804.1_440	CATGGTGCTAATGATCATTGCGACCATTTGGATCTGCCTGCTCTTGCTAGTCAAG	glk	Glucokinase	WP_011129804.: 476670..477731	Glycolysis	COG0837
WP_011129804.1_440	CUST_2421_PI442190945	MIT9313	LLIV	WP_011129804.1_440	GATTCAATTCTTCAAGAAGCCTTACAGCTCTGGCTCGCTGCTTACGGCTCTG	glk	Glucokinase	WP_011129804.: 476670..477731	Glycolysis	COG0837
WP_011129804.1_440	CUST_2422_PI442190945	MIT9313	LLIV	WP_011129804.1_440	CTTCAAGGTCTTCGCTCAAGCACCTTTCTTGAAGCCTTCCGCAACAAGG	glk	Glucokinase	WP_011129804.: 476670..477731	Glycolysis	COG0837
WP_011129804.1_440	CUST_2423_PI442190945	MIT9313	LLIV	WP_011129804.1_440	CCGATTTGCAGCTCGAACGCCTTTCATTGGAACGTGTTGTCAGTGGAAC	glk	Glucokinase	WP_011129804.: 476670..477731	Glycolysis	COG0837
WP_011129804.1_440	CUST_2424_PI442190945	MIT9313	LLIV	WP_011129804.1_440	CTTCCGTCCGTTTCTAGAGCAATTGCCAGTGATGGCAGTGATCGATCCC	glk	Glucokinase	WP_011129804.: 476670..477731	Glycolysis	COG0837
WP_011129911.1_548	CUST_3014_PI442190945	MIT9313	LLIV	WP_011129911.1_548	GCGAAAAAATGGGGCATCGACTTTGGAGATCTGCTCAACATGATTAATGACTCGTTTATGT	prk	Phosphoribulokinase	WP_011129911.: 580255..581157	Carbon Fixation	COG3954
WP_011129911.1_548	CUST_3015_PI442190945	MIT9313	LLIV	WP_011129911.1_548	CCTGATTACATCAACCACATCTGTCCACAATTCAGCCGTACAGACATCAATTTCCAGAGA	prk	Phosphoribulokinase	WP_011129911.: 580255..581157	Carbon Fixation	COG3954
WP_011129911.1_548	CUST_3016_PI442190945	MIT9313	LLIV	WP_011129911.1_548	GCAAGATGGGTTTTGCAATGGAATTAATTTTGACCCCGATCATTCATCGCCTGATTGAAG	prk	Phosphoribulokinase	WP_011129911.: 580255..581157	Carbon Fixation	COG3954
WP_011129911.1_548	CUST_3017_PI442190945	MIT9313	LLIV	WP_011129911.1_548	AACCCATTCATTTGTAGGAACATCCCTACGCCGGATGAAAGCTTTGTGATCATTCACTTC	prk	Phosphoribulokinase	WP_011129911.: 580255..581157	Carbon Fixation	COG3954
WP_011129911.1_548	CUST_3018_PI442190945	MIT9313	LLIV	WP_011129911.1_548	GTCAGTTCACCCCTTGGGAGGAAATTCCAACCAACACGGATGTTCTCTTCTATGAAG	prk	Phosphoribulokinase	WP_011129911.: 580255..581157	Carbon Fixation	COG3954
WP_011129911.1_548	CUST_3019_PI442190945	MIT9313	LLIV	WP_011129911.1_548	GGTGGCAAGAAGCGTTATTACTTGCATAGCACTGAAGAGGCAGCAGAACATAACAG	prk	Phosphoribulokinase	WP_011129911.: 580255..581157	Carbon Fixation	COG3954
WP_011129916.1_554	CUST_3048_PI442190945	MIT9313	LLIV	WP_011129916.1_554	TATCAACTATTACTCAGCCCTTTATGAGAAAGGGCAGCTTGATCCAAAAAACGCTGTTGC	cbbA	Fructose-1,6-bisphosphate aldolase class II	WP_011129916.: 585077..586150	Glycolysis/Gluconeogenesis	COG0191
WP_011129916.1_554	CUST_3049_PI442190945	MIT9313	LLIV	WP_011129916.1_554	GGTGAAGTATTAGCGATCAGTCGAATTGCAGAAATCCATAAGGCCATCCCTAACACTCAT	cbbA	Fructose-1,6-bisphosphate aldolase class II	WP_011129916.: 585077..586150	Glycolysis/Gluconeogenesis	COG0191
WP_011129916.1_554	CUST_3050_PI442190945	MIT9313	LLIV	WP_011129916.1_554	CAACAAACCTGCGCGTAAATATATGAAGCAGGTCTGTCTTGATCGCTATCAGCAGTTTTG	cbbA	Fructose-1,6-bisphosphate aldolase class II	WP_011129916.: 585077..586150	Glycolysis/Gluconeogenesis	COG0191
WP_011129916.1_554	CUST_3051_PI442190945	MIT9313	LLIV	WP_011129916.1_554	CAAATGATCAATAAATACGGAGGTGCAATCCCTGAGACCTATGGCGTTCCTGTCGAAGAG	cbbA	Fructose-1,6-bisphosphate aldolase class II	WP_011129916.: 585077..586150	Glycolysis/Gluconeogenesis	COG0191
WP_011129916.1_554	CUST_3052_PI442190945	MIT9313	LLIV	WP_011129916.1_554	GACTTCGTCGCTAAAACCAAAGTTGACGCTTTAGCAATCGCGATTGGAACCAG	cbbA	Fructose-1,6-bisphosphate aldolase class II	WP_011129916.: 585077..586150	Glycolysis/Gluconeogenesis	COG0191
WP_011129916.1_554	CUST_3053_PI442190945	MIT9313	LLIV	WP_011129916.1_554	GAAGAGATCCAAGAAGGGATTCGCAATGGCGTGCGTAAGGTCAATATCGACAC	cbbA	Fructose-1,6-bisphosphate aldolase class II	WP_011129916.: 585077..586150	Glycolysis/Gluconeogenesis	COG0191
WP_011129944.1_585	CUST_3219_PI442190945	MIT9313	LLIV	WP_011129944.1_585	GAAGTATGTTTGCGCACCAAGGATCCTGACGATGGAAGTGACCTTGTTGAGAAAATTATG	edd	phosphogluconate dehydrogenase (NADP(+)-dependent, decarboxyla	WP_011129944.: 618105..619523	Pentose Phosphate/Entner-Dudorof	COG2084
WP_011129944.1_585	CUST_3220_PI442190945	MIT9313	LLIV	WP_011129944.1_585	CTCAAAGAATTACCGCTGAGCAGATTTTGCATGGGTCAGCGATCAAACCATTTGATCTTG	edd	phosphogluconate dehydrogenase (NADP(+)-dependent, decarboxyla	WP_011129944.: 618105..619523	Pentose Phosphate/Entner-Dudorof	COG2084
WP_011129944.1_585	CUST_3221_PI442190945	MIT9313	LLIV	WP_011129944.1_585	CTGATGTCTTGGCGCAATGGAATGCCACAGAAGAGCTCTCTTCTTATCTGGTTGAGATC	edd	phosphogluconate dehydrogenase (NADP(+)-dependent, decarboxyla	WP_011129944.: 618105..619523	Pentose Phosphate/Entner-Dudorof	COG2084
WP_011129944.1_585	CUST_3222_PI442190945	MIT9313	LLIV	WP_011129944.1_585	CTCGATTACATCAATAGCTATCGCACGGCACGATTACCACAGAATTTGGTGCAGG	edd	phosphogluconate dehydrogenase (NADP(+)-dependent, decarboxyla	WP_011129944.: 618105..619523	Pentose Phosphate/Entner-Dudorof	COG2084
WP_011129944.1_585	CUST_3223_PI442190945	MIT9313	LLIV	WP_011129944.1_585	CTTTAATGCTGACCCTCAGCTTTCCAACTTGTTGATCGATCCTTGGTTCGCTGAG	edd	phosphogluconate dehydrogenase (NADP(+)-dependent, decarboxyla	WP_011129944.: 618105..619523	Pentose Phosphate/Entner-Dudorof	COG2084
WP_011129944.1_585	CUST_3224_PI442190945	MIT9313	LLIV	WP_011129944.1_585	GTTGTCAGTGCCTTGGAGTTAGGAGCTTCAGTGCCAACCATTTACGCCTCTCTAAATG	edd	phosphogluconate dehydrogenase (NADP(+)-dependent, decarboxyla	WP_011129944.: 618105..619523	Pentose Phosphate/Entner-Dudorof	COG2084
WP_011129945.1_586	CUST_3225_PI442190945	MIT9313	LLIV	WP_011129945.1_586	TTCAAAGCTATGTGTTCGATGAGTACTGGGAAGACATCGGCACGATAGGAGCTTTTTATG	glgC	Glucose-1-phosphate adenylyltransferase	WP_011129945.: 619700..620995	Glycogen Metabolism	COG0448
WP_011129945.1_586	CUST_3226_PI442190945	MIT9313	LLIV	WP_011129945.1_586	CCACCTTTCAGTTTCTATGACGAGAAGTTCCCGATCTATACAAGACCCCGTTATCTACCG	glgC	Glucose-1-phosphate adenylyltransferase	WP_011129945.: 619700..620995	Glycogen Metabolism	COG0448
WP_011129945.1_586	CUST_3227_PI442190945	MIT9313	LLIV	WP_011129945.1_586	GGAATCAATTATTGGAGAGGGATCCATCCTCAAATCCTGCAGTATTCATCATTGCGTCCT	glgC	Glucose-1-phosphate adenylyltransferase	WP_011129945.: 619700..620995	Glycogen Metabolism	COG0448
WP_011129945.1_586	CUST_3228_PI442190945	MIT9313	LLIV	WP_011129945.1_586	GATTCCCTGGTGATGGGCTCTGATTTCTATGAATCATCCGAGGAACGCACTCTTCTAAG	glgC	Glucose-1-phosphate adenylyltransferase	WP_011129945.: 619700..620995	Glycogen Metabolism	COG0448
WP_011129945.1_586	CUST_3229_PI442190945	MIT9313	LLIV	WP_011129945.1_586	CTCTCTTCGATTTGCTGCATAAGAACCCTTCTCACAAGGATTTCGGCAAGGAGGTGATTC	glgC	Glucose-1-phosphate adenylyltransferase	WP_011129945.: 619700..620995	Glycogen Metabolism	COG0448
WP_011129945.1_586	CUST_3230_PI442190945	MIT9313	LLIV	WP_011129945.1_586	CTTCTACATCCGCAATGGGATCGTGGTAGTGGTCAAAAATGCCACCATTTCTGATG	glgC	Glucose-1-phosphate adenylyltransferase	WP_011129945.: 619700..620995	Glycogen Metabolism	COG0448
WP_011129980.1_620	CUST_3426_PI442190945	MIT9313	LLIV	WP_011129980.1_620	AGTCTTCACTGAAGACTTCATTGACAACTGGATTGACATCAAATACGAAGAGGTGCAGCA	glnA	Glutamine synthetase/ glutamate--ammonialigase	WP_011129980.: 657545..658966	Nitrogen Metabolism	COG0174
WP_011129980.1_620	CUST_3431_PI442190945	MIT9313	LLIV	WP_011129980.1_620	CATGTTCACCAGAGCCTCTTTAAAGGAGGCCAACCCCTGTTCTTTGGAGAAG	glnA	Glutamine synthetase/ glutamate--ammonialigase	WP_011129980.: 657545..658966	Nitrogen Metabolism	COG0174
WP_011129997.1_638	CUST_3515_PI442190945	MIT9313	LLIV	WP_011129997.1_638	AATTGGAGAATTTCACGATCATTCAGCCTCATCAGATTGTTGAAGCGGCTAAGCAGATTG	pdhB	Pyruvate dehydrogenase E1 component beta subunit	WP_011129997.: 672575..673558	Glycolysis/Krebs cycle	COG0022
WP_011129997.1_638	CUST_3516_PI442190945	MIT9313	LLIV	WP_011129997.1_638	ATGTGGAATTGATCGATTTGATTAGTCTCAAGCCCTTCGATATGGAGACCATTGTTCGCT	pdhB	Pyruvate dehydrogenase E1 component beta subunit	WP_011129997.: 672575..673558	Glycolysis/Krebs cycle	COG0022
WP_011129997.1_638	CUST_3517_PI442190945	MIT9313	LLIV	WP_011129997.1_638	GTTGAAGAGTGTATGAAAACTGGTGGGATTGGTGCTGAGTTGATTGCGCTGATTACTGAG	pdhB	Pyruvate dehydrogenase E1 component beta subunit	WP_011129997.: 672575..673558	Glycolysis/Krebs cycle	COG0022
WP_011129997.1_638	CUST_3518_PI442190945	MIT9313	LLIV	WP_011129997.1_638	GAGACAACAATCCAGTTCTCTTTTTCGAGCATGTGCTGCTCTACAACCTGATTGAGGAG	pdhB	Pyruvate dehydrogenase E1 component beta subunit	WP_011129997.: 672575..673558	Glycolysis/Krebs cycle	COG0022
WP_011129997.1_638	CUST_3519_PI442190945	MIT9313	LLIV	WP_011129997.1_638	CAGATCTCCAACAACATGGGAATGCTTCGTTACACCAGTGGCGGAAATTTCACAATTC	pdhB	Pyruvate dehydrogenase E1 component beta subunit	WP_011129997.: 672575..673558	Glycolysis/Krebs cycle	COG0022
WP_011129997.1_638	CUST_3520_PI442190945	MIT9313	LLIV	WP_011129997.1_638	GATTATGTCTGTGCCCTAGATCAAGCAGATCTGGTTCGTGAGGGTAAAGACGTCACGATC	pdhB	Pyruvate dehydrogenase E1 component beta subunit	WP_011129997.: 672575..673558	Glycolysis/Krebs cycle	COG0022
WP_011130047.1_689	CUST_3797_PI442190945	MIT9313	LLIV	WP_011130047.1_689	ACGTCTTGCTGGTGATCCGATGTTTTTTAAGGTGCTGATCAATACTTTGATCTACCTGGT		sugar ABC transporter substrate-binding protein	WP_011130047.: 724886..725794	Other sugar transporters	
WP_011130047.1_689	CUST_3798_PI442190945	MIT9313	LLIV	WP_011130047.1_689	GGCTTGGTTATTACATGGTGATATTTCTTGCTGGCCTCCAAGGAATTCCTAAAGAGCTTT		sugar ABC transporter substrate-binding protein	WP_011130047.: 724886..725794	Other sugar transporters	
WP_011130047.1_689	CUST_3799_PI442190945	MIT9313	LLIV	WP_011130047.1_689	GGTGTTGTTGAACCGTTCCTTACCAGGTATTCATTTTTTACGCGGTGCGTTCTATACAC		sugar ABC transporter substrate-binding protein	WP_011130047.: 724886..725794	Other sugar transporters	
WP_011130047.1_689	CUST_3800_PI442190945	MIT9313	LLIV	WP_011130047.1_689	GTGGTTTCATCGATTGCGGCTACAAAAGTGTTCGAAGAAGTGTTCTTGATGACTCAGG		sugar ABC transporter substrate-binding protein	WP_011130047.: 724886..725794	Other sugar transporters	
WP_011130047.1_689	CUST_3801_PI442190945	MIT9313	LLIV	WP_011130047.1_689	CTCGAACCCTTGTCTACTACGTTTACGACCAGGCTTTTGCTGAGCTTGAGATCAGTTATG		sugar ABC transporter substrate-binding protein	WP_011130047.: 724886..725794	Other sugar transporters	
WP_011130047.1_689	CUST_3802_PI442190945	MIT9313	LLIV	WP_011130047.1_689	CTCTTTTGATGAGCTTCACCAAAACAGGTCTCGACGTTACAGAGCCCCTTGTCTTTG		sugar ABC transporter substrate-binding protein	WP_011130047.: 724886..725794	Other sugar transporters	
WP_041384965.1_699	CUST_3846_PI442190945	MIT9313	LLIV	WP_041384965.1_699	CAAGAAGGAGAGGAATACCACGCAGAAATGATCTCAAAACGACTAGGGATTCCGTTCATC	pykF	Pyruvate kinase	WP_041384965.: 735528..737312	Glycolysis	COG0469
WP_041384965.1_699	CUST_3847_PI442190945	MIT9313	LLIV	WP_041384965.1_699	GATGCAATTGCTGTTGCCCAGGAATTGGGTCTTCTCAAAGAAGGAGATCTAACAATTCAA	pykF	Pyruvate kinase	WP_041384965.: 735528..737312	Glycolysis	COG0469
WP_041384965.1_699	CUST_3848_PI442190945	MIT9313	LLIV	WP_041384965.1_699	AAAAATTGAACCAGGCGAGATTCTGGTCATTCATGAAACAAGCGATGACTATCTCGATGC	pykF	Pyruvate kinase	WP_041384965.: 735528..737312	Glycolysis	COG0469
WP_041384965.1_699	CUST_3849_PI442190945	MIT9313	LLIV	WP_041384965.1_699	CACAGATCTTGTCAAAGTAGGAATTGTCAGTGCAGTACTTGGCCAAGGGATCGGTATTG	pykF	Pyruvate kinase	WP_041384965.: 735528..737312	Glycolysis	COG0469
WP_041384965.1_699	CUST_3850_PI442190945	MIT9313	LLIV	WP_041384965.1_699	GAGAAACCTGCTTGAAGGAGAGGTGGTAACAATGCAGCTCAGTAATGGCAAAGTTCATC	pykF	Pyruvate kinase	WP_041384965.: 735528..737312	Glycolysis	COG0469
WP_041384965.1_699	CUST_3851_PI442190945	MIT9313	LLIV	WP_041384965.1_699	CAATTTTGGCCGTAACCAACAAATTGGCTGTAGCAAGAAAACTTCAATTGGCATGGG	pykF	Pyruvate kinase	WP_041384965.: 735528..737312	Glycolysis	COG0469
WP_011130071.1_713	CUST_3922_PI442190945	MIT9313	LLIV	WP_011130071.1_713	TACGGACCTATTGAGCAGTTCACAGAACTGATTGGACTACAACCTCTAAACATCCTCTCG		sugar ABC transporter substrate-binding protein	WP_011130071.: 748007..748873	Other sugar transporters	
WP_011130071.1_713	CUST_3923_PI442190945	MIT9313	LLIV	WP_011130071.1_713	TTTGGCTATAGCTCCACTGTAATGATCAGCGGGTTTCTTCTACTTGTTATTGCCTGTCTG		sugar ABC transporter substrate-binding protein	WP_011130071.: 748007..748873	Other sugar transporters	
WP_011130071.1_713	CUST_3924_PI442190945	MIT9313	LLIV	WP_011130071.1_713	AGTCAGCCTAGAACTATTACTTGCTATAGCTATAGCCCTCTTACTCGATCAGCATTGGCG		sugar ABC transporter substrate-binding protein	WP_011130071.: 748007..748873	Other sugar transporters	
WP_011130071.1_713	CUST_3925_PI442190945	MIT9313	LLIV	WP_011130071.1_713	GGCAGGAAGCACGGAAAGCCTTGCTTTATATGCTTATTTAAACGCAATGAGATTCCTAGA		sugar ABC transporter substrate-binding protein	WP_011130071.: 748007..748873	Other sugar transporters	
WP_011130071.1_713	CUST_3926_PI442190945	MIT9313	LLIV	WP_011130071.1_713	TCTTTTGGCTGGCTTGCAAACTATTCCCAATGATCTCTACGAAGCTTTCTATCTCGAAGG		sugar ABC transporter substrate-binding protein	WP_011130071.: 748007..748873	Other sugar transporters	
WP_011130071.1_713	CUST_3927_PI442190945	MIT9313	LLIV	WP_011130071.1_713	AGCCGCACTATTTAAGATCACCTTGCCGCTCTTGCAACCTTATTTACTCCTTAGTCTTTT		sugar ABC transporter substrate-binding protein	WP_011130071.: 748007..748873	Other sugar transporters	
WP_011130072.1_714	CUST_3928_PI442190945	MIT9313	LLIV	WP_011130072.1_714	AAGCCCTAGAAAGCACTGATGAACGTCCATCAACTCCTCTTTATGCACAGATTAGTGATG		sugar ABC transporter substrate-binding protein	WP_011130072.: 748870..750138	Other sugar transporters	
WP_011130072.1_714	CUST_3929_PI442190945	MIT9313	LLIV	WP_011130072.1_714	CCGTCAATATGAAGGCCTAAGTTGTGTATTTCTAGAAGTTCTTGACGGATTTGGAGGGCA		sugar ABC transporter substrate-binding protein	WP_011130072.: 748870..750138	Other sugar transporters	
WP_011130072.1_714	CUST_3930_PI442190945	MIT9313	LLIV	WP_011130072.1_714	CCAAAGTTGAACTATTCAAAGACCAAGAGCTGCTCTCAAAGTCACAAATTCTTCCGGAGC		sugar ABC transporter substrate-binding protein	WP_011130072.: 748870..750138	Other sugar transporters	
WP_011130072.1_714	CUST_3931_PI442190945	MIT9313	LLIV	WP_011130072.1_714	CAGTGATCAACTATGCAGAAAATGAAGCTCTACAAGCGTTTAAGTCTGGAGATGTTGCGC		sugar ABC transporter substrate-binding protein	WP_011130072.: 748870..750138	Other sugar transporters	
WP_011130072.1_714	CUST_3932_PI442190945	MIT9313	LLIV	WP_011130072.1_714	TTACAACGAAATCTTAGTTCGATTTTAACAGGCCAATCCAGTGTTAGCGATGCAATGGCC		sugar ABC transporter substrate-binding protein	WP_011130072.: 748870..750138	Other sugar transporters	
WP_011130072.1_714	CUST_3933_PI442190945	MIT9313	LLIV	WP_011130072.1_714	GCCTTCCTCACTTCAACATCTGCACAAAAAACACTGTTCTTGAACGAAAGTTATACGCCA		sugar ABC transporter substrate-binding protein	WP_011130072.: 748870..750138	Other sugar transporters	
WP_041384970.1_734	CUST_4043_PI442190945	MIT9313	LLIV	WP_041384970.1_734	CAGAAGAAGCCATCTTTTGGATCATGAGGCATCTAACTGCGAATAAGCGTGGTTATGTTG	pgi	Glucose-6-phosphate isomerase	WP_041384970.: 770925..772523	Glycolysis/Gluconeogenesis	COG0166
WP_041384970.1_734	CUST_4044_PI442190945	MIT9313	LLIV	WP_041384970.1_734	CATGCTTATGTGCAGCAATTACGCGATGGTGTTGACAATTTCTTCGCCACATTTATTGAG	pgi	Glucose-6-phosphate isomerase	WP_041384970.: 770925..772523	Glycolysis/Gluconeogenesis	COG0166
WP_041384970.1_734	CUST_4045_PI442190945	MIT9313	LLIV	WP_041384970.1_734	GTGACATGGTTGTTCTTCCATATAGAGATCGCCTTGAGGTCTTTAGTCGTTACCTGCAAC	pgi	Glucose-6-phosphate isomerase	WP_041384970.: 770925..772523	Glycolysis/Gluconeogenesis	COG0166
WP_041384970.1_734	CUST_4046_PI442190945	MIT9313	LLIV	WP_041384970.1_734	CATCCTTAACTTGCAGTCTAGAGTTGAAGACTTGCTTGCTGATGGTGTCGAACGTTCAG	pgi	Glucose-6-phosphate isomerase	WP_041384970.: 770925..772523	Glycolysis/Gluconeogenesis	COG0166
WP_041384970.1_734	CUST_4047_PI442190945	MIT9313	LLIV	WP_041384970.1_734	CTTGAGGATGTTGAGAACATACCGGCGATCAACAATGAGCATCCAGGTGATTTTCTC	pgi	Glucose-6-phosphate isomerase	WP_041384970.: 770925..772523	Glycolysis/Gluconeogenesis	COG0166
WP_041384970.1_734	CUST_4048_PI442190945	MIT9313	LLIV	WP_041384970.1_734	GATGGCAATGTTGTTCATCAAGGCCTTGCTGTATATGGCAACAAGGGTTCAACTGATCAG	pgi	Glucose-6-phosphate isomerase	WP_041384970.: 770925..772523	Glycolysis/Gluconeogenesis	COG0166
WP_041384389.1_950	CUST_5169_PI442190945	MIT9313	LLIV	WP_041384389.1_950	ATTCATAACAGCTCTAGGTCGAGATTCATGCGGTGAAAAGAGTTTTCAAAGACTCCAGCA	rbsK	Sugar kinase, ribokinase family	WP_041384389.: 993667..994518	Pentose Phosphate	COG0524
WP_041384389.1_950	CUST_5170_PI442190945	MIT9313	LLIV	WP_041384389.1_950	CTACAGATCAATTACCCCAGCCTGGGGTAATCAGCCATGCAGATCATTCTTTTGAAGAG	rbsK	Sugar kinase, ribokinase family	WP_041384389.: 993667..994518	Pentose Phosphate	COG0524
WP_041384389.1_950	CUST_5171_PI442190945	MIT9313	LLIV	WP_041384389.1_950	TAGAGACACTTGAGCAGGCGAATATTCAGCTTGATGCGCTGATTGGTAGTGGTCTTGATC	rbsK	Sugar kinase, ribokinase family	WP_041384389.: 993667..994518	Pentose Phosphate	COG0524
WP_041384389.1_950	CUST_5172_PI442190945	MIT9313	LLIV	WP_041384389.1_950	CTTGTTGATTCTCATGGGGAACGGGCAATTACAGTGATTGGCGAGCGTTTACAAC	rbsK	Sugar kinase, ribokinase family	WP_041384389.: 993667..994518	Pentose Phosphate	COG0524
WP_041384389.1_950	CUST_5173_PI442190945	MIT9313	LLIV	WP_041384389.1_950	CAGATAAAGCGCTCTTTCCAACTCCCCGGCTGAGAATTGCAACTGAAGGAGCTTTAG	rbsK	Sugar kinase, ribokinase family	WP_041384389.: 993667..994518	Pentose Phosphate	COG0524
WP_041384389.1_950	CUST_5174_PI442190945	MIT9313	LLIV	WP_041384389.1_950	CTATCAGGCTGTTTCACTGAAGTCTCCAGTCGTGGATGCTTATGGCTGTGGCGATAG	rbsK	Sugar kinase, ribokinase family	WP_041384389.: 993667..994518	Pentose Phosphate	COG0524
WP_011130474.1_1181	CUST_6208_PI442190945	MIT9313	LLIV	WP_011130474.1_1181	GAACTGATTGAAGACAGCCCATGGCAATTGCCCATTCATCCCTATGAATCACGTAC	zwf	glucose-6-phosphate dehydrogenase zwf	WP_011130474.: 1193456..1194979	Pentose Phosphate	COG0364
WP_011130474.1_1181	CUST_6209_PI442190945	MIT9313	LLIV	WP_011130474.1_1181	GATACGTCCGACTTTTAGCCGATGCCATGCTCAGTGACCCAACTTTATTCACC	zwf	glucose-6-phosphate dehydrogenase zwf	WP_011130474.: 1193456..1194979	Pentose Phosphate	COG0364
WP_011130474.1_1181	CUST_6210_PI442190945	MIT9313	LLIV	WP_011130474.1_1181	CAGTAGAGATGGAATTCTCCTATGACGAATCCTTCGGTGAGCCATCCGATGAGGGATAC	zwf	glucose-6-phosphate dehydrogenase zwf	WP_011130474.: 1193456..1194979	Pentose Phosphate	COG0364
WP_011130474.1_1181	CUST_6211_PI442190945	MIT9313	LLIV	WP_011130474.1_1181	GTCTTAGTGAAGTGGTTCTCACCTTTCGAGAAGCACCCGTGCATCTTTTCGATG	zwf	glucose-6-phosphate dehydrogenase zwf	WP_011130474.: 1193456..1194979	Pentose Phosphate	COG0364
WP_011130474.1_1181	CUST_6212_PI442190945	MIT9313	LLIV	WP_011130474.1_1181	GATCCCAGCAGCACCACCGAGACCTATGTAGCGATGAAACTTTTCATCGACAACTG	zwf	glucose-6-phosphate dehydrogenase zwf	WP_011130474.: 1193456..1194979	Pentose Phosphate	COG0364
WP_011130474.1_1181	CUST_6213_PI442190945	MIT9313	LLIV	WP_011130474.1_1181	AAATCACAGCCGCTGAAACCGTAGGGGTTGAAGAACGAGCCAACTACTACGAATC	zwf	glucose-6-phosphate dehydrogenase zwf	WP_011130474.: 1193456..1194979	Pentose Phosphate	COG0364
WP_011130475.1_1182	CUST_6214_PI442190945	MIT9313	LLIV	WP_011130475.1_1182	CAATGCCCTTAGTCATGTTGATCAGCTCGAAATCGACGTTGAGGGTCATCACC	opcA	Glucose-6-P dehydrogenase subunit opcA	WP_011130475.: 1195021..1196310	Pentose Phosphate	COG3429
WP_011130475.1_1182	CUST_6215_PI442190945	MIT9313	LLIV	WP_011130475.1_1182	CTGATTGAAGAAGTCGTCCCCATGCAGGTCGCCTCTGTAGAGATGGATGTAG	opcA	Glucose-6-P dehydrogenase subunit opcA	WP_011130475.: 1195021..1196310	Pentose Phosphate	COG3429
WP_011130475.1_1182	CUST_6216_PI442190945	MIT9313	LLIV	WP_011130475.1_1182	CAATTACAAGCCACCCAGACAATAAAAGGGGAAGGCATTGCTGCCGAATTCC	opcA	Glucose-6-P dehydrogenase subunit opcA	WP_011130475.: 1195021..1196310	Pentose Phosphate	COG3429
WP_011130475.1_1182	CUST_6217_PI442190945	MIT9313	LLIV	WP_011130475.1_1182	GAATGATCTGAATTGGTTAAGGCTGCGCACTTGGCGAGAACACCTCGCAATGGTGTTTG	opcA	Glucose-6-P dehydrogenase subunit opcA	WP_011130475.: 1195021..1196310	Pentose Phosphate	COG3429
WP_011130475.1_1182	CUST_6218_PI442190945	MIT9313	LLIV	WP_011130475.1_1182	GATCGATACAGCACTAGGAGATCCCCATCGCTGCATTGAGCTGCTCAATG	opcA	Glucose-6-P dehydrogenase subunit opcA	WP_011130475.: 1195021..1196310	Pentose Phosphate	COG3429
WP_011130475.1_1182	CUST_6219_PI442190945	MIT9313	LLIV	WP_011130475.1_1182	CTAACAGATGGGCTGGGAATCCTTCAACCTCTACTACCTGCAGAACTGCCCTC	opcA	Glucose-6-P dehydrogenase subunit opcA	WP_011130475.: 1195021..1196310	Pentose Phosphate	COG3429
WP_011130575.1_1287	CUST_6778_PI442190945	MIT9313	LLIV	WP_011130575.1_1287	CATGGTTGGTTATGACGCATACACCCAAAGTCAAGGAACCGCATTCGTGGTATTTGAAG	rbcS	Ribulose bisphosphate carboxylase small subunit	WP_011130575.: 1292937..1293278	Carbon fixation	COG4451
WP_011130575.1_1287	CUST_6779_PI442190945	MIT9313	LLIV	WP_011130575.1_1287	AAGTTACCTTTCTTTGGCGAGAAGGATCTGAACATGGTTGTAAGTGAGCTAGAAGCTTGC	rbcS	Ribulose bisphosphate carboxylase small subunit	WP_011130575.: 1292937..1293278	Carbon fixation	COG4451
WP_011130575.1_1287	CUST_6780_PI442190945	MIT9313	LLIV	WP_011130575.1_1287	GACGAAATTTACGATCAAATTGCTTACATCATTGCTCAGGGATGGAGCCCTGTCATTGAG	rbcS	Ribulose bisphosphate carboxylase small subunit	WP_011130575.: 1292937..1293278	Carbon fixation	COG4451
WP_011130575.1_1287	CUST_6781_PI442190945	MIT9313	LLIV	WP_011130575.1_1287	CAGTTGGTGACTATCAAACAGTCGCCACCCTGGAAACCTTCGGCTTCTTAC	rbcS	Ribulose bisphosphate carboxylase small subunit	WP_011130575.: 1292937..1293278	Carbon fixation	COG4451
WP_011130576.1_1288	CUST_6782_PI442190945	MIT9313	LLIV	WP_011130576.1_1288	CAGGAGAAGTCAAAGGTCATTATCTCAATTGCACTGCGACAACTCCTGAGGAGATGTATG	rbcL	Ribulose 1,5-bisphosphate carboxylase large subunit	WP_011130576.: 1293386..1294798	Carbon fixation	COG1850
WP_011130576.1_1288	CUST_6783_PI442190945	MIT9313	LLIV	WP_011130576.1_1288	CTTGAGACTTGGAAAGAAATCAAGTTCGAGTTCGATACTGTCGACAAGCTCGACGTTCAG	rbcL	Ribulose 1,5-bisphosphate carboxylase large subunit	WP_011130576.: 1293386..1294798	Carbon fixation	COG1850
WP_011130576.1_1288	CUST_6784_PI442190945	MIT9313	LLIV	WP_011130576.1_1288	TAAAGAACTCGACATGCCAATCATCATGCATGACTACATCACTGGTGGATTTACCGCTAA	rbcL	Ribulose 1,5-bisphosphate carboxylase large subunit	WP_011130576.: 1293386..1294798	Carbon fixation	COG1850
WP_011130576.1_1288	CUST_6785_PI442190945	MIT9313	LLIV	WP_011130576.1_1288	ATCGTCAGACCACTCTTGGTTATATCGACAACCTGCGTGAGTCTTTTGTTCCAGAAGATC	rbcL	Ribulose 1,5-bisphosphate carboxylase large subunit	WP_011130576.: 1293386..1294798	Carbon fixation	COG1850
WP_011130576.1_1288	CUST_6786_PI442190945	MIT9313	LLIV	WP_011130576.1_1288	CAATTTTTGGAGACGACTCCTGTCTACAGTTTGGTGGTGGTACTCATGGTCATCCATG	rbcL	Ribulose 1,5-bisphosphate carboxylase large subunit	WP_011130576.: 1293386..1294798	Carbon fixation	COG1850
WP_011130576.1_1288	CUST_6787_PI442190945	MIT9313	LLIV	WP_011130576.1_1288	CTAACACAGGTTTGGCGAATTGGTGCCGTAAAAACGGCATGCTGTTGCATATC	rbcL	Ribulose 1,5-bisphosphate carboxylase large subunit	WP_011130576.: 1293386..1294798	Carbon fixation	COG1850
WP_011130617.1_1332	CUST_7029_PI442190945	MIT9313	LLIV	WP_011130617.1_1332	TCTCGATGGTGATGGTTGTATTACTAGAGAAGAATGGCTCGGTAGTGATGCGGTATTTGA	tal	Transaldolase	WP_011130617.: 1335673..1336845	Pentose Phosphate	COG0176
WP_011130617.1_1332	CUST_7030_PI442190945	MIT9313	LLIV	WP_011130617.1_1332	ATGCAGGTCCAGAAGACCCTGGTGTGATCTCGGTGACCAAGATCTTTAATTATTTCAAAA	tal	Transaldolase	WP_011130617.: 1335673..1336845	Pentose Phosphate	COG0176
WP_011130617.1_1332	CUST_7031_PI442190945	MIT9313	LLIV	WP_011130617.1_1332	AGAAGGATGGAATTCATTGCAATTTAACTCTGCTATTTGGCTTTTCTCAGGCTGTCGCTT	tal	Transaldolase	WP_011130617.: 1335673..1336845	Pentose Phosphate	COG0176
WP_011130617.1_1332	CUST_7032_PI442190945	MIT9313	LLIV	WP_011130617.1_1332	GCAATCTTGACGAAATCATTGAATTGGCTGGATGTGATTTGTTGACCATCTCACCAAAGC	tal	Transaldolase	WP_011130617.: 1335673..1336845	Pentose Phosphate	COG0176
WP_011130617.1_1332	CUST_7033_PI442190945	MIT9313	LLIV	WP_011130617.1_1332	AATCCTGTTGCTGCAGAGTCTCAGATCCATGTCGATAAGGAGAGTTTTGAGTCAATGATG	tal	Transaldolase	WP_011130617.: 1335673..1336845	Pentose Phosphate	COG0176
WP_011130617.1_1332	CUST_7034_PI442190945	MIT9313	LLIV	WP_011130617.1_1332	GAGTCACTTTGATCTCTCCTTTTGTTGGGCGAATCCTTGATTGGTACAAAGCGTCTACTG	tal	Transaldolase	WP_011130617.: 1335673..1336845	Pentose Phosphate	COG0176
WP_011130701.1_1419	CUST_7515_PI442190945	MIT9313	LLIV	WP_011130701.1_1419	GTTTCATCACAACAGACATGACAAAAGAGTTGGATTCAGAACCGATTCTTGCCGCTATCC	gdh	3-oxoacyl-acyl-carrier-protein reductase	WP_011130701.: 1418549..1419301	Entner-Dudoroff	COG1028
WP_011130701.1_1419	CUST_7516_PI442190945	MIT9313	LLIV	WP_011130701.1_1419	CAACTGATCAAAACCGTGCTTGAACGCAGCAGCAGACTGGATATTTTGATCAACAATG	gdh	3-oxoacyl-acyl-carrier-protein reductase	WP_011130701.: 1418549..1419301	Entner-Dudoroff	COG1028
WP_011130701.1_1419	CUST_7517_PI442190945	MIT9313	LLIV	WP_011130701.1_1419	GAAGTAGTCGTGAACTACTCAAGCTCAGCTGATGCAGCAGAAGAAGTTGTTCAAGCGATC	gdh	3-oxoacyl-acyl-carrier-protein reductase	WP_011130701.: 1418549..1419301	Entner-Dudoroff	COG1028
WP_011130701.1_1419	CUST_7518_PI442190945	MIT9313	LLIV	WP_011130701.1_1419	CTGTTGCTCGCACGATGCTTAAGCAAAAAAGCGGCAGAATTATCAATATCACCTCC	gdh	3-oxoacyl-acyl-carrier-protein reductase	WP_011130701.: 1418549..1419301	Entner-Dudoroff	COG1028
WP_011130701.1_1419	CUST_7519_PI442190945	MIT9313	LLIV	WP_011130701.1_1419	GAAGACTGGCAAGCCGTGGTCAATCTCAACCTCACAGGTGTATTTCTATGCACTC	gdh	3-oxoacyl-acyl-carrier-protein reductase	WP_011130701.: 1418549..1419301	Entner-Dudoroff	COG1028
WP_011130701.1_1419	CUST_7520_PI442190945	MIT9313	LLIV	WP_011130701.1_1419	GGCTTACATCACTGGACAAGTGCTACAAGTTGATGGCGGCATGCTGATG	gdh	3-oxoacyl-acyl-carrier-protein reductase	WP_011130701.: 1418549..1419301	Entner-Dudoroff	COG1028
WP_011130709.1_1427	CUST_7559_PI442190945	MIT9313	LLIV	WP_011130709.1_1427	GTTCCATTAGCCCATGACTCCCATGACCATCAGATGATGGACATGAAGGAACAAATCTG	ctaC/coxB	Cytochrome c oxidase subunit II	WP_011130709.: 1426678..1427502	Respiration	COG1622
WP_011130709.1_1427	CUST_7560_PI442190945	MIT9313	LLIV	WP_011130709.1_1427	TATACGACGAACTCTTCAGAGTCCTTTTCAGCATCGGAGCAATCCTTTTTCTCGGCATTG	ctaC/coxB	Cytochrome c oxidase subunit II	WP_011130709.: 1426678..1427502	Respiration	COG1622
WP_011130709.1_1427	CUST_7561_PI442190945	MIT9313	LLIV	WP_011130709.1_1427	GAATTCCGCATCAAGCAAGACATCATTCCTGGTCAGCCCACTCTCTTGAACTTC	ctaC/coxB	Cytochrome c oxidase subunit II	WP_011130709.: 1426678..1427502	Respiration	COG1622
WP_011130709.1_1427	CUST_7562_PI442190945	MIT9313	LLIV	WP_011130709.1_1427	GTTGGTTGTATTCAGCCTGATTAAGTTCCGCAGACGTCCTGGTCAATTGGAGGAC	ctaC/coxB	Cytochrome c oxidase subunit II	WP_011130709.: 1426678..1427502	Respiration	COG1622
WP_011130709.1_1427	CUST_7563_PI442190945	MIT9313	LLIV	WP_011130709.1_1427	GAACTCTGCGGTCCATACCACGGGAACATGCATACGAAAGTGATTGTCGAAGAG	ctaC/coxB	Cytochrome c oxidase subunit II	WP_011130709.: 1426678..1427502	Respiration	COG1622
WP_011130709.1_1427	CUST_7564_PI442190945	MIT9313	LLIV	WP_011130709.1_1427	GTGACTACGACACCTGGTTCAGCAACAACGCCAAAACAACGGTTTCAGAAG	ctaC/coxB	Cytochrome c oxidase subunit II	WP_011130709.: 1426678..1427502	Respiration	COG1622
WP_011130710.1_1428	CUST_7565_PI442190945	MIT9313	LLIV	WP_011130710.1_1428	TTCATTTTTTTATCACTTTCATCGGCTTCAATCTTTGCTTTGCCCCACAGCATTGGCTAG	cyoB/coxA	Cytochrome c oxidase subunit I	WP_011130710.: 1427499..1429175	Respiration	COG0843
WP_011130710.1_1428	CUST_7566_PI442190945	MIT9313	LLIV	WP_011130710.1_1428	TACCTACTTTGTTGTTGCCCATTTTCACTACATCGTCTATGGAGGTTCGGTGTTTGTGAT	cyoB/coxA	Cytochrome c oxidase subunit I	WP_011130710.: 1427499..1429175	Respiration	COG0843
WP_011130710.1_1428	CUST_7567_PI442190945	MIT9313	LLIV	WP_011130710.1_1428	AGGAACTCAACCTAGAAAAAACCAGCAACAGTGATAGTGATCTAGGGAGCAAAAGCCAAT	cyoB/coxA	Cytochrome c oxidase subunit I	WP_011130710.: 1427499..1429175	Respiration	COG0843
WP_011130710.1_1428	CUST_7568_PI442190945	MIT9313	LLIV	WP_011130710.1_1428	TTTTCACCATTGCCACATCCTTCATTGCCGTACCTACAGGAATTAAGTTCTTCAATTGGC	cyoB/coxA	Cytochrome c oxidase subunit I	WP_011130710.: 1427499..1429175	Respiration	COG0843
WP_011130710.1_1428	CUST_7569_PI442190945	MIT9313	LLIV	WP_011130710.1_1428	CTTTGCCTCTATTTATCACTGGTATCCAAAATTCACTGGGCGCATGCTCAATGAATCTCT	cyoB/coxA	Cytochrome c oxidase subunit I	WP_011130710.: 1427499..1429175	Respiration	COG0843
WP_011130710.1_1428	CUST_7570_PI442190945	MIT9313	LLIV	WP_011130710.1_1428	CATCTCTCTAAACAGTGCTGTGCTTTTCTCTTGTGGCTTCATTGTCAACTTTGTGCTTGG	cyoB/coxA	Cytochrome c oxidase subunit I	WP_011130710.: 1427499..1429175	Respiration	COG0843
WP_011130711.1_1429	CUST_7571_PI442190945	MIT9313	LLIV	WP_011130711.1_1429	TCTATTGGCACTTCGTAGACGGCATCTGGGTGATCTTATTCATCATCTTCTACCTTCTCT	cyoC	heme-copper oxidase subunit III	WP_011130711.: 1429172..1429795	Respiration	COG1845
WP_011130711.1_1429	CUST_7572_PI442190945	MIT9313	LLIV	WP_011130711.1_1429	CTTCCAACGATCGACCATGACTATGAGGAGCAGGCTGATCTAGCAGATGATCATG	cyoC	heme-copper oxidase subunit III	WP_011130711.: 1429172..1429795	Respiration	COG1845
WP_011130711.1_1429	CUST_7573_PI442190945	MIT9313	LLIV	WP_011130711.1_1429	CTATCTCACATTTCGGGCTGTCAATCCCCTGCTGCCTGATGCGGTTTATGAATTAGAG	cyoC	heme-copper oxidase subunit III	WP_011130711.: 1429172..1429795	Respiration	COG1845
WP_011130711.1_1429	CUST_7574_PI442190945	MIT9313	LLIV	WP_011130711.1_1429	GTTACAGGCTTTCACGGGCTACACGTCACCCTAGGGACGATCATGATATTGATC	cyoC	heme-copper oxidase subunit III	WP_011130711.: 1429172..1429795	Respiration	COG1845
WP_011130711.1_1429	CUST_7575_PI442190945	MIT9313	LLIV	WP_011130711.1_1429	CCAAATGGTCGAGTACTTCACACTGCCATTTGGCCTCACAGACAACCTTTACG	cyoC	heme-copper oxidase subunit III	WP_011130711.: 1429172..1429795	Respiration	COG1845
WP_011130711.1_1429	CUST_7576_PI442190945	MIT9313	LLIV	WP_011130711.1_1429	GACAAGCTCTGAAGCGCAATCAATCAGACCAGTGTCAACGTTGGCTGTTCATCACTG	cyoC	heme-copper oxidase subunit III	WP_011130711.: 1429172..1429795	Respiration	COG1845
WP_052646185.1_1490	CUST_7893_PI442190945	MIT9313	LLIV	WP_052646185.1_1490	GATTTGGAGCCGCAAATGCTTATCTGATCCCTTGGTGTCTGCTACCAGATGCAATTGATG	glcH	GPH family sugar transporter	WP_052646185.: 1484637..1486079	GlcH sugar transporter	COG2211
WP_052646185.1_1490	CUST_7894_PI442190945	MIT9313	LLIV	WP_052646185.1_1490	GTTCAGAAAATCGGTATTGGTGTGAGCATCCAACTGCTCGGCATCCTGCTTTCACTGTC	glcH	GPH family sugar transporter	WP_052646185.: 1484637..1486079	GlcH sugar transporter	COG2211
WP_052646185.1_1490	CUST_7895_PI442190945	MIT9313	LLIV	WP_052646185.1_1490	GAGGATCATCAGCAACAGACGCTTCCTCAAAGTTCTGGGGCTTTACCTGTTG	glcH	GPH family sugar transporter	WP_052646185.: 1484637..1486079	GlcH sugar transporter	COG2211
WP_052646185.1_1490	CUST_7896_PI442190945	MIT9313	LLIV	WP_052646185.1_1490	CTTTGCAGTTGATGCAGCCAGTTGCTCTGATCTACCTCGTTCAGTTGATGCG	glcH	GPH family sugar transporter	WP_052646185.: 1484637..1486079	GlcH sugar transporter	COG2211
WP_052646185.1_1490	CUST_7897_PI442190945	MIT9313	LLIV	WP_052646185.1_1490	CTACTCGGATCAACCCGAATCCGCTCTGATCACCATTCGCATGTGCATGGGATTCATTC	glcH	GPH family sugar transporter	WP_052646185.: 1484637..1486079	GlcH sugar transporter	COG2211
WP_052646185.1_1490	CUST_7898_PI442190945	MIT9313	LLIV	WP_052646185.1_1490	CTTTGGCCTACAGCCGGCACTGATGGTCTGAGATTGATGGCTTTGTTG	glcH	GPH family sugar transporter	WP_052646185.: 1484637..1486079	GlcH sugar transporter	COG2211
WP_011130786.1_1512	CUST_8016_PI442190945	MIT9313	LLIV	WP_011130786.1_1512	GGACTGGTAAAACCCTTTTGGTGTCGAAGTTTATTGAGGATGCTTTCCGAAATCAGGAAC	kaiC	circadian clock protein KaiC	WP_011130786.: 1506391..1507890	Circadian Rhythm	COG0467
WP_011130786.1_1512	CUST_8017_PI442190945	MIT9313	LLIV	WP_011130786.1_1512	TCATTCGATTACCGACTCCCATATCTCCACAATCACAGACACTATTCTGCTGCTTCAGTA	kaiC	circadian clock protein KaiC	WP_011130786.: 1506391..1507890	Circadian Rhythm	COG0467
WP_011130786.1_1512	CUST_8018_PI442190945	MIT9313	LLIV	WP_011130786.1_1512	AGATTATTAAAACGGCGATTGGACAATTCAAGCCATCGAGGATGGCGATTGATTCACTTT	kaiC	circadian clock protein KaiC	WP_011130786.: 1506391..1507890	Circadian Rhythm	COG0467
WP_011130786.1_1512	CUST_8019_PI442190945	MIT9313	LLIV	WP_011130786.1_1512	CTGGTGTCCCTAATCTTGATGAGATGTGTGGTGGAGGTTTCTTCAAGGATTCGATCATTC	kaiC	circadian clock protein KaiC	WP_011130786.: 1506391..1507890	Circadian Rhythm	COG0467
WP_011130786.1_1512	CUST_8020_PI442190945	MIT9313	LLIV	WP_011130786.1_1512	AAAGACTCCTTCTCTAACTTCGAACGGATCTTCAGTGGTGCTCCTCATCGTATTACTGAC	kaiC	circadian clock protein KaiC	WP_011130786.: 1506391..1507890	Circadian Rhythm	COG0467
WP_011130786.1_1512	CUST_8021_PI442190945	MIT9313	LLIV	WP_011130786.1_1512	ATCGGAGTAACAGGTTATGCAAAGCAGGAAGAAATAGCGGGATTCTTTACCAATACTGCT	kaiC	circadian clock protein KaiC	WP_011130786.: 1506391..1507890	Circadian Rhythm	COG0467
WP_011130787.1_1513	CUST_8022_PI442190945	MIT9313	LLIV	WP_011130787.1_1513	ATGAGCCCAAGAAAAACCTACATCCTCAAGTTGTACGTAGCAGGGAATACCCCTAATTC	kaiB	Circadian clock protein KaiB	WP_011130787.: 1507957..1508316	Circadian Rhythm	COG4251
WP_011130787.1_1513	CUST_8023_PI442190945	MIT9313	LLIV	WP_011130787.1_1513	GAACGGGTTTTGATTGGTCTTGACCTCCTCTATGACGAGATCTCTGAGGAGATGCTC	kaiB	Circadian clock protein KaiB	WP_011130787.: 1507957..1508316	Circadian Rhythm	COG4251
WP_011130787.1_1513	CUST_8024_PI442190945	MIT9313	LLIV	WP_011130787.1_1513	CTCAAGGTGATCGATGTGCTCAAGAATCCTCAGCTTGCAGAGGCAGACAAAATTCTG	kaiB	Circadian clock protein KaiB	WP_011130787.: 1507957..1508316	Circadian Rhythm	COG4251
WP_011130787.1_1513	CUST_8025_PI442190945	MIT9313	LLIV	WP_011130787.1_1513	TAATTCGATGCGGGCGCTTAAAACTCTGCGCGATATCCTCGAGAACGAGTTC	kaiB	Circadian clock protein KaiB	WP_011130787.: 1507957..1508316	Circadian Rhythm	COG4251
WP_011130787.1_1513	CUST_8026_PI442190945	MIT9313	LLIV	WP_011130787.1_1513	GGAGCTCGATACGTTGGCAGACGATGACATTGCGTCGCCAGATTCTTAA	kaiB	Circadian clock protein KaiB	WP_011130787.: 1507957..1508316	Circadian Rhythm	COG4251
WP_011130787.1_1513	CUST_8027_PI442190945	MIT9313	LLIV	WP_011130787.1_1513	GACAAAATTCTGGCCACACCAACCTTGGCCAAGATCCTGCCGCCA	kaiB	Circadian clock protein KaiB	WP_011130787.: 1507957..1508316	Circadian Rhythm	COG4251
WP_011130820.1_1548	CUST_8211_PI442190945	MIT9313	LLIV	WP_011130820.1_1548	CAAATGGAAGGCCAGGAACCCTCAACCGAAAAAATAAAGGCACAACAGAACTTCAATC	atpC	ATP synthase epsilon subunit	WP_011130820.: 1539251..1539652	Energy Metabolism	COG0355
WP_011130820.1_1548	CUST_8212_PI442190945	MIT9313	LLIV	WP_011130820.1_1548	CTGATGGGTGGATTCGCCGAAGTTGAAGCAGACGATGTCACAGTGTTGGTGAATG	atpC	ATP synthase epsilon subunit	WP_011130820.: 1539251..1539652	Energy Metabolism	COG0355
WP_011130820.1_1548	CUST_8213_PI442190945	MIT9313	LLIV	WP_011130820.1_1548	CTGCTGAAGAGGTGATTCTTCCTAGCACCACAGGCCTGATTGGCATCTTG	atpC	ATP synthase epsilon subunit	WP_011130820.: 1539251..1539652	Energy Metabolism	COG0355
WP_011130820.1_1548	CUST_8214_PI442190945	MIT9313	LLIV	WP_011130820.1_1548	GAACTCGGCAAGAGCATTGATGCAACAACTGCTGAAGCAGAGCTGGAACAAG	atpC	ATP synthase epsilon subunit	WP_011130820.: 1539251..1539652	Energy Metabolism	COG0355
WP_011130820.1_1548	CUST_8215_PI442190945	MIT9313	LLIV	WP_011130820.1_1548	CATATCGCTTGTCACGGCCCTGGATATCGGTGTGATGAGGGTTCGAG	atpC	ATP synthase epsilon subunit	WP_011130820.: 1539251..1539652	Energy Metabolism	COG0355
WP_011130820.1_1548	CUST_8216_PI442190945	MIT9313	LLIV	WP_011130820.1_1548	ATGTCTCTCACCCTCCGCGTTCTGGCACCTGACCAGAGTGTGTTC	atpC	ATP synthase epsilon subunit	WP_011130820.: 1539251..1539652	Energy Metabolism	COG0355
WP_011130835.1_1563	CUST_8291_PI442190945	MIT9313	LLIV	WP_011130835.1_1563	TCAATCACGGATGGTCAGATTTTCTTGAGTTCAGATCTTTTCAACTCAGGATTGCGTCCT	atpA	ATP synthase alpha subunit	WP_011130835.: 1553023..1554540	Energy Metabolism	COG0056
WP_011130835.1_1563	CUST_8292_PI442190945	MIT9313	LLIV	WP_011130835.1_1563	AGTTTGATGAATTGGCTGCTTTCTCTCAGTTCGCCTCTGATTTAGATGAAGCAACTCAGA	atpA	ATP synthase alpha subunit	WP_011130835.: 1553023..1554540	Energy Metabolism	COG0056
WP_011130835.1_1563	CUST_8293_PI442190945	MIT9313	LLIV	WP_011130835.1_1563	CTGAGGATCAGGTCACTCAGTTTTCACGTGAATTGCGTGATTACCTCAAGACCAACAAG	atpA	ATP synthase alpha subunit	WP_011130835.: 1553023..1554540	Energy Metabolism	COG0056
WP_011130835.1_1563	CUST_8294_PI442190945	MIT9313	LLIV	WP_011130835.1_1563	CTGAGTCGTTCATGTACAAGGGCAAGGCGACTTTGGTCATCTACGACGATCTAACTAAG	atpA	ATP synthase alpha subunit	WP_011130835.: 1553023..1554540	Energy Metabolism	COG0056
WP_011130835.1_1563	CUST_8295_PI442190945	MIT9313	LLIV	WP_011130835.1_1563	TACATCACCAAAGTTCAGACTGAGAAGGTGCTCAACGAGGATGCTGAGACCATTCTCAAG	atpA	ATP synthase alpha subunit	WP_011130835.: 1553023..1554540	Energy Metabolism	COG0056
WP_011130835.1_1563	CUST_8296_PI442190945	MIT9313	LLIV	WP_011130835.1_1563	CTGAATTTGGCTGAGCAGGTTGCGATCGTTTATGCAGGAGTGAAAGGCTTGATTGATGAG	atpA	ATP synthase alpha subunit	WP_011130835.: 1553023..1554540	Energy Metabolism	COG0056
WP_011130840.1_1568	CUST_8319_PI442190945	MIT9313	LLIV	WP_011130840.1_1568	GTGATCCACACGGTGTTCAAAATCTTCTCGAGTTCCTCTGGGATTACATCCGTGATTTAG	atpI	ATP synthase subunit A	WP_011130840.: 1556671..1557396	Energy Metabolism	COG0356
WP_011130840.1_1568	CUST_8320_PI442190945	MIT9313	LLIV	WP_011130840.1_1568	AAGCCTTTATCACTCTCCTTTCGTCTGTTTGGAAACATCCTTGCTGATGAATTGGTTGTG	atpI	ATP synthase subunit A	WP_011130840.: 1556671..1557396	Energy Metabolism	COG0356
WP_011130840.1_1568	CUST_8321_PI442190945	MIT9313	LLIV	WP_011130840.1_1568	TTGTTCTTGTTCATCTTTGTAAGCAATTGGGGCGGGTCTTTGGTCCCATGGAAATTAATC	atpI	ATP synthase subunit A	WP_011130840.: 1556671..1557396	Energy Metabolism	COG0356
WP_011130840.1_1568	CUST_8322_PI442190945	MIT9313	LLIV	WP_011130840.1_1568	CTGACATCAATACCACCGTGGCACTTGCTCTACTTGTGTCGCTCTCTTACTTCTATG	atpI	ATP synthase subunit A	WP_011130840.: 1556671..1557396	Energy Metabolism	COG0356
WP_011130840.1_1568	CUST_8323_PI442190945	MIT9313	LLIV	WP_011130840.1_1568	GTACTTCGAGTACTACGTCCATCCAACCCCGATCATGATCCCCTTCAAGATCGTG	atpI	ATP synthase subunit A	WP_011130840.: 1556671..1557396	Energy Metabolism	COG0356
WP_011130840.1_1568	CUST_8324_PI442190945	MIT9313	LLIV	WP_011130840.1_1568	CAGATTGGCAATCTTCAGCTCCATGGCCAGGTGTTCATGACATCCTGGATTGTGATTG	atpI	ATP synthase subunit A	WP_011130840.: 1556671..1557396	Energy Metabolism	COG0356
WP_041384566.1_1581	CUST_8388_PI442190945	MIT9313	LLIV	WP_041384566.1_1581	GTGAAGCAGCACTAAAACTCGGCTATGTGAGCAAAGAAGACTTCGATCGCATTGTTAATC	fumC	class II fumarate hydratase	WP_041384566.: 1567711..1569108	Krebs cycle and related genes	COG0114
WP_041384566.1_1581	CUST_8389_PI442190945	MIT9313	LLIV	WP_041384566.1_1581	CAAACCGAATCAAGATCCAGCATGATCTAGAGCAATCGCTGATGCTAGTGACTGCTTTAG	fumC	class II fumarate hydratase	WP_041384566.: 1567711..1569108	Krebs cycle and related genes	COG0114
WP_041384566.1_1581	CUST_8390_PI442190945	MIT9313	LLIV	WP_041384566.1_1581	CAAATGAACGTCTACAAACCCCTGATTGGCTTCAACCTGCTTCACAGCATTGAATTG	fumC	class II fumarate hydratase	WP_041384566.: 1567711..1569108	Krebs cycle and related genes	COG0114
WP_041384566.1_1581	CUST_8391_PI442190945	MIT9313	LLIV	WP_041384566.1_1581	GATCAAATTGCCTCAGCCCATAGTCGTATTCAGTCAAGCCTGATAGAGCTCTACCC	fumC	class II fumarate hydratase	WP_041384566.: 1567711..1569108	Krebs cycle and related genes	COG0114
WP_041384566.1_1581	CUST_8392_PI442190945	MIT9313	LLIV	WP_041384566.1_1581	CTCAGTTGCGCATGTTGGCGGTTGCCCTTTTCAAGATCTCCAATGATCTG	fumC	class II fumarate hydratase	WP_041384566.: 1567711..1569108	Krebs cycle and related genes	COG0114
WP_041384566.1_1581	CUST_8393_PI442190945	MIT9313	LLIV	WP_041384566.1_1581	CTTTAGCCCCAGAAATCGGTTACGACAAAGCCAGTGAGATTGCCCACCTC	fumC	class II fumarate hydratase	WP_041384566.: 1567711..1569108	Krebs cycle and related genes	COG0114
WP_011131019.1_1760	CUST_9318_PI442190945	MIT9313	LLIV	WP_011131019.1_1760	GACTTGAAGGAGACACAATCATGATCGAGCCTGCTAGGGCTAACAGATTCAACGGTTAA	minC	septum site-determining protein MinC	WP_011131019.: 1759770..1760447	Cell Division	COG0850
WP_011131019.1_1760	CUST_9319_PI442190945	MIT9313	LLIV	WP_011131019.1_1760	GACACCAAAGCAAAAATTGTGGCCCTACAACTCAGACCACTTCAGCTGCGTATTG	minC	septum site-determining protein MinC	WP_011131019.: 1759770..1760447	Cell Division	COG0850
WP_011131019.1_1760	CUST_9320_PI442190945	MIT9313	LLIV	WP_011131019.1_1760	CTGAACAAAGAAGGAGTGACGCTGACCAGAATTCACGCCAACTTGCGAGAAAC	minC	septum site-determining protein MinC	WP_011131019.: 1759770..1760447	Cell Division	COG0850
WP_011131019.1_1760	CUST_9321_PI442190945	MIT9313	LLIV	WP_011131019.1_1760	CATATGGCCTCTCCACAGGGAAACAGCTCCAAAACACGCTCTAGCGATACACAAC	minC	septum site-determining protein MinC	WP_011131019.: 1759770..1760447	Cell Division	COG0850
WP_011131019.1_1760	CUST_9322_PI442190945	MIT9313	LLIV	WP_011131019.1_1760	GAGAAGCACATTGGCGAGAAACATTGCCGGCCATGCTCAATGAACTTGATC	minC	septum site-determining protein MinC	WP_011131019.: 1759770..1760447	Cell Division	COG0850
WP_011131019.1_1760	CUST_9323_PI442190945	MIT9313	LLIV	WP_011131019.1_1760	CAAAAACTGCTCTTCCACCAAGGCACTCTGCGATCAGGCGACCATC	minC	septum site-determining protein MinC	WP_011131019.: 1759770..1760447	Cell Division	COG0850
WP_011131020.1_1761	CUST_9324_PI442190945	MIT9313	LLIV	WP_011131020.1_1761	CAATTCGACTATGTATTGATTGATTGCCCCGCGGGGATCGAAGACGGCTTCAAAAATG	minD	septum site-determining protein MinD	WP_011131020.: 1760601..1761416	Cell Division	COG2894
WP_011131020.1_1761	CUST_9325_PI442190945	MIT9313	LLIV	WP_011131020.1_1761	CCCTTGCTTGGTTTAGTTCTAGAGGACGAACAGGTGATTGTGAGCACCAACC	minD	septum site-determining protein MinD	WP_011131020.: 1760601..1761416	Cell Division	COG2894
WP_011131020.1_1761	CUST_9326_PI442190945	MIT9313	LLIV	WP_011131020.1_1761	GTATCGCTCTTGCCAAGCTTGGAGTTCAAACCGTTGTGTTGGATGCCGATTTC	minD	septum site-determining protein MinD	WP_011131020.: 1760601..1761416	Cell Division	COG2894
WP_011131020.1_1761	CUST_9327_PI442190945	MIT9313	LLIV	WP_011131020.1_1761	CAAGGAAGGACTGGGACTACGCGAAAAGGTGTGGCGCTTGATGCAAACCAAGATCTTTTG	minD	septum site-determining protein MinD	WP_011131020.: 1760601..1761416	Cell Division	COG2894
WP_011131020.1_1761	CUST_9328_PI442190945	MIT9313	LLIV	WP_011131020.1_1761	CTGGTGCTCAACAGGGTGCGTCCAAAGATGATGGCCAATCAAGAGATG	minD	septum site-determining protein MinD	WP_011131020.: 1760601..1761416	Cell Division	COG2894
WP_011131020.1_1761	CUST_9329_PI442190945	MIT9313	LLIV	WP_011131020.1_1761	TTCAAAAATGCTGTTGCTGCCGCCAAGGAAGCAATCGTGATCACAACG	minD	septum site-determining protein MinD	WP_011131020.: 1760601..1761416	Cell Division	COG2894
WP_011131021.1_1762	CUST_9330_PI442190945	MIT9313	LLIV	WP_011131021.1_1762	CAAGAGGCTGTCAGCAGCCAAGAATCCGTGTCAACTCCTGGAGCAATGGAATAG	minE	septum site-determining protein MinE	WP_011131021.: 1761421..1761777	Cell Division	COG0851
WP_011131021.1_1762	CUST_9331_PI442190945	MIT9313	LLIV	WP_011131021.1_1762	CTAACTTGCCGATCAAACGTTCCCAAGCCAAGGCTGTCAGTAGCCAAGAGAATG	minE	septum site-determining protein MinE	WP_011131021.: 1761421..1761777	Cell Division	COG0851
WP_011131021.1_1762	CUST_9332_PI442190945	MIT9313	LLIV	WP_011131021.1_1762	GAAATCTTTGAGGTCGTTGCCAAGTACGTGGAGATTGACCTTGAGGAAGGCGAAGTAAG	minE	septum site-determining protein MinE	WP_011131021.: 1761421..1761777	Cell Division	COG0851
WP_011131021.1_1762	CUST_9333_PI442190945	MIT9313	LLIV	WP_011131021.1_1762	CTTCAGCTTGTGCTTGCCCATGACCGCAGCGATCTAAGCCCTGAACTGCTCGATCAAATG	minE	septum site-determining protein MinE	WP_011131021.: 1761421..1761777	Cell Division	COG0851
WP_011131021.1_1762	CUST_9334_PI442190945	MIT9313	LLIV	WP_011131021.1_1762	CAACCACTCTGCGAGACATTCTTGACAAATTGCTGGGCCGCCAACC	minE	septum site-determining protein MinE	WP_011131021.: 1761421..1761777	Cell Division	COG0851
WP_052646240.1_1825	CUST_9655_PI442190945	MIT9313	LLIV	WP_052646240.1_1825	CATTCTTCCGAATGCTGATCTCCAAGGTGGAGATGACCCTTTCAAAAGTAGACCTAGAC	ppc	Phosphoenolpyruvate carboxylase	WP_052646240.: 1813364..1816342	Krebs cycle and related genes	COG2352
WP_052646240.1_1825	CUST_9656_PI442190945	MIT9313	LLIV	WP_052646240.1_1825	GAAGATTACCGTGAAGCCTTTAACCGTATCTTCGAGATCATCGAAACGGAGTACAGCCTC	ppc	Phosphoenolpyruvate carboxylase	WP_052646240.: 1813364..1816342	Krebs cycle and related genes	COG2352
WP_052646240.1_1825	CUST_9657_PI442190945	MIT9313	LLIV	WP_052646240.1_1825	CTTCTTCCAACAGGTCACGCCGATCGAAGAAATCAGCAAATTGCAAATCTCCAG	ppc	Phosphoenolpyruvate carboxylase	WP_052646240.: 1813364..1816342	Krebs cycle and related genes	COG2352
WP_052646240.1_1825	CUST_9658_PI442190945	MIT9313	LLIV	WP_052646240.1_1825	CTACAGCTATCAGTAGATCTTCGCAATCGCACGATCGTGCCCCTTGGGTTTCTG	ppc	Phosphoenolpyruvate carboxylase	WP_052646240.: 1813364..1816342	Krebs cycle and related genes	COG2352
WP_052646240.1_1825	CUST_9659_PI442190945	MIT9313	LLIV	WP_052646240.1_1825	AACCAACCAGCTAGATGCCACCCCAAGTTGGAATCAACTGATGACCAGAC	ppc	Phosphoenolpyruvate carboxylase	WP_052646240.: 1813364..1816342	Krebs cycle and related genes	COG2352
WP_052646240.1_1825	CUST_9660_PI442190945	MIT9313	LLIV	WP_052646240.1_1825	CTGAAGTCGAATCAGACGCTGACCAGCTTGACCTTCTACGCAGACTGC	ppc	Phosphoenolpyruvate carboxylase	WP_052646240.: 1813364..1816342	Krebs cycle and related genes	COG2352
WP_011131136.1_1884	CUST_9979_PI442190945	MIT9313	LLIV	WP_011131136.1_1884	GTTCAGGGATTATCTCTGGATCAATAGTTCCCAGATCATCAATGGCTACAACCCATTCGG	psaB	Photosystem I P700 chlorophyll A apoprotein A2 PsaB	WP_011131136.1	Photosystem I	COG2885
WP_011131136.1_1884	CUST_9980_PI442190945	MIT9313	LLIV	WP_011131136.1_1884	GGCAACATATTTACCTTCGGTGCCTTTGTGATTGCATCTACAGCTAGCAAGTTTGGATAA	psaB	Photosystem I P700 chlorophyll A apoprotein A2 PsaB	WP_011131136.1	Photosystem I	COG2885
WP_011131136.1_1884	CUST_9981_PI442190945	MIT9313	LLIV	WP_011131136.1_1884	ATCACTACTGGATGGATCTGATTAATGCCAGTCCAGAGGTTTCCAACTTCATGCCTATTG	psaB	Photosystem I P700 chlorophyll A apoprotein A2 PsaB	WP_011131136.1	Photosystem I	COG2885
WP_011131136.1_1884	CUST_9982_PI442190945	MIT9313	LLIV	WP_011131136.1_1884	CTCCACTTACATGGCTATTTTCTGGGCACTGAATACCATTGCTTGGGCAACTTATTACTG	psaB	Photosystem I P700 chlorophyll A apoprotein A2 PsaB	WP_011131136.1	Photosystem I	COG2885
WP_011131136.1_1884	CUST_9983_PI442190945	MIT9313	LLIV	WP_011131136.1_1884	ATTGATCTTGATCAAGGGAGCACTTGATGCAAGAGGTTCCAAGCTGATGCCCGATAAAAA	psaB	Photosystem I P700 chlorophyll A apoprotein A2 PsaB	WP_011131136.1	Photosystem I	COG2885
WP_011131136.1_1884	CUST_9984_PI442190945	MIT9313	LLIV	WP_011131136.1_1884	GATTAATAATCTCTCCCCTTGGGCATACATGTTCCTTGCAGGCCATTTGGTCTGG	psaB	Photosystem I P700 chlorophyll A apoprotein A2 PsaB	WP_011131136.1	Photosystem I	COG2885
WP_011131137.1_1885	CUST_9985_PI442190945	MIT9313	LLIV	WP_011131137.1_1885	GTGATCTTCTACTTCTCGTGGAAGATGCAAAGCGATGTATGGGGCACTGTTAATTCAGAC	psaA	Photosystem I P700 chlorophyll A apoprotein A1 PsaA	WP_011131137.1	Photosystem I	COG2885
WP_011131137.1_1885	CUST_9986_PI442190945	MIT9313	LLIV	WP_011131137.1_1885	CTACTGGCAGGAGCTATTTGAATCCATTATCTGGGCTCATAACAAGCTCAAGCTTGCTC	psaA	Photosystem I P700 chlorophyll A apoprotein A1 PsaA	WP_011131137.1	Photosystem I	COG2885
WP_011131137.1_1885	CUST_9987_PI442190945	MIT9313	LLIV	WP_011131137.1_1885	TTGATGATTCACCACATCCATGCCTTCACCATTCACGTAACTTGCCTCATCCTTCTCAAG	psaA	Photosystem I P700 chlorophyll A apoprotein A1 PsaA	WP_011131137.1	Photosystem I	COG2885
WP_011131137.1_1885	CUST_9988_PI442190945	MIT9313	LLIV	WP_011131137.1_1885	GACTACAGGTCTGCCAGGTGCAGTTAGTGATGTGTTCAACGGAAGTGTTGTAGCAGTAG	psaA	Photosystem I P700 chlorophyll A apoprotein A1 PsaA	WP_011131137.1	Photosystem I	COG2885
WP_011131137.1_1885	CUST_9989_PI442190945	MIT9313	LLIV	WP_011131137.1_1885	GAGTGTGACTCATCTCGTTTCTGGCAACTTTGCTCAAAGCGCCATCACTGTGAATG	psaA	Photosystem I P700 chlorophyll A apoprotein A1 PsaA	WP_011131137.1	Photosystem I	COG2885
WP_011131137.1_1885	CUST_9990_PI442190945	MIT9313	LLIV	WP_011131137.1_1885	CTATTCCTTGGTGGCCACTTCGTGTGGGCCTTTAGCTTGATGTTCTTGTTCAG	psaA	Photosystem I P700 chlorophyll A apoprotein A1 PsaA	WP_011131137.1	Photosystem I	COG2885
WP_011131144.1_1892	CUST_10024_PI442190945	MIT9313	LLIV	WP_011131144.1_1892	ACAAAGAGATTGTTGAGATCTGCTTGCTAACCACTATTGAGCAGGAAGCCATTCTCAAGA	glsF	Ferredoxin-dependent glutamate synthase	WP_011131144.: 1871737..1876320	Nitrogen Metabolism	COG0069
WP_011131144.1_1892	CUST_10025_PI442190945	MIT9313	LLIV	WP_011131144.1_1892	TCAGTTGGAAAGCACTTTTTAAGGTGCTTGTTCCACCTAGTGAAAAGGTCAATGTTGGTT	glsF	Ferredoxin-dependent glutamate synthase	WP_011131144.: 1871737..1876320	Nitrogen Metabolism	COG0069
WP_011131144.1_1892	CUST_10026_PI442190945	MIT9313	LLIV	WP_011131144.1_1892	CAATGATTATGTCGGTAAAGGCATGAATGGTGGTCGGATCACACTGGTACCACCTTC	glsF	Ferredoxin-dependent glutamate synthase	WP_011131144.: 1871737..1876320	Nitrogen Metabolism	COG0069
WP_011131144.1_1892	CUST_10027_PI442190945	MIT9313	LLIV	WP_011131144.1_1892	GATTTCTGGTGAGATAGCTGAACGCCATGGCAACAAAGGTTTTCGAGGTCAGTTCGATC	glsF	Ferredoxin-dependent glutamate synthase	WP_011131144.: 1871737..1876320	Nitrogen Metabolism	COG0069
WP_011131144.1_1892	CUST_10028_PI442190945	MIT9313	LLIV	WP_011131144.1_1892	CTGTTGTTGAAGGTGCGGGGGATCATTGCTGTGAATACATGACCGGTG	glsF	Ferredoxin-dependent glutamate synthase	WP_011131144.: 1871737..1876320	Nitrogen Metabolism	COG0069
WP_011131144.1_1892	CUST_10029_PI442190945	MIT9313	LLIV	WP_011131144.1_1892	GATTTTGGGCAACACTTGCCTGTATGGCGGACGGTGGAGAGTTGTGCTTTAGGTAG	glsF	Ferredoxin-dependent glutamate synthase	WP_011131144.: 1871737..1876320	Nitrogen Metabolism	COG0069
WP_011131270.1_2022	CUST_10736_PI442190945	MIT9313	LLIV	WP_011131270.1_2022	TTGAGAAAGACATCCTCAATCTGGTTTATCTTTGTGATCATTTGGTTTGCCGGATGTGG	ugd	UDP-glucose 6-dehydrogenase/nucleotide sugar dehydrogenase	WP_011131270.: 2007329..2008777	Amino sugar and nucleotide sugar r	COG1004
WP_011131270.1_2022	CUST_10737_PI442190945	MIT9313	LLIV	WP_011131270.1_2022	CTGTTGTCGATCCAGAAGTTGGTCCAGGTTTGATCGGCTTTGGCGAGTTTGGAGATG	ugd	UDP-glucose 6-dehydrogenase/nucleotide sugar dehydrogenase	WP_011131270.: 2007329..2008777	Amino sugar and nucleotide sugar r	COG1004

WP_011131270.1_2022	CUST_10737_PI442190945	MIT9313	LLIV	WP_011131270.1_2022	GACTATTGGGAAAATGTGGTAGCTCTCAATACTTGGCAGCAGCACCGCATTTCTCGTTTG	ugd	UDP-glucose 6-dehydrogenase/nucleotide sugar dehydrogenase	WP_011131270.:	2007329..2008777	Amino sugar and nucleotide sugar r	COG1004
WP_011131270.1_2022	CUST_10738_PI442190945	MIT9313	LLIV	WP_011131270.1_2022	CTCAGCTGGCTATTTATGATCCGAAGGTGGATCCGCAACAGATTGCGTTTGATTTG	ugd	UDP-glucose 6-dehydrogenase/nucleotide sugar dehydrogenase	WP_011131270.:	2007329..2008777	Amino sugar and nucleotide sugar r	COG1004
WP_011131270.1_2022	CUST_10739_PI442190945	MIT9313	LLIV	WP_011131270.1_2022	GCTGATCCTGACTGAGTGGCATGAGTTTAGACAGCTTGATTGGGTCACCTTG	ugd	UDP-glucose 6-dehydrogenase/nucleotide sugar dehydrogenase	WP_011131270.:	2007329..2008777	Amino sugar and nucleotide sugar r	COG1004
WP_011131270.1_2022	CUST_10740_PI442190945	MIT9313	LLIV	WP_011131270.1_2022	TCTCGTTTGGTGGTTCAAAAGCTTTTTGGAACGGTTACAGGTAAGCGGCTG	ugd	UDP-glucose 6-dehydrogenase/nucleotide sugar dehydrogenase	WP_011131270.:	2007329..2008777	Amino sugar and nucleotide sugar r	COG1004
WP_011131291.1_2041	CUST_10849_PI442190945	MIT9313	LLIV	WP_011131291.1_2041	GACAAACCGATCTCATTGAAGCAATGGCCAAAACTGGCGCAGTCATCAACATCAAAAAG	eda	2-dehydro-3-deoxyphosphogluconate aldolase	WP_011131291.:	2033006..2033857	Entner-Dudoroff	COG0800
WP_011131291.1_2041	CUST_10850_PI442190945	MIT9313	LLIV	WP_011131291.1_2041	CAACTTCGGCTATGACAATCTTGTGGTTGACATGCTCGCCTTCGGAGTAATGAAG	eda	2-dehydro-3-deoxyphosphogluconate aldolase	WP_011131291.:	2033006..2033857	Entner-Dudoroff	COG0800
WP_011131291.1_2041	CUST_10851_PI442190945	MIT9313	LLIV	WP_011131291.1_2041	CAAATGTCAAACGTGGTGGAAAAGTTCCGCGAATGCGGCAACGAGAATTTGCTTATCTG	eda	2-dehydro-3-deoxyphosphogluconate aldolase	WP_011131291.:	2033006..2033857	Entner-Dudoroff	COG0800
WP_011131291.1_2041	CUST_10852_PI442190945	MIT9313	LLIV	WP_011131291.1_2041	CTTGAACCTTTTCTCCAGCAGGTCAAAGCGATCGATGAGGTTGTGAAAGCACTC	eda	2-dehydro-3-deoxyphosphogluconate aldolase	WP_011131291.:	2033006..2033857	Entner-Dudoroff	COG0800
WP_011131291.1_2041	CUST_10853_PI442190945	MIT9313	LLIV	WP_011131291.1_2041	GAGATTCTTCAAGTGGTGAAAGACACCCACGGGATCCCCGTGATTACCGATG	eda	2-dehydro-3-deoxyphosphogluconate aldolase	WP_011131291.:	2033006..2033857	Entner-Dudoroff	COG0800
WP_011131291.1_2041	CUST_10854_PI442190945	MIT9313	LLIV	WP_011131291.1_2041	GATCTAGCACGTTCTGGCATGGCAGTAGGCCTGGCAGGACTTTTCCTAGAATCTCAC	eda	2-dehydro-3-deoxyphosphogluconate aldolase	WP_011131291.:	2033006..2033857	Entner-Dudoroff	COG0800
WP_011131318.1_2068	CUST_10996_PI442190945	MIT9313	LLIV	WP_011131318.1_2068	GATCAACGCTGCGAAGTTTTAGCTAACCGCTTCGCTGATACTCAGGATGTGATCTTTTTG	glmS	Glutamine--fructose-6-phosphatetransaminase(isomerizing)	WP_011131318.:	2063076..2064980	Amino Acid metabolism	COG0449
WP_011131318.1_2068	CUST_10997_PI442190945	MIT9313	LLIV	WP_011131318.1_2068	CATCAACTATCCCATTGCCCTTGAGGGAGCTCTCAAGCTTAAGGAGATCAGCTATATC	glmS	Glutamine--fructose-6-phosphatetransaminase(isomerizing)	WP_011131318.:	2063076..2064980	Amino Acid metabolism	COG0449
WP_011131318.1_2068	CUST_10998_PI442190945	MIT9313	LLIV	WP_011131318.1_2068	CTTGTTCCGATACAGCTACTGAGCTATCACATTGCAGCTCATAGGGGTCTTGATGTG	glmS	Glutamine--fructose-6-phosphatetransaminase(isomerizing)	WP_011131318.:	2063076..2064980	Amino Acid metabolism	COG0449
WP_011131318.1_2068	CUST_10999_PI442190945	MIT9313	LLIV	WP_011131318.1_2068	CTCATTGAGTGAGATTGCCAGCCTCAAAGACGAACTAAGGGCTTTACCGGATCAG	glmS	Glutamine--fructose-6-phosphatetransaminase(isomerizing)	WP_011131318.:	2063076..2064980	Amino Acid metabolism	COG0449
WP_011131318.1_2068	CUST_11000_PI442190945	MIT9313	LLIV	WP_011131318.1_2068	GAATCGGCCAGAAAGCTCCCTGACTCGTCAGGTTCCATACATTCTCGACATC	glmS	Glutamine--fructose-6-phosphatetransaminase(isomerizing)	WP_011131318.:	2063076..2064980	Amino Acid metabolism	COG0449
WP_011131318.1_2068	CUST_11001_PI442190945	MIT9313	LLIV	WP_011131318.1_2068	CTACAAAGACCTTCTTGGGGCAGTTGTTGGCTTTCTATGGGCTTGCACTGGCATTTG	glmS	Glutamine--fructose-6-phosphatetransaminase(isomerizing)	WP_011131318.:	2063076..2064980	Amino Acid metabolism	COG0449
WP_011131447.1_2207	CUST_11756_PI442190945	MIT9313	LLIV	WP_011131447.1_2207	GCAGGGAATTGATCAAAACGTAGCCAATTCCATCCTGATTAAAGTGAATCAGATCGGCTC	eno	Enolase	WP_011131447.:	2196566..2197861	Glycolysis/Gluconeogenesis	COG0148
WP_011131447.1_2207	CUST_11757_PI442190945	MIT9313	LLIV	WP_011131447.1_2207	TTGATCGCTACCCAATTATTTCGATCGAAGATGGACTAGCAGAAGATGATTGGCAAGGTT	eno	Enolase	WP_011131447.:	2196566..2197861	Glycolysis/Gluconeogenesis	COG0148
WP_011131447.1_2207	CUST_11758_PI442190945	MIT9313	LLIV	WP_011131447.1_2207	AATTGGGGAAGCGTATCCAACTGATAGGTGACGATATTTTTGTAACGAGTACTAAGCGCC	eno	Enolase	WP_011131447.:	2196566..2197861	Glycolysis/Gluconeogenesis	COG0148
WP_011131447.1_2207	CUST_11759_PI442190945	MIT9313	LLIV	WP_011131447.1_2207	GATCTGCTCATTCAAGCAATCGAAAAAGCAGGCTATTCGCCAGGTAAGGATATTTCCTTG	eno	Enolase	WP_011131447.:	2196566..2197861	Glycolysis/Gluconeogenesis	COG0148
WP_011131447.1_2207	CUST_11760_PI442190945	MIT9313	LLIV	WP_011131447.1_2207	TTCCTTGGCACTAGATGTAGCCAGCACAGAGTTCTATCAAGACGGCTGCTATG	eno	Enolase	WP_011131447.:	2196566..2197861	Glycolysis/Gluconeogenesis	COG0148
WP_011131447.1_2207	CUST_11761_PI442190945	MIT9313	LLIV	WP_011131447.1_2207	CTAAGTCGCAGTGAACGGGTAGCGAAATACAACCAACTGCTGCGAATCGAAGACGAACTC	eno	Enolase	WP_011131447.:	2196566..2197861	Glycolysis/Gluconeogenesis	COG0148
WP_011375577.1_12	CUST_67_PI442190940	MIT9312	HLII	WP_011375577.1_12	AATGTGCGACGGGTAGTAGTTTAATGCCGCAGAAAAAAAATCCAGATGTTCCAGAATTGA	argH	Argininosuccinate lyase	WP_011375577.:	14583..15962	Amino Acid metabolism	COG0165
WP_011375577.1_12	CUST_68_PI442190940	MIT9312	HLII	WP_011375577.1_12	AAGAACAAGTGAGGGTGGAACAGGTTTTACTCAAGTAGAAAAGGAGGTCAATAATTGGCA	argH	Argininosuccinate lyase	WP_011375577.:	14583..15962	Amino Acid metabolism	COG0165
WP_011375577.1_12	CUST_69_PI442190940	MIT9312	HLII	WP_011375577.1_12	TGCTGTGAGTGATAGGGATTTTTGTATAGAGTTTGTTTCTGCATCTGCTTTGGCAATGTC	argH	Argininosuccinate lyase	WP_011375577.:	14583..15962	Amino Acid metabolism	COG0165
WP_011375577.1_12	CUST_70_PI442190940	MIT9312	HLII	WP_011375577.1_12	TGCCACTGATTTGGCAGATTACTTAGTTTGTAAACAGGTTCCTTTTAGGACTGCTTATCA	argH	Argininosuccinate lyase	WP_011375577.:	14583..15962	Amino Acid metabolism	COG0165
WP_011375577.1_12	CUST_71_PI442190940	MIT9312	HLII	WP_011375577.1_12	ACGGGAAGAGTTTATGGAAATCTCCAAGCATTATTAACGATGGTTAAAGGTGTACCACTT	argH	Argininosuccinate lyase	WP_011375577.:	14583..15962	Amino Acid metabolism	COG0165
WP_011375577.1_12	CUST_72_PI442190940	MIT9312	HLII	WP_011375577.1_12	TCATCCCGAATTTGATGAGGATGTTTTTGTGGATCTTGAACCTTTTAATGTTGTTAAGTC	argH	Argininosuccinate lyase	WP_011375577.:	14583..15962	Amino Acid metabolism	COG0165
WP_011375776.1_210	CUST_1169_PI442190940	MIT9312	HLII	WP_011375776.1_210	TAGGCTTTTAAAAATTGAGGAGGAGTTGGGAAATCAAGCAAGATTCGCTGGAGCTTTAGG	eno	Enolase	WP_011375776.:	202018..203310	Glycolysis/Gluconeogenesis	COG0148
WP_011375776.1_210	CUST_1170_PI442190940	MIT9312	HLII	WP_011375776.1_210	AGTGGTGAGACTGAAGATACAACAATTGCTGATTTATCTGTCGCCACAAGATCTGGTCAG	eno	Enolase	WP_011375776.:	202018..203310	Glycolysis/Gluconeogenesis	COG0148
WP_011375776.1_210	CUST_1171_PI442190940	MIT9312	HLII	WP_011375776.1_210	TATCCAATAGTTTCAATAGAGGACGGTTTAGCAGAGGATGATTGGGAGGGTTGGTCAGAA	eno	Enolase	WP_011375776.:	202018..203310	Glycolysis/Gluconeogenesis	COG0148
WP_011375776.1_210	CUST_1172_PI442190940	MIT9312	HLII	WP_011375776.1_210	GCAATTCAAAAAGCCGGATTTATTCCTGGTGAGCAAGTATCTTTAGCTTTAGATGCTGCT	eno	Enolase	WP_011375776.:	202018..203310	Glycolysis/Gluconeogenesis	COG0148
WP_011375776.1_210	CUST_1173_PI442190940	MIT9312	HLII	WP_011375776.1_210	GAGGGAGGATTTGCACCTAATTTGTCATCCAGCGAAGAAGCAGGGGACTTATTATTAGAA	eno	Enolase	WP_011375776.:	202018..203310	Glycolysis/Gluconeogenesis	COG0148
WP_011375776.1_210	CUST_1174_PI442190940	MIT9312	HLII	WP_011375776.1_210	AAATGGTGGTGCTCATGCTCCAAATAGTCTTGATTTTCAGGAATTTATGCTTGTCCCACA	eno	Enolase	WP_011375776.:	202018..203310	Glycolysis/Gluconeogenesis	COG0148
WP_011375793.1_227	CUST_1258_PI442190940	MIT9312	HLII	WP_011375793.1_227	AGATTTTGTATCCGATATCAAAGTCAAATATCCAAGAATTAATTTAGGCTCTGCTTCCGT	eda	2-dehydro-3-deoxyphosphogluconate aldolase	WP_011375793.:	219901..220527	Entner-Dudoroff	COG0800
WP_011375793.1_227	CUST_1259_PI442190940	MIT9312	HLII	WP_011375793.1_227	AATTCCCGGAATTAAAAATTTAAAAGATCTTGAGGAAGCGATAAATTTAAATTGCAACAT	eda	2-dehydro-3-deoxyphosphogluconate aldolase	WP_011375793.:	219901..220527	Entner-Dudoroff	COG0800
WP_011375793.1_227	CUST_1260_PI442190940	MIT9312	HLII	WP_011375793.1_227	TGATTTCATTGCTGCTGGAGGACTATCAATCAATGATGTAAAAAATTATAAATCTTTAGG	eda	2-dehydro-3-deoxyphosphogluconate aldolase	WP_011375793.:	219901..220527	Entner-Dudoroff	COG0800
WP_011375793.1_227	CUST_1261_PI442190940	MIT9312	HLII	WP_011375793.1_227	TGGATTAAATTTTTCGATGATGAAATTTTGGGATAAAGATCTTTTCAATTATGCGAAGTC	eda	2-dehydro-3-deoxyphosphogluconate aldolase	WP_011375793.:	219901..220527	Entner-Dudoroff	COG0800
WP_011375793.1_227	CUST_1262_PI442190940	MIT9312	HLII	WP_011375793.1_227	TCAAATACCTCTATAAGGAATTCATTTTTCGACGAATTAGAAAGCTTAGTAAAAGTAGGA	eda	2-dehydro-3-deoxyphosphogluconate aldolase	WP_011375793.:	219901..220527	Entner-Dudoroff	COG0800
WP_011375793.1_227	CUST_1263_PI442190940	MIT9312	HLII	WP_011375793.1_227	AGATTCTTTTTCGGAGTTACTGAAAAAAGAATCTTTTTTTTACTTATAAAACCTGAAGA	eda	2-dehydro-3-deoxyphosphogluconate aldolase	WP_011375793.:	219901..220527	Entner-Dudoroff	COG0800
WP_011375887.1_326	CUST_1784_PI442190940	MIT9312	HLII	WP_011375887.1_326	CAAAAGAACTATTTCAGGAGAAATAAAATTCAAAAAAAATGATAAAACTGATCAAGCTGA	minE	septum site-determining protein MinE	WP_011375887.:	309737..310072	Cell Division	COG0851
WP_011375887.1_326	CUST_1785_PI442190940	MIT9312	HLII	WP_011375887.1_326	GAGGTGGCAGTAAGCTTAGAGACTGAGGATAGAATGACTGCATTAGTTGCCAATTTACCA	minE	septum site-determining protein MinE	WP_011375887.:	309737..310072	Cell Division	COG0851
WP_011375887.1_326	CUST_1786_PI442190940	MIT9312	HLII	WP_011375887.1_326	AGTTCCTTAACGACTGATCTTTTGGATAAAATGAGAAAAGAAATTCTCGATGTTGTTGCA	minE	septum site-determining protein MinE	WP_011375887.:	309737..310072	Cell Division	COG0851
WP_011375887.1_326	CUST_1787_PI442190940	MIT9312	HLII	WP_011375887.1_326	TGCCAGTGCAAATACAGCTAGAGAAAGATTACAACTTGTACTAGCTCATGACAGAGTCGA	minE	septum site-determining protein MinE	WP_011375887.:	309737..310072	Cell Division	COG0851
WP_011375888.1_327	CUST_1788_PI442190940	MIT9312	HLII	WP_011375888.1_327	CCTGCTGGAGTAGAAGATGGTTTTAAAAATGCCCTTGCAGCTTGTAAAGAGGCTATTGTT	minD	septum site-determining protein MinD	WP_011375888.:	310075..310890	Cell Division	COG2894
WP_011375888.1_327	CUST_1789_PI442190940	MIT9312	HLII	WP_011375888.1_327	GGATGTTGGATTGGATGAAGCCCGACGATATGAAAAAAATTAGCGAGCTGCTTAGTGAAA	minD	septum site-determining protein MinD	WP_011375888.:	310075..310890	Cell Division	COG2894
WP_011375888.1_327	CUST_1790_PI442190940	MIT9312	HLII	WP_011375888.1_327	CATAGCACTTGCCAACAGTGGAGCAACTACTGCTGTATTAGATGCTGATTTTGGCTTAAG	minD	septum site-determining protein MinD	WP_011375888.:	310075..310890	Cell Division	COG2894
WP_011375888.1_327	CUST_1791_PI442190940	MIT9312	HLII	WP_011375888.1_327	TCCAGCTTGTAATCAATAGAGTTCGCCCTAACATGATGGCAAGTCAAGAAATGCTATCCA	minD	septum site-determining protein MinD	WP_011375888.:	310075..310890	Cell Division	COG2894
WP_011375888.1_327	CUST_1792_PI442190940	MIT9312	HLII	WP_011375888.1_327	ACTTACAGGAAAGGATGTCCCAATTATTGACCCCAAAAATGAAGGGAAAAGCCTCAAAGA	minD	septum site-determining protein MinD	WP_011375888.:	310075..310890	Cell Division	COG2894
WP_011375888.1_327	CUST_1793_PI442190940	MIT9312	HLII	WP_011375888.1_327	TGACCAAGCATTAGTCAGACATAAAAAAGAACCTAATCTGGCTCTTTTACCTGCAGGAGA	minD	septum site-determining protein MinD	WP_011375888.:	310075..310890	Cell Division	COG2894
WP_011375889.1_328	CUST_1794_PI442190940	MIT9312	HLII	WP_011375889.1_328	CAAAGAACAATATTTACGTTTGGGGCAAACTACTAGGTATCGCCTTCGCAGGAAAAGGTG	minC	septum site-determining protein MinC	WP_011375889.:	311008..311667	Cell Division	COG0850
WP_011375889.1_328	CUST_1795_PI442190940	MIT9312	HLII	WP_011375889.1_328	TCCTTCACGAAGGAACAGTAAGATCTGGAGACAGAATATCTGCAAATGGAGACCTATGCA	minC	septum site-determining protein MinC	WP_011375889.:	311008..311667	Cell Division	COG0850
WP_011375889.1_328	CUST_1796_PI442190940	MIT9312	HLII	WP_011375889.1_328	ACCTTTACAGTTAAGAATTGCTAATGTGGTAGCTATTGGACCAAAGGATAAGCCCAAGAA	minC	septum site-determining protein MinC	WP_011375889.:	311008..311667	Cell Division	COG0850
WP_011375889.1_328	CUST_1797_PI442190940	MIT9312	HLII	WP_011375889.1_328	GACATCCGTTCCTTATGCATTTACTCGAATAATAGACATACGATATTAGCAGGAAAGTCT	minC	septum site-determining protein MinC	WP_011375889.:	311008..311667	Cell Division	COG0850
WP_011375889.1_328	CUST_1798_PI442190940	MIT9312	HLII	WP_011375889.1_328	ACAATATCGATGAAACTTTCAAAGAATTTTCTTCGATAAAAGAACCTCTGGAAGCAAAGA	minC	septum site-determining protein MinC	WP_011375889.:	311008..311667	Cell Division	COG0850
WP_011375889.1_328	CUST_1799_PI442190940	MIT9312	HLII	WP_011375889.1_328	TTAAAGAACAAGAAGTTAAAAAAAAGTTACTTCTACTCGATTCAAAAAATAAAGACGGCA	minC	septum site-determining protein MinC	WP_011375889.:	311008..311667	Cell Division	COG0850
WP_011376003.1_445	CUST_2407_PI442190940	MIT9312	HLII	WP_011376003.1_445	TGTTTGGTCTTATAACTTTCTTAATTGCGGACGGGATGACTTTTGCTGGATTCTTCGCTG	cyoC	heme-copper oxidase subunit III	WP_011376003.:	418772..419374	Respiration	COG1845
WP_011376003.1_445	CUST_2408_PI442190940	MIT9312	HLII	WP_011376003.1_445	TGGCGGAAGAGTAACTAGTCAAAATATGTTTCCATTAGAAGCTGTTGAATTGTACTGGCA	cyoC	heme-copper oxidase subunit III	WP_011376003.:	418772..419374	Respiration	COG1845
WP_011376003.1_445	CUST_2409_PI442190940	MIT9312	HLII	WP_011376003.1_445	AGTGCAACTTTTCATAAAGCAGGTAAAGCACTTTTGAAAGATAAAAACCCTGATGCTCAA	cyoC	heme-copper oxidase subunit III	WP_011376003.:	418772..419374	Respiration	COG1845
WP_011376003.1_445	CUST_2410_PI442190940	MIT9312	HLII	WP_011376003.1_445	AGCAGTAAATCCATTACCTGATGGTGCTATCTATGAATTAGAGCTTCCAATACCTACACT	cyoC	heme-copper oxidase subunit III	WP_011376003.:	418772..419374	Respiration	COG1845
WP_011376003.1_445	CUST_2411_PI442190940	MIT9312	HLII	WP_011376003.1_445	ACCTTTTGGATTAACCGACAATTTATTTGCAAGTACTTTTTATGCTCTTACTGGTTTTCA	cyoC	heme-copper oxidase subunit III	WP_011376003.:	418772..419374	Respiration	COG1845
WP_011376003.1_445	CUST_2412_PI442190940	MIT9312	HLII	WP_011376003.1_445	TGGATTACATGTAACTTTAGGTACTTTAATGATTTTAATTATTGCTTGGCAATCGAGAAT	cyoC	heme-copper oxidase subunit III	WP_011376003.:	418772..419374	Respiration	COG1845
WP_011376004.1_446	CUST_2413_PI442190940	MIT9312	HLII	WP_011376004.1_446	CTCCTCCAGTTGAAAATTGGGAAGGAGAGGCTCCATTAGTGGAAGAACCTTATGGTTATG	cyoB/coxA	Cytochrome c oxidase subunit I	WP_011376004.:	419381..421006	Respiration	COG0843
WP_011376004.1_446	CUST_2414_PI442190940	MIT9312	HLII	WP_011376004.1_446	ACTCCCCCTTGGATGAGATTGTTCTTTACTATCGCTACAGCATTTATTGCTGTTCCAACA	cyoB/coxA	Cytochrome c oxidase subunit I	WP_011376004.:	419381..421006	Respiration	COG0843
WP_011376004.1_446	CUST_2415_PI442190940	MIT9312	HLII	WP_011376004.1_446	TTTCAATTGGGTTGCGACATTATGGGGCGGCAAGATATCCATAAATAGTGCGATGTTATT	cyoB/coxA	Cytochrome c oxidase subunit I	WP_011376004.:	419381..421006	Respiration	COG0843
WP_011376004.1_446	CUST_2416_PI442190940	MIT9312	HLII	WP_011376004.1_446	TTACTGGAGTTGCTTTAGCACAGGTCCCTTTCGATATTCACGTGCATGATACCTATTTCG	cyoB/coxA	Cytochrome c oxidase subunit I	WP_011376004.:	419381..421006	Respiration	COG0843
WP_011376004.1_446	CUST_2417_PI442190940	MIT9312	HLII	WP_011376004.1_446	CATTGGCTCGGTTTAAATGGAATGCCAAGAAGAGTGGCAGAATATGATCCTCAATTCCAA	cyoB/coxA	Cytochrome c oxidase subunit I	WP_011376004.:	419381..421006	Respiration	COG0843
WP_011376004.1_446	CUST_2418_PI442190940	MIT9312	HLII	WP_011376004.1_446	GTATGAGAAACGGTCAAGTTTCTGGAGATAACCCTTGGAAAGCTCTAACACCTGAATGGT	cyoB/coxA	Cytochrome c oxidase subunit I	WP_011376004.:	419381..421006	Respiration	COG0843
WP_011376005.1_447	CUST_2419_PI442190940	MIT9312	HLII	WP_011376005.1_447	TTTGCGCAGAATTATGTGGCCCATATCATGGAGGAATGAGAGCCTCAATAATTGTTGAAG	ctaC/coxB	Cytochrome c oxidase subunit II	WP_011376005.:	421003..421806	Respiration	COG1622
WP_011376005.1_447	CUST_2420_PI442190940	MIT9312	HLII	WP_011376005.1_447	CGAATGGGAGGAATGAAGGAACTAAACCACAATCACGAAATGATGCGTTCAAATCCTGAA	ctaC/coxB	Cytochrome c oxidase subunit II	WP_011376005.:	421003..421806	Respiration	COG1622
WP_011376005.1_447	CUST_2421_PI442190940	MIT9312	HLII	WP_011376005.1_447	CATGCTTTCTGGGTGCCTGAGTTCAGAATTAAACAGGATATTATTCCTGGTCAACCAACT	ctaC/coxB	Cytochrome c oxidase subunit II	WP_011376005.:	421003..421806	Respiration	COG1622
WP_011376005.1_447	CUST_2422_PI442190940	MIT9312	HLII	WP_011376005.1_447	TCTTTTTGGATTGGATTTAATGTCAATTTGCTCCCAATTGAAGCAAGTATTAATGCACCA	ctaC/coxB	Cytochrome c oxidase subunit II	WP_011376005.:	421003..421806	Respiration	COG1622
WP_011376005.1_447	CUST_2423_PI442190940	MIT9312	HLII	WP_011376005.1_447	AGGAAAAGAAATGATCAGATAGGCGATGGTATAGCTTTAGAGGGAAACTTAAGCTTAGAA	ctaC/coxB	Cytochrome c oxidase subunit II	WP_011376005.:	421003..421806	Respiration	COG1622
WP_011376005.1_447	CUST_2424_PI442190940	MIT9312	HLII	WP_011376005.1_447	AGGAGAACTACACGTTCCTGTTGATCAAAAAGTATCGATGAAAATGGAATCTAAAGATGT	ctaC/coxB	Cytochrome c oxidase subunit II	WP_011376005.:	421003..421806	Respiration	COG1622
WP_011376013.1_455	CUST_2462_PI442190940	MIT9312	HLII	WP_011376013.1_455	TTGTTGGAATAATTGGTAATCCCGGTCAAGCAAATTATTCTGCAGCCAAAGCTGGAGTTA	gdh	3-oxoacyl-acyl-carrier-protein reductase	WP_011376013.:	428388..429137	Entner-Dudoroff	COG1028
WP_011376013.1_455	CUST_2463_PI442190940	MIT9312	HLII	WP_011376013.1_455	GCTAGCAGAGGAATTGGTAAAGAAATTGCTTTAGAATTAAGCCGCTTGGGAGCAGAAGTT	gdh	3-oxoacyl-acyl-carrier-protein reductase	WP_011376013.:	428388..429137	Entner-Dudoroff	COG1028
WP_011376013.1_455	CUST_2464_PI442190940	MIT9312	HLII	WP_011376013.1_455	ACTTGCGCCAAAGAATTTGCTTCAAGAGGTATAAACGTAAATGCAATAGCTCCAGGTTTC	gdh	3-oxoacyl-acyl-carrier-protein reductase	WP_011376013.:	428388..429137	Entner-Dudoroff	COG1028
WP_011376013.1_455	CUST_2465_PI442190940	MIT9312	HLII	WP_011376013.1_455	TGCTGGTATTACTAGAGATGGACTATTGATGAGAATGAAGTCGGAACAATGGGATGACGT	gdh	3-oxoacyl-acyl-carrier-protein reductase	WP_011376013.:	428388..429137	Entner-Dudoroff	COG1028
WP_011376013.1_455	CUST_2466_PI442190940	MIT9312	HLII	WP_011376013.1_455	TCAAGTAATGCAGGAAGTTACATTACAGGACAAACAATTAGTATTGACGGAGGTATGAGT	gdh	3-oxoacyl-acyl-carrier-protein reductase	WP_011376013.:	428388..429137	Entner-Dudoroff	COG1028
WP_011376013.1_455	CUST_2467_PI442190940	MIT9312	HLII	WP_011376013.1_455	TACTCTTCATCCGATGAAAAAGCTGAAGAAGTCATAAATTCAATAAAAAATTCGGGAGGT	gdh	3-oxoacyl-acyl-carrier-protein reductase	WP_011376013.:	428388..429137	Entner-Dudoroff	COG1028
WP_011376078.1_521	CUST_2822_PI442190940	MIT9312	HLII	WP_011376078.1_521	AATTAAGATTGCCGCAACGTGGGAAGGAATTAAAGCAGCTGAAATTTTGGAAAAAGAGGG	tal	Transaldolase	WP_011376078.:	485328..486329	Pentose Phosphate	COG0176
WP_011376078.1_521	CUST_2823_PI442190940	MIT9312	HLII	WP_011376078.1_521	ATTGAAAATTATTTCAGGGCGCGTATCGACAGAAGTTGACGCAAGACTTAGCTTTGACAC	tal	Transaldolase	WP_011376078.:	485328..486329	Pentose Phosphate	COG0176
WP_011376078.1_521	CUST_2824_PI442190940	MIT9312	HLII	WP_011376078.1_521	CCAAGCGGTAACTTGTGCGAATGCAAATATAACCTTAATTTCTCCGTTCGTTGGTCGTAT	tal	Transaldolase	WP_011376078.:	485328..486329	Pentose Phosphate	COG0176
WP_011376078.1_521	CUST_2825_PI442190940	MIT9312	HLII	WP_011376078.1_521	TTAGAAGATCAAATGGCCAGTGAGAAGCTTAGTGAAGGTATCACAGGATTCAGTAAGGCT	tal	Transaldolase	WP_011376078.:	485328..486329	Pentose Phosphate	COG0176
WP_011376078.1_521	CUST_2826_PI442190940	MIT9312	HLII	WP_011376078.1_521	AAACTGGTAAAACTAGTTTTATAGGTGCTGAAGACCCAGGTGTTATTTCGGTTACGCAGA	tal	Transaldolase	WP_011376078.:	485328..486329	Pentose Phosphate	COG0176
WP_011376078.1_521	CUST_2827_PI442190940	MIT9312	HLII	WP_011376078.1_521	AGAAGTAATGGGAGCTAGTTTTAGAAATCTTGATGAAATAAAAGAATTAGCAGGTTGCGA	tal	Transaldolase	WP_011376078.:	485328..486329	Pentose Phosphate	COG0176
WP_002805854.1_552	CUST_2998_PI442190940	MIT9312	HLII	WP_002805854.1_552	GGTGGTATTCACGTATGGCATATGCCTGCACTTTTAGCGATTTTTGGAGATGATTCTTGT	rbcL	Ribulose 1,5-bisphosphate carboxylase large subunit	WP_002805854.:	513733..515148	Carbon fixation	COG1850
WP_002805854.1_552	CUST_3002_PI442190940	MIT9312	HLII	WP_002805854.1_552	CGAAACTTGGAAGGAAATCAAGTTTGAGTTTGATACCGTCGACAAGCTTGACGTTCAAGG	rbcL	Ribulose 1,5-bisphosphate carboxylase large subunit	WP_002805854.:	513733..515148	Carbon fixation	COG1850
WP_011376108.1_553	CUST_3004_PI442190940	MIT9312	HLII	WP_011376108.1_553	ACACACAAAGCCAAGGAACAGCTTTTGTAGTTTTCCAAGGACGCTA	rbcS	Ribulose bisphosphate carboxylase small subunit	WP_011376108.:	515243..515584	Carbon fixation	COG4451
WP_011376108.1_553	CUST_3005_PI442190940	MIT9312	HLII	WP_011376108.1_553	AGAGCATACCCTGATCATCATGTAAGAATCATCGGTTACGATGCTTACACACAAAGCCAA	rbcS	Ribulose bisphosphate carboxylase small subunit	WP_011376108.:	515243..515584	Carbon fixation	COG4451
WP_011376108.1_553	CUST_3006_PI442190940	MIT9312	HLII	WP_011376108.1_553	TGGAAACTCCCATTCTTTGGGGAAAAAGATCTTAACTTGGTTGTAAGTGAATTAGAGGCA	rbcS	Ribulose bisphosphate carboxylase small subunit	WP_011376108.:	515243..515584	Carbon fixation	COG4451
WP_011376108.1_553	CUST_3007_PI442190940	MIT9312	HLII	WP_011376108.1_553	ACGATCAAATTGCATACATAATTGCTCAAGGTTGGAGTCCTGTCATTGAGCATGTTCACC	rbcS	Ribulose bisphosphate carboxylase small subunit	WP_011376108.:	515243..515584	Carbon fixation	COG4451
WP_011376108.1_553	CUST_3008_PI442190940	MIT9312	HLII	WP_011376108.1_553	AAACAGTCGCAACCCTGGAAACATTCGGTTTCTTACCACCGATGACCCAGGAAGAAATAT	rbcS	Ribulose bisphosphate carboxylase small subunit	WP_011376108.:	515243..515584	Carbon fixation	COG4451
WP_011376152.1_598	CUST_3245_PI442190940	MIT9312	HLII	WP_011376152.1_598	GTACTAATAATGATTTGCATGCCATAGTTGGAGCTGGGACAGGTTTAGGCATTGCAAGAG	glk	Glucokinase	WP_011376152.:	559922..560959	Glycolysis	COG0837
WP_011376152.1_598	CUST_3246_PI442190940	MIT9312	HLII	WP_011376152.1_598	TGTTAGTGGTATTGGTTTATCGAGAATTGCCGAATGGAGACTGAGCAAACCTGATGCTAA	glk	Glucokinase	WP_011376152.:	559922..560959	Glycolysis	COG0837
WP_011376152.1_598	CUST_3247_PI442190940	MIT9312	HLII	WP_011376152.1_598	TCAAGAATTATGCTTTGGAGGGTTATGGATTTCAGGAGGAACGGCACCAAAACATTTCAA	glk	Glucokinase	WP_011376152.:	559922..560959	Glycolysis	COG0837
WP_011376152.1_598	CUST_3248_PI442190940	MIT9312	HLII	WP_011376152.1_598	CCTCTGAAAGTGATTTTAGATGAAGAGTTTGGGCTTTTTAGTGCAGCCTGCAGAGCAAAA	glk	Glucokinase	WP_011376152.:	559922..560959	Glycolysis	COG0837
WP_011376152.1_598	CUST_3249_PI442190940	MIT9312	HLII	WP_011376152.1_598	AGTTTTAGCTAGCGAAGGTGGTCATGTTGAATACTCGCCAAAGTCAAAATTAGAATGGGA	glk	Glucokinase	WP_011376152.:	559922..560959	Glycolysis	COG0837
WP_011376152.1_598	CUST_3250_PI442190940	MIT9312	HLII	WP_011376152.1_598	CGAATAAGGGGGATCAGTTAATGATTGAAGTAGAGAGGATATGGTTAGGTGCTTATGCCT	glk	Glucokinase	WP_011376152.:	559922..560959	Glycolysis	COG0837
WP_011376332.1_784	CUST_4194_PI442190940	MIT9312	HLII	WP_011376332.1_784	TTGGACTAACTAAGGAATCGGCTGCAGAAAAACCTTATCTTGCCTCTATGGGGATTTACG	glgC	Glucose-1-phosphate adenylyltransferase	WP_011376332.:	725026..726321	Glycogen Metabolism	COG0448
WP_011376332.1_784	CUST_4195_PI442190940	MIT9312	HLII	WP_011376332.1_784	AAGGTGGTGGAACACCTCTTGGAGTAGGTGAAGGAACTACTGTAAAAAGAGCAATTCTTG	glgC	Glucose-1-phosphate adenylyltransferase	WP_011376332.:	725026..726321	Glycogen Metabolism	COG0448
WP_011376332.1_784	CUST_4196_PI442190940	MIT9312	HLII	WP_011376332.1_784	CCATTGGGGCATTCTTTGAGTCAAATTTGGCATTAACAGAGCAACCAAAACCTCCATTTA	glgC	Glucose-1-phosphate adenylyltransferase	WP_011376332.:	725026..726321	Glycogen Metabolism	COG0448
WP_011376332.1_784	CUST_4197_PI442190940	MIT9312	HLII	WP_011376332.1_784	TTCCTTCCTCCTTCTAAGCTTGTAGATGCCCAAATTACTGATTCAATAGTCTGTGAAGGT	glgC	Glucose-1-phosphate adenylyltransferase	WP_011376332.:	725026..726321	Glycogen Metabolism	COG0448
WP_011376332.1_784	CUST_4198_PI442190940	MIT9312	HLII	WP_011376332.1_784	TCCTGAAGCTCTTAAAAGGGGTGATACTCTTAAGAGTTATGTTTTCGACGATTATTGGGA	glgC	Glucose-1-phosphate adenylyltransferase	WP_011376332.:	725026..726321	Glycogen Metabolism	COG0448
WP_011376332.1_784	CUST_4199_PI442190940	MIT9312	HLII	WP_011376332.1_784	AGGATAGGTGATAACGTCGTTATTATTAATAAAGATCGAGTAGAAGAGGCAGATAAGCCA	glgC	Glucose-1-phosphate adenylyltransferase	WP_011376332.:	725026..726321	Glycogen Metabolism	COG0448
WP_011376333.1_785	CUST_4200_PI442190940	MIT9312	HLII	WP_011376333.1_785	CCAATAGACTTCCTCAGAATCTTGTTCAGGCAATGAGAGACTGTTTTGGCTCTCACACAT	edd	phosphogluconate dehydrogenase (NADP(+)-dependent, decarboxyla	WP_011376333.:	726416..727834	Pentose Phosphate/Entner-Dudorof	COG2084
WP_011376333.1_785	CUST_4201_PI442190940	MIT9312	HLII	WP_011376333.1_785	GCTAAAGTCGTTTCTTTATCCACAAAAGCTGGTATACCAGTCCCATGTCTATCCAGTACT	edd	phosphogluconate dehydrogenase (NADP(+)-dependent, decarboxyla	WP_011376333.:	726416..727834	Pentose Phosphate/Entner-Dudorof	COG2084
WP_011376333.1_785	CUST_4202_PI442190940	MIT9312	HLII	WP_011376333.1_785	GCTGTAGTCCTTGCCACAATAGCTAGCTATGCTCAAGGTATGGACATTTTAAGAGAAGCA	edd	phosphogluconate dehydrogenase (NADP(+)-dependent, decarboxyla	WP_011376333.:	726416..727834	Pentose Phosphate/Entner-Dudorof	COG2084
WP_011376333.1_785	CUST_4203_PI442190940	MIT9312	HLII	WP_011376333.1_785	ATGGACTGTTGTAAGTGCTTTAGAACTGGGGGTGTCAGTCCCAACTATTTATGCTTCTTT	edd	phosphogluconate dehydrogenase (NADP(+)-dependent, decarboxyla	WP_011376333.:	726416..727834	Pentose Phosphate/Entner-Dudorof	COG2084
WP_011376333.1_785	CUST_4204_PI442190940	MIT9312	HLII	WP_011376333.1_785	AACTGGTGATGATGTCGTGGAGAAAATATTAGATAAAGCTGGCCAGAAAGGTACTGGTTT	edd	phosphogluconate dehydrogenase (NADP(+)-dependent, decarboxyla	WP_011376333.:	726416..727834	Pentose Phosphate/Entner-Dudorof	COG2084
WP_011376333.1_785	CUST_4205_PI442190940	MIT9312	HLII	WP_011376333.1_785	TGCAGAAGCTTATGACCTTATGAAGAGAGTCAAAGGTATGAACGGTCAGCAAATGTCAGA	edd	phosphogluconate dehydrogenase (NADP(+)-dependent, decarboxyla	WP_011376333.:	726416..727834	Pentose Phosphate/Entner-Dudorof	COG2084
WP_011376344.1_796	CUST_4263_PI442190940	MIT9312	HLII	WP_011376344.1_796	AGCAAGTTTGTCTTGATAGATACAAGCAGTTCTGGTGCGAAGGTCAAGCAAGTAAGATCA	cbbA	Fructose-1,6-bisphosphate aldolase class II	WP_011376344.:	735100..736173	Glycolysis/Gluconeogenesis	COG0191
WP_011376344.1_796	CUST_4265_PI442190940	MIT9312	HLII	WP_011376344.1_796	CAGCATTTGCTGATAAGGCAAACTTTGACCCAAGACATTTCAACAAGCCTGCTAGAAAAT	cbbA	Fructose-1,6-bisphosphate aldolase class II	WP_011376344.:	735100..736173	Glycolysis/Gluconeogenesis	COG0191
WP_011376344.1_796	CUST_4266_PI442190940	MIT9312	HLII	WP_011376344.1_796	GTTAGGAAAGTCAACATTGATACCGATAACAGACTTGCTTTCACTGCCGCGGTTAGAGAG	cbbA	Fructose-1,6-bisphosphate aldolase class II	WP_011376344.:	735100..736173	Glycolysis/Gluconeogenesis	COG0191
WP_011376344.1_796	CUST_4267_PI442190940	MIT9312	HLII	WP_011376344.1_796	AGCACTTCCAAATACCCATCTTGTAATGCATGGATCTAGTTCAGTTCCTCAAGAATGGTT	cbbA	Fructose-1,6-bisphosphate aldolase class II	WP_011376344.:	735100..736173	Glycolysis/Gluconeogenesis	COG0191
WP_011376348.1_800	CUST_4285_PI442190940	MIT9312	HLII	WP_011376348.1_800	TAGAAGGTGATGGATACAATGTGGCATCCTATGCTGATTTACTTGTTGGTGTTGTTCCGA	prk	Phosphoribulokinase	WP_011376348.:	738787..739686	Carbon Fixation	COG3954
WP_011376348.1_800	CUST_4286_PI442190940	MIT9312	HLII	WP_011376348.1_800	AACCAGTATCGTTGTAAATGGTGGGAAAATGGGTTTCGCAATGGAACTTATTCTTACCCC	prk	Phosphoribulokinase	WP_011376348.:	738787..739686	Carbon Fixation	COG3954
WP_011376348.1_800	CUST_4287_PI442190940	MIT9312	HLII	WP_011376348.1_800	TGGGACATCCTTAGAACCAGGTCAATTTACTCCATGGGAAGATATTCCTAAGGGAACAGA	prk	Phosphoribulokinase	WP_011376348.:	738787..739686	Carbon Fixation	COG3954
WP_011376348.1_800	CUST_4288_PI442190940	MIT9312	HLII	WP_011376348.1_800	CACAGGTTTGAGAGAATGCCTATGAAAAAAGCTATGGCAGAAGCTCTTTCAAAGGGTGAA	prk	Phosphoribulokinase	WP_011376348.:	738787..739686	Carbon Fixation	COG3954
WP_011376348.1_800	CUST_4289_PI442190940	MIT9312	HLII	WP_011376348.1_800	TGGTGAAACTGGAGGAGGGAAGAAAAGATATTATCTCCATAGTCCTGAAGAAGCGGAAGA	prk	Phosphoribulokinase	WP_011376348.:	738787..739686	Carbon Fixation	COG3954
WP_011376348.1_800	CUST_4290_PI442190940	MIT9312	HLII	WP_011376348.1_800	AACGCAGAGAGAGGTTACTCAGCTGAAACCATTGTGGATACTATATTGAGAAGGATGCCT	prk	Phosphoribulokinase	WP_011376348.:	738787..739686	Carbon Fixation	COG3954
WP_011376424.1_876	CUST_4693_PI442190940	MIT9312	HLII	WP_011376424.1_876	TGGGTGCAGCAATGACTGGCTTAAGACCAATAGTAGAAGGAATGAATATGGGTTTTTTGC	pdhB	Pyruvate dehydrogenase E1 component beta subunit	WP_011376424.:	802953..803936	Glycolysis/Krebs cycle	COG0022
WP_011376424.1_876	CUST_4694_PI442190940	MIT9312	HLII	WP_011376424.1_876	TGCTGAGCACAGTCAAAGACTTGAAGCATATTTTCATGCAGTTCCTGGCATAAAGATTGT	pdhB	Pyruvate dehydrogenase E1 component beta subunit	WP_011376424.:	802953..803936	Glycolysis/Krebs cycle	COG0022
WP_011376424.1_876	CUST_4695_PI442190940	MIT9312	HLII	WP_011376424.1_876	CCTGAGGGTGATTATACTTGCGCTTTAGATCAGGCTGACGTTGTAAAAGAAGGGAAAGAT	pdhB	Pyruvate dehydrogenase E1 component beta subunit	WP_011376424.:	802953..803936	Glycolysis/Krebs cycle	COG0022
WP_011376424.1_876	CUST_4696_PI442190940	MIT9312	HLII	WP_011376424.1_876	GGTGCAGAATTAATTGCCTTGATAACAGAAGAGTGTTTTGATGATCTTGATGCCCGACCA	pdhB	Pyruvate dehydrogenase E1 component beta subunit	WP_011376424.:	802953..803936	Glycolysis/Krebs cycle	COG0022
WP_011376424.1_876	CUST_4697_PI442190940	MIT9312	HLII	WP_011376424.1_876	TATACTAGTGGCGGAAATTATAAAATACCAGCAGTAGTTCGAGGACCTGGAGGAGTTGGT	pdhB	Pyruvate dehydrogenase E1 component beta subunit	WP_011376424.:	802953..803936	Glycolysis/Krebs cycle	COG0022
WP_011376424.1_876	CUST_4698_PI442190940	MIT9312	HLII	WP_011376424.1_876	TGGCATGTAGTACACCCACAAATGCTAAAGGTTTAATGAAAGCAGCTATAAGAGATGAT	pdhB	Pyruvate dehydrogenase E1 component beta subunit	WP_011376424.:	802953..803936	Glycolysis/Krebs cycle	COG0022
WP_011376435.1_887	CUST_4751_PI442190940	MIT9312	HLII	WP_011376435.1_887	GTGCCGGCCAACACGAGCTTGGAATGAAATTTGATTCTTTAATAAAAGCTGCTGATAACG	glnA	Glutamine synthetase/ glutamate--ammonialigase	WP_011376435.:	811714..813135	Nitrogen Metabolism	COG0174
WP_011376435.1_887	CUST_4752_PI442190940	MIT9312	HLII	WP_011376435.1_887	ATGGAAGAGTGGACAGCCACTATTCTTTGGTGAAAGTGCCTATGCAAATTTATCCCAAAC	glnA	Glutamine synthetase/ glutamate--ammonialigase	WP_011376435.:	811714..813135	Nitrogen Metabolism	COG0174
WP_011376435.1_887	CUST_4753_PI442190940	MIT9312	HLII	WP_011376435.1_887	TTCTGAGGGTAATAGATCGGCTGCAGTAAGAATACCTTTAACTGGTCCAAGTCCAAAAGC	glnA	Glutamine synthetase/ glutamate--ammonialigase	WP_011376435.:	811714..813135	Nitrogen Metabolism	COG0174
WP_011376435.1_887	CUST_4754_PI442190940	MIT9312	HLII	WP_011376435.1_887	TGGGAAAACTGCAACATTTATGCCTAAGCCTGTATTTAACGATAATGGGACTGGAATGCA	glnA	Glutamine synthetase/ glutamate--ammonialigase	WP_011376435.:	811714..813135	Nitrogen Metabolism	COG0174
WP_011376435.1_887	CUST_4755_PI442190940	MIT9312	HLII	WP_011376435.1_887	TGCTCGCTGGTATTGATGGGATCAAAAATCAAATAGATCCTGGTGATGGTGTAGATGTTG	glnA	Glutamine synthetase/ glutamate--ammonialigase	WP_011376435.:	811714..813135	Nitrogen Metabolism	COG0174
WP_011376435.1_887	CUST_4756_PI442190940	MIT9312	HLII	WP_011376435.1_887	TGGTATATCGGAGGAATACTTAAACATGCCCCTTCATTCCTAGCGTTTACTAATCCAACA	glnA	Glutamine synthetase/ glutamate--ammonialigase	WP_011376435.:	811714..813135	Nitrogen Metabolism	COG0174
WP_011376443.1_895	CUST_4796_PI442190940	MIT9312	HLII	WP_011376443.1_895	AAATTTAGACCGCCAACTCCTATTTTGGCAACTACAACTGAAAGGAGTGTAGCGAGAAGA	pykF	Pyruvate kinase	WP_011376443.:	820354..822147	Glycolysis	COG0469
WP_011376443.1_895	CUST_4797_PI442190940	MIT9312	HLII	WP_011376443.1_895	AAAGCTATTGAGAGCCACTTACCTAGCACGATACCAAATGCTATTAGTGGAGCTGTAAGT	pykF	Pyruvate kinase	WP_011376443.:	820354..822147	Glycolysis	COG0469
WP_011376443.1_895	CUST_4798_PI442190940	MIT9312	HLII	WP_011376443.1_895	ACAAGGAGATTTAGTCGTTCAAACTGCAGGTACACTAACTGGTATAAGTGGCTCTACAGA	pykF	Pyruvate kinase	WP_011376443.:	820354..822147	Glycolysis	COG0469
WP_011376443.1_895	CUST_4799_PI442190940	MIT9312	HLII	WP_011376443.1_895	AGCTAGACAACTAGATGCAGGAGCAATAATTCCTTTAACAAAATCAGGTTCTACTGCTCG	pykF	Pyruvate kinase	WP_011376443.:	820354..822147	Glycolysis	COG0469
WP_011376443.1_895	CUST_4800_PI442190940	MIT9312	HLII	WP_011376443.1_895	CAACTTGTTTGGGGAGTCACCCCGATAGTAGTAAATAATGATGAAAGAACAGCAAAAACA	pykF	Pyruvate kinase	WP_011376443.:	820354..822147	Glycolysis	COG0469
WP_011376443.1_895	CUST_4801_PI442190940	MIT9312	HLII	WP_011376443.1_895	AGGAAGATACTTTCAAGAGGGATCTCAATAGGTGAAATTGGAGTTACAGGTAAAGCAAGA	pykF	Pyruvate kinase	WP_011376443.:	820354..822147	Glycolysis	COG0469
WP_011376464.1_917	CUST_4915_PI442190940	MIT9312	HLII	WP_011376464.1_917	AAATGCCTATGATCAACCTGGAGTTGAAGCCGGAAAGAAAGCAGCTGCAAATATTATTGA	pgi	Glucose-6-phosphate isomerase	WP_011376464.:	841041..842624	Glycolysis/Gluconeogenesis	COG0166
WP_011376464.1_917	CUST_4916_PI442190940	MIT9312	HLII	WP_011376464.1_917	GGTGCATCATTAATGGATGAGGCCACACGTACTAGTGATTTTAAAAATAATCCCGCAGCA	pgi	Glucose-6-phosphate isomerase	WP_011376464.:	841041..842624	Glycolysis/Gluconeogenesis	COG0166
WP_011376464.1_917	CUST_4917_PI442190940	MIT9312	HLII	WP_011376464.1_917	TCATCTGCATGGTATTTAACTGGAGATGGCGTTGGAAAGAGAGATATGGTTGTTTTACCT	pgi	Glucose-6-phosphate isomerase	WP_011376464.:	841041..842624	Glycolysis/Gluconeogenesis	COG0166
WP_011376464.1_917	CUST_4918_PI442190940	MIT9312	HLII	WP_011376464.1_917	AGGGATATCCGTTTTGGTAATAAAGGATCTACAGATCAACATGCTTATGTTCAGCAACT	pgi	Glucose-6-phosphate isomerase	WP_011376464.:	841041..842624	Glycolysis/Gluconeogenesis	COG0166
WP_011376464.1_917	CUST_4919_PI442190940	MIT9312	HLII	WP_011376464.1_917	ACTTCGTGAAATGTGTTTCGGTAATGAAGATTATTTAGTTAAGGGCGATTGGTCAAATCC	pgi	Glucose-6-phosphate isomerase	WP_011376464.:	841041..842624	Glycolysis/Gluconeogenesis	COG0166
WP_011376464.1_917	CUST_4920_PI442190940	MIT9312	HLII	WP_011376464.1_917	TGTTTTTCACTAGGTGCCTTAATCGCTTTATTTGAGAGATCTGTATCTTTCTACGCTGAA	pgi	Glucose-6-phosphate isomerase	WP_011376464.:	841041..842624	Glycolysis/Gluconeogenesis	COG0166
WP_011376605.1_1072	CUST_5653_PI442190940	MIT9312	HLII	WP_011376605.1_1072	AAAAGTTGATTCTTATGGATGTGGCGACTCTTTCGCTGCTGGTATTCTTTATGGAATGGC	rbsK	Sugar kinase, ribokinase family	WP_011376605.:	966202..967071	Pentose Phosphate	COG0524
WP_011376605.1_1072	CUST_5654_PI442190940	MIT9312	HLII	WP_011376605.1_1072	CCAGCAGGTGGAGGCACTATTATCGCGAAAACACTTTCTGAGTTGACTTTAAACCAAATT	rbsK	Sugar kinase, ribokinase family	WP_011376605.:	966202..967071	Pentose Phosphate	COG0524
WP_011376605.1_1072	CUST_5655_PI442190940	MIT9312	HLII	WP_011376605.1_1072	TATCGGTAAGGCCCAAATATACTATTAAAACTGAGGGGGATAAGGGGGGCATGATTTTCC	rbsK	Sugar kinase, ribokinase family	WP_011376605.:	966202..967071	Pentose Phosphate	COG0524
WP_011376605.1_1072	CUST_5656_PI442190940	MIT9312	HLII	WP_011376605.1_1072	TCAGAGATTTTTAAAATGGCTAGATCTGCTTCTATACTTGTACCACACCAAGGGTGGGA	rbsK	Sugar kinase, ribokinase family	WP_011376605.:	966202..967071	Pentose Phosphate	COG0524
WP_011376605.1_1072	CUST_5657_PI442190940	MIT9312	HLII	WP_011376605.1_1072	TGCTTAAATCTTGCTAAAATAATAGGAAGAGACGCTAGTGAATTTTTCGGCCCATATGCA	rbsK	Sugar kinase, ribokinase family	WP_011376605.:	966202..967071	Pentose Phosphate	COG0524
WP_011376605.1_1072	CUST_5658_PI442190940	MIT9312	HLII	WP_011376605.1_1072	AGGTTAGCACCCAATTATAAGGACAACTTAGACTGGAGCATTTTAAAAAAAATGGACGGA	rbsK	Sugar kinase, ribokinase family	WP_011376605.:	966202..967071	Pentose Phosphate	COG0524
WP_011376634.1_1101	CUST_5816_PI442190940	MIT9312	HLII	WP_011376634.1_1101	TGGATGTGAATCAGTGGAATGTATGAGACTTGAAGCAGGGGGAATGGCTAATATGGAATT	opcA	Glucose-6-P dehydrogenase subunit opcA	WP_011376634.:	994703..996004	Pentose Phosphate	COG3429
WP_011376634.1_1101	CUST_5817_PI442190940	MIT9312	HLII	WP_011376634.1_1101	TTAGGTTGAAAAATTGGAGGGAATCATTGGCAATGATTTTTGATCCCCCATCGAGGAGAC	opcA	Glucose-6-P dehydrogenase subunit opcA	WP_011376634.:	994703..996004	Pentose Phosphate	COG3429
WP_011376634.1_1101	CUST_5818_PI442190940	MIT9312	HLII	WP_011376634.1_1101	TTATCTATTGATCAATTACCTTCTTGGTTGTGGTGGAATGGGAGCTTGGATGAATCGCCT	opcA	Glucose-6-P dehydrogenase subunit opcA	WP_011376634.:	994703..996004	Pentose Phosphate	COG3429
WP_011376634.1_1101	CUST_5819_PI442190940	MIT9312	HLII	WP_011376634.1_1101	CGCAGGAGATCATATGATTCAAGCTTGTTTTTGATCTCATGGATTAGTGATAAACTTGG	opcA	Glucose-6-P dehydrogenase subunit opcA	WP_011376634.:	994703..996004	Pentose Phosphate	COG3429
WP_011376634.1_1101	CUST_5820_PI442190940	MIT9312	HLII	WP_011376634.1_1101	ACTGCGCTTGGATCGCCTAACAGATGTTTAAAAGTTTTAGATCAACTAAAGAAATCAAAT	opcA	Glucose-6-P dehydrogenase subunit opcA	WP_011376634.:	994703..996004	Pentose Phosphate	COG3429
WP_011376634.1_1101	CUST_5821_PI442190940	MIT9312	HLII	WP_011376634.1_1101	TCTTTGGGAAATCCAAGTATTCATTTAGGACAAGTTATTGGATTGAGGTTGATTTCAAGA	opcA	Glucose-6-P dehydrogenase subunit opcA	WP_011376634.:	994703..996004	Pentose Phosphate	COG3429
WP_011376635.1_1102	CUST_5822_PI442190940	MIT9312	HLII	WP_011376635.1_1102	ATATGCTTCAAAATCATATGACTCAAATGCTCGCTGTTACTGCTATGGAACCTCCTGGGA	zwf	glucose-6-phosphate dehydrogenase zwf	WP_011376635.:	996007..997530	Pentose Phosphate	COG0364
WP_011376635.1_1102	CUST_5823_PI442190940	MIT9312	HLII	WP_011376635.1_1102	AGAAACTTATATTGCGACAAAAGTTTTCGTTGATAACTGGCGTTGGCAAGGGGTACCTTT	zwf	glucose-6-phosphate dehydrogenase zwf	WP_011376635.:	996007..997530	Pentose Phosphate	COG0364
WP_011376635.1_1102	CUST_5824_PI442190940	MIT9312	HLII	WP_011376635.1_1102	TGCTACTTTTAACTTTGAGGTAAAATCCCCTGGTTCTGGGATGAAATCCAGACCTGTTGA	zwf	glucose-6-phosphate dehydrogenase zwf	WP_011376635.:	996007..997530	Pentose Phosphate	COG0364
WP_011376635.1_1102	CUST_5825_PI442190940	MIT9312	HLII	WP_011376635.1_1102	TGACGTGGGGACCTCCTGAGTCTGATCAATTACTTTCAAAAGATAATATTTTCTGGCGCA	zwf	glucose-6-phosphate dehydrogenase zwf	WP_011376635.:	996007..997530	Pentose Phosphate	COG0364
WP_011376635.1_1102	CUST_5826_PI442190940	MIT9312	HLII	WP_011376635.1_1102	GATGCAATGCTTTCTGATCCAACCTTATTTACACGAAGTGATGAAGTAGAGGCAGCTTGG	zwf	glucose-6-phosphate dehydrogenase zwf	WP_011376635.:	996007..997530	Pentose Phosphate	COG0364
WP_011376635.1_1102	CUST_5827_PI442190940	MIT9312	HLII	WP_011376635.1_1102	AAACTTGCTGACGAAAATGAACCGTGGAATTGTTGTATAAGAGGTCAATATGGGGAGGGA	zwf	glucose-6-phosphate dehydrogenase zwf	WP_011376635.:	996007..997530	Pentose Phosphate	COG0364
WP_011376857.1_1340	CUST_7032_PI442190940	MIT9312	HLII	WP_011376857.1_1340	ACTCCATAAGTGCTTTATGTGAAGCGACTGGAGCTGATATCAGAGAAGTTTCTAGAGCGA	ugd	UDP-glucose 6-dehydrogenase/nucleotide sugar dehydrogenase	WP_011376857.:	1221700..1223124	Amino sugar and nucleotide sugar r	COG1004
WP_011376857.1_1340	CUST_7033_PI442190940	MIT9312	HLII	WP_011376857.1_1340	AATTAATTGGGCTTCCGCAGCATTTTTGATGAGAAAACCTGCCTGGATTTTTGACGTAAG	ugd	UDP-glucose 6-dehydrogenase/nucleotide sugar dehydrogenase	WP_011376857.:	1221700..1223124	Amino sugar and nucleotide sugar r	COG1004
WP_011376857.1_1340	CUST_7034_PI442190940	MIT9312	HLII	WP_011376857.1_1340	GGATCTGATAGCAGAATTGGATCCAAATTTCTAGAATCTGGACCAGGTTTTGGTGGAAGT	ugd	UDP-glucose 6-dehydrogenase/nucleotide sugar dehydrogenase	WP_011376857.:	1221700..1223124	Amino sugar and nucleotide sugar r	COG1004
WP_011376857.1_1340	CUST_7035_PI442190940	MIT9312	HLII	WP_011376857.1_1340	AGATTTGATTGAAGAAGGCGCAGAGCTTTTTATTCATGACCCTAAAGTTACCCCTAAACA	ugd	UDP-glucose 6-dehydrogenase/nucleotide sugar dehydrogenase	WP_011376857.:	1221700..1223124	Amino sugar and nucleotide sugar r	COG1004
WP_011376857.1_1340	CUST_7036_PI442190940	MIT9312	HLII	WP_011376857.1_1340	AGTATATTTATCGAACTACTTCGGACTACCTGAAGTTGCAAGATTTTGGAGGAGGTTGT	ugd	UDP-glucose 6-dehydrogenase/nucleotide sugar dehydrogenase	WP_011376857.:	1221700..1223124	Amino sugar and nucleotide sugar r	COG1004
WP_011376857.1_1340	CUST_7037_PI442190940	MIT9312	HLII	WP_011376857.1_1340	TGGAGTAGTGAATTAGCTAAATTAACTGCGAACGCTTTTTAGCTCAACGAATAAGTTCT	ugd	UDP-glucose 6-dehydrogenase/nucleotide sugar dehydrogenase	WP_011376857.:	1221700..1223124	Amino sugar and nucleotide sugar r	COG1004
WP_011376862.1_1345	CUST_7062_PI442190940	MIT9312	HLII	WP_011376862.1_1345	AATTCTTGGAGGATTATCTTATTGCGATACTTTAATCCAATAGGCTCACACCCTAGCGGA	galE	UDP-glucose 4-epimerase	WP_011376862.:	1228713..1229771	Galactose metabolism	COG1087
WP_011376862.1_1345	CUST_7063_PI442190940	MIT9312	HLII	WP_011376862.1_1345	TGAACAACCTAGGAAAGGAGATGTAGGGGTATGTTTTGCTGATAACACTAAGGCAAAAAA	galE	UDP-glucose 4-epimerase	WP_011376862.:	1228713..1229771	Galactose metabolism	COG1087
WP_011376862.1_1345	CUST_7064_PI442190940	MIT9312	HLII	WP_011376862.1_1345	TGTGAAGTTGCGATTAGGGAGAACAATGCGTTTAAAATTTATGGTAATGATTGGCCTACA	galE	UDP-glucose 4-epimerase	WP_011376862.:	1228713..1229771	Galactose metabolism	COG1087
WP_011376862.1_1345	CUST_7065_PI442190940	MIT9312	HLII	WP_011376862.1_1345	TCTGTCTCATGATCCTGAAATAAATGCTTTTAATTTAGGGACTGGTAAAGGTACAAGTGT	galE	UDP-glucose 4-epimerase	WP_011376862.:	1228713..1229771	Galactose metabolism	COG1087
WP_011376862.1_1345	CUST_7066_PI442190940	MIT9312	HLII	WP_011376862.1_1345	TCCCAATGGAATATTGGGAAACAAACCTCTTAGGAACAATTAATTTGATTAGGGTAATGA	galE	UDP-glucose 4-epimerase	WP_011376862.:	1228713..1229771	Galactose metabolism	COG1087
WP_011376862.1_1345	CUST_7067_PI442190940	MIT9312	HLII	WP_011376862.1_1345	TTATGAATTGGTACCCAAAAAAGTCAATAAATGAAATGTGTAAAGATGGATGGAGATGGT	galE	UDP-glucose 4-epimerase	WP_011376862.:	1228713..1229771	Galactose metabolism	COG1087
WP_011376921.1_1403	CUST_7389_PI442190940	MIT9312	HLII	WP_011376921.1_1403	TTCCACTTGAAGCTGCATTATCAGATGCGATTGATCTTACTGGACCAATCAAACCTCTCT		polysaccharide biosynthesis/export family protein	WP_011376921.:	1288756..1290003	Other sugar transporters	COG1596
WP_011376921.1_1403	CUST_7390_PI442190940	MIT9312	HLII	WP_011376921.1_1403	TCGGGTATTTCTCCAAGGTTTATTAGCGTAAGTCTTTTTGGTAGAGTCGAAAGTCCTGGA		polysaccharide biosynthesis/export family protein	WP_011376921.:	1288756..1290003	Other sugar transporters	COG1596
WP_011376921.1_1403	CUST_7391_PI442190940	MIT9312	HLII	WP_011376921.1_1403	TGTACCTCTTGGAAAAGGTGGAGGGAAAAAGAGAGCAATTGTTGATTTTAATGCCTTTGT		polysaccharide biosynthesis/export family protein	WP_011376921.:	1288756..1290003	Other sugar transporters	COG1596
WP_011376921.1_1403	CUST_7392_PI442190940	MIT9312	HLII	WP_011376921.1_1403	ACCGCTTTTCTTTAAAGAAGTTAGCTCCATTTGTGGGTATTTATTCAACTAAACAGACA		polysaccharide biosynthesis/export family protein	WP_011376921.:	1288756..1290003	Other sugar transporters	COG1596
WP_011376921.1_1403	CUST_7393_PI442190940	MIT9312	HLII	WP_011376921.1_1403	AGCAAGATGGGACAGTAATAAAGAAAAATATTTCATATTCAGCAAGTGCTAAAAGAGGCT		polysaccharide biosynthesis/export family protein	WP_011376921.:	1288756..1290003	Other sugar transporters	COG1596
WP_011376921.1_1403	CUST_7394_PI442190940	MIT9312	HLII	WP_011376921.1_1403	TCCATTTCTGATGTTATTAGAAATGCTGGTGGTATAACATCCTTAACTGATTTATCTCGA		polysaccharide biosynthesis/export family protein	WP_011376921.:	1288756..1290003	Other sugar transporters	COG1596
WP_011376930.1_1412	CUST_7443_PI442190940	MIT9312	HLII	WP_011376930.1_1412	GTAGTGATTCTGTAACTGCAGCATTCTTTGGAGATGGGACTTGCAATAATGGGCAGTTTT	pdhA	Pyruvate dehydrogenase E1 component alpha subunit			Glycolysis/Krebs cycle	COG1071
WP_011376930.1_1412	CUST_7444_PI442190940	MIT9312	HLII	WP_011376930.1_1412	GGGAGAAGGGCCCACACTTTTAGAATGCTTGACTTATAGATACAGAGGACATTCTCTTGC	pdhA	Pyruvate dehydrogenase E1 component alpha subunit			Glycolysis/Krebs cycle	COG1071
WP_011376930.1_1412	CUST_7445_PI442190940	MIT9312	HLII	WP_011376930.1_1412	CGAGGGGGGTCCATGCACTTATTTTCAAAAGAGCATCACTTACTAGGAGGATATGCATTT	pdhA	Pyruvate dehydrogenase E1 component alpha subunit			Glycolysis/Krebs cycle	COG1071
WP_011376930.1_1412	CUST_7446_PI442190940	MIT9312	HLII	WP_011376930.1_1412	TAATAAATGGGCTATTGGTATGGCTCACGATAGAGCCACTAGCAATCCTGAAATCTGGAG	pdhA	Pyruvate dehydrogenase E1 component alpha subunit			Glycolysis/Krebs cycle	COG1071
WP_011376930.1_1412	CUST_7447_PI442190940	MIT9312	HLII	WP_011376930.1_1412	[illegible]	pdhA	Pyruvate dehydrogenase E1 component alpha subunit			Glycolysis/Krebs cycle	COG1071
WP_011376930.1_1412	CUST_7448_PI442190940	MIT9312	HLII	WP_011376930.1_1412	[illegible]	pdhA	Pyruvate dehydrogenase E1 component alpha subunit			Glycolysis/Krebs cycle	COG1071
WP_011376951.1_1433	CUST_7566_PI442190940	MIT9312	HLII	WP_011376951.1_1433	[illegible]	ftsZ	cell division protein FtsZ	WP_011376951.:	1321567..1322682	Cell Division	COG0206
WP_011376951.1_1433	CUST_7567_PI442190940	MIT9312	HLII	WP_011376951.1_1433	[illegible]	ftsZ	cell division protein FtsZ	WP_011376951.:	1321567..1322682	Cell Division	COG0206
WP_011376951.1_1433	CUST_7568_PI442190940	MIT9312	HLII	WP_011376951.1_1433	[illegible]	ftsZ	cell division protein FtsZ	WP_011376951.:	1321567..1322682	Cell Division	COG0206
WP_011376951.1_1433	CUST_7569_PI442190940	MIT9312	HLII	WP_011376951.1_1433	[illegible]	ftsZ	cell division protein FtsZ	WP_011376951.:	1321567..1322682	Cell Division	COG0206
WP_011376951.1_1433	CUST_7570_PI442190940	MIT9312	HLII	WP_011376951.1_1433	[illegible]	ftsZ	cell division protein FtsZ	WP_011376951.:	1321567..1322682	Cell Division	COG0206
WP_011376951.1_1433	CUST_7571_PI442190940	MIT9312	HLII	WP_011376951.1_1433	[illegible]	ftsZ	cell division protein FtsZ	WP_011376951.:	1321567..1322682	Cell Division	COG0206
WP_011376965.1_1449	CUST_7645_PI442190940	MIT9312	HLII	WP_011376965.1_1449	[illegible]	glcH	GPH family sugar transporter	WP_011376965.:	1334978..1336324	GlcH sugar transporter	COG2211
WP_011376965.1_1449	CUST_7646_PI442190940	MIT9312	HLII	WP_011376965.1_1449	[illegible]	glcH	GPH family sugar transporter	WP_011376965.:	1334978..1336324	GlcH sugar transporter	COG2211
WP_011376965.1_1449	CUST_7647_PI442190940	MIT9312	HLII	WP_011376965.1_1449	[illegible]	glcH	GPH family sugar transporter	WP_011376965.:	1334978..1336324	GlcH sugar transporter	COG2211
WP_011376965.1_1449	CUST_7648_PI442190940	MIT9312	HLII	WP_011376965.1_1449	[illegible]	glcH	GPH family sugar transporter	WP_011376965.:	1334978..1336324	GlcH sugar transporter	COG2211
WP_011376965.1_1449	CUST_7649_PI442190940	MIT9312	HLII	WP_011376965.1_1449	[illegible]	glcH	GPH family sugar transporter	WP_011376965.:	1334978..1336324	GlcH sugar transporter	COG2211
WP_011376965.1_1449	CUST_7650_PI442190940	MIT9312	HLII	WP_011376965.1_1449	[illegible]	glcH	GPH family sugar transporter	WP_011376965.:	1334978..1336324	GlcH sugar transporter	COG2211
WP_036924033.1_1468	CUST_7757_PI442190940	MIT9312	HLII	WP_036924033.1_1468	[illegible]	kaiC	circadian clock protein KaiC	WP_036924033.:	1354118..1355647	Circadian Rhythm	COG0467
WP_036924033.1_1468	CUST_7758_PI442190940	MIT9312	HLII	WP_036924033.1_1468	[illegible]	kaiC	circadian clock protein KaiC	WP_036924033.:	1354118..1355647	Circadian Rhythm	COG0467
WP_036924033.1_1468	CUST_7759_PI442190940	MIT9312	HLII	WP_036924033.1_1468	[illegible]	kaiC	circadian clock protein KaiC	WP_036924033.:	1354118..1355647	Circadian Rhythm	COG0467
WP_036924033.1_1468	CUST_7760_PI442190940	MIT9312	HLII	WP_036924033.1_1468	[illegible]	kaiC	circadian clock protein KaiC	WP_036924033.:	1354118..1355647	Circadian Rhythm	COG0467
WP_036924033.1_1468	CUST_7761_PI442190940	MIT9312	HLII	WP_036924033.1_1468	[illegible]	kaiC	circadian clock protein KaiC	WP_036924033.:	1354118..1355647	Circadian Rhythm	COG0467
WP_036924033.1_1468	CUST_7762_PI442190940	MIT9312	HLII	WP_036924033.1_1468	[illegible]	kaiC	circadian clock protein KaiC	WP_036924033.:	1354118..1355647	Circadian Rhythm	COG0467
WP_011376985.1_1469	CUST_7763_PI442190940	MIT9312	HLII	WP_011376985.1_1469	[illegible]	kaiB	Circadian clock protein KaiB	WP_011376985.:	1355720..1356037	Circadian Rhythm	COG4251
WP_011376985.1_1469	CUST_7764_PI442190940	MIT9312	HLII	WP_011376985.1_1469	[illegible]	kaiB	Circadian clock protein KaiB	WP_011376985.:	1355720..1356037	Circadian Rhythm	COG4251
WP_011376985.1_1469	CUST_7765_PI442190940	MIT9312	HLII	WP_011376985.1_1469	[illegible]	kaiB	Circadian clock protein KaiB	WP_011376985.:	1355720..1356037	Circadian Rhythm	COG4251
WP_011376985.1_1469	CUST_7766_PI442190940	MIT9312	HLII	WP_011376985.1_1469	[illegible]	kaiB	Circadian clock protein KaiB	WP_011376985.:	1355720..1356037	Circadian Rhythm	COG4251
WP_011376985.1_1469	CUST_7767_PI442190940	MIT9312	HLII	WP_011376985.1_1469	[illegible]	kaiB	Circadian clock protein KaiB	WP_011376985.:	1355720..1356037	Circadian Rhythm	COG4251
WP_011377075.1_1562	CUST_8192_PI442190940	MIT9312	HLII	WP_011377075.1_1562	[illegible]	atpC	ATP synthase epsilon subunit	WP_011377075.:	1429514..1429918	Energy Metabolism	COG0355
WP_011377075.1_1562	CUST_8193_PI442190940	MIT9312	HLII	WP_011377075.1_1562	[illegible]	atpC	ATP synthase epsilon subunit	WP_011377075.:	1429514..1429918	Energy Metabolism	COG0355

WP_011377075.1_1562	CUST_8194_PI442190940	MIT9312	HLII	WP_011377075.1_1562	CCCAGTGGCAATCAATAGCTTTAATGGGTGGCTTCGCTGAAATTGAATCAGATGAAGTAA	atpC	ATP synthase epsilon subunit	WP_011377075.:	1429514..1429918	Energy Metabolism	COG0355
WP_011377075.1_1562	CUST_8195_PI442190940	MIT9312	HLII	WP_011377075.1_1562	ACGCAGAAATTGGATCTGATATTGATGTTCAAAATGCTGAACAAGATCTCAAAGAAGCAA	atpC	ATP synthase epsilon subunit	WP_011377075.:	1429514..1429918	Energy Metabolism	COG0355
WP_011377075.1_1562	CUST_8196_PI442190940	MIT9312	HLII	WP_011377075.1_1562	CCTGGACATATTTCTTTAGTTACAGCTATTGATATTGGCGTTTTAAGGCTAAGAATGAAT	atpC	ATP synthase epsilon subunit	WP_011377075.:	1429514..1429918	Energy Metabolism	COG0355
WP_011377075.1_1562	CUST_8197_PI442190940	MIT9312	HLII	WP_011377075.1_1562	ATCTCAAAGAAGCAAAATTAGTAATGACTAAATTTCCTGAAAATG	atpC	ATP synthase epsilon subunit	WP_011377075.:	1429514..1429918	Energy Metabolism	COG0355
WP_011377087.1_1573	CUST_8252_PI442190940	MIT9312	HLII	WP_011377087.1_1573	AGGTGTTAAAGGTCTTATTGACGAAGTTCCCGTGGAAGACGTAACTAAATTTGCTGCTGA	atpA	ATP synthase alpha subunit	WP_011377087.:	1439212..1440729	Energy Metabolism	COG0056
WP_011377087.1_1573	CUST_8253_PI442190940	MIT9312	HLII	WP_011377087.1_1573	TTGAAAGAGCAGCAAAATTATCTGATGATATGGGCGGTGGTTCAATGACAGCATTACCAA	atpA	ATP synthase alpha subunit	WP_011377087.:	1439212..1440729	Energy Metabolism	COG0056
WP_011377087.1_1573	CUST_8254_PI442190940	MIT9312	HLII	WP_011377087.1_1573	AGTTAGTAGAGTTGGAGGAGCTGCTCAAACAAAGGCAATTAAGAAAATTGCTGGAACACT	atpA	ATP synthase alpha subunit	WP_011377087.:	1439212..1440729	Energy Metabolism	COG0056
WP_011377087.1_1573	CUST_8255_PI442190940	MIT9312	HLII	WP_011377087.1_1573	AGTTGCTAAAACAAGCACAATTCTCACCATTAAATCTCGCAGAACAAGTTGCAGTTGTGT	atpA	ATP synthase alpha subunit	WP_011377087.:	1439212..1440729	Energy Metabolism	COG0056
WP_011377087.1_1573	CUST_8256_PI442190940	MIT9312	HLII	WP_011377087.1_1573	GCAAGCTCAAGCTTATAGACAAATGTCTCTCCTTTTAAGAAGACCACCAGGAAGAGAAGC	atpA	ATP synthase alpha subunit	WP_011377087.:	1439212..1440729	Energy Metabolism	COG0056
WP_011377087.1_1573	CUST_8257_PI442190940	MIT9312	HLII	WP_011377087.1_1573	TGGCTTAGAAGCAACACTCAAAGAGGTCATAAAAGAAGTCAAATCTTCAATGCTTGCCAC	atpA	ATP synthase alpha subunit	WP_011377087.:	1439212..1440729	Energy Metabolism	COG0056
WP_011377091.1_1578	CUST_8279_PI442190940	MIT9312	HLII	WP_011377091.1_1578	GTGGAGAATTAGGAGCGCCTACTGCAGATATCAATACCACTATAGCCTTGGCTTTATTGG	atpI	ATP synthase subunit A	WP_011377091.:	1442752..1443477	Energy Metabolism	COG0356
WP_011377091.1_1578	CUST_8280_PI442190940	MIT9312	HLII	WP_011377091.1_1578	GCAGGGTTAAGCAATAAGGGTTGGAGATACTTCGAATACTATGTTCACCCAACTCCGATT	atpI	ATP synthase subunit A	WP_011377091.:	1442752..1443477	Energy Metabolism	COG0356
WP_011377091.1_1578	CUST_8281_PI442190940	MIT9312	HLII	WP_011377091.1_1578	GCATTGATTTTCGCAACTTTAGCGGCCTACTACATTGGAGAAGCTGTTGAAGAACATCAT	atpI	ATP synthase subunit A	WP_011377091.:	1442752..1443477	Energy Metabolism	COG0356
WP_011377091.1_1578	CUST_8282_PI442190940	MIT9312	HLII	WP_011377091.1_1578	AAGGCCTTCAAAACTTGCTTGAGTTTCTCTGGGATTACATAAGAGATCTTGCTAGGACGC	atpI	ATP synthase subunit A	WP_011377091.:	1442752..1443477	Energy Metabolism	COG0356
WP_011377091.1_1578	CUST_8283_PI442190940	MIT9312	HLII	WP_011377091.1_1578	GTTGGTGTTCTAGTCTTTTTAGTTCCGCTCATTCTCCCAATACCTGTTATGTTCCTAGGA	atpI	ATP synthase subunit A	WP_011377091.:	1442752..1443477	Energy Metabolism	COG0356
WP_011377091.1_1578	CUST_8284_PI442190940	MIT9312	HLII	WP_011377091.1_1578	AGCATTAGAAGTTGGTCAACATCTTTATTGGCAAATTGGAAATATCAGACTTCATGGGCA	atpI	ATP synthase subunit A	WP_011377091.:	1442752..1443477	Energy Metabolism	COG0356
WP_011377101.1_1588	CUST_8335_PI442190940	MIT9312	HLII	WP_011377101.1_1588	ATGGTTTGCACTCAGATAATGGGTCTTGAATATGCAGTTTCAATGGCAAATTCTAGCGGT	fumC	class II fumarate hydratase	WP_011377101.:	1451132..1452517	Krebs cycle and related genes	COG0114
WP_011377101.1_1588	CUST_8336_PI442190940	MIT9312	HLII	WP_011377101.1_1588	TTCTTACCTCTGGGAGGAACTGCAGTTGGTACAGGGATTAATTGTCCAAAAGGATTTTGT	fumC	class II fumarate hydratase	WP_011377101.:	1451132..1452517	Krebs cycle and related genes	COG0114
WP_011377101.1_1588	CUST_8337_PI442190940	MIT9312	HLII	WP_011377101.1_1588	TTGGTTACAGCCATAGTGCCAAAAGTTGGTTATGAAAAAGCAGCTGAAATTGCAAACCTT	fumC	class II fumarate hydratase	WP_011377101.:	1451132..1452517	Krebs cycle and related genes	COG0114
WP_011377101.1_1588	CUST_8338_PI442190940	MIT9312	HLII	WP_011377101.1_1588	TGGCCTCGCATGATCGTCTAGCTCAAGTAATGAGTCAGATAAAAATATTAGCAGGTGCAT	fumC	class II fumarate hydratase	WP_011377101.:	1451132..1452517	Krebs cycle and related genes	COG0114
WP_011377101.1_1588	CUST_8339_PI442190940	MIT9312	HLII	WP_011377101.1_1588	TTAATTATCCCTCAAAATGAACCTGGAAGTTCTATCATGCCGGGCAAAGTGAATCCGACT	fumC	class II fumarate hydratase	WP_011377101.:	1451132..1452517	Krebs cycle and related genes	COG0114
WP_011377101.1_1588	CUST_8340_PI442190940	MIT9312	HLII	WP_011377101.1_1588	TGGTCAGAGCAACTTAAAGATGCTGAGAATGCAATTATTATGAGCCTAAATGAATTGTAT	fumC	class II fumarate hydratase	WP_011377101.:	1451132..1452517	Krebs cycle and related genes	COG0114
WP_036924686.1_1633	CUST_8579_PI442190940	MIT9312	HLII	WP_036924686.1_1633	TCAGCTTAAATTTCAAAGGATACGCAGGACAGAGCTTTGGGGCATTTTTATTGAAGGGAA	glsF	Ferredoxin-dependent glutamate synthase	WP_036924686.:	1501424..1505992	Nitrogen Metabolism	COG0069
WP_036924686.1_1633	CUST_8580_PI442190940	MIT9312	HLII	WP_036924686.1_1633	GGAAACACCTGTCTTTATGGCGCAACAGGAGGAAAATTATTTGCATTAGGTAAATCGGGA	glsF	Ferredoxin-dependent glutamate synthase	WP_036924686.:	1501424..1505992	Nitrogen Metabolism	COG0069
WP_036924686.1_1633	CUST_8581_PI442190940	MIT9312	HLII	WP_036924686.1_1633	AGCAGTAACAGAGGGTGCAGGTGATCATTGTTGTGAATACATGACTGGTGGTAAAGTAAT	glsF	Ferredoxin-dependent glutamate synthase	WP_036924686.:	1501424..1505992	Nitrogen Metabolism	COG0069
WP_036924686.1_1633	CUST_8582_PI442190940	MIT9312	HLII	WP_036924686.1_1633	TGCAGGAAGAAATATTGGTGCTGGCATGACTGGTGGAATAGCTTTTATACTCGATGAGAA	glsF	Ferredoxin-dependent glutamate synthase	WP_036924686.:	1501424..1505992	Nitrogen Metabolism	COG0069
WP_036924686.1_1633	CUST_8583_PI442190940	MIT9312	HLII	WP_036924686.1_1633	AGATAGAAGTGTTTGTGCGAAAATATCAGGCGAAATCGCAGAACTTCATGGCAATACTGG	glsF	Ferredoxin-dependent glutamate synthase	WP_036924686.:	1501424..1505992	Nitrogen Metabolism	COG0069
WP_036924686.1_1633	CUST_8584_PI442190940	MIT9312	HLII	WP_036924686.1_1633	CTTTTTTCAAGAGTGCATTCAAATTAATAGTTCCCCCAAGCGAGGAAGAAATGCTTGGCA	glsF	Ferredoxin-dependent glutamate synthase	WP_036924686.:	1501424..1505992	Nitrogen Metabolism	COG0069
WP_011377157.1_1644	CUST_8631_PI442190940	MIT9312	HLII	WP_011377157.1_1644	ATTGCATGGGTAACTTTCTACTGGCACTGGAAGCACCTAACAATTTGGCAAGGTAATATG	psaB	Photosystem I P700 chlorophyll A apoprotein A2 PsaB			Photosystem I	COG2885
WP_011377157.1_1644	CUST_8632_PI442190940	MIT9312	HLII	WP_011377157.1_1644	TCCTTATCTCCTTGGGCTTGGATGTTCCTATTTGGTCATTTAGTTTGGGCTACTGGATTT	psaB	Photosystem I P700 chlorophyll A apoprotein A2 PsaB			Photosystem I	COG2885
WP_011377157.1_1644	CUST_8633_PI442190940	MIT9312	HLII	WP_011377157.1_1644	TTGGACCAGCAGATTTCTTAGTTCATCATGCTATAGCGCTAGGACTTCATACAACAGCAC	psaB	Photosystem I P700 chlorophyll A apoprotein A2 PsaB			Photosystem I	COG2885
WP_011377157.1_1644	CUST_8634_PI442190940	MIT9312	HLII	WP_011377157.1_1644	AGCTAACCTTGTAGGCTGGAGAGATAAGCCAGTTGCACTTTCAATTGTTCAAGCTAGATT	psaB	Photosystem I P700 chlorophyll A apoprotein A2 PsaB			Photosystem I	COG2885
WP_011377157.1_1644	CUST_8635_PI442190940	MIT9312	HLII	WP_011377157.1_1644	TTAATTCCTGATAAAAAGGATTTAGGTTATGCTTTCCCTTGCGATGGACCAGGTCGTGGA	psaB	Photosystem I P700 chlorophyll A apoprotein A2 PsaB			Photosystem I	COG2885
WP_011377157.1_1644	CUST_8636_PI442190940	MIT9312	HLII	WP_011377157.1_1644	TGGCACATTTCACGATAGGAAACATCCTTACATTTGGAGCCTTTGTGATTGCATCAACAT	psaB	Photosystem I P700 chlorophyll A apoprotein A2 PsaB			Photosystem I	COG2885
WP_011377158.1_1645	CUST_8637_PI442190940	MIT9312	HLII	WP_011377158.1_1645	TGGGCTCATAACAAGCTTAAAGTTGCCCCAACAATACAACCAAGAGCACTTTCAATTACT	psaA	Photosystem I P700 chlorophyll A apoprotein A1 PsaA			Photosystem I	COG2885
WP_011377158.1_1645	CUST_8638_PI442190940	MIT9312	HLII	WP_011377158.1_1645	CAAAGTATTCAAGCTTCTGCAGTAGGCACTTCAATTTTAGCTGGTACTCCGGAGGGATTA	psaA	Photosystem I P700 chlorophyll A apoprotein A1 PsaA			Photosystem I	COG2885
WP_011377158.1_1645	CUST_8639_PI442190940	MIT9312	HLII	WP_011377158.1_1645	GTGGGGACTTACAGGAGGAAACTTCTCTCAAAGTGCTATCACCATTAATGGTTGGCTAAG	psaA	Photosystem I P700 chlorophyll A apoprotein A1 PsaA			Photosystem I	COG2885
WP_011377158.1_1645	CUST_8640_PI442190940	MIT9312	HLII	WP_011377158.1_1645	GGAAGAGGTGGTACTTGTCAGGTTTCATCATGGGATCATGTCTTCTTAGCTCTTTTCTGG	psaA	Photosystem I P700 chlorophyll A apoprotein A1 PsaA			Photosystem I	COG2885
WP_011377158.1_1645	CUST_8641_PI442190940	MIT9312	HLII	WP_011377158.1_1645	AGGTGGAAAGGTAGCCATATCTCCAATTCAACTTGGAACTGCTGATTTGATGATCCATCA	psaA	Photosystem I P700 chlorophyll A apoprotein A1 PsaA			Photosystem I	COG2885
WP_011377158.1_1645	CUST_8642_PI442190940	MIT9312	HLII	WP_011377158.1_1645	AGCTCACTTCATATGGGCGTTTAGTTTGATGTTCTTATTCAGTGGAAGAGGCTATTGGCA	psaA	Photosystem I P700 chlorophyll A apoprotein A1 PsaA			Photosystem I	COG2885
WP_011377209.1_1697	CUST_8919_PI442190940	MIT9312	HLII	WP_011377209.1_1697	GAAGTGAATTATTAAGGGGGGCCCTTCTAACTATTAATGGGATAGCAGCAGGCATGAGAA	ppc	Phosphoenolpyruvate carboxylase	WP_011377209.:	1553903..1556872	Krebs cycle and related genes	COG2352
WP_011377209.1_1697	CUST_8921_PI442190940	MIT9312	HLII	WP_011377209.1_1697	ATGCTACTCCAGAATGGAATCAATTAATGTCTAGGTTGGCAGAAACATCAAGGTCTCACT	ppc	Phosphoenolpyruvate carboxylase	WP_011377209.:	1553903..1556872	Krebs cycle and related genes	COG2352
WP_011377209.1_1697	CUST_8922_PI442190940	MIT9312	HLII	WP_011377209.1_1697	TGGGTATTTGGTTGGACACAAAGTAGATTTCTCTTACCTAGTTGGTTTGGAGTAGGTACT	ppc	Phosphoenolpyruvate carboxylase	WP_011377209.:	1553903..1556872	Krebs cycle and related genes	COG2352
WP_011377209.1_1697	CUST_8923_PI442190940	MIT9312	HLII	WP_011377209.1_1697	TGGGATTTTTGCAGGTTTCACTTTTAAGAAGACTTAGAGATCAAACAAGACAACCTCCAA	ppc	Phosphoenolpyruvate carboxylase	WP_011377209.:	1553903..1556872	Krebs cycle and related genes	COG2352
WP_011377209.1_1697	CUST_8924_PI442190940	MIT9312	HLII	WP_011377209.1_1697	ACCCACAACAAATTGAATTATTAAGAGTCCTACATCAAAGATGGCCATTTTTTAGGATGC	ppc	Phosphoenolpyruvate carboxylase	WP_011377209.:	1553903..1556872	Krebs cycle and related genes	COG2352
WP_011377241.1_1729	CUST_9099_PI442190940	MIT9312	HLII	WP_011377241.1_1729	GAAGGCAGGTTTCAAATGTAATTGATATCTGTGCAGGAATAGAAGTTGGAGTTGCGGCAA	glmS	Glutamine--fructose-6-phosphatetransaminase(isomerizing)	WP_011377241.:	1587656..1589551	Amino Acid metabolism	COG0449
WP_011377241.1_1729	CUST_9100_PI442190940	MIT9312	HLII	WP_011377241.1_1729	AGGAGCGTTAAAACTCAAAGAAATTAGTTACATACATGCGGCTGGATATCCAGCTGGTGA	glmS	Glutamine--fructose-6-phosphatetransaminase(isomerizing)	WP_011377241.:	1587656..1589551	Amino Acid metabolism	COG0449
WP_011377241.1_1729	CUST_9101_PI442190940	MIT9312	HLII	WP_011377241.1_1729	ATTGCCTCCCCTGGTGAAGTTTTTGATAAAGTCATCAGTAATGCTCAAGAAGCAAAAGCT	glmS	Glutamine--fructose-6-phosphatetransaminase(isomerizing)	WP_011377241.:	1587656..1589551	Amino Acid metabolism	COG0449
WP_011377241.1_1729	CUST_9102_PI442190940	MIT9312	HLII	WP_011377241.1_1729	TGCAGCTCATAGAGGACTTGACGTAGATCAACCAAGAAATTTAGCTAAAAGTGTAACTGT	glmS	Glutamine--fructose-6-phosphatetransaminase(isomerizing)	WP_011377241.:	1587656..1589551	Amino Acid metabolism	COG0449
WP_011377241.1_1729	CUST_9103_PI442190940	MIT9312	HLII	WP_011377241.1_1729	AGAGATGCATATTTGATTGGGATTGCTCCTGAGTCTAATGGAACTGAAATCTTTGATTAT	glmS	Glutamine--fructose-6-phosphatetransaminase(isomerizing)	WP_011377241.:	1587656..1589551	Amino Acid metabolism	COG0449
WP_011377241.1_1729	CUST_9104_PI442190940	MIT9312	HLII	WP_011377241.1_1729	TGGATTAGCTATAAAATTTGCGCAAATTAAAGGTAGTCGAAGCTCAGATGAAATAGGTAA	glmS	Glutamine--fructose-6-phosphatetransaminase(isomerizing)	WP_011377241.:	1587656..1589551	Amino Acid metabolism	COG0449

Table S3. List of probes (6,834) and their sequences utilized in the arrays for 1,200 genes of different strains of *Prochlorococcus* that dominate at Station ALOHA.

A.

	4h_1control	4h_2control	4h_1glucose	4h_2glucose	12h_control	12h_1glucos	12h_2glucos	24h_1glucos	24h_1control	2D_4h_contr	2D_4h_1gluc	2D_4h_2gluc	2D_12h_cont	2D_12h_gluc	2D_24h_gluc	Ecotype	Strain	Gene.symbol	ID_Gene	Pathway	Protein.ID	Protein_ID2	COG.Functio	Description	Location
GID.CAE19244.1_885	1,71203875	1,90064939	2,25671894	1,90969826	1,88882562	2,19223794	2,056578	2,28690605	2,22272242	1,7372555	1,53767186	1,55902059	1,49663033	1,56345481	2,11494013	HLI	MED4	prk	CAE19244.1_	Carbon fixati	CAE19244.1		COG3954	Phosphoribu	746750..747649
GID.CAE19009.1_607	2,85210399	2,59865426	2,92845908	2,13760651	0,39327925	0,07159744	0,72650358	3,2354929	3,92350825	0,2837859	0,17358615	0,07999723	0,80859438	0,51265218	1,67026707	HLI	MED4	rbcL	CAE19009.1_	Carbon fixati	CAE19009.1		COG1850	Ribulose 1,5-	519087..520502
GID.CAE19010.1_608	5,24340376	5,39850142	5,46601371	4,23210934	1,26369692	0,64068487	0,91474598	6,40974322	6,60107601	2,42690824	2,49472294	2,7423207	1,18870604	2,42103053	4,49233479	HLI	MED4	rbcS	CAE19010.1_	Carbon fixati	CAE19010.1	PMM0551	COG4451	Ribulose bisp	520592..520933
GID.CAE19768.1_1497	4,29151477	4,33324569	4,3267709	4,36294852	4,31696585	4,17838039	4,44465147	3,55342625	3,72486936	4,22394009	4,17384416	4,11939669	3,61062935	3,95877514	3,79089405	HLI	MED4	ftsZ	CAE19768.1_	Cell Division	CAE19768.1	PMM1309	COG0206	cell division	1262202..1263317
GID.CAE19801.1_1531	1,87804585	2,18017596	1,80968775	1,77786231	1,85978459	1,45803568	1,93293372	1,50539047	1,7662762	1,27222824	1,74594283	1,67542272	1,3151827	2,00718176	1,96044635	HLI	MED4	kaiC	CAE19801.1_	Circadian Rh	CAE19801.1	PMM1342	COG0467	possible circa	1293980..1295509
GID.CAE19910.1_1691	3,98725044	3,97487365	4,19700628	3,68479495	3,05956974	2,39323741	2,42219534	4,72262459	4,74621194	3,160924	2,97620776	2,94432546	3,34863352	3,18923676	3,47082228	HLI	MED4	atpA	CAE19910.1_	Energy meta	CAE19910.1	PMM1451	COG0056	ATP synthas	1387064..1388581
GID.CAE19897.1_1678	6,55935045	6,38580029	6,55061305	6,46168903	5,33021922	5,49377896	5,19944568	7,19105403	7,4604024	5,88905588	5,56933909	5,58612945	6,76848944	5,76992744	6,53873925	HLI	MED4	atpB	CAE19897.1_	Energy meta	CAE19897.1	PMM1438	COG0055	ATP synthase	1375858..1377318
GID.CAE18912.1_509	1,78312512	1,57905137	1,74938377	2,92699709	1,94207214	1,78312512	2,30072865	2,07324492	1,74989351	1,7694321	1,56360678	1,38226033	1,80331088	1,46582723	1,72743193	HLI	MED4	gdh	CAE18912.1_	Entner-Dudo	CAE18912.1	PMM0453	COG1028	3-oxoacyl-ac	432615..433364
GID.CAE19668.1_1395	1,14523494	1,19795897	1,47763877	1,72116989	1,48259486	1,75461166	1,6955204	1,69675425	2,16559837	0,79707875	0,85743644	0,87879143	1,77272741	1,23547748	1,62207911	HLI	MED4	galE	CAE19668.1_	Galactose m	CAE19668.1	PMM1209	COG1087	UDP-glucose	1158248..1159339
GID.CAE19055.1_653	3,12480386	3,02457808	2,88594448	2,79707461	3,16599375	3,27012752	2,5769333	3,08334039	2,87554358	2,51012328	2,66374825	2,60603674	2,97014281	2,73800268	2,58317231	HLI	MED4	glk	CAE19055.1_	Glycolysis	CAE19055.1	PMM0596	COG0837	Glucokinase	565152..566189
GID.CAE19371.1_1039	2,04680346	2,01528062	2,15877682	2,66523678	2,37044743	2,7363334	2,5827213	2,28278541	2,65162539	1,9804152	1,6887419	1,73414853	2,41438761	1,75830683	2,46563142	HLI	MED4	pykF	CAE19371.1_	Glycolysis	CAE19371.1	PMM0912	COG0469	Pyruvate kin	872358..874148
GID.CAE18667.1_215	2,10788577	2,0306971	2,0526315	2,58435461	2,57932544	2,48581052	2,50450397	2,4433959	2,07864745	2,04164382	1,96929933	1,86943034	2,68995123	2,22911047	2,28356018	HLI	MED4	eno	CAE18667.1_	Glycolysis/Gl	CAE18667.1	PMM0208	COG0148	Enolase	201456..202748
GID.CAE19240.1_881	5,30643396	5,14437592	5,63470696	5,75520457	3,43776229	4,17725288	3,40495311	6,35862426	6,86094798	4,51779813	4,39647969	4,26926872	5,32019668	4,46365855	5,77866175	HLI	MED4	fda	CAE19240.1_	Glycolysis/Gl	CAE19240.1	PMM0781	COG0191	Fructose-1,6	742975..744048
GID.CAE19747.1_1475	4,12309236	4,02626975	4,29783475	4,52715901	2,65711774	2,62011609	2,93756453	4,04152095	4,16411735	3,50806349	3,58182306	3,52098043	2,87072711	3,08443292	4,26627033	HLI	MED4	pdhA	CAE19747.1_	Glycolysis/Kr	CAE19747.1	PMM1288	COG1071	Pyruvate deh	1239069..1240106
GID.CAE19389.1_1058	2,29574585	1,91607431	2,21583219	1,94373334	1,17559316	0,62182352	0,66790282	2,63577848	1,75530785	1,61595631	1,70365096	1,70533791	1,58697988	1,3401165	2,02824658	HLI	MED4	pdhB	CAE19389.1_	Glycolysis/Kr	CAE19389.1	PMM0930	COG0022	Pyruvate del	890595..891578
GID.CAE19379.1_1047	1,51497614	1,60860406	1,63020801	2,13614845	2,07881926	2,01716967	2,01946489	1,87206687	1,70221141	2,43474983	2,49073084	2,63607919	1,85553001	1,92989939	3,00666999	HLI	MED4	glnA	CAE19379.1_	Nitrogen me	CAE19379.1	PMM0920	COG0174	Glutamine sy	881367..882788
GID.CAE19971.1_1754	1,87673798	1,71239253	1,51867468	2,25376683	2,19964448	2,23465169	2,64333765	1,881412	1,78725783	1,97494294	1,69850103	1,93222426	2,19459263	1,8132028	2,37453257	HLI	MED4	glsF	CAE19971.1_	Nitrogen me	CAE19971.1	PMM1512	COG0069	Ferredoxin-c	1450922..1455487
GID.CAE18978.1_576	1,34242268	1,53616115	0,92034071	2,2488495	3,91492485	3,34209958	3,86229099	1,0232028	1,28330493	4,55425081	4,48221382	4,47716058	3,64228806	4,26650592	2,22440931	HLI	MED4	tal	CAE18978.1_	Pentose Pho	CAE18978.1	PMM0519	COG0176	Transaldolas	490726..491727
GID.CAE19983.1_1766	8,95450655	9,12100809	9,65518131	9,9455001	9,10535929	9,70379283	9,45320822	9,22149872	9,57105476	9,44816214	9,05875047	8,98078708	9,11950206	8,67383895	8,94721494	HLI	MED4	psaA	CAE19983.1_	Photosystem	CAE19983.1	PMM1524	COG2885	Photosystem	1464162..1466465
GID.CAE19982.1_1765	4,64215759	4,68388754	5,51477033	5,93150265	5,09144927	5,1276458	5,03328841	5,42294592	5,68804407	5,47032935	5,05413122	5,02306555	4,7280144	4,70521605	5,09144927	HLI	MED4	psaB	CAE19982.1_	Photosystem	CAE19982.1	PMM1523	COG2885	Photosystem	1461905..1464133
GID.CAE18904.1_500	1,50000143	1,79316649	0,99106572	1,96975129	5,23527568	4,7929001	5,10335727	2,74160579	1,85906702	3,97179023	4,28881596	4,23369701	3,97884197	4,55221168	1,44043143	HLI	MED4	cyoB/coxA	CAE18904.1_	Respiration	CAE18904.1	PMM0445	COG0843	Cytochrome	423492..425117
GID.WP_002805854.1_	9,86064377	9,5149672	9,49691398	8,43173636	6,7807167	6,4875156	6,72821424	10,2953388	10,517414	6,83610898	6,69117817	6,75636531	7,10276354	7,33260609	8,40697159	HLI	MIT9515	rbcL	WP_0028058	Carbon fixati	WP_002805854.1		COG1850	Ribulose 1,5-	549602..551017
GID.WP_011376108.1_	8,24622268	8,38338141	8,23700662	7,28079066	4,54597539	4,48515575	4,12187921	9,09606984	9,1791447	5,61789188	5,29689175	5,45602452	5,00109444	5,09828448	7,52311359	HLI	MIT9515	rbcS	WP_0113761	Carbon fixati	WP_011376108.1		COG4451	Ribulose bisp	551111..551452
GID.WP_011820774.1_	2,36430247	2,26693599	2,34246595	2,89301669	2,51479414	2,56420937	2,58856269	2,39739508	2,47440931	2,36758526	2,17334085	2,10404384	2,62178316	2,15499826	2,27094323	HLI	MIT9515	ftsZ	WP_0118207	Cell Division	WP_011820774.1		COG0206	cell division	1307400..1308515
GID.WP_011820806.1_	2,95644926	2,95369339	2,76998063	2,60453899	2,83796946	2,47867609	2,36567338	2,65269578	2,35307817	1,99223943	2,38524787	2,50745819	1,6625161	2,47867609	2,31052318	HLI	MIT9515	kaiC	WP_0118208	Circadian Rh	WP_011820806.1		COG0467	circadian clo	1339194..1340723
GID.WP_011820932.1_	3,00044849	2,91214205	3,17506404	2,30413092	1,50203187	1,18422506	1,36488284	3,40359183	3,76462435	2,24699038	2,20959551	2,02332909	1,94831168	1,94470167	2,13885247	HLI	MIT9515	atpA	WP_0118209	Energy meta	WP_011820932.1		COG0056	ATP synthas	1424492..1426009
GID.WP_041710653.1_	2,70633546	2,83018584	3,32936736	2,49903488	2,26913767	1,99202423	2,50512943	2,92988805	3,13799849	1,98533019	2,51638311	2,53236237	2,2975771	2,37953833	2,33355113	HLI	MIT9515	atpI	WP_0417106	Energy meta	WP_041710653.1		COG0356	ATP synthase	1428031..1428756
GID.WP_011819975.1_	1,7675606	1,58037955	1,70342106	2,63609549	1,61180774	2,21468303	2,26843125	2,19615946	2,52729304	1,93918169	0,73854749	1,16453648	2,44092851	1,67745886	1,69279107	HLI	MIT9515	glk	WP_0118199	Glycolysis	WP_011819975.1		COG0837	Glucokinase	596236..597273
GID.WP_011820115.1_	2,64597838	2,22448821	2,46627845	3,21285389	0,99467045	2,23636412	2,81065747	2,94270384	3,2215064	2,06059965	1,47488875	1,50592587	2,91876076	1,74966853	2,96599314	HLI	MIT9515	cbbA	WP_0118201	Glycolysis/Gl	WP_011820115.1		COG0191	Fructose-1,6	723412..724485
GID.WP_011819560.1_	1,53619501	1,2884398	1,82407331	1,92670903	1,94615306	2,18846935	1,89195956	2,08606756	1,96931794	1,40529509	1,38777628	1,21032765	2,31225288	1,7342386	2,25953232	HLI	MIT9515	eno	WP_0118195	Glycolysis/Gl	WP_011819560.1		COG0148	Enolase	218871..220163
GID.WP_011820753.1_	4,14606742	4,23808311	4,30949245	4,24063933	2,66613164	2,52238095	3,2401416	4,02195008	4,28847015	3,42001582	3,61740922	3,61795719	2,85093327	3,25391454	4,03767637	HLI	MIT9515	pdhA	WP_0118207	Glycolysis/Kr	WP_011820753.1		COG1071	Pyruvate deh	1284254..1285291
GID.WP_011820321.1_	2,25444625	2,00993863	2,07068226	2,73824982	1,9101567	2,15498072	1,83279217	2,65960388	2,18764218	2,07825677	2,0441776	2,00778655	1,50355768	1,87595059	2,06372907	HLI	MIT9515	pdhB	WP_0118203	Glycolysis/Kr	WP_011820321.1		COG0022	Pyruvate del	906847..907830
GID.WP_011820127.1_	1,34229998	1,5492522	1,86111277	1,50182321	2,46617638	2,27164838	2,64363146	1,74744767	1,33773687	1,72242414	1,7550327	1,51974131	1,97428034	1,75616504	1,62964053	HLI	MIT9515	edd	WP_0118201	Pentose Pho	WP_011820127.1		COG2084	phosphogluc	731774..733192
GID.WP_011819899.1_	1,96854212	1,82129331	1,67411267	2,91438802	3,38892486	2,97393897	3,17275572	2,01034852	2,15149226	4,05908472	3,79241521	3,8830142	3,30346278	3,90067366	2,44542268	HLI	MIT9515	tal	WP_0118198	Pentose Pho	WP_011819899.1		COG0176	Transaldolas	521181..522182
GID.WP_011820471.1_	1,7054127	1,72253201	1,58882706	1,26424568	3,21712141	3,27733122	2,92780427	2,27870361	1,62626253	2,68948372	3,0474396	2,84580421	3,21291453	3,40948135	2,0176941	HLI	MIT9515	zwf	WP_0118204	Pentose Pho	WP_011820471.1		COG0364	glucose-6-ph	1017903..1019426
GID.WP_011821002.1_	7,53903306	7,65045669	8,28136406	8,71346061	7,52421922	8,27672651	7,87159262	7,65651994	8,23306483	8,06797489	7,7248874	7,71602045	7,2626781	7,29126379	7,44347402	HLI	MIT9515	psaA	WP_0118210	Photosystem	WP_011821002.1		COG2885	Photosystem	1501600..1503882
GID.WP_011821001.1_	4,93937271	5,00231036	5,90595383	6,3481684	5,28047215	5,59262254	5,55030416	6,07415503	6,54306102	6,07937051	5,69436508	5,64511765	5,06045476	5,01350776	5,33694312	HLI	MIT9515	psaB	WP_0118210	Photosystem	WP_011821001.1		COG2885	Photosystem	1499343..1501571
GID.WP_011820570.1_	1,63384559	1,92729164	2,21722786	2,12261953	2,53499616	2,60405799	2,81249967	2,15039484	2,57544094	1,56329501	1,53002882	1,67304688	2,44079215	2,08751378	2,04010632	HLI	MIT9515	cyoB/coxA	WP_0118205	Respiration	WP_011820570.1		COG0843	Cytochrome	1109626..1111317
GID.WP_011819824.1_	1,41197325	1,51017571	1,78124512	1,27237652	4,49289596	4,01345565	4,63353792	2,19197901	2,02626707	3,71086187	3,94812065	3,95183915	3,21312281	3,91465298	1,95198154	HLI	MIT9515	cyoB/coxA	WP_0118198	Respiration	WP_011819824.1		COG0843	Cytochrome	454025..455644
GID.WP_011817489.1_	3,07338589	3,29628382	2,92017114	2,51989096	3,84592541	3,5575618	3,41462393	2,95532528	2,94847733	3,2829339	3,09503154	3,10494729	4,37238112	4,08624502	3,12979288	HLII	AS9606	argH	WP_0118174	Amino Acid r	WP_011817489.1		COG0165	Argininosucc	14527..15906
GID.WP_011819220.1_	3,57126893	3,71130641	3,61262558	3,48397569	2,40488253	2,3403839	2,10192971	2,77030225	2,95775762	2,76594191	3,29676234	3,03487171	2,89387372	2,69768921	3,01263635	HLII	AS9606	glmS	WP_0118192	Amino Acid r	WP_011819220.1		COG0449	Glutamine--f	1548284..1550179
GID.WP_011818291.1_	6,49644479	6,53751051	6,8934079	6,59799041	4,18060994	5,10833356	4,18804315	7,03381487	7,14963274	5,1287023	4,89545915	4,92168247	5,92872936	4,75028897	5,40295486	HLII	AS9606	prk	WP_0118182	Carbon fixati	WP_011818291.1		COG3954	Phosphoribu	735446..736342
GID.WP_002805854.1_	10,1185928	9,6431537	9,67627206	8,59260162	6,92511734	6,72963368	6,94921662	10,3936216	10,6113811	6,96005824	6,77767945	6,77059209	7,243805	7,49906178	8,61557553	HLII	AS9606	rbcL	WP_0028058	Carbon fixati	WP_002805854.1		COG1850	Ribulose 1,5-	529175..530590
GID.WP_011376108.1_	6,99664406	7,15403142	7,2095552	6,00215823	3,46220612	1,72292188	0,98314337	8,32426053	8,41659163	4,29573775	4,21387222	4,43806657	1,9570447	3,89611837	6,42236246	HLII	AS9606	rbcS	WP_0113761	Carbon fixati	WP_011376108.1		COG4451	Ribulose bisp	530681..531022
GID.WP_011818927.1_	8,30624546	8,32490596	7,99267581	8,16648349	8,32409279	8,45575193	8,39788287	7,13897461	6,86169444	7,82181563	7,67474815	7,62990699	7,64765532	7,87129995	7,4278438	HLII	AS9606	ftsZ	WP_0118189	Cell Division	WP_011818927.1		COG0206	cell division	1291754..1292869
GID.WP_011817809.1_	2,67831406	2,48620908	2,81121591	2,75901507	2,53733265	2,64151647	2,42203427	2,48084421	2,3549771	2,44195933	2,47534321	2,47900642	2,31689049	2,20426666	2,29517839	HLII	AS9606	minC	WP_0118178	Cell Division	WP_011817809.1		COG0850	septum site-	316457..317119
GID.WP_080512707.1_	4,16086657	4,10354349	3,58004877	3,59052059	5,4482108	6,32842249	5,46512639	3,04375428	2,65936958	5,9616723	5,81689902	5,83486346	4,51402295	4,9802903	2,51180333	HLII	AS9606	minD	WP_0805127	Cell Division	WP_080512707.1		COG2894	septum site-	315530..316345
GID.WP_011817807.1_	1,17361279	0,89095168	0,54250564	0,1743763	1,849834	2,44335471	1,55802225	1,11119014	0,96198278	2,98700946	2,91931638	3,09690234	1,44132352	2,18434525	0,52813671	HLII	AS9606	minE	WP_0118178	Cell Division	WP_011817807.1		COG0851	septum site-	315202..315528
GID.WP_011818959.1_	3,81839781	3,75933623	4,0265602	3,69276762	4,58775876	4,44755839	4,47935754	4,34716517	4,58336076	3,44598808	3,70050582	3,78209762	4,76971946	4,63891297	4,00244342	HLII	AS9606	kaiB	WP_0118189	Circadian Rh	WP_011818959.1		COG4251	Circadian clo	1325093..1325410
GID.WP_011818958.1_	7,42376466	7,55629339	6,92285475	6,68651646	6,77070606	6,29973137	6,63415091	6,52646617	6,45466982	5,95563392	6,11765182	6,16657748	6,48778416	6,71620524	6,91337824	HLII	AS9606	kaiC	WP_0118189	Circadian Rh	WP_011818958.1		COG0467	circadian clo	1323503..1325032
GID.WP_011819066.1_	9,37065869	9,3221924	9,21974773	8,89785233	6,82286278	6,77363061	6,69466705	9,52916257	9,52647737	7,8507717	7,58376468	7,63325753	8,09577713	7,65234464	8,6010912	HLII	AS9606	atpA	WP_0118190	Energy meta	WP_011819066.1		COG0056	ATP synthas	1397246..1398763
GID.WP_011819054.1_	6,15873973	6,02696952	5,94442751	5,5411102	2,91767876	3,75501023	2,70463085	6,21866661	6,20459621	4,69527322	4,54531049	4,63766422	4,19107161	3,89819541	5,02869147	HLII	AS9606	atpC	WP_0118190	Energy meta	WP_011819054.1		COG0355	ATP synthas	1387557..1387961
GID.WP_011819070.1_	6,87806419	6,64618953	6,55983456	6,19943625	4,44039318	4,66883298	4,60635999	6,78260144	6,30885041	5,06783694	5,17139251	5,18376599	5,17327583	5,00196536	6,0256866	HLII	AS9606	atpI	WP_0118190	Energy meta	WP_011819070.1		COG0356	ATP synthase	1400787..1401512
GID.WP_011817718.1_	2,69629789	2,65335283	2,04258067	0,63479217	1,53221665	0,94069363	0,70703262	1,21871623	2,34923713	1,33232129	1,72956202	1,98322839	1,25251088	1,76168283	2,66872275	HLII	AS9606	eda	WP_0118177	Entner-Dudo	WP_011817718.1		COG0800	2-dehydro-3-	229148..229774
GID.WP_011817965.1_	4,15024861	3,91989788	3,59527271	3,49432523	3,91012063	3,38386892	3,77618143	3,53250816	3,36175989	3,58632053	3,65728948	3,73317804	3,39497659	3,70068421	3,70891822	HLII	AS9606	gdh	WP_0118179	Entner-Dudo	WP_011817965.1		COG1028	3-oxoacyl-ac	443774..444523
GID.WP_011818939.1_	4,94770117	4,9369969	4,74857551	4,27930158	4,7396058	4,44629312	4,37959883	4,45001391	4,16915592	4,00276494	4,41718266	4,44001537	3,73417419	4,73416912	3,75860081	HLII	AS9606	glcH	WP_0118189	GlcH sugar tr	WP_011818939.1		COG2211	GPH family	1304362..1305708
GID.WP_011818274.1_	6,64367347	6,50090051	6,40918846	6,39941977	4,4422679	4,79302414	4,43997509	5,79677543	5,85811031	5,39366344	5,49312785	5,52109346	5,32198624	4,68844113	5,32255436	HLII	AS9606	glgC	WP_0118182	Glycogen me	WP_011818274.1		COG0448	Glucose-1-p	720461..721756
GID.WP_011818100.1_	4,83614441	4,78125928	4,62351616	4,39038156	4,52376706	4,58682439	4,55815342	4,6630668	4,26676333	3,52139665	3,93196064	3,94284808	4,23333326	3,84262134	4,12774329	HLII	AS9606	glk	WP_0118181	Glycolysis	WP_011818100.1		COG0837	Glucokinase	575153..576187
GID.WP_011818389.1_	6,89059285	6,99260939	6,84328929	6,53712294	6,11889299	5,9026146	5,78813059	6,79217394	6,5897122	5,36113279	5,32962587	5,38711921	6,39227124	5,79886411	5,97522022	HLII	AS9606	pykF	WP_0118183	Glycolysis	WP_011818389.1		COG0469	Pyruvate kin	817097..818887
GID.WP_011818286.1_	9,72558301	9,7729801	9,57698056	9,44099213	7,75113138	8,50473691	7,63870818	10,507771	10,0747431	8,13838313	8,05539103	8,0109528	9,63556392	8,62085387	9,85976013	HLII	AS9606	cbbA	WP_0118182	Glycolysis/Gl	WP_011818286.1		COG0191	Fructose-1,6	730539..731612
GID.WP_011817700.1_	7,49771877	7,46751061	7,34124287	7,21973484	7,52731862	7,45495773	7,34885954	7,29111793	7,15031902	7,46385165	7,55099981	7,58506442	7,54550857	7,62596368	7,15181078	HLII	AS9606	eno	WP_0118177	Glycolysis/Gl	WP_011817700.1		COG0148	Enolase	211249..212541
GID.WP_011818287.1_	5,30821255	5,13079253	5,07545542	4,82241386	4,31987817	4,1615755	4,26725114	4,83955656	4,64033042	4,12565509	3,96126409	3,97340541	4,43786712	4,55387611	4,42819693	HLII	AS9606	fda	WP_0118182	Glycolysis/Gl	WP_011818287.1		COG0191	Fructose-1,6	731760..732827
GID.WP_011818410.1_	6,46961384	6,3939568	6,45068258	6,51807968	4,2802427	4,08232596	3,79395713	5,04237975	4,93148136	5,99566622	6,26642015	6,26012306	4,15836132	4,77116578	5,96354478	HLII	AS9606	pgi	WP_0118184	Glycolysis/Gl	WP_011818410.1		COG0166	Glucose-6-p	837779..839362
GID.WP_011818906.1_	8,26466463	8,11741933	8,21064238	8,24840374	6,21891808	6,05093863	6,49507836	7,75171053	7,67499528	7,06857957	7,24401803	7,23798205	5,81050658	6,29063428	8,17633511	HLII	AS9606	pdhA	WP_0118189	Glycolysis/Kr	WP_011818906.1		COG1071	Pyruvate deh	1268563..1269636
GID.WP_011818370.1_	10,2900606	10,3793732	9,9297816	9,50514202	7,52111641	7,47325814	7,54257999	9,98833623	9,18961795	8,96262759	9,12809842	9,14103691	9,19616913	9,11573416	9,49760058	HLII	AS9606	pdhB	WP_0118183	Glycolysis/Kr	WP_011818370.1		COG0022	Pyruvate del	799701..800684
GID.WP_011819079.1_	3,77175128	3,71689222	3,36302585	3,30935908	4,57386602	4,10181789	4,07305749	3,92299248	3,23515602	4,16687347	4,3184108	4,32594926	4,05078071	4,6100437	3,64539163	HLII	AS9606	fumC	WP_0118190	Krebs cycle a	WP_011819079.1		COG0114	class II fuma	1409186..1410571
GID.WP_011819187.1_	4,39013814	4,17156083	4,12898494	4,15025101	4,05949386	3,77106423	3,9184861	4,17607847	4,23886304	3,7802051	4,01435346	4,03835314	3,75386365	3,85613719	4,03276628	HLII	AS9606	ppc	WP_0118191	Krebs cycle a	WP_011819187.1		COG2352	Phosphoeno	1513603..1516572
GID.WP_011818380.1_	7,10104236	7,28833337	7,13160738	7,6991043	7,39955496	7,42915145	7,45566718	6,88862119	7,13393194	8,08388284	8,1112878	8,13101712	7,18490372	7,4402905	8,78864703	HLII	AS9606	glnA	WP_0118183	Nitrogen me	WP_011818380.1		COG0174	Glutamine sy	808459..809880
GID.WP_011819125.1_	6,71070565	6,83439463	6,44652592	6,59105783	7,26512672	7,04986954	7,43412859	6,60961502	6,67554012	6,28275597	6,56249689	6,52362368	6,78288864	7,17021837	6,73361148	HLII	AS9606	glsF	WP_0118191	Nitrogen me	WP_011819125.1		COG0069	Ferredoxin-c	1458439..1463013
GID.WP_011818275.1_	4,74469626	4,89665274	4,39279459	4,05776346	6,23904188	6,42229172	6,28965054	4,72675268	4,26968675	5,45931427	5,33515228	5,37279931	6,26729493	5,86370983	4,80271918	HLII	AS9606	edd	WP_0118182	Pentose Pho	WP_011818275.1		COG2084	phosphogluc	721853..723271
GID.WP_011818610.1_	4,03117388	3,85230241	3,64462306	2,02028089	4,96995042	4,64027102	4,63556572	3,90319186	2,83696338	4,10004523	4,15493907	4,19229225	3,68758559	4,21509598	1,38438521	HLII	AS9606	opcA	WP_0118186	Pentose Pho	WP_011818610.1		COG3429	Glucose-6-P	992976..994277
GID.WP_011818582.1_	5,72466491	5,6174364	5,65506132	5,31652781	5,75625607	6,0671373	5,83223642	5,48545386	5,31480916	5,34505735	5,57997298	5,59210092	5,53768966	5,09384009	5,53768966	HLII	AS9606	rbsK	WP_0118185	Pentose Pho	WP_011818582.1		COG0524	Sugar kinase	964467..965336
GID.WP_011818027.1_	5,22425461	5,22487281	5,2596605	5,82351302	8,96345636	8,32800312	8,9193631	5,06040737	5,16415011	9,3669613	9,38023342	9,31580208	8,53587805	9,30729032	6,49166393	HLII	AS9606	tal	WP_0118180	Pentose Pho	WP_011818027.1		COG0176	Transaldolas	500786..501787
GID.WP_011818611.1_	4,71819432	4,62186081	4,35912311	4,0333037	6,74506447	6,79297729	6,68397604	4,70711494	4,19715384	5,51572162	5,59420363	5,56135278	6,04985798	6,34927115	3,87331804	HLII	AS9606	zwf	WP_0118186	Pentose Pho	WP_011818611.1		COG0364	glucose-6-ph	994280..995803
GID.WP_011819136.1_	12,5263245	12,6551458	12,7353594	12,7880829	12,299856	12,6297385	12,5147289	12,5102575	12,6622528	12,4630219	12,2791375	12,2507162	12,2796769	12,0071646	12,0152639	HLII	AS9606	psaA	WP_0118191	Photosystem	WP_011819136.1		COG2885	Photosystem	1471672..1473975
GID.WP_011819135.1_	8,36472012	8,50046392	9,11617202	9,62596918	8,68309749	9,08657189	8,82579096	9,00593493	9,14310055	9,4303728	8,92279325	8,92009857	8,92760406	8,39111219	8,63289335	HLII	AS9606	psaB	WP_0118191	Photosystem	WP_011819135.1		COG2885	Photosystem	1469416..1471644
GID.WP_011817957.1_	3,38042566	3,27867014	3,43488012	2,62970185	6,35302292	6,19522087	6,58547724	3,5544348	3,12111963	6,89883029	6,76727649	6,80053761	5,26170032	6,3355962	3,57708772	HLII	AS9606	ctaC/coxB	WP_0118179	Respiration	WP_011817957.1		COG1622	Cytochrome	436271..437074
GID.WP_011817956.1_	5,71172321	5,66890995	5,78465796	5,99095823	9,79987989	9,37825025	9,82660196	6,71707948	6,43685978	8,79470024	9,18683035	9,19066613	8,0245976	9,09446618	6,56741046	HLII	AS9606	cyoB/coxA	WP_0118179	Respiration	WP_011817956.1		COG0843	Cytochrome	434649..436274
GID.WP_011817955.1_	2,70983485	3,14717811	2,32617744	1,6802535	6,3361923	5,94792491	6,4105799	3,61124851	3,10447288	5,27154382	5,6984166	5,6085698	4,63806326	5,50620093	2,49403263	HLII	AS9606	cyoC	WP_0118179	Respiration	WP_011817955.1		COG1845	heme-copper	434046..434648
GID.WP_042849489.1_	1,5318386	1,95207935	1,10523365	1,13426093	2,89282794	2,41565448	2,22101117	1,81824514	1,86678578	1,78587179	1,93295133	1,8946985	3,21586482	2,97028404	1,70265155	HLII	MIT0604	argH	WP_0428494	Amino Acid r	WP_042849489.1		COG0165	Argininosucc	14513..15892
GID.WP_042851139.1_	2,08872437	2,24507456	1,93321247	1,17478153	1,77575913	1,65234038	1,65234038	1,65234038	1,55979511	1,55940843	1,87886682	1,65234038	1,3188718	1,65234038	1,22486241	HLII	MIT0604	glmS	WP_0428511	Amino Acid r	WP_042851139.1		COG0449	Glutamine--f	1647103..1648998
GID.WP_042850248.1_	5,95045447	5,76284155	6,39486953	6,31108771	3,7682186	4,58385633	4,18862197	6,48008511	6,77527043	4,95987934	4,74690347	4,78490722	5,41145439	4,56626047	5,31016758	HLII	MIT0604	prk	WP_0428502	Carbon fixati	WP_042850248.1		COG3954	Phosphoribu	756168..757064
GID.WP_011376108.1_	7,38291528	7,41805783	7,49178311	6,25799498	3,92738956	2,89223014	2,78077967	8,43518798	8,6151063	4,60283139	4,48124954	4,61733818	3,25914529	4,23397885	6,60034409	HLII	MIT0604	rbcS	WP_0113761	Carbon fixati	WP_011376108.1		COG4451	Ribulose bisp	522365..522706
GID.WP_042850863.1_	5,56691926	5,67646077	5,5509405	5,84235504	5,59161754	5,69112878	5,86651942	5,00907533	5,14655788	5,68752102	5,62537151	5,55603738	5,43957247	5,44179757	5,4451136	HLII	MIT0604	ftsZ	WP_0428508	Cell Division	WP_042850863.1		COG0206	cell division	1357426..1358541
GID.WP_042849817.1_	2,91350769	2,74495941	3,20931626	3,26334606	3,10485523	3,28272368	2,84688565	2,89018431	3,01997672	2,76488294	2,8137594	2,77034793	2,76094338	2,74189275	2,78111903	HLII	MIT0604	minC	WP_0428498	Cell Division	WP_042849817.1		COG0850	septum site-	333021..333683
GID.WP_042849816.1_	4,53367597	4,48763169	4,23398474	4,42823276	5,71951314	6,58277528	5,87645374	3,79787551	3,56690951	6,16032287	5,91783022	5,96807409	4,92181934	5,19643147	3,35216082	HLII	MIT0604	minD	WP_0428498	Cell Division	WP_042849816.1		COG2894	septum site-	332094..332909
GID.WP_042849815.1_	4,49607567	4,35479283	4,37452256	4,24538214	5,44656678	5,39459826	4,94995357	4,50980553	4,41265204	4,78809706	5,66644023	5,23090558	4,86582439	5,14156589	5,03011334	HLII	MIT0604	minE	WP_0428498	Cell Division	WP_042849815.1		COG0851	septum site-	331757..332092
GID.WP_025913788.1_	5,16505286	5,23773365	5,18593719	4,84534253	6,10678533	5,38308974	5,5793712	5,80301168	5,55111575	5,0474326	5,23559566	5,17883691	6,38118648	6,8182331	5,12828857	HLII	MIT0604	kaiB	WP_0259137	Circadian Rh	WP_025913788.1		COG4251	Circadian clo	1390766..1391083
GID.WP_042850895.1_	6,10038295	6,17537955	5,89709455	5,56963546	5,73580672	5,21861259	5,44969266	5,36118732	5,56656641	4,79239796	5,06917652	5,13627383	5,31134242	5,4193305	5,98198949	HLII	MIT0604	kaiC	WP_0428508	Circadian Rh	WP_042850895.1		COG0467	circadian clo	1389164..1390693
GID.WP_011863563.1_	10,295742	10,2966304	10,0777863	9,66166595	7,4098809	7,5138748	7,3743056	10,4686256	10,3239999	8,63959641	8,43190847	8,41873763	9,02639742	8,35698006	9,45417703	HLII	MIT0604	atpA	WP_0118635	Energy meta	WP_011863563.1		COG0056	ATP synthas	1490663..1492180
GID.WP_002806495.1_	5,44719764	5,31530819	5,23696617	5,02691342	2,79413516	3,52028205	2,75985058	5,62800677	5,51066574	3,99727322	3,918336	3,99275812	3,71394014	3,30912874	4,53174625	HLII	MIT0604	atpC	WP_0028064	Energy meta	WP_002806495.1		COG0355	ATP synthas	1480978..1481382
GID.WP_011863567.1_	6,07199607	5,77535336	5,8500551	5,35574789	3,74999048	3,78042675	3,33380445	6,12565674	5,71784202	4,11682162	4,31108853	4,40231976	3,81161282	4,11642678	5,2548305	HLII	MIT0604	atpI	WP_0118635	Energy meta	WP_011863567.1		COG0356	ATP synthase	1494202..1494927
GID.WP_042849917.1_	3,69847499	3,63986336	3,46327444	3,24385801	3,70850212	2,92602212	3,59511913	3,30033929	3,05740208	3,33109866	3,36446528	3,43849095	3,26959084	3,46296281	3,58497818	HLII	MIT0604	gdh	WP_0428499	Entner-Dudo	WP_042849917.1		COG1028	3-oxoacyl-ac	435415..436164
GID.WP_042850518.1_	6,6345797	6,64758216	6,53361188	6,58376343	6,22744189	6,2471747	6,20183176	6,29050835	6,35070314	6,41816359	6,591232	6,52697612	6,37596035	6,32923865	6,63828074	HLII	MIT0604	glcH	WP_0428505	GlcH sugar tr	WP_042850518.1		COG2211	GPH family	987065..988294
GID.WP_042850621.1_	6,5011804	6,6359434	6,3490299	6,56093336	5,67525195	5,75199312	5,90745994	5,6664265	5,45436936	5,56535819	5,70165437	5,73540348	5,05303669	5,73459287	4,63585079	HLII	MIT0604	glcH	WP_0428506	GlcH sugar tr	WP_042850621.1		COG2211	GPH family	1093943..1095199
GID.WP_042851365.1_	5,39782065	5,30284158	5,27535863	5,13789443	5,50213485	5,27344541	5,31691323	5,10356853	5,10477117	4,60242393	4,81315209	4,82706226	4,73330892	5,43156515	4,92202553	HLII	MIT0604	glcH	WP_0428513	GlcH sugar tr	WP_042851365.1		COG2211	GPH family	1370032..1371378
GID.WP_042851238.1_	5,93420982	5,72079896	5,7943482	5,63681228	5,18714689	4,9492314	5,08945741	5,35470971	5,36288654	4,91647009	5,13073296	5,13981865	4,85334674	5,37445593	5,86253934	HLII	MIT0604	glcH	WP_0428512	GlcH sugar tr	WP_042851238.1		COG2211	GPH family	1751241..1752512
GID.WP_042850235.1_	6,69640845	6,45598385	6,43783151	6,34127671	4,45015335	4,85121297	4,52486378	5,91426015	5,797053	5,15406313	5,27181636	5,26464477	5,03541587	4,47458217	5,12538521	HLII	MIT0604	glgC	WP_0428502	Glycogen me	WP_042850235.1		COG0448	Glucose-1-p	741186..742481
GID.WP_042850043.1_	4,44431665	4,53187669	4,21792413	3,88691776	4,47199774	4,57079456	4,01843437	4,31228017	3,87546151	3,2350026	3,820437	3,86393991	3,90988524	3,88020391	3,74453737	HLII	MIT0604	glk	WP_0428500	Glycolysis	WP_042850043.1		COG0837	Glucokinase	566823..567857

GID.WP_042850341.1_	6,43683202	6,52925499	6,52526099	6,37422373	5,45952353	5,51607852	5,49492788	6,62002937	6,66151649	5,20407388	5,0301433	5,02551223	5,91430258	5,39718737	5,81613465	HLII	MIT0604	pykF	WP_0428503	Glycolysis	WP_042850341.1		COG0469	Pyruvate kin	837814..839607
GID.WP_042849707.1_	6,98665247	7,09492115	6,87431595	7,02792871	6,8887407	6,83478832	6,85658927	7,11824479	6,98879214	7,00544876	7,0122103	7,03018726	7,00595033	7,01899455	6,88472152	HLII	MIT0604	eno	WP_0428497	Glycolysis/Gl	WP_042849707.1		COG0148	Enolase	227820..229112
GID.WP_042850244.1_	5,23176578	5,12790392	4,96355628	5,09043505	4,28167578	4,22058852	4,06532789	4,9147737	4,6238421	4,07261618	4,09133268	4,03745588	4,36599777	4,55195558	4,62132388	HLII	MIT0604	fda	WP_0428502	Glycolysis/Gl	WP_042850244.1		COG0191	Fructose-1,6	752482..753549
GID.WP_042850366.1_	5,50997608	5,67443259	5,79287453	5,75912411	3,77909362	3,97691531	4,04501159	4,63388113	4,37012411	5,19670248	5,50155041	5,53736463	3,30946487	3,85827772	5,39439579	HLII	MIT0604	pgi	WP_0428503	Glycolysis/Gl	WP_042850366.1		COG0166	Glucose-6-p	858488..860071
GID.WP_042850841.1_	7,45789953	7,22240756	7,02545876	7,31897603	5,66534148	5,55841675	5,82031263	7,07780126	7,36399412	6,70230271	6,75717896	6,74888035	6,01427084	5,79041465	7,29582326	HLII	MIT0604	pdhA	WP_0428508	Glycolysis/Kr	WP_042850841.1		COG1071	Pyruvate dehydrogenase E1 component alpha subunit	
GID.WP_042850322.1_	9,86858319	9,85707087	9,6198961	9,30418874	7,14951187	7,07446008	7,02385805	9,67253475	8,97619028	8,64311923	8,80558519	8,81039388	8,80081738	8,60037723	9,03715036	HLII	MIT0604	pdhB	WP_0428503	Glycolysis/Kr	WP_042850322.1		COG0022	Pyruvate del	820416..821399
GID.WP_042851007.1_	4,74799215	4,69353219	4,48153092	4,35106982	5,75926551	5,1247455	5,17816585	4,93186104	4,46430449	5,09199867	5,21487491	5,26629496	5,22260112	5,73393941	4,68902899	HLII	MIT0604	fumC	WP_0428510	Krebs cycle a	WP_042851007.1		COG0114	class II fuma	1502608..1503993
GID.WP_042850333.1_	7,27801033	7,39379072	7,33295715	7,81065871	7,64104445	7,74527279	7,65562099	7,0163635	7,23688256	8,2778157	8,21988105	8,23607896	7,41313309	7,65852746	8,79725077	HLII	MIT0604	glnA	WP_0428503	Nitrogen me	WP_042850333.1		COG0174	Glutamine sy	829172..830593
GID.WP_042851053.1_	8,08818154	8,25958124	7,67438678	7,8413636	8,89482612	8,81106418	8,79685906	7,81168367	7,70768347	7,64454852	7,89644801	7,89141538	8,68938212	8,68617663	8,10610792	HLII	MIT0604	glsF	WP_0428510	Nitrogen me	WP_042851053.1		COG0069	Ferredoxin-c	1551678..1556240
GID.WP_042850236.1_	6,6667709	6,77349267	6,3499832	6,27443942	7,77177274	8,09042293	7,5945837	6,62489009	6,23361592	6,72112363	6,83890121	6,91462786	7,63232267	7,33036238	6,63254472	HLII	MIT0604	edd	WP_0428502	Pentose Phos	WP_042850236.1		COG2084	phosphogluc	742578..743996
GID.WP_042850551.1_	3,59265659	3,65650423	3,50433049	2,69353513	4,58148548	4,22941315	4,39122327	3,75150353	3,00084452	3,72883469	3,85536872	3,98018433	3,48618576	3,85536872	2,32571006	HLII	MIT0604	opcA	WP_0428505	Pentose Phos	WP_042850551.1		COG3429	Glucose-6-P	1014295..1015596
GID.WP_042850517.1_	6,38369573	6,24996232	6,2089033	6,11075385	6,68847444	6,9650754	6,6834451	6,23315888	5,83188064	6,05557475	6,40017544	6,37098832	5,45514466	5,98355696	6,23987073	HLII	MIT0604	rbsK	WP_0428505	Pentose Phos	WP_042850517.1		COG0524	Sugar kinase	985792..986655
GID.WP_042851291.1_	6,43483988	6,35761545	6,41422787	6,86392836	10,4665873	9,63840237	10,2851477	6,20503476	6,13768299	10,5261268	10,5728908	10,5657593	10,2298547	10,7240258	7,74773881	HLII	MIT0604	tal	WP_0428512	Pentose Phos	WP_042851291.1		COG0176	Transaldolas	492474..493475
GID.WP_042850552.1_	4,14176121	3,9537092	3,77671142	3,62742223	6,25635279	6,36959935	6,38223406	4,31144077	3,96559223	5,14866014	5,11087567	5,08890748	5,62785247	5,74692851	4,05767489	HLII	MIT0604	zwf	WP_0428505	Pentose Phos	WP_042850552.1		COG0364	glucose-6-ph	1015599..1017122
GID.WP_042851062.1_	12,3906281	12,4493131	12,6943525	12,5534782	12,0816724	12,473056	12,3232196	12,2767671	12,4492954	12,2815353	12,0745359	12,01981	12,2294929	11,786454	11,9353891	HLII	MIT0604	psaA	WP_0428510	Photosystem	WP_042851062.1		COG2885	Photosystem I P700 chlorophyll A apoprotein A1 PsaA	
GID.WP_011819135.1_	9,0578617	9,18299393	9,84047617	10,2062689	9,36980273	9,70169183	9,42386269	9,6323636	9,76438438	10,0315798	9,63350915	9,68708243	9,66279562	9,21070168	9,31572412	HLII	MIT0604	psaB	WP_0118191	Photosystem	WP_011819135.1		COG2885	Photosystem I P700 chlorophyll A apoprotein A2 PsaB	
GID.WP_042849911.1_	2,77498484	2,45015087	1,9642394	1,92136813	5,16289355	4,57647704	5,23751317	2,18094651	1,82782046	5,78283258	5,61052986	5,55416207	3,96680073	4,9604966	3,10322411	HLII	MIT0604	ctaC/coxB	WP_0428499	Respiration	WP_042849911.1		COG1622	Cytochrome c	427996..428799
GID.WP_042849910.1_	6,84998171	6,68960122	6,9298558	7,28501088	11,608589	11,2880751	11,6477501	8,28001628	7,98635773	10,6377142	10,9748834	10,9479249	10,451332	11,0352054	8,15728536	HLII	MIT0604	cyoB/coxA	WP_0428499	Respiration	WP_042849910.1		COG0843	Cytochrome c	426374..427999
GID.WP_042849909.1_	2,85643019	2,96990312	2,97449312	2,54075375	7,54054739	7,27318876	7,71036379	4,5570175	4,24035017	6,51551055	6,52543349	6,53112393	6,01802329	6,45512953	3,97965094	HLII	MIT0604	cyoC	WP_0428499	Respiration	WP_042849909.1		COG1845	heme-copper	425765..426367
GID.WP_012006816.1_	2,63116862	2,82273639	2,44164084	2,34526354	3,56557986	2,80652584	3,03620659	2,67560797	2,22624273	2,43166467	2,74372296	2,70041276	3,75838061	3,59992984	2,61965589	HLII	MIT9215	argH	WP_0120068	Amino Acid r	WP_012006816.1		COG0165	Argininosucc	14488..15867
GID.WP_012008491.1_	2,89441166	3,08902638	2,6294506	2,0098616	1,96260759	1,29277029	1,58102534	1,87696489	1,69450842	1,75627425	2,36377689	2,36054009	0,87051358	1,7758265	1,51680413	HLII	MIT9215	glmS	WP_0120084	Amino Acid r	WP_012008491.1		COG0449	Glutamine--f	1617261..1619156
GID.WP_012007593.1_	3,73336268	3,8957048	4,39423703	3,92877711	2,19469615	2,83454381	2,87428184	4,43873978	4,73803104	2,82968667	2,83899147	2,82753156	3,67844475	2,56897207	3,16635109	HLII	MIT9215	prk	WP_0120075	Carbon fixati	WP_012007593.1		COG3954	Phosphoribu	767116..768015
GID.WP_002805854.1_	9,08261565	8,5800388	8,72330958	7,6534632	5,88575311	5,63207048	5,92896516	9,29395828	9,5537882	5,83990545	5,69859396	5,72036709	5,94725309	6,50649003	7,43204036	HLII	MIT9215	rbcL	WP_0028058	Carbon fixati	WP_002805854.1		COG1850	Ribulose 1,5-	555111..556526
GID.WP_011376108.1_	7,06310481	7,22946969	7,28664881	5,99017727	2,46820323	2,31215654	1,45119189	8,35068879	8,41100562	3,99765718	4,2870527	4,28565933	1,18211874	3,87217317	6,20623096	HLII	MIT9215	rbcS	WP_0113761	Carbon fixati	WP_011376108.1		COG4451	Ribulose bisp	556620..556961
GID.WP_012008198.1_	7,3976038	7,45935656	7,13727009	7,37241027	7,47809453	7,53009783	7,55994795	6,18443348	6,22126263	7,14804007	7,03201072	7,02742163	6,84370994	7,0803212	6,70986315	HLII	MIT9215	ftsZ	WP_0120081	Cell Division	WP_012008198.1		COG0206	cell division p	1333132..1334241
GID.WP_012007115.1_	2,37036258	2,3802628	2,65389804	2,79868608	2,41171119	2,59985233	2,11594622	2,31553167	2,3568646	2,25237427	2,17271139	2,23996681	2,3305001	2,04066687	2,31534855	HLII	MIT9215	minC	WP_0120071	Cell Division	WP_012007115.1		COG0850	septum site-	313351..314013
GID.WP_012007114.1_	4,14606094	4,19153466	3,72074094	3,20010735	5,56109734	6,47348552	5,50782303	3,19882733	2,22706397	5,96500399	5,83942064	5,86979516	4,49533812	5,09007109	2,00389922	HLII	MIT9215	minD	WP_0120071	Cell Division	WP_012007114.1		COG2894	septum site-	312424..313239
GID.WP_002806062.1_	3,2964239	3,21156732	2,81300147	2,8704482	3,86805114	4,16170718	3,60002277	2,78278063	2,68163748	4,43049091	4,48406273	4,66904539	3,98234939	4,23436387	3,5824662	HLII	MIT9215	minE	WP_0028060	Cell Division	WP_002806062.1		COG0851	septum site-	312096..312422
GID.WP_002807968.1_	4,93129181	5,02922086	5,30315821	4,22997665	5,64764971	5,36788591	5,66138032	5,46999888	5,72130726	4,90825649	4,81635945	4,77587753	6,25892775	6,48862764	5,10569735	HLII	MIT9215	kaiB	WP_0028079	Circadian Rh	WP_002807968.1		COG4251	Circadian clo	1366470..1366787
GID.WP_012008224.1_	7,21608772	7,37625057	6,7088686	6,58845619	6,78324473	6,29356973	6,60583273	6,45234947	6,29937342	5,85444442	6,04597992	6,03792415	6,41911752	6,51609966	7,01098258	HLII	MIT9215	kaiC	WP_0120082	Circadian Rh	WP_012008224.1		COG0467	circadian clo	1364860..1366398
GID.WP_002805160.1_	9,81025066	9,79405159	9,5886805	9,24579178	7,02985644	7,10327209	6,97299328	9,99010694	9,95324418	8,32059586	8,07631292	8,10115027	8,64663992	8,10382081	9,10231358	HLII	MIT9215	atpA	WP_0028051	Energy meta	WP_002805160.1		COG0056	ATP synthas	1466582..1468099
GID.WP_002806495.1_	4,15421687	3,77215238	3,84005588	3,42823296	0,61542115	1,50447903	0,22072971	3,51157958	4,51879871	3,07725443	3,08777543	3,08777543	2,40452673	2,08350476	3,05428947	HLII	MIT9215	atpC	WP_0028064	Energy meta	WP_002806495.1		COG0355	ATP synthas	1456892..1457296
GID.WP_012008357.1_	7,10702453	6,74776388	6,88548635	6,43469006	4,73896969	4,64302592	4,40361509	7,07686988	6,73070075	5,19350859	5,2936892	5,280921	5,15167534	5,24654865	6,06283353	HLII	MIT9215	atpI	WP_0120083	Energy meta	WP_012008357.1		COG0356	ATP synthase	1470122..1470847
GID.WP_012008208.1_	5,14458307	5,18667938	5,02924066	4,71690281	5,00729826	4,86344017	4,58274857	4,75503534	4,24077305	4,41164782	4,71368775	4,6590858	3,75960023	4,88717269	4,25276431	HLII	MIT9215	glcH	WP_0120082	GlcH sugar tr	WP_012008208.1		COG2211	GPH family s	1345728..1347074
GID.WP_012007577.1_	5,00679189	4,7769103	4,90880229	4,64734831	2,77651093	2,6763028	2,91525229	4,17173877	4,04208008	3,6237733	3,78559567	3,79521064	2,87320744	2,65683484	2,91740103	HLII	MIT9215	glgC	WP_0120075	Glycogen me	WP_012007577.1		COG0448	Glucose-1-p	753349..754644
GID.WP_012007413.1_	5,66133841	5,63528511	5,33959965	5,11544892	5,5508487	5,82373687	5,45039276	5,52600813	5,26078331	4,65420329	4,90237787	4,92741608	5,30999683	5,11729249	4,97379864	HLII	MIT9215	glk	WP_0120074	Glycolysis	WP_012007413.1		COG0837	Glucokinase	602516..603550
GID.WP_012007684.1_	6,9386873	6,95598482	6,97702762	6,73813341	6,09538795	5,99097946	6,01585687	7,06603396	6,89144149	5,64887054	5,49823266	5,4938635	6,57068087	5,95935935	6,20712216	HLII	MIT9215	pykF	WP_0120076	Glycolysis	WP_012007684.1		COG0469	Pyruvate kin	848739..850532
GID.WP_012007014.1_	6,82320114	6,87329943	6,7217185	6,55686223	6,89746481	6,80645615	6,8244938	6,63830927	6,45323425	6,85357588	6,90559526	6,94912122	6,86089549	6,96303379	6,51254662	HLII	MIT9215	eno	WP_0120070	Glycolysis/Gl	WP_012007014.1		COG0148	Enolase	208199..209491
GID.WP_012007589.1_	2,58894811	2,50668672	2,38802	2,12745524	1,10674695	1,1456193	1,42348089	2,20899443	1,74034108	1,69523178	1,78794162	1,72003	1,30641559	1,86112202	1,03925555	HLII	MIT9215	fda	WP_0120075	Glycolysis/Gl	WP_012007589.1		COG0191	Fructose-1,6	763429..764496
GID.WP_012007704.1_	6,2682418	6,33859575	6,41074418	6,49616119	4,24291467	4,21096263	4,2416622	4,99273639	4,94210745	6,04510665	6,26199853	6,26448567	4,57030964	4,92001375	5,93472725	HLII	MIT9215	pgi	WP_0120077	Glycolysis/Gl	WP_012007704.1		COG0166	Glucose-6-p	869426..871009
GID.WP_012008176.1_	8,19315544	7,99440097	8,16938241	8,39228808	6,28232403	6,30237946	6,58056436	7,97415906	8,04179283	7,20086915	7,23218825	7,21103244	6,50064042	6,4085087	8,10561659	HLII	MIT9215	pdhA	WP_0120081	Glycolysis/Kr	WP_012008176.1		COG1071	Pyruvate deh	1309929..1311002
GID.WP_012007666.1_	9,05376426	8,90885306	8,89778488	8,63278354	6,24264483	5,89920288	6,15145975	8,8256133	8,54009943	8,10109448	8,22776961	8,20826517	8,14827489	7,85328174	8,39727628	HLII	MIT9215	pdhB	WP_0120076	Glycolysis/Kr	WP_012007666.1		COG0022	Pyruvate del	831329..832312
GID.WP_012008366.1_	4,00065863	3,92146563	3,55907126	2,59153873	5,0389668	4,38518578	4,33256342	3,99884255	3,36911369	4,40356237	4,51244077	4,58968132	4,26821394	4,93095932	3,58522263	HLII	MIT9215	fumC	WP_0120083	Krebs cycle a	WP_012008366.1		COG0114	class II fuma	1478521..1479906
GID.WP_012008463.1_	5,45529446	5,32987087	5,31563942	5,51288236	5,439071	5,08165669	5,30169395	5,54157246	5,44115249	4,87632493	5,11500356	5,07259103	4,9422201	5,04571772	5,44812227	HLII	MIT9215	ppc	WP_0120084	Krebs cycle a	WP_012008463.1		COG2352	Phosphoeno	1584117..1587086
GID.WP_012007675.1_	6,88060511	6,95096461	6,84286903	7,2742503	7,23271884	7,36539137	7,3330839	6,45678678	6,76838295	7,87511776	7,85934363	7,89710467	6,94646908	7,23582562	8,42045216	HLII	MIT9215	glnA	WP_0120076	Nitrogen me	WP_012007675.1		COG0174	Glutamine sy	840097..841518
GID.WP_012007578.1_	3,48732273	3,48280388	3,14024429	2,93346014	4,87451227	5,03925499	4,66708902	3,53714484	3,21657647	4,09190506	4,00444543	3,99452879	4,87424892	4,52474656	3,54031959	HLII	MIT9215	edd	WP_0120075	Pentose Phos	WP_012007578.1		COG2084	phosphogluc	754740..756158
GID.WP_012007898.1_	2,7324685	2,75839013	2,6955336	0,27880003	3,56284858	3,26871937	3,14086367	2,64884335	1,68133617	2,75691102	3,35966054	3,31039057	2,17462128	3,02848752	0,26286689	HLII	MIT9215	opcA	WP_0120078	Pentose Phos	WP_012007898.1		COG3429	Glucose-6-P	1024757..1026058
GID.WP_012007871.1_	4,97973022	4,93292731	4,93292731	4,78142472	4,91958814	5,25714084	4,97235058	4,94423463	4,62666582	4,82646668	4,93656344	4,93292731	4,96215021	4,8598653	5,43339729	HLII	MIT9215	rbsK	WP_0120078	Pentose Phos	WP_012007871.1		COG0524	Sugar kinase	996259..997125
GID.WP_041484446.1_	6,06228389	6,03818737	6,19533317	6,68112403	9,90216288	9,26205216	9,77832123	5,94390719	5,92215951	10,0578509	10,0816951	10,0441592	9,37187148	10,1878354	7,27085014	HLII	MIT9215	tal	WP_0414844	Pentose Phos	WP_041484446.1		COG0176	Transaldolas	526690..527691
GID.WP_012007899.1_	5,23866687	5,10764876	4,95972984	4,58019491	7,44866951	7,53039692	7,46762056	5,57016626	4,8605218	6,1998731	6,25353114	6,19774366	6,76810275	6,99155046	5,20208038	HLII	MIT9215	zwf	WP_0120078	Pentose Phos	WP_012007899.1		COG0364	glucose-6-ph	1026061..1027584
GID.WP_012008418.1_	8,29551139	8,3531551	8,8106653	9,17905412	8,32899075	8,92189657	8,64526211	8,28440874	8,70428998	8,56261393	8,27360286	8,25929449	8,58188557	8,22642131	8,63798121	HLII	MIT9215	psaA	WP_0120084	Photosystem	WP_012008418.1		COG2885	Photosystem	1541410..1543695
GID.WP_002807071.1_	6,3405558	6,52237248	7,36071609	7,7849332	6,83491039	7,23761666	6,95624022	7,31851041	7,41035403	7,45553333	6,96850794	7,03013092	6,58210344	6,33959986	6,68888939	HLII	MIT9215	psaB	WP_0028070	Photosystem	WP_002807071.1		COG2885	Photosystem	1539154..1541382
GID.WP_012007273.1_	1,30328374	1,04306407	1,36171397	0,85764015	4,51982007	4,14108736	4,73266232	1,2929882	0,94449587	5,10658399	5,01311951	5,03210017	3,4152156	4,54090674	1,12118194	HLII	MIT9215	ctaC/coxB	WP_0120072	Respiration	WP_012007273.1		COG1622	Cytochrome c	462411..463214
GID.WP_012007272.1_	5,05009585	4,88580948	5,18926939	5,44438904	9,5494054	9,05111583	9,56814209	6,4366313	6,15435828	8,53899501	8,83975261	8,84825888	8,02061771	8,80724442	6,20827868	HLII	MIT9215	cyoB/coxA	WP_0120072	Respiration	WP_012007272.1		COG0843	Cytochrome c	460789..462414
GID.WP_012007271.1_	1,43818526	1,57906762	1,34777898	0,19023529	5,88414905	5,48150033	5,96983371	2,83067157	1,87126088	4,99258484	5,34253039	5,30587803	4,0057746	4,81479381	1,62979651	HLII	MIT9215	cyoC	WP_0120072	Respiration	WP_012007271.1		COG1845	heme-copper	460180..460782
GID.WP_011862039.1_	2,02732616	2,1430631	1,92712798	2,01883704	2,96738244	2,71371608	2,65617776	2,13979667	1,72100743	1,83884444	1,92745822	1,85088491	3,18586291	2,58196083	2,00003396	HLII	MIT9301	argH	WP_0118620	Amino Acid r	WP_011862039.1		COG0165	Argininosucc	14576..15955
GID.WP_011863709.1_	4,44828185	4,5578826	4,41301508	4,78699681	2,81344823	2,41419666	2,58815132	3,48446543	3,10247722	3,58283334	3,85957751	3,84553676	2,86826903	2,90832654	3,40334808	HLII	MIT9301	glmS	WP_0118637	Amino Acid r	WP_011863709.1		COG0449	Glutamine--f	1520256..1522151
GID.WP_011818291.1_	5,46668686	5,47771577	5,94381535	5,73654661	3,48266002	4,37934202	3,79024167	6,06125641	6,32109377	4,41078514	4,10361	4,07101378	5,02383301	3,97453457	4,73419987	HLII	MIT9301	prk	WP_0118182	Carbon fixati	WP_011818291.1		COG3954	Phosphoribu	733884..734780
GID.WP_011862567.1_	10,00493	9,73268429	9,68445104	8,64142135	6,84484602	6,29887302	6,98009217	10,4768926	10,6935225	7,07824272	6,83478461	6,89745293	7,21423758	7,31675963	8,80499373	HLII	MIT9301	rbcL	WP_0118625	Carbon fixati	WP_011862567.1		COG1850	Ribulose 1,5-	503604..505019
GID.WP_011862568.1_	8,7914083	8,8938699	8,7137606	7,79056387	5,24564101	5,24375834	4,83469147	9,5647002	9,95637592	6,22272156	6,00967387	6,12378551	5,55099054	5,84050615	8,14499996	HLII	MIT9301	rbcS	WP_0118625	Carbon fixati	WP_011862568.1		COG4451	Ribulose bisp	505109..505450
GID.WP_011863425.1_	8,80592234	8,86447807	8,38359074	8,42832513	8,87419431	8,83637438	8,93625535	7,49294812	7,23265975	8,22948619	8,16551154	8,15768949	8,20969363	8,40262137	7,89418209	HLII	MIT9301	ftsZ	WP_0118634	Cell Division	WP_011863425.1		COG0206	cell division p	1264859..1265974
GID.WP_011862358.1_	3,64172653	3,59417845	3,90682369	3,99759652	3,83741869	3,86199846	3,51039393	3,62383861	3,68571355	3,52370002	3,5451867	3,62583664	3,35383503	3,4215173	3,47997956	HLII	MIT9301	minC	WP_0118623	Cell Division	WP_011862358.1		COG0850	septum site-	316052..316714
GID.WP_011862357.1_	3,69135068	3,84687184	3,16300684	2,98958015	5,40898935	6,17650337	5,37228841	2,2490964	1,85490824	5,91371677	5,65903551	5,67466852	4,18795455	4,86564663	1,69503696	HLII	MIT9301	minD	WP_0118623	Cell Division	WP_011862357.1		COG2894	septum site-	315125..315940
GID.WP_011862356.1_	3,17489403	3,07139396	2,84184804	2,96055771	4,26552431	4,29622652	3,98801373	3,12706561	3,48939631	4,60006598	4,60420099	4,57895872	3,88338549	4,13320619	3,11609277	HLII	MIT9301	minE	WP_0118623	Cell Division	WP_011862356.1		COG0851	septum site-	314788..315123
GID.WP_011863456.1_	4,1610691	4,19600327	4,31839031	4,05799129	5,07111595	4,64303338	4,81262906	4,80226759	4,68969038	3,95406761	4,16987912	4,1011439	5,40120045	5,57090626	4,26715374	HLII	MIT9301	kaiB	WP_0118634	Circadian Rh	WP_011863456.1		COG4251	Circadian clo	1298194..1298511
GID.WP_011863455.1_	7,87055353	7,95179954	7,51544826	7,38006373	7,38305804	7,06698277	7,36554555	7,06346641	7,15649666	6,79601469	6,97683325	7,00049363	7,40946001	7,53008978	7,91177681	HLII	MIT9301	kaiC	WP_0118634	Circadian Rh	WP_011863455.1		COG0467	circadian clo	1296596..1298125
GID.WP_011863563.1_	9,3742727	9,32639346	9,28294657	8,74355451	6,9001583	6,8720346	6,79572572	9,56660116	9,61417728	7,69401712	7,37586825	7,38046101	8,01043806	7,54908031	8,47909049	HLII	MIT9301	atpA	WP_0118635	Energy meta	WP_011863563.1		COG0056	ATP synthas	1373011..1374528
GID.WP_011863551.1_	6,30212658	6,22305763	6,03942461	5,60319873	3,07014993	3,86823212	2,67945004	6,31967843	6,23562231	4,72298405	4,7180548	4,76541456	4,59796303	3,9859013	5,12152303	HLII	MIT9301	atpC	WP_0118635	Energy meta	WP_011863551.1		COG0355	ATP synthas	1363319..1363723
GID.WP_011863567.1_	7,06476406	6,71644623	6,80163722	6,49250599	5,03020846	5,0704079	5,26905233	7,07095476	6,79786081	5,34951081	5,34804942	5,30301058	5,87083872	5,466439	6,40361033	HLII	MIT9301	atpI	WP_0118635	Energy meta	WP_011863567.1		COG0356	ATP synthase	1376551..1377276
GID.WP_011862476.1_	4,66976582	4,48774463	4,19919828	3,97080469	4,40263595	3,84243378	4,07678771	4,01222269	3,52540219	3,82170889	4,07678771	4,07678771	3,21330775	4,151086	3,98486684	HLII	MIT9301	gdh	WP_0118624	Entner-Dudo	WP_011862476.1		COG1028	3-oxoacyl-ac	418245..418994
GID.WP_011863437.1_	4,79929664	4,88419689	4,70615508	4,22485729	4,76124027	4,52725749	4,44359807	4,54033154	4,36077142	4,12745311	4,44009736	4,51113402	3,77665068	4,72450028	4,14038598	HLII	MIT9301	glcH	WP_0118634	GlcH sugar tr	WP_011863437.1		COG2211	GPH family s	1277456..1278802
GID.WP_011862806.1_	7,42170866	7,20294471	6,97378939	6,86291892	4,77563344	5,1189685	4,83955082	6,49217151	6,27522057	5,94334219	6,05438984	6,12490821	5,5983675	4,94795051	5,64652887	HLII	MIT9301	glgC	WP_0118628	Glycogen me	WP_011862806.1		COG0448	Glucose-1-p	720123..721418
GID.WP_011862612.1_	4,66252069	4,51344983	4,26012551	3,70373799	4,30058943	4,21013441	3,99664126	4,29791979	3,77793184	3,20068062	3,65148401	3,63870703	3,50403147	3,56937783	3,54450064	HLII	MIT9301	glk	WP_0118626	Glycolysis	WP_011862612.1		COG0837	Glucokinase	549579..550613
GID.WP_011862918.1_	5,87121119	5,98027459	5,94639122	5,63218868	4,92383077	4,85581367	4,70270132	6,13023215	6,08293171	4,51599128	4,45412452	4,51460988	5,10222232	4,82694673	5,17539235	HLII	MIT9301	pykF	WP_0118629	Glycolysis	WP_011862918.1		COG0469	Pyruvate kin	815554..817344
GID.WP_011376344.1_	9,12269417	9,16107776	9,06871636	9,06034894	7,4578829	8,21548163	7,29729199	9,93612418	9,49439244	7,55921488	7,47628693	7,53748063	9,07283508	7,88879662	9,1596906	HLII	MIT9301	cbbA	WP_0113763	Glycolysis/Gl	WP_011376344.1		COG0191	Fructose-1,6	730199..731272
GID.WP_011862251.1_	7,27381841	7,38198199	7,18560197	7,17760601	7,3739316	7,30768373	7,31586879	7,30940368	7,08715446	7,47791333	7,46453377	7,49689514	7,83681485	7,5942951	7,12548534	HLII	MIT9301	eno	WP_0118622	Glycolysis/Gl	WP_011862251.1		COG0148	Enolase	212228..213520
GID.WP_011862940.1_	5,69451854	5,69629063	5,80646654	5,878056	3,676469	3,712949	3,67642182	4,50180374	4,43541218	5,48701919	5,6701619	5,68820492	4,17479703	4,42966242	5,4692949	HLII	MIT9301	pgi	WP_0118629	Glycolysis/Gl	WP_011862940.1		COG0166	Glucose-6-p	836239..837822
GID.WP_011863403.1_	8,3207913	8,24401926	8,20133572	8,34377435	6,3467858	6,28799934	6,58620317	8,09001051	8,07401154	7,3886678	7,44239865	7,4265292	6,43693961	6,60156134	8,33703787	HLII	MIT9301	pdhA	WP_0118634	Glycolysis/Kr	WP_011863403.1		COG1071	Pyruvate deh	1241667..1242740
GID.WP_011862899.1_	9,1463169	9,0655179	8,88040141	8,64149994	6,39886949	6,25519195	6,35406861	9,05491767	8,43269179	7,88440562	7,99962312	8,01096922	7,95779475	7,77425045	8,27669703	HLII	MIT9301	pdhB	WP_0118628	Glycolysis/Kr	WP_011862899.1		COG0022	Pyruvate del	798152..799135
GID.WP_011863576.1_	3,58633235	3,5845415	3,25801193	2,85449907	4,25691958	3,87144539	3,7352994	3,30030261	3,21968429	3,7179602	3,94088411	4,02116939	3,62356788	4,2198378	3,40555734	HLII	MIT9301	fumC	WP_0118635	Krebs cycle a	WP_011863576.1		COG0114	class II fuma	1384941..1386326
GID.WP_011863677.1_	6,00855955	5,95007913	5,89823768	5,90345657	5,84424219	5,55416294	5,74812782	6,16073597	5,84539292	5,21308946	5,45543191	5,49709665	5,31925967	5,4094172	5,7161131	HLII	MIT9301	ppc	WP_0118636	Krebs cycle a	WP_011863677.1		COG2352	Phosphoeno	1487262..1490231
GID.WP_011862910.1_	7,41710206	7,51020758	7,44358299	7,69473786	7,32701195	7,34631475	7,35396118	6,80930817	7,09880438	7,96053947	7,86052629	7,92031521	6,96955612	7,28552286	8,53266593	HLII	MIT9301	glnA	WP_0118629	Nitrogen me	WP_011862910.1		COG0174	Glutamine sy	806913..808334
GID.WP_011863621.1_	6,77580835	6,9600317	6,550643	6,5660398	7,33875283	7,12101713	7,45345378	6,76069897	6,81488702	6,32643591	6,52295978	6,50570568	6,78768694	7,20856963	6,78742568	HLII	MIT9301	glsF	WP_0118636	Nitrogen me	WP_011863621.1		COG0069	Ferredoxin-c	1435535..1440106
GID.WP_011862807.1_	3,7189634	3,73343301	3,19274128	2,22406947	5,8064586	6,0495654	5,63555677	3,45510967	3,34986083	4,73598284	4,71970448	4,68739306	5,66841459	5,487344	3,25816414	HLII	MIT9301	edd	WP_0118628	Pentose Phos	WP_011862807.1		COG2084	phosphogluc	721514..722932
GID.WP_011863131.1_	2,34071092	2,12960994	2,07541156	1,94779478	3,20259935	3,05508339	2,80556094	2,10016423	2,00048074	2,41327935	2,55525917	2,65486819	1,94002681	2,47106949	1,43431676	HLII	MIT9301	opcA	WP_0118631	Pentose Phos	WP_011863131.1		COG3429	Glucose-6-P	991842..993143
GID.WP_011863103.1_	6,17026327	5,77164432	6,26869102	6,32323616	5,81322685	5,91687703	5,6643176	5,72989586	5,90164785	5,90753508	5,97966967	5,92048951	4,78532232	5,46872321	6,32576346	HLII	MIT9301	rbsK	WP_0118631	Pentose Phos	WP_011863103.1		COG0524	Sugar kinase	963338..964207
GID.WP_011862538.1_	6,05117994	6,22877406	6,04976274	6,5598726	10,2652978	9,47214987	10,0598434	5,82457482	5,73242892	10,3032153	10,3184523	10,3546645	9,64912611	10,5390781	7,47959746	HLII	MIT9301	tal	WP_0118625	Pentose Phos	WP_011862538.1		COG0176	Transaldolas	475195..476196
GID.WP_011863132.1_	4,40669284	4,28254742	4,0066374	3,9374165	6,49707979	6,52438414	6,58375045	4,58815502	4,07872289	5,36195682	5,42283537	5,41096706	5,83765125	6,19222709	3,98252562	HLII	MIT9301	zwf	WP_0118631	Pentose Phos	WP_011863132.1		COG0364	glucose-6-ph	993146..994669
[truncated: 242,886 more chars]
